# Supplementary material for: Genome-Wide CRISPR Screen Identifies Genes Involved in Metastasis of Pancreatic Ductal Adenocarcinoma
Source: Cancers (Basel). 2024 Oct 31;16(21):3684. doi: 10.3390/cancers16213684 (PMC11545026; doi:10.3390/cancers16213684)
Supplement: Supplementary file 1 [file cancers-3260552-supplementary.pdf › cancers-3260552-supplementary.pdf]

SUPPLEMENTARY INFORMATION

Genome-wide CRISPR screen identifies genes involved in metastasis of pancreatic ductal adenocarcinoma

Risky Oktriani, Anna Chiara Pirona, Lili Kalmár, Ariani S. Rahadian, Beiping Miao, Andrea S. Bauer, Jörg D. Hoheisel, Michael Boettcher and Haoqi Du

1. Supplementary Method Information: pages 2 to 5
2. Supplementary Figures: pages 6 to 10
3. Full pictures of the Western blots pages 11-16
4. Supplementary Tables:
   - Suppl. Table 1: page 17
   - Suppl. Table 2 page 19
   - Suppl. Table 3 page 193
   - Suppl. Table 4 page 226
   - Suppl. Table 5 page 237
   - Suppl. Table 6 page 241
   - Suppl. Table 7 page 245

SUPPLEMENTARY METHOD INFORMATION

Preparation of sgRNA constructs

Plasmid pSicoR MP-783 was from Dr. Michael Böttcher (University of Halle, Germany); plasmids pAW12.lentiguide.GFP and pAW13.lentiguide.mCherry were purchased from Addgene (Watertown, USA). Cloning of sgRNAs into plasmids was carried out as described (references R1 and R2; see below). Transformation was done with One-Shot STBL3 *Escherichia coli* (Thermo Fisher Scientific, Schwerte, Germany) according to the manufacturer's protocol. The success of sgRNA insertion and transformation was confirmed by DNA sequencing.

CRISPR library expansion and lentivirus production

CRISPR library preparation was done prior to lentivirus production to ensure sufficient plasmid viability. One tube of ElectroMAXTM DH5α-E competent cells (Thermo Fisher Scientific) was mixed with 100 ng CRISPR library (R3). For transformation, a Gene Pulser II electroporator (Bio-Rad, Hercules, USA) was used at 2 kV, 200 Ω, and 25 μF. Cells were grown at 37°C overnight and then subjected to plasmid isolation using Maxiprep (Qiagen, Hilden, Germany). Several cell dilutions (1:100 down to $1:10^6$) were prepared; 100 μl each was taken and plated onto the LB-ampicillin agar plates. After overnight incubation at 37°C, colony numbers were counted to determine the transformation efficiency. To ensure an even representation of the sgRNA sequences, 70 clones per sgRNA were required. Lentivirus production was performed as described in much detail before (R3).

Establishment of Cas9 stable cells

Lentivirus containing plasmids lentiCas9-Blast, psPAX2 and pMD2.G (Addgene) were prepared as described previously (R4). 60,000 cells were plated in a 6-well plate and incubated overnight. Transduction was with the Cas9-lentivirus in DMEM medium supplemented with 1% penicillin-streptomycin containing 8 μg/ml polybrene (Merck Millipore, Burlington, USA). The next day, the medium was replaced with DMEM medium. Three days after transduction, cells were treated with blasticidin for selection. Then the cells were cultured until they reached 90% confluency, expanded and stored for further analysis. For validation, Cas9 Western blot and a Cas9 functionality assay were performed. Cas9 functionally was tested by transducing the $Cas9^{+}$-cells with a plasmid containing an sgRNA, which targets EPCAM (**Suppl. Tab. 7**). The loss of EPCAM expression as measured by flow cytometry indicated the Cas9 activity. Anti-EPCAM fluorescein isothiocyanate (FITC) antibody (BD Biosciences, Franklin Lakes, USA) or FITC mouse IgG isotype control antibody (BD Biosciences) were used to this end. Cells were analyzed with a Guava EasyCyte HT flow cytometer (Luminex, Austin, USA).

Protein isolation

Protein isolation was initiated by discarding the medium in the culture dishes. Cells were washed with cold PBS and treated with lysis buffer that was prepared by mixing RIPA buffer (Thermo Fisher Scientific), 0.25 U/µl Benzonase Nuclease (Merck Millipore), and protease-phosphatase inhibitor single use (Cell Signaling Technology, Danvers, USA). Cells were then collected in microcentrifuge tubes, agitated for 30 min at 4°C and subsequently homogenized using a 20 gauge syringe. After centrifugation at 16,000 g for 20 min at 4°C, the supernatant was collected. Protein concentration was determined with the Pierce BCA assay kit (Thermo Fischer Scientific).

Western blot analysis

After gel electrophoresis, proteins were transferred to a nitrocellulose membrane (Cytiva, Marlborough, USA) using the Trans-Blot Turbo Transfer System (Bio-Rad). Subsequently, the membrane was washed and incubated in 5% skimmed milk in 1x Tris-buffered saline-Tween 20 (TBST) blocking solution for 1 h. The membrane was then incubated with the relevant primary antibody overnight at 4°C. The next day, the membrane was incubated for 1 h with the secondary antibody before being prepared for signal detection using Immobilon Western horseradish peroxidase (HRP) substrate luminol reagent (Merck Millipore) following the manufacturer's protocol. Images were acquired with an ECL ChemoStar chemiluminescence imaging system (Intas Science Imaging, Göttingen, Germany).

Primary antibodies used in the study: anti-Cas9, 1:1000 dilution (Active Motif, Carlsbad, USA); anti-thymidylate synthase (TS), 1:750 dilution (Abcam, Cambridge, UK); anti-MYB Proto-Oncogene Like 2 (MYBL2) protein clone LX015.1, 1:750 dilution (Merck Millipore); anti-Menin, 1:500 dilution (Abcam); anti-FOXM1, 1:1,000 dilution (Cell Signaling Technology); anti-GAPDH, 1:1,000 dilution (Merck Millipore); anti-α-tubulin, 1:1,000 dilution (Abcam). Second antibodies were horse anti-mouse Ig (H+L), peroxidase, 1:5,000 to 1:10,000 dilution and goat anti-rabbit Ig (H+L), peroxidase 1:5,000 to 1:10,000 dilution (Vector Laboratories, Burlingame, USA).

CRISPR screening

CRISPR screening was initiated by plating $3x10^6$ cells in 50 petri dishes of 15 cm diameter. After overnight incubation, the cells of 34 plates were transduced with lentivirus at an MOI of 0.3 in DMEM medium supplemented with 10% fetal bovine serum (FBS) containing 8 µg/ml polybrene. Two plates acted as negative controls without virus transduction. After another overnight incubation, the plates were replenished with fresh DMEM medium supplemented with 10% FBS and 1% penicillin-streptomycin. Forty-eight hours after transduction, cells from the 34 plates were harvested and transferred to 170 T175 flasks to prevent over-confluency and loss of screening coverage. Growth was in presence of 5 µg/ml puromycin for 72 h. To preserve coverage, $300 x 10^6$ cells were then plated into 150 T175 flasks, with each flask contained $2x10^6$ cells. The same number of cells (300 x

10^6 cells) were collected (sample at t_{zero}), carefully pelleted, briefly washed and stored frozen at -20°C. The cells in culture were incubated for another 18 days. Every three days, the medium was refreshed. Cells were harvested and pooled every time they reached confluency (about every 6 days). To maintain screening coverage, 300 x 10^6 cells were plated in new flasks. After 18 days, the cells were harvested, pooled and counted once again. Finally, 300 x 10^6 cells were pelleted, briefly washed and stored frozen at -20°C.

DNA isolation

Cell pellets were resuspended in 20 ml P1 buffer (Qiagen), 100 µg/ml RNAse (Qiagen), and 0.5% SDS, and incubated at 37°C for 30 min. 100 µg/ml proteinase K (Thermo Fischer Scientific) was added followed by an incubation at 55°C for 30 min. Cells were homogenised by being pressed through needles six times. The homogenized cells were treated with phenol:chloroform:isoamyl alcohol (Thermo Fischer Scientific) and centrifuged at 1500 g for 5 min. After DNA precipitation with isopropanol, the DNA-pellet was washed with 70% ethanol, dried at 37°C and resuspended in water. The DNA concentration was measured using a NanoDrop spectrophotometer ND-1000 (Thermo Fischer Scientific), adjusted to 1 µg/µl and stored at -20°C until further use.

Recovery of sgRNA sequences

The DNA was fragmented by boiling at 95°C. A first PCR was done by mixing 5 µg DNA, 25 µl NEB Next Ultra II Q5 master mix (New England Biolabs, Frankfurt, Germany), and 0.5 µM each of forward d(GGCTTGGATTTCTATAACTTCGTATAGCA) and reverse d(CGGGGACTGTGGGC GATGTG) primers in 50 µl. Incubation was at 98°C for 30 sec, followed by 16 cycles of 98°C for 30 sec and 65°C for 75 sec as well as a subsequent incubation at 65°C for 5 min. For the second PCR, 2 µl of the first PCR product was mixed with 25 µl NEB Next Ultra II Q5 master mix, 0.5 µM each of forward d(AATGATACGGCGACCACCGAGATCCACAAAAGGAAACTCACCCTAAC) and reverse d(CAAGCAGAAGACGGCATACGAGAT-$(N)_6$-GTGACTGGAGTTCAGACGTG) primer. The 344 bp amplicon was extracted from an agarose gel using NucleoSpin Gel and PCR Clean (Macherey-Nagel, Düren, Germany). The Qubit dsDNA HS Assay kit and Qubit 3 fluorometer (Thermo Fischer Scientific) was used for measuring DNA concentration. The DNA was subjected to sequencing using Hiseq 2000 v4 single-read 50 bp runs with the sequencing primer d(GAGACTATAAGTATCCCTTGGAGAACCACCTTGTTGG).

Double transduction and sorting

Three and a half million S2-028 and S2-007 cells each were plated in a T175 flask, incubated overnight and subjected to double transduction. The next day, the medium was refreshed with DMEM complete medium. Two days after transduction, cells were harvested, diluted in 1 ml BD FACS Pre-Sort Buffer (BD Biosciences) containing 5.4 µg/ml 7-aminoactinomycin D (Merck Millipore), filtered

and sorted using a FACS Aria (BD Biosciences). GFP and mCherry positive, double-transducted cells were sorted and collected in DMEM complete medium, washed with PBS and grown for 3 days for further analyses.

Cell viability measurement using resazurin assay

Cells were plated in 6-well plates. After a given incubation time, the medium was discarded. Resazurin (5 mg/ml) in DMEM complete growth medium (Thermo Fisher Scientific) was then added. The reduction of resazurin (blue to pink fluorescence) by mitochondrial enzymes in viable cells (R5) was detected in a fluorescence microplate reader (Fluostar Galaxy; BMG Labtech, Ortenberg, Germany) at excitation at 544 nm and emission of 590 nm.

SUPPLEMENTARY REFERENCES

R1. Sanjana NE, Shalem O, Zhang F. Improved vectors and genome-wide libraries for CRISPR screening. Nat Meth. 2014;11:783-784.

R2. Shalem O, Sanjana NE, Hartenian E, Shi X, Scott DA, Mikkelsen TS, et al. Genome-scale CRISPR-Cas9 knockout screening in human cells. Science. 2014;343(6166):84–7.

R3. Pirona AC, Oktriani R, Boettcher M, Hoheisel JD. Process for an efficient lentiviral cell transduction. Biol Methods Protoc. 2020;5(1):bpaa005.

R4. Boettcher M, Tian R, Blau JA, Markegard E, Wagner RT, Wu D, et al. Dual gene activation and knockout screen reveals directional dependencies in genetic networks. Nat Biotechnol. 2018;36(2):170-178.

R5. O'Brien J, Wilson I, Orton T, Pognan F. Investigation of the Alamar Blue (resazurin) fluorescent dye for the assessment of mammalian cell cytotoxicity. Eur J Biochem. 2000;267(17):5421-5426.

SUPPLEMENTARY FIGURES

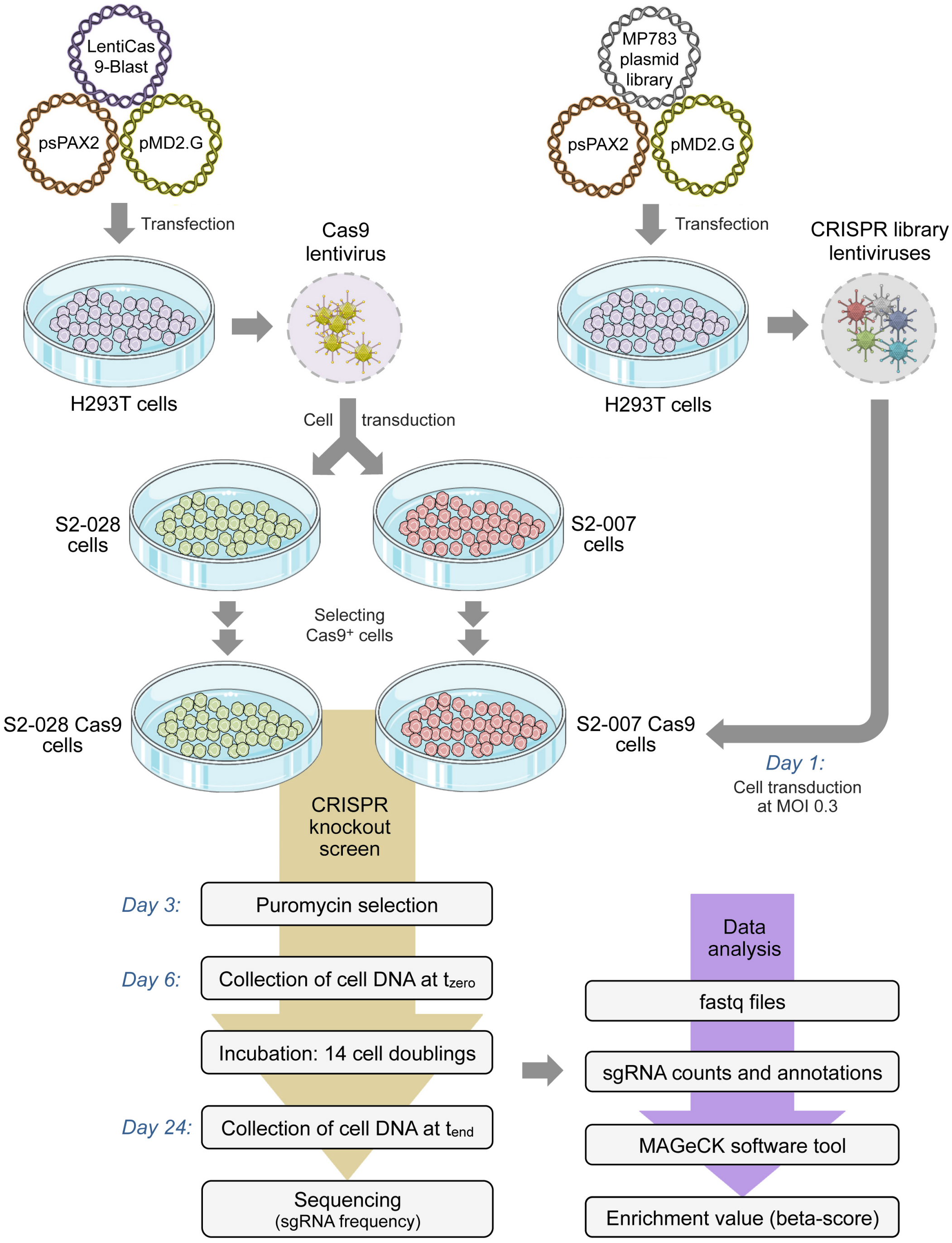

Supplementary Figure S1. Overview on CRISPR-Cas9 screening process. After the creation of stable $Cas9^{+}$ cells and production of the lentivirus library with 259,900 sgRNA constructs, the cells were plated at day 0 followed by transduction with the viral sgRNA library on day 1. The selection of transduced cells was performed on day 3. After another 3 days of growth, an aliquot was taken and used for isolation of genomic DNA. After 18 days, a second sample was collected. Both DNA isolates were studied by sequencing analysis.

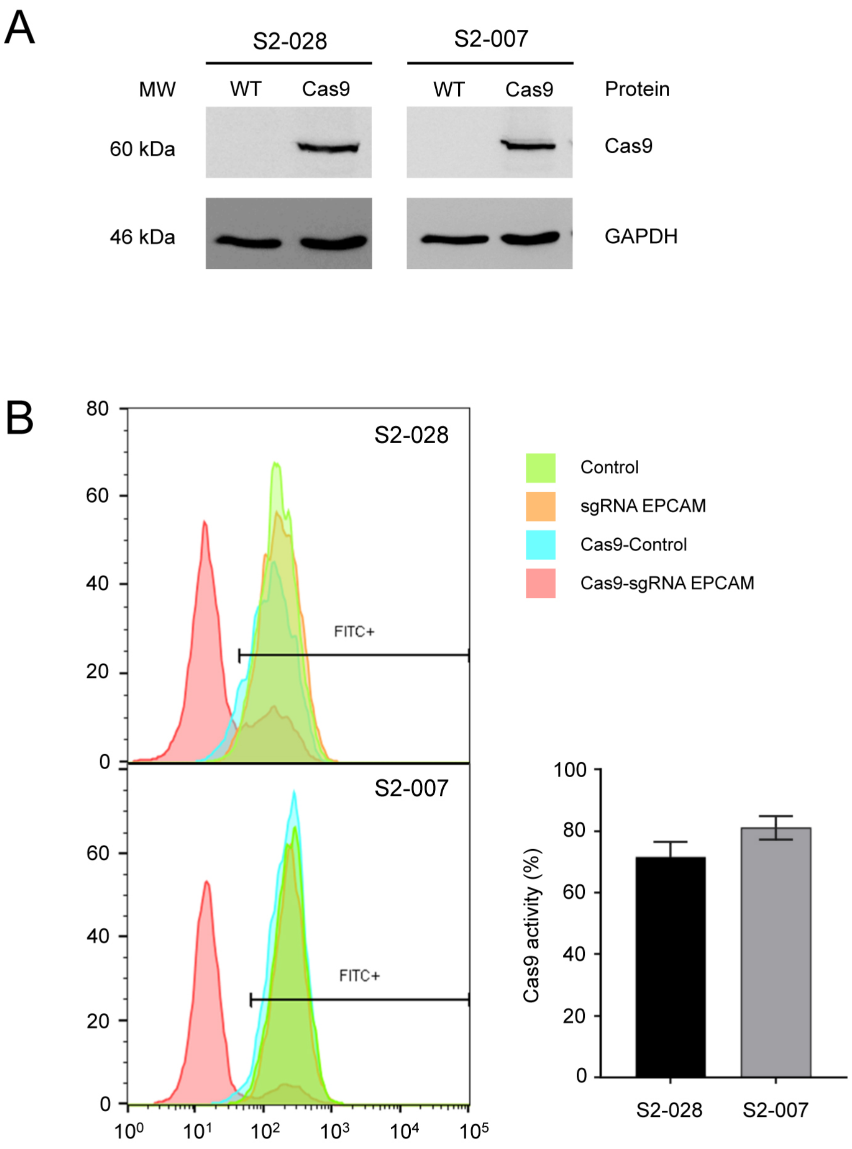

Supplementary Figure S2. Characterisation of Cas9^{+} cells. (**A**) Western blot analysis of S2-007 and S2-028 cells before (WT) and after transduction with Cas9-lentiviruses (Cas9). Stable expression of Cas9 protein was detected by Western blotting in both cell lines. (**B**) Flow cytometry was applied to determine the functionality of Cas9 in both cell lines; an sgRNA construct targeting the gene *EPCAM* was used to this end; transduction was to WT or Cas9^{+} cells. 'Control' stands for cells without transduction. In the bar chart, both the mean and standard deviation are shown as calculated from the data shown left.

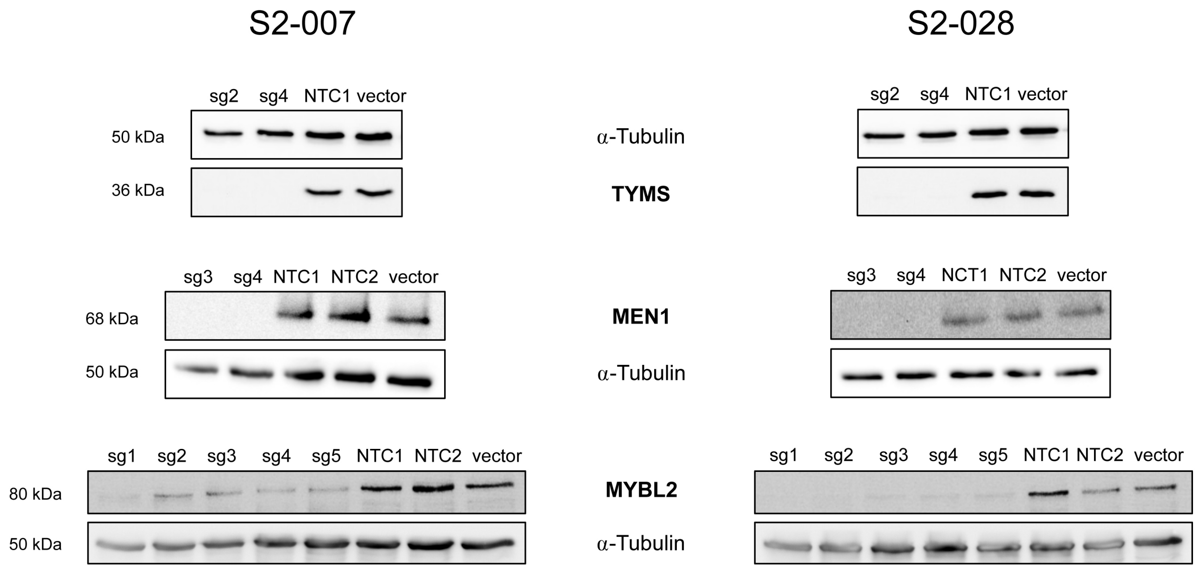

Supplementary Figure S3. Western blot analyses of knockout cells. Typical results are shown for some sgRNA molecules. The identifier of the respective sgRNA construct is shown in a shortened version, e.g., TYMS-sgRNA2 = sg2. NTC stands for non-targeting control.

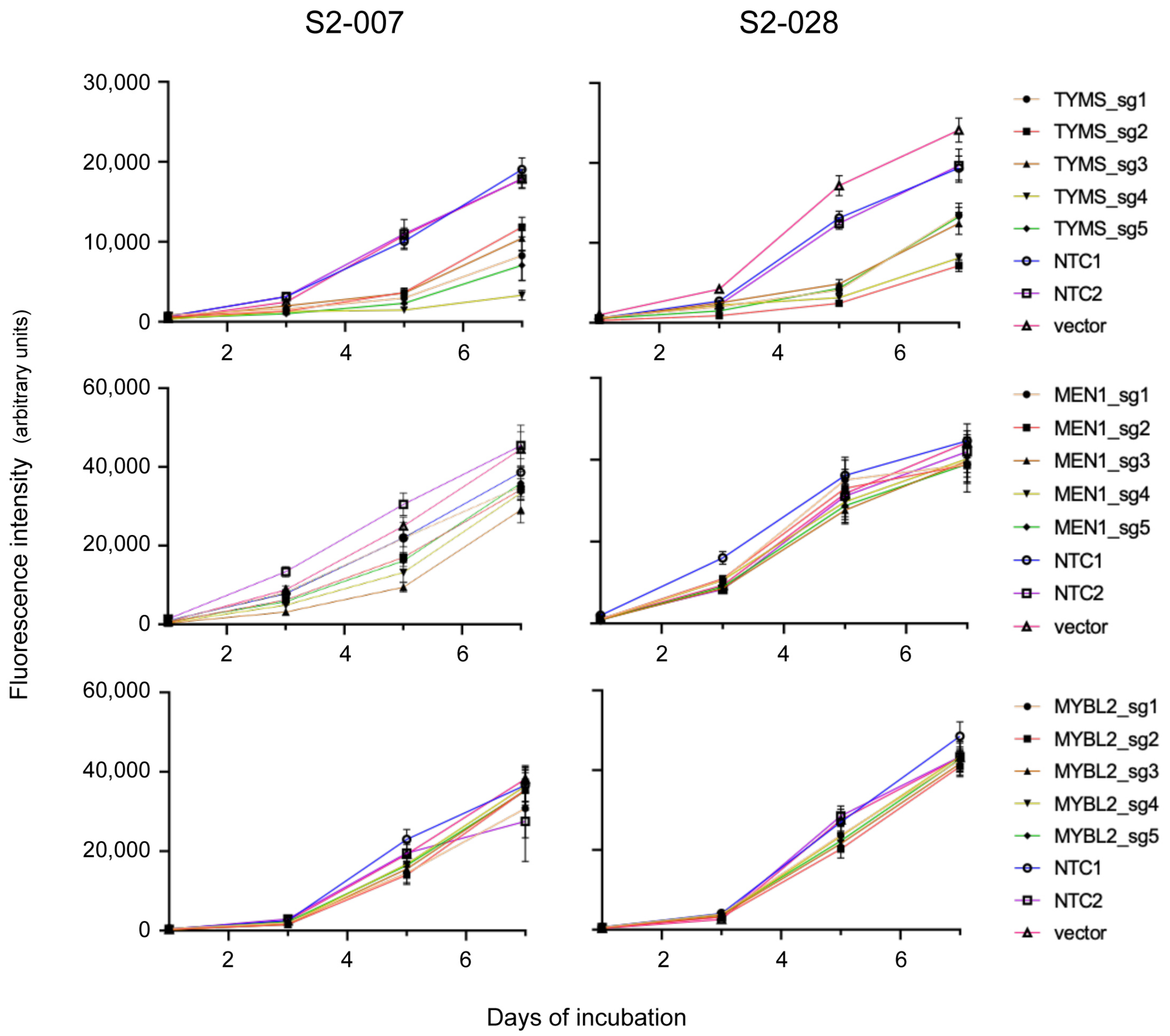

Supplementary Figure S4. Cell proliferation. Cell proliferation was determined with the resazurin assay; eight independent measurements were done for each knockout construct in each cell type. The targeted gene and the identifier of the respective sgRNA construct are shown. NTC stands for non-target control.

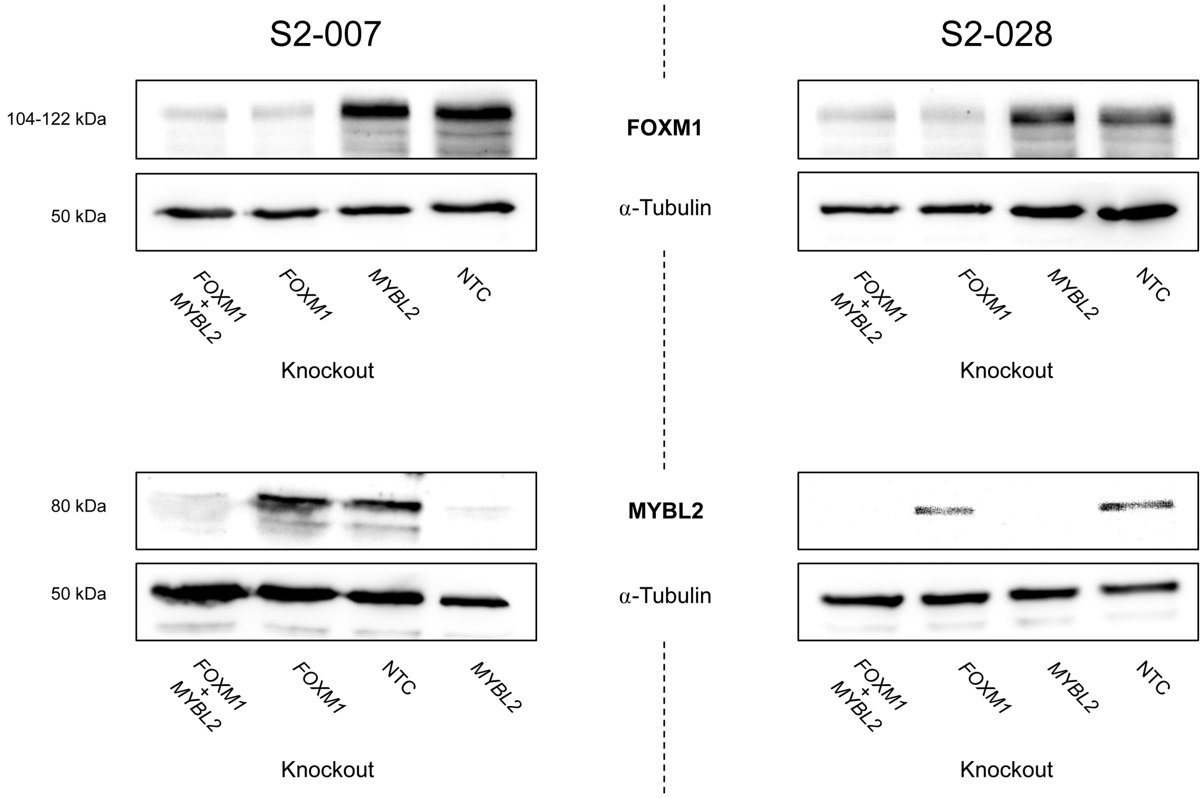

Supplementary Figure S5. Western blot analyses of knockout cells. Results are shown for single- or double-gene knockouts as indicated. NTC stands for non-targeting control. Note the change in the lane order in the MYBL2 analysis in S2-007.

Original Western blots of data shown in Suppl. Fig. S2

S2-028

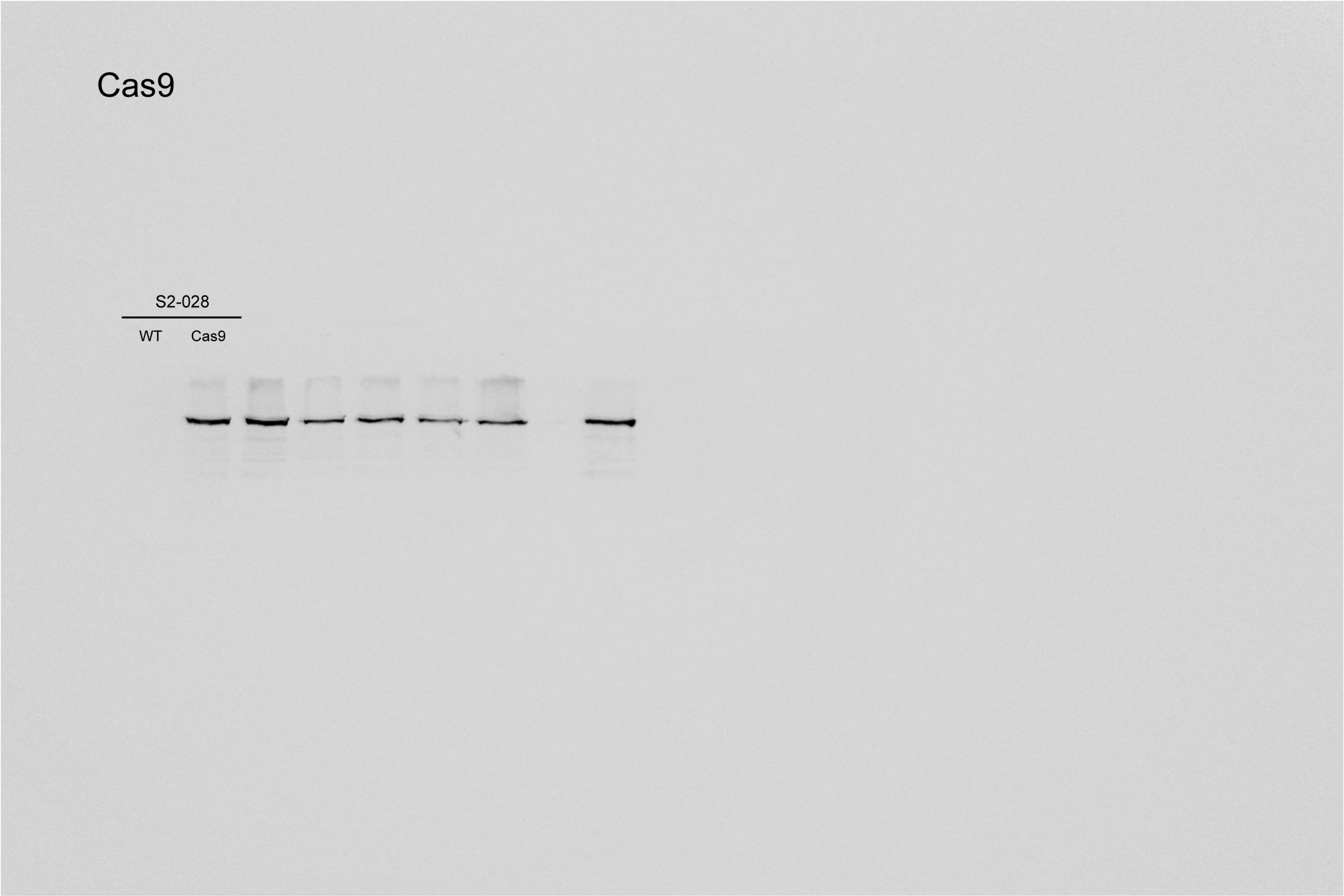

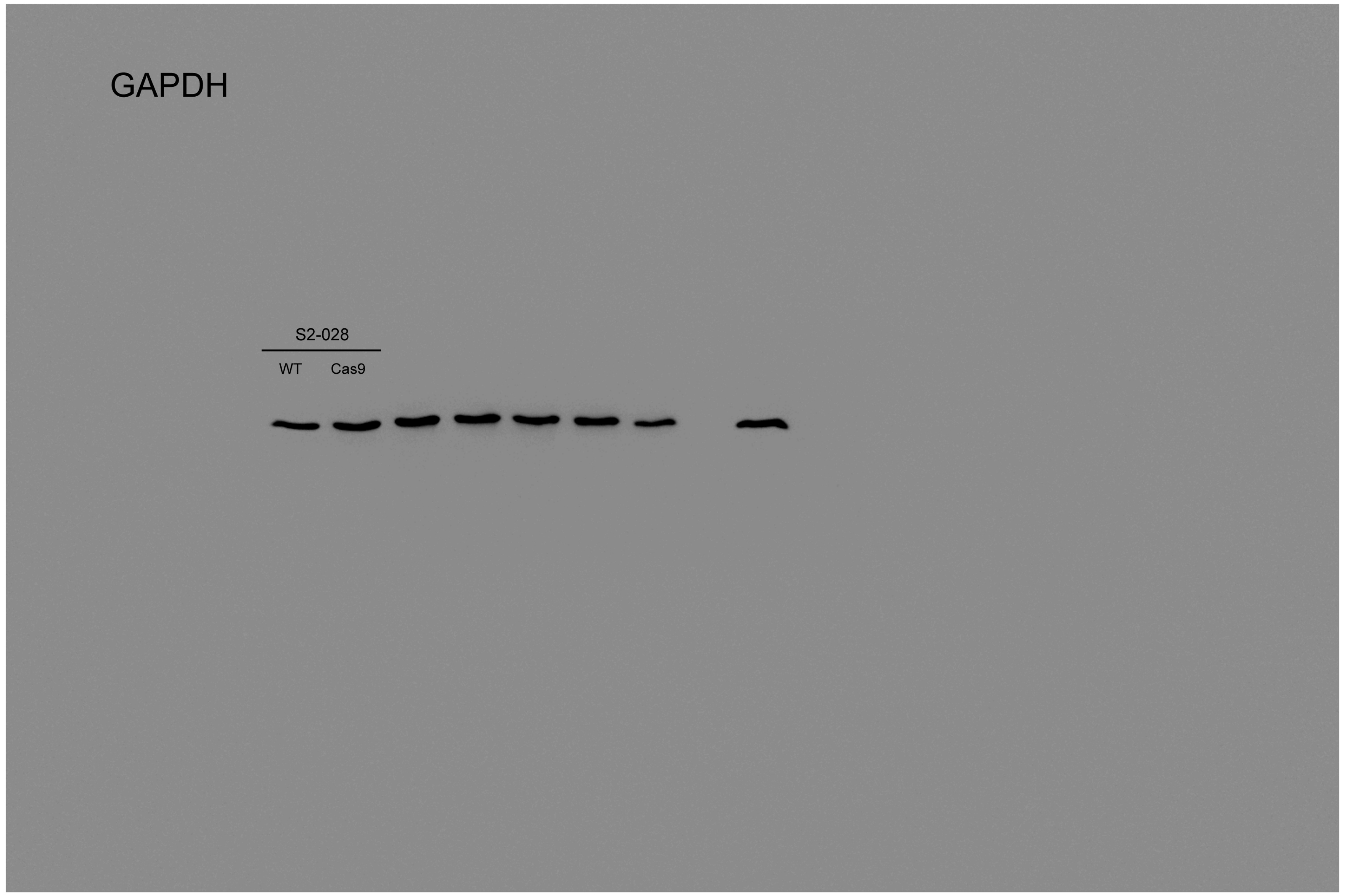

S2-007

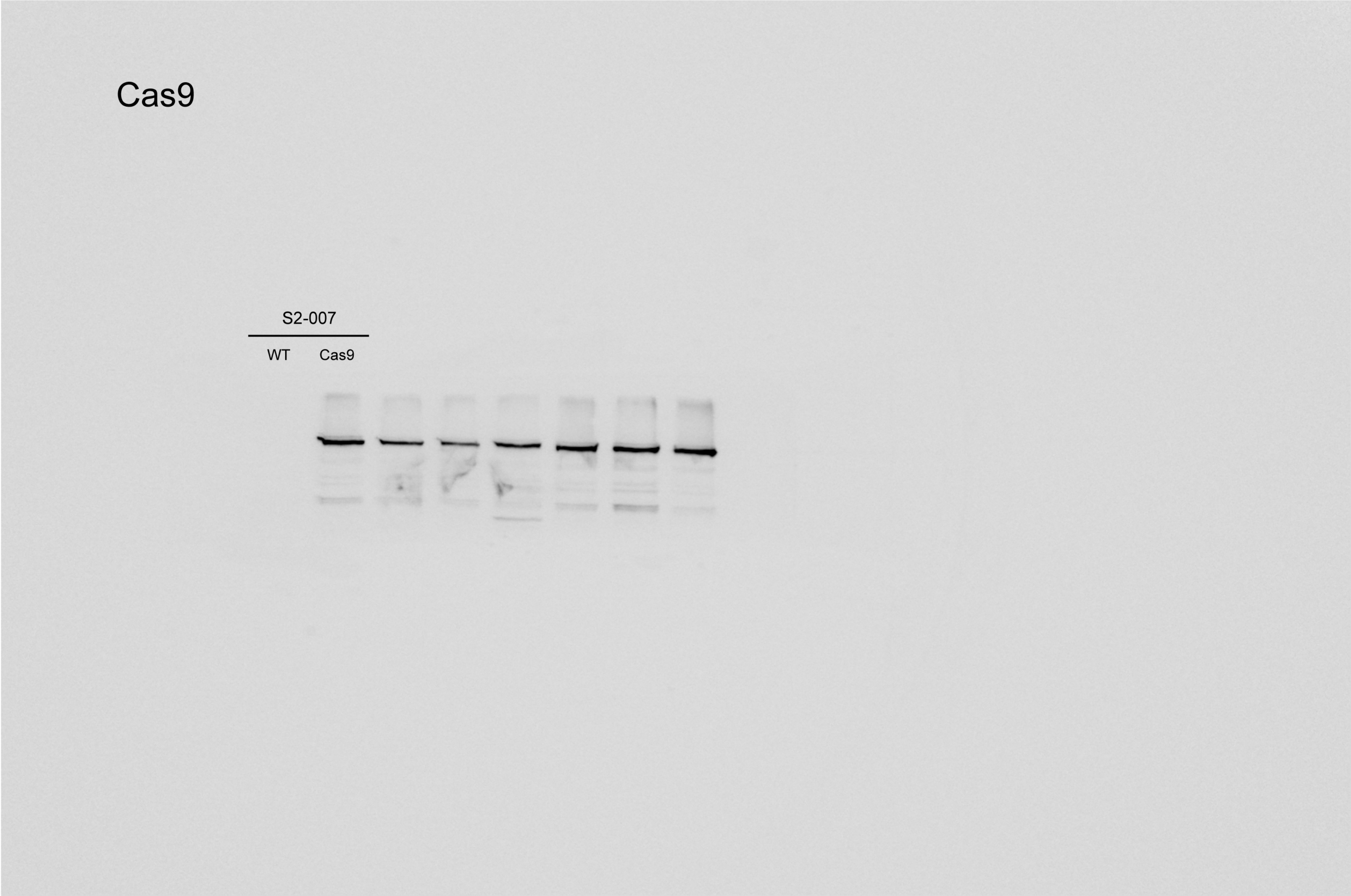

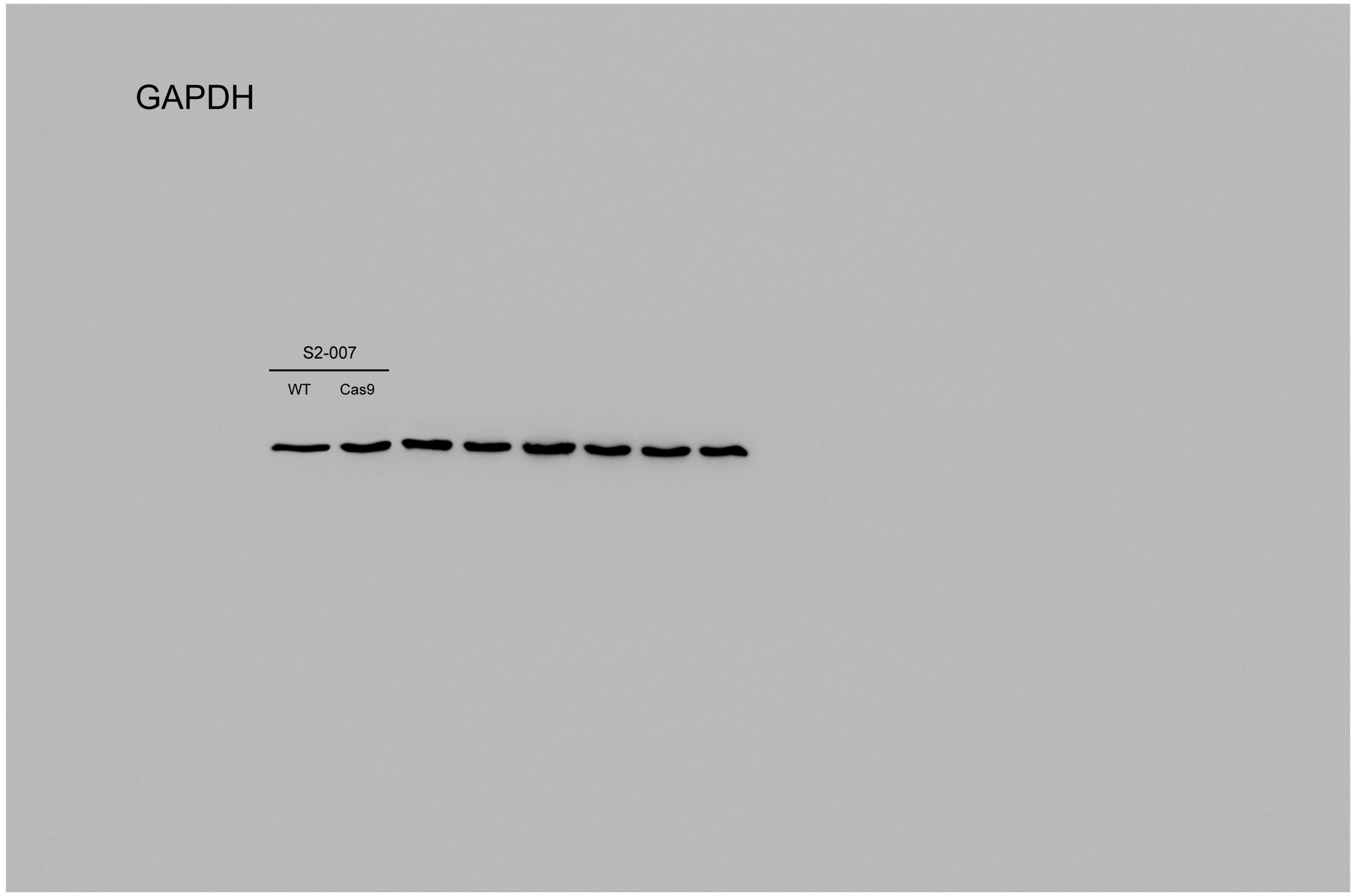

Original Western blots of data shown in Suppl. Fig. S3: MEN1

MEN1

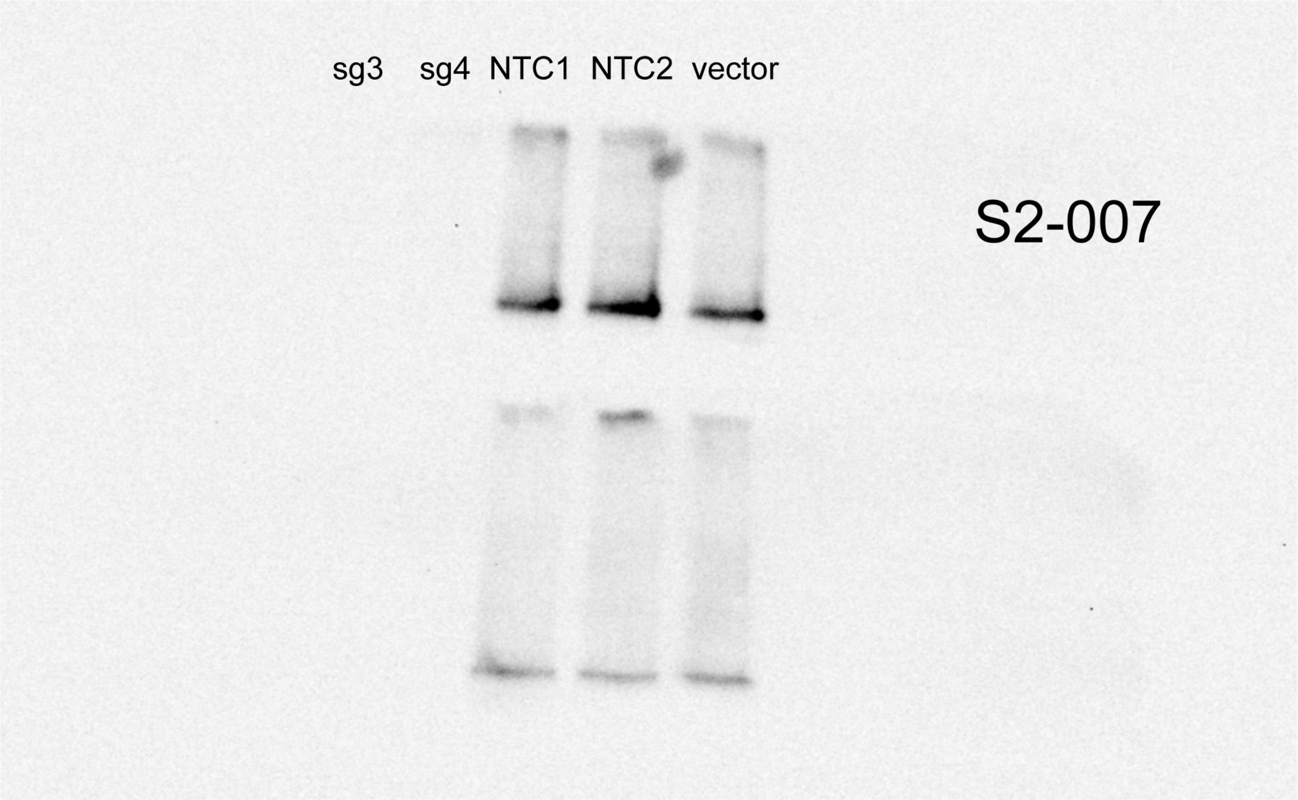

MEN1

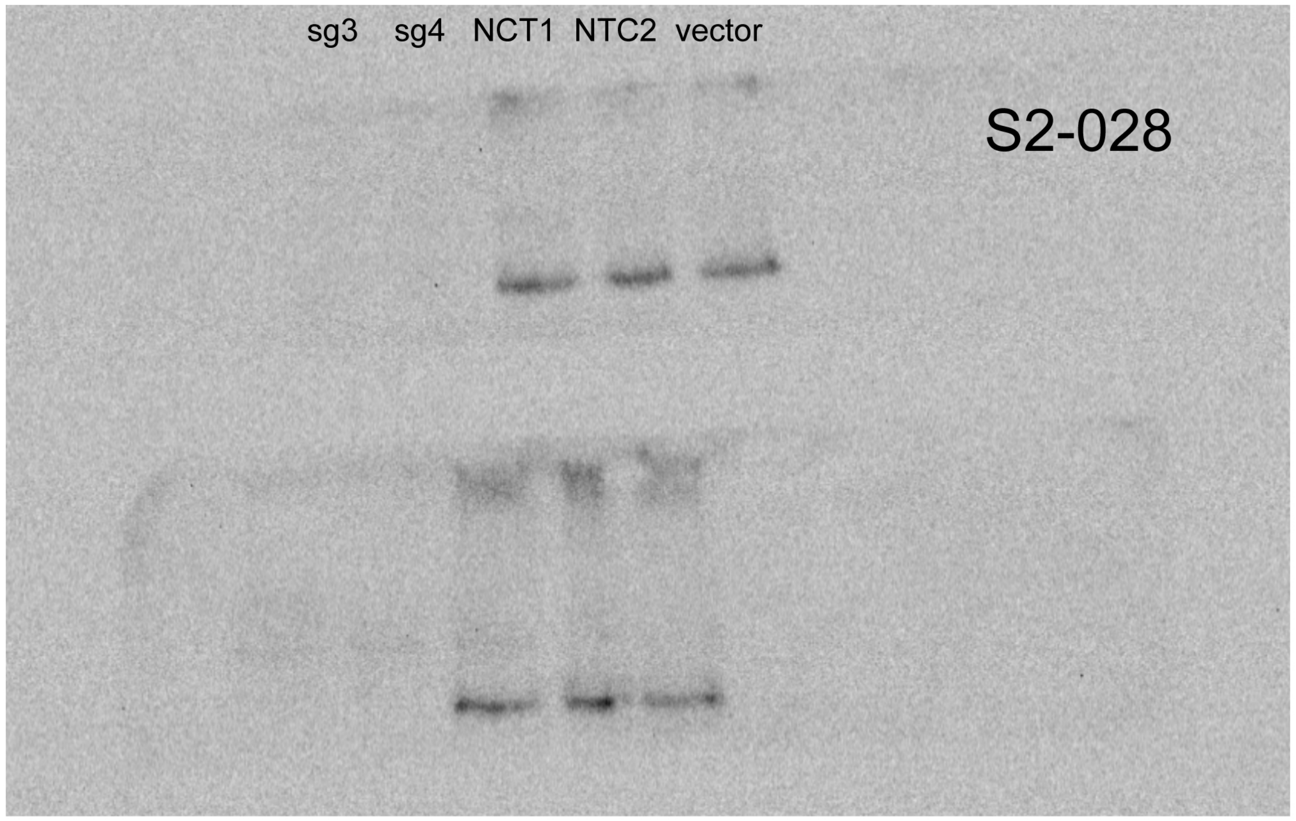

α-Tubulin

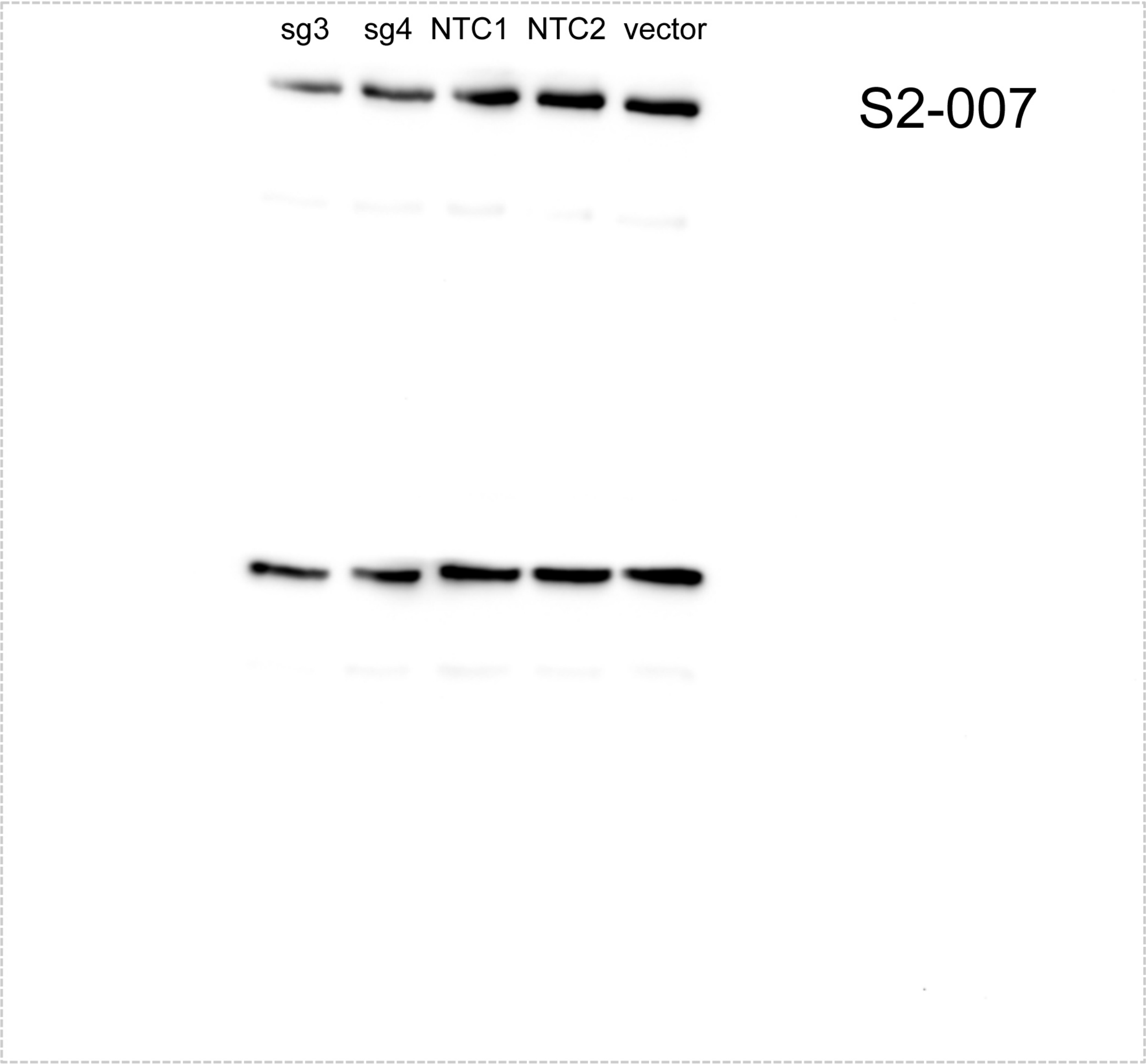

α-Tubulin

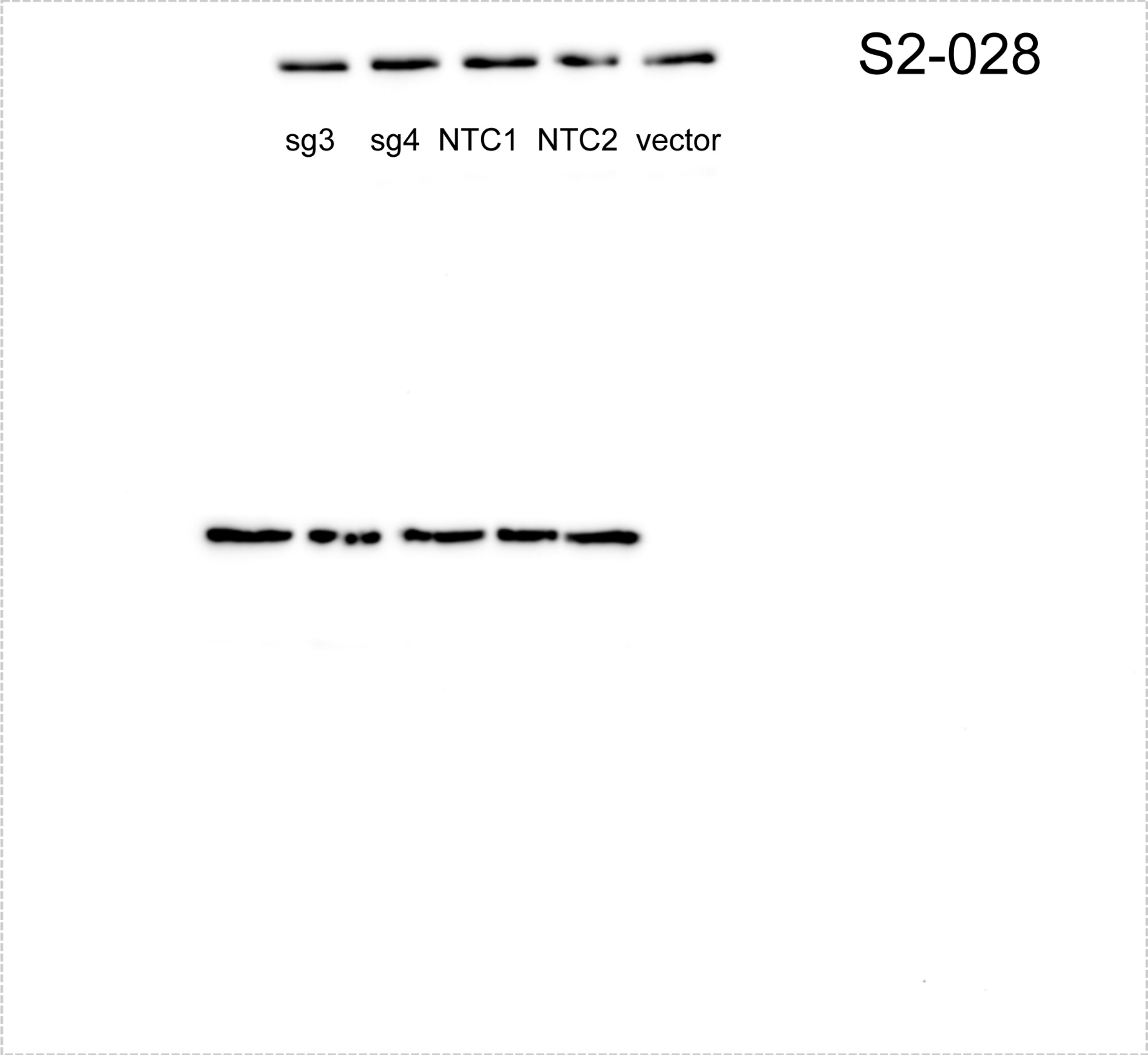

Original Western blots of data shown in Suppl. Fig. S3: MYBL2

MYBL2

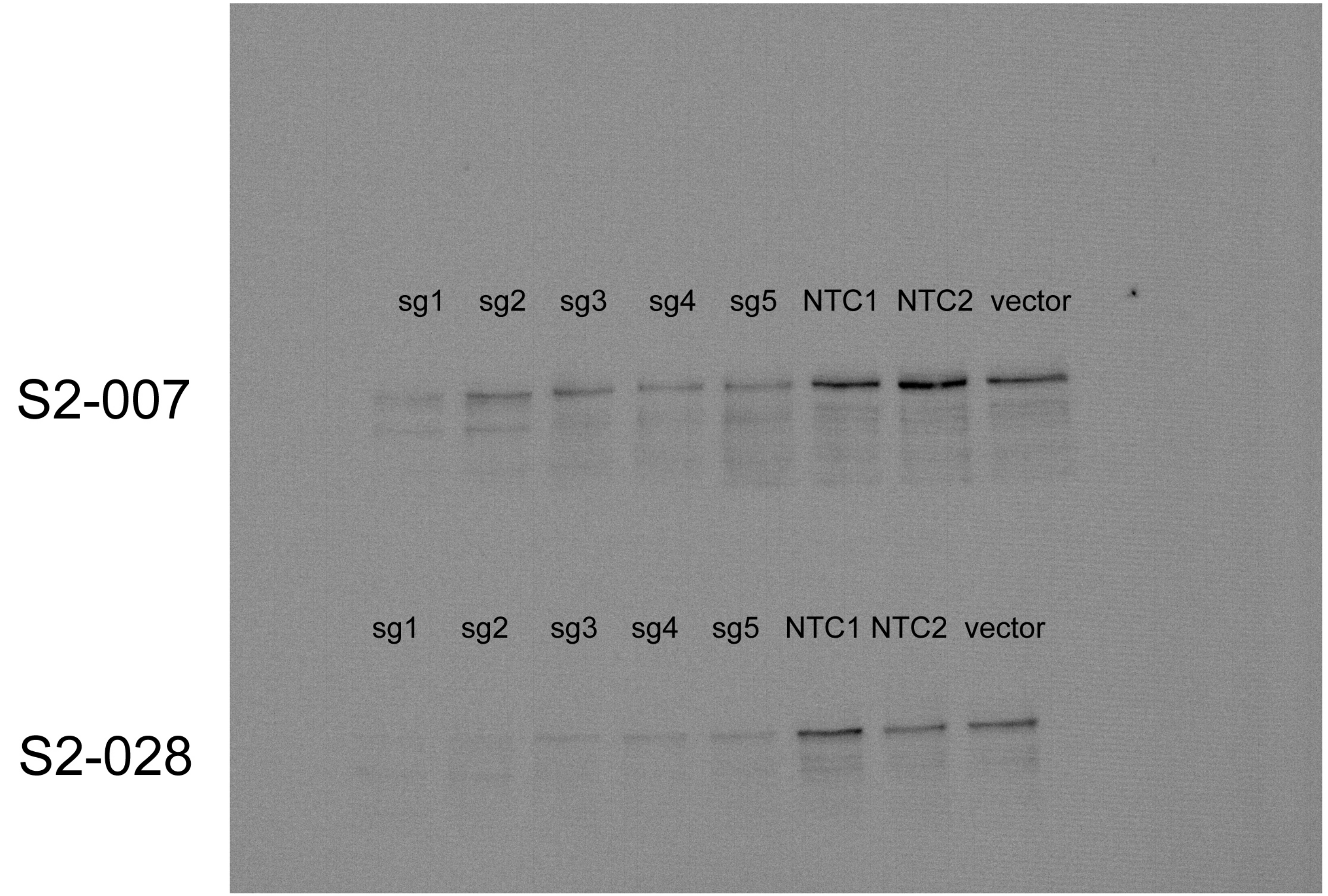

α-Tubulin

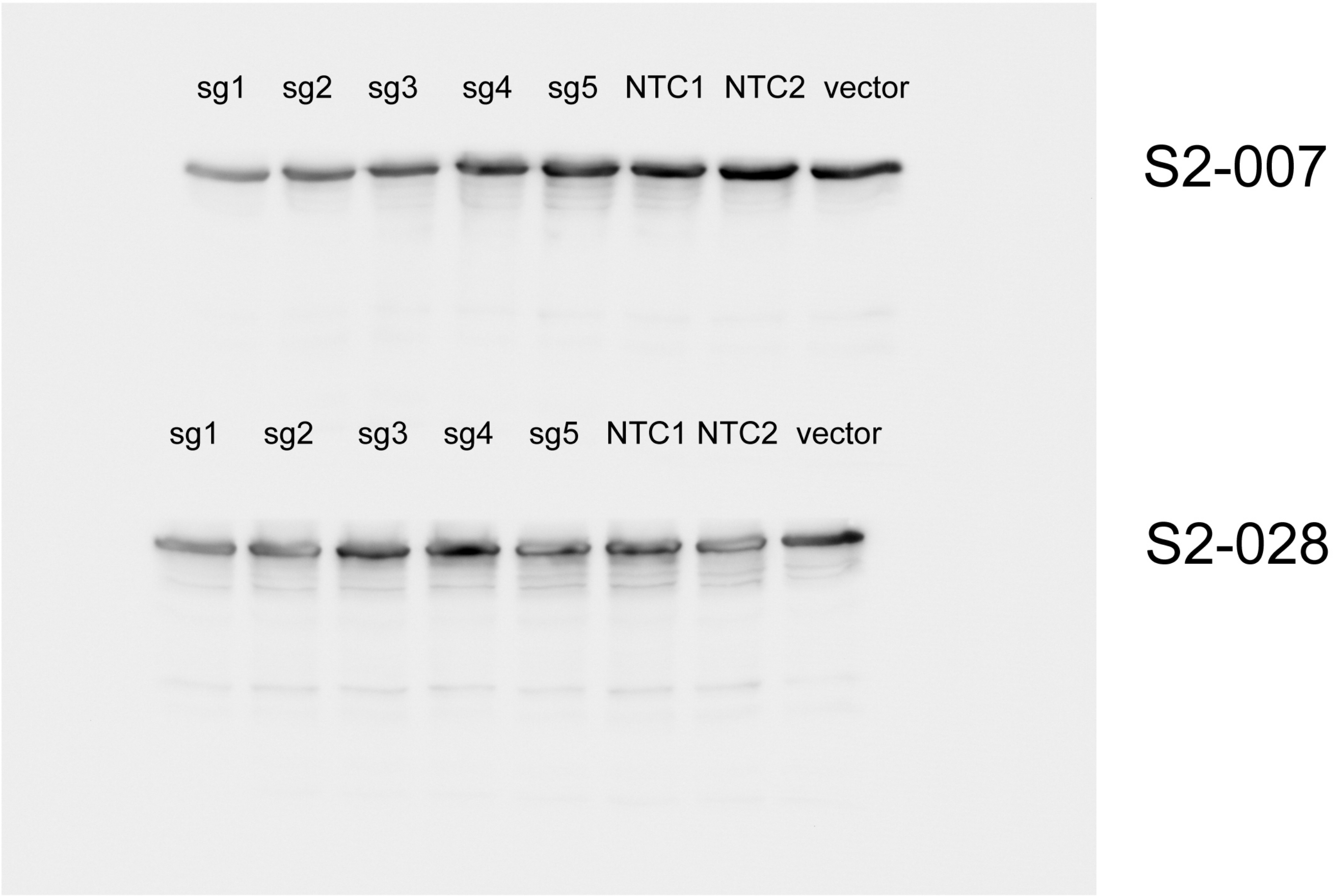

Original Western blots of data shown in Suppl. Fig. S3: TYMS

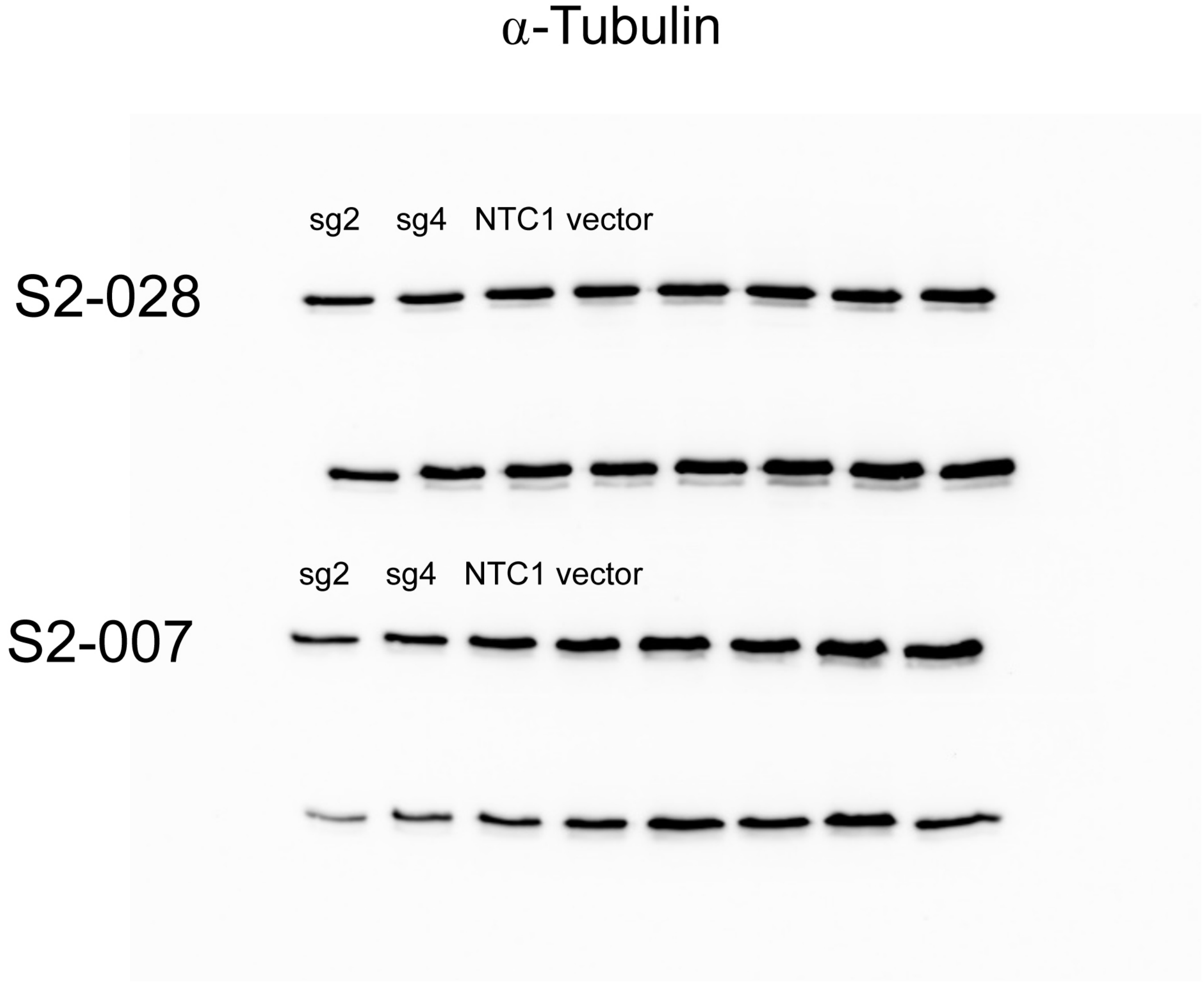

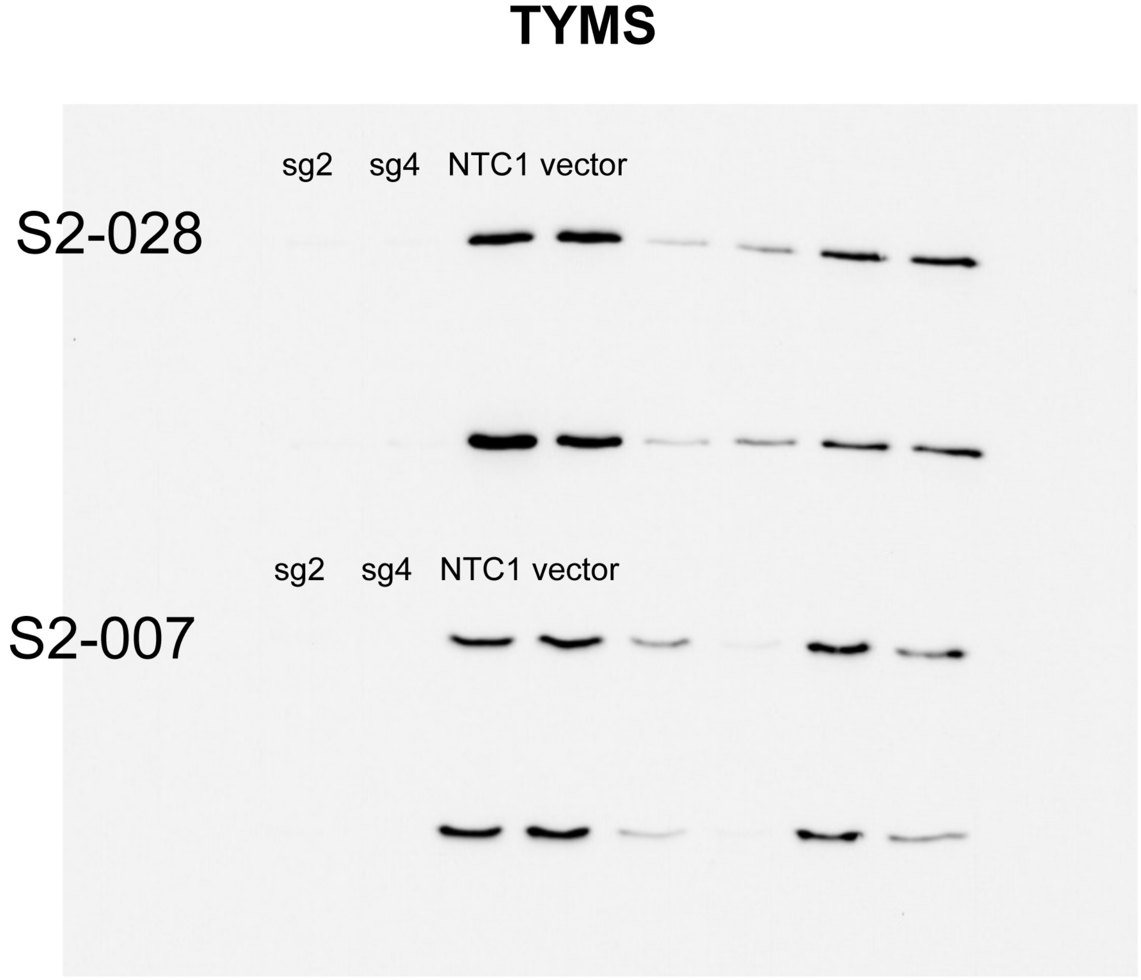

Original Western blots of data shown in Suppl. Fig. S5: FOXM1

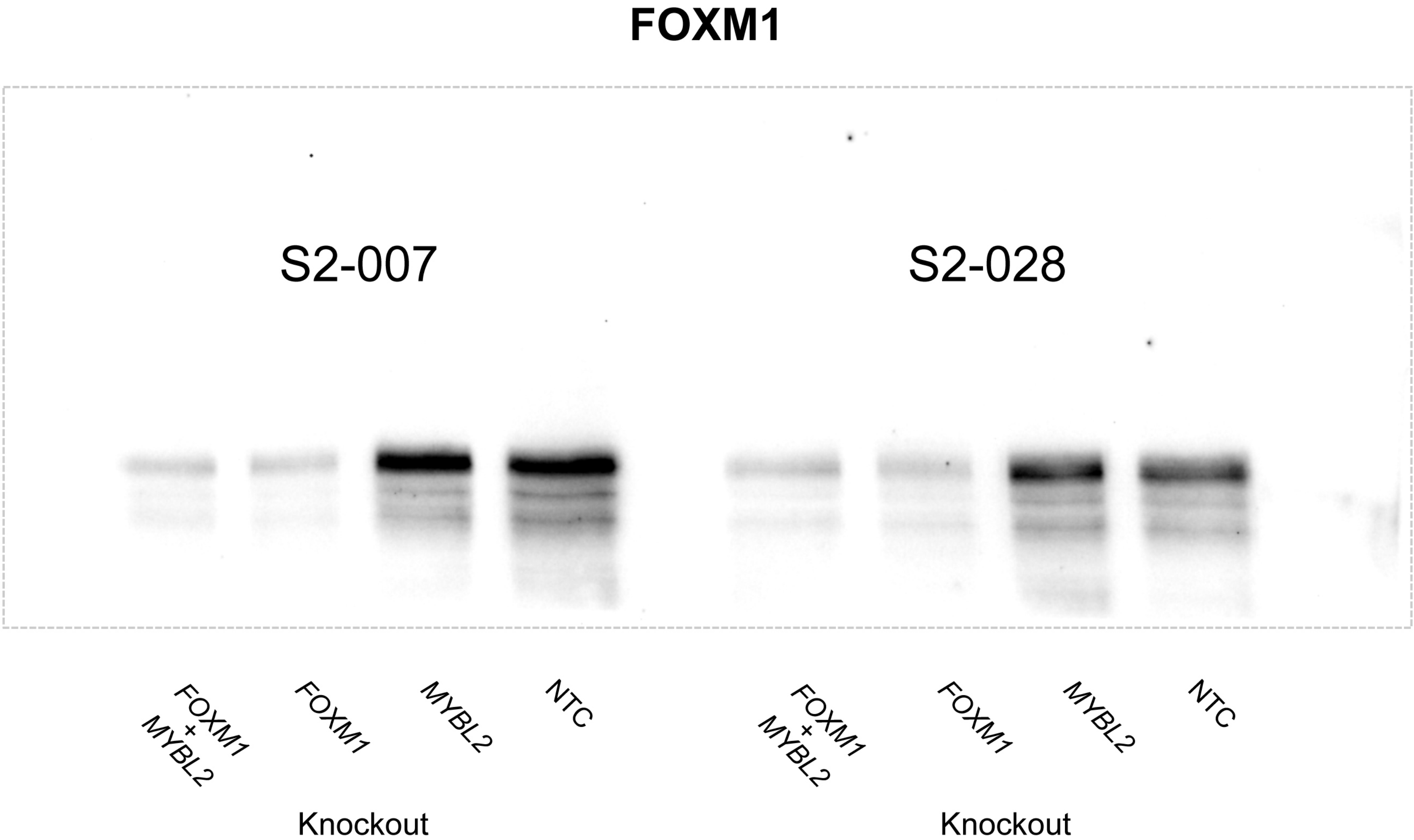

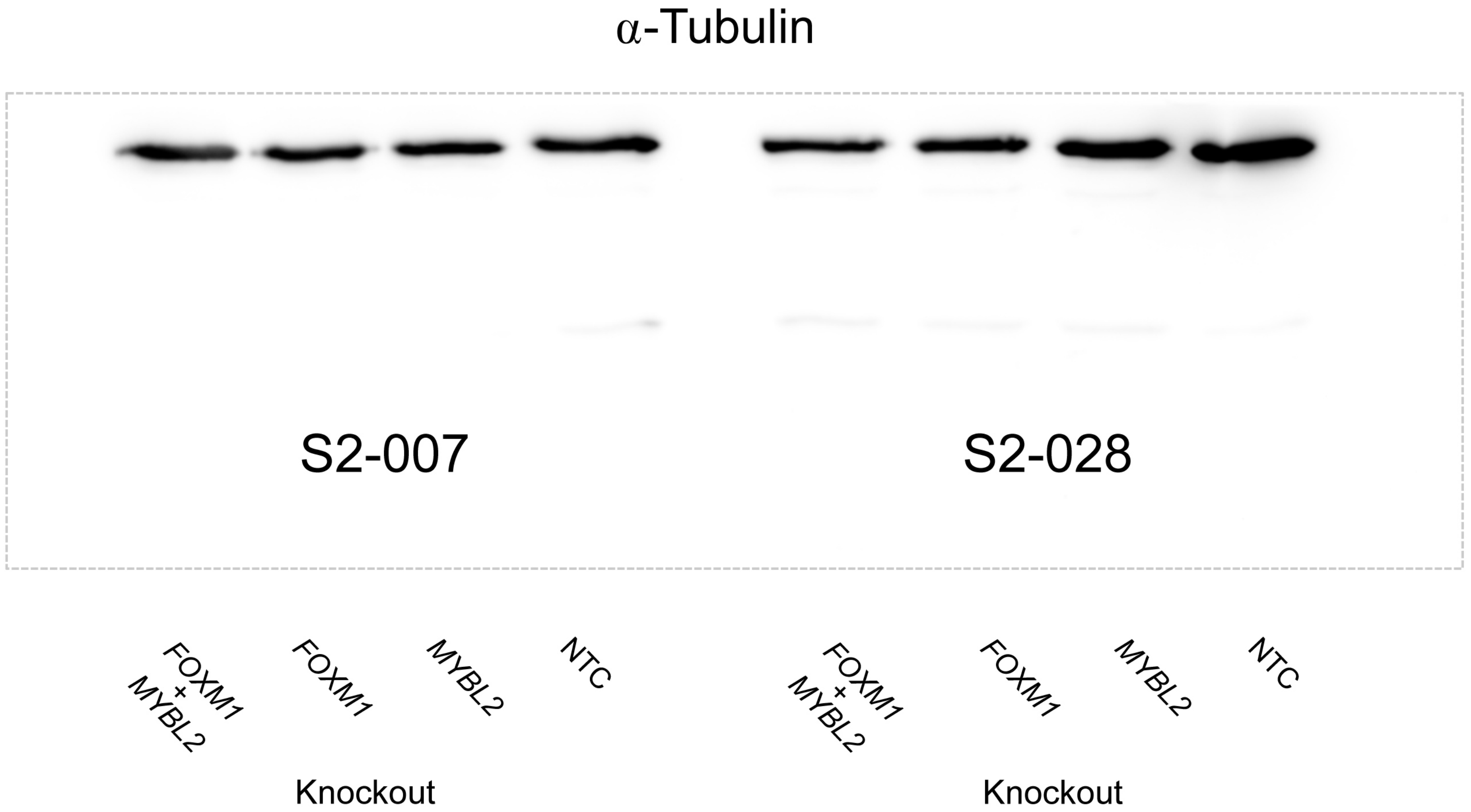

Original Western blots of data shown in Suppl. Fig. S5: MYBL2

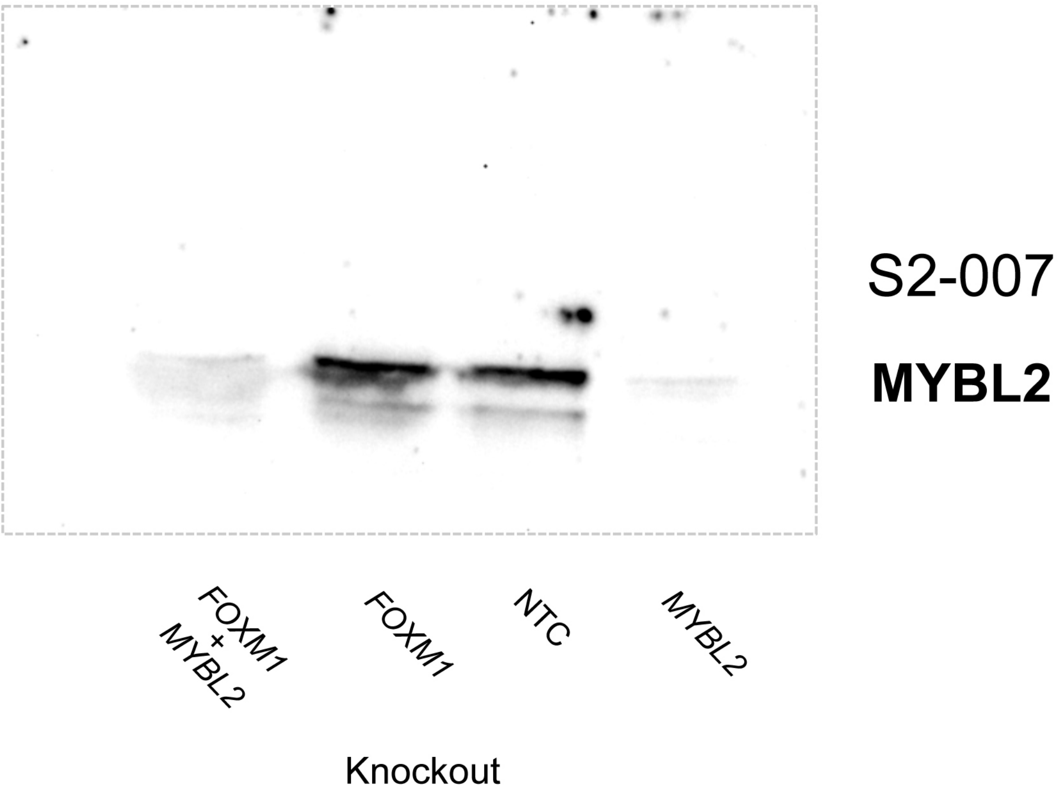

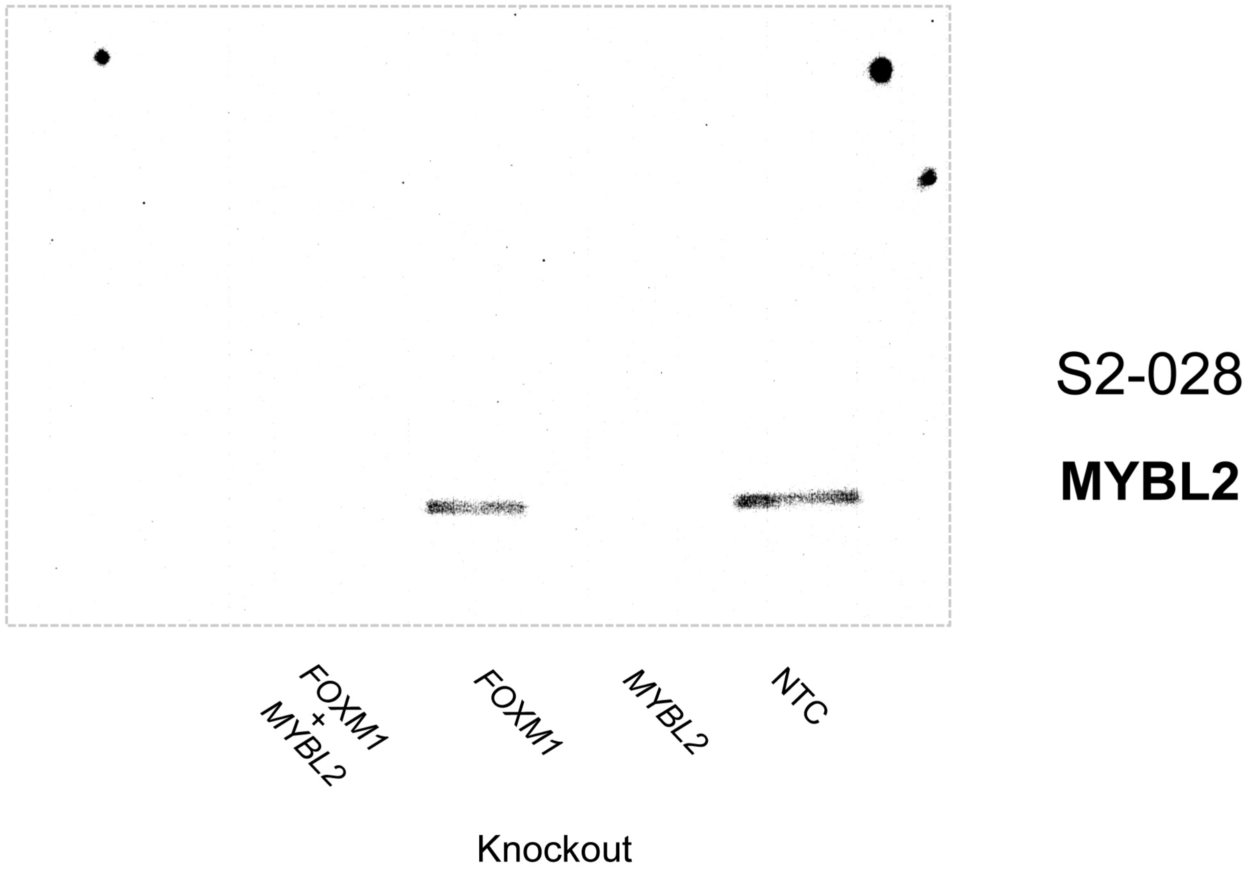

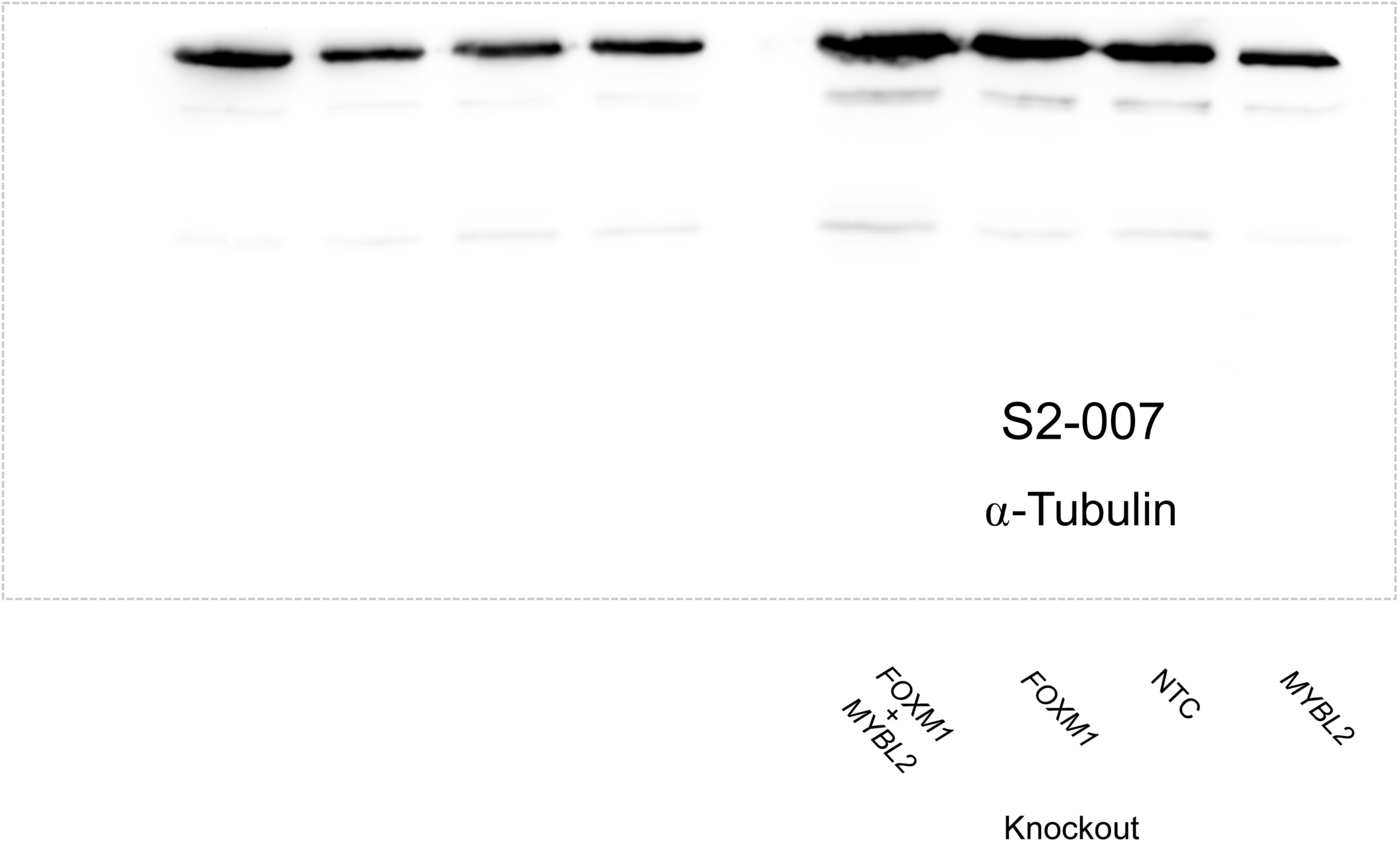

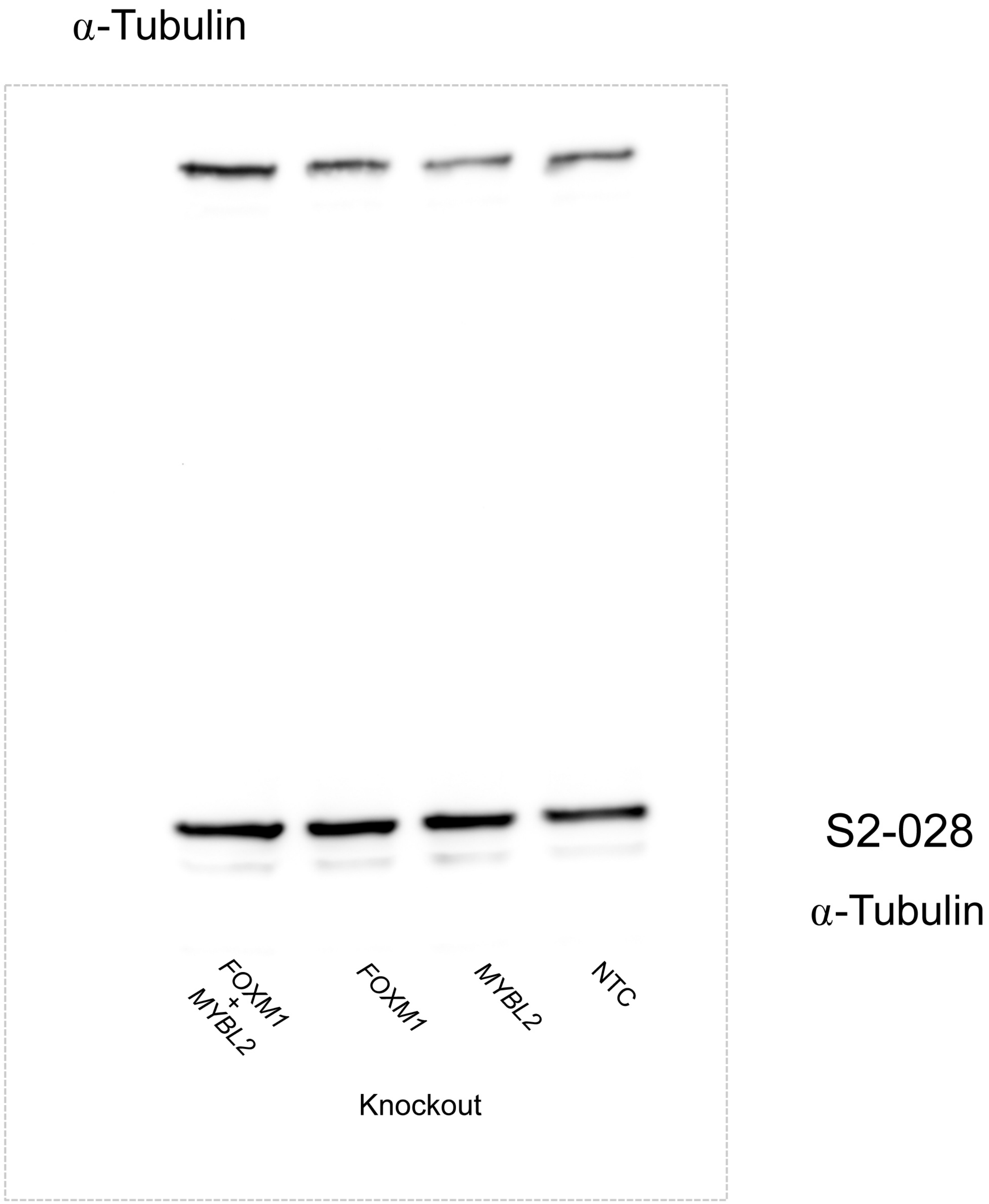

SUPPLEMENTARY TABLES

Supplementary Table S1. Sequences of sgRNAs for validation and double transduction.

Construct name		sgRNA sequence
sgRNAs for validation		
EPCAM	Forward:	5’ - GATCCTGACTGCGATGAGAG - 3’
	Reverse:	5’ - CTCTCATCGCAGTCAGGATC - 3’
*TYMS*_sg1	Forward:	5’ - GTCAGGAAGGACGACCGCAC - 3’
	Reverse:	5’ - GTGCGGTCGTCCTTCCTGAC - 3’
*TYMS*_sg2	Forward:	5’ - CACGGGCACCGGCACCCTGT - 3’
	Reverse:	5’ - ACAGGGTGCCGGTGCCCGTG - 3’
*TYMS*_sg3	Forward:	5’ - ACCGGCACCCTGTCGGTATT - 3’
	Reverse:	5’ - AATACCGACAGGGTGCCGGT - 3’
*TYMS*_sg4	Forward:	5’ - CTGCATGCCGAATACCGACA - 3’
	Reverse:	5’ - TGTCGGTATTCGGCATGCAG - 3’
*TYMS*_sg5	Forward:	5’ - CCTGTCGGTATTCGGCATGC - 3’
	Reverse:	5’ - GCATGCCGAATACCGACAGG - 3’
*MEN1*_sg1	Forward:	5’ - CACACCGGAGCTGTCCAATT - 3’
	Reverse:	5’ - AATTGGACAGCTCCGGTGTG - 3’
*MEN1*_sg2	Forward:	5’ - CACCCCCTTCTCGAGGATAG - 3’
	Reverse:	5’ - CTATCCTCGAGAAGGGGGTG - 3’
*MEN1*_sg3	Forward:	5’ - CTGTCCCTCTATCCTCGAGA - 3’
	Reverse:	5’ - TCTCGAGGATAGAGGGACAG - 3’
*MEN1*_sg4	Forward:	5’ - CAGGTCGGCCACGGGAAAGT - 3’
	Reverse:	5’ - ACTTTCCCGTGGCCGACCTG - 3’
*MEN1*_sg5	Forward:	5’ - CAGGCGCACCACGTCGTCGA - 3’
	Reverse:	5’ - TCGACGACGTGGTGCGCCTG - 3’
*MYBL2*_sg1	Forward:	5’ - CTGACCAGCAATGCCAGTAC - 3’
	Reverse:	5’ - GTACTGGCATTGCTGGTCAG - 3’
*MYBL2*_sg2	Forward:	5’ - TGAATCCAGACCTTGTCAAG - 3’
	Reverse:	5’ - CTTGACAAGGTCTGGATTCA - 3’
*MYBL2*_sg3	Forward:	5’ - ATCGAGCTGGTTAAGAAGTA - 3’
	Reverse:	5’ - TACTTCTTAACCAGCTCGAT - 3’
*MYBL2*_sg4	Forward:	5’ - GGTGGTTGTGCCAGCGTTCA - 3’
	Reverse:	5’ - TGAACGCTGGCACAACCACC - 3’
*MYBL2*_sg5	Forward:	5’ - CAGGCGCACCACGTCGTCGA - 3’
	Reverse:	5’ - CGGTCCAGCAAGACTTCTTC - 3’
NTC1	Forward:	5’ - AACTTCTTAAGCCAACTGAC - 3’
	Reverse:	5’ - GTCAGTTGGCTTAAGAAGTT - 3’
NTC2	Forward:	5’ - CGGTAAAGCCTACCGCAGAA - 3’
	Reverse:	5’ - TTCTGCGGTAGGCTTTACCG - 3’
sgRNAs for double transduction		
MYBL2	Forward:	5’ - CTGACCAGCAATGCCAGTAC - 3’
	Reverse:	5’ - GTACTGGCATTGCTGGTCAG - 3’
FOXM1	Forward:	5’ - CCTGGAGCAGCGACAGGTTA- 3’
	Reverse:	5’ - TAACCTGTCGCTGCTCCAGG- 3’
NTC	Forward:	5’ - AACTTCTTAAGCCAACTGAC - 3’
	Reverse:	5’ - GTCAGTTGGCTTAAGAAGTT - 3’

NTC, non-targeting control.

Supplementary Tables S2 to S7 are shown below. Upon request, they can also be provided as Excel-files. Because of their size and complexity, the table legends are all shown on this page as well as the respective starting page of each table.

Supplementary Table S2. Normalised sgRNA read-counts resulting from the CRISPR-Cas9 screen. The results are shown for all 259,900 constructs at t_{zero} and t_{end} in both S2-007 and S2-028. .... page 19

Supplementary Table S3. Summary of beta-scores for all genes as calculated from the screen data. For all 22,047 genes, the mean beta-score value is shown as calculated from all constructs of a particular gene. Also, the number of sgRNAs is provided. Next to values for both cell lines, also the respective beta-score of their comparison is shown. The continuous colour-code indicates a decrease in red, while yellow represents small or no changes; green stands for an increase. .... page 193

Supplementary Table S4. Genes for which significant changes in beta-score have been recorded in a comparison of S2-007 versus S2-028. Genes are shown for two significance levels: $p < 0.05$ and $p < 0.01$. Also the results of the KEGG enrichment analyses are shown. .... page 226

Supplementary Table S5. Drop-out values calculated from the CRISPR-Cas9 screen data for each sgRNA of the 67 genes that are related to less viability in S2-007 than S2-028 ($p < 0.01$). The actual read-counts and the related drop-out values are shown. The colour-code ranging from red (lowest value) via yellow to green (highest value) is only comparable for the constructs of each gene. .... page 237

Supplementary Table S6. Drop-out values calculated from the CRISPR-Cas9 screen data for *TYMS*, *MEN1* and *MYBL2*. The sgRNA construct, its sequence, its chromosomal position as well as the actual read-counts and the related drop-out values are shown. The colour-code ranging from red (lowest value) via yellow to green (highest value) is only comparable for the constructs of each gene. .... page 241

Supplementary Table S7. Comparison of drop-out values of knockouts of genes *CCNB1* and *MYBL2* as calculated from the CRISPR-Cas9 screen data. .... page 245

Supplementary Table S2. Normalised sgRNA read-counts resulting from the CRISPR-Cas9 screen. The results are shown for all 259,900 constructs at t_{zero} and t_{end} in both S2-007 and S2-028.

Data are shown on the pages below.

Suppl. Tab. S2. Normalized sgRNA read-counts in screen

sgRNA	Transcript	S2-007_baseline	S2-007_end	S2-028_baseline	S2-028_end
A1BG__NM_130786_AAAGAAGATGCGATCTGGGC	A1BG__NM_130786	587	710	771	945
A1BG__NM_130786_ACCTTTCCAGTCCATCAGCC	A1BG__NM_130786	375	472	429	485
A1BG__NM_130786_ACTTCCAGCTGTTCAAGAAT	A1BG__NM_130786	514	251	738	521
A1BG__NM_130786_AGGTGAAAGAAGATGCGATC	A1BG__NM_130786	274	294	275	276
A1BG__NM_130786_CAAGAGAAAGACCACGAGCA	A1BG__NM_130786	709	694	885	854
A1BG__NM_130786_CATCTTCTTTCACCTGAACG	A1BG__NM_130786	712	574	786	940
A1BG__NM_130786_CCATGGTGCATCAGCACAGG	A1BG__NM_130786	423	446	545	703
A1BG__NM_130786_CTTTCTCCCCGCGCCGTAGC	A1BG__NM_130786	57	126	118	131
A1BG__NM_130786_GGTGAAAGAAGATGCGATCT	A1BG__NM_130786	391	329	486	361
A1BG__NM_130786_GTGGAGTGGACTTCCAGCTA	A1BG__NM_130786	104	188	89	125
A1BG__NM_130786_TCAATGGTCACAGTAGCGCT	A1BG__NM_130786	112	138	115	126
A1BG__NM_130786_TGGACTTCCAGCTACGGCGC	A1BG__NM_130786	13	12	18	45
A1CF__NM_001198818_AACAGGAAGAATTCAGTTAT	A1CF__NM_001198818	315	360	423	388
A1CF__NM_001198818_ACAACTGCCGATTATTTGTT	A1CF__NM_001198818	1003	1131	1192	1318
A1CF__NM_001198818_ACAGGAAGAATTCAGTTATG	A1CF__NM_001198818	635	668	765	750
A1CF__NM_001198818_CAACTGCCGATTATTTGTTG	A1CF__NM_001198818	925	1362	1023	1244
A1CF__NM_001198818_CCAGCGCACAGGATATAGCT	A1CF__NM_001198818	194	265	194	280
A1CF__NM_001198818_CTCTGAAGAGATGATTGAAA	A1CF__NM_001198818	40	20	28	42
A1CF__NM_001198818_GAATTCAACAATATCAAACC	A1CF__NM_001198818	332	205	344	318
A1CF__NM_001198818_GAGATGAAAAAGGTTACTGA	A1CF__NM_001198818	398	427	354	492
A1CF__NM_001198818_GCAGAAATGGGCGCCTCTTA	A1CF__NM_001198818	803	596	1060	1165
A1CF__NM_001198818_TCAAATCACAAATCCGGGGA	A1CF__NM_001198818	261	333	369	504
A1CF__NM_001198818_TGCTAGGGTGACTTCAATGG	A1CF__NM_001198818	499	408	456	460
A1CF__NM_001198818_TGGATTGAGCGGCACTCAGA	A1CF__NM_001198818	95	168	109	52
A1CF__NM_001198819_AGTTATGTTAGGTATACCCG	A1CF__NM_001198819	225	165	253	309
A1CF__NM_001198819_GCAATTCCTGGCCTCAAAAA	A1CF__NM_001198819	243	457	173	260
A1CF__NM_001198819_TAGATGGAAGCAGTGTGTCT	A1CF__NM_001198819	376	325	444	448
A1CF__NM_138933_AAAAATGCAGAATGGACCAT	A1CF__NM_138933	492	491	472	477
A1CF__NM_138933_AAATTACACCATTCACTTGT	A1CF__NM_138933	462	451	426	653
A1CF__NM_138933_AACATACTCCCCAAACACTC	A1CF__NM_138933	264	240	291	394
A1CF__NM_138933_AAGCATCTCTGATTGCTAGA	A1CF__NM_138933	600	831	678	989
A1CF__NM_138933_ACAATGGTTGCACGTCAGGT	A1CF__NM_138933	819	554	726	754
A1CF__NM_138933_ACACTAGAATTTCCTTGGAT	A1CF__NM_138933	1329	1129	1447	1065
A1CF__NM_138933_AGATCCACAGGGCACTAGTA	A1CF__NM_138933	457	422	436	473
A1CF__NM_138933_AGGAGAAAAAGGTTAGTTAG	A1CF__NM_138933	277	326	219	240
A1CF__NM_138933_AGTACTGGGGTTATAGGCAT	A1CF__NM_138933	65	0	108	0
A1CF__NM_138933_ATAGGAGAAAAGTCTGTAAG	A1CF__NM_138933	132	98	133	166
A1CF__NM_138933_ATCATTGGGGACCGAGTTAG	A1CF__NM_138933	421	291	435	419
A1CF__NM_138933_ATCTTGTAGCTGGCAAGATT	A1CF__NM_138933	321	381	379	519
A1CF__NM_138933_ATGCCAAACAACACTATGTT	A1CF__NM_138933	205	338	275	363
A1CF__NM_138933_ATGTAGGAGTAACAGGTATT	A1CF__NM_138933	231	443	259	374
A1CF__NM_138933_ATTCACCATGGCTCACTGTG	A1CF__NM_138933	422	550	371	370
A1CF__NM_138933_ATTGACAATGGTTGCACGTC	A1CF__NM_138933	313	206	397	381
A1CF__NM_138933_CACAAATGATACTTTGTCTA	A1CF__NM_138933	812	1341	1000	1205
A1CF__NM_138933_CAGCCTAATGTTGCTTAAGT	A1CF__NM_138933	827	1178	960	1259
A1CF__NM_138933_CATTAAAAAATACTGATGCC	A1CF__NM_138933	382	675	507	640
A1CF__NM_138933_CATTATTATGCCAGGTTAGG	A1CF__NM_138933	62	107	115	77
A1CF__NM_138933_CCAGAGTTTGGCAATAATAG	A1CF__NM_138933	154	193	250	212
A1CF__NM_138933_CCTAATGTTGCTTAAGTAGG	A1CF__NM_138933	739	781	863	993
A1CF__NM_138933_CCTCACATCACGTCATATAA	A1CF__NM_138933	592	827	646	934
A1CF__NM_138933_CTGATCTGTGACAAATCGAC	A1CF__NM_138933	336	405	357	410
A1CF__NM_138933_CTTGCTCTATCTACCTAACT	A1CF__NM_138933	119	208	209	268
A1CF__NM_138933_CTTGGTCTGTAGAGATCCAC	A1CF__NM_138933	217	184	262	244
A1CF__NM_138933_GAAAGTGTAATGTATCTACC	A1CF__NM_138933	285	344	330	360
A1CF__NM_138933_GAAGAGGCCTAGGACTCTGG	A1CF__NM_138933	303	171	291	223
A1CF__NM_138933_GAGATCCACAGGGCACTAGT	A1CF__NM_138933	922	1025	1040	1112
A1CF__NM_138933_GAGATTGATGTAGGAGTAAC	A1CF__NM_138933	284	302	394	383
A1CF__NM_138933_GATACTTTGTCTAAGGGAAT	A1CF__NM_138933	112	99	173	218
A1CF__NM_138933_GATAGAAGTAACTCAACTCA	A1CF__NM_138933	242	158	317	340
A1CF__NM_138933_GCAATAATAGTGGAAACTTA	A1CF__NM_138933	172	300	229	441
A1CF__NM_138933_GCCAAACAACACTATGTTTG	A1CF__NM_138933	374	313	371	432
A1CF__NM_138933_GGACAGTCTTTCATGAGCTC	A1CF__NM_138933	25	82	20	23
A1CF__NM_138933_GGAGGAAATTGATCAGCAAT	A1CF__NM_138933	405	448	384	681
A1CF__NM_138933_GTATATGTAGGCTCATTTGT	A1CF__NM_138933	510	476	617	610
A1CF__NM_138933_GTCTATGTGGATTCTAACTG	A1CF__NM_138933	304	360	377	305
A1CF__NM_138933_GTGTGCCATTTGTAGAATAA	A1CF__NM_138933	239	356	316	523
A1CF__NM_138933_GTTTAAGCTGTTATCTGCTT	A1CF__NM_138933	1176	1187	1236	1543
A1CF__NM_138933_TAAGAACCTCCCTGTCTATG	A1CF__NM_138933	383	526	488	557
A1CF__NM_138933_TCATTATTAGTGAGAGTACA	A1CF__NM_138933	106	198	108	137
A1CF__NM_138933_TCCCAAAGTACTGGGGTTAT	A1CF__NM_138933	80	3	93	4
A1CF__NM_138933_TCTAGATATACTAATGCAGT	A1CF__NM_138933	127	110	145	109
A1CF__NM_138933_TCTATTCTCTTCACTAACAT	A1CF__NM_138933	129	283	184	217
A1CF__NM_138933_TCTGTGACAAATCGACTGGA	A1CF__NM_138933	383	382	374	384
A1CF__NM_138933_TGCTCTATCTACCTAACTTG	A1CF__NM_138933	277	247	457	273
A1CF__NM_138933_TGGAGGCTTTGTTATCTCAT	A1CF__NM_138933	383	360	404	471
A1CF__NM_138933_TGTCTAAACTGGGACACGTT	A1CF__NM_138933	611	622	716	672
A1CF__NM_138933_TTAAAACAATTGCATCATCC	A1CF__NM_138933	184	163	186	195
A1CF__NM_138933_TTCAATGTGCACCTAGGTTT	A1CF__NM_138933	375	407	421	432
A1CF__NM_138933_TTCTAGATATACTAATGCAG	A1CF__NM_138933	16	17	7	5
A1CF__NM_138933_TTCTCTGAAGAATAGATTTG	A1CF__NM_138933	5	2	14	12
A1CF__NM_138933_TTGCTCTATCTACCTAACTT	A1CF__NM_138933	115	123	200	155
A1CF__NM_138933_TTGGCATGATACCAGATTGG	A1CF__NM_138933	208	143	242	240
A1CF__NM_138933_TTGGCCAGTTGTGGTGGCGT	A1CF__NM_138933	428	506	447	560
A1CF__NM_138933_TTTCAATGTGCACCTAGGTT	A1CF__NM_138933	199	240	229	354
A2M__NM_000014_AAGGAAATCGCATCGCACAA	A2M__NM_000014	309	503	383	380
A2M__NM_000014_CACAGTGATGGTTAAGAACG	A2M__NM_000014	207	179	245	270
A2M__NM_000014_CCCAAAGTCTTCATCCAATG	A2M__NM_000014	1093	1251	1224	1503
A2M__NM_000014_CTTGGGTTGGTCCTTTCACT	A2M__NM_000014	1604	1890	1802	1758
A2M__NM_000014_GACAAATCAATCTACAAACC	A2M__NM_000014	261	198	385	310
A2M__NM_000014_GACAGAGCACCCTTTCACCG	A2M__NM_000014	2693	2458	2807	2797
A2M__NM_000014_GATTCCACTAGTATACATTC	A2M__NM_000014	640	526	602	608
A2M__NM_000014_GCTTCCTTGGAGTCTGTCAG	A2M__NM_000014	899	1182	1089	1595
A2M__NM_000014_GGTTAAGAACGAGGACAGTC	A2M__NM_000014	106	125	142	285
A2M__NM_000014_TCACAGCGAAGGCGACACAG	A2M__NM_000014	1064	924	1078	1179
A2M__NM_000014_TCCCAGGCAGTATATGGTTC	A2M__NM_000014	463	561	500	647
A2M__NM_000014_TTCCTCACTGTCCAAGTGAA	A2M__NM_000014	27	10	14	11
A2ML1__NM_001282424_AACACCTCCACTGGGAAAAA	A2ML1__NM_001282424	183	216	177	180
A2ML1__NM_001282424_ACTGACCTTCACTTCGAGAC	A2ML1__NM_001282424	1082	1231	1192	1664
A2ML1__NM_001282424_AGATCACCAGGGAAGGATCA	A2ML1__NM_001282424	240	278	215	317
A2ML1__NM_001282424_AGGGGCCAGTCTCGAAGTGA	A2ML1__NM_001282424	771	1068	864	1093
A2ML1__NM_001282424_ATCATACTCAGCCACTTGAT	A2ML1__NM_001282424	698	855	835	783
A2ML1__NM_001282424_CACCGCCCGGAGCGCACACA	A2ML1__NM_001282424	1109	1331	1348	1412
A2ML1__NM_001282424_CACCTTTCTTCTTAGAGTTC	A2ML1__NM_001282424	537	586	705	1011
A2ML1__NM_001282424_CGAGATGTGCTTTGACAATC	A2ML1__NM_001282424	584	812	922	1159
A2ML1__NM_001282424_GAAAAATGGCATAGATCACC	A2ML1__NM_001282424	28	43	52	34
A2ML1__NM_001282424_TCAGCTACAACACCTCCACT	A2ML1__NM_001282424	1793	1664	2020	1770
A2ML1__NM_001282424_TCATACTCAGCCACTTGATA	A2ML1__NM_001282424	419	405	440	605
A2ML1__NM_001282424_TGGTCACTACCCCTATCAAG	A2ML1__NM_001282424	33	26	40	34
A2ML1__NM_144670_AAACAAACCTTCTGAACGGA	A2ML1__NM_144670	331	343	294	390
A2ML1__NM_144670_AAATTTCCCCTCCGTTCAGA	A2ML1__NM_144670	340	583	407	467
A2ML1__NM_144670_AACTCCCGACACCCGGATTG	A2ML1__NM_144670	1645	1697	1985	1588
A2ML1__NM_144670_ACAAACCTCTCTACACCCCA	A2ML1__NM_144670	1247	1294	1255	1160
A2ML1__NM_144670_AGTGGCCACAATCCGGGTGT	A2ML1__NM_144670	805	1252	955	866
A2ML1__NM_144670_CAAACAAACCTTCTGAACGG	A2ML1__NM_144670	726	1048	932	1107
A2ML1__NM_144670_CAGAAGTTGCTAGAATACTC	A2ML1__NM_144670	264	200	312	338
A2ML1__NM_144670_GAACGGAGGGGAAATTTAGC	A2ML1__NM_144670	183	296	221	129
A2ML1__NM_144670_GCCGGGCTGGTAATGTCACC	A2ML1__NM_144670	1383	1307	1436	1300
A2ML1__NM_144670_GTACAGTGATGTTAAATTCA	A2ML1__NM_144670	283	462	293	390
A2ML1__NM_144670_GTACTCCATGGTGGAACTAC	A2ML1__NM_144670	178	97	147	107
A2ML1__NM_144670_TGTTAAATTCACGGTTACTC	A2ML1__NM_144670	59	121	108	126
A3GALT2__NM_001080438_AAAGAGCCATCCCAAATAAT	A3GALT2__NM_001080438	113	213	178	138
A3GALT2__NM_001080438_CCTGGGGGGCTCCCATTATT	A3GALT2__NM_001080438	1070	1099	1391	1113
A3GALT2__NM_001080438_CGAATGCGCGTCGCGTTCGA	A3GALT2__NM_001080438	305	475	490	605
A3GALT2__NM_001080438_CGGCAGATCCTACTTACACT	A3GALT2__NM_001080438	1142	1168	1360	1230
A3GALT2__NM_001080438_CTGGGGGGCTCCCATTATTT	A3GALT2__NM_001080438	535	485	642	562
A3GALT2__NM_001080438_GAATGCGCGTCGCGTTCGAA	A3GALT2__NM_001080438	257	376	362	569
A3GALT2__NM_001080438_GCAGCAGCCACGACGGCCAG	A3GALT2__NM_001080438	519	466	621	384
A3GALT2__NM_001080438_GCGCTGGCAAGACGTGTCGA	A3GALT2__NM_001080438	1364	1888	1581	2223
A3GALT2__NM_001080438_GCGGAGCAGCACTTCATGGC	A3GALT2__NM_001080438	1018	1028	1303	991
A3GALT2__NM_001080438_GGTACTTCTCCAGGTATCTA	A3GALT2__NM_001080438	327	447	322	366
A3GALT2__NM_001080438_TCCCTCCTTGAGAGCCATAT	A3GALT2__NM_001080438	390	330	445	496
A3GALT2__NM_001080438_TTCTGACCTGTACCCCCTGG	A3GALT2__NM_001080438	9	19	42	33
A4GALT__NM_017436_ACCCCTGGCCGACTGGTACG	A4GALT__NM_017436	1676	2265	1954	2270
A4GALT__NM_017436_ATGATCTACTGGCACGTTGT	A4GALT__NM_017436	190	230	217	90
A4GALT__NM_017436_CATGATCTACTGGCACGTTG	A4GALT__NM_017436	1078	637	1112	915
A4GALT__NM_017436_CCTCCAGGATCGCACTCATG	A4GALT__NM_017436	2099	1586	2398	1701
A4GALT__NM_017436_CTTCCACATGAGTGCGATCC	A4GALT__NM_017436	635	712	703	817
A4GALT__NM_017436_GCGGAACCTGACCAACGTGC	A4GALT__NM_017436	487	629	391	685
A4GALT__NM_017436_GGACCAGCACGTGGGATTCG	A4GALT__NM_017436	952	1037	950	979
A4GALT__NM_017436_GGATTCGGGGTGAGTTCTGG	A4GALT__NM_017436	551	571	606	626
A4GALT__NM_017436_GGGTGCCCAGCACGTTGGTC	A4GALT__NM_017436	832	1246	905	1323
A4GALT__NM_017436_GGGTGGGGTGGGGGGTGTCA	A4GALT__NM_017436	134	89	121	155
A4GALT__NM_017436_TCACCCCGAATCCCACGTGC	A4GALT__NM_017436	1981	1695	2141	1476
A4GALT__NM_017436_TCGTCTCCATCATGATCTAC	A4GALT__NM_017436	822	964	971	903
A4GNT__NM_016161_AAATCTTTGGGGGTGTAAGA	A4GNT__NM_016161	24	26	44	30
A4GNT__NM_016161_ACCCCATCTCCTATCGAGAG	A4GNT__NM_016161	729	977	781	1086
A4GNT__NM_016161_CAAGCAGCAAGGTGACTGAC	A4GNT__NM_016161	0	0	7	9
A4GNT__NM_016161_CACTATAATTCAGCCATTTG	A4GNT__NM_016161	452	676	529	628
A4GNT__NM_016161_CCCTGGTGGGACTTGAAAGA	A4GNT__NM_016161	129	206	155	169
A4GNT__NM_016161_GAGATGGGGTAAAATCTTTG	A4GNT__NM_016161	355	357	357	377
A4GNT__NM_016161_GATTTATCCTGAGTGGCCTG	A4GNT__NM_016161	329	426	364	447
A4GNT__NM_016161_GCCATCATCTGGAAATACGG	A4GNT__NM_016161	1050	969	1077	860
A4GNT__NM_016161_GCTTCTCGGTACTCTAGTAA	A4GNT__NM_016161	340	381	408	580
A4GNT__NM_016161_GGCAGCAGACTCTACGGAAC	A4GNT__NM_016161	48	54	102	91
A4GNT__NM_016161_GTTGTCTATTGCTGACAGGA	A4GNT__NM_016161	252	276	354	387
A4GNT__NM_016161_TCCATGCATTCCCACAAAAA	A4GNT__NM_016161	286	353	220	235
AAAS__NM_001173466_AATTTGCCCAAGTCACAAAT	AAAS__NM_001173466	231	339	199	309
AAAS__NM_001173466_AGATGCATCAACATTTGGTG	AAAS__NM_001173466	94	54	118	68
AAAS__NM_001173466_AGCCAAGACAGAGGCACTAA	AAAS__NM_001173466	721	548	712	460
AAAS__NM_001173466_AGGGAGGGGACTATGGTGCT	AAAS__NM_001173466	378	379	478	325
AAAS__NM_001173466_ATATGAGCACAATAACGAGC	AAAS__NM_001173466	418	256	351	259
AAAS__NM_001173466_CTCGTTATTGTGCTCATATA	AAAS__NM_001173466	379	182	314	195
AAAS__NM_001173466_GAGTGGGTGAAGACGGCATC	AAAS__NM_001173466	147	84	175	122
AAAS__NM_001173466_GCTCGTTATTGTGCTCATAT	AAAS__NM_001173466	828	378	770	404
AAAS__NM_001173466_GGGAGGGGACTATGGTGCTA	AAAS__NM_001173466	460	437	534	448
AAAS__NM_001173466_GGGCCCTGGCACTCTGTCGA	AAAS__NM_001173466	1338	1806	1437	1801
AAAS__NM_001173466_TCCTTTGTCAGTTGTAGGAC	AAAS__NM_001173466	73	21	86	77
AAAS__NM_001173466_TTTCATTTAGCACCCCAAAA	AAAS__NM_001173466	25	20	35	43
AAAS__NM_015665_TCCGTGTGTATAATGCCAGC	AAAS__NM_015665	398	417	445	399
AACS__NM_023928_AATATGACTCAACGGACCAA	AACS__NM_023928	96	189	133	102
AACS__NM_023928_ACTGTTGGGTAAATAACCTG	AACS__NM_023928	420	371	465	523
AACS__NM_023928_ATGCTGGCTGCGGCAAGCAT	AACS__NM_023928	253	175	236	254
AACS__NM_023928_CAACAGTGAGCACGCTGTCG	AACS__NM_023928	1729	1507	1907	1401
AACS__NM_023928_CCAAGGAGGAGCGCCCCGGT	AACS__NM_023928	1544	1274	1778	1339
AACS__NM_023928_CGACTGCCTTTGAACCACTC	AACS__NM_023928	1098	956	1173	1034
AACS__NM_023928_CTCACATTCACACCGAAGTC	AACS__NM_023928	319	505	404	404
AACS__NM_023928_CTTCTCACGTGTGTATGATG	AACS__NM_023928	334	350	447	472
AACS__NM_023928_GACTGCCTTTGAACCACTCG	AACS__NM_023928	594	561	668	521
AACS__NM_023928_GATGTCCCCGAGTGGTTCAA	AACS__NM_023928	95	43	93	35
AACS__NM_023928_TAGGTTGTGGACACATCGAA	AACS__NM_023928	60	40	45	17
AACS__NM_023928_TCACATTCACACCGAAGTCC	AACS__NM_023928	585	702	647	546
AADAC__NM_001086_AGCTCAGAACTGCCATAATT	AADAC__NM_001086	998	1138	1072	1132
AADAC__NM_001086_ATCCAGGGTTCCTAGATGTG	AADAC__NM_001086	552	325	708	392
AADAC__NM_001086_ATGGATTCCTTTAAGGTTGT	AADAC__NM_001086	241	248	251	283
AADAC__NM_001086_ATGGCTCCTCAACGTTATCT	AADAC__NM_001086	229	199	232	137
AADAC__NM_001086_CAGAAATACCGATTCTCTCA	AADAC__NM_001086	1193	1063	1368	1282
[illegible]	[illegible]	[illegible]	[illegible]	[illegible]	[illegible]

ABLIM2__NM_032432_GGAGGAACGCAGCGCATGGA	ABLIM2__NM_032432	705	655	727	823
ABLIM2__NM_032432_GGCGCTAAGGAAACGGAGTA	ABLIM2__NM_032432	428	588	398	488
ABLIM2__NM_032432_GTGAGAAGCCGAGTGCGAAA	ABLIM2__NM_032432	564	603	717	923
ABLIM2__NM_032432_GTTGCTGACACCATCATCGG	ABLIM2__NM_032432	818	901	764	728
ABLIM2__NM_032432_TCTGGGACAGTGCCAAGAGT	ABLIM2__NM_032432	366	262	389	346
ABLIM2__NM_032432_TGAGAAGCCGAGTGCGAAAC	ABLIM2__NM_032432	239	293	302	392
ABLIM2__NM_032432_TGGCTGTGTCGGGCATCAGA	ABLIM2__NM_032432	33	30	43	61
ABLIM2__NM_032432_TGTCATGGGCGCTAAGGAAA	ABLIM2__NM_032432	216	198	264	182
ABLIM2__NM_032432_TTGGCGTGTCATGGGCGCTA	ABLIM2__NM_032432	223	358	264	325
ABLIM2__NM_032432_TTTACAAAGCGTTCTTTAGA	ABLIM2__NM_032432	111	154	105	109
ABLIM3__NM_014945_AAGGTCACCTTGTCTCCAAT	ABLIM3__NM_014945	870	1097	874	873
ABLIM3__NM_014945_AGCAGAATCCTTACAATCCA	ABLIM3__NM_014945	397	443	545	452
ABLIM3__NM_014945_AGCGGGTGCCATAGAGTTGC	ABLIM3__NM_014945	742	558	717	879
ABLIM3__NM_014945_AGCTGCCGGGACTTCATCAC	ABLIM3__NM_014945	1962	2010	2113	2085
ABLIM3__NM_014945_ATCAGATGCTTCACCTGTCA	ABLIM3__NM_014945	347	450	443	466
ABLIM3__NM_014945_CAGAATCCTTACAATCCACG	ABLIM3__NM_014945	712	712	919	657
ABLIM3__NM_014945_CAGGCCACAGCCACATACTT	ABLIM3__NM_014945	830	549	871	753
ABLIM3__NM_014945_GACAGGTGAAGCATCTGATG	ABLIM3__NM_014945	731	938	843	927
ABLIM3__NM_014945_GAGCTGCCCCGTGGATTGTA	ABLIM3__NM_014945	2399	3016	2670	3200
ABLIM3__NM_014945_GCAGAATCCTTACAATCCAC	ABLIM3__NM_014945	909	828	896	909
ABLIM3__NM_014945_GGAAGTGGTTGTTGTGCACG	ABLIM3__NM_014945	603	1104	662	953
ABLIM3__NM_014945_TTGTAAGGATTCTGCTGATA	ABLIM3__NM_014945	156	140	224	261
ABO__NM_020469_AATGTGCCCTCCCAGACAAT	ABO__NM_020469	679	395	831	529
ABO__NM_020469_ACATCCTCAACGAGCAGTTC	ABO__NM_020469	722	735	696	751
ABO__NM_020469_AGACCAAGACAAGCATTATT	ABO__NM_020469	700	723	813	827
ABO__NM_020469_CTTGGCTGGCTCCCATTGTC	ABO__NM_020469	935	777	1155	1271
ABO__NM_020469_GAGCCATGGCCGAGGTGTTG	ABO__NM_020469	592	617	712	806
ABO__NM_020469_GATGGTCTACCCCCAGTCAA	ABO__NM_020469	257	326	223	317
ABO__NM_020469_GGATCATAGGTCGAAGTGCG	ABO__NM_020469	267	213	145	239
ABO__NM_020469_GGCAAACACAGTTAACCCAA	ABO__NM_020469	307	259	316	387
ABO__NM_020469_GGCCAGCGTCCGCAACACCT	ABO__NM_020469	895	900	911	953
ABO__NM_020469_GGCCGAGGTGTTGCGGACGC	ABO__NM_020469	774	1139	1013	1273
ABO__NM_020469_TGAAGACATAGTAGTGGACA	ABO__NM_020469	281	257	442	356
ABO__NM_020469_TTCCTGGCATTAGACTTCTG	ABO__NM_020469	56	31	42	42
ABR__NM_001092_ACGCTTTGTACACACCGAGC	ABR__NM_001092	286	275	316	209
ABR__NM_001092_AGAGGAGGCGATAGGCTTGC	ABR__NM_001092	360	368	364	369
ABR__NM_001092_ATGTCCTCCAGCTCATGGTC	ABR__NM_001092	231	143	262	257
ABR__NM_001092_CGATAACTATAAAGTCGCTC	ABR__NM_001092	160	150	156	187
ABR__NM_001092_CTCCGAGTGACCCGGTCAAT	ABR__NM_001092	1721	1737	1767	1572
ABR__NM_001092_CTTCAGGGGTTTCATGGGCT	ABR__NM_001092	305	151	363	384
ABR__NM_001092_CTTCCATGGTGACAGACGTG	ABR__NM_001092	340	722	428	503
ABR__NM_001092_GCTCCGAGTGACCCGGTCAA	ABR__NM_001092	355	337	330	297
ABR__NM_001092_GTGCCCCAAGGTGCAACAGT	ABR__NM_001092	67	208	40	20
ABR__NM_001092_GTGGCCTTCAGGGGTTTCAT	ABR__NM_001092	88	114	119	105
ABR__NM_001092_TGGATCCTGAACGGGATTGT	ABR__NM_001092	55	97	52	86
ABR__NM_001092_TTCACCAGCTGTCGCGTCTG	ABR__NM_001092	56	36	76	127
ABR__NM_001159746_GATCCACAATCGGAATGGAA	ABR__NM_001159746	646	619	645	681
ABR__NM_001256847_ACACACTGCCGGACGATGTA	ABR__NM_001256847	552	694	624	717
ABR__NM_001256847_ATCTCGGCTGGTGAATTTCA	ABR__NM_001256847	183	173	167	210
ABR__NM_001256847_CACACACTGCCGGACGATGT	ABR__NM_001256847	1406	1525	1536	1669
ABR__NM_001256847_CCAAGGTGCCCTACATCGTC	ABR__NM_001256847	35	17	53	67
ABR__NM_001256847_CTTCAGGCTCATATCTCGGC	ABR__NM_001256847	691	833	834	942
ABR__NM_001256847_CTTCGGTGTGAAGATCAGCG	ABR__NM_001256847	113	85	101	141
ABR__NM_001256847_GATCAGCGTGGTGACGAAGT	ABR__NM_001256847	438	405	445	521
ABR__NM_001256847_GCAGCTGGACCCACAAACCG	ABR__NM_001256847	613	712	725	674
ABR__NM_001256847_GGACGTGATTGAGATGAACG	ABR__NM_001256847	430	543	561	623
ABR__NM_001256847_GGCAGGACGTCGGTCATGCC	ABR__NM_001256847	244	179	258	264
ABR__NM_001256847_GGTGGAGAAGAGGGGTATCG	ABR__NM_001256847	225	217	243	232
ABR__NM_001256847_TTCTTGGTCTCCACGGTTTG	ABR__NM_001256847	14	39	23	23
ABR__NM_001282149_ACAATCGGAATGGAAAGGTC	ABR__NM_001282149	160	244	244	332
ABR__NM_001282149_AGTCCGAGGACAGTAGGAAC	ABR__NM_001282149	197	143	129	222
ABR__NM_001282149_CTCTGACCTCTCGTAGTCCG	ABR__NM_001282149	248	347	262	468
ABR__NM_001282149_TACAGCAGAAGCGAATAATG	ABR__NM_001282149	48	41	36	63
ABR__NM_001282149_TACTGTCCTCGGACTACGAG	ABR__NM_001282149	69	72	125	59
ABR__NM_001282149_TGACCTTTCCATTCCGATTG	ABR__NM_001282149	308	344	386	448
ABR__NM_021962_ACGGACGAGTACGACGGAGA	ABR__NM_021962	590	677	733	611
ABR__NM_021962_AGAGGGTGTCGATCCAGGAC	ABR__NM_021962	361	416	333	283
ABR__NM_021962_GCTGTAGAGGGTGTCGATCC	ABR__NM_021962	834	841	911	928
ABR__NM_021962_TACGGGACGGACGAGTACGA	ABR__NM_021962	354	439	379	288
ABRA__NM_139166_AGCAAGACTTATGAGAGAGG	ABRA__NM_139166	301	368	300	348
ABRA__NM_139166_AGGCAGGCCCAGGAGCCTAC	ABRA__NM_139166	178	332	281	234
ABRA__NM_139166_CAGCATGGCTCCGGGCGAAA	ABRA__NM_139166	0	0	0	0
ABRA__NM_139166_CAGGCGGGGTGGCGACTTTG	ABRA__NM_139166	1643	2007	1977	2214
ABRA__NM_139166_GAAAGAGGTGTCCAAAACGG	ABRA__NM_139166	218	283	253	240
ABRA__NM_139166_GAGCTTTCTGGTGTGAAGTA	ABRA__NM_139166	144	121	187	210
ABRA__NM_139166_GGCTGTGCGTATCTTCCGGA	ABRA__NM_139166	1220	1431	1466	1659
ABRA__NM_139166_GTCAGCAAGACTTATGAGAG	ABRA__NM_139166	274	331	286	427
ABRA__NM_139166_GTGATTGGTTTAGGAGCTTG	ABRA__NM_139166	169	192	123	169
ABRA__NM_139166_TGAGCTTTCTGGTGTGAAGT	ABRA__NM_139166	305	391	362	472
ABRA__NM_139166_TGGCTGTGCGTATCTTCCGG	ABRA__NM_139166	84	10	168	157
ABRA__NM_139166_TGTGAAGTAGGGGGTGTGAT	ABRA__NM_139166	540	779	679	849
ABRACL__NM_021243_AAAGTTAAGCGTGAAATTTG	ABRACL__NM_021243	132	189	170	207
ABRACL__NM_021243_AGTGGAGGAAATTCATCGTT	ABRACL__NM_021243	1260	806	1374	1036
ABRACL__NM_021243_GAAAGTTAAGCGTGAAATTT	ABRACL__NM_021243	204	390	328	386
[illegible]					

ABT1__NM_013375_GGCCTGTGGCAGCAAGAAAC	ABT1__NM_013375	137	230	187	271
ABT1__NM_013375_GTGCCAGGTATTGTGTACCT	ABT1__NM_013375	298	0	243	2
ABT1__NM_013375_TCTTCTTGCGTCTCACGAAC	ABT1__NM_013375	180	453	140	259
ABT1__NM_013375_TGGCCCAGGTACACAATACC	ABT1__NM_013375	13	0	15	0
ABTB1__NM_032548_ATGGAAGGGCAAGAGTGTCG	ABTB1__NM_032548	480	582	564	622
ABTB1__NM_032548_CCTGCTCGTGTCCATCGGTC	ABTB1__NM_032548	1247	1158	1284	1197
ABTB1__NM_032548_CCTGGAGGCCAAGTGCGAGA	ABTB1__NM_032548	0	0	18	9
ABTB1__NM_032548_CCTGGCTCAGATGCTAGACG	ABTB1__NM_032548	774	743	880	729
ABTB1__NM_032548_CGCCTCCTCTATGGCGCTGT	ABTB1__NM_032548	165	253	214	331
ABTB1__NM_032548_CTCGGGGGGCAGGGCACAAT	ABTB1__NM_032548	417	315	476	571
ABTB1__NM_032548_GACGGACTCTATCCCGCTGG	ABTB1__NM_032548	1032	1298	1006	835
ABTB1__NM_032548_GCCCACCTTCTCAATGACCT	ABTB1__NM_032548	1657	2697	1935	2550
ABTB1__NM_032548_GGAGACGGACTCTATCCCGC	ABTB1__NM_032548	1097	1245	1092	896
ABTB1__NM_032548_TAAGCCAGGCACGTGTGTGA	ABTB1__NM_032548	995	1163	993	983
ABTB1__NM_032548_TGACCACTTCCGAGAGAGCG	ABTB1__NM_032548	690	668	663	790
ABTB1__NM_032548_TGTTGAAGCCGTCAGGACAA	ABTB1__NM_032548	64	101	96	97
ABTB1__NM_172027_AGGTACTATGCCTGCTTGTG	ABTB1__NM_172027	564	708	504	604
ABTB1__NM_172027_ATGCCCTGCTCTAGAAGCCT	ABTB1__NM_172027	367	640	458	579
ABTB1__NM_172027_ATGGTGAGCGCTGCCTCTAT	ABTB1__NM_172027	471	366	566	491
ABTB1__NM_172027_CCAGGACGCAGCGATGCACC	ABTB1__NM_172027	417	446	454	430
ABTB1__NM_172027_GCTTGTAATCGCGTAGAGCC	ABTB1__NM_172027	304	248	339	297
ABTB1__NM_172027_GGCTCTACGCGATTACAAGC	ABTB1__NM_172027	74	21	33	44
ABTB1__NM_172027_GGTCACTCAGTGCCCCATAG	ABTB1__NM_172027	0	0	13	1
ABTB1__NM_172027_TGCGAGGCCAACACCTTCGA	ABTB1__NM_172027	1195	1318	1564	1733
ABTB2__NM_145804_CCACAGCCCTGATGGCGGAG	ABTB2__NM_145804	348	475	492	681
ABTB2__NM_145804_CCGCAGGGCACTGATCCGCG	ABTB2__NM_145804	1568	1693	1699	2108
ABTB2__NM_145804_CGAGAAGAGGTCGGCCACTT	ABTB2__NM_145804	2442	3318	2929	3267
ABTB2__NM_145804_CGCCAAGTGCACCCGCTTTG	ABTB2__NM_145804	2301	3135	2469	3169
ABTB2__NM_145804_GACCTTGGACTCCGGGTATG	ABTB2__NM_145804	1007	1180	1027	1155
ABTB2__NM_145804_GCTGCCCCAGTTCTCCGCCG	ABTB2__NM_145804	2258	3306	2621	2976
ABTB2__NM_145804_GGACTCCGGGTATGGGGCCG	ABTB2__NM_145804	579	536	691	931
ABTB2__NM_145804_GGGAGATGGCTGCGTACTCG	ABTB2__NM_145804	959	1079	1202	1486
ABTB2__NM_145804_GGGGAGGGAGAGGACACCTA	ABTB2__NM_145804	220	67	203	245
ABTB2__NM_145804_GTCCTCCAAGTCCAACTCGC	ABTB2__NM_145804	431	396	551	671
ABTB2__NM_145804_GTGGCCCGGGTGCTCCGCAA	ABTB2__NM_145804	144	220	180	160
ABTB2__NM_145804_TGCGGCCTCACCTTCTCAGT	ABTB2__NM_145804	4891	4992	5489	4059
ACAA1__NM_001130410_ACCTCAGAAACTGGGCGATT	ACAA1__NM_001130410	560	453	583	706
ACAA1__NM_001130410_AGTCATGACCGCGGTTCTCA	ACAA1__NM_001130410	649	492	632	400
ACAA1__NM_001130410_ATGACATTGGCATGGCCTGT	ACAA1__NM_001130410	276	157	298	135
ACAA1__NM_001130410_ATTCACGTCCTTGAGAACCG	ACAA1__NM_001130410	137	228	150	169
ACAA1__NM_001130410_CAGGCAGTGGCCAGCATAGC	ACAA1__NM_001130410	0	0	0	0
ACAA1__NM_001130410_CGAACACTGTCTATTGACAG	ACAA1__NM_001130410	148	85	128	216
ACAA1__NM_001130410_CTGCCGAGAGAAGCTCGTCG	ACAA1__NM_001130410	291	243	380	382
ACAA1__NM_001130410_CTGCGGCATCCAGCCGGAAT	ACAA1__NM_001130410	213	303	301	275
ACAA1__NM_001130410_GAGAATGTGGCTGAGCGGTT	ACAA1__NM_001130410	817	785	859	771
ACAA1__NM_001130410_TCTCTCGGCAGTCATGACCG	ACAA1__NM_001130410	475	382	410	325
ACAA1__NM_001130410_TGCCGAGAGAAGCTCGTCGG	ACAA1__NM_001130410	486	755	484	467
ACAA1__NM_001130410_TGTCAATAGACAGTGTTCGT	ACAA1__NM_001130410	487	299	539	503
ACAA1__NM_001607_AAATATTACTTCGCGCTTGA	ACAA1__NM_001607	72	91	158	157
ACAA2__NM_006111_AACAGTTGACAGTGTGATTA	ACAA2__NM_006111	355	393	389	275
ACAA2__NM_006111_ACAGTTGACAGTGTGATTAT	ACAA2__NM_006111	699	888	789	748
ACAA2__NM_006111_ACGCACATTTCTGACACAGT	ACAA2__NM_006111	1937	1730	2152	1929
ACAA2__NM_006111_ACGCCCTTTGGAGCTTACGG	ACAA2__NM_006111	550	848	619	512
ACAA2__NM_006111_AGGCATGTTGGTTTGCGTGT	ACAA2__NM_006111	985	878	1233	651
ACAA2__NM_006111_AGGCCTCCGTAAGCTCCAAA	ACAA2__NM_006111	167	284	255	228
ACAA2__NM_006111_ATAGCTGGAAGATTCTTTAT	ACAA2__NM_006111	262	290	351	177
ACAA2__NM_006111_CAAGCTTGGATCAGATATCA	ACAA2__NM_006111	304	290	383	459
ACAA2__NM_006111_CAGATGCTATATATTTGGCA	ACAA2__NM_006111	676	797	767	727
ACAA2__NM_006111_CGAACGCCCTTTGGAGCTTA	ACAA2__NM_006111	1183	1221	1260	1577
ACAA2__NM_006111_GAAAGGAAAACAGACAATGC	ACAA2__NM_006111	308	280	297	396
ACAA2__NM_006111_GCCTATTAATCGTGAGAGCT	ACAA2__NM_006111	447	613	561	590
ACACA__NM_198834_AATGCATGCGGTCTATCCGT	ACACA__NM_198834	1082	341	1067	330
ACACA__NM_198834_AGAATTTGTTACTCGCTTTG	ACACA__NM_198834	366	519	384	471
ACACA__NM_198834_ATGGCTTGGCCTTGCACATA	ACACA__NM_198834	483	587	521	492
ACACA__NM_198834_CAGACAGTAAGAATTATAAG	ACACA__NM_198834	243	278	241	310
ACACA__NM_198834_CAGCACTCTCGATTCATAAT	ACACA__NM_198834	898	914	1058	1045
ACACA__NM_198834_CCTGGTGAAGTTGGACCTAC	ACACA__NM_198834	851	780	803	842
ACACA__NM_198834_CTATTATGAATCGAGAGTGC	ACACA__NM_198834	349	387	540	451
ACACA__NM_198834_GCAGAATTTGTTACTCGCTT	ACACA__NM_198834	945	1040	1082	1381
ACACA__NM_198834_GGCTTGCACCTAGTAAAGCA	ACACA__NM_198834	275	80	220	192
ACACA__NM_198834_GGTGAAGTTGGACCTACTGG	ACACA__NM_198834	792	715	901	881
ACACA__NM_198834_TGGCTTGCACCTAGTAAAGC	ACACA__NM_198834	69	15	45	39
ACACA__NM_198834_TGTGTCTGAAGATAACTCAG	ACACA__NM_198834	243	322	219	204
ACACA__NM_198836_GCATGCGGTCTATCCGTAGG	ACACA__NM_198836	706	446	775	304
ACACA__NM_198837_ACATTTCATAAGACCACCTA	ACACA__NM_198837	699	287	830	297
ACACA__NM_198837_CAAGAGAAGTTCCGGTAGTT	ACACA__NM_198837	1198	733	1144	742
ACACA__NM_198837_CCAGGCCATGTGGGCTTTAG	ACACA__NM_198837	393	319	480	547
ACACA__NM_198837_CTTATCCCCTAAAGCCCACA	ACACA__NM_198837	278	138	275	147
ACACA__NM_198837_TGCAATTAGATTCGTTGTCA	ACACA__NM_198837	292	336	340	428
ACACA__NM_198837_TTGCAGGTCTTCGTGTGGAC	ACACA__NM_198837	458	176	492	205
ACACB__NM_001093_ACTAGGAGGGTCCCACTGAT	ACACB__NM_001093	634	670	712	611
ACACB__NM_001093_AGCAAACCTCATCCCGAGCC	ACACB__NM_001093	1439	1994	1660	1689
ACACB__NM_001093_AGTGATTCACTAGCGCCATT	ACACB__NM_001093	956	1094	991	1109
ACACB__NM_001093_ATCTCGTGAGTCTACCCGGA	ACACB__NM_001093	458	418	460	444
[illegible]					

[illegible]

ACTL9__NM_178525_GCTGCCACCAAAGACCGGCG	ACTL9__NM_178525	1058	745	1178	786
ACTL9__NM_178525_GGCGTGGTGGTTATTGACAT	ACTL9__NM_178525	144	136	150	240
ACTL9__NM_178525_GTGCTGTCTGTCTACGCCCA	ACTL9__NM_178525	759	971	946	797
ACTL9__NM_178525_TACGCCCACGGTCGTGTCAG	ACTL9__NM_178525	512	231	403	358
ACTL9__NM_178525_TGGTGGCCACGGTGTAGGTG	ACTL9__NM_178525	26	54	55	28
ACTN1__NM_001102_ACAGATCGAGAACATCGAAG	ACTN1__NM_001102	207	332	214	172
ACTN1__NM_001102_AGCCAGCAAAGGCGTCAAAC	ACTN1__NM_001102	128	116	150	163
ACTN1__NM_001102_ATCCAGGGCCTTGTTGACGT	ACTN1__NM_001102	1607	2227	2143	2470
ACTN1__NM_001102_CAACACCTTGCAGATGCGAT	ACTN1__NM_001102	259	360	349	507
ACTN1__NM_001102_CATCGTTGGAACTGCCCGAC	ACTN1__NM_001102	1058	1093	1429	1532
ACTN1__NM_001102_CGGCCCGAGCTGATTGACTA	ACTN1__NM_001102	986	1174	1151	1399
ACTN1__NM_001102_CTGGTGTCCATCGGAGCCGA	ACTN1__NM_001102	570	468	708	439
ACTN1__NM_001102_CTTTGCTGGCTATGAAATCC	ACTN1__NM_001102	311	537	364	490
ACTN1__NM_001102_GCTAGACACGTAAGTCATGA	ACTN1__NM_001102	572	768	842	963
ACTN1__NM_001102_TGGTTTGCTGAGAATCATAA	ACTN1__NM_001102	0	0	0	0
ACTN1__NM_001102_TTCCTTGGCTGAAGTCTCTA	ACTN1__NM_001102	105	232	145	242
ACTN1__NM_001102_TTCTGGCTGCATGTAATCGT	ACTN1__NM_001102	52	30	67	90
ACTN2__NM_001103_AAATGACCCTGGGTATGATC	ACTN2__NM_001103	370	442	433	367
ACTN2__NM_001103_ACATTCAGAACTTCCATACT	ACTN2__NM_001103	1105	852	1310	1296
ACTN2__NM_001103_ACTGCACGCCGGGCTCTATC	ACTN2__NM_001103	963	1006	1057	874
ACTN2__NM_001103_CATCCTTCGCTTTGCTATTC	ACTN2__NM_001103	455	598	462	498
ACTN2__NM_001103_CCAGGTTAATATTTCCTATG	ACTN2__NM_001103	1012	995	1061	1290
ACTN2__NM_001103_CCTTAGGTGGGAGTTACACC	ACTN2__NM_001103	255	187	271	313
ACTN2__NM_001103_GATGGTCCAGATCATACCCA	ACTN2__NM_001103	284	217	264	270
ACTN2__NM_001103_GCCAGGTTAATATTTCCTAT	ACTN2__NM_001103	909	787	1162	1293
ACTN2__NM_001103_GCCGGGCTCTATCTGGTTCA	ACTN2__NM_001103	1434	1906	1644	1727
ACTN2__NM_001103_GGTGCCGGCTTTCCTTAGGT	ACTN2__NM_001103	1841	1486	1857	1372
ACTN2__NM_001103_TGACTACTCAAAGCTTAACA	ACTN2__NM_001103	315	223	289	300
ACTN2__NM_001103_TTCCTCGATGTTCTCAATCT	ACTN2__NM_001103	64	16	49	47
ACTN2__NM_001278344_AGCCCAAGTCTCGTGCGTTG	ACTN2__NM_001278344	1407	1999	1693	2387
ACTN2__NM_001278344_AGTGGTTGCTCAATGAGATT	ACTN2__NM_001278344	185	172	266	235
ACTN2__NM_001278344_CATGTTCATATCAGGAGATG	ACTN2__NM_001278344	237	225	235	366
ACTN2__NM_001278344_CGAGTCGGCGTCGCTGACAG	ACTN2__NM_001278344	675	542	778	809
ACTN2__NM_001278344_CTCCAGGTGAAGCTGATCAA	ACTN2__NM_001278344	181	120	180	258
ACTN2__NM_001278344_CTGAGAAGGGTTACGAGGAG	ACTN2__NM_001278344	249	299	181	316
ACTN2__NM_001278344_GCAAGAGACGTACGTCATGA	ACTN2__NM_001278344	662	749	729	841
ACTN2__NM_001278344_GCAGATCGCAGCCATCGCGC	ACTN2__NM_001278344	638	630	688	667
ACTN2__NM_001278344_GCAGGCTGAGAAGGGTTACG	ACTN2__NM_001278344	631	253	887	727
ACTN2__NM_001278344_GGCCTCAACGCACGAGACTT	ACTN2__NM_001278344	537	586	563	671
ACTN2__NM_001278344_TCTGAGTAAGCGTTCCCAGT	ACTN2__NM_001278344	394	608	513	388
ACTN2__NM_001278344_TGTGACCAGTGGGACCGACT	ACTN2__NM_001278344	875	798	888	834
ACTN3__NM_001104_CACCAGTTTAACCCCCTTGC	ACTN3__NM_001104	1033	786	1218	986
ACTN3__NM_001104_CACCCCAAAGCCGGATGAGA	ACTN3__NM_001104	944	975	1235	1300
ACTN3__NM_001104_CAGTGTTCAGGTTTCCGATG	ACTN3__NM_001104	943	1019	1176	878
ACTN3__NM_001104_CGACTACGCCAAACTGCGAA	ACTN3__NM_001104	46	64	90	73
ACTN3__NM_001104_GCACCAGGCAGTGAAGGTCT	ACTN3__NM_001104	337	433	541	533
ACTN3__NM_001104_GCAGTGTTCAGGTTTCCGAT	ACTN3__NM_001104	928	822	1140	1132
ACTN3__NM_001104_GCGCAGGTGTGAGTTGCACC	ACTN3__NM_001104	753	694	865	758
ACTN3__NM_001104_GGCCTTCTCATCCGGCTTTG	ACTN3__NM_001104	1176	1588	1289	1654
ACTN3__NM_001104_GTCCAGGGCCTTGTTAACGT	ACTN3__NM_001104	690	531	695	501
ACTN3__NM_001104_TGCACGTTGACGTTGCGGTA	ACTN3__NM_001104	236	189	306	184
ACTN3__NM_001104_TGGGGTGTTCACAATGTCTA	ACTN3__NM_001104	122	146	116	75
ACTN3__NM_001104_TGTCCTGGATGGCGAAGCGA	ACTN3__NM_001104	103	84	121	148
ACTN3__NM_001258371_CCAGAGGGCAGCCTCTAGAG	ACTN3__NM_001258371	277	338	296	256
ACTN3__NM_001258371_TCTTCCAGCACCTCGCTGGT	ACTN3__NM_001258371	331	308	428	384
ACTN4__NM_004924_ACTCTCATCTTCCCCCGCTC	ACTN4__NM_004924	385	205	431	342
ACTN4__NM_004924_CAACAATGTGAACAAAGCGC	ACTN4__NM_004924	567	415	744	671
ACTN4__NM_004924_CACATTGACGTTCTTATACG	ACTN4__NM_004924	697	477	837	697
ACTN4__NM_004924_CTGCTCCTGGAGGTCATATC	ACTN4__NM_004924	263	135	302	296
ACTN4__NM_004924_GCACATTGACGTTCTTATAC	ACTN4__NM_004924	554	380	585	802
ACTN4__NM_004924_GCAGAGACCTCGGCCAAGGA	ACTN4__NM_004924	326	208	396	338
ACTN4__NM_004924_GCGAACCAGTCGTACCAGTA	ACTN4__NM_004924	563	614	690	990
ACTN4__NM_004924_GGGCAGCATGGGCGACTACA	ACTN4__NM_004924	59	81	92	175
ACTN4__NM_004924_TGCACATTGACGTTCTTATA	ACTN4__NM_004924	541	361	556	586

[illegible]

ADCY9__NM_001116_TGAAAGACAACGTTAAGCTC	ADCY9__NM_001116	25	26	28	18
ADCY9__NM_001116_TGATGCTTTCCTTGTTCGTG	ADCY9__NM_001116	1042	769	1069	1456
ADCY9__NM_001116_TGCAAACGCGGGGTTTGTTA	ADCY9__NM_001116	448	463	549	525
ADCY9__NM_001116_TGCAGCTCCTTGGTTTCCCG	ADCY9__NM_001116	741	608	793	667
ADCY9__NM_001116_TGCCAGTAATCCGCCAAGAG	ADCY9__NM_001116	260	146	288	305
ADCY9__NM_001116_TGGAAGCTGACTCATCGGAG	ADCY9__NM_001116	109	135	144	166
ADCY9__NM_001116_TGGAGGCCCTCTCGAACAGC	ADCY9__NM_001116	158	285	157	168
ADCY9__NM_001116_TGGGGCTCTCAAGCTATTCG	ADCY9__NM_001116	74	90	83	75
ADCY9__NM_001116_TGTACGCCCGGCATTACGCG	ADCY9__NM_001116	553	664	542	587
ADCY9__NM_001116_TGTCTTGGCCTGCATGCATG	ADCY9__NM_001116	309	324	336	341
ADCY9__NM_001116_TGTTAGGGGCGGAATCCCGA	ADCY9__NM_001116	179	205	213	192
ADCY9__NM_001116_TTCCTCACCCCGGGAAACCA	ADCY9__NM_001116	85	133	74	93
ADCY9__NM_001116_TTCTGCACTGAGGTATGCAT	ADCY9__NM_001116	4	0	19	12
ADCYAP1__NM_001099733_AGGCCAGAGGAAGAGGCGTA	ADCYAP1__NM_001099733	173	76	149	178
ADCYAP1__NM_001099733_AGGCCTCGTTAAGGATCCCG	ADCYAP1__NM_001099733	252	101	287	154
ADCYAP1__NM_001099733_CAGCCGCTACCGGAAACAAA	ADCYAP1__NM_001099733	440	385	466	344
ADCYAP1__NM_001099733_CAGCCTCGCTCCGCTACACA	ADCYAP1__NM_001099733	554	541	794	663
ADCYAP1__NM_001099733_CGCAGAATGACCATGTGTAG	ADCYAP1__NM_001099733	717	663	693	740
ADCYAP1__NM_001099733_CGCCCACGGGATCCTTAACG	ADCYAP1__NM_001099733	1760	1346	1950	1300
ADCYAP1__NM_001099733_CTCTCCAAGCGCCACTCGGA	ADCYAP1__NM_001099733	104	55	93	74
ADCYAP1__NM_001099733_CTTTGCGGTAGGCCTCGTTA	ADCYAP1__NM_001099733	588	180	609	380
ADCYAP1__NM_001099733_GCCGCTCTCCAAGCGCCACT	ADCYAP1__NM_001099733	100	179	115	79
ADCYAP1__NM_001099733_GGCTGGCCCTGCTGGTCTAT	ADCYAP1__NM_001099733	912	1331	1166	1567
ADCYAP1__NM_001099733_GGCTGTCAAGAAATACTTGG	ADCYAP1__NM_001099733	757	874	818	844
ADCYAP1__NM_001099733_TCTCCAAGCGCCACTCGGAC	ADCYAP1__NM_001099733	649	1022	662	881
ADCYAP1__NM_001117_AAATCTGCTTCAGTTTAGTA	ADCYAP1__NM_001117	367	265	377	441
ADCYAP1__NM_001117_AAATGCTTGCAACTAGCCAC	ADCYAP1__NM_001117	350	199	358	202
ADCYAP1__NM_001117_AATCCCAGCCTAACTCAACT	ADCYAP1__NM_001117	681	423	858	496
ADCYAP1__NM_001117_ACATAGAGGTATATTGGAAG	ADCYAP1__NM_001117	204	164	223	198
ADCYAP1__NM_001117_AGATATGGAGAATTGCTGAA	ADCYAP1__NM_001117	255	131	288	257
ADCYAP1__NM_001117_AGGGAGAGGCGTGAATTCTG	ADCYAP1__NM_001117	680	500	826	654
ADCYAP1__NM_001117_AGTCTAATTTAGTGTTCCCG	ADCYAP1__NM_001117	501	353	507	501
ADCYAP1__NM_001117_AGTGCATAATTACTTACTAG	ADCYAP1__NM_001117	612	270	566	555
ADCYAP1__NM_001117_CACACATGATATCTATAACT	ADCYAP1__NM_001117	743	501	1000	826
ADCYAP1__NM_001117_CATACTGATCAAATCCGTAA	ADCYAP1__NM_001117	542	269	625	497
ADCYAP1__NM_001117_CGATGACTGTTGAGTCAGTT	ADCYAP1__NM_001117	1189	1561	1356	1771
ADCYAP1__NM_001117_CGCCCAAAGTCAGGACCATT	ADCYAP1__NM_001117	1627	1277	1754	1445
ADCYAP1__NM_001117_CGTTAGCTTGTAAAGTTTAA	ADCYAP1__NM_001117	244	362	291	390
ADCYAP1__NM_001117_CTAGGGAGAGGCGTGAATTC	ADCYAP1__NM_001117	225	86	255	193
ADCYAP1__NM_001117_CTCCTCAGGCAATGTGACAC	ADCYAP1__NM_001117	856	443	840	607
ADCYAP1__NM_001117_GAGCCCAGTTGAGTTAGGCT	ADCYAP1__NM_001117	1104	942	1143	923
ADCYAP1__NM_001117_GATACAAGCGTAGGTTCTCA	ADCYAP1__NM_001117	86	94	111	98
ADCYAP1__NM_001117_GCGTCAGGGCTGTATACACA	ADCYAP1__NM_001117	876	699	903	754
ADCYAP1__NM_001117_GTATTTGTATCAGCTGTTAG	ADCYAP1__NM_001117	250	275	271	262
ADCYAP1__NM_001117_TATCCCCAAATTAGGGCAAT	ADCYAP1__NM_001117	417	381	601	629
ADCYAP1__NM_001117_TGAAGGACATAGAGGTATAT	ADCYAP1__NM_001117	214	135	242	140
ADCYAP1__NM_001117_TGAGGAGACTGCGGAATTAA	ADCYAP1__NM_001117	334	219	356	274
ADCYAP1__NM_001117_TGCGTCAGGGCTGTATACAC	ADCYAP1__NM_001117	1112	840	1079	667
ADCYAP1R1__NM_001118_AAAACAACCAACTAACCAAT	ADCYAP1R1__NM_001118	249	348	296	256
ADCYAP1R1__NM_001118_AACCACCAGCATCAGGAGTA	ADCYAP1R1__NM_001118	475	473	483	573
ADCYAP1R1__NM_001118_AAGGTTACATGCGTACTGCA	ADCYAP1R1__NM_001118	213	435	208	243
ADCYAP1R1__NM_001118_AAGTTGCCTCATCTCACAAA	ADCYAP1R1__NM_001118	448	409	471	682
ADCYAP1R1__NM_001118_ACAGATGTGGGCTTGTTATT	ADCYAP1R1__NM_001118	712	1213	734	875
ADCYAP1R1__NM_001118_ACAGCAGTCTGAACCCAACT	ADCYAP1R1__NM_001118	980	1168	1191	1181
ADCYAP1R1__NM_001118_ACCACCAGCATCAGGAGTAA	ADCYAP1R1__NM_001118	459	717	499	663
ADCYAP1R1__NM_001118_AGCCACCCACATCCCGCACT	ADCYAP1R1__NM_001118	4206	4536	4858	5255
ADCYAP1R1__NM_001118_AGCTTTGCCTCTATCAGGGC	ADCYAP1R1__NM_001118	230	343	251	303
ADCYAP1R1__NM_001118_AGGGTGGCATGCACACTCGA	ADCYAP1R1__NM_001118	189	103	176	257
ADCYAP1R1__NM_001118_ATGAATATGAATCTGAGACT	ADCYAP1R1__NM_001118	445	476	447	481
ADCYAP1R1__NM_001118_ATGGTTCCTGCCTCACTTGT	ADCYAP1R1__NM_001118	32	4	54	45
ADCYAP1R1__NM_001118_ATTCATCAAAACCTGATATG	ADCYAP1R1__NM_001118	205	108	270	241
ADCYAP1R1__NM_001118_ATTGAAGCCATCAGCTCAT	ADCYAP1R1__NM_001118	580	935	811	775
ADCYAP1R1__NM_001118_ATTGGCATTGTTAGTCCCAC	ADCYAP1R1__NM_001118	290	227	319	274
ADCYAP1R1__NM_001118_CAAAGACTGGATTCTGTATG	ADCYAP1R1__NM_001118	1551	2260	1762	2091
ADCYAP1R1__NM_001118_CAAAGTCAAGACAGGTAAAA	ADCYAP1R1__NM_001118	93	139	105	190
ADCYAP1R1__NM_001118_CAATGTGACTTGGACTTAAG	ADCYAP1R1__NM_001118	468	666	503	758
ADCYAP1R1__NM_001118_CACACCCAAAATCAGATGAT	ADCYAP1R1__NM_001118	1281	1671	1426	1558
ADCYAP1R1__NM_001118_CAGAGGGCCAATGAGCTGAT	ADCYAP1R1__NM_001118	1094	1447	1399	1796
ADCYAP1R1__NM_001118_CAGCAGCAGCCTCTGCTAAT	ADCYAP1R1__NM_001118	755	1075	884	954
ADCYAP1R1__NM_001118_CAGGAAGCAGCACAGTGCAT	ADCYAP1R1__NM_001118	547	441	625	395
ADCYAP1R1__NM_001118_CAGGACTGTGGTATTTGCCT	ADCYAP1R1__NM_001118	1227	1127	1337	1624
ADCYAP1R1__NM_001118_CAGGGTGGCATGCACACTCG	ADCYAP1R1__NM_001118	1022	1076	1231	1206
ADCYAP1R1__NM_001118_CAGTAGCAGTAAGGTCTGTG	ADCYAP1R1__NM_001118	646	950	718	866
ADCYAP1R1__NM_001118_CATGGCTGCTATCACCCATA	ADCYAP1R1__NM_001118	592	703	668	573
ADCYAP1R1__NM_001118_CATGGTTCCTGCCTCACTTG	ADCYAP1R1__NM_001118	712	1048	866	911
ADCYAP1R1__NM_001118_CCACAGGCATCAAAGTAATG	ADCYAP1R1__NM_001118	948	1323	1130	1148
ADCYAP1R1__NM_001118_CCCAACATGCCCATTAGCAG	ADCYAP1R1__NM_001118	1369	1821	1554	1938
ADCYAP1R1__NM_001118_CGGCACCTCTATGAGGATGC	ADCYAP1R1__NM_001118	185	336	133	201
ADCYAP1R1__NM_001118_CTCGTTCTATGCAATCCTAA	ADCYAP1R1__NM_001118	272	319	203	252
ADCYAP1R1__NM_001118_CTCTTCAGCCTTAGCTCACA	ADCYAP1R1__NM_001118	67	62	78	35
ADCYAP1R1__NM_001118_CTGGCAAAAATTCACTCAGA	ADCYAP1R1__NM_001118	490	946	523	685
ADCYAP1R1__NM_001118_CTTGGTCTGGGTTGAAGATT	ADCYAP1R1__NM_001118	714	1045	938	1038
ADCYAP1R1__NM_001118_CTTGTTGCTGTAATTTAGAG	ADCYAP1R1__NM_001118	894	1234	972	1386
ADCYAP1R1__NM_001118_GAAGAGGCGCAAACATGCGT	ADCYAP1R1__NM_001118	363	393	517	457
ADCYAP1R1__NM_001118_GAGTGAGGATACTTCCATAT	ADCYAP1R1__NM_001118	582	558	544	445
ADCYAP1R1__NM_001118_GCCCATTCCAAGTAGATGGC	ADCYAP1R1__NM_001118	863	1021	1007	1439
ADCYAP1R1__NM_001118_GCTCACAAAATACTAAAGCT	ADCYAP1R1__NM_001118	321	325	443	495
ADCYAP1R1__NM_001118_GCTGTAGCCAACCGTGTAGA	ADCYAP1R1__NM_001118	2004	1885	2071	2424
ADCYAP1R1__NM_001118_GGAAAAATCACTTACGTGCA	ADCYAP1R1__NM_001118	427	837	506	764
ADCYAP1R1__NM_001118_GGAAGAGGCGCAAACATGCG	ADCYAP1R1__NM_001118	354	459	461	674
ADCYAP1R1__NM_001118_GGAATGAGCTAGGCCACACA	ADCYAP1R1__NM_001118	480	642	483	443
ADCYAP1R1__NM_001118_GGAATGATCATCAACAGGGG	ADCYAP1R1__NM_001118	40	24	50	105
ADCYAP1R1__NM_001118_GGCCCAAAGAAGGTAAGCCC	ADCYAP1R1__NM_001118	323	470	338	233
ADCYAP1R1__NM_001118_GGCCTCCTAATCTGGGTAAT	ADCYAP1R1__NM_001118	730	960	856	1143
ADCYAP1R1__NM_001118_GGTGGAATGTAAGGCCGTCA	ADCYAP1R1__NM_001118	705	761	761	777
ADCYAP1R1__NM_001118_GTCACATTCTGCTCATGTGC	ADCYAP1R1__NM_001118	574	547	674	707
ADCYAP1R1__NM_001118_GTGAAGGCCCTCTACACGGT	ADCYAP1R1__NM_001118	2304	2694	2703	2328
ADCYAP1R1__NM_001118_TAGAGGTGCCGGGTTCCTAT	ADCYAP1R1__NM_001118	527	603	574	526
ADCYAP1R1__NM_001118_TCAGGATTCGCCGACATCAG	ADCYAP1R1__NM_001118	410	510	445	365
ADCYAP1R1__NM_001118_TCAGGGAGGGACTCTCATCC	ADCYAP1R1__NM_001118	664	852	739	643
ADCYAP1R1__NM_001118_TCATCATTGTGCCTCATATC	ADCYAP1R1__NM_001118	791	1060	1083	1183
ADCYAP1R1__NM_001118_TCCCAGGAATGATCATCAAC	ADCYAP1R1__NM_001118	343	496	447	320
ADCYAP1R1__NM_001118_TCCTCATCATCCATGGGAGT	ADCYAP1R1__NM_001118	1653	1825	1767	1884
ADCYAP1R1__NM_001118_TCCTTGAAGTTGACTCCCAG	ADCYAP1R1__NM_001118	658	886	788	811
ADCYAP1R1__NM_001118_TCTGCAAACAGAATTTGGAC	ADCYAP1R1__NM_001118	452	673	545	584
ADCYAP1R1__NM_001118_TCTTGGGGTGTTTCTGCACT	ADCYAP1R1__NM_001118	870	957	884	1130
ADCYAP1R1__NM_001118_TGAGCCTCCTCAAAGACATA	ADCYAP1R1__NM_001118	129	129	169	170
ADCYAP1R1__NM_001118_TGCAGGTGGAGGGGATCATG	ADCYAP1R1__NM_001118	130	107	194	147
ADCYAP1R1__NM_001118_TGCAGTCAGAATGCATGGCA	ADCYAP1R1__NM_001118	409	301	402	514
ADCYAP1R1__NM_001118_TGCTGTAGCCAACCGTGTAG	ADCYAP1R1__NM_001118	554	946	631	705
ADCYAP1R1__NM_001118_TGGAAAAATCACTTACGTGC	ADCYAP1R1__NM_001118	34	85	34	94
ADCYAP1R1__NM_001118_TGGAAGGAAGGATTAGGTCA	ADCYAP1R1__NM_001118	43	100	25	17
ADCYAP1R1__NM_001118_TGGCAGCAGCAGTAGCAGTA	ADCYAP1R1__NM_001118	22	5	6	12
ADCYAP1R1__NM_001118_TGTGGGACAACATCACGTGT	ADCYAP1R1__NM_001118	1210	863	1379	1115
ADCYAP1R1__NM_001118_TTGAGGAGTATTTACATGTC	ADCYAP1R1__NM_001118	988	1029	1063	1383
ADCYAP1R1__NM_001118_TTGCGAAAGAGACGGAGTTT	ADCYAP1R1__NM_001118	290	265	308	280
ADCYAP1R1__NM_001118_TTGGCGGGGATTATAGATGC	ADCYAP1R1__NM_001118	307	610	414	362
ADCYAP1R1__NM_001118_TTTGGAAATTACACAGATGT	ADCYAP1R1__NM_001118	62	20	47	51
ADD1__NM_001119_AAGAAGGGGAAGAACCCCAC	ADD1__NM_001119	178	102	165	141
ADD1__NM_001119_ACTCCTGTGAACGATCTTAG	ADD1__NM_001119	550	717	557	681
ADD1__NM_001119_AGAACAACCCAGAGTACTTG	ADD1__NM_001119	123	170	185	209
ADD1__NM_001119_ATACAGGAGCAATTTAAGAA	ADD1__NM_001119	364	451	300	314
ADD1__NM_001119_CAATCTGCTGTAATGCCAAT	ADD1__NM_001119	686	1186	790	967
ADD1__NM_001119_GAAGAACCCCACAGGCCTAT	ADD1__NM_001119	471	464	547	673
ADD1__NM_001119_GAAGTACCTCTCCTTGTGAG	ADD1__NM_001119	299	360	383	398
ADD1__NM_001119_GATACAGGAGCAATTTAAGA	ADD1__NM_001119	260	282	284	340
ADD1__NM_001119_GATCCTCTAAGATCGTTCAC	ADD1__NM_001119	263	211	225	211
ADD1__NM_001119_GTAGACATTTGGTACATTCG	ADD1__NM_001119	182	197	229	218
ADD1__NM_001119_TCGAAGTACCTCTCCTTGTG	ADD1__NM_001119	986	672	1029	995
ADD1__NM_001119_TGTAATGCCAATAGGCCTGT	ADD1__NM_001119	136	61	145	97
ADD2__NM_001185054_ACTCGGCCATCTATGCAGCG	ADD2__NM_001185054	879	1090	1019	1357
ADD2__NM_001185054_AGTACATGCGCCTTCGCAAC	ADD2__NM_001185054	503	339	580	636
ADD2__NM_001185054_ATGGCCTATTATGACTTCAA	ADD2__NM_001185054	155	236	252	189
ADD2__NM_001185054_CACTTCCTGATCAGCCCTAA	ADD2__NM_001185054	1500	1772	1899	2247
ADD2__NM_001185054_GAAGCACCGGCCCCATGAGG	ADD2__NM_001185054	472	281	554	477
ADD2__NM_001185054_GCAGATCAAGGTGAACATTC	ADD2__NM_001185054	146	332	151	150
ADD2__NM_001185054_GCCTATGCAGAAGAGTCGGC	ADD2__NM_001185054	602	768	734	879
ADD2__NM_001185054_GCTCCTTGCTGACTCTCAAC	ADD2__NM_001185054	562	678	742	1134
ADD2__NM_001185054_GTCGGCTCTGTCCAGTGCCG	ADD2__NM_001185054	544	560	467	612
ADD2__NM_001185054_GTTGCGAAGGCGCATGTACT	ADD2__NM_001185054	537	601	679	556
ADD2__NM_001185054_TACCGACTCCTGGACCTCTA	ADD2__NM_001185054	733	781	973	956
ADD2__NM_001185054_TGTCGGGAAGACTGCGTGGG	ADD2__NM_001185054	446	351	478	444
ADD3__NM_001121_AAAATGACCCAGAATACATT	ADD3__NM_001121	293	370	374	506
ADD3__NM_001121_ACAGGCCTTTCGGGAAGACT	ADD3__NM_001121	191	156	232	374
ADD3__NM_001121_ACCCAGAATACATTAGGGAG	ADD3__NM_001121	387	593	565	477
ADD3__NM_001121_AGAAAAACCCGAGAAAGAAT	ADD3__NM_001121	73	85	88	91
ADD3__NM_001121_GAGGAGTGGTAATCACGCCT	ADD3__NM_001121	447	328	465	474
ADD3__NM_001121_GCAGATTGCAGATTACATCA	ADD3__NM_001121	486	573	670	794
ADD3__NM_001121_GTCTACAAGTCTGTACAGGC	ADD3__NM_001121	171	179	326	248
ADD3__NM_001121_TATGAGGATGTATCTGCACC	ADD3__NM_001121	858	942	895	769
ADD3__NM_001121_TCAAAATATCTCTCTTTGTG	ADD3__NM_001121	553	656	620	796
ADD3__NM_001121_TGTAATGCTAGTAATCCAGT	ADD3__NM_001121	418	557	456	504
ADD3__NM_001121_TTCCTCTCCCTAATGTATTC	ADD3__NM_001121	9	6	30	9
ADD3__NM_001121_TTGAAGTCTTGTCGTAGATC	ADD3__NM_001121	13	26	31	39
ADGB__NM_024694_AAAGGAAGTAAAAGACGTGA	ADGB__NM_024694	92	195	98	99
ADGB__NM_024694_AAGTTCTTTGACAACACTAA	ADGB__NM_024694	74	129	84	163
ADGB__NM_024694_AGAAGGGGAAATTCCCACTC	ADGB__NM_024694	74	87	106	38
ADGB__NM_024694_AGAGGTGCATCGTATCAACT	ADGB__NM_024694	412	591	464	690
ADGB__NM_024694_ATGCCTTTGTTCAATAGCTA	ADGB__NM_024694	84	54	110	141
ADGB__NM_024694_CATCGTATCAACTCGGCGCA	ADGB__NM_024694	839	1077	909	1209
ADGB__NM_024694_GAAAAGTGGGATGCAGGCAA	ADGB__NM_024694	250	218	316	396
ADGB__NM_024694_GCTCTCTCCTGTCTGCTACA	ADGB__NM_024694	860	779	896	786
ADGB__NM_024694_GGTTCTACTGAACAAAAGAA	ADGB__NM_024694	37	88	24	44
ADGB__NM_024694_GTCTAAAGCTATTATCAAGC	ADGB__NM_024694	378	608	369	527
ADGB__NM_024694_TATCAACTCGGCGCACGGAT	ADGB__NM_024694	426	307	568	485
ADGB__NM_024694_TTTGGCAGTAATGTACAATC	ADGB__NM_024694	203	205	267	212
ADH1A__NM_000667_AGACTACAAGAAACCCATCC	ADH1A__NM_000667	363	331	461	417
ADH1A__NM_000667_ATGGTGGCTGTAGGAATCTG	ADH1A__NM_000667	144	211	175	118
ADH1A__NM_000667_CACACTGAGGAATAGCGAGT	ADH1A__NM_000667	89	40	72	69
ADH1A__NM_000667_CACCTTAATACGAACTTCAT	ADH1A__NM_000667	546	554	714	918
ADH1A__NM_000667_CCACGTGGTTAGTGGTACCA	ADH1A__NM_000667	338	558	431	384
ADH1A__NM_000667_CGAACTTCATGGGCCTTAGG	ADH1A__NM_000667	600	630	548	540
ADH1A__NM_000667_GGCCCATGAAGTTCGTATTA	ADH1A__NM_000667	694	699	816	1013
ADH1A__NM_000667_GGCCTAAAATCACAGGAAGT	ADH1A__NM_000667	308	352	458	525
ADH1A__NM_000667_GTCGGCCTATCTGCTATTAT	ADH1A__NM_000667	1229	1618	1377	1655
ADH1A__NM_000667_GTGGCACAAGTGTCATCGTA	ADH1A__NM_000667	79	152	148	108
ADH1A__NM_000667_TACAGCCCATAATAGCAGAT	ADH1A__NM_000667	298	446	404	367
ADH1A__NM_000667_TCACCTTAATACGAACTTCA	ADH1A__NM_000667	91	62	90	179
ADH1B__NM_000668_ATTGGCTGTGGATTCTCGAC	ADH1B__NM_000668	649	508	661	652
ADH1B__NM_000668_CGAACTTCATAAGCCTTAGG	ADH1B__NM_000668	395	507	462	564
ADH1B__NM_000668_GTGGCACAAGCGTCATCGTA	ADH1B__NM_000668	145	135	165	203
ADH1B__NM_000668_TACAGCCCATAACAGCAGAT	ADH1B__NM_000668	428	664	512	616
ADH1B__NM_000668_TGGCACAAGCGTCATCGTAG	ADH1B__NM_000668	155	71	223	145
ADH1B__NM_000668_TGTGGCACAAGCGTCATCGT	ADH1B__NM_000668	721	629	924	863
ADH1C__NM_000669_ATTTGCAAAGGCTAAAGAGT	ADH1C__NM_000669	209	161	283	385
ADH1C__NM_000669_GGTCGGCCTATCTGTTGTTA	ADH1C__NM_000669	799	971	1137	1695
ADH1C__NM_000669_GTGGCACAAGTGTCATTGTA	ADH1C__NM_000669	249	391	240	219
ADH1C__NM_000669_TAAAGTCAGCCACAAGTTTG	ADH1C__NM_000669	97	95	97	142
[illegible]	[illegible]	[illegible]	[illegible]	[illegible]	[illegible]

AGPAT6__NM_178819_GCATCCGTTTAGACCACCAA	AGPAT6__NM_178819	524	393	594	521
AGPAT6__NM_178819_GCCCGCACAGCGTGCAAAGG	AGPAT6__NM_178819	41	63	47	52
AGPAT6__NM_178819_GCTGCTGATGGGGTTACTAA	AGPAT6__NM_178819	856	1064	866	744
AGPAT6__NM_178819_GCTGTGCGGGCTGAGTGGTT	AGPAT6__NM_178819	1795	1543	1832	1329
AGPAT6__NM_178819_GGAGACCATTATGGATGATG	AGPAT6__NM_178819	160	259	187	176
AGPAT6__NM_178819_GGCACCTGGCGTGTGTTGCG	AGPAT6__NM_178819	1513	1870	1511	1813
AGPAT6__NM_178819_GGCAGGTGGGACCTATGACA	AGPAT6__NM_178819	519	453	617	461
AGPAT6__NM_178819_GGCCCGGTCCGTAGGCTGGA	AGPAT6__NM_178819	1702	1959	1783	2390
AGPAT6__NM_178819_GGGGATCCTTCATGCATCAG	AGPAT6__NM_178819	288	377	448	371
AGPAT6__NM_178819_GGGGGTTAGGAGTGCTGATT	AGPAT6__NM_178819	268	332	321	372
AGPAT6__NM_178819_GGTAACCGAGTTGGTCTTAT	AGPAT6__NM_178819	215	356	199	194
AGPAT6__NM_178819_GTAAAAGGCATACACGGAAA	AGPAT6__NM_178819	235	276	210	305
AGPAT6__NM_178819_GTACATCAGCCTTCGGCTCA	AGPAT6__NM_178819	224	369	211	302
AGPAT6__NM_178819_GTTACTTTCGTGATTTGCAT	AGPAT6__NM_178819	171	108	190	207
AGPAT6__NM_178819_TAAAAGGCATACACGGAAAG	AGPAT6__NM_178819	129	163	160	189
AGPAT6__NM_178819_TACTCTGACTATGGGTCACT	AGPAT6__NM_178819	901	821	953	1189
AGPAT6__NM_178819_TAGTGGAACTAACCCACTTG	AGPAT6__NM_178819	59	71	89	122
AGPAT6__NM_178819_TCAGCCCGCACAGCGTGCAA	AGPAT6__NM_178819	11	99	32	12
AGPAT6__NM_178819_TCCCCATCTGTAATATGAGT	AGPAT6__NM_178819	1499	1347	1686	1613
AGPAT6__NM_178819_TCGGAGAGAGACATATTGAC	AGPAT6__NM_178819	403	334	461	416
AGPAT6__NM_178819_TCGTCGAAGTGGTAGTAGTA	AGPAT6__NM_178819	280	182	338	403
AGPAT6__NM_178819_TGCAGAGAAAAGGAGATTGC	AGPAT6__NM_178819	156	107	141	221
AGPAT6__NM_178819_TGCCCTTCCAGCCTACGGAC	AGPAT6__NM_178819	495	532	515	575
AGPAT6__NM_178819_TTCGGCTCACGGTCCTGTGG	AGPAT6__NM_178819	291	227	285	363
AGPAT6__NM_178819_TTGGGACCCATGCTCCCTCG	AGPAT6__NM_178819	242	183	266	252
AGPAT6__NM_178819_TTTCCACTCATTACACCACT	AGPAT6__NM_178819	311	382	349	369
AGPAT9__NM_001256421_AAAGGGCTCTCTGGTCTACG	AGPAT9__NM_001256421	470	517	535	603
AGPAT9__NM_001256421_AACGGATGGATGTTATGCTA	AGPAT9__NM_001256421	261	329	291	312
AGPAT9__NM_001256421_ATCAATGGGGGAAGTATGGT	AGPAT9__NM_001256421	753	692	834	815
AGPAT9__NM_001256421_CCAAAGAGATGAGTCACCCA	AGPAT9__NM_001256421	432	251	462	453
AGPAT9__NM_001256421_CCTCAGAGGAGCTAGTGTCA	AGPAT9__NM_001256421	731	682	699	786
AGPAT9__NM_001256421_CTCCGAAGACCGAAGGTAAA	AGPAT9__NM_001256421	71	85	137	42
AGPAT9__NM_001256421_GGGATCAGTTTGCTGGTTAT	AGPAT9__NM_001256421	722	1242	772	919
AGPAT9__NM_001256421_TAGAAGATGAAGTGACCCAG	AGPAT9__NM_001256421	285	186	331	405
AGPAT9__NM_001256421_TATTCAGAGAGCTATGGTCA	AGPAT9__NM_001256421	420	477	478	512
AGPAT9__NM_001256421_TCTGCGGCTCACTATGGTGT	AGPAT9__NM_001256421	1153	1319	1466	1449
AGPAT9__NM_001256421_TTATAGGAACTACACTGGTT	AGPAT9__NM_001256421	247	145	184	184
AGPAT9__NM_001256421_TTTCATTGGGATCAGTTTGC	AGPAT9__NM_001256421	82	151	88	71
AGPS__NM_003659_AAAGAAAGTGGTTATTGTAC	AGPS__NM_003659	96	115	86	127
AGPS__NM_003659_AAGGGCCAAATTGAATTGAC	AGPS__NM_003659	172	321	268	211
AGPS__NM_003659_AGGGCCAAATTGAATTGACT	AGPS__NM_003659	532	446	508	481
AGPS__NM_003659_AGGTACCCTCTTAGTGGCAT	AGPS__NM_003659	292	351	315	492
AGPS__NM_003659_AGGTGTATCACTAGGATTTA	AGPS__NM_003659	224	333	208	203
AGPS__NM_003659_AGTTATGAAATGGAATGGAT	AGPS__NM_003659	281	260	283	329
AGPS__NM_003659_CATGTAGAGGCTGGCATAAC	AGPS__NM_003659	1916	1786	1946	2106
AGPS__NM_003659_CTTGGCCATAAAACTATATC	AGPS__NM_003659	376	453	411	525
AGPS__NM_003659_GGTAAACCCATGCCACTAAG	AGPS__NM_003659	190	251	318	426
AGPS__NM_003659_TAGGTACCCTCTTAGTGGCA	AGPS__NM_003659	320	338	281	248
AGPS__NM_003659_TTCTTTGGACACTTCACAAA	AGPS__NM_003659	2	2	22	11
AGPS__NM_003659_TTGACAGCTCATGTAGAGGC	AGPS__NM_003659	251	271	284	255
AGR2__NM_006408_ACAGTTAGAGCCGATATCAC	AGR2__NM_006408	620	769	626	536
AGR2__NM_006408_ATGTCTGAGTCCAGATGAGT	AGR2__NM_006408	513	471	533	514
AGR2__NM_006408_CTCTGGCCAGAGTGTAGGAG	AGR2__NM_006408	108	107	75	154
AGR2__NM_006408_GTTGGGGTGACCAACTCATC	AGR2__NM_006408	118	0	129	0
AGR3__NM_176813_AAGAAACTGTGACGAGTAAG	AGR3__NM_176813	220	282	309	528
AGR3__NM_176813_CTAGGCACATATTGCCCATC	AGR3__NM_176813	584	699	601	531
AGR3__NM_176813_GAGGATTGTCAATACTCTCA	AGR3__NM_176813	135	161	173	198
AGR3__NM_176813_GTGCCTAGAATCATGTTTGT	AGR3__NM_176813	538	561	487	547
AGR3__NM_176813_GTTCATCATGCTAAACCTTA	AGR3__NM_176813	52	36	100	115
AGR3__NM_176813_GTTTAGTAAGAAGCCATTAA	AGR3__NM_176813	55	66	52	71
AGR3__NM_176813_TTACATAGGGGTAAATCCCG	AGR3__NM_176813	136	170	162	286
AGRN__NM_198576_AAGAAGTTCGACGGCCCTTG	AGRN__NM_198576	465	540	613	532
AGRN__NM_198576_ACTGTGTCATGGGCCGATCG	AGRN__NM_198576	1021	1313	1270	1458
AGRN__NM_198576_AGCGACGGCGCCGACTACCC	AGRN__NM_198576	911	967	1079	845
AGRN__NM_198576_CGGGTCTGGCGGTACTTGAA	AGRN__NM_198576	269	186	337	257
AGRN__NM_198576_CTACGAAAACGACTGTGTCA	AGRN__NM_198576	220	155	210	351
AGRN__NM_198576_GCGTGGTGGCGCCTGTGTGT	AGRN__NM_198576	1580	1230	1893	1480
AGRN__NM_198576_GCTGGACGGCGGCAACAAGG	AGRN__NM_198576	89	63	83	42
AGRN__NM_198576_GGCAACAAGGTGGTGATCAG	AGRN__NM_198576	0	0	0	0
AGRN__NM_198576_GGTGGAGGAGATCCTCAACG	AGRN__NM_198576	343	184	319	207
AGRN__NM_198576_GTCAGGGAGGGCGCCCTGAC	AGRN__NM_198576	87	79	44	100
AGRN__NM_198576_GTGTGCGAGCCCAACGCGGA	AGRN__NM_198576	75	99	125	101
AGRN__NM_198576_TCAGGGAGGGCGCCCTGACA	AGRN__NM_198576	82	91	85	90
AGRP__NM_001138_GCTCCTCACGTCGCTGCGTA	AGRP__NM_001138	100	132	130	168
AGRP__NM_001138_GGCAGTAGCACGTGGCACAT	AGRP__NM_001138	892	1141	1042	1055
AGRP__NM_001138_GGGCATCAGAAGGCCTGACC	AGRP__NM_001138	364	448	426	319
AGRP__NM_001138_TCATGGCAGTACCCAGCTTG	AGRP__NM_001138	1133	1339	1226	879
AGT__NM_000029_AGTTGAGGGCCGCAATGGTC	AGT__NM_000029	555	336	596	347
AGT__NM_000029_ATGGCATGCACAGTGAGCTA	AGT__NM_000029	139	181	148	198
AGT__NM_000029_ATTGGAGCAGGTATGAAGGT	AGT__NM_000029	328	538	366	401
AGT__NM_000029_CAAGGCTCCCAGATAGAGAG	AGT__NM_000029	157	390	256	322
AGT__NM_000029_CAGCTGGCAAAGGCCAATGC	AGT__NM_000029	220	297	313	405
AGT__NM_000029_CCCTGCCCTGGGCCACTAGC	AGT__NM_000029	65	127	69	70
AGT__NM_000029_CCTGAAGCAGCCGTTTGTGC	AGT__NM_000029	4545	4747	4693	5111
AGT__NM_000029_GGCATGCACAGTGAGCTATG	AGT__NM_000029	459	320	425	400
AGT__NM_000029_GGGGATGTCTTGGCCTGAAT	AGT__NM_000029	802	964	795	1072
AGT__NM_000029_TGGCATGCACAGTGAGCTAT	AGT__NM_000029	91	62	116	74
AGT__NM_000029_TGTGGATGAAAAGGCCCTAC	AGT__NM_000029	296	371	250	305
AGT__NM_000029_TTCTTGGGCTTCCGTATATA	AGT__NM_000029	1	0	7	4
AGTPBP1__NM_001286715_AGGAGAGAAATGACAGCCAA	AGTPBP1__NM_001286715	216	149	274	283
AGTPBP1__NM_001286715_ATGACAGCCAAAGGTTCTAC	AGTPBP1__NM_001286715	405	400	409	395
AGTPBP1__NM_001286715_ATGTGGGGGAGATTCTTTGC	AGTPBP1__NM_001286715	481	403	523	414
AGTPBP1__NM_001286715_CGATCCTAGAATTATTGGTA	AGTPBP1__NM_001286715	534	525	513	507
AGTPBP1__NM_001286715_CTGTGAATTCAGTATCCTTA	AGTPBP1__NM_001286715	592	685	751	1050

[illegible]

ALDOB__NM_000035_ACGGTGGAGAGCTGTTACGG	ALDOB__NM_000035	519	361	455	503
ALDOB__NM_000035_ATTACCTCTGGTTCAACAAT	ALDOB__NM_000035	200	136	144	191
ALDOB__NM_000035_CCTCAAGGAAAAGGGGATCG	ALDOB__NM_000035	237	360	317	230
ALDOB__NM_000035_GAACCAGAGGTAATTCCTGA	ALDOB__NM_000035	730	861	831	963
ALDOB__NM_000035_GAATGACCATCATGTTTACC	ALDOB__NM_000035	495	735	548	673
ALDOB__NM_000035_GATAGCATTGAGGTTGAGAG	ALDOB__NM_000035	400	512	552	479
ALDOB__NM_000035_GGATTTCTCGGAACTGCCGG	ALDOB__NM_000035	261	180	256	284
ALDOB__NM_000035_GGTGAGGGCTGGAAATCGGT	ALDOB__NM_000035	914	731	940	966
ALDOB__NM_000035_GTAAACATGATGGTCATTCA	ALDOB__NM_000035	116	217	215	194
ALDOB__NM_000035_TAAGAGAAACTTAGTTTCCA	ALDOB__NM_000035	129	212	167	115
ALDOB__NM_000035_TGCCCTCCAGGTAAACATGA	ALDOB__NM_000035	218	276	303	262
ALDOC__NM_005165_ATCCTGGATGGTTCGGACGA	ALDOC__NM_005165	235	110	334	158
ALDOC__NM_005165_CAAGCGGCTGAGCCAAATTG	ALDOC__NM_005165	756	502	604	303
ALDOC__NM_005165_CAGCACCATCCTTCTTGTAT	ALDOC__NM_005165	2543	3284	3023	3405
ALDOC__NM_005165_CCACGACGATGCCCTTATCC	ALDOC__NM_005165	1280	882	1249	828
ALDOC__NM_005165_CTCTGCTCTGCATAGCTATT	ALDOC__NM_005165	0	0	1	29
ALDOC__NM_005165_GAACCTGAAATATTGCCTGA	ALDOC__NM_005165	431	406	585	580
ALDOC__NM_005165_GACCGTGTGAAAAAGTGCAT	ALDOC__NM_005165	616	667	574	376
ALDOC__NM_005165_GATGGAGAAACCACCACTCA	ALDOC__NM_005165	568	470	700	410
ALDOC__NM_005165_GCCAAGCGGCTGAGCCAAAT	ALDOC__NM_005165	879	890	920	1084
ALDOC__NM_005165_GCCCGGGGCTACAATCCGCA	ALDOC__NM_005165	802	724	654	457
ALDOC__NM_005165_TCCCTTCGTCCGAACCATCC	ALDOC__NM_005165	367	448	498	296
ALDOC__NM_005165_TCCTGGATGGTTCGGACGAA	ALDOC__NM_005165	172	64	156	160
ALDOC__NM_005165_TGCCCTTATCCTGGATGGTT	ALDOC__NM_005165	495	239	521	322
ALG1__NM_019109_AACAACAGAATTCAGATTGT	ALG1__NM_019109	752	5	692	9
ALG1__NM_019109_ACAGCAATGCTAGGCAGACC	ALG1__NM_019109	152	28	270	17
ALG1__NM_019109_ACAGCGCGTGGTACTGCATA	ALG1__NM_019109	104	0	71	0
ALG1__NM_019109_AGCGCGTGGTACTGCATACG	ALG1__NM_019109	634	8	665	52
ALG1__NM_019109_CAAGAGCTCATCATGGGGTT	ALG1__NM_019109	674	87	792	318
ALG1__NM_019109_CAGCGCGTGGTACTGCATAC	ALG1__NM_019109	550	3	638	22
ALG1__NM_019109_GAACAACAGAATTCAGATTG	ALG1__NM_019109	379	66	405	112
ALG1__NM_019109_GCGCGTGGTACTGCATACGG	ALG1__NM_019109	595	0	583	0
ALG1__NM_019109_GCGCTGTCGTTGGCCATGCA	ALG1__NM_019109	2676	514	3088	704
ALG1__NM_019109_GTACAACTTTGACTCCGTAC	ALG1__NM_019109	631	163	755	174
ALG1__NM_019109_TGCTGTGGAAGTTGATGTGG	ALG1__NM_019109	486	175	529	138
ALG1__NM_019109_TGGAGAAAGATATAGGCACC	ALG1__NM_019109	68	23	59	6
ALG10__NM_032834_CATTGCACAAAAGTTAACGG	ALG10__NM_032834	721	649	740	686
ALG10__NM_032834_TGATCACTCCAATTGACACC	ALG10__NM_032834	172	156	237	288
ALG10B__NM_001013620_GAGGAGGCAGGACACTAAAA	ALG10B__NM_001013620	137	22	99	41
ALG10B__NM_001013620_TGACCACTCCAACTGACACC	ALG10B__NM_001013620	506	477	485	225
ALG10B__NM_001013620_TGTTTCTGGGTTGTACCTTG	ALG10B__NM_001013620	13	47	3	0
ALG11__NM_001004127_AATGTCTGCACATCACAAGG	ALG11__NM_001004127	257	5	347	7
ALG11__NM_001004127_AATTATCCTTGCACACAATT	ALG11__NM_001004127	359	358	397	353
ALG11__NM_001004127_AGGACAAGTTTAAGCGAAGG	ALG11__NM_001004127	231	55	221	110
ALG11__NM_001004127_CCCCCTATATACTTAAACAG	ALG11__NM_001004127	364	0	240	3
ALG11__NM_001004127_CTTCCTCTGTTTAAGTATAT	ALG11__NM_001004127	564	605	705	713
ALG11__NM_001004127_GAAAAATGGATCCTAGACTT	ALG11__NM_001004127	248	68	323	45
ALG11__NM_001004127_TATATAGGGGGTTGCCAAGT	ALG11__NM_001004127	192	7	180	0
ALG11__NM_001004127_TGGTTTCTGTTGGCCAGTTT	ALG11__NM_001004127	4	0	12	3
ALG11__NM_001004127_TGTTACGACAACCTCCAATG	ALG11__NM_001004127	219	0	147	2
ALG11__NM_001004127_TTAGGGTAAACCAACTGAGA	ALG11__NM_001004127	50	0	40	0
ALG11__NM_001004127_TTAGTGTCAACTAGCAAAAA	ALG11__NM_001004127	16	23	31	64
ALG11__NM_001004127_TTCTCTCACTATGGAAAGTT	ALG11__NM_001004127	12	30	8	0
ALG12__NM_024105_AAGGAAGTGAGACGGCACTT	ALG12__NM_024105	663	545	750	402
ALG12__NM_024105_ACCTGATGTTCTACTGCACG	ALG12__NM_024105	1346	1351	1781	2003
ALG12__NM_024105_AGCGAAAGCACGTAAACCGC	ALG12__NM_024105	562	396	619	568
ALG12__NM_024105_CACTGGCCCGAGGAACGTCC	ALG12__NM_024105	394	232	501	352
ALG12__NM_024105_CAGCGAAAGCACGTAAACCG	ALG12__NM_024105	570	425	702	541
ALG12__NM_024105_CATCTGTCCCTACACCAAAG	ALG12__NM_024105	390	297	419	264
ALG12__NM_024105_CTGTCCCTACACCAAAGTGG	ALG12__NM_024105	218	91	350	210
ALG12__NM_024105_CTTCGCCATCATCGTGTTCA	ALG12__NM_024105	0	0	0	0
ALG12__NM_024105_GCGAAAGCACGTAAACCGCG	ALG12__NM_024105	487	417	523	468
ALG12__NM_024105_GGAAGTGAGACGGCACTTCG	ALG12__NM_024105	607	760	745	780
ALG12__NM_024105_GGCCTGGCTGCGGCACGAGT	ALG12__NM_024105	1858	792	1818	1389
ALG12__NM_024105_TATTAGCTGAGAGTAAAACT	ALG12__NM_024105	49	41	62	39
ALG13__NM_001039210_AAGTGCGTGTTTGTTACCGT	ALG13__NM_001039210	1194	1	1063	4
ALG13__NM_001039210_AGTGCGTGTTTGTTACCGTA	ALG13__NM_001039210	960	0	1065	6
ALG13__NM_001039210_CAAACACGCACTTCATGGCG	ALG13__NM_001039210	670	792	666	835
ALG13__NM_001039210_CAGTACTGAGTCGTTTACTC	ALG13__NM_001039210	616	188	694	169
ALG13__NM_001039210_CGCAATGAGGTCGTCAAAGC	ALG13__NM_001039210	376	42	373	96
ALG13__NM_001039210_GGCAAAGCAGCTACACAAAG	ALG13__NM_001039210	347	465	348	291
ALG13__NM_001039210_GGTAACAAACACGCACTTCA	ALG13__NM_001039210	446	182	527	113
ALG13__NM_001039210_TACCAATTTGCAGGATAAGT	ALG13__NM_001039210	475	0	656	33
ALG13__NM_001039210_TCGTTTATAACCACTACGAG	ALG13__NM_001039210	272	369	406	503
ALG13__NM_001099922_GATCTTGTTATTAGTCACGC	ALG13__NM_001099922	387	19	446	5
ALG13__NM_001099922_GGCTTGCTTATGCAGGTATC	ALG13__NM_001099922	513	690	571	729
ALG13__NM_001099922_TCTGGATGTTTACAGGTACA	ALG13__NM_001099922	434	30	454	22
ALG13__NM_001257230_AGATAATGGCTATGAAGACA	ALG13__NM_001257230	113	81	148	227
ALG13__NM_001257230_CAATGCTGAAAATATACCAG	ALG13__NM_001257230	615	816	743	731
ALG13__NM_001257230_CTACTCTGCTACTCAAGTAG	ALG13__NM_001257230	204	159	224	168
ALG13__NM_001257230_GCTCCGAAATGGCCCGAAAG	ALG13__NM_001257230	183	257	168	129
ALG13__NM_001257230_TAACAAGGGCTTGCTTATGC	ALG13__NM_001257230	225	331	300	427
ALG13__NM_001257230_TAGGATGGGTAGCAGTAACA	ALG13__NM_001257230	137	149	145	173
[illegible]	[illegible]	[illegible]	[illegible]	[illegible]	[illegible]
ALG14__NM_144988_CTCCCGGGGCGTAACGTCCA	ALG14__NM_144988	467	311	497	224
ALG14__NM_144988_GAAGCACTACCCATATTCGC	ALG14__NM_144988	627	487	699	371
ALG14__NM_144988_GGCTTCACCCTGTGAATTAG	ALG14__NM_144988	346	357	371	285
ALG14__NM_144988_GTTCCATGGACGTTACGCCC	ALG14__NM_144988	60	69	59	26
ALG14__NM_144988_TCACAGGGTGAAGCCAGATT	ALG14__NM_144988	481	61	608	30
ALG14__NM_144988_TCTCCTTTCCCCTAATTCAC	ALG14__NM_144988	241	6	385	29
ALG14__NM_144988_TGATAGAGACCCTAGTAACA	ALG14__NM_144988	333	134	350	62
ALG14__NM_144988_TGGCTTCACCCTGTGAATTA	ALG14__NM_144988	17	5	21	7
ALG14__NM_144988_TTTCCTAATCCTGCGAATAT	ALG14__NM_144988	25	58	59	38
ALG1L__NM_001015050_AGCTTCTTCTCGGAGTCTCA	ALG1L__NM_001015050	131	11	113	23
ALG1L__NM_001015050_TGGTGATGCATCTCCGCGAG	ALG1L__NM_001015050	12	115	31	41
ALG2__NM_033087_AGCTACCGGTGCGCTGTGCC	ALG2__NM_033087	1434	756	1414	1057
ALG2__NM_033087_ATTCCTCTATCCAGTCAATT	ALG2__NM_033087	427	82	338	58
ALG2__NM_033087_CGACGTGGTAGTGTGCGACC	ALG2__NM_033087	1898	39	2203	209
ALG2__NM_033087_CGCGGCTCTCGGCGAAACAG	ALG2__NM_033087	532	0	533	6
ALG2__NM_033087_CTGGACAGCGCACTACGACC	ALG2__NM_033087	901	168	1018	192
ALG2__NM_033087_GGACTCGGTTCCCAAGCCGT	ALG2__NM_033087	236	127	250	150
ALG2__NM_033087_GGGTGGAGGAACAGCACCGA	ALG2__NM_033087	540	82	517	137
ALG2__NM_033087_GTCTAGCCAGCCTGAACACT	ALG2__NM_033087	1028	82	1115	76
ALG2__NM_033087_TATACAGGGCCCCAATTGAC	ALG2__NM_033087	799	0	946	9
ALG2__NM_033087_TATTCCTCTATCCAGTCAAT	ALG2__NM_033087	202	20	187	48
ALG2__NM_033087_TGTTCAGGCTGGCTAGACGG	ALG2__NM_033087	249	30	309	26
ALG2__NM_033087_TTCCTCTATCCAGTCAATTG	ALG2__NM_033087	22	0	27	0
ALG3__NM_001006941_AAGATGTACACGAAACCAGC	ALG3__NM_001006941	257	318	316	320
ALG3__NM_001006941_AGACAAGCAGCAAGGTAGCC	ALG3__NM_001006941	38	66	37	55
ALG3__NM_001006941_AGACACAGAGATTGACTGGA	ALG3__NM_001006941	345	174	381	361
ALG3__NM_001006941_AGATGTACACGAAACCAGCT	ALG3__NM_001006941	482	323	386	325
ALG3__NM_001006941_ATTGATGACGCCTTCTACCT	ALG3__NM_001006941	640	718	886	731
ALG3__NM_001006941_CCAGCAGGAGGTTGATACTG	ALG3__NM_001006941	386	523	625	707
ALG3__NM_001006941_GATGGAGTGGACACGGTAAG	ALG3__NM_001006941	261	304	300	350
ALG3__NM_001006941_GCATTCTCCTTCGCCTGCGC	ALG3__NM_001006941	633	840	717	1001
ALG3__NM_001006941_GCTGGCTTCAGCGGTTGTGG	ALG3__NM_001006941	2226	2051	2338	2106
ALG3__NM_001006941_GGTTTCGTGTACATCTTTAT	ALG3__NM_001006941	347	633	489	584
ALG3__NM_001006941_TGGTTTCGTGTACATCTTTA	ALG3__NM_001006941	0	0	0	0
ALG3__NM_001006941_TTTCTTCTCCTCACACAGTT	ALG3__NM_001006941	109	426	216	271
ALG3__NM_005787_GCCACCAGCAGCGTGTAGCG	ALG3__NM_005787	394	476	334	426
ALG3__NM_005787_TCACCTTCTGGGTCATTCAC	ALG3__NM_005787	351	232	295	446
ALG5__NM_001142364_AAAAGGTGGAGCGATTAGAA	ALG5__NM_001142364	0	0	5	5
ALG5__NM_001142364_AAGAAACTTTACCCAGCATA	ALG5__NM_001142364	0	0	0	0
ALG5__NM_001142364_ACCCATACATGAGAAGAGTA	ALG5__NM_001142364	358	157	361	157
ALG5__NM_001142364_ACGACAGAAAGTTGTTTGGT	ALG5__NM_001142364	1574	785	1644	1097
ALG5__NM_001142364_AGAGCTTCATCCATCATCAC	ALG5__NM_001142364	535	270	597	296
ALG5__NM_001142364_ATAACCCTGGTGAAGAATCG	ALG5__NM_001142364	295	296	340	325
ALG5__NM_001142364_CAGGGTATATTCAGTTCTCG	ALG5__NM_001142364	862	1008	999	924
ALG5__NM_001142364_CATCTCTACACGTTGAACGA	ALG5__NM_001142364	836	549	817	562
ALG5__NM_001142364_CCTTTGTGTCAAAGGAATCA	ALG5__NM_001142364	232	148	281	266
ALG5__NM_001142364_CTCCATCAGCATCTGCCATA	ALG5__NM_001142364	349	187	462	309
ALG5__NM_001142364_GGTAGGTGAGTCCCATATGC	ALG5__NM_001142364	209	194	258	106
ALG5__NM_001142364_TCTCACCAGTACGAGGGCTG	ALG5__NM_001142364	201	235	246	316
ALG5__NM_013338_CAGTAAAGATCAGACCTCAA	ALG5__NM_013338	395	141	364	294
ALG6__NM_013339_AAGCTCTTCATGCGTACAAC	ALG6__NM_013339	60	68	85	68
ALG6__NM_013339_AGACTATGATAAGCTGTAAG	ALG6__NM_013339	616	506	566	386
ALG6__NM_013339_ATGGTACTTGATGACAGTAG	ALG6__NM_013339	225	228	170	157
ALG6__NM_013339_ATTGCTCTCCATACATCACG	ALG6__NM_013339	216	88	202	191
ALG6__NM_013339_CAAAGTTTATAAATCCAGAC	ALG6__NM_013339	429	562	444	755
ALG6__NM_013339_GCTGGTAAACCGCCTATGTT	ALG6__NM_013339	683	421	846	577
ALG6__NM_013339_GGCCTTATTCTTATAGACTA	ALG6__NM_013339	252	446	302	378
ALG6__NM_013339_GTGTCTCTTAATTCTTATTC	ALG6__NM_013339	29	38	39	49
ALG6__NM_013339_TAATAGGACTAACAGTACGA	ALG6__NM_013339	197	55	179	85
ALG6__NM_013339_TCTCATATCCACGTGATGTA	ALG6__NM_013339	269	168	342	188
ALG6__NM_013339_TTAATTTACCGGTCAAACAA	ALG6__NM_013339	38	48	58	70
ALG6__NM_013339_TTATGCATCTTGCTGTATCC	ALG6__NM_013339	252	113	407	309
ALG8__NM_001007027_AAACCAATTGCCAGTACCCG	ALG8__NM_001007027	775	586	848	663
ALG8__NM_001007027_AAGAGTCGGGAAAAGACTTG	ALG8__NM_001007027	275	185	242	137
ALG8__NM_001007027_AGCAGATATACACCATAAGC	ALG8__NM_001007027	205	107	269	169
ALG8__NM_001007027_CATAAGCTGGTGCTACATAG	ALG8__NM_001007027	498	350	507	221
ALG8__NM_001007027_CCGACTCTTTCCTTTCAAGA	ALG8__NM_001007027	660	836	803	947
ALG8__NM_001007027_CTGTAGATGGGTCTATTCGA	ALG8__NM_001007027	568	379	723	554
ALG8__NM_001007027_CTTCTCATCCCCACATAGTA	ALG8__NM_001007027	344	150	370	282
ALG8__NM_001007027_GAACTTCGGGTTATTAATTG	ALG8__NM_001007027	160	102	120	82
ALG8__NM_001007027_GGGCCTCTGTCATGCATATT	ALG8__NM_001007027	675	1023	783	858
ALG8__NM_001007027_TACCTGAAATAATCGTGCAA	ALG8__NM_001007027	592	540	637	766
ALG8__NM_001007027_TGTTTCACTGCAAATAAACC	ALG8__NM_001007027	32	5	23	11
ALG8__NM_001007027_TTGCCACGGGTACTGGCAAT	ALG8__NM_001007027	303	231	231	241
ALG9__NM_001077690_ACATACAGAAGCTACTAGGA	ALG9__NM_001077690	764	580	847	619
ALG9__NM_001077690_ACTACGTTGATAGCCATGAC	ALG9__NM_001077690	410	344	450	405
ALG9__NM_001077690_ACTCACCAGAAATAGTATGA	ALG9__NM_001077690	265	222	341	253
ALG9__NM_001077690_ATGAAGCAACAGGTAAGCAT	ALG9__NM_001077690	190	108	234	198
ALG9__NM_001077690_ATGGGGAAGGGTTTCAGACT	ALG9__NM_001077690	432	242	443	148
ALG9__NM_001077690_CAGCTGGGGCTATCTTAGGC	ALG9__NM_001077690	685	689	798	874
ALG9__NM_001077690_CAGGTAAGCATAGGAGCGAA	ALG9__NM_001077690	112	42	131	191
ALG9__NM_001077690_GTCCATATACCATCCAGTCA	ALG9__NM_001077690	598	494	673	524
ALG9__NM_001077690_GTGATGAAACATTCAACTAC	ALG9__NM_001077690	191	235	196	223
ALG9__NM_001077690_GTGTACATACAGAAGCTACT	ALG9__NM_001077690	177	197	211	239
ALG9__NM_001077690_TATAATGTCTTTACTCCTCA	ALG9__NM_001077690	382	482	426	660
ALG9__NM_001077690_TGGGGAAGGGTTTCAGACTT	ALG9__NM_001077690	130	92	202	141
ALG9__NM_001077691_AGAATTTAGGCCACCCGTAT	ALG9__NM_001077691	346	428	443	503
[illegible]	[illegible]	[illegible]	[illegible]	[illegible]	[illegible]
ALK__NM_004304_CTGGGAGGCGCGAATTGCCG	ALK__NM_004304	940	740	972	801
ALK__NM_004304_CTGTCTCATCGCAGCCGATA	ALK__NM_004304	60	68	63	52
ALK__NM_004304_GCACCAACTGCTTGGCAGC	ALK__NM_004304	1242	1499	1417	1716
ALK__NM_004304_GCCAAGGCGAAGGCGACTG	ALK__NM_004304	394	461	472	477
ALK__NM_004304_GCTCCGGCGTGCCAAGCAGT	ALK__NM_004304	491	370	519	490
ALK__NM_004304_GCTCTTCCGTGTCTACGCCC	ALK__NM_004304	400	345	365	219
ALK__NM_004304_GGCGAGGAGGCGATCTTGGA	ALK__NM_004304	1332	1354	1530	1577
ALK__NM_004304_TCAGCGAGCTGTTCAGTTGG	ALK__NM_004304	552	407	568	463
ALK__NM_004304_TTCAGTTGGTGGATTCGCCA	ALK__NM_004304	55	84	75	69
ALKBH1__NM_006020_ACCCAGTGCCACTGGTAACC	ALKBH1__NM_006020	646	836	679	680
ALKBH1__NM_006020_ACGGAGACCCCGAAGTTTAC	ALKBH1__NM_006020	184	283	274	384
ALKBH1__NM_006020_AGTTTCTCCAGTAAACTTCG	ALKBH1__NM_006020	23	80	23	36
ALKBH1__NM_006020_CCCAGTGCCACTGGTAACCT	ALKBH1__NM_006020	532	423	609	724
ALKBH1__NM_006020_CCTTCCTCCCAGGTTACCAG	ALKBH1__NM_006020	456	474	482	571
ALKBH1__NM_006020_GAAACCTAATGTATGTAACC	ALKBH1__NM_006020	240	215	241	243
ALKBH1__NM_006020_GAGCAGAATGCATATAGAGC	ALKBH1__NM_006020	296	316	367	413
ALKBH1__NM_006020_GGTATAAAGAAGCGACTAAA	ALKBH1__NM_006020	103	189	110	79
ALKBH1__NM_006020_GTCTTCAGCCCGTCAGCAAG	ALKBH1__NM_006020	105	109	102	124
ALKBH1__NM_006020_GTTTCTGGGAATATAACTTA	ALKBH1__NM_006020	55	141	49	166
ALKBH1__NM_006020_TAGGCTTGCCACTTGCTGAC	ALKBH1__NM_006020	812	913	922	940
ALKBH1__NM_006020_TTGTCCAGGTTACATACATT	ALKBH1__NM_006020	532	619	523	689
ALKBH2__NM_001001655_AAAGATGGCTGTGACCACAT	ALKBH2__NM_001001655	199	79	188	100
ALKBH2__NM_001001655_AGGCAACGTATGGCGACGCT	ALKBH2__NM_001001655	741	740	827	922
ALKBH2__NM_001001655_CAGGCAACGTATGGCGACGC	ALKBH2__NM_001001655	976	1225	981	955
ALKBH2__NM_001001655_CATCATTAGTAAGCTCCCGT	ALKBH2__NM_001001655	755	587	844	593
ALKBH2__NM_001001655_CCCAGGAAGCAGGCAACGTA	ALKBH2__NM_001001655	200	185	178	234
ALKBH2__NM_001001655_CTGGCCAGAGTCCAGGTATT	ALKBH2__NM_001001655	519	658	783	831
ALKBH2__NM_001001655_GAGTCCAGGTATTCGGGAAG	ALKBH2__NM_001001655	411	221	351	420
ALKBH2__NM_001001655_GCCCTAGCTGGCGGCACATT	ALKBH2__NM_001001655	2246	2420	2618	2552
ALKBH2__NM_001001655_TCACCCCAGAGACGTGATCC	ALKBH2__NM_001001655	707	866	935	903
ALKBH2__NM_001001655_TCCCACGGGAATCCTTATGC	ALKBH2__NM_001001655	1735	1547	1885	1049
ALKBH2__NM_001001655_TCCCGGATGCGCTCTAGAAC	ALKBH2__NM_001001655	359	375	297	635
ALKBH2__NM_001001655_TGCAGTTACACAGTCCTGTT	ALKBH2__NM_001001655	628	869	817	781
ALKBH3__NM_139178_AACTTAGTGAAGCAATAATG	ALKBH3__NM_139178	310	444	364	357
ALKBH3__NM_139178_AGCAACCAAGACTTACAGCA	ALKBH3__NM_139178	910	1005	989	1292
ALKBH3__NM_139178_ATGATGAACCCTCACTAGGG	ALKBH3__NM_139178	135	142	298	226
ALKBH3__NM_139178_ATGGGGCACCTCCCTAGTGA	ALKBH3__NM_139178	590	467	572	540
ALKBH3__NM_139178_ATTGCAGAGTAAGGAGTTGA	ALKBH3__NM_139178	590	583	589	638
ALKBH3__NM_139178_CAATCTTTATCGCAATGAGA	ALKBH3__NM_139178	467	558	525	683
ALKBH3__NM_139178_GAAAGAAGCTGACTGGATAT	ALKBH3__NM_139178	687	790	797	823
ALKBH3__NM_139178_GCGATAAAGATTGCAGAGTA	ALKBH3__NM_139178	307	426	402	466
ALKBH3__NM_139178_GGCTGCCCCTGTTAAAAGCC	ALKBH3__NM_139178	322	412	313	510
ALKBH3__NM_139178_GTGATGATGAACCCTCACTA	ALKBH3__NM_139178	344	319	337	233
ALKBH3__NM_139178_GTGATTCTTGAATAAGTGTA	ALKBH3__NM_139178	100	49	82	33
ALKBH3__NM_139178_TGTCACCCACAGGTGTATCT	ALKBH3__NM_139178	26	16	23	7
ALKBH4__NM_017621_AAACAGAAGCTAAAGACCGA	ALKBH4__NM_017621	84	106	164	131
ALKBH4__NM_017621_AGCTCTCCCAGTCTGGACGG	ALKBH4__NM_017621	232	265	267	203
ALKBH4__NM_017621_CCTCCTGGCATAGCACCGAC	ALKBH4__NM_017621	314	179	309	300
ALKBH4__NM_017621_CCTTCGGGAATGCGGTTGCA	ALKBH4__NM_017621	479	860	502	538
ALKBH4__NM_017621_CCTTGCAACCGCATTCCCGA	ALKBH4__NM_017621	1734	1890	2105	2215
ALKBH4__NM_017621_CGTGATAGCACCCAGCCGGT	ALKBH4__NM_017621	1388	1422	1422	1319
ALKBH4__NM_017621_CTGCTTCCTCCGTCCAGACT	ALKBH4__NM_017621	922	974	1049	744
ALKBH4__NM_017621_GGACCTGTCTGATCTGCGAG	ALKBH4__NM_017621	758	659	801	734
ALKBH4__NM_017621_TGGCATAGCACCGACCGGCT	ALKBH4__NM_017621	16	24	44	5
ALKBH5__NM_017758_CCACCGTGTGCTCGTTGTAC	ALKBH5__NM_017758	3371	3299	3796	3226
ALKBH5__NM_017758_CCCCGAGCGCAGCGACTATG	ALKBH5__NM_017758	295	192	324	368
ALKBH5__NM_017758_CGGGGCCAAGCGCAAGTATC	ALKBH5__NM_017758	337	424	365	350
ALKBH5__NM_017758_GATCGAGGCCCGCATTGACG	ALKBH5__NM_017758	862	829	725	814
ALKBH5__NM_017758_GGACACCACCTCGTCAATGC	ALKBH5__NM_017758	1541	1391	1533	1259
ALKBH5__NM_017758_GGCCAAGCGCAAGTATCAGG	ALKBH5__NM_017758	1770	1564	1911	1671
ALKBH5__NM_017758_GGGACACCACCTCGTCAATG	ALKBH5__NM_017758	832	1020	1040	1152
ALKBH5__NM_017758_GGTGGTGTCCCGCGCTGAGA	ALKBH5__NM_017758	216	329	200	221
ALKBH5__NM_017758_GTCCCGGGACAACTATAAGG	ALKBH5__NM_017758	86	73	88	52
ALKBH5__NM_017758_GTCCTCCTGATACTTGCGCT	ALKBH5__NM_017758	650	747	782	948
ALKBH5__NM_017758_TCCCGGGACAACTATAAGGC	ALKBH5__NM_017758	225	167	211	91
ALKBH5__NM_017758_TGCCCGCCTTATAGTTGTCC	ALKBH5__NM_017758	503	731	494	302
ALKBH6__NM_032878_CAGGCAGATACTGGTTCACG	ALKBH6__NM_032878	1291	1315	1508	1674
ALKBH6__NM_032878_CCAGAGAGCTGGGTCCACTT	ALKBH6__NM_032878	1427	1370	1818	1935
ALKBH6__NM_032878_CGAGGACATGGTTAGCTGGG	ALKBH6__NM_032878	576	397	685	797
ALKBH6__NM_032878_CGCTCAGGAACCATCCCTCG	ALKBH6__NM_032878	1210	1572	1509	1457
ALKBH6__NM_032878_CGTGAACCAGTATCTGCCTG	ALKBH6__NM_032878	716	759	931	969
ALKBH6__NM_032878_GGAGGAGTATTTGCTTCGAC	ALKBH6__NM_032878	550	850	524	637
ALKBH6__NM_032878_GGGACATAGTAGATTACAGG	ALKBH6__NM_032878	151	138	177	103
ALKBH6__NM_032878_GGTCCGTCCTCGTGGGGCTA	ALKBH6__NM_032878	361	280	390	423
ALKBH6__NM_032878_TCAGGGACATAGTAGATTAC	ALKBH6__NM_032878	255	287	230	172
ALKBH6__NM_032878_TGCCCTCCCCAGGCAGATAC	ALKBH6__NM_032878	399	351	338	435
ALKBH6__NM_032878_TGGGAGAAAGTTACAGAACT	ALKBH6__NM_032878	22	34	47	64
ALKBH6__NM_032878_TTCACGAGGACATGGTTAGC	ALKBH6__NM_032878	21	43	20	17
ALKBH6__NM_198867_TGCTGGACTTCTACGAGCCG	ALKBH6__NM_198867	454	580	486	603
ALKBH7__NM_032306_AGTGATCGTATTCGTAGCGG	ALKBH7__NM_032306	477	541	582	524
ALKBH7__NM_032306_CAGGTCCAGCACGTGCACGG	ALKBH7__NM_032306	1102	961	1090	974
ALKBH7__NM_032306_CCCAGTGATCGTATTCGTAG	ALKBH7__NM_032306	1215	901	1264	1006
ALKBH7__NM_032306_CCGCTACGAATACGATCACT	ALKBH7__NM_032306	1080	719	1228	979
ALKBH7__NM_032306_GCAGGCGGCTCAGCACGGAA	ALKBH7__NM_032306	346	368	434	522
ALKBH7__NM_032306_GCCGCTACGAATACGATCAC	ALKBH7__NM_032306	1313	1112	1378	1468
ALKBH7__NM_032306_GGAGACGCTGAGCCGAGAAC	ALKBH7__NM_032306	478	664	549	689
ALKBH7__NM_032306_GTCTCCCAGCGTTATGCGGC	ALKBH7__NM_032306	380	477	452	478
[illegible]	[illegible]	[illegible]	[illegible]	[illegible]	[illegible]
ALKBH8__NM_138775_GATAAAGATAAGCCATTATC	ALKBH8__NM_138775	232	212	218	298
ALKBH8__NM_138775_GCAAATGAGTACGGCTTGTT	ALKBH8__NM_138775	1268	1415	1301	1420
ALKBH8__NM_138775_GGAATGTGTATCAATATGAG	ALKBH8__NM_138775	283	456	372	463
ALKBH8__NM_138775_GTTTGGGTAATGGTGTGAGT	ALKBH8__NM_138775	161	126	148	98
ALLC__NM_018436_AGCATGAATATACGGAGTTT	ALLC__NM_018436	303	353	405	307
ALLC__NM_018436_ATCCGACGACTGGAGTTACT	ALLC__NM_018436	856	761	986	795
ALLC__NM_018436_ATGGCATCTGAATCCGTAGG	ALLC__NM_018436	86	63	129	159
ALLC__NM_018436_ATTGCACGACTTAGAGTATT	ALLC__NM_018436	794	750	801	705
ALLC__NM_018436_CCTGGCTTAAGCTCAGTCAT	ALLC__NM_018436	1826	1626	2159	1967
ALLC__NM_018436_CGACTTAGAGTATTCGGTAC	ALLC__NM_018436	492	638	505	655
ALLC__NM_018436_GACATGGCATCTGAATCCGT	ALLC__NM_018436	706	755	603	531
ALLC__NM_018436_GAGCATGAATATACGGAGTT	ALLC__NM_018436	477	781	495	478
ALLC__NM_018436_GGATTTAGTAATGCTAAGTT	ALLC__NM_018436	396	390	427	581
ALLC__NM_018436_GTGGACGTTTCTTACTTCAC	ALLC__NM_018436	170	187	206	89
ALLC__NM_018436_TCCTGGCTTAAGCTCAGTCA	ALLC__NM_018436	455	601	522	526
ALLC__NM_018436_TGGGGATCCAAGGAGTCATC	ALLC__NM_018436	111	84	97	125
ALMS1__NM_015120_CCGCAGCACCGCTACTCGGA	ALMS1__NM_015120	1350	1018	1264	1054
ALMS1__NM_015120_CGTGGACGACGTAGTGGTCG	ALMS1__NM_015120	606	679	539	597
ALMS1__NM_015120_CTGGGGCCCGTAGTGAGAGT	ALMS1__NM_015120	296	179	336	330
ALMS1__NM_015120_GACTAGGTTTAATGTGAGAA	ALMS1__NM_015120	182	253	231	229
ALMS1__NM_015120_GCCCCCGCAGCACCGCTACT	ALMS1__NM_015120	1053	1118	1273	1262
ALMS1__NM_015120_GGCGAACGTGGACGACGTAG	ALMS1__NM_015120	339	245	330	369
ALMS1__NM_015120_TCCGCTCGCCCTCCGAGTAG	ALMS1__NM_015120	763	720	866	786
ALMS1__NM_015120_TCTGGAAAGTATAGACGACG	ALMS1__NM_015120	237	284	301	268
ALMS1__NM_015120_TCTGGGATGTATCCAAGGTT	ALMS1__NM_015120	573	404	543	592
ALMS1__NM_015120_TGGACTCCGACTCTCACTAC	ALMS1__NM_015120	76	47	76	49
ALMS1__NM_015120_TGGCAACAGATAGTATATCA	ALMS1__NM_015120	65	71	93	98
ALMS1__NM_015120_TTCTCACATTAAACCTAGTC	ALMS1__NM_015120	15	144	10	4
ALOX12__NM_000697_AATGTAGGGGGTTTAACTGG	ALOX12__NM_000697	228	243	229	298
ALOX12__NM_000697_ACTGGCTGGTGGACGACGCG	ALOX12__NM_000697	3305	4140	3653	4595
ALOX12__NM_000697_CCCCGAGGGCAGCACTAGCC	ALOX12__NM_000697	27	0	50	86
ALOX12__NM_000697_CGAGGGCAGCACTAGCCTGG	ALOX12__NM_000697	302	638	285	521
ALOX12__NM_000697_CGGTCTGCAAAGCGTAAAAA	ALOX12__NM_000697	73	141	148	102
ALOX12__NM_000697_CTGCACGCGGTTGTACGACC	ALOX12__NM_000697	317	307	298	396
ALOX12__NM_000697_CTTTGGCTGGTCGGGACGCG	ALOX12__NM_000697	583	682	504	914
ALOX12__NM_000697_GAGGGTGTAAACACGTTTGA	ALOX12__NM_000697	457	441	353	281
ALOX12__NM_000697_GCATGGGGTTGGCACCATTG	ALOX12__NM_000697	1057	1216	1060	1147
ALOX12__NM_000697_GCTAGTGGGATACTAATGTA	ALOX12__NM_000697	508	574	648	723
ALOX12__NM_000697_GGAGGGTGTAAACACGTTTG	ALOX12__NM_000697	559	570	565	585
ALOX12__NM_000697_GGCTAGTGGGATACTAATGT	ALOX12__NM_000697	663	789	787	860
ALOX12__NM_000697_GTCGAGCGTCTCAACAGCAT	ALOX12__NM_000697	480	509	694	708
ALOX12__NM_000697_TAGTGGGATACTAATGTAGG	ALOX12__NM_000697	151	242	209	200
ALOX12__NM_000697_TCCAAATATGAGATTCCATG	ALOX12__NM_000697	332	444	417	465
ALOX12__NM_000697_TCTCCTCGGATCACGTTGGC	ALOX12__NM_000697	673	987	693	944
ALOX12__NM_000697_TTTGAATGGACACTGAAGGC	ALOX12__NM_000697	513	556	572	796
ALOX12B__NM_001139_AAGTGGTAGATACGGCCGTT	ALOX12B__NM_001139	1019	905	1205	1214
ALOX12B__NM_001139_AGTGGTAGATACGGCCGTTG	ALOX12B__NM_001139	778	751	876	914
ALOX12B__NM_001139_CAGGTGGAATGGCTATATTC	ALOX12B__NM_001139	211	349	209	225
ALOX12B__NM_001139_CATGGCCACCTACAAAGTCA	ALOX12B__NM_001139	585	531	692	504
ALOX12B__NM_001139_CCGCACCGGAGGGCGGTAAC	ALOX12B__NM_001139	22	8	23	7
ALOX12B__NM_001139_CCGTGTTCCCGACAAGAGGT	ALOX12B__NM_001139	748	872	789	761
ALOX12B__NM_001139_CCTCCTGGAGCACAGAAAAG	ALOX12B__NM_001139	293	270	293	293
ALOX12B__NM_001139_CGCACCGGAGGGCGGTAACT	ALOX12B__NM_001139	177	71	127	63
ALOX12B__NM_001139_GGCCACCCTGACTTTGTAGG	ALOX12B__NM_001139	691	1070	804	1058
ALOX12B__NM_001139_GTGGTAGATACGGCCGTTGG	ALOX12B__NM_001139	49	1	37	44
ALOX12B__NM_001139_TGCACATAGTTGCAGTACCA	ALOX12B__NM_001139	471	444	520	494
ALOX12B__NM_001139_TGGGAGAGACTTTGCAACTG	ALOX12B__NM_001139	265	181	385	244
ALOX15__NM_001140_ACCTCCTTAAGGACGACGCC	ALOX15__NM_001140	620	749	729	787
ALOX15__NM_001140_ACTGGGGCCTCGCTCTATGC	ALOX15__NM_001140	1074	1149	1100	1127
ALOX15__NM_001140_ACTGTGGCCCGCACGGGGCA	ALOX15__NM_001140	98	191	141	153
ALOX15__NM_001140_CAGGTTCCCTTGTTACCGCT	ALOX15__NM_001140	337	259	403	273
ALOX15__NM_001140_CCACAGCTGCACCTGGTTGT	ALOX15__NM_001140	2186	2492	2201	2290
ALOX15__NM_001140_CCCTCCACCCAGCGGTAACA	ALOX15__NM_001140	601	275	600	332
ALOX15__NM_001140_CTATGCCGGTTCCAACAACC	ALOX15__NM_001140	141	118	114	219
ALOX15__NM_001140_CTGCACCTGGTTGTTGGAAC	ALOX15__NM_001140	357	451	432	558
ALOX15__NM_001140_GGGTTAATTCTGAATATGGC	ALOX15__NM_001140	445	417	437	586
ALOX15__NM_001140_GGTGGAAGTACCGGAGTATC	ALOX15__NM_001140	276	262	227	171
ALOX15__NM_001140_GTTGGAACCGGCATAGAGCG	ALOX15__NM_001140	40	76	42	32
ALOX15__NM_001140_TGTTTGTGAAACTGCGCAAA	ALOX15__NM_001140	70	66	100	147
ALOX15B__NM_001039130_AAAGGATCTTCAACTTCCGG	ALOX15B__NM_001039130	304	371	355	469
ALOX15B__NM_001039130_AGCCTTCGGGGCTGGCACAT	ALOX15B__NM_001039130	993	874	1262	1147
ALOX15B__NM_001039130_AGGTGGAAGGCTTACAACCC	ALOX15B__NM_001039130	81	102	91	74
ALOX15B__NM_001039130_CGGCAGGAGATGTACCAGTG	ALOX15B__NM_001039130	739	779	839	899
ALOX15B__NM_001039130_GAAGGGCTCCCTGTTCTTGG	ALOX15B__NM_001039130	788	786	784	555
ALOX15B__NM_001039130_GCCAGCCTGTAGATAAAAGT	ALOX15B__NM_001039130	808	1226	960	1390
ALOX15B__NM_001039130_GCCATGGTGGCCTCAGTGTT	ALOX15B__NM_001039130	676	728	693	827
ALOX15B__NM_001039130_GCCCCTGGACAATCTCGGCA	ALOX15B__NM_001039130	1744	2082	1839	2126
ALOX15B__NM_001039130_GGAAGGCTTACAACCCAGGT	ALOX15B__NM_001039130	415	492	462	626
ALOX15B__NM_001039130_GGAGTCTGAATGAGATGAAA	ALOX15B__NM_001039130	213	213	218	294
ALOX15B__NM_001039130_TGACAGCGGCGGATCAGGAC	ALOX15B__NM_001039130	227	296	185	237
ALOX15B__NM_001039130_TTCAGGGTCAGGGTGTCCAC	ALOX15B__NM_001039130	8	6	8	1
ALOX5__NM_000698_ACTGGCAGCCAGTGGTTCGC	ALOX5__NM_000698	1251	1033	1284	1121
ALOX5__NM_000698_AGTACTGGCTGAATGACGAC	ALOX5__NM_000698	408	630	550	561
[illegible]	[illegible]	[illegible]	[illegible]	[illegible]	[illegible]
ALOX5AP__NM_001204406_AGCACCCTGGCTACATATT	ALOX5AP__NM_001204406	1512	1020	1365	1178
ALOX5AP__NM_001204406_AGGATTCTTTGCCCATAAAG	ALOX5AP__NM_001204406	35	0	45	27
ALOX5AP__NM_001204406_AGTTAGGGAATGAGAAGTAG	ALOX5AP__NM_001204406	576	479	528	478
ALOX5AP__NM_001204406_ATATGCCAGCAACGGACATG	ALOX5AP__NM_001204406	615	640	672	657
ALOX5AP__NM_001204406_CAGAGCACAGCGAGGAAAGT	ALOX5AP__NM_001204406	415	293	552	590
ALOX5AP__NM_001204406_GCACCCCTGGCTACATATTT	ALOX5AP__NM_001204406	2066	2000	2076	1914
ALOX5AP__NM_001204406_TGGGAAATCCTTGGCACCT	ALOX5AP__NM_001204406	163	191	161	146
ALOX5AP__NM_001204406_TTAGTTCCTGCTGCGTTTGC	ALOX5AP__NM_001204406	96	151	133	192
ALOX5AP__NM_001204406_TTTCCCAAATATGTAGCCAG	ALOX5AP__NM_001204406	194	140	136	211
ALOX5AP__NM_001204406_TTTGGCACCTTGGGGCACAT	ALOX5AP__NM_001204406	326	413	385	484
ALOXE3__NM_001165960_AAATACAGCGCCGGCAAACA	ALOXE3__NM_001165960	256	198	231	230
ALOXE3__NM_001165960_AGTATTAGAGTGCAAGACTG	ALOXE3__NM_001165960	264	365	397	220
ALOXE3__NM_001165960_CAAGCAGCGGCTAGATCGAA	ALOXE3__NM_001165960	235	266	246	306
ALOXE3__NM_001165960_CAGGGTCTGAATGCCCCGCG	ALOXE3__NM_001165960	735	682	811	761
ALOXE3__NM_001165960_CCAGAGCAGAGAATGAGGTC	ALOXE3__NM_001165960	667	510	685	493
ALOXE3__NM_001165960_GAAATACAGCGCCGGCAAAC	ALOXE3__NM_001165960	308	429	323	571
ALOXE3__NM_001165960_GAAGAGCGGCACGCCGGACA	ALOXE3__NM_001165960	819	591	900	849
ALOXE3__NM_001165960_GCGGCTAGATCGAATGGGCA	ALOXE3__NM_001165960	307	412	440	442
ALOXE3__NM_001165960_GGCTCACCCAGATGGATATC	ALOXE3__NM_001165960	367	358	446	499
ALOXE3__NM_001165960_GTATTAGAGTGCAAGACTGT	ALOXE3__NM_001165960	628	554	735	834
ALOXE3__NM_001165960_GTGCGTTGCACAGCGGAGCT	ALOXE3__NM_001165960	0	0	0	0
ALOXE3__NM_001165960_GTTTCTGGGCGGAGGGCTAG	ALOXE3__NM_001165960	224	135	233	198
ALOXE3__NM_021628_AAGTGGGATACACTACCATC	ALOXE3__NM_021628	295	362	303	313
ALOXE3__NM_021628_ATCTGTGTCACCGAACCGGA	ALOXE3__NM_021628	315	301	314	318
ALOXE3__NM_021628_CACAGCAAGAACTATTTGTC	ALOXE3__NM_021628	1301	1343	1371	1745
ALOXE3__NM_021628_CCGCATCTGTGTCACCGAAC	ALOXE3__NM_021628	1243	1285	1511	1323
ALOXE3__NM_021628_CGACAAGAATGCTACCGGTG	ALOXE3__NM_021628	594	481	678	664
ALOXE3__NM_021628_CTCACCGGTAGCATTCTTGT	ALOXE3__NM_021628	2009	1585	2317	2262
ALOXE3__NM_021628_CTGTTGACGTCTACCATGCA	ALOXE3__NM_021628	1111	1634	1233	1512
ALOXE3__NM_021628_CTTCAATCCACTGATAGCAG	ALOXE3__NM_021628	487	317	519	560
ALOXE3__NM_021628_GGATACACTACCATCCGGTT	ALOXE3__NM_021628	1197	1157	1305	1522
ALPI__NM_001631_AACCCACCCAAGGAGGCTAC	ALPI__NM_001631	704	600	653	727
ALPI__NM_001631_ACACACAGGTGTCCTGCCGT	ALPI__NM_001631	123	120	192	134
ALPI__NM_001631_ACGTCCATCCTGTACGGCAA	ALPI__NM_001631	782	848	852	1014
ALPI__NM_001631_AGCCCGGGCCATTGCCGTAC	ALPI__NM_001631	1224	950	1401	1203
ALPI__NM_001631_AGGATTGAAGCTAAGACGGT	ALPI__NM_001631	273	139	247	152
ALPI__NM_001631_ATCCTGGTGGCTGTCACCGT	ALPI__NM_001631	1212	1247	1226	1119
ALPI__NM_001631_CACAAAGAGGTAGAAGCCGC	ALPI__NM_001631	372	477	382	433
ALPI__NM_001631_CAGCCACCAGGATCCTAAAG	ALPI__NM_001631	515	427	600	695
ALPI__NM_001631_CAGGATTGAAGCTAAGACGG	ALPI__NM_001631	830	523	746	505
ALPI__NM_001631_CCAAGGAGGCTACTGGATCG	ALPI__NM_001631	479	394	634	551
ALPI__NM_001631_CCATGGGAAACATGTACTTG	ALPI__NM_001631	1473	1449	1630	1406
ALPI__NM_001631_CCCAGAGACGTCCCCCATCG	ALPI__NM_001631	5725	5874	5747	7082
ALPI__NM_001631_CCGCCTGCTGCTGGTAATCG	ALPI__NM_001631	3431	4006	3844	4754
ALPI__NM_001631_CCTGAGTTGAACACGTAGCC	ALPI__NM_001631	842	912	806	1221
ALPI__NM_001631_CTCTCGCTCTCATTCACGTC	ALPI__NM_001631	236	157	257	344
ALPI__NM_001631_GAGCAGGATAACTCCGGAGT	ALPI__NM_001631	709	760	716	673
ALPI__NM_001631_GCGGCTCAGGACGGGGAACA	ALPI__NM_001631	624	539	874	908
ALPI__NM_001631_GGAGCAGGATAACTCCGGAG	ALPI__NM_001631	290	237	397	275
ALPI__NM_001631_GGAGTGGTCAGCGGTGACGA	ALPI__NM_001631	380	430	385	498
ALPI__NM_001631_GGATTGAAGCTAAGACGGTG	ALPI__NM_001631	131	113	180	149
ALPI__NM_001631_GGCGGCTGCACTCAAGCCGA	ALPI__NM_001631	801	852	883	990
ALPI__NM_001631_GTGTGTCGTGTCCCACGATG	ALPI__NM_001631	176	54	283	336
ALPI__NM_001631_TCCTCTGCTGTCTCCCCACT	ALPI__NM_001631	1802	2160	1977	2046
ALPI__NM_001631_TCTAGGAGGTGAAGGTCCAA	ALPI__NM_001631	127	114	178	145
ALPI__NM_001631_TTGGTGGCTACACCTTGCGA	ALPI__NM_001631	309	268	407	302
ALPK1__NM_001102406_ATCAGGGTCCTTAACTCGCT	ALPK1__NM_001102406	1121	1253	1099	1256
ALPK1__NM_001102406_CGCACAGTCCCGAGCGAGGA	ALPK1__NM_001102406	1356	1510	1446	1818
ALPK1__NM_001102406_GATCAGGGTCCTTAACTCGC	ALPK1__NM_001102406	566	827	661	882
ALPK1__NM_001102406_GGGTTGCAGCAGTTACTGGT	ALPK1__NM_001102406	2152	2124	2208	2249
ALPK1__NM_001102406_GTGGCAGTACAAACAAGCCG	ALPK1__NM_001102406	83	95	112	153
ALPK1__NM_001102406_GTTGGAAGCGCCAGATGTGT	ALPK1__NM_001102406	1558	1729	1511	1549
ALPK1__NM_001102406_TCAGGGTCCTTAACTCGCTG	ALPK1__NM_001102406	478	327	475	658
ALPK1__NM_001102406_TGAAGGATGTGATTGGCGCC	ALPK1__NM_001102406	448	547	543	668
ALPK1__NM_001102406_TGAGGGCCTCCATCCTCGCT	ALPK1__NM_001102406	1707	1350	1805	1909
ALPK1__NM_001102406_TGGCAGTACAAACAAGCCGT	ALPK1__NM_001102406	89	87	48	90
ALPK1__NM_001102406_TTGGTGGACCGGTTCCTGTA	ALPK1__NM_001102406	403	455	347	285
ALPK1__NM_001102406_TTGTCCTCTTCCGACACATC	ALPK1__NM_001102406	113	203	124	128
ALPK1__NM_001253884_AAGTGCCAAATATCCTACAA	ALPK1__NM_001253884	430	465	408	397
ALPK1__NM_001253884_CAGAGACGTCGAGCCCATAC	ALPK1__NM_001253884	1506	1419	1779	1694
ALPK1__NM_001253884_CGAAGCAGCAGAGTTAATAT	ALPK1__NM_001253884	468	468	476	480
ALPK1__NM_001253884_GCCCGAATCTCCGTGAACTC	ALPK1__NM_001253884	1110	1212	1255	1203
ALPK1__NM_001253884_GGCCTCCATTGTAGGATATT	ALPK1__NM_001253884	1330	1372	1488	1877
ALPK1__NM_001253884_GTACAGAAATGAAAGTGACA	ALPK1__NM_001253884	357	557	456	366
ALPK1__NM_001253884_TACCTGAGTTCACGGAGATT	ALPK1__NM_001253884	1082	1270	1206	1328
ALPK1__NM_001253884_TGCCAAATATCCTACAATGG	ALPK1__NM_001253884	548	524	519	559
ALPK2__NM_052947_AAAGGATGAAAGACTCCGAA	ALPK2__NM_052947	201	212	123	111
ALPK2__NM_052947_AATAATGCCACTCCCATCGA	ALPK2__NM_052947	856	973	993	925
ALPK2__NM_052947_AGGGGTTGGAAACATGAAAC	ALPK2__NM_052947	105	67	120	126
ALPK2__NM_052947_CTGGAGGGAGAGTGAATGGT	ALPK2__NM_052947	2707	820	2892	925
ALPK2__NM_052947_CTGTTGTTCTGCTTCCGTTG	ALPK2__NM_052947	680	853	722	989
ALPK2__NM_052947_GAAAGGATGAAAGACTCCGA	ALPK2__NM_052947	363	508	483	671
ALPK2__NM_052947_GGAGAGTGAATGGTTGGATT	ALPK2__NM_052947	1579	1927	1745	2150
ALPK2__NM_052947_GTGCTTCGCTGCATAATATC	ALPK2__NM_052947	223	108	155	266
ALPK2__NM_052947_TCCAGGTTAGGAGACAATTG	ALPK2__NM_052947	402	350	377	469
ALPK2__NM_052947_TGAGAAGGAACATCCTTATA	ALPK2__NM_052947	263	409	308	347
ALPK2__NM_052947_TGCTGCTGTCTATCAAATCT	ALPK2__NM_052947	623	1033	916	1211
ALPK2__NM_052947_TGTAGATAAAAAACACAGCG	ALPK2__NM_052947	84	227	129	80
ALPK3__NM_020778_AAAATATGAGATCACTCATC	ALPK3__NM_020778	494	542	516	525
ALPK3__NM_020778_ACGGAGCTGGACCGCTACTG	ALPK3__NM_020778	1308	1324	1280	1173
ALPK3__NM_020778_AGCAGCAGGTGACGACGGCC	ALPK3__NM_020778	872	951	835	1154
ALPK3__NM_020778_AGGCGAGGGTCAGTCACGGC	ALPK3__NM_020778	813	1073	826	846
ALPK3__NM_020778_ATGTCCCGTGTGGCATATGA	ALPK3__NM_020778	719	513	769	664
ALPK3__NM_020778_CCGGCCACCGGCTATAAATA	ALPK3__NM_020778	40	101	46	34
ALPK3__NM_020778_CGCAGCCTTGCGCTTCAACT	ALPK3__NM_020778	185	314	229	353
ALPK3__NM_020778_CGGCCTGCTCATGTCCCGTG	ALPK3__NM_020778	1371	1675	1548	1901
ALPK3__NM_020778_CGTGGTCTCAAATAGCGGCT	ALPK3__NM_020778	398	420	397	451
ALPK3__NM_020778_GCCAGCTTGGGCTAGACCGC	ALPK3__NM_020778	592	694	679	635
ALPK3__NM_020778_GCGTGGTCTCAAATAGCGGC	ALPK3__NM_020778	207	194	292	324
ALPK3__NM_020778_TGGACCCCTCATATGCCACA	ALPK3__NM_020778	228	292	287	328
ALPL__NM_000478_ACCTTGGAGAGGGCCACGAA	ALPL__NM_000478	104	125	96	99
ALPL__NM_000478_CACTAAGGAGTTAGTAAGGC	ALPL__NM_000478	297	400	327	289
ALPL__NM_000478_CCCGGTGCAACACCACCCAG	ALPL__NM_000478	804	566	962	731
ALPL__NM_000478_CCGGTCAGCCGAGTGGGCGT	ALPL__NM_000478	1393	1550	1509	1541
ALPL__NM_000478_CCTACGCCCACTCGGCTGAC	ALPL__NM_000478	364	513	410	591
ALPL__NM_000478_GAGCTTCAAACCGAGATACA	ALPL__NM_000478	355	334	252	344
ALPL__NM_000478_GATGCGGGCAGCCGTCACTG	ALPL__NM_000478	817	950	965	1015
ALPL__NM_000478_GATTTCACCATTCTTAGTAC	ALPL__NM_000478	784	743	827	1030
ALPL__NM_000478_GGCATGGTTCACTCTCGTGG	ALPL__NM_000478	1273	1317	1583	970
ALPL__NM_000478_GGTGGCATGGTTCACTCTCG	ALPL__NM_000478	922	1110	1119	1177
ALPL__NM_000478_GTTGCACCGGAAACGCTCAG	ALPL__NM_000478	531	573	594	558
ALPL__NM_000478_TGGCGGTGCCGGCACTGTCA	ALPL__NM_000478	72	188	136	219
ALPL__NM_001127501_AATAGGTAGTCCACATTGTG	ALPL__NM_001127501	165	218	154	138
ALPL__NM_001127501_CCACAATGTGGACTACCTAT	ALPL__NM_001127501	109	97	147	125
ALPL__NM_001177520_AAGGAGGCAGAATTGACCAC	ALPL__NM_001177520	499	426	528	304
ALPL__NM_001177520_CACAATGTGGACTACCTATT	ALPL__NM_001177520	77	45	122	90
ALPP__NM_001632_CACAGGGTGCCCGGTATGTG	ALPP__NM_001632	85	18	72	125
ALPP__NM_001632_CAGTGCGGTTCCACACATAC	ALPP__NM_001632	1335	1206	1530	1976
ALPP__NM_001632_CATGGACCGCTTCCCATATG	ALPP__NM_001632	1961	2073	1971	2303
ALPP__NM_001632_CCGGACTTCTGGAACCGCG	ALPP__NM_001632	1974	2438	2551	2377
ALPP__NM_001632_CTCTCGCTCTCGGTAACATC	ALPP__NM_001632	329	163	295	193
ALPP__NM_001632_TGAGCTGCGTAGCGATGTCC	ALPP__NM_001632	119	211	207	234
ALPPL2__NM_031313_ACAGAGCCACGTACGGGAAG	ALPPL2__NM_031313	476	356	620	775
ALPPL2__NM_031313_CAGTGCGGTTCCACACGTAC	ALPPL2__NM_031313	470	322	556	573
ALPPL2__NM_031313_CATGGACCGCTTCCCGTACG	ALPPL2__NM_031313	1656	2180	1878	2087
ALPPL2__NM_031313_CCTTGGACAGAGCCACGTAC	ALPPL2__NM_031313	886	997	930	785
ALPPL2__NM_031313_CGGCGCCCGGCCGGATGTTA	ALPPL2__NM_031313	1094	1508	1159	1531
ALPPL2__NM_031313_GCGTGGGTCTCTCCGTCCAG	ALPPL2__NM_031313	948	782	1338	1077
ALS2__NM_001135745_AACAGAGGCAGAAGGATCCA	ALS2__NM_001135745	278	203	379	212
ALS2__NM_001135745_AACTCCATGTTTCACTCCGA	ALS2__NM_001135745	978	823	1247	1168
ALS2__NM_001135745_AATAACATATTGCCCAACCA	ALS2__NM_001135745	265	176	216	189
ALS2__NM_001135745_AATGGTGTCGCGTACATGTG	ALS2__NM_001135745	300	169	390	319
ALS2__NM_001135745_AGCCAACCAGCAGTATGTGC	ALS2__NM_001135745	579	479	516	549
ALS2__NM_001135745_AGGATCCTGCCTGCCAGATA	ALS2__NM_001135745	453	193	471	462
ALS2__NM_001135745_AGGCCTGGTCCATATCTGGC	ALS2__NM_001135745	873	1080	952	1387
ALS2__NM_001135745_CTGCCTGCCAGATATGGACC	ALS2__NM_001135745	213	266	300	449
ALS2__NM_001135745_GAACTCCATGTTTCACTCCG	ALS2__NM_001135745	1100	1306	1241	1468
ALS2__NM_001135745_GGCAATCTCTCTGGTGTTAT	ALS2__NM_001135745	1425	2194	1562	2333
ALS2__NM_001135745_TCTCTCTGGTGTTATGGGAA	ALS2__NM_001135745	194	127	182	189
ALS2__NM_001135745_TGGGCAATATGTTATTACTG	ALS2__NM_001135745	52	99	91	63
ALS2CL__NM_001190707_AGCACACGCTCAGCCCGCAA	ALS2CL__NM_001190707	774	596	701	425
ALS2CL__NM_001190707_CACGTACTGTTTCACGCTGAT	ALS2CL__NM_001190707	1874	1899	1949	2385
ALS2CL__NM_001190707_CAGCCAGGACGTACCCGTGA	ALS2CL__NM_001190707	1849	1237	1870	1704
ALS2CL__NM_001190707_CCGCAACGGTGCACCCGTCA	ALS2CL__NM_001190707	504	400	465	376
ALS2CL__NM_001190707_CCGTGACGGTCGCACCGTTG	ALS2CL__NM_001190707	1417	968	1582	1306
ALS2CL__NM_001190707_CGACCGTCACGGGTACGTCC	ALS2CL__NM_001190707	820	888	1021	615
ALS2CL__NM_001190707_GCACGTACTGTTCGCACGTGA	ALS2CL__NM_001190707	1163	1446	1173	1471
ALS2CL__NM_001190707_GGAAGGTATATTCTGCGCAG	ALS2CL__NM_001190707	517	525	617	609
ALS2CL__NM_001190707_GGCTCGGTGGGCGCATCGCT	ALS2CL__NM_001190707	691	469	696	601
ALS2CL__NM_001190707_TCCTGAAGGAGCTCTGTGAGC	ALS2CL__NM_001190707	549	275	543	370
ALS2CL__NM_001190707_TCTGCCCCAGGCGATCCGA	ALS2CL__NM_001190707	827	794	877	764
ALS2CL__NM_001190707_TTACCCGGACTCCACCGGTC	ALS2CL__NM_001190707	262	107	188	230
ALS2CL__NM_182775_ACCTTCCAGGCTACCTACGC	ALS2CL__NM_182775	1837	1465	1861	1284
ALS2CL__NM_182775_AGGCTACCTACGCAGGTGTC	ALS2CL__NM_182775	319	374	405	338
ALS2CL__NM_182775_ATCAGGAAAGAGGCTCAAGT	ALS2CL__NM_182775	269	265	336	205
ALS2CL__NM_182775_CCTCACGCTGACGAGCAATC	ALS2CL__NM_182775	309	228	489	457
ALS2CL__NM_182775_CGAGTTGCCTTAGAGCGCAA	ALS2CL__NM_182775	559	342	507	249
ALS2CL__NM_182775_GAAGAGCTCTGAGTAGAAGC	ALS2CL__NM_182775	168	129	148	164
ALS2CL__NM_182775_GAGGACATACGGGGAAATTG	ALS2CL__NM_182775	227	221	308	269
ALS2CL__NM_182775_GCGAGTTGCCTTAGAGCGCA	ALS2CL__NM_182775	575	678	531	659
ALS2CL__NM_182775_GGACCTCCAGGTGCATGGAT	ALS2CL__NM_182775	655	671	702	861
ALS2CL__NM_182775_GGTGCTGGAGAGGACATACG	ALS2CL__NM_182775	474	495	535	523
ALS2CL__NM_182775_GTCAGCGTGAGGTCCTTGAG	ALS2CL__NM_182775	537	327	600	420
ALS2CL__NM_182775_TGCAGAGGTACTCCTGGTC	ALS2CL__NM_182775	1103	704	1237	697
ALS2CR11__NM_001168216_AAAGGCCTATTTGTCTCTTG	ALS2CR11__NM_001168216	301	207	317	390
ALS2CR11__NM_001168216_AGCACACTTGATAACCGCAG	ALS2CR11__NM_001168216	93	37	55	58
ALS2CR11__NM_001168216_ATTCCTCTGCAATAGCGGGG	ALS2CR11__NM_001168216	97	50	83	98
ALS2CR11__NM_001168216_CATGCACATTAAAGGTCGG	ALS2CR11__NM_001168216	607	639	704	612
ALS2CR11__NM_001168216_CCGCCACCCCGCTATTGCAG	ALS2CR11__NM_001168216	3218	2424	3397	2857
ALS2CR11__NM_001168216_CCTTTAATGTGCATGATATC	ALS2CR11__NM_001168216	396	283	363	523
ALS2CR11__NM_001168216_GCACACTTGATAACCGCAGC	ALS2CR11__NM_001168216	314	376	422	383
ALS2CR11__NM_001168216_GCGGGGTGGCGGACAGGACT	ALS2CR11__NM_001168216	671	764	694	847
ALS2CR11__NM_001168216_GGGATTCCTCTGCAATAGCG	ALS2CR11__NM_001168216	45	6	19	30
ALS2CR11__NM_001168216_GTGCCCTGGTTCAGGGCGTA	ALS2CR11__NM_001168216	17	13	12	22
ALS2CR11__NM_001168216_TATCATGCACATTAAAGGT	ALS2CR11__NM_001168216	517	495	482	423
ALS2CR11__NM_001168216_TGCCCTGGTTCAGGGCGTAA	ALS2CR11__NM_001168216	37	91	41	53
ALS2CR12__NM_001127391_AACAGATTTCTGTCACCTTA	ALS2CR12__NM_001127391	288	164	374	291
ALS2CR12__NM_001127391_ACAGATTTCTGTCACCTTAG	ALS2CR12__NM_001127391	271	134	233	249
ALS2CR12__NM_001127391_ACTTACTTTGGGGAAACAAC	ALS2CR12__NM_001127391	59	106	87	69
ALS2CR12__NM_001127391_AGATGTTTGATAGGAAAAAG	ALS2CR12__NM_001127391	19	20	29	29
ALS2CR12__NM_001127391_AGCAGGTGCAGTAGATGAGA	ALS2CR12__NM_001127391	117	71	111	103
ALS2CR12__NM_001127391_GAGTTCTTGCGTGGTAGTTG	ALS2CR12__NM_001127391	156	147	200	227
ALS2CR12__NM_001127391_GGGGTCTTGATTAGCTTCCG	ALS2CR12__NM_001127391	314	700	430	376
ALS2CR12__NM_001127391_GGTGCAGTAGATGAGAGGGT	ALS2CR12__NM_001127391	584	492	747	624
ALS2CR12__NM_001127391_GTGCAGTAGATGAGAGGGTT	ALS2CR12__NM_001127391	612	881	645	760
ALS2CR12__NM_001127391_GTTTGATAGGAAAAAGCGGT	ALS2CR12__NM_001127391	175	382	271	233
ALS2CR12__NM_001127391_TACCACGCAAGAACTCCACA	ALS2CR12__NM_001127391	741	835	752	946
ALS2CR12__NM_001127391_TAGGGGATGAGATGTTTGAT	ALS2CR12__NM_001127391	285	577	344	423
[illegible]	[illegible]	[illegible]	[illegible]	[illegible]	[illegible]

ANKHD1__NM_001197030_AGGAGCAGACTTACGCACTG	ANKHD1__NM_001197030	720	517	792	513
ANKHD1__NM_001197030_ATCCTCAGTTAGTTGTGCAC	ANKHD1__NM_001197030	421	517	533	425
ANKHD1__NM_001197030_CATGCTAATGTTGAAGATCG	ANKHD1__NM_001197030	507	682	658	849
ANKHD1__NM_001197030_CTATATTTGCACCTTCATTA	ANKHD1__NM_001197030	284	377	378	337
ANKHD1__NM_001197030_GTTCGTAAATTGCTAGATGA	ANKHD1__NM_001197030	48	37	60	81
ANKHD1__NM_001197030_GTTGAAGATCGAGGGAATAA	ANKHD1__NM_001197030	153	180	195	119
ANKHD1__NM_001197030_TACCATGCAGGCCTCCATTA	ANKHD1__NM_001197030	981	1057	1058	740
ANKHD1__NM_001197030_TGACGAGGACGAAGTGTCCG	ANKHD1__NM_001197030	354	464	345	394
ANKIB1__NM_019004_AATGGAGTATTGTGCTGGTA	ANKIB1__NM_019004	596	643	700	697
ANKIB1__NM_019004_AATGTAATGGAGTATTGTGC	ANKIB1__NM_019004	387	636	514	601
ANKIB1__NM_019004_AGGGATGAAAGCCTGTGTAG	ANKIB1__NM_019004	443	490	411	401
ANKIB1__NM_019004_ATCTATGCATTTGTTGTGTA	ANKIB1__NM_019004	573	924	620	924
ANKIB1__NM_019004_CACCATTGATGAGTGCTTTA	ANKIB1__NM_019004	514	552	579	676
ANKIB1__NM_019004_CAGGCATTATACCCACTTGG	ANKIB1__NM_019004	548	453	494	527
ANKIB1__NM_019004_CCTCAAATTATGATATCTGA	ANKIB1__NM_019004	1205	1598	1425	1640
ANKIB1__NM_019004_CTTGATCCAAATACATCTTA	ANKIB1__NM_019004	140	188	174	141
ANKIB1__NM_019004_GATGAGTGCTTTACGGAATT	ANKIB1__NM_019004	727	1010	800	898
ANKIB1__NM_019004_GGGAGAAATTACTTGAAGCT	ANKIB1__NM_019004	645	507	711	624
ANKIB1__NM_019004_TCTATGCATTTGTTGTGTAT	ANKIB1__NM_019004	978	1273	1107	1457
ANKIB1__NM_019004_TGATCCAAATACATCTTATG	ANKIB1__NM_019004	329	313	286	351
ANKK1__NM_178510_AACTCCATCACAATACCCAG	ANKK1__NM_178510	374	289	491	401
ANKK1__NM_178510_ACAGGCCGAAGTCTGAAATC	ANKK1__NM_178510	474	507	486	463
ANKK1__NM_178510_ACCTCTCGATGTACTGCATC	ANKK1__NM_178510	182	100	225	185
ANKK1__NM_178510_AGTGGATGGAACAGTCCACC	ANKK1__NM_178510	384	297	428	490
ANKK1__NM_178510_CAGCACATCGTGTCTATCTA	ANKK1__NM_178510	940	1129	1085	1422
ANKK1__NM_178510_CGAGGGCGACTGGCGCCTAG	ANKK1__NM_178510	1246	1227	1647	1531
ANKK1__NM_178510_CTCTCCAGGAACATCTCAGG	ANKK1__NM_178510	324	197	347	257
ANKK1__NM_178510_CTGTCCAGGAGTATGTTGCC	ANKK1__NM_178510	815	1008	955	1198
ANKK1__NM_178510_CTTCGGCCTGTCCAAGTGGA	ANKK1__NM_178510	215	256	206	333
ANKK1__NM_178510_GATGCAGTACATCGAGAGGT	ANKK1__NM_178510	570	357	580	516
ANKK1__NM_178510_GCGACGACTTCGAGGGCGAC	ANKK1__NM_178510	742	676	916	919
ANKK1__NM_178510_TTCACCCGCGACGACTTCGA	ANKK1__NM_178510	77	66	78	85
ANKLE1__NM_001278443_AAGGCGGGGACCCCAACGCT	ANKLE1__NM_001278443	847	681	802	850
ANKLE1__NM_001278443_AAGTAGGAAATCGACCGACC	ANKLE1__NM_001278443	261	353	284	211
ANKLE1__NM_001278443_AGAGGTGACAACAGGGACAT	ANKLE1__NM_001278443	156	158	216	245
ANKLE1__NM_001278443_CCGTCCTCTAGCACCAAATT	ANKLE1__NM_001278443	844	454	921	642
ANKLE1__NM_001278443_CGGCGCTGCGCGACCAGGTT	ANKLE1__NM_001278443	109	152	178	200
ANKLE1__NM_001278443_CGTCCTCTAGCACCAAATTA	ANKLE1__NM_001278443	516	506	495	565
ANKLE1__NM_001278443_CTGCGGCGCGGACCCTAATT	ANKLE1__NM_001278443	1343	1002	1482	1304
ANKLE1__NM_001278443_GAGTCCAGCGTCTCATCGGT	ANKLE1__NM_001278443	1039	537	1279	932
ANKLE1__NM_001278443_GGACCCTAATTTGGTGCTAG	ANKLE1__NM_001278443	274	265	276	414
ANKLE1__NM_001278443_GTCAGTGCCTCGACAGATCT	ANKLE1__NM_001278443	376	223	440	397
ANKLE1__NM_001278443_TCAGTGCCTCGACAGATCTG	ANKLE1__NM_001278443	520	343	718	613
ANKLE1__NM_001278443_TCGATTTCCTACTTCCCGCG	ANKLE1__NM_001278443	293	366	401	505
ANKLE2__NM_015114_AAAAACCAGCCTGATCAGTT	ANKLE2__NM_015114	835	272	1049	321
ANKLE2__NM_015114_AACCTTGTAGTTGATGTAAT	ANKLE2__NM_015114	414	172	438	180
ANKLE2__NM_015114_AGAGAAGAAATCGTCAAAGC	ANKLE2__NM_015114	106	45	211	47
ANKLE2__NM_015114_AGCTGGAGCGACTGCGTCTA	ANKLE2__NM_015114	463	131	643	158
ANKLE2__NM_015114_ATTTCTTCTCTAAGGTCATC	ANKLE2__NM_015114	71	133	63	80
ANKLE2__NM_015114_CCCAGTTCCTCCGCCAAGCG	ANKLE2__NM_015114	209	381	273	373
ANKLE2__NM_015114_GAAAAACCAGCCTGATCAGT	ANKLE2__NM_015114	522	187	569	210
ANKLE2__NM_015114_GCTGGCTTCAAAATCCTTTG	ANKLE2__NM_015114	1639	1477	2105	1478
ANKLE2__NM_015114_GGGAACCCAACTGATCAGGC	ANKLE2__NM_015114	181	45	112	110
ANKLE2__NM_015114_GTCAAAGCCGGATTGAAATG	ANKLE2__NM_015114	382	80	526	127
ANKLE2__NM_015114_GTCTTCTTTCTACCACCATG	ANKLE2__NM_015114	97	34	161	27
ANKLE2__NM_015114_TGCTGCTGATCGCTGTGCGG	ANKLE2__NM_015114	291	228	217	166
ANKMY1__NM_001282771_ACATGTAGGTACCCAGGCCA	ANKMY1__NM_001282771	930	1143	1049	1428
ANKMY1__NM_001282771_ATGGCCTGGGTACCTACATG	ANKMY1__NM_001282771	786	907	668	674
ANKMY1__NM_001282771_CAGCCGCCAACGCCCGCTAG	ANKMY1__NM_001282771	28	17	49	108
ANKMY1__NM_001282771_CAGGATGGTTGCATGTACCA	ANKMY1__NM_001282771	178	236	178	184
ANKMY1__NM_001282771_CTCAGCCACCGAGAAGGCTA	ANKMY1__NM_001282771	127	127	100	61
ANKMY1__NM_001282771_CTGGCCACATGTAGGTACCC	ANKMY1__NM_001282771	547	632	583	512
ANKMY1__NM_001282771_GACTTCGTCCTCTAAGCTAA	ANKMY1__NM_001282771	33	105	33	79
ANKMY1__NM_001282771_GCAGGATGGTTGCATGTACC	ANKMY1__NM_001282771	619	591	780	990
ANKMY1__NM_001282771_GCATGTACCAGGGGGAGTTT	ANKMY1__NM_001282771	1367	1710	1568	2025
ANKMY1__NM_001282771_GTAAAATGTGCCCGTGAAAC	ANKMY1__NM_001282771	56	31	57	60
ANKMY1__NM_001282771_TACAAAGCGGACCAGCGGTT	ANKMY1__NM_001282771	528	756	682	772
ANKMY1__NM_001282771_TTGAACATGAAGCTTGGATA	ANKMY1__NM_001282771	364	232	473	577
ANKMY1__NM_001282780_GAAGGTTGACAATGTCGTTG	ANKMY1__NM_001282780	361	399	465	542
ANKMY1__NM_016552_ACAAAGCGGACCAGCGGTTT	ANKMY1__NM_016552	697	881	875	1136
ANKMY1__NM_016552_GGAAAGAGCTTGTCATGGGC	ANKMY1__NM_016552	362	568	402	704
ANKMY1__NM_016552_TCTCCATCCTCAACGAACGG	ANKMY1__NM_016552	662	761	821	1166
ANKMY1__NM_016552_TGAAATTCCTCCGTTCGTTG	ANKMY1__NM_016552	201	294	317	317
ANKMY2__NM_020319_AAAAGATGGAGAGAATAAAC	ANKMY2__NM_020319	23	17	30	31
ANKMY2__NM_020319_AAACTGCCCCCAAAGTTGGC	ANKMY2__NM_020319	272	379	272	541
ANKMY2__NM_020319_ACAGATGTTGTCAACTCTGT	ANKMY2__NM_020319	468	491	487	648
ANKMY2__NM_020319_ATGCTGATGACAATTTACAT	ANKMY2__NM_020319	478	572	513	457
ANKMY2__NM_020319_CTAATGCATGCAGCATATAA	ANKMY2__NM_020319	44	96	58	65
ANKMY2__NM_020319_CTACTGGAAGTCATCGGGAA	ANKMY2__NM_020319	453	555	446	607
ANKMY2__NM_020319_GAATGTTCGTGTCAACTGTT	ANKMY2__NM_020319	697	743	633	748
ANKMY2__NM_020319_GACAGGATGAAGATTCGTTG	ANKMY2__NM_020319	807	674	899	822
ANKMY2__NM_020319_TCACTCTGTAGCATTTATTC	ANKMY2__NM_020319	494	562	692	788
ANKMY2__NM_020319_TGCAGCGGGCCTGCCAACTT	ANKMY2__NM_020319	786	1068	1176	934
ANKMY2__NM_020319_TTATATGCTGCATGCATTAG	ANKMY2__NM_020319	136	209	175	239
ANKMY2__NM_020319_TTTCTTTCCTCGAGAGAGAC	ANKMY2__NM_020319	28	38	48	13
ANKRA2__NM_023039_ACACTCTTCCACGATAAGCT	ANKRA2__NM_023039	1177	1243	1320	929
ANKRA2__NM_023039_ACCTCAGTTGCAATATTTAC	ANKRA2__NM_023039	988	1142	1007	1305
ANKRA2__NM_023039_AGATCAGGTTAACTCTGACC	ANKRA2__NM_023039	1301	1069	1243	958
ANKRA2__NM_023039_AGCTTTGTCTGTTCACCAGT	ANKRA2__NM_023039	1581	1597	1755	1675
ANKRA2__NM_023039_ATCTAATGAAGTGTTACGGC	ANKRA2__NM_023039	276	315	257	307
ANKRA2__NM_023039_CCAGTTAGGCTATAAGTGCT	ANKRA2__NM_023039	1113	902	1111	1021
ANKRA2__NM_023039_CCCAGCACTTATAGCCTAAC	ANKRA2__NM_023039	991	840	998	1128
ANKRA2__NM_023039_CTGGCTACTCGTATCGAACA	ANKRA2__NM_023039	790	764	842	774
ANKRA2__NM_023039_GACACTCTTCCACGATAAGC	ANKRA2__NM_023039	538	790	806	849
ANKRA2__NM_023039_GCCAGTTAGGCTATAAGTGC	ANKRA2__NM_023039	1055	1244	1428	1530
ANKRA2__NM_023039_GTGGCATCTGTCCTATTTAA	ANKRA2__NM_023039	143	123	215	236
ANKRA2__NM_023039_TATTGGAGCCCAGCTTATCG	ANKRA2__NM_023039	993	762	934	648
ANKRA2__NM_023039_TCAGGGAGAGATGCTCTATC	ANKRA2__NM_023039	539	702	696	645
ANKRA2__NM_023039_TCTGTGTTTGTTGGTTAAAG	ANKRA2__NM_023039	574	581	641	684
ANKRD1__NM_014391_AAAAATTAGCGCCCGAGATA	ANKRD1__NM_014391	190	202	262	226
ANKRD1__NM_014391_AGAGCAGCCTTCAGAAACGT	ANKRD1__NM_014391	634	911	762	680
ANKRD1__NM_014391_AGCTCTTCATAGAGCATGCT	ANKRD1__NM_014391	59	106	71	137
ANKRD1__NM_014391_ATCCGACTCCTGATTATGTA	ANKRD1__NM_014391	546	470	556	698
ANKRD1__NM_014391_CAGAACCTGAAATCATTGTA	ANKRD1__NM_014391	351	408	421	523
ANKRD1__NM_014391_CATGATGGTACTGAAAGTAG	ANKRD1__NM_014391	309	189	308	398
ANKRD1__NM_014391_CCAGATCGAATTCCGTGATA	ANKRD1__NM_014391	863	571	1022	1137
ANKRD1__NM_014391_CCATATCACGGAATTCGATC	ANKRD1__NM_014391	696	670	763	791
ANKRD1__NM_014391_CTTCATAGAGCATGCTTGGA	ANKRD1__NM_014391	355	549	396	564
ANKRD1__NM_014391_GGATGTGCCTACGTTTCTGA	ANKRD1__NM_014391	804	888	856	1176
ANKRD1__NM_014391_TACTTTCAGTACCATCATGT	ANKRD1__NM_014391	9	53	29	22
ANKRD1__NM_014391_TCTCACCGCATCATGCAACG	ANKRD1__NM_014391	427	272	505	504
ANKRD10__NM_001286721_AATGGTTCAGATGACAATTC	ANKRD10__NM_001286721	158	165	184	148
ANKRD10__NM_001286721_ACCCACCTTGCCGAAATGCG	ANKRD10__NM_001286721	720	937	803	850
ANKRD10__NM_001286721_AGACACTGAATTAAAACTGA	ANKRD10__NM_001286721	193	284	328	253
ANKRD10__NM_001286721_AGGAGCCAACATTAACAAAC	ANKRD10__NM_001286721	73	84	136	152
ANKRD10__NM_001286721_CTGGTCTGGCTGATTCAAGC	ANKRD10__NM_001286721	214	128	242	168
ANKRD10__NM_001286721_GTGCACTGGGCCGCGCATTT	ANKRD10__NM_001286721	1102	1088	1016	1045
ANKRD10__NM_001286721_TCAGTGCCCTTGTGGCGAAT	ANKRD10__NM_001286721	335	327	409	340
ANKRD10__NM_001286721_TCTTACCGGTTTGTTAATGT	ANKRD10__NM_001286721	775	958	736	719
ANKRD10__NM_017664_GTAAAGAAAGCTAGAACTGA	ANKRD10__NM_017664	104	53	97	84
ANKRD10__NM_017664_GTCGCCATTCGTGAGTGGCA	ANKRD10__NM_017664	528	495	549	363
ANKRD10__NM_017664_GTTCCCACACTAATATGATT	ANKRD10__NM_017664	245	214	340	254
ANKRD10__NM_017664_TAACAATGGCATCTTAAATG	ANKRD10__NM_017664	73	194	123	79
ANKRD10__NM_017664_TCTGCCGTGCCACTCACGAA	ANKRD10__NM_017664	823	513	908	738
ANKRD11__NM_001256182_ACATGGTGGAGAAGCAGACT	ANKRD11__NM_001256182	80	68	97	75
ANKRD11__NM_001256182_CCAGTCTACAGTGTGTCAGA	ANKRD11__NM_001256182	763	242	972	423
ANKRD11__NM_001256182_CTGCACGAGGCCTGTAACCG	ANKRD11__NM_001256182	852	202	786	301
ANKRD11__NM_001256182_CTTCCTGCTGTGGTGCTTTA	ANKRD11__NM_001256182	205	355	353	462
ANKRD11__NM_001256182_CTTCTGACACACTGTAGACT	ANKRD11__NM_001256182	379	238	348	250
ANKRD11__NM_001256182_GATGAGCTCTTTGATGCGCC	ANKRD11__NM_001256182	787	280	820	453
ANKRD11__NM_001256182_GGGGGCAGACGTCAACGTCA	ANKRD11__NM_001256182	334	384	296	356
ANKRD11__NM_001256182_GTCAACGTCAAGGACTTCGC	ANKRD11__NM_001256182	489	136	484	202
ANKRD11__NM_001256182_GTGAACAAGAGAAACGAGCG	ANKRD11__NM_001256182	47	23	108	32
ANKRD11__NM_001256182_TCTTAATCCTCTTCCGCTCA	ANKRD11__NM_001256182	467	205	509	195
ANKRD11__NM_001256182_TGAGCGGAAGAGGATTAAGA	ANKRD11__NM_001256182	155	95	159	48
ANKRD11__NM_001256182_TGATGAGCTCTTTGATGCGC	ANKRD11__NM_001256182	166	177	211	203
ANKRD12__NM_001083625_AAAAGGAAGTTTACGTTTCA	ANKRD12__NM_001083625	177	262	228	258
ANKRD12__NM_001083625_CGAATAGCAGCCATGTGTAA	ANKRD12__NM_001083625	1047	1359	1056	1240
ANKRD12__NM_001083625_CTCCATGATTCTGCTAGTAG	ANKRD12__NM_001083625	391	340	500	623
ANKRD12__NM_001083625_CTGCATGAAGCTTGCAATGT	ANKRD12__NM_001083625	445	550	499	616
ANKRD12__NM_001083625_GATGATGATGAAAGTTACAC	ANKRD12__NM_001083625	386	406	388	531
ANKRD12__NM_001083625_GCTAAGATACTTATAGCAGC	ANKRD12__NM_001083625	322	258	450	456
ANKRD12__NM_001083625_GGAGCAGATGTTAACACACA	ANKRD12__NM_001083625	720	822	1074	987
ANKRD12__NM_001083625_GTAAAGCTGTTACTTCGTCA	ANKRD12__NM_001083625	733	636	944	769
ANKRD12__NM_001083625_TAACCAAAAAGAATGCTAAC	ANKRD12__NM_001083625	288	170	265	313
ANKRD12__NM_001083625_TCCATGATTCTGCTAGTAGT	ANKRD12__NM_001083625	402	394	549	686
ANKRD12__NM_001083625_TCTGTTTCTGCTACATCCAC	ANKRD12__NM_001083625	352	378	425	353
ANKRD12__NM_001083625_TGGGCAGGCGTTGTTTGTGA	ANKRD12__NM_001083625	371	558	348	622
ANKRD12__NM_015208_GACATACAAGTGAAAATTGG	ANKRD12__NM_015208	276	192	246	373
ANKRD13A__NM_033121_ACCCCCTGCACCTCCTAGTC	ANKRD13A__NM_033121	1697	2262	1741	2328
ANKRD13A__NM_033121_CAAATGATGTCTGTCGCATC	ANKRD13A__NM_033121	601	840	820	1213
ANKRD13A__NM_033121_CAGATGCGACAGACATCATT	ANKRD13A__NM_033121	705	730	803	554
ANKRD13A__NM_033121_GCAATAATGTTCGACCTCGT	ANKRD13A__NM_033121	1810	1935	2078	1917
ANKRD13A__NM_033121_GCGCGTCGATATCACATTGC	ANKRD13A__NM_033121	684	924	838	1066
ANKRD13A__NM_033121_GCTGCTCCAAAAAATTCTCG	ANKRD13A__NM_033121	583	799	775	807
ANKRD13A__NM_033121_GGGCATATTCTAGAAACCAA	ANKRD13A__NM_033121	289	240	222	166
ANKRD13A__NM_033121_TAGTCTGGAAAAACGACTAC	ANKRD13A__NM_033121	111	41	127	110
ANKRD13A__NM_033121_TGCTGTTTCCTTGGGACATT	ANKRD13A__NM_033121	336	449	364	551
ANKRD13A__NM_033121_TGGGCATATTCTAGAAACCA	ANKRD13A__NM_033121	52	19	75	59
ANKRD13A__NM_033121_TGTGATATCGACGCGCAGTT	ANKRD13A__NM_033121	267	298	257	205
ANKRD13A__NM_033121_TGTTGTGGTAGTCTCGATGT	ANKRD13A__NM_033121	531	588	555	400
ANKRD13B__NM_152345_AAATGGGAGTTCACTAGCTG	ANKRD13B__NM_152345	166	162	256	173
ANKRD13B__NM_152345_ACACTTTGTAGGTGTCACTA	ANKRD13B__NM_152345	289	288	289	356
ANKRD13B__NM_152345_ATCCGCTGCACTACCTCGTG	ANKRD13B__NM_152345	1364	1333	1397	1059
ANKRD13B__NM_152345_ATTCTTGGTGTCAAGCTGAG	ANKRD13B__NM_152345	38	12	71	49
ANKRD13B__NM_152345_GAAATGGGAGTTCACTAGCT	ANKRD13B__NM_152345	250	609	344	420
ANKRD13B__NM_152345_GCAGCTGGTGCTTCGGTACC	ANKRD13B__NM_152345	667	583	712	1150
ANKRD13B__NM_152345_GCCGCTCTTCCACACTTTGT	ANKRD13B__NM_152345	211	287	214	140
ANKRD13B__NM_152345_GGCTGTGAGTACCCGGGACC	ANKRD13B__NM_152345	639	854	632	687
ANKRD13B__NM_152345_GTGGGCAGGGAGAATCGCAG	ANKRD13B__NM_152345	181	97	138	43
ANKRD13B__NM_152345_TCAAGCTGAGTGGTGACGAC	ANKRD13B__NM_152345	205	232	201	213
ANKRD13B__NM_152345_TGACGACGGGCGCGGTAAGC	ANKRD13B__NM_152345	223	104	200	188
ANKRD13B__NM_152345_TGCAGCTGGTGCTTCGGTAC	ANKRD13B__NM_152345	436	318	429	441
ANKRD13C__NM_030816_ATTGTGCGTGCGGATGAGAG	ANKRD13C__NM_030816	207	118	206	172
ANKRD13C__NM_030816_CGTGCACCGGGTAGTGTGCG	ANKRD13C__NM_030816	1225	1187	1299	1496
ANKRD13C__NM_030816_CTCATCCGCACGCACAATAT	ANKRD13C__NM_030816	1050	1048	1183	1146
ANKRD13C__NM_030816_CTCGTGCACCGGGTAGTGTG	ANKRD13C__NM_030816	410	346	511	405
ANKRD13C__NM_030816_GCAGAAAGATAATCACGGTG	ANKRD13C__NM_030816	412	387	534	386
ANKRD13C__NM_030816_GCTGTGATGTTAGGAAATAA	ANKRD13C__NM_030816	259	234	341	290
ANKRD13C__NM_030816_GTGCACGAGTGCGTCTTCAA	ANKRD13C__NM_030816	220	214	295	207
ANKRD13C__NM_030816_TCATCCGCACGCACAATATC	ANKRD13C__NM_030816	799	644	857	1021
ANKRD13C__NM_030816_TCGTGCACCGGGTAGTGTGC	ANKRD13C__NM_030816	480	457	442	512
ANKRD13C__NM_030816_TCTGCCCGATATTGTGCGTG	ANKRD13C__NM_030816	2488	3530	2637	3377
ANKRD13C__NM_030816_TCTGGTAAAGGTACCGCCGA	ANKRD13C__NM_030816	280	294	383	235
ANKRD13C__NM_030816_TGCACGAGTGCGTCTTCAAG	ANKRD13C__NM_030816	170	203	148	101
ANKRD13D__NM_207354_CATCTCAACGTAGAAATCGG	ANKRD13D__NM_207354	492	368	530	284
ANKRD13D__NM_207354_CCACGTTGGCATTGTGTCGA	ANKRD13D__NM_207354	1069	1341	1253	1592
ANKRD13D__NM_207354_CCCGCTGCACCGGCTCGTCT	ANKRD13D__NM_207354	2315	2600	2362	2364
ANKRD13D__NM_207354_CCCTCTGGTAGTCCCGATAC	ANKRD13D__NM_207354	878	732	888	760
ANKRD13D__NM_207354_CGGCCTCCAGTTCGCGATGC	ANKRD13D__NM_207354	583	512	703	612
ANKRD13D__NM_207354_CTCCTGTTCAATGTCGTGCT	ANKRD13D__NM_207354	984	862	1095	1480
ANKRD13D__NM_207354_CTTCGACACAATGCCAACGT	ANKRD13D__NM_207354	201	106	192	152
ANKRD13D__NM_207354_GAACCGGCATCGCGAACTGG	ANKRD13D__NM_207354	704	484	773	691
ANKRD13D__NM_207354_GGCGAACCGGCATCGCGAAC	ANKRD13D__NM_207354	531	437	522	553
ANKRD13D__NM_207354_TCATCTCAACGTAGAAATCG	ANKRD13D__NM_207354	229	197	179	224
ANKRD13D__NM_207354_TCCCGCTGCACCGGCTCGTC	ANKRD13D__NM_207354	727	724	850	1113
ANKRD13D__NM_207354_TGCAGCTGGTGCTCCAGTAT	ANKRD13D__NM_207354	534	572	591	699
ANKRD16__NM_001009941_CAGAGGGTCGCCTTCTCGAC	ANKRD16__NM_001009941	5222	4922	5966	5412
ANKRD16__NM_001009941_CCACGGGCATCGGACGTGC	ANKRD16__NM_001009941	811	691	984	819
ANKRD16__NM_001009941_CCATCATCAGAGGAGTCCTA	ANKRD16__NM_001009941	554	469	506	572
ANKRD16__NM_001009941_CCGCTTGTAGTCTCGGTTGG	ANKRD16__NM_001009941	1878	1999	2276	2540
ANKRD16__NM_001009941_GGCCATAGCGCAATGACATA	ANKRD16__NM_001009941	132	160	151	147
ANKRD16__NM_001009941_GGCTGCTCCTCGATGAACAT	ANKRD16__NM_001009941	699	512	842	634
ANKRD16__NM_001009941_GTGGGCACATCGACGTCGCT	ANKRD16__NM_001009941	632	670	651	893
ANKRD16__NM_001009941_TGATGGACGCAATCCAGTGT	ANKRD16__NM_001009941	1190	1507	1198	1213
ANKRD16__NM_001009941_TGCACAGGGCAGCTGTCACA	ANKRD16__NM_001009941	673	391	551	314
ANKRD16__NM_001009941_TTCAGAGACCAAGAATCGGA	ANKRD16__NM_001009941	34	49	63	52
ANKRD16__NM_001009941_TTCCACATTGCCAGTCGAGA	ANKRD16__NM_001009941	44	20	58	111
ANKRD16__NM_001009941_TTGATGGACGCAATCCAGTG	ANKRD16__NM_001009941	277	372	297	310
ANKRD17__NM_001286771_AAGTACTTCAGGATCTGCAA	ANKRD17__NM_001286771	125	51	185	75
ANKRD17__NM_001286771_AGGTGACATTACACCTTTAA	ANKRD17__NM_001286771	196	108	298	152
ANKRD17__NM_001286771_AGTACCTGATAAGAGCAACT	ANKRD17__NM_001286771	313	231	471	338
ANKRD17__NM_001286771_ATCGTCTGTTAGTTGTGCGT	ANKRD17__NM_001286771	891	662	997	814
ANKRD17__NM_001286771_CAGAACAAGCTAAACAAAGG	ANKRD17__NM_001286771	170	96	137	98
ANKRD17__NM_001286771_CAGCAGAACAAGCTAAACAA	ANKRD17__NM_001286771	205	95	289	285
ANKRD17__NM_001286771_CATGCAAATGTGGAAGATAG	ANKRD17__NM_001286771	200	186	274	164
ANKRD17__NM_001286771_CTGAAAGCACAGCAAATGCA	ANKRD17__NM_001286771	64	59	87	141
ANKRD17__NM_001286771_GCATGCAAATGTGGAAGATA	ANKRD17__NM_001286771	454	271	543	464
ANKRD17__NM_001286771_GGTGGAGACAGCGGCTGAAA	ANKRD17__NM_001286771	523	542	544	772
ANKRD17__NM_001286771_GTGCGAAAGTTACTCATTGA	ANKRD17__NM_001286771	340	220	426	335
ANKRD17__NM_001286771_TGTGCTTTCAGCTCTCATAC	ANKRD17__NM_001286771	187	143	184	106
ANKRD17__NM_032217_ACCACAAGGCCAAGCGTAAC	ANKRD17__NM_032217	183	68	286	288
ANKRD17__NM_032217_GGCCTCGTCTCCTCGTGGGA	ANKRD17__NM_032217	311	367	389	372
ANKRD18A__NM_147195_AAAAGAAAAGAACGTGCAAA	ANKRD18A__NM_147195	140	79	122	132
ANKRD18A__NM_147195_AGTGGCCAGGCTAGAATGGA	ANKRD18A__NM_147195	448	294	589	492
ANKRD18A__NM_147195_ATTATGCCGTGTATAATAAG	ANKRD18A__NM_147195	204	69	200	64
ANKRD18A__NM_147195_CAGTCCCGAATGTCGTAACC	ANKRD18A__NM_147195	419	565	723	722
ANKRD18A__NM_147195_CAGTGGCCAGGCTAGAATGG	ANKRD18A__NM_147195	541	592	555	433
ANKRD18A__NM_147195_CCAAACATTGAGGATATCTA	ANKRD18A__NM_147195	840	803	1019	801
ANKRD18A__NM_147195_CCATTATGCCGTGTATAATA	ANKRD18A__NM_147195	549	479	511	722
ANKRD18A__NM_147195_CGGGTCCGGGTTACGACATT	ANKRD18A__NM_147195	997	1224	1113	1104
ANKRD18A__NM_147195_GACCAAGAGTATGCGGGTCC	ANKRD18A__NM_147195	412	613	498	474
ANKRD18A__NM_147195_GGGTCCGGGTTACGACATTC	ANKRD18A__NM_147195	305	303	317	356
ANKRD18A__NM_147195_GTCTGTCACAGATGTCGATC	ANKRD18A__NM_147195	325	201	300	277
ANKRD18A__NM_147195_TGAAGTCCCCTTATTATACA	ANKRD18A__NM_147195	271	86	296	235
ANKRD18B__NM_001244752_AAGAAAGTAGATAGGAGCAA	ANKRD18B__NM_001244752	71	0	87	153
ANKRD18B__NM_001244752_AAGTGAAAATAAACAGCCGC	ANKRD18B__NM_001244752	60	57	165	115
ANKRD18B__NM_001244752_ATTAAGCTGTTGCGAATACT	ANKRD18B__NM_001244752	366	259	460	393
ANKRD18B__NM_001244752_CATCAGGAGTTATTATCTAT	ANKRD18B__NM_001244752	782	634	818	687
ANKRD18B__NM_001244752_GAAGCAGATGCATCCAAATG	ANKRD18B__NM_001244752	317	233	480	348
ANKRD18B__NM_001244752_GACCAAGAGTATGCGGGTCG	ANKRD18B__NM_001244752	712	405	837	535
ANKRD18B__NM_001244752_GCAGTTCCCAGTCCCGAATG	ANKRD18B__NM_001244752	432	392	465	571
ANKRD18B__NM_001244752_GTTTCTAGACATGAAACAAT	ANKRD18B__NM_001244752	109	105	114	169
ANKRD18B__NM_001244752_TCATCAGGAGTTATTATCTA	ANKRD18B__NM_001244752	726	670	893	1014
ANKRD18B__NM_001244752_TGATGATCTTATGGCCGAGA	ANKRD18B__NM_001244752	345	531	264	307
ANKRD18B__NM_001244752_TGGACCAAGAGTATGCGGGT	ANKRD18B__NM_001244752	190	85	208	196
ANKRD18B__NM_001244752_TGGCGCCAATCCAAACATTA	ANKRD18B__NM_001244752	146	151	124	148
ANKRD2__NM_001129981_AGCCGGGGGCTGAGCATAAC	ANKRD2__NM_001129981	608	636	593	611
ANKRD2__NM_001129981_AGGAAAGACCCGACGGACC	ANKRD2__NM_001129981	1141	986	1302	1055
ANKRD2__NM_001129981_ATGGAGCAGACACCAATGTG	ANKRD2__NM_001129981	140	228	191	131
ANKRD2__NM_001129981_CCCTGGAGCATCCTGAGCCG	ANKRD2__NM_001129981	985	891	1121	1002
ANKRD2__NM_001129981_CGGCGGGAGATCATCGATGT	ANKRD2__NM_001129981	1122	984	988	932
ANKRD2__NM_001129981_CTGGAGAAGCTTCTAGATAA	ANKRD2__NM_001129981	311	380	369	360
ANKRD2__NM_001129981_GAGCCGGGGGCTGAGCATAA	ANKRD2__NM_001129981	158	83	113	117
ANKRD2__NM_001129981_GAGCCTCACAGCGTCATGCA	ANKRD2__NM_001129981	724	869	891	870
ANKRD2__NM_001129981_GGAGAAGCTTCTAGATAATG	ANKRD2__NM_001129981	344	240	415	357
ANKRD2__NM_001129981_TCTGGCCTATAAAGCCCCCG	ANKRD2__NM_001129981	414	452	563	726
ANKRD2__NM_001129981_TGGACTGCACAGCCATGCAT	ANKRD2__NM_001129981	83	89	74	85
ANKRD2__NM_001129981_TGGAGAAGCTTCTAGATAAT	ANKRD2__NM_001129981	84	11	59	48
ANKRD2__NM_001291218_AACTCCTGGCCTATTAGAGC	ANKRD2__NM_001291218	571	675	554	557
ANKRD2__NM_001291218_AAGGCAACTGAGTTGCAAAA	ANKRD2__NM_001291218	58	57	105	137
ANKRD2__NM_001291218_CCAGGAGTTGGGGGGGCAAC	ANKRD2__NM_001291218	291	308	342	457
ANKRD2__NM_001291218_CCCCGCTGCACGTGGCAGTC	ANKRD2__NM_001291218	79	127	42	46
ANKRD2__NM_001291218_CCCTCCGGCTCTAATAGGCC	ANKRD2__NM_001291218	276	355	281	354
ANKRD2__NM_020349_TGCACGTGGCAGTCCGGACA	ANKRD2__NM_020349	1901	2145	2120	2249
ANKRD20A3__NM_001012419_TTGCACATGGCCACTGGTAC	ANKRD20A3__NM_001012419	597	829	670	1043
ANKRD22__NM_144590_AGAGTATAGGATTCCCATGC	ANKRD22__NM_144590	787	886	864	722
ANKRD22__NM_144590_AGCTATGCCAACGTTCAAGA	ANKRD22__NM_144590	277	546	245	447
ANKRD22__NM_144590_ATTAAAGCCATCTTGAACGT	ANKRD22__NM_144590	837	698	911	964
ANKRD22__NM_144590_CATGGGAATCCTATACTCTG	ANKRD22__NM_144590	513	478	688	733
ANKRD22__NM_144590_CCTTAGCATTAATTCAATCT	ANKRD22__NM_144590	575	706	592	698
ANKRD22__NM_144590_GTACGAATGCTACTTGATGC	ANKRD22__NM_144590	536	707	570	925
ANKRD22__NM_144590_TCCTTAGCATTAATTCAATC	ANKRD22__NM_144590	307	449	404	349
ANKRD22__NM_144590_TCGCCTGCAAGCACAGATCA	ANKRD22__NM_144590	255	351	329	300
ANKRD22__NM_144590_TTAATGCCTGTTCTGCTTAT	ANKRD22__NM_144590	388	527	428	505
ANKRD22__NM_144590_TTCACAGGCATAATGTAATG	ANKRD22__NM_144590	100	181	103	101
ANKRD23__NM_144994_ACTGTTAAATCTTTCAAGCT	ANKRD23__NM_144994	348	392	312	408
ANKRD23__NM_144994_CAAGTCTCGCGCGTCCACTG	ANKRD23__NM_144994	968	1065	1262	1341
ANKRD23__NM_144994_CAGGTTATCCAGATTAAATC	ANKRD23__NM_144994	171	154	298	221
ANKRD23__NM_144994_CCACCTGAACGCACAGGATA	ANKRD23__NM_144994	334	382	436	564
ANKRD23__NM_144994_CCTCCTCCAGTCACTAGGCC	ANKRD23__NM_144994	690	759	881	937
ANKRD23__NM_144994_CCTTATCCTGTGCGTTCAGG	ANKRD23__NM_144994	784	781	767	1021
ANKRD23__NM_144994_CTCAAACAGCTGCTTAACCA	ANKRD23__NM_144994	251	225	307	241
ANKRD23__NM_144994_GACAAGTACTTGACAGACGG	ANKRD23__NM_144994	259	187	217	207
ANKRD23__NM_144994_GAGACTGGCAGCGCGGCATC	ANKRD23__NM_144994	361	303	288	170
ANKRD23__NM_144994_GAGCCTGGCCTAGTGACTGG	ANKRD23__NM_144994	575	688	614	618
ANKRD23__NM_144994_TAACAGTACCAGATTTAATC	ANKRD23__NM_144994	245	253	312	410
ANKRD23__NM_144994_TTACCTTATCCTGTGCGTTC	ANKRD23__NM_144994	201	249	343	344
ANKRD24__NM_133475_ACAGCTGGCCGCACCCCGCA	ANKRD24__NM_133475	1641	1595	1722	1756
ANKRD24__NM_133475_AGCAATGTCATGAGCGCGGA	ANKRD24__NM_133475	296	159	355	317
ANKRD24__NM_133475_CCCACGAAGCTAGACCCCGA	ANKRD24__NM_133475	1095	801	1183	1117
ANKRD24__NM_133475_CCCTCGGGGTCTAGCTTCGT	ANKRD24__NM_133475	1302	957	1446	1197
ANKRD24__NM_133475_CGAGAGGCTGCTACAAGCCG	ANKRD24__NM_133475	346	415	334	262
ANKRD24__NM_133475_CTGGAGGTGATGATAGCTCA	ANKRD24__NM_133475	9	0	14	4
ANKRD24__NM_133475_GCCCTCGGGGTCTAGCTTCG	ANKRD24__NM_133475	647	591	728	733
ANKRD24__NM_133475_GCCGCCCTCATCGCCCGCAA	ANKRD24__NM_133475	1368	1321	1407	1555
ANKRD24__NM_133475_GGCAGGAGCCAAGGTCAGTG	ANKRD24__NM_133475	242	252	280	348
ANKRD24__NM_133475_GGGCAGGAGCCAAGGTCAGT	ANKRD24__NM_133475	327	279	419	225
ANKRD24__NM_133475_GTGGAGGGCATTGTAACCTG	ANKRD24__NM_133475	197	190	237	125
ANKRD24__NM_133475_TGGAAAACAACGATGCACCT	ANKRD24__NM_133475	106	109	191	204
ANKRD26__NM_001256053_ACGATAGAGACAAGATGAAC	ANKRD26__NM_001256053	161	171	233	277
ANKRD26__NM_001256053_ATGCAGTAGATAAGTTGGAA	ANKRD26__NM_001256053	273	519	361	500
ANKRD26__NM_001256053_CCACTTACTGCAAGTAAAAG	ANKRD26__NM_001256053	245	210	211	282
ANKRD26__NM_001256053_CTAAAGGCTTTCTGGCAAAC	ANKRD26__NM_001256053	123	171	185	143
ANKRD26__NM_001256053_GTCATTAGCTTTGCTAAGCT	ANKRD26__NM_001256053	201	252	173	220
ANKRD26__NM_001256053_TACTACTTCTGGATGACCAT	ANKRD26__NM_001256053	360	450	366	241
ANKRD26__NM_001256053_TCACTATGCTGTCTATAATG	ANKRD26__NM_001256053	351	250	326	468
ANKRD26__NM_001256053_TCATAGGACGGCTCTACATT	ANKRD26__NM_001256053	324	449	391	408
ANKRD26__NM_001256053_TCGGTCTCGGACGTGGTAGC	ANKRD26__NM_001256053	74	98	81	89
ANKRD26__NM_001256053_TGTAAATGCAGTAGATAAGT	ANKRD26__NM_001256053	188	251	220	222
ANKRD26__NM_001256053_TGTGGATCTTGCCGAGATCT	ANKRD26__NM_001256053	238	225	209	212
ANKRD26__NM_001256053_TTTCGCCACATTACCCGCGC	ANKRD26__NM_001256053	415	584	472	423
ANKRD27__NM_032139_AACCTGATCTTTAAATACGT	ANKRD27__NM_032139	368	339	351	358
ANKRD27__NM_032139_AAGGGATCTGAGGGTGCCAA	ANKRD27__NM_032139	161	87	154	118
ANKRD27__NM_032139_ACTCAAACTGACAAGTAGAC	ANKRD27__NM_032139	610	730	584	799
ANKRD27__NM_032139_CAACCTGATCTTTAAATACG	ANKRD27__NM_032139	450	425	477	675
ANKRD27__NM_032139_CAGGTCTTAGTACCCTGCAA	ANKRD27__NM_032139	312	325	365	280
ANKRD27__NM_032139_CGCATTCTCGGAATGTTCGA	ANKRD27__NM_032139	971	1559	1124	1423
ANKRD27__NM_032139_CTTCATCATACAGAGCCATA	ANKRD27__NM_032139	163	184	170	119
ANKRD27__NM_032139_CTTTGCAGGGTACTAAGACC	ANKRD27__NM_032139	280	185	396	326
ANKRD27__NM_032139_GACACTCCGAGCGATTTGAC	ANKRD27__NM_032139	616	605	644	852
ANKRD27__NM_032139_GATGTTCCTGTCAAATCGCT	ANKRD27__NM_032139	684	541	879	673
ANKRD27__NM_032139_TCATGATGGACGTATATCTG	ANKRD27__NM_032139	580	483	802	824
ANKRD27__NM_032139_TCCCTTGAATAAAGACATCC	ANKRD27__NM_032139	165	101	177	168
ANKRD28__NM_001195098_AACATCTTGTCCATTATAGC	ANKRD28__NM_001195098	70	67	139	54
ANKRD28__NM_001195098_AAGATAGGCGTGCTATCCAT	ANKRD28__NM_001195098	835	778	852	612
ANKRD28__NM_001195098_AGAGCTTCTAGTTGGCAATG	ANKRD28__NM_001195098	232	269	248	200
ANKRD28__NM_001195098_AGATAGGCGTGCTATCCATT	ANKRD28__NM_001195098	430	331	408	410
ANKRD28__NM_001195098_ATCCATTGGGCAGCATATAT	ANKRD28__NM_001195098	86	55	114	76
ANKRD28__NM_001195098_CATCAACTCCAAGATCTAGA	ANKRD28__NM_001195098	416	477	458	506
ANKRD28__NM_001195098_GTCAAACTACTCTTGTCTAG	ANKRD28__NM_001195098	1244	1031	1480	1087
ANKRD28__NM_001195098_GTCAAGTACCTTCTAGATCT	ANKRD28__NM_001195098	230	171	263	292
ANKRD28__NM_001195098_GTGAAATTGCTTGTGTCGCA	ANKRD28__NM_001195098	502	839	662	927
ANKRD28__NM_001195098_TAGAGCTTCTAGTTGGCAAT	ANKRD28__NM_001195098	288	356	431	528
ANKRD28__NM_001195098_TGGGGCCGATGTCAATATGA	ANKRD28__NM_001195098	99	34	152	150
ANKRD28__NM_001195098_TTTGCTGCTGCATCAACACA	ANKRD28__NM_001195098	225	377	268	340
ANKRD28__NM_015199_AGCAGCAGCTATATGTAAAG	ANKRD28__NM_015199	303	166	322	185
ANKRD28__NM_015199_AGCAGTTGCATCTTGTAGTG	ANKRD28__NM_015199	495	534	632	585
ANKRD28__NM_015199_AGGTAAGCTGCGGCGTGCAA	ANKRD28__NM_015199	698	562	588	544
ANKRD28__NM_015199_GATGCAACTGCTCTGTGTAA	ANKRD28__NM_015199	693	900	965	843
ANKRD28__NM_015199_GTAAGCTGCGGCGTGCAATG	ANKRD28__NM_015199	484	340	524	599
ANKRD28__NM_015199_TAGCAGCAGCTATATGTAAA	ANKRD28__NM_015199	422	463	590	569
ANKRD28__NM_015199_TGCATCTCCAAGGTAAGCTG	ANKRD28__NM_015199	11	0	35	15
ANKRD28__NM_015199_TTAATGCTCGAGACAAAAAT	ANKRD28__NM_015199	85	54	139	186
ANKRD28__NM_015199_TTAGCAGCAGCTATATGTAA	ANKRD28__NM_015199	537	465	688	602
ANKRD28__NM_015199_TTGCACGCCGCAGCTTACCT	ANKRD28__NM_015199	570	396	595	676
ANKRD29__NM_173505_AGCTGCCCAAGGTGGTTACT	ANKRD29__NM_173505	0	0	0	0
ANKRD29__NM_173505_AGGGGCGCTGTCCCGTCCTG	ANKRD29__NM_173505	830	587	764	780
ANKRD29__NM_173505_CCATCTGGGACGCGATCCAC	ANKRD29__NM_173505	1326	1420	1293	1085
ANKRD29__NM_173505_CTGAAGCTGCTGTTGAACAG	ANKRD29__NM_173505	263	207	228	193
ANKRD29__NM_173505_CTGCATGTGCCCGTACTGAC	ANKRD29__NM_173505	1177	1267	1176	1178
ANKRD29__NM_173505_CTGGCCACATAGACTGTGTG	ANKRD29__NM_173505	776	770	752	1017
ANKRD29__NM_173505_CTGTGGATCGCGTCCCAGAT	ANKRD29__NM_173505	574	848	495	746
ANKRD29__NM_173505_GACATCATTATACCCTTTGT	ANKRD29__NM_173505	1094	1169	1261	1314
ANKRD29__NM_173505_GCATGGCACCACACTCCTGA	ANKRD29__NM_173505	1667	1552	1769	1484
ANKRD29__NM_173505_GGATGTTATTCGATTACTGC	ANKRD29__NM_173505	297	453	306	431
ANKRD29__NM_173505_GTTCCGCGCAGCGTCGCGGT	ANKRD29__NM_173505	94	55	128	201
ANKRD29__NM_173505_TACCTTCAAAATACCAAGAG	ANKRD29__NM_173505	183	113	136	148
ANKRD30A__NM_052997_AAAGAAACACCTGTAAAGAC	ANKRD30A__NM_052997	173	182	240	271
ANKRD30A__NM_052997_AACATCTGAGAAATTTACGT	ANKRD30A__NM_052997	804	867	851	861
ANKRD30A__NM_052997_AGAAAATTCTTGGGATACTG	ANKRD30A__NM_052997	91	19	119	107
ANKRD30A__NM_052997_ATAGAGTCTCCTAATAAAGA	ANKRD30A__NM_052997	216	233	184	196
ANKRD30A__NM_052997_CACATCTACGAGATTTATAT	ANKRD30A__NM_052997	501	519	497	597
ANKRD30A__NM_052997_CGCCATCAAGGACGTCAAGC	ANKRD30A__NM_052997	479	627	483	493
ANKRD30A__NM_052997_GATCACTGTGAACAACGTAC	ANKRD30A__NM_052997	358	381	289	531
ANKRD30A__NM_052997_GCCACGCATCCAGTCTTTAC	ANKRD30A__NM_052997	1077	1099	1047	1231
ANKRD30A__NM_052997_GTCAGCAGAAGAAACACCTA	ANKRD30A__NM_052997	407	442	424	490
ANKRD30A__NM_052997_GTCTTGTATATTAAGGTTGA	ANKRD30A__NM_052997	62	43	62	135
ANKRD30A__NM_052997_TCTGGGCGTCTTGTATATTA	ANKRD30A__NM_052997	709	780	750	881
ANKRD30A__NM_052997_TTCAGAAGACCATCTTTATT	ANKRD30A__NM_052997	119	201	163	252
ANKRD30B__NM_001145029_ACAGGCTCTACAATGCGAGA	ANKRD30B__NM_001145029	214	214	200	292
ANKRD30B__NM_001145029_ACCATCTACTTCGGGGATCT	ANKRD30B__NM_001145029	808	879	816	863
ANKRD30B__NM_001145029_AGTCATCGAGGTGCAAAACA	ANKRD30B__NM_001145029	188	236	279	218
ANKRD30B__NM_001145029_AGTGTAGACCCGTTCGCTGA	ANKRD30B__NM_001145029	1896	1872	2070	2377
ANKRD30B__NM_001145029_ATGTCCTTGATGGCGAAGGG	ANKRD30B__NM_001145029	455	350	532	735
ANKRD30B__NM_001145029_CCATCTACTTCGGGGATCTA	ANKRD30B__NM_001145029	239	287	355	464
ANKRD30B__NM_001145029_CCCCGAACCCCTTCAGCGAA	ANKRD30B__NM_001145029	631	975	735	848
ANKRD30B__NM_001145029_CCCGAACCCCTTCAGCGAAC	ANKRD30B__NM_001145029	1054	1399	1284	1913
ANKRD30B__NM_001145029_GAAAAGAAGCAAGCAAACTG	ANKRD30B__NM_001145029	105	251	257	236
ANKRD30B__NM_001145029_GACAGGCTCTACAATGCGAG	ANKRD30B__NM_001145029	438	522	450	476
ANKRD30B__NM_001145029_GTGTAGACCCGTTCGCTGAA	ANKRD30B__NM_001145029	393	447	465	516
ANKRD30B__NM_001145029_TTTCTGTATGGCCAGTAAAA	ANKRD30B__NM_001145029	26	57	51	12
ANKRD31__NM_001164443_AGATTGTACACAAATAGCAG	ANKRD31__NM_001164443	85	98	54	61
ANKRD31__NM_001164443_AGGGCTTCCAATAACTCAGC	ANKRD31__NM_001164443	92	108	114	123
ANKRD31__NM_001164443_ATCACCAGAAAGCACCCAGG	ANKRD31__NM_001164443	386	504	464	325
ANKRD31__NM_001164443_ATTGGGTCGTTTCGCCAGTC	ANKRD31__NM_001164443	323	439	366	498
ANKRD31__NM_001164443_ATTGTATCCTCGCTAAGAAC	ANKRD31__NM_001164443	182	297	164	103
ANKRD31__NM_001164443_CAGAAATGAATGTTTAGAAG	ANKRD31__NM_001164443	351	520	426	613
ANKRD31__NM_001164443_GATCCGGCTTTAATGATTGA	ANKRD31__NM_001164443	732	1092	979	1070
ANKRD31__NM_001164443_GATGATGCCTGTTCTTAGCG	ANKRD31__NM_001164443	666	769	736	733
ANKRD31__NM_001164443_GGAATTCAAAGGTATTGATA	ANKRD31__NM_001164443	132	142	211	132
ANKRD31__NM_001164443_TCCATCTGGATTGCAAACTT	ANKRD31__NM_001164443	349	331	421	346
ANKRD31__NM_001164443_TGCATTGCCAGCTGAGTTAT	ANKRD31__NM_001164443	354	393	444	473
ANKRD31__NM_001164443_TTACCATCAATCATTAAAGC	ANKRD31__NM_001164443	37	114	82	82
ANKRD32__NM_032290_AAGCATATCATCCAGATGAC	ANKRD32__NM_032290	334	288	397	389
ANKRD32__NM_032290_AAGCCATACATATGAAAATC	ANKRD32__NM_032290	127	59	93	96
ANKRD32__NM_032290_AAGTGATTCTCTTATAAGGT	ANKRD32__NM_032290	67	41	73	55
ANKRD32__NM_032290_AGCGAAGTGATTCTCTTATA	ANKRD32__NM_032290	427	360	482	290
ANKRD32__NM_032290_AGCTTTAATTCTTGCATTAC	ANKRD32__NM_032290	466	498	449	629
ANKRD32__NM_032290_ATTGCATTTGAGGTGAATAA	ANKRD32__NM_032290	132	128	160	188
ANKRD32__NM_032290_CGTGAAGAACTGAAACGCAC	ANKRD32__NM_032290	329	530	460	357
ANKRD32__NM_032290_GATGAAACAACTTATGAATG	ANKRD32__NM_032290	379	226	365	445
ANKRD32__NM_032290_GCACATATATAATAGAGATC	ANKRD32__NM_032290	228	344	267	340
ANKRD32__NM_032290_TTATCCAATTCAGTATCTAG	ANKRD32__NM_032290	158	41	151	154
ANKRD32__NM_032290_TTCAGAGGAGTTATACTTTG	ANKRD32__NM_032290	111	286	140	153
ANKRD32__NM_032290_TTCGCTTATCAGTTCTAACA	ANKRD32__NM_032290	52	25	67	30
ANKRD33__NM_001130015_ACAGCTTCGACACCGTGTGG	ANKRD33__NM_001130015	691	715	707	453
ANKRD33__NM_001130015_AGCTCCTGGTATCGCCAGAG	ANKRD33__NM_001130015	232	174	292	195
ANKRD33__NM_001130015_AGGGCCGTCTTGCCCCGAAC	ANKRD33__NM_001130015	221	160	168	185
ANKRD33__NM_001130015_CAGCCGTTCTAGGAGTGAAG	ANKRD33__NM_001130015	420	442	275	265
ANKRD33__NM_001130015_CCCTGACCATCCACCTTCGC	ANKRD33__NM_001130015	2284	2483	2700	2215
ANKRD33__NM_001130015_CCGACAGCTTCGACACCGTG	ANKRD33__NM_001130015	2655	3264	2865	3214
ANKRD33__NM_001130015_CCTGACCATCCACCTTCGCT	ANKRD33__NM_001130015	2816	2081	2877	1462
ANKRD33__NM_001130015_CTCATGGTCGCATGCTACCA	ANKRD33__NM_001130015	890	895	1061	1499
ANKRD33__NM_001130015_CTCCTGCTCAACTACTATGT	ANKRD33__NM_001130015	852	569	886	417
ANKRD33__NM_001130015_GGGTGGCCACGCACTCACAG	ANKRD33__NM_001130015	570	450	561	540
ANKRD33__NM_001130015_TGGGCCTGGACCTGGAACGC	ANKRD33__NM_001130015	81	250	93	103
ANKRD33__NM_001130015_TGGTTGTGGCAGTCAACAAGG	ANKRD33__NM_001130015	10	14	7	0
ANKRD33__NM_182608_ATCTATGAGGCAGTCAACGC	ANKRD33__NM_182608	229	87	227	102
ANKRD33__NM_182608_CAACGCAGGTAACAGATGGC	ANKRD33__NM_182608	787	569	878	670
ANKRD33__NM_182608_CCCCACCATCCAGTATGGCT	ANKRD33__NM_182608	299	213	306	111
ANKRD33__NM_182608_CTGGGGAGGGATAGTCCACC	ANKRD33__NM_182608	611	491	543	289
ANKRD33__NM_182608_GACCCAGTGATTGTGCTCCG	ANKRD33__NM_182608	890	1066	943	918
ANKRD33__NM_182608_GCTCCGCGGAGCACAATCAC	ANKRD33__NM_182608	101	55	82	55
ANKRD33__NM_182608_TCTATGAGGCAGTCAACGCG	ANKRD33__NM_182608	168	219	235	150
ANKRD33B__NM_001164440_AGGAGACTGACCGCAACGGC	ANKRD33B__NM_001164440	245	336	290	409
ANKRD33B__NM_001164440_CAAACTCTTCGTAGTCAGCG	ANKRD33B__NM_001164440	351	707	476	609
ANKRD33B__NM_001164440_CCGCAGCAGCCCCACGTTGT	ANKRD33B__NM_001164440	2989	3154	3127	3890
ANKRD33B__NM_001164440_CGACTCTTTCTACCCTTTCG	ANKRD33B__NM_001164440	1161	1255	1536	1936
ANKRD33B__NM_001164440_CGGCTTTGTGGATACCGTGG	ANKRD33B__NM_001164440	900	1206	1061	1297
ANKRD33B__NM_001164440_CGTGGTAGCAGGCGACAATA	ANKRD33B__NM_001164440	76	41	101	80
ANKRD33B__NM_001164440_CTCAAACTCTTCGTAGTCAG	ANKRD33B__NM_001164440	274	442	292	471
ANKRD33B__NM_001164440_GCGCAGGAGACTGACCGCAA	ANKRD33B__NM_001164440	492	306	758	483
ANKRD33B__NM_001164440_GCTCCTCCTCGTCCTCGAAA	ANKRD33B__NM_001164440	323	306	317	290
ANKRD33B__NM_001164440_TCAAACTCTTCGTAGTCAGC	ANKRD33B__NM_001164440	125	153	153	175
ANKRD33B__NM_001164440_TGCCAGTTGACGTCAACGTG	ANKRD33B__NM_001164440	236	145	250	210
ANKRD33B__NM_001164440_TTTCTACCCTTTCGAGGACG	ANKRD33B__NM_001164440	111	149	84	189
ANKRD34A__NM_001039888_CCAATATCGCAGACCGATTA	ANKRD34A__NM_001039888	632	484	721	626
ANKRD34A__NM_001039888_CCCAATATCGCAGACCGATT	ANKRD34A__NM_001039888	1050	929	1226	1090
ANKRD34A__NM_001039888_CCTAATCGGTCTGCGATATT	ANKRD34A__NM_001039888	652	942	668	828
ANKRD34A__NM_001039888_CGCAGCTATGTTGCACACCG	ANKRD34A__NM_001039888	1877	1608	1972	2338
ANKRD34A__NM_001039888_CTAATCGGTCTGCGATATTG	ANKRD34A__NM_001039888	792	832	735	811
ANKRD34A__NM_001039888_GAATGAGGGTGATGCGCAGC	ANKRD34A__NM_001039888	873	894	883	798
ANKRD34A__NM_001039888_GCAGCTATGTTGCACACCGA	ANKRD34A__NM_001039888	520	168	454	349
ANKRD34A__NM_001039888_GCAGGGGGAGACTGCGCTAA	ANKRD34A__NM_001039888	688	732	869	739
ANKRD34A__NM_001039888_GGAGGCGCCTACGTGAATGA	ANKRD34A__NM_001039888	578	639	790	653
ANKRD34A__NM_001039888_GGGAGGCGCCTACGTGAATG	ANKRD34A__NM_001039888	873	1032	1010	1155
ANKRD34A__NM_001039888_GTTCTGGGGGTCGTCGTAGC	ANKRD34A__NM_001039888	65	143	75	125
ANKRD34A__NM_001039888_TGAATGAGGGTGATGCGCAG	ANKRD34A__NM_001039888	271	315	312	195
ANKRD34B__NM_001004441_ACTCTGGTGATCGACATGTT	ANKRD34B__NM_001004441	2321	2438	2520	2293
ANKRD34B__NM_001004441_CAATGCCGATCCCAACATAC	ANKRD34B__NM_001004441	684	815	813	879
ANKRD34B__NM_001004441_CCAGATTTGTCCTGTATGTT	ANKRD34B__NM_001004441	1249	1568	1591	1807
ANKRD34B__NM_001004441_CTTCTAGCAAAAGTCTTGTG	ANKRD34B__NM_001004441	239	466	328	380
ANKRD34B__NM_001004441_GATCGGCATTGTTCTCTAAC	ANKRD34B__NM_001004441	625	555	725	950
ANKRD34B__NM_001004441_GGTATGGAAATTTCAAGTGA	ANKRD34B__NM_001004441	300	202	264	292
ANKRD34B__NM_001004441_GTCTTACAAGCGATCATTAA	ANKRD34B__NM_001004441	89	157	163	113
ANKRD34B__NM_001004441_TAATGAGAGCAACGACCGTG	ANKRD34B__NM_001004441	285	213	280	203
ANKRD34B__NM_001004441_TCAAAGCAGTCCATCAGAGC	ANKRD34B__NM_001004441	93	159	125	100
ANKRD34B__NM_001004441_TCTTACAAGCGATCATTAAA	ANKRD34B__NM_001004441	201	184	200	173
ANKRD34B__NM_001004441_TTAATGAGAGCAACGACCGT	ANKRD34B__NM_001004441	155	127	169	153
ANKRD34B__NM_001004441_TTTGTCCTGTATGTTGGGAT	ANKRD34B__NM_001004441	536	830	542	768
ANKRD34C__NM_001146341_CCAAGGCTGTTAATGAGCCT	ANKRD34C__NM_001146341	292	236	238	325
ANKRD34C__NM_001146341_CCAGCACCGAGGGCGCTATC	ANKRD34C__NM_001146341	388	393	419	576
ANKRD34C__NM_001146341_CCAGCCTTGAAGATCGCACT	ANKRD34C__NM_001146341	1063	1135	1242	1148
ANKRD34C__NM_001146341_CCCAGCCTTGAAGATCGCAC	ANKRD34C__NM_001146341	415	293	537	530
ANKRD34C__NM_001146341_CCCAGGCTCATTAACAGCCT	ANKRD34C__NM_001146341	305	312	388	591
ANKRD34C__NM_001146341_CTTCACCCAAAGTAGAAGAC	ANKRD34C__NM_001146341	45	33	53	43
ANKRD34C__NM_001146341_GAAAGTCAGTAATCTCAAAA	ANKRD34C__NM_001146341	57	91	93	44
ANKRD34C__NM_001146341_GACACTGAATTAAGGACTGA	ANKRD34C__NM_001146341	1175	1576	1430	1548
ANKRD34C__NM_001146341_GACCAGGCTGCTCTTGGAAG	ANKRD34C__NM_001146341	189	157	269	161
ANKRD34C__NM_001146341_GGAAAGTCAGTAATCTCAAA	ANKRD34C__NM_001146341	289	417	265	413
ANKRD34C__NM_001146341_TCAGGCCCCAAGGTTCAGAC	ANKRD34C__NM_001146341	100	73	151	86
ANKRD34C__NM_001146341_TGGGGAGGCTCAGGTTGACC	ANKRD34C__NM_001146341	106	139	118	78
ANKRD35__NM_001280799_ACCTTCCATGGACTTCATAC	ANKRD35__NM_001280799	1002	706	1054	993
ANKRD35__NM_001280799_AGCTAATACTGGATTTACTG	ANKRD35__NM_001280799	348	133	441	278
ANKRD35__NM_001280799_CACCCAGCGATGCGATCATC	ANKRD35__NM_001280799	259	139	303	234
ANKRD35__NM_001280799_CATGGAGCAGGGTTGTCCTA	ANKRD35__NM_001280799	578	411	671	446
ANKRD35__NM_001280799_CCAGGATCAAAGCCGATCTG	ANKRD35__NM_001280799	520	610	587	509
ANKRD35__NM_001280799_CTGATGATCGCATCGCTGGG	ANKRD35__NM_001280799	432	447	401	466
ANKRD35__NM_001280799_CTTACCTGGGATGCGAGATC	ANKRD35__NM_001280799	100	75	146	165
ANKRD35__NM_001280799_GCAGGAGAAGATACGCTTCA	ANKRD35__NM_001280799	936	636	1127	796
ANKRD35__NM_001280799_GCTCTCCATGAGCTCTTAGG	ANKRD35__NM_001280799	85	9	82	53
ANKRD35__NM_001280799_GGAGGATGAAGACCCGTGCT	ANKRD35__NM_001280799	1280	1688	1367	1308
ANKRD35__NM_001280799_TCACATTATCCAACACGTCC	ANKRD35__NM_001280799	345	162	268	159
ANKRD35__NM_001280799_TGATGATCGCATCGCTGGGT	ANKRD35__NM_001280799	382	227	347	362
ANKRD35__NM_144698_CCATTCGAGTCAAGCTTGGT	ANKRD35__NM_144698	2045	1341	2354	1676
ANKRD35__NM_144698_CTGGCCATTCGAGTCAAGCT	ANKRD35__NM_144698	442	455	464	439
ANKRD36__NM_001164315_AACCATACTTTACGAACAGA	ANKRD36__NM_001164315	602	783	614	1005
ANKRD36__NM_001164315_ATACGGTTTAATGGGGTATT	ANKRD36__NM_001164315	223	166	296	315
ANKRD36__NM_001164315_CACTCTATTCTTAGCCTCAA	ANKRD36__NM_001164315	399	685	429	476
ANKRD36__NM_001164315_CTGTTGAACAGTGTTTAAAC	ANKRD36__NM_001164315	55	57	93	83
ANKRD36__NM_001164315_GATACGGTTTAATGGGGTAT	ANKRD36__NM_001164315	441	397	473	348
ANKRD36__NM_001164315_GCTGTGCAAAACGGTCCAGT	ANKRD36__NM_001164315	1266	902	1379	1261
ANKRD36__NM_001164315_TACGGTTTAATGGGGTATTG	ANKRD36__NM_001164315	106	95	101	89
ANKRD36__NM_001164315_TCACAGGACCGCCCTACATT	ANKRD36__NM_001164315	1031	423	1323	599
ANKRD36__NM_001164315_TCAGATGATACGGTTTAATG	ANKRD36__NM_001164315	280	116	278	179
ANKRD36__NM_001164315_TCTCCACGATCTATAAAAGA	ANKRD36__NM_001164315	212	236	228	195
ANKRD36__NM_001164315_TGCAGGAGATTATGCCATTG	ANKRD36__NM_001164315	550	975	795	721
ANKRD36__NM_001164315_TTCAGATGATACGGTTTAAT	ANKRD36__NM_001164315	23	15	38	13
ANKRD36B__NM_025190_ATAAAGGAGGGACCTATATC	ANKRD36B__NM_025190	219	172	138	173
ANKRD36B__NM_025190_ATAAAGGATGGACTACAATG	ANKRD36B__NM_025190	205	139	214	179
ANKRD36B__NM_025190_ATATTACGGATGTCTTTGGA	ANKRD36B__NM_025190	297	258	364	265
ANKRD36B__NM_025190_ATCCACAGAGCTGTCTTACG	ANKRD36B__NM_025190	959	670	956	822
ANKRD36B__NM_025190_CCACAAGAATAATGGGCGGT	ANKRD36B__NM_025190	679	856	799	778
ANKRD36B__NM_025190_CCCACCGCCCGTTATTCTTG	ANKRD36B__NM_025190	2887	3247	3448	4223
ANKRD36B__NM_025190_GATGGAGAAAAAACTAGGAG	ANKRD36B__NM_025190	197	101	190	95
ANKRD36B__NM_025190_GATGGAGAAATATCTAGGAC	ANKRD36B__NM_025190	367	492	511	506
ANKRD36B__NM_025190_GCCACAAGAATAACGGGCGG	ANKRD36B__NM_025190	922	1118	895	1339
ANKRD36B__NM_025190_TAAAGGAGGGACCTATATCT	ANKRD36B__NM_025190	256	167	339	185
ANKRD36B__NM_025190_TAAAGGATGGACTACAATGT	ANKRD36B__NM_025190	143	58	228	187
ANKRD36B__NM_025190_TCATAAACAACCATCCTTGA	ANKRD36B__NM_025190	1457	1698	1889	2310
ANKRD37__NM_181726_AAACAGCTGAAGATCTCGCT	ANKRD37__NM_181726	293	328	320	357
ANKRD37__NM_181726_AAGCAAGTGAACACCCTGAC	ANKRD37__NM_181726	810	1018	911	1139
ANKRD37__NM_181726_ATCCGGGGGTGCGTTGACCG	ANKRD37__NM_181726	732	1168	1028	1154
ANKRD37__NM_181726_CACTGGCTACAAGCAGGCTT	ANKRD37__NM_181726	227	364	230	297
ANKRD37__NM_181726_CGACTGCTTGCAGGGATCCG	ANKRD37__NM_181726	847	932	955	1007
ANKRD37__NM_181726_CTTCCTGCGGCTAAGTGGAC	ANKRD37__NM_181726	410	378	405	439
ANKRD37__NM_181726_GGAAGTGTAGAAAATACCAG	ANKRD37__NM_181726	228	356	262	423
ANKRD39__NM_016466_CAGCACTATGCCAGCCGCAA	ANKRD39__NM_016466	575	995	692	739
ANKRD39__NM_016466_CCGTGCCTTTCGGTCCCGGA	ANKRD39__NM_016466	720	971	752	913
ANKRD39__NM_016466_CGCGCTGGGATGCGAGCAGC	ANKRD39__NM_016466	191	280	245	283
ANKRD39__NM_016466_GAAGGCCATCCGGGACCGAA	ANKRD39__NM_016466	355	357	380	287
ANKRD39__NM_016466_GAGGAGATGGACTTCGAGAG	ANKRD39__NM_016466	331	340	352	352
ANKRD39__NM_016466_GGGCTGGGCTGTGTTGCAGG	ANKRD39__NM_016466	804	710	820	845
ANKRD40__NM_052855_AGAGAAATCAGGAAGATTAT	ANKRD40__NM_052855	152	107	99	105
ANKRD40__NM_052855_CCCAAAATGAGGTCAACGGC	ANKRD40__NM_052855	327	333	330	289
ANKRD40__NM_052855_CCGAATGTCCCCTAAGGCCG	ANKRD40__NM_052855	493	455	559	449
ANKRD40__NM_052855_CTCCTTGATGTTAACTGGAC	ANKRD40__NM_052855	590	634	545	681
ANKRD40__NM_052855_CTGCTCCTTCTGCTCTAGGA	ANKRD40__NM_052855	369	371	388	281
ANKRD40__NM_052855_GGCCTTAGGGGACATTCGGG	ANKRD40__NM_052855	478	416	465	329
ANKRD40__NM_052855_GTACAGGACTTGTTTACACT	ANKRD40__NM_052855	856	767	889	1081
ANKRD40__NM_052855_GTCTCTTACCTGTTAAAATC	ANKRD40__NM_052855	179	242	205	191
ANKRD40__NM_052855_TGCCAGTCCAGTTAACATCA	ANKRD40__NM_052855	847	730	938	828
ANKRD40__NM_052855_TGCGGGAGGCCGCGGCCTTA	ANKRD40__NM_052855	203	159	264	174
ANKRD40__NM_052855_TGGGCATGTAAACGAAACCA	ANKRD40__NM_052855	91	39	115	169
ANKRD40__NM_052855_TTTCTCTCCTTGATGTTAAC	ANKRD40__NM_052855	16	46	45	62
ANKRD42__NM_182603_AAAGCAGCTTTCAGAAATAG	ANKRD42__NM_182603	227	299	296	252
ANKRD42__NM_182603_ATAGAAGATGTGGACTACAA	ANKRD42__NM_182603	136	130	275	171
ANKRD42__NM_182603_ATCACACACGTAACAACGAG	ANKRD42__NM_182603	184	155	202	194
ANKRD42__NM_182603_ATGTACTGGATTTGGCCCAG	ANKRD42__NM_182603	264	417	278	232
ANKRD42__NM_182603_CACACGTAACAACGAGAGGT	ANKRD42__NM_182603	325	265	227	273
ANKRD42__NM_182603_CATCCCCAAATCACCCTTAA	ANKRD42__NM_182603	695	953	867	1097
ANKRD42__NM_182603_CATCTCACATAGCTGCAATC	ANKRD42__NM_182603	128	104	144	187
ANKRD42__NM_182603_GCTGCCTTTAAGGGTGATTT	ANKRD42__NM_182603	517	622	640	1067
ANKRD42__NM_182603_GGCTGCCTTTAAGGGTGATT	ANKRD42__NM_182603	685	953	806	1259
ANKRD42__NM_182603_TCTCTCTTATCAGTCACACT	ANKRD42__NM_182603	231	392	332	322
ANKRD42__NM_182603_TGAAAAGCTGCATAATGCAC	ANKRD42__NM_182603	269	243	257	222
ANKRD42__NM_182603_TGAGTTGCAGCAAGATGTAA	ANKRD42__NM_182603	19	18	57	22
ANKRD44__NM_001195144_AGTGCTCAAACTCACCGACC	ANKRD44__NM_001195144	523	373	597	606
ANKRD44__NM_001195144_CATCGCCTGCTACAATGGAC	ANKRD44__NM_001195144	1059	869	1336	1343
ANKRD44__NM_001195144_CGGGGCAGATGTTAACATTC	ANKRD44__NM_001195144	676	652	865	863
ANKRD44__NM_001195144_CGTGGCCGTTCAGAGCCGCA	ANKRD44__NM_001195144	746	804	761	908
ANKRD44__NM_001195144_CTGGGCAGCATACATGGGTA	ANKRD44__NM_001195144	202	187	164	176
ANKRD44__NM_001195144_GTAGCATTGCTCATTAACCA	ANKRD44__NM_001195144	21	18	41	44
ANKRD44__NM_001195144_GTCAATGTCTCCGACCGAGG	ANKRD44__NM_001195144	398	198	321	226
ANKRD44__NM_001195144_GTGAACCAGCCAAACAATAA	ANKRD44__NM_001195144	57	94	68	73
ANKRD44__NM_001195144_GTGTCAATGTCTCCGACCGA	ANKRD44__NM_001195144	438	206	464	371
ANKRD44__NM_001195144_TGAACCAGCCAAACAATAAT	ANKRD44__NM_001195144	223	132	229	254
ANKRD44__NM_001195144_TGAATTGTTAGTAAACAACG	ANKRD44__NM_001195144	73	17	56	93
ANKRD44__NM_001195144_TTTGCTGCTGCCTCCACTCA	ANKRD44__NM_001195144	531	613	712	911
ANKRD45__NM_198493_AAGTGACGTGATTAGAGCTT	ANKRD45__NM_198493	171	92	273	253
ANKRD45__NM_198493_CATGCCGTGCAAAAAATGAG	ANKRD45__NM_198493	407	204	387	345
ANKRD45__NM_198493_CCGTGCAAAAAATGAGTGGT	ANKRD45__NM_198493	1234	1634	1332	1712
ANKRD45__NM_198493_CTGACACAGAAAAGGGATCA	ANKRD45__NM_198493	393	470	411	535
[illegible]	[illegible]	[illegible]	[illegible]	[illegible]	[illegible]

AP2M1__NM_001025205_CGATGTCATCTCGGTAGACT	AP2M1__NM_001025205	1082	1563	1238	1599
AP2M1__NM_001025205_CGCCTGTCTCGGAATTCTGT	AP2M1__NM_001025205	2360	345	2866	236
AP2M1__NM_001025205_GATGTCATCTCGGTAGACTC	AP2M1__NM_001025205	324	392	222	205
AP2M1__NM_001025205_GCAGCAGGGCATCAAGAGTC	AP2M1__NM_001025205	415	155	413	185
AP2M1__NM_001025205_GCGCCTGTCTCGGAATTCTG	AP2M1__NM_001025205	1876	1982	2239	2296
AP2M1__NM_001025205_GGGTCAATGTTATCCATGCC	AP2M1__NM_001025205	429	562	444	715
AP2M1__NM_001025205_TACCCACAGAATTCCGAGAC	AP2M1__NM_001025205	848	264	1137	250
AP2S1__NM_004069_ACTGCATGTACCACTTGGCC	AP2S1__NM_004069	1437	190	1898	348
AP2S1__NM_004069_ATGAGGATAAAGCGGATCTG	AP2S1__NM_004069	202	6	185	27
AP2S1__NM_004069_CAAACACACCAACTTTGTGG	AP2S1__NM_004069	400	90	576	53
AP2S1__NM_004069_CTCCACAAAGTTGGTGTGTT	AP2S1__NM_004069	548	803	603	692
AP2S1__NM_004069_GAAATATTCGTTTAAGACCT	AP2S1__NM_004069	167	112	155	96
AP2S1__NM_004069_GCTGTTTCAGCACCTTCGTC	AP2S1__NM_004069	787	626	884	1216
AP2S1__NM_004069_GGAAATATTCGTTTAAGACC	AP2S1__NM_004069	128	121	126	192
AP2S1__NM_004069_GGATGAGGATAAAGCGGATC	AP2S1__NM_004069	262	76	301	96
AP2S1__NM_004069_GGTAAATGATCTTAAAGTTC	AP2S1__NM_004069	137	13	111	48
AP2S1__NM_004069_TCTCTCGGATTTCGCCAGCC	AP2S1__NM_004069	988	1641	1206	1461
AP3B1__NM_001271769_AAGCGCTTGCACGAATTAGT	AP3B1__NM_001271769	722	475	786	510
AP3B1__NM_001271769_ACTAGTGGATGTTGAAGAGT	AP3B1__NM_001271769	242	288	347	252
AP3B1__NM_001271769_AGCTCGCTGAAAAGTGCTTA	AP3B1__NM_001271769	236	292	249	266
AP3B1__NM_001271769_CAAGCTATGTAACTTACTAG	AP3B1__NM_001271769	805	606	790	829
AP3B1__NM_001271769_CTGACTTATCACCATATGTT	AP3B1__NM_001271769	619	684	796	643
AP3B1__NM_001271769_CTTGCACGAATTAGTTGGTT	AP3B1__NM_001271769	1152	1222	1445	1640
AP3B1__NM_001271769_GAAGTTGGTATATGTTTACC	AP3B1__NM_001271769	201	227	183	202
AP3B1__NM_001271769_GCTGAAAAGTGCTTATGGAC	AP3B1__NM_001271769	1042	1190	1260	1344
AP3B1__NM_001271769_GCTGCATTCTTCCTAACATA	AP3B1__NM_001271769	491	612	481	420
AP3B1__NM_001271769_GTTCTTCAGCATATCGAACC	AP3B1__NM_001271769	3	0	3	2
AP3B1__NM_001271769_TATCATGATGCTTGCTATTA	AP3B1__NM_001271769	360	356	380	298
AP3B1__NM_001271769_TGTGGCCAGTAAAAATATTG	AP3B1__NM_001271769	215	195	334	380
AP3B1__NM_003664_CCTCCGGACTGCTCATTGTA	AP3B1__NM_003664	1139	812	1326	1042
AP3B1__NM_003664_TTTCCTTACAATGAGCAGTC	AP3B1__NM_003664	194	275	201	238
AP3B2__NM_001278511_ACGCCATCCCTAAACTCTAC	AP3B2__NM_001278511	1255	1475	1539	1425
AP3B2__NM_001278511_CCCCGCCTACAGCGAAGACA	AP3B2__NM_001278511	218	467	342	412
AP3B2__NM_001278511_CCCGCCTACAGCGAAGACAA	AP3B2__NM_001278511	62	47	76	66
AP3B2__NM_001278511_CGTGGCCTGTAAGAACATAG	AP3B2__NM_001278511	152	76	171	174
AP3B2__NM_001278511_CTCCTTCAGGTCATCATGCC	AP3B2__NM_001278511	304	435	451	545
AP3B2__NM_001278511_GCGGGGTCGTGGCCGTACTC	AP3B2__NM_001278511	685	944	880	815
AP3B2__NM_001278511_GGGCACTGGCACGAATCAGC	AP3B2__NM_001278511	337	258	359	488
AP3B2__NM_001278511_GTCTTCGCTGTAGGCGGGGG	AP3B2__NM_001278511	110	181	217	301
AP3B2__NM_001278511_TCCTCCGACTACAAGCGGTG	AP3B2__NM_001278511	39	72	35	66
AP3B2__NM_001278511_TCTTCTCCTCCGACTACAAG	AP3B2__NM_001278511	13	16	9	16
AP3B2__NM_001278511_TGCTTCAGACCTGTTTCCCG	AP3B2__NM_001278511	180	96	248	155
AP3B2__NM_001278511_TGTAGAGTTTAGGGATGGCG	AP3B2__NM_001278511	259	307	304	320
AP3B2__NM_001278512_GAAGCTTGTCTATGTGTACC	AP3B2__NM_001278512	939	867	879	856
AP3B2__NM_001278512_GCTCCTCAGCGTAGCGTACC	AP3B2__NM_001278512	38	64	92	105
AP3B2__NM_001278512_GTACCTGGTACGCTACGCTG	AP3B2__NM_001278512	311	389	327	315
AP3D1__NM_001261826_ACCAATCAGATCCGTAAGGT	AP3D1__NM_001261826	378	159	309	229
AP3D1__NM_001261826_AGCCCCAGCCAGTACGACAC	AP3D1__NM_001261826	902	669	998	700
AP3D1__NM_001261826_CACCAATCAGATCCGTAAGG	AP3D1__NM_001261826	839	514	915	601
AP3D1__NM_001261826_CAGCGAGGTAGCCAATTCGC	AP3D1__NM_001261826	1277	835	1460	1111
AP3D1__NM_001261826_CATAGCGGTGAAGGCGAACG	AP3D1__NM_001261826	71	91	125	87
AP3D1__NM_001261826_CCGCGGCATCCGTAACCACA	AP3D1__NM_001261826	1078	983	1212	1147
AP3D1__NM_001261826_CCTTGTGGTTACGGATGCCG	AP3D1__NM_001261826	902	917	969	969
AP3D1__NM_001261826_CGGTGCCTTCGTGAAAGCTC	AP3D1__NM_001261826	53	64	88	121
AP3D1__NM_001261826_GACCACCAATCAGATCCGTA	AP3D1__NM_001261826	1090	558	1307	1056
AP3D1__NM_001261826_GCAGATTCTTGTCGAACATG	AP3D1__NM_001261826	430	635	450	644
AP3D1__NM_001261826_GCAGGACAACATAGCGGTGA	AP3D1__NM_001261826	58	52	59	31
AP3D1__NM_001261826_TGTTGGGATACGACATCAGC	AP3D1__NM_001261826	110	94	109	194
AP3M1__NM_012095_AAGTGTCAGCAACTCGATGT	AP3M1__NM_012095	466	441	490	399
AP3M1__NM_012095_ACAACAGAGCGTAGAATTGT	AP3M1__NM_012095	427	490	532	591
AP3M1__NM_012095_AGGAACTCAATTACAAAGAG	AP3M1__NM_012095	73	81	91	76
AP3M1__NM_012095_AGTTTATGAGAAATAGACTG	AP3M1__NM_012095	133	119	107	124
AP3M1__NM_012095_CTATTTCTCATAAACTGTTC	AP3M1__NM_012095	323	343	352	294
AP3M1__NM_012095_GGCAGCTGTCCAACATACCA	AP3M1__NM_012095	381	259	328	441
AP3M1__NM_012095_GTGTTCAGAGGCTGCAATTA	AP3M1__NM_012095	295	330	296	264
AP3M1__NM_012095_TACAAAGAGAGGTGGCACTT	AP3M1__NM_012095	129	82	135	135
AP3M1__NM_012095_TCTGTTGTCAACTCTATTAC	AP3M1__NM_012095	539	401	612	668
AP3M1__NM_012095_TGAGGTGTTGAAATGACAGG	AP3M1__NM_012095	266	176	281	300
AP3M1__NM_012095_TGTCCAACATACCATGGCGT	AP3M1__NM_012095	615	539	620	494
AP3M1__NM_012095_TTCTAGGCAGTAGTAATGTT	AP3M1__NM_012095	41	13	39	37
AP3M2__NM_001134296_ACAACCGTTCGAAGGATGGT	AP3M2__NM_001134296	366	232	306	262
AP3M2__NM_001134296_ACCGTTCGAAGGATGGTAGG	AP3M2__NM_001134296	315	343	381	449
AP3M2__NM_001134296_ACTACATTGTCTTTGATCAC	AP3M2__NM_001134296	836	571	951	1128
AP3M2__NM_001134296_AGTTGATCAAGAAAAGACTA	AP3M2__NM_001134296	125	116	86	165
AP3M2__NM_001134296_AGTTGTGGTTTATGAGGTAT	AP3M2__NM_001134296	146	279	217	230
AP3M2__NM_001134296_CAAGAAAAGACTATGGATCA	AP3M2__NM_001134296	331	487	431	499
AP3M2__NM_001134296_GTGGTGCCTTGGCGACGGAC	AP3M2__NM_001134296	195	141	118	135
AP3M2__NM_001134296_GTTGACAACCGTTCGAAGGA	AP3M2__NM_001134296	191	112	206	185
AP3M2__NM_001134296_TGGTGAGGGGTAGGGATAAC	AP3M2__NM_001134296	27	39	24	17
AP3M2__NM_001134296_TGGTGTTGACAACCGTTCGA	AP3M2__NM_001134296	39	49	25	14
AP3M2__NM_001134296_TTCTTTAAGAATGTTCGACT	AP3M2__NM_001134296	9	3	5	20
AP3M2__NM_001134296_TTGGAAGAGATGCTTGACAA	AP3M2__NM_001134296	178	263	182	152
AP3S1__NM_001284_AATTGATGCACAAAATAAGC	AP3S1__NM_001284	206	308	204	406
AP3S1__NM_001284_ATTCTTGCAGAAATGGTGAT	AP3S1__NM_001284	173	184	169	218
AP3S1__NM_001284_CATCAGGGAGACTTTCCATT	AP3S1__NM_001284	1254	1001	1107	891
AP3S1__NM_001284_GATTAGGATCGCCTTGATCA	AP3S1__NM_001284	550	216	483	358
AP3S1__NM_001284_TCACAATATTCTTGCAGAAA	AP3S1__NM_001284	154	220	228	280
AP3S2__NM_005829_AAACAGGCTGGAGAAATCCG	AP3S2__NM_005829	384	355	410	377
AP3S2__NM_005829_AGTAGAGGGTAGCATAGTGC	AP3S2__NM_005829	12	11	51	34
AP3S2__NM_005829_CAGACAAACTGGGACAGGTT	AP3S2__NM_005829	439	424	483	510
AP3S2__NM_005829_CATCCCGCTTGAGGACTAGA	AP3S2__NM_005829	615	694	741	860
AP3S2__NM_005829_CCACACAAAATACAAAGTAG	AP3S2__NM_005829	758	797	925	844
AP3S2__NM_005829_CTCAGATTGAGGCTCAAAAC	AP3S2__NM_005829	143	105	126	152
AP3S2__NM_005829_CTTTCCATCTAGTCCTCAAG	AP3S2__NM_005829	256	355	263	316
AP3S2__NM_005829_TCGCCAATGTTGATGTTCCG	AP3S2__NM_005829	1233	1643	1395	1630
AP3S2__NM_005829_TGGTAGAAGCGGACTAGCCG	AP3S2__NM_005829	24	32	31	51
AP3S2__NM_005829_TGTTCCGAGGAATCTCTGGC	AP3S2__NM_005829	177	337	184	138
AP3S2__NM_005829_TTTGATCTTCCATATGGATA	AP3S2__NM_005829	25	33	23	30
AP4B1__NM_001253852_AATGTCAAAACTGGACCAAT	AP4B1__NM_001253852	419	453	566	466
AP4B1__NM_001253852_ACCCCAATCCAATGGTGCGA	AP4B1__NM_001253852	1030	986	1140	736
AP4B1__NM_001253852_ACTCAAGGCTTGGACATGTC	AP4B1__NM_001253852	575	373	584	734
AP4B1__NM_001253852_AGCCTATTCTCAATGGTCTG	AP4B1__NM_001253852	562	737	685	677
AP4B1__NM_001253852_ATCCTCACATTCAAGCTGAT	AP4B1__NM_001253852	495	493	451	581
AP4B1__NM_001253852_ATTTGACATTCTCAATCTGT	AP4B1__NM_001253852	292	413	337	494
AP4B1__NM_001253852_CACCATCTCTTAAATCGGTT	AP4B1__NM_001253852	506	816	579	918
AP4B1__NM_001253852_GACCCCAATCCAATGGTGCG	AP4B1__NM_001253852	576	593	661	774
AP4B1__NM_001253852_GCCCTCGCACCATTGGATTG	AP4B1__NM_001253852	1221	1120	1165	1385
AP4B1__NM_001253852_GTCTTTGCACAGCGTATTGA	AP4B1__NM_001253852	344	414	461	342
AP4B1__NM_001253852_TGCTCCCCTGAAACCAGATC	AP4B1__NM_001253852	128	93	164	182
AP4B1__NM_001253852_TTGCTCACCATCTCTTAAAT	AP4B1__NM_001253852	255	287	310	218
AP4B1__NM_001253853_AAGGACATCAGTTTGTACGT	AP4B1__NM_001253853	790	802	769	527
AP4B1__NM_001253853_AGGACATCAGTTTGTACGTG	AP4B1__NM_001253853	660	657	825	675
AP4B1__NM_001253853_AGTAGCCCAGGTGTGGTGAT	AP4B1__NM_001253853	750	701	905	981
AP4B1__NM_001253853_CAGTAGCCCAGGTGTGGTGA	AP4B1__NM_001253853	399	370	407	317
AP4B1__NM_001253853_CTTTCTGTAGTTTGATGTAG	AP4B1__NM_001253853	359	729	382	535
AP4B1__NM_001253853_GTAGCTCCCATCACCACACC	AP4B1__NM_001253853	400	80	465	148
AP4B1__NM_001253853_TTCTGTAGTTTGATGTAGTG	AP4B1__NM_001253853	5	9	27	68
AP4E1__NM_001252127_AACAGCTGGAATCATTTCGC	AP4E1__NM_001252127	744	1095	1023	827
AP4E1__NM_001252127_AAGAGCTGAATTTGTAACCA	AP4E1__NM_001252127	399	427	554	456
AP4E1__NM_001252127_ACAGCTGGAATCATTTCGCA	AP4E1__NM_001252127	1137	1310	1025	1385
AP4E1__NM_001252127_AGGATCCTGTTGGATAACAT	AP4E1__NM_001252127	281	309	362	257
AP4E1__NM_001252127_ATCACAATGTCACATATGGT	AP4E1__NM_001252127	444	610	504	495
AP4E1__NM_001252127_CCTTGTGAATACAGTTGTAA	AP4E1__NM_001252127	287	329	308	304
AP4E1__NM_001252127_GATTGCATGAATATAGCCAA	AP4E1__NM_001252127	324	320	279	404
AP4E1__NM_001252127_GCAATCAAGTTAGCCCAACA	AP4E1__NM_001252127	11	5	3	0
AP4E1__NM_001252127_TAAATCGGAATTACTTGAGA	AP4E1__NM_001252127	132	100	212	108
AP4E1__NM_001252127_TCTAAGATATATATGCAAGG	AP4E1__NM_001252127	167	236	152	152
AP4E1__NM_001252127_TCTTACCTATGTTATCCAAC	AP4E1__NM_001252127	524	422	536	606
AP4E1__NM_001252127_TTCTCAAGTAATTCCGATTT	AP4E1__NM_001252127	10	6	12	42
AP4E1__NM_007347_GGGCTGTGATGCCGCGGACA	AP4E1__NM_007347	500	623	537	586
AP4M1__NM_004722_AATGAAGAATTGGGAAATCA	AP4M1__NM_004722	115	132	135	137
AP4M1__NM_004722_ACCCTTCTGGGCGATTACTG	AP4M1__NM_004722	428	623	474	579
AP4M1__NM_004722_AGCGACTGGACAGGACGGGG	AP4M1__NM_004722	1093	1022	1179	1213
AP4M1__NM_004722_CAGACACAGCGGCCTCTATT	AP4M1__NM_004722	1287	1367	1196	1208
AP4M1__NM_004722_CCTCACCAGAGCCGCTAGGA	AP4M1__NM_004722	482	333	669	492
AP4M1__NM_004722_CCTCACCTGGTCAGAGCGAC	AP4M1__NM_004722	272	677	324	441
AP4M1__NM_004722_CCTGGTCAGAGCGACTGGAC	AP4M1__NM_004722	351	467	339	351
AP4M1__NM_004722_CCTGTCCAGTCGCTCTGACC	AP4M1__NM_004722	968	1114	1057	825
AP4M1__NM_004722_CTCAAGAGCTTCCTTCCTAG	AP4M1__NM_004722	381	343	602	560
AP4M1__NM_004722_CTCTTTGACCTCAGCAGCGT	AP4M1__NM_004722	140	191	110	131
AP4M1__NM_004722_GTTTCACAGCTCTGTGAATC	AP4M1__NM_004722	132	107	134	124
AP4M1__NM_004722_TGGAGGACAGAATGAAGAAT	AP4M1__NM_004722	117	112	120	111
AP4S1__NM_001128126_CTTATCAAGAATTAGTAGAG	AP4S1__NM_001128126	145	117	172	143
AP4S1__NM_001128126_GCAGCTCTCTTCATTGTGGT	AP4S1__NM_001128126	1244	1734	1371	1982
AP4S1__NM_001128126_GCTGCATTGTGGAAACTAAC	AP4S1__NM_001128126	436	442	467	309
AP4S1__NM_001128126_TCCTCATGGTGAATAAACAA	AP4S1__NM_001128126	155	146	128	85
AP4S1__NM_001254727_AGGATACATCTAATTCACTC	AP4S1__NM_001254727	167	137	206	192
AP4S1__NM_001254727_AGTACTTGGCAAATGCACTC	AP4S1__NM_001254727	121	133	220	244
AP5B1__NM_138368_ACGGATAAGGTCTCCCCAAC	AP5B1__NM_138368	624	509	703	663
AP5B1__NM_138368_CCCAGGGACCACCCCCAGTT	AP5B1__NM_138368	1103	1030	1362	1224
AP5B1__NM_138368_CCTAGCAGCCGTACCAGCTG	AP5B1__NM_138368	106	116	163	289
AP5B1__NM_138368_GCGCAGGGTACTCCATGCTC	AP5B1__NM_138368	366	443	435	435
AP5B1__NM_138368_GGAGGACCAAGGTGTCCAAC	AP5B1__NM_138368	50	50	44	31
AP5B1__NM_138368_GGCCGCCTTGAGCGCAAGCA	AP5B1__NM_138368	322	617	329	398
AP5B1__NM_138368_GGCTGTGCTCTCGTGCTGTA	AP5B1__NM_138368	834	1117	989	1149
AP5B1__NM_138368_GGGGGGACTGCTCACGGATA	AP5B1__NM_138368	368	517	361	309
AP5B1__NM_138368_TCCCTGGGATTGGACACTAG	AP5B1__NM_138368	825	839	968	843
AP5B1__NM_138368_TCGCCCCAGATCGCTACCCG	AP5B1__NM_138368	1525	1298	1611	1324
AP5B1__NM_138368_TGGCCGCGGGTAGCGATCTG	AP5B1__NM_138368	228	302	243	289
AP5B1__NM_138368_TTCCCTGAGAACTGGCCGCT	AP5B1__NM_138368	196	204	134	213
AP5M1__NM_018229_AAAGTGGAGTTCCCGGTTCG	AP5M1__NM_018229	986	789	1088	1143
AP5M1__NM_018229_AACAGGGTATAGCAGATACA	AP5M1__NM_018229	470	574	666	806
AP5M1__NM_018229_ACCCTGTTTATCATATTGCA	AP5M1__NM_018229	1298	1440	1607	1915
AP5M1__NM_018229_ACTCCACTGACACTAATTAG	AP5M1__NM_018229	731	877	831	1001
AP5M1__NM_018229_AGTGTCAGTGGAGTTTCACA	AP5M1__NM_018229	1996	2716	2159	2035
AP5M1__NM_018229_ATCCATGCAATATGATAAAC	AP5M1__NM_018229	158	315	264	300
AP5M1__NM_018229_CCACTGACACTAATTAGTGG	AP5M1__NM_018229	558	468	618	545
AP5M1__NM_018229_CCGCCACTAATTAGTGTCAG	AP5M1__NM_018229	939	1252	892	1247
AP5M1__NM_018229_GACAGAGTTTGTTCAACTAG	AP5M1__NM_018229	228	185	231	240
AP5M1__NM_018229_GCAGAAGCAAGTCAGGCAAC	AP5M1__NM_018229	700	936	833	777
AP5M1__NM_018229_GCTCTTTGAACTTAGATTAT	AP5M1__NM_018229	55	80	80	59
AP5M1__NM_018229_TGGAAAACTGGGACGTACAA	AP5M1__NM_018229	13	1	5	1
AP5S1__NM_001204446_ACTTGCTCATCTGAGGATTG	AP5S1__NM_001204446	378	403	445	463
AP5S1__NM_001204446_AGGAGTACAGCACTCGGCAA	AP5S1__NM_001204446	503	419	659	586
AP5S1__NM_001204446_CCTTCCTCATTCACACCTTG	AP5S1__NM_001204446	1089	1606	1229	1628
AP5S1__NM_001204446_CTGCGGGCTGACCGCATTGA	AP5S1__NM_001204446	396	548	434	437
AP5S1__NM_001204446_CTTGAGGGCCCCGAATACTG	AP5S1__NM_001204446	316	298	387	360
AP5S1__NM_001204446_TCTCGGCACCATGCGGCCGT	AP5S1__NM_001204446	499	629	563	569
AP5Z1__NM_014855_ACTGGTCGTCTGTGAAGCGG	AP5Z1__NM_014855	577	685	613	683
AP5Z1__NM_014855_AGAAGAAGCCGCCGGAGTGT	AP5Z1__NM_014855	1343	1684	1621	1339
AP5Z1__NM_014855_AGTGGCCTCTCGGATGCAGC	AP5Z1__NM_014855	204	303	218	284
AP5Z1__NM_014855_CAGAGGGGGACATCTCTCGC	AP5Z1__NM_014855	1427	1492	1501	1251
AP5Z1__NM_014855_CCCCGTCCACCTCGGTGACG	AP5Z1__NM_014855	149	109	137	202
AP5Z1__NM_014855_CGGGAGGAGCGCCACAAACT	AP5Z1__NM_014855	308	259	385	253
AP5Z1__NM_014855_GACAACCTCCACCTGTTCAG	AP5Z1__NM_014855	1275	1246	1463	1573
AP5Z1__NM_014855_GCACCGCCGTCAAGCAGGGC	AP5Z1__NM_014855	448	465	377	520
AP5Z1__NM_014855_GCCCCGTCCACCTCGGTGAC	AP5Z1__NM_014855	639	523	645	707
AP5Z1__NM_014855_GGTTGGGGAAGCTCAATCTG	AP5Z1__NM_014855	385	388	366	332
AP5Z1__NM_014855_TACCTGGGCCAAGAGAACGG	AP5Z1__NM_014855	243	146	347	162
AP5Z1__NM_014855_TGCTGAGGTGCCCGCTGAAC	AP5Z1__NM_014855	651	537	876	703
APAF1__NM_001160_AGCATTGTAGAATGATACGT	APAF1__NM_001160	356	368	393	306
APAF1__NM_001160_AGTGTTACAGATTCAGTAAT	APAF1__NM_001160	420	192	370	216
APAF1__NM_001160_ATGGATCACATGATTAGTGA	APAF1__NM_001160	97	43	100	91
APAF1__NM_001160_CCAGGATGGGTCACCATACA	APAF1__NM_001160	281	507	438	467
APAF1__NM_001160_GAGTGTTACAGATTCAGTAA	APAF1__NM_001160	295	226	266	317
APAF1__NM_001160_GCAGAATCTTTGCACACGGT	APAF1__NM_001160	815	1037	999	1025
APAF1__NM_001160_GCTGCTCTTTGCTGTTGAGT	APAF1__NM_001160	528	427	683	628
APAF1__NM_001160_GTGAAGGTGGAGTACCACAG	APAF1__NM_001160	125	126	114	114
APAF1__NM_001160_GTTGGGAAACAAGACAAATC	APAF1__NM_001160	60	33	43	35
APAF1__NM_001160_TCCTGGTTCACCTTTCAATT	APAF1__NM_001160	248	150	304	316
APAF1__NM_001160_TGATGCTTCGCAAACACCCA	APAF1__NM_001160	26	86	47	48
APAF1__NM_001160_TTGGGAAACAAGACAAATCT	APAF1__NM_001160	131	112	166	112
APAF1__NM_181861_ACTAACTGAATCTTTACCAC	APAF1__NM_181861	640	584	758	520
APAF1__NM_181861_AGTGGTAAAGATTCAGTTAG	APAF1__NM_181861	115	127	145	118
APAF1__NM_181861_GTTAGTGGAATAACTTCGTA	APAF1__NM_181861	495	585	549	570
APBA1__NM_001163_AACCACACGGACACCGCCGA	APBA1__NM_001163	907	819	878	688
APBA1__NM_001163_AGCACGTGTACGAGGAGATA	APBA1__NM_001163	410	557	431	511
APBA1__NM_001163_AGGCCGCCGTAGTCGGCATA	APBA1__NM_001163	17	5	3	1
APBA1__NM_001163_AGGGCTCGTCCTCACCGCGG	APBA1__NM_001163	483	312	596	484
APBA1__NM_001163_AGGTGGGGAGGTGAACGAGT	APBA1__NM_001163	305	331	288	418
APBA1__NM_001163_CCTCCAGGAGCACGTGTACG	APBA1__NM_001163	979	887	908	1141
APBA1__NM_001163_CTCCGCATCGTAGCCGTCGC	APBA1__NM_001163	741	991	877	940
APBA1__NM_001163_CTCCTGGAGGCCGCCGTAGT	APBA1__NM_001163	794	731	815	794
APBA1__NM_001163_GCCATGAATGCGGCCTACTC	APBA1__NM_001163	400	373	613	457
APBA1__NM_001163_GCCGTAGTCGGCATAGGGCT	APBA1__NM_001163	50	15	39	67
APBA1__NM_001163_GGCCCGCGACGGCTACGATG	APBA1__NM_001163	574	474	664	636
APBA1__NM_001163_TCCGAGCCCTATGCCGACTA	APBA1__NM_001163	734	787	883	956
APBA2__NM_001130414_ACCGGAAGCTTGAGAGCGTG	APBA2__NM_001130414	877	841	961	1090
APBA2__NM_001130414_CACCGGAAGCTTGAGAGCGT	APBA2__NM_001130414	806	741	773	599
APBA2__NM_001130414_CAGGACCTGGTCTCACCCTA	APBA2__NM_001130414	2559	2836	2953	3151
APBA2__NM_001130414_CCACCGGAAGCTTGAGAGCG	APBA2__NM_001130414	880	1209	918	1518
APBA2__NM_001130414_CTCGGGCACATAGCCCTCCA	APBA2__NM_001130414	39	50	69	149
APBA2__NM_001130414_CTTGGAGGGCTATGTGCCCG	APBA2__NM_001130414	123	181	111	106
APBA2__NM_001130414_GAGCCCCGCGCCAGAGGAAC	APBA2__NM_001130414	417	454	523	523
APBA2__NM_001130414_GAGCGGCATGTTGGACCATA	APBA2__NM_001130414	387	537	352	345
APBA2__NM_001130414_GAGGACGACAGCTACCTAGA	APBA2__NM_001130414	194	144	239	265
APBA2__NM_001130414_GCCACAACCACAGCCCCGAT	APBA2__NM_001130414	1545	897	1739	857
APBA2__NM_001130414_GGTGGAGGAGTGGACGGACT	APBA2__NM_001130414	488	386	514	515
APBA2__NM_001130414_TCGGGCACATAGCCCTCCAA	APBA2__NM_001130414	7	0	13	4
APBA2__NM_005503_ACCCGGCTGCATACCCGGGC	APBA2__NM_005503	216	206	143	260
APBA2__NM_005503_AGCTGTTTGGGACCATTGTG	APBA2__NM_005503	757	850	693	908
APBA2__NM_005503_AGGGTGCCCAGAAGAGACGA	APBA2__NM_005503	105	165	115	130
APBA2__NM_005503_CACACATGCCACTACTGCTC	APBA2__NM_005503	122	150	161	128
APBA2__NM_005503_CAGCTGTTTGGGACCATTGT	APBA2__NM_005503	888	1070	959	1248
APBA2__NM_005503_CCCCTTGGCTGGGACGTCTG	APBA2__NM_005503	1089	1494	1240	1605
APBA2__NM_005503_CCCGGCTGCATACCCGGGCA	APBA2__NM_005503	11	5	13	1
APBA2__NM_005503_CCCTGCCCGGGTATGCAGCC	APBA2__NM_005503	678	965	726	792
APBA2__NM_005503_CGACTTGTAATGAAAGTTTG	APBA2__NM_005503	565	694	646	817
APBA2__NM_005503_CGCAGCCAGGACACCGGGCA	APBA2__NM_005503	753	1264	952	1322
APBA2__NM_005503_CTAAAGCTTTAGTTACTGCG	APBA2__NM_005503	385	344	342	482
APBA2__NM_005503_GAGTGGGTTTCGTTTCCTCG	APBA2__NM_005503	756	589	841	879
APBA2__NM_005503_GGAGAAGACAGTCCAGAACG	APBA2__NM_005503	648	765	733	830
APBA2__NM_005503_GGATCGCTAATGAACCGAGC	APBA2__NM_005503	274	307	370	244
APBA2__NM_005503_GGGAGCCAGCGTCCGTGGCT	APBA2__NM_005503	979	1061	955	990
APBA2__NM_005503_GGGTGCCCAGAAGAGACGAC	APBA2__NM_005503	328	536	383	350
APBA2__NM_005503_TGTTGCATGCAGCTACCCGT	APBA2__NM_005503	634	636	709	436
APBA3__NM_004886_AGCTCTGCCAGGCGATCTGG	APBA3__NM_004886	252	242	291	382
APBA3__NM_004886_AGGGGGGCTGCAACAGTCGGG	APBA3__NM_004886	364	186	499	218
APBA3__NM_004886_ATGGCCTGTGGCAATGTGTA	APBA3__NM_004886	247	364	322	324
APBA3__NM_004886_CAATTTCCCGATCCCCTTCG	APBA3__NM_004886	1506	1448	1638	1735
APBA3__NM_004886_CAGGTCATCCCGGCCAGCTT	APBA3__NM_004886	727	682	857	742
APBA3__NM_004886_CGAAGGGGATCGGGAAATTG	APBA3__NM_004886	668	673	875	602
APBA3__NM_004886_CTCCTGGCATAGGGTCCCAC	APBA3__NM_004886	589	563	603	593
APBA3__NM_004886_GAAGGGGATCGGGAAATTGT	APBA3__NM_004886	300	154	300	200
APBA3__NM_004886_GGCTCTTCAGGTGTGACTAG	APBA3__NM_004886	113	63	146	143
APBA3__NM_004886_TCAGGGGGCTGCAACAGTCG	APBA3__NM_004886	411	415	369	490
APBA3__NM_004886_TGTCCCCTACACATTGCCAC	APBA3__NM_004886	369	477	485	403
APBA3__NM_004886_TGTGGCAATGTGTAGGGGAC	APBA3__NM_004886	364	388	398	342
APBB1__NM_001164_AATGCCAAGTGGCTAAAAGA	APBB1__NM_001164	65	48	83	107
APBB1__NM_001164_AGGAAGTGATAACACTGGAG	APBB1__NM_001164	205	219	171	254
APBB1__NM_001164_AGGCAGGGAACCGTAGCTAC	APBB1__NM_001164	298	257	262	257
APBB1__NM_001164_AGGTCCTCGTAGGCCTCGGT	APBB1__NM_001164	384	346	579	594
APBB1__NM_001164_ATCATCAGCACTCAAGAGCA	APBB1__NM_001164	280	217	326	266
APBB1__NM_001164_ATGCCCCTCCCCAGTAGCTA	APBB1__NM_001164	488	426	463	474
APBB1__NM_001164_ATTCTGGTCACGGTGGGCCG	APBB1__NM_001164	452	519	505	683
APBB1__NM_001164_CATTGCGATTCTGGTCACGG	APBB1__NM_001164	908	1006	1029	1596
APBB1__NM_001164_CTTCAAGGAAGTGATAACAC	APBB1__NM_001164	249	266	277	332
APBB1__NM_001164_GATACACCTGTACTCTGAGC	APBB1__NM_001164	901	1057	915	968
APBB1__NM_001164_GCAGGGAACCGTAGCTACTG	APBB1__NM_001164	405	489	479	275
APBB1__NM_001164_GCTCACAACGCAGCCAACCG	APBB1__NM_001164	604	591	719	746
APBB1__NM_001164_GCTCAGAGTACAGGTGTATC	APBB1__NM_001164	488	538	583	687
APBB1__NM_001164_GGAGAGATCTAATCCCTTCA	APBB1__NM_001164	1152	1491	1405	1712
APBB1__NM_001164_GGCAGGGAACCGTAGCTACT	APBB1__NM_001164	486	649	519	542
APBB1__NM_001164_GGGGCCATCTGTAAGGGCTT	APBB1__NM_001164	362	377	370	515
APBB1__NM_001164_TCAAGGTCACATTGCGATTC	APBB1__NM_001164	75	264	104	143
APBB1__NM_001164_TCACATTGCGATTCTGGTCA	APBB1__NM_001164	284	438	385	446
APBB1__NM_001164_TGGGGAGGGGCATATTTGGG	APBB1__NM_001164	180	266	233	208
APBB1__NM_001164_TGTTGGCATTAATGGCCGAC	APBB1__NM_001164	227	175	203	233
APBB1__NM_001257319_AATCCGTCTCGAAGGCGTTG	APBB1__NM_001257319	680	467	635	706
APBB1__NM_001257319_ACACCTCAGGGACCTATTAC	APBB1__NM_001257319	1313	1719	1284	1607
APBB1__NM_001257319_CAGGTCGGAATCCGTCTCGA	APBB1__NM_001257319	1226	1141	1323	1074
APBB1__NM_001257319_CCCCACGGAATACCAACCCA	APBB1__NM_001257319	1183	1381	1501	1908
APBB1__NM_001257319_CCCTGGGTTGGTATTCCGTG	APBB1__NM_001257319	3548	3749	3976	3936
APBB1__NM_001257319_CCTGGGTTGGTATTCCGTGG	APBB1__NM_001257319	1416	1350	1544	1337
APBB1__NM_001257319_CTGCATGACCCCATGTCTGG	APBB1__NM_001257319	490	639	587	617
APBB1__NM_001257319_GGCCCCTGGACGCAGCAGTG	APBB1__NM_001257319	106	136	111	95
APBB1__NM_001257319_GGTCATCTCTACCCAGCCTA	APBB1__NM_001257319	70	59	126	102
APBB1__NM_001257319_GTGCCAGTAATAGGTCCCTG	APBB1__NM_001257319	496	604	549	731
APBB1__NM_001257319_TCCATTGGGGCCTCATCACT	APBB1__NM_001257319	868	1136	1155	1519
APBB1__NM_001257319_TGTTGGGATGTGCCAGTAAT	APBB1__NM_001257319	297	413	404	490
APBB1__NM_001257320_CAGCTTATCACGAGCTACGT	APBB1__NM_001257320	249	288	304	268
APBB1__NM_001257320_TCCCCCCAGCCCCCAGACAT	APBB1__NM_001257320	900	873	1137	894
APBB1__NM_001257325_GGGTGTATGACACAGATGCG	APBB1__NM_001257325	217	198	203	265
APBB1IP__NM_019043_AAAACAAAGCAATAGTCAGT	APBB1IP__NM_019043	289	247	386	477
APBB1IP__NM_019043_ACCAGTGGGTCATGGGAATA	APBB1IP__NM_019043	467	537	701	680
APBB1IP__NM_019043_ATCTACCCGGAACTACAAAT	APBB1IP__NM_019043	442	398	487	471
APBB1IP__NM_019043_CACAAGAACCCTTAACCAGT	APBB1IP__NM_019043	431	331	388	432
APBB1IP__NM_019043_GCTCTTTATTTGAAAGAAGA	APBB1IP__NM_019043	115	139	107	85
APBB1IP__NM_019043_GGAATTTATTATGTACCCAA	APBB1IP__NM_019043	87	22	99	79
APBB1IP__NM_019043_GGCAGTGGAAGGTCTAACAC	APBB1IP__NM_019043	608	804	740	644
APBB1IP__NM_019043_GTGGTAAGTCATCCTCATAT	APBB1IP__NM_019043	137	81	87	81
APBB1IP__NM_019043_TAAAACAAAGCAATAGTCAG	APBB1IP__NM_019043	125	103	106	172
APBB1IP__NM_019043_TCTGGTAGCAGACATAAGTG	APBB1IP__NM_019043	74	110	71	77
APBB1IP__NM_019043_TGGAAGGTCTAACACAGGAT	APBB1IP__NM_019043	61	51	63	77
APBB1IP__NM_019043_TTCCCATGACCCACTGGTTA	APBB1IP__NM_019043	239	160	247	356
APBB2__NM_001166050_AGAACCGAGGCAATCACCAT	APBB2__NM_001166050	1320	1284	1381	1382
APBB2__NM_001166050_AGAAGGATCATCCGAAAACA	APBB2__NM_001166050	240	270	207	239
APBB2__NM_001166050_AGGCAATCACCATGGGACTG	APBB2__NM_001166050	656	923	701	887
APBB2__NM_001166050_ATATGCACTAACTAACATCC	APBB2__NM_001166050	660	673	803	884
APBB2__NM_001166050_CAGTCCCATGGTGATTGCCT	APBB2__NM_001166050	1458	1604	1504	2197
APBB2__NM_001166050_CCCAGCTCCCCGGATGAAAC	APBB2__NM_001166050	739	753	745	1024
APBB2__NM_001166050_GAACCTGGCACCGAGCGATG	APBB2__NM_001166050	471	400	462	595
APBB2__NM_001166050_GAACTACAGACGTCACAAAT	APBB2__NM_001166050	574	663	753	793
APBB2__NM_001166050_GATCGGAGGTTAAGGGTGTT	APBB2__NM_001166050	467	684	439	722
APBB2__NM_001166050_GTGGGAGGATCGGAGGTTAA	APBB2__NM_001166050	64	61	83	60
APBB2__NM_001166050_TGACACCTTGGCAGTGTTTA	APBB2__NM_001166050	178	322	298	424
APBB2__NM_001166050_TGTGGGAGGATCGGAGGTTA	APBB2__NM_001166050	433	429	492	451
APBB2__NM_001166051_AACCTCGATGTCCCTTTGCA	APBB2__NM_001166051	1211	1425	1333	1455
APBB2__NM_001166051_ACATGCCCAAGTACTGCACG	APBB2__NM_001166051	1450	1127	1726	1942
APBB2__NM_001166051_ACCAGCTCAGTCTTTGGTGT	APBB2__NM_001166051	930	1529	1031	1479
APBB2__NM_001166051_CACAACCAATGTAAAACGAG	APBB2__NM_001166051	242	248	301	437
APBB2__NM_001166051_GAAGTTCCACGTGCAGTACT	APBB2__NM_001166051	786	703	715	995
APBB2__NM_001166051_GAATGAAGAGGAAGTCTTAG	APBB2__NM_001166051	201	230	283	309
APBB2__NM_001166051_GTCACAACCAATGTAAAACG	APBB2__NM_001166051	172	318	270	276
APBB2__NM_001166051_GTCCTTCCCAACACCCATGA	APBB2__NM_001166051	923	1016	1014	1038
APBB2__NM_001166051_GTTCAAAATATCCATTCCTG	APBB2__NM_001166051	85	137	110	114
APBB2__NM_001166051_TAGGCTCGCACCAGAAAACG	APBB2__NM_001166051	240	272	204	403
APBB2__NM_001166051_TCACAACCAATGTAAAACGA	APBB2__NM_001166051	336	525	457	430
APBB2__NM_001166051_TTTGCCTTCATCATGGACAC	APBB2__NM_001166051	269	420	274	310
APBB3__NM_006051_AAAAGCGAAGTCCCTAGCAG	APBB3__NM_006051	626	562	675	596
APBB3__NM_006051_ACCTCACCCCAGGCACCATC	APBB3__NM_006051	1524	1540	1469	1472
APBB3__NM_006051_ACTCTCTGTCCTGGTATGGT	APBB3__NM_006051	775	767	785	782
APBB3__NM_006051_CAAAAGCGAAGTCCCTAGCA	APBB3__NM_006051	619	591	715	597
APBB3__NM_006051_CAGGGGATTCACTAGGCTCA	APBB3__NM_006051	259	224	313	319
APBB3__NM_006051_CATCTCCTAGTTCCCAGGTT	APBB3__NM_006051	944	900	1365	905
APBB3__NM_006051_CTCTCTGTCCTGGTATGGTG	APBB3__NM_006051	140	356	162	209
APBB3__NM_006051_CTCTGGTGCACATCCGTGTG	APBB3__NM_006051	254	279	312	281
APBB3__NM_006051_GCTGGGCAAGGATTACATGC	APBB3__NM_006051	452	380	523	459
APBB3__NM_006051_GTAGGATTCCCCACCATACC	APBB3__NM_006051	132	141	183	220
APBB3__NM_006051_GTCCAGTGAACTCTCCAGGC	APBB3__NM_006051	704	715	873	763
APBB3__NM_006051_TCTGGTGCACATCCGTGTGT	APBB3__NM_006051	720	791	734	701
APBB3__NM_133174_ACAAAGCCCATGTAGGGCAC	APBB3__NM_133174	118	81	155	129
APBB3__NM_133174_CCATTGCCAGTGCCCTACAT	APBB3__NM_133174	1306	1388	1416	1359
APC__NM_000038_AAAATGTCCCTCCGTTCTTA	APC__NM_000038	279	368	338	361
APC__NM_000038_AACTGAGGCATCTAATATGA	APC__NM_000038	614	588	699	695
APC__NM_000038_ATTTCCCTGGAGTAAAACTG	APC__NM_000038	86	321	60	42
APC__NM_000038_CCCGGCTTCCATAAGAACGG	APC__NM_000038	613	825	718	542
APC__NM_000038_CCTCCGTTCTTATGGAAGCC	APC__NM_000038	426	712	482	638
APC__NM_000038_CTTCCCGGCTTCCATAAGAA	APC__NM_000038	784	723	867	645
APC__NM_000038_CTTCTTGGAAATGAACCCAT	APC__NM_000038	311	405	389	362
APC__NM_000038_GATTTATTAGAGCGTCTTAA	APC__NM_000038	59	181	92	84
APC__NM_000038_GGAAATGAACCCATAGGAAC	APC__NM_000038	513	496	543	464
APC__NM_000038_GGATCTGTATCAAGCCGTTC	APC__NM_000038	612	840	612	715
APC__NM_000038_TACAAACAGATATGACCAGA	APC__NM_000038	268	144	344	211
APC__NM_000038_TCTGGTCATATCTGTTTGTA	APC__NM_000038	430	417	504	351
APC__NM_001127511_AACTGAGGGTGGTACAGAAG	APC__NM_001127511	111	115	129	79
APC__NM_001127511_AGCCCAGGGAGGCGTACATA	APC__NM_001127511	356	365	394	460
APC__NM_001127511_CCGGCAGGAGACGAAGAGCC	APC__NM_001127511	549	842	638	640
APC__NM_001127511_GAAGCGGGCAAAGGGGCGAC	APC__NM_001127511	539	550	542	590
APC__NM_001127511_GAGCCCAGGGAGGCGTACAT	APC__NM_001127511	290	469	339	325
APC__NM_001127511_GCCCAGGGAGGCGTACATAG	APC__NM_001127511	373	381	392	372
APC2__NM_005883_CATGTTGGCGACGCGCGACC	APC2__NM_005883	1508	1314	1868	1527
APC2__NM_005883_CCAGCACATCCGCTCGCTGA	APC2__NM_005883	976	767	1162	940
APC2__NM_005883_CCAGCAGTTCTCGATGCAGA	APC2__NM_005883	435	490	523	554
APC2__NM_005883_CCATCTGCATCGAGAACTGC	APC2__NM_005883	520	808	577	759
APC2__NM_005883_CCCGAGGGCAGCCCAGTACA	APC2__NM_005883	520	535	637	633
APC2__NM_005883_CTGGTCGCGCGTCGCCAACA	APC2__NM_005883	172	105	204	286
APC2__NM_005883_CTTCGGCACCTCGGACGAGA	APC2__NM_005883	423	374	610	363
APC2__NM_005883_GACAGAGACGTCGGGCATGA	APC2__NM_005883	652	556	800	747
APC2__NM_005883_GAGGTCCTGAAGCACCTACA	APC2__NM_005883	244	259	250	318
APC2__NM_005883_GCTCGTCCAGGCGCTTGGAC	APC2__NM_005883	314	277	364	485
APC2__NM_005883_GGAGGAGCGCTTCGGCACCT	APC2__NM_005883	2441	1846	2590	2180
APC2__NM_005883_TGCCTCACCAGCTGCTCGTA	APC2__NM_005883	181	221	164	161
APCDD1__NM_153000_ACCCAGTGGCCCTCGATTGT	APCDD1__NM_153000	1808	2210	1898	2031
APCDD1__NM_153000_AGGCCTTGAAGGTGTTATTG	APCDD1__NM_153000	558	698	499	702
APCDD1__NM_153000_CACCTACAATCGAGGGCCAC	APCDD1__NM_153000	402	380	461	564
APCDD1__NM_153000_CAGATGCCACCTACAATCGA	APCDD1__NM_153000	303	472	380	521
APCDD1__NM_153000_CAGTGGCCCTCGATTGTAGG	APCDD1__NM_153000	863	997	981	1247
APCDD1__NM_153000_CCCAGCTGTGAAGTAAGGTC	APCDD1__NM_153000	502	405	414	612
APCDD1__NM_153000_CCGGATGATGAGAGTATAAG	APCDD1__NM_153000	448	729	469	589
APCDD1__NM_153000_CGGATGATGAGAGTATAAGT	APCDD1__NM_153000	515	719	481	581
APCDD1__NM_153000_CTGTGATCCTTGCACCATTG	APCDD1__NM_153000	643	599	782	708
APCDD1__NM_153000_GCAGATGCCACCTACAATCG	APCDD1__NM_153000	513	676	606	681
APCDD1__NM_153000_GCTGCCATAATAAAATTGGT	APCDD1__NM_153000	1759	1540	1926	2020
APCDD1__NM_153000_GTTATTGTGGTAGAATCTGT	APCDD1__NM_153000	262	283	316	340
APCDD1L__NM_153360_ACCTACCTCCCAAAAGCACC	APCDD1L__NM_153360	584	811	797	882
APCDD1L__NM_153360_ACTACGAGGACCCCTTCTGC	APCDD1L__NM_153360	1742	870	1969	1346
APCDD1L__NM_153360_ACTGGTGGGCTCGAAAGAGC	APCDD1L__NM_153360	68	13	83	70
APCDD1L__NM_153360_AGCACCAGGACGCAAGCGTA	APCDD1L__NM_153360	261	191	232	152
APCDD1L__NM_153360_AGCCCACCAGTTCTACTACG	APCDD1L__NM_153360	1069	1164	1000	1065
APCDD1L__NM_153360_AGGGGTCCTCGTAGTAGAAC	APCDD1L__NM_153360	52	24	63	60
APCDD1L__NM_153360_CAAGGTCCATTAAGGCGTGG	APCDD1L__NM_153360	426	241	263	292
APCDD1L__NM_153360_CTGGGCACTCTATCTGGCAA	APCDD1L__NM_153360	344	273	350	431
APCDD1L__NM_153360_GCACCAGGACGCAAGCGTAG	APCDD1L__NM_153360	250	311	231	237
APCDD1L__NM_153360_GCAGTGCTGGGCACTCTATC	APCDD1L__NM_153360	429	338	417	420
APCDD1L__NM_153360_GCTGGGCACTCTATCTGGCA	APCDD1L__NM_153360	981	1166	1197	1518
APCDD1L__NM_153360_TGGAGGCAGGATCGCAGTGC	APCDD1L__NM_153360	43	20	69	30
APCS__NM_001639_AAATTCAGCAATACCTGATG	APCS__NM_001639	550	386	560	478
APCS__NM_001639_ACTATAGGCTCGAAAACACA	APCS__NM_001639	77	125	69	94
APCS__NM_001639_AGGGAGAGGGGTACCCTGAT	APCS__NM_001639	269	190	216	276
APCS__NM_001639_CACAAGGTAAAGTTCTGTAG	APCS__NM_001639	14	0	17	10
APCS__NM_001639_CAGTGCACATCTGTGTGAGC	APCS__NM_001639	475	685	566	868
APCS__NM_001639_CTCTTCTCCTACAATACCCA	APCS__NM_001639	142	90	227	195
APCS__NM_001639_GCCAGTCCAGGATATTGGCA	APCS__NM_001639	764	539	934	616
APCS__NM_001639_GGAGAGTATAGTCTATACAT	APCS__NM_001639	592	629	718	448
APCS__NM_001639_GGCACGAGAGAGATCACTAT	APCS__NM_001639	1400	1380	1464	1303
APCS__NM_001639_GTGAGCTGGGAGTCCTCATC	APCS__NM_001639	726	547	832	648
APCS__NM_001639_TGCCAGTCCAGGATATTGGC	APCS__NM_001639	322	308	326	195
APCS__NM_001639_TGTAAACTTGATCACACCGC	APCS__NM_001639	417	343	426	458
APEH__NM_001640_AAGCGGATGCCCAACCGGAA	APEH__NM_001640	346	137	303	258
APEH__NM_001640_AGTCCCCGTCATGGAACACC	APEH__NM_001640	312	268	364	325
APEH__NM_001640_ATCGCTGGCACTGACGTCCA	APEH__NM_001640	296	296	355	254
APEH__NM_001640_CAGGTCAGCCCTGTATTATG	APEH__NM_001640	591	674	736	520
APEH__NM_001640_CAGTGTGCACCGTCCGGTAT	APEH__NM_001640	1843	858	2063	1684
APEH__NM_001640_CCAGCGATGATGAGATAGCC	APEH__NM_001640	134	302	156	232
APEH__NM_001640_CGATGAGGTCCACATAATAC	APEH__NM_001640	396	236	445	483
APEH__NM_001640_GAGGCGGCGGCTCTGTATCG	APEH__NM_001640	801	567	830	705
APEH__NM_001640_GAGGTCACCACGCAGTACGG	APEH__NM_001640	323	344	437	337
APEH__NM_001640_GGCAACATCTCTGTGCTTGA	APEH__NM_001640	2857	3659	3305	4119
APEH__NM_001640_GTACGGCGGCCAATACCGGA	APEH__NM_001640	457	460	540	568
APEH__NM_001640_TGCGTGCTGGATGTCGAGAG	APEH__NM_001640	646	323	617	545
APEX1__NM_001244249_ACAGCATATGTACCTAATGC	APEX1__NM_001244249	355	306	385	307
APEX1__NM_001244249_AGGCCGAGGTCTGGTACGAC	APEX1__NM_001244249	649	834	685	826
APEX1__NM_001244249_CCGAAGCCTTGGCGCTCTTG	APEX1__NM_001244249	1099	998	1206	1006
APEX1__NM_001244249_CTCAAAGTTTCTTACGGCAT	APEX1__NM_001244249	718	952	832	938
APEX1__NM_001244249_CTGTACCCTTCCTTGTCCGA	APEX1__NM_001244249	998	634	1137	936
APEX1__NM_001244249_CTTGAAGGCACAGTATATCT	APEX1__NM_001244249	612	413	508	331
APEX1__NM_001244249_GGTGTAGGCATAGGGTGTGT	APEX1__NM_001244249	730	544	719	661
APEX1__NM_001244249_GTGTAGGCATAGGGTGTGTT	APEX1__NM_001244249	240	278	261	321
APEX1__NM_001244249_TAATCCTTTCTTCTTAATCC	APEX1__NM_001244249	99	53	115	174
APEX1__NM_001244249_TATGTACCTAATGCAGGCCG	APEX1__NM_001244249	443	272	464	403
APEX1__NM_001244249_TCTGCTCTTGGAATGTGGAT	APEX1__NM_001244249	301	209	319	298
APEX1__NM_001244249_TGAATTTGACTCGTTTGTGC	APEX1__NM_001244249	221	610	298	441
APEX2__NM_001271748_AACACAGGACTTTGTTCGAG	APEX2__NM_001271748	1290	1577	1413	1598
APEX2__NM_001271748_AGGCCCCCTCCTGCTTTGGT	APEX2__NM_001271748	1471	1447	1716	1428
APEX2__NM_001271748_CAGCGGTAGCTATCGATGAA	APEX2__NM_001271748	93	60	123	161
APEX2__NM_001271748_CCAGTGGTCAATGGGGCGGT	APEX2__NM_001271748	824	744	888	895
APEX2__NM_001271748_CTGAAAGGTGTCTATGACCA	APEX2__NM_001271748	387	250	352	322
APEX2__NM_001271748_GCAGCACAACAATCAAACCC	APEX2__NM_001271748	739	631	722	629
APEX2__NM_001271748_GCAGCGGTAGCTATCGATGA	APEX2__NM_001271748	491	263	556	621
APEX2__NM_001271748_GGAGCCATAGTTGAGATGGC	APEX2__NM_001271748	170	246	159	157
APEX2__NM_001271748_GGCGCCCGCCATCTCAACTA	APEX2__NM_001271748	721	937	1003	970
APEX2__NM_001271748_TGCAGCACAACAATCAAACC	APEX2__NM_001271748	248	252	293	251
APEX2__NM_001271748_TGGGGCGGTGGGCTGTATTC	APEX2__NM_001271748	421	317	328	230
APEX2__NM_001271748_TTTCAGCCATGTGATCATTC	APEX2__NM_001271748	329	304	258	239
APEX2__NM_014481_AGCTTCAGCCGCAACCGTAG	APEX2__NM_014481	182	162	163	155
APEX2__NM_014481_ATAACCCTCAACGATAGCCA	APEX2__NM_014481	237	280	367	208
APEX2__NM_014481_ATTTACCAGAATAGCCGCTA	APEX2__NM_014481	167	97	104	128
APEX2__NM_014481_CAGAATAGCCGCTACGGTTG	APEX2__NM_014481	291	342	364	257
APEX2__NM_014481_CATGGATGAGTTTACCCAAG	APEX2__NM_014481	780	678	923	747
APEX2__NM_014481_CGCAACCGTAGCGGCTATTC	APEX2__NM_014481	1009	1262	1003	1259
APEX2__NM_014481_GAGCCCCTGGCTATCGTTGA	APEX2__NM_014481	326	356	403	393
APEX2__NM_014481_GGCTCTGGATAGTGAGGGCA	APEX2__NM_014481	305	288	369	616
APEX2__NM_014481_GGGCTCTGGATAGTGAGGGC	APEX2__NM_014481	160	79	239	232
APEX2__NM_014481_GTGGTGAGCTGGAACATCAA	APEX2__NM_014481	24	27	42	14
APEX2__NM_014481_TATAACCCTCAACGATAGCC	APEX2__NM_014481	92	107	137	175
APEX2__NM_014481_TGGTGAGCTGGAACATCAAT	APEX2__NM_014481	55	65	89	66
APH1A__NM_001077628_ACCAGGATGATAACGCGAAG	APH1A__NM_001077628	220	229	211	254
APH1A__NM_001077628_ACCTGAAGTCAGGAAGTAAT	APH1A__NM_001077628	592	522	620	514
APH1A__NM_001077628_AGACAGCAGCACCAAAAATC	APH1A__NM_001077628	384	184	366	323
APH1A__NM_001077628_AGTGATCAAGAAAAGCGCGA	APH1A__NM_001077628	390	356	401	402
APH1A__NM_001077628_CAGCAGCACCAAAAATCAGG	APH1A__NM_001077628	378	223	346	164
APH1A__NM_001077628_CCTGAAGTCAGGAAGTAATA	APH1A__NM_001077628	196	118	231	175
APH1A__NM_001077628_CGCGTTATCATCCTGGTCGC	APH1A__NM_001077628	1117	1159	1491	1203
APH1A__NM_001077628_GACACCACTGATGATACCGA	APH1A__NM_001077628	549	405	622	390
APH1A__NM_001077628_GAGCCGGGCATCTGACCGGT	APH1A__NM_001077628	429	140	348	369
APH1A__NM_001077628_GCCATCTGGCGGATGGAGAT	APH1A__NM_001077628	888	1371	857	1142
APH1A__NM_001077628_GGCTGCACTTTCGTCGCGTT	APH1A__NM_001077628	1341	1683	1584	2070
APH1A__NM_001077628_TCTTGGTCCATGTGACCGAC	APH1A__NM_001077628	185	200	114	92
APH1A__NM_001243771_AGCAGGCTGGCCTCATACCA	APH1A__NM_001243771	1035	750	1074	778
APH1A__NM_001243771_CCAGGGGTTCAGGAATGTCT	APH1A__NM_001243771	399	110	424	363
APH1A__NM_001243771_CCTGTGAGAGGAGACGGTAC	APH1A__NM_001243771	443	368	437	418
APH1A__NM_001243771_CTGTGAGAGGAGACGGTACT	APH1A__NM_001243771	458	506	441	647
APH1A__NM_001243772_GAAACAGTGACTGCATAGAT	APH1A__NM_001243772	176	108	242	188
APH1B__NM_001145646_AGCACCCTCTATGCGACTGC	APH1B__NM_001145646	661	666	893	987
APH1B__NM_001145646_ATAGGCCAGCAGTCGCATAG	APH1B__NM_001145646	127	90	109	142
APH1B__NM_001145646_ATCTTCCTCATCGCCGGGTG	APH1B__NM_001145646	388	358	482	575
APH1B__NM_001145646_ATGAGGAAGATGATACGCAA	APH1B__NM_001145646	184	164	202	184
APH1B__NM_001145646_CTCTTGCCATGAACCAAACA	APH1B__NM_001145646	355	497	448	475
APH1B__NM_001145646_GGATTATAAATGCTGACGCC	APH1B__NM_001145646	498	487	536	656
APH1B__NM_001145646_GGGTCAGGAGAACGATAAGG	APH1B__NM_001145646	134	118	157	138
APH1B__NM_001145646_GGTGGGTCAGGAGAACGATA	APH1B__NM_001145646	25	26	39	2
APH1B__NM_001145646_TACTGATTTCGTCCCTTGTT	APH1B__NM_001145646	1018	928	1059	1622
APH1B__NM_001145646_TAGGCCAGCAGTCGCATAGA	APH1B__NM_001145646	297	296	250	318
APH1B__NM_001145646_TCTTGCCATGAACCAAACAA	APH1B__NM_001145646	76	63	88	108
APH1B__NM_001145646_TTATCTTGCTGCATGTATTC	APH1B__NM_001145646	159	230	230	297
API5__NM_001142930_AAGACAGCAACTTGTAGAGT	API5__NM_001142930	607	454	607	390
API5__NM_001142930_AAGCTCCTCTACTGTCGGCA	API5__NM_001142930	195	107	316	256
API5__NM_001142930_AATTGCTTGACGTCGAATCT	API5__NM_001142930	271	212	407	309
API5__NM_001142930_ACCAGATGAAGTCTTAACAA	API5__NM_001142930	232	95	228	135
API5__NM_001142930_ACCATCCAATATCACTTGAT	API5__NM_001142930	963	413	1040	452
API5__NM_001142930_AGATGCCTATCAAGTGATAT	API5__NM_001142930	219	277	299	221
API5__NM_001142930_CAGGTCCTAGAAGATGTGAC	API5__NM_001142930	275	199	328	272
API5__NM_001142930_CGGCCAGGATGCCATAATTG	API5__NM_001142930	439	153	408	258
API5__NM_001142930_GAGGAGCTTTACCGCAATTA	API5__NM_001142930	376	219	441	305
API5__NM_001142930_GCTCACCATGCCGACAGTAG	API5__NM_001142930	468	235	497	298
API5__NM_001142930_GTTAGTATATCTGCCACTCG	API5__NM_001142930	289	216	320	267
API5__NM_001142930_TAATTGCTTGACGTCGAATC	API5__NM_001142930	165	143	117	181
API5__NM_001142931_GTCAGGATCCGAGGGATTGA	API5__NM_001142931	196	144	268	163
API5__NM_001142931_TCCACACAGTCAGGATCCGA	API5__NM_001142931	974	715	1028	758
API5__NM_001243747_ACATAACTGAACTGTAGCTT	API5__NM_001243747	609	389	626	409
API5__NM_001243747_AGTTTGGCTGTTAAGAAATC	API5__NM_001243747	197	120	199	228
API5__NM_001243747_CAAGCTACAGTTCAGTTATG	API5__NM_001243747	1305	741	1292	624
APIP__NM_015957_ACGTCATGGAGTATATGTGT	APIP__NM_015957	1011	655	964	809
APIP__NM_015957_AGTTCTGGGATCAGGTATCT	APIP__NM_015957	175	75	241	168
APIP__NM_015957_CCATCACAGCAGCTTTAGAG	APIP__NM_015957	479	407	577	336
APIP__NM_015957_GACGTCATGGAGTATATGTG	APIP__NM_015957	827	536	975	713
APIP__NM_015957_GTACTTCCGGAGGGTATTAT	APIP__NM_015957	147	157	176	193
APIP__NM_015957_GTCATGGAGTATATGTGTGG	APIP__NM_015957	551	452	617	483
APIP__NM_015957_TCTGGCTGTGATGCTCGGGA	APIP__NM_015957	448	486	457	564
APIP__NM_015957_TTCCCGGAGATGCGGCGCGC	APIP__NM_015957	33	23	61	119
APITD1-CORT__NM_001243768_AAGATGTGAAGCTCTTAGC	APITD1-CORT__NM_001243768	38	35	15	16
APITD1-CORT__NM_001243768_CACATCTTCAGTGTTAATTG	APITD1-CORT__NM_001243768	404	321	374	429
APITD1-CORT__NM_001243768_CCCGGCGCGCAGATTGCTG	APITD1-CORT__NM_001243768	1951	2866	2103	2753
APITD1-CORT__NM_001243768_TGCAGGAAGCGGCAGGAA	APITD1-CORT__NM_001243768	146	126	180	236
APITD1-CORT__NM_001243768_TGTCCCGGCGCGCAGATTG	APITD1-CORT__NM_001243768	87	80	113	173
APITD1-CORT__NM_001243768_TTCCTCTCCTATGAGGGG	APITD1-CORT__NM_001243768	9	4	4	0
APITD1-CORT__NM_001270517_ACTGCTGCCTTTAGCCTCTG	APITD1-CORT__NM_001270517	618	571	599	652
APITD1-CORT__NM_001270517_CAGCTCCGAAATGGCCGCA	APITD1-CORT__NM_001270517	1769	1827	1902	1760
APITD1-CORT__NM_001270517_GAGCTGACTTTCCGACAGT	APITD1-CORT__NM_001270517	580	578	671	786
APITD1-CORT__NM_001270517_GCAGCAGTTCACTATACTG	APITD1-CORT__NM_001270517	2661	1692	2863	2027
APITD1-CORT__NM_001270517_GGCAGCAGTTCACTATACT	APITD1-CORT__NM_001270517	1095	878	1172	881
APITD1-CORT__NM_001270517_GTTCAGCAAACAGACCATT	APITD1-CORT__NM_001270517	444	423	459	266
APITD1-CORT__NM_001270517_TACTGTGGGTTGTCTTTGC	APITD1-CORT__NM_001270517	676	539	891	808
APLF__NM_173545_AATTTACCTCATGAGACTAC	APLF__NM_173545	181	137	166	217
APLF__NM_173545_ATGAGGTAAATTAATCACGG	APLF__NM_173545	356	404	373	193
APLF__NM_173545_CAGAGGGTATAGAGAGAATG	APLF__NM_173545	257	332	317	372
APLF__NM_173545_CATGAGGTAAATTAATCACG	APLF__NM_173545	529	467	627	551
APLF__NM_173545_CTTGCTGGGTTGTGTTTAGC	APLF__NM_173545	645	711	708	820
APLF__NM_173545_GAGGCACCAGTAGTCTCATG	APLF__NM_173545	829	736	858	673
APLF__NM_173545_TCATGAGGTAAATTAATCAC	APLF__NM_173545	263	416	444	411
APLF__NM_173545_TCTGAAGACTGGTAAAAACA	APLF__NM_173545	362	463	403	527
APLF__NM_173545_TGAAAATTGTTCGAGTGCCC	APLF__NM_173545	237	257	333	349
APLF__NM_173545_TGGAAATGCAATGTACCTTA	APLF__NM_173545	80	117	87	101
APLF__NM_173545_TTCAAATAGCACCATAGATT	APLF__NM_173545	51	48	49	85
APLF__NM_173545_TTGCTGGGTTGTGTTTAGCT	APLF__NM_173545	188	252	247	209
APLN__NM_017413_CCCTGATGCCGCTTCCCGAT	APLN__NM_017413	3378	4335	4114	4656
APLN__NM_017413_GAAAGGCATGGGTCCCTTAT	APLN__NM_017413	638	551	567	611
APLN__NM_017413_GCTCTGGCTCTCCTTGACCG	APLN__NM_017413	1138	1486	1102	1287
APLN__NM_017413_TCCCTGATGCCGCTTCCCGA	APLN__NM_017413	1656	1752	1959	2068
APLNR__NM_005161_AAGACCAACATGTAGATGGC	APLNR__NM_005161	187	410	188	240
APLNR__NM_005161_CATCCCTGCCATCTACATGT	APLNR__NM_005161	2209	1640	2381	1625
APLNR__NM_005161_CCACTGGCCTCACGATGGCC	APLNR__NM_005161	243	201	299	197
APLNR__NM_005161_CGGGCAACGGTCTGGTGCTC	APLNR__NM_005161	157	230	165	240
APLNR__NM_005161_GAGCACCAGACCGTTGCCCG	APLNR__NM_005161	2085	2856	2578	3426
APLNR__NM_005161_GTCATAGTGCCGGTACGTGT	APLNR__NM_005161	4029	3533	3853	3928
APLNR__NM_005161_GTCATGGTGTTACGCACCAC	APLNR__NM_005161	462	460	510	569
APLNR__NM_005161_GTGGGCTACCTACACGTACC	APLNR__NM_005161	292	328	324	250
APLNR__NM_005161_GTGGTGCGTAACACCATGAC	APLNR__NM_005161	103	100	180	129
APLNR__NM_005161_TCATGGTGTTACGCACCACC	APLNR__NM_005161	409	585	535	617
APLNR__NM_005161_TGGTGCTCTGGACCGTGTTT	APLNR__NM_005161	69	118	130	99
APLNR__NM_005161_TGTGGGCTACCTACACGTAC	APLNR__NM_005161	780	1053	956	963
APLP1__NM_001024807_AACTGCTGTCCCAGATGGGT	APLP1__NM_001024807	928	961	1060	1023
APLP1__NM_001024807_AATGTGAGAGTTCAACCCGG	APLP1__NM_001024807	352	225	416	319
APLP1__NM_001024807_ACGTGTGGAGCAGGCTACGC	APLP1__NM_001024807	579	531	617	671
APLP1__NM_001024807_CAGGGACCCCCGACCCATCT	APLP1__NM_001024807	1802	1806	1785	1533
APLP1__NM_001024807_CTGCTGTCCCAGATGGGTCG	APLP1__NM_001024807	319	441	418	481
APLP1__NM_001024807_GAAGGGCACAACCTGGTGGT	APLP1__NM_001024807	284	280	247	285
APLP1__NM_001024807_GCAGATTGCACGTGTGGAGC	APLP1__NM_001024807	440	326	438	558
APLP1__NM_001024807_GCCAGTAGATGATTACTTCG	APLP1__NM_001024807	547	567	579	754
APLP1__NM_001024807_GGTGCGCAGGTCCCGGTGAA	APLP1__NM_001024807	3653	3928	3931	4656
APLP1__NM_001024807_TGCAGCTCCGGGTACATCTG	APLP1__NM_001024807	510	682	628	728
APLP1__NM_001024807_TGCGCTCCTGGTGCAAGAAC	APLP1__NM_001024807	285	310	366	432
APLP1__NM_001024807_TGTGGCTCGGATCGGTTCCG	APLP1__NM_001024807	271	297	432	468
APLP2__NM_001142276_AAATGGGAACCTGATCCAAC	APLP2__NM_001142276	82	87	127	158
APLP2__NM_001142276_AGAAGTTCTTCAGTACTGTC	APLP2__NM_001142276	181	97	161	216
APLP2__NM_001142276_AGCAACAGCAAATCCTGTTC	APLP2__NM_001142276	710	699	799	937
APLP2__NM_001142276_AGTTCTTCAGTACTGTCAGG	APLP2__NM_001142276	115	67	171	146
APLP2__NM_001142276_AGTTGTCAATACTAACCCGC	APLP2__NM_001142276	355	229	364	265
APLP2__NM_001142276_ATGTGATGGAGGCAAACCAG	APLP2__NM_001142276	388	289	342	363
APLP2__NM_001142276_CCACAAAACATTGCGATTTG	APLP2__NM_001142276	667	722	584	722
APLP2__NM_001142276_CTGGCCGGCTACATCGAGGT	APLP2__NM_001142276	3561	3540	3728	4587
APLP2__NM_001142276_GGAATGACCTTATATAGCTA	APLP2__NM_001142276	134	95	183	153
APLP2__NM_001142276_TAGCCGGCCAGCGCCAAGGC	APLP2__NM_001142276	335	659	408	374
APLP2__NM_001142276_TAGGCTCTTGCAGCCAATGC	APLP2__NM_001142276	20	31	76	49
APLP2__NM_001142276_TTAGTATTGACAACTGGTGC	APLP2__NM_001142276	29	42	34	36
APLP2__NM_001142278_GGCACCGCAACCGAATGGAC	APLP2__NM_001142278	604	531	667	717
APLP2__NM_001243299_GAGCGGGTTTCCTCGGTCTA	APLP2__NM_001243299	580	778	722	877
APLP2__NM_001243299_TTTCCTCGGTCTACGGACGC	APLP2__NM_001243299	124	97	168	154
APMAP__NM_020531_AAAGAACCCCCGCTCTTGCT	APMAP__NM_020531	746	793	880	907
APMAP__NM_020531_ATAGAGACCATTGCCCGGTT	APMAP__NM_020531	815	887	937	702
APMAP__NM_020531_CAGGTCGTCACAGACGATGA	APMAP__NM_020531	115	181	88	77
APMAP__NM_020531_CCGGAGTCCATAGCACATAT	APMAP__NM_020531	153	100	93	91
APMAP__NM_020531_CCTGCCGTCCTTAGCCTCCG	APMAP__NM_020531	939	809	1056	989
APMAP__NM_020531_CGAAGCTGCGACAGGCAGAA	APMAP__NM_020531	339	510	495	684
APMAP__NM_020531_CGGAGTCCATAGCACATATT	APMAP__NM_020531	398	500	440	516
APMAP__NM_020531_CTGAGAGGCTGTGGATCTAT	APMAP__NM_020531	693	645	774	702
APMAP__NM_020531_GACCATTGCCCGGTTTGGTT	APMAP__NM_020531	2248	2081	2369	2751
APMAP__NM_020531_GAGTCCATAGCACATATTGG	APMAP__NM_020531	551	491	625	574
APMAP__NM_020531_GCCTGTCGCAGCTTCGTATT	APMAP__NM_020531	2121	2019	2115	1930
APMAP__NM_020531_GGAGTCCATAGCACATATTG	APMAP__NM_020531	237	144	295	203
APOA1__NM_000039_AGCAAGCTGCGCGAACAGCT	APOA1__NM_000039	163	184	211	261
APOA1__NM_000039_CTTGAGAGCCTCAAGGCGCG	APOA1__NM_000039	36	6	48	39
APOA1__NM_000039_GAGCCCCTGGGATCGAGTGA	APOA1__NM_000039	673	678	674	554
APOA1__NM_000039_GATGGAGCTCTACCGCCAGA	APOA1__NM_000039	550	390	488	406
APOA1__NM_000039_GCGCAGCTCGTCGCTGTAGG	APOA1__NM_000039	627	785	768	787
APOA1__NM_000039_GCTGCCAGAAATGCCGAGCC	APOA1__NM_000039	836	1113	987	1274
APOA1__NM_000039_GGACGCGCTGCGCACGCATC	APOA1__NM_000039	51	54	64	95
APOA1__NM_000039_GTTGTCAAGGAGCTTTAGGC	APOA1__NM_000039	145	113	106	122
APOA1__NM_000039_TGGCGCAGCTCGTCGCTGTA	APOA1__NM_000039	143	116	80	112
APOA1__NM_000039_TTGGGAAAACAGCTAAAGTA	APOA1__NM_000039	115	175	163	132
APOA1BP__NM_144772_ATACAGTTCATCAATCGTCA	APOA1BP__NM_144772	822	770	935	766
APOA1BP__NM_144772_ATCTGTGGCCCGGGGAATAA	APOA1BP__NM_144772	215	112	285	158
APOA1BP__NM_144772_CAACCATCTATTACCCCAAA	APOA1BP__NM_144772	1609	1177	1808	1693
APOA1BP__NM_144772_CAGACCATCGCCTGTCGCTC	APOA1BP__NM_144772	416	466	492	384
APOA1BP__NM_144772_CAGCTTCAAGGGCGATGTTC	APOA1BP__NM_144772	502	256	347	191
APOA1BP__NM_144772_CCAGGTGGGTCCCGAGCGAC	APOA1BP__NM_144772	257	167	270	204
APOA1BP__NM_144772_CCTTGGGGAAATGCCCGCAG	APOA1BP__NM_144772	429	291	356	125
APOA1BP__NM_144772_GCCCGGCCAGTTCCATAAGT	APOA1BP__NM_144772	1752	917	1965	1540
APOA1BP__NM_144772_GGGCTCCTGGACATGGACGT	APOA1BP__NM_144772	1106	536	1351	1176
APOA1BP__NM_144772_GGTGACCCAGTGTCAGAAAA	APOA1BP__NM_144772	92	163	123	96
APOA1BP__NM_144772_GTTCAGCGTGGACCAACTTA	APOA1BP__NM_144772	192	108	212	189
APOA1BP__NM_144772_TACAGTTCATCAATCGTCAT	APOA1BP__NM_144772	508	622	661	559
APOA2__NM_001643_GTCTTACTTTGAAAAGTCAA	APOA2__NM_001643	108	45	68	76
APOA2__NM_001643_TAACTTCTTGAGCTATTTCG	APOA2__NM_001643	281	429	299	328
APOA4__NM_000482_ACTTTGAATTCGTCAGCGTA	APOA4__NM_000482	62	120	47	172
APOA4__NM_000482_ATTCGTCAGCGTAGGGCGTA	APOA4__NM_000482	20	49	22	34
APOA4__NM_000482_CAACCTGAGGGGCAACACCG	APOA4__NM_000482	810	1043	911	1196
APOA4__NM_000482_CAGCCTGGCTCCCTATGCTC	APOA4__NM_000482	146	91	163	147
APOA4__NM_000482_CGTAGGGCTCCACCCGGCGT	APOA4__NM_000482	1217	1125	1400	1293
APOA4__NM_000482_GACGCCGGGTGGAGCCCTAC	APOA4__NM_000482	1765	1348	1960	2021
APOA4__NM_000482_GACTTTGAATTCGTCAGCGT	APOA4__NM_000482	427	470	571	791
APOA4__NM_000482_GCCTTCCACGTCCCCCGCAT	APOA4__NM_000482	2669	1639	2917	2055
APOA4__NM_000482_GCCTTGAGCTCGTCGGCGTG	APOA4__NM_000482	986	1140	1055	1487
APOA4__NM_000482_GGCCAAGATCGACCAGAACG	APOA4__NM_000482	864	688	1064	1152
APOA4__NM_000482_TCCTGCGTGTCCTGAGCATA	APOA4__NM_000482	85	12	59	26
APOA5__NM_001166598_CAGGAGCAGTTGCGCGTGGT	APOA5__NM_001166598	1285	1085	1383	1099
APOA5__NM_001166598_CCAGGCTCTCGGCGTATGGG	APOA5__NM_001166598	1036	647	980	926
APOA5__NM_001166598_CGCGAGCCATCTTCTGCTGA	APOA5__NM_001166598	2248	3083	2463	3560
APOA5__NM_001166598_CTGCTGGATGCGTGCGTGCA	APOA5__NM_001166598	664	799	689	912
APOA5__NM_001166598_GCAACTGAAGCCCTACACGA	APOA5__NM_001166598	798	683	968	893
APOA5__NM_001166598_GCAGGAGCAGTTGCGCGTGG	APOA5__NM_001166598	2073	2076	2259	2498
APOA5__NM_001166598_GGAACTTGTTCATATTGTTG	APOA5__NM_001166598	249	268	325	439
APOA5__NM_001166598_TCACCAGGCTCTCGGCGTAT	APOA5__NM_001166598	1023	637	1230	1063
APOA5__NM_001166598_TCGTGCGCCTCTGCCATGTA	APOA5__NM_001166598	1334	1315	1596	1563
APOA5__NM_001166598_TGCCTTTGTCCCCGCTGGTC	APOA5__NM_001166598	522	591	713	842
APOA5__NM_001166598_TGCTGAGCTCTTCGCGCAGC	APOA5__NM_001166598	0	0	4	0
APOB__NM_000384_AGTACACACTGGACGCTAAG	APOB__NM_000384	181	280	262	227
APOB__NM_000384_ATGCCTTTGATGAGAGCAAG	APOB__NM_000384	200	203	194	324
APOB__NM_000384_CAGCCTGGTCTGTCCAAGTA	APOB__NM_000384	559	523	536	812
APOB__NM_000384_CGTCTTGACGGTAAAGTGAG	APOB__NM_000384	295	474	320	412
APOB__NM_000384_CTCACTTTACCGTCAAGACG	APOB__NM_000384	21	9	26	9
APOB__NM_000384_GAAGTACACATACAACTATG	APOB__NM_000384	883	1064	1173	1034
APOB__NM_000384_TACATCCTGAACATCAAGAG	APOB__NM_000384	299	525	409	345
APOB__NM_000384_TACCAGGAATAAGTATGGGA	APOB__NM_000384	245	220	345	297
APOB__NM_000384_TACCTTGCAGTTGATCCTGG	APOB__NM_000384	944	1095	1043	1019
APOB__NM_000384_TGATCAGAGTTGACAAGGGG	APOB__NM_000384	23	37	32	61
APOB__NM_000384_TTGATGTTCAGGATGTAAGT	APOB__NM_000384	77	24	81	83
APOB__NM_000384_TTTCCATACACGGTATCCTA	APOB__NM_000384	201	160	149	114
APOBEC1__NM_001644_AGATCACTAGAGTCACACCA	APOBEC1__NM_001644	341	513	415	425
APOBEC1__NM_001644_AGCCTGGGAGCATTCCCAGC	APOBEC1__NM_001644	48	24	69	47
APOBEC1__NM_001644_ATGGATCAACAAAATCGGCA	APOBEC1__NM_001644	229	132	228	326
APOBEC1__NM_001644_CAGAAACTCTCTAATAGCCT	APOBEC1__NM_001644	153	179	243	371
APOBEC1__NM_001644_CCCCAGAGAACTTCGTAAAG	APOBEC1__NM_001644	405	465	477	379
APOBEC1__NM_001644_CCTTAGAGTATTATCACTGC	APOBEC1__NM_001644	587	658	711	700
APOBEC1__NM_001644_CTCTAGTGATCTACGTAGCT	APOBEC1__NM_001644	24	86	77	70
APOBEC1__NM_001644_CTGCTCTACGAAATCAAGTG	APOBEC1__NM_001644	159	207	130	288
APOBEC1__NM_001644_CTTGATTTCGTAGAGCAGAC	APOBEC1__NM_001644	153	156	214	366
APOBEC1__NM_001644_TAGATCACTAGAGTCACACC	APOBEC1__NM_001644	260	199	302	214
APOBEC1__NM_001644_TAGTTACTCCACTGTTAACA	APOBEC1__NM_001644	98	101	125	86
APOBEC1__NM_001644_TCAGAAACTCTCTAATAGCC	APOBEC1__NM_001644	133	209	165	248
APOBEC2__NM_006789_ACCAGGTGACATTGTACCGC	APOBEC2__NM_006789	304	300	431	386
APOBEC2__NM_006789_ACTTCTTTAAATTCCAGTTC	APOBEC2__NM_006789	136	290	213	258
APOBEC2__NM_006789_ATGTGGAGTACAGTTCCGGG	APOBEC2__NM_006789	887	1016	819	846
APOBEC2__NM_006789_CCAGGTGACATTGTACCGCA	APOBEC2__NM_006789	739	734	726	620
APOBEC2__NM_006789_GCAAGAAGAGGGTGAATCCA	APOBEC2__NM_006789	459	439	539	335
APOBEC2__NM_006789_GGAGAACTTCCTATACTACG	APOBEC2__NM_006789	486	660	478	396
APOBEC2__NM_006789_GGCATCTCGGGGATACCTAG	APOBEC2__NM_006789	428	489	510	487
APOBEC2__NM_006789_GGGGCTGGAGGACACATACC	APOBEC2__NM_006789	408	332	441	439
APOBEC2__NM_006789_GGTGGGTCGACTCTTCATGT	APOBEC2__NM_006789	289	212	368	308
APOBEC2__NM_006789_TACCCTGTGACAATCTCAAA	APOBEC2__NM_006789	385	324	578	437
APOBEC2__NM_006789_TGGTGGGTCGACTCTTCATG	APOBEC2__NM_006789	37	13	76	67
APOBEC2__NM_006789_TTACCCTGTGACAATCTCAA	APOBEC2__NM_006789	125	108	122	174
APOBEC3A_B__NM_001193289_AAGTTGGAAGTGAATATG	APOBEC3A_B__NM_001193289	177	199	229	253
APOBEC3A_B__NM_001193289_GACCTACCTGTGCTACGAA	APOBEC3A_B__NM_001193289	399	344	454	536
APOBEC3A_B__NM_001193289_GGCGCTCCACTTCGTAGCA	APOBEC3A_B__NM_001193289	209	399	295	348
APOBEC3A_B__NM_001193289_TTCACTTCCAACTTTAACAA	APOBEC3A_B__NM_001193289	73	139	59	57
APOBEC3B__NM_001270411_AAGGTCAGCAATTCATGCCT	APOBEC3B__NM_001270411	481	534	590	661
APOBEC3B__NM_001270411_ACATCCCTGGCGGTACACAA	APOBEC3B__NM_001270411	441	476	679	745
APOBEC3B__NM_001270411_AGGAGCCCGCGTGACGATCA	APOBEC3B__NM_001270411	548	782	617	695
APOBEC3B__NM_001270411_ATAGTCCATGATCGTCACGC	APOBEC3B__NM_001270411	576	522	645	910
APOBEC3B__NM_001270411_CACATTTCTGCGTGGTACTG	APOBEC3B__NM_001270411	287	253	376	544
APOBEC3B__NM_001270411_CATGGGCTTTCTATGCAACG	APOBEC3B__NM_001270411	753	782	827	772
APOBEC3B__NM_001270411_CCAGACCTACTTGTGCTATG	APOBEC3B__NM_001270411	1258	1480	1440	1671
APOBEC3B__NM_001270411_TGACATTGGGGTGCTCAGAC	APOBEC3B__NM_001270411	431	535	411	587
APOBEC3C__NM_014508_ACCTATGGAAGCCAACGAT	APOBEC3C__NM_014508	466	436	567	383
APOBEC3C__NM_014508_CCAACGATCGGAACGAAACT	APOBEC3C__NM_014508	682	473	788	788
APOBEC3C__NM_014508_CCAAGTTTCGTTCCGATCGT	APOBEC3C__NM_014508	1536	2035	1589	2180
APOBEC3C__NM_014508_CCGATGAAGGCAATGTATCC	APOBEC3C__NM_014508	586	607	779	619
APOBEC3C__NM_014508_CCTCCTGGTAACATGGATAC	APOBEC3C__NM_014508	842	971	849	858
APOBEC3C__NM_014508_GCGGCGCTTTATACCTTCCA	APOBEC3C__NM_014508	762	1095	1076	1051
APOBEC3C__NM_014508_GTGACCTGGTACTTTGTGTT	APOBEC3C__NM_014508	3786	4646	4145	5414
APOBEC3C__NM_014508_TGGCTGTGCTTCACCGTGGA	APOBEC3C__NM_014508	256	316	143	220
APOBEC3D__NM_152426_ACGTCAGTCGAATCACAGGC	APOBEC3D__NM_152426	435	525	569	664
APOBEC3D__NM_152426_ACTACCGGGATAGAGATTGG	APOBEC3D__NM_152426	634	467	573	516
APOBEC3D__NM_152426_ACTGACGTTTGGGTAGTACC	APOBEC3D__NM_152426	459	356	410	452
APOBEC3D__NM_152426_AGATCACCTGGTTTGTATCA	APOBEC3D__NM_152426	1036	827	1117	1244
APOBEC3D__NM_152426_CCAGCTTTCGTTCCGACCAC	APOBEC3D__NM_152426	870	1120	1087	1238
APOBEC3D__NM_152426_CCGGGATAGAGATTGGCGGT	APOBEC3D__NM_152426	1397	1547	1581	1509
APOBEC3D__NM_152426_CCTCAGGCTGCATAAGGCAG	APOBEC3D__NM_152426	67	199	90	176
APOBEC3D__NM_152426_CCTGTGATTCGACTGACGTT	APOBEC3D__NM_152426	1435	1482	1706	1610
APOBEC3D__NM_152426_CTGTGATTCGACTGACGTTT	APOBEC3D__NM_152426	601	400	560	769
APOBEC3D__NM_152426_GACTGACGTTTGGGTAGTAC	APOBEC3D__NM_152426	625	484	611	717
APOBEC3D__NM_152426_GGGGCCTCCGTGAAGATCAT	APOBEC3D__NM_152426	696	1058	660	809
APOBEC3D__NM_152426_GGGGTGCTCAGCCAAGAATT	APOBEC3D__NM_152426	630	609	668	657
APOBEC3F__NM_001006666_AAAGATCTTTGCGTCCAAAC	APOBEC3F__NM_001006666	91	126	130	117
APOBEC3F__NM_001006666_AAGATCTTTGCGTCCAAACG	APOBEC3F__NM_001006666	75	115	92	143
APOBEC3F__NM_001006666_CTCCCCAGCTGACCGCAGGC	APOBEC3F__NM_001006666	373	482	424	406
APOBEC3F__NM_001006666_GGAACAAGGCAGACCCTAGA	APOBEC3F__NM_001006666	187	151	172	130
APOBEC3F__NM_145298_AAAAACCTACGCAAAGCCTA	APOBEC3F__NM_145298	106	175	94	81
APOBEC3F__NM_145298_ACCTACGCAAAGCCTATGGT	APOBEC3F__NM_145298	1511	1471	1714	1871
APOBEC3F__NM_145298_AGATCAATGGCCAGACTGTT	APOBEC3F__NM_145298	712	863	811	795
APOBEC3F__NM_145298_AGGAAGAGGCTTTAATCAGC	APOBEC3F__NM_145298	404	208	411	267
APOBEC3F__NM_145298_AGGAGACAGACCATGAGGCC	APOBEC3F__NM_145298	860	1028	823	856
APOBEC3F__NM_145298_ATAAAACAAGCGGTCAGGTC	APOBEC3F__NM_145298	97	137	139	172
APOBEC3F__NM_145298_ATCGTCCATAATCTTCACGC	APOBEC3F__NM_145298	1254	1862	1539	1928
APOBEC3F__NM_145298_CAACCCTTCCTAGTGCCCAT	APOBEC3F__NM_145298	1938	1511	2177	1658
APOBEC3F__NM_145298_CAAGCGTGGGCTACGTGACT	APOBEC3F__NM_145298	1512	1777	1656	1871
APOBEC3F__NM_145298_CACCTGTAGTTGAAGCTACT	APOBEC3F__NM_145298	919	1198	943	1287
APOBEC3F__NM_145298_CAGGAGAATCACTTGAACAA	APOBEC3F__NM_145298	35	0	25	0
APOBEC3F__NM_145298_CATCGTCCATAATCTTCACG	APOBEC3F__NM_145298	864	1014	1024	1295
APOBEC3F__NM_145298_CCAGCTTTCGTTCCGACCAT	APOBEC3F__NM_145298	1643	2068	1770	2251
APOBEC3F__NM_145298_CGCTTGAACTCGGAAGGCAG	APOBEC3F__NM_145298	35	0	54	0
APOBEC3F__NM_145298_CGTGATAGATTGTGCAAGCG	APOBEC3F__NM_145298	402	466	333	395
APOBEC3F__NM_145298_GCAGGAGATTCTCGAGTGAG	APOBEC3F__NM_145298	1590	1782	1386	1979
APOBEC3F__NM_145298_GGGAGAACAGAGACAGCAAC	APOBEC3F__NM_145298	79	138	130	129
APOBEC3F__NM_145298_GGTAGCAGCGCCAAACAGTC	APOBEC3F__NM_145298	435	483	400	530
APOBEC3F__NM_145298_GTGATAGATTGTGCAAGCGT	APOBEC3F__NM_145298	176	56	152	67
APOBEC3F__NM_145298_GTGGCCCGCATTATTCATGT	APOBEC3F__NM_145298	384	317	466	382
APOBEC3F__NM_145298_TAGAATTGTAAGCCCTTAAA	APOBEC3F__NM_145298	75	1	78	9
APOBEC3F__NM_145298_TCATTGTCTCCTATTTCAGA	APOBEC3F__NM_145298	265	374	332	404
APOBEC3F__NM_145298_TCCATAGCTTCTGCAATAAA	APOBEC3F__NM_145298	95	132	172	198
APOBEC3F__NM_145298_TCCGACCATAGGCTTTGCGT	APOBEC3F__NM_145298	920	1071	966	990
APOBEC3F__NM_145298_TGCAGGAGATTCTCGAGTGA	APOBEC3F__NM_145298	193	269	276	225
APOBEC3G__NM_021822_AAACCGATTCAAATTGTCGC	APOBEC3G__NM_021822	564	500	589	591
APOBEC3G__NM_021822_AAAGAGAGCTATTTGAGCCT	APOBEC3G__NM_021822	562	627	564	704
APOBEC3G__NM_021822_AAGATCTTTGCGTCCAAAGG	APOBEC3G__NM_021822	135	292	179	249
APOBEC3G__NM_021822_AGGTCACCTGGTACATATCC	APOBEC3G__NM_021822	513	733	533	567
APOBEC3G__NM_021822_ATATTACTGCACATCATGCT	APOBEC3G__NM_021822	552	550	602	626
APOBEC3G__NM_021822_CACAAAGTGTACAAGGGATA	APOBEC3G__NM_021822	35	18	62	99
APOBEC3G__NM_021822_CGAAAGATCTTTGCGTCCAA	APOBEC3G__NM_021822	445	426	568	769
APOBEC3G__NM_021822_CTCTGGGTGGTACTTAAGTT	APOBEC3G__NM_021822	108	141	60	118
APOBEC3G__NM_021822_GCCCCTGCACAAAGTGTACA	APOBEC3G__NM_021822	432	428	499	687
APOBEC3G__NM_021822_GTTCCTGGCCGAGGACCCGA	APOBEC3G__NM_021822	130	179	153	130
APOBEC3G__NM_021822_TATTTAGGCAGATTATTCCA	APOBEC3G__NM_021822	399	528	385	484
APOBEC3G__NM_021822_TCAAGGAAACCGTGTTTATG	APOBEC3G__NM_021822	346	332	366	345
APOBEC3G__NM_021822_TCTTCCACTGGTTCAGCAAG	APOBEC3G__NM_021822	165	199	152	90
APOBEC3H__NM_001166002_AAGTCACCTGTTACCTCACG	APOBEC3H__NM_001166002	1514	1549	1662	1478
APOBEC3H__NM_001166002_ACAGTCGAGCCATAAAGCGA	APOBEC3H__NM_001166002	144	63	115	153
APOBEC3H__NM_001166002_AGGGCCTTCCTCGGGTAGTA	APOBEC3H__NM_001166002	209	221	292	337
APOBEC3H__NM_001166002_ATTAACGAGATCAAGTCCAT	APOBEC3H__NM_001166002	253	219	206	275
APOBEC3H__NM_001166002_CAACCCCTATAAGATGTTAG	APOBEC3H__NM_001166002	720	1076	801	1049

[illegible]

ARFGAP3__NM_001142293_GCTGTGGCTTGATAGTTGTG	ARFGAP3__NM_001142293	1046	1067	1115	1153
ARFGAP3__NM_001142293_GTGAACACCAAGTGACCGGT	ARFGAP3__NM_001142293	152	173	269	281
ARFGAP3__NM_001142293_TCAGGGTCCCACCGGTCACT	ARFGAP3__NM_001142293	919	767	762	671
ARFGAP3__NM_001142293_TGACTCAGACAGACTCGGCA	ARFGAP3__NM_001142293	310	234	368	354
ARFGAP3__NM_001142293_TTACCTAAAGTAGCCTTTGT	ARFGAP3__NM_001142293	284	345	350	417
ARFGAP3__NM_001142293_TTCTAGCTTGGGGCCAAAAA	ARFGAP3__NM_001142293	8	6	5	0
ARFGAP3__NM_001142293_TTCTTCTTTAGATACCACCT	ARFGAP3__NM_001142293	32	21	31	25
ARFGAP3__NM_001142293_TTGCGATGCATGCAAGTCGG	ARFGAP3__NM_001142293	313	254	233	364
ARFGAP3__NM_014570_GTCGTGCTGCTCAGCTCTAT	ARFGAP3__NM_014570	361	289	308	338
ARFGAP3__NM_014570_TCGTGCTGCTCAGCTCTATA	ARFGAP3__NM_014570	180	236	228	285
ARFGAP3__NM_014570_TCTCAAGCAACACGGAAGCA	ARFGAP3__NM_014570	214	550	335	268
ARFGEF1__NM_006421_AAATGTTATCTTTGCACGCA	ARFGEF1__NM_006421	140	175	144	254
ARFGEF1__NM_006421_AAGCGGAAACTGAAAAACAG	ARFGEF1__NM_006421	134	154	147	195
ARFGEF1__NM_006421_ACTAACTATGCGAGGACATT	ARFGEF1__NM_006421	549	433	645	657
ARFGEF1__NM_006421_AGAGATGTACTAACTATGCG	ARFGEF1__NM_006421	308	315	313	396
ARFGEF1__NM_006421_ATTGCTTATGGGCACTTGAC	ARFGEF1__NM_006421	256	205	357	323
ARFGEF1__NM_006421_CATCTGAGTGAGAGTAGCTT	ARFGEF1__NM_006421	189	168	180	125
ARFGEF1__NM_006421_CGTCTTCTTCCCCTCATACA	ARFGEF1__NM_006421	1085	1057	1070	1074
ARFGEF1__NM_006421_CTCGCAAGCTTTGCGCAGCT	ARFGEF1__NM_006421	110	261	149	214
ARFGEF1__NM_006421_GAGGTGATTCAGGCTCGTGA	ARFGEF1__NM_006421	95	32	67	54
ARFGEF1__NM_006421_GGAGAAGATATTGGCCGACA	ARFGEF1__NM_006421	637	371	747	500
ARFGEF1__NM_006421_TACATCTCTAGATTGCTTAC	ARFGEF1__NM_006421	287	319	367	331
ARFGEF1__NM_006421_TTGCCTGGTGTTGTACTATC	ARFGEF1__NM_006421	378	459	495	525
ARFGEF2__NM_006420_ACTCCTGGCATGTTCACCGT	ARFGEF2__NM_006420	1503	1367	2012	1707
ARFGEF2__NM_006420_CAGATTATCTGCTGACGACA	ARFGEF2__NM_006420	181	392	247	197
ARFGEF2__NM_006420_CCATGAGCTGCGTTCCAAGG	ARFGEF2__NM_006420	700	678	735	962
ARFGEF2__NM_006420_CCTGTCATCGGCTATCCCGT	ARFGEF2__NM_006420	1495	1135	1776	1139
ARFGEF2__NM_006420_CTTCAAACGAACGAACTTTG	ARFGEF2__NM_006420	264	253	255	336
ARFGEF2__NM_006420_CTTGTCGTCAGCAGATAATC	ARFGEF2__NM_006420	517	357	563	532
ARFGEF2__NM_006420_CTTTAGAAACTCATCGCATA	ARFGEF2__NM_006420	97	164	187	293
ARFGEF2__NM_006420_GAAGATCCTAGCCGACAAGG	ARFGEF2__NM_006420	187	181	165	155
ARFGEF2__NM_006420_GGAACGCAGCTCATGGGATC	ARFGEF2__NM_006420	712	591	818	703
ARFGEF2__NM_006420_TGCACAGGGAGCGGAACACA	ARFGEF2__NM_006420	564	525	593	649
ARFGEF2__NM_006420_TGCTTCAAACGAACGAACTT	ARFGEF2__NM_006420	104	64	49	140
ARFGEF2__NM_006420_TTTAGAAACTCATCGCATAC	ARFGEF2__NM_006420	83	86	64	103
ARFIP1__NM_001025593_ACTTCTCCATTACTAGTCAC	ARFIP1__NM_001025593	193	322	242	269
ARFIP1__NM_001025593_ATCTCTGAGAAGCTAGGCCG	ARFIP1__NM_001025593	418	480	447	500
ARFIP1__NM_001025593_ATTTCATCTGCTAGAATAAC	ARFIP1__NM_001025593	104	133	115	150
ARFIP1__NM_001025593_CAAAGCCATGAGATGTAATT	ARFIP1__NM_001025593	741	1030	771	1074
ARFIP1__NM_001025593_CAGAGAACACAGACAAAAAG	ARFIP1__NM_001025593	218	248	270	158
ARFIP1__NM_001025593_CTCGTGAACATAGCTTTAAT	ARFIP1__NM_001025593	5	0	1	0
ARFIP1__NM_001025593_GAAACCCAAATTACATCTCA	ARFIP1__NM_001025593	266	209	311	316
ARFIP1__NM_001025593_GAAATTCCAGTGACTAGTAA	ARFIP1__NM_001025593	269	416	387	363
ARFIP1__NM_001025593_GCCTTTGGGTATGTACCATC	ARFIP1__NM_001025593	1002	793	941	754
ARFIP1__NM_001025593_GTACATACCCAAAGGCAACT	ARFIP1__NM_001025593	284	272	287	405
ARFIP1__NM_001025593_GTTCTAGTGACTTCAAACTC	ARFIP1__NM_001025593	26	25	58	71
ARFIP1__NM_001025593_TCGTGAACATAGCTTTAATA	ARFIP1__NM_001025593	28	5	28	0
ARFIP1__NM_001025595_CAGGGTTGCAGCTAGTCGAC	ARFIP1__NM_001025595	428	757	528	718
ARFIP2__NM_001242854_ACAATGGAGATCCCTATCCA	ARFIP2__NM_001242854	791	922	877	870
ARFIP2__NM_001242854_AGTTTGACATCGTCAAGAAA	ARFIP2__NM_001242854	276	493	299	403
ARFIP2__NM_001242854_ATGTGCTCGAAGAGAAGTAT	ARFIP2__NM_001242854	125	76	158	84
ARFIP2__NM_001242854_CAATGGAGATCCCTATCCAC	ARFIP2__NM_001242854	1126	1399	1219	1183
ARFIP2__NM_001242854_CTTCGCCGTTCCCGTGGATA	ARFIP2__NM_001242854	34	6	14	43
ARFIP2__NM_001242854_GACGGACGGGATCCTAGGGA	ARFIP2__NM_001242854	268	178	241	206
ARFIP2__NM_001242854_GAGGTGGCTCGGGGCATTGC	ARFIP2__NM_001242854	33	74	40	58
ARFIP2__NM_001242854_GCATTGTGTCTGGTGGCTAT	ARFIP2__NM_001242854	1609	2030	1955	2329
ARFIP2__NM_001242854_GCTTCGCCGTTCCCGTGGAT	ARFIP2__NM_001242854	127	82	92	154
ARFIP2__NM_001242854_GGATGGCGGCCAGACCCTAA	ARFIP2__NM_001242854	220	98	221	195
ARFIP2__NM_001242854_GGTGGGGCCACAGAGGTGTC	ARFIP2__NM_001242854	452	335	397	427
ARFIP2__NM_001242854_TGCTCGAAGAGAAGTATGGG	ARFIP2__NM_001242854	212	301	313	344
ARFIP2__NM_001242855_CAACTGTTATCAGAACGATT	ARFIP2__NM_001242855	906	754	929	900
ARFIP2__NM_001242855_GGCATCAACACCTATAAGGT	ARFIP2__NM_001242855	57	66	61	109
ARFIP2__NM_001242855_GTTTGACATCGTCAAGAAAT	ARFIP2__NM_001242855	76	103	70	123
ARFIP2__NM_001242855_TGCCTACCGAACAGACTTAG	ARFIP2__NM_001242855	329	482	361	451
ARFIP2__NM_001242855_TGTCTCTAGCATCAACACAT	ARFIP2__NM_001242855	159	127	156	141
ARFIP2__NM_001242855_TTATCAGAACGATTTGGTCG	ARFIP2__NM_001242855	430	562	405	671
ARFIP2__NM_001242855_TTTGACATCGTCAAGAAATG	ARFIP2__NM_001242855	58	130	95	91
ARFIP2__NM_012402_AAGAGCTGCCATTGGCTGAC	ARFIP2__NM_012402	256	237	245	224
ARFIP2__NM_012402_ACAGCACTTTGGTTTAGTCA	ARFIP2__NM_012402	874	984	924	944
ARFIP2__NM_012402_AGCCAGCACCAAGATAGCTA	ARFIP2__NM_012402	163	131	151	179
ARFIP2__NM_012402_AGCTCTAGTCAGATGATTGC	ARFIP2__NM_012402	301	513	388	525
ARFIP2__NM_012402_AGCTGGGACTACAGGACTAC	ARFIP2__NM_012402	99	0	139	33
ARFIP2__NM_012402_AGTGATGAGGGCTAACGTCT	ARFIP2__NM_012402	400	382	499	416
ARFIP2__NM_012402_ATCCATAGCTATCTTGGTGC	ARFIP2__NM_012402	316	265	447	568
ARFIP2__NM_012402_CAGCAGATTCCCATAGTGCA	ARFIP2__NM_012402	388	411	422	513
ARFIP2__NM_012402_CAGTGCTTAGGGCCTACTGA	ARFIP2__NM_012402	783	900	807	673
ARFIP2__NM_012402_CCAGCGAGGTCAATTTGTCG	ARFIP2__NM_012402	1407	1627	1601	1880
ARFIP2__NM_012402_CCAGGGACTGCGTTAGGCCC	ARFIP2__NM_012402	169	201	199	175
ARFIP2__NM_012402_CCCCGACAAATTGACCTCGC	ARFIP2__NM_012402	1283	1658	1505	1615
ARFIP2__NM_012402_CTACTATGCTACCCAAGATG	ARFIP2__NM_012402	91	117	95	111
ARFIP2__NM_012402_CTCCAGCCTGGGCAACAGAG	ARFIP2__NM_012402	10	0	0	0
ARFIP2__NM_012402_CTGCAAGCATGATCCCCGTC	ARFIP2__NM_012402	945	956	1019	1215
ARFIP2__NM_012402_CTGTGAGGATCCCTGCACTA	ARFIP2__NM_012402	731	992	846	1032
ARFIP2__NM_012402_CTTAAGATCCATAGCTATCT	ARFIP2__NM_012402	43	35	60	43
ARFIP2__NM_012402_GAGTGATGAGGGCTAACGTC	ARFIP2__NM_012402	319	314	482	473
ARFIP2__NM_012402_GGTATTAGACGCAACATCAT	ARFIP2__NM_012402	373	522	446	689
ARFIP2__NM_012402_GGTTGTAGGGCCTAAAAACA	ARFIP2__NM_012402	141	242	167	266
ARFIP2__NM_012402_TAAGCACAATTAAACTGGGT	ARFIP2__NM_012402	584	432	600	589
ARFIP2__NM_012402_TCCTTGGCCAAAGGGCATAC	ARFIP2__NM_012402	53	101	78	59
ARFIP2__NM_012402_TGGTTGTAGGGCCTAAAAAC	ARFIP2__NM_012402	0	0	0	0
ARFIP2__NM_012402_TTGTGAAGGAAACCCAGACG	ARFIP2__NM_012402	385	453	414	523
ARFIP2__NM_012402_TTTGCCCACATTGCTATGGT	ARFIP2__NM_012402	2141	3021	2330	2921
ARFRP1__NM_001134758_CACATCCACAGTGCCGACTG	ARFRP1__NM_001134758	60	14	67	1
ARFRP1__NM_001134758_CACCGTGGGCCTAAACAGTA	ARFRP1__NM_001134758	227	30	221	26
ARFRP1__NM_001134758_CGTGACACTCCGCATAATAC	ARFRP1__NM_001134758	648	199	756	355
ARFRP1__NM_001134758_CTGAAGGCCGTCTTGATGTC	ARFRP1__NM_001134758	444	137	524	233
ARFRP1__NM_001134758_GCTGAGTCCAAGCAGGCGTT	ARFRP1__NM_001134758	601	119	713	154
ARFRP1__NM_001134758_GTACAAGTACATGTTTCAGA	ARFRP1__NM_001134758	328	27	310	31
ARFRP1__NM_001134758_GTGACACTCCGCATAATACT	ARFRP1__NM_001134758	680	42	637	42
ARFRP1__NM_001134758_TGAAGGCCGTCTTGATGTCA	ARFRP1__NM_001134758	780	177	816	282
ARFRP1__NM_001267546_GCGGCAGAGGGACATCACGT	ARFRP1__NM_001267546	1173	186	1215	409
ARFRP1__NM_001267546_GGATGGTGAAGTGTGTCGTG	ARFRP1__NM_001267546	512	244	552	251
ARG1__NM_000045_AATATCCTTGGCAGATATAC	ARG1__NM_000045	168	180	241	197
ARG1__NM_000045_ACCCAGATGACTCCAAGATC	ARG1__NM_000045	495	413	442	387
ARG1__NM_000045_AGAGTCCCACTCTATGTATG	ARG1__NM_000045	510	436	534	424
ARG1__NM_000045_AGGAGCTCCAATAATCCCTA	ARG1__NM_000045	299	338	403	417
ARG1__NM_000045_AGTGTGATGTGAAGGATTAT	ARG1__NM_000045	549	585	439	399
ARG1__NM_000045_ATATCCTTGGCAGATATACA	ARG1__NM_000045	178	73	247	134
ARG1__NM_000045_CAAGAGTCCCACTCTATGTA	ARG1__NM_000045	947	893	1057	939
ARG1__NM_000045_CACTCAGCTATCTACTAGGA	ARG1__NM_000045	1030	812	1127	919
ARG1__NM_000045_CCAGCCTTTCTCAATACTGT	ARG1__NM_000045	1018	954	1089	1470
ARG1__NM_000045_GAAACACTCAGCTATCTACT	ARG1__NM_000045	538	531	558	539
ARG1__NM_000045_GACTCCCTGTATATCTGCCA	ARG1__NM_000045	1122	1215	1388	1147
ARG1__NM_000045_GGAGACAAAGCTACCACATG	ARG1__NM_000045	302	236	297	345
ARG1__NM_000045_GTATATTGGCTTGAGAGACG	ARG1__NM_000045	202	271	271	192
ARG1__NM_000045_TCCAGAACCATAGGGATTAT	ARG1__NM_000045	128	266	152	146
ARG1__NM_000045_TTTGAAAGGGACTGTCATTA	ARG1__NM_000045	123	127	177	187
ARG2__NM_001172_ATAGGAGCCCCGTTCTCACA	ARG2__NM_001172	562	501	709	451
ARG2__NM_001172_ATAGTGAATCCACGCTCAGT	ARG2__NM_001172	214	265	297	139
ARG2__NM_001172_CACTAATGGTACCGATTGCC	ARG2__NM_001172	1143	1708	1479	1400
ARG2__NM_001172_CCCGCTGCCATAAGAGAAGC	ARG2__NM_001172	228	548	357	475
ARG2__NM_001172_CTCAGTGGGTCTTGCCAACC	ARG2__NM_001172	305	328	364	427
ARG2__NM_001172_CTGAGCGTGGATTCACTATC	ARG2__NM_001172	786	742	772	799
ARG2__NM_001172_CTGGCAATCGGTACCATTAG	ARG2__NM_001172	229	195	244	305
ARG2__NM_001172_GAAGAAATCCGTCCACTCCG	ARG2__NM_001172	670	701	665	632
ARG2__NM_001172_GCCAGCTTCTCTTATGGCAG	ARG2__NM_001172	784	1089	966	1285
ARG2__NM_001172_TACAGCTGTGTCACACTGGG	ARG2__NM_001172	1353	1264	1558	1802
ARG2__NM_001172_TAGGAGCCCCGTTCTCACAA	ARG2__NM_001172	304	319	268	300
ARG2__NM_001172_TGGAATGCACTCGCGTCTGG	ARG2__NM_001172	68	57	93	39
ARGFX__NM_001012659_AGAGATACTGATACAGACAG	ARGFX__NM_001012659	110	20	140	122
ARGFX__NM_001012659_AGCTTTGAGACTCGACCTAC	ARGFX__NM_001012659	493	568	478	394
ARGFX__NM_001012659_AGGAGAAAAAGCATAAGGAC	ARGFX__NM_001012659	175	257	304	206
ARGFX__NM_001012659_CCAAATGCAAGATACTCAGT	ARGFX__NM_001012659	927	557	735	212
ARGFX__NM_001012659_CCTTTACTGTTGACTCCGGT	ARGFX__NM_001012659	1930	2097	2270	2555
ARGFX__NM_001012659_GAGAAAAAGCATAAGGACTG	ARGFX__NM_001012659	547	557	576	626
ARGFX__NM_001012659_GAGTTCCATGCCCAATTGGA	ARGFX__NM_001012659	355	314	344	204
ARGFX__NM_001012659_GATTGTACAGTTCTATGATT	ARGFX__NM_001012659	382	427	586	437
ARGFX__NM_001012659_GCCTATCTCCTGCACAAACC	ARGFX__NM_001012659	591	544	592	752
ARGFX__NM_001012659_TAGCTCCTCATACTGTTGGT	ARGFX__NM_001012659	242	134	240	152
ARGFX__NM_001012659_TCCAAATGCAAGATACTCAG	ARGFX__NM_001012659	292	270	332	235
ARGFX__NM_001012659_TTTCACCCACCAACAGTATG	ARGFX__NM_001012659	8	0	11	23
ARGLU1__NM_018011_AGAAGAAAGCGGAGTTCGAG	ARGLU1__NM_018011	265	329	284	202
ARGLU1__NM_018011_CATCGACATCTTCGGGCGCA	ARGLU1__NM_018011	991	227	1198	409
ARGLU1__NM_018011_CCGAAGATGTCGATGCGGTC	ARGLU1__NM_018011	1352	1811	1232	1625
ARGLU1__NM_018011_CCGTTTACTTTCCCGAGATT	ARGLU1__NM_018011	2421	2788	2809	2707
ARGLU1__NM_018011_CTCGGGAAAGTAAACGGAAC	ARGLU1__NM_018011	61	109	112	86
ARGLU1__NM_018011_GGAAGAACGTGCAAAACGTG	ARGLU1__NM_018011	295	376	260	353
ARGLU1__NM_018011_GTCCTTCTCATTAAAAACCC	ARGLU1__NM_018011	23	15	25	19
ARGLU1__NM_018011_TGAGGAGCTAGAGCGAATAC	ARGLU1__NM_018011	93	48	85	33
ARGLU1__NM_018011_TGCGCCCGAAGATGTCGATG	ARGLU1__NM_018011	2326	1230	2466	1607
ARHGAP1__NM_004308_AAACCCGGGCTACCAGAGAA	ARHGAP1__NM_004308	289	305	376	315
ARHGAP1__NM_004308_AAAGACCAAATAGATCCGAA	ARHGAP1__NM_004308	261	455	381	292
ARHGAP1__NM_004308_AACGTCTTCTGGTAACAGCG	ARHGAP1__NM_004308	277	296	347	411
ARHGAP1__NM_004308_AAGTCAGGCATTTCATCCGA	ARHGAP1__NM_004308	611	403	664	571
ARHGAP1__NM_004308_ACAGTCTCCCTGAGTACAAT	ARHGAP1__NM_004308	417	427	477	474
ARHGAP1__NM_004308_ACTAAGAGTGCCCGTAGGAG	ARHGAP1__NM_004308	183	82	209	187
ARHGAP1__NM_004308_AGACTAAGAGTGCCCGTAGG	ARHGAP1__NM_004308	57	121	73	119
ARHGAP1__NM_004308_AGATGTTGGAGCCACCCATC	ARHGAP1__NM_004308	576	693	605	449
ARHGAP1__NM_004308_AGCCCATTCCCATTGTACTC	ARHGAP1__NM_004308	681	746	733	648
ARHGAP1__NM_004308_AGGGGAGAGGCTAACCTGGC	ARHGAP1__NM_004308	626	881	763	746
ARHGAP1__NM_004308_AGTGTAATTCTACGAGACAG	ARHGAP1__NM_004308	148	269	212	228
ARHGAP1__NM_004308_AGTGTGACTCCGTAACAGAC	ARHGAP1__NM_004308	79	133	100	140
ARHGAP1__NM_004308_ATCACACGCTGTAGCTCTCG	ARHGAP1__NM_004308	89	176	133	116
ARHGAP1__NM_004308_CAATTTCCCCAGGGGATTAA	ARHGAP1__NM_004308	219	210	202	274
ARHGAP1__NM_004308_CATCGCCCGGCACCAGATCG	ARHGAP1__NM_004308	740	784	940	1025
ARHGAP1__NM_004308_CCAGTTCTTCTCATCGATGG	ARHGAP1__NM_004308	1079	1015	1012	889
ARHGAP1__NM_004308_CCCCACTGGTCTGTACCCCA	ARHGAP1__NM_004308	995	1040	1103	808
ARHGAP1__NM_004308_CCCCCAAGCCCATGCCCCCA	ARHGAP1__NM_004308	1263	1174	1390	1673
ARHGAP1__NM_004308_CTCTGTTTCCCTAGCAAGTG	ARHGAP1__NM_004308	176	297	131	256
ARHGAP1__NM_004308_CTTCTCTGGTAGCCCGGGTT	ARHGAP1__NM_004308	566	879	551	959
ARHGAP1__NM_004308_CTTGCGGTCAAACTCCCGGT	ARHGAP1__NM_004308	3935	2992	4522	3953
ARHGAP1__NM_004308_GAAATACATGATTGGTGCCA	ARHGAP1__NM_004308	405	338	483	435
ARHGAP1__NM_004308_GAAGTCAGGCATTTCATCCG	ARHGAP1__NM_004308	1068	960	1161	859
ARHGAP1__NM_004308_GAGGTTCTTTGAAAGACAAC	ARHGAP1__NM_004308	3	0	2	9
ARHGAP1__NM_004308_GCCGGGCGATGTCATAGTAT	ARHGAP1__NM_004308	1227	1211	1132	1440
ARHGAP1__NM_004308_GCCTCCTCTTCCACACATCC	ARHGAP1__NM_004308	1105	1449	1210	1301
ARHGAP1__NM_004308_GCTGGCTCCGTGATGCCTAC	ARHGAP1__NM_004308	1116	1170	1158	1465
ARHGAP1__NM_004308_GGAGTGAGATCAGCCATCAC	ARHGAP1__NM_004308	644	608	765	780
ARHGAP1__NM_004308_GGCCTGGGCATAACTTCGCT	ARHGAP1__NM_004308	1267	1302	1160	1617
ARHGAP1__NM_004308_GGCTCTGGAATCATCCTGCC	ARHGAP1__NM_004308	333	436	357	392
ARHGAP1__NM_004308_GGGAGCCTTTCGGATCTATT	ARHGAP1__NM_004308	487	519	621	504
ARHGAP1__NM_004308_GGGCCAGTTCTTCTCATCGA	ARHGAP1__NM_004308	1691	2403	1882	1883
ARHGAP1__NM_004308_GGGCCTGGGCATAACTTCGC	ARHGAP1__NM_004308	661	730	700	831
ARHGAP1__NM_004308_GGGGGACTCTTAAAGGGGCT	ARHGAP1__NM_004308	161	120	187	244
ARHGAP1__NM_004308_GTCCTCCCCTCTTGATTGCT	ARHGAP1__NM_004308	2137	1922	2338	2346
ARHGAP1__NM_004308_GTCTTTCTCATGTCCCCCAC	ARHGAP1__NM_004308	190	147	262	220
ARHGAP1__NM_004308_GTGGAGAGGGAGGTTTCAAG	ARHGAP1__NM_004308	177	134	225	221
ARHGAP1__NM_004308_GTGTAATTCTACGAGACAGT	ARHGAP1__NM_004308	204	208	335	251
ARHGAP1__NM_004308_TACCAGTCCCTGCACAACCA	ARHGAP1__NM_004308	337	450	339	291
ARHGAP1__NM_004308_TGGGCTTGGGGGCTGTCGCG	ARHGAP1__NM_004308	192	114	192	251
ARHGAP1__NM_004308_TTCCCAGCAATCAAGAGGGG	ARHGAP1__NM_004308	69	84	58	66
ARHGAP10__NM_024605_AAATTCAGAAAAGAGCAACT	ARHGAP10__NM_024605	185	228	186	262
ARHGAP10__NM_024605_ACAGTTCATAGAAGTGTTGC	ARHGAP10__NM_024605	333	257	393	412
ARHGAP10__NM_024605_ATATCCAAGTAGAGCAGAAC	ARHGAP10__NM_024605	77	54	71	57
ARHGAP10__NM_024605_CTCACGAAGCGGAACTCGAG	ARHGAP10__NM_024605	417	466	521	464
ARHGAP10__NM_024605_GAAAGACTCACATTTACAAG	ARHGAP10__NM_024605	313	288	272	271
ARHGAP10__NM_024605_GAGGATCCGCGCTCACGAAG	ARHGAP10__NM_024605	243	352	330	331
ARHGAP10__NM_024605_GATGGCTGTATTAAAACTAT	ARHGAP10__NM_024605	241	320	401	425
ARHGAP10__NM_024605_GATGTTTACCTTCTATCATC	ARHGAP10__NM_024605	573	410	582	441
ARHGAP10__NM_024605_GTGAATGAGCAAACTTCCGC	ARHGAP10__NM_024605	279	261	304	362
ARHGAP10__NM_024605_GTTGCCGGTTCTGCTCTACT	ARHGAP10__NM_024605	703	895	721	903
ARHGAP10__NM_024605_TAAACTGACTTGCTCGTTTG	ARHGAP10__NM_024605	201	248	255	426
ARHGAP10__NM_024605_TGATGGCTGTATTAAAACTA	ARHGAP10__NM_024605	135	153	135	280
ARHGAP11A__NM_001286479_AAACCATGAGAAGAATCTAC	ARHGAP11A__NM_001286479	119	134	143	111
ARHGAP11A__NM_001286479_AGAAAAGAAGCTACGATTA(	ARHGAP11A__NM_001286479	630	422	676	572
ARHGAP11A__NM_001286479_AGGCGAAGTAAAAGAATTG	ARHGAP11A__NM_001286479	188	189	207	204
ARHGAP11A__NM_001286479_ATAGATATGAAAGTGTTGG	ARHGAP11A__NM_001286479	465	279	570	590
ARHGAP11A__NM_001286479_ATGGTTTCTCAAGTAAGAAA	ARHGAP11A__NM_001286479	24	5	39	9
ARHGAP11A__NM_001286479_CACTGGTGATTCAGATAGCT	ARHGAP11A__NM_001286479	63	0	42	13
ARHGAP11A__NM_001286479_CTTCAATAGCAGTTCTACAC	ARHGAP11A__NM_001286479	285	356	381	264
ARHGAP11A__NM_001286479_GGCAATGTACGCTTAGCATT	ARHGAP11A__NM_001286479	679	593	698	574
ARHGAP11A__NM_001286479_GTCAATAGATATGAAAGTG1	ARHGAP11A__NM_001286479	312	321	338	265
ARHGAP11A__NM_001286479_TCACTCTCTCCTGTACTCAT	ARHGAP11A__NM_001286479	1012	610	1037	868
ARHGAP11A__NM_001286479_TCTTCTTGAGGTGTAATAGA	ARHGAP11A__NM_001286479	56	14	48	59
ARHGAP11A__NM_001286479_TGCGTTTGAAATTCAATCTA	ARHGAP11A__NM_001286479	287	188	346	367
ARHGAP11B__NM_001039841_AAGAGAAGACAACGTGTAG	ARHGAP11B__NM_001039841	267	240	353	415
ARHGAP11B__NM_001039841_AGAAAGAGAAGACAACGTG	ARHGAP11B__NM_001039841	149	150	284	185
ARHGAP12__NM_001270695_AACCACACTGACTATCTGAC	ARHGAP12__NM_001270695	1667	2233	2088	3017
ARHGAP12__NM_001270695_AGCCCGGCAATTCAGATTAA	ARHGAP12__NM_001270695	729	842	811	972
ARHGAP12__NM_001270695_CAAATGAGCGTGTCCTATTC	ARHGAP12__NM_001270695	1389	1540	1382	1816
ARHGAP12__NM_001270695_CCAAAATCCAGGGGATCAAG	ARHGAP12__NM_001270695	261	293	296	383
ARHGAP12__NM_001270695_CTTGCATCCCGAGTCCAACG	ARHGAP12__NM_001270695	1035	1323	1025	1448
ARHGAP12__NM_001270695_GAAACCTCCTCGTTGGACTC	ARHGAP12__NM_001270695	786	1021	972	1282
ARHGAP12__NM_001270695_GGAAACCTCCTCGTTGGACT	ARHGAP12__NM_001270695	867	1331	1068	1613
ARHGAP12__NM_001270695_GGCTGGCACATAAAACGCTT	ARHGAP12__NM_001270695	796	916	987	1171
ARHGAP12__NM_001270695_TCAGAAGAGTTGGATGAACG	ARHGAP12__NM_001270695	338	352	420	355
ARHGAP12__NM_001270695_TCAGACTGTCAACCTAAGCC	ARHGAP12__NM_001270695	585	773	603	688
ARHGAP12__NM_001270695_TCTTGAAGGTTAGCATAGAC	ARHGAP12__NM_001270695	57	90	77	115
ARHGAP12__NM_001270695_TTTGGACCCAGTTATAATCA	ARHGAP12__NM_001270695	206	236	206	334
ARHGAP12__NM_001270697_CAATATTACTACAGTGCAGA	ARHGAP12__NM_001270697	447	452	488	864
ARHGAP12__NM_001270697_CTCAAGCATGTTGATGATCA	ARHGAP12__NM_001270697	849	1075	1026	1541
ARHGAP15__NM_018460_ACTTAGCAATTCAGTGCTAG	ARHGAP15__NM_018460	444	673	566	507
ARHGAP15__NM_018460_ATATCGAGTTAGTGGCAATC	ARHGAP15__NM_018460	227	226	295	321
ARHGAP15__NM_018460_CCAGAAAGTGTGGATTTGTG	ARHGAP15__NM_018460	980	1169	1179	1458
ARHGAP15__NM_018460_GATGGAATATATCGAGTTAG	ARHGAP15__NM_018460	444	377	520	781
ARHGAP15__NM_018460_GTGTGGAGCACACATTGAAT	ARHGAP15__NM_018460	440	396	557	573
ARHGAP15__NM_018460_TACAAATTCTGATACTTCCG	ARHGAP15__NM_018460	501	585	465	643
ARHGAP15__NM_018460_TGGGCACAAACCAGAAAGTG	ARHGAP15__NM_018460	125	404	53	47
ARHGAP15__NM_018460_TGTGTGGAGCACACATTGAA	ARHGAP15__NM_018460	252	186	271	316
ARHGAP15__NM_018460_TTACAAAGAATCCAAGCAAC	ARHGAP15__NM_018460	180	213	170	238
ARHGAP15__NM_018460_TTCAGTGACCTTCCCGACAT	ARHGAP15__NM_018460	313	230	343	367
ARHGAP15__NM_018460_TTGACTTCATCATATTGGAT	ARHGAP15__NM_018460	140	90	160	205
ARHGAP15__NM_018460_TTGCAGATCACAACAGTATC	ARHGAP15__NM_018460	119	99	121	140
ARHGAP17__NM_001006634_AACATGCCACCAAGCGCTTA	ARHGAP17__NM_001006634	326	151	373	385
ARHGAP17__NM_001006634_ACCTTGATGGGCTCGCATTT	ARHGAP17__NM_001006634	1322	1558	1510	1630
ARHGAP17__NM_001006634_CACGTGTCTCCTCTCGGCAT	ARHGAP17__NM_001006634	1998	1665	1931	1813
ARHGAP17__NM_001006634_CCAAGCGCTTATGGAATGG	ARHGAP17__NM_001006634	1577	1548	1767	2371
ARHGAP17__NM_001006634_CCACCAAGCGCTTATGGGAA	ARHGAP17__NM_001006634	657	924	898	916
ARHGAP17__NM_001006634_CCACCATTCCCATAAGCGCT	ARHGAP17__NM_001006634	419	698	543	623
ARHGAP17__NM_001006634_CCATTCCCATAAGCGCTTGG	ARHGAP17__NM_001006634	743	984	807	1163
ARHGAP17__NM_001006634_CCTTAGTGAAGATCTATTAC	ARHGAP17__NM_001006634	906	969	1000	769
ARHGAP17__NM_001006634_CTCTTCAGGTGTTCTTCTAG	ARHGAP17__NM_001006634	122	49	161	137
ARHGAP17__NM_001006634_GAGGAAGCAGCTTGCAAGAT	ARHGAP17__NM_001006634	295	295	391	440
ARHGAP17__NM_001006634_GTATGGCAAATTCTTTGTTA	ARHGAP17__NM_001006634	499	651	690	584
ARHGAP17__NM_001006634_TCTCGGCATCGGTGCCATGC	ARHGAP17__NM_001006634	475	800	646	753
ARHGAP18__NM_033515_AAGACAAAACGGGTACCACA	ARHGAP18__NM_033515	82	151	101	114
ARHGAP18__NM_033515_AGCGGCAAGGACCAGACCGT	ARHGAP18__NM_033515	1461	1383	1526	1335
ARHGAP18__NM_033515_CAAGATCAGAGGAAAGTACC	ARHGAP18__NM_033515	142	149	194	116
ARHGAP18__NM_033515_CAAGGTTAGATGCCTCGTCT	ARHGAP18__NM_033515	657	684	735	577
ARHGAP18__NM_033515_CCCTGAAACAGACATCAACC	ARHGAP18__NM_033515	538	432	569	510
ARHGAP18__NM_033515_GAAATTGATCGATCAAATGG	ARHGAP18__NM_033515	120	101	109	95
ARHGAP18__NM_033515_GAGGGCAGTCAGCTCAATTA	ARHGAP18__NM_033515	281	280	382	383
ARHGAP18__NM_033515_TCAGCTCAATTAGGGCTAAA	ARHGAP18__NM_033515	206	184	152	248
ARHGAP18__NM_033515_TGGGAAATTGATCGATCAAA	ARHGAP18__NM_033515	64	116	53	121
ARHGAP18__NM_033515_TGTCTCTGACGTCAGGAATC	ARHGAP18__NM_033515	50	81	66	87
ARHGAP18__NM_033515_TTCTTTGGATGAACTATCTA	ARHGAP18__NM_033515	0	0	0	0
ARHGAP18__NM_033515_TTTCCTGGTTCATAGTGTAC	ARHGAP18__NM_033515	58	74	55	62
ARHGAP19__NM_001204300_ACTCAATCAGTTGGTATATC	ARHGAP19__NM_001204300	543	606	559	595
ARHGAP19__NM_001204300_AGTTTCTCCACAAAAAAGTC	ARHGAP19__NM_001204300	36	17	40	71
ARHGAP19__NM_001204300_CAGAAAAAGGAGTGATATTT	ARHGAP19__NM_001204300	444	320	488	363
ARHGAP19__NM_001204300_CGACTGAGGCACAGAGTGAA	ARHGAP19__NM_001204300	430	369	529	584
ARHGAP19__NM_001204300_CTCATGTCTCTCAAGCGAAA	ARHGAP19__NM_001204300	60	111	90	137
ARHGAP19__NM_001204300_GAAGTGGACCTTAAGTTGCC	ARHGAP19__NM_001204300	560	652	656	429
ARHGAP19__NM_001204300_GAGGTGCCAGCCCGCGAATC	ARHGAP19__NM_001204300	777	663	905	792
ARHGAP19__NM_001204300_GATAATGGGCTGACCTCGAA	ARHGAP19__NM_001204300	194	216	181	152
ARHGAP19__NM_001204300_GCAATGCCTTCCTCCGTCAG	ARHGAP19__NM_001204300	283	368	380	418
ARHGAP19__NM_001204300_TCAGAAAAAGGAGTGATATT	ARHGAP19__NM_001204300	216	284	247	275
ARHGAP19__NM_001204300_TGGACCTTAAGTTGCCTGGC	ARHGAP19__NM_001204300	118	173	130	138
ARHGAP19__NM_001204300_TGTCTCTCAAGCGAAAGGGT	ARHGAP19__NM_001204300	120	83	110	74
ARHGAP19__NM_001256423_AGAGGGTTTGTTTAGAGTAC	ARHGAP19__NM_001256423	350	271	303	255
ARHGAP19__NM_001256423_GAGGGTTTGTTTAGAGTACC	ARHGAP19__NM_001256423	195	119	233	184
ARHGAP20__NM_001258415_ACATGTCATTACCAATGCTA	ARHGAP20__NM_001258415	752	453	771	734
ARHGAP20__NM_001258415_AGTGGCCACAAAGTTCACTG	ARHGAP20__NM_001258415	1187	1149	1412	1417
ARHGAP20__NM_001258415_ATCAATCAGCAGAGTCCTAT	ARHGAP20__NM_001258415	97	40	114	73
ARHGAP20__NM_001258415_CATCATTAGTGTGCTCCAAT	ARHGAP20__NM_001258415	1472	1149	1698	1653
ARHGAP20__NM_001258415_CATGTGTCAACACTAGCAGA	ARHGAP20__NM_001258415	355	368	337	340
ARHGAP20__NM_001258415_CTGAGAGAGATTACCAGTTG	ARHGAP20__NM_001258415	472	751	634	660
ARHGAP20__NM_001258415_CTTACAGTTATCCCTAGCAT	ARHGAP20__NM_001258415	717	537	878	702
ARHGAP20__NM_001258415_GAGCGCTCCATCTCTTATCC	ARHGAP20__NM_001258415	727	767	833	701
ARHGAP20__NM_001258415_TACCAGTTGTGGGTCAATTC	ARHGAP20__NM_001258415	381	362	475	432
ARHGAP20__NM_001258415_TCCCCTCAAAATCTTCGCCA	ARHGAP20__NM_001258415	1069	1166	1193	1261
ARHGAP20__NM_001258415_TGAGAGAGATTACCAGTTGT	ARHGAP20__NM_001258415	560	499	590	418
ARHGAP20__NM_001258415_TGGATAAAGCCCTACAAAAA	ARHGAP20__NM_001258415	37	14	32	51
ARHGAP20__NM_020809_CCCCCCAGCAGGAGACTCTA	ARHGAP20__NM_020809	262	170	289	216
ARHGAP21__NM_020824_AGACAGACCAGTCCGACGCG	ARHGAP21__NM_020824	1187	997	1311	1181
ARHGAP21__NM_020824_AGCCAGGGATAGCAGATTGG	ARHGAP21__NM_020824	415	489	586	430
ARHGAP21__NM_020824_AGGAAAACAAAGAAACCGCT	ARHGAP21__NM_020824	180	162	179	155
ARHGAP21__NM_020824_ATGATGGCCACGCGTCGGAC	ARHGAP21__NM_020824	459	460	512	395
ARHGAP21__NM_020824_CAGTACTGACACAACCTGGT	ARHGAP21__NM_020824	1604	1125	1634	1173
ARHGAP21__NM_020824_GACCGAATTATAAAAGTCAA	ARHGAP21__NM_020824	224	257	249	312
ARHGAP21__NM_020824_GTCAATGGAGAAAGTGTTAT	ARHGAP21__NM_020824	205	262	317	274
ARHGAP21__NM_020824_TAAAGCAATTACTTGGGAAT	ARHGAP21__NM_020824	609	633	795	925
ARHGAP21__NM_020824_TCCATTCTATAGGCCCTACC	ARHGAP21__NM_020824	155	157	244	171
ARHGAP21__NM_020824_TGGAGGTTCAGGTATATTGC	ARHGAP21__NM_020824	28	15	22	20
ARHGAP21__NM_020824_TTGCTCAATGATGAGTCAGG	ARHGAP21__NM_020824	177	144	218	198
ARHGAP21__NM_020824_TTGTGTCAGTACTGGTGTAC	ARHGAP21__NM_020824	201	232	276	336
ARHGAP22__NM_001256024_ACCTGTGGCCGCAGAATGTT	ARHGAP22__NM_001256024	1418	1468	1463	1318
ARHGAP22__NM_001256024_AGCGCTGGTTTGTGCTGCGT	ARHGAP22__NM_001256024	1572	1662	1744	1982
ARHGAP22__NM_001256024_AGGGTGGGCATGCGATCTAC	ARHGAP22__NM_001256024	112	101	147	93
ARHGAP22__NM_001256024_CAGCACAACAGACGTGCACA	ARHGAP22__NM_001256024	645	612	713	806
ARHGAP22__NM_001256024_GAGGCAAGGGTTCTAGAGG(	ARHGAP22__NM_001256024	139	100	119	227
ARHGAP22__NM_001256024_GAGGGGCTGTTCCGCATGCC	ARHGAP22__NM_001256024	986	1311	1091	1583
ARHGAP22__NM_001256024_GAGGGTGGGCATGCGATCTA	ARHGAP22__NM_001256024	185	165	219	218
ARHGAP22__NM_001256024_GCAGGATTCCTTCGACTGTG	ARHGAP22__NM_001256024	668	881	843	941
ARHGAP22__NM_001256024_GCTGAGCCCAAAGATCAGGC	ARHGAP22__NM_001256024	64	39	72	106
ARHGAP22__NM_001256024_GCTGAGGAAGTCCTCGTACC	ARHGAP22__NM_001256024	870	875	953	923
ARHGAP22__NM_001256024_GGTTGTAATTTGCCTGAGGA	ARHGAP22__NM_001256024	348	465	285	494
ARHGAP22__NM_001256024_TGCAGGATTCCTTCGACTGT	ARHGAP22__NM_001256024	650	412	833	715
ARHGAP22__NM_001256025_CGGAAGGAGGACTTCCAACT	ARHGAP22__NM_001256025	986	1071	1129	1023
ARHGAP22__NM_001256025_GCTGGCTGTGTTTGCGGATG	ARHGAP22__NM_001256025	1725	2494	1961	2338
ARHGAP22__NM_001256025_TACCTTCCATGATGGTTACT	ARHGAP22__NM_001256025	582	605	686	797
ARHGAP22__NM_001256025_TGCGGATGGAGGACGGTCATC	ARHGAP22__NM_001256025	326	309	417	376
ARHGAP22__NM_001256026_AGAGGAGCAGGCGTATGCCC	ARHGAP22__NM_001256026	139	72	164	89
ARHGAP22__NM_001256026_GAGGAGCAGGCGTATGCCCA	ARHGAP22__NM_001256026	122	103	114	85
ARHGAP23__NM_001199417_ACGGCCCTGCCCATAGGGCG	ARHGAP23__NM_001199417	133	132	127	266
ARHGAP23__NM_001199417_AGGCGCCCCTTCCCCTGGCA	ARHGAP23__NM_001199417	1980	1587	1932	1892
ARHGAP23__NM_001199417_ATGGGCTCCAGGCGGTACCG	ARHGAP23__NM_001199417	245	284	247	224
ARHGAP23__NM_001199417_CAGCGCCTCCCAGGACCGGT	ARHGAP23__NM_001199417	1286	1314	1292	1661
ARHGAP23__NM_001199417_CAGGAGGGGCGTAGGTCTTG	ARHGAP23__NM_001199417	43	59	39	85
ARHGAP23__NM_001199417_CATAGGGCGGGGCTTCGCAC	ARHGAP23__NM_001199417	523	546	600	720
ARHGAP23__NM_001199417_CCAGGATGCTTTGAGCCAGC	ARHGAP23__NM_001199417	450	584	524	661
ARHGAP23__NM_001199417_TCATAGCTCTGATCCAGAAT	ARHGAP23__NM_001199417	0	0	0	0
ARHGAP23__NM_001199417_TCCCCAAGGAAGAGTTGTCC	ARHGAP23__NM_001199417	285	223	282	287
ARHGAP23__NM_001199417_TGAATGGGGAAAGCGTCATT	ARHGAP23__NM_001199417	409	421	442	334
ARHGAP23__NM_001199417_TGTGCGAAGCCCCGCCCTAT	ARHGAP23__NM_001199417	1503	1054	1592	1137
ARHGAP23__NM_001199417_TTGCGGGGGTAGCAGATCGG	ARHGAP23__NM_001199417	639	674	682	820
ARHGAP24__NM_001025616_AAGGTCCCCATATGACTCGG	ARHGAP24__NM_001025616	369	290	296	189
ARHGAP24__NM_001025616_AGTCAATCCGCCGAGTCATA	ARHGAP24__NM_001025616	327	273	465	515
ARHGAP24__NM_001025616_CCCAGTCTTCCATATCATTC	ARHGAP24__NM_001025616	1074	1552	1147	1531
ARHGAP24__NM_001025616_CCTCAGCCACCCACACTTGA	ARHGAP24__NM_001025616	682	899	698	811
ARHGAP24__NM_001025616_CGAAAGGTCCCCATATGACT	ARHGAP24__NM_001025616	1127	1125	1272	1075
ARHGAP24__NM_001025616_GAACCGTCTGGCTCCGATGT	ARHGAP24__NM_001025616	1665	2187	1944	1885
ARHGAP24__NM_001025616_GTCAATCCGCCGAGTCATAT	ARHGAP24__NM_001025616	525	467	605	633
ARHGAP24__NM_001025616_GTCATATGGGGACCTTTCGG	ARHGAP24__NM_001025616	498	337	519	378
ARHGAP24__NM_001025616_TCAAGACTTGGCATACTCGC	ARHGAP24__NM_001025616	143	220	157	225
ARHGAP24__NM_001025616_TCAATCCGCCGAGTCATATG	ARHGAP24__NM_001025616	526	727	549	493
ARHGAP24__NM_001025616_TCACAGGAGGCGATCGAGAT	ARHGAP24__NM_001025616	301	386	294	544
ARHGAP24__NM_001025616_TCTGGGTGCTTGCCATGAGG	ARHGAP24__NM_001025616	391	339	439	493
ARHGAP24__NM_001042669_CGTGGACTTTATCCGACAAA	ARHGAP24__NM_001042669	272	389	303	363
ARHGAP24__NM_001042669_GCGTGGACTTTATCCGACAA	ARHGAP24__NM_001042669	312	512	341	563
ARHGAP24__NM_001042669_GTGGACTTTATCCGACAAAG	ARHGAP24__NM_001042669	5	11	11	29
ARHGAP24__NM_031305_AAATCTTCATACTTCGCATA	ARHGAP24__NM_031305	85	30	115	185
ARHGAP24__NM_031305_AGGAGGTTGTAATTTACCAC	ARHGAP24__NM_031305	328	253	223	277
ARHGAP24__NM_031305_ATGCCTGAAGACCGGAATTC	ARHGAP24__NM_031305	450	514	617	553
ARHGAP24__NM_031305_GAGGGTCTCTTTCGACTGCC	ARHGAP24__NM_031305	442	601	638	985
ARHGAP24__NM_031305_GCCTGAAGACCGGAATTCTG	ARHGAP24__NM_031305	792	757	805	836
ARHGAP24__NM_031305_TACTTCGCATAAGGAATAAC	ARHGAP24__NM_031305	323	505	454	499
ARHGAP24__NM_031305_TCCAAGATGCCTTTGACTGT	ARHGAP24__NM_031305	854	1338	1028	1113
ARHGAP24__NM_031305_TGTTAAGGAATTAGCAAAGC	ARHGAP24__NM_031305	17	12	35	38
ARHGAP25__NM_001007231_AAAGTGGAGGCTGCGAAAAT	ARHGAP25__NM_001007231	978	1248	1129	1072
ARHGAP25__NM_001007231_AGTCCTGTCCCATGCGATTC	ARHGAP25__NM_001007231	644	464	607	872
ARHGAP25__NM_001007231_ATCTCCTTGATTGTACATCC	ARHGAP25__NM_001007231	322	344	415	317
ARHGAP25__NM_001007231_ATGGGCCTCTCCAGCGGGTT	ARHGAP25__NM_001007231	727	746	908	970
ARHGAP25__NM_001007231_CAGGGCTGCATGTATCTACC	ARHGAP25__NM_001007231	838	1082	831	827
ARHGAP25__NM_001007231_CCACAGTCTCATCCAAGCGC	ARHGAP25__NM_001007231	594	780	750	716
ARHGAP25__NM_001007231_CTCATGGGACCAGAATCGCA	ARHGAP25__NM_001007231	212	175	260	250
ARHGAP25__NM_001007231_CTGGAGAGGCCCATCAAGAT	ARHGAP25__NM_001007231	266	180	230	212
ARHGAP25__NM_001007231_GGACCAGAATCGCATGGGAC	ARHGAP25__NM_001007231	1115	1202	1192	1426
ARHGAP25__NM_001007231_GGAGGCTGCGAAAATAGGTA	ARHGAP25__NM_001007231	297	458	313	261
ARHGAP25__NM_001007231_GGGGTGGACGATGGATGG	ARHGAP25__NM_001007231	119	9	146	30
ARHGAP25__NM_001007231_TCATGGGACCAGAATCGCAT	ARHGAP25__NM_001007231	636	639	686	565
ARHGAP25__NM_001166276_AGCTATAGAGAGGAACAGAC	ARHGAP25__NM_001166276	260	304	294	262
ARHGAP25__NM_001166276_CAGACAGGCCGACTGACCGA	ARHGAP25__NM_001166276	1837	2403	2219	2129
ARHGAP25__NM_001166276_CCCCATGTCCCTCGGTCAGT	ARHGAP25__NM_001166276	2148	2558	2604	2920
ARHGAP25__NM_001166276_GGCCGACTGACCGAGGGACA	ARHGAP25__NM_001166276	376	468	439	490
ARHGAP25__NM_001166276_GTCTGTTCCTCTCTATAGCT	ARHGAP25__NM_001166276	63	16	34	80
ARHGAP25__NM_001166276_TCCTTGACCGAGCTATAGAG	ARHGAP25__NM_001166276	239	290	232	223
ARHGAP25__NM_001166277_ACCAGGATGGGCACCAGATG	ARHGAP25__NM_001166277	108	95	179	160
ARHGAP25__NM_001166277_GAGGGCATCTTCCGTCTGCC	ARHGAP25__NM_001166277	972	1420	1120	1661
ARHGAP25__NM_001166277_GTGGCCTATGAACAGAAATT	ARHGAP25__NM_001166277	51	32	77	19
ARHGAP25__NM_001166277_TCGGAGGTAGAGCTTTAACA	ARHGAP25__NM_001166277	620	521	683	530
ARHGAP26__NM_001135608_ACAGATGATGAGATGTGTAT	ARHGAP26__NM_001135608	312	579	351	313
ARHGAP26__NM_001135608_ACTCACGTTCTGTATGCTAA	ARHGAP26__NM_001135608	658	969	725	1047
[illegible]	[illegible]	[illegible]	[illegible]	[illegible]	[illegible]
ARHGAP30__NM_001025598_CAGAGGATAAATCCAACAAG	ARHGAP30__NM_001025598	4	0	6	17
ARHGAP30__NM_001025598_CGCTGTCCCATTGAAGCCTG	ARHGAP30__NM_001025598	754	924	807	1162
ARHGAP30__NM_001025598_CTAAGCGTAAACTTCCACGG	ARHGAP30__NM_001025598	291	130	237	98
ARHGAP30__NM_001025598_CTCTCAGGCAGCAATGGGCT	ARHGAP30__NM_001025598	31	49	43	44
ARHGAP30__NM_001025598_CTGCAATCTCGATGATAGTA	ARHGAP30__NM_001025598	300	527	426	333
[illegible]	[illegible]	[illegible]	[illegible]	[illegible]	[illegible]
ARHGAP35__NM_004491_CCATCGCCTAGCGTTTGGGG	ARHGAP35__NM_004491	1365	1147	1614	1355
ARHGAP35__NM_004491_CCCAAGGTAAGGGTACAGCC	ARHGAP35__NM_004491	180	104	250	243
ARHGAP35__NM_004491_CCCCCCAGGCGAGAGCTAGT	ARHGAP35__NM_004491	1572	1952	1663	1585
ARHGAP35__NM_004491_CCGGCCTTCAGCGTCCGGTC	ARHGAP35__NM_004491	176	355	185	205
ARHGAP35__NM_004491_CCGTGGCCTACACGTTCCCA	ARHGAP35__NM_004491	705	660	977	585
[illegible]	[illegible]	[illegible]	[illegible]	[illegible]	[illegible]
ARHGAP40__NM_001164431_GAATGGCGACTCCGGTATGA	ARHGAP40__NM_001164431	1349	1293	1539	1285
ARHGAP40__NM_001164431_GAGGAATGTTTCCACCGTGA	ARHGAP40__NM_001164431	363	185	391	257
ARHGAP40__NM_001164431_GGGGGCCCAGCTCAGTTCCG	ARHGAP40__NM_001164431	578	577	734	641
ARHGAP40__NM_001164431_GTCTTTGGGGTCTTCAATTC	ARHGAP40__NM_001164431	52	34	66	82
ARHGAP40__NM_001164431_TCGTCAGAGAATGGCGACTC	ARHGAP40__NM_001164431	308	269	375	416
[illegible]	[illegible]	[illegible]	[illegible]	[illegible]	[illegible]

ARL14EP__NM_152316_TACAATTTACGAATCCAGGA	ARL14EP__NM_152316	73	35	76	86
ARL14EP__NM_152316_TCATTACAATTTACGAATCC	ARL14EP__NM_152316	301	273	354	390
ARL14EPL__NM_001195581_AAACCCACCGTCGGTTGCAG	ARL14EPL__NM_001195581	191	163	221	255
ARL14EPL__NM_001195581_AGAGCGGCAGTTAAAATGCT	ARL14EPL__NM_001195581	568	748	682	793
ARL14EPL__NM_001195581_AGTGCCGCTGCAACCGACGG	ARL14EPL__NM_001195581	951	921	1033	943
ARL14EPL__NM_001195581_AGTGTAACTCCAACAAGTGT	ARL14EPL__NM_001195581	0	0	0	0
ARL14EPL__NM_001195581_ATGAGGAAGTATGACAAAAG	ARL14EPL__NM_001195581	205	291	281	181
ARL14EPL__NM_001195581_CCGAGTGCCGCTGCAACCGA	ARL14EPL__NM_001195581	1315	1872	1536	1811
ARL14EPL__NM_001195581_CGTCGGTTGCAGCGGCACTC	ARL14EPL__NM_001195581	1163	1174	1358	1030
ARL14EPL__NM_001195581_GGAAGTATGACAAAAGTGGC	ARL14EPL__NM_001195581	244	415	220	352
ARL14EPL__NM_001195581_TGCTTGGCATTTCGAAACCC	ARL14EPL__NM_001195581	184	134	227	212
ARL14EPL__NM_001195581_TGGAGTTACACTTCGGGCAT	ARL14EPL__NM_001195581	58	195	46	22
ARL14EPL__NM_001195581_TGGCATCGTAAACCCACCGT	ARL14EPL__NM_001195581	104	62	63	78
ARL15__NM_019087_AACGTCGTGTCGACCACAGG	ARL15__NM_019087	349	482	407	489
ARL15__NM_019087_ATGCAAACCAGGTCATATTC	ARL15__NM_019087	312	219	284	312
ARL15__NM_019087_CACTTGCACGTGGAAAACGC	ARL15__NM_019087	464	468	366	635
ARL15__NM_019087_GATAACGTCGTGTCGACCAC	ARL15__NM_019087	321	231	380	302
ARL15__NM_019087_GTGGTCGACACGACGTTATC	ARL15__NM_019087	34	27	21	6
ARL15__NM_019087_TGCACGACCAGAATATGACC	ARL15__NM_019087	189	209	209	184
ARL15__NM_019087_TGCTGGCTTGTCTTGATGAT	ARL15__NM_019087	671	813	777	1056
ARL15__NM_019087_TGGTCGACACGACGTTATCG	ARL15__NM_019087	10	3	31	23
ARL15__NM_019087_TGTGGTCGACACGACGTTAT	ARL15__NM_019087	319	258	231	220
ARL16__NM_001040025_ATTCCGTGCTTCGCTCCACC	ARL16__NM_001040025	495	298	358	256
ARL16__NM_001040025_CCGTGGACATGTAACAGGGT	ARL16__NM_001040025	876	537	950	453
ARL16__NM_001040025_CGGGATGGGAAAGGCGACCT	ARL16__NM_001040025	897	499	907	597
ARL16__NM_001040025_CGTGAAGGCACTGGCTTAGC	ARL16__NM_001040025	743	616	783	651
ARL16__NM_001040025_CTCCTCCGTGGACATGTAAC	ARL16__NM_001040025	554	248	681	349
ARL16__NM_001040025_TCCACCCGGCACCCGTAGCT	ARL16__NM_001040025	512	359	410	421
ARL16__NM_001040025_TCCTCCGTGGACATGTAACA	ARL16__NM_001040025	395	156	336	196
ARL16__NM_001040025_TGCTAAGCCAGTGCCTTCAC	ARL16__NM_001040025	680	317	739	516
ARL2__NM_001199745_AGTTCCGCCAGTAGGACCGC	ARL2__NM_001199745	634	612	476	217
ARL2__NM_001199745_CAGATGTTCAGCTTGAATCT	ARL2__NM_001199745	290	7	323	21
ARL2__NM_001199745_CCAGATGTTCAGCTTGAATC	ARL2__NM_001199745	380	504	442	727
ARL2__NM_001199745_CCTGAAGAAGTTCAATGGGG	ARL2__NM_001199745	532	1	754	2
ARL2__NM_001199745_GTTCCGCCAGTAGGACCGCA	ARL2__NM_001199745	245	3	254	18
ARL2__NM_001199745_TCAAGACCCTGGAGCACCGA	ARL2__NM_001199745	667	76	675	50
ARL2__NM_001199745_TGCGGCTGGAAATGTCATCC	ARL2__NM_001199745	651	296	876	255

[illegible]

ASIC3__NM_004769_CGTTGGGCGTTAGGCGCGAG	ASIC3__NM_004769	609	733	516	615
ASIC3__NM_004769_CTGCTCACCACTACTAGGGG	ASIC3__NM_004769	226	372	241	271
ASIC3__NM_004769_CTGCTGGACTGTCGCTTCCG	ASIC3__NM_004769	606	474	547	549
ASIC3__NM_004769_GAGCTGCTCACCACTACTAG	ASIC3__NM_004769	946	1069	1037	935
ASIC3__NM_004769_GGCTGCCTGGCCCGAAGACG	ASIC3__NM_004769	467	767	568	625
ASIC3__NM_004769_TGAGAGGGTGCGCTACTACA	ASIC3__NM_004769	992	1073	1073	1006
ASIC3__NM_004769_TTCCCATCCGGGTGAAGATC	ASIC3__NM_004769	91	76	124	153
ASIC4__NM_182847_AAGCCACAAGGAGACGATCG	ASIC4__NM_182847	359	151	401	426
ASIC4__NM_182847_ACAGCCGGGAAGCCCGCCAC	ASIC4__NM_182847	1655	1994	1811	1968
ASIC4__NM_182847_ACTGGCCCTACTCACCTCGC	ASIC4__NM_182847	2777	3064	3427	3621
ASIC4__NM_182847_AGCTGTGCTGGTGCTGATAA	ASIC4__NM_182847	477	316	481	469
ASIC4__NM_182847_AGGCAGCCAGCGAGGTGAGT	ASIC4__NM_182847	478	327	495	439
ASIC4__NM_182847_AGGGTTCTGCGCAGTCCGTG	ASIC4__NM_182847	185	184	196	213
ASIC4__NM_182847_CAGCCGGGAAGCCCGCCACT	ASIC4__NM_182847	1143	785	1185	884
ASIC4__NM_182847_CATCGCTGAGTGCCGAATGC	ASIC4__NM_182847	1159	972	1221	1477
ASIC4__NM_182847_GCCACCACTGCCACTCGGGA	ASIC4__NM_182847	2401	2636	2717	2440
ASIC4__NM_182847_GCGGCTGCTCTTCTAAACTC	ASIC4__NM_182847	207	253	248	406
ASIC4__NM_182847_GGCAGCCAGCGAGGTGAGTA	ASIC4__NM_182847	148	290	144	228
ASIC4__NM_182847_TGAGTGCCGAATGCCGGAAG	ASIC4__NM_182847	371	400	279	340
ASIC5__NM_017419_AATATTGTGTATCCCATGAA	ASIC5__NM_017419	98	30	116	162
ASIC5__NM_017419_ACCTCAATGGACGTTGTGGT	ASIC5__NM_017419	1537	1924	1582	2262
ASIC5__NM_017419_AGAACCGGAGCAAAATTCGC	ASIC5__NM_017419	886	1588	955	1207
ASIC5__NM_017419_ATGGCAGTGGTTTCTTTGAA	ASIC5__NM_017419	325	389	387	506
ASIC5__NM_017419_CAATTCCACTGGCTCTAGAG	ASIC5__NM_017419	356	384	465	508
ASIC5__NM_017419_GCCAACCACAACGTCCATTG	ASIC5__NM_017419	1450	1204	1571	1987
ASIC5__NM_017419_GCTCAGTCTCACTTGTGACA	ASIC5__NM_017419	1232	970	1267	1309
ASIC5__NM_017419_GCTTGCTCAACTACTTCACA	ASIC5__NM_017419	1630	1799	1849	2517
ASIC5__NM_017419_TCAAACTTCTTTCGCTCAGT	ASIC5__NM_017419	659	899	651	948
ASIC5__NM_017419_TCTCAACAATAGCACTTTGT	ASIC5__NM_017419	544	570	606	586
ASIC5__NM_017419_TGGAAAGCCATGTAGCCCAA	ASIC5__NM_017419	182	337	254	181
ASIC5__NM_017419_TTGAACCTCAATGGACGTTG	ASIC5__NM_017419	285	541	279	380
ASIP__NM_001672_AGGGCGCAGATAGGGGGGTC	ASIP__NM_001672	1137	1276	1197	1254
ASIP__NM_001672_CAGGGTGGCCAGGAGTAAGC	ASIP__NM_001672	100	122	77	43
ASIP__NM_001672_GTCCCTTCTGTCTCTATTGT	ASIP__NM_001672	1082	891	932	1000
ASIP__NM_001672_TGTCCCTTCTGTCTCTATTG	ASIP__NM_001672	253	231	353	303
ASL__NM_000048_AAGGTGCCGGTCGTAGGCAA	ASL__NM_000048	242	233	325	269
ASL__NM_000048_ACGCGTCCATTGCCTACGAC	ASL__NM_000048	774	849	791	1106
ASL__NM_000048_ACTAGTGAGCGGGACTTTGT	ASL__NM_000048	1117	1402	1241	1272
ASL__NM_000048_ATCCAGTGGCTCCAGCGGAT	ASL__NM_000048	309	175	258	239
ASL__NM_000048_CACAAAGTCCCGCTCACTAG	ASL__NM_000048	879	1000	864	721
ASL__NM_000048_CAGGGAAGCTGCACACGGGA	ASL__NM_000048	328	477	354	501
ASL__NM_000048_CGGATCAGCTCCAAACTGTC	ASL__NM_000048	479	477	482	659
ASL__NM_000048_CTCGGCCATCCTGCTGAGAT	ASL__NM_000048	552	650	740	954
ASL__NM_000048_CTCGGGTCAGTGCCACGGCG	ASL__NM_000048	407	378	435	494
ASL__NM_000048_CTCTGCAAATGGGTGTACCC	ASL__NM_000048	4	12	14	15
ASL__NM_000048_GAAGAAAAACCCCGACAGTT	ASL__NM_000048	356	364	488	281
ASL__NM_000048_GATACTCCATGGCCTAGACA	ASL__NM_000048	335	511	491	505
ASL__NM_001024946_GGGCTCCTGATGACCCTCAA	ASL__NM_001024946	241	544	269	338
ASNA1__NM_004317_AAAGTCCCCCCCATAATACA	ASNA1__NM_004317	260	206	288	178
ASNA1__NM_004317_AGCCTGAAGTGGATCTTCGT	ASNA1__NM_004317	1473	721	1666	830
ASNA1__NM_004317_AGTGGCTCCCCCTGTATTAT	ASNA1__NM_004317	1143	1029	1197	966
ASNA1__NM_004317_CAAAGTCCCCCCCATAATAC	ASNA1__NM_004317	1106	849	1079	849
ASNA1__NM_004317_CTCAGTCAGCGAACAGTTCA	ASNA1__NM_004317	1125	1703	1332	1619
ASNA1__NM_004317_CTCATGGCCTCATCGATGCC	ASNA1__NM_004317	422	377	391	625
ASNA1__NM_004317_GAGGACAACATGCTGAGCAT	ASNA1__NM_004317	45	25	17	13
ASNA1__NM_004317_GATCAGCCCTTTCATCTCAC	ASNA1__NM_004317	1247	115	1240	372
ASNA1__NM_004317_GATGCCGGGAAATGCGCTCA	ASNA1__NM_004317	1934	747	2103	997
ASNA1__NM_004317_GCCTGAAGTGGATCTTCGTC	ASNA1__NM_004317	1916	2477	1956	2624
ASNA1__NM_004317_GCTCCCCCTGTATTATGGGG	ASNA1__NM_004317	158	87	127	131
ASNA1__NM_004317_GTGGCTCCCCCTGTATTATG	ASNA1__NM_004317	80	30	93	46
ASNA1__NM_004317_TATGCCGAGGTCATGAGGTC	ASNA1__NM_004317	1038	1077	1023	1260
ASNA1__NM_004317_TCCTCCTCGAAGAACTCGTC	ASNA1__NM_004317	158	47	149	67
ASNA1__NM_004317_TGAGGCCATGAGCTATGCCG	ASNA1__NM_004317	478	332	547	267
ASNA1__NM_004317_TGAGGCCCGTCACAAGATCC	ASNA1__NM_004317	951	134	984	256
ASNA1__NM_004317_TTGCTAAGTGTAGGCTCCAG	ASNA1__NM_004317	390	280	465	515
ASNS__NM_001178075_AAATGGCAAAGTTGCATCCG	ASNS__NM_001178075	508	101	584	203
ASNS__NM_001178075_CAAAGTTGCATCCGTGGAAA	ASNS__NM_001178075	369	573	431	497
ASNS__NM_001178075_CTGCTTTGGATTTCACCGGT	ASNS__NM_001178075	948	335	1248	500
ASNS__NM_001178075_GAATACCAGACCAAAGTGGA	ASNS__NM_001178075	281	25	304	20
ASNS__NM_001178075_GCGGGTCAACTACCGCCAAC	ASNS__NM_001178075	29	0	58	12
ASNS__NM_001178075_GCGGTAGTTGACCCGCTGTT	ASNS__NM_001178075	1250	1369	1320	1694
ASNS__NM_001178075_GCTGTATGTTCAGAAGCTAA	ASNS__NM_001178075	315	18	366	30
ASNS__NM_001178075_GGATATTTCTTCACTCGAAT	ASNS__NM_001178075	250	207	230	308
ASNS__NM_001178075_TATTTGTGGCTCTGTTACAA	ASNS__NM_001178075	64	6	82	41
ASNS__NM_001178075_TCTACAACCATAAGAAGGTG	ASNS__NM_001178075	0	0	2	0
ASNS__NM_001178075_TGAGCAAACAATTTGTATGT	ASNS__NM_001178075	508	69	526	83
ASNS__NM_001178075_TTCTCAAAACGGAATGCATC	ASNS__NM_001178075	0	0	0	0
ASNS__NM_001178076_CCAATTGCAAATGTCTGGAG	ASNS__NM_001178076	699	288	755	449
ASNS__NM_001178076_CCATGCCAATTGCAAATGTC	ASNS__NM_001178076	325	327	448	382
ASNS__NM_001178076_CCTCTCCAGACATTTGCAAT	ASNS__NM_001178076	613	647	733	862
ASNS__NM_001178076_CTGGAGAGGATACTGTACTT	ASNS__NM_001178076	502	85	686	92
ASNS__NM_001178076_GGAAGACAGCCCCGATTTAC	ASNS__NM_001178076	380	34	416	81
ASNS__NM_001178076_TTCTAGCAGCCAGTAAATCG	ASNS__NM_001178076	4	0	0	0
ASNS__NM_001178076_TTTCTAGCAGCCAGTAAATC	ASNS__NM_001178076	43	55	70	112
ASNS__NM_001673_CACTCAGACACTGAACAGAA	ASNS__NM_001673	311	25	346	86
ASNSD1__NM_019048_ACGTGTCTTGTGTTTACCTA	ASNSD1__NM_019048	1518	1656	1822	1721
ASNSD1__NM_019048_AGATCAATTGGTTCATCTAA	ASNSD1__NM_019048	241	146	201	308
ASNSD1__NM_019048_AGGAATATGACGGTCAGCAA	ASNSD1__NM_019048	94	32	150	103
ASNSD1__NM_019048_CTATATAATCTTAAACAGCG	ASNSD1__NM_019048	96	91	65	79
ASNSD1__NM_019048_CTCAGATTCATTCTTACAGG	ASNSD1__NM_019048	149	209	219	287
ASNSD1__NM_019048_GCTACCACTGATACAAATGC	ASNSD1__NM_019048	554	655	680	728
ASNSD1__NM_019048_GTCATTATTTATGGTTTGGT	ASNSD1__NM_019048	323	477	398	489
ASNSD1__NM_019048_GTTTCTTGACTGCTACACTC	ASNSD1__NM_019048	171	139	177	240
ASNSD1__NM_019048_TCAAAACTTCATTTGCTGTC	ASNSD1__NM_019048	1594	2446	1843	2334

[illegible]

ATAD1__NM_032810_CGGTCAGTGTCGAAGGCTGA	ATAD1__NM_032810	419	494	472	621
ATAD1__NM_032810_CTATATCACTCCAAGTAACC	ATAD1__NM_032810	141	175	157	123
ATAD1__NM_032810_CTTCGACACTGACCGATAAG	ATAD1__NM_032810	267	175	280	251
ATAD1__NM_032810_GATGGATGGTTGTAGCTTTA	ATAD1__NM_032810	235	253	241	212
ATAD2__NM_014109_AAAAGCTACTGTTTACTATC	ATAD2__NM_014109	363	520	451	423

[illegible]

ATP5F1__NM_001688_GTACCACCTCTTCCTGAATA	ATP5F1__NM_001688	423	23	448	39
ATP5F1__NM_001688_TACGGAGGAAAAGTTCGTTA	ATP5F1__NM_001688	333	64	349	41
ATP5F1__NM_001688_TAGTACTGATAGGGCAGTGA	ATP5F1__NM_001688	351	176	380	146
ATP5G1__NM_001002027_AAGAGCTGCTGCTTGAGAGA	ATP5G1__NM_001002027	544	202	583	330
ATP5G1__NM_001002027_ACAGATCCGCTGTTGTACCA	ATP5G1__NM_001002027	1369	988	1268	975
ATP5G1__NM_001002027_ACTGGTCTGGAACTCCCGTC	ATP5G1__NM_001002027	1011	847	976	731
ATP5G1__NM_001002027_AGAAATGAATAATGCCCCGG	ATP5G1__NM_001002027	309	396	461	296
ATP5G1__NM_001002027_AGCCAAGTTTATTGGTGCTG	ATP5G1__NM_001002027	536	561	686	580
ATP5G1__NM_001002027_CTCATCCTCTTCGCCATGTG	ATP5G1__NM_001002027	163	209	193	250
ATP5G1__NM_001002027_GTTGTACCAGGGGTCTAATC	ATP5G1__NM_001002027	75	9	95	1
ATP5G2__NM_001002031_AGCCCCAGCTCCAATGAACT	ATP5G2__NM_001002031	19	4	21	2
ATP5G2__NM_001002031_CAGCCAAGTTCATTGGAGCT	ATP5G2__NM_001002031	577	753	792	740
ATP5G2__NM_001002031_CCAAACCAGCGCCATTTCAA	ATP5G2__NM_001002031	2046	1806	2416	1571
ATP5G2__NM_001002031_CTAGAGACAAGTGAGGTAAG	ATP5G2__NM_001002031	132	62	132	71
ATP5G2__NM_001002031_GATGTCCCTTGAAATGGCGC	ATP5G2__NM_001002031	479	707	491	617
ATP5G2__NM_001002031_GCTCTTCTCCTACGCCATTC	ATP5G2__NM_001002031	364	586	396	542
ATP5G2__NM_001002031_GGCTAGAGACAAGTGAGGTA	ATP5G2__NM_001002031	399	351	413	319
ATP5G2__NM_001002031_GTGTTTGGGAGCCTCATCAT	ATP5G2__NM_001002031	383	460	402	146
ATP5G2__NM_001002031_TCCAAACCAGCGCCATTTCA	ATP5G2__NM_001002031	913	985	1104	1121
ATP5G2__NM_001002031_TGCAGTGGTGCTGAAACGAC	ATP5G2__NM_001002031	584	491	427	283
ATP5G2__NM_005176_CAGCGGGAAGAGCGAAAGGA	ATP5G2__NM_005176	278	515	273	333
ATP5G2__NM_005176_GCCAACGAGTCGCTCAGCCA	ATP5G2__NM_005176	1135	1233	1115	618
ATP5G2__NM_005176_GCTCAGCGCATGCGTGACCC	ATP5G2__NM_005176	371	249	416	371
ATP5G2__NM_005176_GGCTGAGCGACTCGTTGGCC	ATP5G2__NM_005176	1254	1577	1407	1949
ATP5G2__NM_005176_TCCGTGGCTGAGCGACTCGT	ATP5G2__NM_005176	860	682	1057	695
ATP5G2__NM_005176_TGTTGCAATCACTCTATCCG	ATP5G2__NM_005176	589	559	571	393
ATP5G3__NM_001002258_AATGTCTCTGCTGATTGCAC	ATP5G3__NM_001002258	304	411	385	345
ATP5G3__NM_001002258_AATTGGTCTGTATGCAACTC	ATP5G3__NM_001002258	335	359	337	390
ATP5G3__NM_001002258_AGGGCTCTACGGTATTTAAT	ATP5G3__NM_001002258	58	30	74	101
ATP5G3__NM_001002258_ATCAGTGTTATCTCGACCAG	ATP5G3__NM_001002258	409	390	486	431
ATP5G3__NM_001002258_CAGGGCTCTACGGTATTTAA	ATP5G3__NM_001002258	506	638	613	648
ATP5G3__NM_001002258_CGACCAGAGGCTAGTAGGAC	ATP5G3__NM_001002258	873	960	1089	1410
ATP5G3__NM_001002258_GATACTGCTGCCAAATTTAT	ATP5G3__NM_001002258	435	598	691	647
ATP5G3__NM_001002258_GCTGTTCTCATATGCTATCC	ATP5G3__NM_001002258	252	365	314	401
ATP5G3__NM_001002258_GTCTTTGGCAGCCTTATCAT	ATP5G3__NM_001002258	355	310	431	426
ATP5G3__NM_001002258_GTGTGTCTCAGCTAATCCAA	ATP5G3__NM_001002258	46	34	72	85
ATP5G3__NM_001002258_TATCTCGACCAGAGGCTAGT	ATP5G3__NM_001002258	442	358	460	494
ATP5H__NM_001003785_ACAAGAAAAAGTATCCCTAT	ATP5H__NM_001003785	556	127	732	387
ATP5H__NM_001003785_AGAATCCACCAGCTATCGAC	ATP5H__NM_001003785	393	94	425	125
ATP5H__NM_001003785_ATTTGATCAGATGACCATTG	ATP5H__NM_001003785	236	119	211	70
ATP5H__NM_001003785_GAATCCACCAGCTATCGACT	ATP5H__NM_001003785	607	66	500	53
ATP5H__NM_001003785_TAAGCCCAGTCGATAGCTGG	ATP5H__NM_001003785	136	6	89	16
ATP5H__NM_001003785_TAGTAAGCCCAGTCGATAGC	ATP5H__NM_001003785	82	27	86	11
ATP5H__NM_001003785_TATCGACTGGGCTTACTACA	ATP5H__NM_001003785	564	242	572	145
ATP5H__NM_001003785_TCAATGGTCATCTGATCAAA	ATP5H__NM_001003785	264	129	256	107
ATP5H__NM_001003785_TGCAAAAGCTACCCAGTCAA	ATP5H__NM_001003785	314	505	329	228
ATP5H__NM_001003785_TTGCTCTAAAAACCATTGAC	ATP5H__NM_001003785	8	2	12	31
ATP5H__NM_001003785_TTTCTCATATTCTACAATCC	ATP5H__NM_001003785	46	50	79	66
ATP5I__NM_007100_ACTGTAGCGCGTGGCTCCGT	ATP5I__NM_007100	2035	901	2233	1029
ATP5I__NM_007100_GCAGGTCTCTCCGCTCATCA	ATP5I__NM_007100	961	544	1237	344
ATP5J__NM_001003696_CACCAATGTTCCTCCGCAAA	ATP5J__NM_001003696	1254	728	1377	620
ATP5J__NM_001003696_GACGGCTGACCGAATGACAG	ATP5J__NM_001003696	578	306	514	379
ATP5J__NM_001003696_TACAGCAGTGGCATTTAATA	ATP5J__NM_001003696	173	91	199	80
ATP5J__NM_001003696_TCCACAAAGAGTTTCTGTAT	ATP5J__NM_001003696	612	471	571	235
ATP5J__NM_001003701_AATGCATTGCGATGGCGGGT	ATP5J__NM_001003701	775	944	893	977
ATP5J__NM_001003701_CCATAATGCATTGCGATGGC	ATP5J__NM_001003701	781	729	712	897
ATP5J__NM_001003701_CCCATAATGCATTGCGATGG	ATP5J__NM_001003701	131	116	170	213
ATP5J__NM_001003701_CCCGCCATCGCAATGCATTA	ATP5J__NM_001003701	1342	1842	1693	2413
ATP5J2__NM_001003713_ACTGAGATGCTTGTAGGAAA	ATP5J2__NM_001003713	190	151	282	227
ATP5J2__NM_001003713_CTCGGGGATTACCATGGTGC	ATP5J2__NM_001003713	474	42	489	57
ATP5J2-PTCD1__NM_001198879_AAACCGCAGGTTATGGAA	ATP5J2-PTCD1__NM_001198879	583	246	698	274
ATP5J2-PTCD1__NM_001198879_ACCGTGTAGTTGCTCTCCA	ATP5J2-PTCD1__NM_001198879	3004	604	2832	496
ATP5J2-PTCD1__NM_001198879_AGCAACTACACGGTGCTG	ATP5J2-PTCD1__NM_001198879	728	161	656	166
ATP5J2-PTCD1__NM_001198879_CAAACCGCAGGTTATGGA	ATP5J2-PTCD1__NM_001198879	633	767	740	796
ATP5J2-PTCD1__NM_001198879_CCGGAGCCCAAATTATGG	ATP5J2-PTCD1__NM_001198879	458	399	590	492
ATP5J2-PTCD1__NM_001198879_CTCCGGCCTCGCCATAATT	ATP5J2-PTCD1__NM_001198879	548	568	566	396
ATP5J2-PTCD1__NM_001198879_GACTCGCTCGACTGTTCGC	ATP5J2-PTCD1__NM_001198879	2560	3846	2854	3363
ATP5J2-PTCD1__NM_001198879_GGAACTGGGCTGCGGATT	ATP5J2-PTCD1__NM_001198879	1076	796	1082	1013
ATP5J2-PTCD1__NM_001198879_GTTCCCCAAACCGCAGGTT	ATP5J2-PTCD1__NM_001198879	187	175	185	189
ATP5J2-PTCD1__NM_001198879_TCTCCGGCCTCGCCATAAT	ATP5J2-PTCD1__NM_001198879	803	729	933	991
ATP5J2-PTCD1__NM_001198879_TGGAACCGGAGCCCAAAT	ATP5J2-PTCD1__NM_001198879	62	33	63	30
ATP5J2-PTCD1__NM_001198879_TTTGCGGAATAGTCTCCGG	ATP5J2-PTCD1__NM_001198879	351	98	435	154
ATP5L__NM_006476_CACAAGGTTACGGACAAATT	ATP5L__NM_006476	8	0	5	1
ATP5L__NM_006476_CCACAAGGTTACGGACAAAT	ATP5L__NM_006476	93	191	151	114
ATP5L__NM_006476_CGGAGAGATTATAGGCAAGC	ATP5L__NM_006476	217	264	166	239
ATP5L__NM_006476_GGAGAGATTATAGGCAAGCG	ATP5L__NM_006476	190	249	222	267
ATP5L__NM_006476_GGGAGGAACCAGCTCAACCT	ATP5L__NM_006476	634	115	637	125
ATP5L2__NM_001165877_GTACTACACCACGGTTGAGC	ATP5L2__NM_001165877	263	130	228	264
ATP5L2__NM_001165877_TCCACAAGGTTACGGACAAA	ATP5L2__NM_001165877	50	15	82	92
ATP5L2__NM_001165877_TGGCCACTGAGGTGTCGACG	ATP5L2__NM_001165877	77	54	52	77
ATP5O__NM_001697_AAGCCTCCTGTTCAGGTATA	ATP5O__NM_001697	226	50	228	119
ATP5O__NM_001697_ACCATTTGCCAAGCTTGTGA	ATP5O__NM_001697	643	127	634	207
ATP5O__NM_001697_ATACCGTATACCTGAACAGG	ATP5O__NM_001697	347	130	313	170
ATP5O__NM_001697_CAAAACAGAAGCAGCCACTT	ATP5O__NM_001697	991	976	1062	1316
ATP5O__NM_001697_CTGATCAGATTGGTAGTGAG	ATP5O__NM_001697	495	625	537	751
ATP5O__NM_001697_GACCATTTGCCAAGCTTGTG	ATP5O__NM_001697	494	216	588	273
ATP5O__NM_001697_GTTCAGGTATACGGTATTGA	ATP5O__NM_001697	269	103	197	90
ATP5O__NM_001697_TCAATACCGTATACCTGAAC	ATP5O__NM_001697	520	254	672	437
ATP5O__NM_001697_TGCAGCAGAATAAAGAGCTG	ATP5O__NM_001697	171	6	193	48
ATP5O__NM_001697_TGCATCAAAACAGAATAAGC	ATP5O__NM_001697	141	96	146	42
ATP5O__NM_001697_TTAATGGAACGCTTCACATA	ATP5O__NM_001697	98	143	115	79
ATP5O__NM_001697_TTTAATGGAACGCTTCACAT	ATP5O__NM_001697	223	73	198	158
ATP5S__NM_001003803_AGAAGGCAGTGCTGTCTTAA	ATP5S__NM_001003803	618	691	769	791
ATP5S__NM_001003803_GAAATGGAAATAATATCCTG	ATP5S__NM_001003803	123	37	162	166
ATP5S__NM_001003803_GAATCTTGTATTTGTCCAGA	ATP5S__NM_001003803	182	272	261	351
ATP5S__NM_001003803_GACTCTTGTATCATGAGCAT	ATP5S__NM_001003803	562	581	685	537
ATP5S__NM_001003803_GCAGTGCTGTCTTAAAGGCT	ATP5S__NM_001003803	1330	1358	1426	1231
ATP5S__NM_001003803_GCTCATGATACAAGAGTCGG	ATP5S__NM_001003803	421	484	458	259
ATP5S__NM_001003803_TCATTGCTTTGCGTCATTTA	ATP5S__NM_001003803	915	1174	995	1225
ATP5S__NM_001003803_TTTGTCTGTGATATTCCCAC	ATP5S__NM_001003803	207	324	268	397
ATP5S__NM_015684_ACAGAAACAAGCTTAGTGAG	ATP5S__NM_015684	514	497	492	515
ATP5S__NM_015684_GAAACAAGCTTAGTGAGAGG	ATP5S__NM_015684	168	117	153	124
ATP5S__NM_015684_TGACTCACGATTATAGTCAT	ATP5S__NM_015684	561	703	612	634
ATP5SL__NM_001167867_AAGACCGAAGGATGAATGTA	ATP5SL__NM_001167867	209	415	300	369
ATP5SL__NM_001167867_ACATGGGGGCGACCAGGCGC	ATP5SL__NM_001167867	656	479	814	580
ATP5SL__NM_001167867_ATCAGGGGCATCCATCGCCT	ATP5SL__NM_001167867	421	348	517	308
ATP5SL__NM_001167867_CATCGTAGAAATAGTTGGTC	ATP5SL__NM_001167867	647	257	646	345
ATP5SL__NM_001167867_CATGTGGAATGGGCGTATCA	ATP5SL__NM_001167867	976	719	1199	827
ATP5SL__NM_001167867_CCATGTGGAATGGGCGTATC	ATP5SL__NM_001167867	815	801	1005	995
ATP5SL__NM_001167867_CCCACTGGCCGACTCGTTGC	ATP5SL__NM_001167867	399	491	437	608
ATP5SL__NM_001167867_CTCCACATCGTAGAAATAGT	ATP5SL__NM_001167867	341	159	402	172
ATP5SL__NM_001167867_CTGATACGCCCATTCCACAT	ATP5SL__NM_001167867	748	539	780	414
ATP5SL__NM_001167867_GAGAAATGGCCATACTTATC	ATP5SL__NM_001167867	528	327	662	249
ATP5SL__NM_001167867_GCCATTTCTCTCAGGAGTTC	ATP5SL__NM_001167867	315	272	398	499
ATP5SL__NM_001167867_TATCAGGGGCATCCATCGCC	ATP5SL__NM_001167867	1046	708	1180	717
ATP5SL__NM_001167868_CTCCCTGCCGTGTCCAACCC	ATP5SL__NM_001167868	866	682	725	834
ATP5SL__NM_001167869_CCCTGGCCTCACTCAGATAT	ATP5SL__NM_001167869	751	389	970	561
ATP5SL__NM_001167871_CGAGAGCTCCTGCAACGAGT	ATP5SL__NM_001167871	901	528	1228	489
ATP6AP1__NM_001183_AGGCGCTCTGGCGCATGCCG	ATP6AP1__NM_001183	45	48	33	30
ATP6AP1__NM_001183_CCTTCATGAGTGTCGGCCGC	ATP6AP1__NM_001183	1331	1019	1463	1495
ATP6AP1__NM_001183_CTCAGATGAGGTCATCGGGC	ATP6AP1__NM_001183	391	437	426	459
ATP6AP1__NM_001183_CTCCAGGGCGGGATCTAAGT	ATP6AP1__NM_001183	207	66	335	65
ATP6AP1__NM_001183_GCGACGGCTCGAGTGCGGAT	ATP6AP1__NM_001183	1679	816	1714	834
ATP6AP1__NM_001183_GCTGACTGCATACCAGTCGA	ATP6AP1__NM_001183	1178	835	1370	820
ATP6AP1__NM_001183_GGAAGGGCGGACCGCTGTGA	ATP6AP1__NM_001183	962	952	974	714
ATP6AP1__NM_001183_GGATGGATCACAGGTGATAC	ATP6AP1__NM_001183	296	210	259	187
ATP6AP1__NM_001183_GGTAGAGAGCTGCAAGTCGC	ATP6AP1__NM_001183	352	170	392	81
ATP6AP1__NM_001183_GTGGCCAGGTCCACATGCAA	ATP6AP1__NM_001183	135	248	219	100
ATP6AP1__NM_001183_TGGTGCTGTGGTCGAGTGAC	ATP6AP1__NM_001183	36	0	32	15
ATP6AP1__NM_001183_TGGTGCTTCCTGCCGTCGAC	ATP6AP1__NM_001183	11	7	44	93
ATP6AP1L__NM_001017971_AGGCGATGAGATCATATTGT	ATP6AP1L__NM_001017971	817	648	859	740
ATP6AP1L__NM_001017971_AGGGAGCATATACGCCAGTT	ATP6AP1L__NM_001017971	683	572	819	491
ATP6AP1L__NM_001017971_CAAGCAGTCTTTAACCCAAC	ATP6AP1L__NM_001017971	332	420	519	411
ATP6AP1L__NM_001017971_CTCCTACCGCTGCCAACGCG	ATP6AP1L__NM_001017971	8	0	0	0
ATP6AP1L__NM_001017971_GAGGGAGCATATACGCCAGT	ATP6AP1L__NM_001017971	1028	718	1265	945
ATP6AP1L__NM_001017971_GATGGACAACTTGTTGTAAT	ATP6AP1L__NM_001017971	475	533	537	590
ATP6AP1L__NM_001017971_GCAGCGGTAGGAGTAACCAG	ATP6AP1L__NM_001017971	658	456	597	587
ATP6AP1L__NM_001017971_GGTAAATCGTCCCCCCTTGA	ATP6AP1L__NM_001017971	577	587	592	477
ATP6AP1L__NM_001017971_TCCTACCGCTGCCAACGCGT	ATP6AP1L__NM_001017971	589	608	638	597
ATP6AP1L__NM_001017971_TGTTGGAAATGATCTCGACT	ATP6AP1L__NM_001017971	217	124	283	228
ATP6AP2__NM_005765_ATCACCATGACGGTAGCCCG	ATP6AP2__NM_005765	341	24	373	6
ATP6AP2__NM_005765_GAAACAGGCGATTACGGAGC	ATP6AP2__NM_005765	271	182	291	165
ATP6AP2__NM_005765_GAGAGTGTATATGGTAGGGA	ATP6AP2__NM_005765	435	290	437	256
ATP6AP2__NM_005765_GCAACACTGTCAAGACTAAA	ATP6AP2__NM_005765	176	128	137	124
ATP6AP2__NM_005765_GCTACATGATATTTCAAGCT	ATP6AP2__NM_005765	615	441	653	472
ATP6AP2__NM_005765_GGGATCCGCTCTCCTGGTAT	ATP6AP2__NM_005765	1562	799	1749	645
ATP6AP2__NM_005765_TAGCCCGAGGACGATGAAAC	ATP6AP2__NM_005765	60	29	88	37
ATP6AP2__NM_005765_TCGTCCTCGGGCTACCGTCA	ATP6AP2__NM_005765	1140	27	1062	47
ATP6AP2__NM_005765_TGCTTATGACGAGACAGCTT	ATP6AP2__NM_005765	64	62	91	45
ATP6AP2__NM_005765_TTCTCCTGATTTATATTCAC	ATP6AP2__NM_005765	0	0	5	0
ATP6AP2__NM_005765_TTCTTGGCCAGGACTCGCAG	ATP6AP2__NM_005765	0	0	0	0
ATP6AP2__NM_005765_TTTCTTGAAACAGGCGATTA	ATP6AP2__NM_005765	22	11	22	30
ATP6V0A1__NM_001130020_AACATTCCGATTATGGACAC	ATP6V0A1__NM_001130020	652	509	682	572
ATP6V0A1__NM_001130020_ATCCAGTTTGCACTCAGAAG	ATP6V0A1__NM_001130020	295	214	330	277
ATP6V0A1__NM_001130020_ATGCTTTGGCGGGTATGCCG	ATP6V0A1__NM_001130020	366	453	321	314
ATP6V0A1__NM_001130020_CATGCTTTGGCGGGTATGCC	ATP6V0A1__NM_001130020	385	376	471	740
ATP6V0A1__NM_001130020_CCGGGACATGATTGACTTAG	ATP6V0A1__NM_001130020	778	589	881	809
ATP6V0A1__NM_001130020_CGTGGCTGGTGTCATTAACC	ATP6V0A1__NM_001130020	926	706	1020	801
ATP6V0A1__NM_001130020_CTCTGACTGTAGAAAAAGCT	ATP6V0A1__NM_001130020	47	109	54	119
ATP6V0A1__NM_001130020_CTTCATTCACAAATTTCCGT	ATP6V0A1__NM_001130020	1099	1662	1440	1927
ATP6V0A1__NM_001130020_GAGTCACATCTATGTTGCAC	ATP6V0A1__NM_001130020	2000	1969	1835	1945
ATP6V0A1__NM_001130020_GTTCAAAATGGAAGGTACAG	ATP6V0A1__NM_001130020	68	99	60	65
ATP6V0A1__NM_001130020_TATTGTTGTGTCAGTGAATT	ATP6V0A1__NM_001130020	223	235	287	397
ATP6V0A1__NM_001130020_TGGAGTCAAGGTCGGTGACA	ATP6V0A1__NM_001130020	74	79	98	96
ATP6V0A2__NM_012463_CAGGGCGCTGAGGCACTCGT	ATP6V0A2__NM_012463	2167	2035	2422	2152
ATP6V0A2__NM_012463_CATCGTGTCCTATGCAGAAC	ATP6V0A2__NM_012463	865	963	1056	825
ATP6V0A2__NM_012463_CATTTCTAGAACCTGTTTCA	ATP6V0A2__NM_012463	289	317	373	300
ATP6V0A2__NM_012463_CTTTGTGAAACGCAATGTTG	ATP6V0A2__NM_012463	301	292	271	382
ATP6V0A2__NM_012463_GAAACAGGTTCTAGAAATGC	ATP6V0A2__NM_012463	139	107	144	121
ATP6V0A2__NM_012463_GAAGAGCTAGAGCGAATATT	ATP6V0A2__NM_012463	273	171	267	128
ATP6V0A2__NM_012463_GACCAGGCCTTTCTCGCCCA	ATP6V0A2__NM_012463	1205	1249	1466	1226
ATP6V0A2__NM_012463_TCTAGAACCTGTTTCAGGGG	ATP6V0A2__NM_012463	144	76	114	97
ATP6V0A2__NM_012463_TGAAGAGCTAGAGCGAATAT	ATP6V0A2__NM_012463	177	193	232	143
ATP6V0A2__NM_012463_TGTTGTGGAGAGTCTGCAAA	ATP6V0A2__NM_012463	131	221	146	184
ATP6V0A2__NM_012463_TGTTTGGATAGGGGTACACG	ATP6V0A2__NM_012463	141	143	170	90
ATP6V0A2__NM_012463_TTTGGAAAGAACTTACGTTC	ATP6V0A2__NM_012463	28	62	34	59
ATP6V0A4__NM_020632_AACTTTCCGGTCATATATGC	ATP6V0A4__NM_020632	328	272	315	379
ATP6V0A4__NM_020632_ACGGGAAATGATTACCCTGG	ATP6V0A4__NM_020632	828	834	942	828
ATP6V0A4__NM_020632_AGTCAGAAGGTGTGAATCAC	ATP6V0A4__NM_020632	158	157	198	154
ATP6V0A4__NM_020632_CATAGCCGGTGTGATCAACA	ATP6V0A4__NM_020632	145	148	177	171
ATP6V0A4__NM_020632_CCAGGGTAATCATTTCCCGT	ATP6V0A4__NM_020632	2120	1939	2181	1451
ATP6V0A4__NM_020632_CCTCTCCCTGTTGATCACAC	ATP6V0A4__NM_020632	990	1390	1074	1248
ATP6V0A4__NM_020632_GCATATATGACCGGAAAGTT	ATP6V0A4__NM_020632	705	663	773	974
ATP6V0A4__NM_020632_GGTGTCTGTGTTTCGAAGCG	ATP6V0A4__NM_020632	215	219	276	312
ATP6V0A4__NM_020632_GTAATCATTTCCCGTGGGAG	ATP6V0A4__NM_020632	302	499	419	504
ATP6V0A4__NM_020632_TCGCCACAGTAACCGCTCAA	ATP6V0A4__NM_020632	1326	1604	1520	1643
ATP6V0A4__NM_020632_TGAGCTCGGAGAGCTCGGAT	ATP6V0A4__NM_020632	7	22	19	1
ATP6V0A4__NM_020632_TTCTAGGAAGCTTTGTTTCA	ATP6V0A4__NM_020632	0	0	0	0
ATP6V0B__NM_001039457_ACCAGGGGCATCTATATTAC	ATP6V0B__NM_001039457	337	0	346	0
ATP6V0B__NM_001039457_CACTCCTCCACCAATGATGG	ATP6V0B__NM_001039457	739	696	831	624
ATP6V0B__NM_001039457_CAGGTTCTTGGTCTTGATCC	ATP6V0B__NM_001039457	172	69	197	124
ATP6V0B__NM_001039457_CCACAGACAGGGAGATAGCT	ATP6V0B__NM_001039457	53	0	51	1
ATP6V0B__NM_001039457_CCCCAAACATGGAGTAGCCT	ATP6V0B__NM_001039457	436	561	475	593
ATP6V0B__NM_001039457_CCCTTCATGTGGTCCAACCT	ATP6V0B__NM_001039457	152	15	160	10
ATP6V0B__NM_001039457_GAAGAGGTTAGACAGGCCTA	ATP6V0B__NM_001039457	291	0	383	12
ATP6V0B__NM_001039457_GCCACAGACCCCAAGGCCAT	ATP6V0B__NM_001039457	346	123	365	109
ATP6V0B__NM_001039457_GCCCTTCATGTGGTCCAACC	ATP6V0B__NM_001039457	86	31	118	70
ATP6V0B__NM_001039457_GGCCATCGGAACTACCATGC	ATP6V0B__NM_001039457	816	153	928	127
ATP6V0B__NM_001039457_GGCTCCTCCATCATTGGTGG	ATP6V0B__NM_001039457	221	312	354	331
ATP6V0B__NM_001039457_GGGGTTCTGAGCATCGGCCA	ATP6V0B__NM_001039457	70	10	59	43
ATP6V0B__NM_004047_ACACTAGGAAGATGGGTTGA	ATP6V0B__NM_004047	693	608	768	839
ATP6V0B__NM_004047_CCAAGCCTCTGAAAGGCTAA	ATP6V0B__NM_004047	109	85	151	61
ATP6V0B__NM_004047_CCTCTTGCTGCGTGTTGATT	ATP6V0B__NM_004047	1233	1604	1695	2053
ATP6V0B__NM_004047_CTTGCTGCGTGTTGATTTGG	ATP6V0B__NM_004047	1614	1715	1776	2005
ATP6V0C__NM_001198569_AGAGGCTGATGTCGTCATTC	ATP6V0C__NM_001198569	860	465	1056	442
ATP6V0C__NM_001198569_AGCGTCCCCCACGATGCCGA	ATP6V0C__NM_001198569	997	3	1266	42
ATP6V0C__NM_001198569_ATCATGCCCACGAATAGTCG	ATP6V0C__NM_001198569	591	65	788	81
ATP6V0C__NM_001198569_CAAGAGCGGTACCGGCATTG	ATP6V0C__NM_001198569	907	3	1016	41
ATP6V0C__NM_001198569_CAGCAGCCCCGACTATTCGT	ATP6V0C__NM_001198569	2580	6	2768	7
ATP6V0C__NM_001198569_CCAGCAGCCCCGACTATTCG	ATP6V0C__NM_001198569	1193	666	1304	915
ATP6V0C__NM_001198569_CGAAAAACGAAGCATACTCG	ATP6V0C__NM_001198569	103	4	145	20
ATP6V0C__NM_001198569_GATCATGCCCACGAATAGTC	ATP6V0C__NM_001198569	259	54	312	90
ATP6V0C__NM_001198569_GCTGGCATCATCGCCATCTA	ATP6V0C__NM_001198569	627	17	746	3
ATP6V0C__NM_001198569_GGCCATGTCTGTCATGCGGC	ATP6V0C__NM_001198569	491	199	488	396
ATP6V0C__NM_001198569_GGCGAAAAACGAAGCATACT	ATP6V0C__NM_001198569	208	36	228	43
ATP6V0C__NM_001198569_GGCTTTGCCATCGGCATCGT	ATP6V0C__NM_001198569	532	162	606	383
ATP6V0D1__NM_004691_AGTCCTGGAAAAAAGCCGCT	ATP6V0D1__NM_004691	946	789	1076	717
ATP6V0D1__NM_004691_ATGCTTCTTAGATACATCAG	ATP6V0D1__NM_004691	106	57	101	69
ATP6V0D1__NM_004691_CAACGAGGCATCACCTCTGA	ATP6V0D1__NM_004691	890	275	1011	328
ATP6V0D1__NM_004691_CAGTCCTGGAAAAAAGCCGC	ATP6V0D1__NM_004691	604	785	520	393
ATP6V0D1__NM_004691_CCCGGAGCTTTACTTTAACG	ATP6V0D1__NM_004691	1069	1442	1335	1462
ATP6V0D1__NM_004691_CTACAAGTTCTGCACCCTAC	ATP6V0D1__NM_004691	705	313	822	521
ATP6V0D1__NM_004691_CTGCCTAGTGGGTGGCACTT	ATP6V0D1__NM_004691	2083	178	2306	355
ATP6V0D1__NM_004691_CTTTACTTTAACGTGGACAA	ATP6V0D1__NM_004691	61	39	90	37
ATP6V0D1__NM_004691_GAAGCTGGCGAGTGGCTCAT	ATP6V0D1__NM_004691	826	66	1056	62
ATP6V0D1__NM_004691_GGCCCCAGGGTTCTCAGGTC	ATP6V0D1__NM_004691	231	222	330	367
ATP6V0D1__NM_004691_GTCCTGGAAAAAAGCCGCTG	ATP6V0D1__NM_004691	722	158	800	137
ATP6V0D1__NM_004691_TACAAGTTCTGCACCCTACT	ATP6V0D1__NM_004691	370	91	395	130
ATP6V0D1__NM_004691_TCTCGATGTTCATCTCGTCA	ATP6V0D1__NM_004691	657	695	920	1123
ATP6V0D1__NM_004691_TCTGCTCGAAGCTGCCTAGT	ATP6V0D1__NM_004691	539	146	572	79
ATP6V0D2__NM_152565_AGCTCCGCACCTTCGAGCAT	ATP6V0D2__NM_152565	189	192	239	155
ATP6V0D2__NM_152565_AGTTACCATAATCAGTAGTC	ATP6V0D2__NM_152565	245	233	236	272
ATP6V0D2__NM_152565_ATGGCATTAAAGAGATCTGA	ATP6V0D2__NM_152565	206	130	265	254
ATP6V0D2__NM_152565_CAGCTCCGCACCTTCGAGCA	ATP6V0D2__NM_152565	326	225	383	468
ATP6V0D2__NM_152565_CCAAAATTGACACTGAGATG	ATP6V0D2__NM_152565	495	500	562	672
ATP6V0D2__NM_152565_CCAGGTTGATATAGTCTTGC	ATP6V0D2__NM_152565	355	461	379	392
ATP6V0D2__NM_152565_CTCCGCACCTTCGAGCATGG	ATP6V0D2__NM_152565	242	54	225	107
ATP6V0D2__NM_152565_GAGATGAGGAAAAGACTATG	ATP6V0D2__NM_152565	215	275	293	271
ATP6V0D2__NM_152565_GCTCCGCACCTTCGAGCATG	ATP6V0D2__NM_152565	312	447	297	383
ATP6V0D2__NM_152565_GTCTATCATATAACTGCACC	ATP6V0D2__NM_152565	184	274	178	266
ATP6V0D2__NM_152565_TAATGGCGTTTCGATCAGAA	ATP6V0D2__NM_152565	183	91	205	290
ATP6V0D2__NM_152565_TGGCCTTGCATCCTCGAACC	ATP6V0D2__NM_152565	60	33	59	86
ATP6V0E1__NM_003945_ACCCCCGGTTAGGACCCTTA	ATP6V0E1__NM_003945	584	654	614	705
ATP6V0E1__NM_003945_AGTGAGGCCGTGATACGCCA	ATP6V0E1__NM_003945	690	974	586	706
ATP6V0E1__NM_003945_CATCCCTAAGGGTCCTAACC	ATP6V0E1__NM_003945	1137	577	1279	664
ATP6V0E1__NM_003945_GGTGCCTTGGTTCATCCCTA	ATP6V0E1__NM_003945	2148	1846	2532	2598
ATP6V0E1__NM_003945_GTGCCTTGGTTCATCCCTAA	ATP6V0E1__NM_003945	273	271	389	442
ATP6V0E1__NM_003945_TCATCCCTAAGGGTCCTAAC	ATP6V0E1__NM_003945	649	761	834	838
ATP6V0E1__NM_003945_TCCCTAAGGGTCCTAACCGG	ATP6V0E1__NM_003945	355	343	350	269
ATP6V0E2__NM_001100592_AAGGGACCCAACCGCGGGTA	ATP6V0E2__NM_001100592	337	245	370	330
ATP6V0E2__NM_001100592_AGCACCCGAAGCGATCAGCC	ATP6V0E2__NM_001100592	700	733	804	704
ATP6V0E2__NM_001100592_CAAGAGGTAACAGCAGACGG	ATP6V0E2__NM_001100592	637	676	583	667
ATP6V0E2__NM_001100592_CGGGCCCTGGTTCGTGCCGA	ATP6V0E2__NM_001100592	1119	1335	1564	1716
ATP6V0E2__NM_001100592_GAGCACCCGAAGCGATCAGC	ATP6V0E2__NM_001100592	71	16	108	115
ATP6V0E2__NM_001100592_GGCGAATGAGTGCGCCGTCA	ATP6V0E2__NM_001100592	1591	1982	1852	1950
ATP6V0E2__NM_001100592_GGGGGAGGGTCCGGACGAAG	ATP6V0E2__NM_001100592	566	765	601	569
ATP6V0E2__NM_001100592_GGGTCCCTTCGGCACGAACC	ATP6V0E2__NM_001100592	58	106	123	148
ATP6V0E2__NM_001100592_GGTCCCTTCGGCACGAACCA	ATP6V0E2__NM_001100592	170	154	186	238
ATP6V0E2__NM_001100592_TCATCATCTTCACCACGTTC	ATP6V0E2__NM_001100592	2027	2178	2205	2542
ATP6V0E2__NM_001100592_TCCCTGACAACCCCTTCGTC	ATP6V0E2__NM_001100592	866	1060	930	782
ATP6V0E2__NM_001100592_TGGGGGAGGGTCCGGACGAA	ATP6V0E2__NM_001100592	105	251	93	122
ATP6V0E2__NM_001289990_CGCAGCTGAACCCCCTGTTC	ATP6V0E2__NM_001289990	3887	4050	4426	4285
ATP6V0E2__NM_001289990_GCGCAGCTGAACCCCCTGTT	ATP6V0E2__NM_001289990	4827	6086	5293	6334
ATP6V0E2__NM_001289990_TTCAGCTGGGGCCCGAACAG	ATP6V0E2__NM_001289990	39	58	59	61
ATP6V1A__NM_001690_CAAAGCATCTACATCCCCAG	ATP6V1A__NM_001690	1249	999	1686	1404
ATP6V1A__NM_001690_CTCTGGGGATGTAGATGCTT	ATP6V1A__NM_001690	399	211	413	144
ATP6V1A__NM_001690_GCACATTTGGTTATGTGCAT	ATP6V1A__NM_001690	923	1041	878	1285
ATP6V1A__NM_001690_GGAGATCCTGTACTTCGCAC	ATP6V1A__NM_001690	1558	440	1587	583
ATP6V1A__NM_001690_GGCTGCACCCGCCATGTCAC	ATP6V1A__NM_001690	1391	895	1573	970
ATP6V1A__NM_001690_GGGTGACATGGCTACTATTC	ATP6V1A__NM_001690	778	25	822	41
ATP6V1A__NM_001690_GGTTGGAGAGATTATTCGAT	ATP6V1A__NM_001690	467	61	598	94
ATP6V1A__NM_001690_GTGGGCCACAGCGAATTGGT	ATP6V1A__NM_001690	233	12	333	15
ATP6V1A__NM_001690_TCTCTCCAACCAATTCGCTG	ATP6V1A__NM_001690	220	243	280	287
ATP6V1A__NM_001690_TGACTGCTGATATCCGACAA	ATP6V1A__NM_001690	472	187	405	90
ATP6V1A__NM_001690_TGGAGAGATTATTCGATTGG	ATP6V1A__NM_001690	212	49	296	77
ATP6V1A__NM_001690_TTACACCTTGCAAAAACCTA	ATP6V1A__NM_001690	103	329	79	97
ATP6V1B1__NM_001692_AAGACCACTTGCGAATTTAC	ATP6V1B1__NM_001692	203	299	227	226
ATP6V1B1__NM_001692_AGACCACTTGCGAATTTACA	ATP6V1B1__NM_001692	600	530	702	670
ATP6V1B1__NM_001692_AGGGGACATCCTACGAACTC	ATP6V1B1__NM_001692	361	245	277	289
ATP6V1B1__NM_001692_AGTGGACGATCTCCGCATAC	ATP6V1B1__NM_001692	375	449	480	565
ATP6V1B1__NM_001692_ATGCTGTTCATGACGTCAAT	ATP6V1B1__NM_001692	1729	1924	1900	2322
ATP6V1B1__NM_001692_CAGATCTGAGCGGCAATCTA	ATP6V1B1__NM_001692	988	1244	963	1018
ATP6V1B1__NM_001692_CGCTCCTCTGAGTCCCATCT	ATP6V1B1__NM_001692	2285	2030	2790	2374
ATP6V1B1__NM_001692_CTCCGGCAAGCCCATTGACA	ATP6V1B1__NM_001692	286	308	448	443
ATP6V1B1__NM_001692_GACCACTGGCCCCTTGTCAA	ATP6V1B1__NM_001692	324	377	370	445
ATP6V1B1__NM_001692_GACCACTTGCGAATTTACAG	ATP6V1B1__NM_001692	554	524	583	389
ATP6V1B1__NM_001692_TGCACACAGTCCTGTAGGCT	ATP6V1B1__NM_001692	849	1117	756	1000
ATP6V1B1__NM_001692_TGGCACCAAGGCGATTGTTC	ATP6V1B1__NM_001692	104	94	110	79
ATP6V1B2__NM_001693_AAAACGTCCTGTGAGTTTAC	ATP6V1B2__NM_001693	316	432	303	403
ATP6V1B2__NM_001693_AAACGTCCTGTGAGTTTACT	ATP6V1B2__NM_001693	1080	864	1170	1082
ATP6V1B2__NM_001693_AAAGATGGAATGCACGGGGT	ATP6V1B2__NM_001693	252	192	259	258
ATP6V1B2__NM_001693_AATTGTCCATTTGACCTTAC	ATP6V1B2__NM_001693	267	0	380	35
ATP6V1B2__NM_001693_ACATGATCTAAGATCACTAG	ATP6V1B2__NM_001693	123	77	216	193
ATP6V1B2__NM_001693_ACCAGTATTTCACAAGAGCG	ATP6V1B2__NM_001693	247	249	271	326
ATP6V1B2__NM_001693_ACCCCAAACAACAACTCTAC	ATP6V1B2__NM_001693	923	1066	1094	1144
ATP6V1B2__NM_001693_ACTGCATAGAAGATCGCTCA	ATP6V1B2__NM_001693	361	556	394	457
ATP6V1B2__NM_001693_AGAGAGACCAACAGGACGCA	ATP6V1B2__NM_001693	178	181	260	285
ATP6V1B2__NM_001693_AGCAGTGAGACCCCTTGTCT	ATP6V1B2__NM_001693	1650	1764	1540	2181
ATP6V1B2__NM_001693_AGCTACCCGTGCCCACCGGT	ATP6V1B2__NM_001693	1364	689	1887	960
ATP6V1B2__NM_001693_AGTGATCTTAGATCATGTTA	ATP6V1B2__NM_001693	120	16	161	15
ATP6V1B2__NM_001693_AGTTTAAAACGTTGCAACAC	ATP6V1B2__NM_001693	105	178	176	257
ATP6V1B2__NM_001693_ATGGACAATTTCAGCATACC	ATP6V1B2__NM_001693	51	1	45	5
ATP6V1B2__NM_001693_CAAACCGAAAGCAAACACTC	ATP6V1B2__NM_001693	456	373	507	573
ATP6V1B2__NM_001693_CATGGTAAACTCCAACACAA	ATP6V1B2__NM_001693	200	340	245	259
ATP6V1B2__NM_001693_CCGGTGGGCACGGGTAGCTC	ATP6V1B2__NM_001693	2	19	15	6
ATP6V1B2__NM_001693_CTCTGCTTGGAAACCCAGTA	ATP6V1B2__NM_001693	321	431	405	480
ATP6V1B2__NM_001693_CTCTTTAGTACTTACGTATT	ATP6V1B2__NM_001693	59	83	76	91
ATP6V1B2__NM_001693_GCTCAGGGCTCACGTCAGGA	ATP6V1B2__NM_001693	451	394	640	538
ATP6V1B2__NM_001693_GGGCAAGTTCTGGAAGTTAG	ATP6V1B2__NM_001693	286	92	312	71
ATP6V1B2__NM_001693_GGGCACGGGTAGCTCGGGTG	ATP6V1B2__NM_001693	211	100	321	180
ATP6V1B2__NM_001693_GTCCATTTGACCTTACCGGA	ATP6V1B2__NM_001693	212	6	275	17
ATP6V1B2__NM_001693_GTGCTGCTGCTCGAAGCAGA	ATP6V1B2__NM_001693	142	257	224	173
ATP6V1B2__NM_001693_TCTCTTTAGTACTTACGTAT	ATP6V1B2__NM_001693	156	260	203	192
ATP6V1B2__NM_001693_TCTGGAAGTTAGTGGTTCCA	ATP6V1B2__NM_001693	118	6	205	0
ATP6V1B2__NM_001693_TGCTTTGGTCTCTACATTAG	ATP6V1B2__NM_001693	359	420	409	537
ATP6V1C1__NM_001695_AACATGGGAGAAATTGCATG	ATP6V1C1__NM_001695	946	257	1105	355
ATP6V1C1__NM_001695_AGACCTTGGAACTACCATTT	ATP6V1C1__NM_001695	571	45	721	113
ATP6V1C1__NM_001695_AGAGTATCTCGTCACATTAC	ATP6V1C1__NM_001695	383	19	484	16
ATP6V1C1__NM_001695_CAAGAGAATCTGTTGGCTAA	ATP6V1C1__NM_001695	562	430	465	385
ATP6V1C1__NM_001695_CATTACACAGGTAACTGTCT	ATP6V1C1__NM_001695	364	0	439	31
ATP6V1C1__NM_001695_CTACTTTCTTAACCACTCTG	ATP6V1C1__NM_001695	261	18	326	65
ATP6V1C1__NM_001695_GCAGGTTAAACCACAACGAC	ATP6V1C1__NM_001695	402	105	384	160
ATP6V1C1__NM_001695_GGTTGGCACGTTGGATGTCT	ATP6V1C1__NM_001695	1019	502	1142	883
ATP6V1C1__NM_001695_GTATGAAACACTAGCCGAAA	ATP6V1C1__NM_001695	349	313	339	387
ATP6V1C1__NM_001695_GTCAGATGAACTGGCTAAAC	ATP6V1C1__NM_001695	406	38	429	52
ATP6V1C1__NM_001695_TAAACAAGGTGACATTACAC	ATP6V1C1__NM_001695	0	0	0	0
ATP6V1C1__NM_001695_TGACTTCCAGTATAATGAAG	ATP6V1C1__NM_001695	101	27	77	51
ATP6V1C2__NM_001039362_ACCTCCTGGCAAACGGAGGT	ATP6V1C2__NM_001039362	240	328	313	448
ATP6V1C2__NM_001039362_CAAGCAGCCGCTCGTGAGTG	ATP6V1C2__NM_001039362	524	663	692	746
ATP6V1C2__NM_001039362_CATGTCTGAGAGAGATTCGT	ATP6V1C2__NM_001039362	990	876	1154	1244
ATP6V1C2__NM_001039362_CGAATTTGGTATTATAAGAC	ATP6V1C2__NM_001039362	387	547	386	443
ATP6V1C2__NM_001039362_CTACGAATCTCTCTCAGACA	ATP6V1C2__NM_001039362	671	974	832	874
ATP6V1C2__NM_001039362_GAAGTCAGGAATAGCGAATT	ATP6V1C2__NM_001039362	17	17	22	43
ATP6V1C2__NM_001039362_GATTGAAGATTTCAAAACCA	ATP6V1C2__NM_001039362	152	211	110	80
ATP6V1C2__NM_001039362_GCAGACCAAACTACTCACAA	ATP6V1C2__NM_001039362	654	921	835	938
ATP6V1C2__NM_001039362_GTGTCCACCACACTCACGAG	ATP6V1C2__NM_001039362	555	404	541	546
ATP6V1C2__NM_001039362_TGTCTTCAGAGTGTTGTAGG	ATP6V1C2__NM_001039362	163	268	173	312
ATP6V1C2__NM_001039362_TGTGGTGGACACAATAGCCA	ATP6V1C2__NM_001039362	279	377	289	240
ATP6V1C2__NM_001039362_TTTGGTATTATAAGACAGGT	ATP6V1C2__NM_001039362	33	4	67	49
ATP6V1D__NM_015994_AAGCAAGAGTACGTTCAATC	ATP6V1D__NM_015994	111	0	189	9
ATP6V1D__NM_015994_AATGGCATTTACACGCCTGT	ATP6V1D__NM_015994	635	97	617	85
ATP6V1D__NM_015994_AGCAAGAGTACGTTCAATCC	ATP6V1D__NM_015994	366	32	371	34
ATP6V1D__NM_015994_ATCATGAAGGCTCGTTTAAA	ATP6V1D__NM_015994	153	1	240	39
ATP6V1D__NM_015994_CATCATGAAGGCTCGTTTAA	ATP6V1D__NM_015994	236	37	330	82
ATP6V1D__NM_015994_CTGTCGAAATCGAAGAGTTA	ATP6V1D__NM_015994	314	505	317	455
ATP6V1D__NM_015994_CTTGGCTTCAGCTAGTGAAA	ATP6V1D__NM_015994	166	4	179	1
ATP6V1D__NM_015994_GAACTGACTGGTTTAGCCAG	ATP6V1D__NM_015994	238	0	274	7
ATP6V1D__NM_015994_GAGAGCGAGAAGAGTTCTAT	ATP6V1D__NM_015994	375	3	464	14
ATP6V1D__NM_015994_GCAAGAGTACGTTCAATCCG	ATP6V1D__NM_015994	1853	48	2311	47
ATP6V1D__NM_015994_GCTGAAGCCAAGTTCACAGC	ATP6V1D__NM_015994	599	876	797	1205
ATP6V1D__NM_015994_TAGGGCACAGACCATCATGA	ATP6V1D__NM_015994	180	12	300	13
ATP6V1E1__NM_001039366_AACCCAAAGACTAAAGATTA	ATP6V1E1__NM_001039366	208	71	296	74
ATP6V1E1__NM_001039366_ACAAGATTTCCCTCTGGTAA	ATP6V1E1__NM_001039366	492	5	524	42
ATP6V1E1__NM_001039366_AGGTGGTAAAAGATACAACC	ATP6V1E1__NM_001039366	14	0	6	1
[illegible]	[illegible]	[illegible]	[illegible]	[illegible]	[illegible]
ATP6V1F__NM_001198909_ATCGAAGACACTTTCCGGTA	ATP6V1F__NM_001198909	974	30	1012	31
ATP6V1F__NM_001198909_CAATTTCATCCCAAAGCGGA	ATP6V1F__NM_001198909	901	778	992	1130
ATP6V1F__NM_001198909_CGGAAGGCTTCCAAGTGAAC	ATP6V1F__NM_001198909	317	667	482	650
ATP6V1F__NM_001198909_CTTCTCCACCACCAGGAAAT	ATP6V1F__NM_001198909	149	15	225	18
ATP6V1F__NM_001198909_GAATCAATTTCATCCCAAAG	ATP6V1F__NM_001198909	72	116	134	204
[illegible]	[illegible]	[illegible]	[illegible]	[illegible]	[illegible]
ATP8A2__NM_016529_TAGCCCAAAGAAGAACGAAC	ATP8A2__NM_016529	199	128	168	155
ATP8A2__NM_016529_TCGAGGATCCGCTCGTCCGT	ATP8A2__NM_016529	1740	1446	1833	1512
ATP8A2__NM_016529_TTCTTCTTTGGGCTATAAGA	ATP8A2__NM_016529	10	21	2	18
[illegible]	[illegible]	[illegible]	[illegible]	[illegible]	[illegible]
ATPAF2__NM_145691_ACGCCGTCTCCTGAATCGGC	ATPAF2__NM_145691	131	132	152	126
ATPAF2__NM_145691_CCAGCATCCCTGCCAAAACT	ATPAF2__NM_145691	1339	1206	1315	1077
ATPAF2__NM_145691_CCAGCGCTTCTATGAGTCCG	ATPAF2__NM_145691	948	634	1175	794
[illegible]	[illegible]	[illegible]	[illegible]	[illegible]	[illegible]

B3GNT3__NM_014256_GACCGGCTGCGCGCACTTAG	B3GNT3__NM_014256	1498	1478	1429	1391
B3GNT3__NM_014256_GCGGCGCACATAGTTGCTAG	B3GNT3__NM_014256	889	611	936	930
B3GNT3__NM_014256_GGCTGCGCGCACTTAGAGGG	B3GNT3__NM_014256	195	256	200	181
B3GNT3__NM_014256_GGGCCGCGAGCGCAAGGTAC	B3GNT3__NM_014256	739	855	856	796
B3GNT3__NM_014256_GTGACACTAGCAGACTGAAG	B3GNT3__NM_014256	130	127	144	64
B3GNT3__NM_014256_GTGCCGGAGATACTTCATCC	B3GNT3__NM_014256	423	260	612	445
B3GNT3__NM_014256_TAGAGGTGTTGGCATGGCAC	B3GNT3__NM_014256	155	103	97	83
B3GNT4__NM_030765_ACCGGCTGTGACGGGGTGTT	B3GNT4__NM_030765	2536	3254	2950	3562
B3GNT4__NM_030765_AGAGACGGTGACGGCTAGGC	B3GNT4__NM_030765	486	520	498	712
B3GNT4__NM_030765_AGCTGCTGGCCTATGAGAGT	B3GNT4__NM_030765	631	751	782	647
B3GNT4__NM_030765_ATCAAACTCCCTACTCTCAT	B3GNT4__NM_030765	1637	1831	1703	1661
B3GNT4__NM_030765_ATCTCCCTTTAGCATGAAAT	B3GNT4__NM_030765	99	162	93	174
B3GNT4__NM_030765_CACCGGCTGTGACGGGGTGT	B3GNT4__NM_030765	1217	1066	1366	1072
B3GNT4__NM_030765_CATCTCCCTTTAGCATGAAA	B3GNT4__NM_030765	345	558	468	656
B3GNT4__NM_030765_CCAGGCCCATTTCATGCTAA	B3GNT4__NM_030765	672	1001	818	1138
B3GNT4__NM_030765_GCTGCTGGCCTATGAGAGTA	B3GNT4__NM_030765	277	420	371	291
B3GNT4__NM_030765_TCCAGGAACTCTAACACGTT	B3GNT4__NM_030765	758	798	907	647
B3GNT4__NM_030765_TGGCCCTGTACATTGAGGGT	B3GNT4__NM_030765	121	165	113	47
B3GNT4__NM_030765_TTTAGCATGAAATGGGCCTG	B3GNT4__NM_030765	38	105	100	96
B3GNT5__NM_032047_ATCGACGTTCCGGAATTAGA	B3GNT5__NM_032047	395	303	476	393
B3GNT5__NM_032047_CAAAGTCATAGCTATTTATG	B3GNT5__NM_032047	26	33	31	43
B3GNT5__NM_032047_CCTTTGTAAAATTAGCTATG	B3GNT5__NM_032047	326	263	279	296
B3GNT5__NM_032047_CTCACAATGTGATTATCGAT	B3GNT5__NM_032047	375	284	355	552
B3GNT5__NM_032047_CTGAGGTGTGCTTAAGAGAC	B3GNT5__NM_032047	212	200	293	418
B3GNT5__NM_032047_GAAAACTATGATCGACGTTC	B3GNT5__NM_032047	360	507	508	613
B3GNT5__NM_032047_GGGGCAATGAAAATTATGTT	B3GNT5__NM_032047	662	839	761	774
B3GNT5__NM_032047_GTGGCAGAAGAGTCAAAAAA	B3GNT5__NM_032047	11	3	8	18
B3GNT5__NM_032047_TACATCACCGGAGATTACAT	B3GNT5__NM_032047	419	510	467	554
B3GNT5__NM_032047_TGTCCTATGAAATGTACCAG	B3GNT5__NM_032047	177	250	189	203
B3GNT5__NM_032047_TTAATCAAGTATTGGTAGCG	B3GNT5__NM_032047	203	252	246	347
B3GNT5__NM_032047_TTCCGGAATTAGAAGGACGT	B3GNT5__NM_032047	15	1	30	47
B3GNT6__NM_138706_AGGAAGTCCTGGATGCGCGC	B3GNT6__NM_138706	1089	1099	1316	1104
B3GNT6__NM_138706_CAGGCGCCGACTTCACCGCC	B3GNT6__NM_138706	1085	1073	1220	1367
B3GNT6__NM_138706_CCAGCCAGTCGAGCAAGTGC	B3GNT6__NM_138706	596	607	651	489
B3GNT6__NM_138706_CCAGGAGCAGGAACACGCCT	B3GNT6__NM_138706	877	738	850	794
B3GNT6__NM_138706_CCCGCTGCTTTGGGACGCAC	B3GNT6__NM_138706	954	946	1015	1249
B3GNT6__NM_138706_CCGTGCCCATCCGCGACAGC	B3GNT6__NM_138706	63	39	115	162
B3GNT6__NM_138706_CTTCCCCGGGTCCGCTTACC	B3GNT6__NM_138706	873	664	905	642
B3GNT6__NM_138706_GAGGCGTTCGCCACGCACGG	B3GNT6__NM_138706	59	19	76	37
B3GNT6__NM_138706_GTACACCGGGTAAGCGGACC	B3GNT6__NM_138706	202	182	295	164
B3GNT6__NM_138706_TACACCGGGTAAGCGGACCC	B3GNT6__NM_138706	226	151	219	223
B3GNT6__NM_138706_TCAGCCGCGGGAGCGTCGGT	B3GNT6__NM_138706	852	821	1127	754
B3GNT6__NM_138706_TTGCTCCAGCTGTCGCGGAT	B3GNT6__NM_138706	169	193	176	252
B3GNT7__NM_145236_ACAGGAAGAAAACCGTCTAC	B3GNT7__NM_145236	253	254	328	418
B3GNT7__NM_145236_AGCACGCTCGGCCCATTCGC	B3GNT7__NM_145236	1671	1337	1911	1820
B3GNT7__NM_145236_ATAGCTGGCCTTGCCGTACA	B3GNT7__NM_145236	372	531	390	306
B3GNT7__NM_145236_CAGGCACAGACTCCGGTAGA	B3GNT7__NM_145236	786	888	758	977
B3GNT7__NM_145236_CCACAGGAAAACCTGTTCGT	B3GNT7__NM_145236	2209	2351	2703	2645
B3GNT7__NM_145236_GACCAGGGGTGAGACTGCGT	B3GNT7__NM_145236	875	799	893	761
B3GNT7__NM_145236_GATAGCTGGCCTTGCCGTAC	B3GNT7__NM_145236	410	465	469	601
B3GNT7__NM_145236_GCCACAGGAAAACCTGTTCG	B3GNT7__NM_145236	1731	1420	1876	2084
B3GNT7__NM_145236_GCCAGCTATCCGCCGTATGC	B3GNT7__NM_145236	643	362	674	795
B3GNT7__NM_145236_GGCTCCAGGACCTGGAACCA	B3GNT7__NM_145236	297	202	381	397
B3GNT7__NM_145236_GGTCAGCCAGAAATTCTAGC	B3GNT7__NM_145236	264	325	330	404
B3GNT7__NM_145236_TTGTTGTCTTTCCTGCGAAT	B3GNT7__NM_145236	185	178	165	241
B3GNT8__NM_198540_CACCCACCGGAGACCCTAGC	B3GNT8__NM_198540	919	1245	1055	1308
B3GNT8__NM_198540_CCCAGGCCACTAGTGAGTCT	B3GNT8__NM_198540	574	869	644	707
B3GNT8__NM_198540_CTGCTGGTTCCAGTAAGCAA	B3GNT8__NM_198540	73	72	73	115
B3GNT8__NM_198540_GAAGTCAGGGATCTCGGTGG	B3GNT8__NM_198540	658	636	765	715
B3GNT8__NM_198540_GCAGCGGGATAGTCTGGCCC	B3GNT8__NM_198540	590	568	759	945
B3GNT8__NM_198540_GCCTCAAAGTGTACATCGAG	B3GNT8__NM_198540	626	551	632	711
B3GNT8__NM_198540_GCGCCCTGGTTCTGACTTGA	B3GNT8__NM_198540	1068	1419	1188	1768
B3GNT8__NM_198540_TCCCGCTGCCCTTTGCTTAC	B3GNT8__NM_198540	1891	1925	1791	2715
B3GNT8__NM_198540_TCTGGTTGAATGGGACGTCG	B3GNT8__NM_198540	293	262	360	366
B3GNT8__NM_198540_TCTTTGAGCGTCTGGTTGAA	B3GNT8__NM_198540	157	311	200	259
B3GNT8__NM_198540_TGGGGTAGGAGGCGAAGTCA	B3GNT8__NM_198540	57	46	13	11
B3GNT8__NM_198540_TTGGCCGTCAAGTCAGAACC	B3GNT8__NM_198540	332	287	297	301
B3GNT9__NM_033309_AAATAGCGGGCGAAGTCAAA	B3GNT9__NM_033309	274	310	174	240
B3GNT9__NM_033309_CAGCCCCGCCGGCCTACGAA	B3GNT9__NM_033309	39	29	41	47
B3GNT9__NM_033309_CGCGTTCCAGTTACCCGACG	B3GNT9__NM_033309	1433	2306	1580	2028
B3GNT9__NM_033309_CGTCGGGTAACTGGAACGCG	B3GNT9__NM_033309	608	921	707	835
B3GNT9__NM_033309_CTGCACCCGCGTCGGGTAAC	B3GNT9__NM_033309	214	270	301	324
B3GNT9__NM_033309_GCGAAGTCAAAGGGTCCCGT	B3GNT9__NM_033309	1494	1701	1868	2142
B3GNT9__NM_033309_GCGTTCCAGTTACCCGACGC	B3GNT9__NM_033309	666	833	735	960
B3GNT9__NM_033309_GGTCCTTGGCGCGCAAATAG	B3GNT9__NM_033309	79	125	89	103
B3GNT9__NM_033309_GTCCTTGGCGCGCAAATAGC	B3GNT9__NM_033309	105	54	74	129
B3GNT9__NM_033309_TCAAAGGGTCCCGTAGGCGT	B3GNT9__NM_033309	424	308	386	372
B3GNT9__NM_033309_TGCGGCTGGTTAATGAGCAG	B3GNT9__NM_033309	578	479	601	719
B3GNT9__NM_033309_TGTCCCCTTCGTAGGCCGGC	B3GNT9__NM_033309	356	396	309	463
B3GNTL1__NM_001009905_ACGGTTCAGCGTTGTGGACT	B3GNTL1__NM_001009905	315	356	397	485
B3GNTL1__NM_001009905_CACGGTTCAGCGTTGTGGAC	B3GNTL1__NM_001009905	332	329	348	549
B3GNTL1__NM_001009905_CAGTCCACAACGCTGAACCG	B3GNTL1__NM_001009905	935	995	1311	1284
B3GNTL1__NM_001009905_CCACAACGCTGAACCGTGGC	B3GNTL1__NM_001009905	636	824	623	890
B3GNTL1__NM_001009905_CCACCGAACGATACACACGT	B3GNTL1__NM_001009905	1725	1491	1996	1690
B3GNTL1__NM_001009905_CGTGCCCCCCAATGATCACG	B3GNTL1__NM_001009905	2237	1610	2399	1983
B3GNTL1__NM_001009905_CGTGGCTGGACGAATGTTTG	B3GNTL1__NM_001009905	1169	1115	1312	1358
B3GNTL1__NM_001009905_GATTCTCCCTCTCCTAGAGG	B3GNTL1__NM_001009905	34	26	32	33
B3GNTL1__NM_001009905_GGACTGGGAGGATAATAGAC	B3GNTL1__NM_001009905	137	74	190	156
B3GNTL1__NM_001009905_GGTGTCCACGTGATCATTGG	B3GNTL1__NM_001009905	1117	1162	1187	1408
B3GNTL1__NM_001009905_GTGTCCACGTGATCATTGGG	B3GNTL1__NM_001009905	707	433	672	492
B3GNTL1__NM_001009905_TGGTGTCCACGTGATCATTG	B3GNTL1__NM_001009905	159	101	136	74
B4GALNT1__NM_001276468_ACCGGGATGTGTGCGTAGCG	B4GALNT1__NM_001276468	218	247	319	230
B4GALNT1__NM_001276468_CCCACGGCGCAAGAGGTAGC	B4GALNT1__NM_001276468	232	305	224	114
B4GALNT1__NM_001276468_CCCGCTCCAGTACCCCCTAC	B4GALNT1__NM_001276468	1105	1228	1237	923

B4GALNT1__NM_001276468_CCGCTCCAGTACCCCCTACA	B4GALNT1__NM_001276468	1874	1878	2124	1173
B4GALNT1__NM_001276468_CCGGCTACCTCTTGCGCCGT	B4GALNT1__NM_001276468	2454	2063	2802	2105
B4GALNT1__NM_001276468_CGTCCCGGGTGCTCGCGTAC	B4GALNT1__NM_001276468	1328	1162	1369	772
B4GALNT1__NM_001276468_GACCGGGATGTGTGCGTAGC	B4GALNT1__NM_001276468	87	98	68	103
B4GALNT1__NM_001276468_GCTACGCACACATCCCGGTC	B4GALNT1__NM_001276468	603	663	634	320
B4GALNT1__NM_001276468_GTCCAGCCCTGGGCTGACAA	B4GALNT1__NM_001276468	26	8	50	11
B4GALNT1__NM_001276468_GTGACTGGAGTTACTCTCAC	B4GALNT1__NM_001276468	442	427	567	449
B4GALNT1__NM_001276468_TCCGGCTACCTCTTGCGCCG	B4GALNT1__NM_001276468	577	549	545	727
B4GALNT1__NM_001276468_TGAACTTCCACACCCTGTAG	B4GALNT1__NM_001276468	164	115	130	58
B4GALNT1__NM_001276469_AGTTCCAGGCCTTTCTGTCG	B4GALNT1__NM_001276469	1195	1186	1293	719
B4GALNT1__NM_001276469_GCAGTTGTGAGTCCAGTGGG	B4GALNT1__NM_001276469	865	694	820	594
B4GALNT2__NM_001159387_AGATTCCATCGTAGGAAAAG	B4GALNT2__NM_001159387	55	22	81	73
B4GALNT2__NM_001159387_ATAGCTTGGGTATGACAGGA	B4GALNT2__NM_001159387	248	332	251	252
B4GALNT2__NM_001159387_ATCCAACAATGCCAAGTACC	B4GALNT2__NM_001159387	336	374	304	270
B4GALNT2__NM_001159387_CACCTCATAGACGGGGGCAT	B4GALNT2__NM_001159387	1057	857	940	1017
B4GALNT2__NM_001159387_GAAGCTGTCAGGGTGACCTA	B4GALNT2__NM_001159387	890	683	786	638
B4GALNT2__NM_001159387_GACTTGGGAGTTCTGGCTTG	B4GALNT2__NM_001159387	880	911	1047	1062
B4GALNT2__NM_001159387_TCCTCGCTTTCACCGCTGGG	B4GALNT2__NM_001159387	930	1149	921	908
B4GALNT2__NM_001159387_TCTGTGGCTCCTCAAGATAT	B4GALNT2__NM_001159387	371	465	367	228
B4GALNT2__NM_001159387_TGGGATGGGAACCGTGTGCA	B4GALNT2__NM_001159387	207	151	252	281
B4GALNT2__NM_001159387_TGTCTCCTCGCTTTCACCGC	B4GALNT2__NM_001159387	115	43	120	116
B4GALNT2__NM_001159387_TGTGAAGCCAACAAAGAGCA	B4GALNT2__NM_001159387	284	197	281	254
B4GALNT2__NM_001159387_TTGGTCATAATCCTGGTACT	B4GALNT2__NM_001159387	209	220	259	237
B4GALNT2__NM_001159388_GGTCCAGGGTCGTATAGCTT	B4GALNT2__NM_001159388	36	2	73	27
B4GALNT2__NM_001159388_TCGTATAGCTTGGGTATGAC	B4GALNT2__NM_001159388	289	454	347	333
B4GALNT2__NM_001159388_TGGTCCAGGGTCGTATAGCT	B4GALNT2__NM_001159388	0	0	0	0
B4GALNT2__NM_153446_CCCGAGCCGATTCCCACACT	B4GALNT2__NM_153446	2921	2191	2896	2851
B4GALNT2__NM_153446_GCGGCCGGGAATGTGTCTCG	B4GALNT2__NM_153446	1263	1090	1209	1133
B4GALNT2__NM_153446_TTCCGTGGGAAAATTCCACG	B4GALNT2__NM_153446	10	6	31	36
B4GALNT3__NM_173593_CACCTCGAAGTAGTACCTGT	B4GALNT3__NM_173593	1111	760	1152	953
B4GALNT3__NM_173593_CAGGGTCGTTCCGTCGCCAC	B4GALNT3__NM_173593	758	577	824	529
B4GALNT3__NM_173593_CAGTGGCGACGGAACGACCC	B4GALNT3__NM_173593	475	462	573	350
B4GALNT3__NM_173593_CTCCCACAGGTACTACTTCG	B4GALNT3__NM_173593	1091	1101	1145	1042
B4GALNT3__NM_173593_GAGGCCATAGTTGGTCCATT	B4GALNT3__NM_173593	667	696	672	785
B4GALNT3__NM_173593_GGACCGCCCCGGGAGAGTTT	B4GALNT3__NM_173593	431	439	369	227
B4GALNT3__NM_173593_GTATCTGGAACTGGTGGCGT	B4GALNT3__NM_173593	248	247	353	296
B4GALNT3__NM_173593_TCAGGGGGTTCCCGCCGACC	B4GALNT3__NM_173593	248	192	254	130
B4GALNT3__NM_173593_TCCCCCAAATGGACCAACTA	B4GALNT3__NM_173593	0	0	0	0
B4GALNT3__NM_173593_TCTCCAGCTGCCGTACCCTG	B4GALNT3__NM_173593	1155	1401	1282	1064
B4GALNT3__NM_173593_TGGAAAGGAGTGGACCGCCC	B4GALNT3__NM_173593	59	119	79	53
B4GALNT3__NM_173593_TGGTCAATGTCGTGGTCCTA	B4GALNT3__NM_173593	20	0	12	13
B4GALNT4__NM_178537_AAAAAGGTATCCCTGGGATC	B4GALNT4__NM_178537	227	256	274	342
B4GALNT4__NM_178537_ACCAGTCCTCAAACACGTGC	B4GALNT4__NM_178537	15	48	29	17
B4GALNT4__NM_178537_CTCAAAGTAGTACCTCCGGG	B4GALNT4__NM_178537	772	756	792	794
B4GALNT4__NM_178537_CTCCTCTTCACGGCTCTCAC	B4GALNT4__NM_178537	350	260	314	301
B4GALNT4__NM_178537_CTTCCCGATCGCCAGATACC	B4GALNT4__NM_178537	385	685	489	685
B4GALNT4__NM_178537_GAACTCCGAGTTGTCGTCTG	B4GALNT4__NM_178537	1119	1313	1281	1369
B4GALNT4__NM_178537_GGCCCTGGTATCTGGCGATC	B4GALNT4__NM_178537	490	727	586	609
B4GALNT4__NM_178537_GGCTCGGACCACGTGGAAGT	B4GALNT4__NM_178537	983	1214	1224	1589
B4GALNT4__NM_178537_TAGGTGGGGGCGTAGGCGCA	B4GALNT4__NM_178537	64	52	43	47
B4GALNT4__NM_178537_TGGCTCACCTACGTGCACCT	B4GALNT4__NM_178537	3	27	8	1
B4GALNT4__NM_178537_TGTGGCCTCAGACGACAACT	B4GALNT4__NM_178537	530	698	625	553
B4GALNT4__NM_178537_TTCACGGTGGTGCGCGTCTG	B4GALNT4__NM_178537	56	78	123	251
B4GALT1__NM_001497_AAAAACACCTGTACGCATTA	B4GALT1__NM_001497	324	343	337	491
B4GALT1__NM_001497_AACACAGAAGTTACCACTCA	B4GALT1__NM_001497	589	659	717	751
B4GALT1__NM_001497_AATTTAGCAATTCTATCACC	B4GALT1__NM_001497	123	92	126	55
B4GALT1__NM_001497_AGACACAGCCCCCTACACAA	B4GALT1__NM_001497	745	551	769	786
B4GALT1__NM_001497_ATCCACACACATAGGACAAA	B4GALT1__NM_001497	802	1084	927	1091
B4GALT1__NM_001497_CACGGACCAGGCAAACATCC	B4GALT1__NM_001497	672	707	736	687
B4GALT1__NM_001497_CAGGCGGCAGGCCCGCTGTA	B4GALT1__NM_001497	301	312	396	370
B4GALT1__NM_001497_CAGTTCCCCACTCTATACCA	B4GALT1__NM_001497	663	663	745	762
B4GALT1__NM_001497_CATTCAGAAATCAGACAATC	B4GALT1__NM_001497	503	640	776	750
B4GALT1__NM_001497_CCAGGGCCAGAATCCACGAC	B4GALT1__NM_001497	710	529	827	855
B4GALT1__NM_001497_CCATCTGAATGATGAGCGAA	B4GALT1__NM_001497	120	247	108	152
B4GALT1__NM_001497_CCCGCGGCATCTCACGGACC	B4GALT1__NM_001497	1466	1142	1598	1316
B4GALT1__NM_001497_CTCAGACTGGTAAAAATGAG	B4GALT1__NM_001497	115	148	145	111
B4GALT1__NM_001497_CTGGCAGGATAATGCTGACC	B4GALT1__NM_001497	474	447	512	496
B4GALT1__NM_001497_CTTGGGTTTCTCGCATTCAG	B4GALT1__NM_001497	418	668	524	615
B4GALT1__NM_001497_GAGGTGGCACAGGACAGTAA	B4GALT1__NM_001497	116	213	157	198
B4GALT1__NM_001497_GATTGAGTTTAACATGCCTG	B4GALT1__NM_001497	458	459	569	519
B4GALT1__NM_001497_GCCTGGTCCGTGAGATGCCG	B4GALT1__NM_001497	2506	2522	2764	2987
B4GALT1__NM_001497_GCGCATCTCAGATGCTAACT	B4GALT1__NM_001497	241	369	316	509
B4GALT1__NM_001497_GGCCAGCCAGGTAGTAAACG	B4GALT1__NM_001497	321	390	401	276
B4GALT1__NM_001497_GGGCCTGAGGGGTCAGTCTA	B4GALT1__NM_001497	397	774	526	626
B4GALT1__NM_001497_GGTTGTAAATAACAGCAGTC	B4GALT1__NM_001497	61	107	42	37
B4GALT1__NM_001497_GTGTATTTACCATTGTGTAG	B4GALT1__NM_001497	285	131	208	282
B4GALT1__NM_001497_GTGTGTATTTACCATTGTGT	B4GALT1__NM_001497	730	646	871	851
B4GALT1__NM_001497_GTTTCTGTGTATCCTATTGC	B4GALT1__NM_001497	289	358	321	408
B4GALT1__NM_001497_TAACACAGAAGTTACCACTC	B4GALT1__NM_001497	113	39	143	121
B4GALT1__NM_001497_TACCACTGAGCGCCTTTGCC	B4GALT1__NM_001497	731	984	1040	1120
B4GALT1__NM_001497_TCATCATTCCATTCCGCAAC	B4GALT1__NM_001497	408	386	463	507
B4GALT1__NM_001497_TCCAGCCCAGTCGTGGATTC	B4GALT1__NM_001497	283	236	354	300
B4GALT1__NM_001497_TGAATGACCATAATGCGTAC	B4GALT1__NM_001497	287	321	399	379
B4GALT1__NM_001497_TGCCAGGCGCGTCCCTACAG	B4GALT1__NM_001497	426	358	507	426
B4GALT1__NM_001497_TGCTCCTGCCGGTTGCGGAA	B4GALT1__NM_001497	221	226	193	214
B4GALT1__NM_001497_TGGGAGCTATTGAGCCACCT	B4GALT1__NM_001497	221	311	337	331
B4GALT1__NM_001497_TGTATTTACCATTGTGTAGG	B4GALT1__NM_001497	121	195	190	139
B4GALT1__NM_001497_TGTCCCCTAAAACTTGACTG	B4GALT1__NM_001497	358	441	462	499
B4GALT1__NM_001497_TGTGGAGACTCCGACCAGTT	B4GALT1__NM_001497	439	452	503	324
B4GALT1__NM_001497_TGTGGGTGGGAGCCTCAACT	B4GALT1__NM_001497	378	450	406	500
B4GALT1__NM_001497_TGTTAAACTCAATCAGCATG	B4GALT1__NM_001497	13	34	16	10
B4GALT1__NM_001497_TTGCTAAATTGTCGTGAAAT	B4GALT1__NM_001497	99	181	130	86
B4GALT2__NM_001005417_AGCCGCTGCCGCCTCAAGAT	B4GALT2__NM_001005417	1024	482	1104	571
B4GALT2__NM_001005417_AGTGGAGCCAGTAGCGCAGG	B4GALT2__NM_001005417	525	232	507	233

B4GALT2__NM_001005417_CCAAGACCAGGTGGCGAGTC	B4GALT2__NM_001005417	132	130	147	206
B4GALT2__NM_001005417_CCAGGTGGCGAGTCAGGACA	B4GALT2__NM_001005417	87	45	121	238
B4GALT2__NM_001005417_CGGGCCAAGCTGCTTAACGT	B4GALT2__NM_001005417	1428	1307	1483	1166
B4GALT2__NM_001005417_GAAGCCCACGTTAAGCAGCT	B4GALT2__NM_001005417	324	317	302	400
B4GALT2__NM_001005417_GACCGCAACCTATACCGCTG	B4GALT2__NM_001005417	1112	1104	1141	738
B4GALT2__NM_001005417_GGGCGTAGACGTCAAAGTAG	B4GALT2__NM_001005417	338	322	347	314
B4GALT2__NM_001005417_GTGGTGTTCCCGGTGTCTAA	B4GALT2__NM_001005417	36	6	35	15
B4GALT2__NM_001005417_TATAGGTTGCGGTCATCCAT	B4GALT2__NM_001005417	340	250	296	213
B4GALT2__NM_001005417_TCAAGATGGGGTGTAGATAG	B4GALT2__NM_001005417	200	172	208	203
B4GALT2__NM_001005417_TGGTGTTCCCGGTGTCTAAA	B4GALT2__NM_001005417	25	65	33	12
B4GALT3__NM_001199873_AATGATGGCTGTTCGGGAGC	B4GALT3__NM_001199873	400	377	623	388
B4GALT3__NM_001199873_AGGAGATCGTTCTGGACAGT	B4GALT3__NM_001199873	367	175	428	213
B4GALT3__NM_001199873_AGGGCAAAACTGTTGAACGT	B4GALT3__NM_001199873	599	464	584	565
B4GALT3__NM_001199873_AGGTTACTGTAGACATCACG	B4GALT3__NM_001199873	376	248	318	312
B4GALT3__NM_001199873_CACTGGCAGAGATTGTGGAG	B4GALT3__NM_001199873	828	731	801	628
B4GALT3__NM_001199873_CCGGCCCCCTGGTTCTACCC	B4GALT3__NM_001199873	811	704	801	480
B4GALT3__NM_001199873_CCTAAGAGAGGAGATCGTTC	B4GALT3__NM_001199873	1173	727	1419	1032
B4GALT3__NM_001199873_CGGCCCCCTGGTTCTACCCG	B4GALT3__NM_001199873	1080	551	1231	685
B4GALT3__NM_001199873_GCTGGAGCGGCCTTGCACGC	B4GALT3__NM_001199873	326	501	329	387
B4GALT3__NM_001199873_TCATGATGTACCTGTCACTG	B4GALT3__NM_001199873	676	603	740	768
B4GALT3__NM_001199873_TCATTGTGCCTCATCGTGCC	B4GALT3__NM_001199873	931	1498	972	1399
B4GALT3__NM_001199873_TCCCTGATCTCGGCCAAATA	B4GALT3__NM_001199873	175	141	225	130
B4GALT3__NM_003779_ATCATGAAACCGAATTCATG	B4GALT3__NM_003779	285	155	272	135
B4GALT3__NM_003779_GAACTCTGGTGCCGTGCTAG	B4GALT3__NM_003779	868	742	952	658
B4GALT3__NM_003779_GGAGTCCCTGCCCCTAGAGT	B4GALT3__NM_003779	697	496	655	547
B4GALT3__NM_003779_GTTTCATGATTAAGGTAGAC	B4GALT3__NM_003779	157	316	180	148
B4GALT3__NM_003779_TGAACTCTGGTGCCGTGCTA	B4GALT3__NM_003779	255	75	356	262
B4GALT3__NM_003779_TTCACAGTCATAAGCCCTAC	B4GALT3__NM_003779	113	122	142	168
B4GALT4__NM_003778_AAGTCAACAGCAACAGTAAT	B4GALT4__NM_003778	242	287	300	503
B4GALT4__NM_003778_AGGAATGTAAAGCTTTACAG	B4GALT4__NM_003778	171	229	226	189
B4GALT4__NM_003778_ATATCCACTGTAACGTAACC	B4GALT4__NM_003778	681	732	558	836
B4GALT4__NM_003778_CTGCTTTATATTCCACGATG	B4GALT4__NM_003778	396	616	473	588
B4GALT4__NM_003778_CTTTGTGCCTGACAGTGGTT	B4GALT4__NM_003778	254	261	245	352
B4GALT4__NM_003778_GCCACCAGTAACTACTTCGT	B4GALT4__NM_003778	2116	2699	2250	2270
B4GALT4__NM_003778_GGAATGTAAAGCTTTACAGA	B4GALT4__NM_003778	155	117	146	107
B4GALT4__NM_003778_GGCCACCAGTAACTACTTCG	B4GALT4__NM_003778	1559	1738	1678	2449
B4GALT4__NM_003778_GTGTTTCTCTCTGTTCCGGT	B4GALT4__NM_003778	50	7	75	58
B4GALT4__NM_003778_TCGAGCCAAACTCTTGAATG	B4GALT4__NM_003778	2514	3828	3062	4078
B4GALT4__NM_003778_TCGCCATCCTCGTTCCCCAC	B4GALT4__NM_003778	1909	2144	2029	2280
B4GALT4__NM_003778_TTTCAAACCAGATCTCACTT	B4GALT4__NM_003778	312	481	441	866
B4GALT5__NM_004776_AGCCCAAGGCATTCTGATCC	B4GALT5__NM_004776	300	296	414	386
B4GALT5__NM_004776_AGCCTTCTGATTGCATGCCT	B4GALT5__NM_004776	316	308	340	352
B4GALT5__NM_004776_ATGTGGCGCCCGGCATAGGT	B4GALT5__NM_004776	73	55	155	117
B4GALT5__NM_004776_CAAGTCAAGAGGATAATCTA	B4GALT5__NM_004776	163	162	221	253
B4GALT5__NM_004776_CACTCATGTTTATGTCTATT	B4GALT5__NM_004776	1753	2556	2165	2550
B4GALT5__NM_004776_CCAGTGACCTCCGAGCTTGA	B4GALT5__NM_004776	943	577	1087	964
B4GALT5__NM_004776_CTCTTCATGATGCAAGCCCA	B4GALT5__NM_004776	393	438	420	530
B4GALT5__NM_004776_GACATAAACATGAGTGAAAT	B4GALT5__NM_004776	399	350	311	419
B4GALT5__NM_004776_GACCCAACCATCAAGCTCGG	B4GALT5__NM_004776	314	304	245	299
B4GALT5__NM_004776_TCAAGTCAAGAGGATAATCT	B4GALT5__NM_004776	145	140	172	204
B4GALT5__NM_004776_TCACTCATGTTTATGTCTAT	B4GALT5__NM_004776	1184	1879	1516	1978
B4GALT5__NM_004776_TGACCTCCGAGCTTGATGGT	B4GALT5__NM_004776	995	1050	1182	1026
B4GALT6__NM_004775_AAAACATAGGTAATCCCGGT	B4GALT6__NM_004775	535	407	486	730
B4GALT6__NM_004775_AAACAATAGGTCATATGATC	B4GALT6__NM_004775	163	125	238	323
B4GALT6__NM_004775_AAATGAAAAGATACCGAATG	B4GALT6__NM_004775	160	200	232	194
B4GALT6__NM_004775_AACAGTACGCTCAACGGTAC	B4GALT6__NM_004775	131	243	191	185
B4GALT6__NM_004775_AACCCGTATATGTGGACCTA	B4GALT6__NM_004775	466	662	542	451
B4GALT6__NM_004775_AACTTGGAGCGCGCTCTTAG	B4GALT6__NM_004775	118	88	210	95
B4GALT6__NM_004775_AATAAAAACAGTACGCTCAA	B4GALT6__NM_004775	325	328	286	258
B4GALT6__NM_004775_ACAGCTTTCTTAGGCTTTAA	B4GALT6__NM_004775	185	187	208	212
B4GALT6__NM_004775_AGGAAAAACATAGGTAATCC	B4GALT6__NM_004775	151	121	167	80
B4GALT6__NM_004775_AGGATTTAGATATTGAGCCA	B4GALT6__NM_004775	125	121	241	159
B4GALT6__NM_004775_AGGTCCCTATGGCACTAACT	B4GALT6__NM_004775	796	792	742	882
B4GALT6__NM_004775_AGTCAAGAAACCCGTATATG	B4GALT6__NM_004775	241	238	306	356
B4GALT6__NM_004775_AGTCCACAGTGTTTACAGAG	B4GALT6__NM_004775	926	1042	997	1235
B4GALT6__NM_004775_ATCAGATGAGATTGCAAGCA	B4GALT6__NM_004775	172	194	301	292
B4GALT6__NM_004775_CAAAAGACTGTAAACCCAGA	B4GALT6__NM_004775	188	268	207	127
B4GALT6__NM_004775_CACATAGATGAAGTACAGAC	B4GALT6__NM_004775	436	642	463	506
B4GALT6__NM_004775_CTCTTTATGGTACAAGCTCG	B4GALT6__NM_004775	5	6	2	0
B4GALT6__NM_004775_GAAGATGTTCATGGCGATTA	B4GALT6__NM_004775	372	434	356	459
B4GALT6__NM_004775_GAAGGCGAGGAGAGAGCGAT	B4GALT6__NM_004775	136	218	175	171
B4GALT6__NM_004775_GAGGTCCCTATGGCACTAAC	B4GALT6__NM_004775	247	412	344	516
B4GALT6__NM_004775_GGGAAGCCACTACTTAAGAT	B4GALT6__NM_004775	534	634	587	833
B4GALT6__NM_004775_GTAATCGGTTATACCTTTAT	B4GALT6__NM_004775	634	924	816	950
B4GALT6__NM_004775_GTGGCGAGCACCGCAGAAAC	B4GALT6__NM_004775	110	127	168	113
B4GALT6__NM_004775_TAACTTCCGATCTTAAGTAG	B4GALT6__NM_004775	275	429	348	333
B4GALT6__NM_004775_TACATGTTTGCATGAGTGTC	B4GALT6__NM_004775	312	402	289	357
B4GALT6__NM_004775_TACCATAAAGAGATATGTGT	B4GALT6__NM_004775	820	1157	1016	1203
B4GALT6__NM_004775_TATGTGGACCTAGGGCTAAT	B4GALT6__NM_004775	765	896	922	958
B4GALT6__NM_004775_TATTGCCTTCGGGATAATCT	B4GALT6__NM_004775	390	434	421	309
B4GALT6__NM_004775_TCTTGTATTTGCAAGTATGC	B4GALT6__NM_004775	100	170	135	166
B4GALT6__NM_004775_TGTGGCGAGCACCGCAGAAA	B4GALT6__NM_004775	279	204	313	321
B4GALT6__NM_004775_TGTTCATGGCGATTACGGAA	B4GALT6__NM_004775	24	175	49	76
B4GALT7__NM_007255_CACTACAAGACCTATGTCGG	B4GALT7__NM_007255	1005	918	1078	796
B4GALT7__NM_007255_CGAGTTCTACCGGCGCATTA	B4GALT7__NM_007255	585	495	442	728
B4GALT7__NM_007255_CGGGCAGCGCTCATCAACGT	B4GALT7__NM_007255	702	625	715	581
B4GALT7__NM_007255_CTCCAAGCAGCACTACCGGC	B4GALT7__NM_007255	480	256	613	537
B4GALT7__NM_007255_GAGCAGGTCAACGTCGTGCA	B4GALT7__NM_007255	820	927	871	838
B4GALT7__NM_007255_GCGAGGACGACGAGTTCTAC	B4GALT7__NM_007255	1057	967	1264	808
B4GALT7__NM_007255_GCTCAGGAAGCGGCGCATGT	B4GALT7__NM_007255	828	684	895	721
B4GALT7__NM_007255_GGGATGTCCAACCGCTTCTG	B4GALT7__NM_007255	635	629	761	632
B4GALT7__NM_007255_TACCACTACAAGACCTATGT	B4GALT7__NM_007255	848	867	977	781
B4GALT7__NM_007255_TCCTCGAAGCGTTCGCGGAA	B4GALT7__NM_007255	166	262	145	138
B4GALT7__NM_007255_TGGGCTTCTTCTCCCTACTC	B4GALT7__NM_007255	67	17	84	42

B4GALT7__NM_007255_TTCCGTCTTCCACCTGTTCG	B4GALT7__NM_007255	130	151	136	271
B4GAT1__NM_006876_ACGGCTGCCTCGTAACGCGA	B4GAT1__NM_006876	248	279	377	337
B4GAT1__NM_006876_CACGGCTGCCTCGTAACGCG	B4GAT1__NM_006876	1196	1129	1465	1801
B4GAT1__NM_006876_CGGCTGCCTCGTAACGCGAG	B4GAT1__NM_006876	170	141	177	70
B4GAT1__NM_006876_GACCTGGTCCACGGACCGTG	B4GAT1__NM_006876	135	101	130	85
B4GAT1__NM_006876_GCCCGGGATTAATTATGCGC	B4GAT1__NM_006876	781	587	682	611
B4GAT1__NM_006876_GCGCATAATTAATCCCGGGC	B4GAT1__NM_006876	1054	1016	1238	1758
B4GAT1__NM_006876_GCGGCGATTACCGCGTCTAC	B4GAT1__NM_006876	348	226	344	291
B4GAT1__NM_006876_GCTGTCCGTGTCGGTGTTCG	B4GAT1__NM_006876	2531	3315	2802	3722
B4GAT1__NM_006876_GGCGATTACCGCGTCTACAG	B4GAT1__NM_006876	219	175	269	217
B4GAT1__NM_006876_GTGGCCAGGATCACATCGTT	B4GAT1__NM_006876	215	178	241	141
B4GAT1__NM_006876_TGACCTGGTCCACGGACCGT	B4GAT1__NM_006876	301	129	311	274
B4GAT1__NM_006876_TTGACCTGGTCCACGGACCG	B4GAT1__NM_006876	64	18	50	47
B9D1__NM_001243473_AAACAGACGGCGTAGCGCGC	B9D1__NM_001243473	50	66	71	59
B9D1__NM_001243473_AACGATGTGGTTCGAGGCTA	B9D1__NM_001243473	122	95	102	98
B9D1__NM_001243473_ACATCGTTCCCGAACACATC	B9D1__NM_001243473	559	738	746	675
B9D1__NM_001243473_ACGATGTGGTTCGAGGCTAT	B9D1__NM_001243473	179	181	227	145
B9D1__NM_001243473_ATGCCACACGCGCACTCGCG	B9D1__NM_001243473	491	732	453	512
B9D1__NM_001243473_CACTCGCGTGGCCTTCGCGA	B9D1__NM_001243473	91	47	72	111
B9D1__NM_001243473_CATCCAAGAGCCAAGATGTG	B9D1__NM_001243473	62	56	73	95
B9D1__NM_001243473_GCCCTTCTCACCTGGCCGGT	B9D1__NM_001243473	1279	1347	1539	1626
B9D1__NM_001243473_GCGCGGTTGCTAAGAGACGC	B9D1__NM_001243473	280	347	252	372
B9D1__NM_001243473_GTAGATTCTGGGACAAACAT	B9D1__NM_001243473	50	28	80	45
B9D1__NM_001243473_GTCTGTTTCTAGGGCAACGC	B9D1__NM_001243473	68	124	83	45
B9D1__NM_001243473_TGCAGTTTAGACGTAGATTC	B9D1__NM_001243473	139	140	179	216
B9D1__NM_001243475_ACCTTGGGGTCTGTGTACTC	B9D1__NM_001243475	585	545	632	556
B9D1__NM_001243475_CACCTTGGGGTCTGTGTACT	B9D1__NM_001243475	1184	1468	1399	1732
B9D1__NM_015681_GGAAACTGGGCTATGACACT	B9D1__NM_015681	543	803	498	701
B9D1__NM_015681_GTGACCCGTGTCCGTTCTCA	B9D1__NM_015681	978	1017	1053	1125
B9D1__NM_015681_TCCTAGCGTCTTTCTACTCA	B9D1__NM_015681	304	207	397	515
B9D2__NM_030578_AGCCATGTCCCCTATCTGCG	B9D2__NM_030578	1272	1084	1446	1577
B9D2__NM_030578_AGTGGACACCCCGCAGATAG	B9D2__NM_030578	400	453	511	446
B9D2__NM_030578_CACCCCGCAGATAGGGGACA	B9D2__NM_030578	619	685	676	754
B9D2__NM_030578_CACGGGCTTTCGTGGGTGGT	B9D2__NM_030578	1662	1693	1965	2416
B9D2__NM_030578_CTGTTCTCGCCAACTGCCCA	B9D2__NM_030578	65	174	72	43
B9D2__NM_030578_GAAGTGCAGGTCGATGGGGT	B9D2__NM_030578	1669	1733	1981	2005
B9D2__NM_030578_GCACGGGCTTTCGTGGGTGG	B9D2__NM_030578	920	1403	1219	1541
B9D2__NM_030578_GCGGGAGGGCCAAACGCAAG	B9D2__NM_030578	168	69	230	239
B9D2__NM_030578_GCGGGGTGTCCACTTGCGTT	B9D2__NM_030578	2683	3222	3153	3782
B9D2__NM_030578_GCTACTTTCCGAGAAACCGC	B9D2__NM_030578	288	442	441	278
B9D2__NM_030578_GGAAGCTCCTGTCAGGCGTG	B9D2__NM_030578	762	950	774	953
B9D2__NM_030578_TAAGCCATGTCCCCTATCTG	B9D2__NM_030578	1027	1151	1244	1393
BAALC__NM_001024372_AGCCCCGCTACTACGAGAGC	BAALC__NM_001024372	199	238	275	218
BAALC__NM_001024372_CGGCGCGTCCGAGTCGGTGT	BAALC__NM_001024372	2964	3022	3088	2620
BAALC__NM_001024372_GGGTCCAGCTCTCGTAGTAG	BAALC__NM_001024372	478	502	448	573
BAALC__NM_001024372_GTCCAGCTCTCGTAGTAGCG	BAALC__NM_001024372	341	294	391	306
BAALC__NM_024812_CCACCTGGGGCTGTAGATCG	BAALC__NM_024812	505	685	522	573
BAALC__NM_024812_GATGCTATCTGTTACATTAA	BAALC__NM_024812	364	603	440	491
BAALC__NM_024812_GCTCTGGGGATTTGGGCACT	BAALC__NM_024812	743	899	884	987
BAAT__NM_001127610_ACACGAATTAAGGTTCGAGA	BAAT__NM_001127610	317	477	417	329
BAAT__NM_001127610_ACCTGGTGTCACACGAATTA	BAAT__NM_001127610	340	232	374	393
BAAT__NM_001127610_ACCTTAATTCGTGTGACACC	BAAT__NM_001127610	586	608	751	889
BAAT__NM_001127610_CCAGTGCATATCCGAGCTAC	BAAT__NM_001127610	539	569	613	775
BAAT__NM_001127610_CGAATTCATTGGCCCTATAG	BAAT__NM_001127610	355	303	501	349
BAAT__NM_001127610_GAATTCATTGGCCCTATAGT	BAAT__NM_001127610	404	386	431	357
BAAT__NM_001127610_GAGAGGTGGTATGTGGCACC	BAAT__NM_001127610	154	70	147	132
BAAT__NM_001127610_GGCCAATGAATTCGGTGAGG	BAAT__NM_001127610	1045	1021	1082	1309
BAAT__NM_001127610_GTCCACCTCACCGAATTCAT	BAAT__NM_001127610	985	1038	1057	1213
BAAT__NM_001127610_TCACAGGGGTAGCTGTCAAC	BAAT__NM_001127610	391	445	407	346
BAAT__NM_001127610_TTATTCTCAAGCCCACTATA	BAAT__NM_001127610	101	293	39	58
BAAT__NM_001127610_TTCACTTGGAGGGGATTATA	BAAT__NM_001127610	32	44	25	30
BABAM1__NM_001033549_AGGTCCAAATTCGGACACCA	BABAM1__NM_001033549	178	121	187	252
BABAM1__NM_001033549_CAGGAGCCGGCCCTAAGTCC	BABAM1__NM_001033549	724	497	818	478
BABAM1__NM_001033549_CCACCACCAGTGCAAACTCG	BABAM1__NM_001033549	854	1010	893	1069
BABAM1__NM_001033549_GACGATTCCCCCGCCATATG	BABAM1__NM_001033549	608	525	929	776
BABAM1__NM_001033549_GCCCAGCAGCCCCACTGAAG	BABAM1__NM_001033549	101	84	86	67
BABAM1__NM_001033549_GGGCTGAGGACCGGGCAGTA	BABAM1__NM_001033549	911	689	1185	1209
BABAM1__NM_001033549_GGTCCAAATTCGGACACCAA	BABAM1__NM_001033549	149	270	174	170
BABAM1__NM_001033549_GGTGCGGACCACATATGGCG	BABAM1__NM_001033549	724	994	871	711
BABAM1__NM_001033549_GGTGGTGGTGAACGATGACA	BABAM1__NM_001033549	83	18	93	111
BABAM1__NM_001033549_GTGCGGACCACATATGGCGG	BABAM1__NM_001033549	685	443	846	423
BABAM1__NM_001033549_TGACCCTTGGTGTCCGAATT	BABAM1__NM_001033549	648	910	774	942
BABAM1__NM_001033549_TGGTGCGGACCACATATGGC	BABAM1__NM_001033549	22	44	38	22
BABAM1__NM_001288757_AGGATGGTGCGGACCACATA	BABAM1__NM_001288757	340	193	406	273
BABAM1__NM_001288757_ATGGTGCGGACCACATATGG	BABAM1__NM_001288757	759	442	679	352
BACE1__NM_001207048_AAAGGCTCCAGGGAGTCGTC	BACE1__NM_001207048	272	191	217	203
BACE1__NM_001207048_AAGTGCTGGCCTCTGTCGGA	BACE1__NM_001207048	903	624	973	620
BACE1__NM_001207048_AGATAGATGAAGGGAACCAT	BACE1__NM_001207048	120	106	163	119
BACE1__NM_001207048_ATCGACCACTCGCTGTACAC	BACE1__NM_001207048	1034	759	1149	1057
BACE1__NM_001207048_CACATACCGGGACCTCCGGA	BACE1__NM_001207048	1081	1228	1162	1316
BACE1__NM_001207048_CCAGCACTTCAGACTGGTTG	BACE1__NM_001207048	1407	2217	1564	2037
BACE1__NM_001207048_CCGGAGGTCCCGGTATGTGC	BACE1__NM_001207048	3330	2747	3509	3411
BACE1__NM_001207048_GCCCACTTTACACTCTGTTC	BACE1__NM_001207048	1007	1033	1155	1571
BACE1__NM_001207048_GCGGGTGGAGATCAATGGAC	BACE1__NM_001207048	444	527	596	520
BACE1__NM_001207048_GTGGGCTTGCAGATAGATGA	BACE1__NM_001207048	121	204	205	187
BACE1__NM_001207048_TGGGCTTGCAGATAGATGAA	BACE1__NM_001207048	22	43	21	19
BACE1__NM_001207048_TTCAGTGATGGCAGCAATGT	BACE1__NM_001207048	120	152	110	156
BACE1__NM_001207049_CTTGGGCAAACGAAGGTTGG	BACE1__NM_001207049	83	109	84	86
BACE1__NM_012104_CTCAACATCCTGGTGGATAC	BACE1__NM_012104	220	288	291	213
BACE1__NM_012104_CTGGTAGTAGCGATGCAGGA	BACE1__NM_012104	252	266	309	290
BACE1__NM_012104_GACAACCTGAGGGGCAAGTC	BACE1__NM_012104	489	367	482	508
BACE1__NM_012104_GCAGCCCCCGCAGACGGTA	BACE1__NM_012104	409	248	439	433
BACE1__NM_012104_GCCTCTGGTAGTAGCGATGC	BACE1__NM_012104	201	204	293	240
BACE1__NM_012104_GGAGATGGTGGACAACCTGA	BACE1__NM_012104	939	783	1072	1014

BACE1__NM_012104_GGCTCTTCGTCGGTCTCCCG	BACE1__NM_012104	307	307	376	334
BACE2__NM_012105_CCGGGGTGGGCGCAACTACG	BACE2__NM_012105	528	714	455	705
BACE2__NM_012105_GAAGCTGGACGGGCTTCGTT	BACE2__NM_012105	1379	1707	1745	1631
BACE2__NM_012105_GCCGGCTCCACACATCTGCA	BACE2__NM_012105	2266	2905	2559	3405
BACE2__NM_012105_GCTTGCCCGTTGCTGGATCT	BACE2__NM_012105	898	1191	1085	1189
[illegible]	[illegible]	[illegible]	[illegible]	[illegible]	[illegible]

[illegible]

BCLAF1__NM_001077440_GAGTGCCTTCTATTCCAGAC	BCLAF1__NM_001077440	962	860	1107	1127
BCLAF1__NM_001077440_GGTAGAGGGTATTATCAAGG	BCLAF1__NM_001077440	228	165	207	118
BCO1__NM_017429_ACCCTGCTCCGCAATGGGCC	BCO1__NM_017429	140	262	202	205
BCO1__NM_017429_AGATACAACCATTGGTTCGA	BCO1__NM_017429	230	298	396	436
BCO1__NM_017429_AGGCAGTTGTCGGTGAAATC	BCO1__NM_017429	312	480	352	388
BCO1__NM_017429_AGGGAACCCTGCTCCGCAAT	BCO1__NM_017429	914	1145	1085	1211
BCO1__NM_017429_CAGGAATAGGAAAGAACAGC	BCO1__NM_017429	291	192	222	227
BCO1__NM_017429_CAGGCAGTTGTCGGTGAAAT	BCO1__NM_017429	1289	1703	1559	1990
BCO1__NM_017429_GGCAGTTGTCGGTGAAATCG	BCO1__NM_017429	933	1069	1288	1450
BCO1__NM_017429_GGGAGTCCAGATACAACCAT	BCO1__NM_017429	518	564	567	491
BCO1__NM_017429_GGGCAAGGCCGTCGAACCAA	BCO1__NM_017429	458	320	482	538
BCO1__NM_017429_GGTTGATTATCGTAAATACG	BCO1__NM_017429	311	282	220	268
BCO1__NM_017429_GTCGAACCAATGGTTGTATC	BCO1__NM_017429	1309	1166	1444	1392
BCO1__NM_017429_TTGCAGGTGAAGTCTATTAC	BCO1__NM_017429	153	84	150	159
BCO2__NM_001037290_AATGGCTCTCTACTTCGAAT	BCO2__NM_001037290	221	202	283	171
BCO2__NM_001037290_AGGTACAATCATTGGTTTGA	BCO2__NM_001037290	188	318	208	331
BCO2__NM_001037290_CAAATTCTGAGATCACAATT	BCO2__NM_001037290	542	577	533	681
BCO2__NM_001037290_CGAATTGTGATCTCAGAATT	BCO2__NM_001037290	1254	1591	1305	1788
BCO2__NM_001037290_CTCTACTTCGAATTGGACCT	BCO2__NM_001037290	250	483	277	283
BCO2__NM_001037290_GGACCTGGGAAATTCGAGTT	BCO2__NM_001037290	647	657	662	649
BCO2__NM_001037290_GGCATCTCTGCTCGAGTCTG	BCO2__NM_001037290	512	488	493	767
BCO2__NM_001037290_GGGCATCTCTGCTCGAGTCT	BCO2__NM_001037290	397	477	510	655
BCO2__NM_001037290_TCTACAGAGTGATACATATA	BCO2__NM_001037290	243	283	227	252
BCO2__NM_001037290_TCTCTACTTCGAATTGGACC	BCO2__NM_001037290	259	162	270	286
BCO2__NM_001037290_TGCACATCCTCATTATGACC	BCO2__NM_001037290	181	166	170	117
BCO2__NM_001037290_TTTACCAGGCAGCTCAAACC	BCO2__NM_001037290	221	226	212	202
BCO2__NM_001256398_CAGAATGACTCCTGATAAAA	BCO2__NM_001256398	415	447	609	651
BCO2__NM_001256400_AGGAACCCGAATAACCTTAT	BCO2__NM_001256400	718	1104	1048	1041
BCO2__NM_001256400_ATGGAACAGCATACAATATG	BCO2__NM_001256400	47	126	93	101
BCO2__NM_001256400_CACTTCTAAAATTCGGGGAA	BCO2__NM_001256400	209	295	258	284
BCO2__NM_001256400_CTATTGCTTCTACAGAGAAA	BCO2__NM_001256400	16	10	46	45
BCO2__NM_001256400_GATGGAACAGCATACAATAT	BCO2__NM_001256400	93	146	124	94
BCO2__NM_001256400_GGATGGAACAGCATACAATA	BCO2__NM_001256400	89	100	130	138
BCO2__NM_001256400_GTTTCTCCTATAAGGTTATT	BCO2__NM_001256400	39	3	67	83
BCOR__NM_001123383_AAGCGTCACCATCATTTACA	BCOR__NM_001123383	488	447	527	581
BCOR__NM_001123383_AAGGCCACCTAGAGTGCTTG	BCOR__NM_001123383	1112	1361	1174	1159
BCOR__NM_001123383_ACAGCTGGATGAACAGCGAG	BCOR__NM_001123383	59	83	138	111
[illegible]					

BPIFC__NM_174932_GTTCTCCGGAAAATGAGACG	BPIFC__NM_174932	53	112	80	77
BPIFC__NM_174932_TCAAGGCAAGGATTACTCAG	BPIFC__NM_174932	103	210	112	129
BPIFC__NM_174932_TCAGTTGGGCGTAGCAATCT	BPIFC__NM_174932	131	141	110	79
BPIFC__NM_174932_TCCAGGGTAAATGGTCTGAG	BPIFC__NM_174932	754	823	943	1243
BPIFC__NM_174932_TTCATTTCCAAATACCTCAT	BPIFC__NM_174932	55	72	81	128
BPIFC__NM_174932_TTCTCCGGAAAATGAGACGT	BPIFC__NM_174932	0	0	0	0
BPNT1__NM_001286149_CAGGACCAGATGCTGTGTTG	BPNT1__NM_001286149	2107	1925	2067	2188
BPNT1__NM_001286149_CAGTAACCAACTTGTTGCTA	BPNT1__NM_001286149	758	1031	838	1067
BPNT1__NM_001286149_CCCCAAACTCACAATTATAG	BPNT1__NM_001286149	690	935	708	666
BPNT1__NM_001286149_CCTCATAGTTGTAATATGGC	BPNT1__NM_001286149	697	898	762	981
BPNT1__NM_001286149_CTGTGTTGGGGAGGACAATC	BPNT1__NM_001286149	319	434	366	386
BPNT1__NM_001286149_CTTATTGGAATTGCTTATGA	BPNT1__NM_001286149	669	788	730	947
BPNT1__NM_001286149_GCTTATGTATTTGCAAGTCC	BPNT1__NM_001286149	1274	1318	1510	1670
BPNT1__NM_001286149_GGAACCAAGGAATATACCGA	BPNT1__NM_001286149	346	217	372	417
BPNT1__NM_001286149_TAATAGCACTGTACTGCGAT	BPNT1__NM_001286149	238	175	264	252
BPNT1__NM_001286149_TATGAAGGAAAAGCCATAGC	BPNT1__NM_001286149	38	51	27	46
BPNT1__NM_001286149_TCGATCCCATAGCAACAAGT	BPNT1__NM_001286149	250	439	336	432
BPNT1__NM_001286149_TGTGTTGGGGAGGACAATCT	BPNT1__NM_001286149	523	390	461	521
BPNT1__NM_001286150_CGCATATTCTATTGCTCAAA	BPNT1__NM_001286150	580	820	700	938
BPNT1__NM_001286150_TAACACTGTGTTGATGCGGT	BPNT1__NM_001286150	739	698	798	893
BPNT1__NM_001286150_TTGAGCAATAGAATATGCGG	BPNT1__NM_001286150	344	238	407	330
BPTF__NM_004459_AGCAGGTCCAAAGGTAGTAT	BPTF__NM_004459	61	118	91	50
BPTF__NM_004459_CCACACTGTATTTCATAGAT	BPTF__NM_004459	498	580	535	485
BPTF__NM_004459_CGACGATGACTCCGATTATC	BPTF__NM_004459	794	603	701	605
BPTF__NM_004459_CGATGACTCCGATTATCCGG	BPTF__NM_004459	970	709	976	655
BPTF__NM_004459_GAATGTCATTGCCATTTACG	BPTF__NM_004459	481	374	546	468
BPTF__NM_004459_GACTTGGGAAATTCAAGGGG	BPTF__NM_004459	64	42	83	48
BPTF__NM_004459_GAGTGCTGCTGTAGGTACTA	BPTF__NM_004459	550	346	465	184
BPTF__NM_004459_GGCGAAAACCAAGAGTACAT	BPTF__NM_004459	816	716	776	354
BPTF__NM_004459_GTTCCGCAGTACCTCGTAAA	BPTF__NM_004459	673	311	911	313
BPTF__NM_004459_TACGAGGTACTGCGGAACTT	BPTF__NM_004459	614	411	465	239
BPTF__NM_004459_TCTTCCAATATAGGAGAACG	BPTF__NM_004459	107	212	175	188
BPTF__NM_004459_TTCCATCTCCTCCGGATAAT	BPTF__NM_004459	3	0	10	3
BRAF__NM_004333_ACAAATGATTAAGTTGACAC	BRAF__NM_004333	650	768	800	1054
BRAF__NM_004333_CCACACCTTGCAGGTACCTA	BRAF__NM_004333	1857	1515	1906	2209
BRAF__NM_004333_CCGGGCCAGGCTCTGTTCAA	BRAF__NM_004333	2173	1973	2590	2494
BRAF__NM_004333_CTTACCTCCAGATATATTGA	BRAF__NM_004333	542	489	699	659
BRAF__NM_004333_GAGAGAAGAAACCAATTGGT	BRAF__NM_004333	1903	1300	2129	1900
BRAF__NM_004333_GCCCTATTGGACAAATTTGG	BRAF__NM_004333	1266	1107	1316	1274
BRAF__NM_004333_GGCAGGAAGACTCTAACGAT	BRAF__NM_004333	2230	2347	2728	2922
BRAF__NM_004333_GGGCCAGGCTCTGTTCAACG	BRAF__NM_004333	2081	1920	2175	1978
BRAF__NM_004333_GTTGACACAGGAACATATAG	BRAF__NM_004333	452	309	507	406
BRAF__NM_004333_TAGCACTGAAAGGCTAGAAG	BRAF__NM_004333	529	443	634	521
BRAF__NM_004333_TCTTCCTGCCCAACAAACAG	BRAF__NM_004333	31	31	38	90
BRAF__NM_004333_TTGGAGTGCATCTAGCTTGC	BRAF__NM_004333	362	464	465	503
BRAP__NM_006768_AAGAAGTAAAGATCACAGTA	BRAP__NM_006768	231	231	251	169
BRAP__NM_006768_AGTGTTCCGCGAGCTCCAAT	BRAP__NM_006768	248	368	294	258
BRAP__NM_006768_ATCAGCACCATATATTGGTT	BRAP__NM_006768	768	642	913	634
BRAP__NM_006768_CCATCAGTTGAAATAGTTCA	BRAP__NM_006768	674	628	768	820
BRAP__NM_006768_CTCTACTCCCAACCAATATA	BRAP__NM_006768	68	90	70	38
BRAP__NM_006768_CTTTACTTCTTTGCGCAGTG	BRAP__NM_006768	93	137	108	106
BRAP__NM_006768_GCTGATAAAGTTTCGTGCAC	BRAP__NM_006768	891	961	1108	1048
BRAP__NM_006768_GGCTTCGGCTTCAGCGCCGC	BRAP__NM_006768	99	147	134	166
BRAP__NM_006768_TATCAGCACCATATATTGGT	BRAP__NM_006768	528	199	577	234
BRAP__NM_006768_TCACATCTGTCATTTCTCGA	BRAP__NM_006768	1309	1300	1497	1132
BRAP__NM_006768_TCTTTACTTCTTTGCGCAGT	BRAP__NM_006768	560	895	729	1094
BRAP__NM_006768_TGGTGACTTGCCTTCTAAAC	BRAP__NM_006768	60	42	58	69
BRAT1__NM_152743_AAAGTCCAGGACCTGAGTTC	BRAT1__NM_152743	529	153	533	171
BRAT1__NM_152743_AGATGGTGTCGACCGCACCT	BRAT1__NM_152743	912	310	1063	296
BRAT1__NM_152743_AGCGCCCGAAGGTCGTGGTC	BRAT1__NM_152743	534	387	661	524
BRAT1__NM_152743_CAGCAGGGGGAGTTACTACC	BRAT1__NM_152743	305	252	343	204
BRAT1__NM_152743_CAGGTCCACGAACGAGTGTG	BRAT1__NM_152743	378	79	432	265
BRAT1__NM_152743_CCCCGCCGCACACTCGTTCG	BRAT1__NM_152743	2648	3069	2761	3552
BRAT1__NM_152743_CCGTCGGAAGAACTGAACAC	BRAT1__NM_152743	676	550	700	818
BRAT1__NM_152743_GGCCCAGGTTGCTCGGCCGA	BRAT1__NM_152743	347	560	446	560
BRAT1__NM_152743_GGGAGCAGCTGGGCGCATTC	BRAT1__NM_152743	505	203	596	297
BRAT1__NM_152743_GTCCACGAACGAGTGTGCGG	BRAT1__NM_152743	442	220	620	302
BRAT1__NM_152743_GTCCTGGCTTTGTCCATGCG	BRAT1__NM_152743	1249	656	1368	829
BRAT1__NM_152743_TCTCCAGCAGACAGGCCACG	BRAT1__NM_152743	742	91	807	204
BRCA1__NM_007294_AACCAGGGATGAAATCAGTT	BRCA1__NM_007294	498	658	665	511
BRCA1__NM_007294_AAGGAGCCAACATAACAGAT	BRCA1__NM_007294	144	30	191	86
BRCA1__NM_007294_CAGTTCCAAGGTTAGAGAGT	BRCA1__NM_007294	272	134	280	190
BRCA1__NM_007294_GACGTCTGTCTACATTGAAT	BRCA1__NM_007294	830	1021	901	1066
BRCA1__NM_007294_GGTGTTCAAATCATTATTAC	BRCA1__NM_007294	302	453	314	290
BRCA1__NM_007294_GGTTTCTGTAGCCCATACTT	BRCA1__NM_007294	144	0	118	53
BRCA1__NM_007294_TAACAGATGGGCTGGAAGTA	BRCA1__NM_007294	265	78	293	51
BRCA1__NM_007294_TGTTGGCTCCTTGCTAAGCC	BRCA1__NM_007294	223	219	154	178
BRCA1__NM_007294_TTAATAAGGCAACTTATTGC	BRCA1__NM_007294	118	30	104	55
BRCA1__NM_007294_TTCCAGCCCATCTGTTATGT	BRCA1__NM_007294	175	172	194	172
BRCA1__NM_007294_TTCTGAAGATACCGTTAATA	BRCA1__NM_007294	15	14	2	7
BRCA1__NM_007294_TTGTTACAAATCACCCCTCA	BRCA1__NM_007294	311	178	414	398
BRCA1__NM_007298_AAGAGTCACTTATGATGGAA	BRCA1__NM_007298	226	102	156	95
BRCA1__NM_007298_AAGCTGTGTTAGAACAGCAT	BRCA1__NM_007298	190	56	187	85
BRCA1__NM_007298_ATGGAAGGGTAGCTGTTAGA	BRCA1__NM_007298	181	195	161	154
BRCA1__NM_007298_GAAGCTGTGTTAGAACAGCA	BRCA1__NM_007298	177	79	201	60
BRCA2__NM_000059_AATAGCTGCAAAGACCACAT	BRCA2__NM_000059	309	193	290	139
BRCA2__NM_000059_ACAAACTCCCACATACCACT	BRCA2__NM_000059	811	731	890	599
BRCA2__NM_000059_ACTTCATCTTCTAGGACATT	BRCA2__NM_000059	649	601	700	506
BRCA2__NM_000059_CCTTGCTCTTTGAATATTAT	BRCA2__NM_000059	175	110	211	194
BRCA2__NM_000059_CTATGAGCACAGTAGAACTA	BRCA2__NM_000059	310	190	339	97
BRCA2__NM_000059_GAAGCCAGCTGATTATAAGA	BRCA2__NM_000059	155	40	172	18
BRCA2__NM_000059_GAGCACAGTAGAACTAAGGG	BRCA2__NM_000059	272	163	336	179
BRCA2__NM_000059_GCAGGTTCAGAATTATAGGG	BRCA2__NM_000059	155	42	113	85
BRCA2__NM_000059_TAGGACCAATAAGTCTTAAT	BRCA2__NM_000059	121	30	151	66
BRCA2__NM_000059_TATGAGCACAGTAGAACTAA	BRCA2__NM_000059	257	211	298	98
BRCA2__NM_000059_TGGTGTACATTGTAGAACAAC	BRCA2__NM_000059	108	98	157	87
BRCA2__NM_000059_TTGGCATTGACTTTCCAATG	BRCA2__NM_000059	361	144	332	232
BRCC3__NM_001018055_AATGGGGCTGTGCATAGGGG	BRCC3__NM_001018055	161	154	251	172
BRCC3__NM_001018055_ACAGAGAAGGAGGAAGTAAT	BRCC3__NM_001018055	651	672	541	482
BRCC3__NM_001018055_ACCAGCCCACAACTCTCATG	BRCC3__NM_001018055	1064	886	1218	503
BRCC3__NM_001018055_AGGCCGCCCCATGAGAGTTG	BRCC3__NM_001018055	666	625	706	445
BRCC3__NM_001018055_AGTTCCAGTATATGCAAATT	BRCC3__NM_001018055	1294	888	1375	1319
BRCC3__NM_001018055_ATACCAGCCCACAACTCTCA	BRCC3__NM_001018055	734	517	757	491
BRCC3__NM_001018055_GAGCGTGGTTGAGACAAACG	BRCC3__NM_001018055	481	632	516	469
BRCC3__NM_001018055_GCCAAACAGTTATATGAGGA	BRCC3__NM_001018055	820	1218	982	1158
BRCC3__NM_001018055_GGCCGCCCCATGAGAGTTGT	BRCC3__NM_001018055	1676	1871	1815	1943
BRCC3__NM_001018055_TACCAGCCCACAACTCTCAT	BRCC3__NM_001018055	1162	989	1351	1122
BRCC3__NM_001018055_TCTAGTTGAACGATGATACA	BRCC3__NM_001018055	200	92	247	315
BRCC3__NM_001018055_TCTTACGACGTTCTGATAAG	BRCC3__NM_001018055	160	106	159	161
BRD1__NM_014577_AAACATGCTCTGCGACACGG	BRD1__NM_014577	836	471	966	806
BRD1__NM_014577_AAATTGAAGGGCGCTTGCAC	BRD1__NM_014577	432	236	435	158
BRD1__NM_014577_ACATGCTCTGCGACACGGCG	BRD1__NM_014577	395	611	346	561
BRD1__NM_014577_AGGTCAGCGTTTCTCGCGTA	BRD1__NM_014577	410	293	433	419
BRD1__NM_014577_ATGGTAGAGATAGAAATTGA	BRD1__NM_014577	153	87	118	85
BRD1__NM_014577_CATCCTTTGAGCTTGAGCGT	BRD1__NM_014577	686	454	760	357
BRD1__NM_014577_CGCTTAGTTCTTAAGCAGAC	BRD1__NM_014577	69	82	115	84
BRD1__NM_014577_GCAACAAGGAAAACAGCGAG	BRD1__NM_014577	127	163	102	168
BRD1__NM_014577_GCAGCAGTCTCTGATCGACG	BRD1__NM_014577	1513	1126	1715	975
BRD1__NM_014577_GGGAGTGTTAACACTGCAT	BRD1__NM_014577	624	486	640	682
BRD1__NM_014577_TGATCCCCTGGAGATCATAT	BRD1__NM_014577	395	124	461	258
BRD1__NM_014577_TTAGTTCTTAAGCAGACAGG	BRD1__NM_014577	4	0	10	1
BRD3__NM_007371_AAAGGGCAAAGGTCGGAAGC	BRD3__NM_007371	130	124	121	79
BRD3__NM_007371_ACGGGCGTCTGGGAGACGGT	BRD3__NM_007371	571	405	741	593
BRD3__NM_007371_AGCTGGTTGGTCTTGCGGCC	BRD3__NM_007371	507	844	412	561
BRD3__NM_007371_CACAGATGACATAGTGCTAA	BRD3__NM_007371	237	179	217	255
BRD3__NM_007371_CATTAGCACTATGTCATCTG	BRD3__NM_007371	1561	1138	1777	1784
BRD3__NM_007371_CCGGGGGGGGTGGGTTCACA	BRD3__NM_007371	501	477	657	687
BRD3__NM_007371_GGCAGCGATGACGGGCGTCT	BRD3__NM_007371	1287	1773	1214	1761
BRD3__NM_007371_GGTGGGGGGCACGCTCTGAA	BRD3__NM_007371	394	525	413	523
BRD3__NM_007371_GTTCAATTTGATTGCGTCCA	BRD3__NM_007371	51	49	81	83
BRD3__NM_007371_TTAAAAACCCAATGGATATG	BRD3__NM_007371	17	54	42	41
BRD3__NM_007371_TTCAATTTGATTGCGTCCAC	BRD3__NM_007371	3	18	8	19
BRD3__NM_007371_TTGCAGTGATGGTTGGTACA	BRD3__NM_007371	233	234	227	302
BRD4__NM_014299_AATCTGCCAGTAATGGGGGA	BRD4__NM_014299	146	86	226	174
BRD4__NM_014299_AGCAAAACCTGGCGTTTCCA	BRD4__NM_014299	622	290	616	264
BRD4__NM_014299_CCTCTTGGGCTTGTTAGGGT	BRD4__NM_014299	897	1296	982	1116
BRD4__NM_014299_GAGAAATCTGCCAGTAATGG	BRD4__NM_014299	352	184	280	127
BRD4__NM_014299_GCTTGGAAAACAACTATTAC	BRD4__NM_014299	264	45	346	55
BRD4__NM_014299_GGGAACAATAAAGAAGCGCT	BRD4__NM_014299	200	100	177	210
BRD4__NM_014299_TAAGATCATTAAAACGCCTA	BRD4__NM_014299	94	3	151	11
BRD4__NM_014299_TATGGAAACACCAGTTTGCA	BRD4__NM_014299	338	27	328	27
BRD4__NM_014299_TGCCTGGACTATCATGATCT	BRD4__NM_014299	838	396	771	369
BRD4__NM_014299_TGGAGATGACATAGTCTTAA	BRD4__NM_014299	10	0	0	0
BRD4__NM_014299_TTCAGCTTGACGGCATCCAC	BRD4__NM_014299	83	37	76	19
BRD4__NM_014299_TTGAGTTGTGTTTGGTACCG	BRD4__NM_014299	406	457	454	643
BRD4__NM_058243_AAAAGGGAATAGTGCCGTGG	BRD4__NM_058243	304	201	281	308
BRD4__NM_058243_AAGGGCCTCTGCCCCGCATG	BRD4__NM_058243	144	65	180	81
BRD4__NM_058243_ACTTTCCATAGTGTTAGGGG	BRD4__NM_058243	110	71	99	84
BRD4__NM_058243_AGAGAGCGGCTCTCAATTAA	BRD4__NM_058243	522	236	495	352
BRD4__NM_058243_AGGAGACGATCACACCATTT	BRD4__NM_058243	1233	820	1276	763
BRD4__NM_058243_AGGGTCGGGGGGTCTGTTCA	BRD4__NM_058243	688	765	735	919
BRD4__NM_058243_CACCACCGCCCCTAACACTA	BRD4__NM_058243	1864	1867	2044	1808
BRD4__NM_058243_CATTGACTTTCCATAGTGTT	BRD4__NM_058243	830	1180	911	1240
BRD4__NM_058243_CCCCGGGCATAGCATGCAGG	BRD4__NM_058243	199	317	275	212
BRD4__NM_058243_CCTCCTGCATGCTATGCCCG	BRD4__NM_058243	1170	833	1138	852
BRD4__NM_058243_CGTCTGTCCGACTGGCCGTA	BRD4__NM_058243	1392	1621	1631	1824
BRD4__NM_058243_CTGCCCCGGGCATAGCATGC	BRD4__NM_058243	44	17	75	89
BRD4__NM_058243_CTGTCTGCCTTACGGCCAGT	BRD4__NM_058243	285	294	372	358
BRD4__NM_058243_GCTCTCAATTAAAGGCTTGT	BRD4__NM_058243	674	1024	830	1325
BRD4__NM_058243_GCTGAGCGGACGTCCTGTGA	BRD4__NM_058243	4119	4025	3985	3575
BRD4__NM_058243_GGCACTGCTTATCTCCGAAA	BRD4__NM_058243	733	1335	957	1278
BRD4__NM_058243_GGCTCTCAATTAAAGGCTTG	BRD4__NM_058243	239	367	260	403
BRD4__NM_058243_GGGAAGGAACTGACTTTGGT	BRD4__NM_058243	793	783	882	883
BRD4__NM_058243_GTAACAAGGCGTGTGCTGAG	BRD4__NM_058243	22	33	14	10
BRD4__NM_058243_TGCAAAAGGGAATAGTGCCG	BRD4__NM_058243	374	380	502	488
BRD4__NM_058243_TTGACTTTCCATAGTGTTAG	BRD4__NM_058243	272	307	339	434
BRD7__NM_001173984_AACTGATGAGACAATTGCAG	BRD7__NM_001173984	195	139	216	287
BRD7__NM_001173984_ACCAGATGGAACAACGACGT	BRD7__NM_001173984	303	460	391	222
BRD7__NM_001173984_CATGATCATTAAACACCCAA	BRD7__NM_001173984	88	28	138	58
BRD7__NM_001173984_CATTGGGTGTTTAATGATCA	BRD7__NM_001173984	491	669	395	344
BRD7__NM_001173984_CCAGATGGAACAACGACGTT	BRD7__NM_001173984	176	212	225	115
BRD7__NM_001173984_CGAAGTCACCGAACTCTCCA	BRD7__NM_001173984	1017	1655	1160	1274
BRD7__NM_001173984_CTCCAGATTCCTTCACGATG	BRD7__NM_001173984	639	729	762	553
BRD7__NM_001173984_CTCTGGTTTATTGTAAATCA	BRD7__NM_001173984	146	91	152	254
BRD7__NM_001173984_GCAGCTTGACCGCATCGTGA	BRD7__NM_001173984	657	636	769	568
BRD7__NM_001173984_GGAACAGACACCTCACAGAG	BRD7__NM_001173984	280	229	271	314
BRD7__NM_001173984_GTCGGACAAACACCTCTACG	BRD7__NM_001173984	954	1023	963	943
BRD7__NM_001173984_GTTGTCATTCCCAGTCTCAC	BRD7__NM_001173984	966	452	919	529
BRD8__NM_001164326_AAGAGGAGGCTGAAGTAAAG	BRD8__NM_001164326	395	197	488	289
BRD8__NM_001164326_AGAGGTAGCCTCTTCCATAG	BRD8__NM_001164326	193	129	165	112
BRD8__NM_001164326_AGGAAACCCAGGAGAGATAT	BRD8__NM_001164326	221	40	210	43
BRD8__NM_001164326_ATCACAGTGGGTAACCTCCG	BRD8__NM_001164326	287	205	312	318
BRD8__NM_001164326_CACAGTGGGTAACCTCCGGG	BRD8__NM_001164326	631	246	610	285
BRD8__NM_001164326_CCAGGAGGTGATTATCACCT	BRD8__NM_001164326	903	429	909	506
BRD8__NM_001164326_CCGGAGGTTACCCACTGTGA	BRD8__NM_001164326	1435	1133	1582	928
BRD8__NM_001164326_GACTCCAACCACTATGGAAG	BRD8__NM_001164326	532	272	605	389
BRD8__NM_001164326_GCATCTTCTGTCATGAGAAG	BRD8__NM_001164326	287	216	330	387
BRD8__NM_001164326_GCATTGTGCTTCCCAGTACT	BRD8__NM_001164326	1063	298	975	284
BRD8__NM_001164326_TTGCAATGACATTGCAACGT	BRD8__NM_001164326	41	177	45	4
BRD8__NM_001164326_TTTGCAATGACATTGCAACG	BRD8__NM_001164326	132	83	142	69
BRD9__NM_001009877_AAGCTCGGGACAGGATCAAC	BRD9__NM_001009877	181	146	188	265
BRD9__NM_001009877_AGGTGGAGCCGCCCCCAGAT	BRD9__NM_001009877	524	395	539	701
BRD9__NM_001009877_CAAGTCAGTTACGGAATTTA	BRD9__NM_001009877	517	486	517	459
BRD9__NM_001009877_CACGCTCGGACTGGCCGATC	BRD9__NM_001009877	183	267	246	362
BRD9__NM_001009877_CCGAGCCGGACGCTGATGGT	BRD9__NM_001009877	2384	1487	2344	2494
BRD9__NM_001009877_CGGACTGGCCGATCGGGGG	BRD9__NM_001009877	751	803	699	925
BRD9__NM_001009877_CTGGCTGTGTCCGGCACGCT	BRD9__NM_001009877	2310	2281	2901	3334
BRD9__NM_001009877_CTTGTACTGGTACAACTTCA	BRD9__NM_001009877	483	418	558	684
BRD9__NM_001009877_GCACGCAGCTGACGAAGCTC	BRD9__NM_001009877	53	88	22	14
BRD9__NM_001009877_GGAGCAATTGCATCCGTGAC	BRD9__NM_001009877	585	666	735	945
BRD9__NM_001009877_TCCAGATGGGCTATCTGAAG	BRD9__NM_001009877	620	774	650	816
BRD9__NM_001009877_TCCTCTTCAGATAGCCCATC	BRD9__NM_001009877	208	107	273	250
BRD9__NM_023924_ACCTTCAGGACTAGCTTTAG	BRD9__NM_023924	349	410	363	616
BRD9__NM_023924_ATAGTAACTGGAGTCGTGGC	BRD9__NM_023924	343	175	391	641
BRD9__NM_023924_GAGTGGCGCTCGTCCTACGA	BRD9__NM_023924	453	525	598	594
BRD9__NM_023924_GCCTCTAAAGCTAGTCCTGA	BRD9__NM_023924	683	864	742	1036
BRDT__NM_001242805_AACGTCAACAGGATTATAAA	BRDT__NM_001242805	173	130	148	106
BRDT__NM_001242805_ACGTCAACAGGATTATAAAA	BRDT__NM_001242805	111	93	99	85
BRDT__NM_001242805_AGAGCAAGTTGTGGGTGTTA	BRDT__NM_001242805	284	335	376	297
BRDT__NM_001242805_AGCATCCACAGGACGTTGAA	BRDT__NM_001242805	201	337	259	373
BRDT__NM_001242805_ATAATAGCTGTTTGTCGACT	BRDT__NM_001242805	589	472	701	581
BRDT__NM_001242805_ATCTATTTCTCCCTTGAACG	BRDT__NM_001242805	213	286	265	238
BRDT__NM_001242805_ATGGCCCTTTCAACGTCCTG	BRDT__NM_001242805	505	424	541	378
BRDT__NM_001242805_GCTCCCTGTACCACGTTCAA	BRDT__NM_001242805	923	838	870	726
BRDT__NM_001242805_GGAGAATAAATATTATGCGA	BRDT__NM_001242805	116	157	189	241
BRDT__NM_001242805_TCCTGTTGACGTTAATGCTT	BRDT__NM_001242805	370	545	562	608
BRDT__NM_001242805_TCTCCCTTGAACGTGGTACA	BRDT__NM_001242805	255	281	204	225
BRDT__NM_001242805_TTCTCCCTTGAACGTGGTAC	BRDT__NM_001242805	0	0	0	0
BRDT__NM_001242807_CCCAAAGCATTAACGTCAAC	BRDT__NM_001242807	829	1125	1180	1301
BRDT__NM_001242810_GTCAAAAATCCGATGGATCT	BRDT__NM_001242810	781	885	947	1073
BRDT__NM_001242810_TGACAACGTCATAGTAGTTA	BRDT__NM_001242810	1154	899	1223	908
BRE__NM_001261840_AGGATAACTGACTTAAAATC	BRE__NM_001261840	193	338	195	334
BRE__NM_001261840_AGTTTAAATCGGTCACAGTT	BRE__NM_001261840	160	138	178	195
BRE__NM_001261840_ATGTCTCTTACTTGTGGTGA	BRE__NM_001261840	164	303	247	378
BRE__NM_001261840_CAGTTTAAATCGGTCACAGT	BRE__NM_001261840	480	541	658	942
BRE__NM_001261840_CATTGGAGATATTCGGTTCA	BRE__NM_001261840	720	858	891	1171
BRE__NM_001261840_CTCCCCTTTCATATCTAGCG	BRE__NM_001261840	245	244	414	714
BRE__NM_001261840_CTTTCATATCTAGCGTGGTC	BRE__NM_001261840	132	170	132	90
BRE__NM_001261840_GGACCACGCTAGATATGAAA	BRE__NM_001261840	304	189	347	189
BRE__NM_001261840_GGGAGGCAGTTCTGGGTATT	BRE__NM_001261840	707	855	819	907
BRE__NM_001261840_TCCTGAATGTCTCTTACTTG	BRE__NM_001261840	723	983	924	993
BRE__NM_001261840_TCTCCAAAGATAAAATCGGG	BRE__NM_001261840	295	144	297	268
BRE__NM_001261840_TCTCCCGGAGGCGGCTACAT	BRE__NM_001261840	1020	904	1103	799
BRF1__NM_001242786_AGAGGCAGGCAGCAATCACG	BRF1__NM_001242786	694	55	802	69
BRF1__NM_001242786_AGCGTGGAATATACAGGCAC	BRF1__NM_001242786	434	0	554	28
BRF1__NM_001242786_ATCAATGGTCAACTGACTGG	BRF1__NM_001242786	293	2	422	93
BRF1__NM_001242786_CCTCTACCTGGTCTGCCGTA	BRF1__NM_001242786	1169	1	1252	35
BRF1__NM_001242786_CTACCTGGTCTGCCGTACGG	BRF1__NM_001242786	743	32	823	33
BRF1__NM_001242786_GACCCCCCCTCGTACACAGC	BRF1__NM_001242786	1138	1474	1342	1704
BRF1__NM_001242786_GCCCAGCTGTGTACGAGGGG	BRF1__NM_001242786	688	192	941	575
BRF1__NM_001242786_TACCTGGTCTGCCGTACGGA	BRF1__NM_001242786	233	0	176	0
BRF1__NM_001242786_TACGCGGCGTGCCCTCCGTA	BRF1__NM_001242786	866	0	1054	15
BRF1__NM_001242786_TCAATGCGCCGGCCATAGGT	BRF1__NM_001242786	203	0	195	4
BRF1__NM_001242786_TCAATGGTCAACTGACTGGT	BRF1__NM_001242786	603	23	803	25
BRF1__NM_001242786_TGCCCAGCTGTGTACGAGGG	BRF1__NM_001242786	799	158	843	300
BRF1__NM_001242788_CACCGTCCAGGGACACGAAC	BRF1__NM_001242788	265	226	359	364
BRF1__NM_001242789_ACCTGAACAAAGACTTATAC	BRF1__NM_001242789	1804	1226	2061	1353
BRF1__NM_001242789_AGGTGAAATATCCAGTTACC	BRF1__NM_001242789	212	71	264	85
BRF1__NM_001242789_CCTCTGCCCAGCTGTGTACG	BRF1__NM_001242789	1160	1456	1330	1724
BRF1__NM_001242789_CTCAATTGCATCCTGGTAAC	BRF1__NM_001242789	289	803	374	507
BRF1__NM_001242789_CTCTGCCCAGCTGTGTACGA	BRF1__NM_001242789	388	622	478	657
BRF1__NM_001242789_TGCCCAGCTGTGTACGAGGG	BRF1__NM_001242789	526	35	614	42
BRF1__NM_001242789_TCTGCCCAGCTGTGTACGAG	BRF1__NM_001242789	1074	1335	1195	1276
BRF1__NM_001242790_AGACCGGCCACTGAGATGCG	BRF1__NM_001242790	1184	1155	1177	1396
BRF1__NM_001242790_CCGGCCACTGAGATGCGTGG	BRF1__NM_001242790	1825	1601	1983	2358
BRF1__NM_001242790_GAGGCACCTGGGGTACACCC	BRF1__NM_001242790	423	489	462	496
BRF1__NM_001242790_GGTGCCTCCACGCATCTCAG	BRF1__NM_001242790	1172	1331	1369	1709
BRF1__NM_001242790_TGTGACCAGGGTTGTGACCG	BRF1__NM_001242790	516	556	681	612
BRF2__NM_018310_ACCAACATTTGAGGATACCG	BRF2__NM_018310	267	38	268	7
BRF2__NM_018310_ACCGCGGTATCCTCAAATGT	BRF2__NM_018310	1022	206	1086	152
BRF2__NM_018310_CAGAGTGCCGATATGCCTGT	BRF2__NM_018310	2503	1041	2367	1385
BRF2__NM_018310_CAGATGGCCCCCATTGTTAG	BRF2__NM_018310	1840	216	2023	246
BRF2__NM_018310_CATAACTGGCCCCTAACAAT	BRF2__NM_018310	615	5	655	7
BRF2__NM_018310_CCGATATGCCTGTTGGTAGT	BRF2__NM_018310	1011	89	1235	95
BRF2__NM_018310_CGTCGCTGAAGGTAGTGGTA	BRF2__NM_018310	455	224	511	411
BRF2__NM_018310_CTGTTGGTAGTAGGCAACCG	BRF2__NM_018310	290	28	303	9
BRF2__NM_018310_GCAGTTGCCACCAACATTTG	BRF2__NM_018310	321	305	373	376
BRF2__NM_018310_TAACTGGCCCCTAACAATGG	BRF2__NM_018310	214	12	196	17
BRF2__NM_018310_TGCAGATGGCCCCCATTGTT	BRF2__NM_018310	914	20	1243	16
BRF2__NM_018310_TTAGGGGCCAGTTATGCTGT	BRF2__NM_018310	189	18	226	0
BRI3__NM_001159491_CAAAGGATACGTGTAAAGCC	BRI3__NM_001159491	405	468	455	689
BRI3__NM_001159491_CAGGATAGCGGGTGACGGTC	BRI3__NM_001159491	117	83	141	156
BRI3__NM_001159491_CTATCCTGCCAACTCTATCG	BRI3__NM_001159491	146	78	76	62
BRI3__NM_001159491_GATAGAGTTGGCAGGATAGC	BRI3__NM_001159491	13	14	35	34
BRI3__NM_001159491_GCCTCCAGGTTGTAGGCGGG	BRI3__NM_001159491	866	783	879	743
BRI3__NM_001159491_GGCCAGGGCGACTACGCGTG	BRI3__NM_001159491	1077	810	1140	1029
BRI3__NM_001159491_TCCTACGACCACGATAGAGT	BRI3__NM_001159491	247	193	241	230
BRI3__NM_015379_GTGGAGCCACCTTCGCTTAA	BRI3__NM_015379	238	373	259	338
BRI3__NM_015379_TGGAGCCACCTTCGCTTAAA	BRI3__NM_015379	443	217	502	329
BRI3BP__NM_080626_ACACGCAGGACATGGAAAAC	BRI3BP__NM_080626	264	281	297	319
BRI3BP__NM_080626_ATGTTCTTGGACTTGACGGT	BRI3BP__NM_080626	112	77	180	140
BRI3BP__NM_080626_CAGGATGTACACGCAGGACA	BRI3BP__NM_080626	582	563	677	557
BRI3BP__NM_080626_CGAGGGCGAGCCGGAGAACG	BRI3BP__NM_080626	878	737	1041	878
BRI3BP__NM_080626_GAAGAACAGCTACCGCCGCA	BRI3BP__NM_080626	357	309	308	323
BRI3BP__NM_080626_GCGTCGTCCTCCTGGCCTAC	BRI3BP__NM_080626	1178	963	1526	1444
BRI3BP__NM_080626_GCTCAACATCCGTCTCAACC	BRI3BP__NM_080626	1229	1105	1365	889
BRI3BP__NM_080626_GCTCGCCCTCGTACTTGTGC	BRI3BP__NM_080626	1307	1588	1457	1772
BRI3BP__NM_080626_GCTGGGAGTGGATATGTTCG	BRI3BP__NM_080626	972	926	1074	1105
BRI3BP__NM_080626_TACATCCTGCACAAGTACGA	BRI3BP__NM_080626	454	212	497	152
BRI3BP__NM_080626_TACTTCATGACCGGGCCCAT	BRI3BP__NM_080626	1052	659	1377	986
BRI3BP__NM_080626_TGCTCAACATCCGTCTCAAC	BRI3BP__NM_080626	284	290	403	372
BRICD5__NM_182563_AGCTGGGCGGTGCTGTTCGA	BRICD5__NM_182563	1354	1212	1014	936
BRICD5__NM_182563_AGTGCAGGGGAGCCTCGAAG	BRICD5__NM_182563	680	568	787	530
BRICD5__NM_182563_CACTGTCCTCCATCAGGCGG	BRICD5__NM_182563	477	816	540	513
BRICD5__NM_182563_CCCGAGCAACATCTGCGTGT	BRICD5__NM_182563	2260	2347	2730	2203
BRICD5__NM_182563_CTGCATCTGTTACCGCCCTG	BRICD5__NM_182563	1178	1310	1283	1501
BRICD5__NM_182563_GCTCGCCGGGCCCAGTAGAT	BRICD5__NM_182563	704	678	940	674
BRICD5__NM_182563_GGACCCCCATCTACTGGGCC	BRICD5__NM_182563	436	655	636	788
BRICD5__NM_182563_TTGGTTGGGCCGGGGCATGT	BRICD5__NM_182563	192	214	173	112
BRINP1__NM_014618_AGATACGGCCCCATATAAAC	BRINP1__NM_014618	429	527	364	266
BRINP1__NM_014618_AGTTTGCCCGTTGGAAGGTG	BRINP1__NM_014618	67	87	42	54
BRINP1__NM_014618_ATCTGGTTGTAAATCCTTGA	BRINP1__NM_014618	441	537	639	718
BRINP1__NM_014618_ATTGGCTCATTTCAGACAGG	BRINP1__NM_014618	465	408	447	501
BRINP1__NM_014618_CAGGAGCTACCTATCCTTTG	BRINP1__NM_014618	3164	2709	3712	3251
BRINP1__NM_014618_CCTCTACTTCCTGTTTATAT	BRINP1__NM_014618	832	806	892	1157
BRINP1__NM_014618_CTTTGAAACTCCGGCATGAG	BRINP1__NM_014618	259	351	320	381
BRINP1__NM_014618_GATACCATCATCAAAAAGTA	BRINP1__NM_014618	255	164	232	351
BRINP1__NM_014618_GATTGGCTCATTTCAGACAG	BRINP1__NM_014618	181	174	218	202
BRINP1__NM_014618_GGAGGCTTTGACCATGTATA	BRINP1__NM_014618	308	345	496	613
BRINP1__NM_014618_GGTGTCTTTCCACAAAGGAT	BRINP1__NM_014618	216	128	245	164
BRINP1__NM_014618_TTTGTGGAAAGACACCGTCA	BRINP1__NM_014618	700	606	764	731
BRINP2__NM_021165_AAGGAGACCCAATCTGCAAC	BRINP2__NM_021165	560	543	639	510
BRINP2__NM_021165_ACAGGAGGTGCCTCTATAAT	BRINP2__NM_021165	555	416	640	723
BRINP2__NM_021165_AGGAGGTGCCTCTATAATCG	BRINP2__NM_021165	325	278	380	280
BRINP2__NM_021165_AGTGTGGCACTCGGTTTAGA	BRINP2__NM_021165	383	284	444	390
BRINP2__NM_021165_CAGTGTGGCACTCGGTTTAG	BRINP2__NM_021165	647	703	825	824
BRINP2__NM_021165_CGAATGTTCCGGATAAACTC	BRINP2__NM_021165	358	563	420	405
BRINP2__NM_021165_CTTCATGGAGCGGTACCGCC	BRINP2__NM_021165	253	216	175	217
BRINP2__NM_021165_GCACTCGGTTTAGAGGGCTT	BRINP2__NM_021165	193	157	144	206
BRINP2__NM_021165_GTTGGCTGTCTCAGCCACGG	BRINP2__NM_021165	142	127	99	164
BRINP2__NM_021165_TGTACCTGGTGGTGAAACCC	BRINP2__NM_021165	25	90	37	45
BRINP2__NM_021165_TTCATGGAGCGGTACCGCCA	BRINP2__NM_021165	47	59	43	20
BRINP2__NM_021165_TTCCAAGGAGGCGAATGTTC	BRINP2__NM_021165	35	6	54	84
BRINP3__NM_199051_AATTGCATCCACTGCCATAA	BRINP3__NM_199051	447	374	632	440
BRINP3__NM_199051_AATTTCTTCTCTCAACTGCA	BRINP3__NM_199051	83	205	157	273
BRINP3__NM_199051_ATGCCACAAGCCCCTTCGAC	BRINP3__NM_199051	1423	1171	1505	1303
BRINP3__NM_199051_CAAGGTTATTTACTTTCCAG	BRINP3__NM_199051	202	160	297	146
BRINP3__NM_199051_CAGGTAACAGAAACACGGAC	BRINP3__NM_199051	419	606	455	639
BRINP3__NM_199051_CAGTCCGTGTTTCTGTTACC	BRINP3__NM_199051	2040	1525	2170	1639
BRINP3__NM_199051_CAGTGGATGCAATTTGAATG	BRINP3__NM_199051	515	494	564	489
BRINP3__NM_199051_CTTATGTTGCGGAAGAATTC	BRINP3__NM_199051	101	86	105	202
BRINP3__NM_199051_GGAAGGGTCCCTTATCAGAG	BRINP3__NM_199051	253	139	227	146
BRINP3__NM_199051_GTATTCCTGTGAGCGATGGA	BRINP3__NM_199051	599	396	684	668
BRINP3__NM_199051_TATTCCTGTGAGCGATGGAA	BRINP3__NM_199051	168	205	247	182
BRINP3__NM_199051_TCAGAGAGGAGCCAGTCGAA	BRINP3__NM_199051	348	204	352	242
BRIP1__NM_032043_AAAGTTATCTGCTAAGAAAC	BRIP1__NM_032043	152	56	170	30
BRIP1__NM_032043_ACCCTTAGAAACTACACAGC	BRIP1__NM_032043	406	197	364	321
BRIP1__NM_032043_ATGTGGTCTGAATATACAAT	BRIP1__NM_032043	279	281	223	148
BRIP1__NM_032043_ATTAGAAAACGTCATTGCTT	BRIP1__NM_032043	152	283	242	219
BRIP1__NM_032043_CAGCAAGCAACATTGTTTGT	BRIP1__NM_032043	820	576	1085	818
BRIP1__NM_032043_CTCCCCTGAAAAAACCACTC	BRIP1__NM_032043	247	358	183	110
BRIP1__NM_032043_GCATGGCAACAATCTCTTAG	BRIP1__NM_032043	355	232	395	392
BRIP1__NM_032043_GGCACTTCATCAACTTGTCA	BRIP1__NM_032043	1176	1766	1300	1659
BRIP1__NM_032043_GGTCTGAATATACAATTGGT	BRIP1__NM_032043	130	125	175	50
BRIP1__NM_032043_GTCTGAATATACAATTGGTG	BRIP1__NM_032043	55	74	82	15
BRIP1__NM_032043_TGGTCTGAATATACAATTGG	BRIP1__NM_032043	4	0	9	5
BRIP1__NM_032043_TTGTTGTGCATGCCATTCAA	BRIP1__NM_032043	216	308	245	374
BRIX1__NM_018321_AAATGGTGAAAGTAAACACG	BRIX1__NM_018321	243	0	254	0
BRIX1__NM_018321_ACCAAGAGGAAACGGCGTGG	BRIX1__NM_018321	430	50	545	27
BRIX1__NM_018321_ACTAGATCTTTAGTACACCA	BRIX1__NM_018321	227	110	253	41
BRIX1__NM_018321_AGATACTAAAATGGATCGTA	BRIX1__NM_018321	119	187	169	65
BRIX1__NM_018321_AGGCTTTCAAATTCACCTCA	BRIX1__NM_018321	505	35	558	44
BRIX1__NM_018321_ATGAGATTTAAGACAAAACG	BRIX1__NM_018321	268	64	268	63
BRIX1__NM_018321_CAGTCATCTTCAGTTCAGCG	BRIX1__NM_018321	819	55	918	99
BRIX1__NM_018321_TAAGCTATTTGTGATTAACG	BRIX1__NM_018321	347	35	427	158
BRIX1__NM_018321_TAGAACAAGACATTTAATGC	BRIX1__NM_018321	397	6	400	5
BRIX1__NM_018321_TCCCAAAAGCCAACCATTTG	BRIX1__NM_018321	239	198	281	430
BRIX1__NM_018321_TTGATGCCTCATTCTAAAGC	BRIX1__NM_018321	120	127	174	57
BRK1__NM_018462_GCAGACTTTCTCAACTCGTT	BRK1__NM_018462	1062	1177	1205	1760
BRK1__NM_018462_GTACTCTATTCTCCGTTCAA	BRK1__NM_018462	355	199	454	261
BRMS1__NM_001024957_AAGATTCGCATTCAGGTGGC	BRMS1__NM_001024957	239	239	205	355
BRMS1__NM_001024957_AAGGGGCTCCGTGTATTCAG	BRMS1__NM_001024957	155	124	120	80
BRMS1__NM_001024957_ATTCTGCTGCTGAGATGAAT	BRMS1__NM_001024957	806	683	1041	981
BRMS1__NM_001024957_CAAGGGGCTCCGTGTATTCA	BRMS1__NM_001024957	712	490	758	832
BRMS1__NM_001024957_CCAAGGGGCTCCGTGTATTC	BRMS1__NM_001024957	247	396	389	319
BRMS1__NM_001024957_CCTCAAGATTCGCATTCAGG	BRMS1__NM_001024957	1054	665	927	710
BRMS1__NM_001024957_CCTCAGAAGAGAAAATCGGA	BRMS1__NM_001024957	865	616	1089	762
BRMS1__NM_001024957_GAAGCATGTACACGATGTAT	BRMS1__NM_001024957	688	479	734	455
BRMS1__NM_001024957_GAGCCTCAAGATTCGCATTC	BRMS1__NM_001024957	429	566	604	590
BRMS1__NM_001024957_GGACACAGCTGCCCTAGCCT	BRMS1__NM_001024957	1174	1672	1171	1660
BRMS1__NM_001024957_TCTCCTCTTCTGACTCTGTC	BRMS1__NM_001024957	367	500	317	445
BRMS1__NM_001024957_TGAGAGAGCCCCTGAATACA	BRMS1__NM_001024957	477	344	625	381
BRMS1L__NM_032352_AACTAGAGGAGAAGATAAGA	BRMS1L__NM_032352	187	181	234	159
BRMS1L__NM_032352_AAGAAGAAGAATGGAATGTT	BRMS1L__NM_032352	334	597	383	431
BRMS1L__NM_032352_AATGCCAGTCCATTCCCGAG	BRMS1L__NM_032352	802	533	860	625
BRMS1L__NM_032352_ACCAGAATACTTGGAACCGC	BRMS1L__NM_032352	288	288	318	276
BRMS1L__NM_032352_CGAGGACACTGAGAGCTCGT	BRMS1L__NM_032352	295	201	253	323
BRMS1L__NM_032352_CTCCATCTCATCGTGATGGT	BRMS1L__NM_032352	666	464	704	686
BRMS1L__NM_032352_GACCAACCATCACGATGAGA	BRMS1L__NM_032352	760	754	758	661
BRMS1L__NM_032352_GGTGGACTACGCCGAAAATG	BRMS1L__NM_032352	654	669	564	664
BRMS1L__NM_032352_TAAAGAACGATTAAGTCAGG	BRMS1L__NM_032352	145	133	192	242
BRMS1L__NM_032352_TGGACTACGCCGAAAATGAG	BRMS1L__NM_032352	46	121	63	28
BRMS1L__NM_032352_TTATCCCCTCGGGAATGGAC	BRMS1L__NM_032352	128	59	146	127
BRMS1L__NM_032352_TTCGTACAAAGGTAGCAGGT	BRMS1L__NM_032352	79	94	44	35
BROX__NM_001288579_AAATGACCCATTGGTTTCAT	BROX__NM_001288579	376	364	499	459
BROX__NM_001288579_AAGTGGACTGATACATTGCA	BROX__NM_001288579	307	357	312	330
BROX__NM_001288579_AATTACTATGGTGTAGTCAC	BROX__NM_001288579	74	23	187	129
BROX__NM_001288579_AGATCATACTTTATCCAGTT	BROX__NM_001288579	361	417	499	548
BROX__NM_001288579_CAGTTCAAGGAGTCGTGCCC	BROX__NM_001288579	411	291	393	368
BROX__NM_001288579_CATCAGGCTTACTGTTACCA	BROX__NM_001288579	1283	1226	1346	1476
BROX__NM_001288579_CATTGCAAGGACAGGTTCCA	BROX__NM_001288579	755	1004	748	1117
BROX__NM_001288579_GCCAATTTCTATCAAAAAGC	BROX__NM_001288579	244	320	306	328
BROX__NM_001288579_GCCAGTGCAGCAATTAGTCC	BROX__NM_001288579	503	534	572	576
BROX__NM_001288579_TGGGTTCCTATGAAACCAAT	BROX__NM_001288579	25	9	7	13
BROX__NM_001288579_TGTCCTGAAGGTTTGACTGT	BROX__NM_001288579	328	488	549	530
BROX__NM_001288579_TTACCATGGTGAGACTTTAT	BROX__NM_001288579	241	192	328	308
BROX__NM_001288581_TGGGATTTAATGTAGCTTTA	BROX__NM_001288581	17	30	13	17
BRPF1__NM_001003694_AATAGGAAGTTGGCCGGGGT	BRPF1__NM_001003694	466	517	566	649
BRPF1__NM_001003694_AGACTTTCTGCCACAACTTG	BRPF1__NM_001003694	371	375	430	505
BRPF1__NM_001003694_AGCTGCCAGAGGTGGTCTAT	BRPF1__NM_001003694	310	261	451	373
BRPF1__NM_001003694_ATGGTGGAGGTGGACTTGCA	BRPF1__NM_001003694	292	330	234	322
BRPF1__NM_001003694_CCAACTTCCTATTACCGGTA	BRPF1__NM_001003694	947	863	1145	1244
BRPF1__NM_001003694_CCCGGCCAACTTCCTATTAC	BRPF1__NM_001003694	276	347	260	321
BRPF1__NM_001003694_CCTTACCGGTAATAGGAAGT	BRPF1__NM_001003694	930	993	950	1057
BRPF1__NM_001003694_GCGGAGTGGAGTTTGTTGTG	BRPF1__NM_001003694	1162	902	1032	1375
BRPF1__NM_001003694_TAAGCCACCATACGAGTGCC	BRPF1__NM_001003694	235	416	317	412
BRPF1__NM_001003694_TCCACCGGGCACCTCGTATGG	BRPF1__NM_001003694	1340	1405	1450	1506
BRPF1__NM_001003694_TGCGGAGTGGAGTTTGTTGT	BRPF1__NM_001003694	2012	2422	2226	3139
BRPF1__NM_001003694_TGTCGTGGTCATAGTGGTAC	BRPF1__NM_001003694	203	239	277	387
BRPF3__NM_015695_ACTTGAGACTGTAGGGGGAC	BRPF3__NM_015695	458	643	484	632
BRPF3__NM_015695_CACCGAATGCAATAGTAACA	BRPF3__NM_015695	815	628	938	695
BRPF3__NM_015695_CATAGATGCTGATACGATGC	BRPF3__NM_015695	172	139	207	213
BRPF3__NM_015695_CCAGGCCAGCGGATTGTCG	BRPF3__NM_015695	960	912	1212	884
BRPF3__NM_015695_CTGGGCTGGGCATATGTCA	BRPF3__NM_015695	517	249	508	319
BRPF3__NM_015695_GCTGGGCTGGGCATATGTC	BRPF3__NM_015695	337	391	376	429
BRPF3__NM_015695_GGAAGCCTCGTCGGAAGTCC	BRPF3__NM_015695	386	405	435	547
BRPF3__NM_015695_GGGTGAGCACTTGAGACTGT	BRPF3__NM_015695	743	571	685	583
BRPF3__NM_015695_GGTGAGCACTTGAGACTGTA	BRPF3__NM_015695	500	444	559	507
BRPF3__NM_015695_GTCTACCTCGACAATCCGCT	BRPF3__NM_015695	846	987	905	892
BRPF3__NM_015695_TGTCTACCTCGACAATCCGC	BRPF3__NM_015695	219	282	294	329
BRPF3__NM_015695_TTCTGCCGGGACTTCGGACG	BRPF3__NM_015695	40	16	73	16
BRS3__NM_001727_AAAGTCTGATTAGGTGAGTG	BRS3__NM_001727	512	608	551	643
BRS3__NM_001727_AATATTTGGAACTGTTTGCA	BRS3__NM_001727	362	444	358	402
BRS3__NM_001727_CACCAACACCAATACCGTTC	BRS3__NM_001727	3986	4794	4010	4452
BRS3__NM_001727_CTTCTTAGAGACAGGATAAG	BRS3__NM_001727	153	210	173	147
BRS3__NM_001727_GAGAATTGCCAGAACGGTAT	BRS3__NM_001727	1035	981	1086	1088
BRS3__NM_001727_GTAAAGGGTCCTAGCAATCA	BRS3__NM_001727	669	802	714	859
BRS3__NM_001727_GTTCCTCAGTAGGTATGTTC	BRS3__NM_001727	495	727	767	720
BRS3__NM_001727_GTTCGGAAGAATTGGTTGTA	BRS3__NM_001727	324	420	424	517
BRS3__NM_001727_TGCAAGAGCTTCTTAGAGAC	BRS3__NM_001727	731	1231	964	1361
BRS3__NM_001727_TGCCAGAACGGTATTGGTGT	BRS3__NM_001727	2155	2223	2384	2640
BRS3__NM_001727_TTCCTCAGTAGGTATGTTCA	BRS3__NM_001727	91	126	147	158
BRS3__NM_001727_TTGGAGGGCTGTCGCTCAAG	BRS3__NM_001727	550	508	446	441
BRSK1__NM_032430_ACCCCAGCACGCCCAATATG	BRSK1__NM_032430	2222	2696	2539	2416
BRSK1__NM_032430_ATATGTGGGCCCCTATCGGC	BRSK1__NM_032430	1315	1597	1386	1348
BRSK1__NM_032430_CCACTGCATCACGGGTCAGA	BRSK1__NM_032430	2321	3251	2726	3089
BRSK1__NM_032430_CCCCAGCACGCCCAATATGT	BRSK1__NM_032430	3868	3576	4315	3488
BRSK1__NM_032430_CCGATAGGGGCCCACATATT	BRSK1__NM_032430	1413	1625	1741	1736
BRSK1__NM_032430_CGAGAACAAGAAATATTTGT	BRSK1__NM_032430	1575	1436	1721	1160
BRSK1__NM_032430_GCCCACATATTGGGCGTGCT	BRSK1__NM_032430	2344	3232	2815	3713
BRSK1__NM_032430_GCCGATAGGGGCCCACATAT	BRSK1__NM_032430	46	6	36	19
BRSK1__NM_032430_GCGTCTCTCCAGCCGATAG	BRSK1__NM_032430	502	476	515	509
BRSK1__NM_032430_GGCCCACATATTGGGCGTGC	BRSK1__NM_032430	718	999	817	1144
BRSK1__NM_032430_TCGCCATCAAGATCGTGAAC	BRSK1__NM_032430	1017	731	1063	1143
BRSK1__NM_032430_TGCAGGGCTGGTTAAACTCG	BRSK1__NM_032430	584	602	696	572
BRSK2__NM_001256627_CGCAGCACGCGCAGTATGTT	BRSK2__NM_001256627	1314	1364	1521	1608
BRSK2__NM_001256627_CGCAGTATGTTGGGCCCTAC	BRSK2__NM_001256627	3380	4861	3389	4715
BRSK2__NM_001256627_CGTCGTGCAGCTTTAGGACG	BRSK2__NM_001256627	2010	2331	2238	2588
BRSK2__NM_001256627_CTCACGGTTGACGATCTTGA	BRSK2__NM_001256627	702	930	889	957
BRSK2__NM_001256627_CTGACACGTGTTCTAGCACC	BRSK2__NM_001256627	1963	1770	2276	2024
BRSK2__NM_001256627_CTGGTGCTAGAACACGTGTC	BRSK2__NM_001256627	541	750	628	758
BRSK2__NM_001256627_GAAGGGGAGGCTGACGCCTA	BRSK2__NM_001256627	281	291	242	242
BRSK2__NM_001256627_GCGCAGCACGCGCAGTATGT	BRSK2__NM_001256627	1117	1056	1290	1388
BRSK2__NM_001256627_GGGGAGGCTGACGCCTAAGG	BRSK2__NM_001256627	14	18	22	23
BRSK2__NM_001256627_GTCGTGCAGCTTTAGGACGT	BRSK2__NM_001256627	3660	4316	3931	3398
BRSK2__NM_001256627_TCGTGCAGCTTTAGGACGTG	BRSK2__NM_001256627	3226	3299	3915	3343
BRSK2__NM_001256627_TCTGCCACAGCCACTCCATA	BRSK2__NM_001256627	1867	1766	2092	2312
BRSK2__NM_001256630_CCTCGGGGCAGGCGTAGTGG	BRSK2__NM_001256630	1259	1083	1542	1560
BRSK2__NM_001256630_CGGGGCAGGCGTAGTGGCGT	BRSK2__NM_001256630	7049	6427	7475	7346
BRSK2__NM_001256630_GGGGACGTGCCCATCGGCTG	BRSK2__NM_001256630	1301	1006	1317	1123
BRSK2__NM_001282218_CGCAAGTTGTCATCGTCGAA	BRSK2__NM_001282218	1524	2461	1906	2695
BRSK2__NM_001282218_CGTCATCCTGTTCGCCTTGC	BRSK2__NM_001282218	2914	3970	3265	3931
BRSK2__NM_001282218_TCGCAAGTTGTCATCGTCGA	BRSK2__NM_001282218	2261	3528	2775	3528
BRWD1__NM_001007246_ACGGGCCCGCCGATAGGTAC	BRWD1__NM_001007246	580	603	604	388
BRWD1__NM_001007246_CCAGCGCATCGGTCCTATGT	BRWD1__NM_001007246	2200	2898	2562	2373
BRWD1__NM_001007246_CCAGTTGTTGCCGAAGAGAT	BRWD1__NM_001007246	666	1077	725	792
BRWD1__NM_001007246_CCCGGTGCCTCTCATCGAGT	BRWD1__NM_001007246	1123	1979	1434	1853
BRWD1__NM_001007246_CCTTATCGCCCGGTACCTAT	BRWD1__NM_001007246	983	1255	1282	1018
BRWD1__NM_001007246_GATGAGAGGCACCGGGCGTC	BRWD1__NM_001007246	856	1171	1047	1207
BRWD1__NM_001007246_GCCGAAGAGATTGGACTGGG	BRWD1__NM_001007246	803	854	1020	1290
BRWD1__NM_001007246_GGAATTTCTTTATCCAACAT	BRWD1__NM_001007246	46	41	41	63
BRWD1__NM_001007246_TATCGCCCGGTACCTATCGG	BRWD1__NM_001007246	528	308	558	292
BRWD1__NM_001007246_TCAAGAGTCACTTCTTTACT	BRWD1__NM_001007246	1145	1540	1281	1438
BRWD1__NM_001007246_TCTTTGCTACGTACAGCAAA	BRWD1__NM_001007246	43	40	34	61
BRWD1__NM_018963_CCTGAAATGCCAGTGAATTA	BRWD1__NM_018963	524	646	620	777
BRWD3__NM_153252_ACCTGAGCGGATTTGTTGCA	BRWD3__NM_153252	1352	1397	1643	1954
BRWD3__NM_153252_ACTGCAAGAACCTAGCGATC	BRWD3__NM_153252	372	359	433	379
BRWD3__NM_153252_ATCGCTAGGTTCTTGCAGTC	BRWD3__NM_153252	376	406	388	516
BRWD3__NM_153252_CCACCGGCTTCGATCTGGGT	BRWD3__NM_153252	1976	2057	2196	1971
BRWD3__NM_153252_CCCTCCCAATCTAAGCGGCG	BRWD3__NM_153252	457	507	465	548
BRWD3__NM_153252_CCGCGCCGCTTAGATTGGGA	BRWD3__NM_153252	1374	1376	1572	1494
BRWD3__NM_153252_CCTACCCAGATCGAAGCCGG	BRWD3__NM_153252	1030	1070	1089	1016
BRWD3__NM_153252_CTTTCCCCTCCCAATCTAAG	BRWD3__NM_153252	200	142	212	210
BRWD3__NM_153252_GATTCCGCGCCGCTTAGATT	BRWD3__NM_153252	872	1070	1019	715
BRWD3__NM_153252_TACCTGAGCGGATTTGTTGC	BRWD3__NM_153252	877	973	1025	971
BRWD3__NM_153252_TCCGCGCCGCTTAGATTGGG	BRWD3__NM_153252	864	1035	1190	959
BRWD3__NM_153252_TGATTCCGCGCCGCTTAGAT	BRWD3__NM_153252	289	284	229	221
BSCL2__NM_001122955_ACCATTTCCTGCTACACCAG	BSCL2__NM_001122955	700	812	839	595
BSCL2__NM_001122955_ACCCTTAGTCAGCGAGAACT	BSCL2__NM_001122955	428	474	579	403
BSCL2__NM_001122955_AGCTCTAAGGTAACACGATA	BSCL2__NM_001122955	74	80	57	59
BSCL2__NM_001122955_CATCTCCACTTCTTCGCGTT	BSCL2__NM_001122955	2813	3416	3118	2959
BSCL2__NM_001122955_CGGAGACCAGATCAAAGGAC	BSCL2__NM_001122955	362	442	345	416
BSCL2__NM_001122955_CGGCATATAGGAATAGTAGA	BSCL2__NM_001122955	117	98	87	51
BSCL2__NM_001122955_CTTTGTCCGGTTCCTTGATC	BSCL2__NM_001122955	674	757	719	765
BSCL2__NM_001122955_GATCAAAGGACCGGACAAAG	BSCL2__NM_001122955	229	231	201	160
BSCL2__NM_001122955_GCCCTGTGCATTTCTACTAC	BSCL2__NM_001122955	1073	1144	1137	1407
BSCL2__NM_001122955_GTGGCTGACTGTCGGCATAT	BSCL2__NM_001122955	133	134	203	154
BSCL2__NM_001122955_TGACTAAGGGTGGACGTGAT	BSCL2__NM_001122955	237	271	232	227
BSCL2__NM_001122955_TGCCAATGTCTCGCTGACTA	BSCL2__NM_001122955	608	670	676	527
BSCL2__NM_001130702_CTGCAAAGCCAAATAGCAGG	BSCL2__NM_001130702	28	14	36	36
BSCL2__NM_001130702_GCTCTGCAAAGCCAAATAGC	BSCL2__NM_001130702	136	133	127	163
BSCL2__NM_001130702_TTCTCTAGCCTCCTGCTATT	BSCL2__NM_001130702	2	0	18	31
BSDC1__NM_001143888_AATACCTTGGTGCCATCATA	BSDC1__NM_001143888	159	119	120	87
BSDC1__NM_001143888_AGCCAGGCGTCAAACAATTC	BSDC1__NM_001143888	108	74	195	150
BSDC1__NM_001143888_CAGGCGTCAAACAATTCCGG	BSDC1__NM_001143888	556	311	487	246
BSDC1__NM_001143888_CCACCTGGGTAAACTCCGTC	BSDC1__NM_001143888	1071	851	1057	1176
BSDC1__NM_001143888_CCAGGCGTCAAACAATTCCG	BSDC1__NM_001143888	1550	1242	1632	1130
BSDC1__NM_001143888_CCGTTCTCTATACAGCGGA	BSDC1__NM_001143888	276	277	375	219
BSDC1__NM_001143888_CTTCCGTCTGTATAGAGAAC	BSDC1__NM_001143888	221	278	270	152
BSDC1__NM_001143888_GGCACAGCTGAGCCCTATGA	BSDC1__NM_001143888	287	254	357	198
BSDC1__NM_001143888_GGCCGTGGCTGCGATGGTAC	BSDC1__NM_001143888	964	1081	915	930
BSDC1__NM_001143888_GGTGATGACATCGCAGTCGA	BSDC1__NM_001143888	876	832	807	883
BSDC1__NM_001143888_TGCGATGTCATCACCCTGAT	BSDC1__NM_001143888	672	684	859	914
BSDC1__NM_001143888_TTTGTCTGGCGAAGGGGCAA	BSDC1__NM_001143888	65	65	149	101
BSDC1__NM_001143889_CCCATTCAGAATTCTGCGGA	BSDC1__NM_001143889	1728	1739	2062	1425
BSDC1__NM_001143889_CGTGACACAGATCGCCAACC	BSDC1__NM_001143889	349	199	317	261
BSDC1__NM_001143889_CTGTTTCCCATTCAGAATTC	BSDC1__NM_001143889	470	389	566	418
BSDC1__NM_001143889_GGTTGGCGATCTGTGTCACG	BSDC1__NM_001143889	839	572	863	609
BSDC1__NM_001143889_GTGTAGAGGGCCGGATGGA	BSDC1__NM_001143889	257	223	395	293
BSDC1__NM_001143889_TAAAGTCCATCAGTTAGAGC	BSDC1__NM_001143889	156	178	111	39
BSDC1__NM_001143889_TGAGGCACGAGTGCTACCCA	BSDC1__NM_001143889	197	135	212	133
BSDC1__NM_001143890_TAGGGGCTTGGAGTGAATAG	BSDC1__NM_001143890	136	114	143	92
BSDC1__NM_001143890_TTAGGGGCTTGGAGTGAATA	BSDC1__NM_001143890	89	68	169	102
BSG__NM_001728_AGCTGGGAGCAGGTGTCGTT	BSG__NM_001728	908	641	1143	1136
BSG__NM_001728_CATCTCCATCGACACGCTCG	BSG__NM_001728	601	622	671	879
BSG__NM_001728_CCAGGACTAGCACGACTGCC	BSG__NM_001728	654	873	871	1389
BSG__NM_001728_CGGGTCAGGTGGTTGCGATC	BSG__NM_001728	283	153	295	338
BSG__NM_001728_CTGCTGGGATTCGCGCTGCT	BSG__NM_001728	1726	1042	1767	1641
BSG__NM_001728_GCTGCTGGGATTCGCGCTGC	BSG__NM_001728	961	695	960	1010
BSG__NM_001728_GGGTCAGGTGGTTGCGATCC	BSG__NM_001728	63	58	119	79
BSG__NM_001728_GTACTCTCCCCACTGGTCGT	BSG__NM_001728	518	555	790	908
BSG__NM_001728_GTCGTCAGAACACATCAACG	BSG__NM_001728	690	306	800	423
BSG__NM_001728_GTTCTTCGTGAGTTCCTCGC	BSG__NM_001728	112	112	154	196
BSG__NM_001728_GTTGCGATCCGGGTCGTTGC	BSG__NM_001728	350	453	392	462
BSG__NM_001728_TGCTCTCGGAGCCGTTCATG	BSG__NM_001728	341	173	292	204
BSG__NM_198589_CCCGGCCAGTACCGGTGCAA	BSG__NM_198589	603	390	660	432
BSG__NM_198589_TGGTGCCGTTGCACCGGTAC	BSG__NM_198589	25	15	54	39
BSN__NM_003458_AGGGACGGAGTAGGGTGATG	BSN__NM_003458	554	386	597	462
BSN__NM_003458_CCCCCGAGAGACAGGGCAC	BSN__NM_003458	551	661	680	565
BSN__NM_003458_CCCTCAGTGTCACCGGACAG	BSN__NM_003458	578	827	785	720
BSN__NM_003458_CCTCTGTCCGGTGACACTGA	BSN__NM_003458	873	486	909	591
BSN__NM_003458_CCTGTGCCCTTGTCTCTCGG	BSN__NM_003458	2560	2511	2801	2554
BSN__NM_003458_CTGCACCCAGTGTCACAACA	BSN__NM_003458	447	676	421	503
BSN__NM_003458_GACAGCAGGACACAGAGATC	BSN__NM_003458	317	301	360	266
BSN__NM_003458_GAGGTCCGAAGTCTTGCATA	BSN__NM_003458	119	208	129	181
BSN__NM_003458_GCAGGACCTTGTTGTGACACT	BSN__NM_003458	1464	1392	1704	1798
BSN__NM_003458_GCTGGCTGGGGGTCGACGTG	BSN__NM_003458	1837	1878	1859	2183
BSN__NM_003458_GGGACGGAGTAGGGTGATGT	BSN__NM_003458	730	511	761	722
BSN__NM_003458_GTGGTAGCAGAAGCCTGCTT	BSN__NM_003458	104	32	49	47
BSND__NM_057176_AGCCGCCCTATGTAAGGCTG	BSND__NM_057176	491	789	522	621
BSND__NM_057176_CACAATGAAGCCGATCCGGA	BSND__NM_057176	744	598	769	733
BSND__NM_057176_CAGCCTTACATAGGGCGGCT	BSND__NM_057176	134	220	181	129
BSND__NM_057176_CATGTGCCAGTGCTACCCCA	BSND__NM_057176	484	39	387	17
BSND__NM_057176_CGTAGACCTGGGGCCGATCA	BSND__NM_057176	1711	1682	2308	1852
BSND__NM_057176_GCTCATGAGGAACGTACCGA	BSND__NM_057176	674	539	835	648
BSND__NM_057176_GGCTCATGAGGAACGTACCG	BSND__NM_057176	382	404	399	368
BSND__NM_057176_TCCCACAGCCTTACATAGGG	BSND__NM_057176	477	429	506	381
BSND__NM_057176_TCCTCCCACAGCCTTACATA	BSND__NM_057176	263	179	298	206
BSND__NM_057176_TCCTTGCTGGCCCCTGAGAT	BSND__NM_057176	364	247	375	420
BSND__NM_057176_TGCCAGTGCTACCCCAAGGT	BSND__NM_057176	0	0	1	0
BSND__NM_057176_TTCCTCCCACAGCCTTACAT	BSND__NM_057176	163	166	166	52
BSPH1__NM_001128326_AAACTTCCAGTATCCTTCGT	BSPH1__NM_001128326	542	467	833	806
BSPH1__NM_001128326_ACAAGACCTACGAAGGATAC	BSPH1__NM_001128326	240	235	267	212
BSPH1__NM_001128326_ACTGATGATGGGGAAGCATT	BSPH1__NM_001128326	244	441	225	405
BSPH1__NM_001128326_GGTACAGACGCTTGATCTAC	BSPH1__NM_001128326	416	453	501	688
BSPH1__NM_001128326_GTACAGACGCTTGATCTACT	BSPH1__NM_001128326	736	1038	694	689
BSPH1__NM_001128326_TACTGGGAGTGTACTGATGA	BSPH1__NM_001128326	403	230	383	516
BSPH1__NM_001128326_TCGTTAAACAAGACCTACGA	BSPH1__NM_001128326	283	420	363	439
BSPRY__NM_017688_AAACTTGACACCATCCGCAC	BSPRY__NM_017688	1321	1487	1251	1589
BSPRY__NM_017688_AGGACATACGGATCGATGAG	BSPRY__NM_017688	376	468	403	300
BSPRY__NM_017688_ATCATCTGACAATTGCAGGA	BSPRY__NM_017688	303	132	326	133
BSPRY__NM_017688_CCCAGAGTTGGGTCAGTAGT	BSPRY__NM_017688	718	716	861	780
BSPRY__NM_017688_CGAGGGAACTGGTTATCCAG	BSPRY__NM_017688	213	151	217	258
BSPRY__NM_017688_GAGGTGGCAGCCAGGGCATT	BSPRY__NM_017688	110	223	81	80
BSPRY__NM_017688_GCCCGGAGCGCTTCGACCAC	BSPRY__NM_017688	2352	3018	2841	3546
BSPRY__NM_017688_GGCCAGTGCAGCGAGGGAAC	BSPRY__NM_017688	350	349	364	277
BSPRY__NM_017688_GGTGGGCATGCTTACTCACC	BSPRY__NM_017688	525	468	691	682
BSPRY__NM_017688_TATGTGGCGGACGTCCTGCC	BSPRY__NM_017688	986	948	987	1384
BSPRY__NM_017688_TCCTCACCAGACTCAACCGC	BSPRY__NM_017688	360	331	370	352
BSPRY__NM_017688_TTCGATCATCTGACAATTGC	BSPRY__NM_017688	13	0	29	45
BST1__NM_004334_ACACGAATGATGACTGCAAG	BST1__NM_004334	347	384	400	482
BST1__NM_004334_AGAAGTTAGTTCTATTTAGC	BST1__NM_004334	77	37	125	160
BST1__NM_004334_ATCTCTGGGAATAGAGTGCC	BST1__NM_004334	231	258	221	309
BST1__NM_004334_CATCTGGGAAGCCTTTAAAG	BST1__NM_004334	173	147	272	294
BST1__NM_004334_CCATACAGAACATCGCTGCA	BST1__NM_004334	559	929	671	800
BST1__NM_004334_CGCTCAGGGGCATAAAACGA	BST1__NM_004334	666	657	615	696
BST1__NM_004334_CTGCAAAGCTGTTAACAAGG	BST1__NM_004334	57	70	45	35
BST1__NM_004334_CTTGTCCAGCGCCACTTTAA	BST1__NM_004334	1244	1417	1407	1660
BST1__NM_004334_GATGTCCCGCAAGTGTGCGC	BST1__NM_004334	368	537	424	639
BST1__NM_004334_GCTCAGGGGCATAAAACGAC	BST1__NM_004334	321	308	442	400
BST1__NM_004334_GGAAGCCTTTAAAGTGGCGC	BST1__NM_004334	167	277	287	344
BST1__NM_004334_GGATTGGTAATCGGATCCTA	BST1__NM_004334	168	220	254	212
BST1__NM_004334_GGGCACCAGCGCACACTTGC	BST1__NM_004334	215	222	209	246
BST1__NM_004334_TGTATAATGACACAAAGCAT	BST1__NM_004334	89	71	99	106
BST1__NM_004334_TGTCTGCAAAGCTGTTAACA	BST1__NM_004334	133	114	110	113
BST1__NM_004334_TTTCAATGAGGCATATGTTC	BST1__NM_004334	14	32	40	58
BST2__NM_004335_CAGACGCGTCCTGAAGCTTA	BST2__NM_004335	474	445	523	446
BST2__NM_004335_CATGGGGCAACACGGTTAGC	BST2__NM_004335	209	155	228	155
BST2__NM_004335_CCCACCCTGAGATTGGGCAT	BST2__NM_004335	717	1460	936	1253
BST2__NM_004335_CCCCCAGAATCACGATGATC	BST2__NM_004335	987	1342	1129	844
BST2__NM_004335_CCTGATCATCGTGATTCTGG	BST2__NM_004335	892	739	1043	824
BST2__NM_004335_CGCTTATCCCCGTCTTCCAT	BST2__NM_004335	1294	753	1585	1147
BST2__NM_004335_CTGCATCCAGGGAAGCCATT	BST2__NM_004335	539	664	603	650
BST2__NM_004335_GACACAGTCGGGTTGACCCA	BST2__NM_004335	446	280	440	365
BST2__NM_004335_GCTTACCACAGTGTGGTTGC	BST2__NM_004335	297	121	355	242
BST2__NM_004335_GGACACAGTCGGGTTGACCC	BST2__NM_004335	301	254	343	341
BST2__NM_004335_GGTCTTAAGCGTGAGAATCG	BST2__NM_004335	144	141	144	100
BST2__NM_004335_TCATGGGGCAACACGGTTAG	BST2__NM_004335	218	50	185	145
BST2__NM_004335_TCCTGATCATCGTGATTCTG	BST2__NM_004335	308	345	314	483
BST2__NM_004335_TCTGCTGGGGATAGGAATTC	BST2__NM_004335	155	127	233	140
BSX__NM_001098169_ACGAACCCAAAGCACCAGAC	BSX__NM_001098169	670	733	789	1061
BSX__NM_001098169_AGACGCCGGGTGTAGAGGAG	BSX__NM_001098169	506	646	484	647
BSX__NM_001098169_AGAGAGCTGGCGAAATGGTC	BSX__NM_001098169	403	251	342	305
BSX__NM_001098169_CACGCCAGAGGAGTCGAGC	BSX__NM_001098169	548	726	635	591
BSX__NM_001098169_CAGCTCCACTCGTTCTGGCG	BSX__NM_001098169	2404	2942	2433	3107
BSX__NM_001098169_CCTGTCCACGCCAGAACGAG	BSX__NM_001098169	933	1360	1110	1277
BSX__NM_001098169_CGAGTCAGAGAAAACCGTGC	BSX__NM_001098169	2296	2109	3010	2596
BSX__NM_001098169_CGGGTGCCTCTGCTAGACTA	BSX__NM_001098169	609	514	684	638
BSX__NM_001098169_GAGAGCTGGCGAAATGGTCT	BSX__NM_001098169	658	1111	693	677
BSX__NM_001098169_GGCCACCTCTCTCAGCGGCT	BSX__NM_001098169	511	912	552	822
BSX__NM_001098169_GGTGGTCTCCCTTATGCAGA	BSX__NM_001098169	52	120	95	204
BSX__NM_001098169_TGGTGGTCTCCCTTATGCAG	BSX__NM_001098169	33	31	34	30
BTAF1__NM_003972_AAATTCAATCGCTCTGTAGT	BTAF1__NM_003972	311	241	371	387
BTAF1__NM_003972_AAGAAACTTGGCCTTAATAT	BTAF1__NM_003972	208	41	233	113
BTAF1__NM_003972_ACAGGAGATTATTTAGTTCA	BTAF1__NM_003972	626	685	777	799
BTAF1__NM_003972_ACCTACTTCCGAAAGTTCTA	BTAF1__NM_003972	487	496	580	478
BTAF1__NM_003972_ATTGACTCAGAGTTTCGAGC	BTAF1__NM_003972	157	152	264	102
BTAF1__NM_003972_CAATTGCTTCTCCCATATTA	BTAF1__NM_003972	578	416	593	325
BTAF1__NM_003972_CAGGAGATTATTTAGTTCAT	BTAF1__NM_003972	1028	327	1149	466
BTAF1__NM_003972_CCTTGTCTGGTTCTCGGCAC	BTAF1__NM_003972	479	161	533	509
BTAF1__NM_003972_CTCGGCACTGGATTCCACTC	BTAF1__NM_003972	454	152	476	230
BTAF1__NM_003972_GAAGAAACTTGGCCTTAATA	BTAF1__NM_003972	269	117	250	203
BTAF1__NM_003972_GGAGTGCAAATTGGATACC	BTAF1__NM_003972	228	38	226	46
BTAF1__NM_003972_TTTGTTAACAAACAACCTGT	BTAF1__NM_003972	87	28	69	10
BTBD1__NM_001011885_ATGGATGCAATAAGTGCAGA	BTBD1__NM_001011885	208	125	161	168
BTBD1__NM_001011885_CATCAGTGGGAACCGGATTA	BTBD1__NM_001011885	432	576	475	504
BTBD1__NM_001011885_CGCCATGTTCAACGGCGGCA	BTBD1__NM_001011885	344	525	395	579
BTBD1__NM_001011885_CGGTCAATGTATTCAACTCG	BTBD1__NM_001011885	404	276	460	696
BTBD1__NM_001011885_GAAAAGCACTTTCCTTAATC	BTBD1__NM_001011885	1542	1819	1635	1712
BTBD1__NM_001011885_GAGCGATGTGCGCTTCGTAC	BTBD1__NM_001011885	886	1057	933	909
BTBD1__NM_001011885_GGGTTTACTGATATTGATAT	BTBD1__NM_001011885	232	396	345	364
BTBD1__NM_001011885_GTCGCCTGCCAGTTGTAGAG	BTBD1__NM_001011885	1028	1175	1234	1241
BTBD1__NM_001011885_GTTCCCACTGATGACAATTG	BTBD1__NM_001011885	140	109	159	163
BTBD1__NM_001011885_TGCCTTTATGTTACTTACTC	BTBD1__NM_001011885	187	279	190	296
BTBD1__NM_001011885_TGGATGCAATAAGTGCAGAA	BTBD1__NM_001011885	80	95	76	125
BTBD1__NM_001011885_TGTATTCAACTCGGGGTTTA	BTBD1__NM_001011885	32	15	37	64
BTBD10__NM_032320_ACTTCCTCGCGTATTGCTAA	BTBD10__NM_032320	556	564	511	722
BTBD10__NM_032320_AGAACGAGTGACACTAATAG	BTBD10__NM_032320	441	314	455	212
BTBD10__NM_032320_AGAGATCAAGAGATAGACGA	BTBD10__NM_032320	141	130	192	90
BTBD10__NM_032320_AGTAGTCAGTCAAGTTCAGA	BTBD10__NM_032320	226	164	232	245
BTBD10__NM_032320_CACACCAAAATGAGTCTACA	BTBD10__NM_032320	552	929	668	729
BTBD10__NM_032320_CCAACATGGTGCTGTCGTGT	BTBD10__NM_032320	1125	1460	1418	1530
BTBD10__NM_032320_GAAGTCACATTTCTAATACA	BTBD10__NM_032320	423	494	405	306
BTBD10__NM_032320_GAGTCTACATGGTGCTAGTG	BTBD10__NM_032320	117	116	141	91
BTBD10__NM_032320_GAGTTACCATCATAGGGATG	BTBD10__NM_032320	112	102	170	175
BTBD10__NM_032320_GGACGGCCTCATCCTATGA	BTBD10__NM_032320	1024	772	1053	786
BTBD10__NM_032320_TCCTCCTTTAGCAATACGCG	BTBD10__NM_032320	328	411	283	293
BTBD10__NM_032320_TCCTCGCGTATTGCTAAAGG	BTBD10__NM_032320	403	779	581	569
BTBD11__NM_001017523_AAAAGCAGTCGTCGGCCCTA	BTBD11__NM_001017523	928	739	1068	816
BTBD11__NM_001017523_AGATGCACAAAAGCAGTCGT	BTBD11__NM_001017523	901	838	934	743
BTBD11__NM_001017523_CAAAGGCTCTCCCACTACTC	BTBD11__NM_001017523	334	271	367	307
BTBD11__NM_001017523_CATCGAAGCCAAGTTTAAGC	BTBD11__NM_001017523	642	626	695	623
BTBD11__NM_001017523_CTGATGTATGCCTGCGTCCG	BTBD11__NM_001017523	2975	3959	3035	3790
BTBD11__NM_001017523_CTGCTTAAACTTGGCTTCGA	BTBD11__NM_001017523	73	131	55	42
BTBD11__NM_001017523_GAACTGTGGACGAACAGACC	BTBD11__NM_001017523	367	417	340	398
BTBD11__NM_001017523_GATCAACACCATGAGCGAAC	BTBD11__NM_001017523	328	277	377	447
BTBD11__NM_001017523_GCTGATGGAGTGGATCCGGG	BTBD11__NM_001017523	1982	2092	2418	1807
BTBD11__NM_001017523_TCCACCCCGGAGACCCGCCAT	BTBD11__NM_001017523	4456	4286	4879	3945
BTBD11__NM_001017523_TGCTGCCGCCGCTGATGGAG	BTBD11__NM_001017523	341	322	420	572
BTBD11__NM_001017523_TGTCTCTGCTGGGGCCCGAT	BTBD11__NM_001017523	199	257	178	122
BTBD11__NM_001018072_AACGCTGGAGGATCTGACGC	BTBD11__NM_001018072	488	780	639	738
BTBD11__NM_001018072_ACGCTGGACTCGGGTTATGG	BTBD11__NM_001018072	904	996	969	976
BTBD11__NM_001018072_CACCCGGCGTCCCCGTATGG	BTBD11__NM_001018072	595	484	598	498
BTBD11__NM_001018072_CAGGGGACCTCGTCTAGGTC	BTBD11__NM_001018072	374	493	418	297
BTBD11__NM_001018072_CCAGCAGCTCCCGCCATACG	BTBD11__NM_001018072	873	648	774	970
BTBD11__NM_001018072_CTGACGCTGGACTCGGGTTA	BTBD11__NM_001018072	291	392	380	366
BTBD11__NM_001018072_GAAGCCCGTGGTGAGAACGC	BTBD11__NM_001018072	435	469	501	622
BTBD11__NM_001018072_GCTCACCAGCGTCGACAGCT	BTBD11__NM_001018072	516	665	655	648
BTBD11__NM_001018072_GCTGGCCAAGCTGTCGACGC	BTBD11__NM_001018072	1019	1284	1191	1226
BTBD11__NM_001018072_GGACTCGGGTTATGGTGGCG	BTBD11__NM_001018072	607	942	639	921
BTBD11__NM_001018072_GTGCTGTTCCGACTCGCACC	BTBD11__NM_001018072	622	489	772	746
BTBD11__NM_001018072_TCGCACCCGGCGTCCCCGTA	BTBD11__NM_001018072	1265	1286	1281	1223
BTBD16__NM_144587_AAGAACAAGGACATCCAAAG	BTBD16__NM_144587	75	72	75	28
BTBD16__NM_144587_AATGAACTTGGTTCCTCTAG	BTBD16__NM_144587	472	296	450	225
BTBD16__NM_144587_ATCAAGAAATTCTACGAGGC	BTBD16__NM_144587	152	169	218	240
BTBD16__NM_144587_CAGTGGGTCATTGATCTTCA	BTBD16__NM_144587	303	338	468	524
BTBD16__NM_144587_GAACAAGGACATCCAAAGTG	BTBD16__NM_144587	155	164	179	168
BTBD16__NM_144587_GAAGAACCTCTACATGAGTG	BTBD16__NM_144587	264	261	319	388
BTBD16__NM_144587_GACCCACTGGTCACTAAAGT	BTBD16__NM_144587	608	607	656	673
BTBD16__NM_144587_GAGTGAGGTGGAGATTAACT	BTBD16__NM_144587	262	214	372	365
BTBD16__NM_144587_GGGCACCACACACCCCCTGA	BTBD16__NM_144587	552	610	534	553
BTBD16__NM_144587_TATCATCACATCCACGCACC	BTBD16__NM_144587	673	845	690	835
BTBD16__NM_144587_TCTCCACCTCACTCATGTAG	BTBD16__NM_144587	1014	717	1124	962
BTBD16__NM_144587_TTTGGGCAAACGCCACCGGT	BTBD16__NM_144587	15	23	14	29
BTBD17__NM_001080466_CACAGAGAGCCGATGTTGGC	BTBD17__NM_001080466	367	201	287	365
BTBD17__NM_001080466_CACCGCCTGGGAGTGGTTGA	BTBD17__NM_001080466	525	556	759	686
BTBD17__NM_001080466_CAGGGAGGACACGCCGTACT	BTBD17__NM_001080466	1517	1425	1401	1367
BTBD17__NM_001080466_CAGGTGCGCGCGCATGTAGT	BTBD17__NM_001080466	813	755	856	767
BTBD17__NM_001080466_CGGTCAGCTCCCGCAGTAC	BTBD17__NM_001080466	924	923	887	860
BTBD17__NM_001080466_CTGGCAGCTCCTGCAACGCT	BTBD17__NM_001080466	716	465	711	634
BTBD17__NM_001080466_GCACAGAGAGCCGATGTTGG	BTBD17__NM_001080466	1018	753	977	926
BTBD17__NM_001080466_GCAGGCTGCGGGCACCGATG	BTBD17__NM_001080466	1155	915	1269	934
BTBD17__NM_001080466_GCTCCTGCAACGCTCGGACC	BTBD17__NM_001080466	640	538	641	583
BTBD17__NM_001080466_GGCTGGTACCACTACGCGGT	BTBD17__NM_001080466	675	567	745	772
BTBD17__NM_001080466_GGGCTGGTACCACTACGCGG	BTBD17__NM_001080466	688	708	758	590
BTBD17__NM_001080466_GTGGGCCTCCCCACCATGC	BTBD17__NM_001080466	200	234	105	83
BTBD18__NM_001145101_AAGGTGGTGCTAGAGCTGGG	BTBD18__NM_001145101	32	0	6	0
BTBD18__NM_001145101_ACTGGCAGGAGAGCACATGT	BTBD18__NM_001145101	634	694	714	834
BTBD18__NM_001145101_AGATTGTCAATGGGACTTGC	BTBD18__NM_001145101	185	248	234	293
BTBD18__NM_001145101_AGGACAAGGAGTTTGATTAG	BTBD18__NM_001145101	346	416	433	456
BTBD18__NM_001145101_CCAGTCCCAAAATCCTATAC	BTBD18__NM_001145101	631	598	618	665
BTBD18__NM_001145101_GATTGTCAATGGGACTTGCT	BTBD18__NM_001145101	645	846	667	807
BTBD18__NM_001145101_GGACAAGGAGTTTGATTAGT	BTBD18__NM_001145101	247	160	310	254
BTBD18__NM_001145101_GGCTTTGATGTACAGATACC	BTBD18__NM_001145101	177	49	249	211
BTBD18__NM_001145101_GGTTCAGCCTCGGCCCTGT	BTBD18__NM_001145101	100	163	81	79
BTBD18__NM_001145101_GTCCACAGAGGGGTACAAGC	BTBD18__NM_001145101	116	216	142	155
BTBD18__NM_001145101_TCCACAGAGGGGTACAAGCT	BTBD18__NM_001145101	575	723	713	634
BTBD18__NM_001145101_TGACACAGTTCTGTTCTAGT	BTBD18__NM_001145101	1214	1575	1408	1511
BTBD19__NM_001136537_AACAACCCGCGATACAGGTG	BTBD19__NM_001136537	192	234	165	202
BTBD19__NM_001136537_ACAGGCCAACAAGCACCGAT	BTBD19__NM_001136537	9	14	7	2
BTBD19__NM_001136537_AGAAAGCGGTGATGCTCCCG	BTBD19__NM_001136537	657	544	638	394
BTBD19__NM_001136537_AGGGCACGGAACATTCGGGC	BTBD19__NM_001136537	1053	851	1060	857
BTBD19__NM_001136537_CCTTGGGACTGGTCGTGCAT	BTBD19__NM_001136537	1260	1202	1399	1094
BTBD19__NM_001136537_CGAGGCTGAACTGGTCCGCG	BTBD19__NM_001136537	4209	4864	4634	4967
BTBD19__NM_001136537_CTGCCCCAGGCCAAAGGTTA	BTBD19__NM_001136537	291	346	296	428
BTBD19__NM_001136537_CTGGAAGAAGTTGCATCTAC	BTBD19__NM_001136537	446	332	373	378
BTBD19__NM_001136537_GCAGGAACCACTCATCCCGG	BTBD19__NM_001136537	2854	1790	3003	1627
BTBD19__NM_001136537_GGATGTGGACTTGGTTGTTG	BTBD19__NM_001136537	585	639	776	763
BTBD19__NM_001136537_TCTCAGCTCTTTCAACCACG	BTBD19__NM_001136537	2	0	5	6
BTBD19__NM_001136537_TGTATCGCGGGTTGTTGACA	BTBD19__NM_001136537	467	413	528	323
BTBD2__NM_017797_AAGGCAGCGGGTTCCACGTC	BTBD2__NM_017797	745	233	965	322
BTBD2__NM_017797_AGCGCGAGGCCGCGTACAAC	BTBD2__NM_017797	115	124	159	94
BTBD2__NM_017797_CACCGACATTGACCTGGGTA	BTBD2__NM_017797	1350	1341	1554	1807
BTBD2__NM_017797_CTTCAGGAACTCCACGCAAT	BTBD2__NM_017797	936	786	858	571
BTBD2__NM_017797_CTTCTTGGCGGTGTATAGCG	BTBD2__NM_017797	83	58	94	110
BTBD2__NM_017797_GCCCACGGCCAGCACGAACC	BTBD2__NM_017797	188	183	217	100
BTBD2__NM_017797_GCTGGCCTGCCAGTTGTACG	BTBD2__NM_017797	860	1035	918	1077
BTBD2__NM_017797_GGCCATTCCCCCGTTGAACA	BTBD2__NM_017797	649	770	664	774
BTBD2__NM_017797_TCTTCAGGAACTCCACGCAA	BTBD2__NM_017797	518	429	499	408
BTBD2__NM_017797_TCTTTGATGCCATGTTCAAC	BTBD2__NM_017797	209	224	198	287
BTBD2__NM_017797_TGCCATGTTCAACGGGGGAA	BTBD2__NM_017797	182	323	161	265
BTBD2__NM_017797_TGTCCTGGAGCGCGACACAC	BTBD2__NM_017797	255	294	281	275
BTBD3__NM_001282550_AAAGCAGCAGGTTCGACATC	BTBD3__NM_001282550	517	744	627	545
BTBD3__NM_001282550_AATCACCTCCCAGCAACGCT	BTBD3__NM_001282550	215	162	238	374
BTBD3__NM_001282550_ACCATTTGCAAAATCATCGA	BTBD3__NM_001282550	578	847	554	692
BTBD3__NM_001282550_ACTAGAAAGTATTCTCCGTA	BTBD3__NM_001282550	666	733	755	857
BTBD3__NM_001282550_CAGCAACGCTGGGTCAGGTC	BTBD3__NM_001282550	616	515	563	717
BTBD3__NM_001282550_CTATTGTGATGAAATTGACT	BTBD3__NM_001282550	314	305	400	389
BTBD3__NM_001282550_CTCCCAGAGCTGCCTGTTCG	BTBD3__NM_001282550	1880	2688	2071	2583
BTBD3__NM_001282550_CTTGATCCGCATACCCACAA	BTBD3__NM_001282550	505	415	564	659
BTBD3__NM_001282550_GAGTTAGCTCTCAAGTCTGA	BTBD3__NM_001282550	299	394	311	444
BTBD3__NM_001282550_GCTCTGGGAGAGGAGCACAC	BTBD3__NM_001282550	370	431	375	317
BTBD3__NM_001282550_GGTCTCCAGGAAATTAACAC	BTBD3__NM_001282550	262	333	296	419
BTBD3__NM_001282550_TTTGTGAGTAAAGCCCGTAA	BTBD3__NM_001282550	115	151	103	74
BTBD3__NM_014962_TTCCAGAGACGGTAAAGAAC	BTBD3__NM_014962	5	8	16	11
BTBD3__NM_181443_GGGAGGGAGCGGAGCGGCAT	BTBD3__NM_181443	233	127	236	202
BTBD6__NM_033271_AAGGCTTCTGTGAGATAGAC	BTBD6__NM_033271	96	42	72	85
BTBD6__NM_033271_ACAGAAGCCTTCGGACCGTA	BTBD6__NM_033271	560	534	612	864
BTBD6__NM_033271_CACAGAAGCCTTCGGACCGT	BTBD6__NM_033271	2277	1873	2432	2331
BTBD6__NM_033271_CACAGGCCGAGATGGCCCTA	BTBD6__NM_033271	80	96	93	77
BTBD6__NM_033271_CAGCAGCGCTGCGTCAGCTC	BTBD6__NM_033271	5	0	0	0
BTBD6__NM_033271_CCGACGTGCACTTCGTCGTG	BTBD6__NM_033271	1941	2584	1898	2395
BTBD6__NM_033271_CGCCAGGTCTCCGTAGAACA	BTBD6__NM_033271	1113	1171	1167	1199
BTBD6__NM_033271_GACCTAGCCAACAGCAACGC	BTBD6__NM_033271	118	99	87	57
BTBD6__NM_033271_GAGATGGCCCTACGGTCCGA	BTBD6__NM_033271	127	162	158	84
BTBD6__NM_033271_GCCGACAGCCAAGACGTACT	BTBD6__NM_033271	1186	1152	1102	1159
BTBD6__NM_033271_GTACAGTGATGAGATCGATC	BTBD6__NM_033271	305	251	303	221
BTBD6__NM_033271_TGTCTCCAGAAAGTTGACAC	BTBD6__NM_033271	265	273	247	227
BTBD7__NM_001002860_AGATGTCAATGCATTAGTGG	BTBD7__NM_001002860	302	424	344	421
BTBD7__NM_001002860_AGTTACCTATAAAAGTCTGT	BTBD7__NM_001002860	326	321	454	596
BTBD7__NM_001002860_CTTTCTTCCTCACCAGAGTA	BTBD7__NM_001002860	300	489	373	531
BTBD7__NM_001002860_GAGGGTAGGGGGAAATTCAC	BTBD7__NM_001002860	84	29	147	76
BTBD7__NM_001002860_GCCTTGCTGAGAATAGGATG	BTBD7__NM_001002860	642	708	558	576
BTBD7__NM_001002860_GTATGGGGCAGAGATAATAA	BTBD7__NM_001002860	177	56	151	107
BTBD7__NM_001002860_TAGAGATGTCAATGCATTAG	BTBD7__NM_001002860	291	38	351	161
BTBD7__NM_001002860_TCATGTTCCCCGAGGGTAGG	BTBD7__NM_001002860	169	133	177	224
BTBD7__NM_001002860_TCGCATCTGCTTGGCATGAT	BTBD7__NM_001002860	421	539	465	590
BTBD7__NM_001002860_TTAAGCGTCGGAAATCTAAT	BTBD7__NM_001002860	295	308	403	457
BTBD7__NM_001002860_TTCTTCCTCACCAGAGTATG	BTBD7__NM_001002860	7	7	6	25
BTBD7__NM_001002860_TTTCTTCCTCACCAGAGTAT	BTBD7__NM_001002860	188	201	191	113
BTBD7__NM_001289133_AAACATCCGAAGTCATGACC	BTBD7__NM_001289133	219	393	326	379
BTBD7__NM_001289133_ACATAGGGAGAGAAGAGTCG	BTBD7__NM_001289133	284	222	341	273
BTBD7__NM_001289133_AGAGGAAATGTAAAGCTTGT	BTBD7__NM_001289133	429	416	431	381
BTBD7__NM_001289133_ATGTGTTCAATTCGCACAAA	BTBD7__NM_001289133	480	379	434	533
BTBD7__NM_001289133_CAAAAGAGGTGTAAAAGAGC	BTBD7__NM_001289133	39	43	72	80
BTBD7__NM_001289133_CCACTTGAGGATGGCAATTA	BTBD7__NM_001289133	504	428	509	405
BTBD7__NM_001289133_CTTAAGACTTCACTGTTTAT	BTBD7__NM_001289133	544	482	631	639
BTBD7__NM_001289133_GCAGTGCCACTCAGTAAGTT	BTBD7__NM_001289133	489	633	731	806
BTBD7__NM_001289133_GGATCTTGTGCGCTTGCGAA	BTBD7__NM_001289133	293	418	348	433
BTBD7__NM_001289133_TACCTTTGCTTCTTCCACAT	BTBD7__NM_001289133	649	611	909	763
BTBD7__NM_001289133_TCTCCTCATTATGAAAGACA	BTBD7__NM_001289133	148	180	190	149
BTBD7__NM_001289133_TGCACCCATTTAGAGCCATA	BTBD7__NM_001289133	611	435	826	722
BTBD8__NM_183242_AAGAGTACGTTACTCTTCAA	BTBD8__NM_183242	111	41	113	29
BTBD8__NM_183242_AGTCAGGAACTCTTGCTAAA	BTBD8__NM_183242	311	316	285	380
BTBD8__NM_183242_CAGAGAAGGTAACATCTGTA	BTBD8__NM_183242	791	1153	1028	1247
BTBD8__NM_183242_CCGCTGGAGTTTGCAGTAAG	BTBD8__NM_183242	839	965	920	1026
BTBD8__NM_183242_CTCAATATGGCTGATATGTA	BTBD8__NM_183242	335	269	315	443
BTBD8__NM_183242_GCGGACCCCAGAAGTACCAT	BTBD8__NM_183242	591	387	702	497
BTBD8__NM_183242_GGACCCCAGAAGTACCATGG	BTBD8__NM_183242	482	506	447	234
BTBD8__NM_183242_TCCGCTGGAGTTTGCAGTAA	BTBD8__NM_183242	753	1097	888	927
BTBD8__NM_183242_TCTTCTCCTAATTCAGATGC	BTBD8__NM_183242	124	125	93	146
BTBD8__NM_183242_TGCAGCAAAATAACTAGATC	BTBD8__NM_183242	260	243	260	302
BTBD8__NM_183242_TTCCAGACAAAACTAATGTT	BTBD8__NM_183242	71	118	85	71
BTBD9__NM_001099272_AGAGGAAACCTTCACTTGAG	BTBD9__NM_001099272	253	257	354	330
BTBD9__NM_001099272_ATATGGCGACGTCACATTCG	BTBD9__NM_001099272	927	689	1248	738
BTBD9__NM_001099272_ATATTCTTCCCCAATCAACA	BTBD9__NM_001099272	158	167	181	129
BTBD9__NM_001099272_ATATTGGTGCCTTGTTGATT	BTBD9__NM_001099272	514	682	619	729
BTBD9__NM_001099272_GACATTCTGAATGTTAAGTA	BTBD9__NM_001099272	71	70	111	110
BTBD9__NM_001099272_GCAGCACATACAAGTTAACT	BTBD9__NM_001099272	849	743	927	782
BTBD9__NM_001099272_GGCTGCTAAAATTACCCTGT	BTBD9__NM_001099272	1140	640	1412	749
BTBD9__NM_001099272_TACACCAGTTTAATAGGAGA	BTBD9__NM_001099272	1749	1252	2190	1751
BTBD9__NM_001099272_TGCAGAGCATTATTATATGG	BTBD9__NM_001099272	375	205	333	175
BTBD9__NM_001099272_TGGCGACGTCACATTCGTGG	BTBD9__NM_001099272	232	154	278	202
BTBD9__NM_001099272_TTACCGAAAATATTGGCACC	BTBD9__NM_001099272	279	219	245	226
BTBD9__NM_001099272_TTGAGCCTGGCTCATAAATA	BTBD9__NM_001099272	144	102	201	179
BTBD9__NM_001172418_AACATTGCAACTATGAAGTA	BTBD9__NM_001172418	296	311	354	270
BTBD9__NM_001172418_ATGTGCAGAGCATTATTATA	BTBD9__NM_001172418	135	60	192	201
BTBD9__NM_001172418_CAGAGCTTCTGAATGTTGTG	BTBD9__NM_001172418	550	615	606	705
BTBD9__NM_001172418_GATATGGACCTCAATTATAG	BTBD9__NM_001172418	200	150	177	105
BTBD9__NM_001172418_GCTCTCAGATCGCACTTTAA	BTBD9__NM_001172418	877	753	995	911
BTBD9__NM_001172418_TGAGCATGCCTCTATAATTG	BTBD9__NM_001172418	398	181	517	370
BTBD9__NM_152733_ATGGAGCCCAAGTTGTAAAG	BTBD9__NM_152733	155	104	176	128
BTBD9__NM_152733_TATGGAGCCCAAGTTGTAAA	BTBD9__NM_152733	160	350	260	224
BTC__NM_001729_AGTAATGCTTGTATTGCTTG	BTC__NM_001729	466	649	436	489
BTC__NM_001729_CAAAGGGAGATGCCGCTTCG	BTC__NM_001729	1366	1697	1618	2058
BTC__NM_001729_GATTTGTTTGATAGCAGTTA	BTC__NM_001729	219	319	202	190
BTC__NM_001729_GCAGTAATGCTTGTATTGCT	BTC__NM_001729	757	1146	864	1067
BTD__NM_000060_AATGGCGCATGCGCATATTC	BTD__NM_000060	80	89	178	379
BTD__NM_000060_ATGGGCATATTCAGGGCGGA	BTD__NM_000060	322	366	402	535
BTD__NM_000060_CAGGCATGCGCATATTCA	BTD__NM_000060	289	195	259	324
BTD__NM_000060_ATGGGTTCCACCTGACCACC	BTD__NM_000060	139	166	162	84
BTD__NM_000060_CATAATGCAGACCACAAATC	BTD__NM_000060	552	368	621	445
BTD__NM_000060_GATGGATGGATGCTCATACA	BTD__NM_000060	443	169	495	472
BTD__NM_000060_GCCTGAGTTGTATGGCCATC	BTD__NM_000060	603	829	746	865
BTD__NM_000060_GCTCCAGCGCCTGAGTTGTA	BTD__NM_000060	883	566	991	699
BTD__NM_000060_GGTGGCCAATCTTGGGACAA	BTD__NM_000060	299	348	237	290
BTD__NM_000060_GTTCATAGATGTCAAGGTTC	BTD__NM_000060	32	40	86	41
BTD__NM_000060_TCACGAGGCTGAATATTATG	BTD__NM_000060	628	767	659	680
BTD__NM_000060_TTTCCTCTGCGGCTGTTACG	BTD__NM_000060	79	85	125	135
BTD__NM_001281723_ACCCAAGGTGCCCAAAAGAT	BTD__NM_001281723	52	30	57	43
BTD__NM_001281723_CTTGTCATAGCAGTGACCCA	BTD__NM_001281723	152	108	184	205
BTD__NM_001281723_TTTACGGTAGCGGTCAACAA	BTD__NM_001281723	241	411	283	216
BTD__NM_001281724_AGATGAATCATTTAGCAAGG	BTD__NM_001281724	158	160	138	160
BTD__NM_001281724_ATGAATCATTTAGCAAGGTG	BTD__NM_001281724	0	0	0	0
BTD__NM_001281725_GTGATGAGATCCACTTTAAG	BTD__NM_001281725	200	147	264	99
BTD__NM_001281726_ACACAGAGGTGATCTAAGTC	BTD__NM_001281726	221	285	233	265
BTD__NM_001281726_CACAGAGGTGATCTAAGTCA	BTD__NM_001281726	221	175	228	288
BTF3__NM_001037637_AGGCACAAGTGCGCATTGGT	BTF3__NM_001037637	492	2	550	16
BTF3__NM_001037637_CCCAGCATCTTAAACCAGCT	BTF3__NM_001037637	196	197	220	188
BTF3__NM_001037637_CCCGCTCAGGCTGACTCTCG	BTF3__NM_001037637	1526	1581	1711	1266
BTF3__NM_001037637_CGGATAGTCTGACTAGTTTA	BTF3__NM_001037637	847	1237	1072	958
BTF3__NM_001037637_CTTTAACAACCCTAAAGTTC	BTF3__NM_001037637	98	0	165	50
BTF3__NM_001037637_GGAAAAGCACCACTTGCTAC	BTF3__NM_001037637	847	254	908	130
BTF3__NM_001037637_GTTCTCCTTAAAGAAGTTAG	BTF3__NM_001037637	7	0	0	0
BTF3__NM_001037637_GTTTACCCCTAACTTCTTTA	BTF3__NM_001037637	172	293	211	244
BTF3__NM_001037637_TCAGCTGCTTTTGTCTCAGCA	BTF3__NM_001037637	266	391	379	403
BTF3__NM_001037637_TCATCCTCTCCAGTAGCAAG	BTF3__NM_001037637	221	14	262	21
BTF3L4__NM_001136497_AACAGTTCCCACGGCAAGGT	BTF3L4__NM_001136497	157	121	160	128
BTF3L4__NM_001136497_AAGCATTTCTGTGATTGGTT	BTF3L4__NM_001136497	490	389	662	417
BTF3L4__NM_001136497_ACTGTTCAGCTAACTTCCTA	BTF3L4__NM_001136497	537	312	641	420
BTF3L4__NM_001136497_CCTGGAATATTAAGATCAGCT	BTF3L4__NM_001136497	366	244	421	506
BTF3L4__NM_001136497_GCTGAACAGTTCCCACGGCA	BTF3L4__NM_001136497	1077	768	1050	855
BTF3L4__NM_001243767_CAGGCTCAGGTCCGGATAGG	BTF3L4__NM_001243767	257	49	299	76
BTF3L4__NM_001243767_CCGGACCTGAGCCTGAAGTT	BTF3L4__NM_001243767	928	525	1037	562
BTF3L4__NM_001243767_CTGGCTGTGAATAATATAGC	BTF3L4__NM_001243767	171	26	161	53
BTF3L4__NM_001243767_CTTCAGGCTCAGGTCCGGAT	BTF3L4__NM_001243767	1009	515	1304	724
BTF3L4__NM_001243767_TCAGGCTCAGGTCCGGATAG	BTF3L4__NM_001243767	133	15	172	19
BTF3L4__NM_001243767_TTCAGGCTCAGGTCCGGATA	BTF3L4__NM_001243767	112	109	142	125
BTG1__NM_001731_AAACAGCACCAACGTGCAAA	BTG1__NM_001731	297	354	244	192
BTG1__NM_001731_ACCCAGAGTGTGAGTTCACT	BTG1__NM_001731	1498	1245	1533	1243
BTG1__NM_001731_ATTCTGTAGGACACTTCATA	BTG1__NM_001731	267	202	273	140
BTG1__NM_001731_GCTGTCTACCATTTGCAGCT	BTG1__NM_001731	1658	1351	1868	1720
BTG1__NM_001731_GGCGATCTCGCCTATCATGG	BTG1__NM_001731	345	392	382	378
BTG1__NM_001731_GGCGGCGATCTCGCCTATCA	BTG1__NM_001731	133	125	252	193
BTG1__NM_001731_GTTCCTCCTTACAGCTGATT	BTG1__NM_001731	278	330	306	297
BTG1__NM_001731_TATGAAGTGTCCTACAGAAT	BTG1__NM_001731	494	370	515	399
BTG1__NM_001731_TATGGTTGATGCGAATACAA	BTG1__NM_001731	145	185	150	125
BTG1__NM_001731_TATTCGCATCAACCATAAAA	BTG1__NM_001731	134	251	140	127
BTG1__NM_001731_TCCAAGTTTCTCCGCACCAA	BTG1__NM_001731	403	538	400	292
BTG2__NM_006763_AAGCAGCACTAGCAAAGCAA	BTG2__NM_006763	133	75	143	266
BTG2__NM_006763_AAGGACAGGGCTAGGGCATT	BTG2__NM_006763	594	513	531	420
BTG2__NM_006763_ACATAGTATTGTTTAAGTCC	BTG2__NM_006763	252	127	381	176
BTG2__NM_006763_ACCCACAGGGTCAGCTCGCT	BTG2__NM_006763	191	211	161	108
BTG2__NM_006763_AGACTACTTGGTATTCTTGT	BTG2__NM_006763	586	479	685	513
BTG2__NM_006763_AGAGGTGGCTCAAAAGCTAC	BTG2__NM_006763	22	16	27	37
BTG2__NM_006763_AGCCAGTGTCTGCCTATAGG	BTG2__NM_006763	700	647	795	533
BTG2__NM_006763_ATGAGGTGTCCTACCGCATT	BTG2__NM_006763	813	773	800	432
BTG2__NM_006763_ATGCGGTAGGACACCTCATA	BTG2__NM_006763	190	173	274	174
BTG2__NM_006763_ATGGTTCCCTAAGCCCACCA	BTG2__NM_006763	52	36	38	44
BTG2__NM_006763_CAAACCGAATCACCTTAAGA	BTG2__NM_006763	370	359	460	306
BTG2__NM_006763_CACTGCGTCTTGTACGAGG	BTG2__NM_006763	475	363	564	329
BTG2__NM_006763_CATGTATTCCTTGGCTGAAT	BTG2__NM_006763	976	1135	1139	940
BTG2__NM_006763_CCAGTGTCTGCCTATAGGAG	BTG2__NM_006763	346	296	347	301
BTG2__NM_006763_CTGGCTGAGTCCGATCTGGC	BTG2__NM_006763	2615	3243	2926	3734
BTG2__NM_006763_CTTCTTAAGGTGATTCGGTT	BTG2__NM_006763	176	199	221	273
BTG2__NM_006763_GAAAAGTGGAAAGCTACACT	BTG2__NM_006763	612	708	533	431
BTG2__NM_006763_GATTCGGTTTGGGAAACTCG	BTG2__NM_006763	362	537	368	449
BTG2__NM_006763_GCACAAGTCTACGACAAATT	BTG2__NM_006763	598	489	776	946
BTG2__NM_006763_GCCAGTGTCTGCCTATAGGA	BTG2__NM_006763	589	322	577	362
BTG2__NM_006763_GCCATCAGCAGTTCTTGGA	BTG2__NM_006763	576	469	695	446
BTG2__NM_006763_GCCGTCCTCCCCAATGCGGT	BTG2__NM_006763	1265	1116	1342	1081
BTG2__NM_006763_GCTGCATGATCCCTTTGAG	BTG2__NM_006763	3117	3121	3294	2967
BTG2__NM_006763_GGCCACCACATACCTCAACC	BTG2__NM_006763	2624	3630	2861	3314
BTG2__NM_006763_GGGGCTGGCTGAGTCCGATC	BTG2__NM_006763	962	924	989	858
BTG2__NM_006763_GTGAGCCAGTGTCTGCCTAT	BTG2__NM_006763	1733	1417	1704	1123
BTG2__NM_006763_GTTCCCCAGGTTGAGGTATG	BTG2__NM_006763	122	142	162	143
BTG2__NM_006763_TATAGGAGGGGGAGCTGTTA	BTG2__NM_006763	284	265	257	253
BTG2__NM_006763_TATGAGGTGTCCTACCGCAT	BTG2__NM_006763	763	730	698	590
BTG2__NM_006763_TCAGCCAAGGAATACATGCA	BTG2__NM_006763	422	492	509	369
BTG2__NM_006763_TGAAAAGAAGGCAGTAATAA	BTG2__NM_006763	141	167	188	192
BTG2__NM_006763_TGAATGCCCCTGAGTAATAG	BTG2__NM_006763	314	254	277	250
BTG3__NM_001130914_AAAAAGCCAGGAGTATGTCG	BTG3__NM_001130914	542	730	521	568
BTG3__NM_001130914_AATCAGAGGTAACCTTATCA	BTG3__NM_001130914	846	1116	993	954
BTG3__NM_001130914_ACCGACAGCACACCTCACAT	BTG3__NM_001130914	2073	3050	2494	2799
BTG3__NM_001130914_AGAAAAACATCGAAAGGAC	BTG3__NM_001130914	119	62	77	117
BTG3__NM_001130914_ATCAGAGGTAACCTTATCAA	BTG3__NM_001130914	345	359	473	361
BTG3__NM_001130914_CTGTAGGCCTGTCCTTTCGA	BTG3__NM_001130914	819	954	882	982
BTG3__NM_001130914_GATCTCCAGGAAAGTTACCA	BTG3__NM_001130914	391	519	592	599
BTG3__NM_001130914_GGATCCTGAATGATAATCAG	BTG3__NM_001130914	127	180	127	160
BTG3__NM_001130914_GTTACCTCTGATTATCATTC	BTG3__NM_001130914	311	294	320	368
BTG3__NM_001130914_TAGTGACCTGGGCTTGCCAA	BTG3__NM_001130914	421	443	499	441
BTG3__NM_001130914_TCTCTGGGTGGACCCATGTG	BTG3__NM_001130914	167	127	173	178
BTG3__NM_001130914_TGCATCTTGTATAGTGACCT	BTG3__NM_001130914	107	295	213	116
BTG4__NM_017589_ACACATGCCCTTTCTAGAAT	BTG4__NM_017589	1102	1308	1262	1712
BTG4__NM_017589_AGAAGCATCCTAAGTGTTAC	BTG4__NM_017589	446	534	516	556
BTG4__NM_017589_ATAAAGATCCCATTCTAGAA	BTG4__NM_017589	167	232	141	157
BTG4__NM_017589_ATAGCAGGCCTGTAACACTT	BTG4__NM_017589	464	558	531	545
BTG4__NM_017589_ATCCCTTTGAAGTATGCTGT	BTG4__NM_017589	355	313	340	606
BTG4__NM_017589_CACCTACAGCATACTTCAAA	BTG4__NM_017589	173	263	208	238
BTG4__NM_017589_CTCCTGGGAAACACTTACCA	BTG4__NM_017589	339	301	361	326
BTG4__NM_017589_CTGAAGGCTTGCCCTTTAGA	BTG4__NM_017589	249	284	348	420
BTG4__NM_017589_GCCAGAGGAAACGTCTGATG	BTG4__NM_017589	620	483	673	719
BTG4__NM_017589_TGAAACAGCCCTTTCAATCT	BTG4__NM_017589	362	284	557	397
BTG4__NM_017589_TTCAAAGGGATCTACCCATA	BTG4__NM_017589	34	68	39	30
BTG4__NM_017589_TTCTCACCTGGGACTTCCGA	BTG4__NM_017589	0	0	2	0
BTK__NM_000061_AATCCGGTACAACAGTGATC	BTK__NM_000061	428	412	461	300
BTK__NM_000061_AATGATCTACAGCTGCGGAA	BTK__NM_000061	203	107	178	127
BTK__NM_000061_AGGAAAGCAACTTACCATGG	BTK__NM_000061	645	536	652	633
BTK__NM_000061_ATGAGTATGACTTTGAACGT	BTK__NM_000061	1184	839	1111	824
BTK__NM_000061_CCCAGGTTGTATATGATGAA	BTK__NM_000061	429	530	482	676
BTK__NM_000061_CCTGGTCCTCCTCAGGCGTT	BTK__NM_000061	2159	2459	2269	2197
BTK__NM_000061_CTGAAGAACTAAGGAAGCGG	BTK__NM_000061	232	297	199	196
BTK__NM_000061_TAAAACCTGGGAGTTCTCAC	BTK__NM_000061	332	204	328	416
BTK__NM_000061_TATGAGTATGACTTTGAACG	BTK__NM_000061	326	333	325	349
BTK__NM_000061_TGGAGGAAAGCAACTTACCA	BTK__NM_000061	274	261	281	331
BTK__NM_000061_TTCAAAGTCATACTCATAGT	BTK__NM_000061	23	18	25	18
BTK__NM_000061_TTCAGCTCACTTGTGGAGAC	BTK__NM_000061	21	25	49	87
BTLA__NM_001085357_AAAGGGATCTCCTGCTAAGA	BTLA__NM_001085357	245	163	166	147
BTLA__NM_001085357_AGCAAACAGAAGCAAGCACC	BTLA__NM_001085357	351	409	367	427
BTLA__NM_001085357_AGCTTGCACCAAGTCACATG	BTLA__NM_001085357	694	637	711	842
BTLA__NM_001085357_AGTTTCTGATAGCAGTACTT	BTLA__NM_001085357	103	139	123	93
BTLA__NM_001085357_CACAGGGCATTCTAGTTCAA	BTLA__NM_001085357	570	713	638	720
BTLA__NM_001085357_CAGTGCTTCCTAATGACAAT	BTLA__NM_001085357	355	472	532	545
BTLA__NM_001085357_CATCTGGAACATCCATGGTA	BTLA__NM_001085357	694	925	735	491
BTLA__NM_001085357_CCAGTGCTTCCTAATGACAA	BTLA__NM_001085357	471	634	566	744
BTLA__NM_001085357_CTGTGAAATACTGTGCTAAC	BTLA__NM_001085357	275	281	358	388
BTLA__NM_001085357_TAATCCCATATCTGGACATC	BTLA__NM_001085357	551	739	672	878
BTLA__NM_001085357_TCAACAACTCTTTATGTGAC	BTLA__NM_001085357	619	827	667	786
BTLA__NM_001085357_TGTTCCAGATGTCCAGATAT	BTLA__NM_001085357	218	218	205	270
BTLA__NM_181780_AGTAGAGGCAATCCCCCCAA	BTLA__NM_181780	324	481	380	434
BTLA__NM_181780_TGCCTCAGAACGACCCTCCA	BTLA__NM_181780	871	1252	910	1230
BTN1A1__NM_001732_AAGTGCTCGGCGCTCGCGTT	BTN1A1__NM_001732	938	767	1051	1030
BTN1A1__NM_001732_AGCAGATGCCCGAGTACCGC	BTN1A1__NM_001732	813	543	918	727
BTN1A1__NM_001732_CCGAGCACTTGGAGCTACGC	BTN1A1__NM_001732	586	707	654	685
BTN1A1__NM_001732_CCGTGCTGGTGCATAGGGAC	BTN1A1__NM_001732	411	493	461	760
BTN1A1__NM_001732_CTCACCCACAACGGCCAGGA	BTN1A1__NM_001732	278	393	373	504
BTN1A1__NM_001732_CTCGGCGTCCTCACCCACAA	BTN1A1__NM_001732	128	219	134	238
BTN1A1__NM_001732_GAGCAGATGCCCGAGTACCG	BTN1A1__NM_001732	558	519	487	476
BTN1A1__NM_001732_GCAGCTCCCTTTGACGTGAT	BTN1A1__NM_001732	760	970	840	1345
BTN1A1__NM_001732_GCCGTGCTGGTGCATAGGGA	BTN1A1__NM_001732	322	213	254	278
BTN1A1__NM_001732_GCGTTCGGAGACAGGCGACA	BTN1A1__NM_001732	432	550	526	624
BTN1A1__NM_001732_GGGGGTCCAATCACGTCAAA	BTN1A1__NM_001732	130	186	189	262
BTN1A1__NM_001732_GTTCCGAAAGAAGGTTTCGC	BTN1A1__NM_001732	83	92	81	84
BTN2A1__NM_001197233_AATAACCACAATGATAGGCA	BTN2A1__NM_001197233	196	114	226	238
BTN2A1__NM_001197233_ATCCTGCACCTCGTAGTGGC	BTN2A1__NM_001197233	2298	2597	2714	3013
BTN2A1__NM_001197233_CAGGGTGTTGTTGATAGAGC	BTN2A1__NM_001197233	397	610	533	517
BTN2A1__NM_001197233_CGGCTGGAGTGCATATCTAG	BTN2A1__NM_001197233	542	585	688	745
BTN2A1__NM_001197233_CTGTGATGTTGTGTATGACC	BTN2A1__NM_001197233	474	449	573	539
BTN2A1__NM_001197233_GCAGGCGCAACCCCACCGTA	BTN2A1__NM_001197233	5477	6163	5858	6745
BTN2A1__NM_001197233_GGCTGGAGTGCATATCTAGA	BTN2A1__NM_001197233	483	470	482	475
BTN2A1__NM_001197233_TGGAGTGCATATCTAGAGGG	BTN2A1__NM_001197233	164	78	142	108
BTN2A1__NM_001197233_TGGCCCCTCATTGAAATGAG	BTN2A1__NM_001197233	3	0	0	0
BTN2A1__NM_001197233_TGTGATGTTGTGTATGACCA	BTN2A1__NM_001197233	380	282	331	307
BTN2A1__NM_001197233_TGTGGAGGGACCCCTACGGT	BTN2A1__NM_001197233	871	540	789	569
BTN2A1__NM_001197233_TTCTTGGAAGTAACAGCGGT	BTN2A1__NM_001197233	5	0	0	0
BTN2A1__NM_001197234_AACAGCCCAGTTTATTGTCG	BTN2A1__NM_001197234	1220	1345	1240	1385
BTN2A1__NM_001197234_ACAGCCCAGTTTATTGTCGT	BTN2A1__NM_001197234	1721	2303	2154	2244
BTN2A1__NM_001197234_CAGCCCAGTTTATTGTCGTG	BTN2A1__NM_001197234	2171	2911	2514	3164
BTN2A1__NM_001197234_GTGGCCAAGATGGGATCAGT	BTN2A1__NM_001197234	59	18	90	42
BTN2A1__NM_001197234_TGGGCCCCACGACAATAAAC	BTN2A1__NM_001197234	91	37	86	122
BTN2A2__NM_001197237_AACAGCCCAGTTTACTGTCG	BTN2A2__NM_001197237	1196	1041	1300	1152
BTN2A2__NM_001197237_ACAGCCCAGTTACTGTCGT	BTN2A2__NM_001197237	2093	2381	2516	2834
BTN2A2__NM_001197237_ATGGCCAGGATGGGATTAGC	BTN2A2__NM_001197237	232	430	248	289
BTN2A2__NM_001197237_CAATGAGGGGCTTAGACCCA	BTN2A2__NM_001197237	478	334	449	415
BTN2A2__NM_001197237_CAGCCCAGTTTACTGTCGTG	BTN2A2__NM_001197237	2437	1804	2518	2486
BTN2A2__NM_001197237_CCATCCAGGGAGATGCGGTC	BTN2A2__NM_001197237	1041	1468	1114	1569
BTN2A2__NM_001197237_GCAGTGTTTGTGTATAAGGG	BTN2A2__NM_001197237	679	463	655	519
BTN2A2__NM_001197237_GCGGGCACAACCTCACCGTA	BTN2A2__NM_001197237	2250	2330	2172	2381
BTN2A2__NM_001197237_GGAAAAGAAAGTTGAACAAG	BTN2A2__NM_001197237	261	293	194	386
BTN2A2__NM_001197237_GGCGGGCACAACCTCACCGT	BTN2A2__NM_001197237	3036	2746	3380	2520
BTN2A2__NM_001197237_TCATCAGAGACAAGTATGTG	BTN2A2__NM_001197237	427	314	497	570
BTN2A2__NM_001197237_TGCCACCACGAGGCGTAGGA	BTN2A2__NM_001197237	225	328	284	235
BTN2A2__NM_001197239_AGATGTTTGGAAACCAATAC	BTN2A2__NM_001197239	248	206	277	294
BTN2A2__NM_001197239_CAGGAAGAGCTCGGGATGAG	BTN2A2__NM_001197239	434	489	402	309
BTN2A2__NM_001197239_GATGGGAGAGCTTCGCCTCA	BTN2A2__NM_001197239	746	786	756	689
BTN2A2__NM_001197239_GGAAAAAGAAATTGCACGTA	BTN2A2__NM_001197239	325	646	365	485
BTN2A2__NM_001197240_ATGGAAACGGCCACTACATG	BTN2A2__NM_001197240	355	346	401	352
BTN2A2__NM_001197240_TCATGGAAACGGCCACTACA	BTN2A2__NM_001197240	349	366	504	507
BTN2A2__NM_001197240_TGTAGTGGCCGTTCCATGA	BTN2A2__NM_001197240	651	548	822	720
BTN3A1__NM_001145008_AATGCCAATTAAATCTCTCA	BTN3A1__NM_001145008	423	650	480	648
BTN3A1__NM_001145008_CAGCTGCTCATGCCTCACTC	BTN3A1__NM_001145008	769	803	787	1034
BTN3A1__NM_001145008_CAGGAAAGAGTGGCATATAG	BTN3A1__NM_001145008	346	379	343	385
BTN3A1__NM_001145008_GAGAATGGATTCTGGACTAT	BTN3A1__NM_001145008	673	887	768	670
BTN3A1__NM_001145008_GAGAGACATTCAGCCTATAA	BTN3A1__NM_001145008	227	217	197	154
BTN3A1__NM_001145008_GAGGGAACTTCTGATGGTAC	BTN3A1__NM_001145008	410	700	463	432
BTN3A1__NM_001145008_GCTGTGAGAGCTTCATATCA	BTN3A1__NM_001145008	904	701	876	1035
BTN3A1__NM_001145008_GGGGAGCCGGTTACTTCCTG	BTN3A1__NM_001145008	1043	893	1267	1075
BTN3A1__NM_001145008_TCACGCTGGACAAATAGTCA	BTN3A1__NM_001145008	756	524	830	801
BTN3A1__NM_001145008_TCCTAAGCCCCCTAAGAAAG	BTN3A1__NM_001145008	625	714	744	1039
BTN3A1__NM_001145008_TGAGAATGGATTCTGGACTA	BTN3A1__NM_001145008	801	943	1068	1386
BTN3A1__NM_001145008_TTCACGCTGGACAAATAGTC	BTN3A1__NM_001145008	32	50	31	55
BTN3A1__NM_001145009_CGTTGATGTGAAGGGTTACA	BTN3A1__NM_001145009	346	376	408	465
BTN3A1__NM_001145009_GATCTTCACGTTGATGTGAA	BTN3A1__NM_001145009	218	252	238	429
BTN3A1__NM_001145009_GGGAGAGAACATCCCGACTG	BTN3A1__NM_001145009	537	510	538	631
BTN3A1__NM_001145009_TGATCTTCACGTTGATGTGA	BTN3A1__NM_001145009	58	77	106	211
BTN3A2__NM_001197246_AAGCAGCAGCAAGATAGGCA	BTN3A2__NM_001197246	292	412	408	394
BTN3A2__NM_001197246_AATCTTCACGTCGAAGTGAA	BTN3A2__NM_001197246	23	36	28	35
BTN3A2__NM_001197246_CAGATGCTGCTACTTCATAT	BTN3A2__NM_001197246	1128	1002	1246	1626
BTN3A2__NM_001197246_GAAGCAGCAGCAAGATAGGC	BTN3A2__NM_001197246	201	164	258	277
BTN3A2__NM_001197246_TAATCTTCACGTCGAAGTGA	BTN3A2__NM_001197246	100	64	103	94
BTN3A2__NM_001197248_TTCTCCAATGGGAATACCAA	BTN3A2__NM_001197248	0	0	0	0
BTN3A3__NM_001242803_ATTCTGAAAACAGGATATAG	BTN3A3__NM_001242803	221	251	198	161
BTN3A3__NM_001242803_CACTTTCATTAGCTAAGCCC	BTN3A3__NM_001242803	658	483	687	870
BTN3A3__NM_001242803_CAGAAAAGAGTGGCATATTG	BTN3A3__NM_001242803	578	385	738	722
BTN3A3__NM_001242803_CAGGCACTAGGTCAGGATCG	BTN3A3__NM_001242803	738	441	759	390
BTN3A3__NM_001242803_CCACTTTCATTAGCTAAGCC	BTN3A3__NM_001242803	200	228	309	436
BTN3A3__NM_001242803_CCGGGCTTAGCTAATGAAAG	BTN3A3__NM_001242803	221	374	305	410
BTN3A3__NM_001242803_CTGAGCCTCCTAGGAAAGTG	BTN3A3__NM_001242803	179	165	255	342
BTN3A3__NM_001242803_GACTGATGGGAATAAGTATC	BTN3A3__NM_001242803	486	361	599	576
BTN3A3__NM_001242803_GGACTATGGGCCTGACTGAT	BTN3A3__NM_001242803	742	549	803	839
BTN3A3__NM_001242803_GGAGAACGGATACTGGACTA	BTN3A3__NM_001242803	723	660	898	1049
BTN3A3__NM_001242803_GGTGTCTCCAGGGAATGATC	BTN3A3__NM_001242803	103	99	109	113
BTN3A3__NM_001242803_TGGGGTATGTAGTAAGAACG	BTN3A3__NM_001242803	27	8	13	9
BTN3A3__NM_006994_AAGCAGCAACGATAGGCA	BTN3A3__NM_006994	444	560	477	481
BTN3A3__NM_006994_ACAGCCAGCATATCCATCGC	BTN3A3__NM_006994	576	429	650	606
BTN3A3__NM_006994_CCTGCCAGGGCCGCGATCCA	BTN3A3__NM_006994	304	348	416	357
BTN3A3__NM_006994_GAGCGCCCAGCCCTGGATCG	BTN3A3__NM_006994	1177	1473	1224	1616
BTN3A3__NM_006994_GCGAGAAGCAGCAACGAGAT	BTN3A3__NM_006994	517	614	462	605
BTN3A3__NM_006994_TGATCTTCACATTGAAGTGA	BTN3A3__NM_006994	71	138	106	114
BTNL10__NM_001287262_ATGTCCTCGGCACTGATATT	BTNL10__NM_001287262	403	193	412	400
BTNL10__NM_001287262_CACGTGGTCACTCATGAAGG	BTNL10__NM_001287262	648	748	913	1100
BTNL10__NM_001287262_CCTGTCCCAGAGCGTCAAGC	BTNL10__NM_001287262	92	23	113	122
BTNL10__NM_001287262_CTCATGCATGTGCACAGCTA	BTNL10__NM_001287262	230	113	254	236
BTNL10__NM_001287262_GAGCAAAAGTGGCAGTACAG	BTNL10__NM_001287262	514	483	453	491
BTNL10__NM_001287262_GGATGGTGTAAAGTTCGGCG	BTNL10__NM_001287262	395	432	424	511
BTNL10__NM_001287262_GGTGGCTGAGGCTGATAGAT	BTNL10__NM_001287262	253	189	315	273
BTNL10__NM_001287262_GTTCCCCAATATCAGTGCCG	BTNL10__NM_001287262	152	246	242	228
BTNL10__NM_001287262_TACTGCTGCCGCTTCAAGGA	BTNL10__NM_001287262	259	234	358	312
BTNL10__NM_001287262_TGCATGTGCACAGCTAGGGA	BTNL10__NM_001287262	73	13	76	76
BTNL10__NM_001287262_TGTCCTCGGCACTGATATTG	BTNL10__NM_001287262	324	399	348	325
BTNL10__NM_001287262_TTCAAGGATGGTGTAAAGTT	BTNL10__NM_001287262	20	23	24	27
BTNL3__NM_197975_AAGCAGATGCCACAGTATCG	BTNL3__NM_197975	299	189	317	200
BTNL3__NM_197975_AGCAGATGCCACAGTATCGA	BTNL3__NM_197975	771	1108	761	664
BTNL3__NM_197975_ATAGGACAGTGGCAAGTCAC	BTNL3__NM_197975	278	309	279	294
BTNL3__NM_197975_CCCTCGGACATCGGCCTGTA	BTNL3__NM_197975	1594	1890	1940	2235
BTNL3__NM_197975_CCTCGGACATCGGCCTGTAT	BTNL3__NM_197975	1197	885	1326	999
BTNL3__NM_197975_CTTCCCCATCTCTGTAGAGG	BTNL3__NM_197975	993	781	1144	979
BTNL3__NM_197975_GGCCTCCTCATCGTAAATCT	BTNL3__NM_197975	558	322	562	561
BTNL3__NM_197975_GTGCAGAGGCTATGGAAGTG	BTNL3__NM_197975	317	218	490	364
BTNL3__NM_197975_TCAGTTCTCCCTCGATACTG	BTNL3__NM_197975	311	259	302	268
BTNL3__NM_197975_TCCATCGTGGGATATGTTGA	BTNL3__NM_197975	706	729	705	686
BTNL3__NM_197975_TTCCCAGATTTACGATGAGG	BTNL3__NM_197975	106	77	91	133
BTNL3__NM_197975_TTGTGAAGGACTCCATTGCA	BTNL3__NM_197975	982	1002	1021	1166
BTNL8__NM_001040462_AACTAATCCTGCACCCATAG	BTNL8__NM_001040462	265	234	332	378
BTNL8__NM_001040462_AGATGCCACAGTATCAAGGC	BTNL8__NM_001040462	415	298	431	383
BTNL8__NM_001040462_ATGAGCATGCCGCATGGAAC	BTNL8__NM_001040462	288	236	281	234
BTNL8__NM_001040462_ATGCAGATGCCACAGTATCA	BTNL8__NM_001040462	585	622	593	557
BTNL8__NM_001040462_CTGACCGTCCAAGAGAACGC	BTNL8__NM_001040462	610	816	661	660
BTNL8__NM_001040462_GAATCCAGGGTACAGATAGG	BTNL8__NM_001040462	204	216	275	406
BTNL8__NM_001040462_GCTGGAAAAGATTACTGTGT	BTNL8__NM_001040462	1157	983	1319	1266
BTNL8__NM_001040462_GGCCTTCTGGTAGTAAGACT	BTNL8__NM_001040462	728	627	702	763
BTNL8__NM_001040462_GGTGGACCACGCTAGAGAAC	BTNL8__NM_001040462	43	116	95	81
BTNL8__NM_001040462_GTATCAAGGCAGGACAAAAC	BTNL8__NM_001040462	134	181	233	309
BTNL8__NM_001040462_TGAAGGATTCTATTGCGGAG	BTNL8__NM_001040462	106	271	157	153
BTNL8__NM_001040462_TGGGCCAGACAAGCCTGTCC	BTNL8__NM_001040462	184	91	144	138
BTNL8__NM_001159707_ACGGAGGACACAATAAAAGG	BTNL8__NM_001159707	678	737	787	765
BTNL8__NM_001159707_AGGACCAGTACCCATGATC	BTNL8__NM_001159707	708	727	816	665
BTNL8__NM_001159707_CACCTGGCTACCAAAGTACT	BTNL8__NM_001159707	1194	1335	1582	1711
BTNL8__NM_001159707_CTCACCTCCTATCTGTACCC	BTNL8__NM_001159707	879	962	881	1030
BTNL8__NM_001159707_GGGAAGACGCTGATAAAACG	BTNL8__NM_001159707	201	227	195	184
BTNL8__NM_001159707_GTAGCCAGGTGCCACGATAT	BTNL8__NM_001159707	781	508	827	844
BTNL8__NM_001159707_GTCTCCCGATCATGGGTACT	BTNL8__NM_001159707	682	614	837	1116
BTNL8__NM_001159707_GTGACTTTGTCTCCCGATCA	BTNL8__NM_001159707	206	246	212	200
BTNL8__NM_001159707_TGACTTTGTCTCCCGATCAT	BTNL8__NM_001159707	611	556	703	698
BTNL9__NM_152547_AACATCTGTGGTTGTCCGAG	BTNL9__NM_152547	408	212	440	430
BTNL9__NM_152547_ACAAGTCCAAAGCTCGTTTG	BTNL9__NM_152547	154	148	111	155
BTNL9__NM_152547_ACCAGCCAAAATGTCAGCGA	BTNL9__NM_152547	387	361	468	308
BTNL9__NM_152547_ACCCCAAGCCTAAGGTTCAG	BTNL9__NM_152547	1502	925	1759	1288
BTNL9__NM_152547_ACTTGGTGACTCTAGGTGAC	BTNL9__NM_152547	267	199	283	264
BTNL9__NM_152547_AGACCCTCACCTCTCCCTTG	BTNL9__NM_152547	1430	977	1656	1325
BTNL9__NM_152547_AGACTGCTGGTCAGCGATAC	BTNL9__NM_152547	293	258	390	444
BTNL9__NM_152547_AGCTGTGGCCATAGGTGGCA	BTNL9__NM_152547	209	155	286	280
BTNL9__NM_152547_AGGTCTGACTCCGGAACCAG	BTNL9__NM_152547	76	102	114	64
BTNL9__NM_152547_ATACACATCTACGTAGGCAA	BTNL9__NM_152547	201	249	283	285
BTNL9__NM_152547_ATACTGGCTTCAAGGAGTCT	BTNL9__NM_152547	579	368	583	452
BTNL9__NM_152547_CAAATTAACTGATCCCTCTA	BTNL9__NM_152547	764	791	826	937
BTNL9__NM_152547_CAGCCAAAATGTCAGCGAGG	BTNL9__NM_152547	529	447	659	572
BTNL9__NM_152547_CCCTCTGACAAGGGCACATA	BTNL9__NM_152547	39	54	24	12
BTNL9__NM_152547_CTCAAAACGTGACGGCAACC	BTNL9__NM_152547	166	134	153	235
BTNL9__NM_152547_GAGTTGGTGGTCCAGATAGC	BTNL9__NM_152547	79	30	72	93
BTNL9__NM_152547_GCAACGAGTTCACCTAAGTA	BTNL9__NM_152547	868	892	976	1192
BTNL9__NM_152547_GGGAAAGGTTACCTTTACAA	BTNL9__NM_152547	247	181	305	157
BTNL9__NM_152547_GTATCAGTTCTGTAATAAAG	BTNL9__NM_152547	155	73	169	214
BTNL9__NM_152547_GTCAAGGACGACATCGCCTA	BTNL9__NM_152547	969	914	1064	1141
BTNL9__NM_152547_GTCCTGGGCATCCCAGACGA	BTNL9__NM_152547	572	578	711	581
BTNL9__NM_152547_GTGACGGCAACCCGGCAAAA	BTNL9__NM_152547	227	212	271	283
BTNL9__NM_152547_TGGAGTTCCCGTGCCACCTA	BTNL9__NM_152547	57	37	54	45
BTNL9__NM_152547_TGTGGGACAATTTGAACATC	BTNL9__NM_152547	325	264	378	373
BTNL9__NM_152547_TGTTCATTGTACTATCAATG	BTNL9__NM_152547	191	151	192	214
BTNL9__NM_152547_TTTAAGTGCGTTATAGAGAC	BTNL9__NM_152547	31	53	84	48
BTRC__NM_001256856_AAGTGCTCTATAAAAAGAGT	BTRC__NM_001256856	202	301	266	224
BTRC__NM_001256856_ACCATCCTCTTCGTTCTGCC	BTRC__NM_001256856	986	1455	1275	1508
BTRC__NM_001256856_AGAGGCCTGGCAGAACGAAG	BTRC__NM_001256856	264	189	233	219
BTRC__NM_001256856_AGGCACTCAAGTTTATGGTG	BTRC__NM_001256856	237	264	195	140
BTRC__NM_001256856_AGTGCTCTATAAAAAGAGTT	BTRC__NM_001256856	127	90	121	161
BTRC__NM_001256856_CACATAGTGATTTGGCATCC	BTRC__NM_001256856	339	260	377	322
BTRC__NM_001256856_CCTTGATTGTGTTGTCTCGA	BTRC__NM_001256856	855	1259	1112	1531
BTRC__NM_001256856_CTGCGATGCCTGTATAACCC	BTRC__NM_001256856	397	214	471	419
BTRC__NM_001256856_TGAGAACATTCTGTCATACC	BTRC__NM_001256856	371	259	500	578
BTRC__NM_001256856_TTATACAGGCATCGCAGCGA	BTRC__NM_001256856	167	196	188	170
BTRC__NM_001256856_TTCAGCAGCACATAGTGATT	BTRC__NM_001256856	17	46	31	28
BTRC__NM_001256856_TTGGGAGGAGCATTCCCGTC	BTRC__NM_001256856	530	356	669	670
BTRC__NM_003939_CTCTGGTATTATCTTCCTAG	BTRC__NM_003939	166	64	164	155
BTRC__NM_003939_GATCTGGGATAAAAACACAT	BTRC__NM_003939	275	300	260	341
BUB1__NM_001278616_ACACTTGGCCCCATACGTGC	BUB1__NM_001278616	1140	304	1157	323
BUB1__NM_001278616_AGTTCCTGCAACAACAATAC	BUB1__NM_001278616	192	65	207	86
BUB1__NM_001278616_CAAGGAGAAGCTTATTCGTG	BUB1__NM_001278616	861	119	888	213
BUB1__NM_001278616_CCCGCCCAGGCAATGTACAG	BUB1__NM_001278616	770	346	917	471
BUB1__NM_001278616_CCGCCCAGGCAATGTACAGA	BUB1__NM_001278616	1229	933	1523	1557
BUB1__NM_001278616_CGCTTCCCAGGAAAGGTCCG	BUB1__NM_001278616	459	170	652	210
BUB1__NM_001278616_GCAAGGAGAAGCTTATTCGT	BUB1__NM_001278616	488	10	516	127
BUB1__NM_001278616_GCAGGTTGTTATGTATTGCA	BUB1__NM_001278616	570	48	630	139
BUB1__NM_001278616_TGATGAATCTTGGGTCATTG	BUB1__NM_001278616	256	387	274	345
BUB1__NM_001278616_TGCAAGGAGAAGCTTATTCG	BUB1__NM_001278616	431	215	483	299
BUB1__NM_001278616_TTGAGTTTCTGTACAACCAT	BUB1__NM_001278616	109	182	126	116
BUB1__NM_001278616_TTTACCTTGAGCTGGCAAAT	BUB1__NM_001278616	46	66	39	65
BUB1__NM_001278617_GCCCTTGTAGCTCTGCATGT	BUB1__NM_001278617	450	391	460	299
BUB1B__NM_001211_AAGTAACTGTACATATCCAA	BUB1B__NM_001211	252	112	293	192
BUB1B__NM_001211_AATGTACAACCTTTAAGGCA	BUB1B__NM_001211	367	19	392	18
BUB1B__NM_001211_ACCCTCTGGATGTTTGGGAT	BUB1B__NM_001211	1929	876	2216	1346
BUB1B__NM_001211_AGCCAGAGATTGAGAAATCG	BUB1B__NM_001211	324	60	363	96
BUB1B__NM_001211_AGGGAAAAGTAGAGACAAAT	BUB1B__NM_001211	117	120	92	82
BUB1B__NM_001211_CTCTGGATGTTTGGGATAGG	BUB1B__NM_001211	147	1	156	10
BUB1B__NM_001211_CTGCTGAAGAGTATTGTTAC	BUB1B__NM_001211	331	82	326	63
BUB1B__NM_001211_GAAATGACCCTCTGGATGTT	BUB1B__NM_001211	472	344	439	351
BUB1B__NM_001211_TACAACCTTTAAGGCAAGGG	BUB1B__NM_001211	161	0	179	13
BUB1B__NM_001211_TACTTGCAGACAACCAGGGAT	BUB1B__NM_001211	163	24	243	38
BUB1B__NM_001211_TCTGGATGTTTGGGATAGGT	BUB1B__NM_001211	228	0	226	2
BUB1B__NM_001211_TGCCAGTGCTCCCTGAAGCG	BUB1B__NM_001211	295	6	290	10
BUB1B__NM_001211_TGCTCAGTTCTATATCTCAT	BUB1B__NM_001211	471	1	582	0
BUB1B__NM_001211_TTCTTCACAAAAGTTAGACA	BUB1B__NM_001211	3	59	0	0
BUB3__NM_001007793_ATGTGATGGTCACTGGAAGT	BUB3__NM_001007793	499	1	636	7
BUB3__NM_001007793_CAGCTTGAACTCGTTAGAAC	BUB3__NM_001007793	214	41	209	60
BUB3__NM_001007793_CAGTATTCAACACATCTGAT	BUB3__NM_001007793	493	10	607	7
BUB3__NM_001007793_CGGCACATCGTAGAGACGCA	BUB3__NM_001007793	163	1	147	11
BUB3__NM_001007793_CTGGACTGCGCCTTCTACGT	BUB3__NM_001007793	1017	6	908	15
BUB3__NM_001007793_GAGAAGGTCCAGCAGCATTACA	BUB3__NM_001007793	122	21	217	16
BUB3__NM_001007793_GGATCAGACAGTTAAACTGT	BUB3__NM_001007793	329	21	375	14
BUB3__NM_001007793_GTACTTGAGCCGCATGGAGT	BUB3__NM_001007793	410	0	371	7
BUB3__NM_001007793_GTATTCAACACATCTGATAG	BUB3__NM_001007793	229	0	214	0
BUB3__NM_001007793_GTCTGGAGACCGGCTGATTG	BUB3__NM_001007793	340	16	288	27
BUB3__NM_001007793_TATAGGATCCAACGCATGCC	BUB3__NM_001007793	175	14	200	37
BUB3__NM_001007793_TTGAGCCGCATGGAGTTGGC	BUB3__NM_001007793	262	0	275	0
BUB3__NM_004725_AACTGTTCAGATACAGCAAC	BUB3__NM_004725	550	626	669	802
BUB3__NM_004725_ACAAACATTTATTTCCACAG	BUB3__NM_004725	219	290	330	211
BUB3__NM_004725_ACAGCAACTGGGCTTACTCA	BUB3__NM_004725	888	1013	854	827
BUB3__NM_004725_ACCACTTTCACAGGATGATT	BUB3__NM_004725	535	592	552	641
BUB3__NM_004725_ACTTTCACAGGATGATTAGG	BUB3__NM_004725	239	235	231	278
BUB3__NM_004725_AGCAGAACACGAACTTAGCT	BUB3__NM_004725	398	446	439	536
BUB3__NM_004725_CAGCGGCTGGTCAACTGAAA	BUB3__NM_004725	716	707	624	613
BUB3__NM_004725_CATTCTTTCTGGACCTTAAA	BUB3__NM_004725	167	192	174	202
BUB3__NM_004725_CTGGGCTGTTAAACAAAGCG	BUB3__NM_004725	215	216	211	196
BUB3__NM_004725_GTATGTCTCTGAGTAGGACT	BUB3__NM_004725	378	455	480	549
BUB3__NM_004725_TAACTCTAACACATCCCTTA	BUB3__NM_004725	642	388	776	606
BUB3__NM_004725_TAATTGCAGTGCATTTAGAC	BUB3__NM_004725	324	409	530	521
BUB3__NM_004725_TAGTAATAAATCCACCATTG	BUB3__NM_004725	201	76	150	150
BUB3__NM_004725_TATTTGGAACTACCTAGAAT	BUB3__NM_004725	436	417	614	679
BUB3__NM_004725_TGTTAAACAAAGCGAGGTTG	BUB3__NM_004725	67	22	75	77
BUB3__NM_004725_TTTGCTGCATTAAAGGTATT	BUB3__NM_004725	316	512	423	583
BUD13__NM_001159736_ACCCTCCTAGGAGATGAATC	BUD13__NM_001159736	571	504	747	679
BUD13__NM_001159736_ACGCTTCAGATACTCGGCCT	BUD13__NM_001159736	1838	2043	2337	2347
BUD13__NM_001159736_AGGATCGGGGTGTCATGAC	BUD13__NM_001159736	459	502	453	410
BUD13__NM_001159736_CCATTTGGCACTGGAACGAA	BUD13__NM_001159736	51	106	64	75
BUD13__NM_001159736_CCCTCCTAGGAGATGGATCC	BUD13__NM_001159736	325	347	310	358
BUD13__NM_001159736_CCTCCTAGGAGATGAATCCG	BUD13__NM_001159736	643	563	783	509
BUD13__NM_001159736_CCTCCTAGGAGATGGATCCG	BUD13__NM_001159736	501	353	532	456
BUD13__NM_001159736_GCACCTGAAGAATTATGCTG	BUD13__NM_001159736	504	424	557	361
BUD13__NM_001159736_GTCGCAAGCGTCGCAAAAAG	BUD13__NM_001159736	152	210	213	106
BUD13__NM_001159736_TATCTGAAGCGTTACTTGTC	BUD13__NM_001159736	484	219	534	250
BUD13__NM_001159736_TCTGAAGCGTTACTTGTCCG	BUD13__NM_001159736	1254	699	1345	729
BUD13__NM_001159736_TTGTGGATGATGATGTGAGC	BUD13__NM_001159736	158	163	226	153
BUD31__NM_003910_AAAAGAGGTCGAAGATGTAG	BUD31__NM_003910	439	103	556	97
BUD31__NM_003910_AGCTGAAACAGAACCGCATG	BUD31__NM_003910	211	48	255	65
BUD31__NM_003910_AGTTGGAGCACGGTCTCTAT	BUD31__NM_003910	878	441	934	516
BUD31__NM_003910_CCCGTGTCTGAATGTGACCGC	BUD31__NM_003910	412	0	637	51
BUD31__NM_003910_GATGCAGTTCGTCCCGAAGT	BUD31__NM_003910	790	110	998	141
BUD31__NM_003910_TGGCTTTCCGCTTGTAAAAG	BUD31__NM_003910	12	11	15	2
BVES__NM_001199563_AAGATTGAAAAGGAACTCAG	BVES__NM_001199563	153	126	76	68
BVES__NM_001199563_AGTCTTCTGAACAAATCTGG	BVES__NM_001199563	251	165	262	207
BVES__NM_001199563_AGTGGCCCAGACGATATAAA	BVES__NM_001199563	177	273	239	159
BVES__NM_001199563_ATATGAAGGTGAAGAGTAGT	BVES__NM_001199563	204	289	245	207
BVES__NM_001199563_ATGTACCCTTTATATCGTCT	BVES__NM_001199563	503	500	453	395
BVES__NM_001199563_CAAGGCACATCGGTAGAGAG	BVES__NM_001199563	275	223	266	335
BVES__NM_001199563_CCTTCAAGAGAATACTCAGA	BVES__NM_001199563	436	441	636	543
BVES__NM_001199563_GATGTACCCTTTATATCGTC	BVES__NM_001199563	648	791	764	1036
BVES__NM_001199563_GATTTGTTCAGAAGACTAAC	BVES__NM_001199563	121	119	84	97
BVES__NM_001199563_GTGCCTTGGATATAATGATC	BVES__NM_001199563	196	246	219	292
BVES__NM_001199563_TCACAAGTGGTCTATTGGA	BVES__NM_001199563	32	13	68	77
BVES__NM_001199563_TGGCAGTTGATTCTCTCAAT	BVES__NM_001199563	86	17	105	48
BYSL__NM_004053_AAACCTGGAGCCTGGTTCAA	BYSL__NM_004053	686	73	646	75
BYSL__NM_004053_AGCCGCGTGGTGCGTTCCCG	BYSL__NM_004053	1170	360	1120	382
BYSL__NM_004053_CAACGTCATCTCGTACTCGA	BYSL__NM_004053	1906	212	2288	356
BYSL__NM_004053_CATGCGTTCCTTCAGGTTAG	BYSL__NM_004053	444	21	597	123
BYSL__NM_004053_CCCACGGGCCGCCTTGAATT	BYSL__NM_004053	29	33	54	69
BYSL__NM_004053_CTCAGAACTAAACAGGGATA	BYSL__NM_004053	164	139	142	131
BYSL__NM_004053_CTCGAGGCCGAGCATGGGAC	BYSL__NM_004053	287	388	248	296
BYSL__NM_004053_CTGATTCCACTGTGCGAGTC	BYSL__NM_004053	496	469	473	702
BYSL__NM_004053_GAAGCGGAGGAAGAGTATGT	BYSL__NM_004053	314	1	448	13
BYSL__NM_004053_GCAACGTCATCTCGTACTCG	BYSL__NM_004053	2121	2566	2258	3132
BYSL__NM_004053_GTATTATCTAAGTACCGCAG	BYSL__NM_004053	8	3	32	17
BYSL__NM_004053_GTCATCTTCAGTCACCAACG	BYSL__NM_004053	226	227	237	219
BYSL__NM_004053_GTGCTTGCTGCAAAATCCGT	BYSL__NM_004053	325	1	339	5
BYSL__NM_004053_TACTACCTGGAGTGCAACAC	BYSL__NM_004053	204	23	250	1
BYSL__NM_004053_TAGCATATGGTATCTCAAGT	BYSL__NM_004053	221	182	309	344
BYSL__NM_004053_TCATCTTCAGTCACCAACGG	BYSL__NM_004053	65	42	93	73
BZRAP1__NM_001261835_ACAACCTACCCGTGATTCTA	BZRAP1__NM_001261835	651	483	583	390
BZRAP1__NM_001261835_AGCAGATTGCTGCCTTGCAG	BZRAP1__NM_001261835	321	223	336	260
BZRAP1__NM_001261835_AGGTGGAGCATGAACAGGCT	BZRAP1__NM_001261835	82	94	85	67
BZRAP1__NM_001261835_CAACCTACCCGTGATTCTAG	BZRAP1__NM_001261835	721	553	777	559
BZRAP1__NM_001261835_CACTGCCCACCTGGCATAGC	BZRAP1__NM_001261835	0	0	0	0
BZRAP1__NM_001261835_CCCAAAGCTCATATTCAGCT	BZRAP1__NM_001261835	956	1248	1187	1802
BZRAP1__NM_001261835_CCCTGGAGCCATGGAGCCAT	BZRAP1__NM_001261835	607	555	562	411
BZRAP1__NM_001261835_GCCGATCGAAGTCCCGCACG	BZRAP1__NM_001261835	1596	956	1351	985
BZRAP1__NM_001261835_GCGCTGTGCCATCCTTAAGG	BZRAP1__NM_001261835	836	611	798	471
BZRAP1__NM_001261835_GCGGAAGAAATGCGAGAGCC	BZRAP1__NM_001261835	236	425	248	293
BZRAP1__NM_001261835_GCTCCCCTAGAATCACGGGT	BZRAP1__NM_001261835	1142	1248	1259	778
BZRAP1__NM_001261835_TTGCTGAGCTCCGATTCCTG	BZRAP1__NM_001261835	1030	984	1198	1110
BZRAP1__NM_024418_ACAGGCTCGGCTCAGCCTAC	BZRAP1__NM_024418	556	321	411	287
BZW1__NM_001207067_AACTGAAACCGCTACTGATT	BZW1__NM_001207067	1587	1458	2042	1342
BZW1__NM_001207067_AGAGTGTTTCTGCATATCGA	BZW1__NM_001207067	183	130	227	189
BZW1__NM_001207067_ATTCAAGGCTTAACTGAAAC	BZW1__NM_001207067	89	133	97	80
BZW1__NM_001207067_CAGCAAAAGCCAACGCTATC	BZW1__NM_001207067	168	90	150	224
BZW1__NM_001207067_CAGTTGTCATCGGAATAGTC	BZW1__NM_001207067	419	229	408	361
BZW1__NM_001207067_CTTGCTGCCTTTACTACTCA	BZW1__NM_001207067	720	1026	945	1349
BZW1__NM_001207067_GCAAACCATCGGAGCTCGTA	BZW1__NM_001207067	298	415	329	577
BZW1__NM_001207067_GTTCGGAATCAGCAAACCAT	BZW1__NM_001207067	169	327	257	180
BZW1__NM_001207067_TACTGCTTCCAAATCAGTAC	BZW1__NM_001207067	171	79	98	146
BZW1__NM_001207067_TCATCTGCCAGTGTACCACC	BZW1__NM_001207067	429	366	409	447
BZW1__NM_001207067_TCCAGCTGAAGTCCTGAGCG	BZW1__NM_001207067	541	590	644	763
BZW1__NM_001207068_GGCGGATGTACGGGGCGCCT	BZW1__NM_001207068	1098	966	1194	1339
BZW1__NM_001207068_TGACTGTGGTCGCTCGTTG	BZW1__NM_001207068	690	553	791	845
BZW2__NM_001159767_AACCATCCGAAACTATGCTC	BZW2__NM_001159767	139	56	104	110
BZW2__NM_001159767_AACCGCTGGCCTGTTAGCAC	BZW2__NM_001159767	612	794	680	770
BZW2__NM_001159767_ACTTGTCCAGGGGCTTAATG	BZW2__NM_001159767	734	876	889	1002
BZW2__NM_001159767_AGACACACTCTTCGATATCC	BZW2__NM_001159767	366	473	366	489
BZW2__NM_001159767_AGGTCTTCAATAAACTCATC	BZW2__NM_001159767	121	157	190	153
BZW2__NM_001159767_ATCTATGCGCGTTCCTCCAG	BZW2__NM_001159767	505	436	626	475
BZW2__NM_001159767_CAGGCCAGCGGTTCAAAACT	BZW2__NM_001159767	619	377	717	522
BZW2__NM_001159767_CATCAGGAGATATAAGTATT	BZW2__NM_001159767	570	645	617	635
BZW2__NM_001159767_CGGTTCAAAACTCGGAAAAG	BZW2__NM_001159767	13	0	19	15
BZW2__NM_001159767_CTGTTTCGGAAAAAGGCTTTA	BZW2__NM_001159767	116	236	148	172
BZW2__NM_001159767_TACCTGAGCATAGTTTCGGA	BZW2__NM_001159767	416	284	366	479
BZW2__NM_001159767_TCAAAACTCGGAAAAGGGGT	BZW2__NM_001159767	133	169	184	287
C10orf10__NM_007021_AACGTGCGGTGATGTCTCGC	C10orf10__NM_007021	944	915	1012	1178
C10orf10__NM_007021_AGAGCTCTGACCTAAGAAGC	C10orf10__NM_007021	238	164	195	207
C10orf10__NM_007021_AGAGTTCATGGATCACCGGG	C10orf10__NM_007021	385	463	421	435
C10orf10__NM_007021_ATCCTGCGGTGGTCTTGCCA	C10orf10__NM_007021	83	32	75	37
C10orf10__NM_007021_CGTGAGGTCTATATCTCGAC	C10orf10__NM_007021	913	984	1057	804
C10orf10__NM_007021_CTCCGTGGTCTCCCGAATTG	C10orf10__NM_007021	1027	1188	1179	1390
C10orf10__NM_007021_CTGCTGGCCACTGAAACGTG	C10orf10__NM_007021	183	139	167	184
C10orf10__NM_007021_CTGGGGTCCACATAGACAGA	C10orf10__NM_007021	400	384	330	416
C10orf10__NM_007021_GACATCACCGCACGGTTCAG	C10orf10__NM_007021	1223	1240	1352	1591
C10orf10__NM_007021_GCCCACAATTCGGGAGACCA	C10orf10__NM_007021	609	752	693	861
C10orf10__NM_007021_GGGTCTCCACAGTATCAGCCAT	C10orf10__NM_007021	830	1011	1007	1322
C10orf10__NM_007021_TGGCCCATCTCGCCCACAAT	C10orf10__NM_007021	85	144	127	126
C10orf105__NM_001164375_AGGGGCTGTCGAGGGACGGT	C10orf105__NM_001164375	510	670	618	666
C10orf105__NM_001164375_AGTTGCCTCTGCAAGGTCC	C10orf105__NM_001164375	99	121	123	132
C10orf105__NM_001164375_CAGGGGCTGTCGAGGGACGG	C10orf105__NM_001164375	724	950	895	941
C10orf105__NM_001164375_GAGCGTCATGAACAGCAGAC	C10orf105__NM_001164375	139	158	158	185
C10orf105__NM_001164375_GGAATCTACCAAGATGTAAT	C10orf105__NM_001164375	482	462	340	466
C10orf105__NM_001164375_TAGATTCCATGTAGTCACAG	C10orf105__NM_001164375	153	251	168	205
C10orf107__NM_173554_ATTTCCGTATCCAATATTGT	C10orf107__NM_173554	238	261	228	244
C10orf107__NM_173554_CATTTCCGTATCCAATATTG	C10orf107__NM_173554	810	983	838	1245
C10orf107__NM_173554_GAAAAATTGAAAAAGGCCTA	C10orf107__NM_173554	55	69	51	63
C10orf107__NM_173554_GAGGTTGTCAAGTCTGCATG	C10orf107__NM_173554	209	246	228	254
C10orf107__NM_173554_GCCAGGGAAGAAATTGTGAT	C10orf107__NM_173554	877	1062	1064	1053
C10orf107__NM_173554_GCCCCCCACAATATTGGATA	C10orf107__NM_173554	896	1063	893	1274
C10orf107__NM_173554_GGAGAAGTTATGGCTGAAAT	C10orf107__NM_173554	584	473	549	761
C10orf11__NM_032024_AAGGGCGTGTAGTGGCGCTC	C10orf11__NM_032024	175	70	182	158
C10orf11__NM_032024_AAGTGTCGCTACGTTTACTA	C10orf11__NM_032024	1041	1367	1223	1561
C10orf11__NM_032024_AGTGTCGCTACGTTTACTAT	C10orf11__NM_032024	1914	1674	2108	2251
C10orf11__NM_032024_CAGAAGGCAAGGGCGTGTAG	C10orf11__NM_032024	682	742	759	827
C10orf11__NM_032024_CATCTTGGACAACAATCAGC	C10orf11__NM_032024	361	341	347	383
C10orf11__NM_032024_CGAGAGGAGGCGTTGGTCAG	C10orf11__NM_032024	1876	1985	2202	2563
C10orf11__NM_032024_CGCCTCCTCTCGTTCTTGTC	C10orf11__NM_032024	3027	3624	3157	4300
C10orf11__NM_032024_GAAAAGTATTTGTCACTCAG	C10orf11__NM_032024	296	332	293	568
C10orf11__NM_032024_GGCATCCAGAAATTTCAAGT	C10orf11__NM_032024	1071	1268	1250	1471
C10orf11__NM_032024_TGGAAGGACTGAGCGCATTC	C10orf11__NM_032024	86	70	194	300
C10orf111__NM_153244_AAATCAAGATAAAAGGGAGA	C10orf111__NM_153244	206	180	190	192
C10orf111__NM_153244_AAGGAGGCAGATACGTAAAG	C10orf111__NM_153244	158	267	155	157
C10orf111__NM_153244_ACCGCGAAAATCAAGATAAA	C10orf111__NM_153244	395	738	489	524
C10orf111__NM_153244_ATGCCTATGGGCAATAATGC	C10orf111__NM_153244	187	144	204	297
C10orf111__NM_153244_ATGGAGACTCAGGCTACCAT	C10orf111__NM_153244	168	356	374	405
C10orf111__NM_153244_CAGAAACTATACCAGAAAAT	C10orf111__NM_153244	539	716	742	735
C10orf111__NM_153244_CTTGGCCAGAACTTAGAAGA	C10orf111__NM_153244	120	107	121	129
C10orf111__NM_153244_GAAGGGGGCCCAAGTAAGGC	C10orf111__NM_153244	109	119	142	131
C10orf111__NM_153244_TGCCTATGGGCAATAATGCA	C10orf111__NM_153244	173	121	245	349
C10orf111__NM_153244_TGGGGTAAAACACATGCCTA	C10orf111__NM_153244	103	113	143	159
C10orf111__NM_153244_TTCCCTGCATTATTGCCCAT	C10orf111__NM_153244	203	161	269	289
C10orf113__NM_001010896_AAAGTTCAACGTATCTAAGA	C10orf113__NM_001010896	110	88	161	150
C10orf113__NM_001010896_ATAGGCCTGGTAAGGCAGAA	C10orf113__NM_001010896	51	54	39	13
C10orf113__NM_001010896_CTCTCTCATATAAGCTAG	C10orf113__NM_001010896	35	3	24	28
C10orf113__NM_001010896_GACCAGAAGGTATAACCAAA	C10orf113__NM_001010896	96	162	169	86
C10orf113__NM_001010896_TGAAAGAAGAATCTACTCTA	C10orf113__NM_001010896	237	201	227	302
C10orf113__NM_001177483_AGCTGTCCGTTCTTCAAGCC	C10orf113__NM_001177483	1307	1494	1259	1680
C10orf113__NM_001177483_TTTATCCCATAGGCCTGGTA	C10orf113__NM_001177483	227	178	228	320
C10orf12__NM_015652_AAAGATGGTTGGTTAGGCCC	C10orf12__NM_015652	164	129	118	89
C10orf12__NM_015652_ACAGCCAGAAAGAGTACTCG	C10orf12__NM_015652	480	400	509	352
C10orf12__NM_015652_CAACCTCCATCAAGACAGCT	C10orf12__NM_015652	486	343	551	499
C10orf12__NM_015652_CATTTACATCCCAGTGATAA	C10orf12__NM_015652	248	296	267	292
C10orf12__NM_015652_CCTTATCACTGGGATGTAAA	C10orf12__NM_015652	299	360	373	355
C10orf12__NM_015652_GACAGCTCGGAAAAGTAAAA	C10orf12__NM_015652	41	97	53	55
C10orf12__NM_015652_GCTGCCAACTGTTCGTACAC	C10orf12__NM_015652	631	554	602	352
C10orf12__NM_015652_GCTTGGAACTCAGGGTTTAT	C10orf12__NM_015652	563	623	630	584
C10orf12__NM_015652_GGCTTGGAACTCAGGGTTTA	C10orf12__NM_015652	286	257	313	442
C10orf12__NM_015652_TCAAACTGGCCCTTATCACT	C10orf12__NM_015652	1212	1154	1283	942
C10orf12__NM_015652_TCAAAGTGGCATTCTAGTTAT	C10orf12__NM_015652	1067	1311	1290	1434
C10orf12__NM_015652_TCCAAAAACACCTTTGCGCC	C10orf12__NM_015652	599	567	567	688
C10orf120__NM_001010912_AATGGAAGAATGACTGTCAG	C10orf120__NM_001010912	271	180	240	381
C10orf120__NM_001010912_AGCCCCAACGTGTTGCCAAG	C10orf120__NM_001010912	287	311	369	362
C10orf120__NM_001010912_ATAGTTGTCTTCCTTCGTC	C10orf120__NM_001010912	404	224	321	456
C10orf120__NM_001010912_ATCCTCTTGGCAACACGTTG	C10orf120__NM_001010912	220	178	207	161
C10orf120__NM_001010912_CGTTGGGGCTTGATCCTGAA	C10orf120__NM_001010912	493	578	751	787
C10orf120__NM_001010912_GATCCTCTTGGCAACACGTT	C10orf120__NM_001010912	242	323	283	350
C10orf120__NM_001010912_GCGTGTAAACCTTCAATGT	C10orf120__NM_001010912	1064	1107	1369	1659
C10orf120__NM_001010912_GGTTGGCATCATCACAATTA	C10orf120__NM_001010912	217	200	212	352
C10orf120__NM_001010912_GTGTACCCCTAGAAAAAATA	C10orf120__NM_001010912	79	87	95	63
C10orf120__NM_001010912_GTTGGCATCATCACAATTAT	C10orf120__NM_001010912	304	508	343	546
C10orf120__NM_001010912_TAAAATGCCACAACGAGAGC	C10orf120__NM_001010912	108	146	92	105
C10orf120__NM_001010912_TATTTCCCAAGGGCAATCCG	C10orf120__NM_001010912	154	129	131	140
C10orf126__NM_001278522_CCTTCAGACTCCACCGCCAC	C10orf126__NM_001278522	1571	1578	1736	2337
C10orf126__NM_001278522_GCAGGACAAGGATCAGTGGC	C10orf126__NM_001278522	534	583	425	567
C10orf126__NM_001278522_TTGGGCTCAGGATTGCGGCT	C10orf126__NM_001278522	369	361	448	456
C10orf128__NM_001010863_AAGACCCCTGGGGCTGAAAT	C10orf128__NM_001010863	378	330	370	448
C10orf128__NM_001010863_ACCCACAGCAGTCCCGATGA	C10orf128__NM_001010863	1169	1123	1270	1119
C10orf128__NM_001010863_CACCCACAGCAGTCCCGATG	C10orf128__NM_001010863	1320	1307	1534	1414
C10orf128__NM_001010863_CCGACCTGAAAAGCACGCCT	C10orf128__NM_001010863	1002	984	1346	1238
C10orf128__NM_001010863_CGACCTGAAAAGCACGCCTG	C10orf128__NM_001010863	2782	2691	2948	2857
C10orf128__NM_001010863_CTTGGGGCTCTCTTCTTTAG	C10orf128__NM_001010863	358	529	537	615
C10orf128__NM_001010863_GGCCAGGAAGCCAGCAGATA	C10orf128__NM_001010863	286	273	332	325
C10orf128__NM_001010863_GTGGGTGTCGCCATATCTGC	C10orf128__NM_001010863	229	241	259	249
C10orf128__NM_001010863_TCCGACCTGAAAAGCACGCC	C10orf128__NM_001010863	459	543	521	804
C10orf128__NM_001010863_TGCACAGGTGATTGAGCTGT	C10orf128__NM_001010863	337	345	410	510
C10orf128__NM_001288742_AAAAGAGAAGTCATTCCTCG	C10orf128__NM_001288742	532	866	622	861
C10orf128__NM_001288742_AAATATGCCTATTAACCTCG	C10orf128__NM_001288742	290	361	330	413
C10orf128__NM_001288742_AGTCATTCCTCGAGGTTAAT	C10orf128__NM_001288742	382	413	394	392
C10orf131__NM_001130446_AACACCAAGTGAAAAGCATT	C10orf131__NM_001130446	242	334	295	380
C10orf131__NM_001130446_TTGATAGCGAAATACATCAA	C10orf131__NM_001130446	315	245	337	440
C10orf131__NM_001130446_TTGGGATAACTACGGTTTAC	C10orf131__NM_001130446	73	86	111	84
C10orf2__NM_001163812_CACGCAGGGGTAACAAGATA	C10orf2__NM_001163812	120	76	123	113
C10orf2__NM_001163812_CAGGGGTAACAAGATACGGA	C10orf2__NM_001163812	122	94	151	123
C10orf2__NM_001163812_CCCCTGCGTGGGGAGTGGAT	C10orf2__NM_001163812	490	156	543	348
C10orf2__NM_001163812_CGTATCTTGTTACCCCTGCG	C10orf2__NM_001163812	696	683	645	1050
C10orf2__NM_001163812_GCAGGGGTAACAAGATACGG	C10orf2__NM_001163812	386	209	363	201
C10orf2__NM_001163812_GCCCTCCTCGCAGACGTTAC	C10orf2__NM_001163812	781	398	788	675
C10orf2__NM_001163812_GTATCTTGTTACCCCTGCGT	C10orf2__NM_001163812	480	131	464	114
C10orf2__NM_001163812_TACGGAGGGGGGTACCCACTT	C10orf2__NM_001163812	669	294	514	253
C10orf2__NM_001163812_TCCTCGCAGACGTTACAGGA	C10orf2__NM_001163812	348	93	360	161
C10orf2__NM_001163812_TCCTGTAACGTCTGCGAGGA	C10orf2__NM_001163812	417	898	434	576
C10orf2__NM_001163812_TCCTTCCTGTAACGTCTGCG	C10orf2__NM_001163812	394	175	444	174
C10orf2__NM_001163812_TGCGTGGGGAGTGGATGGGT	C10orf2__NM_001163812	463	22	414	37
C10orf2__NM_001163813_ACTGGACAAATATGATCACT	C10orf2__NM_001163813	910	48	905	102
C10orf2__NM_001163813_ATGGAAAGTCATGAAATAGA	C10orf2__NM_001163813	149	6	211	18
C10orf2__NM_001163813_CAATAACTGCCATGTGACAC	C10orf2__NM_001163813	1104	857	1393	1304
C10orf2__NM_001163813_CATGCTGACACAGTTTGCCG	C10orf2__NM_001163813	2054	56	2021	105
C10orf2__NM_001163813_CATGGAAAGTCATGAAATAG	C10orf2__NM_001163813	321	16	401	22
C10orf2__NM_001163813_CGGCCTTCACTTTGAACGCT	C10orf2__NM_001163813	584	721	820	1106
C10orf2__NM_001163813_GCCCTCTTCTGGCAAAAAGG	C10orf2__NM_001163813	112	162	176	105
C10orf2__NM_001163813_GGAAGCTGGTAACCGGGCCA	C10orf2__NM_001163813	436	749	431	571
C10orf2__NM_001163813_GTGTCCAAGAACCGCTTTGA	C10orf2__NM_001163813	290	279	356	415
C10orf2__NM_001163813_TGGAAAGTCATGAAATAGAG	C10orf2__NM_001163813	161	0	171	1
C10orf2__NM_001163814_CCAAAGTGAGTGGCCTTTAG	C10orf2__NM_001163814	320	422	324	505
C10orf25__NM_001039380_AAACGAACAACGCTTTCGGG	C10orf25__NM_001039380	691	651	756	863
C10orf25__NM_001039380_AAAGTATGCAGTTTCTAGGT	C10orf25__NM_001039380	400	368	340	221
C10orf25__NM_001039380_AACGAACAACGCTTTCGGGT	C10orf25__NM_001039380	1130	864	1314	808
C10orf25__NM_001039380_AGGGGCGGTTACAGCTCTTG	C10orf25__NM_001039380	84	86	47	64
C10orf25__NM_001039380_AGGTTGGAGAGGCTAGAGTT	C10orf25__NM_001039380	92	80	98	95
C10orf25__NM_001039380_ATGCAGTTTCTAGGTTGGAG	C10orf25__NM_001039380	247	272	268	317
C10orf25__NM_001039380_CACAGGACGCCTTCCGAGTT	C10orf25__NM_001039380	1982	2372	2482	2335
C10orf25__NM_001039380_GGGTGGGCCCGGGACCATAG	C10orf25__NM_001039380	95	73	165	91
C10orf32__NM_001136200_AGGTCAGGCAGCTCGAAACA	C10orf32__NM_001136200	280	217	224	250
C10orf32__NM_001136200_CCAGAGTCTCAAGCGCGGTT	C10orf32__NM_001136200	616	572	643	594
C10orf32__NM_001136200_CGCGATTACGTCAGTACCAC	C10orf32__NM_001136200	447	695	652	955
C10orf32__NM_001136200_GAACGCCAGAGTCTCAAGCG	C10orf32__NM_001136200	465	447	639	495
C10orf32__NM_001136200_GAGCTGCCTGACCTAGCAGC	C10orf32__NM_001136200	105	298	128	106
C10orf32__NM_001136200_GCGGTTCGGTCAGTCCGTGA	C10orf32__NM_001136200	164	258	257	225
C10orf32__NM_001136200_GGTTCGGTCAGTCCGTGAAG	C10orf32__NM_001136200	203	133	211	212
C10orf32__NM_001136200_TGAACAGTCATCGGATCTAC	C10orf32__NM_001136200	316	193	301	319
C10orf35__NM_145306_AAGAGGTAGCATTCCTCGAC	C10orf35__NM_145306	410	482	455	546
C10orf35__NM_145306_AGCTTCTTCAACAGGGGCCA	C10orf35__NM_145306	93	175	119	125
C10orf35__NM_145306_AGGAGCAGGATGGAGGTCAC	C10orf35__NM_145306	415	276	522	344
C10orf35__NM_145306_CTCTTCCTGCTCATGATGCT	C10orf35__NM_145306	170	237	238	294
C10orf35__NM_145306_GGCCAATGGGGAAATCGTGC	C10orf35__NM_145306	470	551	550	754
C10orf35__NM_145306_GGTAGCATTCCTCGACAGGT	C10orf35__NM_145306	359	297	323	464
C10orf35__NM_145306_TGGTGCGGATCTTGGCCAAT	C10orf35__NM_145306	286	417	273	289
C10orf53__NM_001042427_GATGACCTCATGTCCATCTA	C10orf53__NM_001042427	614	882	761	882
C10orf53__NM_001042427_GCAGTGGTCATCCTGCGCTA	C10orf53__NM_001042427	714	710	661	861
C10orf54__NM_022153_ACGTGGTACCGCAGCTCGAG	C10orf54__NM_022153	626	539	666	594
C10orf54__NM_022153_CCTGCCTTTGCTTGTAGACC	C10orf54__NM_022153	1706	2135	2240	2267
C10orf54__NM_022153_CGCTGGGCCACATAGGACAG	C10orf54__NM_022153	309	682	388	515
C10orf54__NM_022153_GACATACAGGGAATACGGCG	C10orf54__NM_022153	410	366	456	444
C10orf54__NM_022153_GCTACGGGTGCCTGCATCGT	C10orf54__NM_022153	2041	1732	1953	1712
C10orf54__NM_022153_GGACAGACATACAGGGAATA	C10orf54__NM_022153	157	329	221	333
C10orf54__NM_022153_GGATGGTGCATCTTTGCCTG	C10orf54__NM_022153	1303	1645	1343	1541
C10orf54__NM_022153_GTAGAGGCCGCTATCCAGCA	C10orf54__NM_022153	280	236	326	392
C10orf54__NM_022153_TCCCTGTATGTCTGTCCCGA	C10orf54__NM_022153	774	465	904	831
C10orf55__NM_001001791_AACTAGATACGAACAGGGTG	C10orf55__NM_001001791	110	91	93	76
C10orf55__NM_001001791_AACTGTGACTGTCTAAATGG	C10orf55__NM_001001791	1074	743	1090	904
C10orf55__NM_001001791_AGCGGAGGCTTGAGAACCAC	C10orf55__NM_001001791	772	604	739	766
C10orf55__NM_001001791_CAGCGGAGGCTTGAGAACCA	C10orf55__NM_001001791	584	156	583	425
C10orf55__NM_001001791_GCGAACTGTGACTGTCTAAA	C10orf55__NM_001001791	891	1009	890	934
C10orf55__NM_001001791_GTTCGTATCTAGTTCTCAAA	C10orf55__NM_001001791	372	359	433	433
C10orf55__NM_001001791_TGCCCTGGAAGAGATTCGGG	C10orf55__NM_001001791	339	269	344	239
C10orf55__NM_001001791_TGTAGGGGTGGATACTCACA	C10orf55__NM_001001791	375	351	454	384
C10orf55__NM_001001791_TTGAGAACTAGATACGAACA	C10orf55__NM_001001791	454	354	530	760
C10orf55__NM_001001791_TTTAGACAGTCACAGTTCGC	C10orf55__NM_001001791	166	229	190	221
C10orf55__NM_001001791_TTTGAGAACTAGATACGAAC	C10orf55__NM_001001791	232	318	239	268
C10orf62__NM_001009997_AATCACACACACACCTTCTA	C10orf62__NM_001009997	578	624	679	782
C10orf62__NM_001009997_ACCAGCAGGCAGAAGACATC	C10orf62__NM_001009997	336	268	371	305
C10orf62__NM_001009997_ACCTGAGTGCTGGTAAGCTA	C10orf62__NM_001009997	487	521	528	671
C10orf62__NM_001009997_AGCGCTGGCATTGTTGTCGA	C10orf62__NM_001009997	1206	1340	1322	1962
C10orf62__NM_001009997_AGGAGATTGACACCAGGGG	C10orf62__NM_001009997	51	20	68	84
C10orf62__NM_001009997_AGTGATCCAAGAGATCCACC	C10orf62__NM_001009997	161	219	177	147
C10orf62__NM_001009997_CAAGAGATCCACCAGGAGTC	C10orf62__NM_001009997	627	834	840	798
C10orf62__NM_001009997_CAGATCTAGGTCTGCGATT	C10orf62__NM_001009997	297	337	378	354
C10orf62__NM_001009997_CAGCGGGAAAACACCATAGC	C10orf62__NM_001009997	89	90	90	123
C10orf62__NM_001009997_GGTAGCATTGCCACTAGCGC	C10orf62__NM_001009997	170	95	173	211
C10orf62__NM_001009997_TCTAGTGTCTGCGATTAGGC	C10orf62__NM_001009997	567	525	618	560
C10orf67__NM_153714_ATCTACGCAAAAGTTAGCAC	C10orf67__NM_153714	400	367	352	421
C10orf67__NM_153714_CATGAGCATAGTTATTAGAT	C10orf67__NM_153714	76	32	116	85
C10orf67__NM_153714_CGCGTAGGAAGCGAGAAGCT	C10orf67__NM_153714	607	634	837	685
C10orf67__NM_153714_CGTGGACCTGCGCATTTGTT	C10orf67__NM_153714	445	767	457	625
C10orf67__NM_153714_GGCCTTGGTCCGGGATCGCA	C10orf67__NM_153714	644	741	778	747
C10orf67__NM_153714_GTGGACCGCGCATTTGCGG	C10orf67__NM_153714	1	0	3	2
C10orf67__NM_153714_GTTTGAGGACCGACTGAAAG	C10orf67__NM_153714	164	149	176	233
C10orf67__NM_153714_TCAACTGAAGCAATTGTTTG	C10orf67__NM_153714	634	1066	612	975
C10orf67__NM_153714_TCATGAGCATAGTTATTAGA	C10orf67__NM_153714	115	22	108	89
C10orf67__NM_153714_TCGTGGACCGCGCATTTGC	C10orf67__NM_153714	359	430	534	610
C10orf67__NM_153714_TGTTATCAAAGGAATGTACC	C10orf67__NM_153714	165	157	156	94
C10orf67__NM_153714_TTCAAGCCCCCGCAAATGCG	C10orf67__NM_153714	15	103	13	31
C10orf71__NM_001135196_AAAAGTCCGAAACACCTGTC	C10orf71__NM_001135196	269	539	414	342
C10orf71__NM_001135196_AACACCTGTCGGGTGATATC	C10orf71__NM_001135196	399	361	368	382
C10orf71__NM_001135196_ACACCTGTCGGGTGATATCC	C10orf71__NM_001135196	254	344	279	319
C10orf71__NM_001135196_AGGGCACGGAACATTCGGGC	C10orf71__NM_001135196	1297	1178	1511	1408
C10orf71__NM_001135196_AGGTGAGCAGCCTAACAGAC	C10orf71__NM_001135196	232	302	333	350
C10orf71__NM_001135196_CACCTGTCGGGTGATATCCG	C10orf71__NM_001135196	540	552	583	706
C10orf71__NM_001135196_CCGTGCCCTGTGACGGTAAC	C10orf71__NM_001135196	674	1018	786	1061
C10orf71__NM_001135196_GCGTGTTGGATGAGCAGAC	C10orf71__NM_001135196	426	441	479	621
C10orf71__NM_001135196_GGGCACGGAACATTCGGGCT	C10orf71__NM_001135196	828	885	840	1030
C10orf71__NM_001135196_GTCCCGGATATCACCCGAC	C10orf71__NM_001135196	970	1262	1144	1291
C10orf71__NM_001135196_TCACAGGGCACGGAACATTC	C10orf71__NM_001135196	859	730	712	840
C10orf71__NM_001135196_TGGAGCAGTTACCGTCACA	C10orf71__NM_001135196	674	504	808	688
C10orf76__NM_024541_AAACCGAGGACTGCACTTAC	C10orf76__NM_024541	1451	1257	1664	1773
C10orf76__NM_024541_AAGAGCTCCTCCAAAACCG	C10orf76__NM_024541	442	785	380	192
C10orf76__NM_024541_ACCCATGCTCCCTACGACTT	C10orf76__NM_024541	1382	986	1284	1066
C10orf76__NM_024541_ACCCTGTGCGCACTCATTCG	C10orf76__NM_024541	3112	3158	3411	3593
C10orf76__NM_024541_CCATGCTCCCTACGACTTGG	C10orf76__NM_024541	1359	1298	1228	1364
C10orf76__NM_024541_CCCATGCTCCCTACGACTTG	C10orf76__NM_024541	1445	1828	1709	1795
C10orf76__NM_024541_CCCCCAAGTCGTAGGGAGCA	C10orf76__NM_024541	466	568	449	648
C10orf76__NM_024541_GGGAGAGGAGCATCCAATTC	C10orf76__NM_024541	836	870	883	783
C10orf76__NM_024541_GTGAATTTAGAGTACCTAGA	C10orf76__NM_024541	64	30	90	58
C10orf76__NM_024541_TCCTCAGATGAGTGCGCACA	C10orf76__NM_024541	317	197	273	194
C10orf76__NM_024541_TGGGAGAGGAGCATCCAATT	C10orf76__NM_024541	319	511	361	245
C10orf76__NM_024541_TTCCCATCCCCCAAGTCGTA	C10orf76__NM_024541	96	95	75	92
C10orf82__NM_144661_ACCGTCTCCTCGGAGTTGAC	C10orf82__NM_144661	534	595	668	719
C10orf82__NM_144661_ACTGAAACCTGTCAACTCCG	C10orf82__NM_144661	586	517	640	611
C10orf82__NM_144661_ACTTCCTGGAGATCACGGAG	C10orf82__NM_144661	276	291	356	350
C10orf82__NM_144661_AGGAGCCCCCGATCCCTGGC	C10orf82__NM_144661	1217	1257	1278	1494
C10orf82__NM_144661_GATTGGCAGGTTTCTCATGA	C10orf82__NM_144661	835	981	986	948
C10orf82__NM_144661_GCCTGCCCAGCCAGGGATCG	C10orf82__NM_144661	464	550	489	693
C10orf82__NM_144661_GTTGACAGGTTTCAGTTTCG	C10orf82__NM_144661	694	874	795	802
C10orf88__NM_024942_ATCAAGGATAGACCTTGACA	C10orf88__NM_024942	417	309	460	361
C10orf88__NM_024942_CCATGGACTCCATTATGGTT	C10orf88__NM_024942	1655	1272	1790	1421
C10orf88__NM_024942_CGACTTGTTTAAGGTTCCTA	C10orf88__NM_024942	667	729	783	832
C10orf88__NM_024942_GAGACGCGGACCGAGGACGG	C10orf88__NM_024942	295	373	355	269
C10orf88__NM_024942_GTTTAGAATTGTATTCCCAT	C10orf88__NM_024942	143	224	214	286
C10orf88__NM_024942_TATGGAAGTGTACTTAGGAG	C10orf88__NM_024942	304	356	302	257
C10orf88__NM_024942_TCCTCCCAGTCGAAGTCACC	C10orf88__NM_024942	249	192	287	248
C10orf88__NM_024942_TCGGCCCCGGTGACTTCGAC	C10orf88__NM_024942	865	928	1015	1312
C10orf88__NM_024942_TCTGGATACAAGCATATGAT	C10orf88__NM_024942	299	267	342	457
C10orf88__NM_024942_TGACAAGGTCCAAACCATAA	C10orf88__NM_024942	241	170	286	249
C10orf88__NM_024942_TGACCCCATGGACTCCATTA	C10orf88__NM_024942	798	579	853	543
C10orf88__NM_024942_TGTGTTCATCAGTAAAGTTG	C10orf88__NM_024942	50	24	80	95
C10orf90__NM_001004298_ACCATCACGGCCAGACGCGT	C10orf90__NM_001004298	1538	1377	1687	1680

C10orf90__NM_001004298_ACTGTCAGGAAAGAGCCCGC	C10orf90__NM_001004298	551	452	621	793
C10orf90__NM_001004298_CAATCTCTGGCTAACCGCAG	C10orf90__NM_001004298	24	35	26	46
C10orf90__NM_001004298_CCTCGCTCATCTGACGGTGC	C10orf90__NM_001004298	1098	1171	870	1051
C10orf90__NM_001004298_CGAGACTGAGCAATGGCACA	C10orf90__NM_001004298	400	559	421	424
C10orf90__NM_001004298_CTGAGCAATGGCACACGGTA	C10orf90__NM_001004298	415	717	536	791
C10orf90__NM_001004298_GTGTGAGAGACGCAAGTACT	C10orf90__NM_001004298	329	282	410	416
C10orf90__NM_001004298_TCCGGGCAGATTAGGAATCC	C10orf90__NM_001004298	225	381	317	302
C10orf90__NM_001004298_TCTACGTAATAGATTGAATC	C10orf90__NM_001004298	170	62	137	156
C10orf90__NM_001004298_TCTCCAGACACCCCACTGTC	C10orf90__NM_001004298	1224	888	1345	1351
C10orf90__NM_001004298_TGAGCAATGGCACACGGTAG	C10orf90__NM_001004298	352	389	418	433
C10orf90__NM_001004298_TGATGGATGCAAACCCTCTG	C10orf90__NM_001004298	1019	1168	1091	1539
C10orf91__NM_173541_CGTGGTAGATCAAGCCTCAC	C10orf91__NM_173541	852	1103	845	931
C10orf91__NM_173541_GAACAGGCGTCTGTTAGTCA	C10orf91__NM_173541	244	204	369	447
C10orf91__NM_173541_GCTCCCAGGGAGGACACGCC	C10orf91__NM_173541	508	784	687	944
C10orf91__NM_173541_GGAACAGGCGTCTGTTAGTC	C10orf91__NM_173541	1425	1751	1565	1922
C10orf91__NM_173541_TAGTCAGGGTTTCAGGAATG	C10orf91__NM_173541	466	501	467	382
C10orf95__NM_024886_AGAAGCGGTGGTATTCCCGT	C10orf95__NM_024886	390	448	356	207
C10orf95__NM_024886_AGGCGGCTGCTGTTCTTACT	C10orf95__NM_024886	1805	1961	2187	2088
C10orf95__NM_024886_CAGCTGTACACATACATGGT	C10orf95__NM_024886	505	684	610	547
C10orf95__NM_024886_CGACCATGTATGTGTACAGC	C10orf95__NM_024886	468	562	565	728
C10orf95__NM_024886_CGCAGCTACAAAGTGCGGAG	C10orf95__NM_024886	278	203	404	408
C10orf95__NM_024886_CGCCGCCCAAACAGGGCGTC	C10orf95__NM_024886	10	25	9	37
C10orf95__NM_024886_CGGCGGCCAGACGCCCTGTT	C10orf95__NM_024886	433	504	504	557
C10orf95__NM_024886_GAAGCGGTGGTATTCCCGTG	C10orf95__NM_024886	214	203	309	260
C10orf95__NM_024886_GAGCCTGCATGCGGGCGAGT	C10orf95__NM_024886	302	341	279	243
C10orf95__NM_024886_GCGGCTGCTGTTCTTACTGG	C10orf95__NM_024886	1356	1284	1538	1847
C10orf95__NM_024886_GGCGGCTGCTGTTCTTACTG	C10orf95__NM_024886	1086	1384	1244	1356
C10orf95__NM_024886_GGGTCGTGGCGTAGGCCGGA	C10orf95__NM_024886	297	238	399	459
C10orf99__NM_207373_ACCTTTCAGGTTTGTTGAGT	C10orf99__NM_207373	778	1027	863	1251
C10orf99__NM_207373_CTTTCAGGTTTGTTGAGTTG	C10orf99__NM_207373	467	763	617	711
C11orf1__NM_022761_AATCTGTCCACACTTCACCA	C11orf1__NM_022761	2448	2817	2684	3043
C11orf1__NM_022761_CATCAGGGTACGGTTTGAAT	C11orf1__NM_022761	495	305	499	490
C11orf1__NM_022761_CCTGGTTCCAGTTGCCCATC	C11orf1__NM_022761	916	957	863	973
C11orf1__NM_022761_CTATTCAAACCGTACCCTGA	C11orf1__NM_022761	400	450	468	451
C11orf1__NM_022761_CTGGTTCCAGTTGCCCATCA	C11orf1__NM_022761	216	378	265	362
C11orf1__NM_022761_GCTATTCAAAGCCTTAAATT	C11orf1__NM_022761	120	177	142	162
C11orf1__NM_022761_TATTCAAACCGTACCCTGAT	C11orf1__NM_022761	658	571	938	746
C11orf1__NM_022761_TCTGTGCATTTGTATCGGGG	C11orf1__NM_022761	855	598	966	683
C11orf1__NM_022761_TGAATAGGTATTCTCATTAG	C11orf1__NM_022761	346	331	374	281
C11orf1__NM_022761_TGATGTCCTGGGAACCAGTG	C11orf1__NM_022761	590	541	697	706
C11orf1__NM_022761_TTTCAAAGTAGTGTCCAAAC	C11orf1__NM_022761	108	133	152	106
C11orf16__NM_020643_AAGGCCATGGAATCCTCCAC	C11orf16__NM_020643	708	973	742	722
C11orf16__NM_020643_CACATGGGTCCTAGCAAGAA	C11orf16__NM_020643	185	281	253	173
C11orf16__NM_020643_CAGCGTGGCCACAAGCCTGA	C11orf16__NM_020643	188	240	221	258
C11orf16__NM_020643_CTCGGGAGTGGCCTTTATTT	C11orf16__NM_020643	1263	1226	1327	1519
C11orf16__NM_020643_GAGACAGGGGGTCCTGCTTG	C11orf16__NM_020643	1146	1111	1272	1027
C11orf16__NM_020643_GAGTGGCCTTTATTTGGGCC	C11orf16__NM_020643	265	441	329	325
C11orf16__NM_020643_GCAGAGAGTGGTCTTAGAAG	C11orf16__NM_020643	531	657	604	557
C11orf16__NM_020643_GGGTCGGCAACGTGGAGACA	C11orf16__NM_020643	311	545	317	331
C11orf16__NM_020643_GTGAAGGAGAGGTCCCAAGG	C11orf16__NM_020643	230	73	318	228
C11orf16__NM_020643_TCCACGTTGCCGACCCAGCA	C11orf16__NM_020643	290	565	330	477
C11orf16__NM_020643_TTACTACCGGGCCCAAATAA	C11orf16__NM_020643	213	209	217	318
C11orf16__NM_020643_TTCGAGGCTCCTCTTGTCGC	C11orf16__NM_020643	157	218	125	160
C11orf21__NM_001142946_ATCCACAGGTTCATGAGCGG	C11orf21__NM_001142946	1351	1504	1534	1384
C11orf21__NM_001142946_CAGTGGCCACACCAAGAACG	C11orf21__NM_001142946	193	179	215	225
C11orf21__NM_001142946_CATCCCAGCCGGATAGCCAG	C11orf21__NM_001142946	368	247	343	351
C11orf21__NM_001142946_CCACTGAGGACGGCGCTGAG	C11orf21__NM_001142946	764	876	1015	1083
C11orf21__NM_001142946_CCTCACTTGTCATCTCAAAG	C11orf21__NM_001142946	759	768	862	1071
C11orf21__NM_001142946_GGCAGTTGCCCCTGGCTATC	C11orf21__NM_001142946	58	48	56	43
C11orf21__NM_001142946_GGGGTTCCCGTCCACCTGTC	C11orf21__NM_001142946	92	139	201	226
C11orf21__NM_001142946_GTGGCGTCGAACCCCCTGAC	C11orf21__NM_001142946	754	664	688	812
C11orf21__NM_001142946_GTTGCCCCTGGCTATCCGGC	C11orf21__NM_001142946	482	427	652	927
C11orf21__NM_001142946_GTTGGAGAGAGACAATTCTA	C11orf21__NM_001142946	164	170	313	218
C11orf21__NM_001142946_TGCTGCTGTCTCAGTTTACC	C11orf21__NM_001142946	732	1053	666	861
C11orf21__NM_001142946_TTGCCCCTGGCTATCCGGCT	C11orf21__NM_001142946	658	577	681	936
C11orf24__NM_022338_AATGGAGGCCACAGTTGTAC	C11orf24__NM_022338	1053	1349	1075	1455
C11orf24__NM_022338_ACCCCGTCCACTACCGCCAC	C11orf24__NM_022338	2651	2917	3008	3147
C11orf24__NM_022338_ACTGGAGGCCACAGTCATAC	C11orf24__NM_022338	591	1035	636	889
C11orf24__NM_022338_ACTGGAGGCCGCAGTCGTAA	C11orf24__NM_022338	796	1015	968	1072
C11orf24__NM_022338_CTCTATGGAAGTCACAACAG	C11orf24__NM_022338	65	40	76	10
C11orf24__NM_022338_GCGGGGAGTGCAAGTGTCAT	C11orf24__NM_022338	1038	1032	1139	1227
C11orf24__NM_022338_GGAGGCCGCAGTCGTACTGG	C11orf24__NM_022338	1740	2644	1834	2241
C11orf24__NM_022338_GGCAGCCCACCTCAACTCTA	C11orf24__NM_022338	834	1179	805	950
C11orf24__NM_022338_GTCCTTATCTGAAAGCCATG	C11orf24__NM_022338	97	51	128	224
C11orf24__NM_022338_TGCAGCCTCCAGTACAACTG	C11orf24__NM_022338	247	419	355	535
C11orf24__NM_022338_TGTGGAAGGGATTAGTCAAG	C11orf24__NM_022338	334	306	422	411
C11orf24__NM_022338_TGTGGCCTCCAGTACGACTG	C11orf24__NM_022338	275	242	256	296
C11orf30__NM_020193_AGCATTAGCTGTTACAGTAA	C11orf30__NM_020193	43	35	106	35
C11orf30__NM_020193_ATTAGCTGTTACAGTAAAGG	C11orf30__NM_020193	86	25	98	73
C11orf30__NM_020193_GGAGAGCAGTAAACGATGAA	C11orf30__NM_020193	216	91	289	143
C11orf30__NM_020193_GGTTGACGTGGTACTTGTGT	C11orf30__NM_020193	488	442	619	451
C11orf30__NM_020193_GTCCATTGAAGGTCGTCGAT	C11orf30__NM_020193	502	330	491	298
C11orf30__NM_020193_GTTACAGTAAAGGCGGTTTG	C11orf30__NM_020193	248	153	198	265
C11orf30__NM_020193_TACCAATCGACGACCTTCAA	C11orf30__NM_020193	532	229	667	357
C11orf30__NM_020193_TCAGTGCACTTCGGGCACAG	C11orf30__NM_020193	141	35	137	79
C11orf30__NM_020193_TCCAAGTGGCAGCATAGCAA	C11orf30__NM_020193	0	0	0	0
C11orf30__NM_020193_TCTTCAGAATGGTCCATTGA	C11orf30__NM_020193	540	303	529	226
C11orf30__NM_020193_TGTTACAGTAAAGGCGGTTT	C11orf30__NM_020193	212	178	243	112
C11orf30__NM_020193_TTAGAATTGGAGGCATATGC	C11orf30__NM_020193	10	0	25	16
C11orf31__NM_170746_AGACGCGTCAGCTAGTGCTA	C11orf31__NM_170746	334	271	359	435
C11orf31__NM_170746_AGAGAAGCTGGCGAACGGCG	C11orf31__NM_170746	582	690	504	688
C11orf31__NM_170746_AGCACTAGCTGACGCGTCTA	C11orf31__NM_170746	156	305	270	338
C11orf31__NM_170746_AGGGAATTTGAGTTTGCGTG	C11orf31__NM_170746	260	337	282	384
C11orf31__NM_170746_CAGGGAATTTGAGTTTGCGT	C11orf31__NM_170746	1495	1405	1831	1557
C11orf31__NM_170746_CGACCGTTGTTATCGAGCAT	C11orf31__NM_170746	1049	1116	1111	1318
C11orf31__NM_170746_CGGGAGGAAGCGTAAGGCTG	C11orf31__NM_170746	427	279	464	332
C11orf31__NM_170746_CTCTGGACTGGGATTAAGAA	C11orf31__NM_170746	17	16	28	83
C11orf31__NM_170746_GCACTAGCTGACGCGTCTAT	C11orf31__NM_170746	827	800	768	665
C11orf31__NM_170746_TAGACGCGTCAGCTAGTGCT	C11orf31__NM_170746	284	291	293	338
C11orf31__NM_170746_TCACCAATGCTCGATAACAA	C11orf31__NM_170746	265	80	194	165
C11orf31__NM_170746_TCAGGGAATTTGAGTTTGCG	C11orf31__NM_170746	427	483	584	726
C11orf40__NM_144663_ACACTAGACGGCACTGTTAG	C11orf40__NM_144663	1254	1548	1474	1814
C11orf40__NM_144663_ACGCTTTCTTTCTGGTTAGC	C11orf40__NM_144663	336	418	360	455
C11orf40__NM_144663_AGGCTCTGTAGAAGTTTATA	C11orf40__NM_144663	57	81	54	18
C11orf40__NM_144663_CACTAGACGGCACTGTTAGT	C11orf40__NM_144663	613	432	593	519
C11orf40__NM_144663_CAGAGCCTTTAACTTAGATA	C11orf40__NM_144663	95	94	183	168
C11orf40__NM_144663_CCAGCTTCTATGTGACTTAA	C11orf40__NM_144663	205	423	249	370
C11orf40__NM_144663_TAAGGATGTGTTCATACACC	C11orf40__NM_144663	133	235	207	189
C11orf40__NM_144663_TAGACGGCACTGTTAGTGGG	C11orf40__NM_144663	695	688	879	867
C11orf40__NM_144663_TATCTGGAACGTGTTTCCAG	C11orf40__NM_144663	274	319	342	331
C11orf40__NM_144663_TCACCCATCACCACACTAGA	C11orf40__NM_144663	739	996	1023	1002
C11orf40__NM_144663_TGACAAAAGACCTTGCTTTA	C11orf40__NM_144663	237	366	419	429
C11orf40__NM_144663_TGGTGTTTCATTGCTCCCAC	C11orf40__NM_144663	0	0	0	0
C11orf42__NM_173525_AAAGCCAGGCGGGTATGGGC	C11orf42__NM_173525	286	291	391	277
C11orf42__NM_173525_ACTCGGGAATACTCACCAAA	C11orf42__NM_173525	388	228	336	383
C11orf42__NM_173525_CACACCCAGTAGGTCATAGC	C11orf42__NM_173525	15	0	10	9
C11orf42__NM_173525_CTATGAAGAGACGCGAATGT	C11orf42__NM_173525	526	442	527	602
C11orf42__NM_173525_CTGCAAGATCCGCCTACATA	C11orf42__NM_173525	688	441	655	788
C11orf42__NM_173525_CTGGCTTTGCCAGGTCGGCA	C11orf42__NM_173525	25	32	44	78
C11orf42__NM_173525_GAAAGGTACTGCTACTGCAT	C11orf42__NM_173525	429	511	512	584
C11orf42__NM_173525_GAAGAGACGCGAATGTTGGA	C11orf42__NM_173525	658	685	785	733
C11orf42__NM_173525_GCAGCCTGCTATGACCTACT	C11orf42__NM_173525	947	963	1061	1385
C11orf42__NM_173525_TGAGTATTCCCGAGTGCAGT	C11orf42__NM_173525	434	383	466	595
C11orf42__NM_173525_TGCAGCCTGCTATGACCTAC	C11orf42__NM_173525	525	404	549	606
C11orf42__NM_173525_TGCTCCAGGAGGCTTGGTAG	C11orf42__NM_173525	1177	1245	1466	1224
C11orf44__NM_001271983_ACCTGAGCTCACAGGACGGC	C11orf44__NM_001271983	793	934	1008	1301
C11orf45__NM_001256088_AAGGCCCATGATAGTAGAGA	C11orf45__NM_001256088	134	151	200	271
C11orf45__NM_001256088_AGCTGAGAAATTCCAGAGTG	C11orf45__NM_001256088	512	320	742	619
C11orf45__NM_001256088_CAAGGCCCATGATAGTAGAG	C11orf45__NM_001256088	297	453	477	393
C11orf45__NM_001256088_GGCGGCCCTCTCTACTATCA	C11orf45__NM_001256088	1424	1809	1699	1685
C11orf45__NM_001256088_TCACTCTGGAATTTCTCAGC	C11orf45__NM_001256088	308	569	436	557
C11orf49__NM_001003676_ACTCGTAGTCCGGTAGGGCT	C11orf49__NM_001003676	309	329	357	339
C11orf49__NM_001003676_AGAACCTGGCAATTCCATAT	C11orf49__NM_001003676	608	825	719	720
C11orf49__NM_001003676_AGGGAAGATATTAGCCAATA	C11orf49__NM_001003676	281	601	175	175
C11orf49__NM_001003676_CAGATACTCGTAGTCCGGTA	C11orf49__NM_001003676	406	457	395	396
C11orf49__NM_001003676_CCAGATACTCGTAGTCCGGT	C11orf49__NM_001003676	1279	1603	1479	1587
C11orf49__NM_001003676_CCTACCGGACTACGAGTATC	C11orf49__NM_001003676	240	421	389	430
C11orf49__NM_001003676_CTGGAGATGCTTCCGAACTG	C11orf49__NM_001003676	1509	1577	1610	1563
C11orf49__NM_001003676_GTAAAAATGATACCCTATTG	C11orf49__NM_001003676	190	178	328	412
C11orf49__NM_001003676_TCCGGAGCGCCTAGCCCTAC	C11orf49__NM_001003676	982	1021	1142	1085
C11orf49__NM_001003676_TGGAGATGCTTCCGAACTGT	C11orf49__NM_001003676	283	384	376	334
C11orf49__NM_001003676_TGTGCTCATGGACGATGCCA	C11orf49__NM_001003676	130	193	166	190
C11orf49__NM_001003676_TTAGCCAATATGGAATTGCC	C11orf49__NM_001003676	172	187	187	226
C11orf49__NM_001278222_CGTCGGCACAATCACTGTGT	C11orf49__NM_001278222	1534	1484	1603	1497
C11orf49__NM_001278222_GACTCAGCATCCTTTGACCG	C11orf49__NM_001278222	1395	1631	1681	1403
C11orf49__NM_001278222_TCAGCATCCTTTGACCGGGG	C11orf49__NM_001278222	149	91	189	171
C11orf52__NM_080659_AACAGGACATACGTATGAAC	C11orf52__NM_080659	303	135	401	296
C11orf52__NM_080659_CAACAGGACATACGTATGAA	C11orf52__NM_080659	1051	1080	1209	1290
C11orf52__NM_080659_GCTTCAGTGTCCGTCTTGTT	C11orf52__NM_080659	2105	2769	2439	3054
C11orf53__NM_198498_AATGACCTACCGCCCAAGGT	C11orf53__NM_198498	121	161	191	157
C11orf53__NM_198498_ACAGGTTCGCCGGTCACGTC	C11orf53__NM_198498	429	470	516	643
C11orf53__NM_198498_AGGAGCCCTACGGAGACTAC	C11orf53__NM_198498	328	203	245	308
C11orf53__NM_198498_ATTTGACACCGTCATGAAAG	C11orf53__NM_198498	64	89	69	103
C11orf53__NM_198498_CATTTGACACCGTCATGAAA	C11orf53__NM_198498	348	438	348	355
C11orf53__NM_198498_GAATGCCGCAGAGCTTATTG	C11orf53__NM_198498	430	437	566	504
C11orf53__NM_198498_GGAGGCGGGGTAGGGTACCC	C11orf53__NM_198498	92	44	175	171
C11orf53__NM_198498_GTCATACTCGCTGCATGCTC	C11orf53__NM_198498	0	0	0	0
C11orf53__NM_198498_GTGGCCCCACCTTGGGCGGT	C11orf53__NM_198498	58	30	23	25
C11orf53__NM_198498_TGGGAGAGGCAACTCGTGCT	C11orf53__NM_198498	326	266	403	430
C11orf53__NM_198498_TTCCCTGTGAGTCCTCCGCA	C11orf53__NM_198498	69	10	82	102
C11orf53__NM_198498_TTGAAAACTGTTTACTGTTG	C11orf53__NM_198498	323	453	427	471
C11orf54__NM_001286067_AGGGTTCCTTAGTCAAATCA	C11orf54__NM_001286067	156	151	211	179
C11orf54__NM_001286067_ATACAAGTCACAAAGTTAAG	C11orf54__NM_001286067	111	61	154	139
C11orf54__NM_001286067_CATTATGGAAATAAGCCTAT	C11orf54__NM_001286067	463	378	485	499
C11orf54__NM_001286067_CCCTTTACCTTTCCTGTAAA	C11orf54__NM_001286067	397	574	508	493
C11orf54__NM_001286067_GCAAAAGAAATCAAGCTGCC	C11orf54__NM_001286067	644	694	824	1055
C11orf54__NM_001286067_GCAAAGTAACTTCCATTTAC	C11orf54__NM_001286067	718	681	825	983
C11orf54__NM_001286067_GGAAATAAGCCTATAGGAAT	C11orf54__NM_001286067	151	165	140	143
C11orf54__NM_001286067_GGTACTTTCATAATTCAGAA	C11orf54__NM_001286067	31	79	70	106
C11orf54__NM_001286067_TGATTGCCCTGATTTGACTA	C11orf54__NM_001286067	232	142	254	408
C11orf54__NM_001286067_TGCAGATGGAGGGTGCCTAC	C11orf54__NM_001286067	1126	924	1271	1480
C11orf54__NM_001286067_TGCTCCTGCTCCAAGAATAA	C11orf54__NM_001286067	111	79	112	116
C11orf54__NM_001286067_TGGAAATAAGCCTATAGGAA	C11orf54__NM_001286067	0	0	5	28
C11orf54__NM_001286071_AAAAGTGAAGTCTCACATTA	C11orf54__NM_001286071	264	389	370	384
C11orf54__NM_001286071_CTGATGAAGAAGTGAATAAA	C11orf54__NM_001286071	224	133	144	135
C11orf54__NM_014039_CCAAGATATTCCACTATATC	C11orf54__NM_014039	507	442	633	777
C11orf54__NM_014039_CCAGATATAGTGGAATATCT	C11orf54__NM_014039	348	393	498	481
C11orf54__NM_014039_TATCTGGAGTAGTGTCATAA	C11orf54__NM_014039	360	312	446	556
C11orf54__NM_014039_TGACACTACTCCAGATATAG	C11orf54__NM_014039	217	136	193	153
C11orf57__NM_001082969_AAAGTCAAAGATACTATTGG	C11orf57__NM_001082969	317	162	367	284
C11orf57__NM_001082969_AGAATGTCCCGGATTCCACT	C11orf57__NM_001082969	882	417	899	538
C11orf57__NM_001082969_AGATGGAAGATGCTTACCGG	C11orf57__NM_001082969	688	147	709	262
C11orf57__NM_001082969_ATCAGCAAGATATTACCAAC	C11orf57__NM_001082969	230	118	295	129
C11orf57__NM_001082969_ATGGGTAGATGACTTTACCT	C11orf57__NM_001082969	444	260	420	170
C11orf57__NM_001082969_CAAAACCATCACTGCGCATC	C11orf57__NM_001082969	679	764	789	848
C11orf57__NM_001082969_CACAGATGCTCACAATAAGG	C11orf57__NM_001082969	297	30	330	21
C11orf57__NM_001082969_CAGATGGAAGATGCTTACCG	C11orf57__NM_001082969	503	230	467	140
C11orf57__NM_001082969_TCTTGTGTTTGCGGGATTCA	C11orf57__NM_001082969	309	356	366	376
C11orf57__NM_001082969_TGGGTAGATGACTTTACCTG	C11orf57__NM_001082969	117	72	100	100
C11orf57__NM_001082969_TTCAGGAGGAATCAGATATG	C11orf57__NM_001082969	83	2	113	2
C11orf57__NM_001082969_TTTAGCCGGATGCGCAGTGA	C11orf57__NM_001082969	551	615	617	704
C11orf58__NM_014267_CAGAGAGTCTCACCCGCATG	C11orf58__NM_014267	1417	1260	1705	1471
C11orf58__NM_014267_CATACTGGTCGTCTTGTTAT	C11orf58__NM_014267	1010	866	1048	922
C11orf58__NM_014267_CCAGAGAGTCTCACCCGCAT	C11orf58__NM_014267	2080	2104	2390	2035
C11orf58__NM_014267_CTAGCTGGGATCTAGCAATT	C11orf58__NM_014267	292	506	373	429
C11orf58__NM_014267_GGAAGATATCGGCGACATTG	C11orf58__NM_014267	420	627	505	393
C11orf58__NM_014267_TCAACATCTCACTTCCGAAC	C11orf58__NM_014267	667	433	719	929
C11orf58__NM_014267_TCTAGCTGGGATCTAGCAAT	C11orf58__NM_014267	196	187	267	173
C11orf58__NM_014267_TTCTTGAGACTTATGGGTGC	C11orf58__NM_014267	0	0	0	0
C11orf63__NM_024806_CGAGCAAAACCACCATACCT	C11orf63__NM_024806	1416	1229	1609	1256
C11orf63__NM_024806_CGGGCTCCATACCGTTGCCC	C11orf63__NM_024806	92	72	161	178
C11orf63__NM_024806_CTTACCTGTTATTGGCGCCC	C11orf63__NM_024806	750	501	900	813
C11orf63__NM_024806_GCCCGACAGCTTAGACGAGG	C11orf63__NM_024806	414	431	523	481
C11orf63__NM_024806_GGACATTTAAGTTGGTATGA	C11orf63__NM_024806	328	402	413	488
C11orf63__NM_024806_GGAGATTGAATAGAGAGCTT	C11orf63__NM_024806	122	192	130	168
C11orf63__NM_024806_GGGACCAGGGCGCCAATAAC	C11orf63__NM_024806	1189	1216	1298	1452
C11orf63__NM_024806_TCCTCCTCGTCTAAGCTGTC	C11orf63__NM_024806	272	496	309	412
C11orf63__NM_024806_TCGAATCCGGGGCAACGGTA	C11orf63__NM_024806	176	349	194	211
C11orf63__NM_024806_TGAGTTTGATGATCGAATCC	C11orf63__NM_024806	223	269	243	226
C11orf63__NM_024806_TTCCTCCTCGTCTAAGCTGT	C11orf63__NM_024806	12	10	33	25
C11orf63__NM_024806_TTTAAGTTGGTATGAAGGAC	C11orf63__NM_024806	107	219	78	107
C11orf65__NM_152587_AACTCTTCGGCTAACTTCAA	C11orf65__NM_152587	245	208	266	267
C11orf65__NM_152587_ATATATTTCACTATCTGACG	C11orf65__NM_152587	79	91	85	196
C11orf65__NM_152587_ATCGTATAGAAAACAATGGC	C11orf65__NM_152587	460	421	533	561
C11orf65__NM_152587_ATTCATGTGCGATTCAGATT	C11orf65__NM_152587	422	635	389	324
C11orf65__NM_152587_CATGTGCGATTCAGATTAGG	C11orf65__NM_152587	763	712	769	711
C11orf65__NM_152587_CTGATTGATTTAAGAAGACA	C11orf65__NM_152587	35	30	62	60
C11orf65__NM_152587_GGAATCAGAATTTACAAAGC	C11orf65__NM_152587	166	244	209	200
C11orf65__NM_152587_TTAACGCCTGATTCTACTTA	C11orf65__NM_152587	130	175	141	172
C11orf65__NM_152587_TTATGAGATGTATGCTTTGC	C11orf65__NM_152587	173	84	178	249
C11orf68__NM_001135635_AGCGGGTAACTTGGGATGGT	C11orf68__NM_001135635	1151	1196	1356	1720
C11orf68__NM_001135635_ATCCCAAGTTACCCGCTATG	C11orf68__NM_001135635	890	1015	1142	904
C11orf68__NM_001135635_CAGCCCACAGGCTCTGAGTT	C11orf68__NM_001135635	390	406	447	468
C11orf68__NM_001135635_CATCCCAAGTTACCCGCTAT	C11orf68__NM_001135635	2228	1679	2705	2244
C11orf68__NM_001135635_CCATCCCAAGTTACCCGCTA	C11orf68__NM_001135635	1068	1227	1423	1608
C11orf68__NM_001135635_CGGGCATCAAACACTAGCCA	C11orf68__NM_001135635	1395	1340	1539	1895
C11orf68__NM_001135635_GCCTTCGCTGCGTTCGACGC	C11orf68__NM_001135635	822	835	1006	1445
C11orf68__NM_001135635_GCGGGCATCAAACACTAGCC	C11orf68__NM_001135635	431	677	631	785
C11orf68__NM_001135635_GCTCTGAGTTGGGTGAACCG	C11orf68__NM_001135635	290	358	378	382
C11orf68__NM_001135635_GTCCCCATAGCGGGTAACTT	C11orf68__NM_001135635	154	189	127	132
C11orf68__NM_001135635_GTGGGCTGGATTGCAGTGTA	C11orf68__NM_001135635	92	142	111	204
C11orf68__NM_001135635_TCGAACGCAGCGAAGGCCGG	C11orf68__NM_001135635	523	564	706	612
C11orf70__NM_001195005_AACGAAATCATCCTTCCGAT	C11orf70__NM_001195005	1172	828	1221	1103
C11orf70__NM_001195005_ATCCTTCCGATAGGACTGAA	C11orf70__NM_001195005	151	140	180	267
C11orf70__NM_001195005_CTTCCAGTCTCTGAGCTCTA	C11orf70__NM_001195005	225	212	317	333
C11orf70__NM_001195005_GACCTTTCAGTCCTATCGGA	C11orf70__NM_001195005	540	285	539	425
C11orf70__NM_001195005_GCTGGGCAGAATCAAGGCGC	C11orf70__NM_001195005	493	291	579	722
C11orf70__NM_032930_ACATGAAGGTGACGCCTGAT	C11orf70__NM_032930	854	923	996	1049
C11orf70__NM_032930_ACTTTATTGTGGATCCTATC	C11orf70__NM_032930	259	276	273	280
C11orf70__NM_032930_CACATCCTCATATTGACAAA	C11orf70__NM_032930	575	850	542	715
C11orf70__NM_032930_GAAGATATTGTACGAGACAG	C11orf70__NM_032930	142	54	240	203
C11orf70__NM_032930_GGAACTCTTCTCTATCTGGT	C11orf70__NM_032930	709	861	871	907
C11orf70__NM_032930_TTATACCACTGTTATGGTGT	C11orf70__NM_032930	201	189	176	179
C11orf70__NM_032930_TTTATACCACTGTTATGGTG	C11orf70__NM_032930	25	41	37	65
C11orf71__NM_001271562_AGGACAGGGACACATTGTTC	C11orf71__NM_001271562	462	513	393	523
C11orf71__NM_001271562_AGGCCTGAAGCGATTCATCT	C11orf71__NM_001271562	446	304	552	727
C11orf71__NM_001271562_CAGCGTTTGATTGCATTAGG	C11orf71__NM_001271562	391	502	523	549
C11orf71__NM_001271562_CCCATGGCGACCTCAGACCG	C11orf71__NM_001271562	2645	2701	3021	3365
C11orf71__NM_001271562_CCTGCTCCTCTGATCACCGG	C11orf71__NM_001271562	1579	1414	1631	1466
C11orf71__NM_001271562_GCCGAGAGCCGTCGAGTGGA	C11orf71__NM_001271562	1274	1457	1319	1594
C11orf71__NM_001271562_GGAGATCGGGTTCAACGGCA	C11orf71__NM_001271562	858	795	890	995
C11orf71__NM_001271562_GGCCGCCACCATCCACTCGA	C11orf71__NM_001271562	3599	4529	3930	5003
C11orf71__NM_001271562_TCGGGCCGAGAGCCGTCGAG	C11orf71__NM_001271562	380	247	396	364
C11orf73__NM_016401_AATGTTCATTCCGGCAAATG	C11orf73__NM_016401	129	42	131	65
C11orf73__NM_016401_ACTCCATCTGTTGCTCAGAT	C11orf73__NM_016401	292	447	362	357
C11orf73__NM_016401_AGTGGAATTATTAGACAGTA	C11orf73__NM_016401	403	380	399	297
C11orf73__NM_016401_ATGGCTCAGCAGACTCCTGT	C11orf73__NM_016401	212	84	241	145
C11orf73__NM_016401_ATTCAAATGGAATGCCAGTA	C11orf73__NM_016401	226	65	261	254
C11orf73__NM_016401_CATTTCAGAACCACATTTGC	C11orf73__NM_016401	697	423	1029	610
C11orf73__NM_016401_CCAGCATAAAAACCACAACA	C11orf73__NM_016401	214	131	266	235
C11orf73__NM_016401_CCGGAATGAACATTTCAGAT	C11orf73__NM_016401	688	263	873	493
C11orf73__NM_016401_CTCAGTTGACTCATTCACTC	C11orf73__NM_016401	433	439	479	561
C11orf73__NM_016401_CTCCCATTCCCTCAGGAAAT	C11orf73__NM_016401	334	166	460	249
C11orf73__NM_016401_TCATGGCTCCAAAAGGATGT	C11orf73__NM_016401	155	6	146	31
C11orf74__NM_001276722_ATTAAACCCCAAATGAGTGA	C11orf74__NM_001276722	199	262	210	264
C11orf74__NM_001276722_CTGGGCCACAAAAGGGATAC	C11orf74__NM_001276722	170	112	131	50
C11orf74__NM_001276722_GAGGATCATGTGTCCAAAAG	C11orf74__NM_001276722	119	133	113	131
C11orf74__NM_001276722_GGAATGTCTGCCCATATGTC	C11orf74__NM_001276722	240	535	243	320
C11orf74__NM_001276722_GGCTTCACTTCACAGGTAGG	C11orf74__NM_001276722	671	791	782	871
C11orf74__NM_001276722_TCAATTAATCAAAGACGTCT	C11orf74__NM_001276722	249	367	302	421
C11orf74__NM_001276722_TCGTACTTCATCACAATGTT	C11orf74__NM_001276722	728	1012	828	956
C11orf74__NM_001276722_TGTCCAAATCTTCTAAATCT	C11orf74__NM_001276722	625	921	837	991
C11orf74__NM_001276722_TTATTTCCAATCCTGACATA	C11orf74__NM_001276722	12	18	20	48
C11orf80__NM_024650_AATGAACCCACTTTGAACCT	C11orf80__NM_024650	793	913	832	732
C11orf80__NM_024650_AGCGGCAACGCCCAGTCGAA	C11orf80__NM_024650	324	207	330	278
C11orf80__NM_024650_AGCTATTCTCAGGATATGAC	C11orf80__NM_024650	122	101	161	192
C11orf80__NM_024650_CAAAGGGAGCATTGCTAGAA	C11orf80__NM_024650	178	269	156	114
C11orf80__NM_024650_CATTTCAGTGTAAAGGTAAA	C11orf80__NM_024650	172	260	200	261
C11orf80__NM_024650_CCATTCCCAAGGTTCAAAGT	C11orf80__NM_024650	698	766	780	772
C11orf80__NM_024650_CGTCCCCTCCATGCCGCGCA	C11orf80__NM_024650	1451	1586	1546	1663
C11orf80__NM_024650_GTGTGACATAGATGTATCAA	C11orf80__NM_024650	117	113	124	157
C11orf80__NM_024650_TCCATTCCCAAGGTTCAAAG	C11orf80__NM_024650	85	75	90	162
C11orf80__NM_024650_TCGACTCCCAGCATTATGTG	C11orf80__NM_024650	209	143	273	245
C11orf80__NM_024650_TGCAGAGATTCCGGGCAGTA	C11orf80__NM_024650	411	358	515	687
C11orf80__NM_024650_TGTTCCCTGCGCGGCATGGA	C11orf80__NM_024650	110	54	114	162
C11orf84__NM_138471_ACCATGGCCCTAAAGGCCGA	C11orf84__NM_138471	43	55	36	37
C11orf84__NM_138471_ACTGCCTGCCTCTAGCGGGC	C11orf84__NM_138471	101	38	103	148
C11orf84__NM_138471_AGACCCAGCCCGCTAGAGGC	C11orf84__NM_138471	161	174	186	179
C11orf84__NM_138471_CACCTCGAAGCAGTCGAGTG	C11orf84__NM_138471	440	407	533	540
C11orf84__NM_138471_CAGCTCTCACGTCTTGCAAG	C11orf84__NM_138471	519	604	490	690
C11orf84__NM_138471_CTGCCTGCCTCTAGCGGGCT	C11orf84__NM_138471	188	168	175	213
C11orf84__NM_138471_GATGTGCGAGCGAGCTGTGT	C11orf84__NM_138471	903	1005	962	939
C11orf84__NM_138471_GCGGGCTGGGTCTAGGCGGT	C11orf84__NM_138471	2162	2662	2355	2899
C11orf84__NM_138471_GGGCTCAGGTCCAAGGTGTG	C11orf84__NM_138471	705	890	856	957
C11orf84__NM_138471_TGCGGCGCCCTCGGCCTTTA	C11orf84__NM_138471	407	311	553	488
C11orf84__NM_138471_TGGTGGTGGCCGTAATTCCG	C11orf84__NM_138471	71	11	96	115
C11orf84__NM_138471_TTCTGAGAAGAGCAATATCC	C11orf84__NM_138471	4	41	34	0
C11orf85__NM_001037225_AATGAAGTGTTCTGTAGCAA	C11orf85__NM_001037225	435	377	498	527
C11orf85__NM_001037225_AGGAGAAGAGATAGAAAATA	C11orf85__NM_001037225	50	77	29	14
C11orf85__NM_001037225_AGGGGTGGGACAATTCGCCC	C11orf85__NM_001037225	216	213	313	255
C11orf85__NM_001037225_ATGAAGTGTTCTGTAGCAAA	C11orf85__NM_001037225	129	86	120	116
C11orf85__NM_001037225_CCCACCTGAAATTCAAACAT	C11orf85__NM_001037225	927	852	1145	1017
C11orf85__NM_001037225_CTACTGCTTTCTTCTCAACT	C11orf85__NM_001037225	1649	2279	2163	2544
C11orf85__NM_001037225_GAGTCTCCTGCAATCCTTGT	C11orf85__NM_001037225	955	1200	935	1063
C11orf85__NM_001037225_GCTGCTCGCTCCTTAGGTGG	C11orf85__NM_001037225	339	370	263	194
C11orf85__NM_001037225_TCTGGAAACGGGTAGGTAAA	C11orf85__NM_001037225	134	216	146	221
C11orf86__NM_001136485_CCACCGGCTGCCTCTTCGCT	C11orf86__NM_001136485	4518	4348	5165	5070
C11orf86__NM_001136485_CGAGAGCCCCGACCCTCCTA	C11orf86__NM_001136485	2951	2203	3179	2564
C11orf86__NM_001136485_GTCTGAGGCTTAGCGCCCTG	C11orf86__NM_001136485	118	91	149	115
C11orf86__NM_001136485_GTGGCTGAGGCGGTACCAAC	C11orf86__NM_001136485	109	104	112	60
C11orf87__NM_207645_CAACGACGAGGCGTTGGAAG	C11orf87__NM_207645	160	103	182	74
C11orf87__NM_207645_CAGCCGGGTTTCCTTTGCGT	C11orf87__NM_207645	4355	3780	4982	4063
C11orf87__NM_207645_CGGGTTTCCTTTGCGTGGGT	C11orf87__NM_207645	619	487	736	894
C11orf87__NM_207645_CTCGCCAGCTGTGTCCAAAC	C11orf87__NM_207645	550	767	615	802
C11orf87__NM_207645_CTTTCCACATCCACAAGCGT	C11orf87__NM_207645	215	42	206	155
C11orf87__NM_207645_GCAGGCCCCAACCCACGCAA	C11orf87__NM_207645	225	175	202	229
C11orf87__NM_207645_GCCAGCTGTGTCCAAACAGG	C11orf87__NM_207645	806	589	755	654
C11orf87__NM_207645_GCTTCTTCATCCTACGCTTG	C11orf87__NM_207645	807	869	945	1075
C11orf87__NM_207645_GGGAAGCAAAGGTCCGGTTG	C11orf87__NM_207645	491	315	387	418
C11orf87__NM_207645_GGGTTTCCTTTGCGTGGGTT	C11orf87__NM_207645	189	166	214	317
C11orf87__NM_207645_TGGAGAGGGACAGCACGATG	C11orf87__NM_207645	188	144	252	258
C11orf88__NM_001100388_AATGTGCCCCCCGGGATTAC	C11orf88__NM_001100388	625	421	507	412
C11orf88__NM_001100388_ACCAGCTGAGATTCGCTAGG	C11orf88__NM_001100388	706	403	805	479
C11orf88__NM_001100388_AGCACCAGCTGAGATTCGCT	C11orf88__NM_001100388	1268	1204	1504	1388
C11orf88__NM_001100388_CAAATACCAGTAATCCCGGG	C11orf88__NM_001100388	974	604	1269	938
C11orf88__NM_001100388_CACCAGCTGAGATTCGCTAG	C11orf88__NM_001100388	799	447	892	653
C11orf88__NM_001100388_CCAACTTGGCTAAGCAGTTC	C11orf88__NM_001100388	552	846	597	1090
C11orf88__NM_001100388_CCAGCAAATACCAGTAATCC	C11orf88__NM_001100388	793	506	815	512
C11orf88__NM_001100388_GCAAATACCAGTAATCCCGG	C11orf88__NM_001100388	830	490	931	554
C11orf88__NM_001100388_TGCCGAAGCCCTAAAGATAC	C11orf88__NM_001100388	1419	1698	1697	1867
C11orf91__NM_001166692_AGGAGATGCCGCGGTCGTAC	C11orf91__NM_001166692	676	805	827	726
C11orf91__NM_001166692_CGTACAGGGACGGGAAATAG	C11orf91__NM_001166692	835	844	796	802
C11orf91__NM_001166692_CTAAAAGACGAAAAGTTACA	C11orf91__NM_001166692	329	366	351	523
C11orf91__NM_001166692_GTACAGGGACGGGAAATAGA	C11orf91__NM_001166692	181	125	171	177
C11orf91__NM_001166692_TTCCCGTCCCTGTACGACCG	C11orf91__NM_001166692	9	0	39	81
C11orf94__NM_001080446_CATCATAGTAATACTCGGCA	C11orf94__NM_001080446	634	670	716	812
C11orf94__NM_001080446_CCAAGCACCCAAGGCCGCGA	C11orf94__NM_001080446	600	803	624	583
C11orf94__NM_001080446_CTAGGGCCCAGCAGCGCAAG	C11orf94__NM_001080446	164	145	157	182
C11orf94__NM_001080446_CTGCGAGAGTTCCAGGGGGG	C11orf94__NM_001080446	457	563	528	520
C11orf94__NM_001080446_GTGTGCATCATAGTAATACT	C11orf94__NM_001080446	344	379	357	336
C11orf94__NM_001080446_TGCGCTGCTGGGCCCTAGAC	C11orf94__NM_001080446	1115	1156	1228	1211
C11orf94__NM_001080446_TGCTGGGCCCTAGACAGGAG	C11orf94__NM_001080446	225	75	219	245
C11orf95__NM_001144936_AGCACCCCTACTCCTTGCAT	C11orf95__NM_001144936	2681	2681	3005	2669
C11orf95__NM_001144936_AGTCCCGGCTCTTTCCGGTC	C11orf95__NM_001144936	1073	1033	1470	1388
C11orf95__NM_001144936_GAGCCAGAGGAAGACGAAGG	C11orf95__NM_001144936	167	65	170	139
C11orf95__NM_001144936_GCATCCCTGGCCGTGACCAC	C11orf95__NM_001144936	1044	893	1064	944
C11orf95__NM_001144936_GCTACTACCACGACCACTGG	C11orf95__NM_001144936	1171	763	1309	976
C11orf95__NM_001144936_GGACTCCAATGCAAGGAGTA	C11orf95__NM_001144936	87	175	139	189
C11orf95__NM_001144936_GGCCCTTGGGCGGGCAAGCT	C11orf95__NM_001144936	48	50	92	94
C11orf95__NM_001144936_GGCGCTACTACCACGACCAC	C11orf95__NM_001144936	766	838	976	1081
C11orf95__NM_001144936_GGGGCTGAGATCCTGAGAGG	C11orf95__NM_001144936	360	66	391	96
C11orf95__NM_001144936_GTCTCATGGACTATGACCCG	C11orf95__NM_001144936	315	256	381	267
C11orf95__NM_001144936_TCTCAGCCCCCCAGACGTAA	C11orf95__NM_001144936	319	236	296	192
C11orf95__NM_001144936_TGTCCCAGCTTGCCCGCCCA	C11orf95__NM_001144936	1019	860	1083	783
C11orf96__NM_001145033_CACGGGCTGCGGGAACTCGT	C11orf96__NM_001145033	1533	1422	1544	1555
C11orf96__NM_001145033_CAGATGCCCATCAGCTCGCC	C11orf96__NM_001145033	428	499	494	495
C11orf96__NM_001145033_GGTGACCTTCGACGAGATCC	C11orf96__NM_001145033	288	251	333	512
C11orf97__NM_001190462_AAGGGATTTGGAGCATTAAA	C11orf97__NM_001190462	128	121	126	211
C11orf97__NM_001190462_ACACTGCTGGCCGTGCTCTA	C11orf97__NM_001190462	468	479	608	481
C11orf97__NM_001190462_ACTGCTGGCCGTGCTCTAGG	C11orf97__NM_001190462	1100	1457	1040	903
C11orf97__NM_001190462_ACTTCCTTAATTCTCTTATG	C11orf97__NM_001190462	426	350	486	383
C11orf97__NM_001190462_CACTGCTGGCCGTGCTCTAG	C11orf97__NM_001190462	552	561	656	553
C11orf97__NM_001190462_CAGTGGCCCTGGAAGGGATT	C11orf97__NM_001190462	487	265	451	431
C11orf97__NM_001190462_CATTAAAAGGAATCTGCCTG	C11orf97__NM_001190462	768	1035	800	1334
C11orf97__NM_001190462_CTGGAGTAGTACTTGGCTTG	C11orf97__NM_001190462	595	811	757	771
C11orf97__NM_001190462_TGAGCCACATAAGAGAATTA	C11orf97__NM_001190462	268	268	324	413
C11orf98__NM_001286086_AGAAGAAGCTGTTCAAACGC	C11orf98__NM_001286086	288	204	339	331
C11orf98__NM_001286086_CGTGCCAACATTACACTGTC	C11orf98__NM_001286086	1557	1826	1642	1923
C11orf98__NM_001286086_CTCCGGGGGGAAAGATCAAC	C11orf98__NM_001286086	263	327	216	203
C11orf98__NM_001286086_GGTCCTTCATTTCTACATCC	C11orf98__NM_001286086	366	449	436	529
C11orf98__NM_001286086_GTCGGGGCTGTGATAGACCA	C11orf98__NM_001286086	608	416	665	537
C12orf10__NM_021640_ATGCGGTGCCGGGTATACAG	C12orf10__NM_021640	405	393	423	289
C12orf10__NM_021640_ATGGGACACCAATTCCTGCG	C12orf10__NM_021640	865	1100	792	1288
C12orf10__NM_021640_CATGCGGTGCCGGGTATACA	C12orf10__NM_021640	661	545	622	420
C12orf10__NM_021640_CCCCGAATCGGACGCACAA	C12orf10__NM_021640	176	147	194	285
C12orf10__NM_021640_CGGTGCCGGGTATACAGGGG	C12orf10__NM_021640	292	291	425	287
C12orf10__NM_021640_CTCATGGCACCGCCCCGAAT	C12orf10__NM_021640	1674	1474	2025	1170
C12orf10__NM_021640_GCATGCGGTGCCGGGTATAC	C12orf10__NM_021640	810	764	843	568
C12orf10__NM_021640_GCCATTGTGCGTCCCGATTC	C12orf10__NM_021640	2066	2515	2010	1489
C12orf10__NM_021640_GCGGCAGCAGCAGCGTTAAG	C12orf10__NM_021640	277	96	327	288
C12orf10__NM_021640_TATACCCGGCACCGCATGCT	C12orf10__NM_021640	793	710	725	679
C12orf10__NM_021640_TTAAGAGGCCGCGCAGGAAT	C12orf10__NM_021640	453	603	427	619
C12orf10__NM_021640_TTGTGCGTCCCGATTCGGGG	C12orf10__NM_021640	230	92	194	116
C12orf29__NM_001009894_AAAACAACAAACAGTATTGC	C12orf29__NM_001009894	23	20	33	29
C12orf29__NM_001009894_AATATCAATGGAAACCCATA	C12orf29__NM_001009894	76	143	81	78
C12orf29__NM_001009894_CAGTGATGGCTGTTTAATAA	C12orf29__NM_001009894	1295	1395	1519	1501
C12orf29__NM_001009894_GGAGTGCTGGATACCAGCAA	C12orf29__NM_001009894	97	138	95	70
C12orf29__NM_001009894_TTCATGTAAGTATCTGGAAT	C12orf29__NM_001009894	73	129	66	111
C12orf29__NM_001009894_TTCTATCTAGTCGAGCCCAA	C12orf29__NM_001009894	1	0	1	9
C12orf4__NM_020374_AGACCACGTTAGAGCATGCA	C12orf4__NM_020374	332	258	228	191
C12orf4__NM_020374_AGAGAATCTGTAGAAGCAAT	C12orf4__NM_020374	428	442	436	417
C12orf4__NM_020374_AGCAATTGGAAGGATTTATG	C12orf4__NM_020374	363	416	481	324
C12orf4__NM_020374_ATCTCTATCATAATCGCTGA	C12orf4__NM_020374	634	728	659	1019
C12orf4__NM_020374_CAAGGCCTGAAGAACCCAGC	C12orf4__NM_020374	241	276	374	439
C12orf4__NM_020374_CAGGAGAATGAATTAAGTCA	C12orf4__NM_020374	410	380	401	273
C12orf4__NM_020374_CATCTCTATCATAATCGCTG	C12orf4__NM_020374	467	373	425	401
C12orf4__NM_020374_CTCTGAAACTCTCTTAAATT	C12orf4__NM_020374	80	104	109	232
C12orf4__NM_020374_GCAAAATCTTCATCCCAGCT	C12orf4__NM_020374	704	808	768	1080
C12orf4__NM_020374_GTTAGTATCTCAGAACTGAT	C12orf4__NM_020374	265	236	391	347
C12orf4__NM_020374_TCCCTGGCAGCTGTGAAATC	C12orf4__NM_020374	794	670	617	814
C12orf4__NM_020374_TGAAGTAGATTTACATCAGC	C12orf4__NM_020374	124	207	103	146
C12orf40__NM_001031748_AACACTCAATACAGCATATT	C12orf40__NM_001031748	88	61	118	178
C12orf40__NM_001031748_AATTCCTATTTCCTAGACTT	C12orf40__NM_001031748	231	212	204	226
C12orf40__NM_001031748_ATAGAAGGTTAGGAGTAGAC	C12orf40__NM_001031748	238	291	211	168
C12orf40__NM_001031748_CAATTCTCTGATAGAAGGTT	C12orf40__NM_001031748	744	568	771	908
C12orf40__NM_001031748_CACATAAAACTACACGATTT	C12orf40__NM_001031748	640	776	743	731
C12orf40__NM_001031748_GGATGAAGATTGTAGAAGCA	C12orf40__NM_001031748	399	553	602	634
C12orf40__NM_001031748_GGGAAGATGAATTGGGTCGG	C12orf40__NM_001031748	568	798	734	769
C12orf40__NM_001031748_GTCCAGGGTTCTGATCAAGC	C12orf40__NM_001031748	329	429	335	275
C12orf40__NM_001031748_TAAAAGCCCTGCTGTAATTA	C12orf40__NM_001031748	430	679	667	633
C12orf40__NM_001031748_TCACATAAAACTACACGATT	C12orf40__NM_001031748	674	899	797	811
C12orf40__NM_001031748_TCTTCATCCATAATTACAGC	C12orf40__NM_001031748	123	231	143	198
C12orf40__NM_001031748_TGGGAAGATGAATTGGGTCG	C12orf40__NM_001031748	182	210	201	147
C12orf42__NM_001099336_AAGTGTAGACTACGAAATTT	C12orf42__NM_001099336	288	376	393	553
C12orf42__NM_001099336_ACTGAAGTTCTTTCATAACA	C12orf42__NM_001099336	379	442	476	648
C12orf42__NM_001099336_ACTGGTTAAAAAGCCTAACT	C12orf42__NM_001099336	359	187	369	283
C12orf42__NM_001099336_CACAATGGGAATATAGCAAG	C12orf42__NM_001099336	214	204	209	137
C12orf42__NM_001099336_CAGTAGACACACAAGACCTA	C12orf42__NM_001099336	579	492	585	810
C12orf42__NM_001099336_CTCTGGCAGTAAAAATCAAT	C12orf42__NM_001099336	363	319	390	466
C12orf42__NM_001099336_CTGAAGTTCTTTCATAACAA	C12orf42__NM_001099336	204	116	129	52
C12orf42__NM_001099336_GAGGAGCACCCAAGCAGGCT	C12orf42__NM_001099336	661	591	754	657
C12orf42__NM_001099336_GCCTCATCAGTTTCACTGGA	C12orf42__NM_001099336	746	885	854	931
C12orf42__NM_001099336_TAGAAGCACACCCAGTGCAA	C12orf42__NM_001099336	221	233	300	234
C12orf42__NM_001099336_TTGTGAGCAGTGCCACCCTG	C12orf42__NM_001099336	745	364	801	760
C12orf42__NM_001099336_TTTACTGAGTGTGCCCAGTT	C12orf42__NM_001099336	343	496	382	559
C12orf42__NM_001278419_AGTAGACACACAAGACCTAA	C12orf42__NM_001278419	277	320	311	228
C12orf42__NM_001278419_CAAGCATCCTCATACCCCGC	C12orf42__NM_001278419	1547	1263	1616	1345
C12orf42__NM_001278419_CTCCTGTCCCGCGGGGTATG	C12orf42__NM_001278419	517	250	489	373
C12orf42__NM_001278419_GAGTCTGCTTTGGATGTCGT	C12orf42__NM_001278419	890	947	849	1176
C12orf42__NM_001278419_GGCGCGTCCGGAAATCCCGT	C12orf42__NM_001278419	2157	3158	2257	2573
C12orf42__NM_001278419_TCCGGAAATCCCGTCGGAAA	C12orf42__NM_001278419	266	231	271	347
C12orf43__NM_001286191_AAAGGAGCCAGCAAAAGCTA	C12orf43__NM_001286191	10	1	9	10
C12orf43__NM_001286191_AAGCCGGCATTGCCGCCTCG	C12orf43__NM_001286191	5	0	24	28
C12orf43__NM_001286191_AGCAACGCCCGCACGTGGCA	C12orf43__NM_001286191	1460	1460	1444	1339
C12orf43__NM_001286191_CAGGGAAGCCAAGAGCCGGT	C12orf43__NM_001286191	441	245	506	405
C12orf43__NM_001286191_CTTGGAGCAACGCCCGCACG	C12orf43__NM_001286191	884	644	913	672
C12orf43__NM_001286191_GAGGCGGCAATGCCGGCTTG	C12orf43__NM_001286191	57	132	58	73
C12orf43__NM_001286191_GCCTCAGGTACTATGCACTT	C12orf43__NM_001286191	1949	1522	2253	1834
C12orf43__NM_001286191_GCGAGGCGGCAATGCCGGCT	C12orf43__NM_001286191	159	58	128	158
C12orf43__NM_001286191_TAACAGCAGTAGCGATGCGG	C12orf43__NM_001286191	100	163	102	94
C12orf43__NM_001286191_TACGTGGGCTCGGAATTCAG	C12orf43__NM_001286191	335	320	348	260
C12orf43__NM_001286191_TCCCAGCTTCTTGGCTACGT	C12orf43__NM_001286191	872	803	867	744
C12orf43__NM_001286191_TGTCCCTGGAGGCCGTGAGA	C12orf43__NM_001286191	147	125	125	76
C12orf43__NM_001286192_CTGTCGCTTTCGGCGGGGTT	C12orf43__NM_001286192	438	479	443	482
C12orf43__NM_001286195_AGCGACAGCCGAGTCGACAC	C12orf43__NM_001286195	859	835	1054	823
C12orf43__NM_001286197_AGAAGTCAGGTGAGCTCAAC	C12orf43__NM_001286197	175	117	156	132
C12orf43__NM_001286198_ACGGGGACCAGGTGTCGCTT	C12orf43__NM_001286198	896	969	914	816
C12orf43__NM_001286198_ATAGCTGTAGCACTCTTTGC	C12orf43__NM_001286198	183	149	199	124
C12orf43__NM_001286198_GAAGTCAGGTGAGCTCAACG	C12orf43__NM_001286198	278	274	239	155
C12orf45__NM_152318_AGGTCTTTCTGGACTTAGGT	C12orf45__NM_152318	124	186	133	121
C12orf45__NM_152318_CGGGTGGGCGACGAACAACT	C12orf45__NM_152318	381	219	479	291
C12orf45__NM_152318_GACTCCTGAGGAATCCCGGG	C12orf45__NM_152318	829	546	1039	401
C12orf45__NM_152318_GGGAAGCTTAATGTTATCTA	C12orf45__NM_152318	503	422	567	435
C12orf45__NM_152318_GTCAGCAGCTCCTTGGACAC	C12orf45__NM_152318	801	294	772	431
C12orf45__NM_152318_TCAATATTGAAACGACCAGG	C12orf45__NM_152318	231	79	218	33
C12orf49__NM_024738_AACTGCACTTTCCACGGGAT	C12orf49__NM_024738	1357	1129	1545	988
C12orf49__NM_024738_AGCAGTACTGCTTCGTGCTA	C12orf49__NM_024738	816	742	771	736
C12orf49__NM_024738_AGGAGAGGGCAGTGAGAGAT	C12orf49__NM_024738	275	200	325	249
C12orf49__NM_024738_CACCTCATCACGGATGAACT	C12orf49__NM_024738	1359	1446	1359	1182
C12orf49__NM_024738_CAGCAGTACTGCTTCGTGCT	C12orf49__NM_024738	1399	1590	1544	1473
C12orf49__NM_024738_CCCATAGCAAAGTATTGCTA	C12orf49__NM_024738	260	126	213	117
C12orf49__NM_024738_CCGAGCTCTTCCCCGCTTGA	C12orf49__NM_024738	3832	3845	4197	3958
C12orf49__NM_024738_GCAGGAGACACAGTACTCAT	C12orf49__NM_024738	777	889	866	691
C12orf49__NM_024738_TCCACGGGATGGGCTGATTA	C12orf49__NM_024738	320	322	371	447
C12orf49__NM_024738_TCCATAGCAATACTTTGCTA	C12orf49__NM_024738	286	308	390	293
C12orf49__NM_024738_TGGAAAGTGCAGTTTAACTT	C12orf49__NM_024738	69	119	59	33
C12orf49__NM_024738_TTTCAGGCTACGTTTGCGAG	C12orf49__NM_024738	140	202	147	206
C12orf5__NM_020375_AAGACACTGGCTGCTAATCC	C12orf5__NM_020375	704	686	727	832
C12orf5__NM_020375_AGGAAGAGAAGTTAAACCAA	C12orf5__NM_020375	107	110	157	118
C12orf5__NM_020375_AGGAGAAAATAATCCAAGGT	C12orf5__NM_020375	141	183	148	193
[illegible]	[illegible]	[illegible]	[illegible]	[illegible]	[illegible]

C17orf80__NM_001100621_GGTCCTATCATCTTACAGTA	C17orf80__NM_001100621	373	346	353	329
C17orf80__NM_001100621_TTAAATGGCTTCTTACAGTA	C17orf80__NM_001100621	83	33	56	42
C17orf80__NM_001100621_TTAGCACGTGGGAGTGTAGC	C17orf80__NM_001100621	104	66	128	150
C17orf82__NM_203425_AGGCTCGGGGTATAAATCCA	C17orf82__NM_203425	143	101	138	121
C17orf82__NM_203425_ATCTGTTCCTCCTATTGGAT	C17orf82__NM_203425	458	420	550	503
C17orf82__NM_203425_CGGTGCTTCCGGAGTGGGTT	C17orf82__NM_203425	540	713	518	657
C17orf82__NM_203425_GCCGCCCTGTCCTGCGAGCA	C17orf82__NM_203425	1932	2274	2001	2482
C17orf82__NM_203425_GGGCCAAGGACAGCGAAGGA	C17orf82__NM_203425	231	223	248	182
C17orf82__NM_203425_GGTCCCCGGCCCCATCCAAT	C17orf82__NM_203425	144	105	171	161
C17orf82__NM_203425_GTTATGGGACGGCCCCTTGA	C17orf82__NM_203425	244	151	206	152
C17orf82__NM_203425_TATAAATCCAGGGCTCGTAG	C17orf82__NM_203425	101	92	145	126
C17orf82__NM_203425_TCAAGGGGCCGTCCCATAAC	C17orf82__NM_203425	445	237	395	562
C17orf85__NM_001114118_AAGGAATTCTTAGCAATTCA	C17orf85__NM_001114118	58	11	65	111
C17orf85__NM_001114118_AGCTGGAAATCGTGCCTGTG	C17orf85__NM_001114118	672	707	783	1078
C17orf85__NM_001114118_ATCATTCCCGTCGTATTCAG	C17orf85__NM_001114118	442	577	409	301
C17orf85__NM_001114118_CACGTCCCGCTGAATACGAC	C17orf85__NM_001114118	660	879	702	677
C17orf85__NM_001114118_CATATTGATAAGTGCTCGTG	C17orf85__NM_001114118	525	483	572	473
C17orf85__NM_001114118_CCTTTAGCAAGTTTGTTAGC	C17orf85__NM_001114118	640	888	644	862
C17orf85__NM_001114118_CGGTGTTGACCGTGGCGAGC	C17orf85__NM_001114118	532	775	575	547
C17orf85__NM_001114118_CTGGAGACAATCTATATTTG	C17orf85__NM_001114118	479	455	452	316
C17orf85__NM_001114118_GATTTACTTCCGATCGAAAA	C17orf85__NM_001114118	276	380	351	475
C17orf85__NM_001114118_TCATTCCCGTCGTATTCAGC	C17orf85__NM_001114118	690	797	707	677
C17orf85__NM_001114118_TGAGAACTCAAGTGATGTAG	C17orf85__NM_001114118	224	180	170	153
C17orf85__NM_001114118_TTTAGGTAATGTAGTTTGGC	C17orf85__NM_001114118	200	396	190	346
C17orf89__NM_001086521_GCTCATGTCGGCTAACGGAG	C17orf89__NM_001086521	228	213	212	121
C17orf89__NM_001086521_TGTCGGCTAACGGAGCGGTG	C17orf89__NM_001086521	98	53	144	42
C17orf96__NM_001130677_AAGCTGGTCGTAGATTCCCG	C17orf96__NM_001130677	391	208	346	320
C17orf96__NM_001130677_AGCTGGTCGTAGATTCCCGA	C17orf96__NM_001130677	1591	1096	1618	1576
C17orf96__NM_001130677_AGTCGATGGAAACCCCCCGC	C17orf96__NM_001130677	1207	1018	1181	1036
C17orf96__NM_001130677_ATTTCGATCGTCTGATCCGC	C17orf96__NM_001130677	180	139	244	200
C17orf96__NM_001130677_CCACGCTGAGGCTCGAGACC	C17orf96__NM_001130677	259	259	340	182
C17orf96__NM_001130677_CGACACGGCTTCCGGGGCGT	C17orf96__NM_001130677	556	515	573	524
C17orf96__NM_001130677_CTGGCGGAGATCTCCGGGTG	C17orf96__NM_001130677	895	892	839	749
C17orf96__NM_001130677_GCGGCGGTGTTAGGAACGCC	C17orf96__NM_001130677	25	24	37	11
C17orf96__NM_001130677_GGAGGCGGTTTCCCGCACTT	C17orf96__NM_001130677	615	370	507	452
C17orf96__NM_001130677_GGCGTTCCTAACACCGCCGC	C17orf96__NM_001130677	685	594	509	434
C17orf96__NM_001130677_GGGCTTGGCAAGGGCGCAGA	C17orf96__NM_001130677	192	283	313	324
C17orf96__NM_001130677_TTCCATCGACTTCGGCAGGC	C17orf96__NM_001130677	44	26	25	93
C17orf97__NM_001013672_AAATAGAGAACCTTGCGAAT	C17orf97__NM_001013672	275	361	268	238
C17orf97__NM_001013672_AGGACTTAAGATGTCATGAA	C17orf97__NM_001013672	130	222	196	125
C17orf97__NM_001013672_AGTCGCCGATTAGTCGGCAT	C17orf97__NM_001013672	592	484	706	612
C17orf97__NM_001013672_ATGGGGTACAAGGACAACGA	C17orf97__NM_001013672	125	176	161	127
C17orf97__NM_001013672_ATTCGCAAGGTTCTCTATTT	C17orf97__NM_001013672	479	400	490	533
C17orf97__NM_001013672_GAGGCCCGATGCCGACTAAT	C17orf97__NM_001013672	209	175	232	208
C17orf97__NM_001013672_GATTAGTCGGCATCGGGCCT	C17orf97__NM_001013672	583	888	681	857
C17orf97__NM_001013672_GCTGAGAGTCGCCGATTAGT	C17orf97__NM_001013672	1174	1151	1346	1417
C17orf97__NM_001013672_GTACAAGGACAACGACGGCG	C17orf97__NM_001013672	233	157	252	262
C17orf97__NM_001013672_GTCGCCGATTAGTCGGCATC	C17orf97__NM_001013672	241	223	234	230
C17orf97__NM_001013672_TCTCTATTTCGGCAAAGTCG	C17orf97__NM_001013672	124	78	139	155
C17orf97__NM_001013672_TTCGGGCCACGCTCTTTGCA	C17orf97__NM_001013672	462	354	429	647
C17orf98__NM_001080465_AAACCGATCGATGGGTTCAA	C17orf98__NM_001080465	255	203	289	305
C17orf98__NM_001080465_AAGGACAAAGATACCTCAAC	C17orf98__NM_001080465	427	367	438	235
C17orf98__NM_001080465_CCTAGAGCGCCCATAAGCGC	C17orf98__NM_001080465	8	31	8	10
C17orf98__NM_001080465_CGATCGATGGGTTCAATGGA	C17orf98__NM_001080465	927	1053	1074	1111
C17orf98__NM_001080465_CTGATCTGAAACCGATCGAT	C17orf98__NM_001080465	305	304	277	452
C17orf98__NM_001080465_CTTCCATTGAACCCATCGAT	C17orf98__NM_001080465	658	700	812	818
C17orf98__NM_001080465_GAAAGGCTTTATCTTGGACG	C17orf98__NM_001080465	328	298	351	297
C17orf98__NM_001080465_GACGGACGTGCTCTGACGGA	C17orf98__NM_001080465	132	203	216	113
C17orf98__NM_001080465_GCACCGCTGCCCGCGCTTAT	C17orf98__NM_001080465	743	878	619	806
C17orf98__NM_001080465_GCCTAGAGCGCCCATAAGCG	C17orf98__NM_001080465	346	370	365	437
C17orf98__NM_001080465_TCCTGCTGCGCGTTGTAGGG	C17orf98__NM_001080465	334	344	349	278
C17orf98__NM_001080465_TTTCAGGATCATGGCGGTAC	C17orf98__NM_001080465	63	66	49	83
C17orf99__NM_001163075_ACAGCCTGATCGGGAAGGAT	C17orf99__NM_001163075	784	600	744	601
C17orf99__NM_001163075_ACCAGAACCAGTCCGATGTC	C17orf99__NM_001163075	1950	2448	2239	2645
C17orf99__NM_001163075_ACTCCTCTTCACTCAGACGG	C17orf99__NM_001163075	784	610	693	552
C17orf99__NM_001163075_CAGGGTGGAGATGATCTGCC	C17orf99__NM_001163075	746	415	792	427
C17orf99__NM_001163075_CCACAGAGGGAATAGGTGAT	C17orf99__NM_001163075	1304	1372	1674	1851
C17orf99__NM_001163075_CGGCCAAGCAGAACAGCCCA	C17orf99__NM_001163075	547	697	587	546
C17orf99__NM_001163075_CTTCCTGCCGAGCCAGACAT	C17orf99__NM_001163075	223	95	264	240
C17orf99__NM_001163075_TAGAGCGGCAAGGCAAGGAT	C17orf99__NM_001163075	530	543	531	463
C17orf99__NM_001163075_TCCTTGCCTTGCCGCTCTAC	C17orf99__NM_001163075	741	466	719	759
C17orf99__NM_001163075_TGCCGAGCCAGACATCGGAC	C17orf99__NM_001163075	909	882	1027	951
C17orf99__NM_001163075_TGGACTTGAGTGTGACGTTG	C17orf99__NM_001163075	139	97	82	111
C17orf99__NM_001163075_TTCCAGGACTTTGTAGGCAA	C17orf99__NM_001163075	15	13	31	17
C18orf21__NM_001201474_AACAGAACAGTGAAACATCA	C18orf21__NM_001201474	239	76	226	28
C18orf21__NM_001201474_AACCCAAATCATGACATGTC	C18orf21__NM_001201474	654	234	705	392
C18orf21__NM_001201474_AGCCAGACATGTCATGATTT	C18orf21__NM_001201474	992	588	1074	394
C18orf21__NM_001201474_ATCCCAAAAGATTCCAAAGG	C18orf21__NM_001201474	55	59	61	30
C18orf21__NM_001201474_GAGCCAGACATGTCATGATT	C18orf21__NM_001201474	1123	809	1295	848
C18orf21__NM_001201474_GATACCTGAAAACCGATGCT	C18orf21__NM_001201474	610	198	673	150
C18orf21__NM_001201476_ACCAACAGCTGGAAACAGTA	C18orf21__NM_001201476	166	18	194	0
C18orf21__NM_001201476_CTTACAGGAGGTAGCGGGCT	C18orf21__NM_001201476	515	594	603	794
C18orf21__NM_001201476_CTTACCGTGCGACGAAGTGT	C18orf21__NM_001201476	555	144	462	98
C18orf21__NM_001201476_GAGACAGAAGCACTACCTTG	C18orf21__NM_001201476	682	0	654	11
C18orf21__NM_001201476_TGGGCCTACACTTCGTCGCA	C18orf21__NM_001201476	139	65	102	26
C18orf25__NM_001008239_ACAAGTCAGGTCACTATCAC	C18orf25__NM_001008239	440	469	545	527
C18orf25__NM_001008239_AGGAGTCACTCTCATAAACT	C18orf25__NM_001008239	542	397	669	461
C18orf25__NM_001008239_AGTAGAGATTGTGGGAGTTC	C18orf25__NM_001008239	177	176	253	141
C18orf25__NM_001008239_CAGGCAGAAACGGCGGTAGA	C18orf25__NM_001008239	534	393	472	491
C18orf25__NM_001008239_CAGTTGGGCGCAAGTCTAGG	C18orf25__NM_001008239	1495	1291	1623	1439
C18orf25__NM_001008239_CCACAGTTGGGCGCAAGTCT	C18orf25__NM_001008239	589	396	630	417
C18orf25__NM_001008239_CCTAGACTTGCGCCCAACTG	C18orf25__NM_001008239	242	216	215	139
C18orf25__NM_001008239_GAGAGTGACAAGCCTACAAC	C18orf25__NM_001008239	320	111	358	179
C18orf25__NM_001008239_TCGTGCTTGAGTCACAAGTC	C18orf25__NM_001008239	422	493	464	360
C18orf25__NM_001008239_TCTGTTTATCACTGGATTGC	C18orf25__NM_001008239	489	488	534	655
C18orf25__NM_001008239_TGCAGGTGTGTTCATCCTCG	C18orf25__NM_001008239	402	289	459	340
C18orf25__NM_001008239_TTGTCACTTGCTAGGAATCC	C18orf25__NM_001008239	69	26	93	75
C18orf25__NM_145055_AAATGTGGACAATTTATTGG	C18orf25__NM_145055	268	325	286	229
C18orf25__NM_145055_GAGGACCAGCGGGCTTCTAG	C18orf25__NM_145055	332	326	395	352
C18orf25__NM_145055_TGACCCAGAAGTGGTAAATG	C18orf25__NM_145055	120	186	166	163
C18orf32__NM_001035005_AGAACTGGAATGACGATACA	C18orf32__NM_001035005	215	194	196	193
C18orf32__NM_001035005_AGGCCATATACGACTAACGA	C18orf32__NM_001035005	328	369	379	319
C18orf32__NM_001035005_ATGAATGGATTACCAACAAA	C18orf32__NM_001035005	5	17	23	28
C18orf32__NM_001035005_CAAAGGCAAAGTAAACTTTA	C18orf32__NM_001035005	105	149	99	96
C18orf32__NM_001035005_CCATATACGACTAACGAAGG	C18orf32__NM_001035005	285	218	336	432
C18orf32__NM_001035005_CCCCCTTCGTTAGTCGTATA	C18orf32__NM_001035005	600	592	736	744
C18orf32__NM_001035005_GCCATATACGACTAACGAAG	C18orf32__NM_001035005	319	366	509	431
C18orf32__NM_001035005_GGCCATATACGACTAACGAA	C18orf32__NM_001035005	286	171	223	310
C18orf32__NM_001035005_TTTGCCTTTGTTTGTATCAT	C18orf32__NM_001035005	697	755	662	806
C18orf42__NM_001145194_ACCACATCCAACTGGGCGTT	C18orf42__NM_001145194	632	797	637	878
C18orf42__NM_001145194_ATTCTGCACAATCTGTTTGC	C18orf42__NM_001145194	279	279	235	233
C18orf42__NM_001145194_GACCACATCCAACTGGGCGT	C18orf42__NM_001145194	695	953	856	972
C18orf42__NM_001145194_TTAACTCCCCAACGCCCAGT	C18orf42__NM_001145194	274	158	289	387
C18orf54__NM_001288980_AAGAAAAACAAGAAATGCCG	C18orf54__NM_001288980	212	344	269	440
C18orf54__NM_001288980_AGAGGAATTACTACCACTCA	C18orf54__NM_001288980	826	945	948	1181
C18orf54__NM_001288980_ATCATCAGTTGTTAGGCTCA	C18orf54__NM_001288980	287	362	446	475
C18orf54__NM_001288980_ATCTGCTTCTCAAGCTCTAC	C18orf54__NM_001288980	230	276	243	335
C18orf54__NM_001288980_ATTAACGGTGTGTCTTCTAC	C18orf54__NM_001288980	286	331	409	445
C18orf54__NM_001288980_CAAGAAATGCCGTGGAAGAC	C18orf54__NM_001288980	1195	1827	1297	1935
C18orf54__NM_001288980_CAAGGACCCTACACTTCCAT	C18orf54__NM_001288980	743	658	892	772
C18orf54__NM_001288980_GATCTAGGCCAAATATATCC	C18orf54__NM_001288980	302	357	391	349
C18orf54__NM_001288980_TAGAGGAATTACTACCACTC	C18orf54__NM_001288980	236	242	322	277
C18orf54__NM_001288980_TAGTTATCCCTGAAACATTA	C18orf54__NM_001288980	131	149	128	143
C18orf54__NM_001288980_TTCTGGCTAGTGAGCCATCT	C18orf54__NM_001288980	13	0	10	0
C18orf54__NM_001288980_TTCTTGCTCGTTCGGTGACT	C18orf54__NM_001288980	20	30	45	81
C18orf54__NM_001288982_TCAAGGGCAAAGAGTGCTAA	C18orf54__NM_001288982	165	259	223	336
C18orf54__NM_001288982_TCAAGTTTATCTAATGAGCA	C18orf54__NM_001288982	224	124	197	371
C18orf63__NM_001174123_AAATCTCCATATGAGTGAAA	C18orf63__NM_001174123	35	108	43	115
C18orf63__NM_001174123_ACTGGTCATCTCTTGATACA	C18orf63__NM_001174123	325	300	313	298
C18orf63__NM_001174123_AGGTGGCAGATACTGAGATT	C18orf63__NM_001174123	123	99	169	148
C18orf63__NM_001174123_AGTTCTTGTGTGTAGTAGCA	C18orf63__NM_001174123	235	197	347	223
C18orf63__NM_001174123_ATGGGAAATCCACATATATG	C18orf63__NM_001174123	124	243	245	267
C18orf63__NM_001174123_CTGCCTGTCATATTCATTCA	C18orf63__NM_001174123	904	1033	1204	1381
C18orf63__NM_001174123_GGTTTACTTGTCATCTTTAT	C18orf63__NM_001174123	1028	1281	1222	1541
C18orf63__NM_001174123_GTAGCACCAGTTGCTTAAAA	C18orf63__NM_001174123	73	55	97	109
C18orf63__NM_001174123_GTTCTTTCCAAGGGTAGATT	C18orf63__NM_001174123	13	24	10	5
C18orf63__NM_001174123_TCTAAACTGCCCCATATATG	C18orf63__NM_001174123	730	474	826	774
C18orf63__NM_001174123_TGACTTCTGATGCAACTGAG	C18orf63__NM_001174123	134	261	209	181
C18orf63__NM_001174123_TTATGGGAAATCCACATATA	C18orf63__NM_001174123	113	191	80	32
C18orf65__NM_001272093_AGTGCAGGTCGTTAGTCACA	C18orf65__NM_001272093	1520	1456	1652	1555
C18orf65__NM_001272093_CCGCGGCCCTCCCCAACTTA	C18orf65__NM_001272093	1052	948	1114	913
C18orf65__NM_001272093_CCTGGCGGTTACCTCAGCGG	C18orf65__NM_001272093	1243	1329	1302	1619
C18orf65__NM_001272093_CCTGGGCACTGCGCCCCATC	C18orf65__NM_001272093	442	710	726	728
C18orf65__NM_001272093_CGGAGCCACGTTGGGCGTCT	C18orf65__NM_001272093	483	458	430	551
C18orf65__NM_001272093_GAAAGCTGGCGATCAGACGG	C18orf65__NM_001272093	105	22	161	184
C18orf65__NM_001272093_GCCACGTTGGGCGTCTTGGA	C18orf65__NM_001272093	1762	2410	2234	2102
C18orf65__NM_001272093_GCGGTGGGGAGTGTGTGTCG	C18orf65__NM_001272093	1303	1076	1255	1201
C18orf65__NM_001272093_TAAACGCCCTAAGTTGGGGA	C18orf65__NM_001272093	116	98	100	91
C18orf65__NM_001272093_TACTCCAGACGCGCTGCAAA	C18orf65__NM_001272093	393	459	445	623
C18orf65__NM_001272093_TCCACTGCGGAGTTATCACG	C18orf65__NM_001272093	744	465	672	711
C18orf65__NM_001272093_TTTGTAAACGCCCTAAGTTG	C18orf65__NM_001272093	149	48	161	147
C18orf8__NM_001276342_AGGCTGGGTTTAGTTGACTT	C18orf8__NM_001276342	489	747	553	511
C18orf8__NM_001276342_ATCTGGTGGAGCTACTGGCG	C18orf8__NM_001276342	372	342	464	429
C18orf8__NM_001276342_CGAAGAAGACGCAGTTGACA	C18orf8__NM_001276342	499	398	497	389
C18orf8__NM_001276342_CTTGCTGTCTACCACGGTCC	C18orf8__NM_001276342	997	986	886	966
C18orf8__NM_001276342_GCGGCCGGTGCAGTTCGAGA	C18orf8__NM_001276342	2014	1428	1953	1460
C18orf8__NM_001276342_GCTACTGGCGTGGTAGTTAA	C18orf8__NM_001276342	616	580	487	543
C18orf8__NM_001276342_GGGCGAGGAGGACTACTATC	C18orf8__NM_001276342	423	246	490	330
C18orf8__NM_001276342_GTTCGATCTGGTGGAGCTAC	C18orf8__NM_001276342	72	67	83	69
C18orf8__NM_001276342_TCGAAGAAGACGCAGTTGAC	C18orf8__NM_001276342	445	358	490	371
C18orf8__NM_001276342_TTAGTTGACTTAGGCGCAGC	C18orf8__NM_001276342	95	25	59	18
C18orf8__NM_001276342_TTCAGGTATTACCAGAGAAA	C18orf8__NM_001276342	16	46	27	17
C18orf8__NM_001276342_TTCGCCTTCTCGAACTGCAC	C18orf8__NM_001276342	107	182	112	117
C18orf8__NM_013326_CCAGCAGAATCCTAGAATGT	C18orf8__NM_013326	1339	429	1124	602
C18orf8__NM_013326_TTCCCAGCTGGAATACACAC	C18orf8__NM_013326	106	41	127	127
C19orf10__NM_019107_AAGGAATGCACGACGCCGCC	C19orf10__NM_019107	382	308	471	591
C19orf10__NM_019107_ACGGCGTCGGCGCGAGCTTG	C19orf10__NM_019107	660	690	850	777
C19orf10__NM_019107_ATGTTCACTTACGCCTCTCA	C19orf10__NM_019107	589	349	713	530
C19orf10__NM_019107_CATTCCTTCTCCCATAACGT	C19orf10__NM_019107	1476	1163	1812	1057
C19orf10__NM_019107_CGACGGTGGCGTTTGACGTG	C19orf10__NM_019107	1085	1262	1378	1413
C19orf10__NM_019107_CGGCGTCGGCGCGAGCTTGT	C19orf10__NM_019107	633	278	687	474
C19orf10__NM_019107_CTGCTTTCCTATGTTTACCC	C19orf10__NM_019107	565	550	679	462
C19orf10__NM_019107_CTTCTCCCATAACGTGGGCC	C19orf10__NM_019107	188	350	279	368
C19orf10__NM_019107_GAACTGTGTGAAGTACAGAT	C19orf10__NM_019107	418	425	503	530
C19orf10__NM_019107_GAAGTGCTGGTGGTCTTCGC	C19orf10__NM_019107	1158	1069	1231	1547
C19orf10__NM_019107_GACTCAGGATCGCAGATCCG	C19orf10__NM_019107	120	45	136	94
C19orf10__NM_019107_GAGGTCCCCTGGGTAAACAT	C19orf10__NM_019107	269	168	212	219
C19orf10__NM_019107_GCATTCCTTCTCCCATAACG	C19orf10__NM_019107	878	1008	946	1225
C19orf10__NM_019107_GGACTCAGGATCGCAGATCC	C19orf10__NM_019107	0	0	1	0
C19orf10__NM_019107_GTACCCCCGGGCCCACGTTA	C19orf10__NM_019107	226	195	212	126
C19orf10__NM_019107_TACCCCCGGGCCCACGTTAT	C19orf10__NM_019107	1529	1085	1715	1266
C19orf10__NM_019107_TGCTTTCCTATGTTTACCCA	C19orf10__NM_019107	205	130	260	92
C19orf10__NM_019107_TGGACTCAGGATCGCAGATC	C19orf10__NM_019107	67	55	45	54
C19orf12__NM_001031726_AAGAGCCTCTGTTGCTCGGC	C19orf12__NM_001031726	332	649	499	706
C19orf12__NM_001031726_ACAGGGGCCATGGCCTTCGT	C19orf12__NM_001031726	543	659	815	758
C19orf12__NM_001031726_CATCTTCCTCTCCCCAGAAA	C19orf12__NM_001031726	702	393	768	732
C19orf12__NM_001031726_CATGGCCTTCGTCGGGGGTT	C19orf12__NM_001031726	680	819	882	1424
C19orf12__NM_001031726_CTAGTCATCATACTGGATCT	C19orf12__NM_001031726	544	290	430	380
[illegible]	[illegible]	[illegible]	[illegible]	[illegible]	[illegible]

C22orf15__NM_182520_CATGGGCAACTCCCTACTGA	C22orf15__NM_182520	1279	767	1109	642
C22orf15__NM_182520_CCAGGAGAGCAATGGTCGCT	C22orf15__NM_182520	868	631	966	612
C22orf15__NM_182520_CCTTACTGATGATCCGAACG	C22orf15__NM_182520	618	732	722	554
C22orf15__NM_182520_CCTTTCTGGGCCTTGAAGTG	C22orf15__NM_182520	115	163	151	108
C22orf15__NM_182520_CTCCAATAGGGACTCATAGC	C22orf15__NM_182520	8	61	26	13
C22orf15__NM_182520_GAGCCATATATGTCCTCGTT	C22orf15__NM_182520	1024	1035	1158	1489
C22orf15__NM_182520_GATCCGAACGAGGACATATA	C22orf15__NM_182520	245	258	276	200
C22orf15__NM_182520_GCCAGGAGAGCAATGGTCGC	C22orf15__NM_182520	606	387	789	547
C22orf15__NM_182520_GGAGGAAGCGTATGGGCACT	C22orf15__NM_182520	1215	1070	1035	945
C22orf15__NM_182520_TCTCCAATAGGGACTCATAG	C22orf15__NM_182520	80	97	100	83
C22orf23__NM_001207062_AGACCATCACTTACACCCCG	C22orf23__NM_001207062	2675	1818	2716	2255
C22orf23__NM_001207062_AGGAGTGAGGAACTTAGGAA	C22orf23__NM_001207062	164	154	200	228
C22orf23__NM_001207062_AGGATGGGAGGCAGGTAGAT	C22orf23__NM_001207062	161	94	169	191
C22orf23__NM_001207062_CAGATGGAGGTAGTGACCAA	C22orf23__NM_001207062	332	253	334	299
C22orf23__NM_001207062_CCCATTGGCTTGACACATGT	C22orf23__NM_001207062	2667	3276	2797	4029
C22orf23__NM_001207062_GAGCCTTCTGTCGTGCAGGA	C22orf23__NM_001207062	383	534	374	309
C22orf23__NM_001207062_GCTGGAGCCTTCTGTCGTGC	C22orf23__NM_001207062	911	1424	1061	1288
C22orf23__NM_001207062_GGACAGGGCAAACAGTACCG	C22orf23__NM_001207062	670	532	607	404
C22orf23__NM_001207062_GGAGCCTTCTGTCGTGCAGG	C22orf23__NM_001207062	763	850	866	770
C22orf23__NM_001207062_TCTTCAAATCGGTCTAGCTC	C22orf23__NM_001207062	0	0	0	0
C22orf23__NM_032561_CTGCTGGATGTTCGTCAGTT	C22orf23__NM_032561	1424	1526	1549	1693
C22orf24__NM_015372_ACAACTCAAGAGGATACAAC	C22orf24__NM_015372	185	161	144	118
C22orf24__NM_015372_CAACTCAAGAGGATACAACA	C22orf24__NM_015372	331	343	375	423
C22orf24__NM_015372_CCCACAGCCTGCTATGAAGT	C22orf24__NM_015372	1052	1089	1285	1210
C22orf24__NM_015372_CCTACTTCATAGCAGGCTGT	C22orf24__NM_015372	309	284	465	364
C22orf24__NM_015372_CTTCGCTCCAGGTCTTGACA	C22orf24__NM_015372	40	82	93	96
C22orf24__NM_015372_GAGAAGATGTTGCTAATGTA	C22orf24__NM_015372	272	258	232	316
C22orf24__NM_015372_GTCAAGACCTGGAGCGAAGA	C22orf24__NM_015372	244	271	178	189
C22orf24__NM_015372_TTGCTAATGTAAGGTGTGGC	C22orf24__NM_015372	231	402	367	466
C22orf29__NM_024627_AAAATCTCTGGGTATTTCCA	C22orf29__NM_024627	33	62	78	42
C22orf29__NM_024627_AACAGCGCAGCGGCTACAGT	C22orf29__NM_024627	608	537	515	318
C22orf29__NM_024627_AAGGACCCTCAGCTCATGCG	C22orf29__NM_024627	182	207	122	141
C22orf29__NM_024627_AAGTTTACCACAGGATAGGC	C22orf29__NM_024627	236	177	159	149
C22orf29__NM_024627_AATAGGAAGTACTAAGGATA	C22orf29__NM_024627	115	66	139	188
C22orf29__NM_024627_ACAAGCAGCCACCCAGCGTG	C22orf29__NM_024627	289	320	217	158
C22orf29__NM_024627_ACAGGAACGAGGTCCCAACA	C22orf29__NM_024627	141	164	166	226
C22orf29__NM_024627_ACCCTCAGCTCATGCGGGGA	C22orf29__NM_024627	792	807	865	910
C22orf29__NM_024627_ACTACTTGCTAGGTGCATGA	C22orf29__NM_024627	350	446	479	464
C22orf29__NM_024627_ACTGTAGCTCTAATCGTGCC	C22orf29__NM_024627	67	28	48	46
C22orf29__NM_024627_AGAACTGCTGGTAATCCCAC	C22orf29__NM_024627	492	438	546	429
C22orf29__NM_024627_AGAAGCGGTCCAGTAGCCAC	C22orf29__NM_024627	346	248	364	254
C22orf29__NM_024627_AGTGGCCACTTTGATTCTGG	C22orf29__NM_024627	1459	1209	1400	770
C22orf29__NM_024627_ATCCACTTCAGGTCCACTAA	C22orf29__NM_024627	304	516	406	313
C22orf29__NM_024627_ATCCGCAATGCATGCGAGTG	C22orf29__NM_024627	145	130	188	205
C22orf29__NM_024627_ATGGGGCCTGAGTCATCGGA	C22orf29__NM_024627	808	855	977	513
C22orf29__NM_024627_ATGGTCTGCCGTGTGTCTGA	C22orf29__NM_024627	271	508	332	196
C22orf29__NM_024627_ATGGTTCGGTACCACTACAT	C22orf29__NM_024627	20	48	59	55
C22orf29__NM_024627_ATGTACTGGCATAAAGAGTG	C22orf29__NM_024627	230	338	232	163
C22orf29__NM_024627_ATGTGCCTAGAATTGTTACG	C22orf29__NM_024627	561	348	642	521
C22orf29__NM_024627_ATGTGCGTTGGCATAATTGG	C22orf29__NM_024627	516	703	605	594
C22orf29__NM_024627_ATTATGCCAACGCACATCCA	C22orf29__NM_024627	329	471	403	331
C22orf29__NM_024627_CAGACACAGAAACCCCATAC	C22orf29__NM_024627	735	599	770	592
C22orf29__NM_024627_CAGCAGGGCCGCTCAATCCA	C22orf29__NM_024627	458	342	336	360
C22orf29__NM_024627_CAGCTGATCATCCTATGGGC	C22orf29__NM_024627	926	915	1091	991
C22orf29__NM_024627_CATGGTCGTTCGCACACACA	C22orf29__NM_024627	758	912	744	635
C22orf29__NM_024627_CCACACACTGGCAGACCCGA	C22orf29__NM_024627	861	904	993	919
C22orf29__NM_024627_CCCAAAGCCCCAATAGGACA	C22orf29__NM_024627	1030	1140	1043	1091
C22orf29__NM_024627_CCCATGAGGTGGGTGTTAGA	C22orf29__NM_024627	219	132	235	163
C22orf29__NM_024627_CCCCAAAGCCCCAATAGGAC	C22orf29__NM_024627	645	910	788	682
C22orf29__NM_024627_CGTGTGTGTGCGAACGACCA	C22orf29__NM_024627	899	803	926	780
C22orf29__NM_024627_CTACAAAACGACCCCCTCGT	C22orf29__NM_024627	1586	933	1549	803
C22orf29__NM_024627_CTCAATCCAGGGATCCACAA	C22orf29__NM_024627	127	180	141	146
C22orf29__NM_024627_CTCACTAGTCTATTGGATCA	C22orf29__NM_024627	524	598	590	758
C22orf29__NM_024627_CTCCGCACTCGCATGCATTG	C22orf29__NM_024627	410	577	550	515
C22orf29__NM_024627_CTGCCCAATATAGCGCTGCC	C22orf29__NM_024627	322	377	341	494
C22orf29__NM_024627_CTGCTCATCACAGGCTATCA	C22orf29__NM_024627	417	510	440	524
C22orf29__NM_024627_CTGTGGCCTGGCTCACCTGT	C22orf29__NM_024627	1173	1121	1107	822
C22orf29__NM_024627_CTTCCACTTTGAGCACTATC	C22orf29__NM_024627	458	629	392	460
C22orf29__NM_024627_GAAAAAGCAGAAATGTGAAT	C22orf29__NM_024627	358	599	503	645
C22orf29__NM_024627_GAAGCGGTCCAGTAGCCACG	C22orf29__NM_024627	711	504	641	536
C22orf29__NM_024627_GACTCAAGGTTGAATGTCAA	C22orf29__NM_024627	122	203	253	301
C22orf29__NM_024627_GAGATCCTCAGGCGCCTAAC	C22orf29__NM_024627	331	309	326	312
C22orf29__NM_024627_GAGCATGGAGAAATATGTCC	C22orf29__NM_024627	503	373	398	186
C22orf29__NM_024627_GATAAGGTCCTATGGGATAT	C22orf29__NM_024627	514	627	657	637
C22orf29__NM_024627_GATCAGCTGCTGCCGTCCTA	C22orf29__NM_024627	916	634	933	559
C22orf29__NM_024627_GCAGCAGCTGATCATCCTAT	C22orf29__NM_024627	899	619	834	503
C22orf29__NM_024627_GCATGCTGTGACTACTTGCT	C22orf29__NM_024627	1925	2320	2312	2319
C22orf29__NM_024627_GCCTGAGGATCTCGCAGACA	C22orf29__NM_024627	1231	1111	1270	701
C22orf29__NM_024627_GCTCACTTGCTTTGCTACCA	C22orf29__NM_024627	731	733	842	726
C22orf29__NM_024627_GCTGCCGTCCTATGGAACTC	C22orf29__NM_024627	700	453	624	431
C22orf29__NM_024627_GGAAATTTAGTACAGGCTAG	C22orf29__NM_024627	290	364	268	389
C22orf29__NM_024627_GGCAGCAGCTGATCATCCTA	C22orf29__NM_024627	2231	1894	2468	1847
C22orf29__NM_024627_GGGCCTGAGTCATCGGACGG	C22orf29__NM_024627	625	990	826	993
C22orf29__NM_024627_GGGCGGAGGTCAAGCTCTGC	C22orf29__NM_024627	167	232	125	218
C22orf29__NM_024627_GGTCTTTAAAGAGGTAATTC	C22orf29__NM_024627	104	148	103	101
C22orf29__NM_024627_GGTGCTCATTAAACACGTGT	C22orf29__NM_024627	0	0	0	0
C22orf29__NM_024627_GTACTAAGGATAAGGTCCTA	C22orf29__NM_024627	277	190	295	230
C22orf29__NM_024627_GTCTTCAAATGTAAGTACTA	C22orf29__NM_024627	149	91	117	48
C22orf29__NM_024627_GTTCCTGGCAGCGCTATATT	C22orf29__NM_024627	359	448	356	275
C22orf29__NM_024627_GTTGGGACCTCGTTCCTGTA	C22orf29__NM_024627	262	257	333	286
C22orf29__NM_024627_TAAGAAATCTTTCCCTACCT	C22orf29__NM_024627	367	191	355	144
C22orf29__NM_024627_TACTAAGGATAAGGTCCTAT	C22orf29__NM_024627	464	324	532	344
C22orf29__NM_024627_TATTACATGTGCATCGCGGT	C22orf29__NM_024627	131	124	240	179
C22orf29__NM_024627_TCAGACCCAGGCACCTTTGA	C22orf29__NM_024627	1271	1202	1451	1219
C22orf29__NM_024627_TCATAAGCTCACTAGTCTAT	C22orf29__NM_024627	465	547	634	590
C22orf29__NM_024627_TCCACCTGTAAGTGTAACTG	C22orf29__NM_024627	407	249	429	322
C22orf29__NM_024627_TCCACTTCAGGTCCACTAAA	C22orf29__NM_024627	372	245	284	250
C22orf29__NM_024627_TCCTGAAGATAGGACATGAT	C22orf29__NM_024627	698	504	657	673
C22orf29__NM_024627_TCCTGGGGGTGTGTGAGTTA	C22orf29__NM_024627	703	682	731	583
C22orf29__NM_024627_TCTAAGTGCTTTAACTATTG	C22orf29__NM_024627	151	208	210	306
C22orf29__NM_024627_TCTTCAAATGTAAGTACTAG	C22orf29__NM_024627	116	202	156	255
C22orf29__NM_024627_TGCCCAGAGTTGTGGAGGTA	C22orf29__NM_024627	853	833	789	854
C22orf29__NM_024627_TGCCTATCCTGTGGTAAACT	C22orf29__NM_024627	671	629	699	554
C22orf29__NM_024627_TGGTTCGGTACCACTACATG	C22orf29__NM_024627	0	0	0	0
C22orf29__NM_024627_TGTCTTCAAATGTAAGTACT	C22orf29__NM_024627	89	125	96	65
C22orf29__NM_024627_TTAGAAAGCAGGTATAGCCC	C22orf29__NM_024627	39	30	8	11
C22orf29__NM_024627_TTCCTGAAGATAGGACATGA	C22orf29__NM_024627	22	12	38	35
C22orf29__NM_024627_TTGCCTTGCTCCTTTATTGC	C22orf29__NM_024627	711	744	730	899
C22orf29__NM_024627_TTGGGACCTCGTTCCTGTAT	C22orf29__NM_024627	256	149	312	243
C22orf31__NM_015370_AAGCCTGGTATTTAATAAGA	C22orf31__NM_015370	88	75	126	108
C22orf31__NM_015370_ACTGTCCTGGGGTAGCGCTT	C22orf31__NM_015370	482	683	413	541
C22orf31__NM_015370_ACTGTCGGAGTCCATAGATA	C22orf31__NM_015370	209	182	257	124
C22orf31__NM_015370_AGAAGGAACTGGCAATTAAT	C22orf31__NM_015370	285	227	272	226
C22orf31__NM_015370_CCTGAGTACAAGCTTAACCA	C22orf31__NM_015370	952	1081	926	911
C22orf31__NM_015370_GACCCCAGCATCCCTATCTA	C22orf31__NM_015370	1091	1090	1231	863
C22orf31__NM_015370_GACTGTCGGAGTCCATAGAT	C22orf31__NM_015370	464	319	567	425
C22orf31__NM_015370_GCCTGAGTACAAGCTTAACC	C22orf31__NM_015370	695	855	727	541
C22orf31__NM_015370_GGGTTTCAGGAAGCAACACT	C22orf31__NM_015370	272	380	212	219
C22orf31__NM_015370_GGTCTCGTCTCACATTGATT	C22orf31__NM_015370	771	760	876	920
C22orf31__NM_015370_GTCCTGGGGTAGCGCTTGGG	C22orf31__NM_015370	352	273	431	473
C22orf31__NM_015370_TGGTTAAGCTTGTACTCAGG	C22orf31__NM_015370	36	18	10	15
C22orf34__NM_001289922_CCCTGAAAGCACCTCGGTCC	C22orf34__NM_001289922	1452	1361	1732	1869
C22orf34__NM_001289922_CCTGAAAGCACCTCGGTCCA	C22orf34__NM_001289922	1126	1023	1252	1295
C22orf34__NM_001289922_CTGAAAGCACCTCGGTCCAG	C22orf34__NM_001289922	566	416	651	536
C22orf34__NM_001289922_CTTGTGCCCTGAAAGCACCT	C22orf34__NM_001289922	635	550	658	594
C22orf34__NM_001289922_GGATTGGGGACAATCCTGCG	C22orf34__NM_001289922	180	69	174	111
C22orf34__NM_001289922_GGCAAGGGGCCTGGATAGAA	C22orf34__NM_001289922	680	576	718	618
C22orf34__NM_001289922_TGGCAAGGGGCCTGGATAGA	C22orf34__NM_001289922	49	52	58	63
C22orf39__NM_001166242_AGCGGCCGGCCTGCGAACAG	C22orf39__NM_001166242	1205	1171	1323	1201
C22orf39__NM_001166242_CCTGGCCAGCTGCCGCGACT	C22orf39__NM_001166242	1388	1026	1149	827
C22orf39__NM_001166242_CGCTGCCACTGTTCGCAGGC	C22orf39__NM_001166242	693	822	807	570
C22orf39__NM_001166242_CGCTGCCGTCCGCCATGTCT	C22orf39__NM_001166242	1924	1409	1847	1433
C22orf39__NM_001166242_CTACACCACTACTACGTCCA	C22orf39__NM_001166242	788	888	854	762
C22orf39__NM_001166242_GAGCTTCCACTCGGCGCGGT	C22orf39__NM_001166242	883	678	870	617
C22orf39__NM_001166242_GCTCGCCGTGGACGTAGTAG	C22orf39__NM_001166242	414	245	407	387
C22orf39__NM_001166242_GTAGTGGTGTAGGAAGTGCC	C22orf39__NM_001166242	101	120	97	44
C22orf39__NM_001166242_GTCCGCCATGTCTGGGCGAC	C22orf39__NM_001166242	431	537	518	497
C22orf39__NM_001166242_TCATTGGCTGGCGGACTGAG	C22orf39__NM_001166242	273	291	449	350
C22orf39__NM_001166242_TTCATTGGCTGGCGGACTGA	C22orf39__NM_001166242	39	17	38	62
C22orf39__NM_173793_GAGCGAGCGGGCACGAGTCC	C22orf39__NM_173793	684	585	475	285
C22orf42__NM_001010859_CACTGCAAAGCCTGCCAAAT	C22orf42__NM_001010859	297	498	324	475
C22orf42__NM_001010859_CTTGAAGACTTCATGACATC	C22orf42__NM_001010859	358	688	448	671
C22orf42__NM_001010859_GCTGAGGTACTGCATGAGTT	C22orf42__NM_001010859	855	652	839	856
C22orf42__NM_001010859_GGAATCTCACACTCATGGCA	C22orf42__NM_001010859	434	520	409	533
C22orf42__NM_001010859_GGCTGAGGTACTGCATGAGT	C22orf42__NM_001010859	1161	1123	1155	816
C22orf42__NM_001010859_TATGGAGACGGCACGGGATT	C22orf42__NM_001010859	508	226	575	230
C22orf42__NM_001010859_TGAGATCTTCGACCAAATCT	C22orf42__NM_001010859	119	125	228	90
C22orf46__NM_001142964_ACTGTTCTGAGGCAGTAAAC	C22orf46__NM_001142964	193	126	144	147
C22orf46__NM_001142964_CTTGGACATGTGTCAAGAGA	C22orf46__NM_001142964	271	235	349	387
C22orf46__NM_001142964_GCCATGTTGTTGTCACTGCT	C22orf46__NM_001142964	1063	1127	1457	1193
C22orf46__NM_001142964_TGGAGCCTGTCATCAGTGTG	C22orf46__NM_001142964	223	247	243	313
C2CD2__NM_015500_ACTTGGCATTCAACTCGCTT	C2CD2__NM_015500	864	839	1027	1004
C2CD2__NM_015500_AGAGCCGCATGTCGTACAGC	C2CD2__NM_015500	445	377	427	433
C2CD2__NM_015500_ATATCCAGCCCAAAGCACTG	C2CD2__NM_015500	253	292	351	350
C2CD2__NM_015500_CAAGGACATCTTGAAGCATT	C2CD2__NM_015500	627	483	616	852
C2CD2__NM_015500_CCTGTCCTCCTAAACCTCCA	C2CD2__NM_015500	983	1399	1223	1409
C2CD2__NM_015500_CGACGCACACTGCATTAATG	C2CD2__NM_015500	1097	1366	1559	1822
C2CD2__NM_015500_GAGAGGACCTCAGATTAGC	C2CD2__NM_015500	202	108	197	117
C2CD2__NM_015500_GTGTGCTGCATCTACTGCTC	C2CD2__NM_015500	108	175	193	186
C2CD2__NM_015500_TCCTCTTCCCACATGAGGTC	C2CD2__NM_015500	123	207	117	76
C2CD2__NM_015500_TGACCAAGAACTGAATAGCC	C2CD2__NM_015500	29	4	48	80
C2CD2__NM_015500_TGAGCTTCCTTTACAGTCGT	C2CD2__NM_015500	710	562	889	808
C2CD2__NM_015500_TGATGAAAGACCAGCTAATC	C2CD2__NM_015500	89	40	94	156
C2CD2__NM_199050_ACCAAGCCTCGCGTCGACGT	C2CD2__NM_199050	4294	4413	4585	4062
C2CD2__NM_199050_CGAGTTGAATGCCAAGTCGA	C2CD2__NM_199050	526	640	643	668
C2CD2L__NM_001290474_AATCTGAGCATACCATGGTA	C2CD2L__NM_001290474	241	235	339	262
C2CD2L__NM_001290474_ATGCTCAGATTGGTCTACGC	C2CD2L__NM_001290474	357	373	613	628
C2CD2L__NM_001290474_CAGTGTCAGAGTGACGTGGT	C2CD2L__NM_001290474	1897	2196	2187	2260
C2CD2L__NM_001290474_CCTGGCTGCTGCAATATGCC	C2CD2L__NM_001290474	411	418	539	636
C2CD2L__NM_001290474_CGCTGCTCACGGTGTTCGCC	C2CD2L__NM_001290474	179	248	214	258
C2CD2L__NM_001290474_CTCCATCCAAATCGCCTTTG	C2CD2L__NM_001290474	321	606	379	525
C2CD2L__NM_001290474_CTCCTCAAAGGCGATTTGGA	C2CD2L__NM_001290474	171	178	175	255
C2CD2L__NM_001290474_CTGGCTGCTGCAATATGCCC	C2CD2L__NM_001290474	459	393	541	342
C2CD2L__NM_001290474_GAGATCTGGGCGATCAGTGA	C2CD2L__NM_001290474	420	410	325	293
C2CD2L__NM_001290474_GCACCTCCTCAAAGGCGATT	C2CD2L__NM_001290474	515	417	584	808
C2CD2L__NM_001290474_TGCTGCGGCTGCGGGCGACT	C2CD2L__NM_001290474	508	483	836	895
C2CD2L__NM_001290474_TGGCTGCTGCAATATGCCCG	C2CD2L__NM_001290474	95	84	108	54
C2CD3__NM_001286577_AAACTTGATGGTCTTCCAAT	C2CD3__NM_001286577	305	180	277	218
C2CD3__NM_001286577_ACACGTTACGCTATTCGTTG	C2CD3__NM_001286577	865	901	1051	1147
C2CD3__NM_001286577_AGCCGTGGGCGCAAAAAAAG	C2CD3__NM_001286577	34	103	7	23
C2CD3__NM_001286577_ATTGGTAGAGTTCAGATCAA	C2CD3__NM_001286577	154	307	277	300
C2CD3__NM_001286577_CAGTTTACCTCTTATCTAAC	C2CD3__NM_001286577	196	259	350	418
C2CD3__NM_001286577_CGGACAAGTACACAAGTGGG	C2CD3__NM_001286577	452	451	484	434
C2CD3__NM_001286577_CTCATCATGAAACAACGAAA	C2CD3__NM_001286577	76	30	92	78
C2CD3__NM_001286577_GAAGTAATCACCAAACTTGA	C2CD3__NM_001286577	472	627	454	442
C2CD3__NM_001286577_GTGTACTTGTCCGAGTGAGA	C2CD3__NM_001286577	455	422	380	358
C2CD3__NM_001286577_TCATCAACGTCTAAGAAACT	C2CD3__NM_001286577	1036	1378	1258	1458
C2CD3__NM_001286577_TTGATCTGAACTCTACCAAT	C2CD3__NM_001286577	130	91	214	186
C2CD3__NM_001286577_TTTCTCCCCACCATCTCACT	C2CD3__NM_001286577	202	215	227	195
C2CD4A__NM_207322_AGTGAGCACATTTGCGCACG	C2CD4A__NM_207322	275	308	328	224
C2CD4A__NM_207322_AGTGCTGCATTGCCGAAGCG	C2CD4A__NM_207322	307	405	359	397
C2CD4A__NM_207322_CCCCGTGTGCGCACCGCCTA	C2CD4A__NM_207322	963	1036	1220	1325
C2CD4A__NM_207322_CCGCCTGGCCGTTCGAGTCA	C2CD4A__NM_207322	529	807	585	737
C2CD4A__NM_207322_CGCGGCGGTGGTGCGAGACT	C2CD4A__NM_207322	422	556	498	397
C2CD4A__NM_207322_CTGAGTTCTGCATCCCGCCA	C2CD4A__NM_207322	549	735	776	688
C2CD4A__NM_207322_GCCTCCAGGCGCTCGGGAAA	C2CD4A__NM_207322	371	284	475	399
C2CD4A__NM_207322_GGGCCTTGACTCGAACGGCC	C2CD4A__NM_207322	212	591	371	303
C2CD4A__NM_207322_TGGCCGTTCGAGTCAAGGCC	C2CD4A__NM_207322	53	131	62	48
C2CD4B__NM_001007595_ATCCCGGGCCTTGACGCGAA	C2CD4B__NM_001007595	899	1271	1118	1101
C2CD4B__NM_001007595_ATTGGAGACTCGAGCGTGCA	C2CD4B__NM_001007595	301	194	354	423
C2CD4B__NM_001007595_CAGAATTCGGGGATGCGATT	C2CD4B__NM_001007595	337	296	484	562
C2CD4B__NM_001007595_GCACGCTCGAGTCTCCAATC	C2CD4B__NM_001007595	1369	1083	1220	1280
C2CD4B__NM_001007595_GGCGCACCTCGTCTTCCGAG	C2CD4B__NM_001007595	678	792	621	558
C2CD4B__NM_001007595_GGGCCACAGGTCGCTTTCAG	C2CD4B__NM_001007595	790	1082	866	706
C2CD4B__NM_001007595_TGAGGTCGCGGCCGGGATC	C2CD4B__NM_001007595	414	487	501	539
C2CD4B__NM_001007595_TTGGAGACTCGAGCGTGCAA	C2CD4B__NM_001007595	248	265	292	379
C2CD4C__NM_001136263_AGACTCCTTGCGCCTCGTGT	C2CD4C__NM_001136263	1150	961	1604	1452
C2CD4C__NM_001136263_CCATGAGAAAAACCAACATG	C2CD4C__NM_001136263	119	72	173	145
C2CD4C__NM_001136263_GACTCCTTGCGCCTCGTGTG	C2CD4C__NM_001136263	1184	1471	1293	1483
C2CD4C__NM_001136263_GAGACTCCTTGCGCCTCGTG	C2CD4C__NM_001136263	1149	1408	1454	2120
C2CD4C__NM_001136263_GAGCCCCCACACGAGGCGCA	C2CD4C__NM_001136263	504	588	583	628
C2CD4C__NM_001136263_GCAGCTGCCAAGGCCAACGG	C2CD4C__NM_001136263	334	321	426	324
C2CD4C__NM_001136263_GGCCAGGTTCTGTTCCGACG	C2CD4C__NM_001136263	664	825	669	644
C2CD4C__NM_001136263_GGCGAAGCCGTAGGACGTCT	C2CD4C__NM_001136263	621	712	744	954
C2CD4C__NM_001136263_GTCAACACATTGCTGTACAG	C2CD4C__NM_001136263	426	200	523	490
C2CD4C__NM_001136263_GTCTGGGCCTTGGCACCGA	C2CD4C__NM_001136263	206	270	245	202
C2CD4C__NM_001136263_TGGGGGGGATGAAAAAGTCG	C2CD4C__NM_001136263	74	150	78	71
C2CD4C__NM_001136263_TTGGGGGGGATGAAAAAGTC	C2CD4C__NM_001136263	129	255	127	94
C2CD4D__NM_001136003_AAGAAATCCTCGTTGAAGAT	C2CD4D__NM_001136003	323	212	330	336
C2CD4D__NM_001136003_CAGGCCGGAAGGCGCCCAAC	C2CD4D__NM_001136003	105	22	76	59
C2CD4D__NM_001136003_CCCGGGCCCGGCCTGATATT	C2CD4D__NM_001136003	649	446	720	341
C2CD4D__NM_001136003_CGATCCGGGGTGAGGACGTT	C2CD4D__NM_001136003	1283	1168	1301	1293
C2CD4D__NM_001136003_CGCTTGGAGAACAGGCCGGA	C2CD4D__NM_001136003	522	578	614	622
C2CD4D__NM_001136003_CTTGGAAAAAGCTGGCTATA	C2CD4D__NM_001136003	136	136	129	219
C2CD4D__NM_001136003_GAAAAAGCTGGCTATAAGGT	C2CD4D__NM_001136003	224	134	249	110
C2CD4D__NM_001136003_GAACTGCGGGATGCGATCGG	C2CD4D__NM_001136003	746	678	741	608
C2CD4D__NM_001136003_GCGATCCGGGGTGAGGACGT	C2CD4D__NM_001136003	819	628	697	338
C2CD4D__NM_001136003_GCGGCTCTCCACCGAATATC	C2CD4D__NM_001136003	118	79	104	68
C2CD4D__NM_001136003_TGGCGCTGCACTTGACCACG	C2CD4D__NM_001136003	221	222	255	200
C2CD4D__NM_001136003_TTGGGGCAGGCGCTTGTGGG	C2CD4D__NM_001136003	866	422	892	665
C2CD5__NM_001286173_ACTGAGGGTTGAGTGACTTA	C2CD5__NM_001286173	464	513	587	703
C2CD5__NM_001286173_AGACTCATTTCGTTAATGTC	C2CD5__NM_001286173	501	440	450	588
C2CD5__NM_001286173_CACACCAAGGGCATCAAATG	C2CD5__NM_001286173	662	767	723	962
C2CD5__NM_001286173_CATCACTGGCAAATGGCGCC	C2CD5__NM_001286173	400	406	563	795
C2CD5__NM_001286173_CATCAGTCAGGTCACTAGCA	C2CD5__NM_001286173	224	419	251	392
C2CD5__NM_001286173_GCAGCTTCACTATACAGTAA	C2CD5__NM_001286173	459	512	581	644
C2CD5__NM_001286173_TATAGAGCTGTGATAATTCA	C2CD5__NM_001286173	180	228	216	182
C2CD5__NM_001286173_TCAAGAACTGTGATCTGTAA	C2CD5__NM_001286173	481	525	615	742
C2CD5__NM_001286173_TCACTAGCACGGTCCATCAC	C2CD5__NM_001286173	777	767	867	1104
C2CD5__NM_001286173_TGACCTGACTGATGCCTTCG	C2CD5__NM_001286173	693	777	874	967
C2CD5__NM_001286173_TGATCGAATTCGCACACCAA	C2CD5__NM_001286173	245	443	310	293
C2CD5__NM_001286173_TTGATCGAATTCGCACACCA	C2CD5__NM_001286173	341	459	397	472
C2orf15__NM_144706_AACACTTCCACTACTTAAAA	C2orf15__NM_144706	154	244	214	303
C2orf15__NM_144706_ATTTGTTCAGCTATCCTAAT	C2orf15__NM_144706	135	142	147	179
C2orf15__NM_144706_CATTTGTTCAGCTATCCTAA	C2orf15__NM_144706	596	537	551	820
C2orf15__NM_144706_GAAAACTTTACAAGGATTGA	C2orf15__NM_144706	83	161	116	98
C2orf15__NM_144706_GTGGATGATCACTTAATACG	C2orf15__NM_144706	74	18	83	121
C2orf15__NM_144706_TGGATGATCACTTAATACGA	C2orf15__NM_144706	19	12	56	28
C2orf15__NM_144706_TTTACAAGGATTGAAGGGAC	C2orf15__NM_144706	39	17	57	74
C2orf16__NM_032266_AAATCTGTGGAATTAGCACC	C2orf16__NM_032266	216	416	304	339
C2orf16__NM_032266_AATCTGACACTAGTCAACAG	C2orf16__NM_032266	231	308	232	283
C2orf16__NM_032266_ACTCCAATGGTAAGGGATCA	C2orf16__NM_032266	244	289	241	276
C2orf16__NM_032266_CTAGTCAACAGTGGTCCTGA	C2orf16__NM_032266	164	199	176	98
C2orf16__NM_032266_CTGAACTAATCCCTAGACCA	C2orf16__NM_032266	615	880	601	747
C2orf16__NM_032266_GAGCCTTGATCCCTTACCAT	C2orf16__NM_032266	668	787	787	915
C2orf16__NM_032266_GATTCTGTGGCGTGATAAAT	C2orf16__NM_032266	84	63	89	110
C2orf16__NM_032266_GATTTAGGGATTTGATACTT	C2orf16__NM_032266	356	361	403	474
C2orf16__NM_032266_GCACAAGGATTGGCATATAA	C2orf16__NM_032266	137	173	131	129
C2orf16__NM_032266_GCCTCAAAGAGTGAAATATG	C2orf16__NM_032266	510	425	520	570
C2orf16__NM_032266_TGCCTCAAAGAGTGAAATAT	C2orf16__NM_032266	110	114	103	97
C2orf16__NM_032266_TGGTCTAGGGATTAGTTCAG	C2orf16__NM_032266	0	0	1	14
C2orf40__NM_032411_ACGGCCACTTTAGTCTTAGT	C2orf40__NM_032411	1317	1200	1582	1358
C2orf40__NM_032411_ACTTTAGTCTTAGTTGGAAC	C2orf40__NM_032411	400	429	494	803
C2orf40__NM_032411_AGCCCCTACGGCTTTAGGCA	C2orf40__NM_032411	410	525	489	516
C2orf40__NM_032411_AGTCTTCATCATAGTGACGT	C2orf40__NM_032411	172	286	276	188
C2orf40__NM_032411_CGAAATGGACATGAATACTA	C2orf40__NM_032411	716	706	719	612
C2orf40__NM_032411_CTACATGGGCTTTGACGAAG	C2orf40__NM_032411	252	229	310	304
C2orf40__NM_032411_GCTCCATGCCTAAAGCCGTA	C2orf40__NM_032411	416	598	587	702
C2orf40__NM_032411_GGCTCCATGCCTAAAGCCGT	C2orf40__NM_032411	1128	925	1288	1151
C2orf40__NM_032411_TCGATCTCTGTTAAGCCAAT	C2orf40__NM_032411	229	258	237	346
C2orf40__NM_032411_TGGCTTAACAGAGATCGAAA	C2orf40__NM_032411	32	34	42	36
C2orf42__NM_017880_AGCTGCCACTCAAGGCGTTG	C2orf42__NM_017880	474	778	563	671
C2orf42__NM_017880_AGCTGTAAGAACAAGACATG	C2orf42__NM_017880	367	195	351	297
C2orf42__NM_017880_CCACAAAGCATCGGTAATCA	C2orf42__NM_017880	1198	2164	1480	2016
C2orf42__NM_017880_CCATTGTATGTGCCACATCG	C2orf42__NM_017880	603	708	555	760
C2orf42__NM_017880_CCCAGCTTTCTTATCTGATT	C2orf42__NM_017880	976	1301	1107	1575
C2orf42__NM_017880_CCCCGAGCTCCACAAAGCAT	C2orf42__NM_017880	427	405	475	644
C2orf42__NM_017880_GCGTGCACCGTAGCGGAATA	C2orf42__NM_017880	501	453	536	681
C2orf42__NM_017880_GTGGCAGCTTTCAGGCATGA	C2orf42__NM_017880	135	129	130	148
C2orf42__NM_017880_TCAGTGCGGCAAAGAGACCG	C2orf42__NM_017880	596	500	660	532
C2orf42__NM_017880_TCCACAAAGCATCGGTAATC	C2orf42__NM_017880	378	562	483	538
C2orf42__NM_017880_TCCATTGTATGTGCCACATC	C2orf42__NM_017880	424	162	333	316

[illegible]

C7orf57__NM_001100159_TCTTCATGAACCATGTAATC	C7orf57__NM_001100159	487	584	492	561
C7orf57__NM_001100159_TGCCTGGGACTCGGAGATAC	C7orf57__NM_001100159	261	173	288	282
C7orf57__NM_001100159_TGTCTCCATGCCGGATTACA	C7orf57__NM_001100159	237	200	262	355
C7orf57__NM_001100159_TTCCTTGCTTGTGTTCCTCA	C7orf57__NM_001100159	109	189	107	119
C7orf57__NM_001267865_AGCGAGCTGACTCAGACAAG	C7orf57__NM_001267865	269	237	296	351
C7orf57__NM_001267865_ATCTCAGTCCCCACGAGACC	C7orf57__NM_001267865	481	539	425	452
C7orf57__NM_001267865_GCAGGATTCTTAGGGCCAAG	C7orf57__NM_001267865	276	253	249	380
C7orf57__NM_001267865_GGGGACCTTCCAGGTCTCGT	C7orf57__NM_001267865	75	101	91	78
C7orf60__NM_152556_AGAAGACTCATGCATATTTA	C7orf60__NM_152556	230	296	252	267
C7orf60__NM_152556_AGGGCGAAGGTCGTATTGAA	C7orf60__NM_152556	333	420	400	328
C7orf60__NM_152556_ATCCATCGCTGGTAAGGAGA	C7orf60__NM_152556	459	607	450	557
C7orf60__NM_152556_CATGAACTGTTAGTGTTAAA	C7orf60__NM_152556	514	691	659	674
C7orf60__NM_152556_CTCCAGGAAGAGAATCAATA	C7orf60__NM_152556	209	181	161	195
C7orf60__NM_152556_GATCTGGCGAGAACACTGTG	C7orf60__NM_152556	295	125	340	268
C7orf60__NM_152556_GTCGTATTGAATGGTGTTGT	C7orf60__NM_152556	678	539	802	908
C7orf60__NM_152556_GTTGCTGAAGCTGTAAGTTC	C7orf60__NM_152556	369	538	490	574
C7orf60__NM_152556_TAAGGCTGGAGTGGTCTTAG	C7orf60__NM_152556	398	377	327	519
C7orf60__NM_152556_TCCCGGGGCGGCGGAGTGTT	C7orf60__NM_152556	430	627	576	688
C7orf60__NM_152556_TGCCGTTGCAATGAAAAATT	C7orf60__NM_152556	99	134	244	291
C7orf60__NM_152556_TTCCATCTCCTTACCAGCGA	C7orf60__NM_152556	33	30	53	51
C7orf61__NM_001004323_ACTGAGCCGGGTTGGGTCGG	C7orf61__NM_001004323	1359	1656	1642	1764
C7orf61__NM_001004323_GATGCCCAAAGTCCCCTCTA	C7orf61__NM_001004323	1518	1745	1629	1580
C7orf61__NM_001004323_GCCAAGCCCGTCTAACCCAT	C7orf61__NM_001004323	555	435	633	511
C7orf61__NM_001004323_GGAGGCTGAGTTGCTGTGGG	C7orf61__NM_001004323	1379	1785	1475	1927
C7orf61__NM_001004323_GTCGGGGGGCAGCATGGCAT	C7orf61__NM_001004323	0	0	0	0
C7orf62__NM_152706_AAAGTAGGCACTAAAGGACC	C7orf62__NM_152706	177	166	165	207
C7orf62__NM_152706_AACTTTGTAGAGAGTGTCGC	C7orf62__NM_152706	147	348	215	181
C7orf62__NM_152706_ACAGTCACAGTCCCTAAAGG	C7orf62__NM_152706	337	225	331	282
C7orf62__NM_152706_ACATCGTCGAGATACATAAC	C7orf62__NM_152706	331	427	460	395
C7orf62__NM_152706_ACTATGAAAGTAGGCACTAA	C7orf62__NM_152706	240	215	177	222
C7orf62__NM_152706_AGTGCAGGTAATTGCTACGT	C7orf62__NM_152706	3850	4223	4345	3599
C7orf62__NM_152706_ATCTGTGATGACCTCCTTTA	C7orf62__NM_152706	526	750	690	503
C7orf62__NM_152706_CTAGAAACATCCTATCAAGA	C7orf62__NM_152706	632	554	719	800
C7orf62__NM_152706_CTTCTGGTTATGGACCGAAA	C7orf62__NM_152706	172	125	169	119
C7orf62__NM_152706_GACACAGTCACAGTCCCTAA	C7orf62__NM_152706	505	675	582	394
C7orf62__NM_152706_GGACTCGAGGATATGCAGAA	C7orf62__NM_152706	225	345	269	286
C7orf62__NM_152706_TTTCTCATAACATTCCAATG	C7orf62__NM_152706	75	70	129	196
C7orf65__NM_001123065_CAAGCCACCAGCCTTCTCTA	C7orf65__NM_001123065	729	809	653	792
C7orf65__NM_001123065_CTTCCTTCTGTTGACTCTGT	C7orf65__NM_001123065	459	375	409	503
C7orf65__NM_001123065_GGAATCACCCTAGAGAAGGC	C7orf65__NM_001123065	489	588	483	616
C7orf65__NM_001123065_GGGTGATTCCACATGAATTC	C7orf65__NM_001123065	172	147	189	161
C7orf65__NM_001123065_TGCAAGGTGCTATACATAAA	C7orf65__NM_001123065	3	2	21	20
C7orf65__NM_001123065_TTCAAGGCAATCAGTGAGGC	C7orf65__NM_001123065	23	47	11	16
C7orf66__NM_001024607_AAGTGAAAGGTGCATATCTA	C7orf66__NM_001024607	374	453	454	495
C7orf66__NM_001024607_AGCTCAATATCGTCACATGA	C7orf66__NM_001024607	484	696	522	698
C7orf66__NM_001024607_AGTTGAGAAAGACCATCACT	C7orf66__NM_001024607	453	552	479	590
C7orf66__NM_001024607_ATACAGAACTAGAATTCATG	C7orf66__NM_001024607	344	317	451	425
C7orf66__NM_001024607_ATCTCACTCATCAGAGACTT	C7orf66__NM_001024607	552	1016	792	1123
C7orf66__NM_001024607_CTGACATCACTGGATGCAAT	C7orf66__NM_001024607	674	978	804	1088
C7orf66__NM_001024607_TCTGACATCACTGGATGCAA	C7orf66__NM_001024607	301	421	331	435
C7orf66__NM_001024607_TTGAGAAGGCATGACATTTG	C7orf66__NM_001024607	382	400	489	499
C7orf69__NM_025031_GCACAGCCTGTGCCAGTAGG	C7orf69__NM_025031	28	66	76	90
C7orf69__NM_025031_GCTGAAAAGTTGGTCATGGA	C7orf69__NM_025031	750	956	784	861
C7orf69__NM_025031_GGCTGTGCACTTGCACGGAT	C7orf69__NM_025031	526	381	476	552
C7orf69__NM_025031_TCAAATGCAGAGCAAATTGG	C7orf69__NM_025031	356	367	453	433
C7orf69__NM_025031_TGGGTGCTGGTTCTTAACTC	C7orf69__NM_025031	3	0	21	3
C7orf71__NM_001145531_ACAGAGCTTTCTCCGCATGT	C7orf71__NM_001145531	1571	1494	2012	1779
C7orf71__NM_001145531_AGAAGCATCGGGGTGCTTAT	C7orf71__NM_001145531	334	531	422	526
C7orf71__NM_001145531_CAAAGATGCCACTCATAATG	C7orf71__NM_001145531	418	549	347	361
C7orf71__NM_001145531_CTTCACAGTCTATACCAGGC	C7orf71__NM_001145531	458	502	569	602
C7orf71__NM_001145531_GAAGCATCGGGGTGCTTATA	C7orf71__NM_001145531	332	427	289	387
C7orf71__NM_001145531_TACAGAGCTTTCTCCGCATG	C7orf71__NM_001145531	2555	2696	3026	3082
C7orf71__NM_001145531_TGGGTTTCACAGAAGCATCG	C7orf71__NM_001145531	3	0	3	39
C7orf72__NM_001161834_AGAACAGACATAACTATGGC	C7orf72__NM_001161834	193	199	220	190
C7orf72__NM_001161834_AGTGGAAATAATGGATAATG	C7orf72__NM_001161834	239	260	213	298
C7orf72__NM_001161834_CACATCACTAAAATCCACTA	C7orf72__NM_001161834	732	958	932	960
C7orf72__NM_001161834_CCCTGAACAGGAATCACCTA	C7orf72__NM_001161834	936	1242	937	1001
C7orf72__NM_001161834_CTTAAGAGATCACAATAGTG	C7orf72__NM_001161834	139	262	198	187
C7orf72__NM_001161834_CTTGAGAACAGACATAACTA	C7orf72__NM_001161834	216	476	282	284
C7orf72__NM_001161834_GGGTGAAATTTAAGAAAAGC	C7orf72__NM_001161834	51	26	46	62
C7orf72__NM_001161834_GGTCTGGGAACTCGATGTTG	C7orf72__NM_001161834	804	837	757	843
C7orf72__NM_001161834_TCACTAAAATCCACTAGGGT	C7orf72__NM_001161834	329	472	432	427
C7orf72__NM_001161834_TGTTCAGGGTTTATGAGTCC	C7orf72__NM_001161834	121	205	170	207
C7orf72__NM_001161834_TTAAGAGATCACAATAGTGT	C7orf72__NM_001161834	236	298	206	267
C7orf72__NM_001161834_TTGAAATCCTGTGTCTGGTC	C7orf72__NM_001161834	185	251	233	369
C7orf73__NM_001130929_TTTCAGCTTGGATTTACACT	C7orf73__NM_001130929	129	151	176	187
C7orf76__NM_001201450_AGGCCGGATGCTTACTCTCA	C7orf76__NM_001201450	1781	1967	1899	2183
C7orf76__NM_001201450_ATGCTTACTCTCATGGATCC	C7orf76__NM_001201450	134	144	109	154
C7orf76__NM_001201450_GGCTAACTCTGTTGTCGCTC	C7orf76__NM_001201450	315	502	314	440
C7orf76__NM_001201450_TGAGCTGGGCTTCTTATGCT	C7orf76__NM_001201450	715	651	793	897
C7orf76__NM_001201450_TGTGCTGTGTTTGCTGTACA	C7orf76__NM_001201450	806	800	822	894
C8A__NM_000562_AAACGCCACCTTGTGTGTAA	C8A__NM_000562	524	486	660	609
C8A__NM_000562_ACACTGGCCCCCATAATAAC	C8A__NM_000562	1007	919	938	923
C8A__NM_000562_ACTCACCTGTCTCCTTACAC	C8A__NM_000562	1301	1067	1364	1027
C8A__NM_000562_AGAAAACTCAATCCATGACC	C8A__NM_000562	280	218	330	257
C8A__NM_000562_AGTGTGAGACGGTATACAAT	C8A__NM_000562	623	366	684	448
C8A__NM_000562_ATGGTTCCCCCAAACTTGTT	C8A__NM_000562	70	72	112	84
C8A__NM_000562_CAGTGTGAGACGGTATACAA	C8A__NM_000562	543	497	504	584
C8A__NM_000562_CCAGTTCTACAACTTGTGTA	C8A__NM_000562	324	376	346	357
C8A__NM_000562_CTCTTGCAGCCAAACAAGTT	C8A__NM_000562	0	0	0	0
C8A__NM_000562_GTGTACGATGCCAGTTATTA	C8A__NM_000562	197	326	203	284
C8A__NM_000562_GTGTGAGACGGTATACAATG	C8A__NM_000562	253	158	221	147
C8A__NM_000562_TCAGCCTGGGGTAACTGCAC	C8A__NM_000562	683	707	696	588
C8A__NM_000562_TTGCAGCCAAACAAGTTTGG	C8A__NM_000562	47	18	97	70
C8B__NM_000066_ATGGACCAATACTGGGGAAT	C8B__NM_000066	440	451	628	650
C8B__NM_000066_CAGAGAAGAAATAGCTCCAC	C8B__NM_000066	173	239	132	99
C8B__NM_000066_CAGATGAAGCAAACTGTAGA	C8B__NM_000066	513	465	433	417
C8B__NM_000066_CATGAAATGGACCAATACTG	C8B__NM_000066	884	910	967	710
C8B__NM_000066_CGGTTCCCCATGGAACTGAG	C8B__NM_000066	13	19	0	0
C8B__NM_000066_CGTGTTCAGGATGTAATGCG	C8B__NM_000066	894	951	900	942
C8B__NM_000066_CTTGATCACAGGTATTATGC	C8B__NM_000066	379	481	485	364
C8B__NM_000066_GCATGAAATGGACCAATACT	C8B__NM_000066	576	596	757	925
C8B__NM_000066_GCTTCCTAAACCTCGTGTTC	C8B__NM_000066	578	771	634	760
C8B__NM_000066_GGAAGTCAAGTGCGATGTGA	C8B__NM_000066	440	530	426	617
C8B__NM_000066_GGGGTCACATGTGGTCCAAG	C8B__NM_000066	325	248	328	293
C8B__NM_000066_TCCACCTGGAGTTCATGCAA	C8B__NM_000066	616	693	827	698
C8B__NM_000066_TTGATTGTGAGCTGTCTAGT	C8B__NM_000066	149	214	191	191
C8B__NM_001278543_TGTGGAAAGCTACACGCCAC	C8B__NM_001278543	375	231	354	277
C8B__NM_001278544_AATGGACACTTGTATGACTC	C8B__NM_001278544	225	145	329	347
C8B__NM_001278544_ATATGAATTCGTATTTGCCT	C8B__NM_001278544	356	479	515	584
C8G__NM_000606_CAGCTGTCAGTGAAGCTCTA	C8G__NM_000606	120	126	145	212
C8G__NM_000606_CTCAGGACCGAGTCGCTCAC	C8G__NM_000606	736	979	746	798
C8G__NM_000606_CTCGCTCCCTGTGAGCGACT	C8G__NM_000606	58	103	67	45
C8G__NM_000606_GACAGCGAAACTCTGGTAGT	C8G__NM_000606	762	667	916	735
C8G__NM_000606_GTGAGCGACTCGGTCCTGAG	C8G__NM_000606	603	596	594	611
C8G__NM_000606_TCAAGAGGGTCGCAGTCCCA	C8G__NM_000606	513	507	654	704
C8G__NM_000606_TCAGGACCGAGTCGCTCACA	C8G__NM_000606	921	629	899	737
C8orf22__NM_001007176_AATGGAAGGTACGGATGCCA	C8orf22__NM_001007176	516	395	628	693
C8orf22__NM_001007176_GAAAAACGATTTAAACCACC	C8orf22__NM_001007176	56	165	94	115
C8orf22__NM_001007176_GCTAGAAGGCAACCAATGGA	C8orf22__NM_001007176	1413	1951	1546	1906
C8orf22__NM_001007176_TAAAAGATCTGTCTGCTACT	C8orf22__NM_001007176	117	149	131	95
C8orf31__NM_173687_AGATGCTTGGCGGTCTTGGA	C8orf31__NM_173687	820	1153	953	1046
C8orf31__NM_173687_CTCCCTTGCAGCAAGAGATC	C8orf31__NM_173687	36	61	29	13
C8orf31__NM_173687_GAGCAGGAGCTGTACGCACA	C8orf31__NM_173687	225	220	233	227
C8orf31__NM_173687_GATGCTTGGCGGTCTTGGAA	C8orf31__NM_173687	447	527	501	623
C8orf31__NM_173687_TCTGCGCTGCTACAGATGCT	C8orf31__NM_173687	493	568	606	795
C8orf33__NM_023080_ACTCCTTGTTTGGAGACTAT	C8orf33__NM_023080	590	391	599	383
C8orf33__NM_023080_ATAAGAAGAAAACGCGGAAC	C8orf33__NM_023080	250	82	211	162
C8orf33__NM_023080_CCGCAGGGCCTCACGCCATT	C8orf33__NM_023080	885	634	928	640
C8orf33__NM_023080_GACTCCAGAGCGCACCCGTT	C8orf33__NM_023080	1750	927	2022	1098
C8orf33__NM_023080_GATGGAAGCCGAATGGCGTG	C8orf33__NM_023080	197	69	170	169
C8orf33__NM_023080_GCCCAGGTGCAACCTGTAGA	C8orf33__NM_023080	609	386	774	355
C8orf33__NM_023080_GCCTGGGCCTCAGCGCTTAG	C8orf33__NM_023080	536	263	513	387
C8orf33__NM_023080_GCGCACCCGTTGGGCGATGA	C8orf33__NM_023080	459	415	448	463
C8orf33__NM_023080_GGGCTCAGATGGAAGCCGAA	C8orf33__NM_023080	304	153	273	278
C8orf33__NM_023080_TGCCGCCTTCATCGCCCAAC	C8orf33__NM_023080	512	477	549	390
C8orf33__NM_023080_TTGGCTCTCCATATAGAGCG	C8orf33__NM_023080	41	30	20	35
C8orf34__NM_001195639_ACCGGACTCCAAATCATGTA	C8orf34__NM_001195639	603	482	717	650
C8orf34__NM_001195639_AGGTGTCTCAGTTATTAACT	C8orf34__NM_001195639	74	12	52	18
C8orf34__NM_001195639_CAACTTCAAAGAACTTTGTC	C8orf34__NM_001195639	453	855	586	719
C8orf34__NM_001195639_CAAGATCGGTCCCCTGTTTG	C8orf34__NM_001195639	1246	1182	1518	1578
C8orf34__NM_001195639_CATGATTTGGAGTCCGGTGA	C8orf34__NM_001195639	679	554	842	813
C8orf34__NM_001195639_CCCCAGTCCGGGTAAAAGGA	C8orf34__NM_001195639	420	535	436	491
C8orf34__NM_001195639_GACCATCTTCAGTCTAAACA	C8orf34__NM_001195639	192	169	154	243
C8orf34__NM_001195639_GCCCCAGTCCGGGTAAAAGG	C8orf34__NM_001195639	409	329	411	286
C8orf34__NM_001195639_GGTTTGCGGATGAGACGCCA	C8orf34__NM_001195639	159	184	224	274
C8orf34__NM_001195639_TCAGAAGCTATGATAAACCT	C8orf34__NM_001195639	1254	1156	1500	1412
C8orf34__NM_001195639_TCCCTTGTTTAGACTGAAGA	C8orf34__NM_001195639	405	778	465	681
C8orf34__NM_001195639_TTCTCCCTGCACTCTCTTCG	C8orf34__NM_001195639	0	0	2	0
C8orf37__NM_177965_AGTTGCAGTCCGGTGTACCT	C8orf37__NM_177965	1594	1272	1869	1478
C8orf37__NM_177965_CACACCTGACCTTCTAAGAC	C8orf37__NM_177965	1241	1137	1390	1593
C8orf37__NM_177965_CCATTGAAGGCCTTGGTAAA	C8orf37__NM_177965	219	199	184	230
C8orf37__NM_177965_CGACCATACCCCGTCTTAGA	C8orf37__NM_177965	1197	1083	1395	1250
C8orf37__NM_177965_CTTGGTTCCGGTCGCTACTG	C8orf37__NM_177965	1149	1272	1176	1391
C8orf37__NM_177965_GAGCTTCCACCAAGGTACAC	C8orf37__NM_177965	27	67	17	17
C8orf37__NM_177965_GCACACCTGACCTTCTAAGA	C8orf37__NM_177965	1812	1542	1904	1560
C8orf37__NM_177965_GCGGCACCCACAGTAGCGAC	C8orf37__NM_177965	329	330	431	414
C8orf37__NM_177965_TGACCTTCTAAGACGGGGTA	C8orf37__NM_177965	168	95	214	198
C8orf37__NM_177965_TGCAGTCCGGTGTACCTTGG	C8orf37__NM_177965	508	514	482	305
C8orf37__NM_177965_TTGGAACAAATATTTCATGG	C8orf37__NM_177965	261	229	246	194
C8orf37__NM_177965_TTGGTTCCGGTCGCTACTGT	C8orf37__NM_177965	61	76	62	61
C8orf4__NM_020130_AGCCTCTCGTAAGAAAGCCG	C8orf4__NM_020130	112	121	228	158
C8orf4__NM_020130_CAGACCAAGAATCACTAGAA	C8orf4__NM_020130	137	223	138	149
C8orf4__NM_020130_CTCCTCCACATCTTGATCTA	C8orf4__NM_020130	474	605	593	690
C8orf4__NM_020130_GCCCACGGCTTTCTTACGAG	C8orf4__NM_020130	1368	1600	1579	2126
C8orf4__NM_020130_TACGAGAGGCTGTGTCGAAG	C8orf4__NM_020130	428	421	364	397
C8orf4__NM_020130_TGGGCTGACTCGTAGCGACG	C8orf4__NM_020130	73	108	69	105
C8orf44__NM_019607_CCCACACTTTCCCTCATGGG	C8orf44__NM_019607	1400	758	1602	1130
C8orf44__NM_019607_GGAATGGGGATAGGGGGTGC	C8orf44__NM_019607	329	308	467	354
C8orf44__NM_019607_GGGAGCACTTCGAAAACCAC	C8orf44__NM_019607	382	560	487	513
C8orf46__NM_152765_ACCTAACTTATGTGAGTGTC	C8orf46__NM_152765	1023	1230	1204	1473
C8orf46__NM_152765_ACCTATGCTGGTCGTAACAG	C8orf46__NM_152765	351	315	456	364
C8orf46__NM_152765_AGATTACAGGACCAGATACC	C8orf46__NM_152765	141	220	170	209
C8orf46__NM_152765_AGTCATCTTCTTGAGATGTC	C8orf46__NM_152765	311	248	334	517
C8orf46__NM_152765_CAAGAAGAAGTCTGAATATG	C8orf46__NM_152765	760	889	799	679
C8orf46__NM_152765_CAAGTCAGTTGCTAGAGAGT	C8orf46__NM_152765	489	256	457	358
C8orf46__NM_152765_CACGCACCTCTGCTAGTAGC	C8orf46__NM_152765	528	786	575	628
C8orf46__NM_152765_CACTTCTTTGTATCAACATA	C8orf46__NM_152765	120	130	191	170
C8orf46__NM_152765_CATCAATCATGTTTAGTGTT	C8orf46__NM_152765	1417	1831	1622	2045
C8orf46__NM_152765_CCAAAACATCAAATCTGTGT	C8orf46__NM_152765	654	888	923	944
C8orf46__NM_152765_CCAACACAGGGCACTTGAAG	C8orf46__NM_152765	355	344	305	448
C8orf46__NM_152765_CCGGCATCCTCCGGAAAATG	C8orf46__NM_152765	776	767	744	755
C8orf46__NM_152765_CTGACAGGCAGTGCTTCCTG	C8orf46__NM_152765	985	1022	1226	1095
C8orf46__NM_152765_CTGTGTGGGAATCGAGCATA	C8orf46__NM_152765	45	113	72	51
C8orf46__NM_152765_CTTTCCTGTGCCCTATCCCA	C8orf46__NM_152765	372	358	448	290
C8orf46__NM_152765_GAAGCCTCTCTACCACTGAC	C8orf46__NM_152765	1140	1476	1434	1437
C8orf46__NM_152765_GAGGTTAAATGATTAGACTA	C8orf46__NM_152765	134	86	155	153
C8orf46__NM_152765_GCGCCTTTGAGGCCGACTAA	C8orf46__NM_152765	190	169	200	150
C8orf46__NM_152765_GGAGGTCTTTAAAATGAGCT	C8orf46__NM_152765	284	321	287	463
C8orf46__NM_152765_GTCCCTGCGGTCTCCGCGGT	C8orf46__NM_152765	380	322	407	377
C8orf46__NM_152765_GTCTCATTCAATAGAGATGT	C8orf46__NM_152765	471	248	511	467
C8orf46__NM_152765_TAGTATCATGCCTGAAACAT	C8orf46__NM_152765	364	402	313	266
C8orf46__NM_152765_TATGTTTCAGGCATGATACT	C8orf46__NM_152765	264	137	314	260
C8orf46__NM_152765_TCACCTGGAGATATGGAAAT	C8orf46__NM_152765	225	219	254	302
C8orf46__NM_152765_TCCCTGCGGTCTCCGCGGTG	C8orf46__NM_152765	2	6	0	0
C8orf46__NM_152765_TCCTCTGTTACGACCAGCAT	C8orf46__NM_152765	208	128	180	155
C8orf46__NM_152765_TCGCTTCTCATGGATTCACC	C8orf46__NM_152765	171	330	172	158
C8orf46__NM_152765_TCTGAAGTGGCATAGTCTAG	C8orf46__NM_152765	106	63	98	153
C8orf46__NM_152765_TGAGTCTGAGACCTGTAATG	C8orf46__NM_152765	214	168	260	265
C8orf46__NM_152765_TTAAGAAGGGGCTATCCCAG	C8orf46__NM_152765	181	188	289	207
C8orf46__NM_152765_TTACGACCAGCATAGGTTAC	C8orf46__NM_152765	0	0	0	0
C8orf46__NM_152765_TTGAAAATTGTACCTTGTCA	C8orf46__NM_152765	136	276	212	177
C8orf46__NM_152765_TTGAATTGGCATCTATTAAG	C8orf46__NM_152765	107	181	135	131
C8orf48__NM_001007090_AAAGACTTTCTCACCCGTAT	C8orf48__NM_001007090	489	741	600	811
C8orf48__NM_001007090_ACTTCCAACAGAAATCACTC	C8orf48__NM_001007090	269	460	325	501
C8orf48__NM_001007090_AGAGTAAGTCGCTTGAGAAA	C8orf48__NM_001007090	560	661	616	647
C8orf48__NM_001007090_ATCAGTGTCCCTATTGTAAC	C8orf48__NM_001007090	430	725	532	625
C8orf48__NM_001007090_ATCGGGTACAGTACAACTAA	C8orf48__NM_001007090	407	628	457	433
C8orf48__NM_001007090_CAGATGACCCCAGAATAATC	C8orf48__NM_001007090	265	322	342	471
C8orf48__NM_001007090_CATGAGGACAACGCCAAAAC	C8orf48__NM_001007090	441	452	486	618
C8orf48__NM_001007090_CCAGTTTGCTCCCAGAGGTC	C8orf48__NM_001007090	155	223	127	123
C8orf48__NM_001007090_TTGATGTGCTTCTCCAATAC	C8orf48__NM_001007090	185	208	187	156
C8orf48__NM_001007090_TTGTCCGTTTGGGCCAATTC	C8orf48__NM_001007090	312	434	381	461
C8orf48__NM_001007090_TTTGAAGAACTCTACTGATG	C8orf48__NM_001007090	179	284	172	238
C8orf58__NM_001013842_AAAGGCCCGGTAAGGCGGCC	C8orf58__NM_001013842	50	65	42	92
C8orf58__NM_001013842_ACCCCAGGCAATCGGGGGCA	C8orf58__NM_001013842	574	576	685	891
C8orf58__NM_001013842_AGGAGTGGAGATGGCAGTTG	C8orf58__NM_001013842	381	249	502	232
C8orf58__NM_001013842_ATTGCCTGGGGTGTGTAACG	C8orf58__NM_001013842	551	633	663	673
C8orf58__NM_001013842_CAGGAGTGGAGATGGCAGTT	C8orf58__NM_001013842	1193	543	1184	886
C8orf58__NM_001013842_CAGGCTTGCTTGCCAGCCGC	C8orf58__NM_001013842	154	65	153	115
C8orf58__NM_001013842_CCCTGGACTTTGAATCCTCA	C8orf58__NM_001013842	774	989	857	1050
C8orf58__NM_001013842_CGGGCAGGGTCTTCGCTATC	C8orf58__NM_001013842	2593	3269	2829	3295
C8orf58__NM_001013842_GCCCCTGCCCCCGATTGCCT	C8orf58__NM_001013842	2054	2377	2278	2866
C8orf58__NM_001013842_GTGTAACGGGGACGGTAAAG	C8orf58__NM_001013842	110	126	99	77
C8orf58__NM_001013842_GTGTGTAACGGGGACGGTAA	C8orf58__NM_001013842	228	139	214	233
C8orf58__NM_001013842_TGTGTAACGGGGACGGTAAA	C8orf58__NM_001013842	48	48	76	150
C8orf59__NM_001099670_ACTCACCTTCTTAAGATTAG	C8orf59__NM_001099670	381	54	417	98
C8orf59__NM_001099670_AGTTACCACTAATCTTAAGA	C8orf59__NM_001099670	327	11	345	60
C8orf59__NM_001099670_ATGGCAAGAACAAATTAAG	C8orf59__NM_001099670	131	1	147	19
C8orf59__NM_001099670_TTCTTAAGATTAGTGGTAAC	C8orf59__NM_001099670	8	19	12	20
C8orf74__NM_001040032_AAGCCCCAAAGAGCGAGCAA	C8orf74__NM_001040032	293	233	365	393
C8orf74__NM_001040032_ATGGCACTCTTAACACCCCA	C8orf74__NM_001040032	123	104	211	237
C8orf74__NM_001040032_CAAACTCTACCAGTATGTCC	C8orf74__NM_001040032	2328	2530	2276	3022
C8orf74__NM_001040032_CCAGAACACATTCGCCATCT	C8orf74__NM_001040032	2938	3803	3217	3909
C8orf74__NM_001040032_CGCAGGCGCTCCCGACCTTG	C8orf74__NM_001040032	687	767	769	973
C8orf74__NM_001040032_CTGGGGTGTTAAGAGTGCCA	C8orf74__NM_001040032	328	434	291	427
C8orf74__NM_001040032_GATCGTCACAGCCTCAGTAA	C8orf74__NM_001040032	488	613	475	565
C8orf74__NM_001040032_GGAACAAGCTTAGAGATTAC	C8orf74__NM_001040032	41	66	140	127
C8orf74__NM_001040032_GGGCAAAGGCTTCCCATGGG	C8orf74__NM_001040032	550	362	557	634
C8orf74__NM_001040032_TACTGAGGCTGTGACGATCC	C8orf74__NM_001040032	356	445	419	426
C8orf76__NM_032847_GAGCTTGGCGCAGTAGGACG	C8orf76__NM_032847	211	184	270	209
C8orf76__NM_032847_GCGAGATGGATTCCGGGTGC	C8orf76__NM_032847	1581	1461	1584	1464
C8orf76__NM_032847_TCCGGGTGCTGGTTGTTCGG	C8orf76__NM_032847	1088	938	1161	1199
C8orf82__NM_001001795_CACGTAGTAGAAATACTCGC	C8orf82__NM_001001795	864	672	943	758
C8orf82__NM_001001795_CCTGGCCGTGCCCTTCGAGC	C8orf82__NM_001001795	3448	2627	3827	2899
C8orf82__NM_001001795_CTCAGCGCCTGCTTCGAGTA	C8orf82__NM_001001795	556	601	583	714
C8orf82__NM_001001795_GCCGGGCCCGTACTCGAAGC	C8orf82__NM_001001795	384	539	399	493
C8orf82__NM_001001795_GCCTGCTTCGAGTACGGGCC	C8orf82__NM_001001795	508	613	594	664
C8orf82__NM_001001795_GGGGGCGTTTCCTACACGCA	C8orf82__NM_001001795	506	583	569	764
C8orf82__NM_001001795_GTCCGGAGCGTCCCGCAAGG	C8orf82__NM_001001795	17	6	11	1
C8orf82__NM_001001795_TCGTAGCGCCCGCTGCGGTT	C8orf82__NM_001001795	555	401	673	536
C8orf82__NM_001001795_TGGGGGCGTTTCCTACACGC	C8orf82__NM_001001795	172	63	268	153
C8orf86__NM_207412_ACAGCCACTTCTACACGTCA	C8orf86__NM_207412	672	783	789	832
C8orf86__NM_207412_CTCACCGTGACGTGTAGAAG	C8orf86__NM_207412	391	492	556	722
C8orf86__NM_207412_CTTATGAGAATACAGGCCAC	C8orf86__NM_207412	81	65	124	182
C8orf86__NM_207412_CTTCCATTTCTATCACATTC	C8orf86__NM_207412	420	543	519	676
C8orf86__NM_207412_CTTCCCTTCTGTCCCAAACT	C8orf86__NM_207412	600	459	615	647
C8orf86__NM_207412_GAAGAATGAGAGATCACTGT	C8orf86__NM_207412	555	523	631	516
C8orf87__NM_001242668_CAACTTCAAAGAAGGCAGTT	C8orf87__NM_001242668	380	282	422	529
C8orf88__NM_001190972_GGATGATCAGGCAGAAAGTC	C8orf88__NM_001190972	241	356	261	339
C8orf89__NM_001243237_CCAAATGCTGTGGTATATTC	C8orf89__NM_001243237	511	636	646	828
C8orf89__NM_001243237_CTTTGCAGTCCTGGTAAATA	C8orf89__NM_001243237	416	521	390	448
C8orf89__NM_001243237_GGAATATGATACCATTCGTC	C8orf89__NM_001243237	297	53	355	308
C8orf89__NM_001243237_GGCTTCAGTGATCCTCTCAC	C8orf89__NM_001243237	970	763	1060	652
C9__NM_001737_AAAAGATGCACCGACGCTGT	C9__NM_001737	1007	1020	1074	960
C9__NM_001737_AAGATGCGACTTCGGTGTAA	C9__NM_001737	433	397	525	692
C9__NM_001737_ATGGACTCTGTAACCGGGAT	C9__NM_001737	373	636	340	589
C9__NM_001737_CTTACCATTTGTCTGAGACA	C9__NM_001737	736	725	886	964
C9__NM_001737_GAGTGTTTCCATCCCGATCC	C9__NM_001737	548	848	667	700
C9__NM_001737_GATGTTGTTTCACTCATAAG	C9__NM_001737	524	367	616	545
C9__NM_001737_GCTACTGTAGTGAGTTCCAT	C9__NM_001737	1366	1495	1401	1890
C9__NM_001737_TAAAGCTGAACAATGTTGTG	C9__NM_001737	187	441	188	204
C9__NM_001737_TCACAGCACAGTACACGACC	C9__NM_001737	680	518	718	810
C9__NM_001737_TCTACAATGGACTCTGTAAC	C9__NM_001737	256	499	347	427
C9__NM_001737_TGACATACTACCGAAGACCT	C9__NM_001737	915	1281	901	995
C9__NM_001737_TTTGCCAACTACCTATGAAA	C9__NM_001737	348	238	310	308
C9orf106__NM_001012715_AAGAGAGGATGCAACCTTAG	C9orf106__NM_001012715	279	288	213	244
C9orf106__NM_001012715_AGAGGATGCAACCTTAGAGG	C9orf106__NM_001012715	331	235	356	194
C9orf106__NM_001012715_AGAGGGGGCAGGGGTAAACA	C9orf106__NM_001012715	361	131	303	122
C9orf106__NM_001012715_AGGTTGCATCCTCTCTTACA	C9orf106__NM_001012715	552	330	568	357
C9orf106__NM_001012715_ATGGCCAAGATGGCTCTACC	C9orf106__NM_001012715	15	73	43	17
C9orf106__NM_001012715_CAGGTGAGGTTTATCGCTCA	C9orf106__NM_001012715	369	401	475	564
C9orf106__NM_001012715_CCCCGGGCATCAGTGCTCTG	C9orf106__NM_001012715	239	377	246	309
C9orf106__NM_001012715_GCACATCGGCAGGCCCTAAG	C9orf106__NM_001012715	317	495	406	320
C9orf106__NM_001012715_TAGGGCTGCCGATGTGCTG	C9orf106__NM_001012715	1406	1599	1319	1328
C9orf106__NM_001012715_TTAGGGCCTGCCGATGTGCT	C9orf106__NM_001012715	356	421	346	388
C9orf106__NM_001012715_TTGTGTGTCCCATGTAAGAG	C9orf106__NM_001012715	142	39	169	141
C9orf114__NM_016390_AAAGGATTGAGTGGCGAAAA	C9orf114__NM_016390	86	40	68	22
C9orf114__NM_016390_ATTCCTCATCCTGACGCATG	C9orf114__NM_016390	131	5	241	12
C9orf114__NM_016390_CAGCCCCCACCACATGCGTC	C9orf114__NM_016390	1294	33	1331	39
C9orf114__NM_016390_CAGGACCCTCGCACCAAAGC	C9orf114__NM_016390	267	22	307	26
C9orf114__NM_016390_CCAGACTGCAAGACCTACCA	C9orf114__NM_016390	862	101	1001	66
C9orf114__NM_016390_CCTCATCCTGACGCATGTGG	C9orf114__NM_016390	1940	170	2225	362
C9orf114__NM_016390_GCCAAGTAGGTGCGAAGCTC	C9orf114__NM_016390	46	0	23	0
C9orf114__NM_016390_GCCGGAGCTTCGCACCTACT	C9orf114__NM_016390	290	49	313	3
C9orf114__NM_016390_GGCAATCTGACCGGCCAAGT	C9orf114__NM_016390	851	97	852	246
C9orf114__NM_016390_TACCTGGAGTGTCCACAGTA	C9orf114__NM_016390	583	0	754	25
C9orf114__NM_016390_TCCAGGGTGAACACGGCCAA	C9orf114__NM_016390	193	62	197	80
C9orf114__NM_016390_TGGCAAAGTGGTATCATCGC	C9orf114__NM_016390	110	36	87	19
C9orf116__NM_001048265_AAACATTGAGAGTATTGTTC	C9orf116__NM_001048265	99	71	138	156
C9orf116__NM_001048265_ACAGGCATCTCGTGCACGGT	C9orf116__NM_001048265	1467	1064	1586	1212
C9orf116__NM_001048265_AGGAGGTGATGCAGTTATCG	C9orf116__NM_001048265	431	414	530	376
C9orf116__NM_001048265_CAATACTCTCAATGTTTACC	C9orf116__NM_001048265	537	698	499	585
C9orf116__NM_001048265_CAGGAGGTGATGCAGTTATC	C9orf116__NM_001048265	786	517	888	711
C9orf116__NM_001048265_CGTGTACAGGACCAGTAACC	C9orf116__NM_001048265	635	613	724	954
C9orf116__NM_001048265_CTGGAGAAAAGCATCGTGAC	C9orf116__NM_001048265	502	719	666	731
C9orf116__NM_001048265_CTGGTTACTGGTCCTGTACA	C9orf116__NM_001048265	203	210	236	230
C9orf116__NM_001048265_GAAGTTGAGCCGGTCACAGG	C9orf116__NM_001048265	541	409	508	344
C9orf116__NM_001048265_GCCTGTTGATGTTGTAACTG	C9orf116__NM_001048265	992	912	894	1175
C9orf116__NM_001048265_GGCCTGTTGATGTTGTAACT	C9orf116__NM_001048265	591	343	670	755
C9orf116__NM_001048265_TGTTGATGTTGTAACTGGGG	C9orf116__NM_001048265	151	47	137	108
C9orf117__NM_001012502_ACAGATGCACCGCGATGAAG	C9orf117__NM_001012502	490	603	579	304
C9orf117__NM_001012502_AGTTCCCCTACGTCCCAAGT	C9orf117__NM_001012502	495	435	617	480
C9orf117__NM_001012502_CTGGAACTCGTGGCGCACCT	C9orf117__NM_001012502	579	642	896	943
C9orf117__NM_001012502_CTGTCATATATCACCCGTGT	C9orf117__NM_001012502	1386	1866	1542	2059
C9orf117__NM_001012502_GATGTGGGAGACAACCAAGC	C9orf117__NM_001012502	376	302	415	267
C9orf117__NM_001012502_GTGCCCCTGTTCTCTAAAA	C9orf117__NM_001012502	52	24	100	67
C9orf117__NM_001012502_GTGTCCTACTTGGGACGTAG	C9orf117__NM_001012502	117	88	111	213
C9orf117__NM_001012502_TCCCACTTCCGCTGGTACCT	C9orf117__NM_001012502	688	625	726	993
C9orf117__NM_001012502_TGTCATATATCACCCGTGTG	C9orf117__NM_001012502	60	58	59	78
C9orf129__NM_001098808_GCGGAGCTGCTTGCTCAGTA	C9orf129__NM_001098808	918	878	1083	1329
C9orf129__NM_001098808_GGAATGTCAGTTGTTGCGCT	C9orf129__NM_001098808	728	844	676	864
C9orf129__NM_001098808_GGCGGAGCTGCTTGCTCAGT	C9orf129__NM_001098808	1259	1252	1432	1620
C9orf131__NM_001040410_ACAAGCTTGCCTTCCTACCT	C9orf131__NM_001040410	1076	981	1146	1097
C9orf131__NM_001040410_ACTGGGCTGAGGAGTGATAC	C9orf131__NM_001040410	386	460	473	488
C9orf131__NM_001040410_AGGGCCTCCTCAAGGCTATA	C9orf131__NM_001040410	101	76	123	156
C9orf131__NM_001040410_CCAGTGGGAGCTTCTTTGGT	C9orf131__NM_001040410	1604	1333	1719	1387
C9orf131__NM_001040410_CTGGTATCCCTATAGCCTTG	C9orf131__NM_001040410	151	247	187	203
C9orf131__NM_001040410_CTTGGGGCTAAGGGGGATAT	C9orf131__NM_001040410	630	521	629	730
C9orf131__NM_001040410_GCTTCTTTGGTAGGAGAACA	C9orf131__NM_001040410	62	106	79	78
C9orf131__NM_001040410_GCTTGGGGCTAAGGGGGATA	C9orf131__NM_001040410	181	251	242	294
C9orf131__NM_001040410_GGAAGCAACAGGTTGGACCT	C9orf131__NM_001040410	124	145	134	132
C9orf131__NM_001040410_GGCTGCATGACTGTTTGCTC	C9orf131__NM_001040410	279	236	317	397
C9orf131__NM_001040410_GTATCCCTATAGCCTTGAGG	C9orf131__NM_001040410	241	255	316	196
C9orf131__NM_001040410_GTGAGGCACAAGATAGCATC	C9orf131__NM_001040410	72	7	67	44
C9orf131__NM_001040411_GGCCCCATGGGTAGAAAACT	C9orf131__NM_001040411	223	166	193	190
C9orf131__NM_001040411_TACCCATGGGGCCCATACTA	C9orf131__NM_001040411	344	403	408	316
C9orf131__NM_203299_CACTATTCTTATTCGTGGTT	C9orf131__NM_203299	1211	1358	1262	1748
C9orf131__NM_203299_GGTGACACTATTCTTATTCG	C9orf131__NM_203299	850	1378	919	987
C9orf135__NM_001010940_AATGAGGAAACAGTGAGCTC	C9orf135__NM_001010940	10	10	28	12
C9orf135__NM_001010940_AATTCAACATATCGGCGACT	C9orf135__NM_001010940	233	213	414	437
C9orf135__NM_001010940_CACCAGGAATACACCAACAC	C9orf135__NM_001010940	547	674	594	648
C9orf135__NM_001010940_CCCAAAGGTTATGATATAGA	C9orf135__NM_001010940	642	770	714	585
C9orf135__NM_001010940_CTGGTTGGAGATCGGACCAC	C9orf135__NM_001010940	185	81	166	144
C9orf135__NM_001010940_GAGCAAGGCTTTATTGAATG	C9orf135__NM_001010940	324	431	389	533
C9orf135__NM_001010940_GATCGGACCACCGGACTGGG	C9orf135__NM_001010940	969	629	1020	1104
C9orf135__NM_001010940_GATCTGTCAAGGCTATCCAT	C9orf135__NM_001010940	565	459	621	560
C9orf135__NM_001010940_GCTTCGAGTATTTCTTGTGA	C9orf135__NM_001010940	778	902	901	1309
C9orf135__NM_001010940_TAATTCAACATATCGGCGAC	C9orf135__NM_001010940	89	75	173	172
C9orf135__NM_001010940_TCACAAGAAATACTCGAAGC	C9orf135__NM_001010940	357	175	333	378
C9orf135__NM_001010940_TCCCAAAGGTTATGATATAG	C9orf135__NM_001010940	307	331	252	305
C9orf139__NM_207511_ATGAGTCAAAGAGAAAGTCG	C9orf139__NM_207511	100	105	127	92
C9orf139__NM_207511_ATTGAGATCTCCAGGACGTC	C9orf139__NM_207511	229	344	236	286
C9orf139__NM_207511_CATGGGGCCCAATCGTGCGT	C9orf139__NM_207511	1433	1521	1713	1981
C9orf139__NM_207511_CGAGCTGGCAAACGGGGCTC	C9orf139__NM_207511	112	132	115	100
C9orf139__NM_207511_CGCAGGTTCCCACGCACGAT	C9orf139__NM_207511	1805	2475	2011	2084
C9orf139__NM_207511_CGTCCTGGAGATCTCAATGC	C9orf139__NM_207511	2048	1945	2341	1796
C9orf139__NM_207511_GAGCTGGCAAACGGGGCTCA	C9orf139__NM_207511	349	345	373	436
C9orf139__NM_207511_GAGTGGGGTGTTGGTTGGCT	C9orf139__NM_207511	1142	1841	1226	1505
C9orf139__NM_207511_GCAGGTTCCCACGCACGATT	C9orf139__NM_207511	556	720	791	829
C9orf139__NM_207511_GTCCTCAAGGAGCAAGCTAG	C9orf139__NM_207511	89	30	76	48
C9orf139__NM_207511_TCAGAACGAAAGCCTCTCAG	C9orf139__NM_207511	604	532	793	777
C9orf142__NM_183241_AGGGCCCCCCTGAAAGCGTC	C9orf142__NM_183241	729	828	737	878
C9orf142__NM_183241_CATCCCTGACGCTTTCAGGG	C9orf142__NM_183241	589	865	719	983
C9orf142__NM_183241_GAGCATCCCTGACGCTTTCA	C9orf142__NM_183241	448	335	428	456
C9orf142__NM_183241_GCATCCCTGACGCTTTCAGG	C9orf142__NM_183241	777	670	775	603
C9orf142__NM_183241_GGAGAGTCGCTCATCAACCC	C9orf142__NM_183241	516	304	583	420
C9orf142__NM_183241_GGGCCCCCCTGAAAGCGTCA	C9orf142__NM_183241	319	308	326	259
C9orf142__NM_183241_TCATCAACCCCGGGTTCAAG	C9orf142__NM_183241	197	546	334	362
C9orf142__NM_183241_TGACCGACGCCGCGGAGCTT	C9orf142__NM_183241	168	98	161	176
C9orf142__NM_183241_TGATGTCCTCAGCCGCACTC	C9orf142__NM_183241	200	216	280	287
C9orf142__NM_183241_TGGGCCTGGCAAAACGCGTG	C9orf142__NM_183241	148	191	244	209
C9orf152__NM_001012993_AGTCATCAAGTACATCATAG	C9orf152__NM_001012993	159	58	142	67
C9orf152__NM_001012993_CAGTCATCAAGTACATCATA	C9orf152__NM_001012993	195	117	225	195
C9orf152__NM_001012993_CATCATAGGGGCAAGCTTGT	C9orf152__NM_001012993	534	350	639	523
C9orf152__NM_001012993_CCTATGATGTACTTGATGAC	C9orf152__NM_001012993	731	938	793	1162
C9orf152__NM_001012993_GCACCTGGAGATGCATTGTT	C9orf152__NM_001012993	1643	1092	1870	1539
C9orf152__NM_001012993_GCCCGGAACCTGGGCTTATA	C9orf152__NM_001012993	455	397	451	487
C9orf152__NM_001012993_GGAAGAGGCAGATTCTGAGG	C9orf152__NM_001012993	361	313	364	272
C9orf153__NM_001276366_GAATGTCATGTCATTCACCA	C9orf153__NM_001276366	646	554	853	645
C9orf153__NM_001276366_TATTTCACTTAACGAAGCAC	C9orf153__NM_001276366	81	86	93	53
C9orf153__NM_001276366_TGGTGAATGACATGACATTC	C9orf153__NM_001276366	29	74	25	62
C9orf156__NM_016481_AACCCCACCATAGCACTAAG	C9orf156__NM_016481	125	177	171	170
C9orf156__NM_016481_AGGGTCAGTCCTATTGCATT	C9orf156__NM_016481	1196	1142	1268	1432
C9orf156__NM_016481_CAGGGTCAGTCCTATTGCAT	C9orf156__NM_016481	946	791	845	919
C9orf156__NM_016481_CCCGTGCGGCTGCGTTAAGC	C9orf156__NM_016481	511	572	647	768
C9orf156__NM_016481_CCTATTGCATTGGGACGATG	C9orf156__NM_016481	1068	928	1070	1127
C9orf156__NM_016481_CGAGAATAGCTACAAATGGA	C9orf156__NM_016481	491	583	620	506
C9orf156__NM_016481_CGGCTGCGTTAAGCGGCTC	C9orf156__NM_016481	111	52	156	114
C9orf156__NM_016481_GAGTCATACTCAGCTATGTA	C9orf156__NM_016481	347	190	456	447
C9orf156__NM_016481_GTGCAGCCTCCTAGGCTGAA	C9orf156__NM_016481	37	37	67	127
C9orf156__NM_016481_TAATCCTGAACATTCCTTGA	C9orf156__NM_016481	817	710	976	978
C9orf156__NM_016481_TAGGGCTTGATGTCTAGTAC	C9orf156__NM_016481	312	327	280	272
C9orf156__NM_016481_TCTTGTTCTCGGCCAAGAA	C9orf156__NM_016481	139	227	127	159
C9orf16__NM_024112_CCGTTGGGGCCCGACATTGC	C9orf16__NM_024112	567	588	700	567
C9orf16__NM_024112_CGCTGCCATCAACTCCATGC	C9orf16__NM_024112	632	598	783	684
C9orf16__NM_024112_CGGCATCCCAGGTCTCCGT	C9orf16__NM_024112	1653	925	1984	1118
C9orf16__NM_024112_CTCCTCCAGGTGGTCCAGAC	C9orf16__NM_024112	86	225	81	95
C9orf16__NM_024112_TCCGTTGGGGCCCGACATTG	C9orf16__NM_024112	309	148	274	330
C9orf163__NM_152571_ACCACTGGGAAGAAGCAGAT	C9orf163__NM_152571	339	283	344	317
C9orf163__NM_152571_AGGGTTTCGGAGGACCCTAG	C9orf163__NM_152571	166	103	149	189
C9orf163__NM_152571_GAGTTAGGGAGCACGTGGTA	C9orf163__NM_152571	478	412	458	417
C9orf163__NM_152571_GCCCAGGGCTCGACCCTCTA	C9orf163__NM_152571	402	559	478	528
C9orf163__NM_152571_GGATGCTGCCTCTCAGGCGT	C9orf163__NM_152571	1170	533	946	748
C9orf163__NM_152571_GGGTTCTTTGAACTCGCGAG	C9orf163__NM_152571	34	78	75	99
C9orf163__NM_152571_GTGCAGAGCGCCTCGACCAC	C9orf163__NM_152571	705	583	881	890
C9orf163__NM_152571_TGGATGCTGCCTCTCAGGCG	C9orf163__NM_152571	35	7	8	26
C9orf163__NM_152571_TTGAACTCGCGAGCGGCAAA	C9orf163__NM_152571	82	85	129	139
C9orf170__NM_001001709_TCCTGCTCAGTCGGTGCAAA	C9orf170__NM_001001709	231	315	327	474
C9orf171__NM_001282957_AATTATATCGCAATGAACCG	C9orf171__NM_001282957	151	120	186	158
C9orf171__NM_001282957_AATTATGGACTCTACATCCG	C9orf171__NM_001282957	460	243	647	499
C9orf171__NM_001282957_ACCGTGGCGGACATCCGTTC	C9orf171__NM_001282957	1643	1236	1912	1767
C9orf171__NM_001282957_AGGCTGAACTCGGCAAGCCC	C9orf171__NM_001282957	352	284	385	413
C9orf171__NM_001282957_GAACACGTTCCAGCGTCCGA	C9orf171__NM_001282957	870	1348	960	1268
C9orf171__NM_001282957_GCCCGGACCTCACGCGATGG	C9orf171__NM_001282957	2179	1742	2409	1819
C9orf171__NM_001282957_GCCGGAACGGATGTCCGCCA	C9orf171__NM_001282957	544	421	570	530
C9orf171__NM_001282957_GCTGCTTCCTCCATCGCGTG	C9orf171__NM_001282957	762	633	884	922
C9orf171__NM_001282957_GGTTCATTGCGATATAATTC	C9orf171__NM_001282957	35	16	39	50
C9orf171__NM_001282957_TAATTCCGGGTCAGCTCGTG	C9orf171__NM_001282957	367	236	448	491
C9orf171__NM_001282957_TCCCTCCAAACATGACATTT	C9orf171__NM_001282957	652	858	884	960
C9orf171__NM_001282957_TTCCTCATCGCGTGAGGTC	C9orf171__NM_001282957	16	38	6	16
C9orf171__NM_207417_GGAGTCATAAACAGAATAAG	C9orf171__NM_207417	225	293	252	173
C9orf172__NM_001080482_AGTGCTCCAGCACGCCACCC	C9orf172__NM_001080482	229	183	171	176
C9orf172__NM_001080482_CAGGTCCGGAGGGTCCGTAC	C9orf172__NM_001080482	554	309	645	439
C9orf172__NM_001080482_CCGTCGCAGTAGAACGCCCG	C9orf172__NM_001080482	1066	1012	1216	1224
C9orf172__NM_001080482_CGGGCCACGGATCGCTCACA	C9orf172__NM_001080482	884	368	890	497
C9orf172__NM_001080482_CTCGCCATGTGAGCGATCCG	C9orf172__NM_001080482	277	344	434	385
C9orf172__NM_001080482_GCCCAGCGACTCATACTGTG	C9orf172__NM_001080482	854	629	957	1032
C9orf172__NM_001080482_GCCCGTCGCAGTAGAACGCC	C9orf172__NM_001080482	726	839	812	1147
C9orf172__NM_001080482_GCTCCTCTGTATAGAAGAAC	C9orf172__NM_001080482	227	336	236	207
C9orf172__NM_001080482_GGTAGTAGGCGTCGAAGGTT	C9orf172__NM_001080482	448	318	658	801
C9orf172__NM_001080482_TCCGCACAGTATGAGTCGCT	C9orf172__NM_001080482	1997	1627	2376	2052
C9orf172__NM_001080482_TGCTGGAGCACTGCGTCGTC	C9orf172__NM_001080482	244	235	283	358
C9orf172__NM_001080482_TGGCGCTTGTTGAAAGGCGC	C9orf172__NM_001080482	34	57	40	92
C9orf173__NM_001004353_AAGATGAAGGAGATCCCAGT	C9orf173__NM_001004353	750	344	794	643
C9orf173__NM_001004353_AAGTCAGCTTTCTGCGTGAA	C9orf173__NM_001004353	1601	1612	1617	2489
C9orf173__NM_001004353_CCACACCCCCACTACAGCAT	C9orf173__NM_001004353	474	613	508	504
C9orf173__NM_001004353_GACTCTCGTACGGACGGGCT	C9orf173__NM_001004353	582	791	588	959
C9orf173__NM_001004353_GAGCGGCTCATCGAGAAGGC	C9orf173__NM_001004353	252	288	418	339
C9orf173__NM_001004353_GCTCCCAGGAAGGATATTGT	C9orf173__NM_001004353	1195	946	1468	1137
C9orf173__NM_001004353_GGGAGGACTCTCGTACGGAC	C9orf173__NM_001004353	564	567	600	758
C9orf173__NM_001004353_GGGCCAGGGCATCTCTTGCC	C9orf173__NM_001004353	946	727	931	1325
C9orf173__NM_001004353_GTGTGGGGAGGACTCTCGTA	C9orf173__NM_001004353	313	183	311	276
C9orf173__NM_001004353_GTTGGCCTCAGGTTACAGAC	C9orf173__NM_001004353	405	287	436	499
C9orf173__NM_001004353_TCTGTAACCTGAGGCCAACT	C9orf173__NM_001004353	526	556	614	668
C9orf173__NM_001004353_TTCGACCAAGAGCAAAGGT	C9orf173__NM_001004353	15	1	27	53
C9orf24__NM_001252195_AATGCCCTCCTAGGCCGGAG	C9orf24__NM_001252195	100	100	56	81
C9orf24__NM_001252195_AGCAAGCACTTTCGTAACCA	C9orf24__NM_001252195	471	543	447	501
C9orf24__NM_001252195_CCACTACTGTCTGCGCGGGA	C9orf24__NM_001252195	1309	925	1329	1167
C9orf24__NM_001252195_CCGCATCGGAGGTGGATCCG	C9orf24__NM_001252195	1136	865	1003	1079
C9orf24__NM_001252195_CCGCGGATCACGCCCCGATG	C9orf24__NM_001252195	1512	1202	1548	1278
C9orf24__NM_001252195_CCGGAGCGGCTCAATGCCTA	C9orf24__NM_001252195	394	272	419	213
C9orf24__NM_001252195_CCGTAGGCATTGAGCCGCTC	C9orf24__NM_001252195	176	103	210	179
C9orf24__NM_001252195_GCGGGTCCACGCACCCGCAT	C9orf24__NM_001252195	22	41	6	4
C9orf24__NM_001252195_GGATGGACTACTACGCCAGC	C9orf24__NM_001252195	399	373	565	513
C9orf24__NM_001252195_GGGATGGACTACTACGCCAG	C9orf24__NM_001252195	371	475	377	278
C9orf24__NM_001252195_GGGTCCACGCACCCGCATCG	C9orf24__NM_001252195	528	397	609	608
C9orf24__NM_001252195_TGAGCCGCTCCGGCCTAGGA	C9orf24__NM_001252195	129	123	175	180
C9orf24__NM_032596_ACTTCTCAGGCAAGTAGGGG	C9orf24__NM_032596	329	322	296	321
C9orf24__NM_032596_AGTCACTGCCCAGGTAGTTT	C9orf24__NM_032596	1049	902	1076	892
C9orf24__NM_032596_AGTCACTGTAGGTACTGATA	C9orf24__NM_032596	204	205	194	143
C9orf24__NM_032596_GAGTCACTGTAGGTACTGAT	C9orf24__NM_032596	544	627	674	639
C9orf24__NM_032596_GATGGCCAAATGTGCTGTAC	C9orf24__NM_032596	880	624	997	951
C9orf24__NM_032596_GTACAGGACTACACTTATAG	C9orf24__NM_032596	415	480	669	553
C9orf24__NM_032596_GTACCTACAGTGACTCCTAC	C9orf24__NM_032596	444	456	503	507
C9orf24__NM_032596_GTAGGTACTGATAGGGGTCC	C9orf24__NM_032596	124	144	179	214
C9orf24__NM_032596_GTCACTGTAGGTACTGATAG	C9orf24__NM_032596	205	234	205	250
C9orf24__NM_032596_TACAGGACTACACTTATAGG	C9orf24__NM_032596	248	242	265	233
C9orf24__NM_032596_TACCTACAGTGACTCCTACA	C9orf24__NM_032596	875	890	1023	1081
C9orf24__NM_032596_TCCTGGTCTTACGGGAGAAC	C9orf24__NM_032596	141	78	151	171
C9orf24__NM_147169_ACTTACCTAAGGTTCGGATA	C9orf24__NM_147169	151	133	175	97
C9orf24__NM_147169_ATTCCGGGCCGGTGCTCGTA	C9orf24__NM_147169	231	213	240	197
C9orf24__NM_147169_CTCGTAGGGAGGTACACATC	C9orf24__NM_147169	475	214	530	286
C9orf24__NM_147169_GGATATGGGTAGTATGACGG	C9orf24__NM_147169	635	467	677	559
C9orf24__NM_147169_TATGGGTAGTATGACGGCGG	C9orf24__NM_147169	390	326	462	496
C9orf24__NM_147169_TTCGGATATGGGTAGTATGA	C9orf24__NM_147169	33	99	51	37
C9orf3__NM_001193329_ACACTTGGAGCTTGCAGATA	C9orf3__NM_001193329	582	633	654	583
C9orf3__NM_001193329_ACTTGTGAAGCACTATGTAC	C9orf3__NM_001193329	518	580	663	619
C9orf3__NM_001193329_ATCTGTGTTAAAAGTCGAGG	C9orf3__NM_001193329	137	53	193	232
C9orf3__NM_001193329_CTGACTTTCCTCATGCTATC	C9orf3__NM_001193329	378	427	413	597
C9orf3__NM_001193329_CTTTGCCTGCAAATCGTTGG	C9orf3__NM_001193329	498	477	620	579
C9orf3__NM_001193329_GCTGTGACACAGGGAATCAT	C9orf3__NM_001193329	229	146	259	297
C9orf3__NM_001193329_TCAAACAAAGCCTGCAAATT	C9orf3__NM_001193329	239	372	357	428
C9orf3__NM_001193329_TGCTGTGACACAGGGAATCA	C9orf3__NM_001193329	122	133	120	148
C9orf3__NM_001193329_TTCCTCATGCTATCAGGATA	C9orf3__NM_001193329	0	0	3	1
C9orf3__NM_001193329_TTGCATTTGTCACGGGAATA	C9orf3__NM_001193329	74	152	69	66
C9orf3__NM_001193329_TTTATCTGTGTTAAAAGTCG	C9orf3__NM_001193329	111	195	155	103
C9orf3__NM_001193329_TTTGCTGCAAATCGTTGGA	C9orf3__NM_001193329	453	550	439	481
C9orf40__NM_017998_CAAGGCACGTGGAACGTCAC	C9orf40__NM_017998	267	283	327	257
C9orf40__NM_017998_CAGCAAGCGCCGTGACAGCG	C9orf40__NM_017998	80	83	91	140
C9orf40__NM_017998_CCTCTCTGGATCCGGCCGCC	C9orf40__NM_017998	758	795	1093	1430
C9orf40__NM_017998_CTGCCCAGCATGCGCGACCC	C9orf40__NM_017998	103	172	152	101
C9orf40__NM_017998_GAAAGCGCAAAATCGACGCA	C9orf40__NM_017998	399	320	507	544
C9orf40__NM_017998_GGAGCCGCTTCCAAGGCACG	C9orf40__NM_017998	747	707	751	658
C9orf40__NM_017998_GGGGTCGCATCGCGCCAGGT	C9orf40__NM_017998	510	554	592	602
C9orf40__NM_017998_TCAATGTCTGCCAGATCAAT	C9orf40__NM_017998	422	356	374	361
C9orf41__NM_152420_AAAGTAAATATTCTGGTACC	C9orf41__NM_152420	44	4	57	30
C9orf41__NM_152420_AGACTGTGGGGGTCAACATC	C9orf41__NM_152420	435	265	355	253
C9orf41__NM_152420_ATGCTAGGTTATGCTTGTCA	C9orf41__NM_152420	841	1432	1091	930
C9orf41__NM_152420_ATTAATGCCTTCCGCTACTA	C9orf41__NM_152420	430	416	429	316
C9orf41__NM_152420_CTTCCAACTTTGTACTCAAC	C9orf41__NM_152420	314	343	342	315
C9orf41__NM_152420_GCCTTCCGCTACTACGGGTA	C9orf41__NM_152420	616	704	837	664
C9orf41__NM_152420_GTTCACCCGCTCATGCATAC	C9orf41__NM_152420	251	314	279	163
C9orf41__NM_152420_TCCATCAGTTTAGCAATAAC	C9orf41__NM_152420	237	245	415	283
C9orf41__NM_152420_TCCATGTCAAATGTAGATGC	C9orf41__NM_152420	284	162	390	237
C9orf41__NM_152420_TTAATGCCTTCCGCTACTAC	C9orf41__NM_152420	431	424	424	302
C9orf41__NM_152420_TTAGCACCAGTATGCATGAG	C9orf41__NM_152420	768	678	732	808
C9orf41__NM_152420_TTTAATGATTGGCTGGTAAC	C9orf41__NM_152420	48	143	63	38
C9orf43__NM_001278629_ACCAGAATGTACCTTTACTA	C9orf43__NM_001278629	740	640	987	745
C9orf43__NM_001278629_ACTCTGAGACAATAAAGAAT	C9orf43__NM_001278629	221	313	234	209
C9orf43__NM_001278629_ATGAGCCGAGGATTCGAGGA	C9orf43__NM_001278629	671	592	713	718
C9orf43__NM_001278629_ATGTCTACCACGGTGAGCAC	C9orf43__NM_001278629	98	110	133	161
C9orf43__NM_001278629_CAACTATCCGCCGCATTGAG	C9orf43__NM_001278629	2198	2414	2471	2658
C9orf43__NM_001278629_CCCTGAAGATGTTAGAAATA	C9orf43__NM_001278629	327	502	299	414
C9orf43__NM_001278629_CTCTCTGGAAATCAGTCCGC	C9orf43__NM_001278629	351	296	405	358
C9orf43__NM_001278629_GATGGCCCCTCTCAATGCGG	C9orf43__NM_001278629	362	379	455	357
C9orf43__NM_001278629_GCCTTAGTAAAGGTACATTC	C9orf43__NM_001278629	836	974	822	844
C9orf43__NM_001278629_GTCTCATTCAAATTCAACAC	C9orf43__NM_001278629	347	420	472	347
C9orf43__NM_001278629_GTGGTAGACATCTTAGATTC	C9orf43__NM_001278629	38	17	34	49
C9orf43__NM_001278629_TAAACTCCCAGTGCTCACCG	C9orf43__NM_001278629	868	974	994	1039
C9orf43__NM_001278630_CTAGAAATGGTTTAGGCCAA	C9orf43__NM_001278630	300	382	333	321
C9orf47__NM_001001938_CACCTGGAGCTCGCCCGATG	C9orf47__NM_001001938	2386	2100	2890	2450
C9orf47__NM_001001938_CCAAGCCCAGACCTCGGACC	C9orf47__NM_001001938	880	1003	990	1178
C9orf47__NM_001001938_CCGGACCTAGGGGATTGGT	C9orf47__NM_001001938	854	876	904	978
C9orf47__NM_001001938_CCTGACAGCGAGGGTTTGTT	C9orf47__NM_001001938	635	636	753	828
C9orf47__NM_001001938_CGCGCCACCCGCTAGGATGC	C9orf47__NM_001001938	1968	2230	2638	2219
C9orf47__NM_001001938_CGGTTCCGGGGAGGGGCAAC	C9orf47__NM_001001938	44	54	43	100
C9orf47__NM_001001938_CTGACAGCGAGGGTTTGTTT	C9orf47__NM_001001938	1828	2113	1949	2354
C9orf47__NM_001001938_GAAGGGCGAGCCGGACCCTA	C9orf47__NM_001001938	465	342	506	332
C9orf47__NM_001001938_GCCTGTCGCCCTGACAGCGA	C9orf47__NM_001001938	232	188	256	83
C9orf47__NM_001001938_GGCCTGGGAACAGGGGCCGA	C9orf47__NM_001001938	181	112	256	173
C9orf47__NM_001001938_TGTTTGGGCCCATTCTTAAT	C9orf47__NM_001001938	149	102	211	171
C9orf47__NM_001001938_TTGGGCCCATTCTTAATAGG	C9orf47__NM_001001938	656	574	851	554
C9orf47__NM_001142413_CGCACCCGGAGCGTTTACAA	C9orf47__NM_001142413	775	886	853	870
C9orf47__NM_001142413_GCCACCGGCATCCTAGCGGG	C9orf47__NM_001142413	824	852	820	677
C9orf47__NM_001142413_TGGGGCCACCGGCATCCTAG	C9orf47__NM_001142413	221	225	215	213
C9orf50__NM_199350_AAATCTTCGTAAGACTATCC	C9orf50__NM_199350	281	258	332	228
C9orf50__NM_199350_AGGACACAACGCTCCGCTAC	C9orf50__NM_199350	691	436	682	575
C9orf50__NM_199350_CCTTTGATGTTGCGGTGCTG	C9orf50__NM_199350	966	1105	1028	1133
C9orf50__NM_199350_CGTCGGAAGTCTCCGTCGCC	C9orf50__NM_199350	2	0	6	27
C9orf50__NM_199350_GCCTTCCCCCGAGGGGTTGT	C9orf50__NM_199350	459	361	574	568
C9orf50__NM_199350_GCTCCAGCAGACACCTATTC	C9orf50__NM_199350	907	666	988	803
C9orf50__NM_199350_GCTCCAGGAGAAGTGCGCCG	C9orf50__NM_199350	896	1256	1129	1127
C9orf50__NM_199350_GGACACAACGCTCCGCTACT	C9orf50__NM_199350	720	646	699	746
C9orf50__NM_199350_GGTTCACAATGACGCTCTGT	C9orf50__NM_199350	416	183	352	397
C9orf50__NM_199350_TCCAGCAGACACCTATTCTG	C9orf50__NM_199350	1272	859	1331	944
C9orf50__NM_199350_TCTCGTCAGCGAATCGCACC	C9orf50__NM_199350	573	716	591	431
C9orf50__NM_199350_TCTGGCGTCGACTTCGCCCA	C9orf50__NM_199350	1026	837	1145	770
C9orf57__NM_001128618_ACACGTCCCACTGATTTCTG	C9orf57__NM_001128618	563	643	654	814
C9orf57__NM_001128618_AGACAAAACCTGGTCAGTAC	C9orf57__NM_001128618	793	829	831	966
C9orf57__NM_001128618_ATCTTATTCGGCCGCTTAGG	C9orf57__NM_001128618	232	312	244	108
C9orf57__NM_001128618_CCTTAATGAAAAAGATAGGT	C9orf57__NM_001128618	235	255	220	155
C9orf57__NM_001128618_CTCTTAGGTGTTATCTTATT	C9orf57__NM_001128618	142	115	147	172
C9orf57__NM_001128618_GTTATCTTATTCGGCCGCTT	C9orf57__NM_001128618	384	356	569	651
C9orf57__NM_001128618_TTCTGATTTCCAAGCCGAAA	C9orf57__NM_001128618	8	0	8	17
C9orf62__NM_173520_CAGCCCTGCTGTCCGCTAGA	C9orf62__NM_173520	275	408	327	221
C9orf62__NM_173520_CATGAGAACAGAGATTTCGG	C9orf62__NM_173520	712	613	893	911
C9orf62__NM_173520_GAAAGAAACCAACCCCATTG	C9orf62__NM_173520	37	14	59	182
C9orf62__NM_173520_GGAGCCGCCACCGCCCCAAT	C9orf62__NM_173520	1909	2824	2612	3271
C9orf62__NM_173520_TGCAGCGTGGACAGAACGGC	C9orf62__NM_173520	428	436	480	508
C9orf62__NM_173520_TGGCAGTCGCTGAAAGTTTA	C9orf62__NM_173520	112	90	80	59
C9orf64__NM_032307_ACAGAGCCCTCGACGAAGGT	C9orf64__NM_032307	360	322	432	347
C9orf64__NM_032307_CCATGCCTTTAGTAGAAGAG	C9orf64__NM_032307	578	544	649	900
C9orf64__NM_032307_CCGACGAGGCCGCGGTCAAC	C9orf64__NM_032307	287	367	386	492
C9orf64__NM_032307_CCTCTCTTCTACTAAAGGCA	C9orf64__NM_032307	322	293	279	320
C9orf64__NM_032307_CGGGATGTGTTTATTGACAG	C9orf64__NM_032307	313	539	285	387
C9orf64__NM_032307_CTCGTCCTGCTCCGACCAAA	C9orf64__NM_032307	490	497	726	655
C9orf64__NM_032307_GATGTGTTTATTGACAGCGG	C9orf64__NM_032307	324	205	389	342
C9orf64__NM_032307_GGTCACTGTCGCGTAGTACG	C9orf64__NM_032307	595	443	589	589
C9orf64__NM_032307_GTCGAGGGCTCTGTTGACGG	C9orf64__NM_032307	643	691	684	424
C9orf64__NM_032307_TAGTACGAGGCACTAGTTAT	C9orf64__NM_032307	460	503	570	491
C9orf64__NM_032307_TATATTCCGAACCTGATCCA	C9orf64__NM_032307	288	269	267	224
C9orf64__NM_032307_TGACAGCGGAGGCGTACGGA	C9orf64__NM_032307	255	249	215	187
C9orf66__NM_152569_ACTCCGTGGCGCGCCCAACA	C9orf66__NM_152569	317	276	364	570
C9orf66__NM_152569_AGGCGCCTGGGTAACCGTGT	C9orf66__NM_152569	1485	1191	1531	1354
C9orf66__NM_152569_CCAGCGCCGACCGACAGACG	C9orf66__NM_152569	1032	1157	1217	1333
C9orf66__NM_152569_CCTAGAAGCCACCGAACCGC	C9orf66__NM_152569	681	580	878	656
C9orf66__NM_152569_CGCAAACCTCGTCTGTCGGT	C9orf66__NM_152569	4935	4608	5496	5487
C9orf66__NM_152569_CGGACGCGAATGGACTCGTC	C9orf66__NM_152569	1233	1250	1616	1959
C9orf66__NM_152569_CGGAGCGGGCGAGGTTGTTC	C9orf66__NM_152569	362	420	503	369
C9orf66__NM_152569_CGTTCGCGCTCAAGATCAAC	C9orf66__NM_152569	956	1302	1204	1418
C9orf66__NM_152569_GACGAGTCCATTCGCGTCCG	C9orf66__NM_152569	873	684	1069	1113
C9orf66__NM_152569_GGCCACCTGTAAGAACCTGG	C9orf66__NM_152569	1093	1040	1150	1209
C9orf66__NM_152569_GGCGCCTGGGTAACCGTGTT	C9orf66__NM_152569	1452	1919	1318	1721
C9orf66__NM_152569_TCCGCCCTCCAGGTTCTTAC	C9orf66__NM_152569	913	912	1020	1112
C9orf69__NM_001256526_ATGGTCTTCGTGGACATGTG	C9orf69__NM_001256526	127	128	110	78
C9orf69__NM_001256526_CGGTCATGCCCATTCCGCGG	C9orf69__NM_001256526	1711	2082	1816	2046
C9orf69__NM_001256526_CTCTTCTGCCTAGAGTACCT	C9orf69__NM_001256526	301	356	275	326
C9orf69__NM_001256526_GAGCGCACCCGCCGCGGAAT	C9orf69__NM_001256526	2064	2561	2363	2428
C9orf69__NM_001256526_TGGTCTTCGTGGACATGTGA	C9orf69__NM_001256526	0	0	4	13
C9orf72__NM_001256054_AGGAAGAATATGGATGCATA	C9orf72__NM_001256054	55	52	62	41
C9orf72__NM_001256054_ATATTCTTGGTCCTAGAGTA	C9orf72__NM_001256054	169	255	221	282
C9orf72__NM_001256054_ATCACTTCTCCAGTAAGCAT	C9orf72__NM_001256054	183	103	161	156
C9orf72__NM_001256054_CACCACTCTCTGCATTTCGA	C9orf72__NM_001256054	1289	1859	1299	1826
C9orf72__NM_001256054_CAGTGCAGAGAAAGTAAATA	C9orf72__NM_001256054	331	224	333	245
C9orf72__NM_001256054_GATGAAAGCAGTTCCATTAC	C9orf72__NM_001256054	663	799	845	1016
C9orf72__NM_001256054_GTCCAGAAGATATTCTTAGA	C9orf72__NM_001256054	296	217	431	431
C9orf72__NM_001256054_GTCCTAGAGTAAGGCACATT	C9orf72__NM_001256054	540	495	684	634
C9orf72__NM_001256054_TGATTCTGCTTCACATAACC	C9orf72__NM_001256054	22	83	25	46
C9orf72__NM_001256054_TGCACTGCTACCTACTACAA	C9orf72__NM_001256054	422	530	364	385
C9orf72__NM_001256054_TGGAGAAGTGATTCCTGTAA	C9orf72__NM_001256054	164	91	194	225
C9orf72__NM_001256054_TGTGGCTGTTCCGTTGTAGT	C9orf72__NM_001256054	1269	948	1365	1472
C9orf78__NM_016520_AAAAAATATCATTTCCACGG	C9orf78__NM_016520	340	400	433	403
C9orf78__NM_016520_AAGGCCCGGCCCTTGAGAGT	C9orf78__NM_016520	575	707	642	674
C9orf78__NM_016520_AGGACGCTTGCGGTTAGGAG	C9orf78__NM_016520	372	275	453	271
C9orf78__NM_016520_AGTTAAGCCAAAGAATGCAG	C9orf78__NM_016520	357	223	458	275
C9orf78__NM_016520_AGTTCAAGAAAATGAATAGG	C9orf78__NM_016520	141	97	189	126
C9orf78__NM_016520_CAAGCGTCCTGCTAACGAGA	C9orf78__NM_016520	407	329	439	385
C9orf78__NM_016520_CTCCGTGTCACCTACTCTCA	C9orf78__NM_016520	1150	1264	1140	1398
C9orf78__NM_016520_GAAAGGGATCGTGGAACATG	C9orf78__NM_016520	417	208	537	308
C9orf78__NM_016520_TCAGTTGCCTTCTCGGTTAGC	C9orf78__NM_016520	583	699	529	902
C9orf78__NM_016520_TCTCGTTAGCAGGACGCTTG	C9orf78__NM_016520	447	437	578	683
C9orf78__NM_016520_TGCATCCTCATCCCTTCGGT	C9orf78__NM_016520	1484	209	1644	358
C9orf78__NM_016520_TTAGCAGGACGCTTGCGGTT	C9orf78__NM_016520	422	190	522	310
C9orf84__NM_001080551_ACTTACACTTCAAAATAGCC	C9orf84__NM_001080551	101	229	110	142
C9orf84__NM_001080551_AGCAGTAGGGAGGGTACACA	C9orf84__NM_001080551	1070	980	1140	1261
C9orf84__NM_001080551_ATTTCCAATTAGCAGTAGGG	C9orf84__NM_001080551	166	150	193	143
C9orf84__NM_001080551_CAAATTTCCAATTAGCAGTA	C9orf84__NM_001080551	187	252	207	354
C9orf84__NM_001080551_CTAATGAATACTACATGATG	C9orf84__NM_001080551	207	191	240	278
C9orf84__NM_001080551_GATGAATTGGAGATATTAAG	C9orf84__NM_001080551	82	58	90	77
C9orf84__NM_001080551_GCATAATAAAGTCGCTCAAA	C9orf84__NM_001080551	348	610	375	463
C9orf84__NM_001080551_GTACCCTCCCTACTGCTAAT	C9orf84__NM_001080551	728	876	680	897
C9orf84__NM_001080551_TAAGCAAAAATGGGTTCAAA	C9orf84__NM_001080551	283	205	288	245
C9orf84__NM_001080551_TCATCAGAAAAACATAGATC	C9orf84__NM_001080551	23	1	13	3
C9orf84__NM_001080551_TCTAATTGAAGCACTTTCGT	C9orf84__NM_001080551	339	275	389	416
C9orf84__NM_001080551_TGTCCTTTGAAATCTAAAAG	C9orf84__NM_001080551	14	30	14	29
C9orf84__NM_173521_CCAGATTAATTGTGAATTTG	C9orf84__NM_173521	348	568	457	669
C9orf84__NM_173521_CCTCAAATTCACAATTAATC	C9orf84__NM_173521	777	1076	769	1154
C9orf84__NM_173521_GGTCAGTTTATACACCCACA	C9orf84__NM_173521	227	144	291	350
C9orf84__NM_173521_TGACCTCCTCTACTTCAATT	C9orf84__NM_173521	663	780	900	958
C9orf85__NM_182505_GAGCTCCCAGAAAGGCAACG	C9orf85__NM_182505	144	155	251	287
C9orf85__NM_182505_TGCGCAAACTTCAAGTTCAC	C9orf85__NM_182505	619	809	692	787
C9orf89__NM_032310_ACAGATCCTTACCAACAAGG	C9orf89__NM_032310	22	0	6	10
C9orf89__NM_032310_ACATGTCTGTCGATGATGAC	C9orf89__NM_032310	1544	1622	1469	2044
C9orf89__NM_032310_CACCTGGCGGATAGCAGTAC	C9orf89__NM_032310	699	648	715	833
C9orf89__NM_032310_CAGGACACGCCTTTCCTGAC	C9orf89__NM_032310	2787	3008	3130	3588
C9orf89__NM_032310_CCAAGGCATCCTTGCGTGTG	C9orf89__NM_032310	755	1218	762	1066
C9orf89__NM_032310_CGCCCATGGCCTGTCAGGAA	C9orf89__NM_032310	777	837	770	795
C9orf89__NM_032310_CTCCTGTACTGCTATCCGCC	C9orf89__NM_032310	789	626	618	642
C9orf89__NM_032310_CTGTACTGCTATCCGCCAGG	C9orf89__NM_032310	976	513	1051	835
C9orf89__NM_032310_GATTGCACAGAGCTAGACTC	C9orf89__NM_032310	70	64	101	42
C9orf89__NM_032310_GCTGCTCACTCAAGCGCCCA	C9orf89__NM_032310	194	197	134	185
C9orf89__NM_032310_GTCACAGAGCCGCACACGCA	C9orf89__NM_032310	543	641	480	568
C9orf89__NM_032310_TGATGACAGGCGAGAAACCG	C9orf89__NM_032310	194	108	178	186
C9orf9__NM_018956_ACTTCTGCTCGATGCTGCGA	C9orf9__NM_018956	476	693	540	768
C9orf9__NM_018956_CTTCTGCTCGATGCTGCGAA	C9orf9__NM_018956	151	166	193	239
C9orf9__NM_018956_GACGAGGCCCGGAACCACTA	C9orf9__NM_018956	468	401	438	373
C9orf9__NM_018956_GGCTGACCACGCCGCCGTAG	C9orf9__NM_018956	394	223	308	192
C9orf9__NM_018956_GGCTGCTTAAGTCGTTGCTT	C9orf9__NM_018956	598	1134	763	957
C9orf9__NM_018956_GTCGTTGCTTTGGCTAACGA	C9orf9__NM_018956	451	288	477	298
C9orf9__NM_018956_TCCATTCTTTCATTAAGTCT	C9orf9__NM_018956	314	348	274	271
C9orf9__NM_018956_TCCTAGACTTAATGAAAGAA	C9orf9__NM_018956	33	21	42	35
C9orf91__NM_153045_ACAGCAAAGGCCAGCCGCAT	C9orf91__NM_153045	961	1011	1197	1052
C9orf91__NM_153045_AGGATGCCAGAGTGTGATTC	C9orf91__NM_153045	405	377	456	564
C9orf91__NM_153045_AGGTCCCGGCTGACCAGTAC	C9orf91__NM_153045	899	981	1020	1034
C9orf91__NM_153045_CAGGCCCCTCCGCTGTTGCA	C9orf91__NM_153045	1828	2204	1985	2630
C9orf91__NM_153045_CCAGCACAAGAGTCAAGGTC	C9orf91__NM_153045	691	732	841	984
C9orf91__NM_153045_CCCCAAGGCAAACATCTGAC	C9orf91__NM_153045	957	1395	1058	1305
C9orf91__NM_153045_CTCGTTAGGACAAGAATTGC	C9orf91__NM_153045	213	272	260	219
C9orf91__NM_153045_GCACCCATCTCCTTCGACCT	C9orf91__NM_153045	2098	1375	2397	2200
C9orf91__NM_153045_GGAGGGGGGCAATGGGCCAG	C9orf91__NM_153045	302	277	444	428
C9orf91__NM_153045_GGTGAGCCCTGCAACAGCGG	C9orf91__NM_153045	373	291	335	253
C9orf91__NM_153045_GTGAACCACAGGTGGACCCG	C9orf91__NM_153045	27	65	44	37
C9orf91__NM_153045_TCTCCATGACAACACACAAC	C9orf91__NM_153045	407	448	367	555
C9orf92__NM_001271829_CATTAAAAGTAAAAAGGGGT	C9orf92__NM_001271829	166	312	174	198
C9orf92__NM_001271829_CGAGCCATCTCGCGTCTAGC	C9orf92__NM_001271829	159	197	196	288
C9orf92__NM_001271829_CTATGTGAGAAAACACCACC	C9orf92__NM_001271829	450	707	470	555
C9orf92__NM_001271829_GCCATCTCGCGTCTAGCTGG	C9orf92__NM_001271829	1349	1118	1492	1668
C9orf92__NM_001271829_GCCCCCAGCTAGAGCGCGAGA	C9orf92__NM_001271829	495	652	611	710
CA1__NM_001128829_AGGGGACTGGTTATTTCCAT	CA1__NM_001128829	354	380	420	382
CA1__NM_001128829_AGGGGTCAAAATTTGTGAAT	CA1__NM_001128829	283	344	320	392
CA1__NM_001128829_CAAGTTACACTCTCATAAAG	CA1__NM_001128829	236	165	300	139
CA1__NM_001128829_CGTTGTCCTCAAAATTTACA	CA1__NM_001128829	153	128	211	230
CA1__NM_001128829_GAGGACAACGATAACCGATC	CA1__NM_001128829	284	124	194	124
CA1__NM_001128829_GCCAAAGAAATTATCAATGT	CA1__NM_001128829	854	1066	943	1137
CA1__NM_001128829_GGTTCAGAACATACAGTGGA	CA1__NM_001128829	332	281	300	358
CA1__NM_001128829_TGCCAAACCATCAGCCTTTG	CA1__NM_001128829	672	652	779	862
CA1__NM_001128829_TGGTTATTTCCATTGGCAAT	CA1__NM_001128829	0	0	0	0
CA1__NM_001128829_TGTAACTTGGATCATCTGTA	CA1__NM_001128829	350	341	378	471
CA1__NM_001128829_TTGTCATCATATCCCCAGTC	CA1__NM_001128829	297	336	402	532
CA1__NM_001128829_TTTAGCTTCACGTAGCTCAC	CA1__NM_001128829	0	0	2	0
CA10__NM_001082533_AAGACACGTATCCCTTCGCC	CA10__NM_001082533	1395	1284	1660	1432
CA10__NM_001082533_AATTTCATCGTCTGCATATC	CA10__NM_001082533	260	411	363	390
CA10__NM_001082533_ACACCTCTTCGCATCAACAC	CA10__NM_001082533	646	556	763	637
CA10__NM_001082533_CACTTGGTCAACATATCTGG	CA10__NM_001082533	1057	1070	1059	1131
CA10__NM_001082533_CCCCCCGGTGTTGATGCGAAG	CA10__NM_001082533	974	1178	1020	1101
CA10__NM_001082533_CCTCTTCGCATCAACACGGG	CA10__NM_001082533	366	393	469	356
CA10__NM_001082533_GAGGATGGAAATAGTCTGGG	CA10__NM_001082533	106	57	154	143
CA10__NM_001082533_GGCCCATGACATACAGCCAC	CA10__NM_001082533	1326	1445	1630	1736
CA10__NM_001082533_GGGGATTGGTGAACTCAGCT	CA10__NM_001082533	191	262	154	314
CA10__NM_001082533_GGGGTCGAAGATCATGTGAC	CA10__NM_001082533	735	936	889	1090
CA10__NM_001082533_GTGTCTTCCAGTGTTGTACA	CA10__NM_001082533	291	449	351	481
CA10__NM_001082533_TGATATGCAGACGATGAAAT	CA10__NM_001082533	161	139	151	108
CA11__NM_001217_CCCAGAGTACCAGCGCTCGA	CA11__NM_001217	298	327	346	365
CA11__NM_001217_CCTCCAGTGCTGAGCCTTAA	CA11__NM_001217	903	808	879	1136
CA11__NM_001217_CGACCTGTGGTCAATGTGTC	CA11__NM_001217	795	905	890	1260
CA11__NM_001217_CGGACCAGCACCTGACCCCG	CA11__NM_001217	1834	2032	2258	2050
CA11__NM_001217_CTCCAGGGAAACTTCGTGCC	CA11__NM_001217	554	511	615	638
CA11__NM_001217_CTCCAGTGCTGAGCCTTAAT	CA11__NM_001217	236	273	283	299
CA11__NM_001217_CTGCTGTTTGGAGCTCGCGA	CA11__NM_001217	218	151	221	211
CA11__NM_001217_GCCCAGAGTACCAGCGCTCG	CA11__NM_001217	374	310	401	540
CA11__NM_001217_GGTGCTGGTCCGATGTGAGC	CA11__NM_001217	236	309	319	342
CA11__NM_001217_GTGCTGGTCCGATGTGAGCT	CA11__NM_001217	523	353	546	572
CA11__NM_001217_GTTGATCTGATGTTCCGAGC	CA11__NM_001217	206	227	364	352
CA11__NM_001217_TCCAGTGCTGAGCCTTAATG	CA11__NM_001217	361	307	516	450
CA12__NM_001218_AAGAAGTACCCGTCGTGTGG	CA12__NM_001218	271	238	289	171
CA12__NM_001218_ATGTCACTGTGCAGGTCTAT	CA12__NM_001218	431	423	494	632
CA12__NM_001218_ATTGTAGCCTTGGAACTCGA	CA12__NM_001218	387	326	381	395
CA12__NM_001218_CAAGAAGTACCCGTCGTGTG	CA12__NM_001218	1415	1714	1369	1835
CA12__NM_001218_CCAAGAAGTACCCGTCGTGT	CA12__NM_001218	1661	1414	1885	1516
CA12__NM_001218_CTCACCTGAATGGCCATTGT	CA12__NM_001218	1141	1003	1372	1338
CA12__NM_001218_CTTACCAAAATAAGTCCACT	CA12__NM_001218	360	289	457	529
CA12__NM_001218_GCAGCAGGCCCCCACACGAC	CA12__NM_001218	1175	1040	1254	1051
CA12__NM_001218_TCCAAGAAGTACCCGTCGTG	CA12__NM_001218	398	373	310	285
CA12__NM_001218_TGAGGCTGGCGTCATACTGG	CA12__NM_001218	680	433	609	578
CA12__NM_001218_TGGCAGACAGATTGTAGCCT	CA12__NM_001218	203	221	144	221
CA12__NM_001218_TTGTAGCCTTGGAACTCGAA	CA12__NM_001218	142	217	194	89
CA13__NM_198584_ACTTGATACTAAGTGGTCGG	CA13__NM_198584	592	377	644	669
CA13__NM_198584_CATACTTGATACTAAGTGGT	CA13__NM_198584	406	335	516	548
CA13__NM_198584_CTTGATACTAAGTGGTCGGA	CA13__NM_198584	156	153	176	162
CA13__NM_198584_GCAAAAGATTACTGACACTT	CA13__NM_198584	1318	1556	1520	1977
CA13__NM_198584_GCACAGGAGACTGCGAAATT	CA13__NM_198584	781	821	889	1006
CA13__NM_198584_GGCTCCGAGCACATAGTAGA	CA13__NM_198584	405	503	389	429
CA13__NM_198584_GGTGGAACTGTAAGAGAACC	CA13__NM_198584	95	120	167	110
CA13__NM_198584_TCACTGGAAGCTACAGGTTA	CA13__NM_198584	540	529	537	615
CA13__NM_198584_TGAATCGAGTTTGTTTACCC	CA13__NM_198584	151	127	141	118
CA13__NM_198584_TGAGCTGCCTCAACAAAGCT	CA13__NM_198584	56	40	74	32
CA13__NM_198584_TGGAAGCTACAGGTTACGGC	CA13__NM_198584	64	58	59	69
CA13__NM_198584_TGGGGATACCGCGAGCACAA	CA13__NM_198584	51	97	56	135
CA14__NM_012113_ACTGTCTGTCTGAATATCGA	CA14__NM_012113	819	556	854	695
CA14__NM_012113_AGGTCAACACTGGACGTATG	CA14__NM_012113	443	288	431	523
CA14__NM_012113_CCACCCAGATACAGGGTAGA	CA14__NM_012113	781	818	837	787
CA14__NM_012113_CCAGGCTGGTCATATCCGTG	CA14__NM_012113	1174	840	1248	1083
CA14__NM_012113_CTGCCCTCTACCCTGTATCT	CA14__NM_012113	1016	909	941	611
CA14__NM_012113_CTGTCTGTCTGAATATCGAT	CA14__NM_012113	636	490	531	433
CA14__NM_012113_GCCAGGCTGGTCATATCCGT	CA14__NM_012113	1954	1118	1726	1181
CA14__NM_012113_GGTCAACACTGGACGTATGA	CA14__NM_012113	532	381	496	360
CA14__NM_012113_GTTTCCACACTCAGGGTAAG	CA14__NM_012113	117	203	158	236
CA14__NM_012113_TCCACCCAGATACAGGGTAG	CA14__NM_012113	395	297	466	512
CA14__NM_012113_TGCCAGGCTGGTCATATCCG	CA14__NM_012113	659	399	743	545
CA14__NM_012113_TGTCCTGGGCATCCTAATTG	CA14__NM_012113	464	180	456	268
CA2__NM_000067_AAACTAGACACAAAGCAACC	CA2__NM_000067	188	184	190	277
CA2__NM_000067_AGCTAAGCACAGATCTACCT	CA2__NM_000067	508	570	581	509
CA2__NM_000067_AGGACCTGAGCACTGGCATA	CA2__NM_000067	187	95	127	130
CA2__NM_000067_CAATGGTCATGCTTTCAACG	CA2__NM_000067	892	1044	1123	1081
CA2__NM_000067_CAGTGAACCAAGTGAAGCTA	CA2__NM_000067	207	148	203	300
CA2__NM_000067_CATTATTTGGATACAGACTA	CA2__NM_000067	239	342	352	396
CA2__NM_000067_GACTTTGTTCACAGCATGTA	CA2__NM_000067	163	269	194	240
CA2__NM_000067_GCACATCAACAACTTTCTGA	CA2__NM_000067	1670	2130	1853	2421
CA2__NM_000067_GCTCAATTTATCTTGTGCTA	CA2__NM_000067	1024	1564	1242	1399
CA2__NM_000067_GTAGATCTGTGCTTAGCTAA	CA2__NM_000067	43	77	30	38
CA2__NM_000067_TAGGTTGGCAGCGCTAAACC	CA2__NM_000067	25	27	19	5
CA2__NM_000067_TATGAGTGTCGATGTCAACA	CA2__NM_000067	419	347	407	291
CA2__NM_000067_TGAGCTAGTTAAGGCAAATC	CA2__NM_000067	480	454	463	650
CA2__NM_000067_TGGGTAGGTCCAGTAATCCA	CA2__NM_000067	86	29	68	81
CA2__NM_000067_TTAGGTTGGCAGCGCTAAAC	CA2__NM_000067	144	182	94	75
CA2__NM_000067_TTCACTGGAACACCAAATAT	CA2__NM_000067	65	97	67	102
CA2__NM_000067_TTTCCCAAAATCCCCATATT	CA2__NM_000067	142	161	217	214
CA3__NM_005181_AGTTGCTCACAAGAGGCACT	CA3__NM_005181	159	95	185	102
CA3__NM_005181_ATCATCAAATACAACTCGGC	CA3__NM_005181	277	366	251	308
CA3__NM_005181_ATTGGACAAGATTAAGACAA	CA3__NM_005181	132	168	126	91
CA3__NM_005181_CATGACCGTGAGCTCTGACC	CA3__NM_005181	762	783	758	698
CA3__NM_005181_CCAAGACCATCCTGAATAAT	CA3__NM_005181	607	760	720	555
CA3__NM_005181_CCATGGTCTGTGTCTTATGA	CA3__NM_005181	741	864	872	1043
CA3__NM_005181_GAAGATGAAACTGGCGAAGT	CA3__NM_005181	507	322	629	499
CA3__NM_005181_GATCCCATCGCGCTGCTTCA	CA3__NM_005181	550	837	799	735
CA3__NM_005181_GCCAAGACCATCCTGAATAA	CA3__NM_005181	555	943	672	664
CA3__NM_005181_TGAAACTGGCGAAGTCGGTA	CA3__NM_005181	207	263	248	237
CA3__NM_005181_TGCATACTTGACTCCATCCA	CA3__NM_005181	257	426	292	330
CA3__NM_005181_TGGGGCTCTTCGGATGATCA	CA3__NM_005181	95	49	57	138
CA4__NM_000717_CAACTGTTTGGCCTGGTATG	CA4__NM_000717	949	707	1112	983
CA4__NM_000717_CGACTTGCCATATAAGGGCT	CA4__NM_000717	1302	1633	1548	2284
CA4__NM_000717_CGAGCCCTTATATGGCAAGT	CA4__NM_000717	975	935	1001	772
CA4__NM_000717_CTGGTCCGACTTGCCATATA	CA4__NM_000717	435	311	421	357
CA4__NM_000717_CTGTGCTCCGAGCCCTTATA	CA4__NM_000717	439	505	451	388
CA4__NM_000717_GCAAAGGTGGACAAAAAACT	CA4__NM_000717	128	171	130	89
CA4__NM_000717_GCAACTGTTTGGCCTGGTAT	CA4__NM_000717	1559	1541	1716	1718
CA4__NM_000717_GTCCAAAATAACGGGCACTC	CA4__NM_000717	269	188	213	218
CA4__NM_000717_TCAGTGCCAGTCAAGTGGGG	CA4__NM_000717	275	335	262	250
CA4__NM_000717_TGCAACTGTTTGGCCTGGTA	CA4__NM_000717	403	451	420	451
CA4__NM_000717_TGGTCCGACTTGCCATATAA	CA4__NM_000717	0	0	0	0
CA4__NM_000717_TGTTTGGCCTGGTATGGGGC	CA4__NM_000717	233	187	262	303
CA5A__NM_001739_ACTGGGGAGCAGTGAACGAG	CA5A__NM_001739	375	216	448	221
CA5A__NM_001739_CACTGGGGAGCAGTGAACGA	CA5A__NM_001739	423	275	437	397
CA5A__NM_001739_CAGGCAGGATGCCGCTTCAT	CA5A__NM_001739	442	365	515	448
CA5A__NM_001739_CCACTGGGGAGCAGTGAACG	CA5A__NM_001739	812	689	965	653
CA5A__NM_001739_CCAGAAGGAGCCCGTTGAAG	CA5A__NM_001739	994	524	1023	389
CA5A__NM_001739_CTCATCGAACGACTCCAGAG	CA5A__NM_001739	411	161	398	259
CA5A__NM_001739_CTCTCTGGAGTCGTTCGATG	CA5A__NM_001739	277	409	404	294
CA5A__NM_001739_CTTGCCGGAAATAAAACATA	CA5A__NM_001739	472	646	527	586
CA5A__NM_001739_GAAATTGCTTCAGTCTGTAG	CA5A__NM_001739	374	387	378	493
CA5A__NM_001739_GACGATGCACCGAGGCATC	CA5A__NM_001739	9	0	8	14
CA5A__NM_001739_GGTCACCTGGATCATCCAGA	CA5A__NM_001739	261	42	240	69
CA5A__NM_001739_TGGGATTACTGGACCTACGC	CA5A__NM_001739	71	64	106	58
CA5B__NM_007220_AATCGGTAGTTGTGTTCCAG	CA5B__NM_007220	341	491	335	430
CA5B__NM_007220_ACCCATCAACATTCGGTGGA	CA5B__NM_007220	637	647	768	756
CA5B__NM_007220_ATACTTGTACTTACAAAACC	CA5B__NM_007220	101	149	133	101
CA5B__NM_007220_CACCCATCAACATTCGGTGG	CA5B__NM_007220	547	462	605	546
CA5B__NM_007220_CCTCTCCAGGCAAATTGCTG	CA5B__NM_007220	536	827	492	487
CA5B__NM_007220_CTGGCATGAATCTCGGAATC	CA5B__NM_007220	618	632	622	840
CA5B__NM_007220_GCCAGTCACCCATCAACATT	CA5B__NM_007220	907	991	993	1294
CA5B__NM_007220_GGTGGCTGGGTCATAAGAGA	CA5B__NM_007220	317	362	367	344
CA5B__NM_007220_GTCCCTCCACCGAATGTTGA	CA5B__NM_007220	825	1018	916	1207
CA5B__NM_007220_TCCCTCCACCGAATGTTGAT	CA5B__NM_007220	968	1001	1189	982
CA5B__NM_007220_TGGGGGGCCATCGATGCCTG	CA5B__NM_007220	256	517	264	271
CA5B__NM_007220_TTCAGCTGCACTTAGTGCAT	CA5B__NM_007220	3	13	8	26
CA6__NM_001215_AGTCGCCTATCAACCTACAG	CA6__NM_001215	656	580	687	654
CA6__NM_001215_CAACATCAAGTACCCAGGTA	CA6__NM_001215	530	483	651	776
CA6__NM_001215_CACATGTCTGATCCCGTCCA	CA6__NM_001215	1825	1805	1966	1977
CA6__NM_001215_CATATTGAGCCCCTTCAAGG	CA6__NM_001215	201	109	183	163
CA6__NM_001215_CCCAGCAGATGCACTTTCAC	CA6__NM_001215	860	910	928	1039
CA6__NM_001215_CGGGATCAGACATGTGATCG	CA6__NM_001215	508	836	685	946
CA6__NM_001215_CTGAACGTCAAGGCCAGTCA	CA6__NM_001215	393	404	465	555
CA6__NM_001215_CTGGCCAACATCAAGTACCC	CA6__NM_001215	127	152	137	175
CA6__NM_001215_CTTACCTGGGTACTTGATGT	CA6__NM_001215	1440	1804	1645	1565
CA6__NM_001215_GGGCACTGGACGAAGCGCAC	CA6__NM_001215	324	384	370	517
CA6__NM_001215_TGATATAGCCCAAGATGCGC	CA6__NM_001215	233	219	252	230
CA6__NM_001215_TTGAAGGGGCTCAATATGAC	CA6__NM_001215	327	363	330	288
CA6__NM_001270501_CCATCCACAACGATTACCGC	CA6__NM_001270501	1124	923	1209	1350
CA6__NM_001270501_CTCACCCTGATTCGGGAAGT	CA6__NM_001270501	926	798	876	854
CA6__NM_001270501_GGAATCCAACTTCCCGAATC	CA6__NM_001270501	314	227	310	219
CA6__NM_001270501_TTCCACCACTCTGTGGTTCA	CA6__NM_001270501	46	78	42	19
CA6__NM_001270502_CAATCTTATGTAGGTAAAAC	CA6__NM_001270502	181	413	252	308
CA7__NM_001014435_ACTGAAGGGCCGCGTGGTAA	CA7__NM_001014435	1443	1585	1412	1367
CA7__NM_001014435_ATCTGTCAGACGATTCATGC	CA7__NM_001014435	371	351	345	332
CA7__NM_001014435_CTGGGGCAAGAAGCACGATG	CA7__NM_001014435	495	377	640	621
CA7__NM_001014435_GAGGCGGTAGGGCCCTTCCA	CA7__NM_001014435	510	90	518	178
CA7__NM_001014435_TCTGTCAGACGATTCATGCT	CA7__NM_001014435	2022	2383	2179	1987
CA7__NM_001014435_TGGGGCAAGAAGCACGATGT	CA7__NM_001014435	155	199	149	103
CA7__NM_005182_ACTCAAGCAATAATTAGAGG	CA7__NM_005182	323	460	312	330
CA7__NM_005182_AGCAGACACCAAACCATCTG	CA7__NM_005182	271	254	397	317
CA7__NM_005182_ATCTGCCTAGCCGGCCACTA	CA7__NM_005182	299	368	281	310
CA7__NM_005182_CAGAAGAGGTAATGCCGAGA	CA7__NM_005182	386	256	476	398
CA7__NM_005182_CTCAAGCAATAATTAGAGGT	CA7__NM_005182	352	437	397	377
CA7__NM_005182_GAAGGGTGCCCCAGACTCAA	CA7__NM_005182	317	287	360	401
CA7__NM_005182_GAGACTCAAGCAATAATTAG	CA7__NM_005182	267	280	237	194
CA7__NM_005182_GCAGAAGAGGTAATGCCGAG	CA7__NM_005182	786	660	891	814
CA7__NM_005182_TAGTGGCCGGCTAGGCAGAT	CA7__NM_005182	254	185	224	377
CA7__NM_005182_TGGATGGGACCCTTGAGTCT	CA7__NM_005182	215	237	213	285
CA7__NM_005182_TTGAGAAGATGGTGCCCTAG	CA7__NM_005182	522	796	681	571
CA8__NM_004056_AAAGTGTTAGGATTAAAGCA	CA8__NM_004056	160	95	105	98
CA8__NM_004056_ACCCTCTGCTGCGGGATTAC	CA8__NM_004056	1044	1241	1158	1094
CA8__NM_004056_ATGAAGCTCAGGTCCGCCAT	CA8__NM_004056	559	491	645	428
CA8__NM_004056_CCCTCTGCTGCGGGATTACT	CA8__NM_004056	827	931	959	1053
CA8__NM_004056_CCTCCAAGATATTCAGTATA	CA8__NM_004056	106	85	123	150
CA8__NM_004056_GACTGTGAAGTCACCAATGA	CA8__NM_004056	134	115	159	177
CA8__NM_004056_GCAATGATGGCGATTCCGTG	CA8__NM_004056	1170	1431	1342	1509
CA8__NM_004056_GGGATATAGTTAAAGGGTAT	CA8__NM_004056	159	156	196	172
CA8__NM_004056_GGTGTTTCCTGATGCTAATG	CA8__NM_004056	214	296	292	301
CA8__NM_004056_TCACCTGGTAATAAAGTGTT	CA8__NM_004056	675	612	681	671
CA8__NM_004056_TCTCTTGAGTTTAGGTTAAT	CA8__NM_004056	337	412	414	505
CA8__NM_004056_TGGTGTTTCCTGATGCTAAT	CA8__NM_004056	73	122	41	70
CA9__NM_001216_AACCCCAGAATAATGCCCAC	CA9__NM_001216	693	490	881	706
CA9__NM_001216_ACAGCAGCAGTTGCACAGTG	CA9__NM_001216	634	429	742	808
CA9__NM_001216_AGCTGGGGGCGGATATCCAC	CA9__NM_001216	660	638	698	753
CA9__NM_001216_CAGGGGATGACCAGAGTCAT	CA9__NM_001216	775	489	778	562
CA9__NM_001216_CCCCAAGGCCTCGTCAACTC	CA9__NM_001216	194	441	226	305
CA9__NM_001216_CGCCTTTGCCAGAGTTGACG	CA9__NM_001216	1051	1400	1271	1817
CA9__NM_001216_GAACTGCGCCTGCGCAACAA	CA9__NM_001216	76	151	39	52
CA9__NM_001216_GACCAGAGTCATTGGCGCTA	CA9__NM_001216	505	461	621	749
CA9__NM_001216_GCTGGGGGCGGATATCCACC	CA9__NM_001216	373	350	499	372
CA9__NM_001216_TCTCCAGGAGCCTCAACAGT	CA9__NM_001216	110	116	144	202
CA9__NM_001216_TGCATCGGGGGCAACCCTG	CA9__NM_001216	786	868	846	687
CA9__NM_001216_TGTCCCTGTGGGCATTATTC	CA9__NM_001216	244	169	212	322
CAAP1__NM_001167575_AGCAGATGATGGTTCTAAGA	CAAP1__NM_001167575	92	160	171	142
CAAP1__NM_001167575_AGCTGCTAGAACTTGAGATG	CAAP1__NM_001167575	454	446	562	556
CAAP1__NM_001167575_CAATGGAATGGACTCTGATA	CAAP1__NM_001167575	249	255	327	250
CAAP1__NM_001167575_CAGCAGTGCTTCTGTATTAT	CAAP1__NM_001167575	203	113	213	227
CAAP1__NM_001167575_GCTTATGACAGCGACATAGA	CAAP1__NM_001167575	302	208	275	222
CAAP1__NM_001167575_TTTAGTGATATGATTCTAGGAC	CAAP1__NM_001167575	216	210	231	107
CAAP1__NM_024828_ACGGCGCAAACGTAGCAGTC	CAAP1__NM_024828	349	314	477	526
CAAP1__NM_024828_CAACGCGGGTACGATGTCCG	CAAP1__NM_024828	908	984	1014	970
CAAP1__NM_024828_CCCGGACATCGTACCCGCGT	CAAP1__NM_024828	2148	1492	2597	2257
CAAP1__NM_024828_GCCAACGCGGGTACGATGTC	CAAP1__NM_024828	805	673	736	664
CAAP1__NM_024828_TACCGACTCTTCCAGCGTCT	CAAP1__NM_024828	511	287	466	358
CAAP1__NM_024828_TTTGCGCCGTTTCTCCCGGG	CAAP1__NM_024828	830	683	954	960
CAB39__NM_001130849_AGGATGGGCTGCGTCTTGTT	CAB39__NM_001130849	1076	809	1141	907
CAB39__NM_001130849_CCAGAAATAGCTCTAAATTG	CAB39__NM_001130849	22	0	20	27
CAB39__NM_001130849_CCGTCATGCCGTTCCCGTTT	CAB39__NM_001130849	3282	3472	3961	4371
CAB39__NM_001130849_GAGCTGTAAATCAGCTACCA	CAB39__NM_001130849	20	1	20	7
CAB39__NM_001130849_GCAGGTTCATCATTAATTTG	CAB39__NM_001130849	466	506	583	810
CAB39__NM_001130849_GCCATGAAAGAAATTCTGTA	CAB39__NM_001130849	424	506	565	427
CAB39__NM_001130849_GCCGTCATGCCGTTCCCGTT	CAB39__NM_001130849	4267	4315	4387	5482
CAB39__NM_001130849_GCTCAAGAACTCTATAATAG	CAB39__NM_001130849	165	235	243	209
CAB39__NM_001130849_GCTGAGGAACTCTATGAGTT	CAB39__NM_001130849	555	364	794	418
CAB39__NM_001130849_GTGCAGATGTATTCAACAGT	CAB39__NM_001130849	349	297	472	386
CAB39__NM_001130849_TGAGACTTCCCAAACGGGAA	CAB39__NM_001130849	0	0	0	0
CAB39__NM_001130849_TTGAGGAGGATGTCTAGGAT	CAB39__NM_001130849	766	572	937	898
CAB39L__NM_001079670_AACATCTTGAGAAGACAGAT	CAB39L__NM_001079670	116	79	124	94
CAB39L__NM_001079670_ACCCTTTGAGGAGCATAAAC	CAB39L__NM_001079670	281	163	265	264
CAB39L__NM_001079670_ACTAACCAGACATAAAGTGT	CAB39L__NM_001079670	1437	1328	1486	1613
CAB39L__NM_001079670_CAGCATAATCCCACAACGTA	CAB39L__NM_001079670	19	9	13	37
CAB39L__NM_001079670_CAGCTATCAGTGTCACTAGC	CAB39L__NM_001079670	369	529	396	503
CAB39L__NM_001079670_CCACAGATTGCCTTACGTTG	CAB39L__NM_001079670	1482	1637	1516	2241
CAB39L__NM_001079670_GAAAATCCTGAAAGACAATT	CAB39L__NM_001079670	236	260	302	377
CAB39L__NM_001079670_GCACTAATATACTCCACAGT	CAB39L__NM_001079670	1434	1452	1590	1417
CAB39L__NM_001079670_TCACAAAACACAGCCTATTG	CAB39L__NM_001079670	599	669	945	809
CAB39L__NM_001079670_TGCTACCAACACTTTATGTC	CAB39L__NM_001079670	462	452	439	578
CABIN1__NM_001199281_AAAGAGGGGTTGAAACACCC	CABIN1__NM_001199281	50	82	59	55
CABIN1__NM_001199281_AAGCTTCGGAGAAGGATTGC	CABIN1__NM_001199281	319	279	280	333
CABIN1__NM_001199281_AAGGATTGCCGGTACAGCAA	CABIN1__NM_001199281	97	47	74	77
CABIN1__NM_001199281_CAAGAGCTCATGGTAGGCTT	CABIN1__NM_001199281	374	311	469	337
CABIN1__NM_001199281_CATGTCCAATCTTATACCAG	CABIN1__NM_001199281	912	783	991	762
CABIN1__NM_001199281_CTCACTTGTGTAATCACTGA	CABIN1__NM_001199281	877	1111	987	1095
CABIN1__NM_001199281_GGAGCAGCCTTGCTCCGGA	CABIN1__NM_001199281	1322	1345	1279	1672
CABIN1__NM_001199281_GGTTATCCAAACAGGGCCAG	CABIN1__NM_001199281	48	53	57	31
CABIN1__NM_001199281_GTCAACCTCTGGTATAAGAT	CABIN1__NM_001199281	478	686	438	620
CABIN1__NM_001199281_GTGATTAGGTTATCCAAACA	CABIN1__NM_001199281	140	88	196	271
CABIN1__NM_001199281_TGGAGTTCTACTTAGAGGTG	CABIN1__NM_001199281	20	3	15	2
CABIN1__NM_001199281_TGTAATCACTGAAGGGTGTAC	CABIN1__NM_001199281	203	138	256	206
CABLES1__NM_001100619_ACATGCGGCAACACGATACC	CABLES1__NM_001100619	692	580	672	784
CABLES1__NM_001100619_ACTTGGTACTGTCGCGATA	CABLES1__NM_001100619	402	450	546	294
CABLES1__NM_001100619_AGGATCTTCTGCCACTGATA	CABLES1__NM_001100619	273	210	317	188
CABLES1__NM_001100619_AGGGACTTGAAGTTGGACGG	CABLES1__NM_001100619	376	336	427	308
CABLES1__NM_001100619_CATTTGTGGGTAACAGAAAT	CABLES1__NM_001100619	228	413	201	306
CABLES1__NM_001100619_CGGCTGCGGGGTTAGCCGCT	CABLES1__NM_001100619	196	241	264	146
CABLES1__NM_001100619_CTCACCAACATCTCGCTGGA	CABLES1__NM_001100619	411	360	386	649
CABLES1__NM_001100619_CTTCCTCACCAACATCTCGC	CABLES1__NM_001100619	824	722	917	803
CABLES1__NM_001100619_GACGGAGGAAGACAATCAAC	CABLES1__NM_001100619	323	235	294	210
CABLES1__NM_001100619_GGAAGCGCGCATGGACCCG	CABLES1__NM_001100619	1327	1101	1396	1485
CABLES1__NM_001100619_GTTCTCCTCAATATCTTCCA	CABLES1__NM_001100619	56	25	105	64
CABLES1__NM_001100619_TTTGAAAGAGATCATTGGTC	CABLES1__NM_001100619	100	103	92	81
CABLES1__NM_001256438_ATCTCTTGGATGACCCCCAG	CABLES1__NM_001256438	119	147	183	157
CABLES1__NM_001256438_CTGAGTGTCAGCTTAATGTG	CABLES1__NM_001256438	315	267	324	218
CABLES1__NM_001256438_GTGGCTCAGGTTACGGAACT	CABLES1__NM_001256438	79	86	49	84
CABLES1__NM_001256438_TAGTGACCTGGGAGACTTTA	CABLES1__NM_001256438	156	221	178	168
CABLES1__NM_001256438_TGACACTCAGCAAAATTAGG	CABLES1__NM_001256438	159	85	162	144
CABLES1__NM_138375_TCCTTGAATGCTTTCTAAGA	CABLES1__NM_138375	225	179	191	170
CABLES2__NM_031215_ACATGAGGCAGTACGACACC	CABLES2__NM_031215	746	608	768	692
CABLES2__NM_031215_ACCTCCTGGATGACCCGCAG	CABLES2__NM_031215	573	700	642	642
CABLES2__NM_031215_ACTTCGCATAAGACACGACC	CABLES2__NM_031215	561	459	660	484
CABLES2__NM_031215_CAGAAGCAGAGGCACCCGTC	CABLES2__NM_031215	1157	944	1326	1318
CABLES2__NM_031215_CCACCGGCCTCGGCCTGGAT	CABLES2__NM_031215	576	776	686	661
CABLES2__NM_031215_CCTCATCTTTGCGTCGTACA	CABLES2__NM_031215	1070	1286	1135	1534
CABLES2__NM_031215_CGTCTCTGTGTCTTCCGAGA	CABLES2__NM_031215	487	334	706	396
CABLES2__NM_031215_CTGGCTGGTGCCGACTTGGT	CABLES2__NM_031215	1166	788	1345	1194
CABLES2__NM_031215_CTGTGCTGGAGCGCATCCCA	CABLES2__NM_031215	431	261	571	475
CABLES2__NM_031215_GCCTTCTCGGTCCTGCCCTA	CABLES2__NM_031215	1335	1085	1767	1432
CABLES2__NM_031215_TCTGGATCACCGAGACATAA	CABLES2__NM_031215	256	146	321	291
CABLES2__NM_031215_TTCTTCAGGCCTTTATGTCT	CABLES2__NM_031215	10	42	37	11
CABP1__NM_001033677_ACAGGGGCCTCCGCGAACTG	CABP1__NM_001033677	1139	1310	1129	1177
CABP1__NM_001033677_AGGCGGACCGTTCCTCCAC	CABP1__NM_001033677	871	670	1013	705
CABP1__NM_001033677_CGGGGCCGCATTTAAGCGGC	CABP1__NM_001033677	3	0	21	49
CABP1__NM_001033677_CTGGGGTCCCCGGACGACTG	CABP1__NM_001033677	200	186	161	138
CABP1__NM_001033677_GCAGAGACAGCAGATATGAT	CABP1__NM_001033677	219	189	202	196
CABP1__NM_001033677_GCGACGGGGCCGCATTTAAG	CABP1__NM_001033677	488	427	576	723
CABP1__NM_001033677_GGATAGATCACTGCGACCAG	CABP1__NM_001033677	522	409	504	575
CABP1__NM_001033677_GGCCGCATTTAAGCGGCCGG	CABP1__NM_001033677	0	0	0	0
CABP1__NM_001033677_GGGCCGCATTTAAGCGGCCG	CABP1__NM_001033677	0	0	0	0
CABP1__NM_001033677_GTCCCCCGGCCGCTTAAATG	CABP1__NM_001033677	780	921	803	603
CABP1__NM_001033677_TACATCAACTGCCGGGATCT	CABP1__NM_001033677	832	769	818	696
CABP1__NM_001033677_TGATGACTTCGTGGAGCTAA	CABP1__NM_001033677	141	125	166	225
CABP1__NM_004276_AGAGGAAATTATCCGAGATG	CABP1__NM_004276	301	171	258	148
CABP1__NM_004276_AGCAGATATGATTGGTGTAA	CABP1__NM_004276	202	56	193	126
CABP1__NM_004276_CCTCAATGGGGATGGACGAG	CABP1__NM_004276	709	695	664	676
CABP1__NM_004276_TTCCTTGAGAGATTTCTCAG	CABP1__NM_004276	16	45	6	5
CABP1__NM_031205_CGTCTGCACCACCATGTAGC	CABP1__NM_031205	670	729	672	497
CABP1__NM_031205_GCAAGGGCTTCGCTGAGAAC	CABP1__NM_031205	160	60	199	93
CABP1__NM_031205_GGTGGTGCAGACGAGCGAGG	CABP1__NM_031205	68	49	100	70
CABP1__NM_031205_GTGGTGCAGACGAGCGAGGA	CABP1__NM_031205	23	39	45	65
CABP2__NM_016366_AGTCCACCTTTCCGCCACCT	CABP2__NM_016366	1651	1908	1947	1637
CABP2__NM_016366_CGAGACCGGGACGGCTACAT	CABP2__NM_016366	1070	934	1146	1389
CABP2__NM_016366_CGGCAGACATGATCGGTGTC	CABP2__NM_016366	622	659	673	790
CABP2__NM_016366_GACGGTCTGGTCGACTTCGA	CABP2__NM_016366	811	703	1000	750
CABP2__NM_016366_GGGGACGGCCGCATCAGCGT	CABP2__NM_016366	955	617	987	721
CABP2__NM_016366_TAGCCCTGGACGCCTGGCGC	CABP2__NM_016366	127	14	117	35
CABP2__NM_016366_TCCCCATTGGTGTCGAACTG	CABP2__NM_016366	397	209	288	312
CABP2__NM_016366_TGATCGGTGTCCGGGAGCTA	CABP2__NM_016366	60	90	108	141
CABP4__NM_145200_CCCGAAGACTCGATTGAGCA	CABP4__NM_145200	1117	1531	1384	1743
CABP4__NM_145200_CGAGGGTCTCGAAAGCGCAC	CABP4__NM_145200	640	551	840	758
CABP4__NM_145200_CTGGGCCCCGAGGAGCTAGA	CABP4__NM_145200	167	113	112	121
CABP4__NM_145200_GACTCCCAAGAGTGATGCAG	CABP4__NM_145200	384	359	382	371
CABP4__NM_145200_GAGTTTGACACTGACCGTGA	CABP4__NM_145200	824	851	715	563
CABP4__NM_145200_GCTCAATCGAGTCTTCGGGA	CABP4__NM_145200	762	446	972	597
CABP4__NM_145200_GGACAGGGATGGACGAATTA	CABP4__NM_145200	194	222	236	209
CABP4__NM_145200_GGAGTCAGGACGATGTCGGT	CABP4__NM_145200	851	588	1055	948
CABP4__NM_145200_GTCAGTGTCAAACTCCTCGA	CABP4__NM_145200	284	351	315	319
CABP4__NM_145200_TCCCGAAGACTCGATTGAGC	CABP4__NM_145200	367	288	351	335
CABP4__NM_145200_TCCCTCACCTTTCCGTCAAACTA	CABP4__NM_145200	453	715	469	782
CABP4__NM_145200_TCCTCTGAGCAGCGTCGTGC	CABP4__NM_145200	170	190	204	277
CABP5__NM_019855_AGCATTTCTTGAGTTCGATA	CABP5__NM_019855	256	169	257	342
CABP5__NM_019855_AGCTGTTTCTGCAAGCAATT	CABP5__NM_019855	165	145	189	174
CABP5__NM_019855_AGTTCGATAAGGACCGAGAT	CABP5__NM_019855	433	455	581	473
CABP5__NM_019855_AGTTTGACACGAATGGAGAT	CABP5__NM_019855	235	185	236	258
CABP5__NM_019855_CAGTTTGACACGAATGGAGA	CABP5__NM_019855	317	380	292	552
CABP5__NM_019855_CTCAATCAGTTCCATCTCCG	CABP5__NM_019855	1339	1282	1533	1760
CABP5__NM_019855_GCTGTTTCTGCAAGCAATTT	CABP5__NM_019855	309	565	326	400
CABP5__NM_019855_GTCCGGGAGGCTGATGTTAA	CABP5__NM_019855	694	609	807	797
CABP5__NM_019855_GTTCATCTCTTGTAAGGATC	CABP5__NM_019855	132	169	210	365
CABP5__NM_019855_TACAAGAGATGAACCCATCT	CABP5__NM_019855	241	136	328	275
CABP5__NM_019855_TGGCCTGCTGTAGCTCCACC	CABP5__NM_019855	37	22	49	34
CABP7__NM_182527_AAGTCCACTTGACCATCACC	CABP7__NM_182527	870	984	1108	890
CABP7__NM_182527_ACCTCCAGCTCCACCTCGTT	CABP7__NM_182527	2142	2097	2325	1732
CABP7__NM_182527_ACGGCGGCGTTGATGTACCG	CABP7__NM_182527	962	1184	823	1004
CABP7__NM_182527_AGATGAAGCCATTGCCGTCA	CABP7__NM_182527	810	705	905	606
CABP7__NM_182527_CACCGGGTGGAACGGCATCT	CABP7__NM_182527	688	576	829	463
CABP7__NM_182527_CATGCTCATTGCGGCCAACC	CABP7__NM_182527	106	180	197	128
CABP7__NM_182527_CATGGACAGGTGCTCGCAGA	CABP7__NM_182527	211	199	166	186
CABP7__NM_182527_CCAGAAGACAGTATCAAAGT	CABP7__NM_182527	1133	1371	1166	1048
CABP7__NM_182527_GAAGGCCTCTCGGATCTCTG	CABP7__NM_182527	602	677	529	439
CABP7__NM_182527_GACTCTTGCGCACGCAAGTC	CABP7__NM_182527	31	24	55	25
CABP7__NM_182527_GGCACAGCCATGCGCTCACT	CABP7__NM_182527	879	750	993	946
CABP7__NM_182527_TGACGGCGGCGTTGATGTAC	CABP7__NM_182527	2036	2134	2216	1674
CABS1__NM_033122_AGGGACAACGAAGTCATATC	CABS1__NM_033122	161	177	210	255
CABS1__NM_033122_ATCAATAGCTACAAACCTAG	CABS1__NM_033122	41	15	77	83
CABS1__NM_033122_ATCTCTCTCCATCCAAATAC	CABS1__NM_033122	199	295	176	200
CABS1__NM_033122_CAACAAACTGACAGCTAAAA	CABS1__NM_033122	67	38	89	272
CABS1__NM_033122_CTGTGTCAATGGTAGCTAAG	CABS1__NM_033122	235	151	171	265
CABS1__NM_033122_GAAACAACTATTACTTCAGA	CABS1__NM_033122	235	255	235	379
CABS1__NM_033122_GATTTCAGCCTCAGGAATAG	CABS1__NM_033122	205	248	192	410
CABS1__NM_033122_GGTAATAGAGTCTCTTGTTA	CABS1__NM_033122	376	581	421	561
CABS1__NM_033122_GGTTTGTAGCTATTGATGCA	CABS1__NM_033122	245	447	226	262
CABS1__NM_033122_TCCTGCCTTTCCACGTAAAA	CABS1__NM_033122	265	194	255	471
CABS1__NM_033122_TCTTCAAGGGCAGTTATGTC	CABS1__NM_033122	158	162	244	339
CABS1__NM_033122_TGTCATCTTCTAAAACACTT	CABS1__NM_033122	165	151	181	135
CABYR__NM_012189_AGCAGAGTCTTGAGGCCATA	CABYR__NM_012189	326	228	392	154
CABYR__NM_012189_AGGGTGGGGTAGTAGTCTTA	CABYR__NM_012189	157	111	137	107
CABYR__NM_012189_CAAGAACTTACTATGTATAG	CABYR__NM_012189	387	295	353	255
CABYR__NM_012189_CAGCTTGCTGCTCAGATGTT	CABYR__NM_012189	340	319	313	326
CABYR__NM_012189_CAGTATGCCTGTTGTTATCA	CABYR__NM_012189	585	941	767	802
CABYR__NM_012189_CCATAGGGTACGACAAGTCT	CABYR__NM_012189	674	332	628	252
CABYR__NM_012189_CCCAGACTTGTCGTACCCTA	CABYR__NM_012189	935	685	1170	987
CABYR__NM_012189_GCTGGGACGTAGGCAAACTC	CABYR__NM_012189	279	172	368	208
CABYR__NM_012189_GGCACCTCCTTGATAACAAC	CABYR__NM_012189	537	665	644	735
CABYR__NM_012189_TGGCCTCAAGACTCTGCTCG	CABYR__NM_012189	63	71	65	59
CABYR__NM_012189_TGGGGTAGTAGTCTTAGGGG	CABYR__NM_012189	38	6	37	6
CABYR__NM_012189_TTTGTCACTATATTCTGTTC	CABYR__NM_012189	197	125	228	230
CABYR__NM_138643_AACTGTGGATCCTTAGGATT	CABYR__NM_138643	317	534	516	521
CABYR__NM_138643_AGGGTCCACATGTTAGAACA	CABYR__NM_138643	670	1009	738	771
CABYR__NM_138643_CCTTAGGATTAGGTAAATAG	CABYR__NM_138643	257	180	249	193
CABYR__NM_138643_CTAACTGATAAGAATCAACA	CABYR__NM_138643	152	179	236	291
CABYR__NM_138643_GCAATGGCAACAAGTGAACG	CABYR__NM_138643	812	1021	843	763
CABYR__NM_138643_GTCCACATGTTAGAACATGG	CABYR__NM_138643	333	265	483	421
CABYR__NM_138643_GTGTCGCCCATCACATAAGT	CABYR__NM_138643	298	404	419	292
CABYR__NM_153770_TTGGAAGTTACGGTATTGCT	CABYR__NM_153770	142	127	110	99
CABYR__NM_153770_TTTGGAAGTTACGGTATTGC	CABYR__NM_153770	119	378	211	204
CACFD1__NM_001135775_AACGCCATCGCCTTTGCTAC	CACFD1__NM_001135775	1236	1844	1499	2064
CACFD1__NM_001135775_ACGCCGGCCGCAATGTTCAG	CACFD1__NM_001135775	1152	1804	1443	1925
CACFD1__NM_001135775_AGGGTCAGGCTGATGACGAT	CACFD1__NM_001135775	632	721	777	665
CACFD1__NM_001135775_CACCCTCTGAACATTGCGGC	CACFD1__NM_001135775	909	1266	1021	1375
CACFD1__NM_001135775_CATCCACCCTCTGAACATTG	CACFD1__NM_001135775	1259	1644	1433	1844
CACFD1__NM_001135775_CGCTGGCTGTGTCGCCTGTC	CACFD1__NM_001135775	1355	1337	1694	2199
CACFD1__NM_001135775_GCTGGCTGTGTCGCCTGTCT	CACFD1__NM_001135775	1455	1656	1806	1913
CACFD1__NM_001135775_GTACAGCACCCCCGTAGCAA	CACFD1__NM_001135775	416	284	295	266
CACFD1__NM_001242369_CATCGAGTTTGCAAACACAG	CACFD1__NM_001242369	183	289	314	333
CACFD1__NM_001242369_CGAGTTTGCAAACACAGTGG	CACFD1__NM_001242369	408	431	432	469
CACFD1__NM_001242369_GCTGTCTTCTACTGCGGGTG	CACFD1__NM_001242369	602	773	722	1053
CACFD1__NM_001242369_TGTTTGCAAACTCGATGAAC	CACFD1__NM_001242369	141	210	164	184
CACHD1__NM_020925_AATAGCCGGGCCTTCAATCC	CACHD1__NM_020925	539	507	608	380
CACHD1__NM_020925_ACTAATTGTTCTAGACACAT	CACHD1__NM_020925	675	568	799	716
CACHD1__NM_020925_AGAGAAGTTCAACCGTTACT	CACHD1__NM_020925	648	585	748	608
CACHD1__NM_020925_AGAGGTTAGGTGAGCCGTAT	CACHD1__NM_020925	280	254	408	326
CACHD1__NM_020925_AGCACACAAGTTCCGGTGTA	CACHD1__NM_020925	729	1054	927	1211
CACHD1__NM_020925_AGCAGTCTTGAATTGCAGTT	CACHD1__NM_020925	1197	1170	1693	1722
CACHD1__NM_020925_CAGTCTTGAATTGCAGTTAG	CACHD1__NM_020925	369	565	388	601
CACHD1__NM_020925_CCAGGACGAGACTTAAATTC	CACHD1__NM_020925	279	380	351	288
CACHD1__NM_020925_CTCGTCCTGGATTGAAGGCC	CACHD1__NM_020925	463	332	489	636
CACHD1__NM_020925_GACAACCTGAAATCCAACCC	CACHD1__NM_020925	220	261	294	284
CACHD1__NM_020925_GATTGACCACATCCAAGTAA	CACHD1__NM_020925	247	122	254	109
CACHD1__NM_020925_TTTCTACTACTGTTAATAGC	CACHD1__NM_020925	33	95	22	67
CACNA1A__NM_000068_AAAAGAAGCAAAGGAATCGG	CACNA1A__NM_000068	176	141	188	212
CACNA1A__NM_000068_ACAAGACCCCGATGTCTGAA	CACNA1A__NM_000068	788	629	971	823
CACNA1A__NM_000068_ACGAGATGCCGGCCCGCTAC	CACNA1A__NM_000068	3435	2585	4018	2901
CACNA1A__NM_000068_ACGATGATTGGTGCTAAGCC	CACNA1A__NM_000068	479	590	541	468
CACNA1A__NM_000068_ACGCCAAAAAGATCACCGAA	CACNA1A__NM_000068	810	811	962	822
CACNA1A__NM_000068_AGAACTGCCTCACGGTTAAC	CACNA1A__NM_000068	444	244	549	648
CACNA1A__NM_000068_AGAGAGACCGGTTAACCGTG	CACNA1A__NM_000068	505	520	643	490
CACNA1A__NM_000068_ATAGTAGGGTTTGGTATAAC	CACNA1A__NM_000068	243	187	242	227
CACNA1A__NM_000068_CACCAGCCGTTCAGACATCG	CACNA1A__NM_000068	1563	1432	1881	2135
CACNA1A__NM_000068_CAGTTCTGTCGGACGGGGAT	CACNA1A__NM_000068	567	963	615	701
CACNA1A__NM_000068_CCACCCGAGAGCCCCTGTCG	CACNA1A__NM_000068	2264	1621	2633	1689
CACNA1A__NM_000068_CCGCTTCGGAGACGAGATGC	CACNA1A__NM_000068	566	821	606	680
CACNA1A__NM_000068_CCTACCTTGTTTGAAAACCG	CACNA1A__NM_000068	559	529	634	663
CACNA1A__NM_000068_CCTCTTCAGGAAGACAACG	CACNA1A__NM_000068	85	119	109	83
CACNA1A__NM_000068_CGAGAGCCCCTGTCGTGGGT	CACNA1A__NM_000068	2905	3134	3320	3448
CACNA1A__NM_000068_CGATCCCCCCACCCACGACA	CACNA1A__NM_000068	1034	920	1379	905
CACNA1A__NM_000068_CGATGATTGGTGCTAAGCCC	CACNA1A__NM_000068	266	247	377	286
CACNA1A__NM_000068_CTACCTTGTTTGAAAACCGT	CACNA1A__NM_000068	575	369	522	477
CACNA1A__NM_000068_GACGAGATGCCGGCCCGCTA	CACNA1A__NM_000068	2695	1686	3214	2121
CACNA1A__NM_000068_GACGATGCAATTCGCTATGA	CACNA1A__NM_000068	854	663	1049	707
CACNA1A__NM_000068_GCAGTTCTGTCGGACGGGGA	CACNA1A__NM_000068	527	356	434	506
CACNA1A__NM_000068_TGGTGGTGGGTGTAGGGCAC	CACNA1A__NM_000068	36	57	39	90
CACNA1B__NM_000718_AACTCAGAGCAAGTGACCGA	CACNA1B__NM_000718	495	284	603	640
CACNA1B__NM_000718_AAGTTGGTGATGCCAAAGTT	CACNA1B__NM_000718	182	259	228	254
CACNA1B__NM_000718_ACACAGCAGCCTGCGGTTTA	CACNA1B__NM_000718	1209	1181	1505	1414
CACNA1B__NM_000718_ACACGGAGCCCTATTTCATC	CACNA1B__NM_000718	1553	747	1807	701
CACNA1B__NM_000718_ACTCACCAGCCGCTCGGACA	CACNA1B__NM_000718	2004	1515	2159	1580
CACNA1B__NM_000718_ATGCTGGGAGGTCCCTTGTT	CACNA1B__NM_000718	1218	926	1547	1460
CACNA1B__NM_000718_ATTGGCCTGGAGTTCTACAT	CACNA1B__NM_000718	717	487	759	827
CACNA1B__NM_000718_CAAAAGATGCCCGATGAAATA	CACNA1B__NM_000718	419	458	582	547
CACNA1B__NM_000718_CACACCAGACCCTAAACCGC	CACNA1B__NM_000718	1058	921	1199	1123
CACNA1B__NM_000718_CACTCTGTGGTATGTGAAAT	CACNA1B__NM_000718	212	145	222	190
CACNA1B__NM_000718_CAGGGGACATATATGATAGC	CACNA1B__NM_000718	309	256	256	283
CACNA1B__NM_000718_CCCAGTTGTGTTGCATGGTT	CACNA1B__NM_000718	1008	1236	1272	1725
CACNA1B__NM_000718_CCTCCTCCCACGAGTGGAAG	CACNA1B__NM_000718	360	134	431	414
CACNA1B__NM_000718_CTCACCAGCCGCTCGGACAT	CACNA1B__NM_000718	1350	1012	1454	1246
CACNA1B__NM_000718_CTCGCTCTTCGTCTTCAGCG	CACNA1B__NM_000718	287	257	370	352
CACNA1B__NM_000718_GACACGGAGCCCTATTTCAT	CACNA1B__NM_000718	1544	607	1602	597
CACNA1B__NM_000718_GAGTGAGCATTCGCTGACAC	CACNA1B__NM_000718	537	476	460	500
CACNA1B__NM_000718_GCAGCTCACTATGAGATTCA	CACNA1B__NM_000718	1043	857	1144	1042
CACNA1B__NM_000718_GCAGTTCTGCTTGACCGGGA	CACNA1B__NM_000718	364	439	373	291
CACNA1B__NM_000718_GCCAGGATCATATACTCGAA	CACNA1B__NM_000718	913	781	1015	847
CACNA1B__NM_000718_GCCCCCAGCCCATGTTATCT	CACNA1B__NM_000718	868	765	844	1031
CACNA1B__NM_000718_GCGCGATCGATTGCTTGTAG	CACNA1B__NM_000718	1734	2096	1927	2453
CACNA1B__NM_000718_GCTGTGAAAAGTACTCGCGA	CACNA1B__NM_000718	660	663	777	758
CACNA1B__NM_000718_GGCAGCTCACTATGAGATTC	CACNA1B__NM_000718	750	837	783	894
CACNA1B__NM_000718_GTGTCTTTGTGGCTTATGCG	CACNA1B__NM_000718	293	406	329	336
CACNA1B__NM_000718_TCATGCTGAACCCCGAGACC	CACNA1B__NM_000718	705	681	735	687
CACNA1B__NM_000718_TCCATTCGAGTATATGATCC	CACNA1B__NM_000718	278	128	391	296
CACNA1B__NM_000718_TGGAAACCCCTTCCACTCGT	CACNA1B__NM_000718	339	190	455	225
CACNA1B__NM_000718_TGTGGATGTCATTAACCCAT	CACNA1B__NM_000718	369	229	431	337
CACNA1B__NM_000718_TTCGACACCCAAGATAACAT	CACNA1B__NM_000718	136	87	161	99
CACNA1B__NM_000718_TTCGTCGTCTAGAGTAAAAG	CACNA1B__NM_000718	22	6	26	43
CACNA1C__NM_000719_AAAGGGAATATAGATCGCTA	CACNA1C__NM_000719	205	72	242	147
CACNA1C__NM_000719_ACTTACCAGGTTGGAATTGG	CACNA1C__NM_000719	524	850	590	698
CACNA1C__NM_000719_AGCCTGCCGGGCTGCGTCGA	CACNA1C__NM_000719	1830	2075	1925	2222
CACNA1C__NM_000719_AGCGGAAGCGGCAGCAATAT	CACNA1C__NM_000719	482	417	664	484
CACNA1C__NM_000719_AGGCGGCCATCGACGCAGCC	CACNA1C__NM_000719	75	199	100	129
CACNA1C__NM_000719_ATATGGGAAACCCAAGAAGC	CACNA1C__NM_000719	82	58	116	23
CACNA1C__NM_000719_CATGAATGCCAATGCGGCAG	CACNA1C__NM_000719	240	112	193	242
CACNA1C__NM_000719_CCATGGTCAATGAGAATACG	CACNA1C__NM_000719	781	1170	808	876
CACNA1C__NM_000719_CCTCGTATTCTCATTGACCA	CACNA1C__NM_000719	1826	1625	2030	2564
CACNA1C__NM_000719_GATGGCCGCCTGCCACGACA	CACNA1C__NM_000719	595	700	638	522
CACNA1C__NM_000719_TGAATGCCAATGCGGCAGCG	CACNA1C__NM_000719	239	185	208	298
CACNA1C__NM_000719_TGACCCTGAAGAACCCCATC	CACNA1C__NM_000719	526	634	682	684
CACNA1D__NM_000720_AAGAGCCTGCATTAGTATAG	CACNA1D__NM_000720	278	396	357	312
CACNA1D__NM_000720_AAGTGGTCGCAACACTCGAA	CACNA1D__NM_000720	302	238	377	341
CACNA1D__NM_000720_ACTCCTGACACTAGTCGAAG	CACNA1D__NM_000720	666	573	718	658
[illegible]	[illegible]	[illegible]	[illegible]	[illegible]	[illegible]

CADM3__NM_001127173_AGTCCTCGTGATCTTTCACT	CADM3__NM_001127173	1051	1102	1335	1098
CADM3__NM_001127173_CCCAGCAGCAGGCGAACAGC	CADM3__NM_001127173	96	86	138	66
CADM3__NM_001127173_CGGTGACATTCCAGGTTACC	CADM3__NM_001127173	213	246	148	197
CADM3__NM_001127173_CTGAATTCGATTATCTCGAA	CADM3__NM_001127173	275	328	323	322
CADM3__NM_001127173_GACATTCCAGGTTACCCGGG	CADM3__NM_001127173	502	485	606	569
CADM3__NM_001127173_GAGCAGGGTTAGACCATTGC	CADM3__NM_001127173	422	369	423	384
CADM3__NM_001127173_GCTGAATTCGATTATCTCGA	CADM3__NM_001127173	1003	1167	1210	1405
CADM3__NM_001127173_GTGCCAAGTGAAAGATCACG	CADM3__NM_001127173	654	415	736	691
CADM3__NM_001127173_TAGCACAGTGACGAGGGACT	CADM3__NM_001127173	592	577	516	464
CADM3__NM_001127173_TCCCAGAAGACTGACAGTTT	CADM3__NM_001127173	664	465	679	386
CADM3__NM_021189_AACTCTGGCTCCACTCGACG	CADM3__NM_021189	651	715	829	888
CADM3__NM_021189_TCCTGCTCCTGCCAGTAGCC	CADM3__NM_021189	125	300	176	212
CADM4__NM_145296_ACAGACAGAGAACGTGACAG	CADM4__NM_145296	373	311	459	381
CADM4__NM_145296_ACGGTTTCGTGTGGACCGTA	CADM4__NM_145296	404	400	475	589
CADM4__NM_145296_CAACTATGGACCCATCATAC	CADM4__NM_145296	778	755	993	970
CADM4__NM_145296_CCGGCAAAGACTCATTCCCG	CADM4__NM_145296	1710	1487	1947	1755
CADM4__NM_145296_CGGTTCCAGCGGATCTGGTT	CADM4__NM_145296	53	39	40	33
CADM4__NM_145296_CGTGTGGACCGTAAGGACGA	CADM4__NM_145296	586	617	621	562
CADM4__NM_145296_CTCTTCTTCAATGGCACCCG	CADM4__NM_145296	154	221	195	206
CADM4__NM_145296_GAGGCATGAATCCGGGCCGT	CADM4__NM_145296	1136	867	1150	1230
CADM4__NM_145296_GCCGTCTGCACCAGTATGAT	CADM4__NM_145296	1472	1687	1613	1633
CADM4__NM_145296_GCTGCCGGGTCTGGTATCCG	CADM4__NM_145296	1582	1578	1784	1843
CADM4__NM_145296_GTGGACCGTAAGGACGACGG	CADM4__NM_145296	101	74	152	123
CADM4__NM_145296_GTTACCTAGTACCGTGAGCG	CADM4__NM_145296	107	91	158	226
CADPS__NM_003716_AGCCCACCGACATGGCTCGC	CADPS__NM_003716	259	167	228	290
CADPS__NM_003716_AGCCCAGATCAGACGAGAGC	CADPS__NM_003716	231	258	296	229
CADPS__NM_003716_ATTCTTCTTCGCTGGACGAA	CADPS__NM_003716	9	7	27	51
CADPS__NM_003716_CAATGGGGAAACCCAGATCA	CADPS__NM_003716	479	279	551	439
CADPS__NM_003716_CATTGAGGAAAGCCTGAAAC	CADPS__NM_003716	426	668	588	577
CADPS__NM_003716_CGCTGTGCAGAGTTACTATG	CADPS__NM_003716	760	1125	980	1148
CADPS__NM_003716_GAGCCCTCGCTGGTACGGCT	CADPS__NM_003716	931	916	938	1047
CADPS__NM_003716_GGTGGGCTGCTTGGCATTAA	CADPS__NM_003716	169	205	218	214
CADPS__NM_003716_GTCTCCCAGCCGTACCAGCG	CADPS__NM_003716	543	432	560	667
CADPS__NM_003716_GTGGCCCGCATGGTTCAGAG	CADPS__NM_003716	111	70	125	94
CADPS__NM_003716_TCTCCCAGCCGTACCAGCGA	CADPS__NM_003716	237	294	300	184
CADPS__NM_003716_TGGGCTGCTTGGCATTAAAG	CADPS__NM_003716	166	187	207	164
CADPS2__NM_001009571_AAAACATAGAAAAACGTGTG	CADPS2__NM_001009571	128	62	126	176
CADPS2__NM_001009571_AACTGGAAGACTTTCCAAAT	CADPS2__NM_001009571	490	691	491	417
CADPS2__NM_001009571_AGCTCTACGTCTTCGTCGTG	CADPS2__NM_001009571	655	769	652	825
CADPS2__NM_001009571_AGTCTTCCAGTTTCGAAAGG	CADPS2__NM_001009571	317	229	276	201
CADPS2__NM_001009571_CGCAGTTCGGAGTTATTATG	CADPS2__NM_001009571	841	883	921	1084
CADPS2__NM_001009571_GCACTTAGGGCCATTCTATT	CADPS2__NM_001009571	910	958	1210	1339
CADPS2__NM_001009571_GCTCCTTTATAATGCATGTC	CADPS2__NM_001009571	242	320	378	423
CADPS2__NM_001009571_GCTGGAAGACGGGTCCAGCA	CADPS2__NM_001009571	221	291	295	325
CADPS2__NM_001009571_GGTACTGTCATTCACCTTAG	CADPS2__NM_001009571	423	404	451	412
CADPS2__NM_001009571_GGTGAATGACAGTACCACGT	CADPS2__NM_001009571	1069	1195	1293	994
CADPS2__NM_001009571_TCAGCTGTCAAAGTCCGACG	CADPS2__NM_001009571	495	465	670	665
CADPS2__NM_001009571_TCCGGACCACCTTTCGAAAC	CADPS2__NM_001009571	412	743	568	770
CAGE1__NM_001170692_AAACCGTTTGCAAATTTCAC	CAGE1__NM_001170692	360	473	400	480
CAGE1__NM_001170692_AACGGTTTCAAATTGTCTCA	CAGE1__NM_001170692	196	360	172	270
CAGE1__NM_001170692_ACACTTTGTGAAGATGCTTA	CAGE1__NM_001170692	810	971	927	942
CAGE1__NM_001170692_ACCTCCAAGCTTCTTATGTA	CAGE1__NM_001170692	664	586	707	705
CAGE1__NM_001170692_AGAAGCTTGGAGGTTGGCTA	CAGE1__NM_001170692	323	223	324	415
CAGE1__NM_001170692_CTTTCCTTACATAAGAAGCT	CAGE1__NM_001170692	122	279	200	188
CAGE1__NM_001170692_GATATGCTGATGGTGTCAAC	CAGE1__NM_001170692	309	368	288	342
CAGE1__NM_001170692_GCAGAAACACTAGTTTCCAT	CAGE1__NM_001170692	1083	1276	1076	1371
CAGE1__NM_001170692_GTATCCACTTCAAAATGTAC	CAGE1__NM_001170692	117	117	132	174
CAGE1__NM_001170692_TAAAATACTCGTTACCAAGT	CAGE1__NM_001170692	57	17	22	32
CAGE1__NM_001170692_TGGAGGTTGGCTAGGGTTGA	CAGE1__NM_001170692	119	165	194	269
CAGE1__NM_001170692_TTCACATTCTCCAATCTGTA	CAGE1__NM_001170692	24	29	70	63
CAGE1__NM_205864_AAAACATACTAACAGAAAGC	CAGE1__NM_205864	124	266	142	121
CAGE1__NM_205864_ACAAGAGTTCTTGTCTTTAC	CAGE1__NM_205864	504	948	563	585
CAGE1__NM_205864_ACTGAGCTGTCTCAAATTTA	CAGE1__NM_205864	428	425	568	574
CAGE1__NM_205864_CTTGTTGAAACGGGAAAAAG	CAGE1__NM_205864	69	24	87	41
CAGE1__NM_205864_GAATGCAAAACTTAAACAGC	CAGE1__NM_205864	177	419	249	269
CAGE1__NM_205864_TGATGTCATCAATAAGCTAA	CAGE1__NM_205864	92	87	158	166
CALB1__NM_004929_AAATAGGAATTGTAGAGGTA	CALB1__NM_004929	164	114	182	146
CALB1__NM_004929_AATCACCGTCAGCGTCGAAA	CALB1__NM_004929	766	728	975	1142
CALB1__NM_004929_AGCCGAGTATACAGACCTAA	CALB1__NM_004929	300	199	233	163
CALB1__NM_004929_ATAATGGCTTTGTCGGATGG	CALB1__NM_004929	477	528	467	429
CALB1__NM_004929_CAAAAGGTTACTACCAGTGC	CALB1__NM_004929	522	882	578	652
CALB1__NM_004929_CAGCCTTCTTTCGCGCCTGC	CALB1__NM_004929	185	236	210	192
CALB1__NM_004929_GCTCCAGCAGGCGCGAAAGA	CALB1__NM_004929	146	120	134	82
CALB1__NM_004929_TAATGGCTTTGTCGGATGGA	CALB1__NM_004929	537	622	694	824
CALB1__NM_004929_TACCATTAGGTCTGTATACT	CALB1__NM_004929	572	638	710	677
CALB1__NM_004929_TGGAAAAATAGGAATTGTAG	CALB1__NM_004929	1	0	14	1
CALB1__NM_004929_TGGAGGGAAGCTGTACCGAA	CALB1__NM_004929	86	143	165	153
CALB1__NM_004929_TGGCTCCATTTCGACGCTGA	CALB1__NM_004929	25	11	8	9
CALB2__NM_001740_ACGGATGTTTGACTTGAACG	CALB2__NM_001740	627	811	678	597
CALB2__NM_001740_ATGTCAAAGAGTGACAACTT	CALB2__NM_001740	404	675	347	372
CALB2__NM_001740_ATGTTTGACTTGAACGGGGA	CALB2__NM_001740	161	374	171	162
CALB2__NM_001740_CAATGTAGCCGCTTCTATCC	CALB2__NM_001740	2995	3390	3266	3760
CALB2__NM_001740_CACCTCCATAAACTCGGCGC	CALB2__NM_001740	892	965	1091	1222
CALB2__NM_001740_CTCAGATGGGAAAATCGAGA	CALB2__NM_001740	81	43	133	114
CALB2__NM_001740_CTCCAGCGCCGAGTTTATGG	CALB2__NM_001740	633	832	456	536
CALB2__NM_001740_CTTGAACGGGGATGGCAAAT	CALB2__NM_001740	783	694	683	683
CALB2__NM_001740_GCTTGGGCTCATCGTACGGC	CALB2__NM_001740	1580	2551	1733	2093
CALB2__NM_001740_GGAAGTTCTCTTCGGTTGGC	CALB2__NM_001740	240	243	303	373
CALB2__NM_001740_GGGCTCCAGCGCCGAGTTTA	CALB2__NM_001740	355	442	385	577
CALB2__NM_001740_TGGAGCTTGGGCTCATCGTA	CALB2__NM_001740	285	323	423	223
CALCA__NM_001033952_AATCTGAGTACTTGCATGCT	CALCA__NM_001033952	173	199	180	218
CALCA__NM_001033952_AGCCCCAGATCTAAGCGGTG	CALCA__NM_001033952	331	486	414	448
CALCA__NM_001033952_ATGGTCTCTCTCCAAGTCGC	CALCA__NM_001033952	103	147	86	156
CALCA__NM_001033952_ATTACCGCACCGCTTAGATC	CALCA__NM_001033952	299	392	261	191

[illegible]

CASP4__NM_033306_CCTAGAGGAAGTATTTCGGA	CASP4__NM_033306	874	1059	1284	1316
CASP4__NM_033306_TATGGTGGGCATTTGAGCTT	CASP4__NM_033306	207	203	324	271
CASP4__NM_033306_TGTCATGGACAGTCGTTCTA	CASP4__NM_033306	173	122	127	179
CASP5__NM_001136109_AGTTAAGATGTTGGAATACC	CASP5__NM_001136109	235	309	254	264
CASP5__NM_001136109_CAAAGAGTCTACCAAGATCA	CASP5__NM_001136109	474	402	530	533
CASP5__NM_001136109_CACAGCTCTTCTGCAAATCG	CASP5__NM_001136109	291	272	254	442
CASP5__NM_001136109_GATCACCAGTGTAAAACGTA	CASP5__NM_001136109	101	46	66	73
CASP5__NM_001136109_GTCCTGGAGAGACCGCACAA	CASP5__NM_001136109	104	84	119	39
CASP5__NM_001136109_TCCGAAATATTTCCATTAGG	CASP5__NM_001136109	148	82	214	168
CASP5__NM_001136109_TCCTGGAGAGACCGCACAAG	CASP5__NM_001136109	0	0	0	0
CASP5__NM_001136109_TGTCAAGGTTGCTCGTTCTA	CASP5__NM_001136109	81	199	112	96
CASP5__NM_001136112_AGTTATCCAATCCACTCTGA	CASP5__NM_001136112	230	161	192	173
CASP5__NM_001136112_ATATTTCTGATAGAAGACAG	CASP5__NM_001136112	94	33	120	138
CASP5__NM_001136112_CCACGTTGTTCTTTAGCATG	CASP5__NM_001136112	311	260	319	393
CASP5__NM_001136112_CCAGACCCTAGTACCTAATA	CASP5__NM_001136112	384	337	438	451
CASP5__NM_001136112_CCGTATTAGGTACTAGGGTC	CASP5__NM_001136112	226	204	252	381
CASP5__NM_001136112_TTCAAAGGTATCCTTCAGAG	CASP5__NM_001136112	69	42	89	90
CASP5__NM_001136112_TTGATCCGTATTAGGTACTA	CASP5__NM_001136112	235	203	285	307
CASP5__NM_001136112_TTTGATCCGTATTAGGTACT	CASP5__NM_001136112	165	363	186	138
CASP6__NM_001226_AAACAGATGCCTTCTATAAA	CASP6__NM_001226	177	267	200	162
CASP6__NM_001226_AAGATTGTCTCTATCTGCGC	CASP6__NM_001226	209	383	188	180
CASP6__NM_001226_ACTACTGCTCAAAATTCATG	CASP6__NM_001226	548	495	642	717
CASP6__NM_001226_CTTTAATCTTCAATCATGAG	CASP6__NM_001226	103	197	110	64
CASP6__NM_001226_GAATGACTGGCACATCGTGC	CASP6__NM_001226	277	312	409	356
CASP6__NM_001226_GAGGAGTTCTGTGAACTCTA	CASP6__NM_001226	80	197	180	156
CASP6__NM_001226_GCTTCTTTCCAATTGCACTT	CASP6__NM_001226	553	577	721	855
CASP6__NM_001226_GTTGGACACCAACATAACTG	CASP6__NM_001226	37	123	20	33
CASP6__NM_001226_TCCGGCAGAAAAGTACAAAA	CASP6__NM_001226	560	670	766	865
CASP6__NM_001226_TGCAAAGACCCAAGTGCAAT	CASP6__NM_001226	751	1190	643	803
CASP6__NM_001226_TGCATCCACCTCAGTTATGT	CASP6__NM_001226	1223	936	1217	1340
CASP6__NM_001226_TGTGAGATGTTGGGAAAATA	CASP6__NM_001226	111	105	132	144
CASP6__NM_032992_TGCTTCTTTCCAATTGCACT	CASP6__NM_032992	109	148	64	81
CASP7__NM_001227_AAGAACTTTGATAAAGTGAC	CASP7__NM_001227	144	232	251	232
CASP7__NM_001227_ACCTGAATGAAGAAGAGTTT	CASP7__NM_001227	728	715	1048	1087
CASP7__NM_001227_AGACAATCACGTCAAAACCC	CASP7__NM_001227	176	112	205	166
CASP7__NM_001227_ATTTGACAGCCCACTTTAGG	CASP7__NM_001227	436	654	590	428
CASP7__NM_001227_CTGGATGCCATCATCAAGCT	CASP7__NM_001227	71	100	99	89
CASP7__NM_001227_GAAGAGGGACGGTACAAACG	CASP7__NM_001227	336	613	308	443
CASP7__NM_001227_GATTTGACAGCCCACTTTAG	CASP7__NM_001227	427	448	431	578
CASP7__NM_001227_GCATCTATCCCCCCTAAAGT	CASP7__NM_001227	462	356	539	629
CASP7__NM_001227_GGGACGGTACAAACGAGGAC	CASP7__NM_001227	283	197	213	296
CASP7__NM_001227_TCCATGGCTTAAGAGGATGC	CASP7__NM_001227	248	218	222	148
CASP7__NM_001227_TGGGCTGTCAAATCCTTTAT	CASP7__NM_001227	274	364	261	414
CASP7__NM_001227_TTAGCATCTGTGTCATTGAT	CASP7__NM_001227	218	137	215	261
CASP7__NM_001267057_GAAAGCGAAACTAAAAGTCT	CASP7__NM_001267057	292	278	364	390
CASP7__NM_001267057_TAGTTTCGCTTTCGCTAAAG	CASP7__NM_001267057	118	111	109	92
CASP7__NM_001267057_TCGGGGTAGTGCGCTCCGCA	CASP7__NM_001267057	434	368	384	482
CASP7__NM_001267057_TGCGGGGACACGGGTCGCTT	CASP7__NM_001267057	1220	1274	1383	1556
CASP7__NM_001267057_TTACCGTTCCCACGGCGGTA	CASP7__NM_001267057	27	32	108	118
CASP7__NM_001267057_TTAGTTTCGCTTTCGCTAAA	CASP7__NM_001267057	145	184	156	209
CASP7__NM_001267057_TTTAGTTTCGCTTTCGCTAA	CASP7__NM_001267057	52	12	38	32
CASP7__NM_001267058_GATACCTGGAACCGTGGAAT	CASP7__NM_001267058	520	431	532	448
CASP7__NM_001267058_TCCACTGGGATCTTGTATCG	CASP7__NM_001267058	702	672	739	634
CASP7__NM_033340_GGAGGAGCACGGAAAAGACC	CASP7__NM_033340	119	135	161	147
CASP7__NM_033340_TTGAAGGCTATTACTCGTGG	CASP7__NM_033340	426	197	385	324
CASP8__NM_001080124_AGTCGTTGATTATCTTCAGC	CASP8__NM_001080124	183	322	266	290
CASP8__NM_001080124_ATGATCAGACAGTATCCCCG	CASP8__NM_001080124	803	723	664	738
CASP8__NM_001080124_CAGCAGGCTCTTGTTGATTT	CASP8__NM_001080124	220	156	262	326
CASP8__NM_001080124_CTACCTAAACACTAGAAAGG	CASP8__NM_001080124	65	33	100	73
CASP8__NM_001080124_GACTATGAAGAATTCAGCAA	CASP8__NM_001080124	319	313	340	417
CASP8__NM_001080124_GAGTCTCTGGAATAACATCA	CASP8__NM_001080124	226	215	330	399
CASP8__NM_001080124_GATGGAGAAGAGGGTCATCC	CASP8__NM_001080124	250	310	353	355
CASP8__NM_001080124_GCAAATCCAGTCTATTAATT	CASP8__NM_001080124	721	970	780	890
CASP8__NM_001080124_GCCTGGACTACATTCCGCAA	CASP8__NM_001080124	1229	1178	1485	1701
CASP8__NM_001080124_GGGCTCAAATTTCTGCCTAC	CASP8__NM_001080124	440	755	523	770
CASP8__NM_001080124_TACCTACCTAAACACTAGAA	CASP8__NM_001080124	333	355	449	504
CASP8__NM_001080124_TCTCCTCCTTTCTAGTGTTT	CASP8__NM_001080124	858	978	1091	1425
CASP8__NM_001080125_CACGTGGAGTTAGGCAGGTT	CASP8__NM_001080125	1258	1305	1387	1784
CASP8__NM_001080125_CGTGGAGTTAGGCAGGTTAG	CASP8__NM_001080125	1665	1836	2059	1979
CASP8__NM_001228_GGGTCGATCATCTATTAATA	CASP8__NM_001228	404	525	477	522
CASP8__NM_001228_TGATCGACCCTCCGCCAGAA	CASP8__NM_001228	371	577	455	447
CASP8AP2__NM_001137667_ACAATTTCTTGATGGTACAC	CASP8AP2__NM_001137667	421	447	384	184
CASP8AP2__NM_001137667_ACTGGACATATACGCTGGGT	CASP8AP2__NM_001137667	825	649	798	903
CASP8AP2__NM_001137667_AGGCAACATATAATGATGTA	CASP8AP2__NM_001137667	284	147	263	64
CASP8AP2__NM_001137667_CAGAGTGGAAATAAACCGCA	CASP8AP2__NM_001137667	104	0	50	0
CASP8AP2__NM_001137667_CAGTGTTTCTCAACAGATGT	CASP8AP2__NM_001137667	201	116	213	213
CASP8AP2__NM_001137667_CCTTCATCATTGTTCTTAAG	CASP8AP2__NM_001137667	231	232	288	208
CASP8AP2__NM_001137667_GCTCACTGGACATATACGCT	CASP8AP2__NM_001137667	624	308	555	478
CASP8AP2__NM_001137667_GGCTCACTGGACATATACGC	CASP8AP2__NM_001137667	371	140	421	283
CASP8AP2__NM_001137667_GTTGAGAAACACTGCACTAA	CASP8AP2__NM_001137667	384	149	479	315
CASP8AP2__NM_001137667_TAGCACTTCACTGCCAAATC	CASP8AP2__NM_001137667	415	254	517	396
CASP8AP2__NM_001137667_TCTTGATGGTACACAGGATT	CASP8AP2__NM_001137667	438	368	529	581
CASP8AP2__NM_001137667_TGTACCATCAAGAAATTGTT	CASP8AP2__NM_001137667	170	30	127	83
CASP9__NM_001229_ACCAGAGATTCGCAAACCAG	CASP9__NM_001229	311	146	394	269
CASP9__NM_001229_CAGGCAGCTGATCATAGATC	CASP9__NM_001229	40	27	26	20
CASP9__NM_001229_CAGGTGCTCTTGAGAGTTTG	CASP9__NM_001229	115	42	105	121
CASP9__NM_001229_CCATGGACGAAGCGGATCGG	CASP9__NM_001229	1256	1554	1505	1604
CASP9__NM_001229_CCGCCGATCCGCTTCGTCCA	CASP9__NM_001229	2260	2278	2794	2771
CASP9__NM_001229_CTCTGGATCTCGGCGGGATC	CASP9__NM_001229	135	76	153	126
CASP9__NM_001229_CTTCGTTTCTGCGAACTAAC	CASP9__NM_001229	98	148	117	148
CASP9__NM_001229_TATGGGGCCTGAACAGCTCG	CASP9__NM_001229	106	136	101	159
CASP9__NM_001229_TCGCCATGGACGAAGCGGAT	CASP9__NM_001229	561	281	623	446
CASP9__NM_001229_TGCTGAGCCGCGAGCTGTTC	CASP9__NM_001229	446	505	502	622
CASP9__NM_001229_TGGATGTCCTCGATCATATG	CASP9__NM_001229	125	46	77	71

[illegible]

CCDC176__NM_025057_CTTTCCCTTCGACGGCATCT	CCDC176__NM_025057	470	528	509	651
CCDC176__NM_025057_GAGTTCTGTGACTTCCAACC	CCDC176__NM_025057	216	309	184	221
CCDC176__NM_025057_GGATCAGGAGAAAGATAATA	CCDC176__NM_025057	216	274	259	255
CCDC176__NM_025057_TACAGGAACAAAAGTATACC	CCDC176__NM_025057	70	98	125	152
CCDC176__NM_025057_TCGTGATACTTCACGGATAC	CCDC176__NM_025057	280	466	332	370
CCDC176__NM_025057_TTAAGTATCGTGATACTTCA	CCDC176__NM_025057	58	90	78	101
CCDC176__NM_025057_TTTCAGCCTCTTGTTCTAGT	CCDC176__NM_025057	305	419	425	541
CCDC177__NM_001271507_AGAACCCGCTGGCGGAGGTT	CCDC177__NM_001271507	445	684	518	402
CCDC177__NM_001271507_AGGCAGGAAGAGCCATTCGC	CCDC177__NM_001271507	516	549	581	481
CCDC177__NM_001271507_CATCGTCGTCATCCTACAGT	CCDC177__NM_001271507	1415	1501	1742	1704
CCDC177__NM_001271507_CCACCGGCTTGACCGCACAG	CCDC177__NM_001271507	586	743	671	656
CCDC177__NM_001271507_CCGCTGGCGGAGGTTGGGTC	CCDC177__NM_001271507	514	403	512	724
CCDC177__NM_001271507_CGCCCGCTGTGCGGTCAAGC	CCDC177__NM_001271507	60	42	42	45
CCDC177__NM_001271507_GCATCGTCGTCATCCTACAG	CCDC177__NM_001271507	1417	2104	1564	2181
CCDC177__NM_001271507_GCGCAGGCCTCTAGCGAGCG	CCDC177__NM_001271507	390	568	499	561
CCDC177__NM_001271507_GCGGCCGGTGATCGGAACCA	CCDC177__NM_001271507	1136	1193	1184	1254
CCDC177__NM_001271507_GGCGCAGGCCTCTAGCGAGC	CCDC177__NM_001271507	508	730	570	737
CCDC177__NM_001271507_GTCCTCCACCACCAACGCTC	CCDC177__NM_001271507	110	87	92	85
CCDC177__NM_001271507_TGACGACGATGCGCCCGACT	CCDC177__NM_001271507	2775	3915	3294	4363
CCDC178__NM_001105528_AAAGTGTCCAGAGTTAAAGC	CCDC178__NM_001105528	291	344	316	370
CCDC178__NM_001105528_AAGTCATGAAAATCAGTATC	CCDC178__NM_001105528	269	260	241	244
CCDC178__NM_001105528_AATGGAAACATTGCTCTCAG	CCDC178__NM_001105528	123	124	136	108
CCDC178__NM_001105528_ACCTTCACTGTTCTCCTTAG	CCDC178__NM_001105528	1237	1190	1295	1169
CCDC178__NM_001105528_AGAGATGATCAAACCAATAT	CCDC178__NM_001105528	153	205	206	251
CCDC178__NM_001105528_AGATGTGGAGTCCAAAATAC	CCDC178__NM_001105528	240	223	192	227
CCDC178__NM_001105528_CACAGCTGTGACGTCGACAT	CCDC178__NM_001105528	1973	1694	2128	2026
CCDC178__NM_001105528_GAGTTTATGACCAACTAACC	CCDC178__NM_001105528	140	153	167	178
CCDC178__NM_001105528_GCACAGCTGTGACGTCGACA	CCDC178__NM_001105528	802	759	894	1293
CCDC178__NM_001105528_TCAGAGAGAAGGCATGGTCA	CCDC178__NM_001105528	261	162	262	201
CCDC178__NM_001105528_TTTAACATGTCAGGAAGTAA	CCDC178__NM_001105528	0	0	0	0
CCDC178__NM_001105528_TTTGGCATGTACAAAACTGA	CCDC178__NM_001105528	233	365	376	451
CCDC179__NM_001195637_ACGGCATGTGCCTGTATTGC	CCDC179__NM_001195637	885	1298	1038	1367
CCDC18__NM_206886_AACAAGGAAGACCGTTGCAT	CCDC18__NM_206886	361	154	357	358
CCDC18__NM_206886_AATTGGTGATTTCGGAATTG	CCDC18__NM_206886	598	579	461	622
CCDC18__NM_206886_ATGGAGTTTGCAGAGTTTAG	CCDC18__NM_206886	139	116	128	92
CCDC18__NM_206886_ATTATGCCCCTAATCCTTCA	CCDC18__NM_206886	231	183	303	308
CCDC18__NM_206886_CTGCTGTGAGGCAAATAAAT	CCDC18__NM_206886	113	130	151	224
CCDC18__NM_206886_GAAACAGAACCTGTAAAGCT	CCDC18__NM_206886	260	242	328	206
CCDC18__NM_206886_GAATGAAAACCGAGAATTAA	CCDC18__NM_206886	148	116	236	198
CCDC18__NM_206886_GGTGAATAATTCAAAAGTCC	CCDC18__NM_206886	252	269	285	346
CCDC18__NM_206886_GTGAATAATTCAAAAGTCCA	CCDC18__NM_206886	161	103	232	257
CCDC18__NM_206886_TATCTTCAGTTCATGTCTTA	CCDC18__NM_206886	257	164	211	157
CCDC18__NM_206886_TCAAAAAAAGCCAAGAGATA	CCDC18__NM_206886	41	158	85	32
CCDC18__NM_206886_TGTTTGAGAAGCTACTCATC	CCDC18__NM_206886	68	10	69	12
CCDC180__NM_020893_AAGGTGACCCAGGTTCCGAA	CCDC180__NM_020893	185	179	166	141
CCDC180__NM_020893_ACTGACGACATCTTGGCCGC	CCDC180__NM_020893	1982	2043	2294	2430
CCDC180__NM_020893_AGGTGACCCAGGTTCCGAAT	CCDC180__NM_020893	256	143	313	163
CCDC180__NM_020893_CAGGAAACGCGTCTAGGCTT	CCDC180__NM_020893	660	467	815	670
CCDC180__NM_020893_CGGCCAAGATGTCGTCAGTG	CCDC180__NM_020893	1207	1141	1263	1332
CCDC180__NM_020893_CGGGGTCTCATGGATACTAT	CCDC180__NM_020893	590	584	598	649
CCDC180__NM_020893_CTGCCGGGAAGCCGTCGTCC	CCDC180__NM_020893	1136	1092	1176	1258
CCDC180__NM_020893_CTTCCCCACTGACGACATCT	CCDC180__NM_020893	1010	568	1094	809
CCDC180__NM_020893_GATGAGCTCTGAACTCGAGC	CCDC180__NM_020893	89	32	108	89
CCDC180__NM_020893_GGCTTTCCCATTCGGAACCT	CCDC180__NM_020893	484	373	447	506
CCDC180__NM_020893_GTCGTCCAGGAAACGCGTCT	CCDC180__NM_020893	866	888	917	988
CCDC180__NM_020893_TAAGCACCAGCACCTTTCAA	CCDC180__NM_020893	560	708	693	610
CCDC181__NM_021179_AAACAAGGATAGGACAATAC	CCDC181__NM_021179	25	11	61	22
CCDC181__NM_021179_AAGAACCGGTGAATGATAAA	CCDC181__NM_021179	74	170	77	33
CCDC181__NM_021179_AAGTGATGCCAGCATAATAG	CCDC181__NM_021179	67	18	20	1
CCDC181__NM_021179_ACAATACTGGTAGAGAGAGA	CCDC181__NM_021179	159	186	183	196
CCDC181__NM_021179_AGAACCGGTGAATGATAAAA	CCDC181__NM_021179	67	33	73	87
CCDC181__NM_021179_GAAAACGAAAGGAATATGTT	CCDC181__NM_021179	490	625	408	498
CCDC181__NM_021179_GGAAGTAAGGAGATATATTA	CCDC181__NM_021179	118	54	127	126
CCDC181__NM_021179_GTAGTAGTGTCTTCTAGTGG	CCDC181__NM_021179	627	362	580	388
CCDC181__NM_021179_GTCAGGATCAGAATGCCGTT	CCDC181__NM_021179	942	964	1134	1090
CCDC181__NM_021179_GTCTTTCTGGCTTTCTAGTT	CCDC181__NM_021179	385	461	289	342
CCDC181__NM_021179_TCACTATCTGAATCTGATAT	CCDC181__NM_021179	207	120	361	384
CCDC181__NM_021179_TTTACAAGACATTGCCAGTC	CCDC181__NM_021179	164	227	216	344
CCDC182__NM_001282544_AGGAGCAGGCGGCTCGCGTG	CCDC182__NM_001282544	978	828	1072	826
CCDC182__NM_001282544_AGGATCTCCAAGTCAGCGGG	CCDC182__NM_001282544	44	25	10	4
CCDC182__NM_001282544_CATGACGGTGAATACCCTAC	CCDC182__NM_001282544	823	804	770	813
CCDC182__NM_001282544_CCGTGGCATCGTGCGCAACA	CCDC182__NM_001282544	1358	1556	1588	1878
CCDC182__NM_001282544_GACGCCTTCTGCGAGATGAA	CCDC182__NM_001282544	361	615	423	743
CCDC182__NM_001282544_GGCGTCTTCAAGGTAATGAA	CCDC182__NM_001282544	195	412	289	439
CCDC182__NM_001282544_GGGAAAAAAATGATAGAGAG	CCDC182__NM_001282544	81	54	143	227
CCDC182__NM_001282544_TCCCGGAGCTGTTTCTCGCG	CCDC182__NM_001282544	1351	1296	1475	1567
CCDC182__NM_001282544_TCGCGTGAGGCAGCGGCTAA	CCDC182__NM_001282544	391	337	436	447
CCDC182__NM_001282544_TCTCGCAGAAGGCGTCTTCA	CCDC182__NM_001282544	734	936	886	768
CCDC182__NM_001282544_TGACGGTGAATACCCTACAG	CCDC182__NM_001282544	307	289	301	144
CCDC183__NM_001039374_AACCTCTATGTCCGGCTGAT	CCDC183__NM_001039374	1294	1729	1348	1550
CCDC183__NM_001039374_CCAGCAGGTCCAAATACAGC	CCDC183__NM_001039374	720	570	697	732
CCDC183__NM_001039374_CCGTGCTCATCAGGTTCGAG	CCDC183__NM_001039374	991	939	1071	1276
CCDC183__NM_001039374_CTCCGTGCTCATCAGGTTCG	CCDC183__NM_001039374	852	849	1055	1210
CCDC183__NM_001039374_CTGAGCAGTAGTTGACCACG	CCDC183__NM_001039374	987	1115	965	1035
CCDC183__NM_001039374_CTGCGCAGCAACATCCGCCG	CCDC183__NM_001039374	373	282	451	387
CCDC183__NM_001039374_CTGTCTTCAGATAATCCAGC	CCDC183__NM_001039374	134	73	194	122
CCDC183__NM_001039374_GCTGTTCAAATCGAGGGTGT	CCDC183__NM_001039374	850	745	1067	826
CCDC183__NM_001039374_GTAGGTTGTGCATGTTCACG	CCDC183__NM_001039374	868	951	972	1015
CCDC183__NM_001039374_TCCAGCTGGCGGATGATCTG	CCDC183__NM_001039374	259	307	263	387
CCDC183__NM_001039374_TCCGTGCTCATCAGGTTCGA	CCDC183__NM_001039374	630	590	857	861
CCDC183__NM_001039374_TGCCCATCAGCCGGACATAG	CCDC183__NM_001039374	178	297	255	262
CCDC184__NM_001013635_AAGCCCTGTTTGAGGACGTG	CCDC184__NM_001013635	397	423	371	430
CCDC184__NM_001013635_AGTTGCAGCAGGGTAATGTC	CCDC184__NM_001013635	556	755	715	604
CCDC184__NM_001013635_CAAGTGGCCCGTCCCCCCCA	CCDC184__NM_001013635	1892	1797	2077	1421
CCDC184__NM_001013635_CAGGGCGGCTTGGGCGTGGT	CCDC184__NM_001013635	2634	2733	2784	2053
CCDC184__NM_001013635_CAGTTGCAGCAGGGTAATGT	CCDC184__NM_001013635	622	416	654	441
CCDC184__NM_001013635_GAGCCGGAGACTCCTTCGCC	CCDC184__NM_001013635	836	962	890	936
CCDC184__NM_001013635_GGGGCGCAGTGGTCATAATC	CCDC184__NM_001013635	417	335	465	346
CCDC184__NM_001013635_GGGTAATGTCGGGCATGTCG	CCDC184__NM_001013635	1128	1408	1546	1630
CCDC184__NM_001013635_GTGTCCACCGTGCCGGCAGT	CCDC184__NM_001013635	535	258	596	362
CCDC184__NM_001013635_TGGGCGTGGTCGGCGGCAAG	CCDC184__NM_001013635	207	295	241	196
CCDC184__NM_001013635_TGGGGGGGACGGGCCACTTG	CCDC184__NM_001013635	508	332	448	394
CCDC184__NM_001013635_TTGGGCGTGGTCGGCGGCAA	CCDC184__NM_001013635	468	609	630	832
CCDC185__NM_152610_CCTGGGCCAGAGGGTAATGC	CCDC185__NM_152610	265	327	361	453
CCDC185__NM_152610_CGCGGGGTGAACGAACAGTG	CCDC185__NM_152610	620	800	665	945
CCDC185__NM_152610_CGGGTGCGGAGAGCGAAGCT	CCDC185__NM_152610	73	68	130	150
CCDC185__NM_152610_CTCCCCTGAGTGGCACATTC	CCDC185__NM_152610	500	532	781	1009
CCDC185__NM_152610_GCCAGAGGGTAATGCGGGCA	CCDC185__NM_152610	817	787	905	1197
CCDC185__NM_152610_GCGGGGTGAACGAACAGTGC	CCDC185__NM_152610	673	725	678	708
CCDC185__NM_152610_GCGGGTGCTCAGATTCACTG	CCDC185__NM_152610	1525	1806	1766	2129
CCDC185__NM_152610_GGCCAGAGGGTAATGCGGGC	CCDC185__NM_152610	414	306	406	511
CCDC185__NM_152610_GGTTCCCAGAGATCCCGGTA	CCDC185__NM_152610	240	290	277	428
CCDC185__NM_152610_GTGCTTCCTGGAACGTTCCA	CCDC185__NM_152610	51	46	35	46
CCDC185__NM_152610_TACCCTGAATGTGCCACTCA	CCDC185__NM_152610	983	1005	1040	1381
CCDC185__NM_152610_TCGGCAGACATCTAGGTCGC	CCDC185__NM_152610	803	586	835	955
CCDC186__NM_018017_AAATACCTGTTCCATCCGTT	CCDC186__NM_018017	820	835	755	864
CCDC186__NM_018017_CACAAATATTGGTGGAATTA	CCDC186__NM_018017	166	245	236	219
CCDC186__NM_018017_CAGAGGAAGTAGAGGCTATG	CCDC186__NM_018017	448	622	566	674
CCDC186__NM_018017_CTTGGTGCAGTCTGTATCAT	CCDC186__NM_018017	986	906	1255	1542
CCDC186__NM_018017_GATGGCCCAACGGATGGAAC	CCDC186__NM_018017	173	51	164	185
CCDC186__NM_018017_GCAAGATAAATATTTGCAGC	CCDC186__NM_018017	285	335	349	371
CCDC186__NM_018017_GCAGAACATCGAGTACCAAA	CCDC186__NM_018017	814	919	949	954
CCDC186__NM_018017_GCATATGAACACAATTAAAC	CCDC186__NM_018017	368	505	477	488
CCDC186__NM_018017_TGCCTCTTCACATTTCTTGT	CCDC186__NM_018017	120	116	211	185
CCDC186__NM_018017_TGGGAAAACACCTGAATTAA	CCDC186__NM_018017	15	42	51	37
CCDC186__NM_018017_TGGTGCAGTCTGTATCATAG	CCDC186__NM_018017	94	35	93	93
CCDC186__NM_018017_TGTTTAGCAAAATTTCCACT	CCDC186__NM_018017	415	550	381	524
CCDC22__NM_014008_AGGTGCCTCAGCCTGTTGGA	CCDC22__NM_014008	889	650	993	982
CCDC22__NM_014008_CAGGTCCCGGGCTTGTATGG	CCDC22__NM_014008	2158	1347	2182	1435
CCDC22__NM_014008_CCACCTCTGGAACTCAATTC	CCDC22__NM_014008	855	687	942	813
CCDC22__NM_014008_CCAGGTCCCGGGCTTGTATG	CCDC22__NM_014008	1441	1140	1486	1370
CCDC22__NM_014008_CCCAGGTCCCGGGCTTGTAT	CCDC22__NM_014008	795	845	975	936
CCDC22__NM_014008_CTGAGCATCTGCGCCAAAGC	CCDC22__NM_014008	219	219	268	341
CCDC22__NM_014008_GCAGCGAATGGATGAGGATT	CCDC22__NM_014008	1113	988	1301	1086
CCDC22__NM_014008_GCATCCTCAGAGGCATCGGT	CCDC22__NM_014008	1345	965	1606	1151
CCDC22__NM_014008_GCGTGTGATCAACCCTGCGG	CCDC22__NM_014008	299	252	342	284
CCDC22__NM_014008_GTGCCAGCTGGTCCCGAATT	CCDC22__NM_014008	876	779	959	762
CCDC22__NM_014008_TCTGCCCACCGATGCCTCTG	CCDC22__NM_014008	1429	1640	1355	1339
CCDC22__NM_014008_TGCATCCTCAGAGGCATCGG	CCDC22__NM_014008	321	374	485	347
CCDC23__NM_199342_CAACAGAGTCATGACAGAAC	CCDC23__NM_199342	421	312	388	370
CCDC23__NM_199342_CTGCTGGGCTGATTTCTGTT	CCDC23__NM_199342	2224	2796	2632	3473
CCDC23__NM_199342_TTCTGTCATGACTCTGTTGA	CCDC23__NM_199342	0	0	0	0
CCDC24__NM_152499_AAGAGGTCTTTAGGAGAGG	CCDC24__NM_152499	165	71	165	157
CCDC24__NM_152499_ATCTGGAATATCTCCTGTTC	CCDC24__NM_152499	341	322	489	396
CCDC24__NM_152499_CAATGTTGGACACGTTCAGT	CCDC24__NM_152499	528	615	622	616
CCDC24__NM_152499_CCGACTGGCCTCGAGGTCGA	CCDC24__NM_152499	381	223	398	727
CCDC24__NM_152499_GAGCCCTACCTTCGACCTCG	CCDC24__NM_152499	1246	1366	1323	1328
CCDC24__NM_152499_GCAGGACCCTGGGGCTATAC	CCDC24__NM_152499	456	182	495	449
CCDC24__NM_152499_GTGGCCAGACACCTGAGGTG	CCDC24__NM_152499	32	11	8	21
CCDC24__NM_152499_TCTGGCCACCTGGTCAATGT	CCDC24__NM_152499	1392	1375	1605	1134
CCDC24__NM_152499_TGGAGGAGCACGTTCCGCTC	CCDC24__NM_152499	69	57	147	147
CCDC24__NM_152499_TGGGCCCACCACCTCTCATC	CCDC24__NM_152499	223	116	266	196
CCDC24__NM_152499_TGGTTGGGAGAGACACAAGA	CCDC24__NM_152499	0	0	0	0
CCDC24__NM_152499_TTGGGTCCAGTATAGCCCCA	CCDC24__NM_152499	61	39	28	10
CCDC25__NM_018246_CAAGATGAACAACGTTAATG	CCDC25__NM_018246	515	484	569	577
CCDC25__NM_018246_GAATATAGAAGACATCCCAA	CCDC25__NM_018246	145	237	149	147
CCDC25__NM_018246_GATGAAGATCTGATCAAGCA	CCDC25__NM_018246	290	144	283	128
CCDC25__NM_018246_GGACTGTGCCCACCTTGTGA	CCDC25__NM_018246	1167	731	1182	870
CCDC25__NM_018246_GTCTGGGAACCGCTCGACTT	CCDC25__NM_018246	613	481	634	552
CCDC25__NM_018246_GTGAAGGCCAATAGCATTCA	CCDC25__NM_018246	271	224	346	431
CCDC25__NM_018246_TAGAAAAGACCAAAGTCGAG	CCDC25__NM_018246	248	204	402	387
CCDC25__NM_018246_TCTGCCTACACTATTTACAT	CCDC25__NM_018246	567	393	741	617
CCDC25__NM_018246_TGTATACCTTCGATTACATA	CCDC25__NM_018246	103	187	120	152
CCDC25__NM_018246_TTAATGTGGTATATACGCCG	CCDC25__NM_018246	107	28	117	81
CCDC27__NM_152492_ACGCCTCATGTTACCTAAGG	CCDC27__NM_152492	511	474	603	553
CCDC27__NM_152492_ATGTGGCCCTGGTGCTGAAC	CCDC27__NM_152492	548	850	679	613
CCDC27__NM_152492_CCCACGCCTCATGTTACCTA	CCDC27__NM_152492	1363	969	1676	1511
CCDC27__NM_152492_CCCACTGAGGCCGATTTGTC	CCDC27__NM_152492	612	456	857	835
CCDC27__NM_152492_GAGAGATCCACGGGAAAAGC	CCDC27__NM_152492	47	17	29	19
CCDC27__NM_152492_GCCACATCCATGTCCCACTG	CCDC27__NM_152492	715	707	713	808
CCDC27__NM_152492_GCGGGGCTCAGACACGAACG	CCDC27__NM_152492	511	618	514	479
CCDC27__NM_152492_GCTACTACAGGAAGACGGTA	CCDC27__NM_152492	182	231	279	229
CCDC27__NM_152492_GTCAATCTCTCCGGACAAAT	CCDC27__NM_152492	502	553	440	557
CCDC27__NM_152492_GTCCAAAATGGAACTTCGAA	CCDC27__NM_152492	309	538	379	500
CCDC27__NM_152492_TCCTTAGGTAACATGAGGCG	CCDC27__NM_152492	272	359	278	354
CCDC27__NM_152492_TGGCCTCCTTAGGTAACATG	CCDC27__NM_152492	8	37	5	12
CCDC28A__NM_015439_AAGCTGGCCGTGGTGCAATA	CCDC28A__NM_015439	392	336	377	355
CCDC28A__NM_015439_ATTGACAACCTGAGTACAGT	CCDC28A__NM_015439	585	520	493	350
CCDC28A__NM_015439_CGGGGCTTCCGCTTGGGGCA	CCDC28A__NM_015439	530	631	634	613
CCDC28A__NM_015439_GAAATTAGCTCGCTTGAATT	CCDC28A__NM_015439	568	625	727	887
CCDC28A__NM_015439_GAACAGATGGAACATGTTCG	CCDC28A__NM_015439	1537	1932	1939	2106
CCDC28A__NM_015439_GGAACATGTTCGGGGAATGC	CCDC28A__NM_015439	329	316	338	399
CCDC28A__NM_015439_GTTTACGGAAGTGACGTAAG	CCDC28A__NM_015439	135	148	116	125
CCDC28A__NM_015439_TACAGCCTCCATGCCCCAAG	CCDC28A__NM_015439	526	456	673	740
CCDC28A__NM_015439_TCCGCAGCTCCGTTTGTTTA	CCDC28A__NM_015439	1812	2490	2143	2534
CCDC28A__NM_015439_TGTCGGGTCTTTGCGGGTTG	CCDC28A__NM_015439	530	719	650	600
CCDC28A__NM_015439_TTAAGTTCCTTATTGCACCA	CCDC28A__NM_015439	103	140	176	198
CCDC28A__NM_015439_TTGAACAGATGGAACATGTT	CCDC28A__NM_015439	359	389	366	440
CCDC28B__NM_024296_AGACATCAGTCACCTCGGTC	CCDC28B__NM_024296	1118	629	1184	706
CCDC28B__NM_024296_CGCTGTGGCTGGTAGGCACA	CCDC28B__NM_024296	91	67	92	46
CCDC28B__NM_024296_GACCCTCCGTAGTGTGCCTG	CCDC28B__NM_024296	1200	1090	1314	1137
CCDC28B__NM_024296_GCACTTCTCTTTGCCTACTC	CCDC28B__NM_024296	990	942	1069	1170
CCDC28B__NM_024296_GGACCAGCTGCTTAGCAATG	CCDC28B__NM_024296	322	194	327	221
CCDC28B__NM_024296_GGACCCTCCGTAGTGTGCCT	CCDC28B__NM_024296	1292	1243	1485	1370
CCDC28B__NM_024296_TGAGCAGCTGGAGCACGTTC	CCDC28B__NM_024296	464	567	541	387
CCDC28B__NM_024296_TGAGGAGCAGTCCGCTGCGT	CCDC28B__NM_024296	2452	1930	2712	2048
CCDC28B__NM_024296_TGGCAGATGAGGAAGTCCTA	CCDC28B__NM_024296	28	10	44	36
CCDC3__NM_001282658_CCACCCTGGAGAAGCGCAAC	CCDC3__NM_001282658	492	441	541	588
CCDC3__NM_001282658_CTGCCGCACATCAATGCCCG	CCDC3__NM_001282658	523	771	574	888
CCDC3__NM_001282658_CTTGCGAACAGTTTGAAAAC	CCDC3__NM_001282658	208	251	190	230
CCDC3__NM_001282658_CTTGCGGCAGGCGCGTAAGA	CCDC3__NM_001282658	196	197	289	377
CCDC3__NM_001282658_TCTTGGAAATTGACTCCGTG	CCDC3__NM_001282658	481	871	440	564
CCDC3__NM_001282658_TTGCGGCAGGCGCGTAAGAA	CCDC3__NM_001282658	133	101	216	228
CCDC3__NM_031455_CCGAGACCATCGTGTACGCC	CCDC3__NM_031455	1860	2398	1975	2596
CCDC3__NM_031455_CGACCTCGGCCGAGTAGAAG	CCDC3__NM_031455	802	864	885	1058
CCDC30__NM_001080850_AACAGAAATTCAAAGCCGAA	CCDC30__NM_001080850	194	219	227	287
CCDC30__NM_001080850_AACTAAAATACAACCAGCAA	CCDC30__NM_001080850	140	114	132	106
CCDC30__NM_001080850_AACTTCACCAGAATTTGCAC	CCDC30__NM_001080850	530	415	441	446
CCDC30__NM_001080850_AGAGGCCCTACTTAAACAAC	CCDC30__NM_001080850	67	101	47	58
CCDC30__NM_001080850_ATGAGCTTCGATATGAACGA	CCDC30__NM_001080850	153	249	228	206
CCDC30__NM_001080850_CAGCTGAGTTGCATAGAATC	CCDC30__NM_001080850	113	136	164	184
CCDC30__NM_001080850_CCTACAGGAAAAGCTGGTTC	CCDC30__NM_001080850	595	504	623	509
CCDC30__NM_001080850_CTGCTTTCTTTCTCCTAATT	CCDC30__NM_001080850	603	731	748	951
CCDC30__NM_001080850_CTTTCTCCTAATTTGGGACT	CCDC30__NM_001080850	241	459	322	349
CCDC30__NM_001080850_TCAGCAGAAAATCAAGGAAC	CCDC30__NM_001080850	313	314	358	472
CCDC30__NM_001080850_TCCTGTATTGCCTTCTCCTA	CCDC30__NM_001080850	714	686	737	849
CCDC30__NM_001080850_TCTGGAGCCGGTGCAAATTC	CCDC30__NM_001080850	113	80	77	80
CCDC33__NM_001287181_ACAGAAGAATGATCGAGAGA	CCDC33__NM_001287181	212	246	200	133
CCDC33__NM_001287181_ACTTGTCTGAAGTACCAGAG	CCDC33__NM_001287181	253	244	203	314
CCDC33__NM_001287181_AGCACCGAGTAAAGTTCAAC	CCDC33__NM_001287181	674	941	709	478
CCDC33__NM_001287181_CCCTGCTGCCTGTTCAGAGG	CCDC33__NM_001287181	794	1021	819	1068
CCDC33__NM_001287181_CTCCCATAGAACCCAAGGGA	CCDC33__NM_001287181	127	117	182	177
CCDC33__NM_001287181_GCTCCATCTTCTCGATCACC	CCDC33__NM_001287181	754	1022	876	859
CCDC33__NM_001287181_GCTGCTGGGCCTGATACAGA	CCDC33__NM_001287181	708	466	650	536
CCDC33__NM_001287181_GGAGCTGCTCCTTCTGTATC	CCDC33__NM_001287181	2197	2306	2531	2761
CCDC33__NM_001287181_GGCCTTCCCTTGGGTTCTAT	CCDC33__NM_001287181	1388	1968	1609	2207
CCDC33__NM_001287181_TGGCAGAAAACGCGAAGCTG	CCDC33__NM_001287181	62	9	98	73
CCDC33__NM_001287181_TGGCCTTCCCTTGGGTTCTA	CCDC33__NM_001287181	41	30	55	42
CCDC33__NM_001287181_TTCTCTCCCATAGAACCCAA	CCDC33__NM_001287181	0	0	0	0
CCDC33__NM_025055_AACGACTGTTGCGTACAACT	CCDC33__NM_025055	183	228	306	393
CCDC33__NM_025055_AGACACGCAGGTACTTGATG	CCDC33__NM_025055	718	672	793	779
CCDC33__NM_025055_AGCCCACCAGAGCCCCTATC	CCDC33__NM_025055	1207	1124	1154	1130
CCDC33__NM_025055_CAGGCAGGTTGGTAGCCCCA	CCDC33__NM_025055	729	295	784	299
CCDC33__NM_025055_CATGATGGTCTCCTTCTTAG	CCDC33__NM_025055	1206	848	1202	1233
CCDC33__NM_025055_CCCACCAGAGCCCCTATCTG	CCDC33__NM_025055	1253	1153	1367	1296
CCDC33__NM_025055_CTCCGAGCCGTGGCCCTATG	CCDC33__NM_025055	514	876	499	745
CCDC33__NM_025055_TACACCACCACATAGGGCCA	CCDC33__NM_025055	303	262	332	369
CCDC33__NM_025055_TCACCGTGTCCCCCCAGATA	CCDC33__NM_025055	661	474	679	510
CCDC33__NM_025055_TCACCTGTCTCCCTCTAAGA	CCDC33__NM_025055	595	525	844	668
CCDC33__NM_025055_TCTGGGTCTTCAGTGTTCTG	CCDC33__NM_025055	334	194	430	236
CCDC33__NM_025055_TTCACCAGCTCAAAGTGGTA	CCDC33__NM_025055	95	172	69	173
CCDC34__NM_030771_AAGAAAAAGAAGAACGTGAC	CCDC34__NM_030771	39	47	109	127
CCDC34__NM_030771_AGAAAGCCGCCTGACACCAT	CCDC34__NM_030771	1130	1123	1107	1176
CCDC34__NM_030771_AGTTGTTGATTTAATTCCTG	CCDC34__NM_030771	312	471	385	471
CCDC34__NM_030771_CACCGTCGTCCTCGTCAAAC	CCDC34__NM_030771	1152	1157	1087	1113
CCDC34__NM_030771_CCATGGGAGGTGTGGTTTAT	CCDC34__NM_030771	982	890	901	1055
CCDC34__NM_030771_CCTGAGAGGAATGGAGTTAC	CCDC34__NM_030771	819	1021	1002	1137
CCDC34__NM_030771_CTCCTGCTCAGTCCCTATGA	CCDC34__NM_030771	707	555	700	604
CCDC34__NM_030771_GCCCACGTGCGCCCGTCATA	CCDC34__NM_030771	476	325	559	362
CCDC34__NM_030771_TCCTCTCAGGCTCGCCACTT	CCDC34__NM_030771	601	562	712	673
CCDC34__NM_030771_TCCTGCTCAGTCCCTATGAC	CCDC34__NM_030771	610	660	774	859
CCDC34__NM_030771_TGAAGATGCCCATGATTCAG	CCDC34__NM_030771	530	583	476	516
CCDC34__NM_030771_TGCCCACGTGCGCCCGTCAT	CCDC34__NM_030771	1879	2028	2140	2137
CCDC36__NM_001135197_AGCCTGGAGAATTTGATGTC	CCDC36__NM_001135197	847	912	1056	942
CCDC36__NM_001135197_ATTGGCTTCTTGAACTGAGA	CCDC36__NM_001135197	94	98	115	75
CCDC36__NM_001135197_CAGGATTCCATGCAGCAGGG	CCDC36__NM_001135197	123	121	155	217
CCDC36__NM_001135197_GCCTGGAGAATTTGATGTCT	CCDC36__NM_001135197	1203	1187	1489	1807
CCDC36__NM_001135197_GCCTGGTGAGATTGAGGCTC	CCDC36__NM_001135197	173	201	188	220
CCDC36__NM_001135197_GCTGCATGGAATCCTGGTAT	CCDC36__NM_001135197	1898	1931	1952	1749
CCDC36__NM_001135197_GGACACTGGGCACATTCAGC	CCDC36__NM_001135197	472	554	626	731
CCDC36__NM_001135197_GGGCAAAAGACAAATGTGAC	CCDC36__NM_001135197	412	485	589	732
CCDC36__NM_001135197_GTCACAACAGAACTATCTGG	CCDC36__NM_001135197	524	122	581	170
CCDC36__NM_001135197_TGAAAGAAATAGGCATGTAA	CCDC36__NM_001135197	74	73	140	111
CCDC36__NM_001135197_TGCTGCATGGAATCCTGGTA	CCDC36__NM_001135197	335	348	391	520
CCDC36__NM_001135197_TGTTAGAGAAAGCATTCTCA	CCDC36__NM_001135197	51	73	56	44
CCDC37__NM_182628_CATGTTCTTGGAGACTATAG	CCDC37__NM_182628	278	321	218	182
CCDC37__NM_182628_CCAGCGGTGGCGACTCCAGA	CCDC37__NM_182628	642	483	627	716
CCDC37__NM_182628_CCCAGATTGTTAATATCAAA	CCDC37__NM_182628	317	411	340	466
CCDC37__NM_182628_CCTTGGGCGACAGCTTGTAT	CCDC37__NM_182628	894	774	1081	792
CCDC37__NM_182628_CCTTGTTCGACGAGTTCGTC	CCDC37__NM_182628	115	112	116	122
CCDC37__NM_182628_GACCTACTCCTCGAAAGTGT	CCDC37__NM_182628	575	660	631	510
CCDC37__NM_182628_GCGAACCCTTTCCACTTATC	CCDC37__NM_182628	884	1035	992	1422
CCDC37__NM_182628_GCTTCCAGGTCGTGTAGTCG	CCDC37__NM_182628	814	874	778	1069
CCDC37__NM_182628_GGGGGCTGCTCAGGTAAGAC	CCDC37__NM_182628	395	609	458	668
CCDC37__NM_182628_TCTTGCTCAGAGATCAGGAG	CCDC37__NM_182628	179	191	224	423
CCDC37__NM_182628_TTCTTGGAGACTATAGTGGA	CCDC37__NM_182628	4	0	7	15
CCDC37__NM_182628_TTTGATATTAACAATCTGGG	CCDC37__NM_182628	21	1	7	3
CCDC38__NM_182496_AAAAACAGAATTCCTCCTCA	CCDC38__NM_182496	1023	1012	1262	1345
CCDC38__NM_182496_AACTAGCTTTCTACCCTAAA	CCDC38__NM_182496	371	164	322	302
CCDC38__NM_182496_CCGATTCCTAGATTAATAGA	CCDC38__NM_182496	262	350	352	342
CCDC38__NM_182496_GTATGAATGACTCTTCATTC	CCDC38__NM_182496	184	259	214	216
CCDC38__NM_182496_TCACAGGTTCCGACACAAAA	CCDC38__NM_182496	155	193	187	240
CCDC38__NM_182496_TCATTTGAAAAGTTTGGGCC	CCDC38__NM_182496	231	260	312	280
CCDC38__NM_182496_TCTGGTAGACTTTCATGTTA	CCDC38__NM_182496	478	429	509	370
CCDC38__NM_182496_TGAAAAAGACATAGCAATGA	CCDC38__NM_182496	119	75	91	60
CCDC38__NM_182496_TTACCTCCAGAATTCAGTGT	CCDC38__NM_182496	612	396	683	641
CCDC38__NM_182496_TTATTGCCAACACTGAATTC	CCDC38__NM_182496	29	20	59	69
CCDC38__NM_182496_TTGGTTGACAAAGCATACTA	CCDC38__NM_182496	46	20	64	38
CCDC38__NM_182496_TTTGGTTGACAAAGCATACT	CCDC38__NM_182496	33	48	20	36
CCDC39__NM_181426_AAAATGTAGAACGGCATATC	CCDC39__NM_181426	121	116	199	189
CCDC39__NM_181426_AAATGAGATGGCTTCAATAC	CCDC39__NM_181426	237	319	298	206
CCDC39__NM_181426_ACTGGAAGTTCTATTCACAG	CCDC39__NM_181426	587	941	698	642
CCDC39__NM_181426_AGACATAGATAACTGTGCTT	CCDC39__NM_181426	379	450	538	524
CCDC39__NM_181426_CCAGTGATTTAGAAGCTCTG	CCDC39__NM_181426	45	95	59	96
CCDC39__NM_181426_CCTCAGAGCTTCTAAATCAC	CCDC39__NM_181426	341	332	484	400
CCDC39__NM_181426_GAAAGATGAAATTCAACGGC	CCDC39__NM_181426	159	213	229	170
CCDC39__NM_181426_GACAAGAACTCATTAAACAA	CCDC39__NM_181426	140	205	134	156
CCDC39__NM_181426_GCAAGAGCTCTCAATTACAC	CCDC39__NM_181426	463	512	472	535
CCDC39__NM_181426_GCAATTAGAAAGACTAACTT	CCDC39__NM_181426	275	308	366	416
CCDC39__NM_181426_TGTTCTCCTCGTTCGCCACC	CCDC39__NM_181426	76	55	59	77
CCDC39__NM_181426_TTGTTCTCCTCGTTCGCCAC	CCDC39__NM_181426	1	0	0	0
CCDC40__NM_001243342_ACAGAAACTTCATCCCCGGA	CCDC40__NM_001243342	1074	960	1175	1146
CCDC40__NM_001243342_ACCTGGCCCATTTGCTCCGA	CCDC40__NM_001243342	1807	2199	2134	2682
CCDC40__NM_001243342_AGGCATACGATAGTGTTAGC	CCDC40__NM_001243342	94	53	76	78
CCDC40__NM_001243342_CCACGGAGTCTTAGGCCCGT	CCDC40__NM_001243342	610	412	488	302
CCDC40__NM_001243342_CCGACGGGCCTAAGACTCCG	CCDC40__NM_001243342	322	372	341	256
CCDC40__NM_001243342_CGACGGGCCTAAGACTCCGT	CCDC40__NM_001243342	1784	1155	1791	1330
CCDC40__NM_001243342_CTTAGGCCCGTCGGAGCAAA	CCDC40__NM_001243342	290	442	364	429
CCDC40__NM_001243342_GAAGATGGATCGGCTTCTGA	CCDC40__NM_001243342	375	392	397	285
CCDC40__NM_001243342_GAGGCATACGATAGTGTTAG	CCDC40__NM_001243342	254	190	205	181
CCDC40__NM_001243342_GGCATACGATAGTGTTAGCG	CCDC40__NM_001243342	301	345	366	331
CCDC40__NM_001243342_TGGGCCAGCAGTGGGCAGAT	CCDC40__NM_001243342	211	137	207	163
CCDC40__NM_001243342_TTAGGCCCGTCGGAGCAAAT	CCDC40__NM_001243342	307	465	362	479
CCDC42__NM_001158261_ACCGGTCTTGGATAAATTGC	CCDC42__NM_001158261	486	629	702	888
CCDC42__NM_001158261_AGAAATCATGCATCAAACTA	CCDC42__NM_001158261	49	67	103	74
CCDC42__NM_001158261_CACCATCTCCTGCTTGCGCT	CCDC42__NM_001158261	1003	1432	1059	1399
CCDC42__NM_001158261_CAGGAAACTCCCCAATGTTG	CCDC42__NM_001158261	878	645	880	726
CCDC42__NM_001158261_CCCAGGAGAACGACCAGAAA	CCDC42__NM_001158261	387	431	393	472
CCDC42__NM_001158261_CCTGCTTGGCACCATTAAGA	CCDC42__NM_001158261	682	775	704	649
CCDC42__NM_001158261_CTGGGAGGAACTGGGCGTTA	CCDC42__NM_001158261	418	501	308	410
CCDC42__NM_001158261_CTTCAACAAGTACCTAGAGA	CCDC42__NM_001158261	209	380	263	334
CCDC42__NM_001158261_GCAATTTATCCAAGACCGGT	CCDC42__NM_001158261	687	514	783	769
CCDC42__NM_001158261_GTTCAGCGTGGCCATCTTAA	CCDC42__NM_001158261	130	180	209	212
CCDC42__NM_001158261_TCCGACGCCCCCTCAACATT	CCDC42__NM_001158261	4259	3706	4840	3797
CCDC42__NM_001158261_TTCCTCCCAGCGCAGGTTCA	CCDC42__NM_001158261	20	28	17	17
CCDC42__NM_144681_TCATGGATCTCCTCGAACTG	CCDC42__NM_144681	622	635	680	557
CCDC42__NM_144681_TGTAGCGTGCAATCACCTCA	CCDC42__NM_144681	363	365	428	417
CCDC42B__NM_001144872_AGGGCTCCAGGCGCTTAAGC	CCDC42B__NM_001144872	31	52	48	54
CCDC42B__NM_001144872_AGTCGGAAATATTCCTCCCA	CCDC42B__NM_001144872	185	291	205	134
CCDC42B__NM_001144872_CAAAAGGAGCGGGAGCTAAA	CCDC42B__NM_001144872	48	83	93	132
CCDC42B__NM_001144872_CCCAGCTCCAGGAGCTACGG	CCDC42B__NM_001144872	412	449	407	396
CCDC42B__NM_001144872_CCGGACGAGGTGCTCGCACA	CCDC42B__NM_001144872	554	393	719	682
CCDC42B__NM_001144872_CTGGGAGGAATATTTCCGAC	CCDC42B__NM_001144872	370	384	406	301
CCDC42B__NM_001144872_CTGTTCCCAACGCTGTTTCA	CCDC42B__NM_001144872	409	587	517	553
CCDC42B__NM_001144872_GCTGGCACACTAGCTGGAAC	CCDC42B__NM_001144872	633	488	598	687
CCDC42B__NM_001144872_GTTTCTCTTGCAAAGCCAGT	CCDC42B__NM_001144872	74	90	38	68
CCDC42B__NM_001144872_TCCGCAGCGCGCGATTGCGC	CCDC42B__NM_001144872	5	0	9	26
CCDC42B__NM_001144872_TTCTCCAGGAGACGCAGTAC	CCDC42B__NM_001144872	14	0	12	4
CCDC43__NM_001099225_ATCTGTCACATCAGCATACT	CCDC43__NM_001099225	58	18	85	66
CCDC43__NM_001099225_CAATGGCCTGTACTTCATCT	CCDC43__NM_001099225	1451	1672	1493	1899
CCDC43__NM_001099225_CCTCCGCCATCGCCTTCGCC	CCDC43__NM_001099225	1208	1591	1495	1692
CCDC43__NM_001099225_CGTTTATGGAGCCTACATCT	CCDC43__NM_001099225	848	762	955	665
CCDC43__NM_001099225_CTGGCTGGACGGACGGTTGG	CCDC43__NM_001099225	1280	1277	1293	1027
CCDC43__NM_001099225_GCAAGGAGATTGTGGAACGA	CCDC43__NM_001099225	468	397	365	305
CCDC43__NM_001099225_GCCTTCGCCAGGGGCTATCG	CCDC43__NM_001099225	295	263	409	293
CCDC43__NM_001099225_GGAGTGGACCGAGCCGTTTA	CCDC43__NM_001099225	535	561	570	427
CCDC43__NM_001099225_TGCCTGCTTCTCAATTAGGG	CCDC43__NM_001099225	354	175	285	211
CCDC43__NM_001099225_TGTAGGCTCCATAAACGGCT	CCDC43__NM_001099225	130	132	119	91
CCDC43__NM_001099225_TTCCCTCCTTAATATCTGCA	CCDC43__NM_001099225	67	149	77	67
CCDC43__NM_001099225_TTTGTGCCTGCTTCTCAATT	CCDC43__NM_001099225	937	1193	1122	957
CCDC47__NM_020198_ACAGTCGCCTTGCACAGGCC	CCDC47__NM_020198	671	484	675	584
CCDC47__NM_020198_AGGCCTGGTTTAACACTCAT	CCDC47__NM_020198	499	321	554	570
CCDC47__NM_020198_ATCCTCAAAATCATCAAACT	CCDC47__NM_020198	590	672	668	620
CCDC47__NM_020198_ATGAGCACATCTATAACCTG	CCDC47__NM_020198	622	248	553	282
CCDC47__NM_020198_CATACCACTTGATCACTCAC	CCDC47__NM_020198	1153	604	1207	871
CCDC47__NM_020198_CTACATCAACAATCGTTATT	CCDC47__NM_020198	298	294	380	318
CCDC47__NM_020198_CTGCCCTTCCAACTCCACAG	CCDC47__NM_020198	542	212	507	388
CCDC47__NM_020198_GGCCTGGTTTAACACTCATA	CCDC47__NM_020198	458	120	506	275
CCDC47__NM_020198_GTTAGTTCCATCATCCCCTA	CCDC47__NM_020198	293	267	231	204
CCDC47__NM_020198_TAATGACTTCGCTGAATTTG	CCDC47__NM_020198	199	291	280	312
CCDC47__NM_020198_TCAGTGATTATGACCCGTTG	CCDC47__NM_020198	341	276	301	269
CCDC47__NM_020198_TCTGGTCGAGTGTGCTGTGA	CCDC47__NM_020198	535	265	501	522
CCDC50__NM_174908_AGAGGAAGATCTGAAAGCGC	CCDC50__NM_174908	263	374	285	218
CCDC50__NM_174908_ATTTGGCATCGAACGTTCAG	CCDC50__NM_174908	266	386	318	494
CCDC50__NM_174908_CCATCTGTCATGATAGGTGG	CCDC50__NM_174908	527	699	660	794
CCDC50__NM_174908_CTGGCCTTTCATTCTTAGAA	CCDC50__NM_174908	645	777	811	1020
CCDC50__NM_174908_CTTTCATTCTTAGAATGGTG	CCDC50__NM_174908	255	369	256	347
CCDC50__NM_174908_GATGCCAAATGATGCTCAAC	CCDC50__NM_174908	374	598	527	639
CCDC50__NM_174908_GATGCTGACTTCAGCCATAC	CCDC50__NM_174908	466	792	436	433
CCDC50__NM_174908_GGCAGAGAGACGACGCATTC	CCDC50__NM_174908	877	1319	897	1154
CCDC50__NM_174908_TCACCATCTGTCATGATAGG	CCDC50__NM_174908	546	314	599	575
CCDC50__NM_174908_TCCCTGTGGGCCATTCGTGA	CCDC50__NM_174908	512	621	635	618
CCDC50__NM_174908_TTCATCTTGAGCTACTTGAG	CCDC50__NM_174908	22	13	26	19
CCDC50__NM_178335_AAACACTTCACCACTGATCG	CCDC50__NM_178335	549	777	671	960
CCDC50__NM_178335_AACACTTCACCACTGATCGT	CCDC50__NM_178335	757	803	752	689
CCDC50__NM_178335_ATCCCTGTCATCCTCTAGCT	CCDC50__NM_178335	674	550	727	902
CCDC50__NM_178335_ATGCTGCTCATTGTTAATAT	CCDC50__NM_178335	551	875	703	856
CCDC50__NM_178335_TGAAAGGAAACGGTCCACTC	CCDC50__NM_178335	236	177	283	281
CCDC50__NM_178335_TGCCCGAGCTAGAGGATGAC	CCDC50__NM_178335	453	424	537	613
CCDC50__NM_178335_TGCTGCTCATTGTTAATATG	CCDC50__NM_178335	337	351	396	409
CCDC51__NM_001256964_AACTGGCTACTCTCGAGCAC	CCDC51__NM_001256964	399	283	387	257
CCDC51__NM_001256964_CCCCAGGACTGAGCCAATGA	CCDC51__NM_001256964	376	339	460	396
CCDC51__NM_001256964_CCGAGAGGCTCGGGAGGACT	CCDC51__NM_001256964	750	638	992	781
CCDC51__NM_001256964_CGTGGGTGTGCCCCACGTAC	CCDC51__NM_001256964	101	92	161	198
CCDC51__NM_001256964_CTCCACAGCCAAGACTTGGT	CCDC51__NM_001256964	227	143	217	93
CCDC51__NM_001256964_GCCCCACGTACTGGTTCGGA	CCDC51__NM_001256964	1406	861	1569	1083
CCDC51__NM_001256964_GCCCTCCGAACCAGTACGT	CCDC51__NM_001256964	1385	1165	1322	974
CCDC51__NM_001256964_GGACAGTCAGTACTTGGAAC	CCDC51__NM_001256964	253	245	325	317
CCDC51__NM_001256964_GGCCCCTCCGAACCAGTACG	CCDC51__NM_001256964	173	228	193	241
CCDC51__NM_001256964_GTGTGCCCCACGTACTGGTT	CCDC51__NM_001256964	907	1075	948	1020
CCDC51__NM_001256964_TGCCCCACGTACTGGTTCGG	CCDC51__NM_001256964	550	287	571	430
CCDC51__NM_001256964_TGTGCGGGAAAGTCATGAGA	CCDC51__NM_001256964	170	148	188	70
CCDC51__NM_001256965_AGAAAAGACTTGTAGCCAAA	CCDC51__NM_001256965	188	190	166	117
CCDC51__NM_001256965_GAGGGACCTCCACAATCTCA	CCDC51__NM_001256965	357	253	396	253
CCDC51__NM_001256965_TAGTCGCACACGGTTCACAT	CCDC51__NM_001256965	926	650	786	567
CCDC51__NM_001256965_TCAGCTCCTGTAGTCGCACA	CCDC51__NM_001256965	384	231	304	205
CCDC51__NM_001256965_TCGCACACGGTTCACATAGG	CCDC51__NM_001256965	411	368	394	173
CCDC51__NM_001256965_TGTGAACCGTGTGCGACTAC	CCDC51__NM_001256965	526	286	472	258
CCDC51__NM_001256965_TTACTCCTGGAGGCGCAGAA	CCDC51__NM_001256965	26	27	49	19
CCDC51__NM_024661_CATGTACAACCTCAATCTGA	CCDC51__NM_024661	837	813	724	519
CCDC51__NM_024661_GAGGTTGTACATGCCCCCAA	CCDC51__NM_024661	199	256	228	138
CCDC53__NM_016053_AGGACGGGCTTCCTCTCATG	CCDC53__NM_016053	1062	970	1026	863
CCDC53__NM_016053_ATATGCAGATATCTCAAAA	CCDC53__NM_016053	101	71	148	117
CCDC53__NM_016053_GTACAGTGTGCACCACAAAT	CCDC53__NM_016053	217	144	214	161
CCDC53__NM_016053_TACACAAGACTCTGGACTAC	CCDC53__NM_016053	603	501	537	594
CCDC54__NM_032600_AATCTCTAGACAATGTATCG	CCDC54__NM_032600	355	328	279	265
CCDC54__NM_032600_CAAGATGATGATAGTTATGA	CCDC54__NM_032600	255	448	362	496
CCDC54__NM_032600_GTACACACTTCACACCAAAA	CCDC54__NM_032600	317	211	339	333
CCDC54__NM_032600_TAGACAATGTATCGTGGAGC	CCDC54__NM_032600	169	139	157	181
CCDC57__NM_198082_ACAGCTGTTGCTTGTACCTA	CCDC57__NM_198082	1119	737	1168	1049
CCDC57__NM_198082_AGATAGAGGCAGCTAAGCTG	CCDC57__NM_198082	46	113	75	50
CCDC57__NM_198082_AGGAAGAGGAAACATTTAAG	CCDC57__NM_198082	143	87	170	142
CCDC57__NM_198082_CCATGCTCAGACCCTGAATC	CCDC57__NM_198082	2375	2311	2676	2783
CCDC57__NM_198082_CCCTTTCAATGTCATCACAG	CCDC57__NM_198082	322	230	435	350
CCDC57__NM_198082_GATCCCAGCAGGACATTGAA	CCDC57__NM_198082	443	171	377	363
CCDC57__NM_198082_GCATGAGGAGCTCGACCGTC	CCDC57__NM_198082	709	849	828	671
CCDC57__NM_198082_GGACCAGATCCAAAAGTCAG	CCDC57__NM_198082	189	147	244	272
CCDC57__NM_198082_GGCATTCCAGGAGCATCGCC	CCDC57__NM_198082	486	397	645	669
CCDC57__NM_198082_TCAAATTGCTCAACTATCTA	CCDC57__NM_198082	147	171	129	185
CCDC57__NM_198082_TGAGCTGCTGCTTCGCAAGG	CCDC57__NM_198082	193	104	221	113
CCDC57__NM_198082_TTGAATCGAAAATGCGGAAG	CCDC57__NM_198082	75	96	78	65
CCDC58__NM_001017928_AGACTCATAAAGTTGTTTAC	CCDC58__NM_001017928	394	315	304	229
CCDC58__NM_001017928_CATAAAGTTGTTTACAGGTT	CCDC58__NM_001017928	659	780	740	785
CCDC58__NM_001017928_TCACAGTTCACACCGCCACT	CCDC58__NM_001017928	1443	1195	1603	852
CCDC59__NM_014167_ACCACCCGCAAGCCTTCGTG	CCDC59__NM_014167	1314	393	1529	481
CCDC59__NM_014167_ACGAAGGCTTGCGGGTGGTT	CCDC59__NM_014167	1330	789	1478	855
CCDC59__NM_014167_ACTGTTCTTCAAGCAACGGC	CCDC59__NM_014167	176	243	137	209
CCDC59__NM_014167_CCACGCGCCTCAATACCACC	CCDC59__NM_014167	945	804	1175	786
CCDC59__NM_014167_GTGGCGGCCTGGTGGTATTG	CCDC59__NM_014167	572	529	673	415
CCDC59__NM_014167_TCCATTTGTACATTCAAGTT	CCDC59__NM_014167	341	224	490	257
CCDC59__NM_014167_TGGTGAACTTGTTCTGACAA	CCDC59__NM_014167	0	0	13	52
CCDC59__NM_014167_TTACAAGAAATTGCTACGGA	CCDC59__NM_014167	133	184	178	176
CCDC59__NM_014167_TTCGTGGGGAGCGTTCGCGA	CCDC59__NM_014167	13	9	17	15
CCDC6__NM_005436_ATCGCCTAGAGATATCTCCA	CCDC6__NM_005436	705	571	682	602
CCDC6__NM_005436_ATCGGGCTGGACACAGTGCG	CCDC6__NM_005436	675	882	876	1123
CCDC6__NM_005436_CATCTCGCCGTTCCGCCTGG	CCDC6__NM_005436	2012	1153	2018	1542
CCDC6__NM_005436_CCAACTTCACTGACTAGAGC	CCDC6__NM_005436	316	406	362	421
CCDC6__NM_005436_CTTTCTCATAATTTACAGCA	CCDC6__NM_005436	216	371	302	468
CCDC6__NM_005436_GAGTGAGTCCAGCTTAGAAA	CCDC6__NM_005436	264	165	352	384
CCDC6__NM_005436_GCCAGCGAGAGCGACACGGA	CCDC6__NM_005436	341	360	393	304
CCDC6__NM_005436_TCAAGCAGGCCTATATCACC	CCDC6__NM_005436	671	838	761	639
CCDC6__NM_005436_TGTCATCTCGCCGTTCCGCC	CCDC6__NM_005436	240	49	211	261
CCDC6__NM_005436_TTCAAGATGCTGTTCTAGTT	CCDC6__NM_005436	129	126	120	126
CCDC6__NM_005436_TTCTGGAGAATCAATCTCCA	CCDC6__NM_005436	0	0	0	0
CCDC6__NM_005436_TTCTGGAGAGCTCATTAGTG	CCDC6__NM_005436	0	0	3	0
CCDC60__NM_178499_AAAAGGACCTTATACGAAGC	CCDC60__NM_178499	79	171	69	87
CCDC60__NM_178499_AATGAAGAGCATCAAGTATA	CCDC60__NM_178499	225	165	230	196
CCDC60__NM_178499_ACGTGAGCCTCGGTTAGCGA	CCDC60__NM_178499	384	393	332	286
CCDC60__NM_178499_ACTATGGGGACACCTTATTG	CCDC60__NM_178499	985	1015	1004	831
CCDC60__NM_178499_CACGTGAGCCTCGGTTAGCG	CCDC60__NM_178499	982	1167	1136	1265
CCDC60__NM_178499_CATTTCTCCCTCGCTAACCG	CCDC60__NM_178499	340	509	445	383
CCDC60__NM_178499_CGGTTGGTGACTCGCATTGT	CCDC60__NM_178499	708	565	743	477
CCDC60__NM_178499_GATCTCAGAAATCCACTATG	CCDC60__NM_178499	969	802	1035	968
CCDC60__NM_178499_GCATTTCATCACAGCGCCAA	CCDC60__NM_178499	462	444	606	751
CCDC60__NM_178499_GGTGGGTGTGGTCAATAGTC	CCDC60__NM_178499	132	165	116	220
CCDC60__NM_178499_TGCCTCGGAGAACCTAAGGC	CCDC60__NM_178499	247	187	277	234
CCDC60__NM_178499_TTTATGCCTCGGAGAACCTA	CCDC60__NM_178499	72	57	110	82
CCDC61__NM_001267723_AGGAACTGGGCCGCCTGCAA	CCDC61__NM_001267723	597	536	611	524
CCDC61__NM_001267723_CAAACTCCACGGAGTAGATG	CCDC61__NM_001267723	516	542	448	661
CCDC61__NM_001267723_CAACATCTTCTGTCATATGC	CCDC61__NM_001267723	670	794	685	921
CCDC61__NM_001267723_CAGGTGGACTACGTCTTCCG	CCDC61__NM_001267723	427	389	458	380
CCDC61__NM_001267723_CCAGCGCCTCCTCGCGCGAT	CCDC61__NM_001267723	1193	1420	1100	1401
CCDC61__NM_001267723_CCGCTGCCCCTCCCGTACCA	CCDC61__NM_001267723	3449	3433	4049	2630
CCDC61__NM_001267723_CTCCGTGGAGTTTGACAGGT	CCDC61__NM_001267723	1137	904	1202	1187
CCDC61__NM_001267723_CTGCGGAACCGCAAGATGGG	CCDC61__NM_001267723	327	173	324	358
CCDC61__NM_001267723_CTGGGCGGCCCCCCATCTTG	CCDC61__NM_001267723	1727	1867	1801	1490
CCDC61__NM_001267723_GGGCATCATCCGGTCACTGA	CCDC61__NM_001267723	943	1356	1058	1046
CCDC61__NM_001267723_GGGGTGTGGAGCATGCCGTG	CCDC61__NM_001267723	555	513	557	570
CCDC61__NM_001267723_TGGCTTGCCCTGGTACGGGA	CCDC61__NM_001267723	71	18	15	30
CCDC62__NM_201435_AACTCTCAGCAACACGTTAG	CCDC62__NM_201435	221	174	196	202
CCDC62__NM_201435_AATCAGGTCACTCACGAAGA	CCDC62__NM_201435	288	279	302	245
CCDC62__NM_201435_ACCCAAAAGTCGATATTAAG	CCDC62__NM_201435	567	696	677	904
CCDC62__NM_201435_CACTATCGAGAAACAACGGA	CCDC62__NM_201435	105	199	146	230
CCDC62__NM_201435_CCCAAAAGTCGATATTAAGA	CCDC62__NM_201435	479	433	427	287
CCDC62__NM_201435_CCGCCATCTTGGCATCACGT	CCDC62__NM_201435	2616	2549	2825	2972
CCDC62__NM_201435_CTGCTTCTCATCAGATAAAG	CCDC62__NM_201435	74	55	82	77
CCDC62__NM_201435_GCAGTTTCTTAATTTCAATG	CCDC62__NM_201435	396	577	376	450
CCDC62__NM_201435_GCATGATGTCAGTGTTTACC	CCDC62__NM_201435	1145	1176	1154	1272
CCDC62__NM_201435_TAAAAAGCAATTCATCGTGC	CCDC62__NM_201435	451	312	534	493
CCDC62__NM_201435_TAATCACATTGCAGATTGTT	CCDC62__NM_201435	798	597	997	961
CCDC62__NM_201435_TTTCCACTATCGAGAAACAA	CCDC62__NM_201435	33	25	30	68
CCDC63__NM_001286243_AAGCAGCCTTCTCAAATCGT	CCDC63__NM_001286243	714	502	723	449
CCDC63__NM_001286243_ACTCCTGCTCCAAACTAAGG	CCDC63__NM_001286243	195	98	178	152
CCDC63__NM_001286243_AGTCCTCCTTAGTTTGGAGC	CCDC63__NM_001286243	373	396	544	421
CCDC63__NM_001286243_ATTTCTGCTGGGATCGCAAG	CCDC63__NM_001286243	245	135	211	224
CCDC63__NM_001286243_CCAGAAAATCTTCAATGAGC	CCDC63__NM_001286243	438	775	485	655
CCDC63__NM_001286243_GAATGATCGCAATGAATTCG	CCDC63__NM_001286243	730	729	810	952
CCDC63__NM_001286243_GACAGAGTGGTTGTCATCGT	CCDC63__NM_001286243	1531	1063	1548	1155
CCDC63__NM_001286243_GGACACCTCTCAGTACAACC	CCDC63__NM_001286243	535	392	624	344
CCDC63__NM_001286243_TCGCAATGAATTCGAGGAGC	CCDC63__NM_001286243	325	454	317	459
CCDC63__NM_001286243_TGAAGACCTACGATTTGAGA	CCDC63__NM_001286243	214	169	178	112
CCDC63__NM_001286243_TGAGTCTCATGAAATCCTCG	CCDC63__NM_001286243	209	174	245	169
CCDC63__NM_001286243_TGCTCGGTTCACGTATGTCA	CCDC63__NM_001286243	1401	1572	1559	1544
CCDC63__NM_001286244_TGGTCTTCCTCAGTTTATCC	CCDC63__NM_001286244	36	32	36	18
CCDC63__NM_152591_AGGCAGAGCTCCGGAAGCTA	CCDC63__NM_152591	15	4	29	11
CCDC64__NM_207311_AGATGCTGTCAGATCGGAAA	CCDC64__NM_207311	392	309	298	421
CCDC64__NM_207311_CAACCAGCACATTATCCGGC	CCDC64__NM_207311	2659	1979	2868	2244
CCDC64__NM_207311_CGAGCAGCGCCTTACCCAGC	CCDC64__NM_207311	973	869	976	699
CCDC64__NM_207311_CGCTTCAGCACCCGCCGAGC	CCDC64__NM_207311	424	505	494	440
CCDC64__NM_207311_GCCCAGGCAGAAAGCGGACA	CCDC64__NM_207311	532	308	623	435
CCDC64__NM_207311_GCCGCCCTCATCTTCCCCGG	CCDC64__NM_207311	4921	3675	4947	3457
CCDC64__NM_207311_GCTCTGGGAAGCCTACTGCC	CCDC64__NM_207311	573	944	557	537
CCDC64__NM_207311_GGAACAGAACCAAAGGCTAT	CCDC64__NM_207311	465	353	554	656
CCDC64__NM_207311_GGAGCTAGAACTGGCGTTAG	CCDC64__NM_207311	308	195	241	201
CCDC64__NM_207311_GTTGGCGGCCCGGCTGGGTA	CCDC64__NM_207311	879	896	1003	1020
CCDC64__NM_207311_TCGTCTCAGCGCTACTTTAG	CCDC64__NM_207311	556	603	605	559
CCDC64__NM_207311_TGCTCTGCTCGATCTCTGAC	CCDC64__NM_207311	395	426	499	523
CCDC64B__NM_001103175_AAGAAGCCCTCGTCGCCGCT	CCDC64B__NM_001103175	1334	1127	1709	1506
CCDC64B__NM_001103175_CCTCACGCAGGCCTCGGATC	CCDC64B__NM_001103175	269	237	245	171
CCDC64B__NM_001103175_CCTCAGTCCAGCGGAGATAC	CCDC64B__NM_001103175	311	332	315	272
CCDC64B__NM_001103175_CCTCCAGGACGCCGACGTGT	CCDC64B__NM_001103175	1309	945	1471	982
CCDC64B__NM_001103175_CGGGGTGTCTCCGGGTGCGT	CCDC64B__NM_001103175	1954	1394	2101	1134
CCDC64B__NM_001103175_GCAGGACAGTGGGCGAGAAC	CCDC64B__NM_001103175	227	163	272	202
CCDC64B__NM_001103175_GCTGGAACGCGCACGGTCAG	CCDC64B__NM_001103175	409	413	619	675
CCDC64B__NM_001103175_GGCCAGTTCTGACTGCAACG	CCDC64B__NM_001103175	1038	900	888	795
CCDC64B__NM_001103175_GGGAGGCGCTGAGTGCGCTG	CCDC64B__NM_001103175	91	67	72	94
CCDC64B__NM_001103175_GGTCGCCGTCGTCGAGGCTG	CCDC64B__NM_001103175	799	1355	930	1096
CCDC64B__NM_001103175_TGAGGGATGTTCGCTTCTTG	CCDC64B__NM_001103175	948	315	948	638
CCDC64B__NM_001103175_TGCCTCGTTGCAGTCAGAAC	CCDC64B__NM_001103175	317	466	292	409
CCDC65__NM_001286957_CCCAGCTGCTAGATATCAAT	CCDC65__NM_001286957	561	400	635	394
CCDC65__NM_001286957_CCCATTGATATCTAGCAGCT	CCDC65__NM_001286957	636	660	651	595
CCDC65__NM_001286957_CCGGTATATCCGTAATGACA	CCDC65__NM_001286957	431	365	369	161
CCDC65__NM_001286957_CCTTGTCATTACGGATATAC	CCDC65__NM_001286957	616	910	719	570
CCDC65__NM_001286957_GATAGTTGACTATAAAGAGT	CCDC65__NM_001286957	301	223	296	194
CCDC65__NM_001286957_GCCCAGCTGCTAGATATCAA	CCDC65__NM_001286957	925	694	1007	769
CCDC65__NM_001286957_GGCAGCCTTTCGATCCTCTG	CCDC65__NM_001286957	1271	1698	1432	1514
CCDC65__NM_001286957_GTGGAATGATCTCAAAAACA	CCDC65__NM_001286957	22	5	40	9
CCDC65__NM_001286957_TAATCTAGAAGAGCTTACTG	CCDC65__NM_001286957	211	500	234	143
CCDC65__NM_001286957_TGATGCCACAGAGGATCGAA	CCDC65__NM_001286957	157	198	174	226
CCDC65__NM_001286957_TGTACAACTGCGAAAACTTA	CCDC65__NM_001286957	123	65	190	243
CCDC65__NM_001286957_TTCATCAGTATTGACTCCTA	CCDC65__NM_001286957	99	103	76	45
CCDC65__NM_033124_ACTGCTCCTCGGCTTCGGAC	CCDC65__NM_033124	810	786	908	492
CCDC65__NM_033124_CAAGACCAGAGAACTTCATA	CCDC65__NM_033124	227	187	309	218
CCDC65__NM_033124_CAGGTCTTTAGCTAAAGACT	CCDC65__NM_033124	93	90	113	21
CCDC65__NM_033124_CCTGGAGGAAAGTTACAACA	CCDC65__NM_033124	515	598	554	480
CCDC65__NM_033124_CTCGTTCAAATGTTTGGCTG	CCDC65__NM_033124	327	367	431	567
CCDC65__NM_033124_CTCTGGTCTTGACTTCCCGA	CCDC65__NM_033124	141	93	182	139
CCDC65__NM_033124_GCAGCTGCTTCTCATCGGAC	CCDC65__NM_033124	634	934	576	576
CCDC66__NM_001012506_ACCTACTAGAGATGTATTTA	CCDC66__NM_001012506	524	488	589	536
CCDC66__NM_001012506_AGTGGAGGAAAGAGCTAGGT	CCDC66__NM_001012506	249	222	239	173
CCDC66__NM_001012506_ATCAAATTCTTGACCAATCT	CCDC66__NM_001012506	233	283	262	390
CCDC66__NM_001012506_ATCTGTCTTAAATGAACATC	CCDC66__NM_001012506	83	100	76	65
CCDC66__NM_001012506_CCTCCTCTGAAGGTTCAACC	CCDC66__NM_001012506	410	197	344	318
CCDC66__NM_001012506_CTCCTTTCCATTCTCAGTCA	CCDC66__NM_001012506	597	586	523	488
CCDC66__NM_001012506_GTCCTTTAAGAACAAAAACT	CCDC66__NM_001012506	197	73	201	214
CCDC66__NM_001012506_TCAAATTCTTGACCAATCTA	CCDC66__NM_001012506	14	4	2	0
CCDC66__NM_001012506_TCAACACAAGATACTTGTAT	CCDC66__NM_001012506	890	670	934	748
CCDC66__NM_001012506_TCTAGTCTTCTGCATATTAG	CCDC66__NM_001012506	87	46	122	82
CCDC66__NM_001012506_TTCTAGTCTTCTGCATATTA	CCDC66__NM_001012506	2	38	2	4
CCDC66__NM_001012506_TTCTGCATATTAGGGGTTGC	CCDC66__NM_001012506	0	0	0	0
CCDC66__NM_001141947_AAGCTAATATTGTCTCCATA	CCDC66__NM_001141947	260	105	259	181
CCDC66__NM_001141947_ATATGGAGACAATATTAGCT	CCDC66__NM_001141947	387	350	507	471
CCDC66__NM_001141947_CTTGAAACTGAATTACTGGA	CCDC66__NM_001141947	474	468	604	617
CCDC67__NM_181645_AACCAAGCCCATAATACGAT	CCDC67__NM_181645	801	690	1132	1184
CCDC67__NM_181645_ACCAAGCCCATAATACGATG	CCDC67__NM_181645	583	569	640	576
CCDC67__NM_181645_AGGACAACTACAAAGCCTAA	CCDC67__NM_181645	227	183	341	497
CCDC67__NM_181645_AGGGTTACTTCGACAGAAAT	CCDC67__NM_181645	127	132	73	100
CCDC67__NM_181645_AGTTACCAAACTCAACTAAA	CCDC67__NM_181645	51	15	81	117
CCDC67__NM_181645_CAATTTGAACCAGAAATTAG	CCDC67__NM_181645	220	311	261	400
CCDC67__NM_181645_GAACCAAGCCCATAATACGA	CCDC67__NM_181645	837	695	771	649
CCDC67__NM_181645_GCAATGACTCAGAATTATGA	CCDC67__NM_181645	253	446	315	411
CCDC67__NM_181645_GGAAAGAAAGATGCGGGCTT	CCDC67__NM_181645	233	330	222	226
CCDC67__NM_181645_TCGACAGAAATTGGACAGTC	CCDC67__NM_181645	900	1227	989	1359
CCDC67__NM_181645_TCTGGGTCACATATCAAACG	CCDC67__NM_181645	266	183	199	271
CCDC67__NM_181645_TTAGAGCAAAGTCAAGAGAA	CCDC67__NM_181645	39	63	38	30
CCDC68__NM_001143829_AGAAACCGAGTATGTGAAAA	CCDC68__NM_001143829	220	227	229	226
CCDC68__NM_001143829_AGCAGCAGCTCTGAGAAACG	CCDC68__NM_001143829	49	23	59	62
CCDC68__NM_001143829_ATCTGAAACTCCTAGGACAA	CCDC68__NM_001143829	64	152	56	64
CCDC68__NM_001143829_CCCCTTATTTAATGTTGATT	CCDC68__NM_001143829	519	562	538	631
CCDC68__NM_001143829_CTCAAATCCAGTGCTACATA	CCDC68__NM_001143829	195	200	289	292
CCDC68__NM_001143829_CTCTGAAGATCCTGGCAACT	CCDC68__NM_001143829	661	792	706	818
CCDC68__NM_001143829_TATCTAAGGCTGTCTCTACA	CCDC68__NM_001143829	326	322	368	501
CCDC68__NM_001143829_TCACTTCTTCAGATTGCGTT	CCDC68__NM_001143829	872	1080	1105	1613
CCDC68__NM_001143829_TGTTCATTTCTAAGAGCTGT	CCDC68__NM_001143829	46	63	63	129
CCDC69__NM_015621_AATTCTCCAGCAACATGAGG	CCDC69__NM_015621	221	178	222	229
CCDC69__NM_015621_ACTCACCTGTGTCCCCATTG	CCDC69__NM_015621	1023	767	1143	859
CCDC69__NM_015621_AGACCAGAGCCCCATGAATT	CCDC69__NM_015621	770	703	745	712
CCDC69__NM_015621_CAGCTGCAAACCCCCGAAAA	CCDC69__NM_015621	902	1485	1105	1304
CCDC69__NM_015621_CATTGAGGGGACCTAATTCA	CCDC69__NM_015621	475	471	567	593
CCDC69__NM_015621_CCATGTCCGAAGCCGCAACC	CCDC69__NM_015621	1315	1289	1454	1685
CCDC69__NM_015621_GGACCACCTGGTTGCGGCTT	CCDC69__NM_015621	1409	1554	1511	1766
CCDC69__NM_015621_GTCTCTTTCCTCGCCACATA	CCDC69__NM_015621	320	329	372	397
CCDC69__NM_015621_TTCTTTCTCCTGTTCATATG	CCDC69__NM_015621	0	0	0	0
CCDC69__NM_015621_TTGAGGGGACCTAATTCATG	CCDC69__NM_015621	764	974	946	1088
CCDC7__NM_001026383_AAGTTCTTTGTCTTACTCGA	CCDC7__NM_001026383	193	276	207	261
CCDC7__NM_001026383_ACCATTATCACCTGAGCTAA	CCDC7__NM_001026383	670	1004	878	915
CCDC7__NM_001026383_AGAAGATTTGGATCAAGTAC	CCDC7__NM_001026383	177	137	214	125
CCDC7__NM_001026383_AGACAACATGACTTTCACGG	CCDC7__NM_001026383	1147	923	1234	1017
CCDC7__NM_001026383_AGGATTTAGACCTCTGTTTA	CCDC7__NM_001026383	431	474	515	806
CCDC7__NM_001026383_CCAGCATTAACTACTAAAAA	CCDC7__NM_001026383	262	370	321	389
CCDC7__NM_001026383_GGGCAAAGCATACCGTAAAA	CCDC7__NM_001026383	55	45	71	68
CCDC7__NM_001026383_GTTGACCACCAGTAACAAAT	CCDC7__NM_001026383	427	547	463	514
CCDC7__NM_001026383_TATGGACATTGCGATCAGAA	CCDC7__NM_001026383	154	154	200	234
CCDC7__NM_001026383_TGAAAACCAGGCAAATATGT	CCDC7__NM_001026383	275	342	333	482
CCDC7__NM_001026383_TGAAGAATCGCCTTAAACAG	CCDC7__NM_001026383	82	83	92	71
CCDC7__NM_001026383_TTTGTCTTACTCGATGGAAT	CCDC7__NM_001026383	79	79	73	127
CCDC7__NM_024688_AAAATCAGAAATGCAAGTGA	CCDC7__NM_024688	200	240	246	212
CCDC7__NM_024688_AAACACTTGAAAGGTGTTAA	CCDC7__NM_024688	91	131	119	225
CCDC7__NM_024688_ACACAATTAAAAAGTCACCC	CCDC7__NM_024688	360	507	351	356
CCDC7__NM_024688_AGAAAGCTTGAGAGGTGCTT	CCDC7__NM_024688	168	234	218	288
CCDC7__NM_024688_AGGAACAAAGAACTCTCAAA	CCDC7__NM_024688	230	213	279	268
CCDC7__NM_024688_CTCTTCAGTTGTGTAGTGAT	CCDC7__NM_024688	313	225	201	251
CCDC7__NM_024688_GAACAATTGACAGATGCATT	CCDC7__NM_024688	338	304	421	528
CCDC7__NM_024688_GAATCTTGATGTTTAAGCAT	CCDC7__NM_024688	260	176	318	357
CCDC7__NM_024688_GAATTCTTGGCAGATGCTAT	CCDC7__NM_024688	309	292	384	610
CCDC7__NM_024688_TACTGGAAGAGGTCTAATGA	CCDC7__NM_024688	212	304	260	290
CCDC7__NM_024688_TCTGTCAATTGTTCTATATC	CCDC7__NM_024688	224	167	286	312
CCDC7__NM_024688_TTAAAGAGAAAGAGTTACCC	CCDC7__NM_024688	68	12	85	94
CCDC70__NM_031290_ACTGGGAGAGGATGCCGCCA	CCDC70__NM_031290	483	647	648	546
CCDC70__NM_031290_ATCTTCCTTATCAGCCGGAA	CCDC70__NM_031290	154	211	186	272
CCDC70__NM_031290_CCCCGCCATTCCGGCTGATA	CCDC70__NM_031290	902	731	814	644
CCDC70__NM_031290_GAAAGAGGACAATGCCTTAT	CCDC70__NM_031290	500	729	541	708
CCDC70__NM_031290_GAAGAGATGTGGACTTTCCG	CCDC70__NM_031290	334	154	373	296
CCDC70__NM_031290_GAGAGCAGATGCTCGAAGAT	CCDC70__NM_031290	205	214	154	97
CCDC70__NM_031290_GCATCTGCTCTCCGGCAATG	CCDC70__NM_031290	643	990	739	904
CCDC70__NM_031290_GGCCCCACAACGCCAACAGA	CCDC70__NM_031290	799	701	831	747
CCDC70__NM_031290_GGCTCTTTCCTCTACCCACA	CCDC70__NM_031290	236	411	334	442
CCDC70__NM_031290_GGGCCCCACAACGCCAACAG	CCDC70__NM_031290	88	113	178	189
CCDC70__NM_031290_GTTCCGGTCTCTTTCCCATA	CCDC70__NM_031290	251	166	208	131
CCDC70__NM_031290_TGAGGGGGAGAAAGCCCTGT	CCDC70__NM_031290	625	101	667	143
CCDC71__NM_022903_AGTCTGCCAGATAGCGGCAT	CCDC71__NM_022903	369	276	358	196
CCDC71__NM_022903_CACGAGCTTGCAGTTTCGTC	CCDC71__NM_022903	1744	1860	1972	2347
CCDC71__NM_022903_CACTGAGATCCTGGCTCATT	CCDC71__NM_022903	818	690	783	479
CCDC71__NM_022903_CACTGCGCAGGATGGTAGGT	CCDC71__NM_022903	837	834	963	830
CCDC71__NM_022903_CAGTCTGCCAGATAGCGGCA	CCDC71__NM_022903	182	253	177	314
CCDC71__NM_022903_CATGCCGCTATCTGGCAGAC	CCDC71__NM_022903	3261	2931	3164	3140
CCDC71__NM_022903_CTTGCAGTTTCGTCTGGCTT	CCDC71__NM_022903	2439	3541	2680	3748
CCDC71__NM_022903_GCACTGAGATCCTGGCTCAT	CCDC71__NM_022903	675	484	932	657
CCDC71__NM_022903_GGTCGCGCATCTCCACGGCA	CCDC71__NM_022903	1306	1168	1382	1271
CCDC71__NM_022903_GTGATGTGGCAGTTGGGTTA	CCDC71__NM_022903	572	815	765	687
CCDC71__NM_022903_TGATGTGGCAGTTGGGTTAG	CCDC71__NM_022903	446	489	471	484
CCDC71__NM_022903_TTGCAGTTTCGTCTGGCTTG	CCDC71__NM_022903	269	596	340	287
CCDC71L__NM_175884_AAGGTGCGGCCCCCGAAGCA	CCDC71L__NM_175884	498	750	511	730
CCDC71L__NM_175884_ACCTTCCCCACCATCCGCGT	CCDC71L__NM_175884	2112	2637	2377	2070
CCDC71L__NM_175884_CAGCACGGAGTTCATGAGCT	CCDC71L__NM_175884	327	305	488	671
CCDC71L__NM_175884_CCAGGTCCTGCGAGTGAACC	CCDC71L__NM_175884	511	655	426	435
CCDC71L__NM_175884_CGCGTCGGCAGCGACGTGTG	CCDC71L__NM_175884	406	526	460	530
CCDC71L__NM_175884_TGGGTTCCAGGTTCACTCGC	CCDC71L__NM_175884	21	20	16	28
CCDC71L__NM_175884_TTCATACTGCGCCGCATCGA	CCDC71L__NM_175884	74	88	90	55
CCDC71L__NM_175884_TTCCCGGTGCCCCGGGCATG	CCDC71L__NM_175884	19	42	36	26
CCDC73__NM_001008391_AACATTGGCAGAGCAACACA	CCDC73__NM_001008391	130	147	133	102
CCDC73__NM_001008391_ACAGAAGAAAGTGAGTGAAA	CCDC73__NM_001008391	178	180	271	246
CCDC73__NM_001008391_ACTTCAAGAAAGACTCAACA	CCDC73__NM_001008391	463	422	565	771
CCDC73__NM_001008391_AGAAAGTGAGTGAAATGGTA	CCDC73__NM_001008391	216	368	180	209
CCDC73__NM_001008391_AGAAGAATTGCGTATGAGAA	CCDC73__NM_001008391	206	212	235	337
CCDC73__NM_001008391_AGTCACATGTCAATATAAGA	CCDC73__NM_001008391	197	174	307	269
CCDC73__NM_001008391_CTTATATTGACATGTGACTT	CCDC73__NM_001008391	357	685	373	495
CCDC73__NM_001008391_GCAGATTGGTAAAATTATTG	CCDC73__NM_001008391	366	352	379	532
CCDC73__NM_001008391_GCCACAATAACAGGTCAATT	CCDC73__NM_001008391	1210	1068	1323	1550
CCDC73__NM_001008391_GTCACATGTCAATATAAGAT	CCDC73__NM_001008391	263	162	331	235
CCDC73__NM_001008391_TAGAAGAATTGCGTATGAGA	CCDC73__NM_001008391	323	318	336	400
CCDC73__NM_001008391_TCCAAATTGACCTGTTATTG	CCDC73__NM_001008391	369	473	451	553
CCDC74A__NM_001258304_CTCAAAGCACTGAGCGATGC	CCDC74A__NM_001258304	128	219	132	170
CCDC74B__NM_001258307_AAAGCAGAGGCCTCTAACGC	CCDC74B__NM_001258307	362	339	401	458
CCDC74B__NM_001258307_ACAGGCAGCTCCTGCGTTAG	CCDC74B__NM_001258307	498	537	638	1009
CCDC74B__NM_001258307_GACAGACTGGAAGCTTGACG	CCDC74B__NM_001258307	767	742	812	737
CCDC74B__NM_207310_ACAGGCCAGGAGGCAAGCAT	CCDC74B__NM_207310	76	197	186	122
CCDC77__NM_001130146_AATTTGTTCTCGAGAAAGTT	CCDC77__NM_001130146	85	204	89	150
CCDC77__NM_001130146_AGAACTTGCCCGAATTCGTG	CCDC77__NM_001130146	784	823	1061	770
CCDC77__NM_001130146_AGGAGGCTCCTTACAAAAAT	CCDC77__NM_001130146	205	239	262	233
CCDC77__NM_001130146_ATGGTGACTCTCATGCATAA	CCDC77__NM_001130146	39	56	33	22
CCDC77__NM_001130146_CTTTCTCGAGAACAAATTGA	CCDC77__NM_001130146	19	14	95	105
CCDC77__NM_001130146_GAAAGCTCTAAGTGATATGC	CCDC77__NM_001130146	65	23	64	41
CCDC77__NM_001130146_GCCTTCCCCCGAAGATCGTC	CCDC77__NM_001130146	548	860	463	729
CCDC77__NM_001130146_GGACAGACGGATTCACCTTG	CCDC77__NM_001130146	999	1012	1137	1034
CCDC77__NM_001130146_GGCCAAACTCCATCCTTCTA	CCDC77__NM_001130146	1849	2813	2263	2852
CCDC77__NM_001130146_GTTTGGCCAGACGATCTTCG	CCDC77__NM_001130146	490	565	549	650
CCDC77__NM_001130146_TGGTGACTCTCATGCATAAC	CCDC77__NM_001130146	36	12	44	99
CCDC77__NM_001130146_TTTGGCCAGACGATCTTCGG	CCDC77__NM_001130146	254	119	380	217
CCDC77__NM_032358_AAACGTGGTGTTGCCGTCAG	CCDC77__NM_032358	135	109	146	130
CCDC77__NM_032358_CGAACAGTTGTCTCCAAACG	CCDC77__NM_032358	983	1172	1247	1550
CCDC78__NM_001031737_ACATTCTCCACCCGCCGAGA	CCDC78__NM_001031737	1725	1099	2020	1345
CCDC78__NM_001031737_ACCAGATCTAGCGCTCAATA	CCDC78__NM_001031737	288	345	336	320
CCDC78__NM_001031737_CCTCATGCTGCTCATGTAGG	CCDC78__NM_001031737	1986	2574	2341	2520
CCDC78__NM_001031737_CCTTGGCTCGTAGCACAACC	CCDC78__NM_001031737	254	371	368	397
CCDC78__NM_001031737_CTCTCCAGCCGAAGGATCTG	CCDC78__NM_001031737	34	16	37	19
CCDC78__NM_001031737_GCCTGGGGTCAGACTCCACT	CCDC78__NM_001031737	1284	1405	1419	1100
CCDC78__NM_001031737_GCCTGTGCTGCTCATTCTCG	CCDC78__NM_001031737	2879	3552	3221	4257
CCDC78__NM_001031737_GTGGTGGCTGCGTGCTCCAT	CCDC78__NM_001031737	248	264	243	246
CCDC78__NM_001031737_TACGGCTGCAACACTGCGCC	CCDC78__NM_001031737	29	13	39	47
CCDC78__NM_001031737_TATTGAGCGCTAGATCTGGT	CCDC78__NM_001031737	238	218	258	214
CCDC78__NM_001031737_TCCTTATTGAGCGCTAGATC	CCDC78__NM_001031737	79	28	131	107
CCDC78__NM_001031737_TGAAGGATGAGTACGTCCTA	CCDC78__NM_001031737	269	212	236	179
CCDC79__NM_001136505_AACCACTGCAAGTTTAGCAC	CCDC79__NM_001136505	663	521	863	1009
CCDC79__NM_001136505_ATTAAAGGACATAAGTGTGA	CCDC79__NM_001136505	274	379	239	211
CCDC79__NM_001136505_ATTACTGCTTCATGAAAGTC	CCDC79__NM_001136505	213	231	347	369
CCDC79__NM_001136505_CATCTCCATGCAGATCGGAT	CCDC79__NM_001136505	823	613	938	930
CCDC79__NM_001136505_CGCCCTATTTGCTCATTTAT	CCDC79__NM_001136505	1067	1460	1263	1756
CCDC79__NM_001136505_CTCATTCTGCGATTCAGTTA	CCDC79__NM_001136505	352	374	403	487
CCDC79__NM_001136505_CTTAACTGAATCGCAGAATG	CCDC79__NM_001136505	128	193	166	194
CCDC79__NM_001136505_GACACTCCAATAATAAGTTC	CCDC79__NM_001136505	442	664	574	590
CCDC79__NM_001136505_GACTGACCTGAACTTATTAT	CCDC79__NM_001136505	939	1227	1030	1173
CCDC79__NM_001136505_GCAGTTGTGAAGCACAAATG	CCDC79__NM_001136505	301	156	293	196
CCDC79__NM_001136505_GGAGTGTCTAAAGTATCAAA	CCDC79__NM_001136505	231	273	219	193
CCDC79__NM_001136505_TGCTTTAGACATGACTCCAT	CCDC79__NM_001136505	204	224	218	133
CCDC8__NM_032040_AGACAGCACCCACAGGTCAG	CCDC8__NM_032040	383	473	498	578
CCDC8__NM_032040_AGAGGTTACAGATAATCAAA	CCDC8__NM_032040	34	44	51	31
CCDC8__NM_032040_AGGGGTATCATCGGCGCCAG	CCDC8__NM_032040	610	668	699	576
CCDC8__NM_032040_CAAGTTCCTGGGAAGCGACC	CCDC8__NM_032040	387	423	477	581
CCDC8__NM_032040_CACAGGGCACAGATAACCAC	CCDC8__NM_032040	530	473	665	879
CCDC8__NM_032040_CCACCGCAGTGGAGAGCGCA	CCDC8__NM_032040	197	140	303	367
CCDC8__NM_032040_CTTGGGCCTCCATCGCCTAG	CCDC8__NM_032040	600	687	817	751
CCDC8__NM_032040_GCACTGGCAGGTTGACGCGG	CCDC8__NM_032040	1978	2235	1929	1745
CCDC8__NM_032040_GGGGATCGCTTGGGAAACGC	CCDC8__NM_032040	176	227	140	144
CCDC8__NM_032040_GTGGCGGGAGTATGTCAGCC	CCDC8__NM_032040	286	245	337	538
CCDC8__NM_032040_TCTTGGGCCTCCATCGCCTA	CCDC8__NM_032040	508	527	726	711
CCDC8__NM_032040_TTGCTGCCCAGGTAGCTGAC	CCDC8__NM_032040	269	210	283	373
CCDC80__NM_199511_ACCCCGTTTCACTATGCTGT	CCDC80__NM_199511	1198	1473	1522	1763
CCDC80__NM_199511_CACCCCCATGCCACTATTAG	CCDC80__NM_199511	966	1397	1139	1240
CCDC80__NM_199511_GCCAACAGCATAGTGAAACG	CCDC80__NM_199511	2	0	0	0
CCDC80__NM_199511_GGCCAACAGCATAGTGAAAC	CCDC80__NM_199511	0	0	0	0
CCDC80__NM_199511_GGGCGAGCTAGTCTCAACAC	CCDC80__NM_199511	446	555	400	584
CCDC80__NM_199511_GTAGGCCAGCTCGGTTTCTG	CCDC80__NM_199511	584	717	617	636
CCDC80__NM_199511_TCCGTGGCTGCCTCTAATAG	CCDC80__NM_199511	1586	1952	1767	1569
CCDC80__NM_199511_TGATCCACACACTAGCCACA	CCDC80__NM_199511	201	259	303	390
CCDC80__NM_199511_TGCCTCTAATAGTGGCATGG	CCDC80__NM_199511	220	261	207	329
CCDC80__NM_199511_TGGCCAACAGCATAGTGAAA	CCDC80__NM_199511	0	0	0	0
CCDC80__NM_199511_TGGGCGAGCTAGTCTCAACA	CCDC80__NM_199511	161	175	183	164
CCDC80__NM_199511_TTGGCCATGTGGCTAGTGTG	CCDC80__NM_199511	158	160	150	135
CCDC81__NM_001156474_AGCTTGCCAGGATCATAACA	CCDC81__NM_001156474	360	316	399	417
CCDC81__NM_001156474_ATGTATCAGAATTTGTGAGA	CCDC81__NM_001156474	248	159	292	154
CCDC81__NM_001156474_CAGAAGTCTCTATTATCTGG	CCDC81__NM_001156474	775	316	771	297
CCDC81__NM_001156474_CCAGAGGCCTGTGTTTATCA	CCDC81__NM_001156474	271	338	300	257
CCDC81__NM_001156474_GAGACCCTTGTAGAAGAATG	CCDC81__NM_001156474	404	541	470	663
CCDC81__NM_001156474_GAGGCCTGTGTTTATCATGG	CCDC81__NM_001156474	585	382	683	557
CCDC81__NM_001156474_GAGTTTACATTCAAAGGAAT	CCDC81__NM_001156474	49	30	123	153
CCDC81__NM_001156474_GCAGTGTGCTTGCGTTTCCA	CCDC81__NM_001156474	1751	2135	1966	2126
CCDC81__NM_001156474_TTACCCTCCTGGCTCAGCGA	CCDC81__NM_001156474	145	89	120	196
CCDC81__NM_001156474_TTCCCTGTTGTACTACAGTG	CCDC81__NM_001156474	25	18	44	115
CCDC81__NM_001156474_TTTGTACCATGGATGGAAGT	CCDC81__NM_001156474	214	128	291	398
CCDC81__NM_001156474_TTTGTCATGATATCCCTGGA	CCDC81__NM_001156474	572	812	460	570
CCDC81__NM_021827_GTATCAGATGTTAAAGGATC	CCDC81__NM_021827	130	92	122	141
CCDC81__NM_021827_TTCCTCACTGTAGTACAACA	CCDC81__NM_021827	70	57	43	65
CCDC81__NM_021827_TTTCCTCACTGTAGTACAAC	CCDC81__NM_021827	91	170	91	129
CCDC82__NM_024725_AAAAGAAAATGGCTGGATTA	CCDC82__NM_024725	274	372	262	332
CCDC82__NM_024725_AGAAATCTCGAGTTGATTGG	CCDC82__NM_024725	333	310	341	282
CCDC82__NM_024725_AGCAGAAATCTCGAGTTGAT	CCDC82__NM_024725	404	450	476	472
CCDC82__NM_024725_AGTAAGTGTCTCATTAACTC	CCDC82__NM_024725	125	112	148	134
CCDC82__NM_024725_ATCACTGTCACACATCACAG	CCDC82__NM_024725	576	779	548	553
CCDC82__NM_024725_ATGATAAATTCTGGTACGGC	CCDC82__NM_024725	226	234	227	196
CCDC82__NM_024725_CAGGAGAGTTGTATAACACC	CCDC82__NM_024725	285	291	406	385
CCDC82__NM_024725_CTCTTTCAAAATGAGTATAG	CCDC82__NM_024725	17	3	20	28
CCDC82__NM_024725_GAAGAAGAAGACATCAAGCG	CCDC82__NM_024725	288	296	439	324
CCDC82__NM_024725_GATACCAAGCTCTCTAGACG	CCDC82__NM_024725	474	457	636	463
CCDC82__NM_024725_TGAAGAGCTTGATAGTAACA	CCDC82__NM_024725	245	453	230	205
CCDC82__NM_024725_TTCATCTTCAACCACTCTAC	CCDC82__NM_024725	34	36	18	23
CCDC83__NM_001286159_AACTCAGTGAAAAGGAATAT	CCDC83__NM_001286159	243	222	237	276
CCDC83__NM_001286159_ATAAAAAGGATACACATGAC	CCDC83__NM_001286159	220	393	299	234
CCDC83__NM_001286159_ATAGTCTAGAAGTGCTTCAC	CCDC83__NM_001286159	130	24	158	137
CCDC83__NM_001286159_CAAGGAAACTTTGTTGCAAC	CCDC83__NM_001286159	477	706	610	797
CCDC83__NM_001286159_CTCAGGGAAAGCAAATAAAA	CCDC83__NM_001286159	134	199	124	153
CCDC83__NM_001286159_GCACATACGGCATCTACTAA	CCDC83__NM_001286159	1472	1928	1437	1819
CCDC83__NM_001286159_GCTGTAAAGCTCATTGACAA	CCDC83__NM_001286159	775	832	981	960
CCDC83__NM_001286159_GCTTAAAAGAAGAACAGATT	CCDC83__NM_001286159	595	805	585	907
CCDC83__NM_001286159_GGATGTTGAAGAAGCGATGA	CCDC83__NM_001286159	251	218	279	339
CCDC83__NM_001286159_GGGCAGTTATCTAGAGATCT	CCDC83__NM_001286159	191	306	229	240
CCDC83__NM_001286159_TGGGAGGAGTACAAGAATGT	CCDC83__NM_001286159	149	185	211	207
CCDC83__NM_001286159_TTGTGGATCTCAAGATACCC	CCDC83__NM_001286159	93	112	100	83
CCDC84__NM_198489_AAACTCCCCCCAGACCGAGT	CCDC84__NM_198489	681	11	765	10
CCDC84__NM_198489_AAAGGAGGATAAAGTGATCA	CCDC84__NM_198489	208	39	242	102
CCDC84__NM_198489_AACTCCCCCCAGACCGAGTT	CCDC84__NM_198489	851	90	915	283
CCDC84__NM_198489_AAGATGAAGAATACATTGCT	CCDC84__NM_198489	445	187	414	286
[illegible]	[illegible]	[illegible]	[illegible]	[illegible]	[illegible]
CCDC88B__NM_032251_GCTGGCACGGGAGCGTGACC	CCDC88B__NM_032251	512	427	610	553
CCDC88B__NM_032251_GGCGTTCTTCGGCTACTGTT	CCDC88B__NM_032251	838	959	692	811
CCDC88B__NM_032251_GTGGAACCTGAACCACCTGT	CCDC88B__NM_032251	307	272	357	417
CCDC88C__NM_001080414_CATCGTGGCCCATATCCAGG	CCDC88C__NM_001080414	772	676	826	686
CCDC88C__NM_001080414_CTAAATCCATGTACATAGTC	CCDC88C__NM_001080414	111	142	140	139
CCDC88C__NM_001080414_CTTACATTTGCAGCATAATT	CCDC88C__NM_001080414	116	78	115	123
CCDC88C__NM_001080414_CTTATTGATGCGTTGATTTG	CCDC88C__NM_001080414	292	524	241	414
CCDC88C__NM_001080414_GGACAACCTGACTATGTACA	CCDC88C__NM_001080414	493	470	554	659
CCDC88C__NM_001080414_GGAGCACCATGCTCCTCGAC	CCDC88C__NM_001080414	203	154	228	291
CCDC88C__NM_001080414_TCCTGGCTGCCGCTTCCAAA	CCDC88C__NM_001080414	856	800	976	909
[illegible]	[illegible]	[illegible]	[illegible]	[illegible]	[illegible]
CCDC93__NM_019044_CAGCAGAAATATGAGGGCAC	CCDC93__NM_019044	326	275	293	361
CCDC93__NM_019044_CAGGCTTCTCCGGAGTTAAA	CCDC93__NM_019044	366	436	510	406
CCDC93__NM_019044_CATCATCTGTCCCCATGGTT	CCDC93__NM_019044	1052	716	1326	1085
CCDC93__NM_019044_CCAAGTGACCAACACTACAT	CCDC93__NM_019044	191	147	152	233
CCDC93__NM_019044_CCCAAGTGACCAACACTACA	CCDC93__NM_019044	153	209	144	180
CCDC93__NM_019044_CCCTGCACTCAAGGCAAGTA	CCDC93__NM_019044	549	769	826	969
CCDC93__NM_019044_CCGTCTCACCGCAAGCTCCG	CCDC93__NM_019044	1369	1039	1456	1782
CCDC93__NM_019044_CCTCACTTAATCATGCATCT	CCDC93__NM_019044	639	966	739	765
CCDC93__NM_019044_CTCAAGGCAAGTAGGGTGTC	CCDC93__NM_019044	127	176	169	191
CCDC93__NM_019044_CTGCTGGGTACTTGTGCAGT	CCDC93__NM_019044	931	910	1140	1152
[illegible]	[illegible]	[illegible]	[illegible]	[illegible]	[illegible]
CCER1__NM_152638_TATAGCCCCAAGACCGAGTA	CCER1__NM_152638	452	465	522	683
CCER1__NM_152638_TGACTCAGACCCTCGACACA	CCER1__NM_152638	1273	1057	1398	1585
CCER2__NM_001243212_AACAAGCCATCCGCATGCAA	CCER2__NM_001243212	361	382	327	268
CCER2__NM_001243212_AGAGAGGACCCACAAGTCTG	CCER2__NM_001243212	169	181	221	148
CCER2__NM_001243212_CCTCGGTGGACGCACAGCCG	CCER2__NM_001243212	307	362	314	487
CCER2__NM_001243212_CTCGGTGGACGCACAGCCGT	CCER2__NM_001243212	346	300	401	405
CCER2__NM_001243212_GAACAAGCCATCCGCATGCA	CCER2__NM_001243212	589	1058	783	850
CCER2__NM_001243212_GCGATGCCCTTGCATGCGGA	CCER2__NM_001243212	111	127	228	246
CCER2__NM_001243212_GCTGGCGATGCCCTTGCATG	CCER2__NM_001243212	571	429	621	723
CCER2__NM_001243212_TTCCTGGACCTCAGACTTGT	CCER2__NM_001243212	62	30	23	60
[illegible]	[illegible]	[illegible]	[illegible]	[illegible]	[illegible]

[illegible]	[illegible]	[illegible]	[illegible]	[illegible]	[illegible]

CD69__NM_001781_GTGGGCCAATACAATTGTCC	CD69__NM_001781	438	445	363	487
CD7__NM_006137_AAAGAAACTGTGCTCGTGGC	CD7__NM_006137	790	719	911	988
CD7__NM_006137_ACTACGGACAGACGGTTCCG	CD7__NM_006137	675	991	785	717
CD7__NM_006137_CAGAGGCTGCTGGCGGGTCA	CD7__NM_006137	1191	1472	1382	1704
CD7__NM_006137_CCACGGGGACAGTCGTGCAG	CD7__NM_006137	759	790	885	661
CD7__NM_006137_CCACTACGGACAGACGGTTC	CD7__NM_006137	909	604	715	683
CD7__NM_006137_CGGAGCCTGTCGGTGGGGCA	CD7__NM_006137	466	428	400	530
CD7__NM_006137_CTCGTGGCGGGATAAGAATT	CD7__NM_006137	368	338	260	229
CD7__NM_006137_GCCTGCGTGGGATCTACCTG	CD7__NM_006137	1251	1234	1328	1467
CD7__NM_006137_GGGGCCGCATCGACTTCTCA	CD7__NM_006137	2422	2535	2697	2714
CD7__NM_006137_GTGGCGGGATAAGAATTCGG	CD7__NM_006137	205	230	191	352
CD7__NM_006137_TGGTGCCCACTACGGACAGA	CD7__NM_006137	29	26	23	133
CD70__NM_001252_AGCAGGACCCCAGGCTATAC	CD70__NM_001252	878	828	856	782
CD70__NM_001252_AGCCCGCAGGACGCACCCAT	CD70__NM_001252	1797	1659	2080	2036
CD70__NM_001252_AGCTGCCGCTCGAGTCACTT	CD70__NM_001252	147	186	199	218
CD70__NM_001252_CAGCTACGTATCCATCGTGA	CD70__NM_001252	1296	883	1492	1372
CD70__NM_001252_CAGCTGCCGCTCGAGTCACT	CD70__NM_001252	375	301	348	315
CD70__NM_001252_CCCCAGGCTATACTGGCAGG	CD70__NM_001252	574	452	605	456
CD70__NM_001252_CCCCCCTGCCAGTATAGCCT	CD70__NM_001252	0	0	0	0
CD70__NM_001252_CCCCCTGCCAGTATAGCCTG	CD70__NM_001252	3	0	7	13
CD70__NM_001252_GCAGCTCAGCTACGTCCCAC	CD70__NM_001252	41	13	66	20
CD70__NM_001252_GCCCGCAGGACGCACCCATA	CD70__NM_001252	1003	906	1029	1132
CD70__NM_001252_GCTGAGCCTGTGCGAAGCGC	CD70__NM_001252	37	54	14	25
CD70__NM_001252_GCTGCCGCTCGAGTCACTTG	CD70__NM_001252	1567	1478	1812	1472
CD72__NM_001782_ACGGGCTGAGCGGATAGGGG	CD72__NM_001782	992	1120	1096	1246
CD72__NM_001782_AGGGTGAAGCCACCTCTAGA	CD72__NM_001782	853	827	970	675
CD72__NM_001782_ATCTCCAGCCGGTTAGGACA	CD72__NM_001782	380	432	482	379
CD72__NM_001782_CATCTCCAGCCGGTTAGGAC	CD72__NM_001782	453	372	553	617
CD72__NM_001782_CCAGAACCCTGTTCGTCTGC	CD72__NM_001782	1822	2047	1744	2268
CD72__NM_001782_CCGCCTCAAGATAACGCAGC	CD72__NM_001782	780	999	910	1065
CD72__NM_001782_CCTCAGATCTGCATAGGTGA	CD72__NM_001782	82	112	120	95
CD72__NM_001782_CGCACGGGCTGAGCGGATAG	CD72__NM_001782	471	594	612	632
CD72__NM_001782_CTTACCCTGTCCTAACCGGC	CD72__NM_001782	770	732	659	647
CD72__NM_001782_CTTGGCTTCTTCTGTACTAG	CD72__NM_001782	838	977	779	1106
CD72__NM_001782_GCAGCTCCAGCAGACGAACA	CD72__NM_001782	51	92	75	75
CD72__NM_001782_GCGCACGGGCTGAGCGGATA	CD72__NM_001782	1546	1991	1596	1868
CD72__NM_001782_GCTTGGCTTCTTCTGTACTA	CD72__NM_001782	1175	1295	1441	1524
CD72__NM_001782_GGCCGAGCAGGAGGTATCGC	CD72__NM_001782	807	661	950	908
CD72__NM_001782_GGGCACTTGAACATTCTCGT	CD72__NM_001782	603	645	666	728
CD72__NM_001782_TCAAGTGCCCGCAGTCCTAG	CD72__NM_001782	837	807	907	787
CD74__NM_001025158_CAAGTATGGCAACATGACAG	CD74__NM_001025158	196	199	257	168
CD74__NM_001025158_GGATGGAAAAGCCTGTGTAC	CD74__NM_001025158	295	368	280	169
CD74__NM_001025159_AGGCCCAAGTGCGACGAGAA	CD74__NM_001025159	203	246	330	310
CD74__NM_001025159_AGTGCCTGCTCATTTCAAAC	CD74__NM_001025159	914	1190	752	876
CD74__NM_001025159_ATGGGAGCATCGGCTACTGC	CD74__NM_001025159	468	749	583	606
CD74__NM_001025159_CTGTCCACCCGGGTTCATTC	CD74__NM_001025159	574	681	643	647
CD74__NM_001025159_GGCTCCTGTTTGAAATGAGC	CD74__NM_001025159	50	69	100	94
CD74__NM_001025159_GTGTGTCTTCCCCAACGGCA	CD74__NM_001025159	104	146	150	146
CD74__NM_001025159_GTTGCCGTTCTCGTCGCACT	CD74__NM_001025159	2141	2603	2125	3010
CD74__NM_001025159_TATCTGCCACTCCAGTGCTA	CD74__NM_001025159	528	442	485	433
CD74__NM_001025159_TGGGGACCTCCGTGCCGTTG	CD74__NM_001025159	184	151	194	229
CD74__NM_001025159_TTGGGGACCTCCGTGCCGTT	CD74__NM_001025159	1003	1194	960	1112
CD79A__NM_001783_AGAAGCTCGGGTTGGATGCC	CD79A__NM_001783	327	506	296	458
CD79A__NM_001783_ATGGGGGCATATACGTGTGC	CD79A__NM_001783	438	201	333	376
CD79A__NM_001783_ATGTATGAGGACATCTCCCG	CD79A__NM_001783	326	203	432	345
CD79A__NM_001783_CGTTGTTGCTGCTATTGTGC	CD79A__NM_001783	1273	1356	1385	1938
CD79A__NM_001783_CTGGATGATCAGCGTACCAT	CD79A__NM_001783	638	890	785	831
CD79A__NM_001783_GAAGCTCGGGTTGGATGCCG	CD79A__NM_001783	611	791	665	761
CD79A__NM_001783_GAGAAGCTCGGGTTGGATGC	CD79A__NM_001783	264	248	246	388
CD79A__NM_001783_GCTGGACATCTCCTATGTTG	CD79A__NM_001783	804	846	1123	1007
CD79A__NM_001783_TCCTCCATGGCAACTACACG	CD79A__NM_001783	484	599	556	474
CD79A__NM_001783_TGCTGCTATTGTGCGGGCAT	CD79A__NM_001783	474	547	485	569
CD79B__NM_000626_ACTGGTCCAGAGCCCGCAAA	CD79B__NM_000626	400	440	434	600
CD79B__NM_000626_AGTACCAGCAGCCAGATCGG	CD79B__NM_000626	453	325	471	319
CD79B__NM_000626_CACGTTTCATAGCCAGGAAA	CD79B__NM_000626	165	160	130	332
CD79B__NM_000626_CATCCAAGGCATCCGGTTTG	CD79B__NM_000626	1126	1077	1425	1275
CD79B__NM_000626_CCAGATCGGAGGACCGGTAC	CD79B__NM_000626	1718	1954	1731	2170
CD79B__NM_000626_CGTTCTGGGACTCTTCCATG	CD79B__NM_000626	844	804	841	881
CD79B__NM_000626_CGTTTCCTGGCTATGAAACG	CD79B__NM_000626	145	123	217	322
CD79B__NM_000626_GAGCCAGCTCACATTGCCGG	CD79B__NM_000626	2029	1431	1818	1149
CD79B__NM_000626_GCAGCCCTGGTAGACCTCCG	CD79B__NM_000626	1008	990	1145	1146
CD79B__NM_000626_GCGCCTCCGGCAATGTGAGC	CD79B__NM_000626	264	86	312	297
CD79B__NM_000626_GGAAGGGCCATGGACCCAAC	CD79B__NM_000626	291	150	242	232
CD79B__NM_000626_GGCACAGAGCTGCGAGTCAT	CD79B__NM_000626	988	950	961	957
CD79B__NM_000626_GGGTGGCGAGAGATTCGTTC	CD79B__NM_000626	547	587	635	807
CD79B__NM_000626_GTTTCCTGGCTATGAAACGT	CD79B__NM_000626	281	296	276	292
CD79B__NM_000626_TCCGTCCCCACACATGGGAT	CD79B__NM_000626	1075	1014	1116	1282
CD79B__NM_000626_TGCCTCCCCCATCCCATGTG	CD79B__NM_000626	510	449	538	303
CD79B__NM_021602_GGAGGAAGATCACACCTACG	CD79B__NM_021602	1066	1303	1125	1176
CD79B__NM_021602_GGGGAAGTGAAGTGGTCTGT	CD79B__NM_021602	931	924	873	843
CD79B__NM_021602_TGGCTGTCTGGTCAATGTCC	CD79B__NM_021602	27	9	19	2
CD80__NM_005191_ATAATTTGCTCAACCTCTGG	CD80__NM_005191	104	317	149	252
CD80__NM_005191_ATGAAAAAGACGCTTTCAAG	CD80__NM_005191	354	598	385	426
CD80__NM_005191_CGTATGTGCCCTCGTCAGAT	CD80__NM_005191	1277	1260	1381	1555
CD80__NM_005191_CTTTCACTTCCTTGGTCACG	CD80__NM_005191	508	598	647	953
CD80__NM_005191_GAAGTGACGTTATCAGTCAA	CD80__NM_005191	523	461	490	551
CD80__NM_005191_GAGCCAGGATCACAATGGAG	CD80__NM_005191	295	289	366	352
CD80__NM_005191_GCTCTGCGCCCATCTGACGA	CD80__NM_005191	161	251	189	168
CD80__NM_005191_GGCTCTGCGCCCATCTGACG	CD80__NM_005191	248	230	301	252
CD80__NM_005191_GGTCACGTGGATAACACCTA	CD80__NM_005191	528	473	481	506
CD80__NM_005191_TGGCACAAACTCGCATCTAC	CD80__NM_005191	73	116	33	99
CD80__NM_005191_TGGTGCTGACTATGATGTCT	CD80__NM_005191	22	13	25	25
CD80__NM_005191_TTGGGAAACTGTTGTGTTGA	CD80__NM_005191	181	142	249	417
CD81__NM_004356_AGACCTTCCACGAGACGGTG	CD81__NM_004356	67	65	77	52
CD81__NM_004356_CAACCTCCTGTATCTGGAGC	CD81__NM_004356	462	723	590	766
CD81__NM_004356_CACCACAGCCTTGGCGTTGT	CD81__NM_004356	1618	1781	1915	2313
CD81__NM_004356_CATGATCACAGCGACCACGA	CD81__NM_004356	854	1424	928	998
CD81__NM_004356_CGGAACAGCTCCGTGTACTG	CD81__NM_004356	1121	1559	1376	1464
CD81__NM_004356_GCTTCACATCCTTGGCGATC	CD81__NM_004356	981	1609	1112	1559
CD81__NM_004356_GGCGCTGTCATGATGTTCGT	CD81__NM_004356	2190	2653	2389	2994
CD81__NM_004356_TCCGGGAAGCTGTACCTCAT	CD81__NM_004356	1340	1583	1435	1239
CD81__NM_004356_TGTCTCCCAGCTCCAGATAC	CD81__NM_004356	233	266	289	244
CD81__NM_004356_TGTGAAGCAGTTCTATGACC	CD81__NM_004356	447	356	424	368
CD82__NM_001024844_AAACCTCCTCCAGCTCGCTT	CD82__NM_001024844	685	618	796	676
CD82__NM_001024844_AGACTACAACAGCAGTCGCG	CD82__NM_001024844	350	341	444	450
CD82__NM_001024844_AGAGATGAAACTGCTCTTGT	CD82__NM_001024844	467	450	547	572
CD82__NM_001024844_CCCAGCCGCAGCACTTCACC	CD82__NM_001024844	1686	1521	1966	2105
CD82__NM_001024844_CCTCCAGCTCGCTTAGGATG	CD82__NM_001024844	904	993	1008	1059
CD82__NM_001024844_CTCCAGCTCGCTTAGGATGG	CD82__NM_001024844	420	594	561	403
CD82__NM_001024844_GAAGAGGAAGAGAAAGTATT	CD82__NM_001024844	231	320	248	259
CD82__NM_001024844_GATCCTGGGCGCAGTGATCC	CD82__NM_001024844	341	462	357	411
CD82__NM_001024844_GGCCTATGTCTTCATCGGCG	CD82__NM_001024844	1254	1723	1446	1826
CD82__NM_001024844_GGGGCAGTCACTATGCTCAT	CD82__NM_001024844	1026	1268	1095	1264
CD82__NM_001024844_GTATTTGGTGACTTTGATAC	CD82__NM_001024844	209	195	244	227
CD82__NM_001024844_TCCTCCAGCTCGCTTAGGAT	CD82__NM_001024844	543	405	477	507
CD82__NM_002231_AGAGGGTCTTCGCAGTGAAG	CD82__NM_002231	245	384	193	260
CD82__NM_002231_AGGCTGGCAGCGGATAGGCC	CD82__NM_002231	213	487	264	434
CD82__NM_002231_ATACAATATACACACCCTTG	CD82__NM_002231	637	643	727	567
CD82__NM_002231_ATAGGCCAGGAGAGATCGCT	CD82__NM_002231	337	429	524	490
CD82__NM_002231_CAAGGGTGTGTATATTGTAT	CD82__NM_002231	261	219	348	281
CD82__NM_002231_GAGGGTCTTCGCAGTGAAGT	CD82__NM_002231	167	158	214	177
CD82__NM_002231_GGAGCAGGAAAGCAAAGTAC	CD82__NM_002231	252	182	306	294
CD82__NM_002231_GGGGCAACTGTATGAAAAAT	CD82__NM_002231	250	240	332	464
CD82__NM_002231_TTTCAGTCAGGATGGGCAAG	CD82__NM_002231	27	27	32	29
CD83__NM_001040280_ACACTACCAGCTGCAACTCG	CD83__NM_001040280	987	550	968	840
CD83__NM_001040280_ACCTCCCCAAATAAGCATTT	CD83__NM_001040280	620	616	644	620
CD83__NM_001040280_ATGCCAGCTTTAGAAAATC	CD83__NM_001040280	83	152	154	161
CD83__NM_001040280_GCAGAGAAACCTAAGTGGCA	CD83__NM_001040280	664	752	634	564
CD83__NM_001040280_GCTGCAACTCGGGGACATAC	CD83__NM_001040280	542	746	678	683
CD83__NM_001040280_GGATCCGCAGGTTCCCTACA	CD83__NM_001040280	239	272	201	256
CD83__NM_001040280_GTATGTCCCCGAGTTGCAGC	CD83__NM_001040280	338	497	303	466
CD83__NM_001040280_GTCTCTTCTTTACGCTGTGC	CD83__NM_001040280	158	216	172	309
CD83__NM_001040280_TATCATCAGAAGGGGCAAAA	CD83__NM_001040280	111	83	131	174
CD83__NM_001040280_TATTGGAGGGTGGTGAAGAG	CD83__NM_001040280	204	299	237	243
CD83__NM_001040280_TCTCTTCTTTACGCTGTGCA	CD83__NM_001040280	275	607	262	274
CD83__NM_004233_AAGTTCATGGGTATAAGTGC	CD83__NM_004233	207	153	197	153
CD83__NM_004233_AGAGCTATTTAATGGCCGGC	CD83__NM_004233	386	390	385	441
CD83__NM_004233_AGATCATGCACCCAGATAGT	CD83__NM_004233	804	577	823	723
CD83__NM_004233_CAAGATAGAGAGCTATTTAA	CD83__NM_004233	204	185	262	249
CD83__NM_004233_CATAGGTAAACTTACATCAT	CD83__NM_004233	253	298	297	190
CD83__NM_004233_CGCCCACTTGTCCCACTATC	CD83__NM_004233	1897	2139	2257	2753
CD83__NM_004233_CTCCCAGACGTAAAACATCT	CD83__NM_004233	528	702	685	890
CD83__NM_004233_CTGGGCTGACGGTGCAGTCT	CD83__NM_004233	843	833	837	969
CD83__NM_004233_GATTACTCCTTAGTAATAGC	CD83__NM_004233	68	42	59	30
CD83__NM_004233_GCTGGGCTGACGGTGCAGTC	CD83__NM_004233	788	1100	1059	1341
CD83__NM_004233_GTGCCTGTCTGTTACACTGG	CD83__NM_004233	979	1359	1163	1138
CD83__NM_004233_TGGCCCGTGACAGACTCCTG	CD83__NM_004233	60	52	74	42
CD83__NM_004233_TTCACTGTCCCCCTAATATT	CD83__NM_004233	251	231	382	340
CD84__NM_001184879_AAAGGAGCAAGATCCATAGG	CD84__NM_001184879	307	449	350	318
CD84__NM_001184879_ATCTGGTCATTAGCGATCTG	CD84__NM_001184879	321	274	398	266
CD84__NM_001184879_CAAAGCAGACATAAATACAC	CD84__NM_001184879	1130	784	1194	827
CD84__NM_001184879_CCCACAGAAATTATTATGAA	CD84__NM_001184879	1366	1602	1701	1957
CD84__NM_001184879_CGGAAGCCCATTGCGATGTC	CD84__NM_001184879	2370	2435	2721	3226
CD84__NM_001184879_CTAATGACCAGATTGTAGTT	CD84__NM_001184879	1740	1395	1915	1899
CD84__NM_001184879_CTGTGGGTCACAGTAACTAC	CD84__NM_001184879	882	810	769	645
CD84__NM_001184879_CTTAGGTCCGAACTACAATC	CD84__NM_001184879	452	374	680	514
CD84__NM_001184879_GCATGTCAGTGTGACATTAC	CD84__NM_001184879	1581	1685	1631	1726
CD84__NM_001184879_GGCAAGTTAAAATCATTGCT	CD84__NM_001184879	1281	1621	1310	1696
CD84__NM_001184879_GGTCATTAGCGATCTGAGGA	CD84__NM_001184879	410	716	444	450
CD84__NM_001184879_GTGGTTCTTGGATATTTACA	CD84__NM_001184879	0	0	0	0
CD86__NM_001206924_AATTCAACTATCGAGTATGA	CD86__NM_001206924	133	109	189	137
CD86__NM_001206924_AGTTTCCAGAATACAGAAGA	CD86__NM_001206924	223	286	156	251
CD86__NM_001206924_CATCAGATCTTTCAGGTATA	CD86__NM_001206924	322	397	411	464
CD86__NM_001206924_CCACATTTATAAGAGTTGCG	CD86__NM_001206924	535	856	518	771
CD86__NM_001206924_CCTCGCAACTCTTATAAATG	CD86__NM_001206924	649	730	822	861
CD86__NM_001206924_GTCATATTGCTCGTAACATC	CD86__NM_001206924	16	50	44	58
CD86__NM_001206924_TCATATTGCTCGTAACATCA	CD86__NM_001206924	45	24	38	11
CD86__NM_001206924_TTATCTTCACCTTTCTCTAT	CD86__NM_001206924	170	167	289	289
CD86__NM_006889_AAATAAGCTTGAATCTTCAG	CD86__NM_006889	177	214	182	103
CD86__NM_006889_ACTTGTGCGCCCATATACT	CD86__NM_006889	1117	1467	992	937
CD86__NM_006889_CAGTGTTCATTCCAAGTATA	CD86__NM_006889	399	425	336	477
CD86__NM_006889_CTACTAGCTCACTCAGGCTT	CD86__NM_006889	349	362	395	424
CD86__NM_006889_GGAAAACTTGGTTCTGAATG	CD86__NM_006889	121	132	178	285
CD8A__NM_001145873_ACGTATCTCGCCGAAAGGCT	CD8A__NM_001145873	324	244	433	527
CD8A__NM_001145873_AGGTTCCAGGTCCGATCCAG	CD8A__NM_001145873	120	167	160	91
CD8A__NM_001145873_CGAGCAGCCCGACGTCGGGT	CD8A__NM_001145873	4075	4157	4694	4956
CD8A__NM_001145873_CGAGGGCTACTATTTCTGCT	CD8A__NM_001145873	3723	3634	3839	4043
CD8A__NM_001145873_CGCCCAGATGTAGATATCAC	CD8A__NM_001145873	1094	952	1082	1456
CD8A__NM_001145873_CTGCTGTCCAACCCGACGTC	CD8A__NM_001145873	1693	1820	1841	2250
CD8A__NM_001145873_GACTTCCGCCGAGAGAACGA	CD8A__NM_001145873	340	426	476	427
CD8A__NM_001145873_GCTGCTGTCCAACCCGACGT	CD8A__NM_001145873	1590	1468	1685	1297
CD8A__NM_001145873_GGCAGGAGCAAGGCGGTCAC	CD8A__NM_001145873	530	385	492	448
CD8A__NM_001145873_GGCGCCGGTGTTGGTGGTCG	CD8A__NM_001145873	2342	3148	2985	3726
CD8A__NM_001145873_TGTCGCCGCTGGATCGGACC	CD8A__NM_001145873	972	1572	1002	1055
CD8A__NM_001145873_TTTCAGGCCTGTGGTCAAAT	CD8A__NM_001145873	99	149	73	182
CD8A__NM_171827_GTTAGACGTATCTCGCCGAA	CD8A__NM_171827	458	461	524	608
CD8B__NM_001178100_AGGAGAAGATAGCTGTGTTT	CD8B__NM_001178100	877	895	1006	780
CD8B__NM_001178100_AGTAGATGCGCATGTTACTG	CD8B__NM_001178100	424	220	458	308
CD8B__NM_001178100_ATCTACTTCTGCATGATCGT	CD8B__NM_001178100	233	128	321	188
CD8B__NM_001178100_CACAGAAGCTGCTTAACCCA	CD8B__NM_001178100	400	392	600	536
CD8B__NM_001178100_CCCGAGCTGACCTTCGGGAA	CD8B__NM_001178100	310	362	446	431
CD8B__NM_001178100_CCTCAAGGGGAAGGTATATC	CD8B__NM_001178100	443	674	486	403
CD8B__NM_001178100_GCCCTCTGGGATTCCGCAAA	CD8B__NM_001178100	450	562	498	331
CD8B__NM_001178100_GTAGTATCCATGCAGGCATT	CD8B__NM_001178100	471	442	640	413
CD8B__NM_001178100_TCAGTAACATGCGCATCTAC	CD8B__NM_001178100	704	603	767	661
CD8B__NM_001178100_TGTAGTATCCATGCAGGCAT	CD8B__NM_001178100	127	184	208	214
CD8B__NM_001178100_TTGCACCTTTATGTATGCAG	CD8B__NM_001178100	118	157	163	143
CD8B__NM_001178100_TTTGCACCTTTATGTATGCA	CD8B__NM_001178100	875	1105	1146	1230
CD8B__NM_004931_ACTGTTTCATGAAACGAAGC	CD8B__NM_004931	115	123	174	206
CD9__NM_001769_AGCCGGCGCCCTCATGATGC	CD9__NM_001769	1897	2229	2072	2037
CD9__NM_001769_ATGGCATCAGGACAGGACTT	CD9__NM_001769	268	229	301	251
CD9__NM_001769_ATTCCACATCATCGGCGCAG	CD9__NM_001769	431	724	482	707
CD9__NM_001769_CAATGGCGAATATCACCAAG	CD9__NM_001769	335	225	376	323
CD9__NM_001769_CATCTGGGGATATTCCCACA	CD9__NM_001769	621	567	620	842
CD9__NM_001769_GAAGATCATGCCAAATATCT	CD9__NM_001769	604	598	660	447
CD9__NM_001769_GCAGGAGTCTATATTCTGAT	CD9__NM_001769	1449	1987	1920	2258
CD9__NM_001769_GCTGAAAGCCATCCACTATG	CD9__NM_001769	293	296	225	230
CD9__NM_001769_GTCTATATTCTGATCGGAGC	CD9__NM_001769	261	198	227	225
CD9__NM_001769_TATCCGCAGGAACCGCGAGA	CD9__NM_001769	409	262	445	244
CD9__NM_001769_TCTTGTGCTGTGCTATCCGC	CD9__NM_001769	489	486	504	417
CD9__NM_001769_TTCCACATCATCGGCGCAGT	CD9__NM_001769	112	62	123	62
CD93__NM_012072_AACCACTGCAACCAGAACGG	CD93__NM_012072	276	463	333	268
CD93__NM_012072_AAGCCCTTCAGCGGCAGACT	CD93__NM_012072	459	586	544	669
CD93__NM_012072_ACAGGGGCCCTCAGACCACT	CD93__NM_012072	168	195	250	278
CD93__NM_012072_AGCCCTCAATGTTACTTCCG	CD93__NM_012072	684	733	867	1096
CD93__NM_012072_AGCCCTTCAGCGGCAGACTA	CD93__NM_012072	235	303	330	338
CD93__NM_012072_AGCGCTCAGCTTGCCCGAGT	CD93__NM_012072	1279	1142	1412	1406
CD93__NM_012072_AGGACACGCCTTACTCTAAC	CD93__NM_012072	426	701	419	565
CD93__NM_012072_CCAAGGAGGAACTGGTGGTC	CD93__NM_012072	534	629	570	675
CD93__NM_012072_CGAGTGGGCCGTGTAGCAGG	CD93__NM_012072	750	975	874	1070
CD93__NM_012072_CTGCGGCCAATGTAGCCTGT	CD93__NM_012072	580	679	638	673
CD93__NM_012072_GCCTTACTCTAACTGGCACA	CD93__NM_012072	421	418	575	504
CD93__NM_012072_TCCTTGTGCCAGTTAGAGTA	CD93__NM_012072	219	297	203	180
CD96__NM_005816_ACGGCTTCTACTGTGCCTAT	CD96__NM_005816	1322	1588	1597	1478
CD96__NM_005816_ACTTACCACCGACCATGCAT	CD96__NM_005816	1603	1892	1541	1540
CD96__NM_005816_AGCAATCAGGTCTATCTTAT	CD96__NM_005816	1205	1746	1306	1808
CD96__NM_005816_CAAAATGGACTCTGCACTTA	CD96__NM_005816	1171	1469	1275	1812
CD96__NM_005816_CCAGGAGGATAATGAACTC	CD96__NM_005816	264	410	268	299
CD96__NM_005816_CCAGTCCAAATCTTCGATGA	CD96__NM_005816	1439	1484	1670	2241
CD96__NM_005816_GCACAGTAGAAGCCGTATTG	CD96__NM_005816	648	570	764	697
CD96__NM_005816_GGCACAGTAGAAGCCGTATT	CD96__NM_005816	3012	3349	3063	3104
CD96__NM_005816_GTTCACCTATGCATGGTCGG	CD96__NM_005816	373	418	501	545
CD96__NM_005816_TACCCTTGACAAAATGTATC	CD96__NM_005816	511	641	620	647
CD96__NM_005816_TGAGTTCACCTATGCATGGT	CD96__NM_005816	55	84	58	39
CD96__NM_005816_TGTTAGGACCGACTCTAATG	CD96__NM_005816	170	172	138	135
CD96__NM_198196_CCTTCTTTCTAAGGGTATAA	CD96__NM_198196	170	115	294	326
CD97__NM_001025160_ACCAATTTGATGACATTCTA	CD97__NM_001025160	1872	1665	2310	2330
CD97__NM_001025160_ACCGTCACAAGTCTCCGTCG	CD97__NM_001025160	4553	5505	5295	6296
CD97__NM_001025160_AGGCCCTGGCGCCACCTGTC	CD97__NM_001025160	1321	1135	1502	1575
CD97__NM_001025160_AGGTGAAGGGGCCTTTAGGC	CD97__NM_001025160	700	780	888	710
CD97__NM_001025160_CCAGAACATGACGACATTGC	CD97__NM_001025160	2464	2287	2568	2648
CD97__NM_001025160_CTAGAATGTCATCAAATTGG	CD97__NM_001025160	967	764	1029	884
CD97__NM_001025160_GGATCCTCATGATATCTTCA	CD97__NM_001025160	954	1218	1207	1224
CD97__NM_001025160_GGGGACAAGAACGTCACTAT	CD97__NM_001025160	1525	1153	1577	1642
CD97__NM_001025160_GGGGGACAAGAACGTCACTA	CD97__NM_001025160	744	824	1063	891
CD97__NM_001025160_GTTCATACCTGAGGATCCGA	CD97__NM_001025160	173	68	205	172
CD97__NM_001025160_TACATTTCCCCTTCGAACAC	CD97__NM_001025160	789	462	797	478
CD97__NM_001025160_TCCTGGAGACGTAGAGGCCC	CD97__NM_001025160	311	172	352	312
CD97__NM_001784_CCAGCAATGTCGTCATGTTC	CD97__NM_001784	2728	3165	3021	3186
CD99L2__NM_001184808_AGGAGAAAGCGAGGCAGACA	CD99L2__NM_001184808	367	392	415	496
CD99L2__NM_001184808_CCTGGAAGCCGTGGTATGTG	CD99L2__NM_001184808	849	1064	977	1038
CD99L2__NM_001184808_CGCAGGAAACCGGGTATAGG	CD99L2__NM_001184808	1031	842	1256	1290
CD99L2__NM_001184808_CTCAACGCAGACTACGTGAA	CD99L2__NM_001184808	458	383	588	717
CD99L2__NM_001184808_GATGATGGCCGCAGGAAACC	CD99L2__NM_001184808	480	458	637	770
CD99L2__NM_001184808_GCCCTGGCCATGGCCCTCAT	CD99L2__NM_001184808	516	407	422	465
CD99L2__NM_001184808_GGTTCCTGGCCTATTGGTTG	CD99L2__NM_001184808	301	320	225	449
CD99L2__NM_001184808_TACGGCAGCAATGACGACCC	CD99L2__NM_001184808	616	453	532	511
CD99L2__NM_001184808_TCTCAACGCAGACTACGTGA	CD99L2__NM_001184808	1756	1054	1794	1288
CD99L2__NM_001184808_TTGGGGTTCCTCACATACCA	CD99L2__NM_001184808	169	104	131	134
CD99L2__NM_001242614_ATGTAACCACCACGACCAAG	CD99L2__NM_001242614	179	196	194	179
CD99L2__NM_001242614_CTCGATCATTTCGATCATCC	CD99L2__NM_001242614	698	622	713	973
CD99L2__NM_001242614_CTTGAAGACATAGTAGGGGG	CD99L2__NM_001242614	450	469	545	660
CD99L2__NM_001242614_GCCCACTGAAGGTAGTGGAT	CD99L2__NM_001242614	972	1692	973	1314
CD99L2__NM_001242614_TCGATCATTTCGATCATCCA	CD99L2__NM_001242614	191	284	232	286
CDA__NM_001785_AAATCATCTTGCATGTCACT	CDA__NM_001785	374	484	490	604
CDA__NM_001785_ACTCAGGCTGGAGTGTAATC	CDA__NM_001785	531	484	529	412
CDA__NM_001785_AGGTGGCTGTTACAGGCAGT	CDA__NM_001785	161	117	220	239
CDA__NM_001785_ATGACAATATACGTACCATC	CDA__NM_001785	428	563	527	411
CDA__NM_001785_CCCAAGGCAGGTTGCTAGGA	CDA__NM_001785	328	418	317	335
CDA__NM_001785_CCCACAGGAAAGTGACTGTA	CDA__NM_001785	697	1363	829	1024
CDA__NM_001785_CGGCGGTGTTCTAAGTCCCA	CDA__NM_001785	298	259	377	372
CDA__NM_001785_GGTCATGTACACGGGCCAGT	CDA__NM_001785	657	473	751	756
CDA__NM_001785_GTATATTGTCATGACGGTCC	CDA__NM_001785	178	198	229	158
CDA__NM_001785_TCAGGGCAATTGCTATCGCC	CDA__NM_001785	536	599	577	753
CDA__NM_001785_TCCGGCTTGGTCATGTACAC	CDA__NM_001785	1143	1097	1185	1397
CDA__NM_001785_TGGTACGTATATTGTCATGA	CDA__NM_001785	44	43	25	26
CDADC1__NM_001193478_AAGATTGAAGTCAAACAGTC	CDADC1__NM_001193478	117	135	112	145
CDADC1__NM_001193478_AATACTGATGACTATAGGTT	CDADC1__NM_001193478	228	439	300	305
CDADC1__NM_001193478_ATCCATACTTTAGCAATCTA	CDADC1__NM_001193478	337	465	371	351
CDADC1__NM_001193478_CATGGTTCAGGCCAGGTTAT	CDADC1__NM_001193478	674	763	708	629
CDADC1__NM_001193478_CTCTTATTAAACATGGGTCA	CDADC1__NM_001193478	8	3	8	17
CDADC1__NM_001193478_CTGTGGCCAAAAGTAGGATA	CDADC1__NM_001193478	94	162	160	153
CDADC1__NM_001193478_GTAAATCTTCACTAGAACAG	CDADC1__NM_001193478	121	135	113	89
CDADC1__NM_001193478_GTGCCGAACTTTAAACACTT	CDADC1__NM_001193478	231	509	294	400
CDADC1__NM_001193478_TAAAAGTCAGTGTTAGCATC	CDADC1__NM_001193478	266	144	281	275
CDADC1__NM_001193478_TCCAGAAATAAGTTTGCTTA	CDADC1__NM_001193478	399	476	550	758
CDADC1__NM_001193478_TCTAGTGAAGATTTACATGC	CDADC1__NM_001193478	195	222	211	160
CDADC1__NM_001193478_TGCACCATATAACACACCAA	CDADC1__NM_001193478	328	223	292	306
CDAN1__NM_138477_AACTGGGGACACAGCTGATT	CDAN1__NM_138477	280	131	368	170
CDAN1__NM_138477_ACGGTACGAATTCTTTCCGC	CDAN1__NM_138477	2481	574	2799	1235
CDAN1__NM_138477_ATGCTCAGGAAGGAGCGGTA	CDAN1__NM_138477	249	41	223	121
CDAN1__NM_138477_CCGGGGGGAACCGAGCCTAC	CDAN1__NM_138477	376	267	452	427
CDAN1__NM_138477_CGCGCGCAGCACCCAGGGTT	CDAN1__NM_138477	1549	1149	1759	1300
CDAN1__NM_138477_GCCCCAAGGGCTAGTGTCCA	CDAN1__NM_138477	383	297	412	386
CDAN1__NM_138477_GCCCTGGACACTAGCCCTTG	CDAN1__NM_138477	1958	1220	1948	1308
CDAN1__NM_138477_GCCGGGGGGAACCGAGCCTA	CDAN1__NM_138477	358	280	363	325
CDAN1__NM_138477_GCCTCCCCAGCGTTATCCTA	CDAN1__NM_138477	1385	245	1457	373
CDAN1__NM_138477_GCTCTTCGCTCACCGGAGTT	CDAN1__NM_138477	738	320	797	841
CDAN1__NM_138477_GGGGAACCGAGCCTACGGGA	CDAN1__NM_138477	273	71	330	252
CDAN1__NM_138477_TGTCGGTCGCAGCCGTCGTG	CDAN1__NM_138477	698	84	754	280
CDC123__NM_006023_ACCTCTTGATGGTAACGCCT	CDC123__NM_006023	714	145	851	97
CDC123__NM_006023_CTTACCTCATATTCTATACA	CDC123__NM_006023	372	91	477	130
CDC123__NM_006023_GAAGCTATCAATTCCCTCGG	CDC123__NM_006023	532	25	528	7
CDC123__NM_006023_GCTTAATTGGAGTGCCCCAA	CDC123__NM_006023	341	144	298	82
CDC123__NM_006023_GGAAGCTATCAATTCCCTCG	CDC123__NM_006023	566	150	623	108
CDC123__NM_006023_GGGGCACTCCAATTAAGCTT	CDC123__NM_006023	236	0	158	3
CDC123__NM_006023_GTCAAGGAAAACAAGCTTAT	CDC123__NM_006023	220	217	200	260
CDC123__NM_006023_GTGTCTTTCCTAAGCTTAAT	CDC123__NM_006023	75	69	103	68
CDC123__NM_006023_TCGGAAGAACGGGTACCACG	CDC123__NM_006023	499	0	363	6
CDC123__NM_006023_TGAGAACACAGCCACGCTTA	CDC123__NM_006023	257	296	380	371
CDC123__NM_006023_TGAGTTTGCCACTAAAGTCC	CDC123__NM_006023	184	84	182	5
CDC123__NM_006023_TTCACTGCCAGTTCTCCGCG	CDC123__NM_006023	148	0	184	5
CDC14A__NM_003672_AAAGCAAAATTGAGAATGGT	CDC14A__NM_003672	313	264	370	401
CDC14A__NM_003672_AATAAGGAAAGTAGGCTTCA	CDC14A__NM_003672	407	340	465	436
CDC14A__NM_003672_ACATCTGTGAGAACACCGAA	CDC14A__NM_003672	471	324	465	466
CDC14A__NM_003672_ACCATTCTCGACTGTTTGCA	CDC14A__NM_003672	2022	2065	2093	2402
CDC14A__NM_003672_ACCCCCCCTATCTTCCATTC	CDC14A__NM_003672	1419	1465	1619	1574
CDC14A__NM_003672_ATAAGGAAAGTAGGCTTCAG	CDC14A__NM_003672	215	113	390	209
CDC14A__NM_003672_CACCATTCTCGACTGTTTGC	CDC14A__NM_003672	1987	2573	2190	2645
CDC14A__NM_003672_CGGAGTCAGGGGAACTAATC	CDC14A__NM_003672	251	153	283	421
CDC14A__NM_003672_CTTCGCACGATGTTGTCACT	CDC14A__NM_003672	856	630	744	715
CDC14A__NM_003672_GGAGTCAGGGGAACTAATCG	CDC14A__NM_003672	680	741	716	788
CDC14A__NM_003672_TACCTGAATGGAAGATAGGG	CDC14A__NM_003672	251	206	309	157
CDC14A__NM_003672_TCCCTGCAAACAGTCGAGAA	CDC14A__NM_003672	814	924	796	830
CDC14B__NM_001077181_CAGAAGGCAATAAATCGGTC	CDC14B__NM_001077181	455	348	672	452
CDC14B__NM_001077181_CCTAGCTGGCCTTGGTCGCA	CDC14B__NM_001077181	1064	1039	1133	1470
CDC14B__NM_001077181_CCTGACCCACGCAATGGTCT	CDC14B__NM_001077181	1549	1673	1567	1607
CDC14B__NM_001077181_GCCAGCATCCGTAAAGCGTT	CDC14B__NM_001077181	860	1012	1075	1030
CDC14B__NM_001077181_GCCATTGCAGTACATTGCAA	CDC14B__NM_001077181	962	1255	1086	908
CDC14B__NM_001077181_GTCCACAGAAGGCAATAAAT	CDC14B__NM_001077181	254	177	302	272
CDC14B__NM_001077181_TCAAGAGCCAGACTTGAAAG	CDC14B__NM_001077181	397	531	476	479
CDC14B__NM_001077181_TCAAGTCTGGCTCTTGAATG	CDC14B__NM_001077181	220	434	270	271
CDC14B__NM_001077181_TCAGAGTGCCCGTGCGACCA	CDC14B__NM_001077181	1113	971	1095	699
CDC14B__NM_001077181_TCTTTGACAATGGCATCAGT	CDC14B__NM_001077181	277	111	277	210
CDC14B__NM_001077181_TGCAGTCCATTACAATGTTA	CDC14B__NM_001077181	551	476	702	819
CDC14B__NM_001077181_TTTGACAATGGCATCAGTAG	CDC14B__NM_001077181	394	269	448	291
CDC14B__NM_003671_CGCTGCTCGTCGACCTCGCC	CDC14B__NM_003671	1297	1456	1340	2075
CDC14B__NM_003671_GATCTTCTTCACACCCGGCG	CDC14B__NM_003671	398	404	524	578
CDC14B__NM_003671_GCGCTGCTCGTCGACCTCGC	CDC14B__NM_003671	1644	1861	1969	2191
CDC14B__NM_003671_TGTCCAGGTACACGTCGTCC	CDC14B__NM_003671	1537	1479	1536	1393
CDC16__NM_001078645_AAAGTAGCTTCACTCTCTCG	CDC16__NM_001078645	779	22	991	5
CDC16__NM_001078645_AAGAACCCCAGGACATCTAT	CDC16__NM_001078645	371	1	539	0
CDC16__NM_001078645_ACCCCAGGACATCTATTGGT	CDC16__NM_001078645	874	1	1446	23
CDC16__NM_001078645_ACGACATGCTTCATACAACT	CDC16__NM_001078645	902	348	1160	505
CDC16__NM_001078645_CAGCCGCTCTAGGTTCATGG	CDC16__NM_001078645	501	41	797	108
CDC16__NM_001078645_CCGCAGCCGCTCTAGGTTCA	CDC16__NM_001078645	470	0	787	47
CDC16__NM_001078645_CGTCCGGCAGTACCTCGACC	CDC16__NM_001078645	982	13	1395	11
CDC16__NM_001078645_GAGCCAACCAATAGATGTCC	CDC16__NM_001078645	470	8	576	57
CDC16__NM_001078645_GGCCCTTGATGTTCTTGACA	CDC16__NM_001078645	607	84	794	408
CDC16__NM_001078645_TCAAAGATCCTTCCAGCGAC	CDC16__NM_001078645	201	326	341	410
CDC16__NM_001078645_TGCACTTCGGTCACGAAAAC	CDC16__NM_001078645	130	214	144	155
CDC16__NM_001078645_TTACATGGCACCTAGCTGCA	CDC16__NM_001078645	195	0	194	0
CDC20__NM_001255_ACCGCCAGGTTTGCTAGGAG	CDC20__NM_001255	1355	779	1477	855
CDC20__NM_001255_ATCTGGGCAGCACTGCGATG	CDC20__NM_001255	32	0	21	6
CDC20__NM_001255_CACTGCGATGGGGGATATAG	CDC20__NM_001255	60	0	105	5
CDC20__NM_001255_CAGACCACTCCTAGCAAACC	CDC20__NM_001255	703	413	672	291
CDC20__NM_001255_CAGCGCGCAGGGGGTGCATT	CDC20__NM_001255	1059	76	1107	238
CDC20__NM_001255_CATCTGGGCAGCACTGCGAT	CDC20__NM_001255	23	0	55	0
CDC20__NM_001255_CCTTGGAACTGGATTTGCCT	CDC20__NM_001255	963	445	1006	445
CDC20__NM_001255_GCAGGGGGTGCATTGGGGAT	CDC20__NM_001255	860	97	1066	276
CDC20__NM_001255_GCTAGGAGTGGTCTGAACCT	CDC20__NM_001255	1128	490	1050	496
CDC20__NM_001255_GGCGCTGTGGGATCGGTTGG	CDC20__NM_001255	1455	922	1562	912
CDC20__NM_001255_TGGCGGCCCGCATGGGTGAG	CDC20__NM_001255	84	24	68	25
CDC20__NM_001255_TTGGCGGCCCGCATGGGTGA	CDC20__NM_001255	243	52	233	114
CDC20B__NM_001145734_AAGAAAGTGTTGCCTCAAAA	CDC20B__NM_001145734	290	225	344	222
CDC20B__NM_001145734_ACTCTTTGTAACCCAGAACG	CDC20B__NM_001145734	252	198	262	198
CDC20B__NM_001145734_CGAGTAGCCCCATTACCACA	CDC20B__NM_001145734	1085	1310	1285	1324
CDC20B__NM_001145734_GCTGCCACCTTGTGGTAATG	CDC20B__NM_001145734	369	686	600	804
CDC20B__NM_001145734_GTAGCCCCATTACCACAAGG	CDC20B__NM_001145734	75	29	121	130
CDC20B__NM_001145734_GTGAGCAATCCGAATGTGTT	CDC20B__NM_001145734	1190	1757	1496	1661
CDC20B__NM_001145734_GTGGCAGCAAAGTCAAACTA	CDC20B__NM_001145734	33	76	33	30
CDC20B__NM_001145734_TAGCCTGCTGAACCACGTTC	CDC20B__NM_001145734	656	906	719	665
CDC20B__NM_001145734_TGTTTCAGAAATTCCTTTGC	CDC20B__NM_001145734	11	30	35	37
CDC20B__NM_001145734_TTAAGAGCAACTTTGCGAAG	CDC20B__NM_001145734	74	15	55	33
CDC20B__NM_001145734_TTGCAAGGCACCTCATGCAA	CDC20B__NM_001145734	220	304	221	212
CDC20B__NM_001145734_TTGCTGCCACCTTGTGGTAA	CDC20B__NM_001145734	208	289	194	245
CDC23__NM_004661_AAAACTGGACTTGGTTAAAG	CDC23__NM_004661	301	177	356	174
CDC23__NM_004661_AAGATGAAGTGTCATATGTT	CDC23__NM_004661	325	430	395	257
CDC23__NM_004661_ACCGCCTCCGCCTATTACAG	CDC23__NM_004661	1210	72	1707	86
CDC23__NM_004661_ATCCATCAAGTTCTCGAGCT	CDC23__NM_004661	686	887	716	983
CDC23__NM_004661_ATCTGAGAAATCGCTGTTTA	CDC23__NM_004661	327	279	365	442
CDC23__NM_004661_CAAACATCATTCTGGACCAT	CDC23__NM_004661	1514	1504	2002	2572
CDC23__NM_004661_CAGATGGCAACAGCTCCATA	CDC23__NM_004661	495	406	431	424
CDC23__NM_004661_CCAACTGACAGAATCCTGGC	CDC23__NM_004661	854	885	1030	991
CDC23__NM_004661_CCTTTACCTCATCTAGATAA	CDC23__NM_004661	388	440	459	381
CDC23__NM_004661_CGAAGCACCACACCATACCT	CDC23__NM_004661	2700	25	3695	95
CDC23__NM_004661_CTTACCTCTGTAATAGGCGG	CDC23__NM_004661	153	153	265	241
CDC23__NM_004661_GAGACAGAAGTACTTCTAAC	CDC23__NM_004661	132	195	179	175
CDC23__NM_004661_GCAAGCTGTATACTAGTTAC	CDC23__NM_004661	400	373	446	553
CDC23__NM_004661_GCTCGGCCAGAGGCAGTGCA	CDC23__NM_004661	464	0	789	1
CDC23__NM_004661_GCTTGTATAATTACCTTCAG	CDC23__NM_004661	354	334	462	291
CDC23__NM_004661_GGATATGGATGCCTATACCC	CDC23__NM_004661	108	0	138	1
CDC23__NM_004661_GTTGCCTATCACAATATCAG	CDC23__NM_004661	595	67	679	81
CDC23__NM_004661_TATTTGCACAGTAGATGCTA	CDC23__NM_004661	317	262	398	456
CDC23__NM_004661_TCTCCAACCTTCTTTATGTC	CDC23__NM_004661	539	519	606	637
CDC23__NM_004661_TGCCCTTGCATTGGGGAGCC	CDC23__NM_004661	250	388	201	380
CDC23__NM_004661_TGTATTCCAGATATCTGGTG	CDC23__NM_004661	271	0	402	2
CDC23__NM_004661_TTTCAGCCTTTATCTAGATG	CDC23__NM_004661	113	151	94	150
CDC25A__NM_001789_ACAGTGGGATGAACCAGCCG	CDC25A__NM_001789	465	264	585	355
CDC25A__NM_001789_ACCGATATCTATTTGGTGGG	CDC25A__NM_001789	703	446	720	406
CDC25A__NM_001789_ACCTCCCACCAAATAGATAT	CDC25A__NM_001789	638	395	741	528
CDC25A__NM_001789_ACTGGCTACCGATATCTATT	CDC25A__NM_001789	1603	817	1575	1132
CDC25A__NM_001789_ATCTGTCTCAATGCGCGTGT	CDC25A__NM_001789	627	249	670	307
CDC25A__NM_001789_ATGGACGGAGGACGTCTAAC	CDC25A__NM_001789	94	90	161	60
CDC25A__NM_001789_CACCAACTCGACCGTCACTA	CDC25A__NM_001789	2012	1322	2153	1473
CDC25A__NM_001789_CACCTGGAAGTACAAAGAGG	CDC25A__NM_001789	257	138	301	171
CDC25A__NM_001789_CCAAATAGCGCCTTCACGAC	CDC25A__NM_001789	1081	979	1293	966
CDC25A__NM_001789_CCACGAGATACAGGTCTTAC	CDC25A__NM_001789	1953	1895	2496	2167
CDC25A__NM_001789_CCAGAGGCTTGCCATGCACG	CDC25A__NM_001789	1034	1052	1198	849
CDC25A__NM_001789_CCCCAAGAAGTTGAGGTAAG	CDC25A__NM_001789	387	367	469	385
CDC25A__NM_001789_CCCGTCGTGAAGGCGCTATT	CDC25A__NM_001789	1734	1906	2021	1867
CDC25A__NM_001789_GCAGAGAAACTTAAGCAAAG	CDC25A__NM_001789	357	393	485	485
CDC25A__NM_001789_GCCAAATAGCGCCTTCACGA	CDC25A__NM_001789	1070	747	1078	808
CDC25A__NM_001789_GGCTACCGATATCTATTTGG	CDC25A__NM_001789	1713	1152	1767	1582
CDC25A__NM_001789_GGCTCCAACCTTGGGAGTGT	CDC25A__NM_001789	651	546	759	580
CDC25A__NM_001789_GTGCTGGAGCTACACAGGGA	CDC25A__NM_001789	433	238	558	268
CDC25A__NM_001789_TCCTCCGAGTCAACAGATTC	CDC25A__NM_001789	323	289	326	346
CDC25A__NM_001789_TCTGTCCGATAACTTATAAC	CDC25A__NM_001789	1143	665	1210	868
CDC25A__NM_001789_TCTTTACTGTCCAATGGCCC	CDC25A__NM_001789	305	220	363	387
CDC25A__NM_001789_TGAACCAGCCGGGGCCTTAT	CDC25A__NM_001789	695	719	729	604
CDC25A__NM_001789_TGCAGGCAGCCACGAGATAC	CDC25A__NM_001789	480	252	653	395
CDC25A__NM_001789_TGCCAGCCTCCTTACCATCA	CDC25A__NM_001789	1092	847	1050	918
CDC25A__NM_001789_TGGGAGATTTAGCTTATGTC	CDC25A__NM_001789	98	30	99	58
CDC25A__NM_001789_TTGTAAGAAAGAGATTCTGT	CDC25A__NM_001789	124	29	136	85
CDC25A__NM_001789_TTTGTAGTTCTCATGACGAG	CDC25A__NM_001789	171	188	246	236
CDC25A__NM_201567_AAGAGGAGGAAGAGCATGTC	CDC25A__NM_201567	314	252	330	354
CDC25A__NM_201567_AGAGGAGGAAGAGCATGTCT	CDC25A__NM_201567	399	84	414	137
CDC25B__NM_001287516_ACAGGGATAGGTGCGTCAGG	CDC25B__NM_001287516	381	575	369	377
CDC25B__NM_001287516_ATTCGGATGCCCGTCGAGAC	CDC25B__NM_001287516	398	642	609	769
CDC25B__NM_001287516_CAGACGCTTCCAGTCTATGC	CDC25B__NM_001287516	446	325	525	462
CDC25B__NM_001287516_CAGCTCGGCCCCCGACCTGA	CDC25B__NM_001287516	864	693	891	803
CDC25B__NM_001287516_CATGTGGGGGTCCATAGGGC	CDC25B__NM_001287516	804	932	883	687
CDC25B__NM_001287516_CGCACCTATCCCTGTCTCGA	CDC25B__NM_001287516	3838	3996	4202	4286
CDC25B__NM_001287516_CTGACTCTTTGGGGTTTCGC	CDC25B__NM_001287516	297	443	373	456
CDC25B__NM_001287516_GAGACAGGGATAGGTGCGTC	CDC25B__NM_001287516	580	668	645	808
CDC25B__NM_001287516_TGGATGGCCTGTTCAAACCT	CDC25B__NM_001287516	128	202	138	144
CDC25B__NM_001287516_TGGTGATGTTCCGAAGCACG	CDC25B__NM_001287516	53	54	55	39
CDC25B__NM_001287516_TTCGGATGCCCGTCGAGACA	CDC25B__NM_001287516	238	218	279	221
CDC25B__NM_001287516_TTGGTGATGTTCCGAAGCAC	CDC25B__NM_001287516	233	284	244	187
CDC25B__NM_001287517_AGAGAAGCGACCTAGGGCCA	CDC25B__NM_001287517	1289	1417	1444	1670
CDC25B__NM_001287519_CGCCCGTGCAGAATAAGCGG	CDC25B__NM_001287519	708	767	738	533
CDC25B__NM_001287519_GGCACTTGCTGTACATGACG	CDC25B__NM_001287519	1599	1870	1929	2647
CDC25B__NM_001287519_GGGTCAGAGAGAAGCGACCT	CDC25B__NM_001287519	455	366	436	654
CDC25B__NM_001287519_GGTCAGAGAGAAGCGACCTA	CDC25B__NM_001287519	230	167	253	293
CDC25B__NM_001287519_TGCCCCACTGGTCAAGACCT	CDC25B__NM_001287519	540	478	730	507
CDC25B__NM_001287522_CCGCCTCCGCTTATTCTGCA	CDC25B__NM_001287522	2727	3335	2764	3370
CDC25B__NM_001287522_CGCCTCCGCTTATTCTGCAC	CDC25B__NM_001287522	4816	6227	5567	7225
CDC25B__NM_001287524_CCGTGCAGAATAAGCGGAGG	CDC25B__NM_001287524	1331	1472	1401	1839
CDC25B__NM_001287524_GCTGATTGGAGATTACTCTA	CDC25B__NM_001287524	660	412	697	863
CDC25B__NM_001287524_GCTTTCCGTCTACTGTCTGT	CDC25B__NM_001287524	1438	1258	1697	1549
CDC25C__NM_001287582_ACTGGAGTTCTAGGGACATC	CDC25C__NM_001287582	815	521	938	960
CDC25C__NM_001287582_AGATGTCCCTAGAACTCCAG	CDC25C__NM_001287582	338	270	352	319
CDC25C__NM_001287582_CTTCAGGAAGTGCATTTAGC	CDC25C__NM_001287582	354	365	326	378
CDC25C__NM_001287582_CTTCTTTGTAGCACTCCGAA	CDC25C__NM_001287582	242	449	248	409
CDC25C__NM_001287582_CTTTGATTAGACCTAAAACT	CDC25C__NM_001287582	103	171	151	97
CDC25C__NM_001287582_GACATCTGGACAGACGGTAA	CDC25C__NM_001287582	642	472	781	560
CDC25C__NM_001287582_GGCTACATTTCATTAGGTGC	CDC25C__NM_001287582	1426	2061	1774	2031
CDC25C__NM_001287582_TCACCAAGAAATTTGCCCAC	CDC25C__NM_001287582	1076	959	1199	1257
CDC25C__NM_001287582_TCACTGGGCTACATTTCATT	CDC25C__NM_001287582	1023	1014	1142	1011
CDC25C__NM_001287582_TGAGAAGAGTTCCGTAGACA	CDC25C__NM_001287582	404	327	496	342
CDC25C__NM_001287582_TGCTAAGATTCGAAAGATCG	CDC25C__NM_001287582	121	226	135	162
CDC25C__NM_001287582_TTCAGGAAGTGCATTTAGCT	CDC25C__NM_001287582	52	65	33	90
CDC25C__NM_001287583_CTCCAAAATATCGCAGAAAG	CDC25C__NM_001287583	120	114	156	147
CDC25C__NM_001287583_GCGCTTTGATTTAGGGATGG	CDC25C__NM_001287583	980	1374	1038	1067
CDC25C__NM_001287583_GTCTCTCGGACAGCGCTGTT	CDC25C__NM_001287583	364	426	357	433
CDC25C__NM_001287583_TTTCAAGATGTGCCTCCAAC	CDC25C__NM_001287583	112	166	107	203
CDC25C__NM_022809_CTGGAGGAAGATTCTAACCA	CDC25C__NM_022809	448	490	468	366
CDC25C__NM_022809_GCATCGACGGGGAGCGATAT	CDC25C__NM_022809	647	579	743	806
CDC25C__NM_022809_GCTGGAGGAAGATTCTAACC	CDC25C__NM_022809	827	802	782	758
CDC25C__NM_022809_TCAAGTTCTCTGGCATCGAC	CDC25C__NM_022809	298	418	332	522
CDC26__NM_139286_AGAGTTTGAGAACATTCGAA	CDC26__NM_139286	185	298	216	228
CDC26__NM_139286_AGATGAACGATTATTGGGCT	CDC26__NM_139286	403	541	604	736
CDC26__NM_139286_CAAATGATCAATGATCGGAT	CDC26__NM_139286	560	557	771	763
CDC26__NM_139286_GGGAACAAATGATCAATGAT	CDC26__NM_139286	278	115	302	192
CDC26__NM_139286_GGGATCACTGCTAAGCCCAA	CDC26__NM_139286	1105	562	1369	597
CDC26__NM_139286_TGAGAACATTCGAAAGGACC	CDC26__NM_139286	140	30	187	64
CDC27__NM_001114091_AAGACAGATCGGCTTGCCAA	CDC27__NM_001114091	149	0	183	1
CDC27__NM_001114091_ACAGCCTGAGACAGTTCTTA	CDC27__NM_001114091	355	236	467	414
CDC27__NM_001114091_AGAGGAAAGGATTTAAACTA	CDC27__NM_001114091	16	0	31	0
CDC27__NM_001114091_ATTCAAGGAGTACTTTGAAT	CDC27__NM_001114091	20	0	17	3
CDC27__NM_001114091_CCACTATGCTTACCGAGATG	CDC27__NM_001114091	438	9	559	42
CDC27__NM_001114091_CCGCATCTCGGTAAGCATAG	CDC27__NM_001114091	21	0	9	0
CDC27__NM_001114091_CTGCGAGGAAAACCGCATCT	CDC27__NM_001114091	87	0	172	1
CDC27__NM_001114091_CTGTGAGATAAACTATGATT	CDC27__NM_001114091	275	170	288	131
CDC27__NM_001114091_GTTCCCAGTGGGACAGTATC	CDC27__NM_001114091	13	0	12	0
CDC27__NM_001114091_TACTTGTGTTGTGCAAGAGT	CDC27__NM_001114091	359	40	553	107
CDC27__NM_001114091_TGAATCAATATAAGACACTG	CDC27__NM_001114091	110	73	139	84
CDC27__NM_001114091_TTCACCTGATACTGTCCCAC	CDC27__NM_001114091	19	0	11	0
CDC27__NM_001256_AACAAATGTAATGGGCATAT	CDC27__NM_001256	440	278	463	555
CDC27__NM_001256_AAGAATAAGAGTACACTGTA	CDC27__NM_001256	187	222	211	221
CDC27__NM_001256_ACCTAAATGACTTTGAAAGT	CDC27__NM_001256	1211	1095	1179	833
CDC27__NM_001256_AGCAGGAATTTATGTGCCAG	CDC27__NM_001256	374	473	397	440
CDC27__NM_001256_AGTAAAGACTCAGTATACAG	CDC27__NM_001256	206	127	287	223
CDC27__NM_001256_AGTAATTGGGTTTAGGATGT	CDC27__NM_001256	470	312	390	482
CDC27__NM_001256_AGTCAGGGTCTTTAGGTATC	CDC27__NM_001256	396	565	494	447
CDC27__NM_001256_ATGACAATTTATGGGGGGTA	CDC27__NM_001256	300	323	385	437
CDC27__NM_001256_ATTCATATGGTGAACTTAAG	CDC27__NM_001256	200	282	161	117
CDC27__NM_001256_CTCCCATAAACAAATGTAAT	CDC27__NM_001256	256	249	286	275
CDC27__NM_001256_CTTGAAGGAACCATTAGATA	CDC27__NM_001256	1674	1614	1927	1918
CDC27__NM_001256_CTTGTTCAGAAGTTATGTTG	CDC27__NM_001256	406	349	499	493
CDC27__NM_001256_CTTTCATTCTGTATACAGTC	CDC27__NM_001256	229	354	254	414
CDC27__NM_001256_GATTCATATGGTGAACTTAA	CDC27__NM_001256	108	93	83	36
CDC27__NM_001256_GCATATTGGGACTCGTATGT	CDC27__NM_001256	1223	996	1284	1150
CDC27__NM_001256_GCCAGAAAGTGTTACTACGT	CDC27__NM_001256	1256	1127	1178	854
CDC27__NM_001256_GGCAATCAAGTCACTGTTAT	CDC27__NM_001256	1091	1142	1422	1808
CDC27__NM_001256_GGGAAAAAACATTCTTATGA	CDC27__NM_001256	415	467	510	655
CDC27__NM_001256_GGTCAGTTACTCTGTTTATC	CDC27__NM_001256	265	585	314	370
CDC27__NM_001256_GGTTGGTGCATAAGTAATTG	CDC27__NM_001256	257	217	314	185
CDC27__NM_001256_GTAAAGACTCAGTATACAGA	CDC27__NM_001256	175	107	189	76
CDC27__NM_001256_GTATGAATGACAATTTATGG	CDC27__NM_001256	268	132	204	83
CDC27__NM_001256_GTGTTACTACGTGGGAAATA	CDC27__NM_001256	100	77	77	77
CDC27__NM_001256_TAACAGTGACTTGATTGCCA	CDC27__NM_001256	467	292	387	294
CDC27__NM_001256_TACTAAGAGTAATTGGGTTT	CDC27__NM_001256	679	659	752	795
CDC27__NM_001256_TATGAATGACAATTTATGGG	CDC27__NM_001256	315	260	253	176
CDC27__NM_001256_TCAGAAATTGTACAGTGTTG	CDC27__NM_001256	975	1328	1035	1213
CDC27__NM_001256_TCATGATACTTTCCAACAAA	CDC27__NM_001256	85	89	97	101
CDC27__NM_001256_TGAACTTAAGGGGTTATTAA	CDC27__NM_001256	16	5	12	9
CDC27__NM_001256_TTTCATTCTGTATACAGTCA	CDC27__NM_001256	52	108	77	98
CDC34__NM_004359_CCCCCCAACACCTACTACGA	CDC34__NM_004359	1354	851	1354	975
CDC34__NM_004359_CCGCCCTCGTAGTAGGTGTT	CDC34__NM_004359	1912	1881	1987	2506
CDC34__NM_004359_CCTCGTCCACCAGTGTCACG	CDC34__NM_004359	738	461	1006	794
CDC34__NM_004359_CGAGGGATTCCGCGTGACAC	CDC34__NM_004359	433	381	457	365
CDC34__NM_004359_GAGAGTATGGGTAGTCGATG	CDC34__NM_004359	337	218	432	291
CDC34__NM_004359_GCCCCCCAACACCTACTACG	CDC34__NM_004359	441	441	556	532
CDC34__NM_004359_GGAGAGTATGGGTAGTCGAT	CDC34__NM_004359	862	978	970	957
CDC34__NM_004359_GGGCGATCTATACAACTGGG	CDC34__NM_004359	347	393	371	376
CDC34__NM_004359_GTGGCACCCTAACATCTACG	CDC34__NM_004359	196	268	215	168
CDC34__NM_004359_TGGAGAGTATGGGTAGTCGA	CDC34__NM_004359	56	63	90	113
CDC34__NM_004359_TTCTGCGAGCTGGGCACTAG	CDC34__NM_004359	27	16	23	26
CDC34__NM_004359_TTGGTCAGGAACCGAAAGGC	CDC34__NM_004359	62	30	78	90
CDC37__NM_007065_AACTACCTGGTAGACCAGCC	CDC37__NM_007065	228	405	270	353
CDC37__NM_007065_AACTGCCGGAAGCAGGCCCG	CDC37__NM_007065	762	146	727	129
CDC37__NM_007065_AAGGGGCCGGCTCTCGTCAC	CDC37__NM_007065	963	888	1061	695
CDC37__NM_007065_AATCGTCATGCAATTATCCC	CDC37__NM_007065	254	58	254	142
CDC37__NM_007065_AGACTGGCCGTGTCGATGTT	CDC37__NM_007065	1277	584	1397	952
CDC37__NM_007065_AGCAGGTGGCGGTGGTAGCT	CDC37__NM_007065	129	148	123	193
CDC37__NM_007065_ATTGCATGACGATTGTCTGG	CDC37__NM_007065	774	104	978	176
CDC37__NM_007065_CACAGGCATGCTTCGCCGCT	CDC37__NM_007065	703	657	738	845
CDC37__NM_007065_CCAGGTGGACGTTGTCTGAC	CDC37__NM_007065	304	14	220	2
CDC37__NM_007065_CGAGGAGACAGCCAATTACC	CDC37__NM_007065	471	3	375	58
CDC37__NM_007065_GAGACTGGCCGTGTCGATGT	CDC37__NM_007065	2008	211	2160	333
CDC37__NM_007065_GCTGGCCAAGAGCCTAAAGG	CDC37__NM_007065	226	33	245	82
CDC37__NM_007065_GGCTGGTCTACCAGGTAGTT	CDC37__NM_007065	707	681	939	756
CDC37__NM_007065_GGGCCGAGCAGCGCAAGTAG	CDC37__NM_007065	430	234	432	292
CDC37__NM_007065_TAAATTGCATGACGATTGTC	CDC37__NM_007065	158	103	234	164
CDC37L1__NM_017913_ACATGATTCTGAACTGTCAT	CDC37L1__NM_017913	323	259	365	448
CDC37L1__NM_017913_ATAGATCCAACACCAGAATG	CDC37L1__NM_017913	23	40	22	16
CDC37L1__NM_017913_CAATTCCATGGCTATATATC	CDC37L1__NM_017913	472	472	605	600
CDC37L1__NM_017913_CATTCTGGTGTTGGATCTAT	CDC37L1__NM_017913	1792	1651	1902	1999
CDC37L1__NM_017913_CTTCACATACAAGGTATGGA	CDC37L1__NM_017913	50	32	41	99
CDC37L1__NM_017913_GGCAAGCCAATTCAATTCCA	CDC37L1__NM_017913	830	922	1078	1433
CDC37L1__NM_017913_GTATAGCCATGGAATTGAAT	CDC37L1__NM_017913	345	167	406	488
CDC37L1__NM_017913_TAATTCTGAGTCCTTGGATC	CDC37L1__NM_017913	125	231	139	164
CDC37L1__NM_017913_TACAGGTATGTTGAGTCGAT	CDC37L1__NM_017913	514	615	563	637
CDC37L1__NM_017913_TATAGATCCAACACCAGAAT	CDC37L1__NM_017913	113	101	194	97
CDC37L1__NM_017913_TTACAGGTATGTTGAGTCGA	CDC37L1__NM_017913	152	121	113	192
CDC37L1__NM_017913_TTTATTAGAATCCTTACCAC	CDC37L1__NM_017913	119	117	184	220
CDC40__NM_015891_AAATCAAGATAGTCCGGACG	CDC40__NM_015891	450	524	555	468
CDC40__NM_015891_AAGGCGTGGGACATACGACC	CDC40__NM_015891	231	324	355	313
CDC40__NM_015891_AATAATAGGGAGAGTAACTG	CDC40__NM_015891	266	184	223	172
CDC40__NM_015891_ACAGACACTCCGTAAGGGCA	CDC40__NM_015891	348	233	424	376
CDC40__NM_015891_AGATTAATCCTTAAACTAGC	CDC40__NM_015891	48	97	52	63
CDC40__NM_015891_AGCCATTTGCTGTGTCCTAA	CDC40__NM_015891	633	996	775	1158
CDC40__NM_015891_ATACTGAAAGCATCTCTTTG	CDC40__NM_015891	299	239	270	243
CDC40__NM_015891_ATTTATGTGGCTATAGGGCC	CDC40__NM_015891	79	141	155	223
CDC40__NM_015891_ATTTCATGTTTGAGCAGCAA	CDC40__NM_015891	136	131	131	177
CDC40__NM_015891_CAAGTGTCTGCTAAATATAT	CDC40__NM_015891	379	251	347	291
CDC40__NM_015891_CATAGGAGATAAAGTACGAG	CDC40__NM_015891	339	241	406	299
CDC40__NM_015891_CTAAAAACAGACACTCCGTA	CDC40__NM_015891	464	799	739	842
CDC40__NM_015891_CTAGAATCCCAAATCCACTA	CDC40__NM_015891	431	376	433	383
CDC40__NM_015891_CTCATAGGAGATAAAGTACG	CDC40__NM_015891	317	324	344	256
CDC40__NM_015891_GATTAATCCTTAAACTAGCT	CDC40__NM_015891	70	119	81	113
CDC40__NM_015891_GCAGCTCTGGCCGCTTCCTA	CDC40__NM_015891	531	311	698	331
CDC40__NM_015891_GCCATTTGCTGTGTCCTAAA	CDC40__NM_015891	678	522	659	469
CDC40__NM_015891_GGCTCCGGAGGTGGCAGTTA	CDC40__NM_015891	316	19	350	101
CDC40__NM_015891_GGTTTAACTGTATTTGAAAC	CDC40__NM_015891	141	191	218	232
CDC40__NM_015891_TAAAAACAGACACTCCGTAA	CDC40__NM_015891	446	361	467	439
CDC40__NM_015891_TCTGGCCGCTTCCTATGGTT	CDC40__NM_015891	2310	1572	2561	1596
CDC40__NM_015891_TGAACCCGAACCATAGGAAG	CDC40__NM_015891	155	25	123	90
CDC40__NM_015891_TGCAATCGCAGCCGATGA	CDC40__NM_015891	379	188	343	196
CDC40__NM_015891_TTCAGGGTCCGAATCGGACT	CDC40__NM_015891	51	81	61	58
CDC40__NM_015891_TTCGGGTTCAGGGTCCGAAT	CDC40__NM_015891	76	58	76	52
CDC42__NM_001039802_ACAGTCGGTACATATTCCGA	CDC42__NM_001039802	740	151	893	326
CDC42__NM_001039802_AGAAAGGAGTCTTTGGACAG	CDC42__NM_001039802	112	8	61	23
CDC42__NM_001039802_CACAACAAACAAATTTCCAT	CDC42__NM_001039802	710	598	951	932
CDC42__NM_001039802_CTGCGGCTCTTCTTCGGTTC	CDC42__NM_001039802	1212	823	1322	1231
CDC42__NM_001039802_GCAAGTTTCTCAATAGTAGA	CDC42__NM_001039802	195	215	313	255
CDC42__NM_001039802_GGCAAGTTTCTCAATAGTAG	CDC42__NM_001039802	683	766	871	935
CDC42__NM_001039802_TGTATTTGACGAAGCAATAT	CDC42__NM_001039802	26	8	55	12
CDC42__NM_044472_CTCCGGAAACTCAACCCAAA	CDC42__NM_044472	316	550	411	305
CDC42BPA__NM_003607_ATATCCATCAGTATATTGTC	CDC42BPA__NM_003607	418	308	440	410
CDC42BPA__NM_003607_ATTCTCGAATACCTAGAATG	CDC42BPA__NM_003607	203	169	239	210
CDC42BPA__NM_003607_ATTTCAGGAGAGATATAATC	CDC42BPA__NM_003607	86	54	106	87
CDC42BPA__NM_003607_CATTCTCGAATACCTAGAAT	CDC42BPA__NM_003607	496	841	606	545
CDC42BPA__NM_003607_CTGGTTATGGATTATTATGT	CDC42BPA__NM_003607	239	329	231	195
CDC42BPA__NM_003607_GAAAGGGATGTATTAGTAGAA	CDC42BPA__NM_003607	237	192	299	289
CDC42BPA__NM_003607_GAATCGCTGGTGGAGACATA	CDC42BPA__NM_003607	264	139	238	294
CDC42BPA__NM_003607_TAAACCTGACAATATACTGA	CDC42BPA__NM_003607	478	520	541	482
CDC42BPA__NM_003607_TAGTGAATGGAGACAATAAA	CDC42BPA__NM_003607	142	301	135	117
CDC42BPA__NM_003607_TGATGTGTCTGAAAATGCTA	CDC42BPA__NM_003607	341	328	318	275
CDC42BPA__NM_003607_TGTCTGGAGGAAGTGCGTTTG	CDC42BPA__NM_003607	964	923	1008	1121
CDC42BPA__NM_003607_TTACTAAGTTATTGTCATCC	CDC42BPA__NM_003607	328	448	432	332
CDC42BPB__NM_006035_ACGTGGCCGAGTTCCTCGAG	CDC42BPB__NM_006035	862	837	924	1138
CDC42BPB__NM_006035_ACTCGGCCACGTACTTGTCG	CDC42BPB__NM_006035	2100	1962	2248	1614
CDC42BPB__NM_006035_AGACAAGCTTCCGGAAGATA	CDC42BPB__NM_006035	273	122	340	251
CDC42BPB__NM_006035_AGCTTCCGGAAGATATGGCG	CDC42BPB__NM_006035	428	658	500	494
CDC42BPB__NM_006035_AGGGCGCTCTCGTTGCGCCA	CDC42BPB__NM_006035	15	13	21	20
CDC42BPB__NM_006035_AGTGGCTGCACTCGGTGTAC	CDC42BPB__NM_006035	412	413	632	429
CDC42BPB__NM_006035_CCTGCGCCGCGACAAGTACG	CDC42BPB__NM_006035	815	750	837	904
CDC42BPB__NM_006035_CGAGCAGCGTTTCCACGCTC	CDC42BPB__NM_006035	630	666	686	788
CDC42BPB__NM_006035_CTTAGTCATGGATTACTATG	CDC42BPB__NM_006035	292	249	463	460
CDC42BPB__NM_006035_GATATGGCGAGGTTCTACAT	CDC42BPB__NM_006035	322	368	524	559
CDC42BPB__NM_006035_GTGGCCGAGTTCCTCGAGTG	CDC42BPB__NM_006035	300	170	258	358
CDC42BPB__NM_006035_TATGACCATTCACGTCCAAA	CDC42BPB__NM_006035	624	596	608	724
CDC42BPG__NM_017525_AGCGGGATGTGCTCGTGAAA	CDC42BPG__NM_017525	530	435	611	713
CDC42BPG__NM_017525_AGCTCAGCAGCGGCCCCCTA	CDC42BPG__NM_017525	865	720	954	1146
CDC42BPG__NM_017525_AGTAGTCCATCACAAGGTAC	CDC42BPG__NM_017525	86	65	102	102
CDC42BPG__NM_017525_CACGCTGCGCTCCCGCCGTA	CDC42BPG__NM_017525	266	224	243	388
CDC42BPG__NM_017525_CAGGTACTCCTCGTCTTGGA	CDC42BPG__NM_017525	995	852	1021	906
CDC42BPG__NM_017525_CTTGTGATGGACTACTATGC	CDC42BPG__NM_017525	1049	1268	1132	1074
CDC42BPG__NM_017525_GACGCTGCTGAGCCGCTTCG	CDC42BPG__NM_017525	875	1282	943	1292
CDC42BPG__NM_017525_GAGCGGGATGTGCTCGTGAA	CDC42BPG__NM_017525	790	807	860	752
CDC42BPG__NM_017525_GCGCCAGCAGCAGATCTAGG	CDC42BPG__NM_017525	555	613	550	606
CDC42BPG__NM_017525_GCGGGATGTGCTCGTGAAAG	CDC42BPG__NM_017525	715	925	746	950
CDC42BPG__NM_017525_TGCCTCACCACGGTGACCTA	CDC42BPG__NM_017525	284	348	245	239
CDC42BPG__NM_017525_TTGGAAGGCATAGTGCAGAG	CDC42BPG__NM_017525	132	68	164	266
CDC42EP1__NM_152243_AACCCAGCTGTTACCCCATT	CDC42EP1__NM_152243	2098	1800	2228	1722
CDC42EP1__NM_152243_ACTACCCAGAGATGGATGCG	CDC42EP1__NM_152243	2111	1922	2070	2260
CDC42EP1__NM_152243_ACTCCTCAGCGAGCTGCTAG	CDC42EP1__NM_152243	212	200	285	200
CDC42EP1__NM_152243_CAACCCAGCTGTTACCCCAT	CDC42EP1__NM_152243	2446	1529	2404	1533
CDC42EP1__NM_152243_CCGTGGCGGGGATGTCTTCG	CDC42EP1__NM_152243	2857	3466	3202	3767
CDC42EP1__NM_152243_GAAGCTGCGGGGTGAGCGAT	CDC42EP1__NM_152243	310	146	240	133
CDC42EP1__NM_152243_GACCCGTGGGGTTTGCAGTA	CDC42EP1__NM_152243	358	478	392	519
CDC42EP1__NM_152243_GGCTGGGGGGTTTGCAGCGG	CDC42EP1__NM_152243	806	791	889	918
CDC42EP1__NM_152243_GGCTGGGTGTCCAGTTCACA	CDC42EP1__NM_152243	1115	1085	1236	1128
CDC42EP1__NM_152243_GGGGGAGATGCCATCCTCCG	CDC42EP1__NM_152243	1585	2174	1942	2523
CDC42EP1__NM_152243_GGGGGGTTTGCAGTAGTGGC	CDC42EP1__NM_152243	525	758	645	890
CDC42EP1__NM_152243_TCCTTCCTCAGCAACCACGG	CDC42EP1__NM_152243	339	296	315	238
CDC42EP2__NM_006779_ATCTCCCTCCCGGTTATCGG	CDC42EP2__NM_006779	409	158	347	259
CDC42EP2__NM_006779_CCCGCACCGCCACCGTGTGT	CDC42EP2__NM_006779	3406	2549	3558	2586
CDC42EP2__NM_006779_CCTGGGGTCCACCGATAACC	CDC42EP2__NM_006779	411	422	433	326
CDC42EP2__NM_006779_CTGAAGCGTGGCAGTCGCAA	CDC42EP2__NM_006779	446	398	531	307
CDC42EP2__NM_006779_CTGCCACGCTTCAGATAGAT	CDC42EP2__NM_006779	765	983	967	993
CDC42EP2__NM_006779_GCCATCTCCCTCCCGGTTAT	CDC42EP2__NM_006779	1820	1965	2032	1791
CDC42EP2__NM_006779_GCCTGGGGTCCACCGATAAC	CDC42EP2__NM_006779	838	557	774	576
CDC42EP2__NM_006779_GGCGGCGGCAGTGACATGTT	CDC42EP2__NM_006779	2236	1988	2440	2276
CDC42EP2__NM_006779_GGGGTCCACCGATAACCGGG	CDC42EP2__NM_006779	251	81	282	233
CDC42EP2__NM_006779_GGTCTCCAGGTGCAGGCGAG	CDC42EP2__NM_006779	192	276	192	179
CDC42EP2__NM_006779_TCTGAAGCGTGGCAGTCGCA	CDC42EP2__NM_006779	214	199	180	243
CDC42EP3__NM_001270436_AATGTCACTGGTGACAATAA	CDC42EP3__NM_001270436	96	74	90	148
CDC42EP3__NM_001270436_AGTCGGGGTACTGTTCGGAC	CDC42EP3__NM_001270436	627	559	627	485
CDC42EP3__NM_001270436_ATCTCCCTCCCGACCATTGG	CDC42EP3__NM_001270436	799	838	983	814
CDC42EP3__NM_001270436_CACCAGGGAGACACCTCGTG	CDC42EP3__NM_001270436	897	614	957	754
CDC42EP3__NM_001270436_CCACCAGGGAGACACCTCGT	CDC42EP3__NM_001270436	1203	918	1378	1050
CDC42EP3__NM_001270436_CGGAAGAACTCATTATGCCC	CDC42EP3__NM_001270436	771	815	1015	877
CDC42EP3__NM_001270436_GCCATCTCCCTCCCGACCAT	CDC42EP3__NM_001270436	2245	2146	2484	2015
CDC42EP3__NM_001270436_GGACTGAGATGCAGAACCGC	CDC42EP3__NM_001270436	536	389	601	459
CDC42EP3__NM_001270436_GGATCCTCCAATGGTCGGGA	CDC42EP3__NM_001270436	643	917	621	730
CDC42EP3__NM_001270436_GGCTGCTTTCAGGTAAATTG	CDC42EP3__NM_001270436	396	570	433	524
CDC42EP3__NM_001270436_TCCACCAGGGAGACACCTCG	CDC42EP3__NM_001270436	657	728	808	874
CDC42EP3__NM_001270436_TTGATGAGCTCGCATGGGGT	CDC42EP3__NM_001270436	301	212	321	299
CDC42EP4__NM_012121_AAGAAGGCCAATGACGGGGA	CDC42EP4__NM_012121	371	331	396	380
CDC42EP4__NM_012121_AGAAGAGGGCTGTTCGTCCA	CDC42EP4__NM_012121	758	989	832	792
CDC42EP4__NM_012121_AGCAAGGCTGGCGAGCCCGA	CDC42EP4__NM_012121	476	356	415	398
CDC42EP4__NM_012121_CAGGCTCTTGGGCAGCTTAC	CDC42EP4__NM_012121	224	244	189	185
CDC42EP4__NM_012121_CATGGCATTCTTGACAAACA	CDC42EP4__NM_012121	199	145	200	233
CDC42EP4__NM_012121_GAGGCAGTGCCCCGTCGGAA	CDC42EP4__NM_012121	652	990	788	900
CDC42EP4__NM_012121_GCCACACCATGCACGTTGGC	CDC42EP4__NM_012121	1320	1492	1562	1763
CDC42EP4__NM_012121_GCCAGCCTTGCTATTGAGGA	CDC42EP4__NM_012121	507	777	566	769
CDC42EP4__NM_012121_GGGCAGCAAGCGGTCACAGT	CDC42EP4__NM_012121	675	618	592	674
CDC42EP4__NM_012121_TCCGGCCCGGCCAACGTGCA	CDC42EP4__NM_012121	185	159	179	265
CDC42EP4__NM_012121_TCCTTCCTCAATAGCAAGGC	CDC42EP4__NM_012121	51	79	47	21
CDC42EP4__NM_012121_TTCCGCCACACCATGCACGT	CDC42EP4__NM_012121	599	682	630	795
CDC42EP5__NM_145057_CTCGAGCTGAACGACGTCAT	CDC42EP5__NM_145057	650	494	996	670
CDC42EP5__NM_145057_GGTGCTGGGCGTCATGGACG	CDC42EP5__NM_145057	655	501	697	609
CDC42EP5__NM_145057_TCCATGCTGGACGCGGTGCT	CDC42EP5__NM_145057	111	85	189	148
CDC42EP5__NM_145057_TTCCGGCACACGCTCACGT	CDC42EP5__NM_145057	34	9	35	96
CDC42SE1__NM_001038707_AGAAGAGAAGACGGATTGAC	CDC42SE1__NM_001038707	1230	928	1444	1311
CDC42SE1__NM_001038707_AGGCCATGGAGCAATTCTAG	CDC42SE1__NM_001038707	441	320	494	432
CDC42SE1__NM_001038707_CGGATTGACCGGACCATGAT	CDC42SE1__NM_001038707	1592	1874	1733	2195
CDC42SE1__NM_001038707_GATCCAAGGGAAACCGAGAT	CDC42SE1__NM_001038707	379	296	421	477
CDC42SE1__NM_001038707_GATTGACCGGACCATGATTG	CDC42SE1__NM_001038707	480	475	447	296
CDC42SE1__NM_001038707_GTCAGGTGAACAAAATTCAT	CDC42SE1__NM_001038707	440	385	445	485
CDC42SE1__NM_001038707_TAGGCCATGGAGCAATTCTA	CDC42SE1__NM_001038707	259	231	325	379
CDC42SE2__NM_001038702_ATGCAGTCCAAGGGAGGTTA	CDC42SE2__NM_001038702	82	55	100	80
CDC42SE2__NM_001038702_CAGAACCAAATGCAGTCCAA	CDC42SE2__NM_001038702	155	213	228	194
CDC42SE2__NM_001038702_CAGCTCGTGGATACGAAGGC	CDC42SE2__NM_001038702	281	356	300	528
CDC42SE2__NM_001038702_CTGAATTCATTCCACTGAAC	CDC42SE2__NM_001038702	231	361	249	200
CDC42SE2__NM_001038702_GCAGCTCGTGGATACGAAGG	CDC42SE2__NM_001038702	128	173	139	67
CDC42SE2__NM_001038702_GCTGTATGCACAAAGTTTGT	CDC42SE2__NM_001038702	1129	946	955	991
CDC45__NM_001178010_AACCGTGTGCGCTTCTCAGA	CDC45__NM_001178010	868	1070	882	1038
CDC45__NM_001178010_ATTTCTTGAGCATAAAGAAC	CDC45__NM_001178010	58	20	102	48
CDC45__NM_001178010_CAATGTATACAACGATACCC	CDC45__NM_001178010	269	7	230	3
CDC45__NM_001178010_CACGTGGTCACACTGGAACA	CDC45__NM_001178010	266	6	271	11
CDC45__NM_001178010_CCGCAAAGAGTTCTACGAGG	CDC45__NM_001178010	382	8	428	7
CDC45__NM_001178010_CTTCATAGGCGGGAACTTCA	CDC45__NM_001178010	155	92	191	73
CDC45__NM_001178010_GAACCAGCGTATATTGCACG	CDC45__NM_001178010	730	22	653	20
CDC45__NM_001178010_GTAGAACTCTTTGCGGAAAT	CDC45__NM_001178010	171	9	202	31
CDC45__NM_001178010_TCTTTGTGTGTGACACCCAT	CDC45__NM_001178010	451	6	652	27
CDC45__NM_001178010_TGACCACGTGCAATATACGC	CDC45__NM_001178010	207	0	218	0
CDC45__NM_001178010_TGACGACATTGACTGGCCTA	CDC45__NM_001178010	799	1040	917	978
CDC45__NM_001178010_TGGAGCTAATGTAGACCTAT	CDC45__NM_001178010	72	11	49	7
CDC45__NM_001178011_ACGAGCAGTATGAATATCAT	CDC45__NM_001178011	858	18	932	19
CDC45__NM_001178011_GAAGCGCACACGGTTAGAAG	CDC45__NM_001178011	507	157	508	135
CDC45__NM_001178011_TACGAGCAGTATGAATATCA	CDC45__NM_001178011	531	28	474	7
CDC45__NM_001178011_TAGTGGAGCAAACCATGCGG	CDC45__NM_001178011	375	105	407	23
CDC5L__NM_001253_AACTGAAAAACCACAGATGG	CDC5L__NM_001253	223	122	238	218
CDC5L__NM_001253_AAGCAGCGGTAATGAAATAT	CDC5L__NM_001253	176	6	235	28
CDC5L__NM_001253_AGCCCGCTTGGCTAATACTC	CDC5L__NM_001253	168	0	298	0
CDC5L__NM_001253_AGGAGATACTAAGTATGTCC	CDC5L__NM_001253	113	92	197	140
CDC5L__NM_001253_AGTGATTCTAACTAGAGTCC	CDC5L__NM_001253	86	208	53	57
CDC5L__NM_001253_AGTGCAAAGATGATTTAATG	CDC5L__NM_001253	541	738	577	483
CDC5L__NM_001253_ATGCCTCGAATTATGATCAA	CDC5L__NM_001253	204	50	285	41
CDC5L__NM_001253_ATTATGAGATAGTGAATGAG	CDC5L__NM_001253	127	130	95	46
CDC5L__NM_001253_ATTTGGGGAATGGATAGAGG	CDC5L__NM_001253	149	140	138	123
CDC5L__NM_001253_CACTGCATCAGGCTCCAGTT	CDC5L__NM_001253	800	581	977	897
CDC5L__NM_001253_CAGCTTATAGTGGTACGGTG	CDC5L__NM_001253	642	512	683	742
CDC5L__NM_001253_CAGTATCTTAAACACTTAAG	CDC5L__NM_001253	20	9	8	12
CDC5L__NM_001253_CCCCCCTTGATCATAATTCG	CDC5L__NM_001253	951	163	923	252
CDC5L__NM_001253_CCGTACCACTATAAGCTGCA	CDC5L__NM_001253	771	788	769	871
CDC5L__NM_001253_CCTCGAATTATGATCAAGGG	CDC5L__NM_001253	908	193	1043	302
CDC5L__NM_001253_CGATGAGAAGATCTAAGCAA	CDC5L__NM_001253	194	137	168	145
CDC5L__NM_001253_CTTAGAATACTAGGGACTAT	CDC5L__NM_001253	157	215	197	338
CDC5L__NM_001253_GAATACTAGGGACTATAGGA	CDC5L__NM_001253	173	182	138	267
CDC5L__NM_001253_GATTTAGTTACAGTATCAGT	CDC5L__NM_001253	417	373	416	277
CDC5L__NM_001253_GCATTAAGCAGTTATAACAT	CDC5L__NM_001253	2056	1324	2090	1327
CDC5L__NM_001253_GCCTCGAATTATGATCAAGG	CDC5L__NM_001253	252	70	398	35
CDC5L__NM_001253_GCGGCCTGATCCAATTGATA	CDC5L__NM_001253	466	7	499	47
CDC5L__NM_001253_GGTAGTGGTGCCACTTGAAA	CDC5L__NM_001253	312	203	289	355
CDC5L__NM_001253_GGTATTGTTCACTAAGGTAT	CDC5L__NM_001253	110	206	153	82
CDC5L__NM_001253_GGTTGTTAAGGCATTCATTA	CDC5L__NM_001253	180	206	169	184
CDC5L__NM_001253_GTAATAGTAGTGGAGATATG	CDC5L__NM_001253	127	234	170	170
CDC5L__NM_001253_GTGTCAGACACTTAAGTGTT	CDC5L__NM_001253	426	496	613	580
CDC5L__NM_001253_TAGAGTCCAGGTAATAGTAG	CDC5L__NM_001253	408	371	431	472
CDC5L__NM_001253_TATAGCCTAAAAAAATCGGC	CDC5L__NM_001253	93	99	133	103
CDC5L__NM_001253_TCATCCATATCAATTGGATC	CDC5L__NM_001253	830	354	949	609
CDC5L__NM_001253_TCCACAGAATCTAAAACTCA	CDC5L__NM_001253	370	332	333	289
CDC5L__NM_001253_TCTTCCAATGATTGGAGCAA	CDC5L__NM_001253	167	0	148	26
CDC5L__NM_001253_TGATCAAGGGGGGCGTATGG	CDC5L__NM_001253	118	0	157	4
CDC5L__NM_001253_TGCCCAGTCTTAGAATACTA	CDC5L__NM_001253	435	344	468	329
CDC5L__NM_001253_TGTCTGTTCACCATTTCAAG	CDC5L__NM_001253	92	56	123	21
CDC5L__NM_001253_TGTGTCAGACACTTAAGTGT	CDC5L__NM_001253	801	735	936	939
CDC5L__NM_001253_TTACCCTCATCCATATCAAT	CDC5L__NM_001253	303	19	334	58
CDC5L__NM_001253_TTAGTGTGTAAAATCTCACC	CDC5L__NM_001253	134	149	178	127
CDC5L__NM_001253_TTATGATCAAGGGGGGCGTA	CDC5L__NM_001253	76	5	99	10
CDC5L__NM_001253_TTATGTGGCTCTATACAACG	CDC5L__NM_001253	47	63	88	139
CDC5L__NM_001253_TTTGGCCGTGCAGCTTATAG	CDC5L__NM_001253	139	87	174	128
CDC6__NM_001254_AAGGTGGGCAGATCACTTGA	CDC6__NM_001254	14	0	4	0
CDC6__NM_001254_CCAGCTGAATACTTTCGGGT	CDC6__NM_001254	1768	1103	1541	1254
CDC6__NM_001254_CCCTCACTCACATACACTTA	CDC6__NM_001254	1030	835	1131	769
CDC6__NM_001254_CCCTTAAGTGTATGTGAGTG	CDC6__NM_001254	603	335	651	606
CDC6__NM_001254_CTCAGCCCCAGGAAACGTCT	CDC6__NM_001254	345	243	347	213
CDC6__NM_001254_GACATTTGTTGGTTCTAGTT	CDC6__NM_001254	220	151	325	387
CDC6__NM_001254_GAGGAGAACAGGTTACGGTT	CDC6__NM_001254	349	402	385	483
CDC6__NM_001254_GATAAATTAGTCCATATGGT	CDC6__NM_001254	379	244	373	326
CDC6__NM_001254_GGAGAACAAGGAGGTAAATG	CDC6__NM_001254	21	8	20	1
CDC6__NM_001254_GTAAATGGGGAGTGTTGCAT	CDC6__NM_001254	429	336	323	269
CDC6__NM_001254_GTTTACCCAGACGTTTCCTG	CDC6__NM_001254	271	103	255	126
CDC6__NM_001254_TACACGAGGAGAACAGGTTA	CDC6__NM_001254	292	269	354	442
CDC6__NM_001254_TCTCAGCCCCAGGAAACGTC	CDC6__NM_001254	447	204	520	286
CDC6__NM_001254_TGTGCCTGGGATCGGGTTTG	CDC6__NM_001254	470	241	462	184
CDC6__NM_001254_TTCTCTTTCTTGCCTTGCTT	CDC6__NM_001254	1	0	14	9
CDC6__NM_001254_TTTGGTTTGGTGGCATTAAA	CDC6__NM_001254	36	0	13	66
CDC7__NM_001134419_AACAAATCCCACATAATCAC	CDC7__NM_001134419	410	269	453	206
CDC7__NM_001134419_AATTGCTGGGCTTAACATCA	CDC7__NM_001134419	208	0	212	0
CDC7__NM_001134419_ACTGAGTGGCCCAGTACCTA	CDC7__NM_001134419	752	284	604	291
CDC7__NM_001134419_AGCCTGAAACCGGTCACGCT	CDC7__NM_001134419	662	364	761	418
CDC7__NM_001134419_AGGTATGCCTTGGTAGACTT	CDC7__NM_001134419	213	0	279	3
CDC7__NM_001134419_AGTTCAGCTGCAATTCTTAT	CDC7__NM_001134419	1190	127	1326	131
CDC7__NM_001134419_CTTCAGTGCCTAACAGTGGC	CDC7__NM_001134419	208	176	245	186
CDC7__NM_001134419_GATCCAGCTCCTTAGGTACT	CDC7__NM_001134419	825	509	845	571
CDC7__NM_001134419_GGCCAAACCAAAGTCTACCA	CDC7__NM_001134419	193	19	263	13
CDC7__NM_001134419_TAGGGGGCAAGATAATGTCA	CDC7__NM_001134419	83	0	71	0
CDC7__NM_001134419_TCTTGTTTCCTGTGATTATG	CDC7__NM_001134419	635	858	608	924
CDC7__NM_001134419_TTAAACACATTACTAAGCTG	CDC7__NM_001134419	16	0	42	15
CDC7__NM_003503_AACACTTCATGAAAGCATTC	CDC7__NM_003503	143	136	139	130
CDC7__NM_003503_ACCTAAGACTTAGTCAAAAC	CDC7__NM_003503	334	350	370	384
CDC7__NM_003503_AGCTAAAAATCTGACCTAAT	CDC7__NM_003503	549	562	753	781
CDC7__NM_003503_ATAATTAGGTAGCTGCTGAA	CDC7__NM_003503	522	418	516	615
CDC7__NM_003503_ATACATACACTGGTTGATAA	CDC7__NM_003503	332	348	378	320
CDC7__NM_003503_ATGTCAGTGAAAGGTAATTA	CDC7__NM_003503	121	94	144	166
CDC7__NM_003503_CAATGAGGAAATGTTGGTCA	CDC7__NM_003503	847	811	1068	1263
CDC7__NM_003503_CACCTAAAATCTACTTAGGT	CDC7__NM_003503	403	586	627	562
CDC7__NM_003503_CATTCTCCTCGGAAATTACA	CDC7__NM_003503	226	320	289	296
CDC7__NM_003503_GACTAAGTCTTAGGTGGGTT	CDC7__NM_003503	312	360	483	471
CDC7__NM_003503_GAGAAAAGGACCCAAGATAG	CDC7__NM_003503	129	119	179	140
CDC7__NM_003503_GATATCTACCTAATCTGTTT	CDC7__NM_003503	426	459	433	501
CDC7__NM_003503_TACAGAATTGACGGTATTAT	CDC7__NM_003503	454	440	424	656
CDC7__NM_003503_TAGTTTGGGGAAACTCAACC	CDC7__NM_003503	81	147	66	97
CDC7__NM_003503_TCAGCACCATGTAATTTCCG	CDC7__NM_003503	715	622	770	444
CDC7__NM_003503_TTATGAGCTAGGTATAGTTT	CDC7__NM_003503	325	242	193	147
CDC73__NM_024529_AAGACCAACTATGTTGTTTG	CDC73__NM_024529	194	26	211	61
CDC73__NM_024529_ACCCAGAGAGTACTACACAT	CDC73__NM_024529	910	13	759	26
CDC73__NM_024529_AGAAGCGCTCCCTTAGAAAT	CDC73__NM_024529	355	293	404	189
CDC73__NM_024529_AGAGAGATTGGCTGCCCGTT	CDC73__NM_024529	1270	320	1380	364
CDC73__NM_024529_AGCAAAGAAACCACGAATTG	CDC73__NM_024529	100	22	136	6
CDC73__NM_024529_CGATCAGGTCTTCTAACCAC	CDC73__NM_024529	763	214	904	496
CDC73__NM_024529_GCGCCTTGATAAAGAGAGAT	CDC73__NM_024529	460	215	489	382
CDC73__NM_024529_GCTGCACGTCGGACATAAAC	CDC73__NM_024529	465	0	500	21
CDC73__NM_024529_GGGAGACGAAGTGATCTTCG	CDC73__NM_024529	309	0	288	2
CDC73__NM_024529_TCGCTGAAGACCTATTTCTA	CDC73__NM_024529	493	296	621	366
CDC73__NM_024529_TGAAGACCAACTATGTTGTT	CDC73__NM_024529	128	1	105	0
CDC73__NM_024529_TTACCCCCAAACAACATAGT	CDC73__NM_024529	197	2	169	10
CDCA2__NM_152562_ACAGTAACCGTAGCAGCAATT	CDCA2__NM_152562	422	508	469	457
CDCA2__NM_152562_ATAGGATATTCTCCGACGTT	CDCA2__NM_152562	956	826	1103	899
CDCA2__NM_152562_CACAGTAACCGTAGCAGCAAT	CDCA2__NM_152562	651	677	811	809
CDCA2__NM_152562_CCCAAGATATCATCTAAACT	CDCA2__NM_152562	843	995	950	1141
CDCA2__NM_152562_GAAAATCTGACGGATGCTGA	CDCA2__NM_152562	267	294	377	467
CDCA2__NM_152562_GAGCAATGAAACGAATCAGA	CDCA2__NM_152562	395	320	412	395
CDCA2__NM_152562_GCAAGGATTAGGAGGTAATT	CDCA2__NM_152562	74	103	113	93
CDCA2__NM_152562_GGATATTCTCCGACGTTTGG	CDCA2__NM_152562	297	314	370	371
CDCA2__NM_152562_GTATCTGGTGTGCAAGGATT	CDCA2__NM_152562	352	356	398	496
CDCA2__NM_152562_TGAGAAAATGACCGGCTGTC	CDCA2__NM_152562	444	433	489	549
CDCA2__NM_152562_TTAACATTTCGATACAGTGC	CDCA2__NM_152562	40	26	59	35
CDCA2__NM_152562_TTCATCAGAGTCTCCTGAT	CDCA2__NM_152562	236	245	178	223
CDCA3__NM_031299_AAGTCCAATTCAGAAGATAA	CDCA3__NM_031299	81	69	121	94
CDCA3__NM_031299_ATGCCAGCACTAGGTGAACG	CDCA3__NM_031299	391	269	367	202
CDCA3__NM_031299_CACTCAGCTGTTTCACCAGT	CDCA3__NM_031299	1598	1921	1935	2234
CDCA3__NM_031299_CTCCTACTCTTGGTATTGCA	CDCA3__NM_031299	636	757	715	796
CDCA3__NM_031299_GAGGCCGCGCTGGTGTGACT	CDCA3__NM_031299	1906	1315	2019	1271
CDCA3__NM_031299_GATGCCAGCACTAGGTGAAC	CDCA3__NM_031299	338	108	364	110
CDCA3__NM_031299_GCCAGATGCTTGTTGTGCGG	CDCA3__NM_031299	1327	1036	1380	923
CDCA3__NM_031299_GGAGGGTCTTAAACATGCCC	CDCA3__NM_031299	33	0	5	1
CDCA3__NM_031299_GGATGCCAGCACTAGGTGAA	CDCA3__NM_031299	120	120	102	27
CDCA3__NM_031299_GGGTACCCAGTTATCTGTTG	CDCA3__NM_031299	596	791	583	711
CDCA3__NM_031299_GTGCGCAGGATGCCAGCACT	CDCA3__NM_031299	589	256	659	250
CDCA3__NM_031299_TCTGTTCCTCAACAGATAAC	CDCA3__NM_031299	63	97	139	103
CDCA4__NM_017955_AAGAGGAGATGACGCAGGAT	CDCA4__NM_017955	326	169	366	312
CDCA4__NM_017955_ACTGTGTCCAGGTCGTAGTA	CDCA4__NM_017955	335	363	378	443
CDCA4__NM_017955_CAAGAGGAGATGACGCAGGA	CDCA4__NM_017955	405	526	484	426
CDCA4__NM_017955_CTGTGTCCAGGTCGTAGTAG	CDCA4__NM_017955	1116	1106	1155	1038
CDCA4__NM_017955_GGAAGAGCTGTTCTCAGACG	CDCA4__NM_017955	443	692	496	526
CDCA4__NM_017955_GGACAGCCCCTACTACGACC	CDCA4__NM_017955	814	697	967	790
CDCA4__NM_017955_GTGGAGACCAAGCGGTCGAG	CDCA4__NM_017955	72	18	93	50
CDCA4__NM_017955_TACTGTGTCCAGGTCGTAGT	CDCA4__NM_017955	372	313	423	496
CDCA4__NM_017955_TCCAGCTGCAAGTCCGACCT	CDCA4__NM_017955	1079	752	1167	1127
CDCA4__NM_017955_TCCTCATTGCCAACACGGTC	CDCA4__NM_017955	92	109	134	214
CDCA5__NM_080668_ACGATCCTCTTTAAGACGAT	CDCA5__NM_080668	387	316	510	456
CDCA5__NM_080668_AGCTTCAAACTCGGCATTCA	CDCA5__NM_080668	321	82	336	47
CDCA5__NM_080668_CACTCCTGTGCCGAACCCTG	CDCA5__NM_080668	11	5	23	16
CDCA5__NM_080668_CCCTGGCAGGGAGCTTACTA	CDCA5__NM_080668	257	104	314	173
CDCA5__NM_080668_CCTCCCTGAAATCTGGCCGA	CDCA5__NM_080668	1259	1504	1732	1527
CDCA5__NM_080668_CGCAGAGGCTTAGTAGGAGA	CDCA5__NM_080668	590	588	757	768
CDCA5__NM_080668_GAAAGCCCATCGTCTTAAAG	CDCA5__NM_080668	182	96	219	228
CDCA5__NM_080668_TCTGGGAGGCGAACGCGGTC	CDCA5__NM_080668	364	231	267	194
CDCA7__NM_031942_AAATGATTCATCGTCAGAGT	CDCA7__NM_031942	256	297	304	262
CDCA7__NM_031942_AGAGGATGACGAGGTTTCCA	CDCA7__NM_031942	177	102	166	135
CDCA7__NM_031942_CAAGATGTATTAGACCATTG	CDCA7__NM_031942	504	500	543	635
CDCA7__NM_031942_CAGGCACTCTGGACCTCTCA	CDCA7__NM_031942	1058	1017	1135	1065
CDCA7__NM_031942_CGGGACCTTGACCTGGTAAG	CDCA7__NM_031942	93	196	140	206
CDCA7__NM_031942_CGTCAAGGGACCCGAGGATC	CDCA7__NM_031942	343	452	353	353
CDCA7__NM_031942_CTCCATGGGTAGAGCGTCAA	CDCA7__NM_031942	74	20	100	89
CDCA7__NM_031942_GAGTGAGATACAAGATGGAA	CDCA7__NM_031942	148	280	175	156
CDCA7__NM_031942_GATGTCACTAACGAACTGGC	CDCA7__NM_031942	1022	938	1160	1332
CDCA7__NM_031942_GCCAGATGTCACTAACGAAC	CDCA7__NM_031942	257	289	270	328
CDCA7__NM_031942_TGTCTTCCACGGAACGAGCC	CDCA7__NM_031942	57	23	58	59
CDCA7__NM_031942_TTCATCTTCTGAATCGGAGT	CDCA7__NM_031942	39	16	57	43
CDCA7__NM_145810_CTGGGCGAATTATATGCGGA	CDCA7__NM_145810	648	771	690	881
CDCA7__NM_145810_GGAAGAGGATAAGTACATGT	CDCA7__NM_145810	790	743	842	684
CDCA7__NM_145810_TCACGGATGATCTGGAGCGA	CDCA7__NM_145810	412	474	428	345
CDCA7__NM_145810_TCCACTGGGCGAATTATATG	CDCA7__NM_145810	627	537	581	684
CDCA7__NM_145810_TCCGCATATAATTCGCCCAG	CDCA7__NM_145810	1003	784	1076	907
CDCA7L__NM_001127370_AGGCTACCCTAGAAGAAGC	CDCA7L__NM_001127370	207	275	240	150
CDCA7L__NM_001127370_AGGTCTAGAAGAAGTAGTAT	CDCA7L__NM_001127370	257	307	372	403
CDCA7L__NM_001127370_AGTTCAATTCCGCCAATAAC	CDCA7L__NM_001127370	169	296	176	299
CDCA7L__NM_001127370_CAAAGCATCTTTGGTGAGCG	CDCA7L__NM_001127370	479	615	570	683
CDCA7L__NM_001127370_CTGAGGTTGGGGTTCGTACT	CDCA7L__NM_001127370	762	973	893	905
CDCA7L__NM_001127370_CTGTAGACAGGTGATACAAA	CDCA7L__NM_001127370	154	215	144	180
CDCA7L__NM_001127370_GAGTCAGATTTGAGTGATGA	CDCA7L__NM_001127370	347	417	411	620
CDCA7L__NM_001127370_GCTGAGGTTGGGGTTCGTAC	CDCA7L__NM_001127370	1283	1492	1564	1584
CDCA7L__NM_001127370_GTGAGGCGGGCCTTCTCGGA	CDCA7L__NM_001127370	2756	2485	2822	2816
CDCA7L__NM_001127370_GTTCAATTCCGCCAATAACT	CDCA7L__NM_001127370	111	166	160	182
CDCA7L__NM_001127370_TCAGCTTGCCCAGTTATTGG	CDCA7L__NM_001127370	602	598	642	485
CDCA7L__NM_001127370_TCTCGGAGGGCAGACAGATCACG	CDCA7L__NM_001127370	227	296	376	294
CDCA8__NM_001256875_ACAGGTAGATGAAATGATAG	CDCA8__NM_001256875	202	67	189	84
CDCA8__NM_001256875_ATGAACTGGCTTGACTACTT	CDCA8__NM_001256875	297	7	299	64
CDCA8__NM_001256875_CCAAGACCAACTCCTTACGG	CDCA8__NM_001256875	444	90	439	117
CDCA8__NM_001256875_CCCAGCCGTGGGCCGATTGG	CDCA8__NM_001256875	302	130	307	220
CDCA8__NM_001256875_CTGAAAGACTTCGACCGTGA	CDCA8__NM_001256875	322	1	468	22
CDCA8__NM_001256875_GCCTGGAGTTGGTTTGACCA	CDCA8__NM_001256875	890	356	922	409
CDCA8__NM_001256875_GGCCTGTTTGTTTCCTCCAA	CDCA8__NM_001256875	626	591	570	605
CDCA8__NM_001256875_GTCGAAGTCTTTCAGAAAGG	CDCA8__NM_001256875	136	0	142	0
CDCA8__NM_001256875_TACAGCACGAAAGGTAATAC	CDCA8__NM_001256875	293	396	338	266
CDCA8__NM_001256875_TACCCCAGCCGTGGGCCGAT	CDCA8__NM_001256875	796	361	852	452
CDCA8__NM_001256875_TCAAGCAAATTGAGTCAGAC	CDCA8__NM_001256875	288	416	313	426
CDCA8__NM_001256875_TGACACCCAGGTTTCGACTCA	CDCA8__NM_001256875	595	261	856	613
CDCP1__NM_022842_ATTACAGTTCTCATAAAGCT	CDCP1__NM_022842	204	330	233	150
CDCP1__NM_022842_CATTACAGTTCTCATAAAGC	CDCP1__NM_022842	351	308	418	553
CDCP1__NM_022842_CCCAGATGAAAGTTCTGTTG	CDCP1__NM_022842	661	631	719	697
CDCP1__NM_022842_CGCCTGAGGCAGATCGGTCC	CDCP1__NM_022842	2664	3514	3005	3395
CDCP1__NM_022842_GGACCGATCTGCCTCAGGCG	CDCP1__NM_022842	221	236	308	237
CDCP1__NM_022842_GGGGTCTCTATCGCACTGCT	CDCP1__NM_022842	350	514	426	512
CDCP1__NM_022842_GGTCTCTATCGCACTGCTAG	CDCP1__NM_022842	415	496	457	506
CDCP1__NM_022842_GTAGGCAACAACGATGTCGA	CDCP1__NM_022842	347	385	467	503
CDCP1__NM_022842_GTCAAAGCTCATAAGAGCAT	CDCP1__NM_022842	298	229	284	225
CDCP1__NM_022842_TCGCCTGAGGCAGATCGGTC	CDCP1__NM_022842	414	467	558	504
CDCP1__NM_022842_TTAGAAATGACGATGTAACA	CDCP1__NM_022842	95	111	94	123
CDCP1__NM_022842_TTTAGAAATGACGATGTAAC	CDCP1__NM_022842	23	0	20	16
CDCP2__NM_201546_ACACCCCCACATTTGACACC	CDCP2__NM_201546	1764	2442	1752	2317
CDCP2__NM_201546_AGCTGCACTCTGTGTTGTAG	CDCP2__NM_201546	583	308	788	466
CDCP2__NM_201546_CACCCCCACATTTGACACCT	CDCP2__NM_201546	2368	2225	2733	2522
CDCP2__NM_201546_CTTCCACTCGGACAAGCATG	CDCP2__NM_201546	238	244	311	301
CDCP2__NM_201546_GATGACAGACATGACATCGC	CDCP2__NM_201546	543	669	564	590
CDCP2__NM_201546_GGCCCAGAGACAGAGGGTG	CDCP2__NM_201546	397	537	535	533
CDCP2__NM_201546_GGCGTCCTGACTGGCCTGTC	CDCP2__NM_201546	2442	3219	2754	3796
CDCP2__NM_201546_GGGGTACAGTCTAGGGAAGT	CDCP2__NM_201546	382	373	575	631
CDCP2__NM_201546_GGGTAGTTGTTGGGATACTC	CDCP2__NM_201546	86	95	120	119
CDCP2__NM_201546_GTCGTGGTACTCTAGGTCAA	CDCP2__NM_201546	231	182	143	193
CDCP2__NM_201546_GTTGTTGGGATACTCAGGAC	CDCP2__NM_201546	613	651	632	657
CDCP2__NM_201546_TGTTGTAGGGGTACAGTCTA	CDCP2__NM_201546	354	322	379	363
CDH1__NM_004360_AAGAAGTTTGTGTCTTTGTC	CDH1__NM_004360	117	182	153	271
CDH1__NM_004360_AAGACAGGGTCTCATTCTAT	CDH1__NM_004360	296	35	385	62
CDH1__NM_004360_AAGATTGCACCGGTCGACAA	CDH1__NM_004360	181	282	191	147
CDH1__NM_004360_ACCACCATGTAAAGAGTGAT	CDH1__NM_004360	948	1007	971	1056
CDH1__NM_004360_ACTGATGCTGAGGATGATTG	CDH1__NM_004360	496	521	513	652
CDH1__NM_004360_ACTTACTCAGAACAAGTCAC	CDH1__NM_004360	304	406	290	294
CDH1__NM_004360_AGAAGAAAATGTGGCCCTAA	CDH1__NM_004360	105	184	166	156
CDH1__NM_004360_AGCTCTCGGCGTCAAAGCCA	CDH1__NM_004360	110	109	130	99
CDH1__NM_004360_AGGGTCTCATTCTATCGGCC	CDH1__NM_004360	356	228	359	237
CDH1__NM_004360_ATAGGCTGTCCTTTGTCGAC	CDH1__NM_004360	831	907	869	845
CDH1__NM_004360_CACTGTATTCAGCGTGACTT	CDH1__NM_004360	472	806	617	517
CDH1__NM_004360_CAGAAACTCATCTCAAGGGA	CDH1__NM_004360	308	496	252	331
CDH1__NM_004360_CAGAGCGGAACTATGAAAAG	CDH1__NM_004360	193	175	182	253
CDH1__NM_004360_CATCAGTTTGCTAAATTGCC	CDH1__NM_004360	634	738	784	1061
CDH1__NM_004360_CCACTTTGAATCGGGTGTCG	CDH1__NM_004360	805	1238	1274	1568
CDH1__NM_004360_CCATTTCTTGGTCTACGCCT	CDH1__NM_004360	663	1064	831	989
CDH1__NM_004360_CCCTAAAGGGGGTTAGTTGA	CDH1__NM_004360	321	453	287	390
CDH1__NM_004360_CCTAAAGGGGGTTAGTTGAG	CDH1__NM_004360	157	249	187	292
CDH1__NM_004360_CCTCGACACCCGATTCAAAG	CDH1__NM_004360	837	1117	915	1231
CDH1__NM_004360_CCTGAGTATGTAACTTGCAA	CDH1__NM_004360	478	707	618	607
CDH1__NM_004360_CGCCGAGAGCTACACGTTCA	CDH1__NM_004360	1141	1286	1273	1571
CDH1__NM_004360_CTGACACCCGATTCAAAGT	CDH1__NM_004360	451	342	550	512
CDH1__NM_004360_CTGAGTATGTAACTTGCAAT	CDH1__NM_004360	656	790	980	1176
CDH1__NM_004360_GAAGAAAATGTGGCCCTAAA	CDH1__NM_004360	304	255	355	395
CDH1__NM_004360_GGAGAGCTTGTCATTGAGCC	CDH1__NM_004360	500	568	668	841
CDH1__NM_004360_TCGGGTGTCGAGGGAAAAAT	CDH1__NM_004360	159	127	190	175
CDH1__NM_004360_TGTAATCCCAATACTCTGGG	CDH1__NM_004360	3	0	6	0
CDH1__NM_004360_TGTATTCAGCGTGACTTTGG	CDH1__NM_004360	274	318	275	347
CDH1__NM_004360_TTACAGTCAAAAGGCCTCTA	CDH1__NM_004360	124	239	89	73
CDH10__NM_006727_ATCTTGGTCTGAATGTAGCT	CDH10__NM_006727	159	273	230	356
CDH10__NM_006727_ATGCCACAAGGCGAATTGAT	CDH10__NM_006727	219	160	170	170
CDH10__NM_006727_ATTCTCCATCGTCAAAAACG	CDH10__NM_006727	170	167	141	220
CDH10__NM_006727_CACAAGGCGAATTGATAGGG	CDH10__NM_006727	29	45	15	15
CDH10__NM_006727_CTCCAGAAATAATGTTCAGA	CDH10__NM_006727	320	491	294	388
CDH10__NM_006727_GGATCTGATTATCAGTACGT	CDH10__NM_006727	298	401	393	414
CDH10__NM_006727_GTTCCCGAAATGTCTGTTGT	CDH10__NM_006727	738	795	672	745
CDH10__NM_006727_TCCATCGTCAAAAACGTGGT	CDH10__NM_006727	358	371	414	542
CDH10__NM_006727_TCTTGGTCTGAATGTAGCTA	CDH10__NM_006727	143	121	110	145
CDH10__NM_006727_TGATTATCAGTACGTAGGCA	CDH10__NM_006727	708	714	639	826
CDH10__NM_006727_TGCCACAAGGCGAATTGATA	CDH10__NM_006727	119	119	98	140
CDH10__NM_006727_TTGTGCGCGTAGAGTATAAA	CDH10__NM_006727	513	560	540	526
CDH11__NM_001797_ACAGCGCTCCAAGCGTGGCT	CDH11__NM_001797	257	173	293	289
CDH11__NM_001797_ACTCCTCTATCACGAAGAAC	CDH11__NM_001797	323	284	367	450
CDH11__NM_001797_CATTAAATACATTCTCTCAG	CDH11__NM_001797	481	296	567	568
CDH11__NM_001797_CCTGAGCCATCAACGTGTAC	CDH11__NM_001797	15	11	16	43
CDH11__NM_001797_CGTGCCTGAGAGGTCCAATG	CDH11__NM_001797	960	1214	1413	1333
CDH11__NM_001797_CTCTTCTCGATCCAACGTCT	CDH11__NM_001797	192	98	187	267
CDH11__NM_001797_CTGAGCCATCAACGTGTACT	CDH11__NM_001797	56	21	41	42
CDH11__NM_001797_CTTCATTCAGATATTGACTC	CDH11__NM_001797	61	144	92	168
CDH11__NM_001797_GTGCTACAGCGCTCCAAGCG	CDH11__NM_001797	171	176	231	208
CDH11__NM_001797_TGGCATGATAGGTCTCGTGC	CDH11__NM_001797	16	15	69	87
CDH11__NM_001797_TTGACAATGAATTCCGACGG	CDH11__NM_001797	463	250	442	354
CDH11__NM_001797_TTTGTGATTGATGACAAATC	CDH11__NM_001797	25	27	47	57
CDH12__NM_004061_AAGAATGCTGTAAACGACTC	CDH12__NM_004061	146	137	145	172
CDH12__NM_004061_ATTACCTTTCCCACATACTG	CDH12__NM_004061	173	82	242	177
CDH12__NM_004061_CATTTCCAACGTGTTAAACG	CDH12__NM_004061	191	295	267	316
CDH12__NM_004061_CCAACGTGTTAAACGTGGCT	CDH12__NM_004061	295	472	367	374
CDH12__NM_004061_CCCAGCCACGTTTAACACGT	CDH12__NM_004061	1173	2067	1390	2130
CDH12__NM_004061_CTCCATTCCGACTAGACAA	CDH12__NM_004061	297	294	314	222
CDH12__NM_004061_TCCAACGTGTTAAACGTGGC	CDH12__NM_004061	268	236	164	239
CDH12__NM_004061_TCTGGAACAGTAGCAACATA	CDH12__NM_004061	182	208	188	206
CDH12__NM_004061_TGAGCACGAAGAGTGTAGAA	CDH12__NM_004061	398	193	371	187
CDH12__NM_004061_TGGAAATGTGACCGTTGTCC	CDH12__NM_004061	202	159	177	166
CDH12__NM_004061_TGGCACTGTTTCCATAGGTC	CDH12__NM_004061	21	6	6	9
CDH12__NM_004061_TTTGTGCTGGAAGAATACGT	CDH12__NM_004061	762	735	820	707
CDH13__NM_001220488_AAACTCTGTTCGTCCATGCA	CDH13__NM_001220488	683	653	921	1371
CDH13__NM_001220488_AAGACAAAAGAGGTCCATTG	CDH13__NM_001220488	357	436	369	325
CDH13__NM_001220488_CAGAACTCGTGATTGTCGGG	CDH13__NM_001220488	1226	815	1351	1088
CDH13__NM_001220488_CAGCCTTTCCCAAGAGATGT	CDH13__NM_001220488	674	548	767	823
CDH13__NM_001220488_CGCCGCCGGGGCTCTTCTAGT	CDH13__NM_001220488	108	103	57	92
CDH13__NM_001220488_GACCCCCCATGCGGAAGATA	CDH13__NM_001220488	550	782	767	1127
CDH13__NM_001220488_GCAGAACTCGTGATTGTCGG	CDH13__NM_001220488	1933	2899	2215	2893
CDH13__NM_001220488_GGCAGAACTCGTGATTGTCG	CDH13__NM_001220488	1820	1967	2025	2996
CDH13__NM_001220488_GTGACCTTCAGTGACTGTAA	CDH13__NM_001220488	204	208	195	132
CDH13__NM_001220488_TACTTCAAGGTGAACAGCGA	CDH13__NM_001220488	61	37	92	64
CDH13__NM_001220488_TCAGCCAGCTGAATTCATTG	CDH13__NM_001220488	445	624	584	752
CDH13__NM_001220488_TGGCAGAACTCGTGATTGTC	CDH13__NM_001220488	100	187	92	130
CDH13__NM_001220489_CAGGGAGCGTCTCCGTGACA	CDH13__NM_001220489	614	422	837	652
CDH13__NM_001220489_TCAGAATCAATGAGAACACA	CDH13__NM_001220489	240	393	276	281
CDH13__NM_001220489_TCTGTCCAAGGTCCGTGTCA	CDH13__NM_001220489	801	806	923	868
CDH13__NM_001220490_AGGAGGGCATTATCGGTGGC	CDH13__NM_001220490	599	724	723	827
CDH13__NM_001220490_ATACCGCAGGAGGGCATTAT	CDH13__NM_001220490	1157	1104	1230	1153
CDH13__NM_001220490_ATCGAGGCTCAAGATATGGC	CDH13__NM_001220490	368	309	448	481
CDH13__NM_001220490_CAAGTATGAACTGATCATCG	CDH13__NM_001220490	1160	983	1339	1291
CDH13__NM_001220490_CCGCAGGAGGGCATTATCGG	CDH13__NM_001220490	1729	1733	1972	1892
CDH13__NM_001220490_GACGGATATTATACCGCAGG	CDH13__NM_001220490	362	344	347	342
CDH13__NM_001220490_GATCATCGAGGCTCAAGATA	CDH13__NM_001220490	272	187	257	325
CDH13__NM_001220490_GCTGACGGATATTATACCGC	CDH13__NM_001220490	827	570	994	768
CDH13__NM_001220490_GGAGGGCATTATCGGTGGCT	CDH13__NM_001220490	744	916	886	1458
CDH13__NM_001220490_GGATCGATGTAGAACATGTT	CDH13__NM_001220490	620	710	568	710
CDH13__NM_001220490_TCGATGATCAGTTCATACTT	CDH13__NM_001220490	443	524	494	448
CDH13__NM_001220490_TGGGTGACCCTTCCATGACG	CDH13__NM_001220490	171	208	161	214
CDH13__NM_001220491_AAGAGAAGTTCGCTGTTAAT	CDH13__NM_001220491	752	496	769	810
CDH15__NM_004933_AATGGACAGAACACCCTCGT	CDH15__NM_004933	676	441	801	743
CDH15__NM_004933_AGATCCGCACAGTGCAAGTG	CDH15__NM_004933	751	790	793	700
CDH15__NM_004933_CAGCGCTGCGTTGTCCGTCT	CDH15__NM_004933	1673	1783	1932	2051
CDH15__NM_004933_GAACACCCTCGTTGGTCTTG	CDH15__NM_004933	792	967	786	1009
CDH15__NM_004933_GAGCCTCTGCCTGTCTTTGG	CDH15__NM_004933	1171	882	1491	1256
CDH15__NM_004933_GGGGGTAGGGGAGACGCTTG	CDH15__NM_004933	315	164	393	259
CDH15__NM_004933_GGTTCTCGGATACGCTGATC	CDH15__NM_004933	46	13	73	51
CDH15__NM_004933_GTTCTCGGATACGCTGATCG	CDH15__NM_004933	182	172	196	224
CDH15__NM_004933_TCCTGGAAGGCGACCCCGAT	CDH15__NM_004933	296	586	544	721
CDH15__NM_004933_TCTCTATCGACAAGTTCACA	CDH15__NM_004933	212	196	242	303
CDH15__NM_004933_TGGTTCTCGGATACGCTGAT	CDH15__NM_004933	0	0	0	0
CDH15__NM_004933_TTCTCTATCGACAAGTTCAC	CDH15__NM_004933	0	0	0	0
CDH16__NM_001204744_AACTGAGGGCCCATTTGCTA	CDH16__NM_001204744	588	821	790	654
CDH16__NM_001204744_AAGGCCAGATCGTGCTGTCA	CDH16__NM_001204744	1389	1235	1545	1294
CDH16__NM_001204744_AATCTGGATCCATAGCAAAT	CDH16__NM_001204744	88	96	137	67
CDH16__NM_001204744_CTCACCTGGGCCATGTGGTG	CDH16__NM_001204744	47	45	57	42
CDH16__NM_001204744_GAAGGCCAGATCGTGCTGTC	CDH16__NM_001204744	738	743	894	1030
CDH16__NM_001204744_GCAGGATGGACATGTCTTGT	CDH16__NM_001204744	1710	1474	1562	1723
CDH16__NM_001204744_GGCCGCATAGTCCTCGCCAT	CDH16__NM_001204744	1631	1532	1676	1604
CDH16__NM_001204744_GGGCCATGTGGTGCGGGTAT	CDH16__NM_001204744	1329	1357	1261	1218
CDH16__NM_001204744_GGGTCACGGGGAGGGCAGAT	CDH16__NM_001204744	361	372	389	354
CDH16__NM_001204744_TCCCTCTGCATTCACTTCAA	CDH16__NM_001204744	622	675	853	991
CDH16__NM_001204744_TCTTCCTTGAGGCTTCAGAC	CDH16__NM_001204744	266	267	301	244
CDH16__NM_001204744_TGCAGGATGGACATGTCTTG	CDH16__NM_001204744	476	247	602	376
CDH16__NM_001204746_AGCTCAGGGATGCTGACTGT	CDH16__NM_001204746	1614	1259	1711	1201
CDH17__NM_001144663_ACCTGGAATATAATTTGACT	CDH17__NM_001144663	971	1226	1040	1273
CDH17__NM_001144663_AGGAGGATTGGCCTTAAACT	CDH17__NM_001144663	414	296	494	409
CDH17__NM_001144663_ATATTTGTGATAGAACGGGA	CDH17__NM_001144663	71	122	99	73
CDH17__NM_001144663_CATATTTGTGATAGAACGGG	CDH17__NM_001144663	279	338	442	379
CDH17__NM_001144663_CGCTAATGGAATTATAGTGG	CDH17__NM_001144663	436	358	657	482
CDH17__NM_001144663_CTATAATTCCATTAGCGTCC	CDH17__NM_001144663	137	139	148	282
CDH17__NM_001144663_GCTAATGGAATTATAGTGGA	CDH17__NM_001144663	709	924	685	686
CDH17__NM_001144663_GGACGCTAATGGAATTATAG	CDH17__NM_001144663	321	462	370	296
CDH17__NM_001144663_GGAGGATTGGCCTTAAACTG	CDH17__NM_001144663	249	165	338	234
CDH17__NM_001144663_GGCAACTGGATATGGCCAAG	CDH17__NM_001144663	535	689	571	499
CDH17__NM_001144663_GTGTCTTCTTATGCTTTATT	CDH17__NM_001144663	140	198	226	229
CDH17__NM_001144663_GTTGCAGCCCTGGACGCTAA	CDH17__NM_001144663	854	1034	922	841
CDH18__NM_001167667_AACTCGGATTCAGGCTCAAG	CDH18__NM_001167667	416	456	497	609
CDH18__NM_001167667_AAGTCCATCATCGTCCCAAA	CDH18__NM_001167667	343	401	452	487
CDH18__NM_001167667_AGATCCTCAGTATGTTGGAA	CDH18__NM_001167667	312	405	415	516
CDH18__NM_001167667_AGTCCATCATCGTCCCAAAA	CDH18__NM_001167667	216	221	250	424
CDH18__NM_001167667_CTACCTATGGAAACAGCGCT	CDH18__NM_001167667	431	676	496	861
CDH18__NM_001167667_GTCCATCATCGTCCCAAAAG	CDH18__NM_001167667	48	18	75	61
CDH18__NM_001167667_TACCTATGGAAACAGCGCTC	CDH18__NM_001167667	26	42	15	7
CDH18__NM_001167667_TAGAACGGCCTTACATAACA	CDH18__NM_001167667	458	468	625	735
CDH18__NM_001167667_TGTTGAGTGGATATCACCCG	CDH18__NM_001167667	293	296	270	310
CDH18__NM_001167667_TTCTCTGTCCATGTTATGTA	CDH18__NM_001167667	0	0	0	0
CDH18__NM_001167667_TTGATGATGAACTCGGATTC	CDH18__NM_001167667	131	167	161	280
CDH18__NM_001167667_TTTATCATTGACGATACCAC	CDH18__NM_001167667	15	67	72	71
CDH19__NM_001271028_ATTCCTAGATGAACCTTATG	CDH19__NM_001271028	300	184	206	201
CDH19__NM_001271028_ATTTGAGAGTGAAGCGTGGC	CDH19__NM_001271028	131	94	110	249
CDH19__NM_001271028_CAGCTCCCAAAAGCTTGTAC	CDH19__NM_001271028	264	317	347	367
CDH19__NM_001271028_CAGGTAATAGACATCGCTAC	CDH19__NM_001271028	668	587	762	906
CDH19__NM_001271028_CTAAGATCTGATTTAGACAA	CDH19__NM_001271028	121	128	151	156
CDH19__NM_001271028_CTGGGCTCTTAAGATGTAGA	CDH19__NM_001271028	22	14	15	37
CDH19__NM_001271028_TAATAGACATCGCTACTGGA	CDH19__NM_001271028	247	230	262	324
CDH19__NM_001271028_TCTGGTACAATGGCCTCATA	CDH19__NM_001271028	374	225	511	510
CDH19__NM_001271028_TGGAGACATCTCTGGTACAA	CDH19__NM_001271028	117	74	108	148
CDH19__NM_001271028_TGGCCTCATAAGGTTCATCT	CDH19__NM_001271028	59	41	71	78
CDH19__NM_001271028_TTAGGAACATTAGTTATCC	CDH19__NM_001271028	21	69	30	37
CDH19__NM_001271028_TTTCCTCTGGTACAAAAAAT	CDH19__NM_001271028	16	9	21	70
CDH2__NM_001792_ACCGACAGCTGACCCGAGAT	CDH2__NM_001792	2042	2024	2145	2254
CDH2__NM_001792_CCTGAAGCCAACCTTAACTG	CDH2__NM_001792	29	56	29	31
CDH2__NM_001792_CGCTCCCGCTATCCGGCACA	CDH2__NM_001792	1078	1344	1225	1377
CDH2__NM_001792_CTTACCCTGACAAGCTCTTG	CDH2__NM_001792	98	71	73	73
CDH2__NM_001792_GAAATCGCATTATGCAAGAC	CDH2__NM_001792	597	701	566	806
CDH2__NM_001792_GACCCAGGAAAAGTGGCAAG	CDH2__NM_001792	411	334	435	440
CDH2__NM_001792_GCACATGCAGTAGATATTAA	CDH2__NM_001792	443	507	495	780
CDH2__NM_001792_GCCTCCATGTGCCGGATAGC	CDH2__NM_001792	366	399	459	475
CDH2__NM_001792_GGGCTATCTGCTCGCGATCC	CDH2__NM_001792	475	800	516	645
CDH2__NM_001792_TCCGCAGCGCTCCCGCTATC	CDH2__NM_001792	1066	937	1157	1228
CDH2__NM_001792_TCTCTATCAGACCTGATCTG	CDH2__NM_001792	667	443	613	668
CDH2__NM_001792_TTACAGTGCAGTCTTATCGA	CDH2__NM_001792	433	386	399	429
CDH20__NM_031891_AGCTTCATTCAGATATGGAC	CDH20__NM_031891	304	462	391	397
CDH20__NM_031891_CATTCAGAGGCTCGACCGAG	CDH20__NM_031891	568	506	576	790
CDH20__NM_031891_CCGAGAGAACGGTGGTCGTA	CDH20__NM_031891	716	901	651	775
CDH20__NM_031891_CGACCCTTTGTATGTCGGCA	CDH20__NM_031891	889	1104	980	1303
CDH20__NM_031891_CTTACCTTGCCGACATACAA	CDH20__NM_031891	216	173	208	185
CDH20__NM_031891_CTTCATTCAGATATGGACAG	CDH20__NM_031891	742	736	812	1139
CDH20__NM_031891_CTTGCCGACATACAAAGGGT	CDH20__NM_031891	1358	1416	1296	1290
CDH20__NM_031891_GAATGAGCAATGCAAAGAAC	CDH20__NM_031891	311	227	266	271
CDH20__NM_031891_TCGGACACCCCAACACCACA	CDH20__NM_031891	357	459	345	478
CDH20__NM_031891_TCGTTCGGAAGAGTACACT	CDH20__NM_031891	110	120	166	194
CDH20__NM_031891_TTACCTTGCCGACATACAAA	CDH20__NM_031891	352	279	397	393
CDH20__NM_031891_TTTACCATCGACGACACCAC	CDH20__NM_031891	268	284	266	267
CDH22__NM_021248_ACACCCAGCCGCGTTTGACG	CDH22__NM_021248	2509	2591	2875	2649
CDH22__NM_021248_CCAGCCGCGTTTGACGCGGC	CDH22__NM_021248	0	0	0	0
CDH22__NM_021248_CCCAGCACCCTCGCCTGAGA	CDH22__NM_021248	823	1093	869	1087
CDH22__NM_021248_CCCGTCACCCTCGTCTGAGT	CDH22__NM_021248	4805	5586	5220	5772
CDH22__NM_021248_CCGACTCGGGCTCCAGTAGG	CDH22__NM_021248	811	820	905	1057
CDH22__NM_021248_CGGCCGCGTCAAACGCGGCT	CDH22__NM_021248	0	0	0	0
CDH22__NM_021248_CTTGATGATGAACTCCGACT	CDH22__NM_021248	775	755	822	932
CDH22__NM_021248_GGAGCCGGCCGCGTCAAACG	CDH22__NM_021248	1561	1491	1657	1973
CDH22__NM_021248_GGCACGGAGCCCCTGTATGT	CDH22__NM_021248	89	62	120	82
CDH22__NM_021248_GGCCGAGGCCCCGAAGGTAGG	CDH22__NM_021248	34	2	59	50
CDH22__NM_021248_GGTGGCGCGATCCCGAGGCT	CDH22__NM_021248	943	1338	1035	1368
CDH22__NM_021248_TCGCGCCACCAACCGCCTAC	CDH22__NM_021248	1128	981	1356	1530
CDH23__NM_001171930_ATCAGCGAGGACACGCCTGT	CDH23__NM_001171930	319	446	337	570
CDH23__NM_001171930_CAATGGCGAAGAATTGGGAG	CDH23__NM_001171930	651	763	738	893
CDH23__NM_001171930_CAGACCCCGACTTGGGGGCA	CDH23__NM_001171930	628	870	600	815
CDH23__NM_001171930_CCTGGTGTTTGGCGTGTCTG	CDH23__NM_001171930	1063	1329	1100	1387
CDH23__NM_001171930_GAATGCCACAGACCCCGACT	CDH23__NM_001171930	1842	2221	2113	2373
CDH23__NM_001171930_GCGTCCCCACTGGTGTATTC	CDH23__NM_001171930	909	1245	991	1134
CDH23__NM_001171930_GGTGGCAACATGGCGCCCCA	CDH23__NM_001171930	58	54	72	81
CDH23__NM_001171930_GTCAATGGCGAAGAATTGGG	CDH23__NM_001171930	1241	1010	1310	1412
CDH23__NM_001171930_GTGGCATTCACATGAAGAT	CDH23__NM_001171930	41	80	69	122
CDH23__NM_001171930_TAGGGCTGATTGTGAAATGT	CDH23__NM_001171930	616	748	678	636
CDH23__NM_001171930_TCAGGGATGCGGACGCTGTA	CDH23__NM_001171930	341	402	381	341
CDH23__NM_001171930_TCTTCTGTGACCCAGTTGC	CDH23__NM_001171930	7	29	8	22
CDH23__NM_001171933_AACCAGCTCCCGATTCATGG	CDH23__NM_001171933	648	591	869	660
CDH23__NM_001171933_ACCCTGACAAGGGCCTTAAT	CDH23__NM_001171933	215	208	195	290
CDH23__NM_001171933_AGAGCGCTCTGGTAGGTCGC	CDH23__NM_001171933	850	1229	1000	905
CDH23__NM_001171933_CAACCAGCTCCCGATTCATG	CDH23__NM_001171933	2435	1890	2815	2244
CDH23__NM_001171933_CAAGCTGACTGTCAACGTCC	CDH23__NM_001171933	879	736	853	950
CDH23__NM_001171933_CAGGATCCGCTCCATGTAGT	CDH23__NM_001171933	3396	3180	3401	3688
CDH23__NM_001171933_CGCTCTGTCAGTGTGCCAAA	CDH23__NM_001171933	254	380	371	480
CDH23__NM_001171933_GATCCCAAAGGGCTTGAACT	CDH23__NM_001171933	610	567	715	701
CDH23__NM_001171933_GGCAACCAGCTCCCGATTCA	CDH23__NM_001171933	1257	1540	1432	1922
CDH23__NM_001171933_TGACAAGGGCCTTAATGGGC	CDH23__NM_001171933	312	398	326	265
CDH23__NM_001171933_TGGTGAAGTCCCCCATGAAT	CDH23__NM_001171933	263	97	252	306
CDH23__NM_001171933_TGTCAGTGTGCCAAATGGTA	CDH23__NM_001171933	226	332	244	292
CDH23__NM_001171935_AGCTCCAGGTTCCGACAGAA	CDH23__NM_001171935	119	76	135	122
CDH23__NM_001171935_ATGGTGGGAGCATGCACCAC	CDH23__NM_001171935	233	287	271	281
CDH23__NM_001171935_CAAACTTCGCGACGTGATCA	CDH23__NM_001171935	602	898	640	813
CDH23__NM_001171935_CACGTCGCGAAGTTTGTGCA	CDH23__NM_001171935	1463	1686	1671	1997
CDH23__NM_001171935_CAGCTCCAGGTTCCGACAGA	CDH23__NM_001171935	1894	1711	2122	2180
CDH23__NM_001171935_CCGTGCTGCCGTGCACCACA	CDH23__NM_001171935	630	586	699	750
CDH23__NM_001171935_CTGGTTCCAGCAGGATCCTA	CDH23__NM_001171935	2	1	8	0
CDH23__NM_001171935_TATGACAACATTGCCAAGCT	CDH23__NM_001171935	184	160	235	309
CDH23__NM_001171935_TGTGGCTGGATCCCTTCTGT	CDH23__NM_001171935	2136	1921	1997	1992
CDH23__NM_001171935_TTCCAGCAGGATCCTATGGT	CDH23__NM_001171935	196	277	228	286
CDH23__NM_001171935_TTGGCCCCTCACGAATGATC	CDH23__NM_001171935	372	239	349	288
CDH23__NM_001171935_TTTGTGCAGGGGTGTGGATT	CDH23__NM_001171935	774	907	827	966
CDH24__NM_022478_AAAGGCGCAATATGTGCTAC	CDH24__NM_022478	313	272	423	482
CDH24__NM_022478_AGCTGCACTCGGATGTTGAC	CDH24__NM_022478	1020	1224	1090	1217
CDH24__NM_022478_ATGCTGATGACCCCAGCTAT	CDH24__NM_022478	320	243	334	335
CDH24__NM_022478_CACCAAGTACCTGTTGACCG	CDH24__NM_022478	1603	1632	1758	1981
CDH24__NM_022478_CGCACCAAGTACCTGTTGAC	CDH24__NM_022478	1157	1252	1242	1438
CDH24__NM_022478_CTCACCAGGCCCCACATGTT	CDH24__NM_022478	1934	1973	2281	1867
CDH24__NM_022478_CTCCCCGGTCAACAGGTACT	CDH24__NM_022478	267	410	397	449
CDH24__NM_022478_CTGCCCCCTCCCCGGTCAAC	CDH24__NM_022478	1255	1246	1259	1444
CDH24__NM_022478_CTGGGGCTGCATGGGGCGTC	CDH24__NM_022478	341	346	406	386
CDH24__NM_022478_GGCCTCATCAATCACAAATA	CDH24__NM_022478	227	320	304	253
CDH24__NM_022478_GGTAACATGAAATATTGCCTG	CDH24__NM_022478	479	683	590	753
CDH24__NM_022478_TTTGTCATTGAGGAAATGC	CDH24__NM_022478	23	95	59	69
CDH26__NM_021810_AGGACCCACGCAGGGAGTTA	CDH26__NM_021810	217	329	225	223
CDH26__NM_021810_CAGACATGGTCAGATGTTGA	CDH26__NM_021810	517	692	678	714
CDH26__NM_021810_CAGGAGTTGTCTGTGCATTG	CDH26__NM_021810	1148	1252	1214	1479
CDH26__NM_021810_CCTACCTCACGTCTACAGCG	CDH26__NM_021810	805	694	909	903
CDH26__NM_021810_CCTCATGAAACCTCTGATAT	CDH26__NM_021810	1169	1037	1412	1249
CDH26__NM_021810_CCTCGCTGTAGACGTGAGGT	CDH26__NM_021810	3929	3877	4119	4988
CDH26__NM_021810_CTCTCTGGCCAGCTTGGAAC	CDH26__NM_021810	325	289	315	540
CDH26__NM_021810_GATGGCAGAGACATTGAATC	CDH26__NM_021810	585	520	736	664
CDH26__NM_021810_GGAGTTGCAACCTGATTTGC	CDH26__NM_021810	1149	1340	1284	1588
CDH26__NM_021810_GTTACCTTAACTCCCTGCGT	CDH26__NM_021810	362	291	456	400
CDH26__NM_021810_TCCTCATGAAACCTCTGATA	CDH26__NM_021810	927	646	1197	1011
CDH26__NM_021810_TTCAAAAGCGACTCCGTTTG	CDH26__NM_021810	39	114	94	83
CDH26__NM_177980_AAGACATATTATTGAACAGC	CDH26__NM_177980	185	296	191	347
CDH26__NM_177980_AAGATGGGTTATCACCACCT	CDH26__NM_177980	336	177	327	353
CDH26__NM_177980_ATATGGCCATGAGATCCGGG	CDH26__NM_177980	608	561	534	483
CDH26__NM_177980_CCTTACCTCACCAATGAGTT	CDH26__NM_177980	1116	1419	1397	1931
CDH26__NM_177980_CTTCTCTTGGATCGCCGTAG	CDH26__NM_177980	191	149	220	170
CDH26__NM_177980_GGTGTTGTGGAGCGCTCAAC	CDH26__NM_177980	441	465	494	573
CDH26__NM_177980_GGTGTCATTTCTCGATCGAC	CDH26__NM_177980	560	789	658	686
CDH26__NM_177980_GTGGATGAATATCCAGAGAT	CDH26__NM_177980	125	157	176	128
CDH26__NM_177980_GTGTCATTTCTCGATCGACA	CDH26__NM_177980	156	235	253	309
CDH26__NM_177980_TAGAAGATCATGAGAACGGA	CDH26__NM_177980	71	57	76	114
CDH26__NM_177980_TGATCTTACTAAGCAAACAA	CDH26__NM_177980	57	69	93	89
CDH26__NM_177980_TTCCGGGTTGATCGCCTTAG	CDH26__NM_177980	80	142	81	62
CDH3__NM_001793_AGGGCAGCCCATGAATACTG	CDH3__NM_001793	793	690	637	643
CDH3__NM_001793_CCACAAGCCCAAGTTTACCC	CDH3__NM_001793	422	399	414	430
CDH3__NM_001793_CCTAAGACACTCCCTCGGA	CDH3__NM_001793	27	24	84	85
CDH3__NM_001793_CTTCCCCCAGAGACTGAATC	CDH3__NM_001793	539	791	794	1049
CDH3__NM_001793_CTTCCGAGGGAGTGTCTTAG	CDH3__NM_001793	240	188	241	245
CDH3__NM_001793_GAAGGTGTCCTGGGTAAACT	CDH3__NM_001793	421	498	512	689
CDH3__NM_001793_GATGACTTCACTGTGCGGAA	CDH3__NM_001793	475	296	480	665
CDH3__NM_001793_GCGGAATGGCGAGACAGTCC	CDH3__NM_001793	422	417	491	430
CDH3__NM_001793_GGGTGTCTTCGCTGTAGAGA	CDH3__NM_001793	323	313	295	324
CDH3__NM_001793_GTGTCTTCGTAGATACGTTC	CDH3__NM_001793	415	485	466	465
CDH3__NM_001793_TTCCGAGGGAGTGTCTTAGA	CDH3__NM_001793	136	114	177	202
CDH3__NM_001793_TTCGTAAGATACGTTTGGAT	CDH3__NM_001793	179	185	191	313
CDH4__NM_001252338_AGAGTGCGGGGACGAGGTCT	CDH4__NM_001252338	947	1252	980	1085
CDH4__NM_001252338_AGCAGAGAAATGGGACGCCG	CDH4__NM_001252338	675	611	655	715
CDH4__NM_001252338_ATGGGACAGTCTTCGCCACC	CDH4__NM_001252338	561	487	699	532
CDH4__NM_001252338_CCCGTGATGCTGTACCGGAT	CDH4__NM_001252338	1478	927	1680	1603
CDH4__NM_001252338_CCGGCCGGATGTACGTCACA	CDH4__NM_001252338	2981	3223	3114	4040
CDH4__NM_001252338_CCGTGATGCTGTACCGGATG	CDH4__NM_001252338	519	681	579	748
CDH4__NM_001252338_CTTCCCGCAGCAGCTCGTGA	CDH4__NM_001252338	214	283	137	110
CDH4__NM_001252338_GCAGAAAGGAAAGAAGGTCG	CDH4__NM_001252338	149	310	255	244
CDH4__NM_001252338_GCCTCAGCCCGTTGGCGTTC	CDH4__NM_001252338	709	912	789	1031
CDH4__NM_001252338_GCGTGCTCCTCCCGGTCCAT	CDH4__NM_001252338	2545	2376	2850	2753
CDH4__NM_001252338_GGACGCCGTGGTGCGGTTGC	CDH4__NM_001252338	923	1336	1111	1482
CDH4__NM_001252338_TGAGGCGGCGCAAACGGGAC	CDH4__NM_001252338	307	386	385	484
CDH4__NM_001794_AGTTGTAAGATCCTCATTAT	CDH4__NM_001794	419	183	448	364
CDH4__NM_001794_ATCTCCCAAAATATTCTAGA	CDH4__NM_001794	231	453	262	331
CDH4__NM_001794_GAAGGGGAAAAGCTACTTCA	CDH4__NM_001794	201	137	279	204
CDH4__NM_001794_GTTCTCTGAAGATGATTACA	CDH4__NM_001794	16	9	11	35
CDH4__NM_001794_TCTCCCAAAATATTCTAGAA	CDH4__NM_001794	372	477	397	416
CDH5__NM_001795_ACTGGCCTGTGTTCACGCAT	CDH5__NM_001795	1197	985	1483	1078
CDH5__NM_001795_ATGATGCTCCTCGCCACATC	CDH5__NM_001795	311	151	386	282
CDH5__NM_001795_CAAAGGAGAATATGTGGGCA	CDH5__NM_001795	981	1093	984	1397
CDH5__NM_001795_CAAGGACACTGGCGAAAACC	CDH5__NM_001795	143	238	238	210
CDH5__NM_001795_CCCATCATGTAGGCAAGGTA	CDH5__NM_001795	285	257	364	412
CDH5__NM_001795_CCTTACCTTGCCTACATGAT	CDH5__NM_001795	1124	957	1054	1113
CDH5__NM_001795_CGTGCCTGAAGACACCCGTG	CDH5__NM_001795	1003	984	1295	930
CDH5__NM_001795_GCCAAAAGAGAGATTGGATT	CDH5__NM_001795	1623	1968	1877	2099
CDH5__NM_001795_GTGCCTGAAGACACCCGTGT	CDH5__NM_001795	65	45	59	62
CDH5__NM_001795_GTGCCTGAGTCGTCGGCTGT	CDH5__NM_001795	1100	1028	1251	1045
CDH5__NM_001795_TATCACAATAACGAAAAGCT	CDH5__NM_001795	192	332	254	180
CDH5__NM_001795_TTCCACCACGATCTCATACC	CDH5__NM_001795	356	295	297	222
CDH6__NM_004932_AGCTGTCTTGATAATACCTG	CDH6__NM_004932	422	389	468	466
CDH6__NM_004932_AGGGATTATAACTGTCAAAA	CDH6__NM_004932	8	8	31	9
CDH6__NM_004932_AGTCAGTGTGATGTTCACGG	CDH6__NM_004932	646	1133	747	846
CDH6__NM_004932_ATGAGCTGAACCGTTCAAAA	CDH6__NM_004932	144	296	230	258
CDH6__NM_004932_CCAAGTGGTGATTCAAGCCA	CDH6__NM_004932	279	352	324	306
CDH6__NM_004932_CCACTAGTCCTCTTTGATAG	CDH6__NM_004932	1524	1531	1589	1711
CDH6__NM_004932_CTGGCTTTGATTCTGCCAAT	CDH6__NM_004932	482	466	373	654
CDH6__NM_004932_GATGCAGATGATCCAACATA	CDH6__NM_004932	53	54	51	46
CDH6__NM_004932_GGATCCGATTATCAGTATGT	CDH6__NM_004932	589	515	574	479
CDH6__NM_004932_GTTGTCTACAGTATTCTACA	CDH6__NM_004932	758	1055	887	843
CDH6__NM_004932_TCCCAGATAATCCTCCCATC	CDH6__NM_004932	815	919	877	849
CDH6__NM_004932_TGTTGTCCAAGTCACTGCGA	CDH6__NM_004932	125	127	102	153
CDH7__NM_004361_AATACATCTTGTCAGGCGAA	CDH7__NM_004361	95	150	111	156
CDH7__NM_004361_AGACCCCCTCTATGTAGGAA	CDH7__NM_004361	559	679	549	796
CDH7__NM_004361_ATACATCTTGTCAGGCGAAG	CDH7__NM_004361	207	287	135	333
CDH7__NM_004361_CCCTACCTTTCCTACATAGA	CDH7__NM_004361	830	858	936	961
CDH7__NM_004361_CCTACCTTTCCTACATAGAG	CDH7__NM_004361	433	419	556	753
CDH7__NM_004361_CTACCTTTCCTACATAGAGG	CDH7__NM_004361	291	323	313	331
CDH7__NM_004361_CTCGGGCTCCACGGGTTTGT	CDH7__NM_004361	1930	1704	2117	2161
CDH7__NM_004361_GACTCATCCCAGAAAAACAC	CDH7__NM_004361	308	352	330	480
CDH7__NM_004361_GAGACTGGATCGTGAGGAGC	CDH7__NM_004361	191	131	159	148
CDH7__NM_004361_TCATTATTGATGAGAACACT	CDH7__NM_004361	142	197	144	195
CDH7__NM_004361_TCCATCAAATACATCTTGTC	CDH7__NM_004361	1036	1028	928	982
CDH7__NM_004361_TTTGTGCTGGAGGAATACAT	CDH7__NM_004361	287	249	278	318
CDH8__NM_001796_AGTCCACTGCTTGAGCTGTT	CDH8__NM_001796	962	1222	1072	1213
CDH8__NM_001796_ATAAACTGGGTCATCAGCGT	CDH8__NM_001796	236	383	338	292
CDH8__NM_001796_CCCTTTGGAACTAAACAGTC	CDH8__NM_001796	320	301	324	448
CDH8__NM_001796_CGGAGCCATGTAAATGCAAG	CDH8__NM_001796	376	432	426	615
CDH8__NM_001796_CGGCCAACAAGAATCGGTTC	CDH8__NM_001796	31	40	43	27
CDH8__NM_001796_GACGCTGATGACCCAGTTTA	CDH8__NM_001796	681	705	887	903
CDH8__NM_001796_GGAGCCATGTAAATGCAAGG	CDH8__NM_001796	168	84	231	189
CDH8__NM_001796_TAACAGCTAACCAGCAGTGGAC	CDH8__NM_001796	339	223	397	388
CDH8__NM_001796_TATAAAAAGACTTGACCGGG	CDH8__NM_001796	238	487	276	216
CDH8__NM_001796_TCTGGCACAGTAGCATGATA	CDH8__NM_001796	394	356	466	466
CDH8__NM_001796_TTCTGGCACAGTAGCATGAT	CDH8__NM_001796	22	27	0	0
CDH8__NM_001796_TTGGAATCAAATGTTTGTCC	CDH8__NM_001796	271	257	273	222
CDH9__NM_016279_AATACATACTAACAGGAGAT	CDH9__NM_016279	80	104	132	174
CDH9__NM_016279_ATGCTACGTCGCACCAAGCG	CDH9__NM_016279	487	405	525	626
CDH9__NM_016279_CAATATGCTATAGACCACTT	CDH9__NM_016279	459	465	520	453
CDH9__NM_016279_GATGCAGATGACGCCAACTA	CDH9__NM_016279	1251	1428	1304	1451
CDH9__NM_016279_GCGGCAGGTGGAACCGGAAT	CDH9__NM_016279	647	936	734	1056
CDH9__NM_016279_GCGGGTCTGACAAAAGATGA	CDH9__NM_016279	223	437	303	299
CDH9__NM_016279_GGTACTGACACACAATATGT	CDH9__NM_016279	204	218	195	150
CDH9__NM_016279_GTCAACTGTATGGAACATAT	CDH9__NM_016279	539	493	552	865
CDH9__NM_016279_GTGGAATCAGTTCTTCTTAT	CDH9__NM_016279	182	147	139	198
CDH9__NM_016279_GTTCCTGAAATGTCTGGAGT	CDH9__NM_016279	166	284	158	177
CDH9__NM_016279_TGACACACAATATGTAGGCA	CDH9__NM_016279	345	670	505	673
CDH9__NM_016279_TTATCAAGCAAAAAGATAGC	CDH9__NM_016279	41	116	66	66
CDHR1__NM_001171971_AGAAAAGACGCTCTAGTGC	CDHR1__NM_001171971	358	429	436	491
CDHR1__NM_001171971_CACGGTGATGTAGTGGGTCC	CDHR1__NM_001171971	516	409	518	586
CDHR1__NM_001171971_CACTTCTTCGACAACGGGGT	CDHR1__NM_001171971	187	341	295	270
CDHR1__NM_001171971_CCCGCACTTCTTCGACAACG	CDHR1__NM_001171971	1570	2276	1872	2406
CDHR1__NM_001171971_CGGCAGCACCAACGGAAACA	CDHR1__NM_001171971	398	384	372	450
CDHR1__NM_001171971_CTCACGTATACAACCCTGAAT	CDHR1__NM_001171971	771	494	951	876
CDHR1__NM_001171971_CTGTGGCGGTCCACGGCAAA	CDHR1__NM_001171971	480	644	530	584
CDHR1__NM_001171971_GAACAGAGCCATGTTTCCGT	CDHR1__NM_001171971	1601	1556	1909	1826
CDHR1__NM_001171971_GTGGCGGTCCACGGCAAATG	CDHR1__NM_001171971	279	105	215	164
CDHR1__NM_001171971_TAAGGCTCCTGGATGAACCT	CDHR1__NM_001171971	157	212	167	132
CDHR1__NM_001171971_TCGGGAACCAGGGCAACATA	CDHR1__NM_001171971	39	38	65	121
CDHR1__NM_001171971_TCTCACGTATACAACCCTGAA	CDHR1__NM_001171971	472	400	493	726
CDHR2__NM_001171976_ACAGCGAAGAAGTAGGCATT	CDHR2__NM_001171976	647	650	702	460
CDHR2__NM_001171976_AGACCCCATGTCTTTATCCA	CDHR2__NM_001171976	1233	1054	1121	1102
CDHR2__NM_001171976_ATTGGGGCCGCTCATCCCAT	CDHR2__NM_001171976	878	694	1118	884
CDHR2__NM_001171976_CAGCGAAGAAGTAGGCATTG	CDHR2__NM_001171976	883	748	791	619
CDHR2__NM_001171976_CCAGGCCTTCTGGTTGGTAG	CDHR2__NM_001171976	764	737	966	752
CDHR2__NM_001171976_CCGCTCATCCCATATAGGTCAG	CDHR2__NM_001171976	223	223	226	296
CDHR2__NM_001171976_CGCTGTCACTCCGAAAACTG	CDHR2__NM_001171976	848	1051	1136	1392
CDHR2__NM_001171976_CTGGCCGTGGATAAAGACAT	CDHR2__NM_001171976	656	770	829	769
CDHR2__NM_001171976_CTTCCTGCCCTCGTGGTGTC	CDHR2__NM_001171976	328	365	400	577
CDHR2__NM_001171976_TGGCCGTGGATAAAGACATG	CDHR2__NM_001171976	62	97	61	49
CDHR2__NM_001171976_TGGTGGAGAAAGCGGTGTTC	CDHR2__NM_001171976	21	15	19	27
CDHR2__NM_001171976_TTCGCTGTCACTCCGAAAAC	CDHR2__NM_001171976	119	120	85	165
CDHR3__NM_152750_ACCTCAGTTTCAAGGCAACT	CDHR3__NM_152750	664	933	772	990
CDHR3__NM_152750_AGCTTCAGAATGTCTGCTAA	CDHR3__NM_152750	410	626	546	577
CDHR3__NM_152750_AGGACTGTGTACACTCGTGT	CDHR3__NM_152750	1160	1494	1053	1077
CDHR3__NM_152750_AGGATCCTCCGCTGTGATAT	CDHR3__NM_152750	356	495	508	547
CDHR3__NM_152750_AGTGACTGGTACAATCCAAG	CDHR3__NM_152750	397	330	413	427
CDHR3__NM_152750_CTGTAGCAGGTAAGAGGATT	CDHR3__NM_152750	462	579	540	673
CDHR3__NM_152750_GGACTGTGTACACTCGTGTC	CDHR3__NM_152750	670	1015	710	750
CDHR3__NM_152750_GGATTTGAGTTGACTATCTG	CDHR3__NM_152750	111	160	106	162
CDHR3__NM_152750_GGGGATTTGAGTTGACTATC	CDHR3__NM_152750	146	191	175	156
CDHR3__NM_152750_TGGATTCATTTACCAGGTTG	CDHR3__NM_152750	15	42	20	20
CDHR3__NM_152750_TTGCTCTTTCTACTATGTAG	CDHR3__NM_152750	9	9	27	15
CDHR3__NM_152750_TTTAGGGTGAATTGGCTGTC	CDHR3__NM_152750	340	468	332	423
CDHR4__NM_001007540_ACCTGAACCACCTCACTACC	CDHR4__NM_001007540	1508	1448	1682	1657
CDHR4__NM_001007540_AGGTGGCAAGGGACCTATGT	CDHR4__NM_001007540	391	336	449	387
CDHR4__NM_001007540_CAAGCTGGGTGGGTTGAAGA	CDHR4__NM_001007540	134	87	149	184
CDHR4__NM_001007540_CAGAGACCACCGGAGCAAAG	CDHR4__NM_001007540	250	158	291	182
CDHR4__NM_001007540_CAGCCAACCTGGTCATTGGA	CDHR4__NM_001007540	1735	1720	1995	1817
CDHR4__NM_001007540_CCAAGGGATGGTGATAGTGA	CDHR4__NM_001007540	388	367	478	360
CDHR4__NM_001007540_CGTGTTCCTCTTTGCTCCGG	CDHR4__NM_001007540	4157	3077	4438	3379
CDHR4__NM_001007540_CTGAACCACCTCACTACCGG	CDHR4__NM_001007540	1440	489	1437	857
CDHR4__NM_001007540_CTTAGCCATATCCAGTGTGC	CDHR4__NM_001007540	631	985	818	981
CDHR4__NM_001007540_GCTCCGTGGAGTTCTAGGCC	CDHR4__NM_001007540	503	803	578	753
CDHR4__NM_001007540_GTGGGCTGGGCACCGGAGAC	CDHR4__NM_001007540	406	441	490	415
CDHR4__NM_001007540_TGTGGCAACCATGTGATGGA	CDHR4__NM_001007540	501	435	618	430
CDHR5__NM_001171968_CCCTGGATCCGAAATGCAAA	CDHR5__NM_001171968	650	713	710	876
CDHR5__NM_001171968_CCCTTTGCATTTCGGATCCA	CDHR5__NM_001171968	1594	1962	1875	2003
CDHR5__NM_001171968_CGAATTCCCCTTTAAGACCA	CDHR5__NM_001171968	501	301	585	650
CDHR5__NM_001171968_CGAGCAGCCCGGTGAACAGC	CDHR5__NM_001171968	1003	1044	854	1311
CDHR5__NM_001171968_CGGGACGGTTTACACTCACC	CDHR5__NM_001171968	879	1029	987	983
CDHR5__NM_001171968_CTGACACGAACACCCTTAGC	CDHR5__NM_001171968	680	656	637	586
CDHR5__NM_001171968_CTTATCTCCTTGGTCTTAAA	CDHR5__NM_001171968	65	258	101	130
CDHR5__NM_001171968_GCAGGCTGAGGACCGCGACA	CDHR5__NM_001171968	580	604	643	586
CDHR5__NM_001171968_GGGACGGTTTACACTCACCA	CDHR5__NM_001171968	620	818	742	677
CDHR5__NM_001171968_TCCCTGGATCCGAAATGCAA	CDHR5__NM_001171968	382	654	393	695
CDHR5__NM_001171968_TGACTCCTGATTACGAGGTG	CDHR5__NM_001171968	106	122	144	147
CDHR5__NM_001171968_TGGAGTTCACTTTCGTGTCC	CDHR5__NM_001171968	62	81	136	119
CDIP1__NM_001199054_ACCCGGCGGCTCATAGGGTG	CDIP1__NM_001199054	830	613	849	607
CDIP1__NM_001199054_CCCCCACCCTATGAGCCGCC	CDIP1__NM_001199054	4031	3887	4083	4111
CDIP1__NM_001199054_CCCTGGGGGGTAGTAGCCCA	CDIP1__NM_001199054	534	429	543	531
CDIP1__NM_001199054_CCTCCCCCTCCTTATCCTGG	CDIP1__NM_001199054	3363	2670	3864	2769
CDIP1__NM_001199054_GACCCGGCGGCTCATAGGGT	CDIP1__NM_001199054	1437	905	1492	971
CDIP1__NM_001199054_GCAGGGGGCAGTGGCATGCC	CDIP1__NM_001199054	1091	1323	1118	1549
CDIP1__NM_001199054_GGGGGGTAGTAGCCCATGGG	CDIP1__NM_001199054	564	592	627	770
CDIP1__NM_001199054_GGTGGGGGGCCAATGTCCGC	CDIP1__NM_001199054	915	873	1009	1083
CDIP1__NM_001199054_GTGCCATCTGCACTCATGTG	CDIP1__NM_001199054	402	438	389	521
CDIP1__NM_001199054_GTGGGGGGCCAATGTCCGCA	CDIP1__NM_001199054	310	434	357	333
CDIP1__NM_001199054_TGACCCGGCGGCTCATAGGG	CDIP1__NM_001199054	850	615	935	647
CDIP1__NM_001199054_TGGGGGGCCAATGTCCGCAG	CDIP1__NM_001199054	200	187	173	202
CDIP1__NM_001199055_ACCAAGATCTCCTACGAGAT	CDIP1__NM_001199055	1598	1470	1876	1548
CDIP1__NM_001199055_GGCTTGATGAATTTCGTGCT	CDIP1__NM_001199055	1643	2274	1833	2488
CDIP1__NM_001199056_GCGCTTGTACGTGTAGATGT	CDIP1__NM_001199056	1861	1996	2144	1970
CDIPT__NM_001286585_CACAAGATGATCGACTTGTC	CDIPT__NM_001286585	188	453	157	220
CDIPT__NM_001286585_CAGGGCAGCCATGTTGCGGG	CDIPT__NM_001286585	1287	18	1562	73
CDIPT__NM_001286585_CATCCAAACTCATGCTGATT	CDIPT__NM_001286585	1234	1567	1575	1748
CDIPT__NM_001286585_GCTGATGAGCGACTTCAGCA	CDIPT__NM_001286585	423	6	526	10
CDIPT__NM_001286585_TACCTGTTCCATTTCTCTGA	CDIPT__NM_001286585	578	667	670	691
CDIPT__NM_001286585_TCGGATCAACCACTACACCTCGA	CDIPT__NM_001286585	649	100	756	23
CDIPT__NM_001286585_TGCCTCGGACCACAGAACTT	CDIPT__NM_001286585	715	63	685	34
CDIPT__NM_001286585_TGCGGGCGGCCGTGATCAGG	CDIPT__NM_001286585	1934	81	2175	57
CDIPT__NM_001286585_TTCGGATCTACTACACCTCG	CDIPT__NM_001286585	302	322	369	236
CDIPT__NM_006319_AAATGATGGCGAAGACAATC	CDIPT__NM_006319	179	4	103	3
CDIPT__NM_006319_GCTGCTCGCGCTCTTAATCA	CDIPT__NM_006319	182	7	231	13
CDIPT__NM_006319_GGCCTGCTGGACGCTTTCGA	CDIPT__NM_006319	1431	70	1554	143
CDK1__NM_001170406_AAGGGTAGACACAAAACTAC	CDK1__NM_001170406	269	241	192	110
CDK1__NM_001170406_ACACAAAACTACAGGTCAAG	CDK1__NM_001170406	786	106	912	99
CDK1__NM_001170406_ATTTCCCGAATTGCAGTACT	CDK1__NM_001170406	429	99	480	94
CDK1__NM_001170406_CATGGATTCTTCACTTGTTA	CDK1__NM_001170406	793	25	911	59
CDK1__NM_001170406_GAAGAATCCATGTACTGACC	CDK1__NM_001170406	272	229	197	36
CDK1__NM_001170406_GGGTTCCTAGTACTGCAATT	CDK1__NM_001170406	158	228	194	174
CDK1__NM_001170406_TCTTCAGGATGTGCTTATGC	CDK1__NM_001170406	398	122	490	67
CDK1__NM_001170406_TTCGTCATCCAAATATAGTC	CDK1__NM_001170406	93	0	85	0
CDK1__NM_001786_ACTGAAGGAGTTGCTTATCT	CDK1__NM_001786	1126	2012	1678	2428
CDK1__NM_001786_ATCAAGGGCTGTCCGCAACA	CDK1__NM_001786	435	558	490	432
CDK1__NM_001786_ATGTTGTTAACTATACAACC	CDK1__NM_001786	83	132	99	87
CDK1__NM_001786_CCAGTTGACATTTGGAGTAT	CDK1__NM_001786	890	20	723	14
CDK1__NM_001786_GGATTTGGTATAAATAACTC	CDK1__NM_001786	17	3	27	10
CDK1__NM_001786_TATCAGAGTATATACACATG	CDK1__NM_001786	99	30	70	14
CDK1__NM_001786_TGACAAAGGAACAATTAAAC	CDK1__NM_001786	117	1	208	25
CDK1__NM_001786_TTAAATCATCTCAGTCCTTA	CDK1__NM_001786	179	223	191	220
CDK1__NM_001786_TTGGTCAGAGTAATAACTGA	CDK1__NM_001786	149	103	165	160
CDK1__NM_033379_GCTTGGATTTGCTCTCGGTA	CDK1__NM_033379	927	226	1162	452
CDK1__NM_033379_TTGCCAGAAATTCGTTTGGC	CDK1__NM_033379	440	663	466	487
CDK10__NM_001098533_ACCGGCCAGATGTTCTCACT	CDK10__NM_001098533	3712	2440	4164	3297
CDK10__NM_001098533_ACGCACCTGAGCCTCCGAGA	CDK10__NM_001098533	970	397	960	623
CDK10__NM_001098533_AGTAAAGCCAATGACCCCCA	CDK10__NM_001098533	417	390	334	448
CDK10__NM_001098533_AGTGAGAACATCTGGCCGGT	CDK10__NM_001098533	2058	1654	2403	1978
CDK10__NM_001098533_CCTGCGTCATCCGAACATCG	CDK10__NM_001098533	1512	1916	1607	1726
CDK10__NM_001098533_GCACGATCAAGTCGATCTGG	CDK10__NM_001098533	1555	1367	1933	1575
CDK10__NM_001098533_GCACGCCCAGTGAGAACATC	CDK10__NM_001098533	495	561	600	632
CDK10__NM_001098533_GCTCACAGTAACCCATCACC	CDK10__NM_001098533	1614	1447	1833	1549
CDK10__NM_001098533_GGAACTTCATTATCCACAGG	CDK10__NM_001098533	291	332	360	501
CDK10__NM_001098533_TGAAGTTCCTGTGCAGATAC	CDK10__NM_001098533	168	192	253	326
CDK10__NM_001098533_TGTCTTCACACAACCCTTGT	CDK10__NM_001098533	399	223	420	304
CDK10__NM_001098533_TTGGGGGTCATTGGCTTTAC	CDK10__NM_001098533	354	198	309	256
CDK10__NM_052988_CCGTCTGAAGTGTATTCGTA	CDK10__NM_052988	1223	1132	1349	1378
CDK10__NM_052988_CTGAAGTGTATTCGTAAGGA	CDK10__NM_052988	1135	901	1505	1277
CDK10__NM_052988_GAAGCTGAACCGCATTGGAG	CDK10__NM_052988	477	343	574	541
CDK10__NM_052988_GTGCGACAATCTCATCTGTC	CDK10__NM_052988	876	672	972	999
CDK10__NM_052988_TCTGAAGTGTATTCGTAAGG	CDK10__NM_052988	637	553	847	597
CDK10__NM_052988_TGAGATTGTCGCACTGAAGA	CDK10__NM_052988	376	554	518	529
CDK10__NM_052988_TGGGTGTCCCGGGCCCGATC	CDK10__NM_052988	183	155	200	180
CDK11A__NM_024011_GATCGATTTCCGAATTCCCG	CDK11A__NM_024011	602	719	586	669
CDK11A__NM_024011_TCAGATCAACAAAGTGTTCA	CDK11A__NM_024011	24	116	35	16
CDK11B__NM_001291345_AAGAAAGAGAGCACGAACGT	CDK11B__NM_001291345	159	16	135	32
CDK11B__NM_001291345_AAGAACAGGATAAAGCTCGC	CDK11B__NM_001291345	128	3	221	50
CDK11B__NM_001291345_AGAACAGGATAAAGCTCGCC	CDK11B__NM_001291345	107	19	167	38
CDK11B__NM_001291345_AGAAGAGAAGGGAAATGGCA	CDK11B__NM_001291345	211	75	320	233
CDK11B__NM_001291345_ATCTCCCTCAGCGACGTGAT	CDK11B__NM_001291345	414	268	581	487
CDK11B__NM_001291345_GGATAAAGCTCGCCGGGAAT	CDK11B__NM_001291345	382	130	431	140
CDK11B__NM_001291345_GTCAGTGGGTTGCATCTTCG	CDK11B__NM_001291345	937	296	1129	635
CDK11B__NM_001291345_TCAGGAAAAGAAACGAAGGA	CDK11B__NM_001291345	113	66	165	196
CDK11B__NM_001291345_TCTCCCTCAGCGACGTGATC	CDK11B__NM_001291345	74	32	77	99
CDK11B__NM_001291345_TGAGTCTTCTCTTCTATACG	CDK11B__NM_001291345	196	47	141	33
CDK11B__NM_001291345_TTCTTCAGGAAAAGAAACGA	CDK11B__NM_001291345	24	0	28	39
CDK11B__NM_001291345_TTCTTTCCGAGACATTTGCT	CDK11B__NM_001291345	0	0	0	0
CDK11B__NM_001787_ACAGGGCTGGTCTGGACGAG	CDK11B__NM_001787	752	266	911	761
CDK11B__NM_001787_AGCCCCGTGCGTGTCGAGAG	CDK11B__NM_001787	776	501	944	874
CDK11B__NM_001787_CTCCCACTCTCGACACGCAC	CDK11B__NM_001787	673	842	796	729
CDK11B__NM_001787_GATTGTTTAATCTTCGGAGC	CDK11B__NM_001787	183	92	381	188
CDK11B__NM_001787_GCCCCGTGCGTGTCGAGAGT	CDK11B__NM_001787	2348	1511	2223	1298
CDK11B__NM_001787_GCTGGTCTGGACGAGTGGGT	CDK11B__NM_001787	962	538	1039	621
CDK11B__NM_001787_GCTGTGCCAGCAGACTCGAC	CDK11B__NM_001787	193	102	216	296
CDK11B__NM_001787_TAAAAACCTGTCGAGTCTGC	CDK11B__NM_001787	151	139	147	161
CDK11B__NM_001787_TCCCACTCTCGACACGCACG	CDK11B__NM_001787	1225	651	1192	844
CDK11B__NM_001787_TCTCCCACTCTCGACACGCA	CDK11B__NM_001787	335	262	401	449
CDK12__NM_015083_AGGGAGAACGACGAACGTCG	CDK12__NM_015083	47	41	71	50
CDK12__NM_015083_ATCGATGAAGGACCGGATAT	CDK12__NM_015083	721	516	911	894
CDK12__NM_015083_CAGCAAGTCGGGATCGATGA	CDK12__NM_015083	686	194	913	378
CDK12__NM_015083_CAGCAGAGAGCGTCACCGCT	CDK12__NM_015083	693	564	756	759
CDK12__NM_015083_CTCCCTTCGGTCTAGTTTGA	CDK12__NM_015083	500	115	543	213
CDK12__NM_015083_GAAGGCCATGTCATCGGAGA	CDK12__NM_015083	369	179	414	283
CDK12__NM_015083_GCTTGTGCTTCGATACCAAG	CDK12__NM_015083	414	246	368	364
CDK12__NM_015083_GGCCTTCAAACTAGACCGAA	CDK12__NM_015083	173	81	274	188
CDK12__NM_015083_TCGATGAAGGACCGGATATC	CDK12__NM_015083	516	177	563	287
CDK12__NM_015083_TGCCCAGGGATGCTGCTTCG	CDK12__NM_015083	1455	1166	1499	835
CDK12__NM_015083_TGGAACTTTGCAGCCGTCAT	CDK12__NM_015083	142	184	157	217
CDK12__NM_015083_TGGCCTTCAAACTAGACCGA	CDK12__NM_015083	51	1	51	27
CDK12__NM_016507_AACTGGCAGGGTACACATGC	CDK12__NM_016507	622	626	641	567
CDK12__NM_016507_AAGGGAAGGGCTGTCAGGAT	CDK12__NM_016507	681	462	588	692
CDK12__NM_016507_ACACACACATACAAGTGGTT	CDK12__NM_016507	1122	1235	1038	1063
CDK12__NM_016507_AGCAAAAGTGGTATAGTAAC	CDK12__NM_016507	242	189	155	143
CDK12__NM_016507_ATAAAGCAGGTTGTAGAACG	CDK12__NM_016507	237	162	234	228
CDK12__NM_016507_ATAGACAAGATACACCCAAT	CDK12__NM_016507	355	250	337	244
CDK12__NM_016507_CATAGACAAGATACACCCAA	CDK12__NM_016507	417	283	366	432
CDK12__NM_016507_CATTGCCTGCACTTACTGAT	CDK12__NM_016507	1232	1202	1117	1230
CDK12__NM_016507_CCATTCCCTCATCCCTACTA	CDK12__NM_016507	1415	1005	1344	1313
CDK12__NM_016507_CCCCTTCCCTAGTAGGGATG	CDK12__NM_016507	249	175	294	272
CDK12__NM_016507_CTAGCTATATCTCATTAACA	CDK12__NM_016507	142	175	205	163
CDK12__NM_016507_CTTGCTCACTTGCTACTAGC	CDK12__NM_016507	173	246	221	294
CDK12__NM_016507_GAGAGAAAGGAATCAGTGTC	CDK12__NM_016507	1673	1803	2026	1988
CDK12__NM_016507_GCTGTCAGGATGGGATAATT	CDK12__NM_016507	402	451	404	309
CDK12__NM_016507_GGGTCAAAAACGAGAGAGAA	CDK12__NM_016507	109	173	176	184
CDK12__NM_016507_GTAAGCCTAGTAAATCTAAC	CDK12__NM_016507	67	177	126	124
CDK12__NM_016507_GTATGTTCAAGATGCTCAAT	CDK12__NM_016507	112	166	186	194
CDK12__NM_016507_TAGCTATATCTCATTAACAG	CDK12__NM_016507	265	132	288	181
CDK12__NM_016507_TCAAATACTTATCGTTCCTA	CDK12__NM_016507	206	292	324	358
CDK12__NM_016507_TCAATGCCAAAATAGCTAGA	CDK12__NM_016507	355	603	434	442
CDK12__NM_016507_TCCATTCCCTCATCCCTACT	CDK12__NM_016507	1265	1283	1200	1262
CDK12__NM_016507_TGCAGATGATTACCTCGCTC	CDK12__NM_016507	161	91	199	157
CDK12__NM_016507_TTCTCTACTTGTATCTACTA	CDK12__NM_016507	0	0	1	0
CDK13__NM_003718_ACAGGAATCGCCTCCGCTTG	CDK13__NM_003718	1445	1126	1527	1237
CDK13__NM_003718_ACTACCACCTTCTCTTGATA	CDK13__NM_003718	1109	756	1143	897
CDK13__NM_003718_AGCAGCTCGGACACGGCGCT	CDK13__NM_003718	464	396	617	525
CDK13__NM_003718_ATCCTCGTATTCCACCAGCG	CDK13__NM_003718	190	201	307	286
CDK13__NM_003718_ATGAAGGTTCATAAGTTAGA	CDK13__NM_003718	179	259	207	196
CDK13__NM_003718_CACTGCTGCTGCGGCGGTCC	CDK13__NM_003718	1238	1644	1665	1901
CDK13__NM_003718_CAGCCACAGCGGCGAGGAAC	CDK13__NM_003718	303	417	437	488
CDK13__NM_003718_CATTTGCCATGGATAATTGG	CDK13__NM_003718	239	280	313	396
CDK13__NM_003718_CCGCAAGGACCGGGACTCGA	CDK13__NM_003718	1906	1733	1961	1739
CDK13__NM_003718_CCTAAATGTGTTAAAGTTCA	CDK13__NM_003718	787	1358	1038	1383
CDK13__NM_003718_CTACCACCTTCTCTTGATAG	CDK13__NM_003718	803	956	879	931
CDK13__NM_003718_GACCTTAAGCTTACCTGTTA	CDK13__NM_003718	1933	1942	2090	2251
CDK13__NM_003718_GAGAAAGTGTGACAAGCAGA	CDK13__NM_003718	182	164	142	110
CDK13__NM_003718_GCAGAGGGAATGGACCTAAC	CDK13__NM_003718	720	667	844	1116
CDK13__NM_003718_GCGAGAGGCAAGAGGCGCGC	CDK13__NM_003718	1721	1418	1976	1612
CDK13__NM_003718_GCTCCTTGGACGACTTAGTC	CDK13__NM_003718	0	0	4	11
CDK13__NM_003718_GGCAGGAGGCGGAGAAGCGT	CDK13__NM_003718	663	667	823	578
CDK13__NM_003718_GGCGGTGGCGGTGCTAGTAG	CDK13__NM_003718	752	994	807	836
CDK13__NM_003718_GGGAAAGTAAGTGCCTTAAC	CDK13__NM_003718	364	430	279	328
CDK13__NM_003718_GGGTTAACTGGCTTTAGAAC	CDK13__NM_003718	422	570	379	456
CDK13__NM_003718_GGTGGCGGTGCTAGTAGCGG	CDK13__NM_003718	321	320	310	366
CDK13__NM_003718_GTGTTTCCATTATCACTAAA	CDK13__NM_003718	23	11	15	50
CDK13__NM_003718_GTTCATAAGTTAGAGGGCAC	CDK13__NM_003718	25	2	22	26
CDK13__NM_003718_TGCCTTAACAGGTAAGCTTA	CDK13__NM_003718	117	103	172	135
CDK13__NM_003718_TGGCGGTGCTAGTAGCGGCG	CDK13__NM_003718	152	106	143	149
CDK13__NM_003718_TTAGAGGGCACAGGGTTAAC	CDK13__NM_003718	72	44	124	87
CDK13__NM_003718_TTATATGAAAACTATGGGTG	CDK13__NM_003718	10	0	19	6
CDK13__NM_003718_TTCATAAGTTAGAGGGCACA	CDK13__NM_003718	38	14	22	27
CDK14__NM_001287135_AAGAGTCACCTAAAGTTAGG	CDK14__NM_001287135	129	120	85	94
CDK14__NM_001287135_AAGCTGGAAAAACTAGGGGA	CDK14__NM_001287135	566	645	503	525
CDK14__NM_001287135_ATCCTCAGGAATTGTGTCCA	CDK14__NM_001287135	1187	1321	1312	1455
CDK14__NM_001287135_CCGCAGCCGGCCGAGAAGAT	CDK14__NM_001287135	77	96	59	90
CDK14__NM_001287135_CCTTTCACAGCTATCAGGGA	CDK14__NM_001287135	794	900	822	1087
CDK14__NM_001287135_CTTATGCTACAGTATACAAA	CDK14__NM_001287135	143	121	110	87
CDK14__NM_001287135_GAAGTTGCGGAGAACTTTGT	CDK14__NM_001287135	611	855	674	964
CDK14__NM_001287135_GACACCTTTCACAGCTATCA	CDK14__NM_001287135	742	889	893	937
CDK14__NM_001287135_GATTAGCTGGTTTCTCAAAT	CDK14__NM_001287135	281	237	306	387
CDK14__NM_001287135_GCAAAGAGTCACCTAAAGTT	CDK14__NM_001287135	1016	1295	1067	1160
CDK14__NM_001287135_GCTCAATGAGGTCACACATC	CDK14__NM_001287135	450	327	449	413
CDK14__NM_001287135_TAGCCAACAAGTCCCAAATT	CDK14__NM_001287135	25	7	20	19
CDK14__NM_001287136_CAGTACATGGACAAGCACCC	CDK14__NM_001287136	931	927	968	963
CDK14__NM_001287136_TCATGACATCATCCATACCA	CDK14__NM_001287136	1141	1148	1155	1313
CDK14__NM_001287136_TGTGCAAAATATAACGCTGG	CDK14__NM_001287136	985	747	1048	569
CDK14__NM_001287137_ATCTTTGTTGAAATGATCCA	CDK14__NM_001287137	55	91	42	38
CDK14__NM_001287137_CCACAAGGTAACCACTTCGT	CDK14__NM_001287137	432	518	558	467
CDK14__NM_001287137_CCCCCGTGTCACTGATCAGA	CDK14__NM_001287137	2784	2957	2908	3129
CDK14__NM_001287137_CTAAAAACCTTAGACAAGCA	CDK14__NM_001287137	194	209	224	468
CDK14__NM_001287137_TGGGGAAACATTGTAGGAGCT	CDK14__NM_001287137	159	83	170	217
CDK14__NM_012395_CCCACGAAGGCAGAGTAACC	CDK14__NM_012395	383	500	528	498
CDK14__NM_012395_CCCGGTTACTCTGCCTTCGT	CDK14__NM_012395	4892	5781	5615	6207
CDK14__NM_012395_TGCCTGTGGAGTTCCCACGA	CDK14__NM_012395	680	620	737	592
CDK15__NM_001261435_AAACAATCACTGTTACTCCG	CDK15__NM_001261435	416	449	489	547
CDK15__NM_001261435_ACCACCAACACGTTCTTCAC	CDK15__NM_001261435	1125	1061	1183	1088
CDK15__NM_001261435_CCTCGCCTCCCTCTGAACAA	CDK15__NM_001261435	680	908	888	899
CDK15__NM_001261435_CTCCAAGTTCAAGTAAGATG	CDK15__NM_001261435	325	382	364	390
CDK15__NM_001261435_CTGGATGCTGAGACATATAC	CDK15__NM_001261435	646	814	741	708
CDK15__NM_001261435_CTTATGCGACAGTTTACAAG	CDK15__NM_001261435	248	268	263	286
CDK15__NM_001261435_CTTCTCGGATAGCTGTAAAT	CDK15__NM_001261435	221	265	203	180
CDK15__NM_001261435_GAGTCAGCCTGAGACCACGG	CDK15__NM_001261435	772	706	912	987

CDK15__NM_001261435_GCTGAGACATATACTGGGCC	CDK15__NM_001261435	1361	2164	1539	1894
CDK15__NM_001261435_TAGAATAAATGGACAACTAG	CDK15__NM_001261435	33	5	67	153
CDK15__NM_001261435_TCTTATGCGACAGTTTACAA	CDK15__NM_001261435	278	108	248	293
CDK15__NM_001261435_TGGATGCTGAGACATATACT	CDK15__NM_001261435	71	114	76	82
CDK15__NM_139158_CCACCAACACGTTCTTCACA	CDK15__NM_139158	1768	1863	2100	2038
CDK15__NM_139158_TGACTGATGAGTAAGTTCTG	CDK15__NM_139158	284	271	362	357
CDK16__NM_001170460_AGACGGTGGCATAGGTACCC	CDK16__NM_001170460	21	13	55	42
CDK16__NM_001170460_ATCCAGGCCTATCTGCTCAG	CDK16__NM_001170460	990	1049	1042	1002
CDK16__NM_001170460_ATCCCAACAATCCATCGTTA	CDK16__NM_001170460	738	667	895	898
CDK16__NM_001170460_CAGCTGTCAATGACACTCCG	CDK16__NM_001170460	1255	1638	1269	1493
CDK16__NM_001170460_CCTCAGTGGAGATCTTGCGT	CDK16__NM_001170460	1361	1302	1605	1069
CDK16__NM_001170460_CCTGAGTTCTCAGATTTAGG	CDK16__NM_001170460	559	622	686	504
CDK16__NM_001170460_CTCAGTGGAGATCTTGCGTG	CDK16__NM_001170460	685	916	849	985
CDK16__NM_001170460_GAGGGCAAGGGCCCTAACGA	CDK16__NM_001170460	109	110	102	118
CDK16__NM_001170460_GATCCCAACAATCCATCGTT	CDK16__NM_001170460	757	1406	805	746
CDK16__NM_001170460_GATGGATTGTTGGGATCTAG	CDK16__NM_001170460	358	308	393	361
CDK16__NM_001170460_GGGCCCTAACGATGGATTGT	CDK16__NM_001170460	879	1180	942	1219
CDK16__NM_001170460_TCATCCAGGCCTATCTGCTC	CDK16__NM_001170460	173	282	172	163
CDK16__NM_006201_AACATGCACAACGTGAAAGT	CDK16__NM_006201	483	433	495	512
CDK16__NM_006201_ACCTATGCCACCGTCTACAA	CDK16__NM_006201	1011	1133	1361	1743
CDK16__NM_006201_CAACATGCACAACGTGAAAG	CDK16__NM_006201	142	233	201	352
CDK16__NM_006201_GGGTGAGGGACTTCTCCGTG	CDK16__NM_006201	1726	2360	2102	2209
CDK16__NM_006201_GTCATGTAGCGTAACGATGT	CDK16__NM_006201	1005	1226	1132	1344
CDK16__NM_006201_TTACCAGGTACTCAAAGACA	CDK16__NM_006201	496	543	494	601
CDK17__NM_001170464_ACACTGTGTGGTCTCCGCAA	CDK17__NM_001170464	756	582	808	504
CDK17__NM_001170464_AGACCACACAGTGTTATTGG	CDK17__NM_001170464	1523	1211	1348	1312
CDK17__NM_001170464_CACTGTGTGGTCTCCGCAAT	CDK17__NM_001170464	1570	1667	1699	1631
CDK17__NM_001170464_CGGAGACCACACAGTGTTAT	CDK17__NM_001170464	1598	2064	1918	1996
CDK17__NM_001170464_CTGACTTCCTCGGAGTGTGA	CDK17__NM_001170464	756	756	856	1068
CDK17__NM_001170464_CTTTCAAGAAGCCCCCATTG	CDK17__NM_001170464	669	650	677	633
CDK17__NM_001170464_GAAAACAGCAGCAAGGATAA	CDK17__NM_001170464	169	208	176	163
CDK17__NM_001170464_GAATGCATACTGTGAGACGT	CDK17__NM_001170464	1596	1895	1851	1800
CDK17__NM_001170464_GATGAAGTCCAGTCACCTAC	CDK17__NM_001170464	1364	1203	1524	1322
CDK17__NM_001170464_TCTTGAAAGATCCTGTGTAC	CDK17__NM_001170464	369	388	421	552
CDK17__NM_001170464_TGATGAATCATTGTCTGAAT	CDK17__NM_001170464	589	711	780	1025
CDK17__NM_001170464_TGCATACTGTGAGACGTTGG	CDK17__NM_001170464	810	860	975	809
CDK17__NM_002595_AAACGTCTACTGCATATTAC	CDK17__NM_002595	446	609	518	658
CDK17__NM_002595_ATTTGCTTAGTAATTTCTGC	CDK17__NM_002595	305	412	314	433
CDK17__NM_002595_CAGCAGTAAAACAATTCGTG	CDK17__NM_002595	615	747	736	710
CDK17__NM_002595_CTGTAACCTACTGGCTCTAA	CDK17__NM_002595	217	250	336	303
CDK17__NM_002595_GAAAGGATGATATTTCATTG	CDK17__NM_002595	301	179	269	305
CDK17__NM_002595_GAAGTCTGTTCAAACGCGAA	CDK17__NM_002595	112	244	130	246
CDK17__NM_002595_GAGTCCTTGATTGGTAAGAA	CDK17__NM_002595	426	629	433	664
CDK17__NM_002595_GGGATGAAGGCTGTGCCGAG	CDK17__NM_002595	219	274	241	274
CDK17__NM_002595_GTGAGATCCTCAAAATGTCA	CDK17__NM_002595	307	442	327	254
CDK17__NM_002595_TTTGCTTAGTAATTTCTGCT	CDK17__NM_002595	74	70	78	119
CDK18__NM_002596_CCTTCTCCCCAACAGACAGC	CDK18__NM_002596	311	308	327	273
CDK18__NM_002596_CTGGGGCAGGCGGATATCCA	CDK18__NM_002596	649	679	611	675
CDK18__NM_002596_GAAGCTGGCCGACTTTGGTG	CDK18__NM_002596	392	357	339	188
CDK18__NM_002596_GACATGCGGCTGAGCGGCTT	CDK18__NM_002596	616	636	701	815
CDK18__NM_002596_GCCGCTGGAACTGCACGCCA	CDK18__NM_002596	1177	1041	1438	1073
CDK18__NM_002596_GCTCCTCCCCGCTGTCTGTT	CDK18__NM_002596	5028	6095	5724	7190
CDK18__NM_002596_GGCTCCTCCCCGCTGTCTGT	CDK18__NM_002596	775	731	752	584
CDK18__NM_002596_GGGTGAGGGACCGATCTGTG	CDK18__NM_002596	411	417	495	338
CDK18__NM_002596_GTCATGCAGGGTCACAATAT	CDK18__NM_002596	1065	943	1120	696
CDK18__NM_002596_TCAACCAGCTCCACAACCGG	CDK18__NM_002596	971	1152	960	647
CDK18__NM_002596_TGCGGTGGTGACAGTAGGCG	CDK18__NM_002596	192	250	230	192
CDK18__NM_002596_TGGGGCAGGCGGATATCCAT	CDK18__NM_002596	137	274	136	165
CDK18__NM_212503_TCCAGAGGCCCTTACCTCCA	CDK18__NM_212503	420	320	417	323
CDK19__NM_015076_AAAGTGGGACGCGGCACCTA	CDK19__NM_015076	571	453	475	487
CDK19__NM_015076_AAGGATTTAACCATAGATCT	CDK19__NM_015076	0	0	0	0
CDK19__NM_015076_AGGCACAGGAATATCCATGT	CDK19__NM_015076	973	863	1120	876
CDK19__NM_015076_ATCAAGAATCTGGTAAAGTA	CDK19__NM_015076	268	374	244	249
CDK19__NM_015076_CATGCAGTTGCCAAGATCTA	CDK19__NM_015076	925	774	960	803
CDK19__NM_015076_CGCCTTGTAGACGTGACCGT	CDK19__NM_015076	2615	2499	2815	2518
CDK19__NM_015076_GAAGCACCCAATTTGCATGG	CDK19__NM_015076	580	351	669	705
CDK19__NM_015076_GAAGGGTGCAAAGTGGGACG	CDK19__NM_015076	219	159	185	149
CDK19__NM_015076_GAGGATTTGTTTGAGTACGA	CDK19__NM_015076	272	280	304	386
CDK19__NM_015076_TCCATTACCTCCATGCAAAT	CDK19__NM_015076	638	962	652	979
CDK19__NM_015076_TTACTTTACCAGATTCTTGA	CDK19__NM_015076	152	168	187	325
CDK19__NM_015076_TTATGCAGAGCATGACTTGT	CDK19__NM_015076	424	328	498	374
CDK2__NM_001290230_AAAGCCAGAAACAAGTTGAC	CDK2__NM_001290230	188	119	178	77
CDK2__NM_001290230_AAAGGTGGAAAAGATCGGAG	CDK2__NM_001290230	49	30	57	27
CDK2__NM_001290230_AAGATCGGAGAGGGCACGTA	CDK2__NM_001290230	225	131	260	79
CDK2__NM_001290230_AATCTCCAGGGAATAGGGCC	CDK2__NM_001290230	402	377	424	352
CDK2__NM_001290230_CACAGAAAATAAACTCTACC	CDK2__NM_001290230	753	1170	862	1191
CDK2__NM_001290230_CAGAAACAAGTTGACGGGAG	CDK2__NM_001290230	357	140	292	202
CDK2__NM_001290230_CATGGGTGTAAGTACGAACA	CDK2__NM_001290230	370	209	279	181
CDK2__NM_001290230_GAATCTGCTTATTAACACAG	CDK2__NM_001290230	118	59	149	61
CDK2__NM_001290230_GGTGGAGGACCCGATGAGAA	CDK2__NM_001290230	187	25	238	73
CDK2__NM_001290230_TAAGCAGATTCTGAGGTTTA	CDK2__NM_001290230	1129	591	1192	539
CDK2__NM_001290230_TAGCTTTCTGCCATTCTCAT	CDK2__NM_001290230	700	360	619	378
CDK2__NM_001290230_TCATGGGTGTAAGTACGAAC	CDK2__NM_001290230	129	29	141	102
CDK2__NM_001798_ATCTCTCGGATGGCAGTACT	CDK2__NM_001798	487	336	518	506
CDK2__NM_001798_GGCACACCCTCAGTCTCACT	CDK2__NM_001798	1616	1386	1844	1616
CDK20__NM_001039803_ACCCAGCAGATCCAATGCCT	CDK20__NM_001039803	1638	1701	1832	1644
CDK20__NM_001039803_ATGACCAGGGCGTCGATCTG	CDK20__NM_001039803	1095	988	1209	1030
CDK20__NM_001039803_CCAGGCCCTGCGGGAGATTA	CDK20__NM_001039803	423	325	516	497
CDK20__NM_001039803_CCCAAACCCTCAAGTCTGGC	CDK20__NM_001039803	1728	1979	1939	2843
CDK20__NM_001039803_CGAGATAGTTGCCCTCAAGA	CDK20__NM_001039803	430	677	548	612
CDK20__NM_001039803_GAAGGTGGCCCTAAGGCGGT	CDK20__NM_001039803	50	52	77	42
CDK20__NM_001039803_GCAGAGCCTTAATCTCCCGC	CDK20__NM_001039803	543	468	573	727
CDK20__NM_001039803_GCCGTCCTCCAACCGCCTTA	CDK20__NM_001039803	1253	1936	1411	2016
CDK20__NM_001039803_GGTAGCTCTTGACCTGTGCC	CDK20__NM_001039803	124	273	140	157
CDK20__NM_001039803_TCAAGAAGGTGGCCCTAAGG	CDK20__NM_001039803	48	114	53	44
CDK20__NM_001039803_TCTCCCGCAGGGCCTGGTTA	CDK20__NM_001039803	903	1067	989	768
CDK20__NM_001039803_TTGCTATGTGCTTCGCATCT	CDK20__NM_001039803	131	230	120	150
CDK20__NM_001170639_TGCCGATTCCTCAGCGTCTA	CDK20__NM_001170639	483	645	556	570
CDK20__NM_001170640_GAGCTGTGAAGAAGTACTGA	CDK20__NM_001170640	606	550	769	635
CDK20__NM_001170640_GCCGATTCCTCAGCGTCTAG	CDK20__NM_001170640	1471	1755	1600	1828
CDK2AP1__NM_001270433_ACATACCTGGGTGTAGCCTA	CDK2AP1__NM_001270433	265	373	317	276
CDK2AP1__NM_001270433_ACTCTTGCTCCCTGCGTACG	CDK2AP1__NM_001270433	3044	3353	3172	3185
CDK2AP1__NM_001270433_AGATCAGACCCACGTACGCA	CDK2AP1__NM_001270433	836	907	918	769
CDK2AP1__NM_001270433_AGCTCCGCGTATTTGCTTTG	CDK2AP1__NM_001270433	930	1208	1013	1377
CDK2AP1__NM_001270433_AGGACTGGTTCGGGAGTGCT	CDK2AP1__NM_001270433	315	244	352	457
CDK2AP1__NM_001270433_CTCTTGCTCCCTGCGTACGT	CDK2AP1__NM_001270433	382	499	422	682
CDK2AP1__NM_001270433_CTGTGAAGACGTTGCCATGC	CDK2AP1__NM_001270433	573	518	683	640
CDK2AP1__NM_001270433_GAGATCAGACCCACGTACGC	CDK2AP1__NM_001270433	843	638	819	790
CDK2AP1__NM_001270433_TCAGGCATCATTCACGCTAG	CDK2AP1__NM_001270433	707	510	766	482
CDK2AP1__NM_004642_GGCATGTGCGCGGCCAAGTT	CDK2AP1__NM_004642	1157	1286	1087	1189
CDK2AP2__NM_001271849_CCCGGGCATGGATGATACCT	CDK2AP2__NM_001271849	878	562	876	555
CDK2AP2__NM_001271849_CCGGGCATGGATGATACCTG	CDK2AP2__NM_001271849	904	466	1020	585
CDK2AP2__NM_001271849_CTGTCAGTCATAGAGGAGAT	CDK2AP2__NM_001271849	321	415	426	350
CDK2AP2__NM_001271849_GAGATCCGGCCTACCTATGC	CDK2AP2__NM_001271849	591	24	604	63
CDK2AP2__NM_001271849_GCGGAACGCCCGCACGTAAC	CDK2AP2__NM_001271849	119	130	218	197
CDK2AP2__NM_001271849_TGCTGCCAGCATAGGTAGGC	CDK2AP2__NM_001271849	0	0	0	0
CDK2AP2__NM_005851_AGACCGCTGTTTAACGACTT	CDK2AP2__NM_005851	898	693	992	776
CDK2AP2__NM_005851_GCTCCTGGCACTGAGCCCGA	CDK2AP2__NM_005851	74	115	85	144
CDK2AP2__NM_005851_GGTCCAAAGTCGTTAAACAG	CDK2AP2__NM_005851	359	62	279	154
CDK2AP2__NM_005851_TCGTTAAACAGCGGTCTGAA	CDK2AP2__NM_005851	132	163	116	173
CDK3__NM_001258_AGGCCAAGAACAGGGAGACA	CDK3__NM_001258	197	330	258	250
CDK3__NM_001258_CAACGAGAGGAAGCTCTATC	CDK3__NM_001258	217	229	299	290
CDK3__NM_001258_CCTGCGCACCTACACCCATG	CDK3__NM_001258	2660	2249	2591	2210
CDK3__NM_001258_CGGAGAGGGCACCTATGGGG	CDK3__NM_001258	693	586	856	769
CDK3__NM_001258_CTTTCGTATCTTTCGTATGC	CDK3__NM_001258	378	537	341	497
CDK3__NM_001258_GAAGGTAGAGAAGATCGGAG	CDK3__NM_001258	368	158	468	361
CDK3__NM_001258_GCTGCCCAAGAGAATCTCGG	CDK3__NM_001258	1213	777	1317	895
CDK3__NM_001258_GGCCCTGAAGAAGATCAGAC	CDK3__NM_001258	529	592	619	780
CDK3__NM_001258_GGTGGATGACCCGATGTGAG	CDK3__NM_001258	448	449	438	449
CDK3__NM_001258_TACCTTCTGGAACATATCCA	CDK3__NM_001258	445	318	420	349
CDK3__NM_001258_TGAAGCACCCCAACATCGTC	CDK3__NM_001258	314	326	279	289
CDK3__NM_001258_TGAGTTTCTGCCACTCACAT	CDK3__NM_001258	819	1031	911	746
CDK4__NM_000075_AAATTGGTGTCGGTGCCTAT	CDK4__NM_000075	383	191	329	111
CDK4__NM_000075_AATCTAGGCCTCTTAGAAAC	CDK4__NM_000075	74	16	79	9
CDK4__NM_000075_AGCCACTGGCTCATATCGAG	CDK4__NM_000075	156	31	157	36
CDK4__NM_000075_AGGACCCCAAATATAAAGGT	CDK4__NM_000075	243	64	286	190
CDK4__NM_000075_CATCAGCACAGTTCGTGAGG	CDK4__NM_000075	319	159	337	93
CDK4__NM_000075_CCTTGATCTCCCGGTCAGTT	CDK4__NM_000075	2885	1998	3163	1888
CDK4__NM_000075_CCTTTCCCTACCTTTATATT	CDK4__NM_000075	3060	3491	3484	3159
CDK4__NM_000075_CTCACCTTGATCGTTTCGGC	CDK4__NM_000075	636	736	618	668
CDK4__NM_000075_CTCCATAGCCTCAGAGATAA	CDK4__NM_000075	116	199	204	208
CDK4__NM_000075_CTTCCCATTTCTCTACACTA	CDK4__NM_000075	646	636	827	834
CDK4__NM_000075_CTTGATCTCCCGGTCAGTTC	CDK4__NM_000075	947	606	977	412
CDK4__NM_000075_CTTGCCAGCCGAAACGATCA	CDK4__NM_000075	161	182	150	132
CDK4__NM_000075_GAAATTGGTGTCGGTGCCTA	CDK4__NM_000075	579	302	560	242
CDK4__NM_000075_TCCCATCAGCACAGTTCGTG	CDK4__NM_000075	426	222	330	160
CDK4__NM_000075_TCCCATTTCTCTACACTAAG	CDK4__NM_000075	305	555	404	388
CDK4__NM_000075_TTGAGCATCCCAATGTTGTC	CDK4__NM_000075	526	319	523	281
CDK4__NM_000075_TTTCGGCTGGCAAGCCTGGT	CDK4__NM_000075	245	84	211	111
CDK5__NM_001164410_AAAAAGAGGCCGCCCAGCAT	CDK5__NM_001164410	237	332	313	363
CDK5__NM_001164410_ACACAGTTCCGTAGGTGCCT	CDK5__NM_001164410	607	730	767	1106
CDK5__NM_001164410_ATCTCCCGGAGGGCGGAACT	CDK5__NM_001164410	164	204	133	187
CDK5__NM_001164410_ATGTCGATGACCAGTTGAAG	CDK5__NM_001164410	173	120	179	206
CDK5__NM_001164410_CACAGTTCCGTAGGTGCCTA	CDK5__NM_001164410	170	257	229	300
CDK5__NM_001164410_CCCCAAAGAGGACATCCGGT	CDK5__NM_001164410	1962	2296	2166	2746
CDK5__NM_001164410_CCGGGAGACTCATGAGATCG	CDK5__NM_001164410	704	972	909	1010
CDK5__NM_001164410_CTCAGCTGAGTAACAGCGGA	CDK5__NM_001164410	141	92	202	242
CDK5__NM_001164410_GACCAAGCTGCCAGACTATA	CDK5__NM_001164410	700	883	706	918
CDK5__NM_001164410_GGTACCACAGTGTGACCACC	CDK5__NM_001164410	422	707	530	734
CDK5__NM_001164410_TCAGCTGAGTAACAGCGGAC	CDK5__NM_001164410	189	236	226	225
CDK5__NM_001164410_TCTGGCAGCTTGGTCATAGA	CDK5__NM_001164410	757	937	868	1045
CDK5__NM_004935_GGTCCCTGTGTAGCACATTG	CDK5__NM_004935	561	602	543	858
CDK5__NM_004935_TTCCAGCTACTAAAAGGGCT	CDK5__NM_004935	51	30	40	112
CDK5__NM_004935_TTCCTCTTCCAGCTACTAAA	CDK5__NM_004935	8	0	23	33
CDK5R1__NM_003885_AGACCTGTGTGAAGTAGTGT	CDK5R1__NM_003885	938	888	1021	927
CDK5R1__NM_003885_AGAGAGGCAACGGTCCCAAA	CDK5R1__NM_003885	560	663	525	467
CDK5R1__NM_003885_AGAGGTACAGGCATGTCAGC	CDK5R1__NM_003885	172	167	174	211
CDK5R1__NM_003885_CAGGAAGGGGCTTGAGCGGT	CDK5R1__NM_003885	1335	1039	1094	889
CDK5R1__NM_003885_CGCGCCATCCTCAAACAGCG	CDK5R1__NM_003885	933	1045	1067	1106
CDK5R1__NM_003885_CGCTGCGCAGCCAGAGCACG	CDK5R1__NM_003885	83	37	59	77
CDK5R1__NM_003885_GCCTGGACGATGACCCGTTT	CDK5R1__NM_003885	1473	1359	1795	1804
CDK5R1__NM_003885_GCTTCAGGCGGTAGCACCGG	CDK5R1__NM_003885	539	569	616	650
CDK5R1__NM_003885_GGCCACCGTGGGCCACTATA	CDK5R1__NM_003885	145	49	118	51
CDK5R1__NM_003885_GGCGTTCTTGCTGTTCTGTA	CDK5R1__NM_003885	1017	1332	931	1286
CDK5R1__NM_003885_GGGGCTGGGCGAATGTGGAC	CDK5R1__NM_003885	1829	1994	1720	2552
CDK5R1__NM_003885_TGTTCTGTACGGCCGTATAG	CDK5R1__NM_003885	88	68	59	57
CDK5R2__NM_003936_ACAGCAGGTACACGAACACC	CDK5R2__NM_003936	494	618	651	816
CDK5R2__NM_003936_CAGGTTTGCAGGCGTAATGA	CDK5R2__NM_003936	348	533	356	442
CDK5R2__NM_003936_CCGCGAGAACCTTCTCCGCA	CDK5R2__NM_003936	1597	2009	1597	1818
CDK5R2__NM_003936_CCTTGCGGAGAAGGTTCTCG	CDK5R2__NM_003936	535	662	746	593
CDK5R2__NM_003936_GATCCCGGCCCTTGCGGAGA	CDK5R2__NM_003936	505	527	567	650
CDK5R2__NM_003936_GCACGGACGGCCGCTTGAGT	CDK5R2__NM_003936	2282	1476	2561	2488
CDK5R2__NM_003936_GCGTGGGGACGAGCTGGCGT	CDK5R2__NM_003936	1778	1882	1987	2197
CDK5R2__NM_003936_GGTGAGCGCCGAGATGAGCA	CDK5R2__NM_003936	362	393	412	432
CDK5R2__NM_003936_GGTGGGCTGGTTCCGCGGTG	CDK5R2__NM_003936	489	655	523	633
CDK5R2__NM_003936_GTGCCGCGAGTCGCTGCGTG	CDK5R2__NM_003936	954	1101	1235	1210
CDK5R2__NM_003936_TGTAGGAGTAGGCGGAGGTAG	CDK5R2__NM_003936	193	172	163	154
CDK5R2__NM_003936_TGTGCCGCGAGTCGCTGCGT	CDK5R2__NM_003936	971	854	1133	1036
CDK5RAP1__NM_001278167_ACGTGGCACTGGCGCTTGTC	CDK5RAP1__NM_001278167	1383	1191	1386	1592
CDK5RAP1__NM_001278167_ATGTACCTCTTGGAGGTTAC	CDK5RAP1__NM_001278167	93	82	83	65
CDK5RAP1__NM_001278167_CTGATGTAAACGGTTCCAGA	CDK5RAP1__NM_001278167	216	147	317	282
CDK5RAP1__NM_001278167_CTTCACTTCCTCTAGAATGG	CDK5RAP1__NM_001278167	598	658	722	743
CDK5RAP1__NM_001278167_CTTGGAGGTTACTGGTCCGC	CDK5RAP1__NM_001278167	1359	1288	1339	1237
CDK5RAP1__NM_001278167_GCAGTCTACCTCGAGACCTA	CDK5RAP1__NM_001278167	1063	658	1098	1011
CDK5RAP1__NM_001278167_GTCTCTGGGTGGGGACCAT	CDK5RAP1__NM_001278167	658	547	634	680
CDK5RAP1__NM_001278167_TCATCCATCATGAGATAGGG	CDK5RAP1__NM_001278167	185	126	211	204
CDK5RAP1__NM_001278167_TCCTTGTCACATGCTCTATC	CDK5RAP1__NM_001278167	172	172	198	240
CDK5RAP1__NM_001278167_TCTGGCAGCCATAGGTCTCG	CDK5RAP1__NM_001278167	89	81	101	130
CDK5RAP1__NM_001278167_TGAATGACACAGAGATAGCC	CDK5RAP1__NM_001278167	34	77	61	38
CDK5RAP1__NM_001278167_TGGCTGGTCCTGATGCCTAC	CDK5RAP1__NM_001278167	35	35	65	25
CDK5RAP1__NM_001278169_GGCCTGAAGTAGCGGCGGAA	CDK5RAP1__NM_001278169	832	1041	887	1448
CDK5RAP2__NM_001011649_ACTGTTTCTTCTGACACATT	CDK5RAP2__NM_001011649	852	949	1148	1073
CDK5RAP2__NM_001011649_AGATGAAGAAGATGCACGAG	CDK5RAP2__NM_001011649	345	128	316	172
CDK5RAP2__NM_001011649_AGCCATGATGGACTTGGTGT	CDK5RAP2__NM_001011649	863	937	882	740
CDK5RAP2__NM_001011649_AGCCATTCAGGGTTTAACCA	CDK5RAP2__NM_001011649	170	89	144	101
CDK5RAP2__NM_001011649_CACCAGAGCACGGAACATGA	CDK5RAP2__NM_001011649	527	348	666	490
CDK5RAP2__NM_001011649_CCTTGAGGAAAGAATGCAAC	CDK5RAP2__NM_001011649	480	465	537	673
CDK5RAP2__NM_001011649_GAAAGAAGATGCTCGAAAGA	CDK5RAP2__NM_001011649	208	214	214	123
CDK5RAP2__NM_001011649_GAAGGCTCTTCGGTTGCGTT	CDK5RAP2__NM_001011649	613	584	715	793
CDK5RAP2__NM_001011649_GACTATGAGACTGCTCTATC	CDK5RAP2__NM_001011649	412	179	494	246
CDK5RAP2__NM_001011649_GCACGAGGGGAACTTGGCGA	CDK5RAP2__NM_001011649	626	645	772	735
CDK5RAP2__NM_001011649_TCCAGGTCATCTGGTACACT	CDK5RAP2__NM_001011649	664	497	714	556
CDK5RAP2__NM_001011649_TGCCATGGTTAAACCCTGAA	CDK5RAP2__NM_001011649	190	216	226	209
CDK5RAP3__NM_001278197_ATACTCTTCACAGCGGATGA	CDK5RAP3__NM_001278197	173	237	264	175
CDK5RAP3__NM_001278197_ATGGTAAAGCGACGCAAGGA	CDK5RAP3__NM_001278197	11	0	7	14
CDK5RAP3__NM_001278197_CATAGTTGACATTCCGAACC	CDK5RAP3__NM_001278197	283	223	315	145
CDK5RAP3__NM_001278197_CGTGATGCCATACTGCTTGC	CDK5RAP3__NM_001278197	1018	1310	1205	1375
CDK5RAP3__NM_001278197_CTGCAAGCAGTATGGCATCA	CDK5RAP3__NM_001278197	287	302	337	296
CDK5RAP3__NM_001278197_CTGGATGGTAAAGCGACGCA	CDK5RAP3__NM_001278197	286	348	256	336
CDK5RAP3__NM_001278197_CTTTCACTGCCTAAGAATCC	CDK5RAP3__NM_001278197	439	181	374	245
CDK5RAP3__NM_001278197_GATGGTAAAGCGACGCAAGG	CDK5RAP3__NM_001278197	276	313	288	321
CDK5RAP3__NM_001278197_GCCAAAAATATTCTTCGTGG	CDK5RAP3__NM_001278197	601	1095	805	1142
CDK5RAP3__NM_001278197_TACCACTCCTGCAAGCAGTA	CDK5RAP3__NM_001278197	227	151	219	124
CDK5RAP3__NM_001278197_TCATCGCTGTGAAGAGTAT	CDK5RAP3__NM_001278197	578	455	610	313
CDK5RAP3__NM_001278197_TTGCTGGTCTGGATGTCGAT	CDK5RAP3__NM_001278197	335	256	323	192
CDK5RAP3__NM_001278198_ACTCAACGGTGTACGAGTGG	CDK5RAP3__NM_001278198	580	178	487	266
CDK5RAP3__NM_001278198_CAAAGTCGCCCCAGTCAATC	CDK5RAP3__NM_001278198	561	880	710	852
CDK5RAP3__NM_001278198_CAGATTGACTGGGGCGACTT	CDK5RAP3__NM_001278198	526	555	572	513
CDK5RAP3__NM_001278198_CCGAGGCTGCTGGAATCGAC	CDK5RAP3__NM_001278198	1214	1032	1544	1264
CDK5RAP3__NM_001278198_CTGGAGGTGATGGGATAGAC	CDK5RAP3__NM_001278198	337	463	457	450
CDK5RAP3__NM_001278198_GAAACTCAACGGTGTACGAG	CDK5RAP3__NM_001278198	619	727	758	848
CDK5RAP3__NM_001278198_GAGGCTGCTGGAATCGACTG	CDK5RAP3__NM_001278198	565	245	547	319
CDK5RAP3__NM_001278198_GATTGGCCCTGTGGTCTATC	CDK5RAP3__NM_001278198	652	852	671	774
CDK5RAP3__NM_001278198_TTCTGCACGAACCGCAGCAT	CDK5RAP3__NM_001278198	31	21	25	5
CDK5RAP3__NM_001278216_AGGTTCCCATCAGGTTCACA	CDK5RAP3__NM_001278216	67	35	87	34
CDK5RAP3__NM_001278216_GTCACAGAGAGGTTCCCATC	CDK5RAP3__NM_001278216	358	243	380	178
CDK5RAP3__NM_001278217_CTCTAGGGCGAAAATGTCCG	CDK5RAP3__NM_001278217	219	257	182	166
CDK5RAP3__NM_001278217_GGAAGCCATTGACGTGTACC	CDK5RAP3__NM_001278217	660	862	778	927
CDK6__NM_001145306_AAGCGCGTGCGGGTGCAGAC	CDK6__NM_001145306	802	395	682	578
CDK6__NM_001145306_AGATCGGGGAGGGCGCCTAT	CDK6__NM_001145306	1307	529	1302	531
CDK6__NM_001145306_CAAATAAAACTCGCTGACTT	CDK6__NM_001145306	1426	1907	1708	2027
CDK6__NM_001145306_CATCTGGAAACTATAGATGC	CDK6__NM_001145306	731	407	664	385
CDK6__NM_001145306_CGCGCTTCAACGCCACGAAA	CDK6__NM_001145306	516	300	522	356
CDK6__NM_001145306_CGGGGAGGGCGCCTATGGGA	CDK6__NM_001145306	867	480	832	446
CDK6__NM_001145306_CGGGTGCAGACCGGCGAGGA	CDK6__NM_001145306	786	630	843	494
CDK6__NM_001145306_GAAGAACGGAGGCCGTTTCG	CDK6__NM_001145306	490	203	483	387
CDK6__NM_001145306_GCCCGCGACTTGAAGAACGG	CDK6__NM_001145306	694	478	786	411
CDK6__NM_001145306_GGCCTCCGTTCTTCAAGTCG	CDK6__NM_001145306	816	1045	1040	1344
CDK6__NM_001145306_TGACCAGCAGTACGAATGCG	CDK6__NM_001145306	384	347	517	412
CDK6__NM_001145306_TTAGATCGCGATGCACTACT	CDK6__NM_001145306	180	70	217	163
CDK6__NM_001259_AAAATGCAGCTGTTCTGAAC	CDK6__NM_001259	283	504	364	452
CDK6__NM_001259_AAATGCAACAACTACACCGA	CDK6__NM_001259	648	839	804	731
CDK6__NM_001259_AAGACACCCGTTTGCTACTA	CDK6__NM_001259	595	454	574	653
CDK6__NM_001259_AAGATATATCATGCACTGTG	CDK6__NM_001259	147	228	202	175
CDK6__NM_001259_AATCCACAGCATTCAAGGTC	CDK6__NM_001259	86	140	97	110
CDK6__NM_001259_AATGCATGCAGCTTATTTGG	CDK6__NM_001259	1097	1043	1117	891
CDK6__NM_001259_ACAAATTATCTCATGTAGCA	CDK6__NM_001259	547	630	579	546
CDK6__NM_001259_ACACAAGCAGTCTTGCCGTT	CDK6__NM_001259	4614	5436	5321	6253
CDK6__NM_001259_ACACAATTCTCCGAAAGTTC	CDK6__NM_001259	381	635	458	373
CDK6__NM_001259_ACACAGTCTTATAAGAATGT	CDK6__NM_001259	64	151	78	82
CDK6__NM_001259_ACAGGTAATTAGTTTGCTT	CDK6__NM_001259	412	585	550	515
CDK6__NM_001259_ACATTCTGGCATTCAATCGT	CDK6__NM_001259	1181	1631	1343	1126
CDK6__NM_001259_ACCCACAGGGTGGACCCGAC	CDK6__NM_001259	414	244	450	451
CDK6__NM_001259_AGAAAGAAAGCTTATCTCTA	CDK6__NM_001259	201	199	144	182
CDK6__NM_001259_AGAGAAAATCATGAGAATAC	CDK6__NM_001259	218	243	205	145
CDK6__NM_001259_AGCTGTGGAGGATTGCTATC	CDK6__NM_001259	368	571	428	472
CDK6__NM_001259_AGGTAACAAAAGTAAACACC	CDK6__NM_001259	168	196	205	176
CDK6__NM_001259_AGTGTCACTTGAAATTTAGT	CDK6__NM_001259	527	453	492	418
CDK6__NM_001259_AGTTAAAGCCCCTATGCGAA	CDK6__NM_001259	259	202	280	318
CDK6__NM_001259_AGTTTCTGTGTTTGAAATCA	CDK6__NM_001259	82	39	107	145
CDK6__NM_001259_ATCAGGGTACAGACTTATAA	CDK6__NM_001259	132	126	99	84
CDK6__NM_001259_ATCTAGAAAAGTAAGGTAAC	CDK6__NM_001259	51	167	109	126
CDK6__NM_001259_ATGGTATAGACATAGCAAGA	CDK6__NM_001259	21	41	27	36
CDK6__NM_001259_ATTCTCCTCCCAGATAGGA	CDK6__NM_001259	33	73	10	51
CDK6__NM_001259_CAAATGCATGCAGCTTATTT	CDK6__NM_001259	577	787	566	830
CDK6__NM_001259_CACAAGCAGTCTTGCCGTTA	CDK6__NM_001259	645	638	741	578
CDK6__NM_001259_CACAGTGGAAAAACTTATTG	CDK6__NM_001259	97	142	110	140
CDK6__NM_001259_CAGCATTTCTAATTACTCTA	CDK6__NM_001259	346	377	462	543
CDK6__NM_001259_CAGTTGGCTCTAGTAACCAG	CDK6__NM_001259	76	127	118	154
CDK6__NM_001259_CATGGCAAAGGGTAATAAAA	CDK6__NM_001259	254	240	249	292
CDK6__NM_001259_CCACAAATGTTAAAATTACC	CDK6__NM_001259	247	276	266	468
CDK6__NM_001259_CCACCAAGGGTGTTCTCCGC	CDK6__NM_001259	220	246	302	350

[illegible]

CENPO__NM_024322_GGGCCGTGGGATGGTTGTAG	CENPO__NM_024322	623	789	714	735
CENPO__NM_024322_GTAAATCTGCATATGGGGGG	CENPO__NM_024322	368	360	414	492
CENPO__NM_024322_GTTCTCATGACTAAGGGGAC	CENPO__NM_024322	17	29	25	19
CENPO__NM_024322_TAAAGAAACACGTCCAAGGC	CENPO__NM_024322	108	181	174	156
CENPO__NM_024322_TAACAAGCACACGGATAAAC	CENPO__NM_024322	165	322	261	357
CENPO__NM_024322_TCCATGGGTCCCAAGCAGTA	CENPO__NM_024322	58	97	104	96
CENPO__NM_024322_TCCCTACACCTAGATGTTCA	CENPO__NM_024322	672	719	752	573
CENPO__NM_024322_TGCCTCTGGACTTACTGTTC	CENPO__NM_024322	291	279	296	312
CENPO__NM_024322_TGGAGCCGAGCACTGATCCA	CENPO__NM_024322	531	411	485	500
CENPO__NM_024322_TGGAGGGGGATGACCCGCCT	CENPO__NM_024322	301	441	340	445
CENPO__NM_024322_TTTAGTCATCTAGAGTCGTC	CENPO__NM_024322	74	155	97	150
CENPO__NM_024322_TTTCTGTTCTCATGACTAAG	CENPO__NM_024322	55	21	45	37
CENPO__NM_024322_TTTGGACTGTTGCAGGCTCG	CENPO__NM_024322	48	62	42	36
CENPP__NM_001012267_AGATGCCGTGTACCTCTCGG	CENPP__NM_001012267	2569	1150	2945	2233
CENPP__NM_001012267_AGCCATACACCAATTCAATT	CENPP__NM_001012267	667	644	735	879
CENPP__NM_001012267_CAAAGTCCCACAGCGAGGTA	CENPP__NM_001012267	357	351	347	322
CENPP__NM_001012267_CAAGTTGAAATGTAACCATG	CENPP__NM_001012267	229	112	267	179
CENPP__NM_001012267_CCGAGAGGTACACGGCATCT	CENPP__NM_001012267	1869	529	2056	1471
CENPP__NM_001012267_CTTAATTCTGAGCATTCTGT	CENPP__NM_001012267	346	64	305	296
CENPP__NM_001012267_GATGCCGTGTACCTCTCGGA	CENPP__NM_001012267	1394	656	1858	1316
CENPP__NM_001012267_GGTTTGAATTAGTCATTGTT	CENPP__NM_001012267	446	640	534	783
CENPP__NM_001012267_TACTGACCTCAACATAATAA	CENPP__NM_001012267	209	213	164	191
CENPP__NM_001012267_TCATTTCTAAGTACGCTTAC	CENPP__NM_001012267	164	207	110	169
CENPP__NM_001012267_TGGGCTCCATTATTATGTTG	CENPP__NM_001012267	183	233	175	277
CENPP__NM_001012267_TTCCAAATTGAATTGGTGTA	CENPP__NM_001012267	0	0	0	0
CENPP__NM_001286969_CAAGCAGTCCTACCAGGGTT	CENPP__NM_001286969	1461	203	1364	979
CENPP__NM_001286969_CTCAGCTTCCGAACCCTGGT	CENPP__NM_001286969	743	352	952	808
CENPP__NM_001286971_CCTTTGCAGAACAGACCAGC	CENPP__NM_001286971	348	380	394	423
CENPP__NM_001286971_TGGTCTGTTCTGCAAAGGTC	CENPP__NM_001286971	0	0	0	0
CENPQ__NM_018132_AAGCACACTAACCTAAAACA	CENPQ__NM_018132	274	373	268	280
CENPQ__NM_018132_AGAAGGTCTGGCATTACTAC	CENPQ__NM_018132	152	233	201	163
CENPQ__NM_018132_CAAGTCTACTGAATATGGAA	CENPQ__NM_018132	541	256	515	364
CENPQ__NM_018132_GAATGCTCTTCTAAAGGACT	CENPQ__NM_018132	200	239	301	479
CENPQ__NM_018132_GAGACCACAGAGTTAATGAC	CENPQ__NM_018132	502	368	591	602
CENPQ__NM_018132_TGTATCAAGTCTACTGAATA	CENPQ__NM_018132	193	107	210	164
CENPT__NM_025082_AAACAAGGACGATAGCCAGA	CENPT__NM_025082	27	8	44	12
CENPT__NM_025082_ACTGGCTTCACTACCGACTC	CENPT__NM_025082	670	425	909	469
CENPT__NM_025082_AGTGCCCACTGGCCTGAATA	CENPT__NM_025082	454	349	507	382
CENPT__NM_025082_CTCTGGCTATCGTCCTTGTT	CENPT__NM_025082	432	602	593	721
CENPT__NM_025082_CTGAAGAACATCCTACTAAC	CENPT__NM_025082	361	82	493	67
CENPT__NM_025082_GACCCCGGAGTGCTCGGGCT	CENPT__NM_025082	781	660	890	618
CENPT__NM_025082_GAGGCATCAGCATTCCCTTG	CENPT__NM_025082	601	245	689	180
CENPT__NM_025082_GGGGGAAGCCGTTTCAAGCA	CENPT__NM_025082	443	459	605	520
CENPT__NM_025082_GTGCCCACTGGCCTGAATAT	CENPT__NM_025082	547	278	524	195
CENPT__NM_025082_TCCAGACAAGAGAGCAGTTG	CENPT__NM_025082	263	281	347	398
CENPT__NM_025082_TCGGCCCATATTCAGGCCAG	CENPT__NM_025082	430	148	657	353
CENPT__NM_025082_TGGCAGATCGGCCCATATTC	CENPT__NM_025082	99	23	84	69
CENPU__NM_024629_AAATCAAGAAGTAAAGCCAT	CENPU__NM_024629	516	260	641	489
CENPU__NM_024629_AGAACCAAACGTAAAGGAAA	CENPU__NM_024629	89	99	84	122
CENPU__NM_024629_AGAAGTAAAGCCATAGGCTC	CENPU__NM_024629	11	17	6	9
CENPU__NM_024629_AGCTCAAGAACCAAACGTAA	CENPU__NM_024629	301	344	255	248
CENPU__NM_024629_ATAGCTGTGCTATGTAAAGG	CENPU__NM_024629	67	40	145	54
CENPU__NM_024629_GAAATATGCTGCATTCCTAA	CENPU__NM_024629	1011	160	1053	490
CENPU__NM_024629_GATGGTTGATATTTCGCAGA	CENPU__NM_024629	542	431	541	632
CENPU__NM_024629_GCATTGAAGAAAGTGATACA	CENPU__NM_024629	714	666	631	801
CENPU__NM_024629_GGAAAGTCGAACACGTCAAT	CENPU__NM_024629	642	272	884	860
CENPU__NM_024629_GTTACCGTTTCCTTTACGTT	CENPU__NM_024629	2279	2926	2414	3536
CENPU__NM_024629_TATATAGCTGTGCTATGTAA	CENPU__NM_024629	314	38	247	168
CENPU__NM_024629_TCTGAAAAGGAAGAATGCTA	CENPU__NM_024629	350	216	423	405
CENPV__NM_181716_AAGCCTCCGGGGTTTGATCG	CENPV__NM_181716	432	567	405	496
CENPV__NM_181716_AGAGCTCCTCGATCGCCGCA	CENPV__NM_181716	944	815	1062	1033
CENPV__NM_181716_AGGAATTCAATGGCAGCGAT	CENPV__NM_181716	79	42	54	37
CENPV__NM_181716_CAGGTCCAGGTTGGACGCCG	CENPV__NM_181716	242	260	314	371
CENPV__NM_181716_GATGGTCTTGTGCTCTTTCA	CENPV__NM_181716	161	99	157	290
CENPV__NM_181716_TATACTCCACGATCAAACCC	CENPV__NM_181716	397	332	462	417
CENPW__NM_001012507_AAAGAAGCCTCAACTTCGTC	CENPW__NM_001012507	647	204	767	173
CENPW__NM_001012507_ACTCGCACAAGCGTTTGTCC	CENPW__NM_001012507	738	385	831	340
CENPW__NM_001012507_GACTCGCTTTAGAAAGCCAC	CENPW__NM_001012507	226	22	288	42
CENPW__NM_001012507_GAGGAAGCAGATAAAGCGGA	CENPW__NM_001012507	124	89	167	37
CEP104__NM_014704_AGCGAGGACCCTGCTCTAGA	CEP104__NM_014704	1315	958	1351	1150
CEP104__NM_014704_AGCTCATCTGGACACGAAGA	CEP104__NM_014704	375	246	392	365
CEP104__NM_014704_AGGGTCTCCAATGATATTTA	CEP104__NM_014704	29	24	52	14
CEP104__NM_014704_AGGTTGGTGAACGCCTTGGG	CEP104__NM_014704	224	231	225	208
CEP104__NM_014704_CTCTAGAAGGAACGTACGCC	CEP104__NM_014704	89	103	80	220
CEP104__NM_014704_GATGGAGCAGTATCGTGCCG	CEP104__NM_014704	955	848	1034	1017
CEP104__NM_014704_GGACAATTTCTTGTGGAAAC	CEP104__NM_014704	168	247	216	309
CEP104__NM_014704_GGACTGCCAGAACGAGCGAG	CEP104__NM_014704	201	151	157	228
CEP104__NM_014704_GGTCCTCGCTGTTGTGCCCA	CEP104__NM_014704	1022	848	993	1080
CEP104__NM_014704_TCCACGCGCCAACTGTCAGT	CEP104__NM_014704	1153	1183	1220	884
CEP104__NM_014704_TGAACGCCTTGGGAGGTATG	CEP104__NM_014704	245	272	328	365
CEP104__NM_014704_TGCAGAATGCCCCACAAGAT	CEP104__NM_014704	0	0	0	0
CEP112__NM_001037325_ATTAGAAAACCGTTCTAATC	CEP112__NM_001037325	96	66	64	161
CEP112__NM_001037325_CAAGCTGAAGCAGATTGAGA	CEP112__NM_001037325	180	143	148	135
CEP112__NM_001037325_GAAGAACTGACTACATATCA	CEP112__NM_001037325	325	409	275	445
CEP112__NM_001037325_TCTCATCTCTATCAGTCTCA	CEP112__NM_001037325	317	303	299	321
CEP112__NM_001199165_ACATACCTGTAGACATCAGT	CEP112__NM_001199165	1123	1084	1301	1083
CEP112__NM_001199165_AGCAAAAGGTTCAAGCCCAG	CEP112__NM_001199165	619	370	582	554
CEP112__NM_001199165_AGTCGCCAACTGATGTCTAC	CEP112__NM_001199165	1448	1230	1604	1637
CEP112__NM_001199165_CAGGTGCAGGAATAATGGGG	CEP112__NM_001199165	338	381	424	412
CEP112__NM_001199165_CCAAATCCAGCACGAGCAAA	CEP112__NM_001199165	70	131	140	143
CEP112__NM_001199165_GCCCAGAGGGATTACCAGCC	CEP112__NM_001199165	415	614	606	946
CEP112__NM_001199165_GGCTTCTGTCTCAGGTATCG	CEP112__NM_001199165	474	723	556	764
CEP112__NM_001199165_GGTTCAGGTCGGTGTGTAAA	CEP112__NM_001199165	167	268	153	160
CEP112__NM_001199165_GTTCAGGTCGGTGTGTAAAA	CEP112__NM_001199165	94	111	134	177

CEP112__NM_001199165_TCAAGGAGTGTGATCTCACT	CEP112__NM_001199165	427	332	419	413
CEP112__NM_001199165_TTAGCAGGGAACAGTACACT	CEP112__NM_001199165	229	206	195	211
CEP112__NM_001199165_TTATTGCATATGCTTAAACG	CEP112__NM_001199165	74	110	110	60
CEP120__NM_001166226_AACCATAGGTTACATCGTTC	CEP120__NM_001166226	477	696	459	632
CEP120__NM_001166226_AATTTGCTACTGAGTTAGCT	CEP120__NM_001166226	137	199	201	379
CEP120__NM_001166226_ACTTCAGCCAAGGAAACCAT	CEP120__NM_001166226	551	604	681	696
CEP120__NM_001166226_AGTGGATAGCTTTAAAGCAA	CEP120__NM_001166226	29	77	54	50
CEP120__NM_001166226_ATCCACTGGTGGCTTTGTAT	CEP120__NM_001166226	459	439	566	353
CEP120__NM_001166226_ATCCAGAACGATGTAACCTA	CEP120__NM_001166226	340	467	448	372
CEP120__NM_001166226_ATTTGCTACTGAGTTAGCTT	CEP120__NM_001166226	96	159	134	222
CEP120__NM_001166226_CAACTGGGTAGCAAATGCTA	CEP120__NM_001166226	310	224	274	177
CEP120__NM_001166226_CAGGATCCAAGGCAAAACAT	CEP120__NM_001166226	594	585	660	512
CEP120__NM_001166226_CCTGGCTGGACTTGACCCCA	CEP120__NM_001166226	1952	1521	2006	1278
CEP120__NM_001166226_GAGGGAGGCTACCATCAGAT	CEP120__NM_001166226	757	748	834	542
CEP120__NM_001166226_GATACAGATAAGTATTGCTT	CEP120__NM_001166226	224	296	204	125
CEP120__NM_153223_AGCATATGCTTTGGACGTTT	CEP120__NM_153223	247	221	294	410
CEP120__NM_153223_ATCGTCGTGTCCATCCTAGA	CEP120__NM_153223	780	610	845	713
CEP120__NM_153223_CACGACGATGAGCAATTGGT	CEP120__NM_153223	1292	1375	1456	1229
CEP120__NM_153223_CGTTTGGGGAAATGCCGACC	CEP120__NM_153223	452	220	383	285
CEP120__NM_153223_GATGAGCAATTGGTCGGATT	CEP120__NM_153223	957	1374	987	1062
CEP120__NM_153223_GCATATGCTTTGGACGTTTG	CEP120__NM_153223	435	367	544	794
CEP120__NM_153223_TCCAAAGCATATGCTTGTAG	CEP120__NM_153223	267	224	443	236
CEP120__NM_153223_TCCACTACAAGCATATGCTT	CEP120__NM_153223	77	162	99	85
CEP120__NM_153223_TGGACACGACGATGAGCAAT	CEP120__NM_153223	88	67	134	59
CEP128__NM_152446_AGAATACAGTAATGGACAGG	CEP128__NM_152446	396	553	448	366
CEP128__NM_152446_AGTTCCCCTGTGCGTTGATC	CEP128__NM_152446	442	473	443	372
CEP128__NM_152446_CACTATAACAAGTACTTTAC	CEP128__NM_152446	331	317	365	450
CEP128__NM_152446_GCGGAAGTGATCTGATTCGC	CEP128__NM_152446	1107	1412	1229	1630
CEP128__NM_152446_GCTGCCAGATCAACGCACAG	CEP128__NM_152446	609	933	791	860
CEP128__NM_152446_GGCTGCCAGATCAACGCACA	CEP128__NM_152446	639	647	797	826
CEP128__NM_152446_GGGCTGCCAGATCAACGCAC	CEP128__NM_152446	436	269	432	517
CEP128__NM_152446_GTGACCTTGATGGTGGCACT	CEP128__NM_152446	250	271	296	318
CEP128__NM_152446_TACAGTAGAAGTTACCGAGA	CEP128__NM_152446	312	274	344	500
CEP128__NM_152446_TGGCAGCCCATGGACTCAAT	CEP128__NM_152446	248	171	201	207
CEP128__NM_152446_TTCTCAATAAACGTTGACTC	CEP128__NM_152446	1	33	22	31
CEP128__NM_152446_TTCTCGGTAACTTCTACTGT	CEP128__NM_152446	0	0	0	0
CEP131__NM_001009811_ACGGAGACAGAGCGGACGAT	CEP131__NM_001009811	409	583	440	427
CEP131__NM_001009811_ACTCCGGGCATTGGATGGCA	CEP131__NM_001009811	908	767	1084	897
CEP131__NM_001009811_AGTCAAGGGCACTGGAACTC	CEP131__NM_001009811	243	175	261	259
CEP131__NM_001009811_AGTGCCCTTGACTCACCAGC	CEP131__NM_001009811	879	471	767	499
CEP131__NM_001009811_CAGTGCCCTTGACTCACCAG	CEP131__NM_001009811	959	981	992	740
CEP131__NM_001009811_CCAACTTCACTGCTAACAAC	CEP131__NM_001009811	446	295	396	301
CEP131__NM_001009811_CCACCTGGAACGTCCTGGTA	CEP131__NM_001009811	1294	778	1380	1346
CEP131__NM_001009811_CCTTACCAGGACGTTCCAGG	CEP131__NM_001009811	433	292	390	285
CEP131__NM_001009811_CGGGCCCGCTGGTGAGTCAA	CEP131__NM_001009811	715	680	736	482
CEP131__NM_001009811_CTGGAACTCCGGGCATTGGA	CEP131__NM_001009811	344	278	385	338
CEP131__NM_001009811_CTGTTGTTAGCAGTGAAGTT	CEP131__NM_001009811	233	187	321	272
CEP131__NM_001009811_GACTTCCTGATGCTCTTCGA	CEP131__NM_001009811	466	411	632	485
CEP131__NM_014984_CAGCCACAGGTGCTTAGCCG	CEP131__NM_014984	716	512	675	674
CEP131__NM_014984_CCGGGAACACTGTCGAAGAA	CEP131__NM_014984	200	263	308	366
CEP131__NM_014984_GAACACTGTCGAAGAACGGA	CEP131__NM_014984	298	365	385	257
CEP131__NM_014984_TGCCCACGGCTAAGCACCTG	CEP131__NM_014984	278	208	268	180
CEP135__NM_025009_ACCTGTTATCAGCCACTTGA	CEP135__NM_025009	3384	2830	3950	2917
CEP135__NM_025009_AGAAATGTATCTAAACGAGA	CEP135__NM_025009	248	308	361	278
CEP135__NM_025009_AGAACGACAACTTACTCTGG	CEP135__NM_025009	997	768	967	489
CEP135__NM_025009_ATCTTCGCTGTATTATATGT	CEP135__NM_025009	610	447	696	491
CEP135__NM_025009_ATGGAATAAAACGTCGAGAC	CEP135__NM_025009	24	50	18	39
CEP135__NM_025009_CATTCAGACCAACACGTTAA	CEP135__NM_025009	347	290	488	432
CEP135__NM_025009_GAAGCGTATACGAGAGCTTA	CEP135__NM_025009	253	278	272	164
CEP135__NM_025009_GTTCATCAATTTGCATACGC	CEP135__NM_025009	49	31	92	118
CEP135__NM_025009_GTTTGAGTTTATGAGCATAT	CEP135__NM_025009	101	291	150	305
CEP135__NM_025009_TCAGCAAGCTAATAAAGACC	CEP135__NM_025009	159	98	197	123
CEP135__NM_025009_TCTGCAATGTAAGGGTCATC	CEP135__NM_025009	341	354	380	290
CEP135__NM_025009_TGCTCATAAACTCAAACTGT	CEP135__NM_025009	94	60	161	134
CEP152__NM_001194998_AACTTTAGGCCATATACCAA	CEP152__NM_001194998	172	82	261	196
CEP152__NM_001194998_ACAGGAAGTGACACATTCGA	CEP152__NM_001194998	1215	584	1260	672
CEP152__NM_001194998_AGATGAAGAGTATGACGAAG	CEP152__NM_001194998	195	184	260	235
CEP152__NM_001194998_CAGTCTTCTGCCCAGAATAA	CEP152__NM_001194998	122	83	59	55
CEP152__NM_001194998_CATCCAGCATGTCATGGGGA	CEP152__NM_001194998	705	291	803	580
CEP152__NM_001194998_CTCTCCAGAGCTCCAGTATT	CEP152__NM_001194998	340	155	335	266
CEP152__NM_001194998_GAAATTCAGAGTTTATATGC	CEP152__NM_001194998	125	78	151	103
CEP152__NM_001194998_GAAGTCCAGAACAACTGAAA	CEP152__NM_001194998	115	118	114	156
CEP152__NM_001194998_GGGCTGGTGAGCCATTATTC	CEP152__NM_001194998	847	384	916	774
CEP152__NM_001194998_TCACCAGCCCAGGAGATAAC	CEP152__NM_001194998	446	186	369	135
CEP152__NM_001194998_TGCTTCTGACCATTGGTATA	CEP152__NM_001194998	32	55	21	26
CEP152__NM_001194998_TTCTGGGCAGAAGACTGATA	CEP152__NM_001194998	0	0	0	0
CEP162__NM_001286206_AAAAATTGAGAGTAACACAG	CEP162__NM_001286206	10	0	30	41
CEP162__NM_001286206_AAAACTGGCATGCTTGCTAA	CEP162__NM_001286206	261	495	260	294
CEP162__NM_001286206_AAAGACCACAAATGAGAGTA	CEP162__NM_001286206	158	297	176	340
CEP162__NM_001286206_AACATCTCTACTATGGAATC	CEP162__NM_001286206	196	191	241	268
CEP162__NM_001286206_ATAAAATAACACCTAAGCCA	CEP162__NM_001286206	256	163	222	166
CEP162__NM_001286206_ATGACAATAAGCTTGATGTA	CEP162__NM_001286206	209	140	230	351
CEP162__NM_001286206_ATGACTGAGAATGAAATGAC	CEP162__NM_001286206	151	53	231	265
CEP162__NM_001286206_ATTCAATTTCTTAAGAGCAG	CEP162__NM_001286206	161	173	96	127
CEP162__NM_001286206_CCAAAGGAATCTATTCTGAT	CEP162__NM_001286206	1041	867	1112	1456
CEP162__NM_001286206_GTCATCAGTTAGTTCTGCGT	CEP162__NM_001286206	471	543	636	465
CEP162__NM_001286206_GTCATTTCTGGTAGGCACCT	CEP162__NM_001286206	291	266	361	429
CEP162__NM_001286206_TGTCTTCATCTCCCAATGAA	CEP162__NM_001286206	100	41	113	116
CEP162__NM_014895_AGAAGAAAGATACAGTGCCT	CEP162__NM_014895	434	259	582	541
CEP162__NM_014895_GAAGACTTCTCAGCCTGTTA	CEP162__NM_014895	395	333	482	530
CEP162__NM_014895_GTTTGAACAGTTTATGAAAG	CEP162__NM_014895	121	164	130	176
CEP162__NM_014895_TCATCTTCAGTTATCCACCA	CEP162__NM_014895	496	634	772	823
CEP164__NM_001271933_ACCTACATTCCTAGTGAGCA	CEP164__NM_001271933	1858	2046	2072	2586
CEP164__NM_001271933_ACCTTGCTCACTAGGAATGT	CEP164__NM_001271933	477	199	546	352

CEP70__NM_001288964_TGTCTATCCAAGACGGTATG	CEP70__NM_001288964	100	314	101	106
CEP70__NM_001288966_ACTGGAATCCTGGGGTTTAG	CEP70__NM_001288966	372	632	482	521
CEP70__NM_001288966_GAGTCTGTCTGATGGTTGAC	CEP70__NM_001288966	346	616	387	403
CEP70__NM_001288967_AATGAAGTTTATACTAGGCT	CEP70__NM_001288967	501	486	552	602
CEP70__NM_001288967_GGATAGACTCCATTTAAAGA	CEP70__NM_001288967	74	35	108	184
CEP70__NM_001288967_TCTGCCCACATTTCTATTAC	CEP70__NM_001288967	854	999	906	998
CEP70__NM_001288967_TGCTTCTTCAAATTAAGCCA	CEP70__NM_001288967	0	0	0	0
CEP72__NM_018140_AGCACGAGCTTCAGCCAGAA	CEP72__NM_018140	277	195	255	352
CEP72__NM_018140_CATTCAGTACCTGACTGCAT	CEP72__NM_018140	979	1029	1227	1320
CEP72__NM_018140_CATTCTCTGATGAGTTTAAC	CEP72__NM_018140	351	372	349	378
CEP72__NM_018140_CCACGAGCTCGGTTAAGGCG	CEP72__NM_018140	628	563	656	787
CEP72__NM_018140_CCTCATTGCAGAGTGCGAGT	CEP72__NM_018140	770	852	933	788
CEP72__NM_018140_GCGCAACTCCTTGGTTAGTC	CEP72__NM_018140	1486	1826	1514	2008
CEP72__NM_018140_GGAAGCAGGGCCGTGAGACG	CEP72__NM_018140	570	698	652	682
CEP72__NM_018140_GGGCGTGAGGCCGACTCTCG	CEP72__NM_018140	1248	1379	1469	1697
CEP72__NM_018140_GTTCGGGCGAAGAGCGGCTT	CEP72__NM_018140	543	406	509	541
CEP72__NM_018140_TCCGCTCGCTTGCTCTCACG	CEP72__NM_018140	109	203	80	188
CEP72__NM_018140_TGAGACTCTCCAATGCAGTC	CEP72__NM_018140	176	164	197	228
CEP72__NM_018140_TTCGGGCGAAGAGCGGCTTA	CEP72__NM_018140	60	27	37	23
CEP76__NM_001271989_AAATCCTTGCTGAGACTATA	CEP76__NM_001271989	238	483	258	275
CEP76__NM_001271989_AATCCTTGCTGAGACTATAC	CEP76__NM_001271989	307	321	315	217
CEP76__NM_001271989_AATGATTCCTCGACGTCTAA	CEP76__NM_001271989	195	204	214	212
CEP76__NM_001271989_ACTGTGGAACTTATGGGTGT	CEP76__NM_001271989	1582	1750	1943	1974
CEP76__NM_001271989_ATCAAAGCCCTTAGACGTCG	CEP76__NM_001271989	328	356	451	499
CEP76__NM_001271989_CAATGATTCCTCGACGTCTA	CEP76__NM_001271989	704	991	782	931
CEP76__NM_001271989_GCTAGGTGATGGAACTAGAA	CEP76__NM_001271989	362	393	377	308
CEP76__NM_001271989_GGAACTTATGGGTGTAGGTA	CEP76__NM_001271989	224	204	224	308
CEP76__NM_001271989_GGATTTCTCTTATTCTACCA	CEP76__NM_001271989	136	109	168	188
CEP76__NM_001271989_TGAGATAACGTTTGATTGAG	CEP76__NM_001271989	142	153	184	291
CEP76__NM_001271989_TTCTTGTATATGCTAAGCAG	CEP76__NM_001271989	0	0	0	0
CEP76__NM_001271989_TTGATTAGCACCATATGAAT	CEP76__NM_001271989	143	267	236	297
CEP76__NM_024899_ACTTGTCCAGGTAAAGGCTC	CEP76__NM_024899	84	103	104	76
CEP76__NM_024899_AGGTAAAGATACCTCCGTGT	CEP76__NM_024899	652	479	773	638
CEP76__NM_024899_CAGCTAATATTGATCCAACA	CEP76__NM_024899	277	298	232	246
CEP76__NM_024899_CTAATATTGATCCAACACGG	CEP76__NM_024899	53	74	92	49
CEP78__NM_001098802_ACATCTTTGTATCTTATCGC	CEP78__NM_001098802	907	797	899	1049
CEP78__NM_001098802_ATGGACTAATTCTGAGAGAG	CEP78__NM_001098802	86	187	142	137
CEP78__NM_001098802_CAGGTCTTTATTGATCTTGA	CEP78__NM_001098802	310	356	315	265
CEP78__NM_001098802_CCTGATCTTGACTGTATGGC	CEP78__NM_001098802	805	788	988	1254
CEP78__NM_001098802_CTGCAAAAGCACATGCACCT	CEP78__NM_001098802	1040	1228	1273	1396
CEP78__NM_001098802_GAGACCTGATCTTGACTGTA	CEP78__NM_001098802	927	1057	843	1011
CEP78__NM_001098802_GCAAATTGTCCAATTGGAGA	CEP78__NM_001098802	233	196	265	332
CEP78__NM_001098802_GGATGTAATCTGACATGGCA	CEP78__NM_001098802	387	413	462	665
CEP78__NM_001098802_GGGCTGAGAGTCTTCGCTAT	CEP78__NM_001098802	1150	653	1190	934
CEP78__NM_001098802_TCACAGGATGTAATCTGACA	CEP78__NM_001098802	205	223	234	144
CEP78__NM_001098802_TCACATGGCCAAGATCTTAA	CEP78__NM_001098802	364	330	480	525
CEP78__NM_001098802_TTGGACAATTTGCAAGAGAC	CEP78__NM_001098802	208	391	259	222
CEP83__NM_001042399_ACCATTTCCTCCTCATATGC	CEP83__NM_001042399	452	453	543	413
CEP83__NM_001042399_AGAAAACAACTAGAGGAACT	CEP83__NM_001042399	263	261	253	234
CEP83__NM_001042399_AGATGGCAGAAGAGATCACC	CEP83__NM_001042399	296	227	266	285
CEP83__NM_001042399_AGCCTTGTATAGTCTTCAAA	CEP83__NM_001042399	204	210	235	443
CEP83__NM_001042399_GCTGATGACTGAAAGCTAAC	CEP83__NM_001042399	354	508	438	683
CEP83__NM_001042399_GGCTGAAGTAGCGGAATTAA	CEP83__NM_001042399	375	575	374	472
CEP83__NM_001042399_GGTGGAGACAGTGGATTGAC	CEP83__NM_001042399	373	402	415	394
CEP83__NM_001042399_TAATTTACGGGCAGAACGTT	CEP83__NM_001042399	576	716	639	794
CEP83__NM_001042399_TGAAAATTCTGACCTAAAAC	CEP83__NM_001042399	93	117	127	223
CEP83__NM_001042399_TGAAAGTGGAACATGATGTC	CEP83__NM_001042399	421	553	412	535
CEP83__NM_001042399_TGTCAGCACATTTACCGATA	CEP83__NM_001042399	287	297	326	223
CEP83__NM_001042399_TTAAGGAGGAACAACATCAA	CEP83__NM_001042399	226	207	285	273
CEP85__NM_001281517_AACAGGTGTAGAATTTGGCT	CEP85__NM_001281517	104	39	83	76
CEP85__NM_001281517_AGGAGAAATATCCAACTGAG	CEP85__NM_001281517	131	10	93	25
CEP85__NM_001281517_ATGAGATCTCTAGATAGGCC	CEP85__NM_001281517	260	85	334	233
CEP85__NM_001281517_CCACTTGAGCTGCAAAAATC	CEP85__NM_001281517	334	356	415	578
CEP85__NM_001281517_CCCTGACAAAGGGAGTTTAG	CEP85__NM_001281517	277	114	406	243
CEP85__NM_001281517_CCTCTAAACTCCCTTTGTCA	CEP85__NM_001281517	610	429	657	408
CEP85__NM_001281517_CTAAAGTAGAAGGCATCACA	CEP85__NM_001281517	228	242	343	145
CEP85__NM_001281517_GAGAATGTTTCATTGCACCG	CEP85__NM_001281517	423	123	421	110
CEP85__NM_001281517_GCAGGAGAAATATCCAACTG	CEP85__NM_001281517	275	143	444	158
CEP85__NM_001281517_GGAGAGGTCCCTAAAGTAGA	CEP85__NM_001281517	372	321	378	324
CEP85__NM_001281517_GGGCTGTTGGAATGGTTACG	CEP85__NM_001281517	295	99	406	190
CEP85__NM_001281517_TTAGAGGAAGAGGGTCCAAC	CEP85__NM_001281517	122	51	133	95
CEP85__NM_001281518_GCAGGGCTGAATCCCTTCGC	CEP85__NM_001281518	856	501	896	706
CEP85__NM_001281518_GCGATACGATTCGCTCCAAA	CEP85__NM_001281518	244	206	297	283
CEP85__NM_001281518_GTCGTGACATTGAGGACTTA	CEP85__NM_001281518	280	16	330	25
CEP85__NM_001281518_GTGCAGCTCAAGTGCCAGCT	CEP85__NM_001281518	189	58	176	153
CEP85__NM_001281518_TCAGTTGCGCTCACAAGTAC	CEP85__NM_001281518	539	520	583	408
CEP85__NM_001281518_TGAAGGTGGAGACCCTGGTT	CEP85__NM_001281518	260	30	286	91
CEP85__NM_022778_AAAGGGCTCCGAGATAACTG	CEP85__NM_022778	158	0	211	3
CEP85__NM_022778_CGAAAGGGCTCCGAGATAAC	CEP85__NM_022778	242	27	371	44
CEP85__NM_022778_CGGCTGAAGCGGCTCCGAAA	CEP85__NM_022778	471	130	626	148
CEP85__NM_022778_GAAAGGGCTCCGAGATAACT	CEP85__NM_022778	127	0	161	6
CEP85__NM_022778_GACAGTGGGGACACAGCCAT	CEP85__NM_022778	711	361	791	372
CEP85__NM_022778_GTTCAAGTGTAGCCGACAGT	CEP85__NM_022778	232	63	215	145
CEP85__NM_022778_TGTTCAAGTGTAGCCGACAG	CEP85__NM_022778	148	88	129	67
CEP85L__NM_001042475_AAGTTTGGAAATTGAAGCGC	CEP85L__NM_001042475	128	248	133	137
CEP85L__NM_001042475_ACAAGCCTCATGCAAACATT	CEP85L__NM_001042475	704	368	684	513
CEP85L__NM_001042475_ACCTTCCACGCTATCAGAAC	CEP85L__NM_001042475	820	1042	921	1202
CEP85L__NM_001042475_AGGGAATCATTGACACCAGA	CEP85L__NM_001042475	343	365	494	479
CEP85L__NM_001042475_ATTGAAGCGCTGGAATTAGA	CEP85L__NM_001042475	261	273	302	332
CEP85L__NM_001042475_CCACTTGAAGTTGAATGATC	CEP85L__NM_001042475	380	198	460	399
CEP85L__NM_001042475_CCGCAGCTTCCCTGCCGGTA	CEP85L__NM_001042475	1391	833	1605	983
CEP85L__NM_001042475_GGAATTAGACGGCATCACAT	CEP85L__NM_001042475	571	530	674	607
CEP85L__NM_001042475_TCCTGATATGGTTATTTCGA	CEP85L__NM_001042475	252	388	362	323
CEP85L__NM_001042475_TCCTGTTCTGATAGCGTGGA	CEP85L__NM_001042475	176	189	240	269

CEP85L__NM_001042475_TGCCACAATGATTCATTAGC	CEP85L__NM_001042475	344	491	451	418
CEP85L__NM_001042475_TGTCAATGATTCCCTAAGTT	CEP85L__NM_001042475	465	329	500	431
CEP85L__NM_001178035_AATAATTGGAAGTCTACCAC	CEP85L__NM_001178035	188	148	224	144
CEP85L__NM_001178035_ATAATTGGAAGTCTACCACA	CEP85L__NM_001178035	255	189	310	271
CEP89__NM_032816_AAAGGTGCCATTGTTACATG	CEP89__NM_032816	185	100	194	270
CEP89__NM_032816_AGAGAAGAGAAAGCTCAAAG	CEP89__NM_032816	147	122	175	144
CEP89__NM_032816_CCCATAGTCCAGTGTTTCAA	CEP89__NM_032816	537	661	610	592
CEP89__NM_032816_CCTTTGAAACACTGGACTAT	CEP89__NM_032816	287	548	347	289
CEP89__NM_032816_GAGAAGGCGTCGTCACAGGA	CEP89__NM_032816	396	557	411	612
CEP89__NM_032816_GTAATTCAGGTGCAGCAACT	CEP89__NM_032816	223	163	180	163
CEP89__NM_032816_GTTTCATTGTGTTTAGTTCA	CEP89__NM_032816	220	601	286	496
CEP89__NM_032816_TCAGCTGTCATCCAGCGGCA	CEP89__NM_032816	183	266	312	261
CEP89__NM_032816_TCATCCAGCGGCAAGGAATT	CEP89__NM_032816	423	607	356	393
CEP89__NM_032816_TGAGTTCTACATTCAAACGA	CEP89__NM_032816	45	57	43	53
CEP89__NM_032816_TTCTGTGTGGCACAGCGTAC	CEP89__NM_032816	4	1	3	1
CEP89__NM_032816_TTTCGCAGATAAAGTAATTC	CEP89__NM_032816	181	123	127	202
CEP95__NM_138363_ACTGGCCTTGGACTACTTGC	CEP95__NM_138363	914	272	976	641
CEP95__NM_138363_ACTGGTGAAATCATTAGACT	CEP95__NM_138363	384	262	431	403
CEP95__NM_138363_CCAGGAGAAAATATAGTGAA	CEP95__NM_138363	253	289	229	287
CEP95__NM_138363_CGAGGCTCTGAAGGGTGGTA	CEP95__NM_138363	221	157	248	227
CEP95__NM_138363_CTACTCTTGGACAAAACACT	CEP95__NM_138363	204	332	162	128
CEP95__NM_138363_CTCCAGTAGAATTTACGGCT	CEP95__NM_138363	1322	627	1460	851
CEP95__NM_138363_GCGGAAACCCTTTCTGTGAG	CEP95__NM_138363	561	990	760	684
CEP95__NM_138363_GGAAATATTTGATGGTTTGT	CEP95__NM_138363	436	416	368	586
CEP95__NM_138363_TCCCCAGCCGTAAATTCTAC	CEP95__NM_138363	861	620	870	767
CEP95__NM_138363_TCTTCATGTGATTTAGAACT	CEP95__NM_138363	273	324	362	332
CEP95__NM_138363_TCTTGGACAAAACACTAGGA	CEP95__NM_138363	161	35	168	122
CEP95__NM_138363_TTCCAAATGCTAGGAAGCTA	CEP95__NM_138363	45	10	39	77
CEP97__NM_024548_AATCGGCTGGTTCGGATGAT	CEP97__NM_024548	501	466	535	323
CEP97__NM_024548_AATTTGCCTCATAATAGCAT	CEP97__NM_024548	264	114	234	126
CEP97__NM_024548_ATCAGTAGCTAATAATCGGC	CEP97__NM_024548	287	367	347	465
CEP97__NM_024548_GAAATGCAAACGATTAATAC	CEP97__NM_024548	286	200	352	197
CEP97__NM_024548_GACAATAATATATCCCAGAT	CEP97__NM_024548	348	174	374	172
CEP97__NM_024548_GCTAAAGGAACTAGTACATC	CEP97__NM_024548	872	270	832	296
CEP97__NM_024548_GGATCAGTGGTCAATTGGTC	CEP97__NM_024548	390	142	426	252
CEP97__NM_024548_TACACGAAGCAACGTCAGCT	CEP97__NM_024548	683	501	655	478
CEP97__NM_024548_TAGCTAATAATCGGCTGGTT	CEP97__NM_024548	360	308	488	568
CEP97__NM_024548_TCAGCTTCACAGGGTAAATT	CEP97__NM_024548	416	415	632	297
CEP97__NM_024548_TGCAAACGATTAATACAGGT	CEP97__NM_024548	489	206	443	216
CEP97__NM_024548_TTTAGATAGATCACCTATCT	CEP97__NM_024548	60	30	62	47
CEPT1__NM_001007794_AGCCCTTAATGCAAGGGTAT	CEPT1__NM_001007794	280	491	420	420
CEPT1__NM_001007794_CATCAATAGCATCCAAAGAC	CEPT1__NM_001007794	407	275	462	259
CEPT1__NM_001007794_CCAAAGACTGGTAAATGAAA	CEPT1__NM_001007794	666	598	807	845
CEPT1__NM_001007794_CCAATGATGGTGATGAGATT	CEPT1__NM_001007794	850	894	973	1035
CEPT1__NM_001007794_CTGCTTGAGCCCTTAATGCA	CEPT1__NM_001007794	242	107	291	234
CEPT1__NM_001007794_GCCCTTAATGCAAGGGTATT	CEPT1__NM_001007794	505	713	542	751
CEPT1__NM_001007794_GTTCTTCTAGCCGCTTTAGT	CEPT1__NM_001007794	35	18	82	39
CEPT1__NM_001007794_TGAGTGGGCATCGATCAACA	CEPT1__NM_001007794	331	240	376	282
CEPT1__NM_001007794_TGTCAAGACACCAACTAAAG	CEPT1__NM_001007794	0	0	0	0
CEPT1__NM_001007794_TTATGTTCTATTGTGCGCAC	CEPT1__NM_001007794	63	31	68	51
CEPT1__NM_001007794_TTCCCAATACCCTTGCATTA	CEPT1__NM_001007794	29	37	34	7
CEPT1__NM_001007794_TTTAGTTGGTGTCTTGACAA	CEPT1__NM_001007794	43	28	44	84
CER1__NM_005454_ACAGTGAGAGCAGGAGGTAT	CER1__NM_005454	652	576	723	814
CER1__NM_005454_AGGAAAGACCACACGGCACC	CER1__NM_005454	378	305	411	585
CER1__NM_005454_ATTCCTTGGCAGGAGTACGG	CER1__NM_005454	148	117	170	131
CER1__NM_005454_GAGAAGATGCTGTCCAGATT	CER1__NM_005454	650	737	679	827
CER1__NM_005454_GAGAGAAATGCATCCATCCA	CER1__NM_005454	841	1122	929	1026
CER1__NM_005454_GCTCCGTCTTCACCTTGCAC	CER1__NM_005454	909	724	1057	1078
CER1__NM_005454_GTACCAGCAGCTGAAATAAG	CER1__NM_005454	495	403	431	444
CER1__NM_005454_GTTCAGAACAACCTTTGCTT	CER1__NM_005454	352	376	335	362
CER1__NM_005454_TGAACTTTCCTCCGTGATCA	CER1__NM_005454	208	235	268	308
CERCAM__NM_001286760_AAGACGATGATGTCGTCGAA	CERCAM__NM_001286760	191	206	215	202
CERCAM__NM_001286760_AAGCCGCAGAGTCTGATTGT	CERCAM__NM_001286760	1479	1469	1655	1206
CERCAM__NM_001286760_CGGCGCTGGCGGTTCTTGGT	CERCAM__NM_001286760	1247	857	1302	1074
CERCAM__NM_001286760_CTAGTGCTTCTAAGATCAGG	CERCAM__NM_001286760	155	116	175	135
CERCAM__NM_001286760_CTGGGGGGCCGACTATATCC	CERCAM__NM_001286760	745	528	761	631
CERCAM__NM_001286760_GCACATTCATGTACCCATAA	CERCAM__NM_001286760	75	84	105	134
CERCAM__NM_001286760_GTGCTCATTGCACACGTGGA	CERCAM__NM_001286760	253	168	240	443
CERCAM__NM_001286760_TCGAAAGGCCAAGTGTAGTT	CERCAM__NM_001286760	648	586	663	606
CERCAM__NM_001286760_TCTGCGGCTTCTCATGGGGC	CERCAM__NM_001286760	1091	1184	1175	1199
CERCAM__NM_001286760_TGAGCTGAGGCCTGCATGCG	CERCAM__NM_001286760	182	167	178	200
CERCAM__NM_001286760_TGCCGGTGAAATCCCACCAG	CERCAM__NM_001286760	295	130	263	264
CERCAM__NM_001286760_TGTGCAATGAGCACCGTTAT	CERCAM__NM_001286760	921	513	990	770
CERCAM__NM_016174_ACACTCGCTGCCCCACTACC	CERCAM__NM_016174	1585	1335	1713	1483
CERCAM__NM_016174_CTGTGGTGTTGTCCACATTG	CERCAM__NM_016174	911	920	1009	1169
CERCAM__NM_016174_GTAGTGGGGCAGCGAGTGTT	CERCAM__NM_016174	433	347	485	579
CERK__NM_022766_ACGGCGATGATCTCAGATAC	CERK__NM_022766	792	627	870	746
CERK__NM_022766_AGGAGCAGCTGTGTCACTTG	CERK__NM_022766	1677	1462	2136	1600
CERK__NM_022766_AGTTACTGAACATGCTAATC	CERK__NM_022766	410	271	435	293
CERK__NM_022766_ATCTGAGATCATCGCCGTTG	CERK__NM_022766	747	707	664	612
CERK__NM_022766_CTGGTATTTATCAACCCGTT	CERK__NM_022766	511	488	591	631
CERK__NM_022766_CTTCTCCAGCATCTCCCGCA	CERK__NM_022766	758	697	1048	927
CERK__NM_022766_GTATTTATCAACCCGTTTGG	CERK__NM_022766	146	154	117	138
CERK__NM_022766_GTCAGTGGTGATGGAGGCTA	CERK__NM_022766	369	332	524	540
CERK__NM_022766_TACCAGTAAATGCTTTGGTC	CERK__NM_022766	636	512	725	447
CERK__NM_022766_TGAACATGCTAATCAGGCCA	CERK__NM_022766	777	761	987	793
CERK__NM_022766_TTAACATAGACAAATACGAC	CERK__NM_022766	187	161	208	175
CERK__NM_022766_TTGAGGAAACAGACGTTCAC	CERK__NM_022766	225	276	254	239
CERKL__NM_001030311_AACCATTGTTCGCAATACAT	CERKL__NM_001030311	690	790	924	1046
CERKL__NM_001030311_AACCTTCTCATAATAAACCT	CERKL__NM_001030311	225	181	244	361
CERKL__NM_001030311_CTCCAATGTATTGCGAACAA	CERKL__NM_001030311	190	196	305	260
CERKL__NM_001030311_CTCCGGGGCATCTTCGAGAT	CERKL__NM_001030311	672	791	784	1043
CERKL__NM_001030311_GCCCCGGAGCAGAATCCGCT	CERKL__NM_001030311	2730	3008	2934	3081
CERKL__NM_001030311_GCCGAGCGGATTCTGCTCCG	CERKL__NM_001030311	1439	1512	1413	1589

CETP__NM_000078_GCCTTCTCGCCCGTGATATC	CETP__NM_000078	482	397	422	428
CETP__NM_000078_GGAAAGACAGGTAGCAGTCA	CETP__NM_000078	736	708	681	864
CETP__NM_000078_GGAGCAGCTTATGGAAAGAC	CETP__NM_000078	385	588	397	593
CETP__NM_000078_GGCTGCTGGCGATGGACAAG	CETP__NM_000078	630	569	702	823
CETP__NM_000078_TCAGCTGTGTGTTGATCTGG	CETP__NM_000078	1322	1220	1416	1182
CETP__NM_000078_TCCTTGGCCAAGTCAAGTAT	CETP__NM_000078	216	315	254	241
CETP__NM_000078_TGTGCAACCCATACTTGACT	CETP__NM_000078	1650	1802	1852	2061
CFAP126__NM_001013625_AGCACCAGCAGTTGTACGGG	CFAP126__NM_001013625	1407	1059	1226	1030
CFAP126__NM_001013625_AGCTGGGGAGCTTGGAATTA	CFAP126__NM_001013625	308	229	325	293
CFAP126__NM_001013625_CAAATTATTGCCAACGATCG	CFAP126__NM_001013625	23	52	17	5
CFAP126__NM_001013625_CAGCAGTTGTACGGGAGGTC	CFAP126__NM_001013625	708	682	828	914
CFAP126__NM_001013625_CAGTAGATGACCACGATCGT	CFAP126__NM_001013625	647	718	666	601
CFAP126__NM_001013625_CTGACCTCCCGTACAACTGC	CFAP126__NM_001013625	470	459	655	613
CFAP126__NM_001013625_CTGAGTTTCTTCTGACTGTC	CFAP126__NM_001013625	683	676	741	962
CFAP126__NM_001013625_CTGGTTGGCACTGTAGTTAG	CFAP126__NM_001013625	231	183	337	265
CFAP126__NM_001013625_GTGTCCTGAAATCTTAGGCA	CFAP126__NM_001013625	256	211	218	236
CFAP126__NM_001013625_TTTCTGTATCCATTTGGTGA	CFAP126__NM_001013625	141	171	161	226
CFAP20__NM_013242_AGATTGAAGGGACAAATGTA	CFAP20__NM_013242	205	3	219	30
CFAP20__NM_013242_ATGCAAATTGTCGCATCCGA	CFAP20__NM_013242	321	376	241	256
CFAP20__NM_013242_CAAATCTGGGACAAAAAGGT	CFAP20__NM_013242	65	50	109	94
CFAP20__NM_013242_CATCGAGACCCTCAGAGTGC	CFAP20__NM_013242	1018	61	1102	131
CFAP20__NM_013242_CTAGCAAGTTGAACTGAATC	CFAP20__NM_013242	327	508	497	670
CFAP20__NM_013242_CTCTCCATCCTCTACAGCAT	CFAP20__NM_013242	121	5	132	64
CFAP20__NM_013242_GACTTCACACGGCGAGCATA	CFAP20__NM_013242	37	0	44	5
CFAP20__NM_013242_GCAGACCCCAAGAAGACGCT	CFAP20__NM_013242	778	386	693	461
CFAP20__NM_013242_GCAGATGAAGGGTTTGACCC	CFAP20__NM_013242	214	16	236	28
CFAP20__NM_013242_TCCCTGGTGCTAGAGATTGA	CFAP20__NM_013242	278	31	350	41
CFAP20__NM_013242_TCTGAGGGTCTCGATGTAAT	CFAP20__NM_013242	982	128	1182	152
CFAP20__NM_013242_TTCAAAAACACGTTCCAGAG	CFAP20__NM_013242	50	48	34	20
CFAP221__NM_001271049_AAAATGTAACGGTGACCGTG	CFAP221__NM_001271049	328	526	361	325
CFAP221__NM_001271049_AATCTGTCAAATGTTCTACT	CFAP221__NM_001271049	228	374	190	336
CFAP221__NM_001271049_AGCATTCTTTAGTCCTCTGC	CFAP221__NM_001271049	372	393	397	569
CFAP221__NM_001271049_ATGACTGGATAGGCATGAAT	CFAP221__NM_001271049	279	257	315	360
CFAP221__NM_001271049_CAGGAATAATTCCGGCTAAT	CFAP221__NM_001271049	507	494	460	519
CFAP221__NM_001271049_CCTCCAAAATGTATTATGCC	CFAP221__NM_001271049	574	631	644	612
CFAP221__NM_001271049_CTCAAAGTATTTGGTTTGCG	CFAP221__NM_001271049	1556	2234	1514	2046
CFAP221__NM_001271049_TAATAGTATCGCCACTCATC	CFAP221__NM_001271049	125	212	169	101
CFAP221__NM_001271049_TAGTGAGTTCATGACTGGAT	CFAP221__NM_001271049	515	473	546	378
CFAP221__NM_001271049_TCTCAAAGTATTTGGTTTGC	CFAP221__NM_001271049	624	787	701	874
CFAP221__NM_001271049_TCTCTTGAAGAACCTAGTGG	CFAP221__NM_001271049	105	59	152	128
CFAP221__NM_001271049_TGGGGTGATGCATTATTAAA	CFAP221__NM_001271049	12	1	25	19
CFAP36__NM_001282761_AAGATGGGGATGGACCAGTC	CFAP36__NM_001282761	560	613	614	743
CFAP36__NM_001282761_ACACAGGCCACCAAAATCAC	CFAP36__NM_001282761	528	641	704	810
CFAP36__NM_001282761_GAGCCATCGGTTAAGCAGTC	CFAP36__NM_001282761	481	398	523	469
CFAP36__NM_001282761_GATGTATGGGTCTTTGCAAG	CFAP36__NM_001282761	927	654	1016	1104
CFAP36__NM_001282761_GCTTCACTGGAATGCACTGT	CFAP36__NM_001282761	2573	2096	2759	2422
CFAP36__NM_001282761_GGGTAGTGGAGAGCATCGCG	CFAP36__NM_001282761	148	224	232	268
CFAP36__NM_001282761_TAGGTTGAAAAGCTGTTAGA	CFAP36__NM_001282761	238	231	130	96
CFAP36__NM_001282761_TCTCTCTTGAATTATTCGAA	CFAP36__NM_001282761	165	352	185	411
CFAP36__NM_001282761_TTACCTGACTGCTTAACCGA	CFAP36__NM_001282761	292	204	357	264
CFAP36__NM_001282761_TTCCAAGTACTGATAAGTTC	CFAP36__NM_001282761	61	58	58	176
CFAP36__NM_001282761_TTCCTGATGAATCTCTGTAT	CFAP36__NM_001282761	35	60	79	59
CFAP43__NM_025145_AACACCATTTGCTACCCTTG	CFAP43__NM_025145	1455	1782	1596	1975
CFAP43__NM_025145_ACCAGAAGGACCAAATTGAA	CFAP43__NM_025145	255	180	244	177
CFAP43__NM_025145_AGAGGAGTAACTAGCCAGGT	CFAP43__NM_025145	700	608	828	706
CFAP43__NM_025145_CAAAATGAACATTCTGCTTA	CFAP43__NM_025145	243	345	322	388
CFAP43__NM_025145_CAGTGTAGTAATGGAATTGT	CFAP43__NM_025145	862	1014	1071	1150
CFAP43__NM_025145_GATCTTTCGGCAACGACTGG	CFAP43__NM_025145	257	177	293	318
CFAP43__NM_025145_GCAGTGTAGTAATGGAATTG	CFAP43__NM_025145	482	556	563	734
CFAP43__NM_025145_GGAGAGAGGAGTAACTAGCC	CFAP43__NM_025145	384	601	453	565
CFAP43__NM_025145_GTGAAATTACCTCTAGAAGA	CFAP43__NM_025145	74	40	83	119
CFAP43__NM_025145_TGAAATTACCTCTAGAAGAT	CFAP43__NM_025145	235	128	209	177
CFAP43__NM_025145_TGAGATCTTTCGGCAACGAC	CFAP43__NM_025145	82	100	104	61
CFAP43__NM_025145_TTGGTCCTTCTGGTCAATCC	CFAP43__NM_025145	53	55	57	78
CFAP44__NM_001164496_AATGAAGGAACCAGATGATC	CFAP44__NM_001164496	224	173	273	268
CFAP44__NM_001164496_AGACTCAGATGAGGAACGTT	CFAP44__NM_001164496	681	953	669	759
CFAP44__NM_001164496_AGATGATCAGGATACTGATG	CFAP44__NM_001164496	614	554	725	574
CFAP44__NM_001164496_AGTATCGCCATATACATAGC	CFAP44__NM_001164496	39	42	42	34
CFAP44__NM_001164496_CAGATGAAACATTTACCAAA	CFAP44__NM_001164496	355	452	470	543
CFAP44__NM_001164496_CAGTTACATCAAAGAGTGAT	CFAP44__NM_001164496	314	343	248	308
CFAP44__NM_001164496_CCATCAGTATCCTGATCATC	CFAP44__NM_001164496	1417	1970	1557	1643
CFAP44__NM_001164496_CTTCACCACTGCTACTTCGC	CFAP44__NM_001164496	580	537	683	822
CFAP44__NM_001164496_GAGCTTCTTCTATGATTATA	CFAP44__NM_001164496	15	29	24	26
CFAP44__NM_001164496_GTATCGCCATATACATAGCT	CFAP44__NM_001164496	9	3	9	14
CFAP44__NM_001164496_GTGAAGGAATTGGCGTCATT	CFAP44__NM_001164496	683	818	706	701
CFAP44__NM_001164496_TTGGTTCCCAGCTATGTATA	CFAP44__NM_001164496	10	3	13	10
CFAP45__NM_012337_AGAAGGAGATCGCACGCTTG	CFAP45__NM_012337	643	716	737	703
CFAP45__NM_012337_AGCCTTATTCCTTGACCTGT	CFAP45__NM_012337	1226	1251	1337	1068
CFAP45__NM_012337_AGGAGTTTGAGCGAATCAAA	CFAP45__NM_012337	159	207	229	319
CFAP45__NM_012337_CAAGGAATAAGGCTCGCTAT	CFAP45__NM_012337	620	510	515	426
CFAP45__NM_012337_CCTCTTCTGCCGCTTCCAAC	CFAP45__NM_012337	747	614	854	570
CFAP45__NM_012337_CTCCTCAGGGCTGATGATTA	CFAP45__NM_012337	714	596	578	363
CFAP45__NM_012337_GCTCCAAGAGGAAGATCTAA	CFAP45__NM_012337	378	269	390	332
CFAP45__NM_012337_GGAGCAGATGCTGGAATATA	CFAP45__NM_012337	348	284	328	405
CFAP45__NM_012337_GGAGTTTGAGCGAATCAAAT	CFAP45__NM_012337	105	139	96	103
CFAP45__NM_012337_TGGCATGGCACTTAGCATTG	CFAP45__NM_012337	9	17	26	6
CFAP45__NM_012337_TTCCAACAGGTCAAGGAATA	CFAP45__NM_012337	20	46	25	58
CFAP45__NM_012337_TTTGAAGGGTATGCTTATCT	CFAP45__NM_012337	654	550	675	618
CFAP46__NM_001200049_ACTCAGCACGTTTATGATTT	CFAP46__NM_001200049	1087	1220	1303	1558
CFAP46__NM_001200049_AGGAAGGCCTACAGACACTT	CFAP46__NM_001200049	442	420	566	600
CFAP46__NM_001200049_ATCAGATGGTGACGATATCC	CFAP46__NM_001200049	420	337	514	489
CFAP46__NM_001200049_ATCGGCCAACCTAGGGAAAT	CFAP46__NM_001200049	386	460	348	458
CFAP46__NM_001200049_CCAGCTCCTGCGTGATGACC	CFAP46__NM_001200049	408	339	556	441
CFAP46__NM_001200049_CCGACTTCGGGGCACACATC	CFAP46__NM_001200049	1251	1402	1410	1345

CFTR__NM_000492_CTCCTTGTTATCCGGGTCAT	CFTR__NM_000492	1946	2445	2394	2988
CFTR__NM_000492_TCCTTTCCTCAAAATTGGTC	CFTR__NM_000492	345	330	350	499
CFTR__NM_000492_TTCCTATGACCCGGATAACA	CFTR__NM_000492	83	91	103	181
CFTR__NM_000492_TTCTGGAGATTTATGTTCTA	CFTR__NM_000492	23	30	21	10
CFTR__NM_000492_TTGCATTGGCACATTTCGTG	CFTR__NM_000492	823	1034	1027	1007
CGA__NM_000735_AGTAATGGGGGGTTTCAAAG	CGA__NM_000735	154	146	155	106
CGA__NM_000735_ATCGTCTTCTTGGACCTTAG	CGA__NM_000735	314	341	349	348
CGA__NM_000735_TTGCCCAGAATGCACGCTAC	CGA__NM_000735	394	290	411	357
CGGBP1__NM_001008390_AAACTCAGTGACTCGATCCA	CGGBP1__NM_001008390	637	687	819	771
CGGBP1__NM_001008390_AAAGACTCATACCAAGAGGA	CGGBP1__NM_001008390	113	175	140	133
CGGBP1__NM_001008390_AGTCAAAGACTCATACCAAG	CGGBP1__NM_001008390	9	22	14	10
CGGBP1__NM_001008390_CAAACTCAGTGACTCGATCC	CGGBP1__NM_001008390	903	1268	1185	1536
CGGBP1__NM_001008390_CATACAAAGCAGTCTTAGAA	CGGBP1__NM_001008390	167	296	346	309
CGGBP1__NM_001008390_CATTCTTCACATGGCGAGAT	CGGBP1__NM_001008390	232	284	292	188
CGGBP1__NM_001008390_CTAAGTCAGACCAGCTACGG	CGGBP1__NM_001008390	260	111	274	260
CGGBP1__NM_001008390_CTTCTGCACTTCTTGCAATG	CGGBP1__NM_001008390	788	1005	929	1045
CGGBP1__NM_001008390_GTCAACAATCTTGTGAGTTG	CGGBP1__NM_001008390	287	231	332	388
CGGBP1__NM_001008390_TAGCTGGTCTGACTTAGGTA	CGGBP1__NM_001008390	351	312	542	414
CGGBP1__NM_001008390_TGACTTGAGGTGGTCACTAA	CGGBP1__NM_001008390	25	14	35	25
CGGBP1__NM_001008390_TTAGAACGGTTTCGAGCAGG	CGGBP1__NM_001008390	257	363	254	198
CGN__NM_020770_AATCCCTTGACCTGAGAGTA	CGN__NM_020770	155	153	189	131
CGN__NM_020770_AATCTGGACTCCATGGTCTA	CGN__NM_020770	153	76	141	75
CGN__NM_020770_ATGGGCACTCTACGTCGAGG	CGN__NM_020770	498	452	732	380
CGN__NM_020770_CACACGCACAGCAACCCCGT	CGN__NM_020770	1741	1646	1587	1342
CGN__NM_020770_CTCCTTTGGGGTCCAAATCA	CGN__NM_020770	98	152	183	154
CGN__NM_020770_GAGATGGGCACTCTACGTCG	CGN__NM_020770	948	678	902	603
CGN__NM_020770_GAGTAGCTTTCCATTCCAGT	CGN__NM_020770	2086	1235	2121	1100
CGN__NM_020770_GCTCTGTGATGAAGCGAATC	CGN__NM_020770	124	95	231	259
CGN__NM_020770_GTGCGTGTGCAGGGAATCGC	CGN__NM_020770	305	444	337	324
CGN__NM_020770_TCCTGAGGCCCCTTGGTCAT	CGN__NM_020770	982	1026	1062	1313
CGN__NM_020770_TCCTTTGGGGTCCAAATCAA	CGN__NM_020770	63	37	58	76
CGN__NM_020770_TGCGTGTGCAGGGAATCGCT	CGN__NM_020770	686	690	634	586
CGNL1__NM_001252335_ACCAGCCTTCTGAGAGTAAT	CGNL1__NM_001252335	417	556	568	648
CGNL1__NM_001252335_AGATTTGGCCGGGTCTTCCA	CGNL1__NM_001252335	511	529	642	716
CGNL1__NM_001252335_AGGCTGGGAATTGCTAGGTT	CGNL1__NM_001252335	1163	1036	1118	1120
CGNL1__NM_001252335_CAACATGTGCAGCAGGAATA	CGNL1__NM_001252335	299	465	422	409
CGNL1__NM_001252335_CAGAAAACGATGTGCCTGCT	CGNL1__NM_001252335	1005	1216	1175	1404
CGNL1__NM_001252335_CTCCTACGGTGTCAGTATTC	CGNL1__NM_001252335	121	321	181	371
CGNL1__NM_001252335_GACCCGAATACTGACACCGT	CGNL1__NM_001252335	1845	1977	2112	1769
CGNL1__NM_001252335_GAGTTCTAGATCGCAAAGAC	CGNL1__NM_001252335	97	114	91	161
CGNL1__NM_001252335_TGTGTTATTCAGGACAATAT	CGNL1__NM_001252335	143	148	88	61
CGNL1__NM_001252335_TTCAGGTTTCTTATTGGGCT	CGNL1__NM_001252335	65	44	103	133
CGNL1__NM_001252335_TTGGAAGACCCGGCCAAATC	CGNL1__NM_001252335	197	139	252	206
CGNL1__NM_001252335_TTGGGCTAGGCTGGGCGTAT	CGNL1__NM_001252335	503	651	535	466
CGREF1__NM_001166239_ACTCTGGTCATAGTCATGGA	CGREF1__NM_001166239	409	324	492	419
CGREF1__NM_001166239_AGGAGGCCAAGGAACTTCCA	CGREF1__NM_001166239	209	177	198	171
CGREF1__NM_001166239_CACTCTGGTCATAGTCATGG	CGREF1__NM_001166239	805	1049	843	1009
CGREF1__NM_001166239_CGGGTTGGTGGTAGGAGAGT	CGREF1__NM_001166239	372	78	380	229
CGREF1__NM_001166239_GGCCAGAGCAGCTGTCAACA	CGREF1__NM_001166239	690	731	678	559
CGREF1__NM_001166239_GTCCACTCTGGTCATAGTCA	CGREF1__NM_001166239	183	399	280	323
CGREF1__NM_001166239_TGAGGTGCACATTGTTCAAG	CGREF1__NM_001166239	264	380	307	286
CGRRF1__NM_006568_ACCAATGGATAGCGAGATCT	CGRRF1__NM_006568	97	31	133	145
CGRRF1__NM_006568_ACCCAGATCTCGCTATCCAT	CGRRF1__NM_006568	2629	2592	2933	2763
CGRRF1__NM_006568_ACCCCCAGTAGCATGTAAGG	CGRRF1__NM_006568	565	563	550	476
CGRRF1__NM_006568_AGCCTCCTTACATGCTACTG	CGRRF1__NM_006568	660	688	696	687
CGRRF1__NM_006568_CCATCCCAATACCAGGCCGG	CGRRF1__NM_006568	2062	2031	2294	2099
CGRRF1__NM_006568_CGTGACCACCGGCCTGGTAT	CGRRF1__NM_006568	1916	1875	2149	1832
CGRRF1__NM_006568_CTTCAGCTTCAATTACAAGT	CGRRF1__NM_006568	493	551	549	488
CGRRF1__NM_006568_GCCTCCTTACATGCTACTGG	CGRRF1__NM_006568	2110	1703	2174	1676
CGRRF1__NM_006568_TACCAATGGATAGCGAGATC	CGRRF1__NM_006568	56	48	31	65
CGRRF1__NM_006568_TAGCCTCCTTACATGCTACT	CGRRF1__NM_006568	582	599	533	557
CGRRF1__NM_006568_TGCACCCCCAGTAGCATGTA	CGRRF1__NM_006568	650	548	600	754
CGRRF1__NM_006568_TTCAAGGCAATCTGTTGTCA	CGRRF1__NM_006568	112	248	137	132
CH25H__NM_003956_AAAGCCAGATGTTGACCACG	CH25H__NM_003956	804	847	777	782
CH25H__NM_003956_CAACGCAGTATATGAGCGTC	CH25H__NM_003956	547	421	595	676
CH25H__NM_003956_CAGGCAAAAGCCCACGTATG	CH25H__NM_003956	434	172	469	388
CH25H__NM_003956_CCGGGAAGAAGGGCGACTGT	CH25H__NM_003956	1253	1468	1366	1185
CH25H__NM_003956_CGAACGAGGACGAGTTCTGG	CH25H__NM_003956	1503	939	1446	1194
CH25H__NM_003956_CTGCGTTGCCAGCGCGAACG	CH25H__NM_003956	488	779	452	503
CH25H__NM_003956_CTGGTGCCCTTCGGGTGGTA	CH25H__NM_003956	72	85	149	126
CH25H__NM_003956_CTTCTCCATCACCACATACG	CH25H__NM_003956	347	411	309	288
CH25H__NM_003956_GCCCGGAGCTGCAAAGGACC	CH25H__NM_003956	466	607	763	719
CH25H__NM_003956_TCGACATGGAGTTCTTCGTG	CH25H__NM_003956	1075	1075	1166	1239
CH25H__NM_003956_TGGTGCCCTTCGGGTGGTAC	CH25H__NM_003956	31	78	25	23
CH25H__NM_003956_TTCTCCATCACCACATACGT	CH25H__NM_003956	47	17	31	47
CHAC1__NM_001142776_CGAAAATCCACAGCGCTTGA	CHAC1__NM_001142776	774	1009	773	1298
CHAC1__NM_001142776_CGCCTACAGCGACAGCCGTG	CHAC1__NM_001142776	105	111	66	187
CHAC1__NM_001142776_CTGTCGCTGTAGGCGAAGTC	CHAC1__NM_001142776	140	134	104	229
CHAC1__NM_001142776_CTTGAGGGTCGCCGTCGTTT	CHAC1__NM_001142776	2130	2503	2523	2449
CHAC1__NM_001142776_GAGGGTCGCCGTCGTTTCGG	CHAC1__NM_001142776	679	530	775	722
CHAC1__NM_001142776_GCCTACAGCGACAGCCGTGT	CHAC1__NM_001142776	1644	1601	1705	1591
CHAC1__NM_001142776_GCTCAGCTGGAGCTACCGAG	CHAC1__NM_001142776	505	512	637	543
CHAC1__NM_001142776_GCTCAGTTCCCCCGAAACGA	CHAC1__NM_001142776	386	349	358	365
CHAC1__NM_001142776_GCTGTCGCTGTAGGCGAAGT	CHAC1__NM_001142776	518	866	543	543
CHAC1__NM_001142776_GGAACTGAGCGGACGGCGTA	CHAC1__NM_001142776	407	582	449	402
CHAC1__NM_001142776_GGCCAGGTGTGTGCGTCCGT	CHAC1__NM_001142776	1546	1757	1744	2189
CHAC1__NM_001142776_TGAGGGTCGCCGTCGTTTCG	CHAC1__NM_001142776	621	772	792	1083
CHAC2__NM_001008708_ACTTCTTCTTCCTTTCCTAC	CHAC2__NM_001008708	385	426	551	532
CHAC2__NM_001008708_CAGGGCAGCACGGACCACCG	CHAC2__NM_001008708	747	537	932	777
CHAC2__NM_001008708_CCGACCAGCTTGTCCTGATA	CHAC2__NM_001008708	1397	1346	1443	1614
CHAC2__NM_001008708_GGTGCAGGACCAAGATAATC	CHAC2__NM_001008708	483	472	482	507
CHAC2__NM_001008708_TAGTAAAGGAACGTTTAGAA	CHAC2__NM_001008708	183	315	212	259
CHAC2__NM_001008708_TTACAGATTGCCAGTAGGAA	CHAC2__NM_001008708	97	95	83	71
CHAD__NM_001267_AGAGGTAGGTCAGCTCGGTC	CHAD__NM_001267	444	400	373	552

CHD9__NM_025134_ATCAGATGGCAGTCCAATGT	CHD9__NM_025134	391	356	397	321
CHD9__NM_025134_CAAGAAGTCATATTTGGGGA	CHD9__NM_025134	969	1090	1243	1372
CHD9__NM_025134_GAGGACTGAAGTAGACTGTT	CHD9__NM_025134	500	631	556	590
CHD9__NM_025134_GATGATGCATTTGTACAACC	CHD9__NM_025134	679	825	802	950
CHD9__NM_025134_GTGTTGGAGTACCTTGAACA	CHD9__NM_025134	561	601	590	922
CHD9__NM_025134_GTTTGGTGATATATGATTAC	CHD9__NM_025134	278	402	235	293
CHD9__NM_025134_TACATCTGGAAACAAACCAT	CHD9__NM_025134	458	763	554	611
CHD9__NM_025134_TCAATGATGTATTGGGGTTT	CHD9__NM_025134	699	954	846	1181
CHD9__NM_025134_TGGTTAACATTGACTCTATG	CHD9__NM_025134	5	38	0	0
CHD9__NM_025134_TTAGATGCATTAGTCATCTG	CHD9__NM_025134	178	181	182	176
CHDC2__NM_173695_AACCAGAAGATTATAAGAGG	CHDC2__NM_173695	323	387	422	413
CHDC2__NM_173695_AAGCCTGGAGTAAACGGGCA	CHDC2__NM_173695	551	635	643	703
CHDC2__NM_173695_ACTCACAGGCATCGTATTAG	CHDC2__NM_173695	863	917	1076	1217
CHDC2__NM_173695_CCAGATGAATTGCCATATAT	CHDC2__NM_173695	789	842	1009	968
CHDC2__NM_173695_CTGTAGATAACCATGAAAAA	CHDC2__NM_173695	214	273	247	325
CHDC2__NM_173695_GCAAAGGGAAACTTATTTAT	CHDC2__NM_173695	503	696	556	673
CHDC2__NM_173695_TAAGAGGTGGATTGAAATTA	CHDC2__NM_173695	91	102	38	91
CHDC2__NM_173695_TCTGGTTCAAGCAAAAATTC	CHDC2__NM_173695	65	61	42	61
CHDC2__NM_173695_TCTTACCAAGAATGGGCAAT	CHDC2__NM_173695	281	140	270	338
CHDC2__NM_173695_TTATGAAATTGACTTTGACG	CHDC2__NM_173695	181	188	158	223
CHDC2__NM_173695_TTGAACCAGAAGATTATAAG	CHDC2__NM_173695	39	15	52	80
CHDH__NM_018397_AATGCCATGGTCTACGTCCG	CHDH__NM_018397	1825	2240	1970	2359
CHDH__NM_018397_AGCATGTGGTGTCTCCTACG	CHDH__NM_018397	684	449	534	489
CHDH__NM_018397_AGTTGTACCTGTCGTCGCAC	CHDH__NM_018397	650	422	736	645
CHDH__NM_018397_ATGCCATGGTCTACGTCCGT	CHDH__NM_018397	2038	1689	2113	1975
CHDH__NM_018397_CACCCTGCTCACAAGCGTCT	CHDH__NM_018397	1337	1077	1329	1104
CHDH__NM_018397_CCATTCATGTCCTCGGTGAG	CHDH__NM_018397	460	591	656	822
CHDH__NM_018397_CGCGTGCTGTACTGGCCACG	CHDH__NM_018397	2008	1208	2167	1918
CHDH__NM_018397_GGGCCCAAGGACGTGCTCGC	CHDH__NM_018397	299	382	323	324
CHDH__NM_018397_GTACCTGTCGTCGCACAGGT	CHDH__NM_018397	515	637	446	588
CHDH__NM_018397_TGCAGGCAAACGGTGGAGCG	CHDH__NM_018397	215	260	270	358
CHDH__NM_018397_TGGACGGCCGCGTGCTGTAC	CHDH__NM_018397	312	116	316	294
CHDH__NM_018397_TTGCCCCGGGACACCCGCAG	CHDH__NM_018397	159	86	216	305
CHEK1__NM_001114121_AAATCTTCTGTTGGATGAAA	CHEK1__NM_001114121	175	188	211	260
CHEK1__NM_001114121_AGAAGTTCTGGAGCAACATA	CHEK1__NM_001114121	11	0	22	0
CHEK1__NM_001114121_AGGCCCCGAGTCACTTCAGG	CHEK1__NM_001114121	760	217	741	403
CHEK1__NM_001114121_CATAAGGAAAGACCTGTGCG	CHEK1__NM_001114121	399	151	352	119
CHEK1__NM_001114121_CCACCCCTGCCATGAGTTGA	CHEK1__NM_001114121	1589	38	1372	128
CHEK1__NM_001114121_CTTCCATCAACTCATGGCAG	CHEK1__NM_001114121	288	84	267	53
CHEK1__NM_001114121_GAGATTCTTCCATCAACTCA	CHEK1__NM_001114121	166	0	152	4
CHEK1__NM_001114121_GTACTTACTGCAATGCTCGC	CHEK1__NM_001114121	127	284	150	195
CHEK1__NM_001114121_TAAGCACATTCAATCCAATT	CHEK1__NM_001114121	612	301	644	347
CHEK1__NM_001114121_TCTCAGCCAGAACCCCGCAC	CHEK1__NM_001114121	552	121	511	216
CHEK1__NM_001114121_TGCAGTAAGTACTATTCCAC	CHEK1__NM_001114121	403	21	482	27
CHEK1__NM_001114121_TGGTATTGGAATAACTCACA	CHEK1__NM_001114121	0	0	0	0
CHEK1__NM_001274_AGAGGAGGATCGCGTGAACC	CHEK1__NM_001274	364	60	415	128
CHEK2__NM_001005735_AAGGGCCCATAATCGAGCCC	CHEK2__NM_001005735	109	274	138	205
CHEK2__NM_001005735_AATACAGAGCTTGTAGGGAA	CHEK2__NM_001005735	190	246	279	439
CHEK2__NM_001005735_AATGTGTGAATGACAACTAC	CHEK2__NM_001005735	314	283	391	307
CHEK2__NM_001005735_AGGGCCCATAATCGAGCCCA	CHEK2__NM_001005735	245	282	236	234
CHEK2__NM_001005735_CTGCCCCCTGGGCTCGATTA	CHEK2__NM_001005735	107	91	83	109
CHEK2__NM_001005735_GGACTGGGTAACGCTGCCAT	CHEK2__NM_001005735	1567	1612	1417	1359
CHEK2__NM_001005735_GGGACTGGGTAACGCTGCCA	CHEK2__NM_001005735	625	395	563	526
CHEK2__NM_001005735_GGGCCCATAATCGAGCCCAG	CHEK2__NM_001005735	193	100	227	150
CHEK2__NM_001005735_GTCTCGGGAGTCGGATGTTG	CHEK2__NM_001005735	621	658	804	688
CHEK2__NM_001005735_GTGAATGACAACTACTGGTT	CHEK2__NM_001005735	212	153	244	296
CHEK2__NM_001005735_TGAATGACAACTACTGGTTT	CHEK2__NM_001005735	327	469	325	251
CHEK2__NM_001005735_TGCCCCCTGGGCTCGATTAT	CHEK2__NM_001005735	256	473	390	476
CHEK2__NM_001257387_AAGAACTTCAGGCGCCAAGT	CHEK2__NM_001257387	298	307	313	156
CHEK2__NM_001257387_AATACCTGGGCTAGAACCTG	CHEK2__NM_001257387	402	759	483	522
CHEK2__NM_001257387_ACATAAGGTTCTCATGAGAG	CHEK2__NM_001257387	215	354	282	272
CHEK2__NM_001257387_AGGTAAAGCTGGCTTTCGAG	CHEK2__NM_001257387	594	729	763	511
CHEK2__NM_001257387_CTGAAGCCACGGGTGTCTTA	CHEK2__NM_001257387	46	49	18	3
CHEK2__NM_001257387_CTTCAGGCGCCAAGTAGGTG	CHEK2__NM_001257387	0	0	0	0
CHEK2__NM_001257387_GAGCTGTTTGACAAAGTGGT	CHEK2__NM_001257387	156	218	142	124
CHEK2__NM_001257387_GTAGGTGGGGGTTCCACATA	CHEK2__NM_001257387	63	97	80	75
CHEK2__NM_001257387_TAGGACTCAAGTGTCACTGA	CHEK2__NM_001257387	387	50	399	168
CHEK2__NM_001257387_TCTACCCCAGGTTCTAGCCC	CHEK2__NM_001257387	526	428	462	665
CHEK2__NM_001257387_TCTCTCATGAGAACCTTATG	CHEK2__NM_001257387	125	106	127	124
CHEK2__NM_001257387_TGCTGGGTATAACCGTGCTG	CHEK2__NM_001257387	558	718	752	665
CHERP__NM_006387_AAGAAGCTGGTGCAGTAGAT	CHERP__NM_006387	151	15	123	22
CHERP__NM_006387_ATCACGTTGGGTTGAAAAGT	CHERP__NM_006387	333	562	475	400
CHERP__NM_006387_CAAACTCGTTCATGTCTAGC	CHERP__NM_006387	620	908	656	862
CHERP__NM_006387_CAACCTGAAGTGTCTTAGGC	CHERP__NM_006387	695	762	747	829
CHERP__NM_006387_CCAAGTGCAAAACTCAAGCA	CHERP__NM_006387	645	602	739	724
CHERP__NM_006387_CCCAGTGCCATGACTGCCCG	CHERP__NM_006387	1963	2022	1974	2892
CHERP__NM_006387_CCCAGTTCGTGGCTCGCAAT	CHERP__NM_006387	536	54	567	168
CHERP__NM_006387_CCGAAAAGAAACGAGAATTT	CHERP__NM_006387	351	34	475	108
CHERP__NM_006387_CGAAAAGAAACGAGAATTTG	CHERP__NM_006387	661	295	708	487
CHERP__NM_006387_CTAATTCTTCGAAGGCGTAC	CHERP__NM_006387	187	250	171	194
CHERP__NM_006387_CTATAAAGGAAAACGAGCCT	CHERP__NM_006387	326	260	405	307
CHERP__NM_006387_CTCCTCGTGCTGCGTCTTGA	CHERP__NM_006387	724	443	811	684
CHERP__NM_006387_CTGAGGAGTACTCGTTGATG	CHERP__NM_006387	873	85	1149	226
CHERP__NM_006387_GAAGCTGGTGGGTGACCGTT	CHERP__NM_006387	805	971	896	777
CHERP__NM_006387_GCCCAGTTCGTGGCTCGCAA	CHERP__NM_006387	416	155	445	187
CHERP__NM_006387_GCTATAAAGGAAAACGAGCC	CHERP__NM_006387	369	579	524	466
CHERP__NM_006387_GCTTTGTCCCTACACAGCCG	CHERP__NM_006387	482	475	430	426
CHERP__NM_006387_GGAACCGCATCACGGCTGAT	CHERP__NM_006387	604	121	759	178
CHERP__NM_006387_GGCTTCCCCTCGGCTGTGTA	CHERP__NM_006387	568	726	622	710
CHERP__NM_006387_GGTGCAGCACGTCATTGATC	CHERP__NM_006387	518	18	466	50
CHERP__NM_006387_GGTGGGTGACCGTTGGGAAT	CHERP__NM_006387	276	129	272	154
CHERP__NM_006387_GTGCCCCATCAGCCGTGATG	CHERP__NM_006387	482	34	595	125
CHERP__NM_006387_TCCGAAAAGAAACGAGAATT	CHERP__NM_006387	361	281	423	280
CHERP__NM_006387_TCTGAAACCTAATTCTTCGA	CHERP__NM_006387	793	660	777	719

[illegible]

CITED4__NM_133467_AACCCGGGGCCCTGGCGTAC	CITED4__NM_133467	846	893	995	891
CITED4__NM_133467_CCCGAAGGCCCCGTACGCCA	CITED4__NM_133467	74	23	85	116
CITED4__NM_133467_CCGGAGCGCATGAGGGCCAT	CITED4__NM_133467	776	815	813	735
CITED4__NM_133467_CGACCACCTGATGCTCGCCG	CITED4__NM_133467	359	329	402	319
CITED4__NM_133467_CGCCTCCTCGTCGATGAGTT	CITED4__NM_133467	2192	2833	2531	3190
CITED4__NM_133467_GACCACCTGATGCTCGCCGA	CITED4__NM_133467	2745	2521	3136	3373
CITED4__NM_133467_GGTAGCCCTCGGCGAGCATC	CITED4__NM_133467	88	78	127	84
CIZ1__NM_001131015_AATTTGGAGTGGCCAGTTGT	CIZ1__NM_001131015	1031	1343	1136	1174
CIZ1__NM_001131015_CCGTCCTGAAAGGTTGAACT	CIZ1__NM_001131015	836	493	911	957
CIZ1__NM_001131015_CTGGGCAGGGCGGTAAATCT	CIZ1__NM_001131015	636	415	642	765
CIZ1__NM_001131015_GAACTGTTGCAAATTTGGAG	CIZ1__NM_001131015	364	200	310	310
CIZ1__NM_001131015_GCAGCATGGAGCCGTTGAGG	CIZ1__NM_001131015	219	89	226	230
CIZ1__NM_001131015_GGAAAGAACTGTTGCAAATT	CIZ1__NM_001131015	576	995	819	858
CIZ1__NM_001131015_GGCAGCTCGGACGCCTCACA	CIZ1__NM_001131015	758	681	800	658
CIZ1__NM_001131015_TCCAGAGCAAACACCAGTTG	CIZ1__NM_001131015	878	816	952	859
CIZ1__NM_001131015_TCCAGGGCAGTTACAGGTGA	CIZ1__NM_001131015	460	469	429	416
CIZ1__NM_001131015_TCCGTCCTGAAAGGTTGAAC	CIZ1__NM_001131015	261	230	361	292
CIZ1__NM_001131015_TCTGGGGGTTCCGTCCTGAA	CIZ1__NM_001131015	239	224	330	240
CIZ1__NM_001131015_TCTGTTTCGGTACTGTCATC	CIZ1__NM_001131015	535	622	568	489
CIZ1__NM_001257976_CCGCAGACATGAACCACAAC	CIZ1__NM_001257976	738	529	685	713
CKAP2__NM_001098525_AAGTCATCACAGTAATACCC	CKAP2__NM_001098525	256	246	232	265
CKAP2__NM_001098525_AATGTCATATGTTGACTTTG	CKAP2__NM_001098525	1338	1327	1419	1770
CKAP2__NM_001098525_AGGGCGCAGTCCGCATTCAA	CKAP2__NM_001098525	350	377	320	237
CKAP2__NM_001098525_AGTATGTCTTCGCTGGTCAA	CKAP2__NM_001098525	456	501	592	577
CKAP2__NM_001098525_CCCTTTCCGGATTGTAACAT	CKAP2__NM_001098525	1049	916	999	895
CKAP2__NM_001098525_CCTGTGCTTGGATCTTATCG	CKAP2__NM_001098525	1800	1359	1730	1390
CKAP2__NM_001098525_GAAGTCATCACAGTAATACC	CKAP2__NM_001098525	120	125	173	84
CKAP2__NM_001098525_GAGCAAAAATAATACAGTGG	CKAP2__NM_001098525	247	156	286	251
CKAP2__NM_001098525_GGACTGCGCCCTCTGACTCG	CKAP2__NM_001098525	680	419	760	547
CKAP2__NM_001098525_GTTACACTGCTGGTGTTTAC	CKAP2__NM_001098525	190	118	235	171
CKAP2__NM_001098525_TTATCATTAGACAATGAAGC	CKAP2__NM_001098525	62	78	84	115
CKAP2__NM_001098525_TTTAGTAGTGGCAGTCATAT	CKAP2__NM_001098525	125	118	179	169
CKAP2__NM_001286686_GCAAAGGAAGAGTGCTAAAA	CKAP2__NM_001286686	305	373	260	281
CKAP2__NM_001286686_TGAGTAACTACTGAATTAGG	CKAP2__NM_001286686	522	368	631	507
CKAP2L__NM_152515_AAGGGTCCGAATAAAGTGAG	CKAP2L__NM_152515	81	113	133	74
CKAP2L__NM_152515_AGCGGCAGCAGCGGTAGGCC	CKAP2L__NM_152515	361	284	362	458
CKAP2L__NM_152515_AGGGTCCGAATAAAGTGAGA	CKAP2L__NM_152515	103	129	142	100
CKAP2L__NM_152515_ATAGTGCTGTTCTGAAAGAT	CKAP2L__NM_152515	670	541	773	794
CKAP2L__NM_152515_CTGGTATATAATTTAGGATC	CKAP2L__NM_152515	296	134	376	376
CKAP2L__NM_152515_GAAGCTGGATCGTCCACAAC	CKAP2L__NM_152515	368	266	356	292
CKAP2L__NM_152515_GACCAGGAGTAAAACCCTCA	CKAP2L__NM_152515	292	364	501	403
CKAP2L__NM_152515_GTGAGAGGGAACCGTCCTTG	CKAP2L__NM_152515	776	697	797	768
CKAP2L__NM_152515_TGAGAGGGAACCGTCCTTGA	CKAP2L__NM_152515	1074	1245	1291	1320
CKAP2L__NM_152515_TGCTAGAAGGCTTAGAGTAT	CKAP2L__NM_152515	199	161	167	137
CKAP2L__NM_152515_TGTTGAAAACTCTTGCTAGA	CKAP2L__NM_152515	44	25	46	81
CKAP2L__NM_152515_TTGCTAGAAGGCTTAGAGTA	CKAP2L__NM_152515	51	28	78	109
CKAP4__NM_006825_AGCTGAAGTCCGATTCCCAC	CKAP4__NM_006825	1956	1747	2068	1524
CKAP4__NM_006825_AGGAGGCGGCCGACACGGAG	CKAP4__NM_006825	672	597	832	755
CKAP4__NM_006825_CACCTTCTGGAGTGATTCCA	CKAP4__NM_006825	302	309	338	314
CKAP4__NM_006825_CAGAGAAAGCTGTGAAGCAA	CKAP4__NM_006825	329	412	397	485
CKAP4__NM_006825_CTCCTTCGGCCCGTGGGAAT	CKAP4__NM_006825	441	222	415	401
CKAP4__NM_006825_GAGAAGTACCCTTCAGACTA	CKAP4__NM_006825	447	984	697	475
CKAP4__NM_006825_GAGCCCCTTTGTTTGGCCGA	CKAP4__NM_006825	757	651	721	636
CKAP4__NM_006825_GGAGCCCCTTTGTTTGGCCG	CKAP4__NM_006825	1029	1383	1096	1327
CKAP4__NM_006825_GGAGTCTGACATCTACACCG	CKAP4__NM_006825	1058	1128	1102	803
CKAP4__NM_006825_GGCACTCCAGCAAAAGAGTC	CKAP4__NM_006825	393	723	492	490
CKAP4__NM_006825_GGCTCCAGCACGTGGAGGAT	CKAP4__NM_006825	729	659	664	899
CKAP4__NM_006825_TGTCAGACTCCATAGTCTGA	CKAP4__NM_006825	55	55	27	35
CKAP5__NM_001008938_AGCTCATAAGCATCTATTTG	CKAP5__NM_001008938	355	95	338	170
CKAP5__NM_001008938_ATTTAGCTTCTAGTTCTTGT	CKAP5__NM_001008938	201	248	195	312
CKAP5__NM_001008938_CAAAGCCACCAACATGACAT	CKAP5__NM_001008938	149	9	144	48
CKAP5__NM_001008938_CCTAAAAATTTGGACCACTC	CKAP5__NM_001008938	349	398	422	474
CKAP5__NM_001008938_CTGTGGAAAGCAAGGTTAAG	CKAP5__NM_001008938	69	1	106	0
CKAP5__NM_001008938_GGACACCAATGTCATGTTGG	CKAP5__NM_001008938	1208	733	1118	746
CKAP5__NM_001008938_TATACAGGCCACTATGATCT	CKAP5__NM_001008938	274	0	176	3
CKAP5__NM_001008938_TGATCAGAAATGTGAACACA	CKAP5__NM_001008938	134	0	129	3
CKAP5__NM_001008938_TGTCACTGATTCCAATGCAG	CKAP5__NM_001008938	163	0	127	2
CKAP5__NM_001008938_TGTGGAAAGCAAGGTTAAGT	CKAP5__NM_001008938	57	0	27	0
CKAP5__NM_001008938_TTTCCTCAGTGTCTCTATAC	CKAP5__NM_001008938	68	0	79	0
CKAP5__NM_001008938_TTTGGCAACACTTTGATAAT	CKAP5__NM_001008938	216	245	235	223
CKB__NM_001823_AGCCCACGGTCATGATGTAC	CKB__NM_001823	1610	1297	1884	1354
CKB__NM_001823_AGCGCAGCTTCAGTGCGTTG	CKB__NM_001823	2228	1872	2511	2509
CKB__NM_001823_ATCATCGAGGACCGGCACGG	CKB__NM_001823	572	568	522	567
CKB__NM_001823_CAGCCCACGGTCATGATGTA	CKB__NM_001823	2023	2334	2480	2909
CKB__NM_001823_CCACGGTCATGATGTACGGG	CKB__NM_001823	1010	1057	1012	1237
CKB__NM_001823_CCGCGCCATCGAGAAGCTCG	CKB__NM_001823	283	272	296	401
CKB__NM_001823_CCGTGCCGGTCCTCGATGAT	CKB__NM_001823	4299	4489	4753	5088
CKB__NM_001823_CGCGCCAAGAGCACGCCGAG	CKB__NM_001823	17	42	10	17
CKB__NM_001823_GAGATCCTTGAACACTTCGT	CKB__NM_001823	113	97	180	187
CKB__NM_001823_GGAGTCCTACGAAGTGTTCA	CKB__NM_001823	423	298	548	635
CKB__NM_001823_GTCATGATGTACGGGTGGCC	CKB__NM_001823	458	545	502	627
CKB__NM_001823_TGCTCTTGAGCGCGTAGTAT	CKB__NM_001823	189	177	232	238
CKLF__NM_001040138_ACCACAACATTGACAGTTGG	CKLF__NM_001040138	949	1750	905	1035
CKLF__NM_001040138_CATGCTCATCGTATCTGTGT	CKLF__NM_001040138	1080	1015	1393	1227
CKLF__NM_001040138_CCAGCCGCAGCATCTTCACG	CKLF__NM_001040138	2138	1903	2397	1999
CKLF__NM_001040138_GAACCATATATTGTTATCAC	CKLF__NM_001040138	388	407	544	767
CKLF__NM_001040138_GACTTGATCGATTAATGAAG	CKLF__NM_001040138	221	245	261	329
CKLF__NM_001040138_GTGATAACAATATATGGTTC	CKLF__NM_001040138	311	367	272	393
CKLF__NM_001040138_TCCACCAACTGTCAATGTTG	CKLF__NM_001040138	379	426	477	462
CKLF__NM_016326_ACAGAAGCTTCCGGTAAATA	CKLF__NM_016326	646	710	736	804
CKLF__NM_016326_CAGAAGCTTCCGGTAAATAA	CKLF__NM_016326	432	481	474	638
CKLF__NM_016326_CAGTATGCTGTCTTGCCGAC	CKLF__NM_016326	1167	1556	1344	1654
CKLF__NM_016326_CCGACGGGGCCCTTATTTAC	CKLF__NM_016326	525	477	484	365
CKLF__NM_016326_GCAGTATGCTGTCTTGCCGA	CKLF__NM_016326	1079	1041	1028	857
CKLF-CMTM1__NM_001202509_AAGGACGCCTACCCCGAAA	CKLF-CMTM1__NM_001202509	388	450	400	274
CKLF-CMTM1__NM_001202509_AAGGCATTTACTCTATGTCG	CKLF-CMTM1__NM_001202509	304	274	296	334
CKLF-CMTM1__NM_001202509_AGAAGGCATTTACTCTATGT	CKLF-CMTM1__NM_001202509	928	691	1037	712
CKLF-CMTM1__NM_001202509_AGGCATTTACTCTATGTCGG	CKLF-CMTM1__NM_001202509	391	489	425	405
CKLF-CMTM1__NM_001202509_GAAGGCATTTACTCTATGTC	CKLF-CMTM1__NM_001202509	440	553	555	653
CKLF-CMTM1__NM_001202509_GCATTTACTCTATGTCGGG	CKLF-CMTM1__NM_001202509	794	1166	1079	1284
CKLF-CMTM1__NM_001202509_GGCATTTACTCTATGTCGG	CKLF-CMTM1__NM_001202509	1222	1432	1257	1455
CKLF-CMTM1__NM_001202509_GTGTTGCATCGATGCGTTT	CKLF-CMTM1__NM_001202509	452	516	570	685
CKM__NM_001824_CAGCAAACATAACAACCACA	CKM__NM_001824	793	767	737	816
CKM__NM_001824_CAGCTTCTTGTAGAGTTCAA	CKM__NM_001824	549	524	585	696
CKM__NM_001824_CATCATCTCGGATCGCCACG	CKM__NM_001824	1569	1361	1937	1700
CKM__NM_001824_CCATCATCTCGGATCGCCAC	CKM__NM_001824	1611	1431	1716	1717
CKM__NM_001824_CCCATCATCTCGGATCGCCA	CKM__NM_001824	1675	1139	1907	1439
CKM__NM_001824_CCGTGGCGATCCGAGATGAT	CKM__NM_001824	2711	3049	2977	3171
CKM__NM_001824_GAGTTCCTTGAAAACTTCGT	CKM__NM_001824	520	610	658	443
CKM__NM_001824_GCAGCTTCTTGTAGAGTTCA	CKM__NM_001824	606	628	778	1010
CKM__NM_001824_GGCTGCTGAGCACGTAGTTA	CKM__NM_001824	982	1206	1113	1610
CKM__NM_001824_TGAGCACGTAGTTAGGGTCC	CKM__NM_001824	486	417	569	450
CKM__NM_001824_TGTGGTTGTTATGTTTGCTG	CKM__NM_001824	233	268	211	193
CKM__NM_001824_TTGTTGTGGGTGTTACCGAA	CKM__NM_001824	329	527	465	387
CKMT1A__NM_001015001_AGACTCCAAAAGCGTGGTAC	CKMT1A__NM_001015001	109	99	131	89
CKMT1B__NM_020990_AACCTAAGACTCCAAAAACG	CKMT1B__NM_020990	109	68	129	227
CKMT1B__NM_020990_AGACTCCAAAAACGTGGTAC	CKMT1B__NM_020990	37	52	42	33
CKMT1B__NM_020990_AGGAGTCTTTACTAGGATGG	CKMT1B__NM_020990	213	76	201	217
CKMT1B__NM_020990_AGGTCCAGAGCAAGTAGAAT	CKMT1B__NM_020990	121	70	207	98
CKMT1B__NM_020990_CATAGCAAGGAGTCTTTACT	CKMT1B__NM_020990	375	321	310	265
CKMT1B__NM_020990_GCAAGGAGTCTTTACTAGGA	CKMT1B__NM_020990	456	285	554	402
CKMT2__NM_001099735_AAGCTTGGCATAAATGGCGG	CKMT2__NM_001099735	883	808	1120	825
CKMT2__NM_001099735_CACCCCTTCATAAAGACTGT	CKMT2__NM_001099735	1179	855	1121	887
CKMT2__NM_001099735_CAGGGTGATGAAGCACACAA	CKMT2__NM_001099735	536	614	650	755
CKMT2__NM_001099735_CATGCCCACAGTCTTTATGA	CKMT2__NM_001099735	2560	1193	2630	1424
CKMT2__NM_001099735_CTGGTCCAGCGTGTAGCCGT	CKMT2__NM_001099735	710	638	505	415
CKMT2__NM_001099735_GAAGCTTGGCATAAATGGCG	CKMT2__NM_001099735	243	280	250	310
CKMT2__NM_001099735_GTCATCAAACTAAGACACAA	CKMT2__NM_001099735	333	347	387	420
CKMT2__NM_001099735_GTTGCGAAGCTTGGCATAAA	CKMT2__NM_001099735	291	252	341	425
CKMT2__NM_001099735_GTTGTGTCTTAGTTTGATGA	CKMT2__NM_001099735	700	960	716	1011
CKMT2__NM_001099735_GTTTGATGACGGGGTCAAAA	CKMT2__NM_001099735	115	203	99	159
CKMT2__NM_001099735_TGCCCACAGTCTTTATGAAG	CKMT2__NM_001099735	724	905	782	616
CKMT2__NM_001099735_TTATGCCAAGCTTCGCAACA	CKMT2__NM_001099735	156	188	129	146
CKS1B__NM_001826_GCCCAAGGACATAGCCAAGC	CKS1B__NM_001826	132	11	158	2
CKS1B__NM_001826_TCTTTGGTTTCTTGGGTAGT	CKS1B__NM_001826	329	244	393	259
CKS1B__NM_001826_TTCGGACAAATACGACGACG	CKS1B__NM_001826	82	52	62	52
CKS1B__NM_001826_TTCTTTGGTTTCTTGGGTAG	CKS1B__NM_001826	0	0	0	0
CKS2__NM_001827_ACGAACACTACGAGTACCGG	CKS2__NM_001827	1171	679	1070	747
CKS2__NM_001827_CGAACACTACGAGTACCGGT	CKS2__NM_001827	788	632	863	611
CLASP1__NM_001142273_AACTCTGCTGCTAAAGATCA	CLASP1__NM_001142273	140	135	178	244
CLASP1__NM_001142273_AAGAATTTCCGTACTCGAGA	CLASP1__NM_001142273	131	91	128	140
CLASP1__NM_001142273_ACGCATTGAGTGTTGCTATA	CLASP1__NM_001142273	441	343	533	446
CLASP1__NM_001142273_CAAGTTTATCTAACATGGTC	CLASP1__NM_001142273	115	97	157	173
CLASP1__NM_001142273_CCAAGTAAGTTGCATATATG	CLASP1__NM_001142273	551	572	773	570
CLASP1__NM_001142273_CCAAGTCTAATAGACAGACT	CLASP1__NM_001142273	341	340	429	443
CLASP1__NM_001142273_CCACATATATGCAACTTACT	CLASP1__NM_001142273	573	464	514	697
CLASP1__NM_001142273_CTCCATGCGAGGCTCCATAG	CLASP1__NM_001142273	96	170	96	74
CLASP1__NM_001142273_GACAGATGCCTTCTCGAGTA	CLASP1__NM_001142273	918	972	1222	1020
CLASP1__NM_001142273_GACATGTAGGAGAACGTGTG	CLASP1__NM_001142273	831	628	876	1039
CLASP1__NM_001142273_GTATGGGACAGAATGCTTGG	CLASP1__NM_001142273	338	334	415	309
CLASP1__NM_001142273_GTGGGGAAACGATTGCAGGT	CLASP1__NM_001142273	187	173	231	140
CLASP2__NM_001207044_ACCAGGCTTCCTTGCACTGT	CLASP2__NM_001207044	1760	1054	1872	1439
CLASP2__NM_001207044_ATCAGATTTATCATTCGGGT	CLASP2__NM_001207044	278	79	354	174
CLASP2__NM_001207044_CAACATTTACGATTGTTGGA	CLASP2__NM_001207044	565	420	580	362
CLASP2__NM_001207044_CAGCAATCAGATTTATCATT	CLASP2__NM_001207044	999	585	1123	525
CLASP2__NM_001207044_GAAATCCTGCCAACAGTGCA	CLASP2__NM_001207044	171	116	203	155
CLASP2__NM_001207044_GCAGGTGGCCCTAAGGTTGG	CLASP2__NM_001207044	96	94	114	54
CLASP2__NM_001207044_GGCTTCCTTGCACTGTTGGC	CLASP2__NM_001207044	776	749	816	1319
CLASP2__NM_001207044_GTGCAATTGCTTGTTATTAA	CLASP2__NM_001207044	1094	1526	1055	1294
CLASP2__NM_001207044_GTTCCTGCACCTAAAACATC	CLASP2__NM_001207044	125	204	132	63
CLASP2__NM_001207044_TAAGGATCTTAGATCCCAGG	CLASP2__NM_001207044	81	65	70	27
CLASP2__NM_001207044_TTCCCAAAACTGTTGAAAGG	CLASP2__NM_001207044	69	78	71	44
CLASP2__NM_001207044_TTGGGAAACAAGTTTGATCA	CLASP2__NM_001207044	366	446	362	383
CLASP2__NM_015097_AAGACAGTCGACGCGCTCAC	CLASP2__NM_015097	961	901	1162	941
CLASP2__NM_015097_ACTTTGCACTTCATCAAATT	CLASP2__NM_015097	2062	2616	2322	2683
CLASP2__NM_015097_ATCAATTTGCTGATGACTAG	CLASP2__NM_015097	195	229	260	338
CLASP2__NM_015097_CCAAACAGGATACACAAATG	CLASP2__NM_015097	572	528	661	509
CLASP2__NM_015097_CGTCGACTGTCTTGCCTAGG	CLASP2__NM_015097	888	768	925	691
CLASP2__NM_015097_CTTTAAATCCTATGTAGCAA	CLASP2__NM_015097	165	210	186	182
CLASP2__NM_015097_GGCAAATATCATTTCTAATC	CLASP2__NM_015097	753	1305	989	1242
CLASP2__NM_015097_GTAGGTATCATTAATGGGAT	CLASP2__NM_015097	187	252	197	135
CLASP2__NM_015097_GTGCAGCAGAAGGACGTCGG	CLASP2__NM_015097	486	581	603	370
CLASRP__NM_001278439_AACAAACAGGCACGACTTA	CLASRP__NM_001278439	555	136	687	494
CLASRP__NM_001278439_AAGCATGAGCGGAAGCTTCG	CLASRP__NM_001278439	224	220	220	219
CLASRP__NM_001278439_CTGGTGCAGAACGACTTTGC	CLASRP__NM_001278439	406	211	598	149
CLASRP__NM_001278439_CTTCTTCTTCTCATCTTCGC	CLASRP__NM_001278439	626	630	751	1011
CLASRP__NM_001278439_GCGGAAGCTTCGAGGCATGA	CLASRP__NM_001278439	1059	1355	1302	1490
CLASRP__NM_001278439_GGCCACCGTGCTGTCCTCGT	CLASRP__NM_001278439	3326	1504	3797	2525
CLASRP__NM_001278439_GGTGGCAGATCTCAACAAAC	CLASRP__NM_001278439	180	18	175	94
CLASRP__NM_001278439_GTCCTCGTAGGTATAACCGA	CLASRP__NM_001278439	211	53	256	166
CLASRP__NM_001278439_GTCGGCCATGCCATAAGTCG	CLASRP__NM_001278439	603	465	702	641
CLASRP__NM_001278439_TGGCCGACGGTGACTTCGTC	CLASRP__NM_001278439	173	175	169	234
CLASRP__NM_001278439_TGTAACTACGAGCGCTACAG	CLASRP__NM_001278439	173	33	134	23
CLASRP__NM_001278439_TTCCATCGGTTATACCTACG	CLASRP__NM_001278439	110	1	144	101
CLASRP__NM_007056_AGCAGAGGGGGGGTGTAGTC	CLASRP__NM_007056	874	1033	944	1207
CLASRP__NM_007056_CAGAACAGGAGTCGGACGAA	CLASRP__NM_007056	180	68	279	178
CLASRP__NM_007056_CGTTCGTCCGACTCCTGTTC	CLASRP__NM_007056	1032	1107	1102	1588
CLASRP__NM_007056_GAATCGGTCAATCATGTTGT	CLASRP__NM_007056	1387	1603	1503	2458
CLC__NM_001828_AGCTGACATTAAATTTGGTC	CLC__NM_001828	400	671	454	688
CLC__NM_001828_AGGCTTGATTCTATGGTCAA	CLC__NM_001828	291	476	224	243
CLC__NM_001828_AGTGCTGCCAGATAAGTACC	CLC__NM_001828	276	161	243	235
CLC__NM_001828_CATGAACAGCCGTGAGTATG	CLC__NM_001828	768	796	884	1008
CLC__NM_001828_GCTGACATTAAATTTGGTCA	CLC__NM_001828	767	1042	705	894
CLC__NM_001828_GGTCAAAGGTGTAAGAGGAT	CLC__NM_001828	140	126	260	253
CLC__NM_001828_GTCATGAACAGCCGTGAGTA	CLC__NM_001828	555	716	529	682
CLC__NM_001828_GTTCTACTGTGACAATCAAA	CLC__NM_001828	21	53	47	38
CLC__NM_001828_TCATGAACAGCCGTGAGTAT	CLC__NM_001828	2156	2283	2634	2594
CLCA1__NM_001285_CCATGCTTCCAGATTTGTCA	CLCA1__NM_001285	1847	2269	1959	2755
CLCA1__NM_001285_CTCAATCGACTGAATCAAGC	CLCA1__NM_001285	243	414	303	561
CLCA1__NM_001285_CTGAACAACAATGGCTATGA	CLCA1__NM_001285	293	292	321	463
CLCA1__NM_001285_CTGCTTGATTCAGTCGATTG	CLCA1__NM_001285	1091	1127	1062	1337
CLCA1__NM_001285_GAGAAATTCTACTTATCCAA	CLCA1__NM_001285	167	210	249	191
CLCA1__NM_001285_GAGTGGGCTCATCTACGATG	CLCA1__NM_001285	814	683	968	673
CLCA1__NM_001285_GCTGAATGAGTGAATTACTC	CLCA1__NM_001285	267	141	271	256
CLCA1__NM_001285_GTAACAGGACTCTATGAAAA	CLCA1__NM_001285	152	135	162	160
CLCA1__NM_001285_TAGGGTTCATCATTACCTGG	CLCA1__NM_001285	144	188	151	181
CLCA1__NM_001285_TGAGTGGGCTCATCTACGAT	CLCA1__NM_001285	529	410	603	396
CLCA1__NM_001285_TGGCTGTGTTGTCATAGGAG	CLCA1__NM_001285	29	16	74	86
CLCA1__NM_001285_TTGATTCTTCACCTTCTAGA	CLCA1__NM_001285	163	193	172	134
CLCA2__NM_006536_ACAGCTGGCTACGGATCACG	CLCA2__NM_006536	915	1226	1073	959
CLCA2__NM_006536_ACTGACTGGTATGGGGCACA	CLCA2__NM_006536	963	857	989	708
CLCA2__NM_006536_CATACACCCTACAATACAGA	CLCA2__NM_006536	686	473	711	478
CLCA2__NM_006536_CATAGTGACTGACTGGTATG	CLCA2__NM_006536	1059	1101	1370	1630
CLCA2__NM_006536_CCTCTGTATTGTAGGGTGTA	CLCA2__NM_006536	970	1105	1116	1333
CLCA2__NM_006536_GAATGGGAGTTCTGAACTTA	CLCA2__NM_006536	122	103	237	186
CLCA2__NM_006536_GAGTACAGCTTCAAGACAAT	CLCA2__NM_006536	750	615	895	809
CLCA2__NM_006536_GCCGAGTGTTTGTCCATGAA	CLCA2__NM_006536	1719	2219	1931	2615
CLCA2__NM_006536_GTCATAGTGACTGACTGGTA	CLCA2__NM_006536	1977	1552	1918	1766
CLCA2__NM_006536_GTTATTATTAGCTTTCCATG	CLCA2__NM_006536	510	284	453	476
CLCA2__NM_006536_GTTCTGAACTTAAGGCAACC	CLCA2__NM_006536	110	42	103	121
CLCA2__NM_006536_TTCTCTTGGTAGCATTAAAT	CLCA2__NM_006536	0	0	0	0
CLCA4__NM_012128_ACCAAGCAGTTCACAGAATG	CLCA4__NM_012128	1807	2035	2165	2005
CLCA4__NM_012128_CTGCTTGATTCATTCGATTT	CLCA4__NM_012128	1596	1733	2164	2450
CLCA4__NM_012128_CTTGATTCATTCGATTTAGG	CLCA4__NM_012128	205	118	271	252
CLCA4__NM_012128_GCAGCAGGCACAGAACTAAG	CLCA4__NM_012128	394	369	509	465
CLCA4__NM_012128_GGAACAATGGGGTTATTCAG	CLCA4__NM_012128	95	89	78	36
CLCA4__NM_012128_TAATGAAGGAAGTATTTGAC	CLCA4__NM_012128	444	461	449	535
CLCA4__NM_012128_TACTTCGGATCCATCGAGTT	CLCA4__NM_012128	766	787	950	909
CLCA4__NM_012128_TCTGTGAACTGCTTGGTGTA	CLCA4__NM_012128	429	393	436	528
CLCA4__NM_012128_TGAGTGGGCTCACCTCCGGT	CLCA4__NM_012128	312	192	350	234
CLCA4__NM_012128_TGCTTAGTTCTTGATAAGTC	CLCA4__NM_012128	221	313	254	307
CLCA4__NM_012128_TTACCTACATATCCTCTGGG	CLCA4__NM_012128	265	214	393	272
CLCA4__NM_012128_TTTGACTTAGCACGGTAGAA	CLCA4__NM_012128	113	52	106	78
CLCC1__NM_001048210_ACTAGTCAACCCTATTTGGT	CLCC1__NM_001048210	564	355	654	221
CLCC1__NM_001048210_AGCTGTGGACATATGTACGT	CLCC1__NM_001048210	1712	1137	1916	940
CLCC1__NM_001048210_ATCAGCTTTCTGTTCAGTTT	CLCC1__NM_001048210	990	715	1226	1511
CLCC1__NM_001048210_ATCATAGTTAAGCATGTCTG	CLCC1__NM_001048210	166	53	181	94
CLCC1__NM_001048210_CATCAGCTTTCTGTTCAGTT	CLCC1__NM_001048210	1561	1719	2071	2154
CLCC1__NM_001048210_CCAGCAACAGACATTCACAA	CLCC1__NM_001048210	468	264	594	361
CLCC1__NM_001048210_GGCAAAATATGGTATTTCAG	CLCC1__NM_001048210	397	266	391	216
CLCC1__NM_001048210_TCAGGCTGAAGTCGCCAAGA	CLCC1__NM_001048210	226	140	226	94
CLCC1__NM_001048210_TGTGTGCCAAAAAGATGGAC	CLCC1__NM_001048210	178	72	146	86
CLCC1__NM_001048210_TTGGCACACACATTGTTTAA	CLCC1__NM_001048210	178	104	157	190
CLCC1__NM_001048210_TTGGCATGGGTCATCCTTAT	CLCC1__NM_001048210	595	365	505	344
CLCC1__NM_001048210_TTGGTGGGACCAACCAAATA	CLCC1__NM_001048210	0	0	0	0
CLCC1__NM_001278202_ACGGAGCCATTGAAGCATAT	CLCC1__NM_001278202	299	161	351	107
CLCC1__NM_001278202_CAATGGCTCCGTTACAAATG	CLCC1__NM_001278202	624	418	631	289
CLCC1__NM_001278202_TCTTCCAGTGCTGATAATTA	CLCC1__NM_001278202	216	102	247	61
CLCC1__NM_001278203_AATGCCATAATTATCAGCAC	CLCC1__NM_001278203	278	159	466	123
CLCC1__NM_001278203_CATGTGCTGAGACATATAGG	CLCC1__NM_001278203	448	283	507	200
CLCC1__NM_001278203_TCCCGTGGCCGAAGTGCCTG	CLCC1__NM_001278203	170	109	254	193
CLCC1__NM_001278203_TCCTGCCGTCTTCTATCCCG	CLCC1__NM_001278203	2401	2080	2905	1630
CLCC1__NM_001278203_TGATGCCGATTTCCATTATA	CLCC1__NM_001278203	618	740	615	826
CLCC1__NM_001278203_TTCGGCCACGGGATAGAAGA	CLCC1__NM_001278203	55	49	83	78
CLCF1__NM_001166212_AAGTCTGGCTCGTTGAAAGG	CLCF1__NM_001166212	167	231	131	87
CLCF1__NM_001166212_CACCTTCTGTGTTACTTGCG	CLCF1__NM_001166212	1248	1689	1255	1677
CLCF1__NM_001166212_CAGTGGCAGCCTGACGGTTG	CLCF1__NM_001166212	895	1283	983	952
CLCF1__NM_001166212_CCACACCTCCAAGTCAACAG	CLCF1__NM_001166212	691	740	838	829
CLCF1__NM_001166212_CTTGCGTGGCCTCAACCGTC	CLCF1__NM_001166212	798	878	1081	844
CLCF1__NM_001166212_GGCTGTAGGCCTCGTAGTTC	CLCF1__NM_001166212	835	998	774	1150
CLCF1__NM_001166212_GTGCCAGCTCTCAATCGCAC	CLCF1__NM_001166212	350	389	508	458
CLCF1__NM_001166212_TGAAGTCTGGCTCGTTGAAA	CLCF1__NM_001166212	68	139	100	80
CLCF1__NM_001166212_TGCCAGCTCTCAATCGCACA	CLCF1__NM_001166212	827	989	942	879
CLCF1__NM_001166212_TTGAAGTCTGGCTCGTTGAA	CLCF1__NM_001166212	219	236	211	277
CLCF1__NM_013246_CCATGGACCTCCGAGCAGGT	CLCF1__NM_013246	999	1148	1049	1139
CLCN1__NM_000083_AAAGGGCATATACTGGTACT	CLCN1__NM_000083	385	459	583	586
CLCN1__NM_000083_AGTGTTCAAAGGGCATATAC	CLCN1__NM_000083	348	405	350	363
CLCN1__NM_000083_CCAGGCGGTGGATACAATCT	CLCN1__NM_000083	560	580	699	607
CLCN1__NM_000083_CCTAGATTGTATCCACCGCC	CLCN1__NM_000083	934	977	976	1458
CLCN1__NM_000083_CCTGTGTGGGGTGGACGTTG	CLCN1__NM_000083	1667	1994	1899	2046
CLCN1__NM_000083_CTTGCTGTCCACGGTAGAAC	CLCN1__NM_000083	556	645	583	676
CLCN1__NM_000083_GAAGAAAATTAGGGGAAGAC	CLCN1__NM_000083	183	215	193	276
CLCN1__NM_000083_GATCACTATTCTAAATGTCA	CLCN1__NM_000083	595	818	831	848
CLCN1__NM_000083_GGTGGTGAGAAGAAAATTAG	CLCN1__NM_000083	73	47	49	68
CLCN1__NM_000083_GTAGCTGGTGCAGTGTTCAA	CLCN1__NM_000083	464	488	471	529
CLCN1__NM_000083_TGGGGTGGACGTTGTGGCGG	CLCN1__NM_000083	227	324	195	261
CLCN1__NM_000083_TGTGGGGTGGACGTTGTGGC	CLCN1__NM_000083	359	368	381	385
CLCN2__NM_001171087_AAGTTCCTAGTATCCAGGGT	CLCN2__NM_001171087	602	825	691	628
CLCN2__NM_001171087_AGCAGCTTCCTCTTTGGCAA	CLCN2__NM_001171087	1109	1666	1329	1796
CLCN2__NM_001171087_CCACAAGTTCCTAGTATCCA	CLCN2__NM_001171087	887	1084	972	893
CLCN2__NM_001171087_CCCCAAGGTCCTGAGTGTAC	CLCN2__NM_001171087	811	919	1074	897
CLCN2__NM_001171087_GAAGCTGCTCGGATTCGCCT	CLCN2__NM_001171087	2721	3482	3084	3674
CLCN2__NM_001171087_GAGTGTACCGGCCATACATC	CLCN2__NM_001171087	1057	1024	1152	1194
CLCN2__NM_001171087_GATCCAATCTTCACCAACCC	CLCN2__NM_001171087	450	638	492	415
CLCN2__NM_001171087_GATGTATGGCCGGTACACTC	CLCN2__NM_001171087	1302	1365	1507	1234
CLCN2__NM_001171087_GCCACAAGTTCCTAGTATCC	CLCN2__NM_001171087	512	361	643	812
CLCN2__NM_001171087_GCTGCTCGGATTCGCCTGGG	CLCN2__NM_001171087	1383	1604	1726	1740
CLCN2__NM_001171087_GGAAGCTGCTCGGATTCGCC	CLCN2__NM_001171087	1063	1315	1366	1855
CLCN2__NM_001171087_GGGGGACCTTTCCAGGGTTC	CLCN2__NM_001171087	526	788	570	653
CLCN2__NM_001171088_ACTCAAGACCTTTATAGCTA	CLCN2__NM_001171088	215	276	304	329
CLCN2__NM_001171088_CCCAATGACCTTAGCTATAA	CLCN2__NM_001171088	500	721	481	759
CLCN2__NM_001171088_GACAACAGGGTAGGTGACCC	CLCN2__NM_001171088	216	297	178	290
CLCN2__NM_001171088_GAGATGAAGACCATCTTGCG	CLCN2__NM_001171088	835	818	981	974
CLCN3__NM_001243372_AGACAGAGAAAGGCATAGAC	CLCN3__NM_001243372	81	118	134	93
CLCN3__NM_001243372_AGTTTGTATGATGCGTGGTC	CLCN3__NM_001243372	278	221	318	310
CLCN3__NM_001243372_CATTATACAATGACAAATGG	CLCN3__NM_001243372	219	120	267	217
CLCN3__NM_001243372_CTCGCACCCAATCAATAGTA	CLCN3__NM_001243372	918	1051	1023	1474
CLCN3__NM_001243372_GCATTTGCCTTAGTGCGTTG	CLCN3__NM_001243372	648	772	748	902
CLCN3__NM_001243372_GGTTGTACCACAACGCACTA	CLCN3__NM_001243372	103	113	136	224
CLCN3__NM_001243372_TAGTTCATGATATAAGAACC	CLCN3__NM_001243372	37	24	42	19
CLCN3__NM_001243372_TCTGAGCAGCTGTTCCATAG	CLCN3__NM_001243372	303	345	397	404
CLCN3__NM_001243372_TGCAAGAAAGGCAAAACTCA	CLCN3__NM_001243372	464	656	573	668
CLCN3__NM_001243372_TGGAGCAAATACCTTTACCA	CLCN3__NM_001243372	231	404	263	207
CLCN3__NM_001243372_TGGCTAGTAGTAACACTAAC	CLCN3__NM_001243372	31	20	83	92
CLCN3__NM_001243372_TTGGATGACTGACCTAAAGG	CLCN3__NM_001243372	88	142	87	97
CLCN3__NM_001243374_ACCCTTATTTGCCTTATGAC	CLCN3__NM_001243374	1376	1502	1461	1729
CLCN3__NM_001243374_AGGAGACAGTATTCCCCTGA	CLCN3__NM_001243374	1515	1327	1762	1678
CLCN3__NM_001243374_CCCCCGTCATAAGGCAAATA	CLCN3__NM_001243374	471	641	584	703
CLCN3__NM_001243374_CCCCGTCATAAGGCAAATAA	CLCN3__NM_001243374	283	219	239	338
CLCN3__NM_001243374_CCCTTATTTGCCTTATGACG	CLCN3__NM_001243374	927	904	961	1045
CLCN3__NM_001243374_CCTTATTTGCCTTATGACGG	CLCN3__NM_001243374	852	1087	1027	1237
CLCN3__NM_001243374_CTGTCTCCTCCCCCGTCATA	CLCN3__NM_001243374	683	686	905	894
CLCN3__NM_001243374_GTCATAAGGCAAATAAGGGT	CLCN3__NM_001243374	1113	948	1107	1323
CLCN4__NM_001256944_ACGAGACGGCTATTCCCAAA	CLCN4__NM_001256944	1031	1039	1091	1113
CLCN4__NM_001256944_ATGGTGCAAATACACGCACC	CLCN4__NM_001256944	231	266	267	366
CLCN4__NM_001256944_ATGTGGAATACCACACGCCC	CLCN4__NM_001256944	309	290	317	345
CLCN4__NM_001256944_CGTGACTGTCTTGATTAGCA	CLCN4__NM_001256944	1515	1711	1693	1995
CLCN4__NM_001256944_CTCCGACCATTTCTGCCACA	CLCN4__NM_001256944	1879	2541	2253	3046
CLCN4__NM_001256944_CTGAAAAGCACGCCTCCAAT	CLCN4__NM_001256944	1514	1877	1719	1858
CLCN4__NM_001256944_CTGCTCATGGCTATACCAGA	CLCN4__NM_001256944	503	419	559	547
CLCN4__NM_001256944_GGAAGGGCCGCTAGTGCACG	CLCN4__NM_001256944	810	1070	787	882
CLCN4__NM_001256944_GGACTGGATGACGGACCTGA	CLCN4__NM_001256944	931	1042	732	935
CLCN4__NM_001256944_GTAATTCAGAATGTAAGCAC	CLCN4__NM_001256944	985	960	1125	1330
CLCN4__NM_001256944_TCATCCGCTGCAACATCGCC	CLCN4__NM_001256944	1311	1706	1599	2109
CLCN4__NM_001256944_TGCACCAGGCGATGTTGCAG	CLCN4__NM_001256944	803	965	918	1142
CLCN4__NM_001830_ACTGGCTAAGGGAAAAGTCA	CLCN4__NM_001830	519	385	535	505
CLCN4__NM_001830_AGCCTGCTGGATGCCTGGTC	CLCN4__NM_001830	2052	2238	2495	2978
CLCN4__NM_001830_AGGACTTCCACACCATCGAC	CLCN4__NM_001830	1351	1504	1643	1829
CLCN4__NM_001830_ATGAGCCGTTCCCTGATGTG	CLCN4__NM_001830	1409	1431	1569	1349
CLCN4__NM_001830_CATCAGGGAACGGCTCATCG	CLCN4__NM_001830	1560	1804	1922	2131
CLCN4__NM_001830_CCACACCATCGACTGGCTAA	CLCN4__NM_001830	1586	2252	1818	1927
CLCN4__NM_001830_GAGCCTGCTGGATGCCTGGT	CLCN4__NM_001830	1773	1939	1935	2239
CLCN4__NM_001830_GCAAGAGCAAGGAGTCCATA	CLCN4__NM_001830	332	349	499	538
CLCN4__NM_001830_GCTCTTGATGAACTCCCATA	CLCN4__NM_001830	720	688	708	920
CLCN4__NM_001830_TACGTCCCCACATCAGGGAA	CLCN4__NM_001830	572	539	702	653
CLCN4__NM_001830_TCCACACCATCGACTGGCTA	CLCN4__NM_001830	1462	2275	1539	1925
CLCN5__NM_000084_AATGTCCAGAGTGGAATAGT	CLCN5__NM_000084	550	644	735	722
CLCN5__NM_000084_AGGAGCCAATCCCTGGTGTA	CLCN5__NM_000084	1147	1395	1275	1418
CLCN5__NM_000084_ATAATTGACTATGTAGGCAA	CLCN5__NM_000084	170	337	211	176
CLCN5__NM_000084_ATCACTCACACTGTGAATTA	CLCN5__NM_000084	718	847	865	1103
CLCN5__NM_000084_ATCTGTGCTGATGATAAGCT	CLCN5__NM_000084	170	245	294	350
CLCN5__NM_000084_GAGAGAGAAGTCTCGAGACC	CLCN5__NM_000084	469	513	494	599
CLCN5__NM_000084_TAAAAGAAGGTATATGCACA	CLCN5__NM_000084	302	554	279	285
CLCN5__NM_000084_TAAAAGCAAAGAGTCAACAT	CLCN5__NM_000084	311	165	389	414
CLCN5__NM_000084_TCATCATAGGTCCCTACACC	CLCN5__NM_000084	530	634	469	578
CLCN5__NM_000084_TGGATGACAGACTTAAAAGA	CLCN5__NM_000084	50	55	41	43
CLCN5__NM_000084_TTAAAAGAAGGTATATGCAC	CLCN5__NM_000084	70	75	57	143
CLCN5__NM_000084_TTAACCATGAACATTGTTGC	CLCN5__NM_000084	390	316	277	315
CLCN5__NM_001127898_ACAGGTGCCATGGATAACAG	CLCN5__NM_001127898	196	154	150	200
CLCN5__NM_001127898_CCTACTTCTCGGTCTAAGGG	CLCN5__NM_001127898	348	480	391	229
CLCN5__NM_001127898_CCTCCTACTTCTCGGTCTAA	CLCN5__NM_001127898	789	881	1027	1248
CLCN5__NM_001127898_GAGAAATCCATAGCGGTTGC	CLCN5__NM_001127898	139	305	194	226
CLCN6__NM_001256959_ACAGAAAGGTCGAAGATATG	CLCN6__NM_001256959	380	289	511	477
CLCN6__NM_001256959_ACCTGTCGCTTCGGAAATAG	CLCN6__NM_001256959	640	731	861	976
CLCN6__NM_001256959_AGTTTCAGAGCATCTCCTTA	CLCN6__NM_001256959	766	538	736	542
CLCN6__NM_001256959_CCCCATGATCCACAGTGGTT	CLCN6__NM_001256959	1263	1190	1097	1081
CLCN6__NM_001256959_CTGAACAAGGTACCCCCGAT	CLCN6__NM_001256959	475	490	450	480
CLCN6__NM_001256959_CTGAATGGCGTAAAGGTGCC	CLCN6__NM_001256959	175	464	275	346
CLCN6__NM_001256959_GAAAGGTCGAAGATATGAGG	CLCN6__NM_001256959	152	249	191	135
CLCN6__NM_001256959_GAGGTCAAATGCTATCTGAA	CLCN6__NM_001256959	267	179	324	227
CLCN6__NM_001256959_GCAGCTTTCGGGGCGCCAAT	CLCN6__NM_001256959	746	854	851	988
CLCN6__NM_001256959_TACCTGTCGCTTCGGAAATA	CLCN6__NM_001256959	544	414	616	544
CLCN6__NM_001256959_TCCCCTATTTCCGAAGCGAC	CLCN6__NM_001256959	81	42	145	122
CLCN6__NM_001256959_TTTGCCATTGGAGTCTGCAC	CLCN6__NM_001256959	220	144	270	157
CLCN7__NM_001114331_AACGCCGCCTTCGTGCTCGT	CLCN7__NM_001114331	1462	1426	1511	1717
CLCN7__NM_001114331_ACAGGAGCTTCTCGTTGTGT	CLCN7__NM_001114331	1189	1633	1327	1453
CLCN7__NM_001114331_CACAGAGCCCACGAGCACGA	CLCN7__NM_001114331	1054	1443	1250	1769
CLCN7__NM_001114331_CATGGCCAACGTCTCTAAGA	CLCN7__NM_001114331	122	40	110	50
CLCN7__NM_001114331_CGCCGCTGCTGAACGGGGCT	CLCN7__NM_001114331	2034	2288	2376	3343
CLCN7__NM_001114331_CTCAAGTACAGGGTCATCAA	CLCN7__NM_001114331	456	551	583	612
CLCN7__NM_001114331_GACAGGAGCTTCTCGTTGTG	CLCN7__NM_001114331	586	731	733	884
CLCN7__NM_001114331_GACGGTGGAGATCAAGCGCT	CLCN7__NM_001114331	195	177	292	288
CLCN7__NM_001114331_GGACACCTTCTTAGAGACGT	CLCN7__NM_001114331	850	985	1160	1075
CLCN7__NM_001114331_GGAGCGGCGGATCAATCACA	CLCN7__NM_001114331	1309	1201	1475	1529
CLCN7__NM_001114331_GTGTGATCCTGTCCGTGGTC	CLCN7__NM_001114331	791	1125	737	880
CLCN7__NM_001114331_TTGAGGAAGCACTTGATCTG	CLCN7__NM_001114331	634	536	670	536
CLCN7__NM_001287_AGTCGGACATATGAGCAGCG	CLCN7__NM_001287	765	902	648	795
CLCN7__NM_001287_CGCTGCTCATATGTCCGACT	CLCN7__NM_001287	2087	2578	2190	2849
CLCNKA__NM_001042704_ACCAATCCCATCATGCCCGG	CLCNKA__NM_001042704	1033	622	1265	963
CLCNKA__NM_001042704_ACTTCTCTGTCCGGGATTAC	CLCNKA__NM_001042704	537	378	613	585
CLCNKA__NM_001042704_CAAACTGCTGGCTACTAGGT	CLCNKA__NM_001042704	1123	1070	1331	1318
CLCNKA__NM_001042704_CAGTTTGGAGCTGTACCGAT	CLCNKA__NM_001042704	2053	2014	2301	2369
CLCNKA__NM_001042704_CATGTTGGCGGGTGTGATCT	CLCNKA__NM_001042704	1599	1697	1818	1928
CLCNKA__NM_001042704_CCAATCCCATCATGCCCGGG	CLCNKA__NM_001042704	2848	2462	3152	2979
CLCNKA__NM_001042704_CGTCAGGGTCACTGGTTCCG	CLCNKA__NM_001042704	2948	2487	3458	2886
CLCNKA__NM_001042704_GCCATCGGGTGTGTGGTCCG	CLCNKA__NM_001042704	1889	2104	1926	1854
CLCNKA__NM_001042704_GGTCTCTGAGAATAGCGTCA	CLCNKA__NM_001042704	194	118	238	195
CLCNKA__NM_001042704_GTCAGGGTCACTGGTTCCGT	CLCNKA__NM_001042704	1210	1074	1405	1015
CLCNKA__NM_001042704_GTGCTCCACCCTCACATGGT	CLCNKA__NM_001042704	121	107	115	100
CLCNKA__NM_001042704_TGCCAGGAGCCGGAATATGA	CLCNKA__NM_001042704	447	262	507	415
CLCNKB__NM_000085_CCCAGAGACTTCCCTGCATG	CLCNKB__NM_000085	1816	1383	2049	1448
CLCNKB__NM_000085_CCTGGTGGGCATAGTGCGAA	CLCNKB__NM_000085	141	68	119	102
CLCNKB__NM_000085_CGCATCCGCCGAGGCATCCG	CLCNKB__NM_000085	1941	2555	1890	2355
CLCNKB__NM_000085_CGGATGCCTCGGCGGATGCG	CLCNKB__NM_000085	878	1384	1035	1245
CLCNKB__NM_000085_GCCCCGCGACGTCACAAAGA	CLCNKB__NM_000085	799	636	786	545
CLCNKB__NM_000085_GTGCTCCACCCTCACGCGGT	CLCNKB__NM_000085	168	315	188	176
CLCNKB__NM_000085_TCCTGGTGGGCATAGTGCGA	CLCNKB__NM_000085	108	108	196	248
CLCNKB__NM_000085_TCGGATGCCTCGGCGGATGC	CLCNKB__NM_000085	538	525	530	712
CLCNKB__NM_000085_TGAAGTGCTCCACCCTCACG	CLCNKB__NM_000085	858	682	1006	661
CLCNKB__NM_000085_TGTACAGCCACTGGTGCGCT	CLCNKB__NM_000085	432	560	487	573
CLCNKB__NM_000085_TTCCCTCTTTGTGACGTCGC	CLCNKB__NM_000085	83	111	99	54
CLCNKB__NM_001165945_AGGGAGGAGGTGACATGGGG	CLCNKB__NM_001165945	343	199	396	206
CLCNKB__NM_001165945_GGGCGACACTGATCTCAGGT	CLCNKB__NM_001165945	1430	1725	1702	2200
CLCNKB__NM_001165945_GTCAGGGAGGAGGTGACATG	CLCNKB__NM_001165945	370	357	464	465
CLDN1__NM_021101_ACCGCCCAGGCCATGTACGA	CLDN1__NM_021101	1924	2651	1959	2010
CLDN1__NM_021101_CCGCCCAGGCCATGTACGAG	CLDN1__NM_021101	316	333	336	267
CLDN1__NM_021101_CTATGACCCCAGTCAATGCC	CLDN1__NM_021101	168	149	226	251
CLDN1__NM_021101_GGGACAGGAACAGCAAAGTA	CLDN1__NM_021101	59	167	139	272
CLDN1__NM_021101_GGGAGTGATAGCAATCTTTG	CLDN1__NM_021101	656	815	614	813
CLDN10__NM_001160100_ACATGTCCAGGGCGCAGATC	CLDN10__NM_001160100	360	573	463	593
CLDN10__NM_001160100_AGCCGCTCTGTTTATTGGAT	CLDN10__NM_001160100	1117	1290	1363	1768
CLDN10__NM_001160100_CAAAGCTAAAATTGCTTGTT	CLDN10__NM_001160100	1009	1217	1115	1610
CLDN10__NM_001160100_CACTTTCCACTCATTGGACG	CLDN10__NM_001160100	1027	1563	1062	1465
CLDN10__NM_001160100_CAGACCCTGGTAAACCCAAG	CLDN10__NM_001160100	166	153	174	204
CLDN10__NM_001160100_CATTCCAAAGAGCGCAAATA	CLDN10__NM_001160100	199	236	171	240
CLDN10__NM_001160100_GCCTCACTGTGCATAATTGG	CLDN10__NM_001160100	2156	2143	2476	2744
CLDN10__NM_001160100_GCTAAAATTGCTTGTTTGGC	CLDN10__NM_001160100	1183	1769	1111	1595
CLDN10__NM_001160100_GCTCTGGTGTCTGGTGTCGG	CLDN10__NM_001160100	1345	1388	1219	1589
CLDN10__NM_001160100_GCTCTGTTTATTGGATGGGC	CLDN10__NM_001160100	236	324	264	351
CLDN10__NM_001160100_GGAATGAAGTGTACCAAAGT	CLDN10__NM_001160100	314	357	423	267
CLDN10__NM_001160100_GGTCTGTGGATGAACTGCGC	CLDN10__NM_001160100	190	192	214	186
CLDN10__NM_006984_AGACACCTTCCAGTAGTCGG	CLDN10__NM_006984	448	485	432	387
CLDN10__NM_006984_CAGGTTGGCCCAATAGGTGG	CLDN10__NM_006984	175	232	156	117
CLDN10__NM_006984_CATCACAACCGCCACCTATT	CLDN10__NM_006984	2004	1974	2025	1876
CLDN10__NM_006984_CCACAGGTTGGCCCAATAGG	CLDN10__NM_006984	87	120	120	84
CLDN10__NM_006984_GCTGCCCACCGACTACTGGA	CLDN10__NM_006984	1054	1256	1120	1079
CLDN10__NM_006984_GGTAGACACCTTCCAGTAGT	CLDN10__NM_006984	375	353	434	421
CLDN10__NM_006984_GGTGTCTACCATCGACGGCA	CLDN10__NM_006984	262	287	275	262
CLDN10__NM_006984_TCATCACAACCGCCACCTAT	CLDN10__NM_006984	1061	1057	1501	1385
CLDN10__NM_006984_TGGAAGGTGTCTACCATCGA	CLDN10__NM_006984	111	189	177	194
CLDN10__NM_006984_TGTGATGACCGTGCCGTCGA	CLDN10__NM_006984	580	651	563	629
CLDN10__NM_182848_AACTGCGCAGGTAACGCGTT	CLDN10__NM_182848	1163	1209	1167	1104
CLDN10__NM_182848_GAACTGCGCAGGTAACGCGT	CLDN10__NM_182848	747	834	926	810
CLDN10__NM_182848_TTTACTATCTTCAAAGTAGC	CLDN10__NM_182848	0	0	0	0
CLDN11__NM_001185056_AGCCCGGTGTGGCTAAGTAC	CLDN11__NM_001185056	591	480	661	660
CLDN11__NM_001185056_CCGCCTGTACTTAGCCACAC	CLDN11__NM_001185056	1023	1127	1161	1465
CLDN11__NM_001185056_CGGTGTGGCTAAGTACAGGC	CLDN11__NM_001185056	558	481	597	484
CLDN11__NM_001185056_GACCGAGGCAGCAATCATCA	CLDN11__NM_001185056	669	694	769	1026
CLDN11__NM_001185056_GAGACCACCATCGTGAGCTT	CLDN11__NM_001185056	553	537	559	395
CLDN11__NM_001185056_GCTACTCCCTGTATGCAGGC	CLDN11__NM_001185056	718	942	841	1165
CLDN11__NM_001185056_GGAGTAGCCAAAGCTCACGA	CLDN11__NM_001185056	592	850	742	654
CLDN11__NM_001185056_GGGCACTCTTCGCATGAGTC	CLDN11__NM_001185056	368	511	502	569
CLDN11__NM_001185056_GTTCTTCCCTGCATCCGGAT	CLDN11__NM_001185056	75	143	133	164
CLDN11__NM_001185056_TCACGATGGTGGTCTCACGG	CLDN11__NM_001185056	172	170	194	203
CLDN11__NM_001185056_TCCTGGCCCATCCGGATGCA	CLDN11__NM_001185056	525	721	512	589
CLDN11__NM_001185056_TTTGGCTACTCCCTGTATGC	CLDN11__NM_001185056	251	338	359	245
CLDN11__NM_005602_ACAAAAGGGAAAGTTTCCCA	CLDN11__NM_005602	371	627	366	356
CLDN11__NM_005602_ACACTGTGACGAGCAAACCT	CLDN11__NM_005602	625	806	949	668
CLDN11__NM_005602_ATAGTGGAACGAACTTTCCA	CLDN11__NM_005602	217	166	207	95
CLDN11__NM_005602_CCAGAAACTTCATCAAGTTA	CLDN11__NM_005602	489	573	504	528
CLDN11__NM_005602_CCCAAGAAAAGAGCTAATAG	CLDN11__NM_005602	382	320	439	365
CLDN11__NM_005602_CCTGGCCCTCAACTTACAAA	CLDN11__NM_005602	750	1190	885	943
CLDN11__NM_005602_CGGGAGGATACTTTGAGTCC	CLDN11__NM_005602	495	55	549	77
CLDN11__NM_005602_CTACTTCTAACACTTAGTAA	CLDN11__NM_005602	176	79	194	217
CLDN11__NM_005602_GAAAAGCATCTTGGCCGGTG	CLDN11__NM_005602	268	534	458	405
CLDN11__NM_005602_GACGAGCAAACCTAGGGCCC	CLDN11__NM_005602	631	593	588	484
CLDN11__NM_005602_GAGACAGAAAAGATAGCAT	CLDN11__NM_005602	183	135	168	102
CLDN11__NM_005602_GCCCAAGAAAAGAGCTAATA	CLDN11__NM_005602	213	342	335	307
CLDN11__NM_005602_GGCTTCGTCACGAGCTTCGT	CLDN11__NM_005602	888	1174	1069	995
CLDN11__NM_005602_GGGCTTCGTCACGAGCTTCG	CLDN11__NM_005602	382	513	447	668
CLDN11__NM_005602_GGTATTGCAGTGGTAATTTG	CLDN11__NM_005602	541	726	576	652
CLDN11__NM_005602_TCGTCACGAGCTTCGTGGGC	CLDN11__NM_005602	645	664	757	761
CLDN11__NM_005602_TGCCCAAGAAAAGAGCTAAT	CLDN11__NM_005602	326	326	344	347
CLDN11__NM_005602_TGCTCTCTAGGTTTCTTAAG	CLDN11__NM_005602	124	79	100	125
CLDN12__NM_001185072_AAGAACCTGACTGTTTACAC	CLDN12__NM_001185072	403	407	432	331
CLDN12__NM_001185072_ATGTCACTATTGCTAGTGCT	CLDN12__NM_001185072	333	512	354	424
CLDN12__NM_001185072_CACTTGGCCAGTTTGATGTT	CLDN12__NM_001185072	1035	1455	1274	1497
CLDN12__NM_001185072_CAGGGACTCTGCTTCCCAAC	CLDN12__NM_001185072	396	627	567	610
CLDN12__NM_001185072_CAGTGTTGCACATTCCAATC	CLDN12__NM_001185072	676	996	669	976
CLDN12__NM_001185072_CTCAGCATGCTGATCGCCAT	CLDN12__NM_001185072	253	345	265	286
CLDN12__NM_001185072_GCCTGATGTACGACACTACT	CLDN12__NM_001185072	982	1367	966	1546
CLDN12__NM_001185072_GTCACTGCTCCCGTCATACC	CLDN12__NM_001185072	849	1159	873	993
CLDN12__NM_001185072_GTGAAATGTGCCCGGTATGA	CLDN12__NM_001185072	587	658	608	663
CLDN12__NM_001185072_TATGTCACTATTGCTAGTGC	CLDN12__NM_001185072	355	683	494	580
CLDN12__NM_001185072_TGAAATGTGCCCGGTATGAC	CLDN12__NM_001185072	517	535	633	699
CLDN12__NM_001185072_TGCTGAGGGGTAGGGCAAAC	CLDN12__NM_001185072	136	309	121	243
CLDN14__NM_001146077_CCAGCAGGGATCGGTAGATC	CLDN14__NM_001146077	133	147	176	182
CLDN14__NM_001146077_CGATTGTCTTTGTAGGCAGC	CLDN14__NM_001146077	463	756	594	714
CLDN14__NM_001146077_GCCAGGACGAGGCACCCTAC	CLDN14__NM_001146077	1232	1093	1148	1447
CLDN15__NM_001185080_ACTTGGTTCCGGGGTACAAG	CLDN15__NM_001185080	187	121	179	163
CLDN15__NM_001185080_CCACCATCCCGCAGATACCT	CLDN15__NM_001185080	1463	1375	1449	1189
CLDN15__NM_001185080_CGGCAGGCCTGAATATACCC	CLDN15__NM_001185080	577	564	603	423
CLDN15__NM_001185080_CTGCGCTGCACCAACATTGG	CLDN15__NM_001185080	32	13	10	0
CLDN15__NM_001185080_GAGCTCCAGGCCCCCAATGT	CLDN15__NM_001185080	335	185	256	210
CLDN15__NM_001185080_GCAGGCCTGAATATACCCTG	CLDN15__NM_001185080	671	857	699	673
CLDN15__NM_001185080_GGCCCGCTATGCCTAGCAAG	CLDN15__NM_001185080	315	139	337	264
CLDN15__NM_001185080_GGCCTCTTGCTAGGCATAGC	CLDN15__NM_001185080	0	0	0	0
CLDN15__NM_001185080_GGCTTCTTCATGGCAACTGT	CLDN15__NM_001185080	417	670	525	633
CLDN15__NM_001185080_GGGGCCCTCCACATTCTGGC	CLDN15__NM_001185080	345	449	416	378
CLDN15__NM_001185080_TCACTTGGTTCCGGGGTACA	CLDN15__NM_001185080	484	583	590	571
CLDN15__NM_001185080_TGACTCTGCCAAACAGCTAC	CLDN15__NM_001185080	153	236	178	153
CLDN16__NM_006580_AACAGGCTGTAACCAACAGT	CLDN16__NM_006580	112	66	102	39
CLDN16__NM_006580_ACTGACACCCGCCACTTAAG	CLDN16__NM_006580	1098	908	1248	953
CLDN16__NM_006580_ACTTTAATGTACGGCTCATC	CLDN16__NM_006580	492	445	666	491
CLDN16__NM_006580_CACCATCCAACAGTCAGTCC	CLDN16__NM_006580	788	984	1009	1095
CLDN16__NM_006580_CAGATGCGGACTTTAATGTA	CLDN16__NM_006580	709	632	643	626
CLDN16__NM_006580_CCTGGCCCCACTTAAGTGGC	CLDN16__NM_006580	470	574	519	652
CLDN16__NM_006580_CTTTAATGTACGGCTCATCA	CLDN16__NM_006580	279	174	358	669
CLDN16__NM_006580_GCAGTAAGAATAATACAAAC	CLDN16__NM_006580	384	501	470	364
CLDN16__NM_006580_GTATGTGGAACGTTCTACTT	CLDN16__NM_006580	669	674	633	611
CLDN16__NM_006580_TAGGTGAGCACAAAATGCCG	CLDN16__NM_006580	331	429	331	259
CLDN16__NM_006580_TGAGTACGATTCCATACTTG	CLDN16__NM_006580	364	359	443	314
CLDN16__NM_006580_TTGAAGAAGATCCCTCATTG	CLDN16__NM_006580	541	484	624	442
CLDN17__NM_012131_AGTGCACAGGCTCTAACGAG	CLDN17__NM_012131	292	295	397	513
CLDN17__NM_012131_AGTTCCCAGAAGGTATGCTT	CLDN17__NM_012131	224	223	191	266
CLDN17__NM_012131_GAATTGCATCCGACAAGCCA	CLDN17__NM_012131	685	918	758	891
CLDN17__NM_012131_GGGCATCTTCGTTCTGATTC	CLDN17__NM_012131	348	330	483	913
CLDN17__NM_012131_GGGTACAGATATCCAGTGCC	CLDN17__NM_012131	800	1312	829	1074
CLDN17__NM_012131_TATAGAACTTGCATTGCAAC	CLDN17__NM_012131	53	66	49	122
CLDN17__NM_012131_TCCTTGATCGCCCTGCTTAT	CLDN17__NM_012131	767	1307	843	1345
CLDN17__NM_012131_TCGTTTCTGACCTATGTGGA	CLDN17__NM_012131	459	424	457	531
CLDN17__NM_012131_TTGGGTTCCTTGGCATGGTG	CLDN17__NM_012131	15	33	26	20
CLDN18__NM_001002026_ATTTGGTGCGGCTCTGTTCG	CLDN18__NM_001002026	517	595	545	851
CLDN18__NM_001002026_CACGAACCCCAAGCCCTGAC	CLDN18__NM_001002026	1543	1403	1749	1808
CLDN18__NM_001002026_CTGTCAGGGCTTGGGGTTCG	CLDN18__NM_001002026	1218	1462	1430	1704
CLDN18__NM_001002026_GACGATGCCTACGATCATCA	CLDN18__NM_001002026	697	584	743	925
CLDN18__NM_001002026_GCTAACATGTACACCGGCAT	CLDN18__NM_001002026	1038	1134	1039	1078
CLDN18__NM_001002026_GCTGGAGGCCTCACACTAAT	CLDN18__NM_001002026	672	775	799	863
CLDN18__NM_001002026_GGACGATGCCTACGATCATC	CLDN18__NM_001002026	327	296	315	264
CLDN18__NM_001002026_GGGTTCGTGGTTTCACTGAT	CLDN18__NM_001002026	226	276	224	355
CLDN18__NM_001002026_GTAGTTGAAAACAGCTGTTA	CLDN18__NM_001002026	188	260	106	132
CLDN18__NM_001002026_GTTTCACTGATTGGGATTGC	CLDN18__NM_001002026	147	278	204	162
CLDN18__NM_001002026_TCCACAGCTAACATGTACAC	CLDN18__NM_001002026	1303	1089	1401	1152
CLDN18__NM_001002026_TGGTGCAGACTGTTCAGACC	CLDN18__NM_001002026	81	131	110	75
CLDN18__NM_016369_ACCTCCGTGTTCCAGTACGA	CLDN18__NM_016369	603	527	775	553
CLDN18__NM_016369_CAGGCCCTATTTCACCATCC	CLDN18__NM_016369	1126	1184	1142	1460
CLDN18__NM_016369_GCTCCACATGTCCATCCCGG	CLDN18__NM_016369	1850	1895	1708	1350
CLDN18__NM_016369_GTTCAGGCTTCACCGAATGC	CLDN18__NM_016369	134	108	179	158
CLDN18__NM_016369_TGTTCCAGTACGAAGGGCTC	CLDN18__NM_016369	4	0	19	22
CLDN19__NM_001123395_AGCCCACAGCCCTATCGGCC	CLDN19__NM_001123395	1862	1640	1928	1964
CLDN19__NM_001123395_AGTTGGCATGAAGTGTACGC	CLDN19__NM_001123395	838	701	939	766
CLDN19__NM_001123395_ATTTGGCCCAGCCCTGTTCG	CLDN19__NM_001123395	743	1221	786	1045
CLDN19__NM_001123395_CGCTGATTGGATGTGACCTA	CLDN19__NM_001123395	777	508	803	568
CLDN19__NM_001123395_CTGCCGTGGGCCTCTATGAA	CLDN19__NM_001123395	320	305	347	314
CLDN19__NM_001123395_GACGCCATCATCACTGCCGT	CLDN19__NM_001123395	2770	2180	3097	2362
CLDN19__NM_001123395_GATGATGGCGTCGCCTGCGT	CLDN19__NM_001123395	792	474	758	342
CLDN19__NM_001123395_GCCCAGCCCACGAACAGGGC	CLDN19__NM_001123395	230	393	225	272
CLDN19__NM_001123395_GGCATGAAGTGTACGCGGGT	CLDN19__NM_001123395	1191	979	1440	1313
CLDN19__NM_001123395_TAGTTGGCATGAAGTGTACG	CLDN19__NM_001123395	262	329	199	205
CLDN19__NM_001123395_TGAGGCCCAGCCCACGAACA	CLDN19__NM_001123395	284	183	171	142
CLDN19__NM_001123395_TGGGCCTCTATGAAGGGCTC	CLDN19__NM_001123395	2	1	8	0
CLDN2__NM_001171092_AAAGACAGAGTGGCGGTAGC	CLDN2__NM_001171092	75	43	45	37
CLDN2__NM_001171092_ACGGGACTTCTACTCACCAC	CLDN2__NM_001171092	958	1056	871	964
CLDN2__NM_001171092_ATTGGAGAGGCTCTTTACTT	CLDN2__NM_001171092	144	79	94	106
CLDN2__NM_001171092_CCATGAAGATTCCAGGCAAC	CLDN2__NM_001171092	789	802	735	766
CLDN2__NM_001171092_CTAGGATGTAGCCCACAAGT	CLDN2__NM_001171092	8	13	14	25
CLDN2__NM_001171092_GACAGCATGAAATTTGAGAT	CLDN2__NM_001171092	390	436	365	424
CLDN2__NM_001171092_GAGGATGATTCCAGCTATCA	CLDN2__NM_001171092	260	420	350	319
CLDN2__NM_001171092_GATTGGAGAGGCTCTTTACT	CLDN2__NM_001171092	501	348	501	667
CLDN2__NM_001171092_GTGGTGAGTAGAAGTCCCGT	CLDN2__NM_001171092	181	127	162	156
CLDN2__NM_001171092_TCCCTGTTCTCCCTGATAGC	CLDN2__NM_001171092	170	165	223	182
CLDN2__NM_001171092_TTGGTAGGCATCGTAGTAGT	CLDN2__NM_001171092	535	658	527	619
CLDN2__NM_020384_ACGCCTGAAGAGTTTCTAGG	CLDN2__NM_020384	715	901	661	699
CLDN2__NM_020384_AGGTCAGGTGCCCTAGAGTG	CLDN2__NM_020384	602	607	580	571
CLDN2__NM_020384_AGTGAGGGGTCATACACCAA	CLDN2__NM_020384	576	557	657	687
CLDN2__NM_020384_ATTCTCTCATATTTGTGGGC	CLDN2__NM_020384	88	87	69	86
CLDN2__NM_020384_CACCTTCTGACACGATCCAG	CLDN2__NM_020384	1136	1375	1135	1153
CLDN2__NM_020384_CCTGGGATTGCAGCTGAGGT	CLDN2__NM_020384	381	712	564	478
CLDN2__NM_020384_GAAGCCAGTCATGCCTAGAC	CLDN2__NM_020384	480	475	481	493
CLDN2__NM_020384_GAAGCCCCTGCCTCACTCTA	CLDN2__NM_020384	1293	1586	1165	1273
CLDN2__NM_020384_GAGCATCTTCCGATGGACAG	CLDN2__NM_020384	296	405	320	340
CLDN2__NM_020384_GAGGCTCTGTAGTGCTCTGT	CLDN2__NM_020384	324	336	414	366
CLDN2__NM_020384_GAGTGGCTAGAGGGCATCTA	CLDN2__NM_020384	397	437	365	516
CLDN2__NM_020384_GGAATGGGGTTTCAAGTTGA	CLDN2__NM_020384	434	572	512	571
CLDN2__NM_020384_GGCACTGTTACAGATAGTCT	CLDN2__NM_020384	376	458	372	574
CLDN2__NM_020384_GGCCTCTTGCCTAAACCACA	CLDN2__NM_020384	341	454	376	547
CLDN2__NM_020384_GGGCTAGTGTAACAGCATGC	CLDN2__NM_020384	25	7	38	31
CLDN2__NM_020384_GTCAGTGGGATGTGATTAGT	CLDN2__NM_020384	661	846	656	864
CLDN2__NM_020384_GTTCCCTGGATACCGTAGGA	CLDN2__NM_020384	130	146	92	103
CLDN2__NM_020384_GTTGGCTCTTAGCTCATTGC	CLDN2__NM_020384	33	100	73	139
CLDN2__NM_020384_TACCACTGGATCGTGTCAGA	CLDN2__NM_020384	474	512	493	537
CLDN2__NM_020384_TAGATGCCCTCTAGCCACTC	CLDN2__NM_020384	448	507	523	542
CLDN2__NM_020384_TCTGCTGACAATGAGGTTCG	CLDN2__NM_020384	361	298	421	439
CLDN2__NM_020384_TGCTGAGGATAGACTGACTT	CLDN2__NM_020384	329	380	412	348
CLDN2__NM_020384_TGGCACTGTTACAGATAGTC	CLDN2__NM_020384	8	30	6	23
CLDN2__NM_020384_TGGGATGTGATTAGTGGGTT	CLDN2__NM_020384	155	384	171	189
CLDN20__NM_001001346_ATAATCCTTCAGATTGTGTG	CLDN20__NM_001001346	207	213	246	234
CLDN20__NM_001001346_ATGAAATGTACTCGCTTAGG	CLDN20__NM_001001346	348	166	421	291
CLDN20__NM_001001346_CACTCCAGAGACCCCAGATA	CLDN20__NM_001001346	2005	2677	2101	2809
CLDN20__NM_001001346_CCTGGAGGAGCTATCTATAT	CLDN20__NM_001001346	990	1181	1199	1464
CLDN20__NM_001001346_CTAGTTTAATCTCGACAGTG	CLDN20__NM_001001346	171	200	277	160
CLDN20__NM_001001346_CTTTCATCCTGGCCTTATCT	CLDN20__NM_001001346	752	841	910	1030
CLDN20__NM_001001346_GAAATGTACTCGCTTAGGAG	CLDN20__NM_001001346	190	147	291	170
CLDN20__NM_001001346_GAGCCCGTGCAGCTGTACAA	CLDN20__NM_001001346	1629	1072	1567	1248
CLDN20__NM_001001346_GGAATGAAATGTACTCGCTT	CLDN20__NM_001001346	430	409	559	708
CLDN20__NM_001001346_GGACAGAATGGAGTGTTTCA	CLDN20__NM_001001346	312	163	384	357
CLDN20__NM_001001346_TCGAGCTGTGTGTTAGAGAT	CLDN20__NM_001001346	1377	1749	1449	2099
CLDN22__NM_001111319_AGCTTCGGGCCACTACGCAG	CLDN22__NM_001111319	436	655	566	553
CLDN22__NM_001111319_GCGTAGTGGCCCGAAGCTAG	CLDN22__NM_001111319	135	163	175	133
CLDN22__NM_001111319_TGGCAGGTAGTTTGTAAGAC	CLDN22__NM_001111319	43	97	74	189
CLDN23__NM_194284_ACGCCGGTGGTGATGACGCT	CLDN23__NM_194284	325	489	386	547
CLDN23__NM_194284_AGTAGCCCCACTGGTCCGTC	CLDN23__NM_194284	846	964	801	1003
CLDN23__NM_194284_CAAGGCCTACACCAACTCGG	CLDN23__NM_194284	435	694	568	382
CLDN23__NM_194284_CACCGAGTTGGTGTAGGCCT	CLDN23__NM_194284	934	1195	923	966
CLDN23__NM_194284_CCCCAAGAAGTGGTTGTACC	CLDN23__NM_194284	260	498	328	441
CLDN23__NM_194284_CGACAACGCTCGTCGCACCA	CLDN23__NM_194284	854	929	926	998
CLDN23__NM_194284_GACGTCCACCGAGTTGGTGT	CLDN23__NM_194284	2161	2425	2307	2444
CLDN23__NM_194284_GCCCAAGGCCTACACCAACT	CLDN23__NM_194284	404	697	479	643
CLDN23__NM_194284_GCTGGTGGCGCGGGCACTCA	CLDN23__NM_194284	1761	2043	1970	2221
CLDN23__NM_194284_GTGCGGCCAGACGGACCAGT	CLDN23__NM_194284	643	765	765	763
CLDN23__NM_194284_GTGGACGTGGAGTTGTACCA	CLDN23__NM_194284	106	127	165	198
CLDN23__NM_194284_TGGGCTTGGGCTTGGGCTTG	CLDN23__NM_194284	231	301	401	455
CLDN25__NM_001101389_AGAAGTCTTGGATTGTGGCA	CLDN25__NM_001101389	472	550	440	668
CLDN25__NM_001101389_CCAGTGGAAGACTCTTAATC	CLDN25__NM_001101389	397	513	385	394
CLDN25__NM_001101389_CCCATGGGCTGGGCCTATTG	CLDN25__NM_001101389	27	56	20	37
CLDN25__NM_001101389_CTCCCATGGGCTGGGCCTAT	CLDN25__NM_001101389	291	248	354	490
CLDN25__NM_001101389_GAGATGGAGACCTGGATCAT	CLDN25__NM_001101389	622	419	711	651
CLDN25__NM_001101389_GCATCCCTGACATCATACCT	CLDN25__NM_001101389	811	704	924	802
CLDN25__NM_001101389_GCCCATGGGAGGCTACCATG	CLDN25__NM_001101389	537	595	711	547
CLDN25__NM_001101389_GGAGACTGGAAGGAGGGTAG	CLDN25__NM_001101389	225	73	184	95
CLDN25__NM_001101389_GGGCAGGATGGTGGTAACAC	CLDN25__NM_001101389	297	326	394	467
CLDN25__NM_001101389_TAATCTGGAACTGAACGAGA	CLDN25__NM_001101389	122	95	161	106
CLDN25__NM_001101389_TCAGATGGGTATTCAAGAGG	CLDN25__NM_001101389	323	334	324	236
CLDN25__NM_001101389_TCCCTGACATCATACCTCGG	CLDN25__NM_001101389	1141	1174	1091	1262
CLDN3__NM_001306_CAGCGCCACTAGCAGCCCGA	CLDN3__NM_001306	623	646	775	876
CLDN3__NM_001306_CCGACCAGGACACCGGCACG	CLDN3__NM_001306	630	668	676	730
CLDN3__NM_001306_CCGTGTACTTCTTCTCGCGT	CLDN3__NM_001306	4372	5509	4895	5967
CLDN3__NM_001306_CGCGGAGTAGACGACCTTGG	CLDN3__NM_001306	1757	2110	1868	2069
CLDN3__NM_001306_CGTGTACTTCTTCTCGCGTG	CLDN3__NM_001306	2467	3216	3014	3509
CLDN3__NM_001306_CTTCGGGCTGCTAGTGGCGC	CLDN3__NM_001306	527	642	503	759
CLDN3__NM_001306_GACCGCAAGGACTACGTCTA	CLDN3__NM_001306	544	640	615	579
CLDN3__NM_001306_GATAATGGTGTTGGCCGACC	CLDN3__NM_001306	424	409	493	356
CLDN3__NM_001306_GGTCGGCCAACACCATTATC	CLDN3__NM_001306	348	210	339	368
CLDN3__NM_001306_GTGTACTTCTTCTCGCGTGG	CLDN3__NM_001306	201	284	297	339
CLDN3__NM_001306_GTTGCCCATGTGGCGCGTGT	CLDN3__NM_001306	1153	1444	1151	1278
CLDN4__NM_001305_CCTGGAGGATGAAAGCGCCA	CLDN4__NM_001305	303	238	316	293
CLDN4__NM_001305_GAGTAAGGCTTGTCTGTGCG	CLDN4__NM_001305	1359	1909	1620	1942
CLDN4__NM_001305_GCTGCCAGCAACTACGTGTA	CLDN4__NM_001305	674	859	708	844
CLDN4__NM_001305_TCCATGGGGCTACAGGTAAT	CLDN4__NM_001305	416	411	447	541
CLDN4__NM_001305_TCCGGGCAGAAGCGGGAGAT	CLDN4__NM_001305	1015	1460	1249	1533
CLDN4__NM_001305_TGCCTCGCTCTACGTCGGCT	CLDN4__NM_001305	484	584	599	680
CLDN4__NM_001305_TTGTCACCTCGCAGACCATC	CLDN4__NM_001305	383	453	342	214
CLDN5__NM_001130861_AAACTCGCGGACGACAATGT	CLDN5__NM_001130861	507	430	621	358
CLDN5__NM_001130861_ACGATGACCCGCGCACGGAT	CLDN5__NM_001130861	3022	3228	3289	2659
CLDN5__NM_001130861_AGCCATGGGGTCCGCAGCGT	CLDN5__NM_001130861	539	421	566	410
CLDN5__NM_001130861_CGCGTCTCGCCTCTAGCCAT	CLDN5__NM_001130861	1479	1721	1712	1469
CLDN5__NM_001130861_GACCCGCGGGGCAAACGGAC	CLDN5__NM_001130861	239	245	330	282
CLDN5__NM_001130861_GAGGCGAGACGCGCACCCGA	CLDN5__NM_001130861	434	369	445	460
CLDN5__NM_001130861_GCAGTGCAAAGTGTACGACT	CLDN5__NM_001130861	911	973	776	671
CLDN5__NM_001130861_GCGTCTCGCCTCTAGCCATG	CLDN5__NM_001130861	345	500	415	631
CLDN5__NM_001130861_GGGGCTGTGGATGTCGTGCG	CLDN5__NM_001130861	698	784	672	899
CLDN5__NM_001130861_GGTGCTGGCTCTGAGCACCG	CLDN5__NM_001130861	227	248	194	232
CLDN5__NM_001130861_TACCCTCTTTGAAGGTTCGG	CLDN5__NM_001130861	204	133	233	107
CLDN5__NM_001130861_TCTGCGACACGGGCACAGAC	CLDN5__NM_001130861	314	381	327	450
CLDN6__NM_021195_AAGCTATGGAACTAATGCGG	CLDN6__NM_021195	363	381	448	286
CLDN6__NM_021195_AGCACACGGGGATTAGCGTC	CLDN6__NM_021195	520	643	554	876
CLDN6__NM_021195_CATTAGTTCCATAGCTTGAC	CLDN6__NM_021195	125	227	159	179
CLDN6__NM_021195_CCAGGGCCCCAGCCATTACA	CLDN6__NM_021195	364	310	366	313
CLDN6__NM_021195_CGCCCAGCCCAAGTAGAGGG	CLDN6__NM_021195	463	358	534	532
CLDN6__NM_021195_CTCCACACAGGTGGTACACT	CLDN6__NM_021195	40	17	17	3
CLDN6__NM_021195_CTTGCTGGTCTACCTTGCTG	CLDN6__NM_021195	1070	450	935	463
CLDN6__NM_021195_GATGCTGTTGCCGATGAAAG	CLDN6__NM_021195	571	385	576	596
CLDN6__NM_021195_GCTTGCTGGTCTACCTTGCT	CLDN6__NM_021195	2423	1958	2362	2008
CLDN6__NM_021195_GGAGCTCCAGAGACCTGAGT	CLDN6__NM_021195	1327	1294	1567	1032
CLDN6__NM_021195_GGCTTGCTGGTCTACCTTGC	CLDN6__NM_021195	939	880	1074	1039
CLDN6__NM_021195_GGGCGGGGGTCTACATAGCT	CLDN6__NM_021195	2	0	28	31
CLDN6__NM_021195_GTAGACCAGCAAGCCGAACA	CLDN6__NM_021195	104	91	206	123
CLDN6__NM_021195_GTCAAGCTATGGAACTAATG	CLDN6__NM_021195	295	251	335	274
CLDN6__NM_021195_TCCAGAGACCTGAGTAGGAT	CLDN6__NM_021195	296	444	419	475
CLDN6__NM_021195_TGACGCTAATCCCCGTGTGC	CLDN6__NM_021195	762	632	724	921
CLDN6__NM_021195_TGGAAGGTGACCGCTTTCAT	CLDN6__NM_021195	337	300	503	229
CLDN6__NM_021195_TGGACGTCTTATCAGGACGG	CLDN6__NM_021195	74	38	102	78
CLDN6__NM_021195_TGGGGGGTGGACGTCTTATC	CLDN6__NM_021195	165	111	136	67
CLDN6__NM_021195_TGTGGCCCTGTTCGGCTTGC	CLDN6__NM_021195	590	746	795	972
CLDN7__NM_001185022_AAGAAGGCCCGTATAGCCAT	CLDN7__NM_001185022	587	589	764	593
CLDN7__NM_001185022_ACTGCGTCACGCAGAGCACG	CLDN7__NM_001185022	130	156	114	159
CLDN7__NM_001185022_ATGAAGTGCACGCGCTGTGG	CLDN7__NM_001185022	838	641	869	816
CLDN7__NM_001185022_GAAGAAGGCCCGTATAGCCA	CLDN7__NM_001185022	331	255	463	401
CLDN7__NM_001185022_GACCACCATTAGGGCTCGAG	CLDN7__NM_001185022	486	639	618	666
CLDN7__NM_001185022_GAGCTGCAAAATGTACGACT	CLDN7__NM_001185022	536	592	600	584
CLDN7__NM_001185022_GCAGGCCACTCGAGCCCTAA	CLDN7__NM_001185022	276	323	440	545
CLDN7__NM_001185022_GCCTCCACCCATGGCTATAC	CLDN7__NM_001185022	606	664	776	587
CLDN7__NM_001185022_GGCCACTCGAGCCCTAATGG	CLDN7__NM_001185022	486	506	484	453
CLDN7__NM_001185022_GTGGCAGATGAGCTCCTATG	CLDN7__NM_001185022	139	121	211	253
CLDN7__NM_001185022_TGCCTCCACCCATGGCTATA	CLDN7__NM_001185022	316	480	253	240
CLDN7__NM_001185022_TGGCAGATGAGCTCCTATGC	CLDN7__NM_001185022	14	5	10	43
CLDN8__NM_199328_AAACAGCCCAGCGATTTCTA	CLDN8__NM_199328	304	370	347	337
CLDN8__NM_199328_CCCATGCCTTAGAAATCGCT	CLDN8__NM_199328	1399	1819	1498	1846
CLDN8__NM_199328_CCTCTGGCTGCCTGTAGGTC	CLDN8__NM_199328	708	1045	805	1178
CLDN8__NM_199328_CTTAGGATGGACCACGGCAC	CLDN8__NM_199328	261	422	312	481
CLDN8__NM_199328_CTTCATTGAAAACAACATCG	CLDN8__NM_199328	148	227	202	266
CLDN8__NM_199328_CTTGGAGAAGCTCTCTACTT	CLDN8__NM_199328	1303	1765	1528	1478
CLDN8__NM_199328_GACTGTGGATGAATTGCGTG	CLDN8__NM_199328	939	813	981	1207
CLDN8__NM_199328_GCGTGAGGCAGGCTAACATC	CLDN8__NM_199328	648	924	898	976
CLDN8__NM_199328_GGCTCTTTCTCCGGACCTAC	CLDN8__NM_199328	165	272	199	289
CLDN8__NM_199328_TCTCTACTTAGGATGGACCA	CLDN8__NM_199328	141	146	157	232
CLDN8__NM_199328_TCTTGGTGGTGTTGGAATGG	CLDN8__NM_199328	146	187	194	271
CLDN8__NM_199328_TGACTTCTGGAGTAGACGCT	CLDN8__NM_199328	58	86	137	237
CLDN9__NM_020982_CCCCCGCGGTGAGCACGATA	CLDN9__NM_020982	705	670	825	701
CLDN9__NM_020982_CCCCGCGGTGAGCACGATAC	CLDN9__NM_020982	837	593	929	858
CLDN9__NM_020982_CTGCACTGCTTATGCTGGGC	CLDN9__NM_020982	1273	1291	1317	1729
CLDN9__NM_020982_GACAAGAGGGACTACGTGTG	CLDN9__NM_020982	308	300	361	328
CLDN9__NM_020982_GCCCGTATCGTGCTCACCGC	CLDN9__NM_020982	1014	959	1192	1009
CLDN9__NM_020982_GGCGGCTGCACTGCTTATGC	CLDN9__NM_020982	938	913	1099	1432
CLDN9__NM_020982_GTGCATCTGGACTGGACAAG	CLDN9__NM_020982	170	141	167	188
CLDN9__NM_020982_TGCACTGCTTATGCTGGGCG	CLDN9__NM_020982	454	417	480	838
CLDN9__NM_020982_TGCCCAGTGTACCACGTGTG	CLDN9__NM_020982	238	208	304	321
CLDND1__NM_001040181_AATGGAGGCTGCCATGTAGA	CLDND1__NM_001040181	438	559	361	339
CLDND1__NM_001040181_CAATGGTGGGATATAAGCTT	CLDND1__NM_001040181	298	287	315	313
CLDND1__NM_001040181_CAGGGAGCTCTAGTTTCTGG	CLDND1__NM_001040181	808	751	972	893
CLDND1__NM_001040181_GACAATGTATCCGGTGAATT	CLDND1__NM_001040181	504	591	499	516
CLDND1__NM_001040181_GAGCTCCCTGACAATGTATC	CLDND1__NM_001040181	486	453	317	331
CLDND1__NM_001040181_GATCCCGGAAACCACAATAG	CLDND1__NM_001040181	216	286	286	467
CLDND1__NM_001040181_GCAGAAGCCATGAACTGTAA	CLDND1__NM_001040181	251	300	198	353
CLDND1__NM_001040181_GGAGATCAATCCCGCTATTG	CLDND1__NM_001040181	776	845	765	782
CLDND1__NM_001040181_GGATGAATTCATTAGTGATG	CLDND1__NM_001040181	300	399	374	316
CLDND1__NM_001040181_TTACAAATGCTGTAGCAAAA	CLDND1__NM_001040181	26	13	26	33
CLDND1__NM_001040181_TTATATCCCACCATTGCCAC	CLDND1__NM_001040181	283	231	258	298
CLDND1__NM_001040181_TTTAGGTTTGATGTGCTTTG	CLDND1__NM_001040181	171	184	144	198
CLDND1__NM_001040182_CCAGGATGCTACGGAGACAG	CLDND1__NM_001040182	507	731	568	617
CLDND1__NM_001040182_CCTCTGTCTCCGTAGCATCC	CLDND1__NM_001040182	454	878	497	569
CLDND1__NM_001040200_CTTAATGAAGGCATATCGTG	CLDND1__NM_001040200	345	351	333	383
CLDND1__NM_001040200_GAAAGAGTACACCTTAATGA	CLDND1__NM_001040200	802	821	931	999
CLDND1__NM_001040200_TAAGGTGTACTCTTTCCGGT	CLDND1__NM_001040200	175	198	235	117
CLDND2__NM_152353_ACACACGGGGAATCCACTGA	CLDND2__NM_152353	719	533	807	782
CLDND2__NM_152353_AGAGGAAGGCGCTCGTGGTC	CLDND2__NM_152353	1186	1088	1377	1266
CLDND2__NM_152353_CTGCTGCTGACCGCCTTGAT	CLDND2__NM_152353	1150	1048	1224	1559
CLDND2__NM_152353_CTTACCCTTCTCAATTCTCG	CLDND2__NM_152353	890	1066	1110	1437
CLDND2__NM_152353_CTTCACGGTGTAGCCTATCA	CLDND2__NM_152353	332	211	360	501
CLDND2__NM_152353_GGAGAGCACCATGAGGACGT	CLDND2__NM_152353	1182	1097	1307	1288
CLDND2__NM_152353_GGGGAATCCACTGATGGCGT	CLDND2__NM_152353	564	667	654	618
CLDND2__NM_152353_TACTGGACCCGCCAACAAGA	CLDND2__NM_152353	136	98	110	80
CLDND2__NM_152353_TTGTTGGCGGGTCCAGTAGT	CLDND2__NM_152353	515	487	574	487
CLEC10A__NM_006344_AGGCTCCGCATACACCTGGA	CLEC10A__NM_006344	476	731	553	667
CLEC10A__NM_006344_CCAGGTGGGCGTTCTTCAGC	CLEC10A__NM_006344	334	589	414	525
CLEC10A__NM_006344_CCGTGCCCCTGCCAGTCGTC	CLEC10A__NM_006344	3420	4125	3663	4404
CLEC10A__NM_006344_CTTCACCTCAAACACTGTGG	CLEC10A__NM_006344	254	327	425	323
CLEC10A__NM_006344_CTTCCAGTACTTGGAGAATA	CLEC10A__NM_006344	317	320	416	556
CLEC10A__NM_006344_GATGGAACAGACTATGCGAC	CLEC10A__NM_006344	698	845	809	992
CLEC10A__NM_006344_GCCCCTGCCAGTCGTCTGGC	CLEC10A__NM_006344	1054	1540	1229	1609
CLEC10A__NM_006344_GGCTCCGCATACACCTGGAT	CLEC10A__NM_006344	1257	1866	1459	1559
CLEC10A__NM_006344_GGTGCTCCACCCAGTTGACA	CLEC10A__NM_006344	214	213	241	237
CLEC10A__NM_006344_TCTGCCAGAGGCCCTACCAC	CLEC10A__NM_006344	810	835	928	978
CLEC10A__NM_006344_TGAGGTGGAGGGTTTCAAGC	CLEC10A__NM_006344	179	117	175	225
CLEC10A__NM_182906_CCAGGAACACACTACGCAGA	CLEC10A__NM_182906	1224	1365	1475	1419
CLEC10A__NM_182906_GAACTGGGACACACACAGAT	CLEC10A__NM_182906	793	908	1234	1397
CLEC11A__NM_002975_CAGGGCTTGCACGGCATCGC	CLEC11A__NM_002975	554	693	490	572
CLEC11A__NM_002975_CCTCCCAGCAGGCAGTCCTA	CLEC11A__NM_002975	506	296	481	574
CLEC11A__NM_002975_CGAGCGTGCCACCGTTGGGC	CLEC11A__NM_002975	1239	1313	1532	1515
CLEC11A__NM_002975_CTCCCTACAACTGGCCCGTG	CLEC11A__NM_002975	724	978	901	1158
CLEC11A__NM_002975_CTGCGAGTTCCCCTTCTAGC	CLEC11A__NM_002975	1127	1624	1592	1623
CLEC11A__NM_002975_GAATCCTGCGGAACTGTTG	CLEC11A__NM_002975	640	729	625	647
CLEC11A__NM_002975_GGCCTCTACCTCTTCGAAAA	CLEC11A__NM_002975	398	534	439	706
CLEC11A__NM_002975_GGGTGTCCAACGCGTGCAGA	CLEC11A__NM_002975	271	482	261	322
CLEC11A__NM_002975_GGTGGGACCACGACTGCCAG	CLEC11A__NM_002975	178	187	208	255
CLEC11A__NM_002975_TGGTCCGGGCTGAGCGGATG	CLEC11A__NM_002975	0	0	2	4
CLEC11A__NM_002975_TGTGGCTGGGCGTGCACGAT	CLEC11A__NM_002975	1284	1383	1436	1360
CLEC11A__NM_002975_TTTCCCTCAACAGTTCCGGC	CLEC11A__NM_002975	171	205	235	242
CLEC12A__NM_001207010_AGAGTAGATCATATGACTAT	CLEC12A__NM_001207010	297	222	423	494
CLEC12A__NM_001207010_ATATAGCTCACGACATAATT	CLEC12A__NM_001207010	31	7	34	27
CLEC12A__NM_001207010_ATGGAAAAAATCCCAGAAAT	CLEC12A__NM_001207010	134	169	170	187
CLEC12A__NM_001207010_CCTGAAGAAGATTCCACTCG	CLEC12A__NM_001207010	1302	1321	1589	1468
CLEC12A__NM_001207010_CCTTCTGTTGCTCATTGGAT	CLEC12A__NM_001207010	1026	1429	1264	1653
CLEC12A__NM_001207010_GGAGAGGTTCCTGATCTTGT	CLEC12A__NM_001207010	2380	2314	2757	2947
CLEC12A__NM_001207010_TAGCTCACGACATAATTTGG	CLEC12A__NM_001207010	305	472	380	399
CLEC12A__NM_001207010_TGATGATGTCCAAACATGGC	CLEC12A__NM_001207010	405	520	370	419
CLEC12A__NM_001207010_TGCTGGACGCCATACATGAG	CLEC12A__NM_001207010	576	525	541	665
CLEC12A__NM_001207010_TTCCACTCGTGGTATGAGAG	CLEC12A__NM_001207010	10	12	23	9
CLEC12B__NM_001129998_AACCTGGGCTAACAGTAGAA	CLEC12B__NM_001129998	244	236	268	378
CLEC12B__NM_001129998_ACTGTCTATCTTCACTAGGG	CLEC12B__NM_001129998	951	639	1022	737
CLEC12B__NM_001129998_AGAGCAGCATGACGCCAAAT	CLEC12B__NM_001129998	280	234	374	272
CLEC12B__NM_001129998_CAAACTGTCTATCTTCACTA	CLEC12B__NM_001129998	1452	1499	1594	1956
CLEC12B__NM_001129998_CATTGCCACATCTTAGGACA	CLEC12B__NM_001129998	2302	2592	2597	3526
CLEC12B__NM_001129998_CCTAGTGAAGATAGACAGTT	CLEC12B__NM_001129998	735	920	791	1300
CLEC12B__NM_001129998_CTGAAATGTGAGTGTCGCGT	CLEC12B__NM_001129998	1382	1852	1490	1821
CLEC12B__NM_001129998_GGAGCAAGGAATAACCGAGA	CLEC12B__NM_001129998	335	565	515	484
CLEC12B__NM_001129998_GTAATCCATGTCCTAAGATG	CLEC12B__NM_001129998	157	249	191	144
CLEC12B__NM_001129998_GTCCTTTCTACTGTTAGCCC	CLEC12B__NM_001129998	152	173	144	193
CLEC12B__NM_001129998_TAGCTCTTGGCACAGTTTGA	CLEC12B__NM_001129998	31	20	37	47
CLEC12B__NM_205852_TTGTACGTCTCTAACTATTG	CLEC12B__NM_205852	608	639	551	489
CLEC14A__NM_175060_AGCCAGGAGAAACCCCGCAA	CLEC14A__NM_175060	705	677	872	657
CLEC14A__NM_175060_AGGCCTGCATCCTGCGAGGT	CLEC14A__NM_175060	469	559	492	703
CLEC14A__NM_175060_CACCCCACTGCCGACCGTGC	CLEC14A__NM_175060	1932	1870	2223	2507
CLEC14A__NM_175060_CAGCCAGCACGGTCGGCAGT	CLEC14A__NM_175060	2010	2056	2241	1849
CLEC14A__NM_175060_CCAAGTCGTCTAGGCAGTTA	CLEC14A__NM_175060	344	441	352	418
CLEC14A__NM_175060_CCCTAACTGCCTAGACGACT	CLEC14A__NM_175060	2180	2376	2499	3061
CLEC14A__NM_175060_CCCTGGAGAACGAGCCTTTG	CLEC14A__NM_175060	748	1210	881	1362
CLEC14A__NM_175060_CCTGGAGAACGAGCCTTTGC	CLEC14A__NM_175060	1081	1815	1278	1245
CLEC14A__NM_175060_CGATGCAAGTAACTGAGATC	CLEC14A__NM_175060	683	668	735	693
CLEC14A__NM_175060_CTGGAGAACGAGCCTTTGCG	CLEC14A__NM_175060	1436	1543	1658	2064
CLEC14A__NM_175060_GAGGCCTGCATCCTGCGAGG	CLEC14A__NM_175060	827	1020	858	777
CLEC14A__NM_175060_GCAGCCAGCACGGTCGGCAG	CLEC14A__NM_175060	789	912	1088	1177
CLEC16A__NM_001243403_AACATCTTGCGGCAAAAGTC	CLEC16A__NM_001243403	142	119	132	122
CLEC16A__NM_001243403_ACGGCCGAAAATTAGCCTGC	CLEC16A__NM_001243403	467	376	557	435
CLEC16A__NM_001243403_AGTCATCGAGTTCGATCACA	CLEC16A__NM_001243403	478	465	494	647
CLEC16A__NM_001243403_ATCACTGAGATCCTGATCTG	CLEC16A__NM_001243403	998	901	1035	983
CLEC16A__NM_001243403_CCGACATGTTTGGCCGCTCG	CLEC16A__NM_001243403	2905	3179	3258	4466
CLEC16A__NM_001243403_CTTACTTCTCCAATTTGGTC	CLEC16A__NM_001243403	274	507	349	496
CLEC16A__NM_001243403_GAACATCTTGCGGCAAAAGT	CLEC16A__NM_001243403	419	312	458	451
CLEC16A__NM_001243403_GACAGCTCTGTATTTGAGTA	CLEC16A__NM_001243403	166	135	214	203
CLEC16A__NM_001243403_GATCTCAGTGATGGAACGGA	CLEC16A__NM_001243403	245	139	227	284
CLEC16A__NM_001243403_GATGTATAATTAAGAAGACC	CLEC16A__NM_001243403	172	73	157	118
CLEC16A__NM_001243403_GCAGCTGAACGCACACGTAA	CLEC16A__NM_001243403	276	242	277	285
CLEC16A__NM_001243403_TCTCCAGTGAGTACACGTAG	CLEC16A__NM_001243403	879	659	974	890
CLEC17A__NM_001204118_AGCCCCAGCCAGTACACCCG	CLEC17A__NM_001204118	2046	1790	2305	2173
CLEC17A__NM_001204118_AGCCTTCAGGACAGGTAATT	CLEC17A__NM_001204118	816	852	976	1142
CLEC17A__NM_001204118_ATGGCTCTCCACGGGTGTAC	CLEC17A__NM_001204118	10	4	16	23
CLEC17A__NM_001204118_CAGACCTCTGATTGAGCCAG	CLEC17A__NM_001204118	876	916	1072	697
CLEC17A__NM_001204118_CCCTGGCTCAATCAGAGGTC	CLEC17A__NM_001204118	247	167	244	300
CLEC17A__NM_001204118_CTTCAGGACAGGTAATTCGG	CLEC17A__NM_001204118	621	552	712	632
CLEC17A__NM_001204118_CTTGGTGGAGCACTTGAACC	CLEC17A__NM_001204118	272	255	286	463
CLEC17A__NM_001204118_GACTGTGCTACCATGAACAA	CLEC17A__NM_001204118	333	344	395	300
CLEC17A__NM_001204118_GTTGGTGTCAGCTCTTACAC	CLEC17A__NM_001204118	8	4	37	70
CLEC17A__NM_001204118_TCCGGGGCTTGCAAGGAAGT	CLEC17A__NM_001204118	350	441	317	282
CLEC17A__NM_001204118_TCCTCATCGTGGATGTTATT	CLEC17A__NM_001204118	314	380	357	417
CLEC17A__NM_001204118_TGTGCTACCATGAACAAAGG	CLEC17A__NM_001204118	65	44	79	41
CLEC18A__NM_001136214_ACAAAGGACACCAAGCCCGC	CLEC18A__NM_001136214	918	1227	844	1228
CLEC18A__NM_001136214_TAGGCTGACCACTTCGACAA	CLEC18A__NM_001136214	301	393	256	218
CLEC18B__NM_001011880_CAAAGGCTTCTATCGCTGTC	CLEC18B__NM_001011880	374	519	437	536
CLEC18B__NM_001011880_GGCCCTCCTTGGCACCACCT	CLEC18B__NM_001011880	379	554	456	492
CLEC18B__NM_001011880_GTGTGGCTGAGTGCTGCCAT	CLEC18B__NM_001011880	1022	577	1239	754
CLEC19A__NM_001256720_CTTCCCTCTCAATAAGACCT	CLEC19A__NM_001256720	265	256	271	257
CLEC19A__NM_001256720_TCAGCCCAGGTCTTATTGAG	CLEC19A__NM_001256720	555	292	583	541
CLEC19A__NM_001256720_TCTTCCCTCTCAATAAGACC	CLEC19A__NM_001256720	106	115	135	123
CLEC19A__NM_001256720_TGGTACAGGCCTACCAGTGG	CLEC19A__NM_001256720	26	33	38	17
CLEC1A__NM_016511_AATGGTGTCTTGACCAGTAT	CLEC1A__NM_016511	536	708	631	530
CLEC1A__NM_016511_ACTGTGTGGCCATCCTTAAT	CLEC1A__NM_016511	1147	1153	1350	926
CLEC1A__NM_016511_ATTTCCATTGTTCTGTACAA	CLEC1A__NM_016511	288	246	229	190
CLEC1A__NM_016511_CATGTCCCCCCTGAAACATT	CLEC1A__NM_016511	1131	759	1233	956
CLEC1A__NM_016511_CCTTCGCCTAATGTTTCAGG	CLEC1A__NM_016511	808	949	929	1120
CLEC1A__NM_016511_CGTGAGCTGTATAACAAAGC	CLEC1A__NM_016511	403	435	629	618
CLEC1A__NM_016511_CTTGACCAGTATTGGAGAGC	CLEC1A__NM_016511	385	430	408	396
CLEC1A__NM_016511_CTTGGTGCTGCTGATAGGGC	CLEC1A__NM_016511	585	751	537	564
CLEC1A__NM_016511_GCAGCCCTTGTACAGAACAA	CLEC1A__NM_016511	324	348	296	224
CLEC1A__NM_016511_GGGCTCTGGATGCCGAGTTG	CLEC1A__NM_016511	184	230	279	308
CLEC1A__NM_016511_TACTACCAGCTCTCCAATAC	CLEC1A__NM_016511	333	503	350	252
CLEC1A__NM_016511_TTGAGAAGATCATCCCATTA	CLEC1A__NM_016511	810	931	833	780
CLEC1B__NM_001099431_AAAGCGCTTCTGTCAATATG	CLEC1B__NM_001099431	618	597	520	537
CLEC1B__NM_001099431_ACTCATTTAATTCGTTGGGT	CLEC1B__NM_001099431	1213	1544	1330	1672
CLEC1B__NM_001099431_ATCTCCAGTTTGTGTCACAG	CLEC1B__NM_001099431	550	450	712	523
CLEC1B__NM_001099431_ATCTCGCCAGAAGTCGAATG	CLEC1B__NM_001099431	462	653	516	622
CLEC1B__NM_001099431_CAGGACTCATTTAATTCGTT	CLEC1B__NM_001099431	1316	1121	1350	1233
CLEC1B__NM_001099431_CCAGGACTCATTTAATTCGT	CLEC1B__NM_001099431	1114	1583	1464	1594
CLEC1B__NM_001099431_CTTCAGGCACAACTTAACAT	CLEC1B__NM_001099431	400	453	473	449
CLEC1B__NM_001099431_GACACAAACTGGAGATATTA	CLEC1B__NM_001099431	304	370	301	378
CLEC1B__NM_001099431_GCAGCCCCTGTGACACAAAC	CLEC1B__NM_001099431	621	665	809	1085
CLEC1B__NM_001099431_GGTGAGAATGAAAATCGCAC	CLEC1B__NM_001099431	275	477	296	488
CLEC1B__NM_001099431_TATCTCCAGTTTGTGTCACA	CLEC1B__NM_001099431	683	719	828	844
CLEC1B__NM_001099431_TCTTCAGGCACAACTTAACA	CLEC1B__NM_001099431	355	458	444	561
CLEC1B__NM_016509_CGACAACCATCCCCACGCAC	CLEC1B__NM_016509	2598	2712	3039	2593
CLEC1B__NM_016509_CTCCTCCTGGTGGCGTGTGA	CLEC1B__NM_016509	421	520	451	522
CLEC2A__NM_001130711_AGGTTTGAAATTATAGGAA	CLEC2A__NM_001130711	29	30	59	65
CLEC2A__NM_001130711_ATGGCACCACATTCAATGGT	CLEC2A__NM_001130711	544	792	632	751
CLEC2A__NM_001130711_CCGCAGCTCTGGATTAATCA	CLEC2A__NM_001130711	370	284	450	531
CLEC2A__NM_001130711_GAGCTGATGGCTTCATACAT	CLEC2A__NM_001130711	1008	853	1135	991
CLEC2A__NM_001130711_GAGGATTTATTGATATCAAG	CLEC2A__NM_001130711	99	138	98	148
CLEC2A__NM_001130711_GATGGCTTCATACATCGGAT	CLEC2A__NM_001130711	372	422	388	294
CLEC2A__NM_001130711_TTGATACAAAACTGGAAGAT	CLEC2A__NM_001130711	197	277	276	344
CLEC2A__NM_001130711_TTTAGCATGCTTGGACCATG	CLEC2A__NM_001130711	207	139	247	250
CLEC2B__NM_005127_AAAAAATCGAACAGGACAAT	CLEC2B__NM_005127	142	174	125	118
CLEC2B__NM_005127_AAGATGGCAAAAAATCGAAC	CLEC2B__NM_005127	170	139	173	192
[illegible]	[illegible]	[illegible]	[illegible]	[illegible]	[illegible]
CLEC4F__NM_001258027_GAACGAGCCTCGGTATCTTG	CLEC4F__NM_001258027	762	443	909	617
CLEC4F__NM_001258027_GAGCTCCACGTGCTAAGCAG	CLEC4F__NM_001258027	310	434	448	402
CLEC4F__NM_001258027_GATGGTAAAAGGAGTTCTAA	CLEC4F__NM_001258027	136	161	163	197
CLEC4F__NM_001258027_GCCTGTGCAAGCCGTAATTC	CLEC4F__NM_001258027	1189	1093	1263	1506
CLEC4F__NM_001258027_GGTCACAGCCATAAATGCCG	CLEC4F__NM_001258027	398	503	430	370
[illegible]	[illegible]	[illegible]	[illegible]	[illegible]	[illegible]
CLECL1__NM_001253750_AGTGGAAACGCGAGTTCTAA	CLECL1__NM_001253750	364	308	444	345
CLECL1__NM_001253750_CAAAGACTGGAAGGTGCATA	CLECL1__NM_001253750	146	81	184	158
CLECL1__NM_001253750_CTTTGGCAGGACATGATTTA	CLECL1__NM_001253750	317	354	413	419
CLECL1__NM_001253750_GTGGAAACGCGAGTTCTAAC	CLECL1__NM_001253750	109	21	112	142
CLECL1__NM_001253750_GTGTTCAGTCTTACTAATCA	CLECL1__NM_001253750	153	110	178	157
[illegible]	[illegible]	[illegible]	[illegible]	[illegible]	[illegible]
CLIC6__NM_053277_GGCGGAGGTCCCGGGGGTAA	CLIC6__NM_053277	1647	2317	1644	2179
CLIC6__NM_053277_GGGGGACAACATACAAGCCG	CLIC6__NM_053277	398	516	441	585
CLIC6__NM_053277_GGTAAAGGGGTCCGAAGAAG	CLIC6__NM_053277	360	542	347	385
CLIC6__NM_053277_GGTTATGATGGTGAGAGTAT	CLIC6__NM_053277	360	439	475	420
[illegible]	[illegible]	[illegible]	[illegible]	[illegible]	[illegible]

CLPSL2__NM_001286550_GCATCTTCCTCTAAATCATA	CLPSL2__NM_001286550	216	489	210	281
CLPSL2__NM_001286550_GCTCGTGGCGGGGGTCCTGT	CLPSL2__NM_001286550	1828	1834	2268	1987
CLPTM1__NM_001282175_AAAGGCTCATATAACCGAAA	CLPTM1__NM_001282175	84	144	88	44
CLPTM1__NM_001282175_CACTCTTGGTGAAGTAAACG	CLPTM1__NM_001282175	323	333	293	397
CLPTM1__NM_001282175_CCAGCAATGGCAGCATCGGG	CLPTM1__NM_001282175	159	127	188	164
CLPTM1__NM_001282175_CTTGTATTTGTTGATCACTG	CLPTM1__NM_001282175	562	606	568	590
CLPTM1__NM_001282175_GGCAGAAGGCCCTGTACCGC	CLPTM1__NM_001282175	336	248	253	236
CLPTM1__NM_001282175_GGCTCATATAACCGAAAAGG	CLPTM1__NM_001282175	73	17	45	85
CLPTM1__NM_001282175_TCAGCAGTTGGTTCCGCCGA	CLPTM1__NM_001282175	424	289	543	446
CLPTM1__NM_001282175_TCATCAAAGGTGTGCTGTTT	CLPTM1__NM_001282175	700	698	818	921
CLPTM1__NM_001282175_TGCTGAGCTCGATATCCCAC	CLPTM1__NM_001282175	311	166	240	152
CLPTM1__NM_001282175_TGTGGACTGTGGCAAGCCGG	CLPTM1__NM_001282175	313	344	357	250
CLPTM1__NM_001282175_TTACCATTAAAGTGTCTTTG	CLPTM1__NM_001282175	93	109	103	131
CLPTM1__NM_001282175_TTCCCAGAAGAGTGCCGACG	CLPTM1__NM_001282175	164	100	149	109
CLPTM1__NM_001282176_CTGGCCAGGCTCTCGTTGAT	CLPTM1__NM_001282176	1819	1816	2108	2127
CLPTM1__NM_001282176_CTGTGGAGGTGATCTCCCAT	CLPTM1__NM_001282176	604	320	760	498
CLPTM1__NM_001282176_GCCAGCGCCAAAGCGAGAGT	CLPTM1__NM_001282176	247	198	294	260
CLPTM1__NM_001282176_TCATCTACTTCAATGACTAC	CLPTM1__NM_001282176	295	386	330	474
CLPTM1__NM_001282176_TCGTGGACGACCACACGCCG	CLPTM1__NM_001282176	2	8	3	0
CLPTM1__NM_001282176_TGGTGATGTTGGGGTGCCAA	CLPTM1__NM_001282176	10	14	5	12
CLPTM1L__NM_030782_ACCACGACGAGGTCCCACCT	CLPTM1L__NM_030782	10	55	22	0
CLPTM1L__NM_030782_AGGTGGTCAGAGGACTGACC	CLPTM1L__NM_030782	3	1	2	9
CLPTM1L__NM_030782_CAAAGAAAACGAGAAACAAT	CLPTM1L__NM_030782	396	505	481	597
CLPTM1L__NM_030782_CAGTTGGCGTCGCCGGAGCA	CLPTM1L__NM_030782	301	312	323	436
CLPTM1L__NM_030782_CCAAAGAAAACGAGAAACAA	CLPTM1L__NM_030782	80	105	79	196
CLPTM1L__NM_030782_CTGGATGCAGTTGGCGTCGC	CLPTM1L__NM_030782	895	1103	937	1180
CLPTM1L__NM_030782_CTTGGTGGTGGGCGTGTTCG	CLPTM1L__NM_030782	679	764	641	834
CLPTM1L__NM_030782_GCAGGACCCCAGCGTGATGG	CLPTM1L__NM_030782	632	630	853	667
CLPTM1L__NM_030782_GGGACCATGTAGGTGGTCAG	CLPTM1L__NM_030782	72	163	79	144
CLPTM1L__NM_030782_TCCAGCCCTACCTGGCGCGG	CLPTM1L__NM_030782	934	816	963	1273
CLPTM1L__NM_030782_TGAGCGTGTACACCACGACG	CLPTM1L__NM_030782	312	260	280	396
CLPTM1L__NM_030782_TGGCTTGGGGACCATGTAGG	CLPTM1L__NM_030782	61	51	37	38
CLPX__NM_006660_ACCACCACCTCCCCCTAAGA	CLPX__NM_006660	1105	744	1209	807
CLPX__NM_006660_AGCAGCAGAAGCTGTAAAAT	CLPX__NM_006660	359	392	382	338
CLPX__NM_006660_CAAGTACAAGCACCGCAGCT	CLPX__NM_006660	599	652	644	705
CLPX__NM_006660_CGGTGCTTGTACTTGCGGCG	CLPX__NM_006660	471	653	482	482
CLPX__NM_006660_GAGGGAAGTAGTAAGAAATC	CLPX__NM_006660	104	129	109	60
CLPX__NM_006660_GGCCAACAACATACTTGTCG	CLPX__NM_006660	1040	862	1158	1106
CLPX__NM_006660_GTTTCTGTAAAGGATCTAAG	CLPX__NM_006660	100	151	105	89
CLPX__NM_006660_TACCTCGACAAGTATGTTGT	CLPX__NM_006660	806	836	996	896
CLPX__NM_006660_TCAGAATTGCTTCAGATTGC	CLPX__NM_006660	519	977	529	606
CLPX__NM_006660_TGGCCAGTCATTTGCTAAGA	CLPX__NM_006660	51	44	58	61
CLPX__NM_006660_TGTACAACTTTGACTCAGGC	CLPX__NM_006660	313	370	317	451
CLPX__NM_006660_TTCACACTTGACAAAACGGG	CLPX__NM_006660	28	18	8	12
CLRN1__NM_001195794_AAACTCTGCATGGTCCCCTA	CLRN1__NM_001195794	742	1029	879	791
CLRN1__NM_001195794_ACAGTGGCTTTGATCCACAA	CLRN1__NM_001195794	525	833	617	604
CLRN1__NM_001195794_CAAGGCTGTCACAACTCCGA	CLRN1__NM_001195794	544	675	533	357
CLRN1__NM_001195794_CAGCCTTGGGGACACCGTTG	CLRN1__NM_001195794	671	524	780	638
CLRN1__NM_001195794_CAGGAGCTGGACAAGTTTAT	CLRN1__NM_001195794	446	462	408	480
CLRN1__NM_001195794_CATGGTTAACACAATAAGGA	CLRN1__NM_001195794	124	92	116	93
CLRN1__NM_001195794_CCACCATGGTTAACACAATA	CLRN1__NM_001195794	484	441	473	778
CLRN1__NM_001195794_CCTTATTGTGTTAACCATGG	CLRN1__NM_001195794	469	501	484	514
CLRN1__NM_001195794_CTCAAAAGGTACAGCCCTAG	CLRN1__NM_001195794	291	298	348	423
CLRN1__NM_001195794_GCTGCCAGCTACCAGGCACC	CLRN1__NM_001195794	1046	1763	1068	1367
CLRN1__NM_001195794_TTATGGGTGAAATGCAGTAC	CLRN1__NM_001195794	251	260	306	245
CLRN1__NM_001195794_TTTATGGGTGAAATGCAGTA	CLRN1__NM_001195794	101	54	93	90
CLRN1__NM_174878_GGGCTCCTAATACGACTTGC	CLRN1__NM_174878	402	516	398	381
CLRN2__NM_001079827_ACATGATCCAGGTCCCGTAC	CLRN2__NM_001079827	731	927	769	1094
CLRN2__NM_001079827_AGTTGGCGATTCGTTCCGTC	CLRN2__NM_001079827	467	539	606	724
CLRN2__NM_001079827_CACTCGTGAATTGGGATTGG	CLRN2__NM_001079827	1659	1766	1839	2189
CLRN2__NM_001079827_GACCTGGATCATGTTAAGAA	CLRN2__NM_001079827	229	312	271	301
CLRN2__NM_001079827_GAGAGCTGGTCAAGTTCATT	CLRN2__NM_001079827	1121	1255	1328	1239
CLRN2__NM_001079827_GGTGAAATTTCACGACCTGA	CLRN2__NM_001079827	380	371	365	526
CLRN2__NM_001079827_GGTTCAAAAAGGCGTGGTAT	CLRN2__NM_001079827	62	39	41	29
CLRN2__NM_001079827_GTCCTGGGGGCATCTGCCTA	CLRN2__NM_001079827	731	975	817	702
CLRN2__NM_001079827_TCATTGGGGACATTTACTAC	CLRN2__NM_001079827	1425	1435	1494	1760
CLRN2__NM_001079827_TGCCATTCTTAACATGATCC	CLRN2__NM_001079827	357	424	355	413
CLRN2__NM_001079827_TGGTTCAAAAAGGCGTGGTA	CLRN2__NM_001079827	0	0	0	0
CLRN2__NM_001079827_TTTACTACGGGCTCTTCCGA	CLRN2__NM_001079827	89	115	116	108
CLRN3__NM_152311_AAAGGAACGACCCACAGTTA	CLRN3__NM_152311	1176	1308	1157	1043
CLRN3__NM_152311_ACAGTTACGGATACTCGTTC	CLRN3__NM_152311	660	581	751	620
CLRN3__NM_152311_ACGTGATCAAACTCAGGACC	CLRN3__NM_152311	549	775	604	601
CLRN3__NM_152311_ACTGTGGGTCGTTCCTTTAC	CLRN3__NM_152311	854	909	950	962
CLRN3__NM_152311_AGAACGAGTATCCGTAACTG	CLRN3__NM_152311	774	586	969	1043
CLRN3__NM_152311_GAACGAGTATCCGTAACTGT	CLRN3__NM_152311	1036	936	1211	1002
CLRN3__NM_152311_GTTCGCCACAAACAGTATCA	CLRN3__NM_152311	351	320	432	326
CLRN3__NM_152311_TACACCTGGAACGGGCTCGG	CLRN3__NM_152311	721	922	699	724
CLRN3__NM_152311_TACCCGGCAACCACCAGTAA	CLRN3__NM_152311	648	827	725	594
CLRN3__NM_152311_TATCATGGTCACAAAAACGA	CLRN3__NM_152311	333	664	415	539
CLSPN__NM_001190481_ACAATTGGACCCTTGAGTGA	CLSPN__NM_001190481	864	1089	1005	930
CLSPN__NM_001190481_AGACAGCCACTGTCATTAAA	CLSPN__NM_001190481	295	215	236	160
CLSPN__NM_001190481_AGACAGTGATTCCGAAACAG	CLSPN__NM_001190481	161	225	214	209
CLSPN__NM_001190481_AGTTATCAAAAGGAACCACG	CLSPN__NM_001190481	144	144	195	172
CLSPN__NM_001190481_CACTTCCTTCCTCAAATGAA	CLSPN__NM_001190481	272	306	336	211
CLSPN__NM_001190481_GAACAGCCATTTAATGACAG	CLSPN__NM_001190481	554	502	507	313
CLSPN__NM_001190481_GAGACTCAGCGCCTTATTCG	CLSPN__NM_001190481	1337	1383	1664	1996
CLSPN__NM_001190481_GCAGATAGTCCTTCAGATAG	CLSPN__NM_001190481	385	261	520	354
CLSPN__NM_001190481_GGATGAAAATAACTCTCCAT	CLSPN__NM_001190481	616	445	783	672
CLSPN__NM_001190481_TCTCAGGCATATGATATGGA	CLSPN__NM_001190481	253	122	211	114
CLSPN__NM_001190481_TGAAGACTCAGCTCTAAGCA	CLSPN__NM_001190481	99	44	98	71
CLSPN__NM_001190481_TTTGGAACTCTTTCTGTCAG	CLSPN__NM_001190481	154	228	185	192
CLSTN1__NM_001009566_ACCATCCAGGCCTATGATTG	CLSTN1__NM_001009566	1491	1468	1692	1608
CLSTN1__NM_001009566_AGGTCCCTTCCCACAATCAT	CLSTN1__NM_001009566	507	417	618	551
CLSTN1__NM_001009566_CAGAGCAACTGTTCATATTC	CLSTN1__NM_001009566	547	777	688	765
CLSTN1__NM_001009566_CCATCCAGGCCTATGATTGT	CLSTN1__NM_001009566	758	813	778	1015
CLSTN1__NM_001009566_CCTTCCCACAATCATAGGCC	CLSTN1__NM_001009566	440	679	355	453
CLSTN1__NM_001009566_CGACCCCCCACTGATCGCGC	CLSTN1__NM_001009566	2346	3400	2779	3446
CLSTN1__NM_001009566_CGTTCTCTGTGACTATGCCG	CLSTN1__NM_001009566	452	781	534	459
CLSTN1__NM_001009566_CTTTATCCAGCGCGATCAGT	CLSTN1__NM_001009566	160	211	190	269
CLSTN1__NM_001009566_GTGACTATGCCGTGGTAGGT	CLSTN1__NM_001009566	666	558	743	792
CLSTN1__NM_001009566_TACAAAGCCACGGTCATCGA	CLSTN1__NM_001009566	1532	1155	1750	1647
CLSTN1__NM_001009566_TCTTTATCCAGCGCGATCAG	CLSTN1__NM_001009566	317	219	302	288
CLSTN1__NM_001009566_TTTATCCAGCGCGATCAGTG	CLSTN1__NM_001009566	172	148	241	195
CLSTN2__NM_022131_AACCGGTGCATCTTTATCCA	CLSTN2__NM_022131	414	605	426	421
CLSTN2__NM_022131_CAGGGCCGTGGTCCATATAC	CLSTN2__NM_022131	847	912	839	790
CLSTN2__NM_022131_CGCCGCCTCCTCGCGGCTAA	CLSTN2__NM_022131	8	5	34	11
CLSTN2__NM_022131_CTGGTGACCGCCTACGACTG	CLSTN2__NM_022131	699	755	781	896
CLSTN2__NM_022131_CTTCACCTGTATATGGACCA	CLSTN2__NM_022131	944	1206	1140	1383
CLSTN2__NM_022131_CTTCTGTCCACAGTCGTAGG	CLSTN2__NM_022131	300	381	303	342
CLSTN2__NM_022131_CTTGTAGGCTGGCTCTTTGA	CLSTN2__NM_022131	281	365	317	240
CLSTN2__NM_022131_GTCACAACAGCCTTGTAGGC	CLSTN2__NM_022131	124	116	152	262
CLSTN2__NM_022131_TACAAGGCTGTTGTGACGGA	CLSTN2__NM_022131	40	77	66	55
CLSTN2__NM_022131_TGATATGAAGTCTCGATCCA	CLSTN2__NM_022131	149	162	215	253
CLSTN2__NM_022131_TGGATCGAGACTTCATATCA	CLSTN2__NM_022131	13	87	24	20
CLSTN2__NM_022131_TGGGGAGCAGTCCTCGTCAA	CLSTN2__NM_022131	183	398	145	163
CLSTN3__NM_014718_ACCACTCTTTGCCTTGGACA	CLSTN3__NM_014718	1164	945	1343	1184
CLSTN3__NM_014718_AGACCTGTGATGAACCACTC	CLSTN3__NM_014718	555	513	689	601
CLSTN3__NM_014718_AGGTCTCCAGGCGGATACCA	CLSTN3__NM_014718	104	119	148	129
CLSTN3__NM_014718_CAGTGACAGCTTATGACTGT	CLSTN3__NM_014718	931	1020	966	945
CLSTN3__NM_014718_CCTCTCCTGTCGCCTTGTCA	CLSTN3__NM_014718	1473	1216	1826	1693
CLSTN3__NM_014718_GCGTCCTGCTCCTGTAACAA	CLSTN3__NM_014718	728	629	871	903
CLSTN3__NM_014718_TGGATTGAGGCAGAGTACCA	CLSTN3__NM_014718	39	24	13	21
CLSTN3__NM_014718_TGTGGAACGGCTGTATCGTG	CLSTN3__NM_014718	760	649	763	736
CLSTN3__NM_014718_TTCTGGGCCTCGCAGTCCAC	CLSTN3__NM_014718	27	35	32	39
CLSTN3__NM_014718_TTCTTGGTGTTGGCCCCGTC	CLSTN3__NM_014718	43	30	11	13
CLSTN3__NM_014718_TTGCCAGCTGGGTTTACAGG	CLSTN3__NM_014718	276	316	279	276
CLSTN3__NM_014718_TTTGTTCCCTGGTATCCGCC	CLSTN3__NM_014718	95	160	113	130
CLTA__NM_001076677_AGAAGAATGGTATGCAAGAC	CLTA__NM_001076677	366	335	419	418
CLTA__NM_001076677_AGCCTGAAAGTATCCGTAAA	CLTA__NM_001076677	195	239	301	307
CLTA__NM_001076677_ATAGCTGCATAACTGTCTGT	CLTA__NM_001076677	477	207	542	433
CLTA__NM_001076677_CAACCCTTCGCTGACGTGAT	CLTA__NM_001076677	1610	1699	1768	2182
CLTA__NM_001076677_CTCGTCAATGTCATTTACAA	CLTA__NM_001076677	580	636	613	673
CLTA__NM_001076677_TTGGCCTGCTTGCTAGACTT	CLTA__NM_001076677	426	301	378	310
CLTA__NM_001076677_TTTGGCCTGCTTGCTAGACT	CLTA__NM_001076677	741	889	907	1013
CLTB__NM_001834_GCAGACGCCACTGTCCCGCT	CLTB__NM_001834	1766	1500	1908	2041
CLTB__NM_001834_GTAGCCATCAGCAGGACCGT	CLTB__NM_001834	645	857	875	590
CLTB__NM_001834_TCTGCTCCTCTCGCCACTTG	CLTB__NM_001834	1089	759	1243	1145
CLTB__NM_007097_CAGCCAGATGCTGATATCAT	CLTB__NM_007097	832	703	884	966
CLTB__NM_007097_TAGCCGATGATATCAGCATC	CLTB__NM_007097	61	125	94	87
CLTC__NM_001288653_AGATGCTCCTGAAAACGAAT	CLTC__NM_001288653	229	13	254	58
CLTC__NM_001288653_AGCAAAGTAATTGCACTGAA	CLTC__NM_001288653	378	11	345	18
CLTC__NM_001288653_ATCGCCATTCTAGCCTTGCA	CLTC__NM_001288653	931	133	1046	179
CLTC__NM_001288653_CATATCAATGATTACCACCT	CLTC__NM_001288653	578	126	653	276
CLTC__NM_001288653_GAATACGGTTGCTCTTGTTA	CLTC__NM_001288653	261	6	330	34
CLTC__NM_001288653_GCTGAAATTGGTCTTCGAAT	CLTC__NM_001288653	848	1017	854	1178
CLTC__NM_001288653_GGTCTTCGAATTGGATTACT	CLTC__NM_001288653	271	209	294	267
CLTC__NM_001288653_GTTTATCACTGGAGTATGGA	CLTC__NM_001288653	81	13	83	43
CLTC__NM_001288653_TAGCTTTGCACAGTTTAAGA	CLTC__NM_001288653	170	53	119	38
CLTC__NM_001288653_TCTGACTGGTATATCTGCAC	CLTC__NM_001288653	440	23	492	55
CLTC__NM_001288653_TGCAGCTATATTCTGTAGAT	CLTC__NM_001288653	296	51	365	144
CLTC__NM_001288653_TGCAGTTTATCACTGGAGTA	CLTC__NM_001288653	179	45	187	277
CLTC__NM_004859_AAAGCAGTGCAAGACTAACT	CLTC__NM_004859	479	326	561	466
CLTC__NM_004859_AAAGGCCCTCTAACCTATGC	CLTC__NM_004859	298	245	406	348
CLTC__NM_004859_AAAGTGTTTAATGTGAGTGC	CLTC__NM_004859	321	279	387	318
CLTC__NM_004859_AATGACCTTAGACCTTGAGA	CLTC__NM_004859	459	368	560	570
CLTC__NM_004859_ACCTTTGGCCAGTATGGGTC	CLTC__NM_004859	211	192	238	227
CLTC__NM_004859_AGGTCATTGGTGGAGTATTG	CLTC__NM_004859	120	140	166	206
CLTC__NM_004859_AGTAGCAGCTAGGATCTTAT	CLTC__NM_004859	64	64	19	35
CLTC__NM_004859_ATCTCAAGGTCTAAGGTCAT	CLTC__NM_004859	326	326	394	429
CLTC__NM_004859_CACACACAAGATTGGAATCA	CLTC__NM_004859	384	317	463	320
CLTC__NM_004859_CTAATCAGCACATCGTACAC	CLTC__NM_004859	559	255	690	421
CLTC__NM_004859_CTGAAACTTGAGTAACCTTA	CLTC__NM_004859	374	407	424	511
CLTC__NM_004859_GAGGTGGAGCCACTTAAAAT	CLTC__NM_004859	92	73	85	49
CLTC__NM_004859_GATTAACCTTTGGCCAGTAT	CLTC__NM_004859	344	372	400	290
CLTC__NM_004859_GCAGTTCTTTAAAGAAAGAG	CLTC__NM_004859	0	0	15	48
CLTC__NM_004859_GGCCAAAGGTTAATCAGCAG	CLTC__NM_004859	375	405	432	649
CLTC__NM_004859_GGCTTCATTGTTACAAGAAA	CLTC__NM_004859	88	79	85	162
CLTC__NM_004859_GTGTAGCTATTGAGGATTAA	CLTC__NM_004859	116	141	147	86
CLTC__NM_004859_GTGTCACTTGGTTAAAATAT	CLTC__NM_004859	168	84	279	200
CLTC__NM_004859_GTTTGCCTCAGATAGACTGA	CLTC__NM_004859	48	39	52	48
CLTC__NM_004859_TAGCACCATCTCAAGGTCTA	CLTC__NM_004859	374	306	387	366
CLTC__NM_004859_TATTGCAGTTAACCATAGAG	CLTC__NM_004859	149	139	226	254
CLTC__NM_004859_TCCATCTGTTAAGGTCCATA	CLTC__NM_004859	217	280	171	146
CLTC__NM_004859_TCTGGCTCAGGTAATTTCTG	CLTC__NM_004859	693	527	786	768
CLTC__NM_004859_TCTGTGGGACCATTCCATTC	CLTC__NM_004859	555	422	593	513
CLTC__NM_004859_TGGAGCCATCAGTCTATCTG	CLTC__NM_004859	310	356	325	310
CLTC__NM_004859_TGTGTTGATATTTGCACTGA	CLTC__NM_004859	462	400	458	406
CLTC__NM_004859_TTAGTAAACACACACAAGAT	CLTC__NM_004859	69	97	108	87
CLTC__NM_004859_TTATAAAGGTGCCCAAATTA	CLTC__NM_004859	141	110	169	159
CLTC__NM_004859_TTGCTCACTTCATATGTAAC	CLTC__NM_004859	104	99	145	193
CLTC__NM_004859_TTGGCACTGACAGTCTTGCA	CLTC__NM_004859	253	351	321	335
CLTCL1__NM_001835_ACCCAATGGCTCCGATCCGA	CLTCL1__NM_001835	822	580	878	649
CLTCL1__NM_001835_AGAGCTATCACCTTAGAGGC	CLTCL1__NM_001835	406	352	357	239
CLTCL1__NM_001835_ATATGTATCCGAGAGAAAGT	CLTCL1__NM_001835	51	43	74	30
CLTCL1__NM_001835_CATGCTCCAGTGGTAGACCG	CLTCL1__NM_001835	619	658	620	429
CLTCL1__NM_001835_CCGAGACCGCGGTCTACCAC	CLTCL1__NM_001835	1000	755	977	483
CLTCL1__NM_001835_CCGAGAGAAAGTTGGTGAGC	CLTCL1__NM_001835	574	424	597	453
CLTCL1__NM_001835_GCAGAGATAGGCCGTCGGAT	CLTCL1__NM_001835	1522	1239	1768	1356
CLTCL1__NM_001835_GCCGTCGGATCGGAGCCATT	CLTCL1__NM_001835	1435	1065	1341	989
CLTCL1__NM_001835_GGAATTAATCCAGCTAACAT	CLTCL1__NM_001835	207	324	303	290
CLTCL1__NM_001835_GTGCTGAATCCAATGTTAGC	CLTCL1__NM_001835	179	289	197	302
CLTCL1__NM_001835_GTTTGATAGACATACCAGTC	CLTCL1__NM_001835	161	169	288	268
CLTCL1__NM_001835_TAAAATGAAGGCTCATACTA	CLTCL1__NM_001835	96	196	133	69
CLU__NM_001831_AAATTCAAAATGCTGTCAAC	CLU__NM_001831	166	124	202	107
CLU__NM_001831_AGGATGCCCTAAATGAGACC	CLU__NM_001831	217	239	209	155
CLU__NM_001831_CCAAGAAGAAGAAAGAGGTC	CLU__NM_001831	135	290	171	137
CLU__NM_001831_CCGGCAGCAGACGCACATGC	CLU__NM_001831	262	295	312	304
CLU__NM_001831_CCTTCAGCCTGCCCCACCGG	CLU__NM_001831	604	510	627	471
CLU__NM_001831_CTCAGGCCTGGTTGGCCGCC	CLU__NM_001831	106	79	151	249
CLU__NM_001831_CTGCGGACGATGCGGGACTT	CLU__NM_001831	928	1196	1085	1535
CLU__NM_001831_GAAGAAGTGAGGCCTCCGGT	CLU__NM_001831	2728	3114	3094	3188
CLU__NM_001831_GACGTACTTACTTCCCTGAT	CLU__NM_001831	1061	1265	1416	1581
CLU__NM_001831_GGAATCAGAGACAAAGCTGA	CLU__NM_001831	483	439	543	633
CLU__NM_001831_GGGAAGTAAGTACGTCAATA	CLU__NM_001831	412	323	533	642
CLU__NM_001831_TGATTCCCTGGTCTCATTTA	CLU__NM_001831	101	74	87	98
CLUAP1__NM_015041_AAAGCAGTATGACACTTATC	CLUAP1__NM_015041	285	215	427	253
CLUAP1__NM_015041_ATACCCTCGACATATTTCTA	CLUAP1__NM_015041	333	194	336	291
CLUAP1__NM_015041_CAAGTCCAAAATTGGGTGTA	CLUAP1__NM_015041	286	231	272	303
CLUAP1__NM_015041_CGTCAGGCGGGATGTCAGTC	CLUAP1__NM_015041	724	820	775	881
CLUAP1__NM_015041_GCCTGACGTGGATACTGAAC	CLUAP1__NM_015041	642	560	805	554
CLUAP1__NM_015041_TATTTCCAGAGGTCTGGCAA	CLUAP1__NM_015041	106	57	137	110
CLUAP1__NM_015041_TCAGATACAAGTCCAAAATT	CLUAP1__NM_015041	140	112	193	97
CLUAP1__NM_015041_TCCTGTTCAGTATCCACGTC	CLUAP1__NM_015041	536	478	598	708
CLUAP1__NM_015041_TCTGTATGACTTGCTCGGCA	CLUAP1__NM_015041	579	446	600	627
CLUAP1__NM_015041_TGGTCTTCATAGCATTATAA	CLUAP1__NM_015041	0	0	0	0
CLUAP1__NM_015041_TTATCAAGCAGATGGGTATG	CLUAP1__NM_015041	110	104	153	229
CLUAP1__NM_015041_TTCAGATACAAGTCCAAAAT	CLUAP1__NM_015041	63	265	71	84
CLUAP1__NM_024793_AAGACCTGGCAAACGCATTG	CLUAP1__NM_024793	488	365	499	623
CLUAP1__NM_024793_AGACCTGGCAAACGCATTGT	CLUAP1__NM_024793	922	851	995	847
CLUAP1__NM_024793_ATTGTGGGCACGATGCAAGG	CLUAP1__NM_024793	431	359	478	365
CLUAP1__NM_024793_CCGGACCCTTCGATTGGGCT	CLUAP1__NM_024793	1739	2216	1834	2468
CLUAP1__NM_024793_CCTTGAGCTTGTTCTGTATC	CLUAP1__NM_024793	966	1353	1130	1155
CLUAP1__NM_024793_GATTTCCGGACCCTTCGATT	CLUAP1__NM_024793	649	627	802	556
CLUAP1__NM_024793_GTGCCCACAATGCGTTTGCC	CLUAP1__NM_024793	335	316	418	504
CLUH__NM_015229_CCACCCGAGTACATCCTGCC	CLUH__NM_015229	1506	1557	1728	1986
CLUH__NM_015229_CCCCGTTGAAGGCATCGGAT	CLUH__NM_015229	1078	688	1220	1258
CLUH__NM_015229_CGCACCTCCCAGGTCGCCGT	CLUH__NM_015229	228	365	211	290
CLUH__NM_015229_CGGGTGGTGTGCAGTCGATG	CLUH__NM_015229	479	338	436	476
CLUH__NM_015229_CTCGGGTGGTGTGCAGTCGA	CLUH__NM_015229	359	386	440	459
CLUH__NM_015229_GAAAATGGGCTTGACGAGGC	CLUH__NM_015229	303	274	357	399
CLUH__NM_015229_GGCCCGGGAGATGAGACCAC	CLUH__NM_015229	399	366	425	390
CLUH__NM_015229_GGGAGAAGGGCTCGATCCCA	CLUH__NM_015229	313	373	329	236
CLUH__NM_015229_GGTCCAGCACGTTGCCATCC	CLUH__NM_015229	564	576	705	898
CLUH__NM_015229_GTTGCAGTCAACCCCGTTGA	CLUH__NM_015229	680	618	798	632
CLUH__NM_015229_TCGGAGCTGCGCAGCGTCGA	CLUH__NM_015229	100	161	175	86
CLUH__NM_015229_TTCCTGAGTGTCTTCACCGA	CLUH__NM_015229	94	103	154	101
CLUL1__NM_001289036_AAGCAGATCTTGAGCAATGT	CLUL1__NM_001289036	231	222	290	336
CLUL1__NM_001289036_AATGTATCCAATCAGCAGTA	CLUL1__NM_001289036	44	62	30	34
CLUL1__NM_001289036_AGAGGACCAGCTAGGTTGGC	CLUL1__NM_001289036	895	778	966	876
CLUL1__NM_001289036_AGCAGATCTTGAGCAATGTT	CLUL1__NM_001289036	359	352	493	345
CLUL1__NM_001289036_AGGAAGAAGAAAGGCTATGC	CLUL1__NM_001289036	130	70	200	108
CLUL1__NM_001289036_CACACAATAAACACCAAGAG	CLUL1__NM_001289036	815	930	900	935
CLUL1__NM_001289036_CAGAATTAGACGAGGCGATC	CLUL1__NM_001289036	161	286	193	239
CLUL1__NM_001289036_CTGAAAAACCGTTCAATCTG	CLUL1__NM_001289036	465	526	416	586
CLUL1__NM_001289036_CTTCAGGGTGCTCATTAGAT	CLUL1__NM_001289036	390	260	436	386
CLUL1__NM_001289036_TCTGCACACAGAATTAGACG	CLUL1__NM_001289036	357	380	407	274
CLUL1__NM_001289036_TGCACAATTGACCCAAATGG	CLUL1__NM_001289036	275	260	292	206
CLUL1__NM_001289036_TGTCAGGCTCACCTATCTGA	CLUL1__NM_001289036	661	475	701	623
CLUL1__NM_199167_AATGCAATAAACACAGTTGC	CLUL1__NM_199167	616	719	652	947
CLUL1__NM_199167_GTGATAGAACATAGACACCT	CLUL1__NM_199167	279	162	289	285
CLVS1__NM_173519_AAAACCGAGACCATTACGGC	CLVS1__NM_173519	505	568	543	667
CLVS1__NM_173519_AAACACTTGGAACGGAGATT	CLVS1__NM_173519	458	343	436	318
CLVS1__NM_173519_AGATGATCCCGGCATTAAGA	CLVS1__NM_173519	232	162	214	149
CLVS1__NM_173519_AGCCTGTAAATGGGTCATCT	CLVS1__NM_173519	436	541	508	524
CLVS1__NM_173519_ATCATCACCAGGCCTGACAT	CLVS1__NM_173519	670	506	721	775
CLVS1__NM_173519_ATTAAGAGGGCTCTGATCGA	CLVS1__NM_173519	249	369	318	325
CLVS1__NM_173519_GAAGCTCCGGATCTTCGATT	CLVS1__NM_173519	825	933	728	697
CLVS1__NM_173519_GGAAGTCCTAATCGAAGATC	CLVS1__NM_173519	53	54	71	121
CLVS1__NM_173519_TATCCTGATGTAAAACATCG	CLVS1__NM_173519	214	180	231	180
CLVS1__NM_173519_TCAGGATATTCAGCAAGTCA	CLVS1__NM_173519	165	130	164	195
CLVS1__NM_173519_TTAAGAGGGCTCTGATCGAT	CLVS1__NM_173519	226	244	228	174
CLVS1__NM_173519_TTAGGACTTCCAATGACAGC	CLVS1__NM_173519	46	20	79	92
CLVS2__NM_001010852_AAGCTCAGGATCTTCAATCA	CLVS2__NM_001010852	287	320	295	225
CLVS2__NM_001010852_ATAGCTAATCGCAGCATACT	CLVS2__NM_001010852	15	0	7	11
CLVS2__NM_001010852_ATGCTGCGATTAGCTATTGA	CLVS2__NM_001010852	827	731	854	1022
CLVS2__NM_001010852_CAGAAAGCCAATGTCCGGCC	CLVS2__NM_001010852	766	971	915	1317
CLVS2__NM_001010852_CCAATCTGGACCACTATGGC	CLVS2__NM_001010852	946	973	1127	1365
CLVS2__NM_001010852_CCGCAAGAAGCGTAAGATGA	CLVS2__NM_001010852	364	285	463	564
CLVS2__NM_001010852_CCTTCATCTTACGCTTCTTG	CLVS2__NM_001010852	1094	1462	1229	1802
CLVS2__NM_001010852_GATAGTTTCCCAGCGCGATT	CLVS2__NM_001010852	176	130	195	246
CLVS2__NM_001010852_GGTACTCAAAGTACTGCGCC	CLVS2__NM_001010852	103	155	169	216
CLVS2__NM_001010852_TCTTACGCTTCTTGCGGGCT	CLVS2__NM_001010852	834	1379	1015	1269
CLVS2__NM_001010852_TGGCGCAGTACTTTGAGTAC	CLVS2__NM_001010852	83	69	145	84
CLVS2__NM_001010852_TGTCCTGGTGCAGCGTGTCT	CLVS2__NM_001010852	360	313	387	329
CLYBL__NM_206808_AGATGGCGCTACGTCTGCTG	CLYBL__NM_206808	1331	1951	1360	1462
CLYBL__NM_206808_AGGGCAGTGCTTTATGTACC	CLYBL__NM_206808	518	616	675	632
CLYBL__NM_206808_CCCTGGATATTCTCTACGCC	CLYBL__NM_206808	1032	919	1306	1287
CLYBL__NM_206808_CCGGGCGTAGAGAATATCCA	CLYBL__NM_206808	428	407	518	533
CLYBL__NM_206808_CTGAAGGTCGGGCCTCAAGT	CLYBL__NM_206808	864	388	1061	1013
CLYBL__NM_206808_CTGCTTAGACAGTCACGAGA	CLYBL__NM_206808	230	219	259	246
CLYBL__NM_206808_GAAGACTTTCGAGCCAGCAT	CLYBL__NM_206808	112	158	104	114
CLYBL__NM_206808_GAGGAACTATATCCAAGTCT	CLYBL__NM_206808	1005	1103	1224	1078
CLYBL__NM_206808_GTGTACATTGACTTTCGAGA	CLYBL__NM_206808	290	284	284	306
CLYBL__NM_206808_TCTAGAAAGAGACCTACTTG	CLYBL__NM_206808	59	84	86	42
CLYBL__NM_206808_TGTACACCAGATCTATGGCT	CLYBL__NM_206808	392	556	473	589
CLYBL__NM_206808_TTCCAGCCTGATGCTACCAA	CLYBL__NM_206808	28	50	35	18
CMA1__NM_001836_ACCTGGGAGAATGTGCCGGG	CMA1__NM_001836	1017	1322	1281	1226
CMA1__NM_001836_AGGGCCGGTAATGGGAGATT	CMA1__NM_001836	1777	1736	1717	1764
CMA1__NM_001836_ATTTCCAGGTAGGCCATGTA	CMA1__NM_001836	446	325	445	525
CMA1__NM_001836_CATCGTATCCTATGGACGGT	CMA1__NM_001836	2113	2449	2449	2510
CMA1__NM_001836_CCCACCTGGGAGAATGTGCC	CMA1__NM_001836	451	659	501	669
CMA1__NM_001836_CTTCTTCCTCTGTTATGTTA	CMA1__NM_001836	219	291	265	346
CMA1__NM_001836_CTTTGCATCCGACCGTCCAT	CMA1__NM_001836	1257	1935	1555	1473
CMA1__NM_001836_GAAGACAAAATCTGCATTTA	CMA1__NM_001836	924	1059	1155	1318
CMA1__NM_001836_GCACAAAGTTCCGTCTTATA	CMA1__NM_001836	474	519	533	609
CMA1__NM_001836_GCCCAGGGCATCGTATCCTA	CMA1__NM_001836	923	1154	1042	1026
CMA1__NM_001836_GTCCATAGGATACGATGCCC	CMA1__NM_001836	487	308	457	308
CMA1__NM_001836_TTCTTCCTCTGTTATGTTAT	CMA1__NM_001836	23	13	20	18
CMAS__NM_018686_AATGCGAGCTGAACATAGTG	CMAS__NM_018686	315	206	334	181
CMAS__NM_018686_ACCTCCGTCTCCAACCCGCG	CMAS__NM_018686	400	350	390	310
CMAS__NM_018686_GAGAATGTGGCCAAACAATT	CMAS__NM_018686	196	109	194	123
CMAS__NM_018686_GATAGAGATGGGTTACTTGC	CMAS__NM_018686	200	90	270	190
CMAS__NM_018686_GCACCTGGCAGCCCTAATTC	CMAS__NM_018686	787	267	794	251
CMAS__NM_018686_GCAGAAATGATTCGAGAAGA	CMAS__NM_018686	324	371	285	441
CMAS__NM_018686_GCAGCCCTAATTCTGGCCCG	CMAS__NM_018686	1067	507	1309	788
CMAS__NM_018686_GCCTCCCCGGGCCAGAATTA	CMAS__NM_018686	100	44	115	66
CMAS__NM_018686_TGAGACGCCATCAGTTTCGA	CMAS__NM_018686	1296	737	1651	845
CMAS__NM_018686_TGGATTTAAATTCAGAGGTT	CMAS__NM_018686	9	0	8	0
CMAS__NM_018686_TTGCAGAGCAAAGAGTATTA	CMAS__NM_018686	149	66	157	118
CMAS__NM_018686_TTTCACTCCATCGAAACTGA	CMAS__NM_018686	80	41	100	147
CMBL__NM_138809_AAATACTCAGAATTCAGGGC	CMBL__NM_138809	349	399	410	506
CMBL__NM_138809_AAGATAGACCAGTCGCCAGA	CMBL__NM_138809	344	453	379	217
CMBL__NM_138809_ACCAAATCCCCCGTTGATGC	CMBL__NM_138809	1118	1363	1238	1335
CMBL__NM_138809_AGATAGACCAGTCGCCAGAG	CMBL__NM_138809	280	509	295	208
CMBL__NM_138809_AGCTTTGCCTGCATCAACGG	CMBL__NM_138809	429	241	457	386
CMBL__NM_138809_ATTGGCCACAGACTTGAGTA	CMBL__NM_138809	182	128	302	305
CMBL__NM_138809_CAGCAAAAATGAACAAAGTG	CMBL__NM_138809	116	106	104	128
CMBL__NM_138809_GAAATGCCCAGAAGATCGAT	CMBL__NM_138809	1503	1784	1741	1808
CMBL__NM_138809_GATGAAATACTCAGAATTCA	CMBL__NM_138809	202	238	298	217
CMBL__NM_138809_GCCCAGAAAATTGGCATCGT	CMBL__NM_138809	1860	2020	2294	2100
CMBL__NM_138809_GTCAGCTATATATCTGGTAT	CMBL__NM_138809	340	394	361	597
CMBL__NM_138809_TGATGAAATACTCAGAATTC	CMBL__NM_138809	319	367	443	593
CMC1__NM_182523_TCCAGAGTTCTTGCAACATT	CMC1__NM_182523	575	1024	692	911
CMC1__NM_182523_TGCGGGGTCGAGCGCCATCT	CMC1__NM_182523	1377	1362	1287	1194
CMC1__NM_182523_TTCTTATGGTAGTAAAATGC	CMC1__NM_182523	7	0	8	25
CMC1__NM_182523_TTGCATTCTTCATAAAAGGC	CMC1__NM_182523	206	198	233	259
CMC2__NM_020188_CGTCTTGATTAACTTGCTTA	CMC2__NM_020188	599	658	556	680
CMC4__NM_001018024_GAGATACAGAAATGTTTACA	CMC4__NM_001018024	296	326	354	240
CMC4__NM_001018024_GGAAGATCTGTCGTCTGTTC	CMC4__NM_001018024	612	904	614	884
CMC4__NM_001018024_TAAACATTTCTGTATCTCAC	CMC4__NM_001018024	141	204	174	201
CMC4__NM_001018024_TTGATGCAGACTTCCGTGTT	CMC4__NM_001018024	1102	1201	1187	1478
CMIP__NM_030629_AACGTCTTCAATTGCGCTGT	CMIP__NM_030629	1232	1301	1474	1321
CMIP__NM_030629_AAGAGGAGCATCAGCGGGTC	CMIP__NM_030629	396	392	520	671
CMIP__NM_030629_AAGATCCCCTTGCTCCACTA	CMIP__NM_030629	747	784	781	819
CMIP__NM_030629_AAGCTAAAAGAATTCGGCGG	CMIP__NM_030629	291	259	316	246
CMIP__NM_030629_AAGGAGTGTCTTCGTTGCAG	CMIP__NM_030629	271	225	392	406
CMIP__NM_030629_AATGGAACCACTGGTCTCGC	CMIP__NM_030629	1400	1680	1540	1638
CMIP__NM_030629_ACCTTTGACCTGATCTAAAG	CMIP__NM_030629	164	154	262	284
CMIP__NM_030629_ACGGGTGCAGTGATGTGACT	CMIP__NM_030629	883	735	978	904
CMIP__NM_030629_ACGTGAAGCACACGATGGCA	CMIP__NM_030629	1824	2100	1825	1828
CMIP__NM_030629_AGAAAGAGCTTGGGCCTTAG	CMIP__NM_030629	288	168	292	253
CMIP__NM_030629_AGAGGAGCATCAGCGGGTCG	CMIP__NM_030629	493	405	583	682
CMIP__NM_030629_AGGGGTGACAGAGTCTCCGT	CMIP__NM_030629	327	188	366	319
CMIP__NM_030629_AGTATAAAACGACAAAAGCA	CMIP__NM_030629	189	249	191	299
CMIP__NM_030629_AGTGCTGTGAAGTATGGGAC	CMIP__NM_030629	565	514	680	681
CMIP__NM_030629_ATGAGGCAGTCGATGAGGTT	CMIP__NM_030629	2417	2736	2489	2539
CMIP__NM_030629_ATGTTGACAAATTGCGGGTC	CMIP__NM_030629	410	500	420	484
CMIP__NM_030629_CACGGAAAATGAATTAAGAC	CMIP__NM_030629	530	918	636	798
CMIP__NM_030629_CCAGTCAGCTGTCGGGTTAG	CMIP__NM_030629	1518	1811	1687	1943
CMIP__NM_030629_CTCCAACTCCCTCACGAGGT	CMIP__NM_030629	707	972	799	940
CMIP__NM_030629_CTGTGAAGTATGGGACCGGA	CMIP__NM_030629	776	833	864	963
CMIP__NM_030629_CTTGGGCCTTAGTGGAGCAA	CMIP__NM_030629	355	530	364	364
CMIP__NM_030629_CTTGGGGAATTGTCGCGGCT	CMIP__NM_030629	2621	2919	2940	3928
CMIP__NM_030629_GAGCTGTAAACAGTACTTCG	CMIP__NM_030629	848	788	952	723
CMIP__NM_030629_GCCTGAGCCCCACCTCGTGA	CMIP__NM_030629	262	209	351	459
CMIP__NM_030629_GCTTGGGCCTTAGTGGAGCA	CMIP__NM_030629	506	977	497	621
CMIP__NM_030629_GCTTGGGGAATTGTCGCGGC	CMIP__NM_030629	1296	1552	1405	1917
CMIP__NM_030629_GGAAGAAAGCATCCTTCGTC	CMIP__NM_030629	514	608	767	787
CMIP__NM_030629_GGCCTCCAGTGAGATCCACG	CMIP__NM_030629	1978	2037	2041	1891
CMIP__NM_030629_GGGGAGGGCAGTCGGACTGG	CMIP__NM_030629	1085	1657	1109	1311
CMIP__NM_030629_GGGTCTTCTTGTCCCAAGGG	CMIP__NM_030629	99	54	98	132
CMIP__NM_030629_GGTGGGGAGGGCAGTCGGAC	CMIP__NM_030629	239	279	331	435
CMIP__NM_030629_GTCTGAGGCCCGGCTCAAGT	CMIP__NM_030629	1868	2043	1802	1799
CMIP__NM_030629_GTGAGCTGTAAACAGTACTT	CMIP__NM_030629	331	243	386	385
CMIP__NM_030629_GTTTCGAGACGATTTCCAGT	CMIP__NM_030629	1316	1048	1422	961
CMIP__NM_030629_TACAAAGTCCACTTTAGATC	CMIP__NM_030629	519	471	546	688
CMIP__NM_030629_TCAGGAAAAGCAACAGTGCG	CMIP__NM_030629	203	372	313	419
CMIP__NM_030629_TCAGGCTTGGGGAATTGTCG	CMIP__NM_030629	1142	1195	1184	1262
CMIP__NM_030629_TCCAACTCCCTCACGAGGTG	CMIP__NM_030629	15	8	63	81
CMIP__NM_030629_TCGTTGCAGTGGTCACATGT	CMIP__NM_030629	1222	1180	1422	1720
CMIP__NM_030629_TGAGTAAACAGCCTCAGTCG	CMIP__NM_030629	1454	1545	1435	1158
CMIP__NM_030629_TGTTGACAAATTGCGGGTCT	CMIP__NM_030629	116	96	191	203
CMIP__NM_030629_TTAAAAAAATGAGGGCATAG	CMIP__NM_030629	16	9	11	10
CMIP__NM_030629_TTGTGTTTGATGCCGTCGTG	CMIP__NM_030629	236	324	371	488
CMIP__NM_198390_CGCCCATCTTCGTGCCTTCG	CMIP__NM_198390	6107	8093	6994	9090
CMIP__NM_198390_GCCTCAGGAATTTCGAGGTG	CMIP__NM_198390	791	1061	898	1032

[illegible]

COBLL1__NM_001278458_ATGTGGACTCACTCTCACTA	COBLL1__NM_001278458	549	681	642	753
COBLL1__NM_001278458_CAGCACGGCCGCACATAAGT	COBLL1__NM_001278458	614	514	694	648
COBLL1__NM_001278458_GACAAAATCTCTTAATGACC	COBLL1__NM_001278458	338	608	398	448
COBLL1__NM_001278458_GCAGCACGGCCGCACATAAG	COBLL1__NM_001278458	227	232	218	201
COBLL1__NM_001278458_TGAAGAGACATCAGTATATT	COBLL1__NM_001278458	298	248	298	397
COBLL1__NM_001278458_TTCAACAATAGTGTATGCAA	COBLL1__NM_001278458	8	2	8	11
COBLL1__NM_001278458_TTTAAGCCAAACACACCAAT	COBLL1__NM_001278458	111	140	93	117
COBLL1__NM_001278461_ACTAAGAGAATTATATGCGA	COBLL1__NM_001278461	349	308	392	443
COBLL1__NM_001278461_GCGGTGCTTATTTACTAGAG	COBLL1__NM_001278461	204	67	257	293
COCH__NM_001135058_AGGGGAGTAATCAGCAACTC	COCH__NM_001135058	110	189	87	123
COCH__NM_001135058_AGTCTCAAATGCTTTCTAGA	COCH__NM_001135058	333	173	319	402
COCH__NM_001135058_ATGTGGGCCTTGTTCAAGCC	COCH__NM_001135058	379	377	351	323
COCH__NM_001135058_CTTCTGTATCGAGCATATGT	COCH__NM_001135058	257	144	245	315
COCH__NM_001135058_CTTCTTTCACAGTAACTAGT	COCH__NM_001135058	180	384	241	274
COCH__NM_001135058_CTTGAGGAATTCTCTGTGTA	COCH__NM_001135058	694	933	793	1089
COCH__NM_001135058_GAGAAGAAAACTGGCAATAA	COCH__NM_001135058	136	156	113	109
COCH__NM_001135058_GCTTCTGTATCGAGCATATG	COCH__NM_001135058	379	548	399	445
COCH__NM_001135058_GGAAAAGTGGCTCTAATGTT	COCH__NM_001135058	480	602	580	838
COCH__NM_001135058_GGTAGGCTATAGACTCGTAC	COCH__NM_001135058	229	136	232	160
COCH__NM_001135058_GTAAAACATGTGATAGCAAT	COCH__NM_001135058	89	94	93	86
COCH__NM_001135058_GTACGAGTCTATAGCCTACC	COCH__NM_001135058	184	201	243	237
COCH__NM_004086_ATCACACTGAATAAGAGAGC	COCH__NM_004086	190	239	189	222
COG1__NM_018714_AAGATGTTGCTCAAATGCCA	COG1__NM_018714	301	50	269	149
COG1__NM_018714_ACGGTACCGCGACCTGATCG	COG1__NM_018714	1015	455	913	610
COG1__NM_018714_CACGGCGTCCACTAGCCCCA	COG1__NM_018714	1104	628	1114	747
COG1__NM_018714_CCGGATGAGTATAGGAAACC	COG1__NM_018714	607	461	631	474
COG1__NM_018714_CTAAGAGCATTATAGAGCAC	COG1__NM_018714	221	89	219	38
COG1__NM_018714_GCGTCTCGAAAAGAGCCGCA	COG1__NM_018714	43	76	29	18
COG1__NM_018714_GCTGGATCTGCGCGACCCTG	COG1__NM_018714	654	262	777	477
COG1__NM_018714_GGAGAGGACGGGACTGTATC	COG1__NM_018714	486	213	415	221
COG1__NM_018714_GGGAGAGGACGGGACTGTAT	COG1__NM_018714	746	306	749	371
COG1__NM_018714_GTTTCCTATACTCATCCGGC	COG1__NM_018714	339	199	409	213
COG1__NM_018714_TAAGAGCATTATAGAGCACA	COG1__NM_018714	376	147	408	177
COG1__NM_018714_TTCTAAGAGTAGCTTGATCT	COG1__NM_018714	8	0	26	6
COG2__NM_001145036_ACGCCACGATTGACAAGACA	COG2__NM_001145036	789	807	963	690
COG2__NM_001145036_ACGTCTGAAGGCCTTCTAAT	COG2__NM_001145036	1178	903	1286	1145
COG2__NM_001145036_AGAGGACATTAGGAAAAAAA	COG2__NM_001145036	8	14	0	0
COG2__NM_001145036_CCAAAATTTGTCCAGTCAAA	COG2__NM_001145036	356	409	392	602
COG2__NM_001145036_CGTCTGACGTCGATATAATA	COG2__NM_001145036	351	464	422	246
COG2__NM_001145036_CTGGAAGGTCTCCTATTAGA	COG2__NM_001145036	409	391	512	365
COG2__NM_001145036_CTTCACTGACAGACGATCTA	COG2__NM_001145036	439	208	500	326
COG2__NM_001145036_CTTGTTGATGAGTTCGACCA	COG2__NM_001145036	559	251	665	495
COG2__NM_001145036_GTCCCGTGTCTTGTCAATCG	COG2__NM_001145036	1040	490	1071	528
COG2__NM_001145036_TGAACGAATGTCTAAACAAG	COG2__NM_001145036	71	108	76	123
COG2__NM_001145036_TGAGGCTTATACAAGTTATT	COG2__NM_001145036	204	132	247	289
COG2__NM_001145036_TTTGGGACAATTACGAGAAG	COG2__NM_001145036	36	0	26	28
COG3__NM_031431_AAGAATTGAAACCGCACAGC	COG3__NM_031431	44	44	40	11
COG3__NM_031431_ACATTGTCGGTGAATAGTGA	COG3__NM_031431	350	148	367	180
COG3__NM_031431_ACTACTGAAGTCAGTTCAAT	COG3__NM_031431	788	398	858	374
COG3__NM_031431_ACTATTCACCGACAATGTAG	COG3__NM_031431	184	146	239	118
COG3__NM_031431_AGATGAGGGATTACTTGTCT	COG3__NM_031431	578	207	667	340
COG3__NM_031431_CACTACTGAAGTCAGTTCAA	COG3__NM_031431	516	288	660	336
COG3__NM_031431_CACTATTCACCGACAATGTA	COG3__NM_031431	225	137	239	165
COG3__NM_031431_CAGGCAGACGGACTCGGTAT	COG3__NM_031431	1511	550	1650	810
COG3__NM_031431_GACCGACAGGCAGACGGACT	COG3__NM_031431	1027	768	1145	1242
COG3__NM_031431_GGCTCTCTGGGATCGGAGAC	COG3__NM_031431	124	118	233	185
COG3__NM_031431_TCACTATTCACCGACAATGT	COG3__NM_031431	316	120	395	127
COG3__NM_031431_TGACGGATTTATACCTATGC	COG3__NM_031431	266	225	259	170
COG4__NM_001195139_AGCTGGAGGCTGTATACGAA	COG4__NM_001195139	323	314	319	268
COG4__NM_001195139_CAGATTCTCCTCAGCTTTAC	COG4__NM_001195139	427	414	413	621
COG4__NM_001195139_CAGGGAGCGAATGAGCTCAG	COG4__NM_001195139	165	34	227	49
COG4__NM_001195139_CCCACTGCTGGGTTTGCATG	COG4__NM_001195139	2975	939	3606	1754
COG4__NM_001195139_CCGCCATCTTGGTCCCCATT	COG4__NM_001195139	2592	3315	3091	3869
COG4__NM_001195139_CTCTCTGAATGGCCTGATAG	COG4__NM_001195139	81	107	95	94
COG4__NM_001195139_GAGGAGTGAAGATTATGAGC	COG4__NM_001195139	159	86	175	83
COG4__NM_001195139_GATGGTCACTCTCCACCGAA	COG4__NM_001195139	295	140	313	139
COG4__NM_001195139_GTCAAGCTGACGAACTTTGC	COG4__NM_001195139	498	515	667	776
COG4__NM_001195139_TACTCATCGCTACTTGTGCC	COG4__NM_001195139	479	893	554	943
COG4__NM_001195139_TCTGAATGGCCTGATAGAGG	COG4__NM_001195139	392	159	471	245
COG4__NM_001195139_TGGGGACAGACATGAGTGAT	COG4__NM_001195139	183	65	195	63
COG4__NM_015386_AAGCGGGCTCCTAATGAGGT	COG4__NM_015386	253	351	368	420
COG4__NM_015386_AGCCCAGACCTTTCTACGGC	COG4__NM_015386	1615	1692	1842	1941
COG4__NM_015386_CAAAGTCAAGCCTAACTGCT	COG4__NM_015386	398	475	416	580
COG4__NM_015386_CAAGTTGTCTAGGACTGGCA	COG4__NM_015386	443	515	464	667
COG4__NM_015386_CATCTGGGGACCTAGCAGTT	COG4__NM_015386	729	662	709	565
COG4__NM_015386_CTAGACAACTTGGGTACATC	COG4__NM_015386	391	501	418	493
COG4__NM_015386_CTCTGCTGCCAGCCGTAGAA	COG4__NM_015386	281	267	328	503
COG4__NM_015386_CTTGCAGGCCTGTTCCCTAA	COG4__NM_015386	1022	1069	1275	1090
COG4__NM_015386_GCCTGCAAGTGTGATGAGCC	COG4__NM_015386	495	732	741	801
COG4__NM_015386_TTGCAGGCCTGTTCCCTAAG	COG4__NM_015386	308	253	226	217
COG4__NM_015386_TTGTTTGCTGTAGTGTTTGA	COG4__NM_015386	235	247	143	214
COG5__NM_001161520_ACGAGAATTGGGGCTTTACA	COG5__NM_001161520	896	430	1046	913
COG5__NM_001161520_ACTGGGATTGAGTCGTTGGA	COG5__NM_001161520	307	192	396	308
COG5__NM_001161520_AGCAACTGGGATTGAGTCGT	COG5__NM_001161520	1277	850	1137	1257
COG5__NM_001161520_AGGTTGCCTGTGATTTGCTT	COG5__NM_001161520	568	214	561	427
COG5__NM_001161520_CAGATGATGCAGACGAGAAT	COG5__NM_001161520	371	320	371	424
COG5__NM_001161520_CAGGTGGTGACGCAGAGATCC	COG5__NM_001161520	118	172	152	134
COG5__NM_001161520_CATACAATAAGATAGTTGCC	COG5__NM_001161520	337	194	419	329
COG5__NM_001161520_CGGGCAACTATCTTATTGTA	COG5__NM_001161520	831	344	1029	1079
COG5__NM_001161520_CTTTACAGGGAGCTGTTGAT	COG5__NM_001161520	597	326	818	695
COG5__NM_001161520_GACGAGAATTGGGGCTTTAC	COG5__NM_001161520	612	397	688	596
COG5__NM_001161520_GGACAGAGAACTACACTTAC	COG5__NM_001161520	640	719	691	691
COG5__NM_001161520_TGCAAGAGCCCGACTTGAAG	COG5__NM_001161520	382	657	383	552
COG6__NM_001145079_CCTTTCAGATTGAAGCTTAG	COG6__NM_001145079	227	342	232	330
COG6__NM_001145079_CGTATCTCCAGTATTGACAC	COG6__NM_001145079	496	231	556	444
COG6__NM_001145079_CTGCCAACGGCCTCAACAAT	COG6__NM_001145079	274	283	355	366
COG6__NM_001145079_CTTATGAAAGACTTTACCGA	COG6__NM_001145079	523	410	634	488
COG6__NM_001145079_CTTGCGTACAAATCAACAAA	COG6__NM_001145079	223	155	223	157
COG6__NM_001145079_GACGCGGCTGGACAACGACA	COG6__NM_001145079	567	649	600	569
COG6__NM_001145079_GCGCAAGCTGCATAAGATCC	COG6__NM_001145079	331	175	473	474
COG6__NM_001145079_GGTCGCAGTGTCTGCGACCG	COG6__NM_001145079	0	0	0	0
COG6__NM_001145079_TATGCAGCTTGCGCGACAGC	COG6__NM_001145079	13	19	2	0
COG6__NM_001145079_TCCCGCCTGCCCCATTGTTG	COG6__NM_001145079	1306	1369	1581	1335
COG6__NM_001145079_TTATGAAAGACTTTACCGAT	COG6__NM_001145079	229	133	300	314
COG6__NM_001145079_TTATGCAGCTTGCGCGACAG	COG6__NM_001145079	74	68	67	98
COG7__NM_153603_AGCCAGTCCACAGATTGTAG	COG7__NM_153603	484	437	471	465
COG7__NM_153603_CACGTTGAGCGCCGATATTG	COG7__NM_153603	754	335	833	599
COG7__NM_153603_CGTGAAGGAGTGGATCAATG	COG7__NM_153603	969	218	1139	489
COG7__NM_153603_CTCTTCAGGAAGCAGATAAG	COG7__NM_153603	36	12	38	35
COG7__NM_153603_CTTAAATGTCTCCTCAATAT	COG7__NM_153603	830	549	739	456
COG7__NM_153603_GGCAGACGACTTCGACGTGA	COG7__NM_153603	1258	451	1264	732
COG7__NM_153603_GTGAATGCCGCTACAATCTG	COG7__NM_153603	333	457	331	255
COG7__NM_153603_GTGATTTCTGCCAAGCTAAC	COG7__NM_153603	34	39	45	18
COG7__NM_153603_TACAATCTGTGGACTGGCTA	COG7__NM_153603	214	538	279	345
COG7__NM_153603_TCACGTCGAAGTCGTCTGCC	COG7__NM_153603	754	386	716	592
COG7__NM_153603_TGATGTTGAAGCCCTAAAAC	COG7__NM_153603	121	27	92	62
COG7__NM_153603_TGTTGAAGCCCTAAAACAGG	COG7__NM_153603	395	94	305	248
COG8__NM_032382_AAACAGGCGGTGGATGCGCT	COG8__NM_032382	145	65	118	94
COG8__NM_032382_ACGCCTCCACGTCGCCAAAC	COG8__NM_032382	765	282	964	531
COG8__NM_032382_CCACCGCCTGTTTGGCGACT	COG8__NM_032382	374	225	441	253
COG8__NM_032382_CCGGAGGTAGCGGCCCACAT	COG8__NM_032382	167	100	176	191
COG8__NM_032382_CCTCCGGGAGTTGAGCGGCT	COG8__NM_032382	1004	671	1120	782
COG8__NM_032382_GAAGGTCTTGTAGTTAGCGA	COG8__NM_032382	47	24	74	67
COG8__NM_032382_GACCGCGGCGACTATCCCAT	COG8__NM_032382	2082	1078	1928	1704
COG8__NM_032382_GCACTCGGCGCCGCGGATGA	COG8__NM_032382	1097	284	1164	510
COG8__NM_032382_GCTGTGGCCGTGGCTACCGA	COG8__NM_032382	906	808	1041	1017
COG8__NM_032382_GGTCCCGGAACAGCGACGCC	COG8__NM_032382	161	158	194	225
COG8__NM_032382_TACCGATGGGATAGTCGCCG	COG8__NM_032382	520	512	710	700
COG8__NM_032382_TGGCGCGAGCGGCCCGATGT	COG8__NM_032382	179	86	181	122
COIL__NM_004645_AAAGCAACCTGTGGCACAGT	COIL__NM_004645	498	465	461	435
COIL__NM_004645_AAGCCGTAGCCTAACCGTCT	COIL__NM_004645	568	558	602	462
COIL__NM_004645_AATTCTGTAGTCATCAGTAA	COIL__NM_004645	256	191	304	196
COIL__NM_004645_AGTCTCATCCGCCAGCGCTT	COIL__NM_004645	476	309	481	481
COIL__NM_004645_AGTGCGGGGTAGCTGGCGGC	COIL__NM_004645	524	283	630	435
COIL__NM_004645_AGTGGGTGATGATAACGAAG	COIL__NM_004645	164	223	161	160
COIL__NM_004645_CGGGCATTTCAGTTAGAGGA	COIL__NM_004645	361	440	448	500
COIL__NM_004645_GAAGCGGGCATTTCAGTTAG	COIL__NM_004645	1023	477	1089	705
COIL__NM_004645_GCCAGAAGGCCGTACAGTGC	COIL__NM_004645	263	253	254	249
COIL__NM_004645_TAATGAGAAGGTCTTGGATC	COIL__NM_004645	241	407	237	272
COIL__NM_004645_TAATGAGATCTGTGACGACT	COIL__NM_004645	385	354	562	493
COIL__NM_004645_TGGCAGCTTCCGAGACGGTT	COIL__NM_004645	98	147	101	51
COL10A1__NM_000493_AAACCAGGCTACGGAAGTCC	COL10A1__NM_000493	494	481	540	507
COL10A1__NM_000493_AAGAACTGTGTCTTGGTGTT	COL10A1__NM_000493	613	449	643	586
COL10A1__NM_000493_AGCAGCAAAAAGGGTATTTG	COL10A1__NM_000493	584	533	836	663
COL10A1__NM_000493_CAAATGCCCACAGGCATAAA	COL10A1__NM_000493	72	39	71	112
COL10A1__NM_000493_CCACCAGGAAAACCAGGCTA	COL10A1__NM_000493	474	209	442	387
COL10A1__NM_000493_CCCTACACCATAAAGAGTAA	COL10A1__NM_000493	281	503	305	270
COL10A1__NM_000493_CCTTTACTCTTTATGGTGTA	COL10A1__NM_000493	470	719	675	729
COL10A1__NM_000493_CTGGGAGACCATGGCTACCC	COL10A1__NM_000493	320	369	428	418
COL10A1__NM_000493_CTTTCTGTCCATTCATACCA	COL10A1__NM_000493	558	652	684	611
COL10A1__NM_000493_GAAGAACTGTGTCTTGGTGT	COL10A1__NM_000493	1100	1230	1174	1497
COL10A1__NM_000493_GTATCCTTGAACTTGGTTCA	COL10A1__NM_000493	196	163	346	282
COL10A1__NM_000493_TTGTTCCTGGAATCCCTGGC	COL10A1__NM_000493	206	297	245	177
COL11A1__NM_001190709_ACTTCTATCAAGTGGTTTCG	COL11A1__NM_001190709	345	322	347	345
COL11A1__NM_001190709_AGAACTGTTAACATCGCTGA	COL11A1__NM_001190709	588	564	704	929
COL11A1__NM_001190709_AGAGCAATTGTTGATACCAA	COL11A1__NM_001190709	906	861	858	1099
COL11A1__NM_001190709_CCTCTAGGTGGAAAACGAAA	COL11A1__NM_001190709	36	18	24	77
COL11A1__NM_001190709_CTAGGTGGAAAACGAAACGG	COL11A1__NM_001190709	84	72	106	117
COL11A1__NM_001190709_CTCCTCTGACCTCTCTAGCT	COL11A1__NM_001190709	363	358	473	553
COL11A1__NM_001190709_CTTCCTCTTCCAAGCTAGAG	COL11A1__NM_001190709	115	60	105	117
COL11A1__NM_001190709_GCATCGGGTAGCAATCAGCG	COL11A1__NM_001190709	734	728	793	946
COL11A1__NM_001190709_GGTCAATGCGAGGGTTGTTA	COL11A1__NM_001190709	513	734	642	815
COL11A1__NM_001190709_TCCAAAAACCGTGATTCCAT	COL11A1__NM_001190709	1166	1148	1265	1261
COL11A1__NM_001190709_TGTTGATACCAATGGAATCA	COL11A1__NM_001190709	73	131	95	105
COL11A1__NM_001190709_TTTGATCACAGGTGATCCCA	COL11A1__NM_001190709	35	32	64	34
COL12A1__NM_004370_AAAACCAGTTGATCCAATTG	COL12A1__NM_004370	157	291	274	308
COL12A1__NM_004370_AAACCAGTTGATCCAATTGT	COL12A1__NM_004370	669	715	835	536
COL12A1__NM_004370_AATAACGGTGGACCCTACAA	COL12A1__NM_004370	154	164	209	189
COL12A1__NM_004370_ATAACGGTGGACCCTACAAC	COL12A1__NM_004370	180	101	125	81
COL12A1__NM_004370_ATCAACACCTGAGCACACCT	COL12A1__NM_004370	462	586	480	556
COL12A1__NM_004370_CTTGTTAGTTGTCCTATAAC	COL12A1__NM_004370	578	610	511	569
COL12A1__NM_004370_GAAGAAAGTGTACCAGTTAT	COL12A1__NM_004370	496	559	629	628
COL12A1__NM_004370_GCAAGAGAGCTTCGTAATGT	COL12A1__NM_004370	1303	1807	1452	1895
COL12A1__NM_004370_GGCCAACTTTGATGCAATTG	COL12A1__NM_004370	716	839	715	1086
COL12A1__NM_004370_GTTATTCTGTAACCCACAAT	COL12A1__NM_004370	44	37	54	53
COL12A1__NM_004370_TGTGGGTTACAGAATAACGG	COL12A1__NM_004370	81	243	96	74
COL12A1__NM_004370_TTTCAGTGGTACTAGCTGAA	COL12A1__NM_004370	135	223	194	314
COL12A1__NM_080645_AAAGGAGGCAATACTCTCAC	COL12A1__NM_080645	390	175	440	398
COL12A1__NM_080645_AATCTGCCACATTGTATGCA	COL12A1__NM_080645	431	510	532	456
COL12A1__NM_080645_AGACCTCGAGCTCGAAAAAT	COL12A1__NM_080645	119	213	163	253
COL12A1__NM_080645_CCAAAAGAGTACAAATTGGT	COL12A1__NM_080645	962	1350	1043	1574
COL12A1__NM_080645_GAGTTTCATTTCTCGTATTG	COL12A1__NM_080645	276	356	267	399
COL12A1__NM_080645_GCAAACTTGCCGTACAAAGG	COL12A1__NM_080645	60	9	63	32
COL12A1__NM_080645_GCTCACTCTAAAAGAACGAT	COL12A1__NM_080645	404	450	373	413
COL12A1__NM_080645_GTGGATGGATCATGGAGCAT	COL12A1__NM_080645	63	53	110	115
COL12A1__NM_080645_GTGGCAAACTTGCCGTACAA	COL12A1__NM_080645	47	66	74	46
COL12A1__NM_080645_TAGCTCTTGCTCAGTATAGT	COL12A1__NM_080645	33	48	48	76
COL12A1__NM_080645_TGATACCCATGCATACAATG	COL12A1__NM_080645	280	259	314	330
COL12A1__NM_080645_TTAGCTCTTGCTCAGTATAG	COL12A1__NM_080645	2	0	2	0
COL13A1__NM_001130103_AAACCTTCAGGGCAATTATT	COL13A1__NM_001130103	192	347	353	572
COL13A1__NM_001130103_AGGGGTCCCCCGGAGACGCT	COL13A1__NM_001130103	236	224	189	140
COL13A1__NM_001130103_AGTCGCACTGAATTGAGGAG	COL13A1__NM_001130103	388	383	434	521
COL13A1__NM_001130103_CAGCTCGGCCGTCCGAAAGT	COL13A1__NM_001130103	1136	826	1029	1034
COL13A1__NM_001130103_CATTGGGCTGGACGGCAAAC	COL13A1__NM_001130103	302	364	214	339
COL13A1__NM_001130103_CTCCACCCCCGGTCATAAAA	COL13A1__NM_001130103	230	277	237	349
COL13A1__NM_001130103_CTTCAGGGCAATTATTTGGC	COL13A1__NM_001130103	438	499	548	752
COL13A1__NM_001130103_GCAGCTCGGCCGTCCGAAAG	COL13A1__NM_001130103	382	383	410	645
COL13A1__NM_001130103_GGGACGAGTCAATCAACTGC	COL13A1__NM_001130103	488	448	769	636
COL13A1__NM_001130103_GGTTTGCCGTCCAGCCCAAT	COL13A1__NM_001130103	639	562	596	772
COL13A1__NM_001130103_GTCAGCAGCCCGCACGACCC	COL13A1__NM_001130103	714	610	840	928
COL13A1__NM_001130103_TGCCGAGTCCAGGGTCGTGC	COL13A1__NM_001130103	817	1043	956	975
COL13A1__NM_080798_CAGAGGTCCACTTGGTCCAG	COL13A1__NM_080798	1338	1033	1326	876
COL13A1__NM_080798_CCCTGGTGGACCTTGGATGC	COL13A1__NM_080798	763	937	777	900
COL13A1__NM_080798_GGCGATGCTGGCAACTCCAT	COL13A1__NM_080798	1390	1289	1639	1463
COL14A1__NM_021110_AATTGCTGACATTGTAATCC	COL14A1__NM_021110	783	827	894	831
COL14A1__NM_021110_ACAACTTTGACTCTGGGCTT	COL14A1__NM_021110	818	945	864	961
COL14A1__NM_021110_CAGTATACAGATTTCATGGA	COL14A1__NM_021110	628	662	825	820
COL14A1__NM_021110_CCACATCGAATGCTGTAACC	COL14A1__NM_021110	578	694	702	507
COL14A1__NM_021110_CCTGGTTACAGCATTCGATG	COL14A1__NM_021110	281	387	377	367
COL14A1__NM_021110_CTGGTTACAGCATTCGATGT	COL14A1__NM_021110	285	344	362	237
COL14A1__NM_021110_GATTCAACTTCAGACTGGTT	COL14A1__NM_021110	607	562	699	668
COL14A1__NM_021110_GCAACTAAAGCAATTATTCA	COL14A1__NM_021110	676	908	780	1113
COL14A1__NM_021110_GTCGATGGTTCATGGAGTAT	COL14A1__NM_021110	303	534	353	378
COL14A1__NM_021110_TAATCCTGGTCGATGGTTCA	COL14A1__NM_021110	287	441	395	412
COL14A1__NM_021110_TGAACTGTGTAATTCTGGTC	COL14A1__NM_021110	289	314	379	398
COL14A1__NM_021110_TGTAATTCTGGTCTGGCATA	COL14A1__NM_021110	75	57	91	102
COL15A1__NM_001855_AGCAGCCTCTTGGGACACAT	COL15A1__NM_001855	792	853	853	991
COL15A1__NM_001855_AGTAGAGGATGATCCGCTGG	COL15A1__NM_001855	817	572	979	588
COL15A1__NM_001855_ATAGCCTGTGACAAAGGATA	COL15A1__NM_001855	227	187	233	192
COL15A1__NM_001855_ATCCTCTACTACACGGAGCC	COL15A1__NM_001855	189	253	183	364
COL15A1__NM_001855_CCTGGCTGGGCGGCCAACAT	COL15A1__NM_001855	0	0	0	0
COL15A1__NM_001855_CTCCTCACAGTTCACGAGGA	COL15A1__NM_001855	293	335	231	293
COL15A1__NM_001855_GGCACCAGGCCGAAACTGT	COL15A1__NM_001855	434	285	423	476
COL15A1__NM_001855_GGCTTCCCGGCCTACAGTTT	COL15A1__NM_001855	645	898	803	1000
COL15A1__NM_001855_GTGACAAAGGATACGGACGA	COL15A1__NM_001855	231	284	230	269
COL15A1__NM_001855_TACCTGTCGCACCGCGGGTC	COL15A1__NM_001855	340	336	457	409
COL15A1__NM_001855_TGGCGAAGAGCACGCCACCA	COL15A1__NM_001855	10	34	8	5
COL15A1__NM_001855_TGTGACAAAGGATACGGACG	COL15A1__NM_001855	205	96	225	207
COL16A1__NM_001856_ACAGGCCTCATGGGTCGTCG	COL16A1__NM_001856	974	1161	1011	887
COL16A1__NM_001856_AGCCCAGGATACCCACATCC	COL16A1__NM_001856	706	589	826	625
COL16A1__NM_001856_CATCCAAGCCTAGAAATACA	COL16A1__NM_001856	444	581	568	540
COL16A1__NM_001856_CCCAGCTCTTCGACTTGCGT	COL16A1__NM_001856	623	775	966	967
COL16A1__NM_001856_CTGTGGATACCCATTTGCAT	COL16A1__NM_001856	1495	1658	1532	1723
COL16A1__NM_001856_GGGCCATGTATTTCTAGGCT	COL16A1__NM_001856	1006	1443	889	1380
COL16A1__NM_001856_GGGCCTCTCATCCTGCGCCT	COL16A1__NM_001856	1570	1929	2057	2396
COL16A1__NM_001856_TCAGTGTGCAGAAGGCCCGA	COL16A1__NM_001856	1079	994	1153	994
COL16A1__NM_001856_TCCAGAAGAGTATTCCCTCG	COL16A1__NM_001856	793	792	829	713
COL16A1__NM_001856_TGGGGCCCCGACGACCCATG	COL16A1__NM_001856	177	125	248	206
COL16A1__NM_001856_TGTTGACTTCCAGGGATATC	COL16A1__NM_001856	208	191	245	179
COL16A1__NM_001856_TTCAAGTGACCGATGCAAAT	COL16A1__NM_001856	86	57	116	154
COL17A1__NM_000494_ACCAAGAAAAACAAACGAGA	COL17A1__NM_000494	76	104	101	76
COL17A1__NM_000494_AGAGGACGGAGTCAAACACG	COL17A1__NM_000494	302	291	307	292
COL17A1__NM_000494_AGCCCTCCTGTAACTAGAGG	COL17A1__NM_000494	457	706	513	596
COL17A1__NM_000494_ATGCCTCCACCTCTAGTTAC	COL17A1__NM_000494	404	467	410	441
COL17A1__NM_000494_CCTCCACCTCTAGTTACAGG	COL17A1__NM_000494	906	864	1110	830
COL17A1__NM_000494_GAGTACCCTCGGAAGGAATT	COL17A1__NM_000494	375	316	477	477
COL17A1__NM_000494_GATACCTTCATACGCATGGC	COL17A1__NM_000494	206	255	154	211
COL17A1__NM_000494_GATGTTAAGCGTTTGCTCAA	COL17A1__NM_000494	386	272	324	390
COL17A1__NM_000494_GGAGTGTGTTGGAGGAATTC	COL17A1__NM_000494	192	104	213	307
COL17A1__NM_000494_GTTACCCGCCATGCGTATGA	COL17A1__NM_000494	1058	1117	1240	1332
COL17A1__NM_000494_TGATACCTTCATACGCATGG	COL17A1__NM_000494	232	200	300	372
COL17A1__NM_000494_TGGTAAGGATGTAAGTCTTG	COL17A1__NM_000494	23	12	15	14
COL18A1__NM_030582_ACAGGGGTAGGGAGCCATCG	COL18A1__NM_030582	592	395	760	501
COL18A1__NM_030582_AGACGGATGACCCCGACGTC	COL18A1__NM_030582	808	728	890	937
COL18A1__NM_030582_ATTCCGGGGGGTCACGTGTG	COL18A1__NM_030582	555	953	621	833
COL18A1__NM_030582_CAGGCCCTGGGCACCACTCA	COL18A1__NM_030582	1374	1132	1451	1737
COL18A1__NM_030582_GAAGGCCCAGCGAGGGCAAG	COL18A1__NM_030582	253	235	223	240
COL18A1__NM_030582_GAGCTAGGAATGCCACGGCT	COL18A1__NM_030582	1190	1477	1270	1471
COL18A1__NM_030582_GAGGAGAACATTGCCGGTGT	COL18A1__NM_030582	792	794	971	1082
COL18A1__NM_030582_GCTCTGTGGAACCATTCCGG	COL18A1__NM_030582	382	231	397	339
COL18A1__NM_030582_GGAAGTGGTACCGGGCCACT	COL18A1__NM_030582	645	795	673	876
COL18A1__NM_030582_GGCGCCCACACAACCGAGGC	COL18A1__NM_030582	1113	1404	1239	1719
COL18A1__NM_030582_GGCTCTGTGGAACCATTCCG	COL18A1__NM_030582	209	312	274	268
COL18A1__NM_030582_TGGGCCCCCTTGCCCTCGCT	COL18A1__NM_030582	646	765	708	935
COL18A1__NM_130445_ACCACGGTGGCTTCGTTAGG	COL18A1__NM_130445	104	191	149	203
COL18A1__NM_130445_CCCTGGGGGCCGCGTGAAAG	COL18A1__NM_130445	9	47	18	18
COL18A1__NM_130445_CCGCTTGCTCGGTCCTCACG	COL18A1__NM_130445	1904	2421	1871	2366
COL18A1__NM_130445_CTCACTGGGCACGGGAGACC	COL18A1__NM_130445	147	260	204	233
COL18A1__NM_130445_GCCGCTTGCTCGGTCCTCAC	COL18A1__NM_130445	973	1234	1090	1513
COL18A1__NM_130445_GCTGTTCGCCATCACGGACT	COL18A1__NM_130445	412	500	513	618
COL18A1__NM_130445_TCCTCGTCCAGGCAGTGCAT	COL18A1__NM_130445	266	285	379	406
COL18A1__NM_130445_TGCTCGGTCCTCACGGGGCC	COL18A1__NM_130445	91	258	58	128
COL18A1__NM_130445_TGTCCACTGGCCGACGAAGG	COL18A1__NM_130445	191	369	281	253
COL18A1__NM_130445_TTCCGGCTCCCCGCCTTCGT	COL18A1__NM_130445	108	208	115	109
COL19A1__NM_001858_AGTACGAAGAAACGCCAAAA	COL19A1__NM_001858	57	93	57	77
COL19A1__NM_001858_CATATAAAGTGAAATGACCT	COL19A1__NM_001858	520	433	548	572
COL19A1__NM_001858_CCAGGTCATTTCACTTTATA	COL19A1__NM_001858	512	663	821	916
COL19A1__NM_001858_CCATATAAAGTGAAATGACC	COL19A1__NM_001858	457	528	629	893
COL19A1__NM_001858_CTTCCACTTCCGTGACCGTT	COL19A1__NM_001858	588	943	696	1081
COL19A1__NM_001858_GATACCTGTCTTGTCCCTAA	COL19A1__NM_001858	764	1095	911	856
COL19A1__NM_001858_GTCCCTCTATTCTCAGGATA	COL19A1__NM_001858	197	220	221	324
COL19A1__NM_001858_GTCCTTTGTTTGATCGTCAG	COL19A1__NM_001858	459	353	465	460
COL19A1__NM_001858_GTTTCTTCGTACTCGAAACA	COL19A1__NM_001858	146	120	211	284
COL19A1__NM_001858_TGCCACTGACGATCAAACAA	COL19A1__NM_001858	122	82	216	259
COL19A1__NM_001858_TGTCTTGTCCCTAACGGTCA	COL19A1__NM_001858	278	219	194	157
COL19A1__NM_001858_TTCCACTTCCGTGACCGTTA	COL19A1__NM_001858	25	2	27	4
COL1A1__NM_000088_ACCGGAGGAATTTCCGTGCC	COL1A1__NM_000088	622	887	703	871
COL1A1__NM_000088_ATCATAGCCATAAGACAGCT	COL1A1__NM_000088	772	943	824	696
COL1A1__NM_000088_CATCACACCAGCCTGACCAC	COL1A1__NM_000088	1521	1191	1719	1486
COL1A1__NM_000088_CATCATAGCCATAAGACAGC	COL1A1__NM_000088	166	145	176	174
COL1A1__NM_000088_CATCATCTCCATTCTTTCCA	COL1A1__NM_000088	1566	1486	1781	1415
COL1A1__NM_000088_CCTGAGCCGTCGGGGCAGAC	COL1A1__NM_000088	588	548	640	532
COL1A1__NM_000088_CGGATCTGCGTCTGCGACAA	COL1A1__NM_000088	333	185	297	242
COL1A1__NM_000088_CTCTTGGCCGTGCGTCAGGA	COL1A1__NM_000088	237	305	189	267
COL1A1__NM_000088_GCCGGTCAAGATGGTCGCCC	COL1A1__NM_000088	976	1039	1060	908
COL1A1__NM_000088_GGGCGACCATCTTGACCGGC	COL1A1__NM_000088	1562	2022	1784	2391
COL1A1__NM_000088_GGTACCATGACCGAGACGTG	COL1A1__NM_000088	428	400	340	265
COL1A1__NM_000088_TTTGCTCCCCAGCTGTCTTA	COL1A1__NM_000088	400	173	456	236
COL1A2__NM_000089_AACAGGGCCAGGAATACCGC	COL1A2__NM_000089	388	460	461	655
COL1A2__NM_000089_ACAGGGCCAGGAATACCGCG	COL1A2__NM_000089	987	1154	1098	1130
COL1A2__NM_000089_ATTGGAGCTGTTGGTAACGC	COL1A2__NM_000089	720	1111	852	1157
COL1A2__NM_000089_CAACAGGGCCAGGAATACCG	COL1A2__NM_000089	573	546	631	644
COL1A2__NM_000089_CAGGTCCTTGGAAACCTTGA	COL1A2__NM_000089	436	640	547	576
COL1A2__NM_000089_CGTGGTCCTCTATCTCCGGC	COL1A2__NM_000089	1318	1704	1656	2179
COL1A2__NM_000089_GATGGAAAAGGAGTTGGACT	COL1A2__NM_000089	552	845	702	896
COL1A2__NM_000089_GTTGGTGCTGCCGGTGCTAC	COL1A2__NM_000089	462	622	461	772
COL1A2__NM_000089_TCCTCTGGCACCAGTAGCAC	COL1A2__NM_000089	311	278	362	431
COL1A2__NM_000089_TGCTCAGCTTTGTGGATACG	COL1A2__NM_000089	678	797	726	1091
COL1A2__NM_000089_TGGCCCCCCTGGTCTCGGTG	COL1A2__NM_000089	115	225	100	74
COL1A2__NM_000089_TTGGCATGTTGCTAGGCATA	COL1A2__NM_000089	41	46	70	37
COL20A1__NM_020882_AGGGTCTGGGCTCCCCGTGT	COL20A1__NM_020882	1076	539	1282	838
COL20A1__NM_020882_CAAGACCCCTAAGGCCACAG	COL20A1__NM_020882	373	467	428	388
COL20A1__NM_020882_CACCAGGAGTGCGCGGATCC	COL20A1__NM_020882	464	320	468	531
COL20A1__NM_020882_CGCACCCTTTGAAATCGGGC	COL20A1__NM_020882	2956	3501	3341	3829
COL20A1__NM_020882_CGCCGCCTCCGCTACAAGGG	COL20A1__NM_020882	157	79	112	110
COL20A1__NM_020882_CTTATCCGGCCCGATTTCAA	COL20A1__NM_020882	575	685	667	487
COL20A1__NM_020882_CTTTGAAATCGGGCCGGATA	COL20A1__NM_020882	474	579	458	647
COL20A1__NM_020882_GGACGGTGATGTCCCTCGGC	COL20A1__NM_020882	459	627	539	538
COL20A1__NM_020882_GTGCGCCGCCTCCGCTACAA	COL20A1__NM_020882	947	655	927	876
COL20A1__NM_020882_TGCAAGGTGTAGCCCTTGGA	COL20A1__NM_020882	91	95	115	143
COL20A1__NM_020882_TGTGCGCCGCCTCCGCTACA	COL20A1__NM_020882	1297	941	1432	1035
COL20A1__NM_020882_TGTTCCCCCCCTTGTAGCGG	COL20A1__NM_020882	619	368	517	385
COL21A1__NM_030820_AAAGGTAATCGAGCGCAAAC	COL21A1__NM_030820	85	77	144	103
COL21A1__NM_030820_ATAGCAGTGGTACTTACGGA	COL21A1__NM_030820	131	27	138	110
COL21A1__NM_030820_ATCGTGAGGACTTGGCAAAA	COL21A1__NM_030820	274	317	381	478
COL21A1__NM_030820_ATTCTGTGTTAGCTGAAGAT	COL21A1__NM_030820	173	129	130	112
COL21A1__NM_030820_ATTTCTGACTAAGATAGCAG	COL21A1__NM_030820	135	97	118	190
COL21A1__NM_030820_GAATCCATACTCTACTTAGG	COL21A1__NM_030820	427	375	501	460
COL21A1__NM_030820_GTAATCGAGCGCAAACTGGA	COL21A1__NM_030820	354	438	508	567
COL21A1__NM_030820_GTGGAATCCATACTCTACTT	COL21A1__NM_030820	124	133	150	166
COL21A1__NM_030820_GTTTCCTCCTAAGTAGAGTA	COL21A1__NM_030820	127	65	85	131
COL21A1__NM_030820_TAAGATAGCAGTGGTACTTA	COL21A1__NM_030820	221	215	245	200
COL21A1__NM_030820_TAGGAGGAAACACAAAGACA	COL21A1__NM_030820	607	393	653	506
COL21A1__NM_030820_TTAGATGGCTCTTATAGTGT	COL21A1__NM_030820	38	4	29	16
COL22A1__NM_152888_ACTTTGAGAAGGTCCGGCAG	COL22A1__NM_152888	196	147	184	157
COL22A1__NM_152888_AGGAGGACTTTGAGAAGGTC	COL22A1__NM_152888	189	163	210	221
COL22A1__NM_152888_CAAAAGTGTCCACTACGATC	COL22A1__NM_152888	1698	1141	1859	1476
COL22A1__NM_152888_CCCCGTGGTAGGCGAGACGC	COL22A1__NM_152888	765	639	803	774
COL22A1__NM_152888_CCGTGTTGGTGTTGCCCCCG	COL22A1__NM_152888	1238	1349	1582	1581
COL22A1__NM_152888_CTGGTGGACACCTTCGAGGT	COL22A1__NM_152888	247	221	272	269
COL22A1__NM_152888_CTTTGAGAAGGTCCGGCAGT	COL22A1__NM_152888	556	446	513	389
COL22A1__NM_152888_GACCCCCACACGGGTGCGGT	COL22A1__NM_152888	547	709	537	578
COL22A1__NM_152888_GCCCGGCGTCTCGCCTACCA	COL22A1__NM_152888	1027	806	1018	810
COL22A1__NM_152888_GGCATCCGCATCTTTGCCGT	COL22A1__NM_152888	3287	4699	3994	4967
COL22A1__NM_152888_GGGCGTGGGCGAGGCACTCA	COL22A1__NM_152888	556	633	711	737
COL22A1__NM_152888_TGGCATTGAAGTCGGACACG	COL22A1__NM_152888	79	16	81	63
COL23A1__NM_173465_AAAGGAGAGCCCGGACAAGA	COL23A1__NM_173465	280	485	288	380
COL23A1__NM_173465_AAAGGGGAAGCAGGCGAGAT	COL23A1__NM_173465	219	195	245	300
COL23A1__NM_173465_AGGGACTTCCAGGCCCGAAA	COL23A1__NM_173465	37	11	50	36
COL23A1__NM_173465_AGGGCGCTGACGGCCTCAAG	COL23A1__NM_173465	73	91	93	73
COL23A1__NM_173465_CAGGGCGCTGACGGCCTCAA	COL23A1__NM_173465	287	190	297	252
COL23A1__NM_173465_CCCGGTGCCCCAGGAATCGA	COL23A1__NM_173465	609	526	634	745
COL23A1__NM_173465_CCTGCTGCTCTCCGTGGGCT	COL23A1__NM_173465	3472	4125	3902	4933
COL23A1__NM_173465_CCTGGGCCAGCAGGACTCAA	COL23A1__NM_173465	178	207	236	277
COL23A1__NM_173465_CTTCTCTCCATCGATTCCTG	COL23A1__NM_173465	317	274	362	185
COL23A1__NM_173465_CTTTCCCAGTGTCGCCAGGA	COL23A1__NM_173465	643	895	636	823
COL23A1__NM_173465_GGGGGGCAGACACATTCGGA	COL23A1__NM_173465	676	445	877	731
COL23A1__NM_173465_TGGACGGACTAGCGAAGATC	COL23A1__NM_173465	26	30	25	25
COL24A1__NM_152890_AAATGGACCCCCTGAGGTAA	COL24A1__NM_152890	654	480	714	721
COL24A1__NM_152890_AGCCGTTTACAATATTAACT	COL24A1__NM_152890	738	577	739	432
COL24A1__NM_152890_CAACAAACATTGAGACACTT	COL24A1__NM_152890	1383	1526	1533	2020
COL24A1__NM_152890_CAGCCGTTTACAATATTAAC	COL24A1__NM_152890	243	248	305	302
COL24A1__NM_152890_CAGGTTGGTATTTGTCTGCT	COL24A1__NM_152890	530	373	552	746
COL24A1__NM_152890_CATCTACACCGTTACCTCAG	COL24A1__NM_152890	888	451	938	569
COL24A1__NM_152890_GAGTGTTCCGGTATCTTTGT	COL24A1__NM_152890	825	910	808	740
COL24A1__NM_152890_GCATCTACACCGTTACCTCA	COL24A1__NM_152890	1207	791	1223	817
COL24A1__NM_152890_GTCTCAATGTTTGTTGAGTG	COL24A1__NM_152890	296	329	387	625
COL24A1__NM_152890_GTTGGTATGAGAGTTGTACA	COL24A1__NM_152890	334	433	282	308
COL24A1__NM_152890_TAACTGGGTTACAGTCACAT	COL24A1__NM_152890	602	584	583	466
COL24A1__NM_152890_TTGGGCGGGGGAGAGTGTTC	COL24A1__NM_152890	527	274	522	268
COL25A1__NM_001256074_ACAGAAATCCTATGAACATA	COL25A1__NM_001256074	301	266	396	468
COL25A1__NM_001256074_AGGGCCGCCAGGACGGCACA	COL25A1__NM_001256074	32	90	29	30
COL25A1__NM_001256074_CAGGACGGCACACGGGGGCA	COL25A1__NM_001256074	966	1262	1145	1335
COL25A1__NM_001256074_CCGCTCTCGAATCCGCCAAA	COL25A1__NM_001256074	945	998	1204	1259
COL25A1__NM_001256074_CGCTCTCGAATCCGCCAAAG	COL25A1__NM_001256074	338	456	381	411
COL25A1__NM_001256074_GAGGATCTGATTACTGTGCA	COL25A1__NM_001256074	422	324	411	429
COL25A1__NM_001256074_GATCCTCGCCTGGAGGTCGT	COL25A1__NM_001256074	796	657	747	850
COL25A1__NM_001256074_GGGGTCGGGTCCTCGGATCT	COL25A1__NM_001256074	798	1131	852	893
COL25A1__NM_001256074_GTGGTGTCTTGCCTGTACCT	COL25A1__NM_001256074	181	92	167	221
COL25A1__NM_001256074_TCAGGCAGCAGATGAATGGA	COL25A1__NM_001256074	763	841	860	580
COL25A1__NM_001256074_TCCTGGTCCAAAAGGCGATA	COL25A1__NM_001256074	67	51	73	63
COL25A1__NM_001256074_TGCTGCTGAAGAAGCACGCA	COL25A1__NM_001256074	97	117	131	217
COL25A1__NM_032518_CCAGGCAGTGCCCTTATATC	COL25A1__NM_032518	719	558	734	783
COL25A1__NM_032518_GTACTCCTGGCATTCCACGG	COL25A1__NM_032518	916	586	1004	632
COL26A1__NM_001278563_ACTCCAGCACCGTCACCGTG	COL26A1__NM_001278563	1558	1571	1453	1633
COL26A1__NM_001278563_AGCAAGGACCCCATCGGGTT	COL26A1__NM_001278563	615	589	706	669
COL26A1__NM_001278563_AGGGTGAACCTGGCGTGAAG	COL26A1__NM_001278563	168	127	163	162
COL26A1__NM_001278563_CAGCAAGGACCCCATCGGGT	COL26A1__NM_001278563	1141	1484	1172	1368
COL26A1__NM_001278563_CCCGTGGGCCTTCTCTGTCG	COL26A1__NM_001278563	254	341	386	297
COL26A1__NM_001278563_CCTTCTTCCCCTTCACGCC	COL26A1__NM_001278563	1571	1975	1820	1893
COL26A1__NM_001278563_CGGTCACCTTTAGACCCGGG	COL26A1__NM_001278563	600	694	782	867
COL26A1__NM_001278563_CTCGGTCACCTTTAGACCCG	COL26A1__NM_001278563	610	669	768	777
COL26A1__NM_001278563_GGCAGCTCTGGTACACGCGC	COL26A1__NM_001278563	481	852	547	682
COL26A1__NM_001278563_GGCCCTGCGCCAACCTCGTA	COL26A1__NM_001278563	226	225	313	392
COL26A1__NM_001278563_TCACTGTCTTTGTCTGTAGG	COL26A1__NM_001278563	1256	1468	1567	1674
COL26A1__NM_001278563_TCGGTCACCTTTAGACCCGG	COL26A1__NM_001278563	250	243	335	404
COL27A1__NM_032888_AAGATGAAGCCCGACTGGAA	COL27A1__NM_032888	451	474	499	485
COL27A1__NM_032888_ACAATTACTGTACCCACCTG	COL27A1__NM_032888	507	650	673	743
COL27A1__NM_032888_AGGGACCCTGCACTCGACCC	COL27A1__NM_032888	780	522	753	657
COL27A1__NM_032888_AGGTCGGACTGGAAGGTAAA	COL27A1__NM_032888	118	160	116	194
COL27A1__NM_032888_CAGGGCGAGGTCGGACTGGA	COL27A1__NM_032888	1296	1492	1486	1700
COL27A1__NM_032888_GAAGGCATGGTTCACCCGGT	COL27A1__NM_032888	980	972	918	894
COL27A1__NM_032888_GACCCTGCACTCGACCCTGG	COL27A1__NM_032888	1423	1459	1626	1432
COL27A1__NM_032888_GGCAAACGAGACTAAGATCC	COL27A1__NM_032888	345	284	343	332
COL27A1__NM_032888_GTCGTGCATGTCGAGGTCGA	COL27A1__NM_032888	1339	1596	1286	1683
COL27A1__NM_032888_TCGACATGCACGACGGGCGC	COL27A1__NM_032888	777	865	669	1054
COL27A1__NM_032888_TGTCCTGCTGCCTTTCCACA	COL27A1__NM_032888	817	646	736	653
COL27A1__NM_032888_TGTGCGGCCTCGGAGCTCGA	COL27A1__NM_032888	244	334	320	293
COL28A1__NM_001037763_AAGAAAAAGGTGGATCAATT	COL28A1__NM_001037763	28	16	39	45
COL28A1__NM_001037763_AAGATTTGTGAATGTGAGAA	COL28A1__NM_001037763	43	39	5	5
COL28A1__NM_001037763_ACTTAAGAGAGAAGGGCGTA	COL28A1__NM_001037763	419	328	398	472
COL28A1__NM_001037763_AGATAAAATTCAAGATCGTC	COL28A1__NM_001037763	343	329	415	434
COL28A1__NM_001037763_AGGAGCGACCAGGAGTCAAT	COL28A1__NM_001037763	682	435	719	717
COL28A1__NM_001037763_CCCAGAAATCAAACGAAGTT	COL28A1__NM_001037763	387	382	484	665
COL28A1__NM_001037763_CCCAGGGCCATATGGACCAA	COL28A1__NM_001037763	26	23	72	169
COL28A1__NM_001037763_CTTAAGTAGCCTAGTGGCAT	COL28A1__NM_001037763	388	307	457	621
COL28A1__NM_001037763_GGAAATGGCATAATAAGAGA	COL28A1__NM_001037763	52	83	104	156
COL28A1__NM_001037763_TAGCCTAGTGGCATTGGAAA	COL28A1__NM_001037763	539	818	583	841
COL28A1__NM_001037763_TCGTAAACGCTGACAAAAGC	COL28A1__NM_001037763	0	0	0	0
COL28A1__NM_001037763_TTCTCTCTTAAGTAGCCTAG	COL28A1__NM_001037763	0	0	0	0
COL2A1__NM_001844_AAGGCTGGTGGCGCCCAGTT	COL2A1__NM_001844	747	699	778	416
COL2A1__NM_001844_CAAGGACCCAGAGGGGATCG	COL2A1__NM_001844	369	330	411	252
COL2A1__NM_001844_CAGGGTGCCCGAGGCAACGA	COL2A1__NM_001844	206	236	330	363
COL2A1__NM_001844_CCAGGCCTGGACGGTGCTAA	COL2A1__NM_001844	1560	1411	1627	1128
COL2A1__NM_001844_CCTCTCCCTTAGCACCGTCC	COL2A1__NM_001844	1401	1129	1527	905
COL2A1__NM_001844_CGGAGATCCGTTCTCACCCG	COL2A1__NM_001844	1769	1868	1781	1016
COL2A1__NM_001844_CTGGCAGCTGTGTGGAGGAT	COL2A1__NM_001844	805	1024	935	1051
COL2A1__NM_001844_CTGGCAGTGGCGAGGTCAGT	COL2A1__NM_001844	928	738	833	642
COL2A1__NM_001844_TCCAGGCCTGGACGGTGCTA	COL2A1__NM_001844	304	204	223	202
COL2A1__NM_001844_TCCTTGCATTACTCCCAACT	COL2A1__NM_001844	747	665	746	464
COL2A1__NM_001844_TGGCAGTGGCGAGGTCAGTT	COL2A1__NM_001844	52	35	59	31
COL2A1__NM_001844_TGGGCAGAGGTATAATGATA	COL2A1__NM_001844	161	197	107	100
COL2A1__NM_033150_CCTGGCCCTCAAGGTGCAAC	COL2A1__NM_033150	5	15	14	18
COL2A1__NM_033150_CTGGCTGACCATCGTTGCCT	COL2A1__NM_033150	1848	2335	2240	2700
COL2A1__NM_033150_GAGGACCAGTTGCACCTTGA	COL2A1__NM_033150	1310	1229	1335	1261
COL2A1__NM_033150_GGAGTACCAGGTTCACCGCG	COL2A1__NM_033150	792	833	751	587
COL3A1__NM_000090_ACATCATATGAATCATACTG	COL3A1__NM_000090	699	494	832	669
COL3A1__NM_000090_ACTCGCCCTCCTAATGGTCA	COL3A1__NM_000090	795	951	1043	1498
COL3A1__NM_000090_CATGATGAGCTTTGTGCAAA	COL3A1__NM_000090	652	892	874	1155
COL3A1__NM_000090_CCAAATGGAATTTCTGGGTT	COL3A1__NM_000090	743	1139	879	1107
COL3A1__NM_000090_CCAGGACCTCCTGGTGCTAT	COL3A1__NM_000090	2600	2495	2712	3423
COL3A1__NM_000090_CCCAACCCAGAAATTCCATT	COL3A1__NM_000090	1857	2496	2082	2817
COL3A1__NM_000090_CCTATGCGGATAGAGATGTC	COL3A1__NM_000090	544	685	618	714
COL3A1__NM_000090_CTGAGTCACAGACACATATT	COL3A1__NM_000090	694	553	664	622
COL3A1__NM_000090_GACATCATATGAATCATACT	COL3A1__NM_000090	661	640	757	684
COL3A1__NM_000090_GGAGGACTCGCAGGCTATCC	COL3A1__NM_000090	898	928	931	1026
COL3A1__NM_000090_TGAGGTCCTTGACCATTAGG	COL3A1__NM_000090	104	84	129	166
COL3A1__NM_000090_TGTTGTGCCAAAATAATAGT	COL3A1__NM_000090	343	457	291	416
COL4A1__NM_001845_ACATGGCCAAGTATCTCACC	COL4A1__NM_001845	1063	1004	1271	841
COL4A1__NM_001845_ACGGGATTTCCAGCGAAACC	COL4A1__NM_001845	62	251	158	125
COL4A1__NM_001845_AGGGACCACCAGGCTTACCA	COL4A1__NM_001845	371	417	482	509
COL4A1__NM_001845_CAGGGCTTCAAGGTCCTGTT	COL4A1__NM_001845	928	945	1127	1114
COL4A1__NM_001845_CCAGGATTTATAGGCGAAAT	COL4A1__NM_001845	540	607	539	753
COL4A1__NM_001845_CCCTGGCGGCCTATGAGTCC	COL4A1__NM_001845	326	211	294	255
COL4A1__NM_001845_CGGGGTTACAAGGTGTCATT	COL4A1__NM_001845	164	176	223	246
COL4A1__NM_001845_GGTGATCCAGGTGAGATACT	COL4A1__NM_001845	248	243	296	230
COL4A1__NM_001845_GGTGGTCCGGTAAATCCTGG	COL4A1__NM_001845	62	30	59	62
COL4A1__NM_001845_GTTGGGCCTCCAGGATTTAC	COL4A1__NM_001845	257	201	298	201
COL4A1__NM_001845_TCTGTGATATAGACGGATAT	COL4A1__NM_001845	388	605	532	477
COL4A1__NM_001845_TGACACCTTGTAACCCCGGG	COL4A1__NM_001845	327	332	275	542
COL4A2__NM_001846_AAGGGGAGCCCCCGGAGTAA	COL4A2__NM_001846	229	598	172	228
COL4A2__NM_001846_AGGGGAAGGCCGGGCTACGA	COL4A2__NM_001846	686	571	816	673
COL4A2__NM_001846_AGGGGAGCCCCCGGAGTAAC	COL4A2__NM_001846	187	151	156	236
COL4A2__NM_001846_CAGGGAGACTCAGGTCCACA	COL4A2__NM_001846	756	960	874	831
COL4A2__NM_001846_CAGGGGTACAATGGGCCACC	COL4A2__NM_001846	364	424	382	319
COL4A2__NM_001846_CCCGGCTCTGAGGGGTTCAC	COL4A2__NM_001846	661	567	607	632
COL4A2__NM_001846_CCGGCTCTGAGGGGTTCACC	COL4A2__NM_001846	300	163	364	248
COL4A2__NM_001846_CGATGGCTGCAACGGAACCC	COL4A2__NM_001846	582	459	571	520
COL4A2__NM_001846_CTTGGGTCCCGTTACTCCGG	COL4A2__NM_001846	537	474	536	416
COL4A2__NM_001846_GATGGCTGCAACGGAACCCA	COL4A2__NM_001846	631	655	679	544
COL4A2__NM_001846_GTGGAGGAAGAGATTGCAGT	COL4A2__NM_001846	406	315	399	256
COL4A2__NM_001846_TGGAGGAAGAGATTGCAGTG	COL4A2__NM_001846	217	58	306	52
COL4A3__NM_000091_ACCGGGCCTTACGGACTTGT	COL4A3__NM_000091	1555	1479	1737	1689
COL4A3__NM_000091_AGGCCAGTGCTTCTGTGACG	COL4A3__NM_000091	763	683	843	782
COL4A3__NM_000091_CCAGGACTCACGGGTTCCAA	COL4A3__NM_000091	374	567	411	516
COL4A3__NM_000091_CCGTAAGGCCCGGTATTGCC	COL4A3__NM_000091	208	323	196	283
COL4A3__NM_000091_CGTAAGGCCCGGTATTGCCT	COL4A3__NM_000091	1128	1085	1358	1657
COL4A3__NM_000091_CTTAGGTCCTCTAGGTCCCA	COL4A3__NM_000091	139	103	233	210
COL4A3__NM_000091_CTTGATGCAAAAGGCGACCC	COL4A3__NM_000091	286	299	317	373
COL4A3__NM_000091_GATATAGAACTTGATGCAAA	COL4A3__NM_000091	201	332	331	444
COL4A3__NM_000091_GGTTGTGTCTGTAAAGACAA	COL4A3__NM_000091	56	63	54	55
COL4A3__NM_000091_GTAAGGCCCGGTATTGCCTG	COL4A3__NM_000091	682	725	901	856
COL4A3__NM_000091_TACGGACTTGTCGGTGTACC	COL4A3__NM_000091	290	255	328	330
COL4A3__NM_000091_TGCTTCTGTGACGGGGCCAA	COL4A3__NM_000091	0	0	0	0
COL4A3BP__NM_001130105_ACCCGGGAGAGCCGGAGGTA	COL4A3BP__NM_001130105	332	345	318	314
COL4A3BP__NM_001130105_AGGATCCATCTGTCTTAGCA	COL4A3BP__NM_001130105	163	191	237	224
COL4A3BP__NM_001130105_ATCCATTGCTGTCTATGATC	COL4A3BP__NM_001130105	219	197	234	208
COL4A3BP__NM_001130105_CCACAGTTTACGTGAGAAGT	COL4A3BP__NM_001130105	587	767	800	811
COL4A3BP__NM_001130105_CCAGCTCTGATTATCCGACA	COL4A3BP__NM_001130105	1074	998	1243	1170
COL4A3BP__NM_001130105_CCATGTCGGATAATCAGAGC	COL4A3BP__NM_001130105	324	313	430	270
COL4A3BP__NM_001130105_CCGGCTCTCCCGGGTGACGA	COL4A3BP__NM_001130105	481	455	466	524
COL4A3BP__NM_001130105_CGACATTCATCAAAATCGTG	COL4A3BP__NM_001130105	414	443	519	722
COL4A3BP__NM_001130105_CGGCTCTCCCGGGTGACGAC	COL4A3BP__NM_001130105	97	106	101	147
COL4A3BP__NM_001130105_GGAAGGAGGACGAGCGGTGA	COL4A3BP__NM_001130105	471	449	644	615
COL4A3BP__NM_001130105_TCCTCGGGGTGAAGGGTCGG	COL4A3BP__NM_001130105	193	118	178	234
COL4A3BP__NM_001130105_TGGAGGCTCGACAACCGAGC	COL4A3BP__NM_001130105	38	53	43	49
COL4A3BP__NM_005713_AGAGGCACTGACTAGATCAA	COL4A3BP__NM_005713	343	372	379	326
COL4A3BP__NM_005713_ATGGAGGAAGAGCGACTATA	COL4A3BP__NM_005713	178	238	246	165
COL4A3BP__NM_005713_CTTCATTAATCAGACTGTTA	COL4A3BP__NM_005713	192	324	245	291
COL4A3BP__NM_005713_GACTTCTTGCATAGTACCAA	COL4A3BP__NM_005713	141	175	202	202
COL4A3BP__NM_005713_TACAATGAGAAAGTGTTGCA	COL4A3BP__NM_005713	340	525	359	237
COL4A3BP__NM_005713_TGAACTAATGGTTAAACGTG	COL4A3BP__NM_005713	50	30	59	48
COL4A3BP__NM_031361_CCACATGACTTACTCATTAC	COL4A3BP__NM_031361	1124	1211	1157	1032
COL4A3BP__NM_031361_CCTGTAATGAGTAAGTCATG	COL4A3BP__NM_031361	1158	1178	1290	1321
COL4A4__NM_000092_AGAGGCAAACCTGGTATGAG	COL4A4__NM_000092	314	189	277	242
COL4A4__NM_000092_ATGTGCAGAGACCACATCGC	COL4A4__NM_000092	963	712	1083	1176
COL4A4__NM_000092_CAAGGACTTGGTCAATCTGA	COL4A4__NM_000092	733	346	757	611
COL4A4__NM_000092_CCAGGGCCACAGGGTCCAAT	COL4A4__NM_000092	167	207	163	236
COL4A4__NM_000092_CCATTGTGGCCACTCATACC	COL4A4__NM_000092	97	66	78	66
COL4A4__NM_000092_CCTGGTATGAGTGGCCACAA	COL4A4__NM_000092	39	35	53	80
COL4A4__NM_000092_CTCTGCACATAGTACTAATG	COL4A4__NM_000092	834	646	903	969
COL4A4__NM_000092_GGAAATCCAGGAACACCAGT	COL4A4__NM_000092	364	249	361	426
COL4A4__NM_000092_GGGGCTCCACGGGGTCCAAT	COL4A4__NM_000092	362	385	465	532
COL4A4__NM_000092_TGGGAGCCCCAGGACCCATT	COL4A4__NM_000092	152	151	161	261
COL4A4__NM_000092_TTCTCTCCTGAAAGCCCAAT	COL4A4__NM_000092	1	5	8	7
COL4A4__NM_000092_TTTCTCTCCTGAAAGCCCAA	COL4A4__NM_000092	15	50	40	5
COL4A5__NM_000495_AATATGGGCTTAAATTTCCA	COL4A5__NM_000495	187	163	212	146
COL4A5__NM_000495_AATTGGCCCTGGTATACCAG	COL4A5__NM_000495	37	24	33	22
COL4A5__NM_000495_AGGGAATGCCAGGCCACGAT	COL4A5__NM_000495	766	1232	915	1024
COL4A5__NM_000495_AGGTGGTGGAAAGGTGAGA	COL4A5__NM_000495	34	24	54	41
COL4A5__NM_000495_ATTGGCCCTGGTATACCAGT	COL4A5__NM_000495	583	490	576	510
COL4A5__NM_000495_CCCAGGACCACCAGGTTTGA	COL4A5__NM_000495	1007	1117	1139	1114
COL4A5__NM_000495_CCTGGTCCCACTGGTATACC	COL4A5__NM_000495	825	782	958	1006
COL4A5__NM_000495_GGAGGACCACGTATCCCTGG	COL4A5__NM_000495	1069	1518	1184	1139
COL4A5__NM_000495_GGTGGTCCTGGGGGACCAAT	COL4A5__NM_000495	7	0	13	29
COL4A5__NM_000495_TCCGGGGCCTCGGGGACAAA	COL4A5__NM_000495	146	134	188	135
COL4A5__NM_000495_TTACCCTGGTGAACCCGGAA	COL4A5__NM_000495	148	142	142	185
COL4A5__NM_000495_TTCTGTTCACTGATCTGCCC	COL4A5__NM_000495	40	10	27	44
COL4A6__NM_001287758_ACTGGTTTATCGGGATTGAA	COL4A6__NM_001287758	158	446	200	200
COL4A6__NM_001287758_CCTAGCCCCATATTCCCCTA	COL4A6__NM_001287758	1020	1158	1243	1165
COL4A6__NM_001287758_CCTGGAGGGCCACTTATGCC	COL4A6__NM_001287758	57	69	116	169
COL4A6__NM_001287758_CCTTGGAATCCATTAAGCCC	COL4A6__NM_001287758	723	768	842	745
COL4A6__NM_001287758_CGGTCAGGCACAACGTAACC	COL4A6__NM_001287758	368	474	440	517
COL4A6__NM_001287758_CTGCTGCCAGTTCCTCGGTC	COL4A6__NM_001287758	1705	2066	1921	1811
COL4A6__NM_001287758_CTTGCTCCAGGTAGTTTCAA	COL4A6__NM_001287758	335	275	364	381
COL4A6__NM_001287758_GGACACCAGAAGGGCCACGT	COL4A6__NM_001287758	2864	3328	3322	3269
COL4A6__NM_001287758_GTTGGGCCTTGAATTCCAAT	COL4A6__NM_001287758	567	940	797	1085
COL4A6__NM_001287758_GTTGTGCCTGACCGAGGAAC	COL4A6__NM_001287758	336	319	451	328
COL4A6__NM_001287758_TCTTATGGAAAGCCATGTGG	COL4A6__NM_001287758	92	50	140	100
COL4A6__NM_001287758_TGGACTCCTTCTGAACCCAT	COL4A6__NM_001287758	155	163	186	147
COL5A1__NM_000093_ATCAATGGCATCATCGTGTT	COL5A1__NM_000093	1241	1571	1236	1542
COL5A1__NM_000093_ATGCCATTGATGTCGATCAT	COL5A1__NM_000093	496	591	584	707
COL5A1__NM_000093_CGGGGCATCAACCTGTCAGA	COL5A1__NM_000093	1399	676	1365	838
COL5A1__NM_000093_CTTCGGGGTCTTCGTAGTAG	COL5A1__NM_000093	458	524	452	436
COL5A1__NM_000093_GATGCCATTGATGTCGATCA	COL5A1__NM_000093	1019	1461	1177	1437
COL5A1__NM_000093_GCCACGCGGCGATCTTCCAA	COL5A1__NM_000093	2454	1537	2848	2218
COL5A1__NM_000093_GCGGCGATCTTCCAAAGGCC	COL5A1__NM_000093	453	229	522	440
COL5A1__NM_000093_GTAGGGGTATTCGTAGTAAT	COL5A1__NM_000093	283	235	253	271
COL5A1__NM_000093_GTCGCTGCGGTCGAGGAATT	COL5A1__NM_000093	830	632	790	825
COL5A1__NM_000093_GTCTCCATCTACAACGAGCA	COL5A1__NM_000093	244	454	220	297
COL5A1__NM_000093_TCTTGGCTTTCACAGTTGTT	COL5A1__NM_000093	582	607	691	784
COL5A1__NM_000093_TGACTCTGTAAGCGACATCC	COL5A1__NM_000093	148	88	177	153
COL5A2__NM_000393_AAAGGTCTTGAAGGCCCTAA	COL5A2__NM_000393	57	63	67	29
COL5A2__NM_000393_ACCTTCTATCATGTATGTAC	COL5A2__NM_000393	732	672	749	753
COL5A2__NM_000393_AGAGGAGAGCGAGGGCCAAA	COL5A2__NM_000393	208	257	225	160
COL5A2__NM_000393_AGCAAAATAAACATGTGTTG	COL5A2__NM_000393	311	312	398	587
COL5A2__NM_000393_ATGGCACCCATTGGACCAGT	COL5A2__NM_000393	742	781	800	727
COL5A2__NM_000393_ATTCCCAGGGGGCGTTACA	COL5A2__NM_000393	308	313	225	256
COL5A2__NM_000393_CATGACCAAAGTTGTATGCA	COL5A2__NM_000393	620	666	660	676
COL5A2__NM_000393_CCCAGGGGGCGTTACAGGGT	COL5A2__NM_000393	94	75	148	140
COL5A2__NM_000393_CTGTGACAAGATAGAATGCC	COL5A2__NM_000393	247	318	327	382
COL5A2__NM_000393_GGATGATGAGATTGTACATG	COL5A2__NM_000393	539	550	531	499
COL5A2__NM_000393_GGTCAAATATAACCAGTTT	COL5A2__NM_000393	1355	1605	1432	1623
COL5A2__NM_000393_TAGGGACAACGAGGTGCACA	COL5A2__NM_000393	59	155	81	75
COL5A2__NM_000393_TAGGGTCCGATTGGTTCACG	COL5A2__NM_000393	445	386	444	445
COL5A2__NM_000393_TAGGGTCCTCTTGGGATACC	COL5A2__NM_000393	149	134	163	125
COL5A2__NM_000393_TAGGGTGGTGCAGGACCTAC	COL5A2__NM_000393	104	19	137	82
COL5A2__NM_000393_TCTGGTCCACGTGAACCAAT	COL5A2__NM_000393	455	264	483	468
COL5A2__NM_000393_TGCTCATTGTCGATGTGTCT	COL5A2__NM_000393	346	632	413	484
COL5A2__NM_000393_TGTACTTAAACAGGGACATT	COL5A2__NM_000393	33	18	26	37
COL5A3__NM_015719_AAGAAAGGGCGAGGTCGCAA	COL5A3__NM_015719	420	439	473	446
COL5A3__NM_015719_ACCCCTCGTCCTCGGCGGAA	COL5A3__NM_015719	849	803	871	870
COL5A3__NM_015719_AGAAAGGGCGAGGTCGCAAG	COL5A3__NM_015719	249	258	174	143
COL5A3__NM_015719_CATAGCTGGACTCACTGTGC	COL5A3__NM_015719	324	223	367	267
COL5A3__NM_015719_CCTGGAAAGAGTTCCCACGT	COL5A3__NM_015719	1683	891	1869	919
COL5A3__NM_015719_CCTTGGGGAAAAGACTTTCG	COL5A3__NM_015719	332	260	406	259
COL5A3__NM_015719_CGTGTGGCCGTCAGCATAGA	COL5A3__NM_015719	0	0	0	0
COL5A3__NM_015719_GAAGAAAGGGCGAGGTCGCA	COL5A3__NM_015719	428	531	543	557
COL5A3__NM_015719_GGAGGTACCGCTCACAAGCC	COL5A3__NM_015719	438	328	443	595
COL5A3__NM_015719_GGCACCCCTTTCATCATAAA	COL5A3__NM_015719	554	645	660	941
COL5A3__NM_015719_GGGGAAAAGACTTTCGAGGT	COL5A3__NM_015719	388	355	301	468
COL5A3__NM_015719_TGCTGTCCATTTATGATGAA	COL5A3__NM_015719	108	237	157	150
COL6A1__NM_001848_ACCGACTGCGCTATCAAGAA	COL6A1__NM_001848	380	502	556	746
COL6A1__NM_001848_CACCGACTGCGCTATCAAGA	COL6A1__NM_001848	876	809	1010	771
COL6A1__NM_001848_CACGGGCCGCCCTCATGTCT	COL6A1__NM_001848	4136	5162	4776	4706
COL6A1__NM_001848_CACGTACCGGCGCAACTTCA	COL6A1__NM_001848	1546	1636	1618	1880
COL6A1__NM_001848_CCATCGTGGACATGATCGTG	COL6A1__NM_001848	880	936	982	1015
COL6A1__NM_001848_CCGACTGCGCTATCAAGAAG	COL6A1__NM_001848	430	654	585	767
COL6A1__NM_001848_CCGGGGACCTCGGACCTGTT	COL6A1__NM_001848	1070	1408	1318	1683
COL6A1__NM_001848_CTACCGCTGTGACCGAAACC	COL6A1__NM_001848	465	348	549	712
COL6A1__NM_001848_GGCTGGAGCAGCTCCTCGTG	COL6A1__NM_001848	1359	1504	1656	1996
COL6A1__NM_001848_GGGCGTCAAAGTCTTCTCGG	COL6A1__NM_001848	1270	1353	1589	1869
COL6A1__NM_001848_GTGGCGATGATGCTCAGACG	COL6A1__NM_001848	313	688	380	340
COL6A1__NM_001848_TTGTCCACGAGGGCCCCGTA	COL6A1__NM_001848	268	307	302	428
COL6A2__NM_001849_AGCACGAAGTACACGTGGAT	COL6A2__NM_001849	1021	971	1091	1144
COL6A2__NM_001849_CCAACATGACGGAGCAGATC	COL6A2__NM_001849	558	707	607	827
COL6A2__NM_001849_CCGGATCTGCTCCGTCATGT	COL6A2__NM_001849	2964	3189	3147	3264
COL6A2__NM_001849_GAGAGAAACAACAACTGCCC	COL6A2__NM_001849	301	274	353	437
COL6A2__NM_001849_GCACGAAGTACACGTGGATG	COL6A2__NM_001849	1101	1260	1231	1373
COL6A2__NM_001849_GCATGGTGGCGTAGTCGTTG	COL6A2__NM_001849	1008	993	1106	1341
COL6A2__NM_001849_GGTGGAGGTGTTCAGCCCAC	COL6A2__NM_001849	379	489	397	376
COL6A2__NM_001849_GTGGCGCTGAGCTGGCGCTA	COL6A2__NM_001849	206	185	315	316
COL6A2__NM_001849_TCGTTGCGGTAGAGCTCGTG	COL6A2__NM_001849	388	313	345	447
COL6A2__NM_001849_TGCAGCTTGATGCCCCCGCA	COL6A2__NM_001849	558	728	637	988
COL6A2__NM_001849_TGTCCAGCACGAAGTACACG	COL6A2__NM_001849	441	581	413	451
COL6A2__NM_001849_TTGTTTCTCTCGGTAGTGTC	COL6A2__NM_001849	38	45	39	6
COL6A3__NM_004369_ACATCGGCACTTGCCCTTAG	COL6A3__NM_004369	848	910	878	826
COL6A3__NM_004369_ACATGTTGGAAATATGAGAA	COL6A3__NM_004369	160	96	179	141
COL6A3__NM_004369_AGGCAAAAGACGGCCACTAA	COL6A3__NM_004369	173	203	207	143
COL6A3__NM_004369_ATACGTATTTAACAGGAACT	COL6A3__NM_004369	128	103	171	128
COL6A3__NM_004369_ATAGAAACTCTCGAACAAGT	COL6A3__NM_004369	187	308	249	286
COL6A3__NM_004369_GTGGATTCCTCTTGGACCAT	COL6A3__NM_004369	33	53	30	28
COL6A3__NM_004369_GTTGTAAAATCCTTAGCTGT	COL6A3__NM_004369	183	373	231	266
COL6A3__NM_004369_TAGTACGATACGTATTTAAC	COL6A3__NM_004369	148	172	142	138
COL6A3__NM_004369_TATGTGGGTTTCCGTTGAAC	COL6A3__NM_004369	409	369	419	417
COL6A3__NM_004369_TGCTGCTGGGCATGAGTTGT	COL6A3__NM_004369	302	315	364	514
COL6A3__NM_004369_TTAACAGGAACTCGGTATGT	COL6A3__NM_004369	379	313	518	414
COL6A3__NM_004369_TTCCTCTTGGACCATTGGAG	COL6A3__NM_004369	21	0	52	26
COL6A3__NM_057164_AGGCTGGCTGATTTCATCTA	COL6A3__NM_057164	858	598	832	913
COL6A3__NM_057164_CACGAGTTCAGCCGGCTACC	COL6A3__NM_057164	1099	1167	1206	1400
COL6A3__NM_057164_CCAGAGGCTGGAAATCGGAC	COL6A3__NM_057164	638	853	717	823
COL6A3__NM_057164_GGGAAGTCATAACCGCTGTG	COL6A3__NM_057164	501	728	480	729
COL6A3__NM_057164_GTCATCCAGAGGCTGGAAAT	COL6A3__NM_057164	469	429	505	644
COL6A3__NM_057164_GTGGATGGCTCATCTGCACT	COL6A3__NM_057164	256	218	251	276
COL6A3__NM_057164_TAGAGCAGAGCCCGTGTACA	COL6A3__NM_057164	453	632	511	715
COL6A3__NM_057164_TCAATACCCATCCAACAAAA	COL6A3__NM_057164	104	141	104	114
COL6A3__NM_057164_TGGGAACAAGGGTGCCGATC	COL6A3__NM_057164	16	31	22	1
COL6A3__NM_057165_ATCAAGGGCGAGGCCGATAT	COL6A3__NM_057165	707	812	787	639
COL6A3__NM_057165_CCACCACGAAATCAAGGGCG	COL6A3__NM_057165	2195	2603	2499	3064
COL6A3__NM_057165_CCTCGCCCTTGATTTCGTGG	COL6A3__NM_057165	1914	1637	2313	2423
COL6A3__NM_057165_CGGATCTGCTGAGTTCCAAT	COL6A3__NM_057165	502	1006	641	887
COL6A3__NM_057165_CGGCCTCGCCCTTGATTTCG	COL6A3__NM_057165	736	946	871	1181
COL6A3__NM_057165_CTCCTTGAGAAACTCCCAAT	COL6A3__NM_057165	577	773	596	674
COL6A3__NM_057165_GGATCTGCTGAGTTCCAATT	COL6A3__NM_057165	626	573	718	788
COL6A3__NM_057165_TGTTGTTTGATCCATCAATA	COL6A3__NM_057165	62	162	88	111
COL6A3__NM_057165_TTCTGGGCTCATCGCTAAAC	COL6A3__NM_057165	14	74	12	7
COL6A5__NM_001278298_AAGAAGAACTTTCAGTTCAT	COL6A5__NM_001278298	203	268	246	339
COL6A5__NM_001278298_AATCATTTGGACTGAAACAT	COL6A5__NM_001278298	214	181	348	416
COL6A5__NM_001278298_ACTTCTCTGCACCCATAAAT	COL6A5__NM_001278298	968	872	842	778
COL6A5__NM_001278298_ATGTTTCAGTCCAAATGATT	COL6A5__NM_001278298	190	199	189	227
COL6A5__NM_001278298_CACCCATAAATGGGAGAGAC	COL6A5__NM_001278298	444	634	477	555
COL6A5__NM_001278298_CGGGGTGAAAATTATCTCCG	COL6A5__NM_001278298	834	1336	1115	874
COL6A5__NM_001278298_GCCAGGACCACCAAAATTGG	COL6A5__NM_001278298	721	694	766	580
COL6A5__NM_001278298_GGGGTGAAAATTATCTCCGT	COL6A5__NM_001278298	707	701	667	770
COL6A5__NM_001278298_GGGTGAAAATTATCTCCGTG	COL6A5__NM_001278298	636	1056	693	831
COL6A5__NM_001278298_TACTTCTCTGCACCCATAAA	COL6A5__NM_001278298	229	126	253	330
COL6A5__NM_001278298_TTCACGAATGGGAATGACTT	COL6A5__NM_001278298	63	165	94	172
COL6A5__NM_001278298_TTCCTGTCTCTCCCATTTAT	COL6A5__NM_001278298	40	59	34	12
COL6A6__NM_001102608_AATCTTGGTTCACAGAAATA	COL6A6__NM_001102608	79	69	96	103
COL6A6__NM_001102608_ACGGTATTTGTCGGCCTCTA	COL6A6__NM_001102608	547	446	592	756
COL6A6__NM_001102608_AGAACTTTGGATTCATTGGC	COL6A6__NM_001102608	132	164	204	317
COL6A6__NM_001102608_AGGAAGAACTTTGGATTCAT	COL6A6__NM_001102608	817	723	858	886
COL6A6__NM_001102608_ATTTCTCTGCACCCGCAAAT	COL6A6__NM_001102608	849	1299	1057	1197
COL6A6__NM_001102608_ATTTCTGTGAACCAAGATTC	COL6A6__NM_001102608	119	69	117	101
COL6A6__NM_001102608_CGGTATTTGTCGGCCTCTAT	COL6A6__NM_001102608	345	532	469	593
COL6A6__NM_001102608_GTGAAGTTTATCACTGTACT	COL6A6__NM_001102608	224	320	245	398
COL6A6__NM_001102608_GTGGACAGCTCTGATCGCCT	COL6A6__NM_001102608	168	170	197	147
COL6A6__NM_001102608_TATGGGAACAAATTATCACG	COL6A6__NM_001102608	321	333	385	403
COL6A6__NM_001102608_TCCCCCAATTCTAGTGGTCC	COL6A6__NM_001102608	374	370	442	426
COL6A6__NM_001102608_TGTGAAGTTTATCACTGTAC	COL6A6__NM_001102608	443	393	577	681
COL7A1__NM_000094_AAGAAGAAGAAGTCACTGGT	COL7A1__NM_000094	719	435	583	532
COL7A1__NM_000094_AAGGGGGGCAACACTCGCAC	COL7A1__NM_000094	754	537	761	688
COL7A1__NM_000094_AGGTCCGCAGCTTTCTCGAA	COL7A1__NM_000094	973	667	1097	936
COL7A1__NM_000094_CAACAGGACAGAGTTCGGCC	COL7A1__NM_000094	422	246	540	358
COL7A1__NM_000094_CAGTAAGAACACAATGTCAG	COL7A1__NM_000094	341	232	313	243
COL7A1__NM_000094_CATCCGTGAGCTTAGCTACA	COL7A1__NM_000094	271	117	221	100
COL7A1__NM_000094_CCGTGAGCTTAGCTACAAGG	COL7A1__NM_000094	220	70	205	137
COL7A1__NM_000094_CGGAGAGTGTGCACGACTGC	COL7A1__NM_000094	608	665	735	873
COL7A1__NM_000094_CTTGTAGCTAAGCTCACGGA	COL7A1__NM_000094	260	99	322	168
COL7A1__NM_000094_TCCGTGAGCTTAGCTACAAG	COL7A1__NM_000094	112	39	130	109
COL7A1__NM_000094_TGCACACTCTCCGGGAAACG	COL7A1__NM_000094	247	279	365	210
COL7A1__NM_000094_TGGCCGCAGCAATTTCCGCG	COL7A1__NM_000094	228	181	304	365
COL8A1__NM_001850_ACCATTAGCCAGTTTACGAG	COL8A1__NM_001850	1036	922	1067	742
COL8A1__NM_001850_ACCTTGTTCCCCTCGTAAAC	COL8A1__NM_001850	466	630	472	628
COL8A1__NM_001850_ACTCTTTGCCATACTGCAAG	COL8A1__NM_001850	391	449	365	474
COL8A1__NM_001850_AGCCTGTAATTGCGAATGAT	COL8A1__NM_001850	719	841	793	798
COL8A1__NM_001850_ATACCATTAGCCAGTTTACG	COL8A1__NM_001850	898	951	1036	1098
COL8A1__NM_001850_ATCCCATCATTCGCAATTAC	COL8A1__NM_001850	778	961	876	1052
COL8A1__NM_001850_ATTCCAGGTATCCCATGACC	COL8A1__NM_001850	422	294	440	385
COL8A1__NM_001850_CACTGGGGCGGTAACACTAC	COL8A1__NM_001850	363	344	381	341
COL8A1__NM_001850_CAGCCTGTAATTGCGAATGA	COL8A1__NM_001850	1842	2293	1942	2392
COL8A1__NM_001850_CAGCGGCTTGATCCCATAGT	COL8A1__NM_001850	1193	1119	1309	1233
COL8A1__NM_001850_CCACTGGGGCGGTAACACTA	COL8A1__NM_001850	520	978	587	612
COL8A1__NM_001850_CCTGGTAATGCTTTAGTCAA	COL8A1__NM_001850	475	930	603	642
COL8A1__NM_001850_CTCTTTGCCATACTGCAAGT	COL8A1__NM_001850	439	394	475	474
COL8A1__NM_001850_GAGATGCCCCACTTGCAGTA	COL8A1__NM_001850	679	1032	900	896
COL8A1__NM_001850_GCCAGTTTACGAGGGGAACA	COL8A1__NM_001850	554	885	501	749
COL8A1__NM_001850_TACCATTAGCCAGTTTACGA	COL8A1__NM_001850	933	717	833	815
COL8A1__NM_001850_TCTGAATAAGCAGGTTACTG	COL8A1__NM_001850	221	193	196	180
COL8A1__NM_001850_TGACCCAGGGGCTGGTATTG	COL8A1__NM_001850	490	592	609	739
COL8A1__NM_001850_TTTCCTTCATATATTGGGGT	COL8A1__NM_001850	65	96	71	97
COL8A1__NM_001850_TTTGACATGGAATATATGGT	COL8A1__NM_001850	181	172	218	204
COL8A2__NM_005202_AAAGGTCGCTCTACCACTAA	COL8A2__NM_005202	549	291	672	493
COL8A2__NM_005202_AACCTTCTCAAGTGTCACTT	COL8A2__NM_005202	387	259	465	283
COL8A2__NM_005202_AAGGACTCTCAAACCATACC	COL8A2__NM_005202	127	109	163	180
COL8A2__NM_005202_AAGGCAGAGCACCTCTCGTT	COL8A2__NM_005202	1279	1153	1500	1075
COL8A2__NM_005202_AAGGCAGTTTGTGAGGGCTT	COL8A2__NM_005202	589	490	663	447
COL8A2__NM_005202_AAGGTTTGAGGAATGAAATT	COL8A2__NM_005202	56	26	49	27
COL8A2__NM_005202_ACCCCAGCCAGGTATCCCCA	COL8A2__NM_005202	922	763	900	627
COL8A2__NM_005202_ACGCCCAAGAAAACCAACTA	COL8A2__NM_005202	237	252	268	194
COL8A2__NM_005202_ACTCTCAAACCATACCTGGT	COL8A2__NM_005202	697	456	902	436
COL8A2__NM_005202_AGCTCTGGCTGTTTAGCTTG	COL8A2__NM_005202	460	763	591	733
COL8A2__NM_005202_AGTCCTGGCTTTCCCATGCC	COL8A2__NM_005202	1210	1059	1341	1226
COL8A2__NM_005202_ATCAAAGCTAGTCAGTTAAG	COL8A2__NM_005202	321	278	505	471
COL8A2__NM_005202_ATGATAGTGGCTTTGACCTA	COL8A2__NM_005202	417	386	495	411
COL8A2__NM_005202_ATGGGCTGGATGTACTTCAC	COL8A2__NM_005202	597	494	673	603
COL8A2__NM_005202_ATGTGTCAATACAGTTAACA	COL8A2__NM_005202	178	91	187	170
COL8A2__NM_005202_CAGGGATAGTAATGCCTGAG	COL8A2__NM_005202	551	330	529	537
COL8A2__NM_005202_CCAAATGTCTGTGCAGTGTC	COL8A2__NM_005202	1077	1245	1327	1955
COL8A2__NM_005202_CCACCATATGGTTCTTATTA	COL8A2__NM_005202	303	379	374	442
COL8A2__NM_005202_CCAGGAATACGAGGGGACCA	COL8A2__NM_005202	562	579	583	462
COL8A2__NM_005202_CCCCCTGGCTTCTCCCGGAT	COL8A2__NM_005202	2928	2375	3534	2993
COL8A2__NM_005202_CCTGGCAAGGTCGGGCCACC	COL8A2__NM_005202	296	303	313	294
COL8A2__NM_005202_CCTGGTGGCCCGACCTTGCC	COL8A2__NM_005202	327	495	390	373
COL8A2__NM_005202_CCTTTGCCCTCACGGAAGGG	COL8A2__NM_005202	395	624	492	567
COL8A2__NM_005202_CGGGGGGAGCCAGGAATACG	COL8A2__NM_005202	241	199	287	292
COL8A2__NM_005202_CTAGAAGTATCTAGTGCAGA	COL8A2__NM_005202	384	254	362	276
COL8A2__NM_005202_CTGGTGGCCCGACCTTGCCA	COL8A2__NM_005202	856	725	935	771
COL8A2__NM_005202_CTGTGTGCCTCAGGGCCTAT	COL8A2__NM_005202	574	609	679	594
COL8A2__NM_005202_GCCCAGCCCCTTGTGAGTCC	COL8A2__NM_005202	565	487	651	641
COL8A2__NM_005202_GCCCTAGTCCTGCTTAGAAC	COL8A2__NM_005202	727	860	643	740
COL8A2__NM_005202_GGAGCTCTGATCGGGGAACA	COL8A2__NM_005202	323	195	322	213
COL8A2__NM_005202_GGGAGCCCACGGAACTAAGT	COL8A2__NM_005202	563	349	569	397
COL8A2__NM_005202_GGGGGGAGCCAGGAATACGA	COL8A2__NM_005202	202	168	183	230
COL8A2__NM_005202_TATCCACTCCGCTTGACGTA	COL8A2__NM_005202	248	197	378	285
COL8A2__NM_005202_TCACGGAAGGGCGGTCCCAC	COL8A2__NM_005202	1440	1723	1656	1439
COL8A2__NM_005202_TCTGGCCTGTAGGTGTCCCA	COL8A2__NM_005202	450	261	573	272
COL8A2__NM_005202_TGAACAAGGTGCACGCGAGC	COL8A2__NM_005202	2	0	1	4
COL8A2__NM_005202_TGCCTTGGGGATACCTGGCT	COL8A2__NM_005202	746	635	888	873
COL8A2__NM_005202_TGGGACCCTACTTAGTTCCG	COL8A2__NM_005202	180	175	115	78
COL8A2__NM_005202_TGGGTGTATGTGTGTACAAG	COL8A2__NM_005202	139	252	147	131
COL8A2__NM_005202_TGTTCAGAGGGCCAAACGAG	COL8A2__NM_005202	104	96	98	126
COL8A2__NM_005202_TTATCCACTCCGCTTGACGT	COL8A2__NM_005202	1597	875	1598	979
COL9A1__NM_001851_AATCATGATCTTATGCCACT	COL9A1__NM_001851	1043	1043	1223	1381
COL9A1__NM_001851_ATACATTGTAATCATGCTGA	COL9A1__NM_001851	383	240	409	381
COL9A1__NM_001851_ATACCTATGTGTCTTATCGT	COL9A1__NM_001851	1490	1140	1573	1196
COL9A1__NM_001851_ATTGCAGGTGGCTTACAAGT	COL9A1__NM_001851	682	673	724	711
COL9A1__NM_001851_CAAGTTGGCATAAAGATTAA	COL9A1__NM_001851	64	53	100	111
COL9A1__NM_001851_CATTAGAATTGGAATTGACA	COL9A1__NM_001851	352	403	409	383
COL9A1__NM_001851_CATTCGAAACGTCGTCAAGA	COL9A1__NM_001851	472	628	528	811
COL9A1__NM_001851_CCATTAGAATTGGAATTGAC	COL9A1__NM_001851	999	1424	1212	1677
COL9A1__NM_001851_CCCACTACATTCAATGGAAT	COL9A1__NM_001851	416	362	645	735
COL9A1__NM_001851_CTCTGCTGTCTTCCCTCCAA	COL9A1__NM_001851	683	284	688	413
COL9A1__NM_001851_CTGTCTGCATGAACCACGCC	COL9A1__NM_001851	390	255	448	556
COL9A1__NM_001851_GACAATCACATATAGAAGAA	COL9A1__NM_001851	101	124	152	134
COL9A1__NM_001851_GCATGAACCACGCCTGGTGA	COL9A1__NM_001851	1219	1394	1221	1562
COL9A1__NM_001851_GCTCAAAATATTAACAGGTT	COL9A1__NM_001851	480	824	570	718
COL9A1__NM_001851_TAGACTTCAGGATTCCAACT	COL9A1__NM_001851	183	335	263	271
COL9A1__NM_001851_TATACCTATGTGTCTTATCG	COL9A1__NM_001851	392	342	494	367
COL9A1__NM_001851_TATTCTTCAGGCAGTCCACT	COL9A1__NM_001851	235	317	290	273
COL9A1__NM_001851_TCAAAAAGAACTGGAACATT	COL9A1__NM_001851	535	791	754	990
COL9A1__NM_001851_TGTCAAGCGTCGCCCCAGTA	COL9A1__NM_001851	707	422	661	553
COL9A1__NM_001851_TTGACGACGTTTCGAATGAC	COL9A1__NM_001851	96	71	116	145
COL9A1__NM_001851_TTGCAGGTGGCTTACAAGTT	COL9A1__NM_001851	209	140	260	310
COL9A1__NM_078485_ACCGGGTCCAAAGGGTGAAC	COL9A1__NM_078485	52	71	76	68
COL9A1__NM_078485_AGAGGAGAATTAGGACCAGT	COL9A1__NM_078485	463	332	589	435
COL9A1__NM_078485_AGGCATCACCGGCATAGTTG	COL9A1__NM_078485	179	145	197	172
COL9A1__NM_078485_ATCCAGGCCTACCAGGCATG	COL9A1__NM_078485	64	118	98	74
COL9A1__NM_078485_CAGGGTGACCGAGGTCCTAA	COL9A1__NM_078485	369	272	405	262
COL9A1__NM_078485_CCCAGGCCTACCAGGTAAAC	COL9A1__NM_078485	104	176	132	132
COL9A1__NM_078485_CCTGGATATCCTGAGCGACC	COL9A1__NM_078485	1117	1181	1371	1321
COL9A1__NM_078485_CGAGGCATCACCGGCATAGT	COL9A1__NM_078485	361	408	423	357
COL9A1__NM_078485_CGGTGATGCCTCGCAAACCC	COL9A1__NM_078485	442	225	246	277
COL9A1__NM_078485_GAGGCATCACCGGCATAGTT	COL9A1__NM_078485	245	306	312	297
COL9A1__NM_078485_GTCGGTGAACCGGGTCCAAA	COL9A1__NM_078485	360	546	359	364
COL9A1__NM_078485_TAGGGAGATATAGGATTACC	COL9A1__NM_078485	251	108	241	145
COL9A2__NM_001852_AAAGGCATGGTGGGCGCTAT	COL9A2__NM_001852	351	217	266	284
COL9A2__NM_001852_AAGGGGAGGTAGAGCACTGA	COL9A2__NM_001852	290	238	386	227
COL9A2__NM_001852_ACCATGCCTTTATATCCATG	COL9A2__NM_001852	909	1121	1218	1098
COL9A2__NM_001852_ATAGAGTAACTCCAATATCC	COL9A2__NM_001852	177	278	173	178
COL9A2__NM_001852_CAGGGCCCTCATGGATATAA	COL9A2__NM_001852	301	246	286	233
COL9A2__NM_001852_CAGGGGCATGCGGGCAAACG	COL9A2__NM_001852	648	795	687	784
COL9A2__NM_001852_CATGGATATAAAGGCATGGT	COL9A2__NM_001852	625	456	683	587
COL9A2__NM_001852_CCCAGCAGCAGTCACCTGGT	COL9A2__NM_001852	4069	3540	3798	3710
COL9A2__NM_001852_CCCTGGACCAGATGGACCAT	COL9A2__NM_001852	1331	1190	1257	1117
COL9A2__NM_001852_CCGGGAGTGCCTGGATCCGA	COL9A2__NM_001852	1047	634	1033	597
COL9A2__NM_001852_CCTGGACCAGATGGACCATC	COL9A2__NM_001852	612	660	689	535
COL9A2__NM_001852_CCTGGGGTCCACGAATACCT	COL9A2__NM_001852	476	464	517	501
COL9A2__NM_001852_CCTGGTGAGATTGGAATCCG	COL9A2__NM_001852	939	876	972	934
COL9A2__NM_001852_GAAGGTCCTAGGGGACCGCC	COL9A2__NM_001852	29	5	17	35
COL9A2__NM_001852_GCATCCCTGGACCACCGGTA	COL9A2__NM_001852	1411	1709	1756	2086
COL9A2__NM_001852_TCCCAAGTTGGGTACAAACC	COL9A2__NM_001852	47	7	78	65
COL9A2__NM_001852_TCCTTCCAGCGAGCATCATT	COL9A2__NM_001852	124	110	193	180
COL9A2__NM_001852_TTAAGGGTATAGCCGCCTCA	COL9A2__NM_001852	219	122	165	154
COL9A3__NM_001853_AAAGCCGGGGAAACCAGGAG	COL9A3__NM_001853	108	38	85	65
COL9A3__NM_001853_AGGCGGCCCTCGGAACCCAA	COL9A3__NM_001853	335	185	363	296
COL9A3__NM_001853_CAGGGCCCCCGGGGATTACG	COL9A3__NM_001853	372	403	383	494
COL9A3__NM_001853_CAGGGGGAAGTCGGCAAGGA	COL9A3__NM_001853	243	248	257	141
COL9A3__NM_001853_CCGGGCAAGGACGGCCAGAA	COL9A3__NM_001853	4	6	15	11
COL9A3__NM_001853_CGAGTCCTGGCACGCCATTC	COL9A3__NM_001853	676	915	820	736
COL9A3__NM_001853_GAAGGTCAGTAGCGCCTTCA	COL9A3__NM_001853	340	360	322	444
COL9A3__NM_001853_GACGGCCAGAATGGCGTGCC	COL9A3__NM_001853	199	378	167	255
COL9A3__NM_001853_GCAGTCCTCGTAATCCCCGG	COL9A3__NM_001853	1272	997	1461	1394
COL9A3__NM_001853_GGCAGTCCTCGTAATCCCCG	COL9A3__NM_001853	1174	989	1208	1289
COL9A3__NM_001853_GGCGGCCCTCGGAACCCAAT	COL9A3__NM_001853	964	1067	1129	947
COL9A3__NM_001853_TCCAGGGTGACCGAGGCGAG	COL9A3__NM_001853	245	239	319	321
COLCA2__NM_001136105_AGGACAAGTGTTGGTAGGAA	COLCA2__NM_001136105	255	367	394	507
COLCA2__NM_001136105_CACCAGTGATTTCTATAAGA	COLCA2__NM_001136105	241	136	242	291
COLCA2__NM_001136105_CCACCAGTGATTTCTATAAG	COLCA2__NM_001136105	420	457	545	400
COLCA2__NM_001136105_CGATGCGCAGTAGCAGATGG	COLCA2__NM_001136105	506	437	520	467
COLCA2__NM_001136105_CTGCTACTGCGCATCGTGTG	COLCA2__NM_001136105	400	272	425	618
COLCA2__NM_001136105_GTCTTCTGGGGGGTAGCCAC	COLCA2__NM_001136105	107	56	120	109
COLCA2__NM_001271458_ACGCCCAATTATTTGCAACG	COLCA2__NM_001271458	928	1043	1093	1378
COLCA2__NM_001271458_AGGAATCAAAGATCTGGTCG	COLCA2__NM_001271458	305	180	396	324
COLCA2__NM_001271458_CGTTGCAAATAATTGGGCG	COLCA2__NM_001271458	1050	1062	1056	1620
COLCA2__NM_001271458_TTCTCCCCGTTGCAAATAAT	COLCA2__NM_001271458	14	0	23	26
COLEC10__NM_006438_AAATTGGTTTCTTCGAAGCA	COLEC10__NM_006438	245	263	223	302
COLEC10__NM_006438_AACTGGATATTAGTATTGCT	COLEC10__NM_006438	283	379	341	376
COLEC10__NM_006438_ACTGGGTGATATGGGAGATC	COLEC10__NM_006438	458	314	509	383
COLEC10__NM_006438_ATAGTACCAGGAGGATAAAT	COLEC10__NM_006438	250	186	252	196
COLEC10__NM_006438_ATGGCAAAGTGGGACGCATG	COLEC10__NM_006438	252	134	324	364
COLEC10__NM_006438_CATGGCAAAGTGGGACGCAT	COLEC10__NM_006438	801	594	937	1222
COLEC10__NM_006438_CTCATGACCATAGGGGTCGC	COLEC10__NM_006438	240	341	368	416
COLEC10__NM_006438_GGGGAACCCAGCGACCCCTA	COLEC10__NM_006438	495	416	499	617
COLEC10__NM_006438_TAAAGGAGAACTGGGTGATA	COLEC10__NM_006438	200	217	255	334
COLEC10__NM_006438_TCATGACCATAGGGGTCGCT	COLEC10__NM_006438	733	765	788	1178
COLEC10__NM_006438_TCTGTGATTGTGGAAGATAC	COLEC10__NM_006438	161	288	157	205
COLEC10__NM_006438_TTTGCAAAAGAAATAGTACC	COLEC10__NM_006438	140	265	154	148
COLEC11__NM_001255982_AAAGGAGATTCCGGTGACAT	COLEC11__NM_001255982	292	309	333	330
COLEC11__NM_001255982_AAAGGCAGTGTGGGTCGTCA	COLEC11__NM_001255982	483	394	661	847
COLEC11__NM_001255982_ATTGGTCCCATTGGCTCTAA	COLEC11__NM_001255982	192	130	171	244
COLEC11__NM_001255982_GCAGCTGGCTGCACTCACAT	COLEC11__NM_001255982	1507	2069	1542	1705
COLEC11__NM_001255982_GCCGCATACCTGGCGCAAGC	COLEC11__NM_001255982	319	380	304	305
COLEC11__NM_001255982_GCCGGCTTGCGCCAGGTATG	COLEC11__NM_001255982	60	110	69	138
COLEC11__NM_001255982_GCGCCAGGTATGCGGCCATC	COLEC11__NM_001255982	482	521	441	807
COLEC11__NM_001255982_GCTGGCTGCACTCACATGGG	COLEC11__NM_001255982	1018	536	1089	719
COLEC11__NM_001255982_GGAGTGGTCAGAGTACACGA	COLEC11__NM_001255982	372	550	460	466
COLEC11__NM_001255982_GGCCTGGCCCGTGTCTTCAT	COLEC11__NM_001255982	2748	2274	3163	2898
COLEC11__NM_001255982_GTATGCGGCCATCAGGCCAT	COLEC11__NM_001255982	104	127	112	96
COLEC11__NM_001255982_TGGCCTGATGGCCGCATACC	COLEC11__NM_001255982	43	68	73	53
COLEC11__NM_001255983_ACTTGTTGAAGGTCCGCATG	COLEC11__NM_001255983	304	264	379	398
COLEC11__NM_001255983_CACTTGTTGAAGGTCCGCAT	COLEC11__NM_001255983	966	984	1056	1045
COLEC11__NM_001255986_GCGCAGGGAAGACAACCGTA	COLEC11__NM_001255986	694	378	696	576
COLEC11__NM_001255986_GGAAGACAACCGTAGGGACT	COLEC11__NM_001255986	287	255	291	389
COLEC11__NM_001255986_TGGGTGCCTCCGAGTCCCTA	COLEC11__NM_001255986	91	74	87	81
COLEC11__NM_199235_CACCTCGTCCTTTGTAGAAA	COLEC11__NM_199235	690	928	855	1056
COLEC11__NM_199235_CTTTGTAGAAATGGGATCAC	COLEC11__NM_199235	217	278	268	357
COLEC11__NM_199235_TCCCATTTCTACAAAGGACG	COLEC11__NM_199235	74	205	72	147
COLEC12__NM_130386_ACCAAACCGCTTGTAACCGA	COLEC12__NM_130386	963	962	1216	1155
COLEC12__NM_130386_ATCTAGAATGTCTGATCTGA	COLEC12__NM_130386	304	171	312	267
COLEC12__NM_130386_CTGGAGAAGTTACAGGCGAG	COLEC12__NM_130386	448	448	578	587
COLEC12__NM_130386_GAAAGTGACCTGAAAAAATT	COLEC12__NM_130386	323	460	455	437
COLEC12__NM_130386_GAAGGTGGAGAGTTCTGAGT	COLEC12__NM_130386	105	37	107	32
COLEC12__NM_130386_GAGAAAATGGACAATGTCAC	COLEC12__NM_130386	403	512	536	437
COLEC12__NM_130386_GGAGAAGTTACAGGCGAGCG	COLEC12__NM_130386	528	571	554	605
COLEC12__NM_130386_GGCTACTGTGATTGTTAGCA	COLEC12__NM_130386	467	414	521	727
COLEC12__NM_130386_TCCTTCGGTTACAAGCGGTT	COLEC12__NM_130386	190	108	219	166
COLEC12__NM_130386_TGAGCTTGTCATCATAGGTT	COLEC12__NM_130386	197	179	216	152
COLEC12__NM_130386_TGCAATCCTTCGGTTACAAG	COLEC12__NM_130386	45	25	84	159
COLEC12__NM_130386_TGGAGAAGTTACAGGCGAGC	COLEC12__NM_130386	72	130	68	99
COLGALT1__NM_024656_AAAGACGATGATGTCGTCAA	COLGALT1__NM_024656	176	221	199	306
COLGALT1__NM_024656_ACGAGGAAGGCCCGAAACAC	COLGALT1__NM_024656	984	623	982	670
COLGALT1__NM_024656_AGAAGTTGGAGTACGCAGCC	COLGALT1__NM_024656	128	278	146	232
COLGALT1__NM_024656_CAAAGGACCAGGTGTAGTCA	COLGALT1__NM_024656	579	479	780	1009
COLGALT1__NM_024656_CACCAAGACACCGGACAAGA	COLGALT1__NM_024656	868	1165	936	1182
COLGALT1__NM_024656_CACCTCACCCTGACTACACC	COLGALT1__NM_024656	1570	2027	1857	2197
COLGALT1__NM_024656_CAGCCCGGGAATCCAGCATG	COLGALT1__NM_024656	132	145	89	19
COLGALT1__NM_024656_CCGCCTTCCGCAGGTCGATC	COLGALT1__NM_024656	903	862	1145	1353
COLGALT1__NM_024656_CCTCGTCCGGGTAGGACCTA	COLGALT1__NM_024656	232	301	211	255
COLGALT1__NM_024656_CGTGAGTCAGACCAGTGTTT	COLGALT1__NM_024656	1451	1019	1851	1159
COLGALT1__NM_024656_GCCTTCTCCTGCAAGCAGGC	COLGALT1__NM_024656	287	351	342	493
COLGALT1__NM_024656_GGCGGTCTCGCTTGCGGATA	COLGALT1__NM_024656	800	993	946	1341
COLGALT2__NM_015101_AAAAACAGAAAGAGCATCTG	COLGALT2__NM_015101	155	237	158	129
COLGALT2__NM_015101_ACAAACTAGACCAATAAGTG	COLGALT2__NM_015101	29	24	37	21
COLGALT2__NM_015101_ACAGAGACTCTATCACTATG	COLGALT2__NM_015101	588	671	742	635
COLGALT2__NM_015101_ACAGTTTGTGTAGGTATTTG	COLGALT2__NM_015101	422	713	448	508
COLGALT2__NM_015101_ACCAATAAGTGTGGTCTAGT	COLGALT2__NM_015101	507	495	594	637
COLGALT2__NM_015101_AGACAGGGTTTAGCCTCCAG	COLGALT2__NM_015101	525	564	460	347
COLGALT2__NM_015101_AGCTTGCTGGTCAGTGTAGA	COLGALT2__NM_015101	300	244	344	414
COLGALT2__NM_015101_AGGACCGCCACGAGCACCGT	COLGALT2__NM_015101	1558	1161	1465	1021
COLGALT2__NM_015101_ATAGAGCATCCTGACTCGAG	COLGALT2__NM_015101	776	480	718	520
COLGALT2__NM_015101_ATGGGGGGATTTGAACTATT	COLGALT2__NM_015101	362	485	338	332
COLGALT2__NM_015101_ATTATCCACATTGTGATCAG	COLGALT2__NM_015101	173	105	154	96
COLGALT2__NM_015101_ATTTCTCTTGGGATCTAACG	COLGALT2__NM_015101	29	6	29	54
COLGALT2__NM_015101_ATTTGGGTCCGACTGAGGAC	COLGALT2__NM_015101	409	473	525	392
COLGALT2__NM_015101_CAGAGGATGTTGAACTGATG	COLGALT2__NM_015101	819	1138	971	1154
COLGALT2__NM_015101_CATCACATGGGCAAACCGGG	COLGALT2__NM_015101	699	618	929	808
COLGALT2__NM_015101_CATCACCAAATAGGTAGTCA	COLGALT2__NM_015101	298	430	360	387
COLGALT2__NM_015101_CATCAGCAAATATGTAGTCA	COLGALT2__NM_015101	531	465	550	320
COLGALT2__NM_015101_CATCCTAGTATAGATCATGG	COLGALT2__NM_015101	399	230	440	237
COLGALT2__NM_015101_CATGCACCATGCAGCCATAT	COLGALT2__NM_015101	529	672	597	588
COLGALT2__NM_015101_CCATGTGATGAAACTACGAC	COLGALT2__NM_015101	447	611	461	494
COLGALT2__NM_015101_CGGTGGGTTCCCAGGCTAAG	COLGALT2__NM_015101	110	166	120	53
COLGALT2__NM_015101_CTGCCTCTTGTCCTAAAGTG	COLGALT2__NM_015101	309	393	423	397
COLGALT2__NM_015101_GAAAGCAGAAAGGACTCTTA	COLGALT2__NM_015101	400	275	412	187
COLGALT2__NM_015101_GAGCGCGCTTCGTCGCCGAG	COLGALT2__NM_015101	260	600	263	464
COLGALT2__NM_015101_GCAGACTTCCACGGCACCCG	COLGALT2__NM_015101	1158	1207	1404	1647
COLGALT2__NM_015101_GCAGAGCCCCACGGTGCTCG	COLGALT2__NM_015101	15	2	6	0
COLGALT2__NM_015101_GCCCCGCTTGCGGTTCATTC	COLGALT2__NM_015101	1071	1330	1217	1396
COLGALT2__NM_015101_GGAGAAACAAACCGGGCTAA	COLGALT2__NM_015101	337	403	506	418
COLGALT2__NM_015101_GGAGAGGAGCAGTAGCGACC	COLGALT2__NM_015101	80	79	79	66
COLGALT2__NM_015101_GGCTGCCTTGACTACCTATT	COLGALT2__NM_015101	2517	2498	3001	2950
COLGALT2__NM_015101_GTACCCACAGTGAGTCGGGA	COLGALT2__NM_015101	374	356	457	398
COLGALT2__NM_015101_GTATAGAGGCCTTTGCATCT	COLGALT2__NM_015101	720	533	853	640
COLGALT2__NM_015101_GTCTTGGCTTAGGACTATAA	COLGALT2__NM_015101	109	142	115	64
COLGALT2__NM_015101_TCGGCAGCCTTCGCGGAGCA	COLGALT2__NM_015101	280	367	259	352
COLGALT2__NM_015101_TCTATCACACTAGATCACTT	COLGALT2__NM_015101	370	195	328	309
COLGALT2__NM_015101_TCTGGAGGCTAAACCCTGTC	COLGALT2__NM_015101	455	586	579	484
COLGALT2__NM_015101_TGAGGGTCTGTGGATTAGTC	COLGALT2__NM_015101	721	900	984	1142
COLGALT2__NM_015101_TGGGTTCCCAGGCTAAGGGG	COLGALT2__NM_015101	0	0	0	0
COLGALT2__NM_015101_TGTGGCAATCAGTATCACAT	COLGALT2__NM_015101	538	504	512	408
COLGALT2__NM_015101_TTAGTCCCTGTTATCCAGTA	COLGALT2__NM_015101	351	223	362	387
COLGALT2__NM_015101_TTATTATGGCCACTCGAGTC	COLGALT2__NM_015101	252	186	315	411
COLGALT2__NM_015101_TTCTGCAATCAGTAGATTGA	COLGALT2__NM_015101	0	0	0	0
COLGALT2__NM_015101_TTGCAGAGGACACGCTGCAT	COLGALT2__NM_015101	1415	1335	1409	1310
COLGALT2__NM_015101_TTTCATCACATGGGCAAACC	COLGALT2__NM_015101	77	84	55	81
COLGALT2__NM_015101_TTTCTCTTGGGATCTAACGT	COLGALT2__NM_015101	125	200	121	113
COLQ__NM_005677_AAAGGGGATAGTGGAGTTAT	COLQ__NM_005677	228	207	268	152
COLQ__NM_005677_AAAGGTGAACCTGGGATAGC	COLQ__NM_005677	4	0	15	1
COLQ__NM_005677_AGATAAATTCCCAAAGTCAT	COLQ__NM_005677	309	267	459	591
COLQ__NM_005677_AGTGGAGTTATGGGCCCACC	COLQ__NM_005677	841	801	782	632
COLQ__NM_005677_CACCTCTGAAGAATGGTGGT	COLQ__NM_005677	741	824	823	950
COLQ__NM_005677_CCAGGATTTCCTGGAATGTT	COLQ__NM_005677	495	853	625	742
COLQ__NM_005677_CCTGGGCCCATCGGTTGGCC	COLQ__NM_005677	598	676	643	802
COLQ__NM_005677_CTAGCGAGCCTTGCATGCAC	COLQ__NM_005677	273	187	335	227
COLQ__NM_005677_GAAAGGGGATAGTGGAGTTA	COLQ__NM_005677	177	109	221	99
COLQ__NM_005677_GGGTTATTCACATTCATAGT	COLQ__NM_005677	72	65	96	49
COLQ__NM_005677_GGTCGGCCAAGCTCCCCCTA	COLQ__NM_005677	181	162	212	98
COLQ__NM_005677_TAGCGAGCCTTGCATGCACG	COLQ__NM_005677	614	296	626	370
COLQ__NM_080538_GGACTGCTCTGTTACTCGGC	COLQ__NM_080538	861	810	981	1105
COLQ__NM_080539_ACACAGATTCCCCGTAGGAA	COLQ__NM_080539	447	373	572	480
COLQ__NM_080539_TACACAGATTCCCCGTAGGA	COLQ__NM_080539	475	489	615	342
COMMD1__NM_152516_AACCAGAGCCGCTGGAATAG	COMMD1__NM_152516	712	786	698	739
COMMD1__NM_152516_ACCTGTTGCCATTATAGAGC	COMMD1__NM_152516	574	559	654	839
COMMD1__NM_152516_ACGGAGCCAGCTATATCCAG	COMMD1__NM_152516	1251	1016	1335	873
COMMD1__NM_152516_ACTGCTCAAACCAAAAAGCA	COMMD1__NM_152516	112	119	176	210
COMMD1__NM_152516_ATGGCGGCGGCGGAGCTTGA	COMMD1__NM_152516	577	432	701	611
COMMD1__NM_152516_CCCGAAGCCCGCTATTCCAG	COMMD1__NM_152516	2116	1659	2279	2051
COMMD1__NM_152516_GAGCTGGAATTAGGCAAATA	COMMD1__NM_152516	356	453	467	507
COMMD1__NM_152516_TCCAGCTCTATAATGGCAAC	COMMD1__NM_152516	52	75	41	26
COMMD10__NM_016144_AGAATCCGAGTGAGCAACCG	COMMD10__NM_016144	249	247	232	200
COMMD10__NM_016144_ATGGCAGCTTAACCTTCAGA	COMMD10__NM_016144	534	188	545	353
COMMD10__NM_016144_CCTCAAGCTGTGTTACAACT	COMMD10__NM_016144	1105	897	1229	1051
COMMD10__NM_016144_CTGATAAATGCAATAGATAC	COMMD10__NM_016144	283	356	249	289
COMMD10__NM_016144_GATTTCCACGGTTGCTCACT	COMMD10__NM_016144	704	634	684	717
COMMD10__NM_016144_TCCCCGCGGCGCTGATCCTA	COMMD10__NM_016144	693	492	864	749
COMMD2__NM_016094_CAAGGTTATGATAACTGGGA	COMMD2__NM_016094	76	66	92	97
COMMD2__NM_016094_CAGAACGATTCTGAGTGAAT	COMMD2__NM_016094	579	685	742	736
COMMD2__NM_016094_CATTCAAGGTTATGATAACT	COMMD2__NM_016094	161	55	196	119
COMMD2__NM_016094_CCAGTTATCATAACCTTGAA	COMMD2__NM_016094	673	665	762	775
COMMD2__NM_016094_CCATTCAAGGTTATGATAAC	COMMD2__NM_016094	368	335	462	346
COMMD2__NM_016094_CGAGTTTGGGCGGATTGCTG	COMMD2__NM_016094	963	840	1078	782
COMMD2__NM_016094_GATGCTGCTGGAATTGTCCG	COMMD2__NM_016094	1502	1673	1836	1401
COMMD2__NM_016094_GCAAACCCAAAAATCTACGA	COMMD2__NM_016094	494	635	526	665
COMMD2__NM_016094_GGAATTGTCCGAGGAGCATA	COMMD2__NM_016094	92	12	85	30
COMMD2__NM_016094_GTTCCAGTTGTTGAACCAAA	COMMD2__NM_016094	252	209	199	222
COMMD2__NM_016094_TGAATGGCGACTAGATGTAC	COMMD2__NM_016094	264	184	242	121
COMMD2__NM_016094_TGTAGCTTTATAGTCACTGC	COMMD2__NM_016094	100	72	175	116
COMMD3__NM_012071_AAGAAACATCCGTTATATGA	COMMD3__NM_012071	434	262	499	154
COMMD3__NM_012071_AGAGCAACTCAGTTGTAACT	COMMD3__NM_012071	370	607	466	414
COMMD3__NM_012071_CAGATCTCTCCCTCATATAA	COMMD3__NM_012071	445	250	534	347
COMMD3__NM_012071_CTTAAGGTCACCAAATATGC	COMMD3__NM_012071	303	315	310	333
COMMD3__NM_012071_TTTGGTGACCTTAAGTGTAC	COMMD3__NM_012071	64	222	84	142
COMMD3-BMI1__NM_001204062_AACGTGTATTGTTCGTTA	COMMD3-BMI1__NM_001204062	553	635	654	801
COMMD3-BMI1__NM_001204062_ACAAATAGGACAATACTT	COMMD3-BMI1__NM_001204062	427	704	465	498
COMMD3-BMI1__NM_001204062_AGCTGCAACTTACATACT	COMMD3-BMI1__NM_001204062	384	213	488	424
COMMD3-BMI1__NM_001204062_CTTGAAACATATCGACCC	COMMD3-BMI1__NM_001204062	793	776	805	698
COMMD3-BMI1__NM_001204062_GATGGGGAATCAGTGTT	COMMD3-BMI1__NM_001204062	750	465	811	617
COMMD3-BMI1__NM_001204062_GCAACTTACATACTAGAG	COMMD3-BMI1__NM_001204062	496	606	557	618
COMMD3-BMI1__NM_001204062_GGAATGTAGACATTCTA	COMMD3-BMI1__NM_001204062	298	591	366	494
COMMD3-BMI1__NM_001204062_GGGTCGATATGTTTCAA	COMMD3-BMI1__NM_001204062	236	211	280	305
COMMD3-BMI1__NM_001204062_GTGGTCTGGTCTTGTGA	COMMD3-BMI1__NM_001204062	16	11	24	43
COMMD3-BMI1__NM_001204062_TGAACTTGGACATCACAA	COMMD3-BMI1__NM_001204062	103	126	84	134
COMMD3-BMI1__NM_001204062_TTAGAGTTGACTTGTCAC	COMMD3-BMI1__NM_001204062	111	128	120	100
COMMD3-BMI1__NM_001204062_TTGCAGCATGGAACAATT	COMMD3-BMI1__NM_001204062	87	34	73	53
COMMD4__NM_001284377_CAGCCTGTGCCGCTGTTATG	COMMD4__NM_001284377	758	377	888	433
COMMD4__NM_001284377_CCAGCAGCTCCTTTAGTACC	COMMD4__NM_001284377	906	352	879	333
COMMD4__NM_001284377_CTCACGGCTGACGCCAAGTT	COMMD4__NM_001284377	122	111	168	275
COMMD4__NM_001284377_CTCAGAGTCAGGCGATGTGA	COMMD4__NM_001284377	215	248	255	168
COMMD4__NM_001284377_GTGATCTGGACTGTCCCGAC	COMMD4__NM_001284377	268	241	329	224
COMMD4__NM_001284377_TGATCTGGACTGTCCCGACT	COMMD4__NM_001284377	281	115	360	179
COMMD4__NM_001284377_TTTGCTTCTCCTCATAACAG	COMMD4__NM_001284377	117	6	78	30
COMMD4__NM_001284379_TGAGGGCTGTGAGTCTGGTA	COMMD4__NM_001284379	348	499	334	274
COMMD4__NM_017828_CTGGAACTTGTCTGCTGAGA	COMMD4__NM_017828	433	413	457	400
COMMD4__NM_017828_GAGGGACATGGCAACAGGCT	COMMD4__NM_017828	433	397	380	280
COMMD4__NM_017828_TCCAGCCGCAGGTGCACCAT	COMMD4__NM_017828	1164	877	1129	780
COMMD5__NM_001081003_ACTTGGCCAGCGTGGTATTT	COMMD5__NM_001081003	588	344	583	585
COMMD5__NM_001081003_CAGCAATGGCCCGGCTACTA	COMMD5__NM_001081003	957	762	1140	900
COMMD5__NM_001081003_CGCATGTTGCTGACTTTCGG	COMMD5__NM_001081003	2313	2203	2619	2680
COMMD5__NM_001081003_GACTTGGCCAGCGTGGTATT	COMMD5__NM_001081003	491	353	570	669
COMMD5__NM_001081003_GCTCCTGGAACTTGGCTGTG	COMMD5__NM_001081003	963	677	880	801
COMMD5__NM_001081003_GTCTAGGTCCCCTAGTAGCC	COMMD5__NM_001081003	452	689	459	736
COMMD5__NM_001081003_TGCATCCCCCAAGACCTGGT	COMMD5__NM_001081003	597	187	623	251
COMMD5__NM_001081003_TGGGTCAGCATACCGCTTTG	COMMD5__NM_001081003	24	5	29	11
COMMD5__NM_001081003_TGTCTAGGTCCCCTAGTAGC	COMMD5__NM_001081003	63	21	64	86
COMMD5__NM_001081003_TTCAGCTGCATCAGGACGCT	COMMD5__NM_001081003	93	81	67	51
COMMD6__NM_001287392_CAAAGCACTTGGTCTTTACT	COMMD6__NM_001287392	920	1202	1071	1262
COMMD6__NM_001287392_CGTTGCAGTGATGCTAAAAG	COMMD6__NM_001287392	176	105	253	177
COMMD6__NM_001287392_TTTAGCATCACTGCAACGTA	COMMD6__NM_001287392	76	63	109	195
COMMD7__NM_001099339_AACTGAGGCAAGGTTAATTC	COMMD7__NM_001099339	216	237	156	163
COMMD7__NM_001099339_AATCATCAGAGTCTGACCTA	COMMD7__NM_001099339	565	350	686	496
COMMD7__NM_001099339_ACCTATGGCCCATCGAGCAA	COMMD7__NM_001099339	302	144	237	233
COMMD7__NM_001099339_AGCCTCCTTCTGGTTCCAAA	COMMD7__NM_001099339	290	428	407	504
COMMD7__NM_001099339_CAAACCGAAAATGTGTATAT	COMMD7__NM_001099339	716	629	690	675
COMMD7__NM_001099339_CGGATTTCATAACTCTGGGT	COMMD7__NM_001099339	467	423	661	564
COMMD7__NM_001099339_CTGAGGCAAGGTTAATTCTG	COMMD7__NM_001099339	402	152	497	367
COMMD7__NM_001099339_GACATCTGGGAGCAGCGAAT	COMMD7__NM_001099339	320	341	311	169
COMMD7__NM_001099339_GATTAACCAGCTCATAGATA	COMMD7__NM_001099339	233	279	258	249
COMMD7__NM_001099339_TATGGCCCATCGAGCAAGGG	COMMD7__NM_001099339	171	150	228	146
COMMD7__NM_001099339_TCAGCAGAAACACTCCATGC	COMMD7__NM_001099339	1097	872	1232	908
COMMD7__NM_001099339_TGCTCCCACCCTTGCTCGAT	COMMD7__NM_001099339	635	820	584	865
COMMD8__NM_017845_ACCAAGATTATCACACTGTT	COMMD8__NM_017845	1191	1620	1343	1957
COMMD8__NM_017845_CCAAGATTATCACACTGTTT	COMMD8__NM_017845	1271	1094	1387	1439
COMMD8__NM_017845_CGGAAGAGGGGACGCCCTTG	COMMD8__NM_017845	739	583	879	768
COMMD8__NM_017845_GTCCTTGGAAGCAGCGAATA	COMMD8__NM_017845	106	94	140	171
COMMD8__NM_017845_TAATGCAGCAATCTTGTCAC	COMMD8__NM_017845	1035	1081	1207	1277
COMMD8__NM_017845_TAGGAAAGATGAAATCAAAC	COMMD8__NM_017845	120	111	153	94
COMMD8__NM_017845_TCATGAAATGCGTGAAAAGT	COMMD8__NM_017845	1028	1266	1056	1273
COMMD8__NM_017845_TTCTATTGAAATGAGTAGAG	COMMD8__NM_017845	0	0	0	0
COMMD9__NM_001101653_AAAGAGAGCCAGAATTGCCT	COMMD9__NM_001101653	202	213	253	155
COMMD9__NM_001101653_ACACTGGACACCATGTTAGA	COMMD9__NM_001101653	806	777	939	685
COMMD9__NM_001101653_CACGCCTGGTCGATCTGGAC	COMMD9__NM_001101653	3618	4646	4053	4370
COMMD9__NM_001101653_CCTCGGCAGAGGACAGGTCA	COMMD9__NM_001101653	98	93	86	133
COMMD9__NM_001101653_CTCTCCAGTCCAGATCGACC	COMMD9__NM_001101653	269	346	388	320
COMMD9__NM_001101653_GACACCATGTTAGATGGCCT	COMMD9__NM_001101653	1151	1493	1226	1807
COMMD9__NM_001101653_GGACACCATGTTAGATGGCC	COMMD9__NM_001101653	594	864	628	927
COMMD9__NM_001101653_GGCAGGTGGGGACGGCCATG	COMMD9__NM_001101653	475	502	485	374
COMMD9__NM_001101653_GGGTTTGTCTCCGCATAGGC	COMMD9__NM_001101653	37	20	20	59
COMMD9__NM_001101653_GTCCTCTGCCGAGGCAATTC	COMMD9__NM_001101653	68	108	68	69
COMMD9__NM_001101653_TCTGCACCGCCTCACTAGGC	COMMD9__NM_001101653	67	116	54	31
COMMD9__NM_001101653_TCTGCCACGCCTGGTCGATC	COMMD9__NM_001101653	650	615	854	795
COMMD9__NM_014186_CTGTCTGACAACATCTTTCG	COMMD9__NM_014186	512	635	606	647
COMP__NM_000095_ACTCCGTGTGCATCAACACC	COMP__NM_000095	632	378	604	329
COMP__NM_000095_ATGCAGCAGTCAGTACGCAC	COMP__NM_000095	504	357	495	439
COMP__NM_000095_CACCCTCACCGCGGCTGGAT	COMP__NM_000095	1486	1836	1908	1818
COMP__NM_000095_CCCGCGGGCTTCACGGGCAA	COMP__NM_000095	380	481	384	497
COMP__NM_000095_GCACCGGCCTACCCAGCGTG	COMP__NM_000095	559	474	566	571
COMP__NM_000095_GCACTGCACCGACGTCAACG	COMP__NM_000095	2864	2745	3371	3445
COMP__NM_000095_GCCGCTCTCCGTCTGGATGC	COMP__NM_000095	1218	1558	1458	1389
COMP__NM_000095_GCTTGCCCGCCGGGGTACAG	COMP__NM_000095	394	397	560	545
COMP__NM_000095_GTGTTGATGCACACGGAGTT	COMP__NM_000095	423	370	502	460
COMP__NM_000095_GTTGATGTCCGTGCAAACCT	COMP__NM_000095	341	338	441	337
COMP__NM_000095_TGAGGGTGGCTGTCATCCGG	COMP__NM_000095	668	562	683	314
COMP__NM_000095_TGCAGTTCCCGAAGCATCTG	COMP__NM_000095	259	365	342	497
COMP__NM_000095_TGGTGTTGATACAGCGGACT	COMP__NM_000095	12	19	6	15
COMP__NM_000095_TGTTGATGCACACGGAGTTG	COMP__NM_000095	237	181	322	228
COMT__NM_000754_ACAGTCGGGGTTGATCTCGA	COMT__NM_000754	799	523	763	387
COMT__NM_000754_ACGGACGCTAACGCTAAGGG	COMT__NM_000754	124	36	91	44
COMT__NM_000754_AGCCACCTCAGAGGCTCCAA	COMT__NM_000754	0	0	0	0
COMT__NM_000754_AGGCCGGCAGTCGTCCCCAT	COMT__NM_000754	1524	843	1531	794
COMT__NM_000754_ATGGGGACAGCTCTAGGTGT	COMT__NM_000754	299	234	284	127
COMT__NM_000754_CAGCGGATGGTGGATTTCGC	COMT__NM_000754	1273	890	1361	957
COMT__NM_000754_CAGGCCGGCAGTCGTCCCCA	COMT__NM_000754	2591	1195	2495	1164
COMT__NM_000754_CCCTGTATTCCAGGAACGAT	COMT__NM_000754	714	605	815	687
COMT__NM_000754_CGGACGCTAACGCTAAGGGC	COMT__NM_000754	44	34	56	14
COMT__NM_000754_CTCCTTCCCTACTCCCCACT	COMT__NM_000754	1261	983	1407	979
COMT__NM_000754_CTTAGCGTTAGCGTCCGTCA	COMT__NM_000754	426	374	553	362
COMT__NM_000754_CTTGACGGACGCTAACGCTA	COMT__NM_000754	310	195	338	266
COMT__NM_000754_GCGGAAGGGGACAGTGCTAC	COMT__NM_000754	371	214	375	172
COMT__NM_000754_GGACAGCTCTAGGTGTAGGT	COMT__NM_000754	480	311	557	270
COMT__NM_000754_GGTCTGTCTATCCATAGGAG	COMT__NM_000754	345	468	413	269
COMT__NM_000754_GTTCCTGGAATACAGGGAGG	COMT__NM_000754	119	91	107	81
COMT__NM_000754_TCACTGGTCTGTCTATCCAT	COMT__NM_000754	2515	1933	2086	1261
COMT__NM_000754_TCTCCTTCCCTACTCCCCAC	COMT__NM_000754	411	405	422	198
COMT__NM_000754_TGCTCCCGCGCACGTGTGCT	COMT__NM_000754	242	447	362	460
COMT__NM_000754_TGGGCCAGGGAGGCAACTAC	COMT__NM_000754	177	151	161	142
COMT__NM_000754_TTAGCGTTAGCGTCCGTCAA	COMT__NM_000754	189	161	185	96
COMT__NM_000754_TTGACGGACGCTAACGCTAA	COMT__NM_000754	55	4	80	28
COMT__NM_000754_TTTCTTGTCGCCCACGTTCA	COMT__NM_000754	203	150	242	257
COMTD1__NM_144589_AAGGTGCTGCAACCTCCGAA	COMTD1__NM_144589	219	168	285	261
COMTD1__NM_144589_ACCTAAACGAACGCATCCGG	COMTD1__NM_144589	1076	1194	1208	1150
COMTD1__NM_144589_AGCTCTTGGCCAACCTGGCG	COMTD1__NM_144589	157	174	178	146
COMTD1__NM_144589_AGGTGCTGCAACCTCCGAAA	COMTD1__NM_144589	73	36	86	30
COMTD1__NM_144589_CAGGGCGGAGTAGCCCGTGA	COMTD1__NM_144589	846	1009	1061	948
COMTD1__NM_144589_CCCGCCGGATGCGTTCGTTT	COMTD1__NM_144589	3564	3757	3963	3747
COMTD1__NM_144589_CCTAAACGAACGCATCCGGC	COMTD1__NM_144589	301	306	299	305
COMTD1__NM_144589_CTCCTTGTCCGCATCCACCA	COMTD1__NM_144589	1326	1262	1350	1616
COMTD1__NM_144589_GAAACCTAAACGAACGCATC	COMTD1__NM_144589	127	177	259	251
COMTD1__NM_144589_GGTGCTGCAACCTCCGAAAG	COMTD1__NM_144589	303	226	330	249
COMTD1__NM_144589_GTCATCATAGAATCCCCCTG	COMTD1__NM_144589	502	295	482	489
COPA__NM_001098398_ACATCCGATAGTCCCATAAC	COPA__NM_001098398	127	0	88	0
COPA__NM_001098398_ACCAAGAGCGCGCGGGTCAA	COPA__NM_001098398	319	2	266	1
COPA__NM_001098398_AGCGCGCGGGTCAAAGGTAG	COPA__NM_001098398	488	5	366	38
COPA__NM_001098398_AGGAGATGACTATAAGATTA	COPA__NM_001098398	153	6	132	0
COPA__NM_001098398_ATCCTGACTAGTTTACATAA	COPA__NM_001098398	275	52	274	53
COPA__NM_001098398_ATGATCAGACCATCCGAGTG	COPA__NM_001098398	130	3	138	21
COPA__NM_001098398_CACTCGGATGGTCTGATCAT	COPA__NM_001098398	67	0	42	0
COPA__NM_001098398_CAGCCACTGTTCGTCTCTGG	COPA__NM_001098398	2783	296	2954	581
COPA__NM_001098398_CCCCATTATGTAAACTAGTC	COPA__NM_001098398	561	832	587	866
COPA__NM_001098398_TCATCCAGTTATGGGACTAT	COPA__NM_001098398	516	0	439	12
COPA__NM_001098398_TCTCCTCCAGAGACGAACAG	COPA__NM_001098398	0	0	2	0
COPA__NM_001098398_TGGAAGTCAATGCCTCGCAC	COPA__NM_001098398	21	0	32	0
COPA__NM_004371_CATTCTCTGGGGGGAACATA	COPA__NM_004371	458	405	446	431
COPA__NM_004371_GAGATGATTAAGAGTTGGTG	COPA__NM_004371	178	89	208	166
COPB1__NM_001144061_AGGTAGCACAAAACGAATGA	COPB1__NM_001144061	133	25	115	10
COPB1__NM_001144061_ATAACATTTGCAGCCATATC	COPB1__NM_001144061	128	36	162	78
COPB1__NM_001144061_CACGTTAATTAACGTGCCAA	COPB1__NM_001144061	0	0	0	0
COPB1__NM_001144061_CAGAAATTGGTTACTGAAAT	COPB1__NM_001144061	292	508	327	318
COPB1__NM_001144061_CCGAGGAGCATTATGGATCC	COPB1__NM_001144061	610	54	709	157
COPB1__NM_001144061_CCTGAAGAAGAAATAACTGT	COPB1__NM_001144061	767	464	811	577
COPB1__NM_001144061_CTGAAGAAGAAATAACTGTA	COPB1__NM_001144061	251	171	340	191
COPB1__NM_001144061_TAGTGCACCAACTGCAATCA	COPB1__NM_001144061	238	96	262	64
COPB1__NM_001144061_TCATGTAAAAGTCTCCCATC	COPB1__NM_001144061	254	108	276	171
COPB1__NM_001144061_TCCAAACATGCACGAATAGC	COPB1__NM_001144061	208	107	214	106
COPB1__NM_001144061_TTACCTATAGATGGTATAGA	COPB1__NM_001144061	38	27	66	61
COPB1__NM_001144061_TTTAAGCTAATTTCAGATGG	COPB1__NM_001144061	48	74	30	96
COPB1__NM_016451_TAATTTAGGTATGGGCTTAC	COPB1__NM_016451	237	151	214	182
COPB2__NM_004766_ACAAACTTTGCAGCTCGAAC	COPB2__NM_004766	94	95	144	177
COPB2__NM_004766_AGTGTTCAATTACAATACTC	COPB2__NM_004766	58	20	81	42
COPB2__NM_004766_ATCTGATCGAGTTAAGAGTG	COPB2__NM_004766	89	15	262	17
COPB2__NM_004766_ATGACATGCTTATTAAGCTC	COPB2__NM_004766	22	0	28	2
COPB2__NM_004766_ATGTTGGCAAGTCTTTACAA	COPB2__NM_004766	105	8	106	32
COPB2__NM_004766_CACAATCTGCATAACATAAT	COPB2__NM_004766	468	254	592	245
COPB2__NM_004766_CCACTGCTAGTTAGAATGAA	COPB2__NM_004766	463	73	439	47
COPB2__NM_004766_CCTTTCATTCTAACTAGCAG	COPB2__NM_004766	104	54	205	141
COPB2__NM_004766_GATCTGTTTAGTCAGGTAAA	COPB2__NM_004766	278	405	334	506
COPB2__NM_004766_GCTTAATAAGCATGTCATCT	COPB2__NM_004766	474	103	491	300
COPB2__NM_004766_TAAAGACTTGCCAACATCCA	COPB2__NM_004766	76	0	32	0
COPB2__NM_004766_TCACAATCTGCATAACATAA	COPB2__NM_004766	163	197	168	160
COPB2__NM_004766_TCACCAAACTTCACTTTGGA	COPB2__NM_004766	1590	1717	1884	2255
COPE__NM_007263_ACTACCTCGCCCACGAGAGT	COPE__NM_007263	470	67	373	25
COPE__NM_007263_CCCCTGGTGCAGCGCACGCA	COPE__NM_007263	194	21	262	30
COPE__NM_007263_CTACCAGCAGTGCATAAACG	COPE__NM_007263	249	42	253	50
COPE__NM_007263_CTCTCTGGGCTTGATAGCTG	COPE__NM_007263	60	118	73	117
COPE__NM_007263_CTGGTAGCTGCCGATGTAGA	COPE__NM_007263	301	63	409	0
COPE__NM_007263_CTTCCTGTATAGAGCGTACC	COPE__NM_007263	134	11	234	61
COPE__NM_007263_GATGAAGGGATGGGACCTGT	COPE__NM_007263	2395	1093	2410	1216
COPE__NM_007263_GCGCCAGGTACGCTCTATAC	COPE__NM_007263	865	71	701	33
COPE__NM_007263_GGTGCTGGGACAGGACGATG	COPE__NM_007263	529	25	481	37
COPE__NM_007263_GTAAAGAACGCCTTCTACAT	COPE__NM_007263	358	115	325	14
COPG1__NM_016128_AGGATGTGGGCACATTTCCG	COPG1__NM_016128	543	105	541	100
COPG1__NM_016128_CCGCACTCTTCTCAAGGTGC	COPG1__NM_016128	56	11	87	80
COPG1__NM_016128_CTGGGAAAGAAGACAACTAC	COPG1__NM_016128	215	94	217	119
COPG1__NM_016128_GATTCTTTATCTCATAAACC	COPG1__NM_016128	50	131	91	73
COPG1__NM_016128_GCCTAACAAAAGACATGACT	COPG1__NM_016128	1373	556	1273	661
COPG1__NM_016128_GGAGATGTCTTGCATTGCAG	COPG1__NM_016128	323	50	488	133
COPG1__NM_016128_GGGAGTTTCATTAAATACAC	COPG1__NM_016128	68	77	103	119
COPG1__NM_016128_GGTACAGGAGCCCTAGTGCG	COPG1__NM_016128	288	21	220	41
COPG1__NM_016128_GTAGCACATCCGACGGAGTG	COPG1__NM_016128	140	17	138	27
COPG1__NM_016128_GTCGAATTTCTTCAACATAG	COPG1__NM_016128	120	131	99	141
COPG1__NM_016128_TAGCACATCCGACGGAGTGT	COPG1__NM_016128	185	61	93	12
COPG1__NM_016128_TGGGAGTTTCATTAAATACA	COPG1__NM_016128	51	36	117	33
COPG2__NM_001290033_AAACATCTTCTTGGATTGAT	COPG2__NM_001290033	178	220	194	352
COPG2__NM_001290033_AAGTGATAATATTATGGTCC	COPG2__NM_001290033	168	272	193	266
COPG2__NM_001290033_AGAAGATGATATACCGAGGCC	COPG2__NM_001290033	316	368	324	379
COPG2__NM_001290033_CATTCTTTCTAAGGTGATAC	COPG2__NM_001290033	51	44	50	73
COPG2__NM_001290033_CATTGAAAGATACATGAAGC	COPG2__NM_001290033	390	441	430	628
COPG2__NM_001290033_GATACAGGACTCCCAATGCA	COPG2__NM_001290033	565	412	625	640
COPG2__NM_001290033_GCTATGATGTGGTTAAGCGC	COPG2__NM_001290033	212	155	281	192
COPG2__NM_012133_CACCATGAAGTTTACAGTCC	COPG2__NM_012133	636	368	649	601
COPG2__NM_012133_CAGAGGTGGCTATGATTTAT	COPG2__NM_012133	363	474	321	503
COPG2__NM_012133_GAATTCCCATTCGCTCTATC	COPG2__NM_012133	504	533	523	633
COPG2__NM_012133_GCACCATGAAGTTTACAGTC	COPG2__NM_012133	908	1046	780	819
COPG2__NM_012133_TGGGCATGCAGCCATGTGAG	COPG2__NM_012133	128	248	161	83
COPRS__NM_018405_ATCAAGGGAATCCATATGGT	COPRS__NM_018405	644	456	554	514
COPRS__NM_018405_CTGATGGAGAACTGAATACC	COPRS__NM_018405	88	73	171	139
COPRS__NM_018405_GAGAAGGCTATGGATCGACT	COPRS__NM_018405	464	407	378	323
COPRS__NM_018405_GCAGATCAAGGGAATCCATA	COPRS__NM_018405	200	271	220	216
COPRS__NM_018405_TTGGAATAATAGGGTATCAG	COPRS__NM_018405	12	1	28	35
COPS2__NM_001143887_AAACATAGATGTAGCTGATG	COPS2__NM_001143887	228	153	347	246
COPS2__NM_001143887_AATAAATCCATTTGACTCAC	COPS2__NM_001143887	374	30	380	55
COPS2__NM_001143887_AGCAGCTATTGACCTATATT	COPS2__NM_001143887	600	22	720	96
COPS2__NM_001143887_ATGCTAAGAATGATAGACTG	COPS2__NM_001143887	97	0	153	31
COPS2__NM_001143887_CATCCCTCATCCACTGATTA	COPS2__NM_001143887	1387	42	1725	79
COPS2__NM_001143887_CCAGTTACATCAGTCGTGCC	COPS2__NM_001143887	568	6	768	38
COPS2__NM_001143887_CGTACCTCCTGTGAGTCAAA	COPS2__NM_001143887	321	24	345	35
COPS2__NM_001143887_CTTGCTGGTGCAGTGCATAT	COPS2__NM_001143887	659	21	895	22
COPS2__NM_001143887_GGAATATGTATCTTGTGTA	COPS2__NM_001143887	723	19	641	102
COPS2__NM_001143887_GGAATTCTATGAAACAACAC	COPS2__NM_001143887	118	26	157	74
COPS2__NM_001143887_GGAGAAGATGATCTGAAAAA	COPS2__NM_001143887	77	8	133	49
COPS2__NM_001143887_TATTTAGAACGAGAGGAATA	COPS2__NM_001143887	23	0	23	0
COPS2__NM_004236_AAAACTTCCACCTAACTTTG	COPS2__NM_004236	153	247	145	234
COPS2__NM_004236_AAAAGAACGCACCTATATCT	COPS2__NM_004236	590	625	621	637
COPS2__NM_004236_AAAGACAAAATGTCTATGGT	COPS2__NM_004236	218	194	259	135
COPS2__NM_004236_AAATGAGCTGAGTTCCTTTA	COPS2__NM_004236	681	544	752	748
COPS2__NM_004236_AAGAAGGGCTTTAGCTTTCA	COPS2__NM_004236	667	556	753	665
COPS2__NM_004236_AAGACAAAATGTCTATGGTA	COPS2__NM_004236	206	227	235	198
COPS2__NM_004236_ACAGTATGCCACTATAATTA	COPS2__NM_004236	403	614	494	398
COPS2__NM_004236_ACGAAACCCCATAATTATAG	COPS2__NM_004236	268	223	289	446
COPS2__NM_004236_ACTATGATGTTGTACGTTTA	COPS2__NM_004236	360	458	491	644
COPS2__NM_004236_AGAAGGGCTTTAGCTTTCAA	COPS2__NM_004236	262	160	301	332
COPS2__NM_004236_AGCTTTGTTTACCCAGATAT	COPS2__NM_004236	719	748	807	947
COPS2__NM_004236_AGGGGAAGTTAAAAATCGAT	COPS2__NM_004236	392	493	460	616
COPS2__NM_004236_ATACACTGACACGCTACTGT	COPS2__NM_004236	381	260	525	367
COPS2__NM_004236_CATCAACTCAAAAGTTAAGA	COPS2__NM_004236	471	617	567	639
COPS2__NM_004236_CCTGAAAATTATTGGAGTTA	COPS2__NM_004236	1043	1286	1222	1592
COPS2__NM_004236_GAACTGGGCACTGTAAGAAT	COPS2__NM_004236	612	733	759	591
COPS2__NM_004236_GGGAATGGCAAAACTACTGT	COPS2__NM_004236	391	449	522	440
COPS2__NM_004236_GTAAGAGCAAGAGTGAGTAA	COPS2__NM_004236	308	255	269	245
COPS2__NM_004236_GTACAAAGCAAAGATAGCAC	COPS2__NM_004236	228	268	267	176
COPS2__NM_004236_TACTATGATGTTGTACGTTT	COPS2__NM_004236	373	333	486	361
COPS2__NM_004236_TATGATGTAAACCACTGGTA	COPS2__NM_004236	219	172	243	159
COPS2__NM_004236_TATGCATTTCAAGTTATTGC	COPS2__NM_004236	500	642	537	728
COPS2__NM_004236_TGAGCAGTGGCAGTATACTT	COPS2__NM_004236	563	557	585	627
COPS3__NM_001199125_AAGGATGCCAATTCCTCGCA	COPS3__NM_001199125	430	615	508	714
COPS3__NM_001199125_AGGATGCCAATTCCTCGCAG	COPS3__NM_001199125	397	1	270	37
COPS3__NM_001199125_ATCATATCACGTCAAGATA	COPS3__NM_001199125	182	0	161	33
COPS3__NM_001199125_CAAGTGCATTTGTTAGCTGA	COPS3__NM_001199125	695	538	626	499
COPS3__NM_001199125_GAGAATAGCGTTTCGAAGTC	COPS3__NM_001199125	187	143	157	157
COPS3__NM_001199125_GCAGAGATCAGCATGTATTG	COPS3__NM_001199125	518	37	479	54
COPS3__NM_001199125_TAAGGATGCCAATTCCTCGC	COPS3__NM_001199125	367	0	498	5
COPS3__NM_001199125_TCTTCATCAGCACTTGTAAT	COPS3__NM_001199125	331	90	360	142
COPS3__NM_001199125_TCTTGTCTATGGCTTGCTTA	COPS3__NM_001199125	254	277	308	235
COPS3__NM_001199125_TGAACTGATCAACAAGAGTG	COPS3__NM_001199125	0	0	0	0
COPS3__NM_001199125_TGACAGCCCCTGCGAGGAAT	COPS3__NM_001199125	619	70	715	33
COPS3__NM_001199125_TGCCCTTCCATATCTTGACG	COPS3__NM_001199125	720	641	767	838
COPS3__NM_003653_GCAAACTTTAAAGTCTGTCG	COPS3__NM_003653	812	1379	1006	1406
COPS3__NM_003653_GCCAGTGACAAGTGTTCGGA	COPS3__NM_003653	959	928	957	800
COPS3__NM_003653_GGCCAGTGACAAGTGTTCGG	COPS3__NM_003653	1094	1145	1140	1084
COPS3__NM_003653_TTCTCTTGTTTAGCTCAGGA	COPS3__NM_003653	0	0	0	0
COPS3__NM_003653_TTGGAAGATTTGGATCTGAA	COPS3__NM_003653	156	234	185	222
COPS4__NM_001258006_AAGAGCTCCAAGTTCTTCGA	COPS4__NM_001258006	230	258	299	425
COPS4__NM_001258006_ACGAGAAGCCCTGCCAACGT	COPS4__NM_001258006	1976	349	2132	348
COPS4__NM_001258006_ATGTCAGTCTCGTGATCTCG	COPS4__NM_001258006	943	57	1089	112
COPS4__NM_001258006_GAGCGGCTCTCATAAAGATC	COPS4__NM_001258006	214	165	299	134
COPS4__NM_001258006_GATTGCTAGGCTATATCTGG	COPS4__NM_001258006	513	27	729	77
COPS4__NM_001258006_GCCAGCAACTTGCTGCCTAT	COPS4__NM_001258006	527	200	598	222
COPS4__NM_001258006_GGCAGCCGTGCGACAGGATT	COPS4__NM_001258006	920	958	1043	1298
COPS4__NM_001258006_TAATCTACATTGTACTGTCT	COPS4__NM_001258006	376	67	491	146
COPS4__NM_001258006_TATCTAGATAGGATCATCAG	COPS4__NM_001258006	119	14	148	7
COPS4__NM_001258006_TGCCAGCAACTTGCTGCCTA	COPS4__NM_001258006	344	151	430	190
COPS4__NM_001258006_TGGATCTGCTTATCCCACGT	COPS4__NM_001258006	136	3	146	20
COPS4__NM_001258006_TGGCAGGGCTTCTCGTGCTA	COPS4__NM_001258006	118	4	106	15
COPS4__NM_016129_ACAGAGACCCTGAAATATAT	COPS4__NM_016129	417	305	515	658
COPS4__NM_016129_ATAACCGAAGGACGTATGAA	COPS4__NM_016129	387	45	334	113
COPS4__NM_016129_GCATCTCAAATGATAACCGA	COPS4__NM_016129	300	110	435	210
COPS5__NM_006837_AAAACTGTCCATAATGATCA	COPS5__NM_006837	197	67	279	275
COPS5__NM_006837_AAGCCCTGGACTAAGGAGTG	COPS5__NM_006837	336	40	422	118
COPS5__NM_006837_ATGGTGATGCATGCCAGATC	COPS5__NM_006837	139	6	160	18
COPS5__NM_006837_CAACAAGAACAATATCCGCA	COPS5__NM_006837	404	28	543	18
COPS5__NM_006837_CCAACAAGAACAATATCCGC	COPS5__NM_006837	492	74	507	115
COPS5__NM_006837_CCAGGAACCATTTGTAGCAG	COPS5__NM_006837	673	6	695	29
COPS5__NM_006837_GATGGGTCTGATGCTAGGAA	COPS5__NM_006837	94	0	80	0
COPS5__NM_006837_GATGGTGATGCATGCCAGAT	COPS5__NM_006837	502	26	626	26
COPS5__NM_006837_GCCTTGAAAATGCAATCGGG	COPS5__NM_006837	632	2	735	5
COPS5__NM_006837_TCCGCAGGGAAAGTGAATCT	COPS5__NM_006837	692	29	797	18
COPS5__NM_006837_TGAAACCCGAGTAAATGCTC	COPS5__NM_006837	5	0	39	0
COPS5__NM_006837_TGGTGAAACCATGATCATTA	COPS5__NM_006837	48	0	50	5
COPS6__NM_006833_AATCTTCTCTTCCACGGTGT	COPS6__NM_006833	260	97	343	117
COPS6__NM_006833_ACATCTGTGTGCTTGGTCAT	COPS6__NM_006833	1223	515	1189	675
COPS6__NM_006833_CAAGGAATATTATTACACCA	COPS6__NM_006833	247	67	310	172
COPS6__NM_006833_CAATCAGAGCCCCAATCACT	COPS6__NM_006833	1937	77	1974	169
COPS6__NM_006833_CATCACGCTGGGGACTACTA	COPS6__NM_006833	589	597	592	532
COPS6__NM_006833_CTGGGTTGGTATACCACAGG	COPS6__NM_006833	159	12	195	16
COPS6__NM_006833_CTTATGGACGTGGATGTCCG	COPS6__NM_006833	388	349	399	425
COPS6__NM_006833_GAGGGTACTTGAAGGGCTGA	COPS6__NM_006833	254	217	285	273
COPS6__NM_006833_GCAGGAGGGCCGAAATATCG	COPS6__NM_006833	450	11	521	57
COPS6__NM_006833_GCTGCCTGCTTCCACCTAAG	COPS6__NM_006833	1255	1187	1176	1128
COPS6__NM_006833_GGACATCCACGTCCATAAGC	COPS6__NM_006833	434	2	342	12
COPS6__NM_006833_GTGATGGCCTGCGGAGTGAC	COPS6__NM_006833	378	74	389	114
COPS6__NM_006833_TAGAACAGAGACCACTTAGG	COPS6__NM_006833	146	30	151	67
COPS6__NM_006833_TCTGGGTTGGTATACCACAG	COPS6__NM_006833	93	0	94	0
COPS6__NM_006833_TGGTGGCTCTGTCCTCTGTT	COPS6__NM_006833	97	106	103	128
COPS6__NM_006833_TTCTGGGTTGGTATACCACA	COPS6__NM_006833	8	1	35	13
COPS7A__NM_001164093_CAGCTGCCGCACATTACGCA	COPS7A__NM_001164093	209	373	266	230
COPS7A__NM_001164093_CCAGCTGCCGCACATTACGC	COPS7A__NM_001164093	747	1029	878	1183
COPS7A__NM_001164093_CCAGCTGCTGCTCCTTGTGT	COPS7A__NM_001164093	2683	2984	2832	2815
COPS7A__NM_001164093_CTCCTAGCCAAGTCGGCCAA	COPS7A__NM_001164093	137	256	106	119
COPS7A__NM_001164093_CTCTTGCCCTGCGTAATGTG	COPS7A__NM_001164093	268	554	328	367
COPS7A__NM_001164093_CTGCTCACAGTGTTTGCTTA	COPS7A__NM_001164093	341	584	465	523
COPS7A__NM_001164093_GGACATGCCCAATGTTAGAG	COPS7A__NM_001164093	233	308	226	396
COPS7A__NM_001164093_GGTCGTGCTGTCAGGCATTG	COPS7A__NM_001164093	384	446	386	541
COPS7A__NM_001164093_GTGCGGAAGTGAAGGTGACA	COPS7A__NM_001164093	316	310	323	285
COPS7A__NM_001164093_TCTCCAAACACGTAGACACC	COPS7A__NM_001164093	284	264	231	230
COPS7A__NM_001164093_TGCTCACAGTGTTTGCTTAT	COPS7A__NM_001164093	433	492	500	728
COPS7A__NM_001164093_TGCTGCCGTCGTAACTTTAA	COPS7A__NM_001164093	345	429	375	496
COPS7B__NM_001282949_AAACAGAAGTTAACTCTCTG	COPS7B__NM_001282949	316	181	385	396
COPS7B__NM_001282949_AACAGAAGTTAACTCTCTGC	COPS7B__NM_001282949	570	293	794	584
COPS7B__NM_001282949_CCTGCTGCTGAGTTCGGTTG	COPS7B__NM_001282949	1581	2102	1625	1919
COPS7B__NM_001282949_GGTATGTCCCATAGGCAAAC	COPS7B__NM_001282949	118	96	172	206
COPS7B__NM_001282949_GTAATCTGGGTATGTCCCAT	COPS7B__NM_001282949	359	274	368	388
COPS7B__NM_001282949_GTCTTTCAGCAACACGGAGT	COPS7B__NM_001282949	9	40	18	3
COPS7B__NM_001282949_TCTTGATGCCAAGCTCACGA	COPS7B__NM_001282949	349	330	283	237
COPS7B__NM_001282949_TCTTTCAGCAACACGGAGTA	COPS7B__NM_001282949	110	116	217	137
COPS7B__NM_001282949_TGTAGACAGCCTCAATGATA	COPS7B__NM_001282949	188	91	135	69
COPS7B__NM_001282950_AAGCTGTTGTTATAAGATTC	COPS7B__NM_001282950	206	150	132	69
COPS7B__NM_001282950_CCTCACTGCTCTCATAAGCC	COPS7B__NM_001282950	417	536	436	478
COPS7B__NM_001282950_CTCCGGGAGCCTCTAAGACC	COPS7B__NM_001282950	89	100	89	146
COPS7B__NM_001282950_TGCTCCAGGAGATTACTTGA	COPS7B__NM_001282950	741	664	729	799
COPS7B__NM_001282950_TTCTCCAAAGACATACACTC	COPS7B__NM_001282950	4	0	25	6
COPS8__NM_006710_AAACTGGTTTAGTATGGAAA	COPS8__NM_006710	209	213	279	246
COPS8__NM_006710_AACAAGTTTATTCCCTTATC	COPS8__NM_006710	193	260	224	267
COPS8__NM_006710_AAGTGTGGAATGCATTATAT	COPS8__NM_006710	483	407	530	408
COPS8__NM_006710_AATGGATATACTCACACTAT	COPS8__NM_006710	574	495	748	478
COPS8__NM_006710_ACTGCTGTTCATTGGGTATT	COPS8__NM_006710	1466	841	1758	1243
COPS8__NM_006710_AGAAGCTGACCATACACTGG	COPS8__NM_006710	462	33	421	35
COPS8__NM_006710_AGCCAGACTGACGGATTATG	COPS8__NM_006710	1009	1025	1089	870
COPS8__NM_006710_AGTGATTCAGATACTGTGCC	COPS8__NM_006710	221	262	364	332
COPS8__NM_006710_CGTACTCTTATATGTACCAT	COPS8__NM_006710	633	518	712	478
COPS8__NM_006710_CTGCTGTTCATTGGGTATTG	COPS8__NM_006710	400	358	436	465
COPS8__NM_006710_GCTAGAAGCTGACCATACAC	COPS8__NM_006710	344	38	389	15
COPS8__NM_006710_GTTAACAAACTGGTTTAGTA	COPS8__NM_006710	46	51	41	66
COPS8__NM_006710_TATAAGAGTACGGCATGTGC	COPS8__NM_006710	149	236	195	150
COPS8__NM_006710_TCTGGGCAGAACCATTCTTG	COPS8__NM_006710	627	474	593	355
COPS8__NM_006710_TGATGGTTGTATAGATCCCA	COPS8__NM_006710	123	0	76	17
COPS8__NM_006710_TGCTGTTCATTGGGTATTGG	COPS8__NM_006710	200	241	196	280
COPS8__NM_006710_TTCTACGTTTGTCAGTATAC	COPS8__NM_006710	0	0	0	0
COPZ1__NM_001271734_ACACGGTCTGCTCCGTAAGG	COPZ1__NM_001271734	239	114	192	59
COPZ1__NM_001271734_AGGGGGACATCTTCACCCTA	COPZ1__NM_001271734	180	209	265	194
COPZ1__NM_001271734_CCAGCAGGTGGTACACCGGG	COPZ1__NM_001271734	156	63	252	172
COPZ1__NM_001271734_GACACGGTCTGCTCCGTAAG	COPZ1__NM_001271734	182	164	240	130
COPZ1__NM_001271734_GATCTCTATTTCTATGTGAT	COPZ1__NM_001271734	379	379	397	344
COPZ1__NM_001271734_GGTACACCGGGCATTAA	COPZ1__NM_001271734	84	169	82	110
COPZ1__NM_001271734_GGTAGGTGTCGTCATAGTAC	COPZ1__NM_001271734	172	45	113	31
COPZ1__NM_001271735_GATGGCTTTGACAGTATACA	COPZ1__NM_001271735	219	60	263	23
COPZ1__NM_001271735_GCTTTGACAGTATACAGGGA	COPZ1__NM_001271735	376	165	482	181
COPZ1__NM_001271735_GGATGGCTTTGACAGTATAC	COPZ1__NM_001271735	285	23	181	4
COPZ2__NM_016429_AATGCTGTTCTTGTAGACGA	COPZ2__NM_016429	491	381	551	592
COPZ2__NM_016429_AGCCCACCACGTATAGGAAG	COPZ2__NM_016429	561	693	403	390
COPZ2__NM_016429_AGTCTCTGAACCACATGTTA	COPZ2__NM_016429	544	609	637	584
COPZ2__NM_016429_CACCTTCTGGATCACTTGCT	COPZ2__NM_016429	1489	1522	1529	1369
COPZ2__NM_016429_CATTGACCTCTTCCTATACG	COPZ2__NM_016429	982	1045	1117	928
COPZ2__NM_016429_GACCTCTTCCTATACGTGGT	COPZ2__NM_016429	1026	1192	1028	1257
COPZ2__NM_016429_GCCGGCGCCCGTCATTATCT	COPZ2__NM_016429	634	516	690	599
COPZ2__NM_016429_GTTCAGAGACTCAAACAGGC	COPZ2__NM_016429	154	114	115	184
COPZ2__NM_016429_TGACCTCTTCCTATACGTGG	COPZ2__NM_016429	500	461	583	527
COQ10A__NM_001099337_ATCTCCTGCATTGAGTACCT	COQ10A__NM_001099337	476	266	491	397
COQ10A__NM_001099337_CAACAAGCGAAAGGCTTACT	COQ10A__NM_001099337	904	537	618	331
COQ10A__NM_001099337_CCCATGAAGGAACGGCTCGA	COQ10A__NM_001099337	582	627	544	539
COQ10A__NM_001099337_CCTTCGAGCCGTTCCTTCAT	COQ10A__NM_001099337	961	623	1210	787
COQ10A__NM_001099337_CGAGTAAGCCTTTCGCTTGT	COQ10A__NM_001099337	2599	3548	3112	4144
COQ10A__NM_001099337_CTCAATGCAGGAGATGTATG	COQ10A__NM_001099337	244	308	287	245
COQ10A__NM_001099337_GGGAGCAGCAAATCCCATGA	COQ10A__NM_001099337	1290	1361	1384	960
COQ10A__NM_001099337_TACTCGGAGCGTAGAATCAT	COQ10A__NM_001099337	214	197	319	117
COQ10A__NM_001099337_TCCCTTCACCAACAAGCGAA	COQ10A__NM_001099337	0	0	6	0
COQ10A__NM_001099337_TCTGGGCAGCCCGAGGCAAG	COQ10A__NM_001099337	43	139	108	35
COQ10A__NM_001099337_TGAGGTGGTGTCCAACGTCC	COQ10A__NM_001099337	108	49	122	94
COQ10A__NM_001099337_TTACTCGGAGCGTAGAATCA	COQ10A__NM_001099337	96	52	135	87
COQ10B__NM_025147_AAAAAAGAGTGTGGCAAGCT	COQ10B__NM_025147	139	124	103	103
COQ10B__NM_025147_ATATACATCAGTAGTAACCT	COQ10B__NM_025147	128	91	185	113
COQ10B__NM_025147_CCGGCCGCTGTCGCCGACTT	COQ10B__NM_025147	1165	1364	1223	1255
COQ10B__NM_025147_GAAATGTATGATGTAGTATC	COQ10B__NM_025147	858	704	825	865
COQ10B__NM_025147_GGCAAAATTGAGGTATGTAG	COQ10B__NM_025147	356	378	499	484
COQ10B__NM_025147_GGCCTTGAGAAGGGTAGTCT	COQ10B__NM_025147	197	218	245	222
COQ10B__NM_025147_GGTGAAACCTCATTTAGTAA	COQ10B__NM_025147	576	624	537	481
COQ10B__NM_025147_GTATGATGTAGTATCGGGAG	COQ10B__NM_025147	259	211	343	433
COQ10B__NM_025147_GTCCAGAAACAAATATACCT	COQ10B__NM_025147	488	550	496	402
COQ10B__NM_025147_TCCCGAGACTACCCTTCTCA	COQ10B__NM_025147	910	761	1052	865
COQ10B__NM_025147_TGATGTAGTATCGGGAGTGG	COQ10B__NM_025147	584	860	689	712
COQ10B__NM_025147_TGTAGGCATCTTGTACTGAT	COQ10B__NM_025147	505	498	513	507
COQ2__NM_015697_AACCGCATGAGGCGCAAGTA	COQ2__NM_015697	368	186	460	149
COQ2__NM_015697_AGACTAGGATTGCATCCTGC	COQ2__NM_015697	430	393	372	397
COQ2__NM_015697_AGCCGTACTTGCGCCTCATG	COQ2__NM_015697	1000	416	1114	272
COQ2__NM_015697_CAATTTCACAAGTAAGGATG	COQ2__NM_015697	167	98	157	197
COQ2__NM_015697_CCAGCTCAGTTTGTCCGCGG	COQ2__NM_015697	1424	767	1787	925
COQ2__NM_015697_CCGCCGCGGACAAACTGAGC	COQ2__NM_015697	698	397	795	353
COQ2__NM_015697_GATTGACGTCATTCCCCGGC	COQ2__NM_015697	910	1078	953	1183
COQ2__NM_015697_GCGCCAGCTCAGTTTGTCCG	COQ2__NM_015697	3730	3375	4168	4613
COQ2__NM_015697_GGATGCAATCCTAGTCTGCC	COQ2__NM_015697	616	671	633	599
COQ2__NM_015697_GGGCTGCAAGTCACCACCGT	COQ2__NM_015697	1036	563	1094	732
COQ2__NM_015697_TGACCCCAATTTCACAAGTA	COQ2__NM_015697	265	177	383	346
COQ2__NM_015697_TGGGCTTGTCCAACCGCATG	COQ2__NM_015697	44	10	76	20
COQ3__NM_017421_AACCACTGTCGACAGCGGTG	COQ3__NM_017421	45	25	25	41
COQ3__NM_017421_ACAGGAAAAGATCGTCCTGT	COQ3__NM_017421	886	1153	972	1071
COQ3__NM_017421_ACCTGAGGGTGCCATTTATT	COQ3__NM_017421	976	622	1149	557
COQ3__NM_017421_AGCCAGGAAAACCTTTGTTG	COQ3__NM_017421	922	708	869	588
COQ3__NM_017421_ATCCCACCATTTGTGAGCCA	COQ3__NM_017421	553	614	645	527
COQ3__NM_017421_ATGTGGAGTGGCCGTAAGCT	COQ3__NM_017421	856	407	924	428
COQ3__NM_017421_CATCCCACCATTTGTGAGCC	COQ3__NM_017421	959	1025	1022	1453
COQ3__NM_017421_CCATATGGTTCAAATCCTAC	COQ3__NM_017421	481	383	497	363
COQ3__NM_017421_GATGTGGAGTGGCCGTAAGC	COQ3__NM_017421	359	281	459	210
COQ3__NM_017421_GGACTCTACAGATTAAACCA	COQ3__NM_017421	195	78	225	85
COQ3__NM_017421_GGGACTCTACAGATTAAACC	COQ3__NM_017421	372	319	296	294
COQ3__NM_017421_TCCCAAACCACTGTCGACAG	COQ3__NM_017421	855	421	923	399
COQ4__NM_016035_AACGGAGATACTCGCGACCG	COQ4__NM_016035	667	113	717	64
COQ4__NM_016035_ACGGAGATACTCGCGACCGA	COQ4__NM_016035	570	141	721	69
COQ4__NM_016035_AGGGTGGATGTCGAAATCCG	COQ4__NM_016035	200	260	192	334
COQ4__NM_016035_CCCCTCCGGGCTAGGAGCGA	COQ4__NM_016035	33	16	45	96
COQ4__NM_016035_CGAGGGTGGATGTCGAAATC	COQ4__NM_016035	675	922	702	923
COQ4__NM_016035_CGTATGTGATTCAGCGGTAC	COQ4__NM_016035	504	205	523	240
COQ4__NM_016035_GAGGGTGGATGTCGAAATCC	COQ4__NM_016035	94	236	221	284
COQ4__NM_016035_GGGAGGTGGTGCGAGTATAG	COQ4__NM_016035	52	18	50	45
COQ4__NM_016035_GTGGTGCGAGTATAGCGGGC	COQ4__NM_016035	3	0	1	7
COQ4__NM_016035_TCGACATCCACCCTCGACCT	COQ4__NM_016035	1527	719	1687	1501
COQ4__NM_016035_TGCTGCGCCCTGTCCTCCGT	COQ4__NM_016035	579	236	551	389
COQ4__NM_016035_TTCGACATCCACCCTCGACC	COQ4__NM_016035	183	169	244	252
COQ5__NM_032314_ACAAGAGCCGAGCACTTAGT	COQ5__NM_032314	344	186	357	164
COQ5__NM_032314_ACTCTCTACAAGGTACTGAT	COQ5__NM_032314	106	50	117	49
COQ5__NM_032314_ATATGAGGGGATTGTTCACT	COQ5__NM_032314	699	722	708	258
COQ5__NM_032314_CATCGGGTGCTGAAACCAGG	COQ5__NM_032314	508	91	383	45
COQ5__NM_032314_CCACAGGTGACATTGCATTC	COQ5__NM_032314	400	87	462	77
COQ5__NM_032314_CCGGGGACCTACTAAGTGCT	COQ5__NM_032314	2591	2095	2719	1938
COQ5__NM_032314_CGGGCAGCGGAAACGCACTT	COQ5__NM_032314	844	972	1045	692
COQ5__NM_032314_CTTGGGCGGGTCTCGTGTCG	COQ5__NM_032314	1856	1916	2104	1844
COQ5__NM_032314_GCCAAGAGCTACGAAGCCCG	COQ5__NM_032314	597	419	636	302
COQ5__NM_032314_TATGGAGCTATTGCGGCCGT	COQ5__NM_032314	1214	965	1310	619
COQ5__NM_032314_TGAACAATCCCCTCATATCC	COQ5__NM_032314	443	175	412	94
COQ5__NM_032314_TGGTATCCATCGTGTTTGGA	COQ5__NM_032314	26	6	28	57
COQ6__NM_182476_AAAATCCTGTTGCTCGAAGC	COQ6__NM_182476	134	306	207	211
COQ6__NM_182476_ACAAGGCCAGGTATAGCGAA	COQ6__NM_182476	5	13	26	10
COQ6__NM_182476_AGACACCGTGTATGACGTGG	COQ6__NM_182476	930	684	974	381
COQ6__NM_182476_ATGGTCACAACTCCGGAGTA	COQ6__NM_182476	387	298	451	165
COQ6__NM_182476_CCGGCTTGTCAGCCGATGCG	COQ6__NM_182476	4	17	18	12
COQ6__NM_182476_CCTAGGGTAATATGAACCCA	COQ6__NM_182476	767	698	808	473
COQ6__NM_182476_CTGCACGCACAGCCCCGCAT	COQ6__NM_182476	550	354	757	420
COQ6__NM_182476_GACGTGGTGGTGTCGGGTGG	COQ6__NM_182476	793	171	837	223
COQ6__NM_182476_GCAAAGCCATTCGCTATACC	COQ6__NM_182476	268	429	275	553
COQ6__NM_182476_GTTCATATTACCCTAGGTGA	COQ6__NM_182476	11	0	10	20
COQ6__NM_182476_TGTATCTCATGTTGCAGATA	COQ6__NM_182476	70	72	132	94
COQ6__NM_182476_TGTCAGAAACTTACAGCAAC	COQ6__NM_182476	93	116	120	75
COQ6__NM_182480_TCACAACTCCGGAGTACGGC	COQ6__NM_182480	351	220	363	237
COQ7__NM_001190983_AAACCCCAGCACGTTCCACA	COQ7__NM_001190983	1029	488	1015	474
COQ7__NM_001190983_ACCTTCCTTCCCGAGCAAGG	COQ7__NM_001190983	178	54	195	82
COQ7__NM_001190983_ATGAGTTGATGGTTACGTTC	COQ7__NM_001190983	460	604	516	766
COQ7__NM_001190983_CAGCTGTGGATCGAATAATC	COQ7__NM_001190983	320	148	361	184
COQ7__NM_001190983_CTCTTCCACCGCCACGGTGC	COQ7__NM_001190983	101	86	93	86
COQ7__NM_001190983_GAAAAAGTTCAATGAGTTGA	COQ7__NM_001190983	436	736	531	547
COQ7__NM_001190983_GACTTTAGACAATATCAGTC	COQ7__NM_001190983	254	232	301	345
COQ7__NM_001190983_GTCCTGGGTCGGACCAGCGT	COQ7__NM_001190983	230	226	237	180
COQ7__NM_001190983_GTGGATCATGCAGGCGAATA	COQ7__NM_001190983	0	0	0	0
COQ7__NM_001190983_TGAGTTGATGGTTACGTTCA	COQ7__NM_001190983	248	560	379	497
COQ7__NM_001190983_TGCCCTTGTGGAACGTGCTG	COQ7__NM_001190983	537	503	711	549
COQ7__NM_001190983_TGTGGATCGAATAATCCGGG	COQ7__NM_001190983	424	199	367	179
COQ9__NM_020312_ATGGCGGCGGCGGCGGTATC	COQ9__NM_020312	434	345	492	424
COQ9__NM_020312_CAAGGGCTGCCGTCAGGATG	COQ9__NM_020312	191	91	220	112
COQ9__NM_020312_CACCAGGGCTTGTCGGCAAC	COQ9__NM_020312	688	758	833	776
COQ9__NM_020312_CGGGGCAGTGCTCAATGTA	COQ9__NM_020312	526	641	622	672
COQ9__NM_020312_CGGTATCTGGTGCGCTTGGC	COQ9__NM_020312	191	247	212	143
COQ9__NM_020312_CTCTGCTGTCCACCCGTGGG	COQ9__NM_020312	225	281	328	322
COQ9__NM_020312_GCGGCGGTATCTGGTGCGCT	COQ9__NM_020312	1100	984	1073	1152
COQ9__NM_020312_GCTCACACGTGTGCTAGAAG	COQ9__NM_020312	599	334	636	690
COQ9__NM_020312_GGAGGAGGACTATGAAAGTG	COQ9__NM_020312	219	202	267	313
COQ9__NM_020312_GTGTGAGCCGGGTATTGCAC	COQ9__NM_020312	913	1199	964	1048
COQ9__NM_020312_TGATCCCATACATTGAGCAC	COQ9__NM_020312	298	182	364	337
COQ9__NM_020312_TTGTGACCCAGTGCAATACC	COQ9__NM_020312	430	577	549	388
CORIN__NM_001278585_ACCATCACATTGAAACGTGC	CORIN__NM_001278585	1425	1807	1693	2309
CORIN__NM_001278585_AGGAAAACGGAAAGCAATGT	CORIN__NM_001278585	474	549	428	652
CORIN__NM_001278585_CACAGAAGGACCTACAGGGC	CORIN__NM_001278585	538	477	487	696
CORIN__NM_001278585_CAGAGCCGGGTCCCCAAAGC	CORIN__NM_001278585	508	611	557	677
CORIN__NM_001278585_CCATCACATTGAAACGTGCT	CORIN__NM_001278585	3298	3850	4028	4260
CORIN__NM_001278585_GAGCACAGATACATGGAATC	CORIN__NM_001278585	274	266	337	303
CORIN__NM_001278585_GATGGTGAATTACTCCTGGC	CORIN__NM_001278585	525	902	620	774
CORIN__NM_001278585_GCCTCACAGAAGGACCTACA	CORIN__NM_001278585	181	273	209	229
CORIN__NM_001278585_GCTGTGGGTGATGTTCATAC	CORIN__NM_001278585	584	392	468	551
CORIN__NM_001278585_GGAGAGGTGTCAGCGTGGCG	CORIN__NM_001278585	757	1044	905	1397
CORIN__NM_001278585_GTGATCCTGCTTTCCTATGT	CORIN__NM_001278585	772	752	984	971
CORIN__NM_001278585_TCCTGCTTTCCTATGTTGGT	CORIN__NM_001278585	751	1006	724	1078
CORIN__NM_001278586_CCAGGCTGGAACGTGTTGGT	CORIN__NM_001278586	2117	2520	2306	2767
CORIN__NM_001278586_GAGAGAAGCATCCGTAGTCC	CORIN__NM_001278586	774	965	993	1015
CORIN__NM_001278586_TCACTGATGGTGAAATCCAA	CORIN__NM_001278586	346	435	420	447
CORO1A__NM_001193333_CACGCGCTTGTCACGGCAGG	CORO1A__NM_001193333	1286	1212	1320	1339
CORO1A__NM_001193333_CCAGGCGATGTCTAGCACAG	CORO1A__NM_001193333	434	407	480	417
CORO1A__NM_001193333_CCCCTGTGCTAGACATCGCC	CORO1A__NM_001193333	1806	1992	1784	2277
CORO1A__NM_001193333_CCCGCTGGCCTCACAGATCA	CORO1A__NM_001193333	292	341	355	528
CORO1A__NM_001193333_CGTGCGTGCAGTGTTCGTGT	CORO1A__NM_001193333	3720	3855	3779	3700
CORO1A__NM_001193333_CTGCACTGAGCAGCACGTTC	CORO1A__NM_001193333	122	125	145	170
CORO1A__NM_001193333_GACACGAACACTGCACGCAC	CORO1A__NM_001193333	531	448	558	501
CORO1A__NM_001193333_GCTACGACAGTGCCTTTGCG	CORO1A__NM_001193333	1089	1264	1144	1335
CORO1A__NM_001193333_TCCACACTGTAGATCGTGTC	CORO1A__NM_001193333	695	799	747	1010
CORO1A__NM_001193333_TGATGCGCACGCGCTTGTCA	CORO1A__NM_001193333	327	206	338	298
CORO1A__NM_001193333_TGGCCGGCTGTCCAAACACG	CORO1A__NM_001193333	77	58	77	71
CORO1A__NM_001193333_TGTGGACAAGAATGCGCCCA	CORO1A__NM_001193333	367	354	356	274
CORO1B__NM_001018070_AAGGCCTACCCGACGGTGTG	CORO1B__NM_001018070	351	433	383	405
CORO1B__NM_001018070_AGCAAATTCGGGCATGTGTT	CORO1B__NM_001018070	864	739	879	1075
CORO1B__NM_001018070_AGGCCTACCCGACGGTGTGT	CORO1B__NM_001018070	1104	1212	1115	916
CORO1B__NM_001018070_AGGTGCTGTCCCAGGTAACA	CORO1B__NM_001018070	228	127	227	165
CORO1B__NM_001018070_CATTGACAAGGCCTACCCGA	CORO1B__NM_001018070	886	762	849	641
CORO1B__NM_001018070_CCGTGTGCCCACACACCGTC	CORO1B__NM_001018070	631	733	585	679
CORO1B__NM_001018070_CTGCCGGACCACTTTGCGGA	CORO1B__NM_001018070	1151	1300	1343	1354
CORO1B__NM_001018070_GTGCTCCCCCTAAGCAAGGT	CORO1B__NM_001018070	12	15	32	28
CORO1B__NM_001018070_TAGCACTGGTCGTTCTTGAC	CORO1B__NM_001018070	225	160	231	148
CORO1B__NM_001018070_TCGCGTGTCCCGTGTTACCT	CORO1B__NM_001018070	1569	762	1513	1481
CORO1B__NM_001018070_TCGGGTAGGCCTTGTCAATG	CORO1B__NM_001018070	738	594	921	987
CORO1B__NM_001018070_TTCGCGTGTCCCGTGTTACC	CORO1B__NM_001018070	206	134	234	220
CORO1C__NM_001105237_AAAGTCTGGCAATAACAGCT	CORO1C__NM_001105237	260	314	282	362
CORO1C__NM_001105237_ACTTTCACACATGCGTTGGA	CORO1C__NM_001105237	606	797	888	818
CORO1C__NM_001105237_AGCAAGTTTCGGCATGTATT	CORO1C__NM_001105237	621	936	760	1062
CORO1C__NM_001105237_CCATAATCATAGAGGCAAGT	CORO1C__NM_001105237	531	668	795	904
CORO1C__NM_001105237_CCCACTTGCCTCTATGATTA	CORO1C__NM_001105237	1423	1799	1674	1748
CORO1C__NM_001105237_CCGGGTTTCTCGTGTGACCT	CORO1C__NM_001105237	1082	1245	1244	1467
CORO1C__NM_001105237_CTGTGTGGCCACATACTGTA	CORO1C__NM_001105237	503	304	459	384
CORO1C__NM_001105237_GAAAGTCTGGCAATAACAGC	CORO1C__NM_001105237	208	241	246	180
CORO1C__NM_001105237_GGCCACTTTCACACATGCGT	CORO1C__NM_001105237	534	480	485	683
CORO1C__NM_001105237_TATGATTATGGCAACAAATC	CORO1C__NM_001105237	43	62	56	47
CORO1C__NM_001105237_TATGGCCCTGGTAGTCAGCT	CORO1C__NM_001105237	758	620	719	652
CORO1C__NM_001105237_TCCGGGTTTCTCGTGTGACC	CORO1C__NM_001105237	460	509	436	607
CORO1C__NM_001276471_CAATGACCTGATCGTTATGT	CORO1C__NM_001276471	2181	1977	2389	1672
CORO1C__NM_001276471_CCTGTGTGCCCACATACTGT	CORO1C__NM_001276471	216	256	180	211
CORO1C__NM_001276471_CTCAAAGAGAGTCGGCATCG	CORO1C__NM_001276471	451	354	488	485
CORO1C__NM_001276471_CTGGTGCCCACATAACGATC	CORO1C__NM_001276471	339	150	431	259
CORO1C__NM_001276471_GAAGGCCACTCAAAGAGAGT	CORO1C__NM_001276471	264	270	304	194
CORO2A__NM_003389_AACTTGGAGCTCCGGTACTG	CORO2A__NM_003389	469	309	698	378
CORO2A__NM_003389_AAGTTCCTGAGTCAACAGAA	CORO2A__NM_003389	109	134	93	105
CORO2A__NM_003389_ACTTGAGATTGTATCATAGA	CORO2A__NM_003389	351	231	420	515
CORO2A__NM_003389_ACTTGGAGCTCCGGTACTGG	CORO2A__NM_003389	1209	650	1273	1071
CORO2A__NM_003389_AGATTCAGCTTCAGTTGGTC	CORO2A__NM_003389	539	632	597	629
CORO2A__NM_003389_AGGAGGCCACCTAGTCTGGT	CORO2A__NM_003389	1134	926	1399	1440
CORO2A__NM_003389_AGGTCTTTGATTCTGAACAT	CORO2A__NM_003389	271	313	381	353
CORO2A__NM_003389_AGTTTCTTCATCTGAACCAG	CORO2A__NM_003389	36	68	37	28
CORO2A__NM_003389_CAATTATTGTAGCGTTAAGG	CORO2A__NM_003389	238	267	310	284
CORO2A__NM_003389_CAGGCAGGACTTAAGAGATC	CORO2A__NM_003389	263	310	255	169
CORO2A__NM_003389_CATTGACTGCCAAGCGGTAG	CORO2A__NM_003389	70	42	69	29
CORO2A__NM_003389_CCAGGAACCTCACGGCCTAC	CORO2A__NM_003389	945	579	1071	951
CORO2A__NM_003389_CGGCGCTCACCTCGTAGTAG	CORO2A__NM_003389	378	330	423	402
CORO2A__NM_003389_CTAAGAGAAAACTACCTGTG	CORO2A__NM_003389	356	296	371	468
CORO2A__NM_003389_CTCGGAACACAGATTCACTA	CORO2A__NM_003389	296	289	392	443
CORO2A__NM_003389_CTGGTGTCCGCGTCATAGAA	CORO2A__NM_003389	262	385	319	403
CORO2A__NM_003389_GAAAAGCTTTCAGGCTAAAA	CORO2A__NM_003389	156	411	211	212
CORO2A__NM_003389_GAACTTGGAGCTCCGGTACT	CORO2A__NM_003389	934	790	1041	817
CORO2A__NM_003389_GCAACCCCACCTCTACCGCT	CORO2A__NM_003389	2374	1949	2732	2523
CORO2A__NM_003389_GCACAATTATTGTAGCGTTA	CORO2A__NM_003389	1118	1082	1384	1662
CORO2A__NM_003389_GCCAAATCCCAGGCATTGTC	CORO2A__NM_003389	459	575	424	500
CORO2A__NM_003389_GGAACTTGCCTAGCACATTC	CORO2A__NM_003389	750	980	712	845
CORO2A__NM_003389_GGACACCACTCGGCTCCATG	CORO2A__NM_003389	1462	1276	1530	1590
CORO2A__NM_003389_GGACAGCATCTATCCATATC	CORO2A__NM_003389	836	562	874	1122
CORO2A__NM_003389_GGCCCGGTGCCCTTTGTAGC	CORO2A__NM_003389	326	373	397	542
CORO2A__NM_003389_GGTCAATAACCCGAATCTTG	CORO2A__NM_003389	447	458	499	509
CORO2A__NM_003389_GTGACAGCTAATCGTACTCA	CORO2A__NM_003389	549	733	746	747
CORO2A__NM_003389_GTGGTGGCAGTCAGCAGTCA	CORO2A__NM_003389	79	35	134	77
CORO2A__NM_003389_GTTCCTTCCTGTAGGCCGTG	CORO2A__NM_003389	315	398	386	404
CORO2A__NM_003389_TAGGGGAAGACTAAGCAGGA	CORO2A__NM_003389	92	167	123	114
CORO2A__NM_003389_TATGGTCATATAAGCTCCAC	CORO2A__NM_003389	304	405	393	464
CORO2A__NM_003389_TCAGGGCAGCAGAGCTCTAC	CORO2A__NM_003389	0	0	0	0
CORO2A__NM_003389_TCTCAGCCTCCCGAATAGCA	CORO2A__NM_003389	51	0	42	0

CORO2A__NM_003389_TCTCCATTTACTCAGTCACT	CORO2A__NM_003389	583	434	538	606
CORO2A__NM_003389_TCTTCTGAAATGACTATACG	CORO2A__NM_003389	155	112	190	238
CORO2A__NM_003389_TGACAGCTAATCGTACTCAT	CORO2A__NM_003389	591	376	654	666
CORO2A__NM_003389_TGCACAGGGCGATTAGATTC	CORO2A__NM_003389	496	361	674	675
CORO2A__NM_003389_TGCAGTGCAGTCCGCAATCG	CORO2A__NM_003389	1269	870	1186	1075
CORO2A__NM_003389_TGGAGCTTATATGACCATAC	CORO2A__NM_003389	108	46	83	84
CORO2A__NM_003389_TGGCAACATCCGCTACTACG	CORO2A__NM_003389	112	141	142	117
CORO2A__NM_003389_TGTAGTCCCTGCTATTCGGG	CORO2A__NM_003389	17	0	29	0
CORO2A__NM_003389_TTCTCCATTTACTCAGTCAC	CORO2A__NM_003389	20	0	22	17
CORO2B__NM_001190456_AACTTGGAGCTACGGTATTG	CORO2B__NM_001190456	552	454	584	578
CORO2B__NM_001190456_CCACCTTCCCGTAGACATTC	CORO2B__NM_001190456	643	970	581	696
CORO2B__NM_001190456_CCCGTAGACATTCCGGAACT	CORO2B__NM_001190456	883	775	964	742
CORO2B__NM_001190456_CCCTGCGCTCTCGGTGACGA	CORO2B__NM_001190456	1077	1421	1361	1970
CORO2B__NM_001190456_CCGCAGACCTTGGGGTAGTT	CORO2B__NM_001190456	1374	1846	1681	2142
CORO2B__NM_001190456_CCTGGTGGCCGCAGACCTTG	CORO2B__NM_001190456	188	69	225	239
CORO2B__NM_001190456_GATTGAACCCAACTACCCCA	CORO2B__NM_001190456	115	115	105	98
CORO2B__NM_001190456_GCAATGATGTTGTCGATGAA	CORO2B__NM_001190456	621	808	685	809
CORO2B__NM_001190456_GCAATGTGCTGGATATCAAA	CORO2B__NM_001190456	370	360	342	319
CORO2B__NM_001190456_GCTACGGTATTGCGGACGCC	CORO2B__NM_001190456	439	948	466	669
CORO2B__NM_001190456_GTTCCGGAATGTCTACGGGA	CORO2B__NM_001190456	227	203	255	257
CORO2B__NM_001190456_TCCAAGTTCCGGAATGTCTA	CORO2B__NM_001190456	221	139	326	323
CORO6__NM_032854_AGCAAGTTCCGCCATGTGTT	CORO6__NM_032854	1047	1670	1342	1484
CORO6__NM_032854_AGGTTCCGTAATGTTGCGCA	CORO6__NM_032854	148	279	166	181
CORO6__NM_032854_AGTGGATAAGAACTACCCAC	CORO6__NM_032854	145	114	152	102
CORO6__NM_032854_CAAATTCCTGGCCATTATTG	CORO6__NM_032854	418	285	473	332
CORO6__NM_032854_GAGGGCCACTCCAAGCGTGT	CORO6__NM_032854	655	393	549	270
CORO6__NM_032854_GCCTCCAGCCTCCACAATAA	CORO6__NM_032854	319	186	236	197
CORO6__NM_032854_GGTTCCGTAATGTTGCGCAT	CORO6__NM_032854	256	143	191	149
CORO6__NM_032854_GTTCCGTAATGTTGCGCATG	CORO6__NM_032854	276	379	302	354
CORO6__NM_032854_TACCCCCATGCGCAACATTA	CORO6__NM_032854	1035	969	1134	897
CORO6__NM_032854_TCTGGGGTCAATGATGCGCA	CORO6__NM_032854	355	465	386	530
CORO6__NM_032854_TGGCCCTCAAGTGTGATGAT	CORO6__NM_032854	55	76	25	41
CORO6__NM_032854_TTGCGCATGGGGGTATAGTC	CORO6__NM_032854	129	79	194	168
CORO7__NM_001201472_ACCAGAATGCCGTCAGAGGT	CORO7__NM_001201472	1160	666	1457	909
CORO7__NM_001201472_AGGAGAGGACAAGCGACGCG	CORO7__NM_001201472	333	315	384	299
CORO7__NM_001201472_CAACTCCGACCGTCCTGGTA	CORO7__NM_001201472	2386	1500	2728	1537
CORO7__NM_001201472_CCAGAATGCCGTCAGAGGTG	CORO7__NM_001201472	16	127	53	3
CORO7__NM_001201472_GAACAGCAGGGATAGCCGGC	CORO7__NM_001201472	1256	963	1347	1106
CORO7__NM_001201472_GCCTTCAACTCCGACCGTCC	CORO7__NM_001201472	997	719	1041	1070
CORO7__NM_001201472_GCTCACCAGAATGCCGTCAG	CORO7__NM_001201472	366	303	369	305
CORO7__NM_001201472_TCAACTCCGACCGTCCTGGT	CORO7__NM_001201472	1019	875	1052	1048
CORO7__NM_001201472_TCTACTGGATTCAACCAGGT	CORO7__NM_001201472	189	195	160	175
CORO7__NM_001201472_TGCTCGAATGTCACTGATCC	CORO7__NM_001201472	364	471	427	334
CORO7__NM_001201472_TGGATCAGTGACATTCGAGC	CORO7__NM_001201472	65	84	109	53
CORO7__NM_001201472_TGGGAGCACCTTGTGTCTAC	CORO7__NM_001201472	308	290	356	302
CORO7__NM_001201473_CTCACCTTGGACACCTCGCT	CORO7__NM_001201473	1140	1580	1327	1667
CORO7__NM_001201473_CTTGGACACCTCGCTTGGGT	CORO7__NM_001201473	968	698	1130	675
CORO7__NM_001201473_TCACCTTGGACACCTCGCTT	CORO7__NM_001201473	1653	1287	1737	1496
CORO7-PAM16__NM_001201479_AGAAGTCCAAGTCGGTG	CORO7-PAM16__NM_001201479	381	234	432	338
CORO7-PAM16__NM_001201479_CAAGTCGGTGACTAGGTC	CORO7-PAM16__NM_001201479	200	238	271	290
CORT__NM_001302_AGCAGGAGGCCGGGGGACAA	CORT__NM_001302	898	620	916	569
COTL1__NM_021149_AGGAGTTTGTGATCAGTGAT	COTL1__NM_021149	194	303	279	323
COTL1__NM_021149_CCAAGTTTGCCCTCATCACG	COTL1__NM_021149	1341	1544	1428	1843
COTL1__NM_021149_CCTGGTGCGCGACGACGGCT	COTL1__NM_021149	1103	1261	1164	1278
COTL1__NM_021149_CTCCGTCTGGGCGTCGTAAT	COTL1__NM_021149	439	310	457	503
COTL1__NM_021149_GATCGACAAAGAGGCTTGCC	COTL1__NM_021149	103	119	139	244
COTL1__NM_021149_GCAAGCCTCTTTGTCGATCT	COTL1__NM_021149	1125	1508	1161	1353
COTL1__NM_021149_GGATCGGTGAGAACGTCAGC	COTL1__NM_021149	317	297	328	299
COTL1__NM_021149_GGCCACCAAGATCGACAAAG	COTL1__NM_021149	257	495	388	501
COTL1__NM_021149_TACAACCTGGTGCGCGACGA	COTL1__NM_021149	216	190	231	222
COX10__NM_001303_AGAAGATGTAAGAACTTGTG	COX10__NM_001303	97	81	98	69
COX10__NM_001303_CAGCTCAGGGCGTTGAAATG	COX10__NM_001303	959	50	1135	46
COX10__NM_001303_CATTTGACTCAAACATGAAT	COX10__NM_001303	204	227	217	337
COX10__NM_001303_CCCGGGAGTAGTCTTCACGG	COX10__NM_001303	721	7	838	48
COX10__NM_001303_CGCCCCGGGAGTAGTCTTCA	COX10__NM_001303	1350	585	1518	776
COX10__NM_001303_CTGCAAGTGTATGATTTGCC	COX10__NM_001303	860	1352	1011	1369
COX10__NM_001303_CTGTCCCAACAGAAGTAAGC	COX10__NM_001303	125	6	94	46
COX10__NM_001303_GAGTCTTCAATTACTGAGTC	COX10__NM_001303	206	344	278	303
COX10__NM_001303_GGAAACAGGGCCAGTCAAAA	COX10__NM_001303	205	52	144	34
COX10__NM_001303_GTCCAGAAAGCCAAATGAAA	COX10__NM_001303	168	111	170	114
COX10__NM_001303_TAGGAGATGTACGCATTGAT	COX10__NM_001303	368	11	431	4
COX10__NM_001303_TTGAAGACTCAATAGATGTA	COX10__NM_001303	70	95	76	28
COX11__NM_001162861_AATATTGTTCCATTTGAAGC	COX11__NM_001162861	268	205	308	305
COX11__NM_001162861_AGCCGTTTCTTAGGCCAGAG	COX11__NM_001162861	274	159	339	275
COX11__NM_001162861_ATAAAGGGGTACGGCAGCGT	COX11__NM_001162861	602	4	401	3
COX11__NM_001162861_CAAGACGACCCTCACTTACG	COX11__NM_001162861	2478	6	2398	46
COX11__NM_001162861_CCTACTGACAAACCAGTAAT	COX11__NM_001162861	624	144	924	169
COX11__NM_001162861_CCTGGCAATAGAGCCGATAA	COX11__NM_001162861	455	34	447	55
COX11__NM_001162861_CGACAGCGGCCACGTAAGTG	COX11__NM_001162861	96	0	79	13
COX11__NM_001162861_CTCCACTCTGGCCTAAGAAA	COX11__NM_001162861	155	139	214	106
COX11__NM_001162861_CTGGCAATAGAGCCGATAAA	COX11__NM_001162861	156	6	188	1
COX11__NM_001162861_GCTGAGGTCTAAAGTTCCAC	COX11__NM_001162861	339	1	322	5
COX11__NM_001162861_GGGACAGGAGGTGCCGAGAG	COX11__NM_001162861	698	127	632	219
COX11__NM_001162861_TTCGAGCTCTTAGGCCGCCG	COX11__NM_001162861	0	0	7	5
COX14__NM_001257133_ATGATGCTTCTCACTGTGTA	COX14__NM_001257133	489	707	461	599
COX14__NM_001257133_CCCTGCGCCACTGGAAATAG	COX14__NM_001257133	774	930	867	802
COX15__NM_004376_ATTGCTCTTTCCGCCGTTGA	COX15__NM_004376	393	545	414	465
COX15__NM_004376_CGAATGTGGGGTCGCCTTGT	COX15__NM_004376	2540	144	3011	248
COX15__NM_004376_CTACAAGGCGACCCCACATT	COX15__NM_004376	440	13	477	5
COX15__NM_004376_GAGTACTCACACCGAATGTG	COX15__NM_004376	232	6	262	20
COX15__NM_004376_GATTGCTCTTTCCGCCGTTG	COX15__NM_004376	271	257	340	397
COX15__NM_004376_GCTGGAGCAGTTATTCTTGG	COX15__NM_004376	1545	1195	1611	1390
COX15__NM_004376_GGAGTACTCACACCGAATGT	COX15__NM_004376	541	36	533	31
COX15__NM_004376_GGGTCTGTTGGGATGGTATA	COX15__NM_004376	409	117	391	152
COX15__NM_004376_GTAGCTTTGCAATCTGGAAG	COX15__NM_004376	140	72	133	108
COX15__NM_004376_TAGGGCAGCGCCTAGAGCAC	COX15__NM_004376	156	107	222	47
COX15__NM_004376_TCCCATTCCTCTTGGCTTGT	COX15__NM_004376	842	847	917	1084
COX15__NM_004376_TGTCATATCATGATTCAAGC	COX15__NM_004376	74	61	112	71
COX16__NM_001204090_AAGAACAAGACTCTCGGCTA	COX16__NM_001204090	317	103	275	127
COX17__NM_005694_ACACTGTGGACATCTAATTG	COX17__NM_005694	775	337	767	332
COX17__NM_005694_CCAAGAAGGCGCGCGATGCG	COX17__NM_005694	847	371	871	462
COX17__NM_005694_GGCTCTCATGCATTCCTTGT	COX17__NM_005694	458	634	579	832
COX17__NM_005694_GGGTTTGAGTCAACCAGACC	COX17__NM_005694	176	46	155	116
COX18__NM_173827_AACTTCTTGGTTAAGATGCC	COX18__NM_173827	100	104	110	197
COX18__NM_173827_ACGGGGGCAGCACATTCAGA	COX18__NM_173827	966	556	958	661
COX18__NM_173827_ACTGCCCACACTGGGAGAGT	COX18__NM_173827	1019	888	946	833
COX18__NM_173827_CTCTCCACCGTGGCCTTACG	COX18__NM_173827	145	91	127	145
COX18__NM_173827_CTCTCCGGAATTTAAGCACG	COX18__NM_173827	202	136	263	207
COX18__NM_173827_CTGCCCCCGTGCTTAAATTC	COX18__NM_173827	592	549	702	520
COX18__NM_173827_GCTCTCCGGAATTTAAGCAC	COX18__NM_173827	1157	946	1268	967
COX18__NM_173827_GTTCAGGAACAGTTAGCTAC	COX18__NM_173827	105	53	61	30
COX18__NM_173827_TCAAAGCCACTGTGTTGGTT	COX18__NM_173827	655	634	680	799
COX18__NM_173827_TGCAGTTCGTGCAAATCAGT	COX18__NM_173827	307	509	389	398
COX18__NM_173827_TGGATTCTGCCTATCTCTGT	COX18__NM_173827	12	4	25	19
COX18__NM_173827_TGGCCGCGTCTTCGCCGGTG	COX18__NM_173827	149	123	161	114
COX19__NM_001031617_ATGTCGACCGCCATGAATTT	COX19__NM_001031617	549	600	756	682
COX19__NM_001031617_ATTCATGGCGGTCGACATGT	COX19__NM_001031617	825	713	786	829
COX19__NM_001031617_ATTGATGCTACAAGAACCAT	COX19__NM_001031617	116	25	140	76
COX19__NM_001031617_GCTCTTGGTCCCGAAATTCA	COX19__NM_001031617	455	321	578	282
COX19__NM_001031617_GGATTTGGAGACTTGACTAG	COX19__NM_001031617	248	403	359	328
COX20__NM_198076_AACCATACAATATTGAATGC	COX20__NM_198076	323	28	264	38
COX20__NM_198076_ACCATACAATATTGAATGCC	COX20__NM_198076	537	143	658	254
COX20__NM_198076_ATTAGAAGATCATGTGATGT	COX20__NM_198076	385	380	361	511
COX20__NM_198076_CAATATTGAATGCCGGGCGC	COX20__NM_198076	455	103	495	193
COX20__NM_198076_CATGTGATGTTGGAGTAGGA	COX20__NM_198076	614	308	717	260
COX20__NM_198076_TAAAAATCCTAGGAGCTTAA	COX20__NM_198076	134	147	103	147
COX20__NM_198076_TGGAGTAGGAGGGTTTATCT	COX20__NM_198076	134	10	168	28
COX20__NM_198076_TGGTTCATTAGGATCTGTTG	COX20__NM_198076	4	0	0	0
COX4I1__NM_001861_AACGAGTGGAAGACGGTTGT	COX4I1__NM_001861	928	54	935	73
COX4I1__NM_001861_ACATGGGCCACCTCCGGCAA	COX4I1__NM_001861	676	241	899	324
COX4I1__NM_001861_ACCAGGGTATTTAGCCTAGT	COX4I1__NM_001861	648	245	685	276
COX4I1__NM_001861_AGTGTATCGCATTAAGTTCA	COX4I1__NM_001861	829	47	984	112
COX4I1__NM_001861_CACGCCGATCCATATAAGCT	COX4I1__NM_001861	931	263	945	270
COX4I1__NM_001861_CGCTCCCAGCTTATATGGAT	COX4I1__NM_001861	1161	1212	1269	1578
COX4I1__NM_001861_GAACTTAATGCGATACACTG	COX4I1__NM_001861	362	0	386	4
COX4I1__NM_001861_GAGCTTTGCTGAGATGAACA	COX4I1__NM_001861	117	52	178	130
COX4I1__NM_001861_TCACGCCGATCCATATAAGC	COX4I1__NM_001861	276	110	347	68
COX4I1__NM_001861_TGAACTTAATGCGATACACT	COX4I1__NM_001861	195	43	206	121
COX4I1__NM_001861_TGGATGAGAAAGTCGAGTGT	COX4I1__NM_001861	152	6	143	2
COX4I1__NM_001861_TTGAACTTAATGCGATACAC	COX4I1__NM_001861	213	214	228	214
COX4I2__NM_032609_CGATCACCTTGACGGACGAG	COX4I2__NM_032609	1000	1543	1350	1599
COX4I2__NM_032609_CTTCATTGGATTCGCAGCTC	COX4I2__NM_032609	5	4	9	12
COX4I2__NM_032609_GATTCGCAGCTCTGGTGATT	COX4I2__NM_032609	894	900	972	1195
COX4I2__NM_032609_GGCTTTCCGCTCGTCCGTCA	COX4I2__NM_032609	1292	1130	1289	1574
COX4I2__NM_032609_TGAACCGTCGCTCCAATGAG	COX4I2__NM_032609	283	198	314	250
COX4I2__NM_032609_TTGAACTGGAGCCGGTACAC	COX4I2__NM_032609	245	146	274	276
COX5A__NM_004255_ATGAGGAGTTTGATGCTCGC	COX5A__NM_004255	458	418	576	710
COX5A__NM_004255_CAAGCCAGATATAGATGCCT	COX5A__NM_004255	1417	92	1484	77
COX5A__NM_004255_CAAGTTACCATTACATGGCT	COX5A__NM_004255	205	131	265	128
COX5A__NM_004255_CGCTGTGGCCGCAACCACCC	COX5A__NM_004255	511	195	532	193
COX5A__NM_004255_GATGCCTGGGAATTGCGTAA	COX5A__NM_004255	436	578	443	368
COX5A__NM_004255_GGGAATAGCAGCGAACTGAC	COX5A__NM_004255	227	83	233	52
COX5A__NM_004255_TTGAGTACCAAGCCATGTAA	COX5A__NM_004255	208	216	272	309
COX5B__NM_001862_ATGGCTTCAAGGTTACTTCG	COX5B__NM_001862	132	1	104	6
COX5B__NM_001862_CTGCAAAGAAGGGACTGGTA	COX5B__NM_001862	664	586	720	808
COX5B__NM_001862_CTGTGGAGCCCATTACAAGC	COX5B__NM_001862	399	175	431	298
COX5B__NM_001862_GAGATGGAGGGGACTAAATT	COX5B__NM_001862	380	124	357	184
COX5B__NM_001862_TCCACAGCGGGGGCATCGCT	COX5B__NM_001862	907	38	951	95
COX5B__NM_001862_TTGGGGCCAGTACATTGTAT	COX5B__NM_001862	488	242	568	442
COX6A1__NM_004373_ACCCAGCAGCCGAGAAACCG	COX6A1__NM_004373	1693	2017	1946	2071
COX6A1__NM_004373_AGTCTTCCACATGCGAGCTG	COX6A1__NM_004373	125	164	132	140
COX6A1__NM_004373_CCATGGGCGCCACTCGACAT	COX6A1__NM_004373	1455	657	1324	435
COX6A1__NM_004373_CCCGGGGAGCGCGACGAAGA	COX6A1__NM_004373	338	141	301	168
COX6A1__NM_004373_CCTATGTCGAGTGGCGCCCA	COX6A1__NM_004373	524	194	623	218
COX6A1__NM_004373_CTGGGGCGGCCTATGTCGAG	COX6A1__NM_004373	762	619	753	487
COX6A1__NM_004373_CTTGGTCCTGATGCGGAGAT	COX6A1__NM_004373	371	356	405	433
COX6A1__NM_004373_GTGGATTCACATGAGGGTTA	COX6A1__NM_004373	33	7	25	11
COX6A1__NM_004373_GTGTACCTGAAGTCGCACCA	COX6A1__NM_004373	846	635	989	782
COX6A1__NM_004373_GTTGGAAGTGGATTCACATG	COX6A1__NM_004373	417	369	496	175
COX6A1__NM_004373_TGGGGGTAGGCGATGAACTC	COX6A1__NM_004373	48	30	61	23
COX6A1__NM_004373_TTGGAAGTGGATTCACATGA	COX6A1__NM_004373	596	417	517	307
COX6A2__NM_005205_CAGACGCCAGGTACGAGCTG	COX6A2__NM_005205	248	125	319	264
COX6A2__NM_005205_CAGGGGTGTTCGTAGCCCGT	COX6A2__NM_005205	513	618	652	610
COX6A2__NM_005205_CCGTGGGCAGAGGGTTCACG	COX6A2__NM_005205	546	546	607	516
COX6A2__NM_005205_GAGGGTTCACGTGGCTATTG	COX6A2__NM_005205	460	427	435	460
COX6A2__NM_005205_GTGTGGTTGCCGTCCCCCCA	COX6A2__NM_005205	624	837	704	703
COX6A2__NM_005205_GTGTTGGTAGGGACGGAACT	COX6A2__NM_005205	147	98	206	275
COX6A2__NM_005205_TAGGGACGGAACTCGGGGCG	COX6A2__NM_005205	274	197	291	394
COX6A2__NM_005205_TCAGGGGTGTTCGTAGCCCG	COX6A2__NM_005205	360	524	428	471
COX6A2__NM_005205_TGCGGAGGTGTTGGTAGGGA	COX6A2__NM_005205	628	507	658	526
COX6B1__NM_001863_AACTGCTGGCAGAACTACCT	COX6B1__NM_001863	368	2	509	99
COX6B1__NM_001863_AAGGCAATGACCGCTAAAGG	COX6B1__NM_001863	296	52	250	55
COX6B1__NM_001863_AGAACCAGACTAGAAACTGC	COX6B1__NM_001863	106	0	150	25
COX6B1__NM_001863_CAGAAGGCAATGACCGCTAA	COX6B1__NM_001863	1652	1180	2038	2236
COX6B2__NM_144613_ACGCGGAAATAGTACTCGCA	COX6B2__NM_144613	939	1019	905	961
COX6B2__NM_144613_CACGCGGAAATAGTACTCGC	COX6B2__NM_144613	456	422	562	496
COX6B2__NM_144613_CGCGGCGGGTCCTGGTCTTG	COX6B2__NM_144613	4194	3195	4714	3928
COX6B2__NM_144613_CTGGGGAAGCGCGGGTCGAA	COX6B2__NM_144613	625	843	685	660
COX6B2__NM_144613_GGAAGTTCTGGTAGCAGTTA	COX6B2__NM_144613	182	226	237	232
COX6B2__NM_144613_GGCACAGCGAGTGGTACACG	COX6B2__NM_144613	512	632	622	451
COX6B2__NM_144613_GGGAAGCGCGGGTCGAAGGG	COX6B2__NM_144613	1219	1117	1296	1508
COX6B2__NM_144613_TCTGGTAGCAGTTACGGATC	COX6B2__NM_144613	195	162	238	217
COX6C__NM_004374_CAGGCGTCTGCGAAATCATA	COX6C__NM_004374	393	3	374	2
COX6C__NM_004374_GCCAGAAGGCCACGCATCCG	COX6C__NM_004374	364	102	451	122
COX6C__NM_004374_TTGCCAAAACCTCGGATGCG	COX6C__NM_004374	243	379	253	243
COX7A1__NM_001864_CAGCTTGTACTCCCTTGGCT	COX7A1__NM_001864	1270	1190	1556	1344
COX7A1__NM_001864_CTCGGTTCTGAAAGCGGTTC	COX7A1__NM_001864	95	80	144	234
COX7A1__NM_001864_GCGTCATTGTCACTCGGTAC	COX7A1__NM_001864	1034	1384	1182	1532
COX7A1__NM_001864_GCTTCTGTTTCTCGCGCACT	COX7A1__NM_001864	2763	3267	3183	4167
COX7A1__NM_001864_GTGACAATGACGCTGTGTCT	COX7A1__NM_001864	543	509	699	739
COX7A2__NM_001865_ACCAACTGTAAGAATCATGG	COX7A2__NM_001865	359	124	412	213
COX7A2__NM_001865_ACCATGATTCTTACAGTTGG	COX7A2__NM_001865	337	192	399	347
COX7A2__NM_001865_ATTCCACTGTATCTAAAGGG	COX7A2__NM_001865	181	186	190	65
COX7A2__NM_001865_CATGGTGGCTCTATACAGGA	COX7A2__NM_001865	27	2	42	29
COX7A2__NM_001865_CTGAACACCACCAACGAAAA	COX7A2__NM_001865	124	230	172	312
COX7A2__NM_001865_GAAATTCCACTGTATCTAAA	COX7A2__NM_001865	221	141	163	161
COX7A2__NM_001865_GTGAATGCATACGCAAGACT	COX7A2__NM_001865	243	163	223	179
COX7A2__NM_001865_TCATGGTGGCTCTATACAGG	COX7A2__NM_001865	37	39	25	18
COX7A2__NM_001865_TCCACTGTATCTAAAGGGTG	COX7A2__NM_001865	127	46	143	141
COX7A2__NM_001865_TTCCACTGTATCTAAAGGGT	COX7A2__NM_001865	48	8	65	30
COX7A2L__NM_004718_ATCATACACTGTGGAATCGG	COX7A2L__NM_004718	354	370	388	453
COX7A2L__NM_004718_CCACAGTGTATGATTATGCT	COX7A2L__NM_004718	769	1111	1030	1261
COX7A2L__NM_004718_CTGCCTGATCGCCCTCTACA	COX7A2L__NM_004718	901	489	1052	807
COX7A2L__NM_004718_GAATCGGAGGTCAGTTTAGT	COX7A2L__NM_004718	687	462	721	727
COX7A2L__NM_004718_GGAGGTCAGTTTAGTTGGTG	COX7A2L__NM_004718	418	285	402	403
COX7A2L__NM_004718_GTCATGTACTACAAGTTTAG	COX7A2L__NM_004718	195	121	217	177
COX7A2L__NM_004718_GTCCGGTAAAGCATTTGGTC	COX7A2L__NM_004718	60	58	43	35
COX7A2L__NM_004718_GTGCCCGTCTACCTGAAACG	COX7A2L__NM_004718	386	346	419	579
COX7A2L__NM_004718_TAGTGGCTTCACGCAGAAGT	COX7A2L__NM_004718	314	406	364	362
COX7A2L__NM_004718_TGCCTGACCAAATGCTTTAC	COX7A2L__NM_004718	417	267	447	547
COX7A2L__NM_004718_TTCGGAGGCCTATAGCCCGC	COX7A2L__NM_004718	180	122	193	194
COX7B__NM_001866_AATCAGGTGTACGTTTCTGG	COX7B__NM_001866	343	88	335	76
COX7B__NM_001866_CAACACAAGTCGGAATAGAA	COX7B__NM_001866	302	139	279	138
COX7B__NM_001866_CCACTTTCTGTATTGTTACA	COX7B__NM_001866	582	919	659	991
COX7B__NM_001866_GAATGGAACCTGTCCCCTGT	COX7B__NM_001866	1020	11	1062	28
COX7B2__NM_130902_CCACTCAGATTGGAATAGAA	COX7B2__NM_130902	168	216	212	333
COX7B2__NM_130902_CTGCTTTCTGTGTTGCTACA	COX7B2__NM_130902	512	686	612	860
COX7B2__NM_130902_CTTAGTGCATTTCTGGCCAA	COX7B2__NM_130902	276	368	323	367
COX7C__NM_001867_CACCAACTGCTTAAAACATA	COX7C__NM_001867	304	417	334	376
COX7C__NM_001867_GCAGTTGGTGTCTTACTACA	COX7C__NM_001867	333	332	445	175
COX7C__NM_001867_GCTAAGATGTGTTTGTACTT	COX7C__NM_001867	1281	69	1538	241
COX7C__NM_001867_GTGGCTCCTACGGACCACAG	COX7C__NM_001867	3	7	4	0
COX8A__NM_004074_ATCCATTCGTTGCCGCCGGA	COX8A__NM_004074	561	699	617	713
COX8A__NM_004074_CAAGATCCATTCGTTGCCGC	COX8A__NM_004074	704	834	808	1086
COX8A__NM_004074_TCCCCTCCGGCGGCAACGAA	COX8A__NM_004074	20	88	41	98
COX8A__NM_004074_TGTGACAGGATCCAGCCCGC	COX8A__NM_004074	430	704	451	455
COX8C__NM_182971_CGCTCCCCGCTTCGCCCACT	COX8C__NM_182971	7476	9675	8543	9778
COX8C__NM_182971_GCAACCTGAAGCAGTTCAGA	COX8C__NM_182971	419	561	512	703
COX8C__NM_182971_GCTCCCCGCTTCGCCCACTC	COX8C__NM_182971	429	391	415	372
CP__NM_000096_AATTGGGAGACTATATAAGA	CP__NM_000096	113	118	198	159
CP__NM_000096_ATAGTCTCCCAATTCTATCT	CP__NM_000096	118	193	221	234
CP__NM_000096_ATGAAAGGTGTAGGGCCTAG	CP__NM_000096	332	204	307	330
CP__NM_000096_CAAAATGGCCCAGATAGAAT	CP__NM_000096	388	520	411	364
CP__NM_000096_CAACTATAGAAAAACCGGTC	CP__NM_000096	250	278	279	245
CP__NM_000096_CTTCATTACCCATACCAAAA	CP__NM_000096	492	841	533	571
CP__NM_000096_GAAAATCTGTGGTGTTATCA	CP__NM_000096	209	324	279	320
CP__NM_000096_GAGATGGCAATTGTGTGACT	CP__NM_000096	802	747	805	684
CP__NM_000096_TATAGTCTCCCAATTCTATC	CP__NM_000096	214	293	212	292
CP__NM_000096_TGGAATTATTGAAACGACTT	CP__NM_000096	35	38	22	27
CP__NM_000096_TGGTGGATGAAAATTTCAGC	CP__NM_000096	45	24	53	15
CP__NM_000096_TTGGAATTATTGAAACGACT	CP__NM_000096	71	167	101	135
CPA1__NM_001868_AGGGCACTCGGACGTCGATG	CPA1__NM_001868	628	493	780	623
CPA1__NM_001868_CAATCTGGATCTTGCTGACA	CPA1__NM_001868	313	383	393	556
CPA1__NM_001868_CCGAATCTCTGTAGCCGATG	CPA1__NM_001868	407	628	533	884
CPA1__NM_001868_CCTCATCGGCTACAGAGATT	CPA1__NM_001868	232	259	330	461
CPA1__NM_001868_CGACTGCACGTCCTCGATCA	CPA1__NM_001868	1476	1397	1585	1424
CPA1__NM_001868_GCAGTAAGCGTCCAGCCATC	CPA1__NM_001868	744	714	854	847
CPA1__NM_001868_GCGTCCCATTTACGTGCTGA	CPA1__NM_001868	780	846	847	956
CPA1__NM_001868_GGCGTAGTTAAAAGTGTCGG	CPA1__NM_001868	1038	1450	1067	1229
CPA1__NM_001868_GGGCACTCGGACGTCGATGG	CPA1__NM_001868	1513	1345	1554	1386
CPA1__NM_001868_GTAAATGGGACGCCCTTCAT	CPA1__NM_001868	548	489	564	484
CPA1__NM_001868_GTTACCTTCAGCACGTAAAT	CPA1__NM_001868	226	209	337	217
CPA1__NM_001868_TACTGCCCCCCGTGCTGAAC	CPA1__NM_001868	799	872	794	1237
CPA2__NM_001869_ACTGCCTGGACGTTGACGAA	CPA2__NM_001869	388	421	599	578
CPA2__NM_001869_AGACAAGCCAGCTATCTGGC	CPA2__NM_001869	106	134	85	143
CPA2__NM_001869_AGTTCCCTTCGTCAACGTCC	CPA2__NM_001869	460	412	504	434
CPA2__NM_001869_CCAGAATGGAAGTGATGGAT	CPA2__NM_001869	1912	2083	2218	2632
CPA2__NM_001869_CCGGCCTATGAACGTGCTCA	CPA2__NM_001869	688	925	816	993
CPA2__NM_001869_CTGGGATCCATGCTCGAGAG	CPA2__NM_001869	376	414	359	490
CPA2__NM_001869_GACAGTAGATATGCCCAAAA	CPA2__NM_001869	86	165	65	70
CPA2__NM_001869_GACTGCCTGGACGTTGACGA	CPA2__NM_001869	1076	1262	1263	1663
CPA2__NM_001869_GTGTAACCCACTCTCGAGCA	CPA2__NM_001869	225	338	383	499
CPA2__NM_001869_TAAAAATCTGCTACAATTGG	CPA2__NM_001869	143	258	237	214
CPA2__NM_001869_TCACTTTGCTCACTAGACCA	CPA2__NM_001869	430	579	462	661
CPA2__NM_001869_TGGGATCCATGCTCGAGAGT	CPA2__NM_001869	33	55	60	15
CPA3__NM_001870_AAAACAAGCAGACATCATAA	CPA3__NM_001870	466	646	468	768
CPA3__NM_001870_ATGGTCTCTCGTATTAAAAT	CPA3__NM_001870	91	141	113	114
CPA3__NM_001870_ATTTACCTGTCAAAGCGGAC	CPA3__NM_001870	190	263	173	214
CPA3__NM_001870_CAGCTTGACTTCTGGTATCC	CPA3__NM_001870	751	700	895	756
CPA3__NM_001870_CGTAGCTGCTAATATGATGG	CPA3__NM_001870	584	698	575	722
CPA3__NM_001870_GAGGTCAGTGCCGATGCATT	CPA3__NM_001870	672	636	711	719
CPA3__NM_001870_GCTACGCAAAATACAATAAT	CPA3__NM_001870	598	562	767	764
CPA3__NM_001870_GGATTTCCGAGTTAGTGAGA	CPA3__NM_001870	269	311	328	401
CPA3__NM_001870_GGGATTCCTTCTCACTAACT	CPA3__NM_001870	257	349	357	397
CPA3__NM_001870_GTGGCATTCACGCACGAGAA	CPA3__NM_001870	74	274	90	110
CPA3__NM_001870_TGGCATTCACGCACGAGAAT	CPA3__NM_001870	72	37	46	62
CPA3__NM_001870_TTGATGGATATATTTGGTCA	CPA3__NM_001870	279	239	258	434
CPA4__NM_001163446_ATCCACAGGCCGATTGAAGG	CPA4__NM_001163446	715	688	943	894
CPA4__NM_001163446_CACAGATGCTGGACCCAATA	CPA4__NM_001163446	257	342	352	334
CPA4__NM_001163446_CCGGCCGTTTGGCTGAATGC	CPA4__NM_001163446	998	1194	1109	1046
CPA4__NM_001163446_CCTTCAGTACATACATCGGC	CPA4__NM_001163446	1215	1608	1450	1529
CPA4__NM_001163446_CTCCTCCTTCAATCGGCCTG	CPA4__NM_001163446	771	773	693	840
CPA4__NM_001163446_CTGTTCATTGGGGCCCTTAT	CPA4__NM_001163446	191	294	335	351
CPA4__NM_001163446_GACATCCACAGGCCGATTGA	CPA4__NM_001163446	675	550	754	728
CPA4__NM_001163446_GGGAGATCCACTCTCGGGAA	CPA4__NM_001163446	357	421	337	275
CPA4__NM_001163446_TCCAAGATGGAGGTGATAGC	CPA4__NM_001163446	175	154	172	175
CPA4__NM_001163446_TCTCCCAGGCCACTGCAATC	CPA4__NM_001163446	504	433	465	473
CPA4__NM_001163446_TGAGGTGGATACTGTTCATT	CPA4__NM_001163446	350	235	309	181
CPA4__NM_001163446_TGTTCATTGGGGCCCTTATT	CPA4__NM_001163446	117	182	161	170
CPA4__NM_016352_AGCAGTAATAACTTCAACTA	CPA4__NM_016352	192	187	217	200
CPA4__NM_016352_CATCATCTTCATTGTCTAAA	CPA4__NM_016352	290	336	303	440
CPA5__NM_001127441_AAGGAACTCTCATATCCACA	CPA5__NM_001127441	331	236	378	198
CPA5__NM_001127441_ACTTGTTCTTCCGCCATAAG	CPA5__NM_001127441	320	411	459	499
CPA5__NM_001127441_CGGCATCTGGACTGCCAATA	CPA5__NM_001127441	334	416	316	393
CPA5__NM_001127441_GAACTCTCATATCCACAGGG	CPA5__NM_001127441	325	252	309	418
CPA5__NM_001127441_GAAGCAGCTTTCACTTCTCG	CPA5__NM_001127441	1814	2362	2272	2478
CPA5__NM_001127441_GACAGACATACTGAATGCCA	CPA5__NM_001127441	343	474	470	483
CPA5__NM_001127441_GGACCAGGTTCTTCGAGTCC	CPA5__NM_001127441	260	392	407	365
CPA5__NM_001127441_TGCTTACAGCATCATGATAA	CPA5__NM_001127441	146	161	168	203
CPA5__NM_001127441_TGGCATTCAGTATGTCTGTC	CPA5__NM_001127441	28	18	62	47
CPA5__NM_001127441_TGGCCAGGACTCGAAGAACC	CPA5__NM_001127441	24	28	17	13
CPA5__NM_001127441_TTGCAGATTGTCAGTGATTA	CPA5__NM_001127441	212	421	245	307
CPA5__NM_001127441_TTGGGCCAAATGAATTTCAC	CPA5__NM_001127441	296	161	296	192
CPA6__NM_020361_AACTGGAAAGTGAAGTGGTG	CPA6__NM_020361	205	179	161	158
CPA6__NM_020361_AACTGTTCCCTCTGATACAT	CPA6__NM_020361	1209	956	1284	1292
CPA6__NM_020361_ATGCCTGTGTTTAACGTCGA	CPA6__NM_020361	450	507	508	468
CPA6__NM_020361_CACAGTATGTGTCATCACAA	CPA6__NM_020361	106	85	122	68
CPA6__NM_020361_CAGCATAGCGGTTGTTATAA	CPA6__NM_020361	0	0	0	0
CPA6__NM_020361_CTTCCTGTAAGAAGGCTAAC	CPA6__NM_020361	301	237	317	504
CPA6__NM_020361_CTTTATAACAACCGCTATGC	CPA6__NM_020361	173	179	231	272
CPA6__NM_020361_TAAGAAGGCTAACAGGGCTC	CPA6__NM_020361	19	29	32	66
CPA6__NM_020361_TATCCATCGACGTTAAACAC	CPA6__NM_020361	308	418	333	242
CPA6__NM_020361_TTACATTGGTCCAACTAAAA	CPA6__NM_020361	20	28	38	28
CPA6__NM_020361_TTTATAACAACCGCTATGCT	CPA6__NM_020361	391	526	448	625
CPA6__NM_020361_TTTGGAGAAAAACAAGGTCA	CPA6__NM_020361	27	3	17	19
CPAMD8__NM_015692_AACACAGGCTGGTCGGACAA	CPAMD8__NM_015692	392	217	410	301
CPAMD8__NM_015692_ACACAGGCTGGTCGGACAAG	CPAMD8__NM_015692	92	73	119	78
CPAMD8__NM_015692_ACGCCCGCGCGAAAAACAGA	CPAMD8__NM_015692	428	535	508	413
CPAMD8__NM_015692_CAAAAGACTTGTTGTACGCG	CPAMD8__NM_015692	741	853	782	1135
CPAMD8__NM_015692_CAACACAGGCTGGTCGGACA	CPAMD8__NM_015692	1041	815	1119	1081
CPAMD8__NM_015692_CCCACACTTTCAGAAGCGCT	CPAMD8__NM_015692	289	287	372	536
CPAMD8__NM_015692_GAGGCTCTCGGATGATAGAG	CPAMD8__NM_015692	85	40	115	51
CPAMD8__NM_015692_GAGGTCTGGTTGTGAAAGAG	CPAMD8__NM_015692	257	146	233	135
CPAMD8__NM_015692_GTCAATCAGAAGCTCAAACT	CPAMD8__NM_015692	482	291	589	496
CPAMD8__NM_015692_TAAAGGGACAATCAAACTCA	CPAMD8__NM_015692	314	279	326	249
CPAMD8__NM_015692_TCTGAGGCCTGTCAACGAGA	CPAMD8__NM_015692	367	234	456	317
CPAMD8__NM_015692_TGACCATCTTTAACTCTCCA	CPAMD8__NM_015692	587	670	607	712
CPB1__NM_001871_ATACAGGCTGTTCGTACCTA	CPB1__NM_001871	429	685	525	607
CPB1__NM_001871_CACTTGGATCTCACGTCCAT	CPB1__NM_001871	1503	1301	1734	1413
CPB1__NM_001871_CATTAACATAATCCGCGAGT	CPB1__NM_001871	884	1108	1094	1281
CPB1__NM_001871_CCACAGTCCATGAAAATGGC	CPB1__NM_001871	201	215	210	399
CPB1__NM_001871_CGGAAGTCAACTGTACTGTG	CPB1__NM_001871	286	513	328	533
CPB1__NM_001871_CTCATCTCTCGCAGTGTTAT	CPB1__NM_001871	805	1238	909	1066
CPB1__NM_001871_CTGCCAGTGGTTTGTAAGAG	CPB1__NM_001871	165	345	156	344
CPB1__NM_001871_GATAGCCGGGTTCGTGCAAC	CPB1__NM_001871	298	356	411	457
CPB1__NM_001871_GATGAGGGCTGGATTCTCAG	CPB1__NM_001871	850	477	769	451
CPB1__NM_001871_GGAGGCTCAGTTTGATAGCC	CPB1__NM_001871	189	160	201	385
CPB1__NM_001871_TGGAGGCTCAGTTTGATAGC	CPB1__NM_001871	37	31	69	93
CPB1__NM_001871_TGTGTCCTGTTGCACGAACC	CPB1__NM_001871	310	348	328	252
CPB2__NM_001278541_AAAGCCCATTTAAATGTGAG	CPB2__NM_001278541	141	222	186	172
CPB2__NM_001278541_ACAGTAGGTTTCCGAGCATG	CPB2__NM_001278541	339	462	406	463
CPB2__NM_001278541_ACATCTGCCAGCAAGACACT	CPB2__NM_001278541	487	572	418	589
CPB2__NM_001278541_AGCATGTCTTCGCGTTTCAG	CPB2__NM_001278541	416	528	450	624
CPB2__NM_001278541_ATACTGTTCATAGTACGATG	CPB2__NM_001278541	217	190	266	210
CPB2__NM_001278541_CTGTTCATAGTACGATGCGG	CPB2__NM_001278541	347	318	291	321
CPB2__NM_001278541_GATTCCACAGTCAATCCATA	CPB2__NM_001278541	512	628	636	759
CPB2__NM_001278541_GATTGTTCGCATAGAAAGAA	CPB2__NM_001278541	74	124	92	198
CPB2__NM_001278541_GCATCGGAACAGACCTGAAT	CPB2__NM_001278541	798	1044	862	843
CPB2__NM_001278541_GGATAGAATTTATAACTGAG	CPB2__NM_001278541	147	367	189	238
CPB2__NM_001278541_TCATGCTCGGAAACCTACTG	CPB2__NM_001278541	0	0	0	0
CPB2__NM_001278541_TCGGGGGCTGACTGTGTCGT	CPB2__NM_001278541	2221	2715	2214	2627
CPB2__NM_001872_ACTCAATTCTATGGATAAT	CPB2__NM_001872	407	377	528	669
CPB2__NM_001872_CTCAATTCTATGGGATAATA	CPB2__NM_001872	133	122	125	174
CPB2__NM_001872_GGATTTCTATGTTATGCCAG	CPB2__NM_001872	140	76	127	180
CPB2__NM_001872_TTGCAGATAACTCAATTCTA	CPB2__NM_001872	184	329	273	370
CPD__NM_001199775_AATATCACAACAGGCAGATT	CPD__NM_001199775	216	215	140	62
CPD__NM_001199775_AGAGAATGCAACCATCTCAG	CPD__NM_001199775	612	313	622	408
CPD__NM_001199775_ATTCCAGTGGCCTTATGTTC	CPD__NM_001199775	291	345	335	344
CPD__NM_001199775_CGGCGCACATTGGTATGATG	CPD__NM_001199775	933	926	1014	928
CPD__NM_001199775_CTTCACAGCTTCGACAGGAA	CPD__NM_001199775	155	151	163	248
CPD__NM_001199775_CTTTCTGGAAATCTGCATGG	CPD__NM_001199775	181	209	200	126
CPD__NM_001199775_GAAGACGAGACTTTCAAAGA	CPD__NM_001199775	233	162	255	162
CPD__NM_001199775_GAATCACAAACGGCGCACAT	CPD__NM_001199775	984	952	977	726
CPD__NM_001199775_GCAAGATTACAATTATGTGT	CPD__NM_001199775	660	563	693	474
CPD__NM_001199775_GCACATTGGTATGATGTGGA	CPD__NM_001199775	742	780	981	848
CPD__NM_001199775_TAATGTGATCTCAAAACAGT	CPD__NM_001199775	571	863	606	781
CPD__NM_001199775_TTCACAGCTTCGACAGGAAT	CPD__NM_001199775	69	172	124	182
CPD__NM_001304_AGCGGCCGCGACAATAGTCG	CPD__NM_001304	525	704	540	447
CPD__NM_001304_CGCGGCCGCGACTATTGTCG	CPD__NM_001304	986	790	988	783
CPD__NM_001304_GACTACCACAACTACGAGCG	CPD__NM_001304	442	262	547	522
CPD__NM_001304_GACTGTGGCTTCGGCGACGG	CPD__NM_001304	942	869	748	617
[illegible]	[illegible]	[illegible]	[illegible]	[illegible]	[illegible]
CPED1__NM_001105533_CAGGCAAGCAGATGCAAGAA	CPED1__NM_001105533	231	255	191	181
CPED1__NM_001105533_CTTTCTTGCATCTGCTTGCC	CPED1__NM_001105533	411	455	415	509
CPED1__NM_001105533_GAAAGGAGGCCTGTAGAGTA	CPED1__NM_001105533	465	544	590	592
CPED1__NM_001105533_GCCCAGTGTTCCCTTGTCGT	CPED1__NM_001105533	2928	2511	3302	2595
CPED1__NM_001105533_GGGCCGGGGCTACTAGAACA	CPED1__NM_001105533	315	363	386	383
[illegible]	[illegible]	[illegible]	[illegible]	[illegible]	[illegible]
CPNE3__NM_003909_GTGGTTCAGAAATTGAAATT	CPNE3__NM_003909	0	0	0	0
CPNE3__NM_003909_TTCCATCAGATGTCTGCTTG	CPNE3__NM_003909	190	245	174	269
CPNE3__NM_003909_TTCCTTGACTATATAATGGG	CPNE3__NM_003909	16	38	17	13
CPNE4__NM_001289112_ATCCTCAAGATGCAGTCTCA	CPNE4__NM_001289112	433	545	506	523
CPNE4__NM_001289112_CACGCAGCTCAACTTTGGTC	CPNE4__NM_001289112	1227	1748	1529	1344
[illegible]	[illegible]	[illegible]	[illegible]	[illegible]	[illegible]
CPOX__NM_000097_AGATGTTGCCTAAGACCTCG	CPOX__NM_000097	399	403	385	258
CPOX__NM_000097_AGCTCGCCCAGGTCGGTCAC	CPOX__NM_000097	855	1379	817	705
CPOX__NM_000097_ATCAGCTGTGTACTTCAAGA	CPOX__NM_000097	197	196	262	194
CPOX__NM_000097_CAAAGTATCTGTAGTTGAAA	CPOX__NM_000097	95	201	150	246
CPOX__NM_000097_CACGGCTCGACGTCGAGAGG	CPOX__NM_000097	607	827	572	738
[illegible]	[illegible]	[illegible]	[illegible]	[illegible]	[illegible]

CRIP3__NM_206922_GCTGGCACCTCAGACACGGT	CRIP3__NM_206922	1327	1105	1387	1211
CRIP3__NM_206922_GTGAATGTCTTCATATGGGG	CRIP3__NM_206922	863	827	937	868
CRIP3__NM_206922_TAGCAGGGGACGTGGCAGTA	CRIP3__NM_206922	1134	1511	1260	1510
CRIP3__NM_206922_TGAATGTCTTCATATGGGGA	CRIP3__NM_206922	302	433	344	314
CRIPAK__NM_175918_AGCAGGCGAACACTCCACAT	CRIPAK__NM_175918	1575	1505	1713	1677
CRIPAK__NM_175918_AGCAGGCGGGCACTCCACAA	CRIPAK__NM_175918	666	618	757	527
CRIPAK__NM_175918_ATGGGCACGTGTGATCAGGC	CRIPAK__NM_175918	538	603	601	1027
CRIPAK__NM_175918_CATGGGCACGTGTGATCAGG	CRIPAK__NM_175918	1004	1117	1086	1434
CRIPAK__NM_175918_CCACATGGGCACGTGTGATC	CRIPAK__NM_175918	547	594	579	700
CRIPAK__NM_175918_CCTGCTCACACGTGCCAACG	CRIPAK__NM_175918	59	76	94	137
CRIPAK__NM_175918_CCTGCTCACGTGCCGATGTG	CRIPAK__NM_175918	916	1118	865	1018
CRIPAK__NM_175918_CGCCTGCTCACGTGCCCATA	CRIPAK__NM_175918	1580	1911	1851	2630
CRIPAK__NM_175918_GAGCAGGCGAGCACTCCACA	CRIPAK__NM_175918	670	636	694	777
CRIPAK__NM_175918_GAGCAGGCGGGCACTCCATA	CRIPAK__NM_175918	755	908	903	1133
CRIPAK__NM_175918_GCCTGCTCACGTGCCGATGT	CRIPAK__NM_175918	2030	2692	2440	3242
CRIPAK__NM_175918_TGCCAGGGCTTTGTGTGAGC	CRIPAK__NM_175918	530	585	671	858
CRIPT__NM_014171_GATGGTGCTAGGAATACCAC	CRIPT__NM_014171	224	182	223	122
CRISP1__NM_001131_AAGAATGAACCTTATAAGAC	CRISP1__NM_001131	63	45	74	46
CRISP1__NM_001131_ACGATCTCTTCTTGTACATT	CRISP1__NM_001131	1519	2208	1787	2574
CRISP1__NM_001131_ACTTGGAAGTCTCCTCTCAA	CRISP1__NM_001131	778	638	851	687
CRISP1__NM_001131_ATCGAGGTGATCCTTGTTGG	CRISP1__NM_001131	1498	1248	1508	1543
CRISP1__NM_001131_ATTACACTTGACCATGATAC	CRISP1__NM_001131	245	129	296	305
CRISP1__NM_001131_CATACTTTCATGGACAACAT	CRISP1__NM_001131	286	279	371	424
CRISP1__NM_001131_CATGGGACGCCTGTCTTATA	CRISP1__NM_001131	949	972	984	831
CRISP1__NM_001131_GACATACAAGTCCATTATCT	CRISP1__NM_001131	932	724	1132	1015
CRISP1__NM_001131_GAGTCTACAAGTTTCAAACA	CRISP1__NM_001131	164	180	197	303
CRISP1__NM_001131_GTATCATGGTCAAGTGTAAT	CRISP1__NM_001131	500	631	631	599
CRISP1__NM_001131_TACTTGGGCAGGCTTCACAT	CRISP1__NM_001131	1007	1054	1143	1137
CRISP1__NM_001131_TGGGCCACATCTTACCTGAT	CRISP1__NM_001131	378	397	382	317
CRISP2__NM_001142407_AAGAATACCCCGTACCAACA	CRISP2__NM_001142407	154	154	228	266
CRISP2__NM_001142407_AAGGTGTTCCTTGTTGGTAC	CRISP2__NM_001142407	128	71	103	128
CRISP2__NM_001142407_AGGTAACAACGAATGCCCAA	CRISP2__NM_001142407	325	284	313	431
CRISP2__NM_001142407_AGGTGTTCCTTGTTGGTACG	CRISP2__NM_001142407	354	312	321	336
CRISP2__NM_001142407_AGTTGTTGGACATTATACTC	CRISP2__NM_001142407	301	540	350	276
CRISP2__NM_001142407_TAATGTCCAACAACTGCATT	CRISP2__NM_001142407	339	272	343	478
CRISP2__NM_001142407_TACCATCTTTAGCATGTTAC	CRISP2__NM_001142407	317	345	328	404
CRISP2__NM_001142407_TCATCAGGGCAACCGGCACA	CRISP2__NM_001142407	994	712	954	994
CRISP2__NM_001142407_TGCCAGTAACATGCTAAAGA	CRISP2__NM_001142407	250	260	268	185
CRISP3__NM_001190986_AGTCCATCGTCACAGTTATC	CRISP3__NM_001190986	1006	1170	1077	1264
CRISP3__NM_001190986_CCACCCAAACACAAGTGCAA	CRISP3__NM_001190986	529	881	554	701
CRISP3__NM_001190986_GAAAACAAAACCGGTGGAAC	CRISP3__NM_001190986	140	182	140	276
CRISP3__NM_001190986_GCAGTAAAAGCGGGATCCTA	CRISP3__NM_001190986	394	338	486	433
CRISP3__NM_001190986_GTCCATCGTCACAGTTATCT	CRISP3__NM_001190986	928	1384	940	713
CRISP3__NM_001190986_TAATGTCCAACCACTGCGTT	CRISP3__NM_001190986	967	1356	1114	1529
CRISP3__NM_001190986_TACTGTGTCTGTAATTGCAC	CRISP3__NM_001190986	469	332	551	535
CRISP3__NM_001190986_TGCAGCAAATGCCCAAAAGT	CRISP3__NM_001190986	141	276	220	253
CRISP3__NM_001190986_TGCCCAGATAACTGTGACGA	CRISP3__NM_001190986	720	755	831	827
CRISP3__NM_001190986_TGCTGGGAAAACAAAACCGG	CRISP3__NM_001190986	158	152	222	173
CRISP3__NM_001190986_TGGTACTCTTCATACCTCGT	CRISP3__NM_001190986	0	0	1	0
CRISP3__NM_001190986_TTACAGACACAGTAACCCAA	CRISP3__NM_001190986	28	12	41	51
CRISP3__NM_006061_ATTAACCTGTAAACATCAGT	CRISP3__NM_006061	227	370	349	381
CRISP3__NM_006061_CCTGACCAACTGATGTTTAC	CRISP3__NM_006061	1295	1699	1464	1943
CRISPLD1__NM_001286777_AACACCTGAATGGACAATAT	CRISPLD1__NM_001286777	704	688	788	724
CRISPLD1__NM_001286777_AGCCCCCTCCAAAACTAGGT	CRISPLD1__NM_001286777	91	71	120	121
CRISPLD1__NM_001286777_ATCTGATCTTGTCCGGACAT	CRISPLD1__NM_001286777	819	679	937	672
CRISPLD1__NM_001286777_CATCTGATCTTGTCCGGACA	CRISPLD1__NM_001286777	1973	1637	1776	1852
CRISPLD1__NM_001286777_CATTTGTTTCCTCTTCTCGA	CRISPLD1__NM_001286777	631	740	794	1160
CRISPLD1__NM_001286777_GAACACCTGAATGGACAATA	CRISPLD1__NM_001286777	359	605	559	484
CRISPLD1__NM_001286777_GCCCATGTTTGTAAGGGGCA	CRISPLD1__NM_001286777	580	631	549	838
CRISPLD1__NM_001286777_GGTTGCATTCATGTTCATAT	CRISPLD1__NM_001286777	457	520	535	443
CRISPLD1__NM_001286777_GTGTATAATGTGTACATACA	CRISPLD1__NM_001286777	199	202	139	126
CRISPLD1__NM_001286777_TGGGTAGATATCACTAGACA	CRISPLD1__NM_001286777	119	108	127	100
CRISPLD1__NM_001286777_TGTAGAGCTGCAATTCATTA	CRISPLD1__NM_001286777	480	570	535	431
CRISPLD1__NM_001286777_TGTGTATAATGTGTACATAC	CRISPLD1__NM_001286777	286	199	298	255
CRISPLD1__NM_001286778_CCACATTATGCTCGTGTAAT	CRISPLD1__NM_001286778	340	463	403	575
CRISPLD1__NM_001286778_TGTGAAGCAGGCTTATGAAA	CRISPLD1__NM_001286778	364	284	446	394
CRISPLD1__NM_001286778_TTCACAGTCTCTAAAGTAAC	CRISPLD1__NM_001286778	9	0	4	0
CRISPLD1__NM_001286778_TTGGGGAGTAATTGCACACC	CRISPLD1__NM_001286778	230	183	265	220
CRISPLD1__NM_001286778_TTTGCTTGCATACAGTTACG	CRISPLD1__NM_001286778	204	274	317	294
CRISPLD1__NM_031461_ACGATTGTACATGAAACGTC	CRISPLD1__NM_031461	416	335	332	244
CRISPLD1__NM_031461_AGCCAAACAACGAGGGAAAA	CRISPLD1__NM_031461	57	70	56	84
CRISPLD1__NM_031461_CACGATTGTACATGAAACGT	CRISPLD1__NM_031461	672	503	853	545
CRISPLD1__NM_031461_CCTGGGCTGAAAGTTGCTTG	CRISPLD1__NM_031461	4077	4774	4530	5989
CRISPLD1__NM_031461_CGACGTTTCATGTACAATCG	CRISPLD1__NM_031461	871	1165	1016	1152
CRISPLD1__NM_031461_CGATTGTACATGAAACGTCG	CRISPLD1__NM_031461	726	783	848	886
CRISPLD1__NM_031461_GCAAGCTTGCTTCCATCAAT	CRISPLD1__NM_031461	478	679	556	592
CRISPLD1__NM_031461_GGTGGATAGCCAAACAACGA	CRISPLD1__NM_031461	50	22	57	39
CRISPLD1__NM_031461_GGTTCCCAATGCCACTTTAT	CRISPLD1__NM_031461	311	342	352	331
CRISPLD1__NM_031461_GTATCCAACAGCCTCTAATA	CRISPLD1__NM_031461	280	182	313	175
CRISPLD1__NM_031461_TACCATATACTCCATATTAG	CRISPLD1__NM_031461	550	581	685	495
CRISPLD1__NM_031461_TGGATGAGGATGGTGAGTGG	CRISPLD1__NM_031461	104	90	134	173
CRISPLD2__NM_031476_AGCCTTGGGATCCGCAGACC	CRISPLD2__NM_031476	744	966	896	1020
CRISPLD2__NM_031476_ATGGACACCAGCAGACTGGT	CRISPLD2__NM_031476	17	0	15	1
CRISPLD2__NM_031476_CACAGCACAACCGATCTTGT	CRISPLD2__NM_031476	2189	2644	2446	2296
CRISPLD2__NM_031476_CATGTGCACGCACTACACAC	CRISPLD2__NM_031476	1145	1048	1186	1354
CRISPLD2__NM_031476_CTCCTCTAAGAGAGTGACGT	CRISPLD2__NM_031476	470	554	515	556
CRISPLD2__NM_031476_GCAGACCTGGGATGACGAAC	CRISPLD2__NM_031476	434	559	469	376
CRISPLD2__NM_031476_GCCCAACGTCACTCTCTTAG	CRISPLD2__NM_031476	1690	1483	1768	1530
CRISPLD2__NM_031476_GGGGTTGCACTCGCTCGGGT	CRISPLD2__NM_031476	920	806	856	914
CRISPLD2__NM_031476_GGTTGCACTCGCTCGGGTAG	CRISPLD2__NM_031476	1094	1314	1082	1040
CRISPLD2__NM_031476_GTCCTTCACCTCGTCATACC	CRISPLD2__NM_031476	647	609	691	836
CRISPLD2__NM_031476_TGGGTGGTGTCATCCCCTTG	CRISPLD2__NM_031476	255	341	194	208
CRISPLD2__NM_031476_TGTGCAGTCCTGGTATGACG	CRISPLD2__NM_031476	1454	1514	1573	1304
CRK__NM_005206_AGCCCCTCCAGACTCCGAAT	CRK__NM_005206	673	507	698	401
CRK__NM_005206_CAAACTCTTGATCTCCTATT	CRK__NM_005206	666	598	692	728
CRK__NM_005206_CTGGGGGAGGTTGAGTCGGC	CRK__NM_005206	1164	904	1134	1291
CRK__NM_005206_GAGCCCTCTTTGACTTTAAT	CRK__NM_005206	372	439	460	485
CRK__NM_005206_GGAGCGGAGTAGCTGGTACT	CRK__NM_005206	1061	809	950	947
CRK__NM_005206_GGCGGGCAACTTCGACTCGG	CRK__NM_005206	2063	2047	2282	2587
CRK__NM_005206_GGTCTATACTTCTCGACGTA	CRK__NM_005206	115	147	188	178
CRK__NM_005206_GTCTATACTTCTCGACGTAA	CRK__NM_005206	101	54	133	160
CRK__NM_005206_TACTTCTCGACGTAAGGGAC	CRK__NM_005206	161	223	224	213
CRK__NM_005206_TAGTGGAGTGATTCTCAGGC	CRK__NM_005206	421	410	359	385
CRK__NM_005206_TCCAATCAGAGCCGATACTG	CRK__NM_005206	575	484	479	494
CRK__NM_016823_TTGACGCTGGGTTGGGCATA	CRK__NM_016823	95	110	110	124
CRKL__NM_005207_AAAACTTGTGTATCCCGGGA	CRKL__NM_005207	232	210	235	296
CRKL__NM_005207_AAACATGCAGTATCTCGCCA	CRKL__NM_005207	657	467	673	383
CRKL__NM_005207_ACCGCTCCGCCTGGTATATG	CRKL__NM_005207	745	255	723	220
CRKL__NM_005207_ACCTGATGACATGGTTAACG	CRKL__NM_005207	791	448	769	589
CRKL__NM_005207_ACGTACTAGAGGGCATTGAC	CRKL__NM_005207	393	546	513	691
CRKL__NM_005207_ACTTCAAAGGAGACTAGGAC	CRKL__NM_005207	336	184	350	314
CRKL__NM_005207_AGAACACACAAGTCTAGGCC	CRKL__NM_005207	417	275	567	481
CRKL__NM_005207_AGACACCGGCCCCATATACC	CRKL__NM_005207	2377	467	1925	654
CRKL__NM_005207_AGCACAGAAGGTGTCTAGTC	CRKL__NM_005207	146	115	174	140
CRKL__NM_005207_AGCCAACTGGTATATACGTG	CRKL__NM_005207	741	680	956	831
CRKL__NM_005207_AGTCCTCCGGTCGCAGAGGG	CRKL__NM_005207	173	137	171	145
CRKL__NM_005207_ATATACCTAGAGTATACCTG	CRKL__NM_005207	76	96	154	76
CRKL__NM_005207_ATCTGGTTGAAAAAGGCCTA	CRKL__NM_005207	588	464	694	382
CRKL__NM_005207_ATGGCAAAACAAGGTACTAC	CRKL__NM_005207	216	168	284	264
CRKL__NM_005207_ATTCTGAAGTGCGCCACACA	CRKL__NM_005207	395	223	546	412
CRKL__NM_005207_ATTGCTGCCCGTTTGTACAT	CRKL__NM_005207	854	453	885	565
CRKL__NM_005207_ATTTCCCTGTTAGCTAAAAG	CRKL__NM_005207	65	150	86	91
CRKL__NM_005207_ATTTGGGAAAACTATCGCCA	CRKL__NM_005207	199	167	177	65
CRKL__NM_005207_CAACCCACTGCTCTTATCAC	CRKL__NM_005207	896	1062	803	675
CRKL__NM_005207_CACACAAAATAATGCAGTTG	CRKL__NM_005207	978	1623	1161	1450
CRKL__NM_005207_CATAGTCCTCCGGTCGCAGA	CRKL__NM_005207	117	30	141	26
CRKL__NM_005207_CATTGCTGCCCGTTTGTACA	CRKL__NM_005207	714	951	755	1036
CRKL__NM_005207_CCAGGAATTTGACCATTTGC	CRKL__NM_005207	624	403	697	378
CRKL__NM_005207_CCCGATCTTAAAACGGCGGT	CRKL__NM_005207	356	318	293	226
CRKL__NM_005207_CCCTGGGGACTATGTGCTGT	CRKL__NM_005207	1734	558	1756	563
CRKL__NM_005207_CCGATCTTAAAACGGCGGTT	CRKL__NM_005207	790	199	807	229
CRKL__NM_005207_CCTAGAGTATACCTGTGGTT	CRKL__NM_005207	1395	1153	1449	1364
CRKL__NM_005207_CCTGGTCCCCGATCTTAAAA	CRKL__NM_005207	153	137	148	59
CRKL__NM_005207_CGAGGAACATACCGTGGCGC	CRKL__NM_005207	1017	203	1037	341
CRKL__NM_005207_CGCGGACGAGGAACATACCG	CRKL__NM_005207	1275	302	1181	288
CRKL__NM_005207_CGCTTCTGGTTAGGTGTGTC	CRKL__NM_005207	370	416	347	541
CRKL__NM_005207_CTGACACACCTAACCAGAAG	CRKL__NM_005207	359	340	363	264
CRKL__NM_005207_CTGGGACCAGTAACGTAAAA	CRKL__NM_005207	259	116	347	328
CRKL__NM_005207_GAAACAAATTACGTACTAGA	CRKL__NM_005207	99	246	186	190
CRKL__NM_005207_GACAGGATCGTTGTCCCTAC	CRKL__NM_005207	187	56	245	101
CRKL__NM_005207_GACCGCTCCGCCTGGTATAT	CRKL__NM_005207	1009	309	924	277
CRKL__NM_005207_GAGGCGGTCACCGTTCTCAC	CRKL__NM_005207	802	603	859	744
CRKL__NM_005207_GCACAGAAGGTGTCTAGTCT	CRKL__NM_005207	491	591	576	608
CRKL__NM_005207_GCGCCACACAAGGTGAGTCG	CRKL__NM_005207	17	22	37	58
CRKL__NM_005207_GGAAACAAATTACGTACTAG	CRKL__NM_005207	355	471	334	371
CRKL__NM_005207_GGACCGCTCCGCCTGGTATA	CRKL__NM_005207	529	191	463	192
CRKL__NM_005207_GGCAGTTGCCGCTTCTGGTT	CRKL__NM_005207	1340	1500	1705	1683
CRKL__NM_005207_GGCGTTGTTCATAGAATCGT	CRKL__NM_005207	642	498	657	488
CRKL__NM_005207_GGTCCCCGATCTTAAAACGG	CRKL__NM_005207	121	25	90	15
CRKL__NM_005207_GTCCTAGTCTCCTTTGAAGT	CRKL__NM_005207	465	222	383	189
CRKL__NM_005207_TACAGCAGTTATCTGCCTGA	CRKL__NM_005207	753	410	938	540
CRKL__NM_005207_TAGACTTGTGTGTTCTAAAC	CRKL__NM_005207	113	96	107	126
CRKL__NM_005207_TCAACTCCACCCTCTGCGAC	CRKL__NM_005207	864	989	1113	1011
CRKL__NM_005207_TCAGAGTTGGGGGTACTTTA	CRKL__NM_005207	79	92	86	60
CRKL__NM_005207_TCGTTAACCATGTCATCAGG	CRKL__NM_005207	480	367	544	365
CRKL__NM_005207_TCTGGCAGCCTGACGAGATT	CRKL__NM_005207	884	556	850	495
CRKL__NM_005207_TCTGGTTGAAAAAGGCCTAA	CRKL__NM_005207	64	96	128	61
CRKL__NM_005207_TTCTAAACAGGTTAAGTAGC	CRKL__NM_005207	0	0	0	0
CRKL__NM_005207_TTGCCAGTGATAAGAGCAGT	CRKL__NM_005207	337	214	293	162
CRKL__NM_005207_TTTAAGTCTGAACCTTAAAG	CRKL__NM_005207	2	5	1	4
CRLF1__NM_004750_ACCGCTGCCTGGACCCATTG	CRLF1__NM_004750	1070	888	1158	1039
CRLF1__NM_004750_ACTCCCTCAAGTACAAGCTT	CRLF1__NM_004750	582	318	534	424
CRLF1__NM_004750_ACTCCTCACATGTGTTGTCC	CRLF1__NM_004750	1235	1174	1432	1237
CRLF1__NM_004750_AGCCAGGATGCTGCCGTCAC	CRLF1__NM_004750	1435	1153	1609	1338
CRLF1__NM_004750_AGGTGGAGGCGTTGAGTACA	CRLF1__NM_004750	312	394	387	318
CRLF1__NM_004750_CAGGATCCCACGCTTCTCAT	CRLF1__NM_004750	1894	1424	2016	2073
CRLF1__NM_004750_CCAGCGTGAGTACATCGGAG	CRLF1__NM_004750	852	978	864	657
CRLF1__NM_004750_CGGGCTCCTGATCCGGCTCG	CRLF1__NM_004750	651	785	820	643
CRLF1__NM_004750_GACCAGCAGCTGATGTTGAC	CRLF1__NM_004750	1299	1262	1421	1541
CRLF1__NM_004750_GACGTGCACGTGAGCCGCGT	CRLF1__NM_004750	1567	1726	1793	1383
CRLF1__NM_004750_GGCCTCTACTGGACCCTCAA	CRLF1__NM_004750	146	127	160	227
CRLF1__NM_004750_TGGGTCCAGGCAGCGGTCGG	CRLF1__NM_004750	255	182	206	208
CRLF3__NM_015986_AAAGCTCCTGGATGAGCGAT	CRLF3__NM_015986	145	149	236	119
CRLF3__NM_015986_ATTAGTTCTTCTATCTGTAC	CRLF3__NM_015986	247	146	191	252
CRLF3__NM_015986_CCTCGCACATTCAGTTGGAC	CRLF3__NM_015986	1207	1373	1358	1530
CRLF3__NM_015986_CCTGTCCAACTGAATGTGCG	CRLF3__NM_015986	957	885	1136	1548
CRLF3__NM_015986_CGCTGCCTCCACGTTCTCGC	CRLF3__NM_015986	2242	2616	2274	2467
CRLF3__NM_015986_CTCATCCAGGAGCTTTCCAA	CRLF3__NM_015986	151	132	199	168
CRLF3__NM_015986_CTGGAGGCATCATTGTACGA	CRLF3__NM_015986	562	483	617	573
CRLF3__NM_015986_GCCTTGTTTATCTGCTCAGT	CRLF3__NM_015986	1055	1455	1100	1268
CRLF3__NM_015986_GGAGCTGGGTCACCGGCTTG	CRLF3__NM_015986	691	924	851	915
CRLF3__NM_015986_GGGTCACCAATCGCTCATCC	CRLF3__NM_015986	522	255	656	395
CRLF3__NM_015986_TCCAACTGAGCAGATAAACA	CRLF3__NM_015986	643	633	660	538
CRLF3__NM_015986_TGGAGGCAGCGCAGAGCTAC	CRLF3__NM_015986	36	54	33	51
CRLS1__NM_001127458_AAGCTGCCACCAAGATTAAC	CRLS1__NM_001127458	171	13	147	5
CRLS1__NM_001127458_ACATATTCGGGATTGTCCAT	CRLS1__NM_001127458	403	46	419	62
CRLS1__NM_001127458_ATAATCAAATAGCCCAGAAC	CRLS1__NM_001127458	252	66	211	166
CRLS1__NM_001127458_ATAGCAAGGATTGAAATACT	CRLS1__NM_001127458	242	58	254	100
CRLS1__NM_001127458_ATAGTTACTATCATTATGGC	CRLS1__NM_001127458	112	56	155	73
CRLS1__NM_001127458_ATGGATTTATTGCTCGAAAC	CRLS1__NM_001127458	61	60	42	29
CRLS1__NM_001127458_CTGTCAGCATAGTTGAAAAC	CRLS1__NM_001127458	0	0	0	0
CRLS1__NM_001127458_GAAATGATCATGTAAGTAAG	CRLS1__NM_001127458	187	4	198	4
CRLS1__NM_001127458_GGCCAATCAAAGATCAGCTT	CRLS1__NM_001127458	402	188	413	299
CRLS1__NM_001127458_TACAGCAGTCCAGTTAATCT	CRLS1__NM_001127458	703	5	944	23
CRLS1__NM_001127458_TCGTCATTGACAACATATTC	CRLS1__NM_001127458	92	96	87	134
CRLS1__NM_001127458_TTTAGCTGGACTAACAGATT	CRLS1__NM_001127458	57	9	86	27
CRLS1__NM_019095_ACCAGGCTGGGGGTGCTCGC	CRLS1__NM_019095	901	324	1012	333
CRLS1__NM_019095_CGCCCGAACAGTGGTTCCGC	CRLS1__NM_019095	831	420	854	296
CRLS1__NM_019095_GCTGCCTGGCCGAACGCTGG	CRLS1__NM_019095	1879	690	2120	898
CRLS1__NM_019095_GGGCAGCCGCAAGCCAAGAG	CRLS1__NM_019095	288	87	331	171
CRMP1__NM_001014809_ACCCCGCGCCAGAAGTACGG	CRMP1__NM_001014809	868	748	950	957
CRMP1__NM_001014809_ACTTCTCGAAAGAGGTCAGT	CRMP1__NM_001014809	484	649	437	415
CRMP1__NM_001014809_AGACCATTGAAGCCAACGGG	CRMP1__NM_001014809	366	458	454	360
CRMP1__NM_001014809_CCAGGTAGACGTCAGCATAA	CRMP1__NM_001014809	93	60	173	102
CRMP1__NM_001014809_GACTTCGACGCCTACAGTGT	CRMP1__NM_001014809	912	1218	1099	1386
CRMP1__NM_001014809_GAGAACTTAATCGTTCCTGG	CRMP1__NM_001014809	219	153	166	168
CRMP1__NM_001014809_GGAGGATGGACTTATCAAGT	CRMP1__NM_001014809	623	537	633	661
CRMP1__NM_001014809_GGGAATAACCATCCGCCCGT	CRMP1__NM_001014809	1049	1142	1017	1020
CRMP1__NM_001014809_GGGCGGATGGTTATTCCCGG	CRMP1__NM_001014809	507	427	549	596
CRMP1__NM_001014809_GTGGAGGGAGTAATCACAGC	CRMP1__NM_001014809	117	91	100	133
CRMP1__NM_001014809_TCAATGGTCTTCACTCCACC	CRMP1__NM_001014809	1570	1806	1774	2072
CRMP1__NM_001014809_TGGAGGATGGACTTATCAAG	CRMP1__NM_001014809	132	54	98	112
CRMP1__NM_001288661_GAAAATGAACAACGAAGTGG	CRMP1__NM_001288661	59	157	84	59
CRMP1__NM_001288661_GCAGAAAATGAACAACGAAG	CRMP1__NM_001288661	144	191	193	170
CRMP1__NM_001288662_CAAGCTGGTACGATGGCGTT	CRMP1__NM_001288662	312	378	296	355
CRMP1__NM_001288662_GACATCACAAGCTGGTACGA	CRMP1__NM_001288662	176	259	302	229
CRMP1__NM_001313_GAAGAGCATCCCGCACATCA	CRMP1__NM_001313	1620	1906	1616	1974
CRNKL1__NM_001278625_ACAGGACTGTGATTAGTAAC	CRNKL1__NM_001278625	203	6	346	76
CRNKL1__NM_001278625_ACCTCTCGCTTGAGCCGTGA	CRNKL1__NM_001278625	742	895	1137	1457
CRNKL1__NM_001278625_CCGATTTCTAGCGTCGTTGC	CRNKL1__NM_001278625	467	302	580	510
CRNKL1__NM_001278625_CCGGCACGGACGCTAGAAAT	CRNKL1__NM_001278625	296	187	313	249
CRNKL1__NM_001278625_CCTGGTCATCCCAGTTCCGG	CRNKL1__NM_001278625	1727	2615	1847	2643
CRNKL1__NM_001278625_CGAGTGGCTCTGTCGAGCCG	CRNKL1__NM_001278625	111	72	74	49
CRNKL1__NM_001278625_CTCCACGGCTGCCCAAGG	CRNKL1__NM_001278625	137	104	156	165
CRNKL1__NM_001278625_CTGGACACCTGTCCCCGGCA	CRNKL1__NM_001278625	167	54	163	187
CRNKL1__NM_001278625_GACGCAAGGACTGCGGTGCA	CRNKL1__NM_001278625	261	204	224	459
CRNKL1__NM_001278625_TCCGTCACGGCTCAAGCGAG	CRNKL1__NM_001278625	405	396	454	715
CRNKL1__NM_001278625_TCTCGCTTGAGCCGTGACGG	CRNKL1__NM_001278625	443	653	580	760
CRNKL1__NM_001278625_TGCGTCGCTGCTAGTCAGCA	CRNKL1__NM_001278625	196	162	184	163
CRNKL1__NM_001278626_AACTCGAGGCAGCGTTGTTA	CRNKL1__NM_001278626	609	51	596	152
CRNKL1__NM_001278626_AAGAAGTTTGGTGATAGGCG	CRNKL1__NM_001278626	45	15	76	20
CRNKL1__NM_001278626_ACGTACATGGAGGAAATGTT	CRNKL1__NM_001278626	268	28	301	34
CRNKL1__NM_001278626_ATCCACACAATTATGATGCA	CRNKL1__NM_001278626	356	11	445	5
CRNKL1__NM_001278626_ATTCTTTGGAGATGAACATA	CRNKL1__NM_001278626	11	0	54	10
CRNKL1__NM_001278626_CAACATGCTCGAAATATCT	CRNKL1__NM_001278626	664	597	691	851
CRNKL1__NM_001278626_CCCGGCAGGTGTTTGAGCGC	CRNKL1__NM_001278626	577	74	636	14
CRNKL1__NM_001278626_CTCATACACTTTCCGTGCAT	CRNKL1__NM_001278626	731	555	825	571
CRNKL1__NM_001278626_CTTTGAGCTGAGATACAAAG	CRNKL1__NM_001278626	115	101	175	143
CRNKL1__NM_001278626_GAGGCAGCGTTGTTATGGCC	CRNKL1__NM_001278626	302	6	385	42
CRNKL1__NM_001278626_TATTTCGAGCATGGTTGACT	CRNKL1__NM_001278626	205	14	237	27
CRNKL1__NM_001278626_TCTCATACACTTTCCGTGCA	CRNKL1__NM_001278626	495	228	505	370
CRNKL1__NM_001278628_ACAATGGGAAGAAAGCCTAA	CRNKL1__NM_001278628	125	6	79	11
CRNKL1__NM_001278628_ACTACCGAAATATTACACTC	CRNKL1__NM_001278628	75	0	58	0
CRNKL1__NM_001278628_ACTGGATAAAATACGCACAA	CRNKL1__NM_001278628	471	128	587	224
CRNKL1__NM_001278628_ATCTAAAGCACGCTCGTATA	CRNKL1__NM_001278628	128	0	168	11
CRNKL1__NM_001278628_CTGGATAAAATACGCACAAT	CRNKL1__NM_001278628	301	52	338	35
CRNKL1__NM_016652_CTCCGTTCTTACTCCCTTGC	CRNKL1__NM_016652	612	736	704	671
CRNN__NM_016190_AGGAGGACACACTCAGATAC	CRNN__NM_016190	577	489	567	539
CRNN__NM_016190_CAACTTTAAACACTAAGACC	CRNN__NM_016190	312	210	411	338
CRNN__NM_016190_CACAGTCTCACCTGTCTGGT	CRNN__NM_016190	2076	2004	1876	2225
CRNN__NM_016190_CGTCCTTGCATAGCGCCTGA	CRNN__NM_016190	81	48	67	53
CRNN__NM_016190_CTGGCTCCTGCCTTGACCGT	CRNN__NM_016190	634	755	762	634
CRNN__NM_016190_CTTAGTGTTTAAAGTTGCCC	CRNN__NM_016190	153	189	170	118
CRNN__NM_016190_GCCACCAATGACCAGAACAG	CRNN__NM_016190	642	699	745	889
CRNN__NM_016190_GCCCAGAGAGTTGTATCTGC	CRNN__NM_016190	1172	959	1188	1050
CRNN__NM_016190_GCCTGCGGCTCTCAAGAGTC	CRNN__NM_016190	22	17	10	13
CRNN__NM_016190_GGGACTGAGACCCACGGTCA	CRNN__NM_016190	261	343	300	393
CRNN__NM_016190_GGGACTGAGATCCACGGTCA	CRNN__NM_016190	714	554	681	689
CRNN__NM_016190_GGTGAGACTGTGACTGGATC	CRNN__NM_016190	715	869	858	1052
CROCC__NM_014675_AATGGCGAGCTCATCGCGCT	CROCC__NM_014675	774	505	774	477
CROCC__NM_014675_CAGCCTGCCTGCCCTTATCA	CROCC__NM_014675	323	135	334	177
CROCC__NM_014675_CGCCAGCCCCAAGCTCATGG	CROCC__NM_014675	678	397	701	476
CROCC__NM_014675_GACACTAGAGACGGTTATCC	CROCC__NM_014675	89	112	166	97
CROCC__NM_014675_GCTCGCCATTAAGTACAATG	CROCC__NM_014675	380	278	611	510
CROCC__NM_014675_GCTGACCGCATTGTACTTAA	CROCC__NM_014675	628	625	691	552
CROCC__NM_014675_GGAGAAAGGCTTGGGCGCGC	CROCC__NM_014675	672	536	820	581
CROCC__NM_014675_GGCACCTGGGCTCTCAGGCT	CROCC__NM_014675	1169	474	1177	451
CROCC__NM_014675_GGGGCTGGTACGGCAGAGCG	CROCC__NM_014675	59	32	42	19
CROCC__NM_014675_TACGGCAGAGCGTGGAGTTG	CROCC__NM_014675	275	323	311	264
CROCC__NM_014675_TCGCCAGCCCCAAGCTCATG	CROCC__NM_014675	349	464	482	565
CROCC__NM_014675_TCTCCCTGATAAGGGCAGGC	CROCC__NM_014675	561	520	580	538
CROT__NM_001143935_AAAGTGCCTGTTCATAAAGT	CROT__NM_001143935	979	1294	1001	1189
CROT__NM_001143935_AAGTTGACATTCAAGTTGTGA	CROT__NM_001143935	382	596	456	495
CROT__NM_001143935_ACAGCACTACAATGTGGTTT	CROT__NM_001143935	229	296	266	344
CROT__NM_001143935_AGAACGAACATTTCAGTACC	CROT__NM_001143935	255	356	315	297
CROT__NM_001143935_CAATTGAATGTCAACTTTGC	CROT__NM_001143935	226	260	265	395
CROT__NM_001143935_CACAGCACTACAATGTGGTT	CROT__NM_001143935	60	132	46	105
CROT__NM_001143935_CTCGACACAGCACTACAATG	CROT__NM_001143935	352	455	331	274
CROT__NM_001143935_GATTGCAGCATTAACTAGTG	CROT__NM_001143935	380	582	554	775
CROT__NM_001143935_GTATTTCCAACTTTATGAAC	CROT__NM_001143935	82	260	150	145
CROT__NM_001143935_GTGGCTGAATGTTGCCTATC	CROT__NM_001143935	155	239	190	290
CROT__NM_001143935_TGGCAGCTATTAAGAAAGTA	CROT__NM_001143935	70	37	50	15
CROT__NM_001143935_TTTGGCATAACTTGAACTAC	CROT__NM_001143935	70	119	123	145
CRP__NM_000567_AAGCTGAGAGGACGTGAACC	CRP__NM_000567	700	674	853	888
CRP__NM_000567_AAGGATTGAGTTATGGACTT	CRP__NM_000567	143	79	154	276
CRP__NM_000567_ACAGCCCCACAAGGTTCGTG	CRP__NM_000567	96	79	57	48
CRP__NM_000567_ACCATGCAGTGTAAAAAAGC	CRP__NM_000567	368	395	348	539
CRP__NM_000567_ACGAGAAAATACTGTACCCA	CRP__NM_000567	501	379	541	528
CRP__NM_000567_AGCAGGCCTGCAATGCATAT	CRP__NM_000567	750	947	633	737
CRP__NM_000567_ATCCTTGGGAAGACTGTAGT	CRP__NM_000567	312	188	337	328
CRP__NM_000567_ATGCGGTGTAAAAAACCGGG	CRP__NM_000567	157	139	186	132
CRP__NM_000567_CAAGCTGGGAGTCCGCCTCA	CRP__NM_000567	1145	1091	1104	870
CRP__NM_000567_CACACAGTGAAGGCTTTGAG	CRP__NM_000567	1738	2056	1936	2228
CRP__NM_000567_CAGGAGCAGGATTCCTTCGG	CRP__NM_000567	1067	674	1134	738
CRP__NM_000567_CAGTACACATTTGTACAAGC	CRP__NM_000567	680	670	784	976
CRP__NM_000567_CCAGAACTCCACGATCCCTG	CRP__NM_000567	1715	1179	1454	1037
CRP__NM_000567_CCCGCATTCCCAATATACCC	CRP__NM_000567	676	599	627	592
CRP__NM_000567_CGAGAAAATACTGTACCCAC	CRP__NM_000567	598	432	652	452
CRP__NM_000567_CGGAACTGTCCTCGACCCGT	CRP__NM_000567	1850	1346	1680	1171
CRP__NM_000567_CTCCCCAGCTCCCTATCTGG	CRP__NM_000567	788	313	701	392
CRP__NM_000567_CTCCCTATCTGGAGGATAGT	CRP__NM_000567	582	614	567	629
CRP__NM_000567_CTCTTGTGGCCTGGGTATAT	CRP__NM_000567	200	248	287	436
CRP__NM_000567_CTGAAGAAGGGATACACTGT	CRP__NM_000567	610	442	623	390
CRP__NM_000567_CTGGGTATATTGGGAATGCG	CRP__NM_000567	407	370	400	384
CRP__NM_000567_CTTTATTGGCTTATAGACCT	CRP__NM_000567	334	455	340	432
CRP__NM_000567_GAGAATGGAGGTAAAGTGTC	CRP__NM_000567	347	418	408	357
CRP__NM_000567_GATAGTTACGTGTTCCTAGC	CRP__NM_000567	173	200	179	233
CRP__NM_000567_GCCCATGCGGTGTAAAAAAC	CRP__NM_000567	182	247	212	371
CRP__NM_000567_GCTTCGTTAACGGTGCTTTG	CRP__NM_000567	407	399	456	635
CRP__NM_000567_GGGAGCTCGTTAACTATGCT	CRP__NM_000567	143	123	128	235
CRP__NM_000567_GTGGTTCACGTCCACTCTTG	CRP__NM_000567	0	0	0	0
CRP__NM_000567_TCCTTCAAAGTTCCCACCGA	CRP__NM_000567	875	437	1026	877
CRP__NM_000567_TCGTGGAGTTCTGGGTAGAT	CRP__NM_000567	1148	1248	1311	1137
CRP__NM_000567_TGCTGGATTTCCAAGCTGAG	CRP__NM_000567	65	31	101	113
CRTAC1__NM_001206528_CAAGTTCCGCAATAACCGGT	CRTAC1__NM_001206528	2668	2462	2850	1771
CRTAC1__NM_001206528_CACAGCCTGCGACATCGACG	CRTAC1__NM_001206528	700	639	657	651
CRTAC1__NM_001206528_CCTCTTTGCCGGACGCTCTG	CRTAC1__NM_001206528	213	238	200	315
CRTAC1__NM_001206528_CCTGAGCGATGAGGTCAACG	CRTAC1__NM_001206528	295	360	396	434
CRTAC1__NM_001206528_CGGTCCCGCAGCGCGTAGTA	CRTAC1__NM_001206528	305	311	415	511
CRTAC1__NM_001206528_GCCGTCCCCGTCGATGTCGC	CRTAC1__NM_001206528	2795	3855	2630	3570
CRTAC1__NM_001206528_GCTCACCCTACTACGCGCTG	CRTAC1__NM_001206528	855	1335	921	1392
CRTAC1__NM_001206528_GTCACAGCCTGCGACATCGA	CRTAC1__NM_001206528	627	687	651	905
CRTAC1__NM_001206528_TACATTGCCAATTACGCCTA	CRTAC1__NM_001206528	674	764	735	826
CRTAC1__NM_001206528_TCAAGTTCCGCAATAACCGG	CRTAC1__NM_001206528	411	617	502	564
CRTAC1__NM_001206528_TGTCTTCCCACCGGTTATTG	CRTAC1__NM_001206528	278	94	340	243
CRTAC1__NM_001206528_TGTTCAAGTTCCGCAATAAC	CRTAC1__NM_001206528	645	444	879	777
CRTAM__NM_019604_AGATTACTAAATCACATGAG	CRTAM__NM_019604	264	284	298	258
CRTAM__NM_019604_AGCCCCCTCCGCAGATAACC	CRTAM__NM_019604	674	725	808	769
CRTAM__NM_019604_AGTAGCCAGGTTATCTGCGG	CRTAM__NM_019604	2230	1088	2123	1390
CRTAM__NM_019604_AGTGTGGATTATGAGAGTGC	CRTAM__NM_019604	241	195	246	352
CRTAM__NM_019604_CAATGGAACATGTGATTTCA	CRTAM__NM_019604	474	377	587	743
CRTAM__NM_019604_CACTTATGGCAAAAATTCAA	CRTAM__NM_019604	256	308	364	484
CRTAM__NM_019604_CAGCGACTCTGTAAGCACAA	CRTAM__NM_019604	590	408	646	635
CRTAM__NM_019604_CATCCTACCTCAAATGGAAC	CRTAM__NM_019604	621	738	516	612
CRTAM__NM_019604_CCATCTTGCATTGCACTAAG	CRTAM__NM_019604	386	526	339	465
CRTAM__NM_019604_CCGCAGATAACCTGGCTACT	CRTAM__NM_019604	1918	1766	1908	1678
CRTAM__NM_019604_CCGGGGCAGATCATTATCCG	CRTAM__NM_019604	824	622	974	944
CRTAM__NM_019604_CTGATTATTGACCTAATGGA	CRTAM__NM_019604	332	357	381	509
CRTAM__NM_019604_GAGGATTCTACGAAGCCTTG	CRTAM__NM_019604	135	93	158	156
CRTAM__NM_019604_GCTTCCAGGATTGGCTTGAA	CRTAM__NM_019604	544	823	485	496
CRTAM__NM_019604_GGAAGTGAAAGTGATTGTGC	CRTAM__NM_019604	307	261	287	385
CRTAM__NM_019604_TAGCATGGAAGTGTCCGGTA	CRTAM__NM_019604	291	200	329	297
CRTAM__NM_019604_TCATCTTGCAGGGTTACGTT	CRTAM__NM_019604	710	599	890	1070
CRTAM__NM_019604_TCCCATCAGTTTCAAATTCA	CRTAM__NM_019604	626	717	749	954
CRTAM__NM_019604_TTATGGCAAAAATTCAACGG	CRTAM__NM_019604	169	104	191	128
CRTAP__NM_006371_AAACTGCAAGTAATGATACA	CRTAP__NM_006371	236	190	280	221
CRTAP__NM_006371_AACCTCACCCCAGTTATAGG	CRTAP__NM_006371	523	417	531	553
CRTAP__NM_006371_ACCGGATAGCCTCCTATAAC	CRTAP__NM_006371	766	334	680	423
CRTAP__NM_006371_AGCGGCATCAGCTCGTCCCG	CRTAP__NM_006371	249	268	294	239
CRTAP__NM_006371_CCCAGTTATAGGAGGCTATC	CRTAP__NM_006371	619	562	795	617
CRTAP__NM_006371_CCCGCACTGCTCGGATGAAC	CRTAP__NM_006371	960	665	862	866
CRTAP__NM_006371_CGAGCAGTGCGGGCATACAA	CRTAP__NM_006371	325	147	363	262
CRTAP__NM_006371_GAGGCGCAGCTCGGGATAGC	CRTAP__NM_006371	298	236	294	181
CRTAP__NM_006371_GCAGGAACTTGTAGGGCTCG	CRTAP__NM_006371	1161	991	1383	1221
CRTAP__NM_006371_GCGGCATCAGCTCGTCCCGT	CRTAP__NM_006371	268	125	385	218
CRTAP__NM_006371_GGAAGCTGTAGCGTTCGTAT	CRTAP__NM_006371	927	895	1253	1058
CRTAP__NM_006371_GGCTTTGAAGAAGTCGGGAA	CRTAP__NM_006371	696	501	653	936
CRTC1__NM_001098482_ACTCCCGATCTGGTTCACGT	CRTC1__NM_001098482	1817	1513	2113	1615
CRTC1__NM_001098482_AGCCGAGGCCAGTACTATGG	CRTC1__NM_001098482	1030	688	989	675
CRTC1__NM_001098482_ATCTTCTCGCTGAATTTCCG	CRTC1__NM_001098482	125	223	169	145
CRTC1__NM_001098482_CCCAGCCGAGGCCAGTACTA	CRTC1__NM_001098482	487	285	416	437
CRTC1__NM_001098482_CTGCCCAACGTGAACCAGAT	CRTC1__NM_001098482	588	582	634	992
CRTC1__NM_001098482_GATCTGGTTCACGTTGGGCA	CRTC1__NM_001098482	367	181	364	373
CRTC1__NM_001098482_GCCTCCCCCAGAAATCCGCT	CRTC1__NM_001098482	1795	2163	2462	2552
CRTC1__NM_001098482_GGCCTCCCCCAGAAATCCGC	CRTC1__NM_001098482	871	1146	1026	1079
CRTC1__NM_001098482_GTCCAGGCCCGAGGATTGGA	CRTC1__NM_001098482	250	305	351	329
CRTC1__NM_001098482_TGGGGCCCAGTTGCAGGTAC	CRTC1__NM_001098482	315	152	211	185
CRTC1__NM_001098482_TGGTGTCCAGGCCCGAGGAT	CRTC1__NM_001098482	38	4	62	38
CRTC1__NM_001098482_TGTCAGTGGACAAACACGGA	CRTC1__NM_001098482	205	265	148	170
CRTC1__NM_015321_AAAATAAAATAGAAGCCACC	CRTC1__NM_015321	197	299	278	348
CRTC1__NM_015321_AAATTAACAGCATCTATGAC	CRTC1__NM_015321	876	828	857	942
CRTC1__NM_015321_AACAGTCGGAAAATGCGCCA	CRTC1__NM_015321	429	401	403	388
CRTC1__NM_015321_ACCAGGGTGACAGGGTACTT	CRTC1__NM_015321	494	296	516	243
CRTC1__NM_015321_ACTCACTTCTTACACTCAGG	CRTC1__NM_015321	1210	508	1296	799
CRTC1__NM_015321_AGAATCACAAGCTCGGCCGT	CRTC1__NM_015321	355	519	445	539
CRTC1__NM_015321_AGACCCTCTGCTGGGTACAT	CRTC1__NM_015321	946	889	979	978
[illegible]	[illegible]	[illegible]	[illegible]	[illegible]	[illegible]

CTBP1__NM_001328_TTCGTGATGCCGTCTTCATT	CTBP1__NM_001328	168	252	160	186
CTBP2__NM_001083914_ACTTGCAGGATGGGATCGAG	CTBP2__NM_001083914	556	731	600	603
CTBP2__NM_001083914_ATCAAGGCTGCCGGCGAGCT	CTBP2__NM_001083914	464	487	513	522
CTBP2__NM_001083914_CGCCACGGGCTGCGTTCACA	CTBP2__NM_001083914	995	320	1255	578
CTBP2__NM_001083914_CTCCTTGAGGGCTTGTGCTA	CTBP2__NM_001083914	502	501	547	569
CTBP2__NM_001083914_CTGAGAGTGATCGTGCGGAT	CTBP2__NM_001083914	1274	1586	1306	1482
CTBP2__NM_001083914_CTGTGACGCGCAGTCGACGC	CTBP2__NM_001083914	50	70	33	84
CTBP2__NM_001083914_GACTTTGTGCTTATCCACAA	CTBP2__NM_001083914	492	204	434	186
CTBP2__NM_001083914_GCAGGGGGCCGTTCATGATC	CTBP2__NM_001083914	804	337	921	659
CTBP2__NM_001083914_GCAGGTTCTAAACGAAGCCG	CTBP2__NM_001083914	113	76	109	210
CTBP2__NM_001083914_GCATCTCCACAGTGCAGTCG	CTBP2__NM_001083914	906	1094	879	1317
CTBP2__NM_001083914_GGCGGTTGCAGTTCGAGCCA	CTBP2__NM_001083914	418	479	452	561
CTBP2__NM_001083914_TCAAGGAGGGCAGGATACGA	CTBP2__NM_001083914	300	118	308	139
CTBP2__NM_022802_ACAGGACGCTGCCCTGCCCG	CTBP2__NM_022802	134	253	224	291
CTBP2__NM_022802_ACCAATATTTATATGCCTGC	CTBP2__NM_022802	654	848	677	861
CTBP2__NM_022802_ACGGCCTCCCGGTACAGGTA	CTBP2__NM_022802	789	1260	853	742
CTBP2__NM_022802_AGGAGAAGCTCCCTGACGTA	CTBP2__NM_022802	412	316	372	345
CTBP2__NM_022802_CAAAGAGCCTCCCCTCTATC	CTBP2__NM_022802	2282	2667	2666	3068
CTBP2__NM_022802_CCTCTGCTGTGCCATACGTC	CTBP2__NM_022802	676	849	838	1106
CTBP2__NM_022802_CCTGGGTCCCGATAGAGGGG	CTBP2__NM_022802	215	423	280	147
CTBP2__NM_022802_GATGCTGCTGGGTGGTACGA	CTBP2__NM_022802	609	629	582	621
CTBP2__NM_022802_GCGTCCTGTGGTCGGTGGTT	CTBP2__NM_022802	1275	1480	1320	1683
CTBP2__NM_022802_GGATCACTGTAGTACTCCCG	CTBP2__NM_022802	678	792	810	863
CTBP2__NM_022802_GGATGCTGCTGGGTGGTACG	CTBP2__NM_022802	782	936	868	1159
CTBP2__NM_022802_TGCAGCCACTGAGTTATAAA	CTBP2__NM_022802	208	142	206	261
CTBS__NM_004388_AAATCTGGATGGTGGCGAAT	CTBS__NM_004388	144	155	331	294
CTBS__NM_004388_AGGATCAATGATATCCTTTA	CTBS__NM_004388	607	557	775	767
CTBS__NM_004388_ATGATGAACAAAGTCAGATC	CTBS__NM_004388	235	266	282	257
CTBS__NM_004388_ATGTGCTACGCTCATTCAAA	CTBS__NM_004388	73	62	90	118
CTBS__NM_004388_CACAGGAGATGTATCCTTAA	CTBS__NM_004388	1106	1503	1475	1654
CTBS__NM_004388_CATACCTTTAAGTACTACTC	CTBS__NM_004388	281	343	302	430
CTBS__NM_004388_CTGGATGGTGGCGAATCGGG	CTBS__NM_004388	316	235	219	213
CTBS__NM_004388_GCAGTTAATGCATCATATTC	CTBS__NM_004388	398	386	399	489
CTBS__NM_004388_GCTACATCAAAGGTTACCTG	CTBS__NM_004388	647	649	814	623
CTBS__NM_004388_GGAGCCAGAGTAGTACTTAA	CTBS__NM_004388	376	520	476	575
CTBS__NM_004388_GTGATCCCTCAATTTCACGA	CTBS__NM_004388	584	469	695	611
CTBS__NM_004388_TTTGGCCAAAACACAATATA	CTBS__NM_004388	84	62	111	169
CTC1__NM_025099_AACAGACCTATCGGCAGACT	CTC1__NM_025099	373	389	448	273
CTC1__NM_025099_CAATTACCAATGGAGTCAAC	CTC1__NM_025099	354	492	368	400
CTC1__NM_025099_CAGACTTGGAACAAGAGTGC	CTC1__NM_025099	67	32	98	54
CTC1__NM_025099_CAGGAGGGAGGTAACTCCAA	CTC1__NM_025099	490	4	616	27
CTC1__NM_025099_CTGCCCCTCAGCTATAGGTA	CTC1__NM_025099	232	30	260	170
CTC1__NM_025099_CTTGTTCCAAGTCTGCCGAT	CTC1__NM_025099	596	649	556	495
CTC1__NM_025099_GAGGGAGGTAACTCCAACGG	CTC1__NM_025099	131	0	143	13
CTC1__NM_025099_GGAGGGAGGTAACTCCAACG	CTC1__NM_025099	154	17	241	33
CTC1__NM_025099_GTCAAAGGAAACACTGGCAC	CTC1__NM_025099	522	84	747	158
CTC1__NM_025099_GTGGAGCAGTAGTGCATACC	CTC1__NM_025099	127	12	115	21
CTC1__NM_025099_TAATGTCCAGTTGACTCCAT	CTC1__NM_025099	976	533	945	515
CTC1__NM_025099_TGAGGGGCAGTGTAGAACCT	CTC1__NM_025099	443	60	380	89
CTCF__NM_001191022_AAAGCCGCAGGGACGAAGTT	CTCF__NM_001191022	553	223	688	455
CTCF__NM_001191022_AAGCCGCAGGGACGAAGTTG	CTCF__NM_001191022	474	131	488	253
CTCF__NM_001191022_ATGCCGAACCAATTCTCCAC	CTCF__NM_001191022	637	0	891	5
CTCF__NM_001191022_CGTCACATTCGCTCTCATAC	CTCF__NM_001191022	645	185	549	298
CTCF__NM_001191022_GGCGGAAGGTCTTATCGCAG	CTCF__NM_001191022	842	502	953	604
CTCF__NM_001191022_GTCTACAAGCGTAATCACAC	CTCF__NM_001191022	233	1	219	35
CTCF__NM_001191022_GTGGGAAAACATTTACACGT	CTCF__NM_001191022	177	28	245	67
CTCF__NM_001191022_GTGTGATTACGCTTGTAGAC	CTCF__NM_001191022	259	22	310	46
CTCF__NM_001191022_GTTTGTGCAGTTATGCCAGC	CTCF__NM_001191022	320	399	302	442
CTCF__NM_001191022_TACACTGGCGTAATCGCACA	CTCF__NM_001191022	310	8	441	7
CTCF__NM_001191022_TTAGAACAGACAAAAGCCGC	CTCF__NM_001191022	224	130	251	196
CTCF__NM_001191022_TTTCTTGCCTTGCTCAATAT	CTCF__NM_001191022	97	132	117	170
CTCF__NM_006565_AGGTGGAGACACTAGAACAA	CTCF__NM_006565	59	17	77	41
CTCF__NM_006565_CAACCTGTAAAGTTATAATC	CTCF__NM_006565	451	131	556	240
CTCF__NM_006565_CACCCCAGGAAGATCCTAGT	CTCF__NM_006565	1978	443	2164	924
CTCF__NM_006565_GAAAATGAAGTGTCTAAAGA	CTCF__NM_006565	281	110	277	203
CTCF__NM_006565_GAGGAACAGCCCATAAACAT	CTCF__NM_006565	620	354	755	526
CTCF__NM_006565_GATGAAGACTGAAGTAATGG	CTCF__NM_006565	384	140	441	266
CTCF__NM_006565_GGTTTCAGGTGGTTAAAGTG	CTCF__NM_006565	104	17	133	141
CTCF__NM_006565_TACCCAGATTATAACTTTAC	CTCF__NM_006565	253	48	338	114
CTCF__NM_006565_TGAAAATGAAGTGTCTAAAG	CTCF__NM_006565	170	183	191	115
CTCF__NM_006565_TGAAGTTCTCCTATGTTTAT	CTCF__NM_006565	127	145	156	133
CTCF__NM_006565_TGTCAACAGCAGTGTACAGA	CTCF__NM_006565	319	195	405	147
CTCF__NM_006565_TTACCCCAGAACCAGACGGA	CTCF__NM_006565	167	136	239	139
CTCFL__NM_001269040_CAACACCTCCATCTCTTGCG	CTCFL__NM_001269040	2768	2698	3307	3516
CTCFL__NM_001269040_CAAGAGGATCAACCTACAGC	CTCFL__NM_001269040	469	415	498	488
CTCFL__NM_001269040_CAGCTCTTGCTGGATACTAA	CTCFL__NM_001269040	345	314	362	382
CTCFL__NM_001269040_CAGTGAAGACAGTAAGTTAG	CTCFL__NM_001269040	529	468	630	795
CTCFL__NM_001269040_CCAACTCACTAGGGCTCCGA	CTCFL__NM_001269040	1237	1357	1279	1373
CTCFL__NM_001269040_CCATCGGAGCCCTAGTGAGT	CTCFL__NM_001269040	1762	1637	1633	1760
CTCFL__NM_001269040_GAAGGCCCCAGAGGTACGCT	CTCFL__NM_001269040	494	354	553	656
CTCFL__NM_001269040_GCATCTGCTTGACCAGCTGT	CTCFL__NM_001269040	1597	1337	1949	1766
CTCFL__NM_001269040_GTTGCAGTTCCACGCTCTAG	CTCFL__NM_001269040	501	283	533	406
CTCFL__NM_001269040_TCGGAGCCCTAGTGAGTTGG	CTCFL__NM_001269040	697	694	754	818
CTCFL__NM_001269040_TGAACATGCAGACATCACAG	CTCFL__NM_001269040	412	200	411	579
CTCFL__NM_001269040_TTCTGAAGCTGTGGAGTTGC	CTCFL__NM_001269040	11	25	12	14
CTCFL__NM_001269049_AAAACTTGACATTCTAGAAG	CTCFL__NM_001269049	249	225	255	371
CTCFL__NM_001269049_CAAGTGTAACGACTGCAACA	CTCFL__NM_001269049	448	493	580	629
CTCFL__NM_001269049_CACACTGGCATACTTGCACA	CTCFL__NM_001269049	630	618	808	899
CTCFL__NM_001269049_CAGCAGAGTGACCGTACGGA	CTCFL__NM_001269049	56	43	73	70
CTCFL__NM_001269049_CTGCCTGAAAACCTTCCGTA	CTCFL__NM_001269049	687	745	859	1010
CTCFL__NM_001269049_GTCGTTACACTTGTAGGGCC	CTCFL__NM_001269049	153	361	225	221
CTCFL__NM_001269049_GTGGAGAACTCGTCCGACAC	CTCFL__NM_001269049	381	382	342	179
CTCFL__NM_001269049_TACCTGTGTGGGTGTTAACA	CTCFL__NM_001269049	240	103	284	109
CTCFL__NM_001269049_TCCGCAGCAGAGTGACCGTA	CTCFL__NM_001269049	444	589	369	430
CTCFL__NM_001269054_CCCCAGTGTGGGATCGGACA	CTCFL__NM_001269054	774	638	751	718
CTCFL__NM_001269054_GCCATGTCCGATCCCACACT	CTCFL__NM_001269054	1756	1370	1986	1531
CTCFL__NM_001269054_TGCACTGGCAACACTGAAAG	CTCFL__NM_001269054	151	195	219	381
CTDNEP1__NM_001143775_AAGCCCCTGGGGAGTTATCC	CTDNEP1__NM_001143775	599	121	735	330
CTDNEP1__NM_001143775_ACACCACCAGCTCGTACCAC	CTDNEP1__NM_001143775	595	173	589	241
CTDNEP1__NM_001143775_ATAACTCCCCAGGGGCTTAC	CTDNEP1__NM_001143775	109	81	86	41
CTDNEP1__NM_001143775_ATGGCTCCTGTAAGCCCCTG	CTDNEP1__NM_001143775	209	102	278	157
CTDNEP1__NM_001143775_CCTGTGTCCCGGAATCGGCT	CTDNEP1__NM_001143775	1374	435	1450	841
CTDNEP1__NM_001143775_GATCCTGGATAACTCCCCAG	CTDNEP1__NM_001143775	1233	384	1416	610
CTDNEP1__NM_001143775_GATGGGGATGGCATTGTCTA	CTDNEP1__NM_001143775	477	195	566	286
CTDNEP1__NM_001143775_GCTTCTTCATTTACCTTCTG	CTDNEP1__NM_001143775	396	465	572	654
CTDNEP1__NM_001143775_GGTGAGCCAGTGGTACGAGC	CTDNEP1__NM_001143775	250	73	273	108
CTDNEP1__NM_001143775_GGTGGTGTTTACAGCAAGCA	CTDNEP1__NM_001143775	0	0	0	0
CTDNEP1__NM_001143775_TAAGAGGAGATATTACAGAC	CTDNEP1__NM_001143775	688	236	754	514
CTDNEP1__NM_001143775_TTATGTACAAAAAACCGGAC	CTDNEP1__NM_001143775	103	59	110	91
CTDNEP1__NM_015343_ACTCCATGGAAACCCCAGAC	CTDNEP1__NM_015343	554	614	652	616
CTDNEP1__NM_015343_CCCCCCCACCCCAACTCAGG	CTDNEP1__NM_015343	2576	2335	3035	2228
CTDNEP1__NM_015343_CTGGGCTGTGGGTGGGATAC	CTDNEP1__NM_015343	457	781	564	602
CTDNEP1__NM_015343_GGCATCAGACGGCATCCCAA	CTDNEP1__NM_015343	513	258	610	455
CTDNEP1__NM_015343_TGACAGACATACGGTGATCC	CTDNEP1__NM_015343	309	200	248	184
CTDNEP1__NM_015343_TGCCCAGGCAGTCCTCACAT	CTDNEP1__NM_015343	1750	1096	1778	890
CTDP1__NM_001202504_ACTGCACCGAAACCGGAAGC	CTDP1__NM_001202504	312	48	320	34
CTDP1__NM_001202504_AGACCCTGAGGGGGTAACGC	CTDP1__NM_001202504	570	542	553	342
CTDP1__NM_001202504_AGTCAGTGTGTACACGGACC	CTDP1__NM_001202504	287	0	305	7
CTDP1__NM_001202504_CACAGAACAGCGAGCTGTCG	CTDP1__NM_001202504	733	980	674	1073
CTDP1__NM_001202504_CCCCAGGGAGCCTCTGTGCGCA	CTDP1__NM_001202504	1783	878	1742	903
CTDP1__NM_001202504_CGGTTGTGTGAATCAACGTC	CTDP1__NM_001202504	187	95	218	101
CTDP1__NM_001202504_GGCCAGATGTCCCTCTCGTC	CTDP1__NM_001202504	1440	2258	1582	1944
CTDP1__NM_001202504_GGGCCTGCGTTACCCCCTCA	CTDP1__NM_001202504	268	296	259	246
CTDP1__NM_001202504_TCCTCCTCCTCCGCCTCTGA	CTDP1__NM_001202504	1255	445	1166	356
CTDP1__NM_001202504_TGCAGGTTGCAGAGTAAGAA	CTDP1__NM_001202504	88	100	78	110
CTDP1__NM_001202504_TGGGCAACGGCTGTGCCGAC	CTDP1__NM_001202504	23	14	60	19
CTDP1__NM_001202504_TTCTCACCGAATATTATCAA	CTDP1__NM_001202504	0	0	2	0
CTDP1__NM_004715_CAGTCCCGTGTAGCCTCCGG	CTDP1__NM_004715	1775	776	1438	702
CTDP1__NM_004715_CGCAGCCCCCGGAGGCTACA	CTDP1__NM_004715	595	187	526	235
CTDP1__NM_004715_GGCCTTTCATGACAACCGGG	CTDP1__NM_004715	466	0	380	7
CTDP1__NM_004715_TAGAGCGGTTCTGGTGAGGT	CTDP1__NM_004715	962	6	820	16
CTDSP1__NM_001206878_ACCACGAGGCCACCGGTACC	CTDSP1__NM_001206878	891	1075	1165	1227
CTDSP1__NM_001206878_ACGCTTCAACACGTAGACCT	CTDSP1__NM_001206878	578	489	688	448
CTDSP1__NM_001206878_ACGGCTGAGTTGCTCGAAGA	CTDSP1__NM_001206878	81	186	101	82
CTDSP1__NM_001206878_CAGCTACTGGGTCTGCGTAC	CTDSP1__NM_001206878	643	855	823	762
CTDSP1__NM_001206878_CCACGAGGCCACCGGTACCT	CTDSP1__NM_001206878	132	31	105	96
CTDSP1__NM_001206878_GATCTGCGTGGTCATCGACC	CTDSP1__NM_001206878	383	482	522	508
CTDSP1__NM_001206878_GCAGGACTCTCGAAACAGCC	CTDSP1__NM_001206878	345	235	437	412
CTDSP1__NM_001206878_GCTGTGTCTGCCGGGATGAT	CTDSP1__NM_001206878	1077	1162	1226	1165
CTDSP1__NM_001206878_GGACTCAGACAAGATCTGCG	CTDSP1__NM_001206878	380	261	424	352
CTDSP1__NM_001206878_GGAGAATGGCGCCATCCCTA	CTDSP1__NM_001206878	923	1051	1345	1216
CTDSP1__NM_001206878_TCAGGGAGCAGGTATTGGAC	CTDSP1__NM_001206878	254	295	248	177
CTDSP1__NM_001206878_TTGTCCAGCAGGTCAGCTAC	CTDSP1__NM_001206878	145	116	137	126
CTDSP2__NM_005730_ACTCACGGAATAGGCGGGCC	CTDSP2__NM_005730	563	766	597	855
CTDSP2__NM_005730_ATGAAGTCAGCATTGTTGAT	CTDSP2__NM_005730	107	47	175	140
CTDSP2__NM_005730_ATGAGTTCCTGAGACGCATG	CTDSP2__NM_005730	362	250	375	232
CTDSP2__NM_005730_CACTGAGCTCGCTGCGTATA	CTDSP2__NM_005730	643	783	764	846
CTDSP2__NM_005730_CCTTAAAGGAGCTATGCACA	CTDSP2__NM_005730	475	262	497	283
CTDSP2__NM_005730_CTCAGGTGTATGTGCTCAAG	CTDSP2__NM_005730	58	63	95	79
CTDSP2__NM_005730_GATGAGTTCCTGAGACGCAT	CTDSP2__NM_005730	227	212	244	174
CTDSP2__NM_005730_TAGTGCCTATAGAGATTGAG	CTDSP2__NM_005730	349	317	381	364
CTDSP2__NM_005730_TATGCACAAGGGTTTCATCG	CTDSP2__NM_005730	398	370	465	345
CTDSP2__NM_005730_TGAGCTCGCTGCGTATAAGG	CTDSP2__NM_005730	332	151	397	191
CTDSP2__NM_005730_TGTCACAGGGTCGGCATACT	CTDSP2__NM_005730	334	233	277	156
CTDSP2__NM_005730_TTGAAGATGTTACGTCCACG	CTDSP2__NM_005730	224	198	230	206
CTDSPL__NM_001008392_AATCGATGGAACTATACATC	CTDSPL__NM_001008392	145	100	142	149
CTDSPL__NM_001008392_ACACACCCCAGCGGTCTAGG	CTDSPL__NM_001008392	723	896	813	874
CTDSPL__NM_001008392_ATAGTTCCATCGATTTCAAC	CTDSPL__NM_001008392	91	26	108	112
CTDSPL__NM_001008392_ATGATGGCCGGGCCGTCCAT	CTDSPL__NM_001008392	378	154	363	270
CTDSPL__NM_001008392_ATTGTTCCGGTTGAAATCGA	CTDSPL__NM_001008392	339	440	352	401
CTDSPL__NM_001008392_GAGGTGACGGTGCTTGACTA	CTDSPL__NM_001008392	513	501	588	563
CTDSPL__NM_001008392_GATGGAAGATGTATGAGGCA	CTDSPL__NM_001008392	550	664	640	713
CTDSPL__NM_001008392_GGAAGAAGGTACTTAGCTGG	CTDSPL__NM_001008392	405	413	470	403
CTDSPL__NM_001008392_GGCTGACCTCCTAGACCGCT	CTDSPL__NM_001008392	1657	2498	1823	2254
CTDSPL__NM_001008392_TAGACCGCTGGGGTGTGTTC	CTDSPL__NM_001008392	537	357	536	290
CTDSPL__NM_001008392_TCTGGAAGAAGGTACTTAGC	CTDSPL__NM_001008392	268	236	218	111
CTDSPL__NM_001008392_TGACTATGGAAAGAAATGTG	CTDSPL__NM_001008392	121	233	189	133
CTDSPL2__NM_016396_AACAGATATCTCGAGTAAGA	CTDSPL2__NM_016396	190	122	245	116
CTDSPL2__NM_016396_AAGCCGGTTTCATTCTTCGA	CTDSPL2__NM_016396	1143	1044	1058	1016
CTDSPL2__NM_016396_AGACGGAAAAGTCAAGTAAA	CTDSPL2__NM_016396	108	57	104	63
CTDSPL2__NM_016396_AGCAAAGAGGAAATATTCAG	CTDSPL2__NM_016396	312	341	258	270
CTDSPL2__NM_016396_AGTACTACTACATCAACTAA	CTDSPL2__NM_016396	81	66	93	67
CTDSPL2__NM_016396_ATTAGTTGATGTAGTAGTAC	CTDSPL2__NM_016396	123	239	144	143
CTDSPL2__NM_016396_CGGAAAAGTCAAGTAAATGG	CTDSPL2__NM_016396	371	453	464	345
CTDSPL2__NM_016396_CTCCAAGGACTACTTTGTTG	CTDSPL2__NM_016396	759	894	927	1121
CTDSPL2__NM_016396_CTCTGGCAGTGCGTTGTGTT	CTDSPL2__NM_016396	1121	1672	1235	1563
CTDSPL2__NM_016396_GTGAGACCATCACTAAACAA	CTDSPL2__NM_016396	113	126	85	52
CTDSPL2__NM_016396_TCTAAACCATTGTTTAGTGA	CTDSPL2__NM_016396	590	1085	650	893
CTDSPL2__NM_016396_TTCCCAGTCTTCTTCATAGG	CTDSPL2__NM_016396	65	6	78	49
CTF1__NM_001142544_AGCCGCAGCCGCTCGTGCAC	CTF1__NM_001142544	1344	1291	1517	1576
CTF1__NM_001142544_GAGTGGCTGAGCCGCACCGA	CTF1__NM_001142544	628	501	625	743
CTF1__NM_001142544_GCTGCTCAGCGTATTTGGTG	CTF1__NM_001142544	534	662	366	292
CTF1__NM_001142544_GCTGTGTGTCTGACGGATCT	CTF1__NM_001142544	1477	1907	1691	1936
CTF1__NM_001142544_GGCTCAGCCACTCGCGGTAG	CTF1__NM_001142544	661	864	803	903
CTGF__NM_001901_AACCATGACCGCCGCCAGTA	CTGF__NM_001901	930	812	934	1161
CTGF__NM_001901_ACCAGTGCACGTGCCTGGAC	CTGF__NM_001901	499	343	566	677
CTGF__NM_001901_CAAGCTTTACATTCTACCTA	CTGF__NM_001901	1193	1981	1324	1362
CTGF__NM_001901_CCAGCTGCTTGGCGCAGACG	CTGF__NM_001901	281	263	289	248
CTGF__NM_001901_CCCAAGGACCAAACCGTGGT	CTGF__NM_001901	110	151	186	147
CTGF__NM_001901_CCGAGGAGGGTCAAGCTGCC	CTGF__NM_001901	1412	1630	1440	1900
CTGF__NM_001901_GACGCGGACGGGGCCCATAC	CTGF__NM_001901	245	283	295	255
CTGF__NM_001901_GACGGGGCCCATACTGGCGG	CTGF__NM_001901	286	310	371	266
CTGF__NM_001901_GAGACATTAACTCATTAGAC	CTGF__NM_001901	293	262	378	397
CTGF__NM_001901_GCATGAAATGGATACTTATA	CTGF__NM_001901	398	322	611	649
CTGF__NM_001901_GGCCCTTGTGCGGGTCGCAT	CTGF__NM_001901	376	433	436	605
CTGF__NM_001901_GGTGCTCCCTGCATCTTCGG	CTGF__NM_001901	1005	933	1146	1063
CTGF__NM_001901_GGTGGTACGGTGTACCGCAG	CTGF__NM_001901	29	20	8	55
CTGF__NM_001901_GTGGGTGTGTGACGAGCCCA	CTGF__NM_001901	88	89	112	110
CTGF__NM_001901_TCCCTGCATCTTCGGTGGTA	CTGF__NM_001901	338	403	311	447
CTGF__NM_001901_TGGCAGGCTGATTTCTAGGT	CTGF__NM_001901	466	409	437	286
CTH__NM_001190463_CAGTGATGTTGTAATGGGCC	CTH__NM_001190463	606	895	629	807
CTH__NM_001190463_CGCCCCTTGCTTGAACGTGG	CTH__NM_001190463	1490	1483	1607	1612
CTH__NM_001190463_CTGTCCACCACGTTCAAGCA	CTH__NM_001190463	877	987	967	1160
CTH__NM_001190463_CTTCCAACATTTCGCCACGC	CTH__NM_001190463	427	457	469	615
CTH__NM_001190463_GCACATATTGTCCATAAGCA	CTH__NM_001190463	336	317	416	443
CTH__NM_001190463_GCCACGCAGGCGATCCATGT	CTH__NM_001190463	1181	1465	1360	1513
CTH__NM_001190463_GCCCACATGGATCGCCTGCG	CTH__NM_001190463	1196	1098	1227	1260
CTH__NM_001190463_GTGTGGCAGGAAACCTTGTG	CTH__NM_001190463	623	725	644	723
CTH__NM_001190463_TCCAGAGCAATGGACCTCCA	CTH__NM_001190463	193	187	144	170
CTH__NM_001190463_TCGCCTGCGTGGCGAAATGT	CTH__NM_001190463	72	111	106	20
CTH__NM_001190463_TGTCCACCACGTTCAAGCAA	CTH__NM_001190463	556	639	649	578
CTH__NM_001190463_TTTCACAATTAACAGACACC	CTH__NM_001190463	154	291	175	212
CTH__NM_153742_AGAAACGAAGTCTATTATGA	CTH__NM_153742	344	356	445	399
CTHRC1__NM_001256099_AATGGCATTCCGGGTACACC	CTHRC1__NM_001256099	287	158	313	181
CTHRC1__NM_001256099_ACTTGTTCAGATTACCCAAA	CTHRC1__NM_001256099	424	277	385	388
CTHRC1__NM_001256099_CAGGAGTGCCTGGTCGAGAC	CTHRC1__NM_001256099	738	936	795	815
CTHRC1__NM_001256099_CATGAACACTGCTTGTAGTT	CTHRC1__NM_001256099	1202	1151	1286	1158
CTHRC1__NM_001256099_CTTTCCAGGGGCTCATCTGT	CTHRC1__NM_001256099	687	794	818	568
CTHRC1__NM_001256099_CTTTGAATCCATCCCGACCT	CTHRC1__NM_001256099	426	444	601	371
CTHRC1__NM_001256099_GCTATAATTTATTTGGACCA	CTHRC1__NM_001256099	379	353	421	389
CTHRC1__NM_001256099_TACACCTGGGATCCCAGGTC	CTHRC1__NM_001256099	208	87	194	53
CTHRC1__NM_001256099_TCATGGAGTTCATTGAATTA	CTHRC1__NM_001256099	503	503	537	496
CTHRC1__NM_001256099_TGAGCCCCTGGAAAGCACTC	CTHRC1__NM_001256099	550	555	651	504
CTIF__NM_001142397_ACCACCAGAAATCCTACCAG	CTIF__NM_001142397	911	880	1059	830
CTIF__NM_001142397_AGCACCCACCAGGCGACAAG	CTIF__NM_001142397	156	336	265	218
CTIF__NM_001142397_CACATCGACCGCGAAGGGTA	CTIF__NM_001142397	514	471	477	364
CTIF__NM_001142397_CCTCCCCCTTGTCGCCTGGT	CTIF__NM_001142397	3105	2774	3111	2667
CTIF__NM_001142397_GAAGCACCCACCAGGCGACA	CTIF__NM_001142397	199	155	245	260
CTIF__NM_001142397_GACCACCAGAAATCCTACCA	CTIF__NM_001142397	1045	1181	1110	1180
CTIF__NM_001142397_GAGGAGAAATGATCGAAGGT	CTIF__NM_001142397	467	527	408	354
CTIF__NM_001142397_GCAGGAGGAGAAATGATCGA	CTIF__NM_001142397	847	1238	914	984
CTIF__NM_001142397_GCTGCTGGCTGACAAGACGG	CTIF__NM_001142397	483	224	482	318
CTIF__NM_001142397_GGCAACATGAAGCACCCACC	CTIF__NM_001142397	729	791	598	880
CTIF__NM_001142397_TGCCACACATCGACCGCGAA	CTIF__NM_001142397	309	370	453	448
CTIF__NM_001142397_TGGGCGTGTGTCGGACTTTG	CTIF__NM_001142397	152	156	112	99
CTLA4__NM_001037631_AAAGAAGCCCTCTTACAACA	CTLA4__NM_001037631	221	254	305	180
CTLA4__NM_001037631_CAAGTGAACCTCACTATCCA	CTLA4__NM_001037631	753	691	913	1191
CTLA4__NM_001037631_CCGGGTGACAGTGCTTCGGC	CTLA4__NM_001037631	1392	1563	1552	1917
CTLA4__NM_001037631_CTCACCAATTACATAAATCT	CTLA4__NM_001037631	1969	2241	2308	2311
CTLA4__NM_001037631_CTGAAATCCAAGGCAAGCCA	CTLA4__NM_001037631	232	253	244	309
CTLA4__NM_001037631_GATGTAGAGTCCCGTGTCCA	CTLA4__NM_001037631	1365	1406	1392	1488
CTLA4__NM_001037631_GGGTTCCGTTGCCTATGCCC	CTLA4__NM_001037631	284	275	331	292
CTLA4__NM_001037631_TACCCACCGCCATACTACCT	CTLA4__NM_001037631	1333	1415	1397	1480
CTLA4__NM_001037631_TACTACCTGGGCATAGGCAA	CTLA4__NM_001037631	149	180	184	196
CTLA4__NM_001037631_TGGCTTGCCTTGGATTTCAG	CTLA4__NM_001037631	101	118	92	111
CTLA4__NM_001037631_TGTGCGGCAACCTACATGAT	CTLA4__NM_001037631	784	689	797	808
CTLA4__NM_005214_GATCCTTGCAGCAGTTAGTT	CTLA4__NM_005214	548	375	611	748
CTLA4__NM_005214_TCCTTGCAGCAGTTAGTTCG	CTLA4__NM_005214	635	513	642	696
CTNNA1__NM_001290307_ATCCTAAAAGTCTAGAGATC	CTNNA1__NM_001290307	110	195	127	121
CTNNA1__NM_001290307_ATTAGAGGGCCCTTTACTAT	CTNNA1__NM_001290307	793	606	810	644
CTNNA1__NM_001290307_CAACAGATGCAGCCAAAACA	CTNNA1__NM_001290307	336	286	369	411
CTNNA1__NM_001290307_CAGGCAACATAAACTTCAAG	CTNNA1__NM_001290307	265	320	315	265
CTNNA1__NM_001290307_CGACCTCTCTTCTTATTAGA	CTNNA1__NM_001290307	907	998	1084	1389
CTNNA1__NM_001290307_CTTGTAAACACCAATAGTAA	CTNNA1__NM_001290307	172	198	255	230
CTNNA1__NM_001290307_GAAGAGAGGTCGTTCTAAGA	CTNNA1__NM_001290307	182	226	202	111
CTNNA1__NM_001290307_GAAGGGGGATAAAATTGCGA	CTNNA1__NM_001290307	338	295	490	340
CTNNA1__NM_001290307_GTAGAAGATGTTCGAAAACA	CTNNA1__NM_001290307	219	194	256	323
CTNNA1__NM_001290307_GTTGGAGCCTCTTGTTACAC	CTNNA1__NM_001290307	740	882	758	779
CTNNA1__NM_001290307_TGAAGCGAGGCAACATGGTT	CTNNA1__NM_001290307	236	199	299	281
CTNNA1__NM_001290307_TGTTGGCCATCGTGATCAGA	CTNNA1__NM_001290307	191	196	282	254
CTNNA1__NM_001290309_ACGACGGTCATCACGCGTGC	CTNNA1__NM_001290309	439	406	377	554
CTNNA1__NM_001290309_CCCTGATGTCGCAGCCTATA	CTNNA1__NM_001290309	631	882	763	876
CTNNA1__NM_001290309_CCTTATAGGCTGCGACATCA	CTNNA1__NM_001290309	757	956	1031	1034
CTNNA1__NM_001290309_CGCAGCCTATAAGGCCAACA	CTNNA1__NM_001290309	311	415	347	351
CTNNA1__NM_001290309_CGTCTGGAAAGCATCATTAG	CTNNA1__NM_001290309	348	326	486	233
CTNNA1__NM_001290309_GCAGGACGAGTCGGCCATCA	CTNNA1__NM_001290309	1153	867	1201	1032
CTNNA1__NM_001290309_GCTTTCCAGACGCTCCTCCA	CTNNA1__NM_001290309	1697	2281	1885	1629
CTNNA1__NM_001290309_GTCTGGAAAGCATCATTAGT	CTNNA1__NM_001290309	567	495	531	586
CTNNA1__NM_001290309_TCTGGAAAGCATCATTAGTG	CTNNA1__NM_001290309	227	128	379	302
CTNNA1__NM_001290309_TGACCGTCGTGAGCGAATTG	CTNNA1__NM_001290309	434	365	603	655
CTNNA1__NM_001290310_CTGCCACAATTCGCTCACGA	CTNNA1__NM_001290310	1513	2227	1714	2028
CTNNA1__NM_001290312_ACATGCCAACAAATTGATTG	CTNNA1__NM_001290312	195	256	178	287
CTNNA1__NM_001290312_ACCTTCTTCATTATTTGAGA	CTNNA1__NM_001290312	233	387	368	399
CTNNA1__NM_001290312_AGCAAAACCACAGAGTAAAC	CTNNA1__NM_001290312	149	200	191	152
CTNNA1__NM_001290312_AGGTGACTACGTGAATGACC	CTNNA1__NM_001290312	169	50	172	125
CTNNA1__NM_001290312_CGCACAGCTGGTGCAATTCG	CTNNA1__NM_001290312	2236	2744	2375	3136
CTNNA1__NM_001290312_CTGGCCAGAGTGCCCGGGTA	CTNNA1__NM_001290312	1012	1295	1042	1516
CTNNA1__NM_001290312_GACCAGGGACTTGCGTAGAC	CTNNA1__NM_001290312	1092	930	1186	1421
CTNNA1__NM_001290312_GAGATGGACAACTATGAGCC	CTNNA1__NM_001290312	312	356	296	496
CTNNA1__NM_001290312_GATGCCATCATATACCAGGC	CTNNA1__NM_001290312	624	607	733	503
CTNNA1__NM_001290312_GCTTCAATCAATACCAAAAG	CTNNA1__NM_001290312	218	178	305	315
CTNNA1__NM_001290312_TCTACTTGCTCAGTAAAACG	CTNNA1__NM_001290312	256	241	173	301
CTNNA1__NM_001290312_TCTTGGGCCAGTTTACTCTG	CTNNA1__NM_001290312	248	339	205	214
CTNNA2__NM_001164883_AAAGGGTGAACAGATCGCTA	CTNNA2__NM_001164883	297	328	302	265
CTNNA2__NM_001164883_CCTTGCTCGTCGGTAAAGCG	CTNNA2__NM_001164883	591	589	650	799
CTNNA2__NM_001164883_CGCGCTTTACCGACGAGCAA	CTNNA2__NM_001164883	706	1018	859	1004
CTNNA2__NM_001164883_CTACAGAGGCAGCTAGTACA	CTNNA2__NM_001164883	206	264	236	236
CTNNA2__NM_001164883_CTGAGTGGCTTGCTCTACAG	CTNNA2__NM_001164883	392	377	516	472
CTNNA2__NM_001164883_CTTGTCAACACAAGCAACAA	CTNNA2__NM_001164883	0	0	8	5
CTNNA2__NM_001164883_GCACCATGGTACGGGCGGCA	CTNNA2__NM_001164883	1638	1825	1577	1845
CTNNA2__NM_001164883_GGAAATCCGGACGCTAACAG	CTNNA2__NM_001164883	679	821	756	801
CTNNA2__NM_001164883_GTAGAGGATGTGCGCAAACA	CTNNA2__NM_001164883	378	318	397	362
CTNNA2__NM_001164883_TACAGAGGCAGCTAGTACAT	CTNNA2__NM_001164883	301	268	309	252
CTNNA2__NM_001164883_TCCGGACGCTAACAGTGGAA	CTNNA2__NM_001164883	336	353	374	479
CTNNA2__NM_001164883_TGCAGATGACCCTTGCTCGT	CTNNA2__NM_001164883	543	427	610	442
CTNNA2__NM_001282599_AAGAAGGGGTGAAATTAGTT	CTNNA2__NM_001282599	255	191	244	336
CTNNA2__NM_001282599_ACGCTTCGCTGAACAAGTAG	CTNNA2__NM_001282599	1	2	0	0
CTNNA2__NM_001282599_AGCTGGGGTTTATACTGAGA	CTNNA2__NM_001282599	314	236	352	361
CTNNA2__NM_001282599_AGGGGTGAAATTAGTTCGGA	CTNNA2__NM_001282599	384	360	374	430
CTNNA2__NM_001282599_CAAAGTTGCTCAGGATAACA	CTNNA2__NM_001282599	94	98	149	188
CTNNA2__NM_001282599_CAACGTTCCTCAACCGTTTG	CTNNA2__NM_001282599	685	857	907	1111
CTNNA2__NM_001282599_CCACGGCCTCTGTCAACACT	CTNNA2__NM_001282599	1022	1191	843	1296
CTNNA2__NM_001282599_GCCTCAATGAGAACTAGCAA	CTNNA2__NM_001282599	302	257	346	452
CTNNA2__NM_001282599_GCGATGTGGACACTCTGGAC	CTNNA2__NM_001282599	1490	1684	1789	2229
CTNNA2__NM_001282599_GGAGATGGAACAGGCCAAAT	CTNNA2__NM_001282599	549	623	643	720
CTNNA2__NM_001282599_TCAGCGAAGCGTGGCATCAC	CTNNA2__NM_001282599	105	63	113	134
CTNNA2__NM_001282599_TCTGAGATGCATACCATGTC	CTNNA2__NM_001282599	703	738	729	714
CTNNA2__NM_001282600_TCATTCTCCTCAAACGGTTG	CTNNA2__NM_001282600	79	312	84	196
CTNNA3__NM_001127384_ACTGGAGCCTCTCATAATCC	CTNNA3__NM_001127384	637	836	803	440
CTNNA3__NM_001127384_AGAACCTCAGGCAGCAACCC	CTNNA3__NM_001127384	370	346	469	603
CTNNA3__NM_001127384_CACAGAAGCTAGAAGGACAC	CTNNA3__NM_001127384	203	222	233	209
CTNNA3__NM_001127384_CAGAAAACCTACCAGAAGCT	CTNNA3__NM_001127384	537	690	588	879
CTNNA3__NM_001127384_CAGAGCAGCCCCACTGATAA	CTNNA3__NM_001127384	1083	1272	1265	1690
CTNNA3__NM_001127384_CAGCTGACACATGTTGCAAG	CTNNA3__NM_001127384	366	551	367	457
CTNNA3__NM_001127384_CGAGGGACTTACACCGAGAG	CTNNA3__NM_001127384	320	421	264	313
CTNNA3__NM_001127384_CTTCCTCCACAGAAGCTAGA	CTNNA3__NM_001127384	123	92	152	277
CTNNA3__NM_001127384_GCAGGTCCAAACATTCACCG	CTNNA3__NM_001127384	676	740	878	1057
CTNNA3__NM_001127384_GCCTTGAAGCCATTATCAGT	CTNNA3__NM_001127384	1150	739	1286	864
CTNNA3__NM_001127384_GCTTCTCCACGGTGAATGTT	CTNNA3__NM_001127384	328	404	390	476
CTNNA3__NM_001127384_TCCTCCTCAGTTACTGTGAG	CTNNA3__NM_001127384	443	487	584	669
CTNNA3__NM_001291133_GGAGAATGAAATCAACGCAA	CTNNA3__NM_001291133	145	172	184	255
CTNNAL1__NM_001286974_ACTGGAGATCAAAACTCGCT	CTNNAL1__NM_001286974	801	950	1030	912
CTNNAL1__NM_001286974_AGACAAAACAGGAGTGATAA	CTNNAL1__NM_001286974	149	284	233	187
CTNNAL1__NM_001286974_AGATTCCAGATGGTTCAAGT	CTNNAL1__NM_001286974	256	201	303	345
CTNNAL1__NM_001286974_AGCTATAGCCAATGAAAACT	CTNNAL1__NM_001286974	373	463	562	577
CTNNAL1__NM_001286974_AGTATTTGACCGTATGAAAG	CTNNAL1__NM_001286974	140	168	160	186
CTNNAL1__NM_001286974_CAAGCTGTCAACTTGGCAGT	CTNNAL1__NM_001286974	910	985	940	1470
CTNNAL1__NM_001286974_CTGCAAGCAATTCAGCGTGT	CTNNAL1__NM_001286974	1208	1304	1380	1403
CTNNAL1__NM_001286974_CTTCGCCCTCGACTCGGGAC	CTNNAL1__NM_001286974	39	59	27	67
CTNNAL1__NM_001286974_GATCTCCAGTCCCGAGTCGA	CTNNAL1__NM_001286974	724	802	923	883
CTNNAL1__NM_001286974_GGAGATCAAAACTCGCTCGG	CTNNAL1__NM_001286974	468	597	446	435
CTNNAL1__NM_001286974_GGCGGCGCCGGAGCAGTCTA	CTNNAL1__NM_001286974	947	961	1027	1084
CTNNAL1__NM_001286974_GTCCAAATATTCAGTCAATT	CTNNAL1__NM_001286974	363	610	629	788
CTNNB1__NM_001098209_AGAACGCATGATAGCGTGTC	CTNNB1__NM_001098209	220	156	263	127
CTNNB1__NM_001098209_ATGCAATGACTCGAGCTCAG	CTNNB1__NM_001098209	241	139	339	224
CTNNB1__NM_001098209_CTGGCAGCAACAGTCTTACC	CTNNB1__NM_001098209	344	115	396	99
CTNNB1__NM_001098209_GAAAAGCGGCTGTTAGTCAC	CTNNB1__NM_001098209	572	228	509	306
CTNNB1__NM_001098209_GAAACAGCTCGTTGTACCGC	CTNNB1__NM_001098209	505	704	623	759
CTNNB1__NM_001098209_GACAAAACTGCTAAATGACG	CTNNB1__NM_001098209	657	753	715	865
CTNNB1__NM_001098209_GCAGCATCAAACTGTGTAGA	CTNNB1__NM_001098209	885	1077	930	1374
CTNNB1__NM_001098209_GTTCCCTGAGACATTAGATG	CTNNB1__NM_001098209	183	52	150	99
CTNNB1__NM_001098209_TGCAATGACTCGAGCTCAGA	CTNNB1__NM_001098209	117	86	117	123
CTNNB1__NM_001098209_TTCACTCAAGAACAAGTAGC	CTNNB1__NM_001098209	35	6	17	16
CTNNB1__NM_001098209_TTCCCACTCATACAGGACTT	CTNNB1__NM_001098209	80	44	88	83
CTNNB1__NM_001098209_TTCCCTGAGACATTAGATGA	CTNNB1__NM_001098209	35	28	36	32
CTNNBIP1__NM_001012329_CCACCCCTGCATAGGTGCGC	CTNNBIP1__NM_001012329	333	489	404	461
CTNNBIP1__NM_001012329_CCTCCGCACTCCATCGACCA	CTNNBIP1__NM_001012329	1315	1276	1717	1627
CTNNBIP1__NM_001012329_CCTGCGCACCTATGCAGGGG	CTNNBIP1__NM_001012329	550	574	581	631
CTNNBIP1__NM_001012329_CTGCTCATGCTGCGGAAGAT	CTNNBIP1__NM_001012329	411	703	642	852
CTNNBIP1__NM_001012329_GAGTTCCTGCGCACCTATGC	CTNNBIP1__NM_001012329	298	383	421	385
CTNNBIP1__NM_001012329_GGTCGGAGACGGAAGACCGG	CTNNBIP1__NM_001012329	450	640	451	429
CTNNBIP1__NM_001012329_TCACCCTGGTCGATGGAGTG	CTNNBIP1__NM_001012329	1063	1008	1158	1308
CTNNBL1__NM_001281495_AAGATGCGTCGGAAACAAAC	CTNNBL1__NM_001281495	154	112	148	103
CTNNBL1__NM_001281495_AGAAGTGCTCATCGATGCTC	CTNNBL1__NM_001281495	87	72	91	62
CTNNBL1__NM_001281495_AGAGCATGTCTGTTCGATCC	CTNNBL1__NM_001281495	758	32	842	114
CTNNBL1__NM_001281495_AGCACTGCTGGTACAGAATC	CTNNBL1__NM_001281495	308	338	330	369
CTNNBL1__NM_001281495_AGCTGATGAATCTCATGCTC	CTNNBL1__NM_001281495	168	320	194	103
CTNNBL1__NM_001281495_ATCATTGTCCTGGAGCAATA	CTNNBL1__NM_001281495	233	13	225	10
CTNNBL1__NM_001281495_CCTGGATGAGTCTGTGAAAG	CTNNBL1__NM_001281495	1011	875	1160	1298
CTNNBL1__NM_001281495_CGGCCTGAGATGTGTACAGA	CTNNBL1__NM_001281495	352	104	391	201
CTNNBL1__NM_001281495_CTTCAGGAAGCGCTCACGAT	CTNNBL1__NM_001281495	793	864	784	806
CTNNBL1__NM_001281495_TCATTTCTTCTTCCCGATAG	CTNNBL1__NM_001281495	105	136	127	132
CTNNBL1__NM_001281495_TTAGAAAACAGGGAATTGCT	CTNNBL1__NM_001281495	25	12	52	4
CTNNBL1__NM_001281495_TTCACTGCAATACAGTTTGT	CTNNBL1__NM_001281495	148	5	152	9
CTNND1__NM_001085458_AAGAATGGCAATGGGGGACC	CTNND1__NM_001085458	185	292	362	465
CTNND1__NM_001085458_ATGAGTGGGTTGGCATCTTG	CTNND1__NM_001085458	666	781	837	930
CTNND1__NM_001085458_CCAGGTGGCAGTGATAACTA	CTNND1__NM_001085458	44	31	53	49
CTNND1__NM_001085458_CCGGCGGGTGAGTGTGCCGT	CTNND1__NM_001085458	3255	3087	3413	3651
CTNND1__NM_001085458_CGGCGCAGCTGGAACGCGTC	CTNND1__NM_001085458	452	443	576	816
CTNND1__NM_001085458_GACAGTACAGCCAGTCGCTA	CTNND1__NM_001085458	1026	1311	1187	1143
CTNND1__NM_001085458_GCCGTTGGCCATGAGTGGGT	CTNND1__NM_001085458	199	191	187	309
CTNND1__NM_001085458_GGGACCAGACGGGTTGCCTG	CTNND1__NM_001085458	380	314	508	456
CTNND1__NM_001085458_GGGGGACCTGGTCCCTATGT	CTNND1__NM_001085458	190	202	209	147
CTNND1__NM_001085458_GTGCCAGCTTGCCCCACATA	CTNND1__NM_001085458	614	536	602	554
CTNND1__NM_001085458_TGGCGACTATAACCATCAGG	CTNND1__NM_001085458	68	36	18	5
CTNND1__NM_001085458_TGGGGATGGTGCTATATAGA	CTNND1__NM_001085458	93	39	84	75
CTNND1__NM_001085463_CAACCCCAGGTTCGGGTAGG	CTNND1__NM_001085463	1157	1169	1239	1718
CTNND1__NM_001085463_CCCGCATTGAGGAGCGGTAT	CTNND1__NM_001085463	2514	2568	2607	3310
CTNND1__NM_001085463_CTACCGGGCACCTAGTAGAC	CTNND1__NM_001085463	517	710	659	681
CTNND1__NM_001085463_GGAGCGGTATAGGCCCAGCA	CTNND1__NM_001085463	416	489	456	711
CTNND1__NM_001085463_GTCCCGGGTGACCCGCATTG	CTNND1__NM_001085463	112	76	110	77
CTNND2__NM_001288715_AGGCACACAAGCCCGAGCTA	CTNND2__NM_001288715	1146	1293	1089	1234
CTNND2__NM_001288715_ATCAGGAATACTTGACCCAC	CTNND2__NM_001288715	230	213	294	241
CTNND2__NM_001288715_CAGGGTGGCGTACGTGCCGA	CTNND2__NM_001288715	574	795	657	738
CTNND2__NM_001288715_CTCCTGTATTAGGTCACTAC	CTNND2__NM_001288715	269	438	328	378
CTNND2__NM_001288715_GACCCACAGGATTATTCTAC	CTNND2__NM_001288715	373	378	485	658
CTNND2__NM_001288715_GGGGCGAGGTCACGCGGATC	CTNND2__NM_001288715	460	819	515	737
CTNND2__NM_001288715_GGGGGCGAGGTCACGCGGAT	CTNND2__NM_001288715	1676	2275	1991	2784
CTNND2__NM_001288715_GGTCTGGTTGCTATGGTAGC	CTNND2__NM_001288715	378	472	559	791
CTNND2__NM_001288715_GGTGGCGTACGTGCCGATGG	CTNND2__NM_001288715	663	803	759	1101
CTNND2__NM_001288715_TACAACAGGTCTCGAGCTGG	CTNND2__NM_001288715	347	262	326	403
CTNND2__NM_001288715_TATGGTAGCTGGCCGGATAC	CTNND2__NM_001288715	329	474	295	435
CTNND2__NM_001288715_TTCCTGTAGTGACCTAATAC	CTNND2__NM_001288715	56	71	35	51
CTNND2__NM_001288716_AGGGGACGGAGTCGACACCA	CTNND2__NM_001288716	269	284	224	208
CTNND2__NM_001288716_CACACCTGTGCTCGTGCGGT	CTNND2__NM_001288716	399	327	404	527
CTNND2__NM_001288716_CGGCCCACAGAATGCCGCCG	CTNND2__NM_001288716	1	0	15	34
CTNND2__NM_001288716_GGCACCTACCGCACGAGCAC	CTNND2__NM_001288716	568	778	576	780
CTNND2__NM_001288716_GGTCCGCGTAATTGGAGGCT	CTNND2__NM_001288716	632	857	717	844
CTNND2__NM_001288716_TGGCCGCGGCGGCATTCTGT	CTNND2__NM_001288716	279	218	336	387
CTNND2__NM_001288716_TGGCTGCCTGTGCGCTGCAA	CTNND2__NM_001288716	59	48	114	66
CTNND2__NM_001288717_GAGTCTCCATACAGCAAATC	CTNND2__NM_001288717	181	187	198	325
CTNND2__NM_001288717_GCAGGGCCGGATTTGCTGTA	CTNND2__NM_001288717	843	1015	1075	1210
CTNND2__NM_001288717_GCTGTATGGAGACTCAACAG	CTNND2__NM_001288717	266	326	278	341
CTNND2__NM_001288717_GTAGGGGTCCGCGTAATTGG	CTNND2__NM_001288717	232	338	231	169
CTNND2__NM_001288717_TCGGTAGGGGTCCGCGTAAT	CTNND2__NM_001288717	1284	1329	1262	1272
CTNND2__NM_001332_CAGCTGGAGCGATGCAAGCT	CTNND2__NM_001332	217	232	221	186
CTNND2__NM_001332_CCCGTTGGAGGTGTTTAAGC	CTNND2__NM_001332	520	384	508	365
CTNND2__NM_001332_CCGGCTTAAACACCTCCAAC	CTNND2__NM_001332	336	381	307	331
CTNND2__NM_001332_CGTTGGAGGTGTTTAAGCCG	CTNND2__NM_001332	955	935	897	967
CTNND2__NM_001332_TTCAGAGCCATCCCCGTTGG	CTNND2__NM_001332	137	122	94	114
CTNND2__NM_001332_TTCTACCTGTTCTTTGACTG	CTNND2__NM_001332	8	0	0	0
CTNS__NM_001031681_AAGAAAGCGTATCCTCGGGC	CTNS__NM_001031681	899	651	944	696
CTNS__NM_001031681_ACATTTCTCTACGAGCTTCA	CTNS__NM_001031681	163	436	285	433
CTNS__NM_001031681_ACCAGGGTTGCATTTAATGG	CTNS__NM_001031681	632	509	584	568
CTNS__NM_001031681_AGCATCATAAACCAGGTGAT	CTNS__NM_001031681	384	367	340	369
CTNS__NM_001031681_ATCACCAGGGTTGCATTTAA	CTNS__NM_001031681	358	419	482	418
CTNS__NM_001031681_CACCTGGTTTATGATGCTAA	CTNS__NM_001031681	517	518	638	787
CTNS__NM_001031681_CATTTCTCTACGAGCTTCAG	CTNS__NM_001031681	298	276	373	301
CTNS__NM_001031681_CCGTTCTCCAGCTTTACGAC	CTNS__NM_001031681	498	541	485	608
CTNS__NM_001031681_GGAAATCACTCCAATCAGAC	CTNS__NM_001031681	355	499	498	764
CTNS__NM_001031681_GTGAGGCTGACGCTTGACTC	CTNS__NM_001031681	81	117	95	32
CTNS__NM_001031681_TCACTCCAATCAGACCGGGT	CTNS__NM_001031681	1078	1153	1087	820
CTNS__NM_001031681_TCCAATTCATGATCACCTGA	CTNS__NM_001031681	699	747	747	858
CTPS1__NM_001905_ACATTCTCTCCTTATGAGCA	CTPS1__NM_001905	554	47	651	159
CTPS1__NM_001905_ACGTAGCTATGCCTATTGCT	CTPS1__NM_001905	1619	1923	1661	1612
CTPS1__NM_001905_AGCTACGTGGCACTATATTC	CTPS1__NM_001905	286	175	321	192
CTPS1__NM_001905_ATAACGGTCTAGGTATCCAA	CTPS1__NM_001905	36	17	41	36
CTPS1__NM_001905_ATGAAGTACATTCTGGTTAC	CTPS1__NM_001905	267	2	297	18
CTPS1__NM_001905_ATTCAAAGGGACGGTCAGTT	CTPS1__NM_001905	469	598	599	545
CTPS1__NM_001905_CAAGGAGAGTGTCACGCAAC	CTPS1__NM_001905	415	275	369	338
CTPS1__NM_001905_CCATGCTCGGTAAGTCCATG	CTPS1__NM_001905	686	705	834	735
CTPS1__NM_001905_CCTGCATCAATGTTAATGTA	CTPS1__NM_001905	690	9	841	94
CTPS1__NM_001905_CCTTGGTGAGGCGGATGTCA	CTPS1__NM_001905	782	66	824	74
CTPS1__NM_001905_CGATGGAACCGATGGTGGG	CTPS1__NM_001905	591	527	760	759
CTPS1__NM_001905_CTAGCATCTGCAATGATGCG	CTPS1__NM_001905	289	491	335	312
CTPS1__NM_001905_GAACGGAAAGGAGATTACTT	CTPS1__NM_001905	237	3	252	19
CTPS1__NM_001905_GAGAAACAAGGTGACCGATG	CTPS1__NM_001905	0	0	0	0
CTPS1__NM_001905_GAGGAGCCAGTACACGTGAT	CTPS1__NM_001905	1075	799	1155	1174
CTPS1__NM_001905_GGATTGCATCTGTGATATGA	CTPS1__NM_001905	209	14	300	97
CTPS1__NM_001905_GGCACAATACTCAAGTCATG	CTPS1__NM_001905	837	499	887	597
CTPS1__NM_001905_GGCACACTTTATAACGGTCT	CTPS1__NM_001905	1123	1062	1236	1633
CTPS1__NM_001905_GTTACTGGTGGTGTTATATC	CTPS1__NM_001905	23	0	41	9
CTPS1__NM_001905_GTTGCGGCAGAACATCGCGA	CTPS1__NM_001905	583	613	598	693
CTPS1__NM_001905_TACAGCTCTGACTTTACACT	CTPS1__NM_001905	329	341	361	480
CTPS1__NM_001905_TAGCAATAGGCATAGCTACG	CTPS1__NM_001905	3	0	1	0
CTPS1__NM_001905_TATAACGGTCTAGGTATCCA	CTPS1__NM_001905	345	166	358	181
CTPS1__NM_001905_TCATATCACAGATGCAATCC	CTPS1__NM_001905	396	15	452	34
CTPS1__NM_001905_TCGCGATGGGAACCGATGGT	CTPS1__NM_001905	819	474	869	706
CTPS1__NM_001905_TCTTAATGTGACCTAGCAAT	CTPS1__NM_001905	696	634	759	608
CTPS1__NM_001905_TGGATTGCATCTGTGATATG	CTPS1__NM_001905	31	0	18	0
CTPS1__NM_001905_TGGGGAAGTAGACCTTGACC	CTPS1__NM_001905	197	3	222	9
CTPS1__NM_001905_TTGACCTGGGTAACTATGAG	CTPS1__NM_001905	141	140	192	249
CTPS1__NM_001905_TTGCGGCAGAACATCGCGAT	CTPS1__NM_001905	260	84	277	297
CTPS2__NM_001144002_AGAGAGGCGTGGTGATTACC	CTPS2__NM_001144002	574	618	836	956
CTPS2__NM_001144002_AGCATGTGATCAATAAAGAG	CTPS2__NM_001144002	116	248	101	106
CTPS2__NM_001144002_AGTACATCCTGGTCACGGGT	CTPS2__NM_001144002	1599	1719	1584	1788
CTPS2__NM_001144002_ATGAAGTACATCCTGGTCAC	CTPS2__NM_001144002	192	151	320	226
CTPS2__NM_001144002_CCCTATATTAACATCGATGC	CTPS2__NM_001144002	446	567	539	651
CTPS2__NM_001144002_GAGAGGCGTGGTGATTACCT	CTPS2__NM_001144002	491	584	626	788
CTPS2__NM_001144002_GCCAGCATCGATGTTAATAT	CTPS2__NM_001144002	357	249	481	447
CTPS2__NM_001144002_GGAACGATTCTAAAATCATG	CTPS2__NM_001144002	538	634	640	894
CTPS2__NM_001144002_GGACAGCATCAGTAATGTGA	CTPS2__NM_001144002	221	246	253	250
CTPS2__NM_001144002_GTCATCTCAGGCATTGGTAA	CTPS2__NM_001144002	383	392	398	447
CTPS2__NM_001144002_GTCTTCGTCTTAAATGATGG	CTPS2__NM_001144002	118	331	207	269
CTPS2__NM_001144002_TCATCTCAGGCATTGGTAAA	CTPS2__NM_001144002	267	285	328	345
CTR9__NM_014633_AAAAGACCAGATGACTTGCT	CTR9__NM_014633	668	117	831	262
CTR9__NM_014633_ACTGCACATATGGATTGCTT	CTR9__NM_014633	649	165	730	239
CTR9__NM_014633_ACTTGACTTCGATCAGTTAC	CTR9__NM_014633	553	161	774	245
CTR9__NM_014633_AGAAGGGCTGGAATATTATT	CTR9__NM_014633	669	458	690	511
CTR9__NM_014633_AGCAGGCTCTTCCCAACAAA	CTR9__NM_014633	31	5	44	13
CTR9__NM_014633_AGCCTGCTTCTGCCTACTTG	CTR9__NM_014633	1683	431	1811	462
CTR9__NM_014633_ATGGAGCCCCGCGACATGAT	CTR9__NM_014633	607	340	674	429
CTR9__NM_014633_GACTTCGATCAGTTACCGGA	CTR9__NM_014633	493	98	641	179
CTR9__NM_014633_GATGACTTGCTTGGATACAT	CTR9__NM_014633	263	20	234	32
CTR9__NM_014633_GCCTGCTTCTGCCTACTTGA	CTR9__NM_014633	1012	135	1431	163
CTR9__NM_014633_TGGAGCCCCGCGACATGATG	CTR9__NM_014633	242	64	227	112
CTR9__NM_014633_TTACCTCGTCAGTGTCCCGG	CTR9__NM_014633	448	130	495	59
CTRB1__NM_001906_CGGCCCCCTGGTCTGCCAAA	CTRB1__NM_001906	136	122	126	182
CTRB1__NM_001906_GTGTCACCAAGCTCATACCT	CTRB1__NM_001906	345	246	439	401
CTRB1__NM_001906_TCTCCTGCTTCTCCCTTGTG	CTRB1__NM_001906	696	651	766	605
CTRB1__NM_001906_TGACACGGGCGTACACGCCA	CTRB1__NM_001906	8	8	11	18
CTRB2__NM_001025200_CTGCACCCAGGGTATGAGCT	CTRB2__NM_001025200	348	330	375	484
CTRB2__NM_001025200_GCGACACGGGCGTACACAGC	CTRB2__NM_001025200	1169	1084	1262	1598
CTRB2__NM_001025200_GGCGACACGGGCGTACACAG	CTRB2__NM_001025200	1124	1398	1324	1375
CTRB2__NM_001025200_GGGTATGAGCTTGGCGACAC	CTRB2__NM_001025200	382	670	476	611
CTRC__NM_007272_ACGTGGAGGCATACGTGTGG	CTRC__NM_007272	1643	1513	1859	1772
CTRC__NM_007272_AGTACCTCAAGAACGACACG	CTRC__NM_007272	371	342	333	228
CTRC__NM_007272_CACCACTCGGGCGGATAGGT	CTRC__NM_007272	1022	1023	1036	1321
CTRC__NM_007272_CACGGCCACACGGTAGGTCC	CTRC__NM_007272	47	10	71	21
CTRC__NM_007272_CAGTGATGCCCAACATGGTT	CTRC__NM_007272	134	92	160	87
CTRC__NM_007272_CCACGGCCACACGGTAGGTC	CTRC__NM_007272	2	0	8	14
CTRC__NM_007272_CGGACCTACCGTGTGGCCGT	CTRC__NM_007272	2016	1909	2316	2332
CTRC__NM_007272_CGTGGAGGCATACGTGTGGC	CTRC__NM_007272	1653	1222	1716	1757
CTRC__NM_007272_CTCCCACCACTCGGGCGGAT	CTRC__NM_007272	960	854	1167	948
CTRC__NM_007272_CTCGGGCGGATAGGTTGGGC	CTRC__NM_007272	796	962	911	961
CTRC__NM_007272_GACACGTGGAGGCATACGTG	CTRC__NM_007272	292	411	349	344
CTRC__NM_007272_GCCCAACCTATCCGCCCGAG	CTRC__NM_007272	1766	1149	1723	1278
CTRL__NM_001907_ACTCGCCCAGGACAACAAAA	CTRL__NM_001907	72	28	137	74
CTRL__NM_001907_AGCCGGTGGTGACACACGTG	CTRL__NM_001907	308	203	295	342
CTRL__NM_001907_AGGCCATTACACACCCTAGC	CTRL__NM_001907	483	304	630	819
CTRL__NM_001907_AGGGCTCTGCGTTTGATGAT	CTRL__NM_001907	339	253	245	294
CTRL__NM_001907_ATGCGTGTTGTGTACTGGGC	CTRL__NM_001907	967	1264	1050	1250
CTRL__NM_001907_CAACGGGGAGAATGCAGTGT	CTRL__NM_001907	1302	772	1416	840
CTRL__NM_001907_CGAGATGCGTGTTGTGTACT	CTRL__NM_001907	1419	1976	1733	1854
CTRL__NM_001907_GTGGGCAGCAGTGACCACCC	CTRL__NM_001907	2	16	8	26
CTRL__NM_001907_TCGCCTCAGTGGCGTGGGTA	CTRL__NM_001907	265	303	320	429
CTRL__NM_001907_TGGTCACTGTGAATCAGTGC	CTRL__NM_001907	22	0	7	5
CTRL__NM_001907_TTCAGCCAGAGGATTGTCAA	CTRL__NM_001907	45	13	35	92
CTRL__NM_001907_TTCAGTCAGAGCCTCGTTTG	CTRL__NM_001907	85	76	92	132
CTSA__NM_000308_ATAAAACTTGTCATCGGAGT	CTSA__NM_000308	349	693	466	439
CTSA__NM_000308_ATACCAGATTCCAAGAATAG	CTSA__NM_000308	38	86	88	64
CTSA__NM_000308_ATGGAATCAGTGCATCATAA	CTSA__NM_000308	277	231	340	399
CTSA__NM_000308_CAGCATAGCTCTCCCCGGTC	CTSA__NM_000308	0	0	0	0
CTSA__NM_000308_CCAGGAACCCAACAGAGCTC	CTSA__NM_000308	0	0	15	0
CTSA__NM_000308_CCGTCTTTCCGCCAGTACTC	CTSA__NM_000308	469	557	595	459
CTSA__NM_000308_CTATACCAGATTCCAAGAAT	CTSA__NM_000308	423	286	523	523
CTSA__NM_000308_CTCCTGGAGAGCAAGGACGC	CTSA__NM_000308	336	343	316	268
CTSA__NM_000308_CTGTGGTGCTTTGGCTCAAT	CTSA__NM_000308	378	568	394	541
CTSA__NM_000308_GAGGCGGAAGAAATCTTGAA	CTSA__NM_000308	99	88	135	76
CTSA__NM_000308_GATTGCCAATGTGTTATACC	CTSA__NM_000308	582	362	499	517
CTSA__NM_000308_GTAGTAGGCAAAGTAGACCA	CTSA__NM_000308	346	136	349	221
CTSA__NM_000308_TAGAAGAGGACTCTCCTAGC	CTSA__NM_000308	636	800	668	763
CTSA__NM_000308_TATACCAGATTCCAAGAATA	CTSA__NM_000308	132	136	176	128
CTSA__NM_000308_TGTTCTTGTACTCCGGAAAG	CTSA__NM_000308	0	0	3	1
CTSA__NM_000308_TTAGAAGAGGACTCTCCTAG	CTSA__NM_000308	211	78	198	177
CTSA__NM_001127695_TGATGCCACATCCGCTTGAG	CTSA__NM_001127695	752	963	900	1416
CTSA__NM_001167594_ACCTCAACAACCCGTACGTG	CTSA__NM_001167594	918	947	954	900
CTSA__NM_001167594_CTCAGGGGATAAAGTGCGCA	CTSA__NM_001167594	176	286	279	366
CTSB__NM_001908_ATCCAGAGTTATGTTTACCG	CTSB__NM_001908	571	452	694	726
CTSB__NM_001908_CAATGCGCACGTCAGCGTGG	CTSB__NM_001908	293	372	353	329
CTSB__NM_001908_CACCAATGCGCACGTCAGCG	CTSB__NM_001908	486	723	564	740
CTSB__NM_001908_CCCTGTGAGCACCACGTCAA	CTSB__NM_001908	837	780	881	1046
CTSB__NM_001908_CGCTGACGTGCGCATTGGTG	CTSB__NM_001908	12	3	54	49
CTSB__NM_001908_GGCCCAGAGCTGCCACATGT	CTSB__NM_001908	1159	1152	1172	1286
CTSB__NM_001908_GTTGACCAGCTCATCCGACA	CTSB__NM_001908	498	780	556	738
CTSB__NM_001908_TACCTACATGGGATTCATAG	CTSB__NM_001908	241	173	300	268
CTSB__NM_001908_TATCTCCCTCCCCCGTGCAT	CTSB__NM_001908	1100	1196	1057	1187
CTSB__NM_001908_TCCCGTGCATCGAAGCTTGC	CTSB__NM_001908	230	398	290	313
CTSB__NM_001908_TGCCTGCAAGCTTCGATGCA	CTSB__NM_001908	112	226	116	143
CTSB__NM_001908_TGTTGGCCAATGCCCGGAGC	CTSB__NM_001908	400	336	397	349
CTSC__NM_001114173_ACAGCCATGAACAGACGTTG	CTSC__NM_001114173	1083	1098	1303	1089
CTSC__NM_001114173_CACAGCCATGAACAGACGTT	CTSC__NM_001114173	698	633	728	800
CTSC__NM_001114173_CCGAGCAGTTGACATCGCGC	CTSC__NM_001114173	1603	1637	1654	1753
CTSC__NM_001114173_CGAGCAGTTGACATCGCGCT	CTSC__NM_001114173	1555	1588	1831	2105
CTSC__NM_001114173_CGATGTCAACTGCTCGGTTA	CTSC__NM_001114173	1567	1756	1700	2185
CTSC__NM_001114173_GATGTCAACTGCTCGGTTAT	CTSC__NM_001114173	811	797	920	1110
CTSC__NM_001114173_TGAAATGGCCAGAATTGCCA	CTSC__NM_001114173	110	238	222	241
CTSC__NM_001114173_TTAGCCCCAACGTCTGTTCA	CTSC__NM_001114173	346	308	404	357
CTSC__NM_001114173_TTGTGTTGAATGACTACAAG	CTSC__NM_001114173	7	0	10	10
CTSC__NM_001814_CATTGTCTCGTTGCAGTAAG	CTSC__NM_001814	224	231	289	394
CTSC__NM_001814_CGGAACTGGGCTTGTTTCAC	CTSC__NM_001814	1002	790	1141	1308
CTSC__NM_001814_GGGTGGGTGCATGATGTGTT	CTSC__NM_001814	271	284	293	447
CTSC__NM_001814_TCTCCTAATCATATCTCCCA	CTSC__NM_001814	515	518	640	565
CTSC__NM_001814_TGATGTGTTGGGCCGGAACT	CTSC__NM_001814	890	1104	982	1025
CTSC__NM_001814_TTACCCTGGGAGATATGATT	CTSC__NM_001814	95	35	82	118
CTSD__NM_001909_ACAGACTCCAAGTATTACAA	CTSD__NM_001909	208	274	318	353
CTSD__NM_001909_ATGGGCCCCTCGGTCACGGC	CTSD__NM_001909	734	550	659	592
CTSD__NM_001909_CACCCGCAAGGCCTACTGGC	CTSD__NM_001909	1034	1175	1229	1547
CTSD__NM_001909_CAGAGGCTGACGACGCTGAC	CTSD__NM_001909	1366	1410	1433	2034
CTSD__NM_001909_CGAGGTACCATTCTTCACGT	CTSD__NM_001909	2686	2307	2991	2397
CTSD__NM_001909_CTCCGACATGGTCCGGCGGA	CTSD__NM_001909	487	433	679	780
CTSD__NM_001909_GCCCAGTACTACGGGGAGAT	CTSD__NM_001909	1939	2570	2403	2956
CTSD__NM_001909_GCCGGACCATGTCCGGAGGTT	CTSD__NM_001909	1255	1248	1477	1592
CTSD__NM_001909_GTCCATCCGCCGGACCATGT	CTSD__NM_001909	788	714	853	897
CTSD__NM_001909_TGCCAATCTCCCCGTAGTAC	CTSD__NM_001909	805	1153	829	950
CTSD__NM_001909_TGGGCGGTGTCAAAGTGGAG	CTSD__NM_001909	61	33	88	65
CTSD__NM_001909_TTCACAGTCGTCTTCGACAC	CTSD__NM_001909	17	22	19	6
CTSE__NM_001910_AATCTGCCAGTAAGCTTGCT	CTSE__NM_001910	620	887	770	855
CTSE__NM_001910_ACATCCAAGTAGTTGATGAG	CTSE__NM_001910	428	488	476	491
CTSE__NM_001910_CAATCTTTCTCCATTCAGTA	CTSE__NM_001910	633	913	803	864
CTSE__NM_001910_CCAGGTGGAAGGACTAACCG	CTSE__NM_001910	271	153	165	181
CTSE__NM_001910_GCCAGGCCATTGTGGACACA	CTSE__NM_001910	862	1141	918	1021
CTSE__NM_001910_GCTCCCGGTTCCATACTGAA	CTSE__NM_001910	590	728	616	477
CTSE__NM_001910_GGGAGCTTGTCCGGGATCAT	CTSE__NM_001910	1361	1325	1669	1619
CTSE__NM_001910_GTGGAAGGACTAACCGTGGT	CTSE__NM_001910	336	495	370	378
CTSE__NM_001910_TCTCCATTCAGTATGGAACC	CTSE__NM_001910	136	70	116	91
CTSE__NM_001910_TGAATGGAGAAAGATTGACC	CTSE__NM_001910	149	79	96	132
CTSE__NM_001910_TTCTCCATTCAGTATGGAAC	CTSE__NM_001910	0	0	0	0
CTSE__NM_001910_TTGAGCAGGACTCGGTGAAC	CTSE__NM_001910	810	655	777	673
CTSE__NM_148964_TGCCAACCTTAACGTCATGC	CTSE__NM_148964	236	132	365	385
CTSF__NM_003793_ACAGGGGATCCTCATTCAAC	CTSF__NM_003793	660	875	810	798
CTSF__NM_003793_ACCATGGGGTCGTTGCAGGG	CTSF__NM_003793	541	526	648	568
CTSF__NM_003793_ACGGGGAGCCGGCACACCAT	CTSF__NM_003793	1460	976	1423	1027
CTSF__NM_003793_ACTTTGTCATTACCTATAAC	CTSF__NM_003793	333	304	364	451
CTSF__NM_003793_CACACCATGGGGTCGTTGCA	CTSF__NM_003793	697	829	616	587
CTSF__NM_003793_CAGGGGATCCTCATTCAACA	CTSF__NM_003793	225	147	389	384
CTSF__NM_003793_GACCGTGGCACAGCTCAGTA	CTSF__NM_003793	312	188	313	251
CTSF__NM_003793_GAGTATTCAGGTAGATAGTG	CTSF__NM_003793	82	89	56	96
CTSF__NM_003793_GCGCTGGAGATGTTCAACCG	CTSF__NM_003793	612	551	722	639
CTSF__NM_003793_GTCCGTCTTTGTCAATAACA	CTSF__NM_003793	213	191	215	307
CTSF__NM_003793_TGACTCATATGTCCGGTTAT	CTSF__NM_003793	836	653	832	673
CTSF__NM_003793_TGTTATTGACAAAGACGGAC	CTSF__NM_003793	115	153	132	147
CTSG__NM_001911_ATCTGAAGATACGCCATGTA	CTSG__NM_001911	506	590	564	586
CTSG__NM_001911_ATGGTCCGCTGATTATATTG	CTSG__NM_001911	205	181	226	234
CTSG__NM_001911_CAGGGGAGATCATCGGAGGC	CTSG__NM_001911	481	667	516	532
CTSG__NM_001911_CCCCCTGCTGTGTAACAATG	CTSG__NM_001911	742	977	688	890
CTSG__NM_001911_CGTAGGAACCGAAGATGCGG	CTSG__NM_001911	711	653	683	368
CTSG__NM_001911_CTCCTATGGAAAGTCGTCAG	CTSG__NM_001911	262	200	262	222
CTSG__NM_001911_GAATGACATCATGTTATTGC	CTSG__NM_001911	560	597	670	843
CTSG__NM_001911_GATCTGAAGATACGCCATGT	CTSG__NM_001911	1281	1050	1398	1053
CTSG__NM_001911_GCCCACGGCATCGTCTCCTA	CTSG__NM_001911	1064	1205	1293	1251
CTSG__NM_001911_GCTGACAGCAGCTCATTGCT	CTSG__NM_001911	187	262	270	270
CTSG__NM_001911_GGCCACATTGTTACACAGCA	CTSG__NM_001911	595	527	648	1160
CTSG__NM_001911_GTGTGGGGGACCGGCGGGAA	CTSG__NM_001911	297	206	358	394
CTSG__NM_001911_TACTCTGTGGACGTTTATTA	CTSG__NM_001911	568	598	611	714
CTSH__NM_004390_ACCAAAAGTAACTACCTTCG	CTSH__NM_004390	1226	1484	1145	1310
CTSH__NM_004390_ATCGCTTCCTCGTCATACTG	CTSH__NM_004390	827	894	1059	790
CTSH__NM_004390_CCATGATCCCCTTGTTGTAC	CTSH__NM_004390	1941	2041	1614	2321
CTSH__NM_004390_CCGTAAGACCTACAGTACGG	CTSH__NM_004390	255	208	294	246
CTSH__NM_004390_CCTCCGTACTGTAGGTCTTA	CTSH__NM_004390	259	330	275	256
CTSH__NM_004390_CTTCAAGTCATGGATGTCTA	CTSH__NM_004390	468	513	603	656
CTSH__NM_004390_GGTAGGGACCAGTACCTCGA	CTSH__NM_004390	671	835	743	605
CTSH__NM_004390_TAAGGAGTTCACGCACAGTT	CTSH__NM_004390	513	741	551	717
CTSH__NM_004390_TACATCCTTGACAAAGCCGA	CTSH__NM_004390	216	175	219	138
CTSH__NM_004390_TCCGTGGACTGGCGGAAAAA	CTSH__NM_004390	214	296	246	234
CTSH__NM_004390_TGTCTCACCTGTGAAAAATC	CTSH__NM_004390	75	75	123	128
CTSH__NM_004390_TGTGTGGTTCCCATTGTTGT	CTSH__NM_004390	337	400	351	399
CTSK__NM_000396_AAATCTCTCGGCGTTTAATT	CTSK__NM_000396	188	272	129	184
CTSK__NM_000396_ACACAATCCACTAGGTTCTG	CTSK__NM_000396	220	104	199	179
CTSK__NM_000396_GGGTGTCCAGTATCTCCTCA	CTSK__NM_000396	930	932	1037	970
CTSK__NM_000396_TACAGAGCAAAGCTCACCAC	CTSK__NM_000396	82	79	79	43
CTSK__NM_000396_TCATAGCCAGTTCATATGTA	CTSK__NM_000396	714	542	827	585
CTSK__NM_000396_TCTCAGACACACAATCCACT	CTSK__NM_000396	317	291	396	383
CTSK__NM_000396_TCTGTACCCTGAGGAGATAC	CTSK__NM_000396	149	416	177	168
CTSK__NM_000396_TCTGTCGACTATCGAAAGAA	CTSK__NM_000396	370	465	413	481
CTSK__NM_000396_TCTTCTTCCATAGCTCCCAG	CTSK__NM_000396	16	25	45	9
CTSK__NM_000396_TGAACTGGCTATGAACCACC	CTSK__NM_000396	336	297	337	332
CTSK__NM_000396_TGGACACCCACTGGGAGCTA	CTSK__NM_000396	99	68	34	66
CTSK__NM_000396_TGTTACTCCTGTCAAAAATC	CTSK__NM_000396	28	0	25	0
CTSL__NM_001257971_AACTGCACAATCAGGAATAC	CTSL__NM_001257971	371	594	379	410
CTSL__NM_001257971_ACTGGAAAGCATAATCCATT	CTSL__NM_001257971	271	318	391	317
CTSL__NM_001257971_AGGCCCCCAGATCTGTGGAT	CTSL__NM_001257971	1206	1179	1367	1734
CTSL__NM_001257971_AGGCTGCAATGGTGGCCTAA	CTSL__NM_001257971	236	194	303	318
CTSL__NM_001257971_ATACTTGGGATTGTACTTAC	CTSL__NM_001257971	236	265	202	197
CTSL__NM_001257971_ATCAAATGTTAGAGTAGCTG	CTSL__NM_001257971	225	250	187	214
CTSL__NM_001257971_CTTTCCCCTTCCTGGGCTTA	CTSL__NM_001257971	726	953	932	1025
CTSL__NM_001257971_GCACAACAGATTATACGGCA	CTSL__NM_001257971	739	743	687	824
CTSL__NM_001257971_TACTGTTGCCTCATATGGAT	CTSL__NM_001257971	385	780	499	699
CTSL__NM_001257971_TCATTAGCAACAGAATACTT	CTSL__NM_001257971	343	255	428	235
CTSL__NM_001257971_TTATGCTTTCCAGTATGTTC	CTSL__NM_001257971	418	571	556	648
CTSL__NM_001257971_TTCCAGTATGTTCAGGATAA	CTSL__NM_001257971	23	11	20	11
CTSL__NM_001257973_CATGGGTGGCTACGTAAAGA	CTSL__NM_001257973	288	238	412	313
CTSL__NM_001257973_GATGAAGGCAGTTGCAACTG	CTSL__NM_001257973	894	610	1046	654
CTSL__NM_001257973_GTCATTAGCAACAGAATACT	CTSL__NM_001257973	283	323	281	269
CTSL__NM_001257973_TGAAGGCAGTTGCAACTGTG	CTSL__NM_001257973	332	298	278	388
CTSL__NM_001257973_TTTCAGCTGGGGTGAAGAAT	CTSL__NM_001257973	248	250	260	293
CTSO__NM_001334_AATCTGCTTATGCAATAAAG	CTSO__NM_001334	135	86	214	207
CTSO__NM_001334_AGGCGGCGGCTTCACGCTCG	CTSO__NM_001334	1017	1184	1164	1101
CTSO__NM_001334_AGTGAGCTGGCAAGATTATC	CTSO__NM_001334	488	417	541	689
CTSO__NM_001334_CGCGGCCAGGTCGGGGTGAA	CTSO__NM_001334	3236	4303	3836	4884
CTSO__NM_001334_CTACTCTCAATGCTTTGAAC	CTSO__NM_001334	584	461	560	657
CTSO__NM_001334_CTTTGCCGTTAAGATTTGAC	CTSO__NM_001334	431	615	567	499
CTSO__NM_001334_GAAAACTCCACCGCCTTCTA	CTSO__NM_001334	492	479	533	591
CTSO__NM_001334_GCCGTTAAGATTTGACTGGA	CTSO__NM_001334	1126	1519	1344	1567
CTSO__NM_001334_GGAATCTGCTTATGCAATAA	CTSO__NM_001334	82	145	87	142
CTSO__NM_001334_TGCCGTTAAGATTTGACTGG	CTSO__NM_001334	852	884	838	1034
CTSO__NM_001334_TGCTGGGCCTTCAGCGTGGT	CTSO__NM_001334	433	487	430	414
CTSO__NM_001334_TGCTTTGAACTGGTTAAACA	CTSO__NM_001334	23	3	10	25
CTSS__NM_001199739_AACCTATGGCAAACAATACA	CTSS__NM_001199739	257	370	367	369
CTSS__NM_001199739_AAGCAGTACGACGTCTCATC	CTSS__NM_001199739	441	476	617	538
CTSS__NM_001199739_CACAACCTGGAGCATTCAAT	CTSS__NM_001199739	728	687	815	643
CTSS__NM_001199739_CACCATGGCTTTGTAGGGAT	CTSS__NM_001199739	1459	1954	1860	2396
CTSS__NM_001199739_GCATCTACACCAACAGACAC	CTSS__NM_001199739	492	470	550	381
CTSS__NM_001199739_GGAATGCACTCATACGATCT	CTSS__NM_001199739	817	788	863	806
CTSS__NM_001199739_GGGAATGCACTCATACGATC	CTSS__NM_001199739	200	439	236	230
CTSS__NM_001199739_GTTTGTGATGCTTCACAACC	CTSS__NM_001199739	135	202	70	185
CTSS__NM_001199739_TCACAACCTGGAGCATTCAA	CTSS__NM_001199739	129	81	95	86
CTSS__NM_001199739_TGAAGAAGGATATATTCGGA	CTSS__NM_001199739	229	338	264	192
CTSS__NM_001199739_TTCAGTGTACTTTGAACATG	CTSS__NM_001199739	91	52	121	157
CTSS__NM_001199739_TTGGCTATGGTGATCTTAAT	CTSS__NM_001199739	235	237	233	252
CTSS__NM_004079_AATCAGGCAATATCCGATTA	CTSS__NM_004079	506	380	557	398
CTSV__NM_001201575_ACACAGAAGATTATATGGCG	CTSV__NM_001201575	333	238	337	296
CTSV__NM_001201575_ATCCTATCCATATGTAGCAG	CTSV__NM_001201575	504	560	559	838
CTSV__NM_001201575_ATCTCCGTTGCTATGGATGC	CTSV__NM_001201575	515	394	482	760
CTSV__NM_001201575_ATGAAAGCAGTCGCAACTGT	CTSV__NM_001201575	486	280	464	449
CTSV__NM_001201575_CATGAATCTTTCGCTCGTCC	CTSV__NM_001201575	1445	1226	1461	1910
CTSV__NM_001201575_CTCCGTTCTCCTTGACATAC	CTSV__NM_001201575	540	767	553	689
CTSV__NM_001201575_GATGAAAGCAGTCGCAACTG	CTSV__NM_001201575	459	351	382	294
CTSV__NM_001201575_GGCTTCACAGTGGTCGCACC	CTSV__NM_001201575	772	925	959	1070
CTSV__NM_001201575_TAGGGCCTTCCAGTATGTCA	CTSV__NM_001201575	226	279	231	233
CTSV__NM_001201575_TCTTGATCTTCCCAAATCTG	CTSV__NM_001201575	433	318	548	569
CTSV__NM_001201575_TGAAAGCAGTCGCAACTGTG	CTSV__NM_001201575	273	307	313	345
CTSV__NM_001201575_TTCGCGTCCTCAAGGCAATC	CTSV__NM_001201575	70	104	84	135
CTSW__NM_001335_CAGGCAACATAGAGACCCTG	CTSW__NM_001335	299	208	458	430
CTSW__NM_001335_CCAGCTCTATGGCTATCGGA	CTSW__NM_001335	337	410	360	389
CTSW__NM_001335_CTCTTTCAGCTCTAGCGGCT	CTSW__NM_001335	8	3	6	31
CTSW__NM_001335_GAAAAGGACTACCCGTTCCA	CTSW__NM_001335	526	655	662	501
CTSW__NM_001335_GACTTGGGCACAGCTGAGTT	CTSW__NM_001335	451	351	430	428
CTSW__NM_001335_GCCAGCTCTATGGCTATCGG	CTSW__NM_001335	874	992	1142	999
CTSW__NM_001335_GCGGCTGGGGACCTAGGTCC	CTSW__NM_001335	208	239	178	197
CTSW__NM_001335_GCTGCCACGGTGGCTTCGTC	CTSW__NM_001335	1125	1545	1198	1453
CTSW__NM_001335_TCAGGTAACTCCGGTTGAAC	CTSW__NM_001335	460	476	514	697
CTSW__NM_001335_TCCAGAGCATGCTCACCGCC	CTSW__NM_001335	205	198	303	307
CTSW__NM_001335_TCTTTCAGCTCTAGCGGCTG	CTSW__NM_001335	89	54	113	140
CTSW__NM_001335_TTGGCCAGCTCTATGGCTAT	CTSW__NM_001335	213	147	336	225
CTSZ__NM_001336_AGCTCTTGGCAGATCCGCT	CTSZ__NM_001336	323	380	375	378
CTSZ__NM_001336_AGTTGTTGCAGGTCTCGTCA	CTSZ__NM_001336	624	803	684	569
CTSZ__NM_001336_ATTCATTGCATGTCCCACAT	CTSZ__NM_001336	142	115	161	131
CTSZ__NM_001336_CAGCAGGAGCCGCAGTATTG	CTSZ__NM_001336	220	200	239	241
CTSZ__NM_001336_CCACGCCAGCACCAGCGCTA	CTSZ__NM_001336	320	326	313	290
CTSZ__NM_001336_CGATGACGTTCTGCACGGAC	CTSZ__NM_001336	900	1174	947	1313
CTSZ__NM_001336_CGGATCAACATCAAGAGGAA	CTSZ__NM_001336	216	318	229	305
CTSZ__NM_001336_GCTGGGGACAGGTACTCATG	CTSZ__NM_001336	1028	383	937	356
CTSZ__NM_001336_GGGTAATGACCTGTCCGTGT	CTSZ__NM_001336	1346	1260	1460	1457
CTSZ__NM_001336_GTCATCGACTGCGGTAACGC	CTSZ__NM_001336	230	293	175	115
CTSZ__NM_001336_TAGTTGTTGCAGGTCTCGTC	CTSZ__NM_001336	889	1052	987	1035
CTSZ__NM_001336_TGGGACTGGCGCAATGTGGA	CTSZ__NM_001336	94	141	130	82
CTTN__NM_001184740_AAAGTTTCGACTGATATTCG	CTTN__NM_001184740	224	187	277	306
CTTN__NM_001184740_AGGGTCGGTCTCCCAGTCAT	CTTN__NM_001184740	858	837	998	854
CTTN__NM_001184740_ATGACGCGGGGGCCGATGAC	CTTN__NM_001184740	1411	1602	1601	1974
CTTN__NM_001184740_CAAACTTGCCTCCGAAGCCA	CTTN__NM_001184740	310	271	275	442
CTTN__NM_001184740_CATGGCTATGGAGGGAAATT	CTTN__NM_001184740	261	396	360	266
CTTN__NM_001184740_CGACCGAGTAGACAAGAGCG	CTTN__NM_001184740	526	453	529	536
CTTN__NM_001184740_CGCGCTCTTGTCTACTCGGT	CTTN__NM_001184740	3636	3715	4162	4490
CTTN__NM_001184740_CTGTAGGCTTTGAATACCAG	CTTN__NM_001184740	451	460	520	488
CTTN__NM_001184740_GCGGTGGGCTTCGACTACCA	CTTN__NM_001184740	757	793	685	568
CTTN__NM_001184740_GGAACAAGACCGAATGGATA	CTTN__NM_001184740	159	179	265	292
CTTN__NM_001184740_TCAAGAGCATCAGACCCTTA	CTTN__NM_001184740	471	272	429	299
CTTN__NM_001184740_TGGCGGCAAGTATGGCGTGC	CTTN__NM_001184740	122	250	155	132
CTTNBP2__NM_033427_AAGAGGAGATAGACAAGATG	CTTNBP2__NM_033427	142	101	148	201
CTTNBP2__NM_033427_AGGAGGTATTTATCCAGGAA	CTTNBP2__NM_033427	293	313	336	248
CTTNBP2__NM_033427_ATGATTGAGCAACTGAAAAG	CTTNBP2__NM_033427	197	249	235	166
CTTNBP2__NM_033427_ATGCTCCTCAGCGTGATGGA	CTTNBP2__NM_033427	396	483	423	445
CTTNBP2__NM_033427_CCAGAAGCTCGAAGACGTAA	CTTNBP2__NM_033427	31	30	49	52
CTTNBP2__NM_033427_CGAAGACGTAATGGCCAAAC	CTTNBP2__NM_033427	654	1004	658	802
CTTNBP2__NM_033427_GAAGACAAAAGATAGGCGTT	CTTNBP2__NM_033427	455	568	522	501
CTTNBP2__NM_033427_GAGAGGGGGTTGGTACAAAC	CTTNBP2__NM_033427	190	288	251	300
CTTNBP2__NM_033427_GTATTTATCCAGGAACGGTA	CTTNBP2__NM_033427	100	57	89	101
CTTNBP2__NM_033427_TATTTATCCAGGAACGGTAT	CTTNBP2__NM_033427	586	488	685	734
CTTNBP2__NM_033427_TGTGCTTCTTCGTTTCTCAG	CTTNBP2__NM_033427	259	513	305	278
CTTNBP2__NM_033427_TTCCACGGAAGACAAAAGAT	CTTNBP2__NM_033427	24	18	41	70
CTTNBP2NL__NM_018704_AAAGGTGATCCTAGACCTTG	CTTNBP2NL__NM_018704	27	18	42	5
CTTNBP2NL__NM_018704_ACTTTCATTGAAGAACGCTA	CTTNBP2NL__NM_018704	53	152	90	61
CTTNBP2NL__NM_018704_AGAAAGGCAGCGGCATGCAC	CTTNBP2NL__NM_018704	218	107	220	182
CTTNBP2NL__NM_018704_CAATGAAAGTATCTCTGTGT	CTTNBP2NL__NM_018704	1683	2335	1964	2098
CTTNBP2NL__NM_018704_CATGCACAGGATACGGCTGA	CTTNBP2NL__NM_018704	1496	1394	1601	1418
CTTNBP2NL__NM_018704_CTAAATAGTGTCAGGAGTTC	CTTNBP2NL__NM_018704	188	382	225	306
CTTNBP2NL__NM_018704_GAGAGCAGGCACCGAAAGGT	CTTNBP2NL__NM_018704	57	55	65	26
CTTNBP2NL__NM_018704_GAGAGTGGATTTGTGCAGAC	CTTNBP2NL__NM_018704	286	298	307	435
CTTNBP2NL__NM_018704_GCAGCGGCATGCACAGGATA	CTTNBP2NL__NM_018704	200	216	232	175
CTTNBP2NL__NM_018704_TAACATCAGTGATCCTTTAA	CTTNBP2NL__NM_018704	272	174	370	277
CTTNBP2NL__NM_018704_TGCTGAGAGCAGGCACCGAA	CTTNBP2NL__NM_018704	79	66	89	49
CTTNBP2NL__NM_018704_TGTCACCTACATGCTAGAGA	CTTNBP2NL__NM_018704	140	145	206	132
CTU1__NM_145232_ATGAGGGCATCGGTGGCTAC	CTU1__NM_145232	340	23	432	48
CTU1__NM_145232_ATGTCGTCGGCGTTGTGACC	CTU1__NM_145232	350	5	428	5
CTU1__NM_145232_CACGGTCTCCGCCATGTCGT	CTU1__NM_145232	1434	16	1727	173
CTU1__NM_145232_CGGAGAAGTAGTCGAGGCGG	CTU1__NM_145232	632	26	735	33
CTU1__NM_145232_CTCTCGGGCCAAGCGCTGTG	CTU1__NM_145232	29	0	30	3
CTU1__NM_145232_GCAGCTCGTGGCCGTCGATG	CTU1__NM_145232	462	209	397	437
CTU1__NM_145232_GCCACCGATGCCCTCATCGA	CTU1__NM_145232	1059	7	1023	35
CTU1__NM_145232_GGAGCCACGCACATCGTGAC	CTU1__NM_145232	507	320	505	458
CTU1__NM_145232_GGAGCTGCCGCTCACGGTCG	CTU1__NM_145232	24	6	41	47
CTU1__NM_145232_GTGCTGCACACGGTGCTCGC	CTU1__NM_145232	209	30	235	103
CTU1__NM_145232_TCGTAGGCCACGACCGTGAG	CTU1__NM_145232	49	0	39	16
CTU1__NM_145232_TGAGGGCATCGGTGGCTACC	CTU1__NM_145232	159	32	231	45
[illegible]					
CUL3__NM_001257197_CGAGATCAAGTTGTACGTTA	CUL3__NM_001257197	435	462	476	561
CUL3__NM_001257197_GACCTAAAATCATTAACATC	CUL3__NM_001257197	391	115	583	251
CUL3__NM_001257197_GAGATCAAGTTGTACGTTAT	CUL3__NM_001257197	770	124	972	213
[illegible]					
CUL9__NM_015089_GGTGAGCGGGCACTATCTAA	CUL9__NM_015089	813	574	823	727
CUL9__NM_015089_GGTGGGGGAACGGCATGCTG	CUL9__NM_015089	176	149	200	128
CUL9__NM_015089_TCAGGACACTCCATCGGATC	CUL9__NM_015089	1114	1072	1226	1309
[illegible]					
CWC25__NM_017748_TAGGTGATGGGCTCCTAGTT	CWC25__NM_017748	442	252	366	282
CWC27__NM_005869_AATGCTGGTTCTCATGATAA	CWC27__NM_005869	50	18	57	42
CWC27__NM_005869_ACATACCTTCCAAACAAAGT	CWC27__NM_005869	79	140	113	96
[illegible]					

CYB561D1__NM_001134400_CGCAGCCAGCGGGTCAGTCT	CYB561D1__NM_001134400	712	679	713	841
CYB561D1__NM_001134400_CTACCATCTGACATGTGGAC	CYB561D1__NM_001134400	779	1019	884	1034
CYB561D1__NM_001134400_CTACCTGATGGCTACAGTAA	CYB561D1__NM_001134400	572	457	527	540
CYB561D1__NM_001134400_CTGTGCCAGGACACCAGATG	CYB561D1__NM_001134400	308	306	251	229
CYB561D1__NM_001134400_GCAAGTAGGATCTGGAAATC	CYB561D1__NM_001134400	266	261	276	228
CYB561D1__NM_001134400_TCTTCTGCTCCCGAAAAGCA	CYB561D1__NM_001134400	146	95	142	126
CYB561D1__NM_001134400_TTCTGGGCATGTACTCAGTA	CYB561D1__NM_001134400	2	26	17	6
CYB561D1__NM_001134400_TTTCCAGATCCTACTTGCCG	CYB561D1__NM_001134400	326	421	275	252
CYB561D1__NM_001134403_GCACCCTGTATTCATGGCCT	CYB561D1__NM_001134403	836	1106	905	1384
CYB561D1__NM_001134404_AAGCCAGACATGAGGCTTAC	CYB561D1__NM_001134404	251	210	385	405
CYB561D2__NM_001291284_AGAAAACACCAGTAGTGCCT	CYB561D2__NM_001291284	310	332	438	278
CYB561D2__NM_001291284_AGCTCGGTAGATGTGTGACT	CYB561D2__NM_001291284	572	368	597	484
CYB561D2__NM_001291284_ATACCATGCTACTTCTGGGC	CYB561D2__NM_001291284	476	494	642	847
CYB561D2__NM_001291284_CAGCCAAAGACATAAGCACC	CYB561D2__NM_001291284	633	370	687	381
CYB561D2__NM_001291284_CCAGGCTGCACCAGTGACAG	CYB561D2__NM_001291284	720	751	990	1048
CYB561D2__NM_001291284_CCCACCTGGTTACGCGGCAT	CYB561D2__NM_001291284	1578	1408	1812	1387
CYB561D2__NM_001291284_CCCATGCCGCGTAACCAGGT	CYB561D2__NM_001291284	934	622	947	618
CYB561D2__NM_001291284_CCTGCCCATGCCGCGTAACC	CYB561D2__NM_001291284	1685	1185	1795	1041
CYB561D2__NM_001291284_GCCCACCTGGTTACGCGGCA	CYB561D2__NM_001291284	1782	2019	1865	2388
CYB561D2__NM_001291284_GCCCATGCCGCGTAACCAGG	CYB561D2__NM_001291284	1041	1250	1124	1557
CYB561D2__NM_001291284_GGACAGGGCATAATACAGCC	CYB561D2__NM_001291284	308	241	378	309
CYB561D2__NM_001291284_TGCTCGGCCTTTCCGTGAGA	CYB561D2__NM_001291284	238	264	249	237
CYB5A__NM_001190807_CATGTATAGGCGATACATCA	CYB5A__NM_001190807	748	758	745	1035
CYB5A__NM_001190807_CTCTAGGGTGTAGTACTTCA	CYB5A__NM_001190807	241	311	281	276
CYB5A__NM_001190807_CTTGATGTATCGCCTATACA	CYB5A__NM_001190807	455	468	546	495
CYB5A__NM_001190807_GATGGCAGAGCAGTCGGACG	CYB5A__NM_001190807	639	1001	759	958
CYB5A__NM_001190807_GATGGCAGGGATCACCCAGT	CYB5A__NM_001190807	962	180	955	344
CYB5A__NM_001190807_TTCAGTCCTCTGCCATGTAT	CYB5A__NM_001190807	76	25	96	60
CYB5A__NM_001190807_TTCTAGTTGGTGGACCAACT	CYB5A__NM_001190807	2	0	3	26
CYB5B__NM_030579_ACCCGCTTCCTCAACGAGGT	CYB5B__NM_030579	1302	1215	1522	1073
CYB5B__NM_030579_AGGCAGTATGTCCGGTTCAA	CYB5B__NM_030579	540	398	396	368
CYB5B__NM_030579_AGTCACCTATTACCGGTTGG	CYB5B__NM_030579	636	646	727	576
CYB5B__NM_030579_CACCTCCTCCAACCGGTAAT	CYB5B__NM_030579	911	938	1006	1064
CYB5B__NM_030579_TAAACCTGAAAGTGGTAGCA	CYB5B__NM_030579	170	215	199	190
CYB5B__NM_030579_TAAGAGAACAGCGCCTATGA	CYB5B__NM_030579	431	355	492	270
CYB5B__NM_030579_TGCTACCACTTTCAGGTTTA	CYB5B__NM_030579	314	495	355	587
CYB5D1__NM_144607_ATTCCTGTGCCAATGACGTT	CYB5D1__NM_144607	592	861	700	795
CYB5D1__NM_144607_CCTGCTGCTGAAACCCATCG	CYB5D1__NM_144607	484	417	496	541
CYB5D1__NM_144607_CGACCTAACGTCATTGGCAC	CYB5D1__NM_144607	806	942	796	1168
CYB5D1__NM_144607_CGTGTACGACCTAACGTCAT	CYB5D1__NM_144607	1514	1515	1561	1403
CYB5D1__NM_144607_CTCAGCTGCCCTGTTCGGAC	CYB5D1__NM_144607	690	767	640	814
CYB5D1__NM_144607_GACCTCTGGGTATCTTACCT	CYB5D1__NM_144607	960	924	1041	1040
CYB5D1__NM_144607_GCGTCGCTATTTCACGCCGG	CYB5D1__NM_144607	1337	1626	1549	1951
CYB5D1__NM_144607_GGCACAGGAATACAAGGGTA	CYB5D1__NM_144607	268	368	326	351
CYB5D1__NM_144607_GGTCTTCGGGCCTGTTATGT	CYB5D1__NM_144607	694	867	704	609
[illegible]	[illegible]	[illegible]	[illegible]	[illegible]	[illegible]

DAPK3__NM_001348_GTGCGATGCGCTTAGAGTGC	DAPK3__NM_001348	649	770	720	718
DAPK3__NM_001348_GTGCGGAAGTGCCGGCAGAA	DAPK3__NM_001348	628	622	638	799
DAPK3__NM_001348_GTGGAGGACCATTATGAGAT	DAPK3__NM_001348	279	268	344	314
DAPK3__NM_001348_TGATGACACCGATGCTCCTG	DAPK3__NM_001348	755	536	854	755
DAPK3__NM_001348_TGGCCAAGGACTTCATTCGC	DAPK3__NM_001348	221	181	234	156
DAPK3__NM_001348_TGTTCTCGAAGATGTCGTGC	DAPK3__NM_001348	34	48	67	55
DAPL1__NM_001017920_AAAACAAGAAATTGGCACCT	DAPL1__NM_001017920	1033	1284	1085	1271
DAPL1__NM_001017920_AGCCTCGAAAATGTTAAGCC	DAPL1__NM_001017920	128	200	152	224
DAPL1__NM_001017920_CCTTCTCCAGTGCGTCATTC	DAPL1__NM_001017920	664	587	750	1016
DAPL1__NM_001017920_GAGGATGTCCCCCTTTCCGA	DAPL1__NM_001017920	609	760	877	703
DAPL1__NM_001017920_GGATGCCCTGAATGACGCAC	DAPL1__NM_001017920	586	652	702	856
DAPP1__NM_014395_AACCCGGACAGATTCATAAA	DAPP1__NM_014395	301	274	234	349
DAPP1__NM_014395_CAAGATGGTTTACTCTGCAC	DAPP1__NM_014395	194	341	179	201
DAPP1__NM_014395_CACTTTCATGTTGAATATAC	DAPP1__NM_014395	421	685	512	600
DAPP1__NM_014395_CCGGACAGATTCATAAATGG	DAPP1__NM_014395	790	515	991	955
DAPP1__NM_014395_CCTCCATTTATGAATCTGTC	DAPP1__NM_014395	1105	1103	1393	1619
DAPP1__NM_014395_CTGAACATTCTGTTAGGTCT	DAPP1__NM_014395	339	410	324	381
DAPP1__NM_014395_GGCAGAGCAGAACTTCTAGA	DAPP1__NM_014395	384	340	401	484
DAPP1__NM_014395_GGGACAGCAATGAGACCACC	DAPP1__NM_014395	459	458	573	655
DAPP1__NM_014395_GGTTACCTCACCAAACAGGG	DAPP1__NM_014395	79	52	78	100
DAPP1__NM_014395_GTTAGGTCTAGGATCCGAAT	DAPP1__NM_014395	208	136	243	258
DAPP1__NM_014395_TCTAGGATCCGAATTGGTTC	DAPP1__NM_014395	315	453	418	419
DAPP1__NM_014395_TGCCGTCACATCCATTTGAG	DAPP1__NM_014395	658	777	673	692
DARS__NM_001349_AAACTCAGTTAGATGTCTAT	DARS__NM_001349	582	54	565	27
DARS__NM_001349_ACAGATTTATGTGATCAGTT	DARS__NM_001349	257	0	262	50
DARS__NM_001349_AGAGATGGCAGATGCCAGAC	DARS__NM_001349	83	0	57	7
DARS__NM_001349_CAAACTCAGTTAGATGTCTA	DARS__NM_001349	339	549	359	538
DARS__NM_001349_CAACAGAGTCATTGATCTTA	DARS__NM_001349	154	0	170	0
DARS__NM_001349_CAATTTCTTCCATAACTTCG	DARS__NM_001349	314	571	385	461
DARS__NM_001349_CATTTGCTTATATAGCTGTG	DARS__NM_001349	399	227	379	300
DARS__NM_001349_GATTATGCTAAAGAGAGATA	DARS__NM_001349	238	136	263	187
DARS__NM_001349_GCGGGAGATCATGGACGCGG	DARS__NM_001349	539	204	654	337
DARS__NM_001349_GTTCATACAAGCAGAGCTAA	DARS__NM_001349	0	0	0	0
DARS__NM_001349_TGAAGCATTGGCTATGCTTA	DARS__NM_001349	141	1	245	48
DARS__NM_001349_TTACCATTACCACGAAGTTA	DARS__NM_001349	28	0	62	4
DARS2__NM_018122_AAGGACCACCCAACCGATCT	DARS2__NM_018122	498	177	562	187
DARS2__NM_018122_AGAACCCCAGATCGGTTGGG	DARS2__NM_018122	312	106	293	115
DARS2__NM_018122_AGGACCACCCAACCGATCTG	DARS2__NM_018122	471	139	603	163
DARS2__NM_018122_CTTGGTTAAGTCAGCTGTAC	DARS2__NM_018122	338	282	495	440
DARS2__NM_018122_GAAGGACCACCCAACCGATC	DARS2__NM_018122	286	22	340	142
DARS2__NM_018122_GAGAGAACCCCAGATCGGTT	DARS2__NM_018122	874	342	903	369
DARS2__NM_018122_GAGTTGCGTTCGTCTCACTT	DARS2__NM_018122	2348	109	2299	179
DARS2__NM_018122_GGGTCCTCTTAAACAATGTG	DARS2__NM_018122	15	99	19	15
DARS2__NM_018122_GGTGGTCCTTCTGATGGGTC	DARS2__NM_018122	0	0	21	20
DARS2__NM_018122_GTGGATGGATTCAGTACCGA	DARS2__NM_018122	171	4	138	11
DARS2__NM_018122_TGAAATTAAGAACTTCGTGA	DARS2__NM_018122	160	22	156	18
DARS2__NM_018122_TGGTCCTAAGAGATTTCGAT	DARS2__NM_018122	0	0	0	0
DAW1__NM_178821_AAAATGTTACCATACCTTCA	DAW1__NM_178821	417	580	464	618
DAW1__NM_178821_AAATGTTACCATACCTTCAG	DAW1__NM_178821	141	201	153	179
DAW1__NM_178821_AATGTTGCACTTAACAAATC	DAW1__NM_178821	144	110	176	153
DAW1__NM_178821_AGAACGTGTGATTGCTGTTC	DAW1__NM_178821	553	586	592	727
DAW1__NM_178821_ATAGCATTCAACAATCCTTA	DAW1__NM_178821	31	46	77	47
DAW1__NM_178821_ATTTAACCCTCAAAGCACAT	DAW1__NM_178821	197	344	178	194
DAW1__NM_178821_CACTTAACAAATCGGGCTCA	DAW1__NM_178821	458	619	526	744
DAW1__NM_178821_CTTAGTGACAAAATCGCCAC	DAW1__NM_178821	465	825	416	564
DAW1__NM_178821_GAGGAGCTGAACACGCTGGA	DAW1__NM_178821	1226	1298	1248	1431
DAW1__NM_178821_GCGTACCTGGCGGGTAATAC	DAW1__NM_178821	558	689	657	808
DAW1__NM_178821_GTAAGGATTGTTGAATGCTA	DAW1__NM_178821	144	95	173	181
DAW1__NM_178821_TAGCATTCAACAATCCTTAC	DAW1__NM_178821	429	265	422	411
DAZAP1__NM_018959_AACACGGCCGAAGGAAGGAT	DAZAP1__NM_018959	530	339	644	696
DAZAP1__NM_018959_ACCCCAAGCCATGCACACCC	DAZAP1__NM_018959	1173	1078	1415	1213
DAZAP1__NM_018959_ACTCGGGCGCCGACGAGATC	DAZAP1__NM_018959	489	273	665	505
DAZAP1__NM_018959_ACTGGAGCACGACCCAAGGT	DAZAP1__NM_018959	515	190	647	485
DAZAP1__NM_018959_AGCAGACCGCACACGCTAGA	DAZAP1__NM_018959	363	339	416	429
DAZAP1__NM_018959_CCAAGCCATGCACACCCCGG	DAZAP1__NM_018959	1217	719	1457	968
DAZAP1__NM_018959_CTTGACTGGAGCACGACCCA	DAZAP1__NM_018959	374	198	373	334
DAZAP1__NM_018959_GCCGGAGAGAACACGGCCGA	DAZAP1__NM_018959	252	102	246	280
DAZAP1__NM_018959_GGCGCCCGAGTTGTTCATGG	DAZAP1__NM_018959	1121	693	1616	1433
DAZAP1__NM_018959_GTCGGCGCCCGAGTTGTTCA	DAZAP1__NM_018959	890	798	1101	1246
DAZAP1__NM_018959_TCTTCGTGGGCGGTCTTGAC	DAZAP1__NM_018959	314	270	306	412
DAZAP1__NM_018959_TTTCGGCCATCTAGCGTGTG	DAZAP1__NM_018959	93	158	132	220
DAZAP2__NM_001136264_ACCGACTGGATAATAAGCCA	DAZAP2__NM_001136264	338	400	314	218
DAZAP2__NM_001136264_CAGATGGTGGCTACACCATC	DAZAP2__NM_001136264	26	13	10	4
DAZAP2__NM_001136264_CCAACGTCCTCGTAACTCAG	DAZAP2__NM_001136264	551	761	592	536
DAZAP2__NM_001136264_CCCATGGCTTATTATCCAGT	DAZAP2__NM_001136264	1399	1579	1581	1277
DAZAP2__NM_001136264_CGTCCTCGTAACTCAGCGGA	DAZAP2__NM_001136264	231	196	235	245
DAZAP2__NM_001136264_GCAGGTGGAGCATCGGTATA	DAZAP2__NM_001136264	549	1027	607	977
DAZAP2__NM_001136264_GGCAGGTGGAGCATCGGTAT	DAZAP2__NM_001136264	429	650	530	435
DAZAP2__NM_001136264_GTGGAAGGAGGGTATGATGC	DAZAP2__NM_001136264	80	106	75	96
DAZAP2__NM_001136264_GTGGAGCCAGGTGGATAGAT	DAZAP2__NM_001136264	147	92	179	152
DAZAP2__NM_001136264_TCCCCTTCCGCTGAGTTACG	DAZAP2__NM_001136264	387	324	408	230
DAZAP2__NM_001136266_AGCCTCTCTGTATCTTCCCA	DAZAP2__NM_001136266	2095	1767	2683	1782
DAZAP2__NM_001136266_AGTTTAGACACATGTTGTTG	DAZAP2__NM_001136266	390	524	516	425
DAZAP2__NM_001136266_ATGGGGATTGTGGAACCTAA	DAZAP2__NM_001136266	143	340	121	99
DAZAP2__NM_001136266_GCAGTTTAGACACATGTTGT	DAZAP2__NM_001136266	435	360	532	450
DAZAP2__NM_014764_AAAAGTGGAGGGTGCCTTAT	DAZAP2__NM_014764	398	383	516	461
DAZAP2__NM_014764_AAACATCAAATTAGGTTTGG	DAZAP2__NM_014764	188	364	260	232
DAZAP2__NM_014764_AAGAAGGAAATTAGTCTGAC	DAZAP2__NM_014764	338	326	326	269
DAZAP2__NM_014764_AATTCCTAGACTGGGTTAAT	DAZAP2__NM_014764	98	115	159	177
DAZAP2__NM_014764_ACCTATGAGGAGGGCCCATA	DAZAP2__NM_014764	452	524	591	613
DAZAP2__NM_014764_ACTGTGGTGCACTTAACTTG	DAZAP2__NM_014764	518	591	562	427
DAZAP2__NM_014764_ATTCCTAGACTGGGTTAATA	DAZAP2__NM_014764	444	213	412	426
DAZAP2__NM_014764_ATTCTGGGGAGCCGAATGAG	DAZAP2__NM_014764	123	210	187	138
DAZAP2__NM_014764_CACCTAGCTGAATTCTAGAT	DAZAP2__NM_014764	843	827	1079	950
DAZAP2__NM_014764_GACTGTACCATTGACTGTTA	DAZAP2__NM_014764	1133	1098	1281	1148
DAZAP2__NM_014764_GCACCTAGCTGAATTCTAGA	DAZAP2__NM_014764	625	981	803	819
DAZAP2__NM_014764_GTATGCAGAGACAACCTATG	DAZAP2__NM_014764	338	211	310	319
DAZAP2__NM_014764_GTCTGATGCGGCCACTCATT	DAZAP2__NM_014764	1090	807	1243	738
DAZAP2__NM_014764_GTGTTCAGAGCTAAATTAAG	DAZAP2__NM_014764	71	74	32	48
DAZAP2__NM_014764_TACCTCAAAATGCTTTAGGG	DAZAP2__NM_014764	541	358	541	410
DAZAP2__NM_014764_TTAAAGGAAAATCCTTAGGG	DAZAP2__NM_014764	148	112	160	44
DAZL__NM_001190811_AAGATAATCACTGATCGAAC	DAZL__NM_001190811	218	191	227	206
DAZL__NM_001190811_ACTGGTGAATTTGGGTAAGT	DAZL__NM_001190811	1017	1009	1251	1097
DAZL__NM_001190811_AGAAGCTTCTTTGCTAGATA	DAZL__NM_001190811	151	196	167	245
DAZL__NM_001190811_ATAGGATTCATCGTGGTTGT	DAZL__NM_001190811	205	226	207	270
DAZL__NM_001190811_ATGGCGGCTCCCTCGTGTGG	DAZL__NM_001190811	718	562	778	699
DAZL__NM_001190811_CAGTCACAGATAAATTTCCA	DAZL__NM_001190811	1145	823	1194	868
DAZL__NM_001190811_CCAACCTCGTGCGGCAAAGA	DAZL__NM_001190811	686	983	796	911
DAZL__NM_001190811_CTGAGTTATAGGATTCATCG	DAZL__NM_001190811	239	232	239	292
DAZL__NM_001190811_TATAGGATTCATCGTGGTTG	DAZL__NM_001190811	103	60	79	61
DAZL__NM_001190811_TCATGAACTGAACATTCATT	DAZL__NM_001190811	828	768	1010	733
DAZL__NM_001190811_TGACCTGAACTGGTGAATTT	DAZL__NM_001190811	978	752	1106	1227
DAZL__NM_001190811_TTTCTGTCGCCGCCACACGA	DAZL__NM_001190811	310	303	367	408
DAZL__NM_001351_GGACCGAAGCATACAAACGG	DAZL__NM_001351	460	477	375	300
DBF4__NM_006716_AACTCAGGTTAAACTAAGGT	DBF4__NM_006716	136	114	225	179
DBF4__NM_006716_AAGTACTTCAGTAAGAGATG	DBF4__NM_006716	88	15	70	28
DBF4__NM_006716_AATCTCTGAAAACTGATAAC	DBF4__NM_006716	185	70	237	78
DBF4__NM_006716_AGCAAACTCAGGTTAAACTA	DBF4__NM_006716	301	62	215	89
DBF4__NM_006716_AGCTAAATTTGCACAAACCT	DBF4__NM_006716	393	145	382	220
DBF4__NM_006716_CAAAAGGACATTAAGGATCT	DBF4__NM_006716	253	57	307	128
DBF4__NM_006716_CAAGTACTTCAGTAAGAGAT	DBF4__NM_006716	250	18	261	137
DBF4__NM_006716_CATGAAGGAAGGACTTAACC	DBF4__NM_006716	627	659	791	784
DBF4__NM_006716_CCTGGAAATGTCCTTTACTG	DBF4__NM_006716	454	229	571	152
DBF4__NM_006716_GACTTAACCAGGCTACCAAG	DBF4__NM_006716	440	426	542	561
DBF4__NM_006716_TACAGGAGAAATTCGACCCA	DBF4__NM_006716	154	79	159	143
DBF4__NM_006716_TACCACCAGTGACATTCAAT	DBF4__NM_006716	1560	1701	1494	1680
DBF4__NM_006716_TATTATCAAATGCCTTGTCA	DBF4__NM_006716	309	218	469	341
DBF4__NM_006716_TCCCCAAAGTGGCTTACATT	DBF4__NM_006716	618	360	573	471
DBF4__NM_006716_TGGTGACAGAAGGTAAGTCA	DBF4__NM_006716	21	4	10	3
DBF4__NM_006716_TTACAGAATAAACTTTGTGAC	DBF4__NM_006716	156	238	244	314
DBF4__NM_006716_TTCATCTCCTACCAAATTAG	DBF4__NM_006716	5	45	13	7
DBF4__NM_006716_TTTGGGTCTGTAAACAAAGC	DBF4__NM_006716	57	62	39	39
DBF4B__NM_025104_AGCAGGCAGATCCAAGTAAA	DBF4B__NM_025104	144	91	184	200
DBF4B__NM_025104_CAGAGTGGAAACATCGGCCA	DBF4B__NM_025104	627	493	544	447
DBF4B__NM_025104_CCTCACTGGGGCTAGGGCTA	DBF4B__NM_025104	424	593	469	629
DBF4B__NM_025104_GAATGGCCCCCGTCAAAAAC	DBF4B__NM_025104	256	440	287	416
DBF4B__NM_025104_GAGTCAACGGGTTTCCGTGA	DBF4B__NM_025104	487	278	457	288
DBF4B__NM_025104_GGCCATTCAGCAACTGGGTG	DBF4B__NM_025104	97	88	111	208
DBF4B__NM_025104_GGGCCATTCAGCAACTGGGT	DBF4B__NM_025104	827	767	927	1169
DBF4B__NM_025104_GTAAAAGGACTTTCCGGAAA	DBF4B__NM_025104	355	199	403	331
DBF4B__NM_025104_TGAGGTCAGAGTGGAAACAT	DBF4B__NM_025104	190	132	188	179
DBF4B__NM_025104_TGTCAGGTGCCTCTAAGCAG	DBF4B__NM_025104	81	68	82	67
DBF4B__NM_025104_TTCACGGAAACCCGTTGACT	DBF4B__NM_025104	71	120	79	68
DBF4B__NM_025104_TTTGATGAGCGAACCGGGAA	DBF4B__NM_025104	22	33	59	32
DBH__NM_000787_AAGGCTGCCTCCCGCATGCT	DBH__NM_000787	721	560	796	832
DBH__NM_000787_AGGGGGATGTGATAGGGGAG	DBH__NM_000787	105	21	121	165
DBH__NM_000787_CGGGTCCAGGGGGATGTGAT	DBH__NM_000787	372	429	503	536
DBH__NM_000787_CGTGGTGCTCTGGACCGATG	DBH__NM_000787	499	263	517	352
DBH__NM_000787_CTCAAGGCTGGCGTCCTGTT	DBH__NM_000787	583	900	579	491
DBH__NM_000787_CTCGTGGTGCTCTGGACCGA	DBH__NM_000787	260	275	233	312
DBH__NM_000787_CTGCGTTCTCAAGCTCGCCA	DBH__NM_000787	147	184	228	181
DBH__NM_000787_CTGTTTGGGATGTCCGACCG	DBH__NM_000787	584	535	587	594
DBH__NM_000787_GCTTGAGAACGCAGATCTCG	DBH__NM_000787	531	506	696	815
DBH__NM_000787_GGGTCCAGGGGGATGTGATA	DBH__NM_000787	606	498	555	567
DBH__NM_000787_TCAAGGCTGGCGTCCTGTTT	DBH__NM_000787	1045	1019	948	1045
DBH__NM_000787_TCGTGGTGCTCTGGACCGAT	DBH__NM_000787	633	660	764	994
DBI__NM_001079862_CATCTCCTCATCCGATGGCT	DBI__NM_001079862	837	629	902	874
DBI__NM_001079862_CTCATTCCAGGCATCCCACT	DBI__NM_001079862	913	470	899	686
DBI__NM_001079862_GAGGAGATGCTGTTCATCTA	DBI__NM_001079862	328	345	369	348
DBI__NM_001079862_TAAGACCAAGCCATCGGATG	DBI__NM_001079862	278	276	306	296
DBI__NM_001178017_ACGAGGAGAATCGCGGCCCG	DBI__NM_001178017	2022	1736	2142	2062
DBI__NM_001178017_GGGGAAGGGGTTGCACGGAT	DBI__NM_001178017	479	429	608	733
DBI__NM_001178042_CTCTGTCCCAGTGCCCGGAT	DBI__NM_001178042	1296	1147	1351	1360
DBI__NM_001178042_GCCTCTGCCAATCCGGGCAC	DBI__NM_001178042	767	802	1024	1234
DBN1__NM_004395_AAGAACTCCCTTGGGTTGTC	DBN1__NM_004395	343	309	340	355
DBN1__NM_004395_ATCGGGCAGCGGCTCTCTAA	DBN1__NM_004395	157	250	198	226
DBN1__NM_004395_CCACATGAAGAAGTCAGAGT	DBN1__NM_004395	754	705	812	744
DBN1__NM_004395_CCCAGCGGCCTGACAACCCA	DBN1__NM_004395	332	402	411	373
DBN1__NM_004395_GCAGCAGGAAAGAGTCGCAT	DBN1__NM_004395	991	1028	1152	913
DBN1__NM_004395_GCAGCGGCTCTCTAACGGGC	DBN1__NM_004395	349	347	352	395
DBN1__NM_004395_GCTGCTGGCGGCTTACGAGG	DBN1__NM_004395	539	523	603	541
DBN1__NM_004395_GGAGCTGCTGGCGGCTTACG	DBN1__NM_004395	802	780	1014	1223
DBN1__NM_004395_GGCTGGCACAAGCGCACTTG	DBN1__NM_004395	1029	1410	1219	1488
DBN1__NM_004395_GGTCGATGGTTGAAGGGCGA	DBN1__NM_004395	370	267	390	392
DBN1__NM_004395_TCGGGCAGCGGCTCTCTAAC	DBN1__NM_004395	462	515	522	546
DBN1__NM_004395_TTACGAGGAGGTGATCCGAG	DBN1__NM_004395	43	20	36	101
DBN1__NM_080881_ACTACCTGGTCGATGGTTGA	DBN1__NM_080881	562	833	634	632
DBN1__NM_080881_CGCTCTATCTGCTCAGCGAC	DBN1__NM_080881	384	337	401	504
DBN1__NM_080881_CTACCTGGTCGATGGTTGAA	DBN1__NM_080881	352	480	426	520
DBNDD1__NM_001042610_CAGCTCCTGGTCCGACATGT	DBNDD1__NM_001042610	1460	1360	1588	1346
DBNDD1__NM_001042610_CCCTTGTCCTCGTCCAGGAA	DBNDD1__NM_001042610	711	552	890	861
DBNDD1__NM_001042610_TAAGGAGGCTGAGGTGCCGC	DBNDD1__NM_001042610	454	372	515	369
DBNDD1__NM_001288709_ATGGCGCGAATTAACGCGGG	DBNDD1__NM_001288709	312	242	306	248
DBNDD1__NM_001288709_CAGTTTCATCGAAACTGCCT	DBNDD1__NM_001288709	0	0	0	0
DBNDD1__NM_001288709_CTGGATGGCGCGAATTAACG	DBNDD1__NM_001288709	362	251	355	443
DBNDD1__NM_001288709_CTTCCAGGTACTTGCGTGTG	DBNDD1__NM_001288709	1680	2332	1917	2041
DBNDD1__NM_001288709_GATGGCGCGAATTAACGCGG	DBNDD1__NM_001288709	176	148	200	160
DBNDD1__NM_001288709_GGAGGGGCCTGACTCGCGCT	DBNDD1__NM_001288709	2173	1797	2164	1649
DBNDD1__NM_001288709_GGATGGCGCGAATTAACGCG	DBNDD1__NM_001288709	335	313	417	369
DBNDD1__NM_001288709_TGGATGGCGCGAATTAACGC	DBNDD1__NM_001288709	1	18	10	17
DBNDD1__NM_024043_GAGGCATGGTTGGTGGCTAA	DBNDD1__NM_024043	200	103	245	258
DBNDD2__NM_001048221_CACTTGGATTTGGGCTATGC	DBNDD2__NM_001048221	570	880	668	979
DBNDD2__NM_001048221_CCAGGGCGGCCCGAGGATTT	DBNDD2__NM_001048221	700	707	652	523
DBNDD2__NM_001048221_GGATGAGATACTACCTATGG	DBNDD2__NM_001048221	560	317	645	492
DBNDD2__NM_001048221_TGCCGGTGCCTACATCAGAC	DBNDD2__NM_001048221	651	586	621	739
DBNDD2__NM_001048222_GGGCATACCAGACGACTGGG	DBNDD2__NM_001048222	822	871	1026	1054
DBNDD2__NM_001048225_AAGAGAGATACTCTGTATTC	DBNDD2__NM_001048225	706	574	769	643
DBNDD2__NM_001048225_CCAAGTCTCATGCAGAGGCG	DBNDD2__NM_001048225	408	634	421	547
DBNDD2__NM_001048225_CCCCGCCTCTGCATGAGACT	DBNDD2__NM_001048225	892	1276	1053	1426
DBNDD2__NM_001048225_GCAGCCAGCATCCCAACAGT	DBNDD2__NM_001048225	107	52	123	113
DBNDD2__NM_018478_GGCTACTGGTACTTCCAAGG	DBNDD2__NM_018478	232	183	245	267
DBNL__NM_001014436_ACGCCGGGAGCAGCGCTATC	DBNL__NM_001014436	840	972	955	973
DBNL__NM_001014436_AGCAGCAAGAAGTGGTTTCA	DBNL__NM_001014436	375	401	415	404
DBNL__NM_001014436_AGGGCTCTCTTTACCTATGA	DBNL__NM_001014436	161	238	210	155
DBNL__NM_001014436_CAAATTTGTCCTCATCAACT	DBNL__NM_001014436	784	1131	915	1281
DBNL__NM_001014436_CCCATGTGACCATCAACGCA	DBNL__NM_001014436	223	232	290	330
DBNL__NM_001014436_CCGAGAAGTCCCCGACCGAC	DBNL__NM_001014436	1712	2073	1961	2120
DBNL__NM_001014436_CTCCTCGGCCCGTGCGTTGA	DBNL__NM_001014436	776	861	808	1083
DBNL__NM_001014436_CTCGGTGACCACCCGCACGT	DBNL__NM_001014436	842	1156	844	991
DBNL__NM_001014436_GCGAGGGCGTGAACGATGTG	DBNL__NM_001014436	712	714	847	964
DBNL__NM_001014436_GGAGGAGGAGAACCGTCGGC	DBNL__NM_001014436	1445	1520	1584	1869
DBNL__NM_001014436_TTCAAGGAACCGAAATGAGC	DBNL__NM_001014436	36	20	40	105
DBNL__NM_001014436_TTTAATCTCAGACACGGCAT	DBNL__NM_001014436	58	147	132	77
DBNL__NM_001284313_AAAGGAGATCTCTGTGTCGT	DBNL__NM_001284313	496	694	631	753
DBNL__NM_001284313_CACTCCTCCATGTCTGGTGC	DBNL__NM_001284313	1471	1407	1705	1894
DBNL__NM_001284313_CAGTGGGGGCTGCTCGTAGA	DBNL__NM_001284313	373	434	421	492
DBNL__NM_001284313_GGCATCGAGGTGATCGACGA	DBNL__NM_001284313	614	656	671	829
DBNL__NM_001284313_TACGAGCAGCCCCCACTGGT	DBNL__NM_001284313	2118	1697	2390	1837
DBP__NM_001352_ATCAGCTGGGTCGGGTTCAA	DBP__NM_001352	397	404	449	459
DBP__NM_001352_CACGCTGCCGTTCGGCGATG	DBP__NM_001352	632	762	766	1040
DBP__NM_001352_CGATGTGGAGTACGTAGACC	DBP__NM_001352	650	591	652	701
DBP__NM_001352_CGTGGCCAGGAATGCTTGAT	DBP__NM_001352	1038	976	1319	1157
DBP__NM_001352_CTGGGGCAGTGGTGGGCGGA	DBP__NM_001352	540	419	603	687
DBP__NM_001352_CTTGATAGGGCAAGATCAGC	DBP__NM_001352	344	338	345	450
DBP__NM_001352_GAAGCCGAGCCGCACGAACC	DBP__NM_001352	782	1031	943	1087
DBP__NM_001352_GCGATGTCTTCGAGGGTCAA	DBP__NM_001352	754	1004	725	1052
DBP__NM_001352_GCTCAAGGAGAACCAGATAT	DBP__NM_001352	426	315	525	457
DBP__NM_001352_GGGTCAAAGGTCTCGTGGCC	DBP__NM_001352	640	997	731	1048
DBP__NM_001352_TCCCCAGGGCCGGGTTCGTG	DBP__NM_001352	638	694	873	793
DBP__NM_001352_TGGGAGCGCACGCTGCCGTT	DBP__NM_001352	209	174	254	162
DBR1__NM_016216_AAATACCGAGGTGTAAGGAT	DBR1__NM_016216	275	0	363	0
DBR1__NM_016216_AAGAGTTACCCTATGGTGGC	DBR1__NM_016216	345	18	337	61
DBR1__NM_016216_AGAGTTACCCTATGGTGGCT	DBR1__NM_016216	263	0	316	10
DBR1__NM_016216_CAGCACAGCAAGAGGTCGAC	DBR1__NM_016216	404	25	408	54
DBR1__NM_016216_CATTTGCAAGAGTTACCCTA	DBR1__NM_016216	636	17	724	3
DBR1__NM_016216_CGAGGCGGATCTACGCTGCA	DBR1__NM_016216	723	1	801	12
DBR1__NM_016216_GCTGGTGTGGTAAAATACCG	DBR1__NM_016216	428	0	494	0
DBR1__NM_016216_GGATAAGATCTATGAGACGC	DBR1__NM_016216	503	21	590	36
DBR1__NM_016216_GGTTTGCATGTGACGATACT	DBR1__NM_016216	262	115	331	158
DBR1__NM_016216_GTTTGCATGTGACGATACTT	DBR1__NM_016216	356	61	419	121
DBR1__NM_016216_TAACTCTTGCAAATGATTTG	DBR1__NM_016216	257	1	272	0
DBR1__NM_016216_TGGTAAAATACCGAGGTGTA	DBR1__NM_016216	4	0	11	0
DBT__NM_001918_AAACCCATTGCATTTGCTCG	DBT__NM_001918	95	204	122	159
DBT__NM_001918_ACATACACACCAAGAGATAA	DBT__NM_001918	196	171	243	212
DBT__NM_001918_ACCATCACTAGTCGTTATGA	DBT__NM_001918	708	537	688	710
DBT__NM_001918_AGAAGATATCCTCAACTATT	DBT__NM_001918	304	322	317	281
DBT__NM_001918_ATAGAAACGGAAGCTTTAAA	DBT__NM_001918	132	44	173	82
DBT__NM_001918_ATTAAGCTGAGTGAAGTTGT	DBT__NM_001918	514	898	601	541
DBT__NM_001918_CTAGACGATATTGCCTATGT	DBT__NM_001918	1463	969	1378	850
DBT__NM_001918_GGATGACTATACTTGAATGA	DBT__NM_001918	430	545	456	485
DBT__NM_001918_TAGACGATATTGCCTATGTG	DBT__NM_001918	592	180	566	387
DBT__NM_001918_TCCATCATAACGACTAGTGA	DBT__NM_001918	292	279	374	185
DBT__NM_001918_TCTAGACGATATTGCCTATG	DBT__NM_001918	399	636	560	493
DBT__NM_001918_TGCTCCAGGTTCTCAGCATA	DBT__NM_001918	0	0	0	0
DBX1__NM_001029865_AGCGCGAACTCCTGTCTAGC	DBX1__NM_001029865	692	765	752	1191
DBX1__NM_001029865_AGTTCCGCCATTTCATGCGT	DBX1__NM_001029865	1201	936	1298	1320
DBX1__NM_001029865_CCCACGGCCCTCACCGACAC	DBX1__NM_001029865	303	522	367	531
DBX1__NM_001029865_CGCGAACTCCTGTCTAGCGG	DBX1__NM_001029865	1611	1144	1739	1722
DBX1__NM_001029865_CGCGACAGCCCCCGCTAGAC	DBX1__NM_001029865	1805	2630	2199	2939
DBX1__NM_001029865_CTTCAGAAACGTCGTCTCGC	DBX1__NM_001029865	383	493	386	405
DBX1__NM_001029865_GACTGGGGCAGCGTCAAGGT	DBX1__NM_001029865	421	342	406	520
DBX1__NM_001029865_GCGCGAACTCCTGTCTAGCG	DBX1__NM_001029865	2010	1840	2255	3237
DBX1__NM_001029865_GCTGGGCCTGAAAGACTCGC	DBX1__NM_001029865	980	1001	917	1013
DBX1__NM_001029865_GGAAAGGCGAAGGTCTTGGG	DBX1__NM_001029865	527	434	641	787
DBX1__NM_001029865_TCCAGAACCGACGCATGAAA	DBX1__NM_001029865	257	589	427	576
DBX1__NM_001029865_TCGCCTTTCCCTACTTCGAA	DBX1__NM_001029865	447	931	423	577
DBX2__NM_001004329_AAAAGCCCACCGCGTTCCGT	DBX2__NM_001004329	1344	1018	1440	879
DBX2__NM_001004329_AAGGCTGGAAGGTACAGTCT	DBX2__NM_001004329	523	470	416	206
DBX2__NM_001004329_AGCCCACGCCGGTGCGTACT	DBX2__NM_001004329	1076	1263	1285	808
DBX2__NM_001004329_AGCGGGGAGGTTGAGGAGCG	DBX2__NM_001004329	544	105	568	89
DBX2__NM_001004329_CACAACGTCCCAGTACGCAC	DBX2__NM_001004329	589	428	611	331
DBX2__NM_001004329_CAGCCCACGCCGGTGCGTAC	DBX2__NM_001004329	1049	1263	1130	1192
DBX2__NM_001004329_CCCGCAGCAAATTCTCGATC	DBX2__NM_001004329	1438	1462	1853	1298
DBX2__NM_001004329_CCGAACAAGTTAGCCCCGCC	DBX2__NM_001004329	1310	1456	1381	1949
DBX2__NM_001004329_CGATCAGGAAACTCTTGCCC	DBX2__NM_001004329	3570	3401	3206	1771
DBX2__NM_001004329_CTTGCCCAGGTTGCCAAAGC	DBX2__NM_001004329	518	503	556	636
DBX2__NM_001004329_GGCACAGCTTTAGGGGAACT	DBX2__NM_001004329	1219	1812	1524	1541
DBX2__NM_001004329_GGCGCCCTACGGAACGCGGT	DBX2__NM_001004329	679	782	769	489
DCAF10__NM_001286810_AAGAAATCATTCGTCCATCG	DCAF10__NM_001286810	670	396	649	681
DCAF10__NM_001286810_ACAAGACTCTTAGTAACATC	DCAF10__NM_001286810	131	121	123	114
DCAF10__NM_001286810_ACCATGTAAAGTGCATACTT	DCAF10__NM_001286810	552	641	557	587
DCAF10__NM_001286810_ACGACTTCAAGACTATTTCG	DCAF10__NM_001286810	601	719	776	897
DCAF10__NM_001286810_AGATGCTCAGTCGCTCACAC	DCAF10__NM_001286810	489	403	496	667
DCAF10__NM_001286810_CTAAAGACTTAGTTAAGTCA	DCAF10__NM_001286810	96	100	127	98
DCAF10__NM_001286810_GAAGAAATCATTCGTCCATC	DCAF10__NM_001286810	652	687	662	666
DCAF10__NM_001286810_GTGTGAAAGAATTTCTTATG	DCAF10__NM_001286810	177	234	163	149
DCAF10__NM_001286810_TCATAGACACAAGTACACTA	DCAF10__NM_001286810	227	257	232	260
DCAF10__NM_001286810_TCATTATTTGGGACACTAAC	DCAF10__NM_001286810	173	218	285	314
DCAF10__NM_001286810_TGACACTACAATAGCACTAT	DCAF10__NM_001286810	182	161	219	189
DCAF10__NM_001286810_TTGAAGAAGCCAATGTAGGC	DCAF10__NM_001286810	165	179	178	191
DCAF10__NM_024345_ACCCGTCGGGCGAGTACTCG	DCAF10__NM_024345	602	548	644	654
DCAF10__NM_024345_AGCCCCGGGATGTAGTGGCG	DCAF10__NM_024345	1307	1209	1256	1056
DCAF10__NM_024345_CACCCGAACAGCCTAGCGCC	DCAF10__NM_024345	175	115	145	249
DCAF10__NM_024345_CGCACCATGACTAGCCTCTA	DCAF10__NM_024345	1011	1217	1325	1799
DCAF10__NM_024345_CGGGGTGGATGGAACCGTAG	DCAF10__NM_024345	36	101	53	35
DCAF10__NM_024345_GAGGAGCCGACGCCCCACGA	DCAF10__NM_024345	1099	971	1140	1114
DCAF10__NM_024345_GCCCTGGCGCTAGGCTGTTC	DCAF10__NM_024345	3032	3134	3207	2956
DCAF10__NM_024345_GTGCGAAAATTGTCCCGCGC	DCAF10__NM_024345	1073	1504	1201	1231
DCAF10__NM_024345_TTGGGCCCCATAGCCCTGGA	DCAF10__NM_024345	79	57	115	225
DCAF11__NM_001163484_AGAGCTTGGGATGGTCGTCT	DCAF11__NM_001163484	574	615	568	605
DCAF11__NM_001163484_AGCTTCTTGCCCAATGATCT	DCAF11__NM_001163484	206	252	264	176
DCAF11__NM_001163484_AGTCATAGAGTCGGATTGTC	DCAF11__NM_001163484	446	314	443	428
DCAF11__NM_001163484_ATTCCAGCTCCCGGGTGTCA	DCAF11__NM_001163484	434	384	499	368
DCAF11__NM_001163484_CATATCGGCAGTCATAGAGT	DCAF11__NM_001163484	625	690	539	570
DCAF11__NM_001163484_CCTACCTGGTGCAACATTCG	DCAF11__NM_001163484	840	1027	862	1038
DCAF11__NM_001163484_CGACTCTATGACTGCCGATA	DCAF11__NM_001163484	747	1063	960	1316
DCAF11__NM_001163484_CTCTTCCTCTGAGTCCAAGA	DCAF11__NM_001163484	23	17	37	4
DCAF11__NM_001163484_GATCATTGGGCAAGAAGCTA	DCAF11__NM_001163484	135	203	160	177
DCAF11__NM_001163484_GGCTGGCCTGCGTCGGAGTG	DCAF11__NM_001163484	966	1309	1059	1384
DCAF11__NM_001163484_GGGGATCGATACAACCCACC	DCAF11__NM_001163484	364	451	434	436
DCAF11__NM_001163484_TCAGTGAAGCCCAGATCATT	DCAF11__NM_001163484	595	684	651	483
DCAF12__NM_015397_AACTACTTTCCGGGCCATAG	DCAF12__NM_015397	64	128	91	47
DCAF12__NM_015397_ACACTTTATTAAGGGTCCCA	DCAF12__NM_015397	99	86	110	46
DCAF12__NM_015397_ACTACTTTCCGGGCCATAGT	DCAF12__NM_015397	896	850	1075	1112
DCAF12__NM_015397_AGCTACTCTCGAGTGTTGCA	DCAF12__NM_015397	717	731	888	851
DCAF12__NM_015397_CAGCGTAGGTAGTCGATAGA	DCAF12__NM_015397	146	255	220	264
DCAF12__NM_015397_CATCTCAGTGGTTGAATCAT	DCAF12__NM_015397	465	560	395	363
DCAF12__NM_015397_CGATAGATGGCAAGACTGTT	DCAF12__NM_015397	1403	1744	1361	1567
DCAF12__NM_015397_GAGATGCAAACACTTTATTA	DCAF12__NM_015397	602	441	567	684
DCAF12__NM_015397_GGAGCTGGGAGCGACGCTCA	DCAF12__NM_015397	161	170	166	134
DCAF12__NM_015397_GTTCTTCAAGTAGTATACTA	DCAF12__NM_015397	20	57	16	21
DCAF12__NM_015397_TCGATAGATGGCAAGACTGT	DCAF12__NM_015397	937	795	951	700
DCAF12__NM_015397_TGGCCCGGAAAGTAGTTAGC	DCAF12__NM_015397	0	0	0	0
DCAF12L1__NM_178470_ACAGTGTCATCGAACTTGTC	DCAF12L1__NM_178470	465	731	663	846
DCAF12L1__NM_178470_CAGCGCCACAGTGCCGTCGC	DCAF12L1__NM_178470	1148	1143	1396	1355
DCAF12L1__NM_178470_GCCATCGAGCGATACGTCGC	DCAF12L1__NM_178470	546	402	549	495
DCAF12L1__NM_178470_GCCGAGAGCTCGCCGTCGCA	DCAF12L1__NM_178470	1052	1627	1231	1656
DCAF12L1__NM_178470_GCCGGCGACGTATCGCTCGA	DCAF12L1__NM_178470	939	1236	909	1355
DCAF12L1__NM_178470_GCGGGAGCCGCTCACGGCTA	DCAF12L1__NM_178470	58	143	136	76
DCAF12L1__NM_178470_GCTGCCGCCAAGCCCTGCGA	DCAF12L1__NM_178470	232	181	196	203
DCAF12L1__NM_178470_GGCCTCACTGTCCCGCAAGA	DCAF12L1__NM_178470	585	738	752	672
DCAF12L1__NM_178470_GTTCGGGAGGTAGGCGGGTG	DCAF12L1__NM_178470	220	214	206	189
DCAF12L1__NM_178470_TGCCGCAGCCCTGTTGGTCC	DCAF12L1__NM_178470	3472	4722	3940	5218
DCAF12L1__NM_178470_TGCGGGGCTACGCGGTACAG	DCAF12L1__NM_178470	248	346	225	252
DCAF12L1__NM_178470_TGTTGCCTGGCATAGCGAGG	DCAF12L1__NM_178470	1	1	4	1
DCAF12L2__NM_001013628_ATGGACCCAGACATGTTTAA	DCAF12L2__NM_001013628	183	247	180	156
DCAF12L2__NM_001013628_CCCCAGCAACCGCAAGGTAC	DCAF12L2__NM_001013628	394	431	402	481
DCAF12L2__NM_001013628_CGGCCTCCTTGTCCCGCATG	DCAF12L2__NM_001013628	2243	2922	2507	3138
DCAF12L2__NM_001013628_GCCGGGAGCTCGTCGTCGCA	DCAF12L2__NM_001013628	1043	1372	1298	1786
DCAF12L2__NM_001013628_GCCTCCTTGTCCCGCATGAG	DCAF12L2__NM_001013628	538	668	626	497
DCAF12L2__NM_001013628_GGCCTCCTTGTCCCGCATGA	DCAF12L2__NM_001013628	768	1006	928	1318
DCAF12L2__NM_001013628_GTCGTCGCAGGGTTTAGCGG	DCAF12L2__NM_001013628	658	704	656	645
DCAF12L2__NM_001013628_GTGACGCGAACACCTTGTTG	DCAF12L2__NM_001013628	3060	3052	3223	3342
DCAF12L2__NM_001013628_TGAGGGGGATGCGCGTGATG	DCAF12L2__NM_001013628	483	499	523	493
DCAF12L2__NM_001013628_TGCTGCCATTAAACATGTCT	DCAF12L2__NM_001013628	214	238	260	207
DCAF12L2__NM_001013628_TGGTGCACTATCTGAAGGGC	DCAF12L2__NM_001013628	21	3	25	16
DCAF12L2__NM_001013628_TTTGTGGTGGACGTGCAGTC	DCAF12L2__NM_001013628	275	223	285	421
DCAF13__NM_015420_AGCCGGAGACTCTAGTACTG	DCAF13__NM_015420	634	501	685	629
DCAF13__NM_015420_CCACCTCGGGACCAACGAG	DCAF13__NM_015420	5	0	8	3
DCAF13__NM_015420_CCGGAGACTCTAGTACTGAG	DCAF13__NM_015420	583	795	702	782
DCAF13__NM_015420_CGAGAGGACTCTTATGCCCA	DCAF13__NM_015420	1306	1291	1460	1323
DCAF13__NM_015420_CGCCCCTTGCTAACGTTTGC	DCAF13__NM_015420	3877	4514	4487	5530
DCAF13__NM_015420_CGGAAGAGCAACCGAGATGA	DCAF13__NM_015420	696	320	644	288
DCAF13__NM_015420_GAAAAGCGGAAGTCGCCTGT	DCAF13__NM_015420	4349	4462	4781	3871
DCAF13__NM_015420_GAGGTGGGGTTGCGCTCACA	DCAF13__NM_015420	564	477	682	527
DCAF13__NM_015420_GCAGTCGCTTCCGCGTCCGT	DCAF13__NM_015420	651	584	684	819
DCAF13__NM_015420_GTTTCGCGGACATAATTGTC	DCAF13__NM_015420	158	0	177	15
DCAF13__NM_015420_TTGCCGGCAAAACTACGGAG	DCAF13__NM_015420	237	163	238	237
DCAF13__NM_015420_TTTGCCGGCAAACGTTAGCA	DCAF13__NM_015420	1352	1307	1504	1429
DCAF15__NM_138353_ACTCGCATACGGTCAGGTAC	DCAF15__NM_138353	1153	1252	1278	1266
DCAF15__NM_138353_AGGGACACGCACACCCGGGG	DCAF15__NM_138353	431	198	533	287
DCAF15__NM_138353_ATGAGGACTTCCTCTATGCA	DCAF15__NM_138353	534	457	507	491
DCAF15__NM_138353_CGCCCAGCTCGAAATCGGAG	DCAF15__NM_138353	452	546	475	471
DCAF15__NM_138353_GGGAGAGCTGTCCGCTGATC	DCAF15__NM_138353	732	966	816	1027
DCAF15__NM_138353_GTCGCTGGGCCACTCGCATA	DCAF15__NM_138353	275	127	282	280
DCAF15__NM_138353_GTTCCGCTCCGATTTCGAGC	DCAF15__NM_138353	199	187	230	196
DCAF15__NM_138353_TCTACATCTACCATCTGTAC	DCAF15__NM_138353	736	533	929	902
DCAF15__NM_138353_TGGGCCACTCGCATACGGTC	DCAF15__NM_138353	51	56	49	59
DCAF15__NM_138353_TGGTGTAGGAGAGGACGTAG	DCAF15__NM_138353	0	0	0	0
DCAF15__NM_138353_TGTACCTGACCGTATGCGAG	DCAF15__NM_138353	363	344	440	443
DCAF15__NM_138353_TTCCGCTCCGATTTCGAGCT	DCAF15__NM_138353	155	166	143	154
DCAF16__NM_017741_ACTCTCAAGAGGCGATAAGT	DCAF16__NM_017741	487	444	466	494
DCAF16__NM_017741_AGAGATCATGCCACTCTAAA	DCAF16__NM_017741	188	226	247	306
DCAF16__NM_017741_AGCCGGCTGGGACTTGTAAG	DCAF16__NM_017741	1159	1434	1192	1425
DCAF16__NM_017741_CTCTCAAGAGGCGATAAGTT	DCAF16__NM_017741	184	394	279	367
DCAF16__NM_017741_GACCTATCTCTCGAAGTATA	DCAF16__NM_017741	213	376	376	386
DCAF16__NM_017741_GCATGATACAACCAGGAATT	DCAF16__NM_017741	359	445	388	485
DCAF16__NM_017741_GGGGACACAATGGAACAAT	DCAF16__NM_017741	454	479	428	521
DCAF16__NM_017741_GTCCATATACTTCGAGAGAT	DCAF16__NM_017741	459	446	512	434
DCAF16__NM_017741_GTTGTATCATGCTAAACTGT	DCAF16__NM_017741	635	714	748	664
DCAF16__NM_017741_TCTGATTCTGACAAGTGGTC	DCAF16__NM_017741	58	42	133	177
DCAF16__NM_017741_TCTTGAGAGTCTTGCCTGGC	DCAF16__NM_017741	640	743	877	1048
DCAF16__NM_017741_TGATCTCTAGAGAGCCGGCT	DCAF16__NM_017741	255	284	354	425
DCAF17__NM_001164821_ACAGATGCTACCTTGTCTCA	DCAF17__NM_001164821	1147	888	1114	1250
DCAF17__NM_001164821_ACCAGATGATATGTGACACT	DCAF17__NM_001164821	1019	1137	1226	1090
DCAF17__NM_001164821_CAAGTGAAAAAGGTAGAACT	DCAF17__NM_001164821	424	348	479	437
DCAF17__NM_001164821_CATTCCACTAGTGGAGTCAT	DCAF17__NM_001164821	671	747	801	685
DCAF17__NM_001164821_CCCCAGTGTCACATATCATC	DCAF17__NM_001164821	1121	942	1159	1303
DCAF17__NM_001164821_CGTGGTGCAGAGGACCAACC	DCAF17__NM_001164821	348	243	466	439
DCAF17__NM_001164821_CTCATAGGCTATAGGTGACC	DCAF17__NM_001164821	182	185	177	238
DCAF17__NM_001164821_GGTGTGACGATGTAGTGCCA	DCAF17__NM_001164821	320	263	468	584
DCAF17__NM_001164821_TGCAGATGGGGTGGGACTAC	DCAF17__NM_001164821	519	472	739	811
DCAF17__NM_001164821_TGTTACAGCTTCTGGACGGG	DCAF17__NM_001164821	576	531	469	392
DCAF17__NM_025000_GTTGTTCTCTAGAATCTGAC	DCAF17__NM_025000	28	135	8	6
DCAF17__NM_025000_TCTGGTAGAATAATACATGT	DCAF17__NM_025000	355	374	384	315
DCAF4__NM_001163508_AAAGGTCCAGATTCGAAGCA	DCAF4__NM_001163508	217	216	263	370
DCAF4__NM_001163508_AATCACGGAGTCTGAACCAA	DCAF4__NM_001163508	241	245	234	169
DCAF4__NM_001163508_ACCGTGGCCGGAATCGTGAG	DCAF4__NM_001163508	406	414	503	510
DCAF4__NM_001163508_CAGAAATGAATAAAAGTCGC	DCAF4__NM_001163508	349	434	368	301
DCAF4__NM_001163508_CAGGTTGATGATACCATACT	DCAF4__NM_001163508	427	346	597	509
DCAF4__NM_001163508_CATGAACACCTTGAGCGTAG	DCAF4__NM_001163508	257	220	318	261
DCAF4__NM_001163508_CGACCGATTTAACCTCATAC	DCAF4__NM_001163508	3055	3533	3507	3928
DCAF4__NM_001163508_GAGTCTCCGTCAACCTCGTC	DCAF4__NM_001163508	763	789	743	948
DCAF4__NM_001163508_GGATGCTCTCTTTCGTCAGG	DCAF4__NM_001163508	1313	1733	1754	2161
DCAF4__NM_001163508_GGGTTGCAGTTGTTATGTCC	DCAF4__NM_001163508	709	743	726	940
DCAF4__NM_001163508_TCTGAAGACCCCTACGCTCA	DCAF4__NM_001163508	481	433	492	297
DCAF4__NM_001163508_TGGCAGAGTAGAAGACGACA	DCAF4__NM_001163508	21	3	20	32
DCAF4__NM_001163509_ACCAGGTAAATCGGTGTGC	DCAF4__NM_001163509	428	490	652	690
DCAF4__NM_001163509_GCATGAACACCTTGAGCGTA	DCAF4__NM_001163509	544	808	601	815
DCAF4__NM_181340_AGGACCAGGCACCAGGGATC	DCAF4__NM_181340	463	301	534	356
DCAF4__NM_181340_GAGTCCCATGAGGCATAGCC	DCAF4__NM_181340	0	0	0	0
DCAF4__NM_181340_GGAATCCAAGTGATTCAGCG	DCAF4__NM_181340	168	334	243	396
DCAF4__NM_181340_GTTATTTGCTTGGATATTCA	DCAF4__NM_181340	103	181	117	123
DCAF4__NM_181340_TTAGCAGGAATAGACCGGCC	DCAF4__NM_181340	218	53	296	282
DCAF4L1__NM_001029955_ACGCAGCTCGTTGGCTAAAC	DCAF4L1__NM_001029955	815	1016	922	1066
DCAF4L1__NM_001029955_AGATGCATTAAATCCCATTC	DCAF4L1__NM_001029955	1945	1853	2255	2595
DCAF4L1__NM_001029955_AGCACGTACACGTGGAACGA	DCAF4L1__NM_001029955	391	331	341	417
DCAF4L1__NM_001029955_AGGGATCCAAGCTCCGAATT	DCAF4L1__NM_001029955	416	480	442	379
DCAF4L1__NM_001029955_CCAGCTCTTCGTAGTGAACC	DCAF4L1__NM_001029955	299	198	355	403
DCAF4L1__NM_001029955_CCTCTACGTCCCCAACCGGA	DCAF4L1__NM_001029955	396	470	482	431
DCAF4L1__NM_001029955_CGACCGATTTAACTTCATTC	DCAF4L1__NM_001029955	1057	1414	1303	1787
DCAF4L1__NM_001029955_CTGAAAAAGGTAGCCAGAAT	DCAF4L1__NM_001029955	346	638	442	716
DCAF4L1__NM_001029955_GCACGTACACGTGGAACGAA	DCAF4L1__NM_001029955	123	196	166	130
DCAF4L1__NM_001029955_GCTGAAAAAGGTAGCCAGAA	DCAF4L1__NM_001029955	286	320	341	453
DCAF4L1__NM_001029955_GTTGGCTAAACGGGAGTAAC	DCAF4L1__NM_001029955	165	248	164	221
DCAF4L1__NM_001029955_TGGAGCCTTCGACTTCGACC	DCAF4L1__NM_001029955	712	815	993	964
DCAF4L2__NM_152418_AAAAGGTCCAGATTCATAGC	DCAF4L2__NM_152418	41	66	16	36
DCAF4L2__NM_152418_AGGGATCCAGCTATGAATC	DCAF4L2__NM_152418	146	240	192	162
DCAF4L2__NM_152418_CACGCAGCTCGCGAGCTATA	DCAF4L2__NM_152418	357	171	259	221
DCAF4L2__NM_152418_CAGAAAAAGACAGTCAGAGT	DCAF4L2__NM_152418	664	388	805	558
DCAF4L2__NM_152418_CGACCGATTTAACCGCATAC	DCAF4L2__NM_152418	1202	1267	1263	1494
DCAF4L2__NM_152418_GCGAGCTATACGGCAATAGT	DCAF4L2__NM_152418	1316	1201	1591	1526
DCAF4L2__NM_152418_TACGGCATCATCACCATGCG	DCAF4L2__NM_152418	2234	2817	2730	3153
DCAF4L2__NM_152418_TAGCTGGTTCTTTCGTAGCA	DCAF4L2__NM_152418	3	2	10	10
DCAF4L2__NM_152418_TCGCCAGTATGCGGTTAAAT	DCAF4L2__NM_152418	1014	1310	1002	1288
DCAF4L2__NM_152418_TCTGCTTCCTCGAGCAGTCG	DCAF4L2__NM_152418	333	402	289	423
DCAF4L2__NM_152418_TGGTTCTTTCGTAGCATGGA	DCAF4L2__NM_152418	0	0	0	0
DCAF4L2__NM_152418_TGTTGGTATTCGCCAGTATG	DCAF4L2__NM_152418	159	136	188	230
DCAF5__NM_001284206_AGGCTGATGGATAGTTTGCC	DCAF5__NM_001284206	471	452	461	420
DCAF5__NM_001284206_ATGACCGCCGGGTTCTGCTA	DCAF5__NM_001284206	469	502	586	668
DCAF5__NM_001284206_ATGATGGCCGGGTTCTCATT	DCAF5__NM_001284206	2333	2643	2856	2796
DCAF5__NM_001284206_CCATGTGCCATAGCAGAACC	DCAF5__NM_001284206	223	415	347	267
DCAF5__NM_001284206_CTTTCAGAGGAGACGCCTGC	DCAF5__NM_001284206	229	259	292	310
DCAF5__NM_001284206_GAATGTCCCAAATGAGAACC	DCAF5__NM_001284206	121	77	146	133
DCAF5__NM_001284206_GACATTCGGGAATCCCCCCA	DCAF5__NM_001284206	504	613	607	548
DCAF5__NM_001284206_GCTCATGAAGATGCAGTATA	DCAF5__NM_001284206	71	41	92	91
DCAF5__NM_001284206_GCTGTTGAATCGTACACTCA	DCAF5__NM_001284206	768	808	734	1052
DCAF5__NM_001284206_GTGGGCTTCTTGTCCCAGCG	DCAF5__NM_001284206	41	41	27	26
DCAF5__NM_001284206_TGATGGCCGGGTTCTCATTT	DCAF5__NM_001284206	948	1017	1135	766
DCAF5__NM_001284206_TGCTCTCCTTCAGCTGTAT	DCAF5__NM_001284206	293	270	255	239
DCAF5__NM_001284207_GAATGGATGTCATAGAGCAC	DCAF5__NM_001284207	305	301	409	362
DCAF5__NM_001284207_TGATTGTCAAACTGAAACAC	DCAF5__NM_001284207	172	268	207	280
DCAF5__NM_001284207_TGGATGTCATAGAGCACAGG	DCAF5__NM_001284207	23	19	32	45
DCAF5__NM_001284207_TGTGTTTCAGTTTGACAATC	DCAF5__NM_001284207	120	141	240	212
DCAF6__NM_001017977_AAACAGATTGTATCCTGCTC	DCAF6__NM_001017977	93	33	61	52
DCAF6__NM_001017977_ACAGCAAGGTAATATGGTAT	DCAF6__NM_001017977	417	427	450	436
DCAF6__NM_001017977_ATGGAACTACTTATGAGGTA	DCAF6__NM_001017977	297	341	377	497
DCAF6__NM_001017977_CAACCAACAGCAAGGTAATA	DCAF6__NM_001017977	149	276	148	236
DCAF6__NM_001017977_CAAGGTAATATGGTATTGGT	DCAF6__NM_001017977	266	369	307	228
DCAF6__NM_001017977_CACAACCATCATGCACATTA	DCAF6__NM_001017977	1315	1502	1204	1647
DCAF6__NM_001017977_CACAAGAGAGAAAAGTGTAA	DCAF6__NM_001017977	70	77	96	62
DCAF6__NM_001017977_CGGCTGCGGAGTCGATACCT	DCAF6__NM_001017977	858	1190	839	925
DCAF6__NM_001017977_GCAACCCTTAATGTGCATGA	DCAF6__NM_001017977	925	1195	934	1262
DCAF6__NM_001017977_TCATTATGGAACTACTTATG	DCAF6__NM_001017977	488	485	490	484
DCAF6__NM_001017977_TGCCAATTTACGTGTCATTA	DCAF6__NM_001017977	250	418	441	657
DCAF6__NM_001017977_TTCCATAATGACACGTAAAT	DCAF6__NM_001017977	62	41	82	99
DCAF7__NM_005828_CGCCAAGCGAAAGCGCTTAT	DCAF7__NM_005828	232	308	267	429
DCAF7__NM_005828_GCATGTCGTATCAATGCTTG	DCAF7__NM_005828	1116	1335	1113	1190
DCAF7__NM_005828_GCCAAGCGAAAGCGCTTATC	DCAF7__NM_005828	200	43	194	55
DCAF7__NM_005828_GCCCGATAAGCGCTTTCGCT	DCAF7__NM_005828	1251	1006	1489	945
DCAF7__NM_005828_GGACAGTCTACGCGATGAAC	DCAF7__NM_005828	562	296	562	282
DCAF7__NM_005828_GGGCGAGTGAATCTCGTGTC	DCAF7__NM_005828	955	1270	1078	914
DCAF7__NM_005828_GGTGGGGTATGGGTGGTCAA	DCAF7__NM_005828	230	222	153	117
DCAF7__NM_005828_GTCCCTGCACGGCAAACGGA	DCAF7__NM_005828	136	66	123	47
DCAF7__NM_005828_TCAGCTTGTTGGTTTAGATG	DCAF7__NM_005828	498	430	458	285
DCAF7__NM_005828_TCTCCTTCCGTTTGCCGTGC	DCAF7__NM_005828	364	467	533	606
DCAF7__NM_005828_TGATACGACATGCACCATCT	DCAF7__NM_005828	347	195	364	86
DCAF7__NM_005828_TTTGTCATGGGCGATCAGCT	DCAF7__NM_005828	146	82	182	166
DCAF8__NM_015726_AACAGACTTAGCTAATGGTA	DCAF8__NM_015726	474	374	580	397
DCAF8__NM_015726_AAGCCTGTCTAGCAGTCCAG	DCAF8__NM_015726	215	178	276	269
DCAF8__NM_015726_ACCCAGCTCCCGCTCCCGAA	DCAF8__NM_015726	947	602	893	705
DCAF8__NM_015726_CCACTCTCAAAGTCCAGTAC	DCAF8__NM_015726	457	434	583	648
DCAF8__NM_015726_CTTTCGTGCTGGTGCGGTT	DCAF8__NM_015726	620	872	666	811
DCAF8__NM_015726_GAAGGTGGTGGTGTGGGATT	DCAF8__NM_015726	29	39	35	11
DCAF8__NM_015726_GGCAGAACAGACTTAGCTAA	DCAF8__NM_015726	166	339	259	194
DCAF8__NM_015726_GGCCTCCACTTCAATGCCTG	DCAF8__NM_015726	931	1064	1003	1227
DCAF8__NM_015726_GGCGCCGTGTACAGCGCAAG	DCAF8__NM_015726	192	248	206	285
DCAF8__NM_015726_TAGCCCGCTTGCGCTGTACA	DCAF8__NM_015726	151	48	189	166
DCAF8__NM_015726_TCTCCTCTGGACTGCTAGAC	DCAF8__NM_015726	308	460	340	316
DCAF8__NM_015726_TGGTGTGGGATTGGGTACGG	DCAF8__NM_015726	52	34	39	32
DCAF8L1__NM_001017930_ATGACTTAAGGGTGATAGTG	DCAF8L1__NM_001017930	161	146	112	159
DCAF8L1__NM_001017930_CCGCATCGTGGACACATCCG	DCAF8L1__NM_001017930	1175	1122	1321	1547
DCAF8L1__NM_001017930_CGGAGATGGTGGTGATACCA	DCAF8L1__NM_001017930	448	349	533	574
DCAF8L1__NM_001017930_GCAGCCACAGAGCCATCGAC	DCAF8L1__NM_001017930	1707	1788	1849	2286
DCAF8L1__NM_001017930_GCCACGCTGGTTAAAGTGTA	DCAF8L1__NM_001017930	502	710	561	806
DCAF8L1__NM_001017930_TCACCACCATCTCCGGTCGA	DCAF8L1__NM_001017930	2512	3490	3062	3539
DCAF8L1__NM_001017930_TCTGTCTCCTCTCCCACTAA	DCAF8L1__NM_001017930	457	444	554	418
DCAF8L1__NM_001017930_TGATCAGTGTTTGTTAGACG	DCAF8L1__NM_001017930	655	977	749	1225
DCAF8L1__NM_001017930_TGGAGTAGCAGCGGTGACGG	DCAF8L1__NM_001017930	164	136	150	196
DCAF8L1__NM_001017930_TGGTACTGACAGAACCGGCA	DCAF8L1__NM_001017930	0	0	0	0
DCAF8L1__NM_001017930_TTATTGTTAGCTACCCGTTAGT	DCAF8L1__NM_001017930	249	254	211	144
DCAF8L1__NM_001017930_TTTATTGGTTACCCGTTAG	DCAF8L1__NM_001017930	73	112	102	69
DCAF8L2__NM_001136533_ACAGACGGCTTACCAGACTT	DCAF8L2__NM_001136533	658	788	679	701
DCAF8L2__NM_001136533_AGGCTCCAGAGCCAACTTGT	DCAF8L2__NM_001136533	227	266	341	262
DCAF8L2__NM_001136533_AGGCTTTCAGTCCCTAAGTC	DCAF8L2__NM_001136533	215	189	274	242
DCAF8L2__NM_001136533_ATAAACTTTCACCACTCATG	DCAF8L2__NM_001136533	344	418	502	507
DCAF8L2__NM_001136533_CAGACGGCTTACCAGACTTA	DCAF8L2__NM_001136533	902	844	1021	1033
DCAF8L2__NM_001136533_CAGTAGCGGTGATGACCTAA	DCAF8L2__NM_001136533	335	362	318	505
DCAF8L2__NM_001136533_CCATGAGCAGTATTCGTTAG	DCAF8L2__NM_001136533	1233	1642	1333	1848
DCAF8L2__NM_001136533_GCAAATTGGTAGGTATTGGC	DCAF8L2__NM_001136533	520	839	513	700
DCAF8L2__NM_001136533_GGTCTGCAAGACGATATTGC	DCAF8L2__NM_001136533	441	506	493	596
DCAF8L2__NM_001136533_TGAGCAGTATTCGTTAGAGG	DCAF8L2__NM_001136533	518	506	614	476
DCAF8L2__NM_001136533_TTATTCCATTACCCTTTAGT	DCAF8L2__NM_001136533	307	220	455	365
DCAF8L2__NM_001136533_TTTATTCCATTACCCTTTAG	DCAF8L2__NM_001136533	94	50	93	111
DCAKD__NM_001128631_AGCACACCGTGGTAGTATAC	DCAKD__NM_001128631	228	197	245	218
DCAKD__NM_001128631_ATGTTTCTGGTGGGCCTGAC	DCAKD__NM_001128631	168	240	142	216
DCAKD__NM_001128631_CACCCACCCCGAGATTCGCA	DCAKD__NM_001128631	2198	2059	2263	2312
DCAKD__NM_001128631_CATCATCTCCTTGCGAATCT	DCAKD__NM_001128631	756	746	873	829
DCAKD__NM_001128631_CCTTCTGCCTTACGCCTAGT	DCAKD__NM_001128631	928	1140	1109	1046
DCAKD__NM_001128631_CGCCCGAGTTGTCTAGGACA	DCAKD__NM_001128631	2556	1917	2703	2157
DCAKD__NM_001128631_GACCTCTACGATGCGCCGGT	DCAKD__NM_001128631	1388	1248	1513	1387
DCAKD__NM_001128631_GGATATCCACGAGAATCACG	DCAKD__NM_001128631	309	296	338	420
DCAKD__NM_001128631_GGTCAGGCTGGTTAAAGATC	DCAKD__NM_001128631	245	151	337	229
DCAKD__NM_001128631_GTGGAGTGTCACCAAACGCC	DCAKD__NM_001128631	191	172	150	159
DCAKD__NM_001128631_TCATCTCCTTGCGAATCTCG	DCAKD__NM_001128631	537	623	635	701
DCAKD__NM_001128631_TCTCCTTGCGAATCTCGGGG	DCAKD__NM_001128631	369	572	460	418
DCANP1__NM_130848_AGAATGCAGTGGAGGTTGTT	DCANP1__NM_130848	951	728	1122	1088
DCANP1__NM_130848_AGATGCTTCACTCGAAAGAT	DCANP1__NM_130848	345	296	377	376
DCANP1__NM_130848_ATGCAGTGGAGGTTGTTGGG	DCANP1__NM_130848	814	791	1025	918
DCANP1__NM_130848_CACGGCCTGGAGACTGTACC	DCANP1__NM_130848	1166	1275	1168	1419
DCANP1__NM_130848_GCTGCTCCGTAATGATAGT	DCANP1__NM_130848	287	248	391	194
DCANP1__NM_130848_GCTTCTCGAGTTCTGTATGT	DCANP1__NM_130848	1250	1187	1546	1303
DCANP1__NM_130848_GGCAGTTCAATTCCTCCACA	DCANP1__NM_130848	645	791	790	758
DCANP1__NM_130848_GGCTTCTCGAGTTCTGTATG	DCANP1__NM_130848	481	742	425	594
DCANP1__NM_130848_TCCTAACAGCTGGAACCGTC	DCANP1__NM_130848	268	223	343	336
DCANP1__NM_130848_TGATATCAAGTGCGAAGACC	DCANP1__NM_130848	104	202	120	142
DCANP1__NM_130848_TGCCTTAAAAACTTGGTACA	DCANP1__NM_130848	84	143	139	172
DCANP1__NM_130848_TTTCGAGTGAAGCATCTGCG	DCANP1__NM_130848	49	72	57	90
DCBLD1__NM_173674_AAAACCCCGGCCAGAAATGT	DCBLD1__NM_173674	335	121	306	172
DCBLD1__NM_173674_AAAGGGATCAGTCGATATGA	DCBLD1__NM_173674	317	332	349	437
DCBLD1__NM_173674_AAGGGATCAGTCGATATGAA	DCBLD1__NM_173674	180	414	217	228
DCBLD1__NM_173674_AGACAATTACAGTACCAAAG	DCBLD1__NM_173674	141	123	178	298
DCBLD1__NM_173674_AGGGGAAAAGACTGATTCTG	DCBLD1__NM_173674	202	309	288	386
DCBLD1__NM_173674_CACTATCCTGATAAGTCACT	DCBLD1__NM_173674	1226	925	1420	1393
DCBLD1__NM_173674_CTAGTGACTTATCAGGATAG	DCBLD1__NM_173674	111	62	162	152
DCBLD1__NM_173674_GATTCTGAGGTTGGGAGATT	DCBLD1__NM_173674	475	368	631	535
DCBLD1__NM_173674_GCTGGTTGTAGAGACGTAGC	DCBLD1__NM_173674	109	153	74	124
DCBLD1__NM_173674_GTAACCGTCCGCTTTGAGAG	DCBLD1__NM_173674	607	637	599	586
DCBLD1__NM_173674_TCAGTGTGCTTCAGCGCAAA	DCBLD1__NM_173674	363	390	438	437
DCBLD1__NM_173674_TGGCCAATGGTGTTCTTCG	DCBLD1__NM_173674	56	110	103	84
DCBLD2__NM_080927_AGGAGTAGTGTCAAACACGT	DCBLD2__NM_080927	1210	1530	1114	781
DCBLD2__NM_080927_ATAGGTCTGTGGGTAGTTTA	DCBLD2__NM_080927	27	141	34	86
DCBLD2__NM_080927_ATATCTGGAACAATTCCTCA	DCBLD2__NM_080927	317	341	292	328
DCBLD2__NM_080927_ATGGGAGATCCGTGTAAAGA	DCBLD2__NM_080927	426	423	429	665
DCBLD2__NM_080927_ATGTAAGGGTTCCACTCTCA	DCBLD2__NM_080927	512	633	634	698
DCBLD2__NM_080927_CCAGACCACAGTATTTGCCT	DCBLD2__NM_080927	1332	1726	1647	1705
DCBLD2__NM_080927_CTAGGCAAATACTGTGGTCT	DCBLD2__NM_080927	824	1005	913	1226
DCBLD2__NM_080927_TAAGTCCTCGCCATTGTGCA	DCBLD2__NM_080927	950	1332	1057	1353
DCBLD2__NM_080927_TCAGCAGAACTGAAATAGGT	DCBLD2__NM_080927	442	803	600	562
DCBLD2__NM_080927_TGCACACCAGCCATGCACAA	DCBLD2__NM_080927	690	980	721	912
DCBLD2__NM_080927_TGCTGTTGGGATAGGTCTGT	DCBLD2__NM_080927	77	104	105	85
DCBLD2__NM_080927_TGGGAGATCCGTGTAAAGAT	DCBLD2__NM_080927	106	78	63	128
DCC__NM_005215_AGAATAGTCTTAGATGTGTT	DCC__NM_005215	1647	1745	2132	2435
DCC__NM_005215_AGATGGCATTCATCTGGCCT	DCC__NM_005215	1733	2361	2048	2653
DCC__NM_005215_AGCGCGCATCTTCAAGTAAC	DCC__NM_005215	1393	1711	1652	1941
DCC__NM_005215_ATTCTGGCTCAATTATTAGT	DCC__NM_005215	712	803	906	781
DCC__NM_005215_CTTCTGATGCCGTCACAATG	DCC__NM_005215	775	823	886	961
DCC__NM_005215_GAAGAAAGATGGCATTCATC	DCC__NM_005215	632	781	834	751
DCC__NM_005215_GAATAGTCTTAGATGTGTTT	DCC__NM_005215	585	727	574	592
DCC__NM_005215_GAGGCATCTTTAGGAGATTC	DCC__NM_005215	439	691	546	522
DCC__NM_005215_GATCACTGGAACTCCTCGGT	DCC__NM_005215	4391	4293	5101	5522
DCC__NM_005215_TCTGATGCCGTCACAATGCG	DCC__NM_005215	439	698	588	650
DCC__NM_005215_TGCTCCGCGGAGTCCGACCG	DCC__NM_005215	600	466	659	625
DCC__NM_005215_TTGAAGATGCGCGCTGAACA	DCC__NM_005215	726	943	822	1029
DCD__NM_053283_ACAGCTATAGTACTGAGTCA	DCD__NM_053283	518	379	399	211
DCD__NM_053283_AGACCCAGGGTTAGCCAGAC	DCD__NM_053283	408	400	499	541
DCD__NM_053283_AGTCGAAGATCTAGAAAGCG	DCD__NM_053283	440	521	505	476
DCD__NM_053283_CAAGGACGTCTTTAACGTCA	DCD__NM_053283	111	163	159	194
DCD__NM_053283_GACGGAGCAAAAAAAGCTGT	DCD__NM_053283	312	319	454	365
DCD__NM_053283_GTCGAAGATCTAGAAAGCGT	DCD__NM_053283	447	252	397	258
DCD__NM_053283_GTGGGGGGACTCGGAAAACT	DCD__NM_053283	214	319	227	239
DCDC1__NM_181807_AAACATAAACATCGGCTTCA	DCDC1__NM_181807	536	768	521	690
DCDC1__NM_181807_ACATAAACATCGGCTTCATG	DCDC1__NM_181807	986	956	1179	1262
DCDC1__NM_181807_ATCTGATGCTGTTTGATGTG	DCDC1__NM_181807	684	822	767	762
DCDC1__NM_181807_GATTATTTGCAGTCTCAGTT	DCDC1__NM_181807	598	1081	713	755
DCDC1__NM_181807_GCCCTGAAGGCACTTTGGAT	DCDC1__NM_181807	675	579	788	507
DCDC1__NM_181807_GCCGATGTTTATGTTTCAAC	DCDC1__NM_181807	924	1016	946	1261
DCDC1__NM_181807_GGGGTATATCTTCAGGTTCG	DCDC1__NM_181807	144	247	171	187
DCDC1__NM_181807_TCCCGTTGAAACATAAACAT	DCDC1__NM_181807	184	312	166	179
DCDC1__NM_181807_TGCCAAGAACACTCGTCTTG	DCDC1__NM_181807	608	537	654	477
DCDC1__NM_181807_TGCTTCAGTCAAGAGGGATA	DCDC1__NM_181807	46	44	58	35
DCDC1__NM_181807_TTCCATTGCTTCAGTCAAGA	DCDC1__NM_181807	26	2	47	50
DCDC1__NM_181807_TTCCTACTGATATCAAAAGA	DCDC1__NM_181807	10	2	9	19
DCDC2__NM_001195610_AAAGGGTGCCTGAACGCCGC	DCDC2__NM_001195610	421	389	392	565
DCDC2__NM_001195610_AACGGGGACCCCTTCTACGC	DCDC2__NM_001195610	1934	974	2004	888
DCDC2__NM_001195610_ACAAGCACGCTCTTCACGAC	DCDC2__NM_001195610	715	360	844	762
DCDC2__NM_001195610_ACGCGGCGCCCCGCGTAGAA	DCDC2__NM_001195610	31	64	23	23
DCDC2__NM_001195610_AGCGGGGGCAATTACGTGGC	DCDC2__NM_001195610	748	979	830	966
DCDC2__NM_001195610_ATGTTCCTGACGGCCCCAAA	DCDC2__NM_001195610	248	365	248	398
DCDC2__NM_001195610_CACAAGCACGCTCTTCACGA	DCDC2__NM_001195610	863	831	1128	1003
DCDC2__NM_001195610_CCAGAGCGGGGGCAATTACG	DCDC2__NM_001195610	616	424	734	798
DCDC2__NM_001195610_GGGGGCAATTACGTGGCTGG	DCDC2__NM_001195610	810	853	754	1017
DCDC2__NM_001195610_GGTCTAGCTTCCGGATTCGG	DCDC2__NM_001195610	736	732	864	649
DCDC2__NM_001195610_TAAAACCAGTAATCCATAGC	DCDC2__NM_001195610	37	26	39	29
DCDC2__NM_001195610_TTGATCCTGCTATGGATTAC	DCDC2__NM_001195610	257	285	288	352
DCDC2B__NM_001099434_ACCTGGGTCCTTCCCTCTAT	DCDC2B__NM_001099434	1090	509	1181	727
DCDC2B__NM_001099434_AGAGGGCAGTATGTGGCCGC	DCDC2B__NM_001099434	909	797	862	753
DCDC2B__NM_001099434_CACTTGTGTGATGGGGGCAT	DCDC2B__NM_001099434	1105	919	1149	809
DCDC2B__NM_001099434_CCTGGGTCCTTCCCTCTATG	DCDC2B__NM_001099434	1413	912	1606	960
DCDC2B__NM_001099434_GAAGAACAGAGGGCAGTATG	DCDC2B__NM_001099434	164	184	270	174
DCDC2B__NM_001099434_GCCATGACAAGGTGTGTAGA	DCDC2B__NM_001099434	47	15	67	37
DCDC2B__NM_001099434_GGAAGAATGGGTCCCCATTC	DCDC2B__NM_001099434	96	66	138	49
DCDC2B__NM_001099434_GGGTAGTGGTGTACCGGAAT	DCDC2B__NM_001099434	357	201	332	190
DCDC2B__NM_001099434_GGTAGTGGTGTACCGGAATG	DCDC2B__NM_001099434	269	90	238	128
DCDC2B__NM_001099434_TCAGCTATTTACCCCATAGA	DCDC2B__NM_001099434	223	105	228	134
DCDC2B__NM_001099434_TGACTCGCCACTTGTGTGAT	DCDC2B__NM_001099434	662	730	850	1061
DCDC2B__NM_001099434_TTCAGCTATTTACCCCATAG	DCDC2B__NM_001099434	26	93	31	54
DCDC2C__NM_001287444_AACGGGGACCCGTTCTACGT	DCDC2C__NM_001287444	109	106	133	91
DCDC2C__NM_001287444_AACTTCTTGCCCACGTAGAA	DCDC2C__NM_001287444	152	140	179	267
DCDC2C__NM_001287444_AAGAGGCGGCGCACGCCGAA	DCDC2C__NM_001287444	28	27	46	47
DCDC2C__NM_001287444_ACTTCTTGCCCACGTAGAAC	DCDC2C__NM_001287444	227	263	309	435
DCDC2C__NM_001287444_AGAGGCGGCGCACGCCGAAC	DCDC2C__NM_001287444	26	35	27	43
DCDC2C__NM_001287444_CAAATTTAGTCTGTCCGATT	DCDC2C__NM_001287444	766	814	746	1082
DCDC2C__NM_001287444_CAACGGGGACCCGTTCTACG	DCDC2C__NM_001287444	328	336	430	316
DCDC2C__NM_001287444_CAATCGGACAGACTAAATTT	DCDC2C__NM_001287444	788	809	861	752
DCDC2C__NM_001287444_CCAAATTTAGTCTGTCCGAT	DCDC2C__NM_001287444	1434	2110	1507	2084
DCDC2C__NM_001287444_GAGCAGGTGGACGTCCCGTT	DCDC2C__NM_001287444	980	912	1057	915
DCDC2C__NM_001287444_GCAAGAAGTTCGTGCTGTCG	DCDC2C__NM_001287444	2057	2251	2115	3185
DCDC2C__NM_001287444_GCCTCTTCACGCCCACGCGT	DCDC2C__NM_001287444	176	144	211	200
DCDC5__NM_020869_ATCTGGACCAAGGATAGTAA	DCDC5__NM_020869	226	281	224	208
DCDC5__NM_020869_CAAAACTCTGAGTCGCACAG	DCDC5__NM_020869	375	665	551	581
DCDC5__NM_020869_CAGGTGAGATATCTGGACCA	DCDC5__NM_020869	459	760	588	690
DCDC5__NM_020869_CTGAGTCGCACAGGGGCATA	DCDC5__NM_020869	120	115	101	87
DCDC5__NM_020869_CTTACAGATGGGTTTCTTCA	DCDC5__NM_020869	472	730	536	855
DCDC5__NM_020869_CTTGGTCCAGATATCTCACC	DCDC5__NM_020869	642	535	749	519
DCDC5__NM_020869_GCAAAACTCTGAGTCGCACA	DCDC5__NM_020869	1003	1205	1140	1345
DCDC5__NM_020869_GGAACAAGCAGTCCTTGTAT	DCDC5__NM_020869	301	272	412	490
DCDC5__NM_020869_GGCTCTGTTGTCTTACAGAT	DCDC5__NM_020869	479	681	604	635
DCDC5__NM_020869_TCCCAGTGGCCAACTTAATG	DCDC5__NM_020869	94	71	178	164
DCDC5__NM_020869_TTACAGATGGGTTTCTTCAT	DCDC5__NM_020869	101	60	113	64
DCDC5__NM_020869_TTCCGTCCAGGTGAGATATC	DCDC5__NM_020869	16	0	23	43
DCHS1__NM_003737_CACCAGATAGCGCATAGCCC	DCHS1__NM_003737	24	5	26	18
DCHS1__NM_003737_CACTGTGTTCGTCAATGGCC	DCHS1__NM_003737	967	1180	1171	1689
DCHS1__NM_003737_CGTAGAAGTTACAGTGCGAG	DCHS1__NM_003737	700	931	789	896
DCHS1__NM_003737_CGTCCGTACAGCCCGTGTCT	DCHS1__NM_003737	956	1161	1040	1228
DCHS1__NM_003737_CTGATTGGCGACATCAGTGC	DCHS1__NM_003737	431	435	478	446
DCHS1__NM_003737_CTTCTCCATCGACGCACACA	DCHS1__NM_003737	341	455	284	425
DCHS1__NM_003737_GCGGCTCTGATTGAAAGCCG	DCHS1__NM_003737	107	126	160	116
DCHS1__NM_003737_GCTGTAGCATATAGTGTGAG	DCHS1__NM_003737	510	775	578	812
DCHS1__NM_003737_TCAGAGCCGCTACCATGCTG	DCHS1__NM_003737	562	429	567	631
DCHS1__NM_003737_TCGCCAATCAGTGTACCCGC	DCHS1__NM_003737	709	500	725	755
DCHS1__NM_003737_TGACTTACGAGATCAACCGG	DCHS1__NM_003737	319	247	329	265
DCHS1__NM_003737_TGATTGGCGACATCAGTGCG	DCHS1__NM_003737	705	905	953	1318
DCHS2__NM_001142552_ATGTCACCTACCAGCGTGTC	DCHS2__NM_001142552	508	596	492	563
DCHS2__NM_001142552_CCTCACCCTTTCCGTAGATG	DCHS2__NM_001142552	3478	3780	3723	4221
DCHS2__NM_001142552_CGCCCATCTTCCGCCCACAA	DCHS2__NM_001142552	320	294	314	475
DCHS2__NM_001142552_CTCACCCTTTCCGTAGATGA	DCHS2__NM_001142552	596	747	848	860
DCHS2__NM_001142552_CTCGGAGACGTCGAGTTGCA	DCHS2__NM_001142552	685	687	874	1074
DCHS2__NM_001142552_CTTCCCCCGGACACGCTGGT	DCHS2__NM_001142552	1481	1890	1776	2071
DCHS2__NM_001142552_GCTGCTGCCTGACCGCCCAT	DCHS2__NM_001142552	931	775	1212	851
DCHS2__NM_001142552_GGAGGAGAGATACACCCCAT	DCHS2__NM_001142552	506	562	516	435
DCHS2__NM_001142552_GTGGGCGGAAGATGGGCGAA	DCHS2__NM_001142552	351	281	380	348
DCHS2__NM_001142552_TCACCCTTTCCGTAGATGAG	DCHS2__NM_001142552	558	537	611	613
DCHS2__NM_001142552_TGGTAGGTGACATCCGCGCC	DCHS2__NM_001142552	38	54	55	37
DCHS2__NM_001142552_TGTGGGCGGAAGATGGGCGA	DCHS2__NM_001142552	290	332	337	281
DCHS2__NM_017639_AGCCTTTGTTCGTGTGGACC	DCHS2__NM_017639	488	629	549	868
DCHS2__NM_017639_CACATACACCTCATATACAC	DCHS2__NM_017639	464	377	539	599
DCHS2__NM_017639_CTCAAGATATCGACAGGGAA	DCHS2__NM_017639	172	127	212	361
DCHS2__NM_017639_CTCCAGGTCCACACGAACAA	DCHS2__NM_017639	191	176	245	346
DCHS2__NM_017639_CTTGGGCACTTAGCCCACCC	DCHS2__NM_017639	368	525	451	405
DCHS2__NM_017639_GAGAATGACACATTTAGCCC	DCHS2__NM_017639	259	268	194	143
DCHS2__NM_017639_GCCCATCATGAGGGTCGATC	DCHS2__NM_017639	841	780	1086	1322
DCHS2__NM_017639_GTGTTTCTCAAGATATCGAC	DCHS2__NM_017639	12	18	16	26
DCHS2__NM_017639_TCCTGGTGGAAGCTAAGGAT	DCHS2__NM_017639	323	292	317	418
DCHS2__NM_017639_TGATCTCCTGGTGGAAGCTA	DCHS2__NM_017639	32	18	13	27
DCHS2__NM_017639_TGCCCAAGCCTTTGTTCGTG	DCHS2__NM_017639	1113	1588	1232	1709
DCHS2__NM_017639_TGCTCGTCACATAGGTTGAT	DCHS2__NM_017639	443	624	566	604
DCK__NM_000788_ACTTTGAACATTGCACCATC	DCK__NM_000788	656	790	840	887
DCK__NM_000788_AGAGGTGCCTATCTTAACAC	DCK__NM_000788	357	420	379	435
DCK__NM_000788_AGCTCTTATTCGACTGAGAC	DCK__NM_000788	15	8	17	2
DCK__NM_000788_ATTTGGCCAAAGCCTTGAAT	DCK__NM_000788	123	76	88	93
DCK__NM_000788_CAACTTCGATTATCTTCAAG	DCK__NM_000788	1941	2220	2041	2536
DCK__NM_000788_CAGAGCTGGCTGAGAAAGAC	DCK__NM_000788	590	848	661	717
DCK__NM_000788_CTTACTTCAGTGTCCTATGC	DCK__NM_000788	887	1054	1150	1657
DCK__NM_000788_TGTATGAGAAACCTGAACGA	DCK__NM_000788	182	56	195	257
DCK__NM_000788_TTAACATCCAGTGTTAAGAT	DCK__NM_000788	111	95	124	126
DCLK1__NM_001195415_ACCTTCTATGAGTTCTAACA	DCLK1__NM_001195415	376	477	352	292
DCLK1__NM_001195415_ATGCAGGTATTTGATGGCGC	DCLK1__NM_001195415	835	1202	954	899
DCLK1__NM_001195415_ATGGATGAGAACGATGGCCC	DCLK1__NM_001195415	314	321	296	286
DCLK1__NM_001195415_CAGGGGGCCGTCTACAATGG	DCLK1__NM_001195415	1230	1483	1665	1886
DCLK1__NM_001195415_CTGTGGCACCCCAACATACG	DCLK1__NM_001195415	416	488	510	330
DCLK1__NM_001195415_GTCGGAAGAACAATAGGAGA	DCLK1__NM_001195415	649	813	598	629
DCLK1__NM_001195415_GTGCCACAGACTGTGTACAG	DCLK1__NM_001195415	1080	1141	1253	1212
DCLK1__NM_001195415_TATAAAGTCGGAAGAACAAT	DCLK1__NM_001195415	434	424	442	377
DCLK1__NM_001195415_TATCGTTCTGTTATTGTAGC	DCLK1__NM_001195415	132	82	125	184
DCLK1__NM_001195415_TCAATCAGAAGAACGATATT	DCLK1__NM_001195415	658	488	690	528
DCLK1__NM_001195415_TCTCTCGGTGTATTTGTTAG	DCLK1__NM_001195415	786	1029	996	1159
DCLK1__NM_001195415_TCTTGTCATGGAATTAGTAA	DCLK1__NM_001195415	438	402	380	677
DCLK1__NM_001195430_AATCCAAGTCATCCGAAGAG	DCLK1__NM_001195430	363	489	423	356
DCLK1__NM_004734_AAAGTTCGTTTCTATCGAAA	DCLK1__NM_004734	85	120	89	138
DCLK1__NM_004734_AGCCGAGGGTCGCGGGTGAA	DCLK1__NM_004734	710	915	927	1153
DCLK1__NM_004734_AGCTGGAGCACTTCGACGAG	DCLK1__NM_004734	1064	1644	1259	1437
DCLK1__NM_004734_GATAACGTGAATTTGCCCCA	DCLK1__NM_004734	995	779	1218	818
DCLK1__NM_004734_GCACTTCGACGAGCGGGATA	DCLK1__NM_004734	501	623	584	751
DCLK1__NM_004734_GGGCCTCAAAAGATCGGAAC	DCLK1__NM_004734	200	121	183	303
DCLK1__NM_004734_GTGTACTCCAGTTCTTGAA	DCLK1__NM_004734	427	419	443	477
DCLK1__NM_004734_TCAAAAGATCGGAACCGGTC	DCLK1__NM_004734	935	1067	1299	1654
DCLK1__NM_004734_TCACGTTATCCGACAGAGTT	DCLK1__NM_004734	958	1275	1002	1383
DCLK1__NM_004734_TGGCATCGGTGATATCGGTG	DCLK1__NM_004734	197	325	158	152
DCLK1__NM_004734_TTCAGAGGTGCGAGAGAATA	DCLK1__NM_004734	43	35	52	131
DCLK1__NM_004734_TTCTCGGGCAGTGTCTTCAC	DCLK1__NM_004734	8	0	7	2
DCLK2__NM_001040260_AGGCGCGCTTCTACCGGAAC	DCLK2__NM_001040260	468	650	467	545
DCLK2__NM_001040260_CAGCACCAGGAGTATCGAGC	DCLK2__NM_001040260	529	393	513	745
DCLK2__NM_001040260_CAGGTTCACGTTCTCCGACA	DCLK2__NM_001040260	755	971	887	1053
DCLK2__NM_001040260_GACTTTACGAAATGGTTCAT	DCLK2__NM_001040260	405	262	440	498
DCLK2__NM_001040260_GGCGCGCTTCTACCGGAACG	DCLK2__NM_001040260	106	97	118	181
DCLK2__NM_001040260_GGTCTGTGAACATCAAGGGT	DCLK2__NM_001040260	688	769	675	744
DCLK2__NM_001040260_GTGTAATCGACTTTACGAAA	DCLK2__NM_001040260	80	84	46	64
DCLK2__NM_001040260_TCGAATCACAGTCACTAACT	DCLK2__NM_001040260	1759	1995	1739	2080
DCLK2__NM_001040260_TCTACACCATCGACGGCAGC	DCLK2__NM_001040260	440	428	556	588
DCLK2__NM_001040260_TGAAGCCTAGAAAAGCCGTG	DCLK2__NM_001040260	293	586	452	430
DCLK2__NM_001040260_TGAAGTAGCGGTCCCCGTTC	DCLK2__NM_001040260	1000	1219	1089	825
DCLK2__NM_001040260_TGGTCTGTGAACATCAAGGG	DCLK2__NM_001040260	45	49	41	36
DCLK3__NM_033403_AGCTTCTCCACATCATACAT	DCLK3__NM_033403	477	475	431	471
DCLK3__NM_033403_CACCCTTCCTCCCCACTTGC	DCLK3__NM_033403	3514	3870	4432	5350
DCLK3__NM_033403_CTCTGGAAGAGAGGCACGCA	DCLK3__NM_033403	194	103	100	117
DCLK3__NM_033403_GAAGGGCCAATGTATGATG	DCLK3__NM_033403	621	574	593	710
DCLK3__NM_033403_GGGCATCATTGCCGCCAATG	DCLK3__NM_033403	110	342	98	159
DCLK3__NM_033403_GGGGGCTTGCTACTGGGTTC	DCLK3__NM_033403	464	580	616	956
DCLK3__NM_033403_GGTCGGAAGCCACGGCCCAT	DCLK3__NM_033403	609	469	746	638
DCLK3__NM_033403_GGTGGAGATTGAAAAGACCT	DCLK3__NM_033403	127	43	165	134
DCLK3__NM_033403_GTGGAAAAGCATTATGAGAC	DCLK3__NM_033403	96	71	115	162
DCLK3__NM_033403_TGGGAACTTTGCTGTCGTGA	DCLK3__NM_033403	223	209	213	280
DCLK3__NM_033403_TGTCAGGGCCCGGGCTTTGT	DCLK3__NM_033403	996	777	1065	727
DCLK3__NM_033403_TTTCACTAGATGACAGAGCG	DCLK3__NM_033403	93	80	102	127
DCLRE1A__NM_001271816_AAGTGAACAGCTTCTTAACT	DCLRE1A__NM_001271816	450	484	578	669
DCLRE1A__NM_001271816_ACGAAGACCATCTTTCCTCA	DCLRE1A__NM_001271816	1867	1856	2170	1878
DCLRE1A__NM_001271816_ACGACATGGTCCCTTACTGA	DCLRE1A__NM_001271816	1856	1726	2190	1818
DCLRE1A__NM_001271816_AGGTGTCTGCCCTATCAATG	DCLRE1A__NM_001271816	292	245	305	374
DCLRE1A__NM_001271816_AGTGAACAGCTTCTTAACTC	DCLRE1A__NM_001271816	470	435	488	424
DCLRE1A__NM_001271816_AGTTTCAGTGAGACTAAGTC	DCLRE1A__NM_001271816	698	670	727	848
DCLRE1A__NM_001271816_CTCATCCTGGTCCTTCAGTA	DCLRE1A__NM_001271816	756	899	1012	1215
DCLRE1A__NM_001271816_GAAAAAGGCATCTGGCAATT	DCLRE1A__NM_001271816	280	242	446	449
DCLRE1A__NM_001271816_GTATCTTTGGGGACACGTGT	DCLRE1A__NM_001271816	476	452	517	497
DCLRE1A__NM_001271816_GTTGAGAATGACAAACTAGT	DCLRE1A__NM_001271816	465	536	578	529
DCLRE1A__NM_001271816_TGATAGGGCAGACACCTCGA	DCLRE1A__NM_001271816	448	630	495	485
DCLRE1A__NM_001271816_TGTTGAGAATGACAAACTAG	DCLRE1A__NM_001271816	139	149	83	62
DCLRE1B__NM_022836_AGTCCACTGCGATGGGCGTA	DCLRE1B__NM_022836	173	66	150	80
DCLRE1B__NM_022836_ATCGAGGAGGGTTACGGTCA	DCLRE1B__NM_022836	841	291	820	340
DCLRE1B__NM_022836_CAAGAGGTGGGCTGTAATTG	DCLRE1B__NM_022836	674	102	653	216
DCLRE1B__NM_022836_CCACTGCGATGGGCGTATGG	DCLRE1B__NM_022836	964	172	920	336
DCLRE1B__NM_022836_CCCCCATACGCCCATCGCAG	DCLRE1B__NM_022836	1473	467	1449	619
DCLRE1B__NM_022836_CCTCTTGCATCGTCACCTAC	DCLRE1B__NM_022836	863	1083	1169	1362
DCLRE1B__NM_022836_CTTCTTGTCTCACATGCACT	DCLRE1B__NM_022836	647	356	756	540
DCLRE1B__NM_022836_GCAAGAGGTGGGCTGTAATT	DCLRE1B__NM_022836	454	107	494	158
DCLRE1B__NM_022836_GGGGATCAGGACCCCATTCA	DCLRE1B__NM_022836	255	163	289	227
DCLRE1B__NM_022836_GTATTACCCCTAGATGAAAT	DCLRE1B__NM_022836	89	62	92	80
DCLRE1B__NM_022836_TCCACTGCGATGGGCGTATG	DCLRE1B__NM_022836	441	95	476	286
DCLRE1B__NM_022836_TCTCTTGTCCAATTTCATCT	DCLRE1B__NM_022836	611	508	707	565
DCLRE1C__NM_001033855_AACAACAACTCCTTAGTCAC	DCLRE1C__NM_001033855	541	586	833	681
DCLRE1C__NM_001033855_AAGCGGTCTATGGAGATAGT	DCLRE1C__NM_001033855	309	253	315	234
DCLRE1C__NM_001033855_CTCCATAGACCGCTTCGATA	DCLRE1C__NM_001033855	321	151	318	393
DCLRE1C__NM_001033855_CTCCCTATCGAAGCGGTCTA	DCLRE1C__NM_001033855	667	633	713	1022
DCLRE1C__NM_001033855_GCGCTATGAGTTCTTTCGAG	DCLRE1C__NM_001033855	949	888	1109	1093
DCLRE1C__NM_001033855_GGCGCTATGAGTTCTTTCGA	DCLRE1C__NM_001033855	916	773	1325	1061
DCLRE1C__NM_001033855_TATGGAGATAGTTGGATACT	DCLRE1C__NM_001033855	322	324	408	345
DCLRE1C__NM_001033855_TCAGGTTCTCCCTATCGAAG	DCLRE1C__NM_001033855	69	52	61	98
DCLRE1C__NM_001033855_TCCACTAAAGATATCTGGGT	DCLRE1C__NM_001033855	679	398	661	455
DCLRE1C__NM_001033855_TCCTACCCAGATATCTTTAG	DCLRE1C__NM_001033855	305	203	335	239
DCLRE1C__NM_001033855_TCTCCATAGACCGCTTCGAT	DCLRE1C__NM_001033855	748	610	944	866
DCLRE1C__NM_001033855_TTCATCCACTAAAGATATCT	DCLRE1C__NM_001033855	62	22	137	52
DCLRE1C__NM_001033857_AATGGAACTGTCCTGTACAC	DCLRE1C__NM_001033857	349	268	568	499
DCLRE1C__NM_001033857_ACATTTGGATATGCGTTCAC	DCLRE1C__NM_001033857	548	406	704	706
DCLRE1C__NM_001033857_AGACATCCAAAGTGTATATT	DCLRE1C__NM_001033857	249	270	220	224
DCLRE1C__NM_001033857_ATCTGAAGTCTCCTGTGTAC	DCLRE1C__NM_001033857	927	835	1093	904
DCLRE1C__NM_001033857_ATTTCTGGAAGTAATTCCAC	DCLRE1C__NM_001033857	142	109	152	152
DCLRE1C__NM_001033857_CCTTTCTCCAAACCACATGG	DCLRE1C__NM_001033857	247	181	358	230
DCLRE1C__NM_001033857_CTGAACTGCAAAGCGGCTTA	DCLRE1C__NM_001033857	478	472	703	621
DCLRE1C__NM_001033857_CTTGGAATTTGGTAAAATCT	DCLRE1C__NM_001033857	175	352	194	313
DCLRE1C__NM_001033857_GGAGACTTCAGATTGGCGCA	DCLRE1C__NM_001033857	866	1083	1024	1329
DCLRE1C__NM_001033857_GTGCCAACTGGAATGACATT	DCLRE1C__NM_001033857	919	1057	1011	954
DCLRE1C__NM_001033857_TGCAGTTCAGCCACACAACA	DCLRE1C__NM_001033857	204	148	174	197
DCLRE1C__NM_001033857_TTTAAGTGGAGTCTTAGAGC	DCLRE1C__NM_001033857	16	42	19	25
DCLRE1C__NM_022487_AAAGTACTGGGATTGCAGGC	DCLRE1C__NM_022487	62	0	30	0
DCLRE1C__NM_022487_AAATGTTCAAATAACCTGGT	DCLRE1C__NM_022487	313	389	314	460
DCLRE1C__NM_022487_AACAACCACTTTCGATCATA	DCLRE1C__NM_022487	1435	2230	1422	1983
DCLRE1C__NM_022487_AATGCTCAAGAATTAAATGG	DCLRE1C__NM_022487	278	126	283	242
DCLRE1C__NM_022487_ACTAATGCACAATTACAGCT	DCLRE1C__NM_022487	354	413	343	315
DCLRE1C__NM_022487_ACTGAAAATCATAAGCCGAC	DCLRE1C__NM_022487	595	870	785	850
DCLRE1C__NM_022487_CAAGTGATTGATAGGTTAAA	DCLRE1C__NM_022487	223	302	235	266
DCLRE1C__NM_022487_CAGGATCTACATTAAGAGGA	DCLRE1C__NM_022487	392	330	453	507
DCLRE1C__NM_022487_CAGGCAGGAGTTACTGTGCC	DCLRE1C__NM_022487	819	675	728	649
DCLRE1C__NM_022487_CCATAGTATGTTTACGTACC	DCLRE1C__NM_022487	510	577	673	719
DCLRE1C__NM_022487_CCTAAGCCTTCCAAGTAGCG	DCLRE1C__NM_022487	118	154	206	137
DCLRE1C__NM_022487_CCTTTGTCTCATTTGTACAT	DCLRE1C__NM_022487	931	979	965	1174
DCLRE1C__NM_022487_CTAATTGTCCGCTTCTAATT	DCLRE1C__NM_022487	463	550	507	723
DCLRE1C__NM_022487_GAGATACATTCTGCTAGTTT	DCLRE1C__NM_022487	664	475	774	770
DCLRE1C__NM_022487_GAGCATAGCAGTTAAGATCA	DCLRE1C__NM_022487	38	14	52	35
DCLRE1C__NM_022487_GTCCAGCCTGGGCAACACAC	DCLRE1C__NM_022487	228	20	169	18
DCLRE1C__NM_022487_GTGCCACTGCACCTCTAGTC	DCLRE1C__NM_022487	152	146	161	194
DCLRE1C__NM_022487_GTGCTTCTAATGAACCTGTC	DCLRE1C__NM_022487	20	10	32	73
DCLRE1C__NM_022487_GTGTACCTTATGATCGAAAG	DCLRE1C__NM_022487	86	158	98	142
DCLRE1C__NM_022487_TCTTCAGGAAACCAATATGA	DCLRE1C__NM_022487	288	332	294	272
DCLRE1C__NM_022487_TCTTGTTACCCTGGTTAGTC	DCLRE1C__NM_022487	251	239	280	285
DCLRE1C__NM_022487_TGGGCTCAAGTGATCCTCAA	DCLRE1C__NM_022487	97	81	108	124
DCLRE1C__NM_022487_TGTAGTCCTCGCTACTTGGA	DCLRE1C__NM_022487	73	0	97	0
DCLRE1C__NM_022487_TGTTAGGTGCATATACGTTT	DCLRE1C__NM_022487	183	6	225	19
DCLRE1C__NM_022487_TTGGATCCACCAGACTAACC	DCLRE1C__NM_022487	89	38	158	266
DCLRE1C__NM_022487_TTTCACCATATTGGTCAGAC	DCLRE1C__NM_022487	27	46	61	70
DCN__NM_001920_AAAATTAGCAAAGTTAGTCC	DCN__NM_001920	135	187	190	134
DCN__NM_001920_AGATGGCATTGACAGCGGAA	DCN__NM_001920	117	163	195	117
DCN__NM_001920_AGATGTAATTCCGTAAGGGA	DCN__NM_001920	243	124	246	155
DCN__NM_001920_AGGTCTAGCAGAGTTGTGTC	DCN__NM_001920	268	326	286	226
DCN__NM_001920_ATCAAGATGTAATTCCGTAA	DCN__NM_001920	235	288	358	301
DCN__NM_001920_CTGTCAATGCCATCTTCGAG	DCN__NM_001920	1202	1259	1281	1446
DCN__NM_001920_GCTGGACCGTTTCAACAGAG	DCN__NM_001920	403	384	396	388
DCN__NM_001920_GGCTCGAAGTCGCGGTCATC	DCN__NM_001920	529	791	696	844
DCN__NM_001920_GGTATCAGCAATGCGGATGT	DCN__NM_001920	410	366	266	258
DCN__NM_001920_TCCTGAGCTCTTCAGCGGAT	DCN__NM_001920	165	256	241	384
DCN__NM_001920_TGACTTTATGCTAGAAGATG	DCN__NM_001920	95	134	116	171
DCN__NM_001920_TGTAATTCCGTAAGGGAAGG	DCN__NM_001920	38	74	23	14
DCN__NM_133504_CATCAAGATGTAATTCCGTA	DCN__NM_133504	391	454	505	509
DCN__NM_133504_CTTACGGAATTACATCTTGA	DCN__NM_133504	364	448	520	576
DCN__NM_133505_CCTGAAAGGACTGAATAATT	DCN__NM_133505	619	694	706	620
DCN__NM_133505_TCATCTGAGGGAGCTTCACT	DCN__NM_133505	777	700	874	658
DCN__NM_133506_ACCTGGACACAACACCAAAA	DCN__NM_133506	199	194	186	131
DCN__NM_133506_CAGAGATATTGTTGTTATGA	DCN__NM_133506	360	317	373	511
DCN__NM_133506_GCGCACGTAGACACATCTGA	DCN__NM_133506	1507	1633	1805	1923
DCN__NM_133506_GTGCGCTCTGCCATTCAACT	DCN__NM_133506	476	580	473	466
DCN__NM_133506_TCAGCAACCCGGTCCAGTAC	DCN__NM_133506	903	608	963	854
DCP1A__NM_001290204_AATGGTAAAACCATGGTAAG	DCP1A__NM_001290204	146	71	171	85
DCP1A__NM_001290204_AGAAGGTGTACAGAGCGACC	DCP1A__NM_001290204	87	41	29	15
DCP1A__NM_001290204_AGGAGAAGACTGATATAGAA	DCP1A__NM_001290204	141	70	248	115
DCP1A__NM_001290204_CAATGGTAAAACCATGGTAA	DCP1A__NM_001290204	436	196	502	355
DCP1A__NM_001290204_CATATTTAGTCGATTCACAA	DCP1A__NM_001290204	430	458	465	578
DCP1A__NM_001290204_GATTCACAATGGTAAAACCA	DCP1A__NM_001290204	176	146	252	234
DCP1A__NM_001290204_GGACCTTATTCGTATATCGA	DCP1A__NM_001290204	375	634	441	546
DCP1A__NM_001290204_TCTGCGATGCTGGTGATATA	DCP1A__NM_001290204	323	112	387	273
DCP1A__NM_001290204_TGAACCAGTGAATAAAGATT	DCP1A__NM_001290204	336	285	331	402
DCP1A__NM_001290204_TGGAGGCGCTGAGTCGAGCT	DCP1A__NM_001290204	231	420	281	270
DCP1A__NM_001290204_TGGGCAGGAGATGAGCCTAG	DCP1A__NM_001290204	101	34	152	92
DCP1A__NM_001290204_TTACCTTCGATATACGAATA	DCP1A__NM_001290204	7	0	41	98
DCP1A__NM_001290205_AAGTGTCGATATATAGTATC	DCP1A__NM_001290205	216	327	276	321
DCP1A__NM_001290205_AGCATCTCCAGGATGTCGAT	DCP1A__NM_001290205	411	343	574	481
DCP1A__NM_001290205_AGCCAAGGATGAGTATGAGA	DCP1A__NM_001290205	74	13	52	57
DCP1A__NM_001290205_GAGCCAAGGATGAGTATGAG	DCP1A__NM_001290205	141	236	215	242
DCP1A__NM_001290205_GAGGCTGGAGTGATTAGCAC	DCP1A__NM_001290205	212	63	239	105
DCP1A__NM_001290205_GAGTGCTTGGCTGTAACCCA	DCP1A__NM_001290205	1208	1029	1248	1186
DCP1A__NM_001290205_GCTCCTCCTAACTGCTCAAA	DCP1A__NM_001290205	614	592	732	715
DCP1A__NM_001290205_GGAAATGGTAGGAATGAATT	DCP1A__NM_001290205	375	154	452	406
DCP1A__NM_001290205_GGCTCCTCCTAACTGCTCAA	DCP1A__NM_001290205	283	249	348	384
DCP1A__NM_001290205_GGCTGGAGTGATTAGCACCG	DCP1A__NM_001290205	890	660	898	584
DCP1A__NM_001290205_GGGAAATGGTAGGAATGAAT	DCP1A__NM_001290205	132	99	148	174
DCP1A__NM_001290205_GGGCAGCAGAAGGGACACCC	DCP1A__NM_001290205	303	353	347	207
DCP1A__NM_001290206_AGGGCTCAACGGGATTGTGT	DCP1A__NM_001290206	452	240	528	329
DCP1A__NM_001290207_GGACTGAGAACAGGGCTCAA	DCP1A__NM_001290207	145	164	179	163
DCP1B__NM_152640_AGTGATCCTCAATTCAGGAG	DCP1B__NM_152640	112	307	198	106
DCP1B__NM_152640_ATGGATTCACCATTATGAAT	DCP1B__NM_152640	84	92	99	83
DCP1B__NM_152640_CAGTGTCCATCTATGGAATT	DCP1B__NM_152640	813	1128	769	972
DCP1B__NM_152640_CATGCTCAGCCTATTCATAA	DCP1B__NM_152640	303	519	421	366
DCP1B__NM_152640_CGGCCATCGGGCCAACGAGT	DCP1B__NM_152640	74	46	85	70
DCP1B__NM_152640_GCACCCACTCGTTGGCCCGA	DCP1B__NM_152640	890	1021	952	1093
DCP1B__NM_152640_GCGTATACCTCAGCCCAACC	DCP1B__NM_152640	91	58	64	30
DCP1B__NM_152640_GCTGAGGTATACGCTGTTGC	DCP1B__NM_152640	636	724	664	826
DCP1B__NM_152640_GTCCACGATGCGGTTGATAT	DCP1B__NM_152640	1524	1270	1401	1270
DCP1B__NM_152640_TCAACTGTTCATACTGAGTT	DCP1B__NM_152640	863	841	822	1273
DCP1B__NM_152640_TCCACGATGCGGTTGATATA	DCP1B__NM_152640	393	359	387	352
DCP1B__NM_152640_TGCGGTTGATATAGGGGTCG	DCP1B__NM_152640	269	278	334	277
DCP2__NM_001242377_AGAATAATTGCACCATATGT	DCP2__NM_001242377	568	162	622	179
DCP2__NM_001242377_CCTCATCATGGAGACCAAAC	DCP2__NM_001242377	89	45	124	158
DCP2__NM_001242377_GAAATCCAAGTAAAACCAAT	DCP2__NM_001242377	134	29	146	87
DCP2__NM_001242377_GCCGGGAATCTCCACCCGTT	DCP2__NM_001242377	1777	1911	1985	1870
DCP2__NM_001242377_GTCATATCATTTCTATGACA	DCP2__NM_001242377	323	568	330	437
DCP2__NM_001242377_GTTAGGTGCCAAACCAAGTT	DCP2__NM_001242377	116	132	132	115
DCP2__NM_001242377_TGATGTACAAACGAGCAAGC	DCP2__NM_001242377	23	15	52	46
DCP2__NM_001242377_TGCATTGTCTCTTTCCTCGC	DCP2__NM_001242377	703	705	769	1007
DCP2__NM_001242377_TTATGGCCATTCCCTTTATC	DCP2__NM_001242377	331	114	396	161
DCP2__NM_001242377_TTCAGATTGAACTTGCCCAT	DCP2__NM_001242377	51	30	67	34
DCP2__NM_001242377_TTCCTCAACTGGTAGCACGC	DCP2__NM_001242377	41	115	41	21
DCP2__NM_001242377_TTGTAGGTACTACTAGTTCA	DCP2__NM_001242377	419	489	492	477
DCPS__NM_014026_ATCCCTACGCGACCTTACTC	DCPS__NM_014026	541	0	607	43
DCPS__NM_014026_ATGAGGACAAAACCATCAGA	DCPS__NM_014026	300	55	374	22
DCPS__NM_014026_CAGCTCCTCAACTAGGCAAG	DCPS__NM_014026	224	127	297	116
DCPS__NM_014026_CATCTCCCGTCTCTCGGATC	DCPS__NM_014026	2679	201	3036	252
DCPS__NM_014026_CCGGAGAACGGTAAGCGGAC	DCPS__NM_014026	571	527	549	542
DCPS__NM_014026_CGCTTCCTCTTGCCTAGTTG	DCPS__NM_014026	603	423	739	569
DCPS__NM_014026_CTCCGCCTGATCCGAGAGAC	DCPS__NM_014026	118	85	78	87
DCPS__NM_014026_CTCCGGAGTAAGGTCGCGTA	DCPS__NM_014026	295	50	409	81
DCPS__NM_014026_GAGGAAGCGCGAATTGGACG	DCPS__NM_014026	700	541	552	354
DCPS__NM_014026_GATGCCCCGGCGATGGCAGA	DCPS__NM_014026	275	7	435	35
DCPS__NM_014026_TCCTCATCCCTGACCTCAAG	DCPS__NM_014026	145	126	226	173
DCPS__NM_014026_TCTTGGAGGGAACAAGTGAT	DCPS__NM_014026	176	12	163	19
DCST1__NM_001143687_CATCTCCACAGCCCCCTTAC	DCST1__NM_001143687	1343	943	1462	932
DCST1__NM_001143687_CCAGCATCTTGGGTACTAGT	DCST1__NM_001143687	922	967	832	856
DCST1__NM_001143687_CTTGAACATGGCCCGTAAGG	DCST1__NM_001143687	11	2	6	10
DCST1__NM_001143687_GCATCTCCACAGCCCCCTTA	DCST1__NM_001143687	871	888	1118	1034
DCST1__NM_001143687_GCATCTTGGGTACTAGTAGG	DCST1__NM_001143687	253	266	394	385
DCST1__NM_001143687_GCCCAGGTGAATAGTGAGAC	DCST1__NM_001143687	669	676	602	655
DCST1__NM_001143687_GCCCGTCTCACTATTCACCT	DCST1__NM_001143687	2165	1095	2103	1253
DCST1__NM_001143687_GCGCCACAGGAACCAGCTAC	DCST1__NM_001143687	663	548	820	742
DCST1__NM_001143687_GTCCTTGAACATGGCCCGTA	DCST1__NM_001143687	438	400	440	274
DCST1__NM_001143687_GTTGAGATTGTGTCGCAGAT	DCST1__NM_001143687	423	424	425	445
DCST1__NM_001143687_TCCTTGAACATGGCCCGTAA	DCST1__NM_001143687	280	290	255	201
DCST1__NM_001143687_TGCCCAGGTGAATAGTGAGA	DCST1__NM_001143687	335	321	424	282
DCST1__NM_152494_ATGTTCTTGTACAGCTTGGT	DCST1__NM_152494	73	37	88	74
DCST2__NM_144622_ACAAGATTAAAGCTATTGCC	DCST2__NM_144622	193	241	178	150
DCST2__NM_144622_AGAGCTGTGGTTCGCAGCGT	DCST2__NM_144622	205	165	194	92
DCST2__NM_144622_CAGCCACCAACAGTAGTGTC	DCST2__NM_144622	256	498	277	397
DCST2__NM_144622_CCCACAGGCTACAGCCTCGC	DCST2__NM_144622	589	580	609	604
DCST2__NM_144622_CGATGTGTGCAACTCGGAAC	DCST2__NM_144622	585	585	717	956
DCST2__NM_144622_GATGTTGTGCACCCCTTGGG	DCST2__NM_144622	96	21	52	28
DCST2__NM_144622_GGAGCCACTGCCACACATTC	DCST2__NM_144622	1951	1504	2176	1796
DCST2__NM_144622_GGCCCTCTGTAGCACTTCGG	DCST2__NM_144622	832	596	1020	957
DCST2__NM_144622_GGGCCGGACACTACTGTTGG	DCST2__NM_144622	1116	1086	1103	993
DCST2__NM_144622_TGCACACATCGCCGATGTGC	DCST2__NM_144622	226	91	197	234
DCST2__NM_144622_TTCTTTCGGTCAATCATGGA	DCST2__NM_144622	14	0	2	21
DCST2__NM_144622_TTGTCTTTAGCCACGGCCTA	DCST2__NM_144622	50	154	63	94
DCSTAMP__NM_001257317_AATATATTGAGGCAATTCAG	DCSTAMP__NM_001257317	68	37	106	68
DCSTAMP__NM_001257317_AGGTCATCAAATACACTTAG	DCSTAMP__NM_001257317	451	399	517	649
DCSTAMP__NM_001257317_ATGGTATGACTTGCAACCTA	DCSTAMP__NM_001257317	244	383	244	244
DCSTAMP__NM_001257317_CAGAACACACGTGATAATCC	DCSTAMP__NM_001257317	610	532	525	466
DCSTAMP__NM_001257317_CCTCTGTGGTTGTTGCCATC	DCSTAMP__NM_001257317	1016	867	1093	1307
DCSTAMP__NM_001257317_CGTAAATCTCCCAAAGACTT	DCSTAMP__NM_001257317	972	1014	1010	673
DCSTAMP__NM_001257317_GCAGCGGCCGCTATGATTGA	DCSTAMP__NM_001257317	1586	1815	1786	1948
DCSTAMP__NM_001257317_GGTTGCAAGTCATACCATCT	DCSTAMP__NM_001257317	662	558	644	612
DCSTAMP__NM_001257317_GTTTCTGCCATCAATCATAG	DCSTAMP__NM_001257317	105	735	76	137
DCSTAMP__NM_001257317_TGGTATGACTTGCAACCTAA	DCSTAMP__NM_001257317	0	0	0	0
DCSTAMP__NM_001257317_TTGGTTGCTCTTATTTCAGT	DCSTAMP__NM_001257317	121	181	142	133
DCSTAMP__NM_001257317_TTTGGTTGCTCTTATTTCAG	DCSTAMP__NM_001257317	108	148	178	202
DCT__NM_001129889_AACTTTGCCGGCTATAATTG	DCT__NM_001129889	732	518	889	813
DCT__NM_001129889_ACCACTCCAGGGCCTTGTGT	DCT__NM_001129889	3415	3120	3975	3351
DCT__NM_001129889_ATCCTGGTTTCGTAGGATGT	DCT__NM_001129889	312	320	392	373
DCT__NM_001129889_ATGTTCTGCCGAATCACTGG	DCT__NM_001129889	295	389	275	329
DCT__NM_001129889_GGAAGAAACCACCAGTGATT	DCT__NM_001129889	426	580	514	433
DCT__NM_001129889_GTGGAGACTGCAAGTTTGGC	DCT__NM_001129889	202	214	224	302
DCT__NM_001129889_GTGTGGTGATCACGTAGTCG	DCT__NM_001129889	1227	726	1117	985
DCT__NM_001129889_GTTCCCCGGAGTCTGCATGA	DCT__NM_001129889	317	219	347	242
DCT__NM_001129889_TCCTGGTTTCGTAGGATGTA	DCT__NM_001129889	296	562	363	476
DCT__NM_001129889_TCTCTTCTTCGCGAGATCTA	DCT__NM_001129889	118	38	119	113
DCT__NM_001129889_TGGATGTTCTGCCGAATCAC	DCT__NM_001129889	22	12	16	32
DCT__NM_001129889_TTTCCTGAGGACTCAAGGAA	DCT__NM_001129889	24	67	32	29
DCTD__NM_001012732_AAAAATTCGACCGATGTGAA	DCTD__NM_001012732	112	290	193	240
DCTD__NM_001012732_AAAACAAGATTGTCGGGATT	DCTD__NM_001012732	124	344	240	286
DCTD__NM_001012732_AAGGACAGCAGAGAATAAGC	DCTD__NM_001012732	71	19	68	83
DCTD__NM_001012732_AGGCTCCTGTTTAATATGGC	DCTD__NM_001012732	253	393	297	348
DCTD__NM_001012732_ATTCAGAAAACAAGATTGTC	DCTD__NM_001012732	73	21	83	107
DCTD__NM_001012732_CAAGAAACGGGACGACTATT	DCTD__NM_001012732	1196	936	1261	1544
DCTD__NM_001012732_GAAAACAAGATTGTCGGGAT	DCTD__NM_001012732	165	187	132	157
DCTD__NM_001012732_GCAGGAAACTTCACTCATGT	DCTD__NM_001012732	1333	1410	1457	1672
DCTD__NM_001012732_GCAGTGATGACGTGTTGCCT	DCTD__NM_001012732	1332	1363	1569	1800
DCTD__NM_001012732_GCCACAGCCATAAAATACTC	DCTD__NM_001012732	170	202	168	209
DCTD__NM_001012732_TAAATACCATGATAGTGACG	DCTD__NM_001012732	123	123	231	242
DCTD__NM_001012732_TTGTCGGGATTGGGTACAAT	DCTD__NM_001012732	741	741	784	675
DCTN1__NM_001135040_AACTGCAAAGACTAGCAAAC	DCTN1__NM_001135040	163	42	213	94
DCTN1__NM_001135040_AGTACTTCACTTGTGATGAA	DCTN1__NM_001135040	27	38	55	2
DCTN1__NM_001135040_ATGATGGAACTGTTCAAGGC	DCTN1__NM_001135040	196	121	231	189
DCTN1__NM_001135040_CAAAGCAAAGCTAAAAGAGC	DCTN1__NM_001135040	63	224	140	131
DCTN1__NM_001135040_CAAATGGGTAGGCGTGATTC	DCTN1__NM_001135040	766	186	881	342
DCTN1__NM_001135040_CCCACGCGCCCAGCCAGTAC	DCTN1__NM_001135040	1169	909	1412	1232
DCTN1__NM_001135040_CGAGGCACTGTGGCCTATGT	DCTN1__NM_001135040	1122	232	1386	432
DCTN1__NM_001135040_GCAAGAGGCACGTGTACAGC	DCTN1__NM_001135040	484	533	625	731
DCTN1__NM_001135040_GGAGGAGGGACTAAGGGCTC	DCTN1__NM_001135040	77	35	53	57
DCTN1__NM_001135040_GGAGGCAAAGGAACGCTATA	DCTN1__NM_001135040	232	169	268	193
DCTN1__NM_001135040_GGCTCCCGTGTAGAGGTGAT	DCTN1__NM_001135040	663	767	821	1032
DCTN1__NM_001135040_TTTGCCAGTGGCAAACAGTG	DCTN1__NM_001135040	17	2	19	0
DCTN1__NM_001135041_CAGCAGGGACATGTGTCGAT	DCTN1__NM_001135041	956	376	912	442
DCTN1__NM_001135041_CCAGCTGACCGCCCATCTAC	DCTN1__NM_001135041	1076	152	1291	394
DCTN1__NM_001135041_CTGCTCATAGCGGTGTAGCG	DCTN1__NM_001135041	171	109	194	243
DCTN1__NM_001135041_GGACATGTGTCGATTGGCCT	DCTN1__NM_001135041	1295	1838	1436	2052
DCTN1__NM_001135041_GGCAAAGGAACGCTATATGG	DCTN1__NM_001135041	705	515	738	305
DCTN1__NM_001135041_GTGGATGTGTATAAGAAAGT	DCTN1__NM_001135041	101	6	119	67
DCTN1__NM_001135041_TAAGTGAGAACTGTTCAGAG	DCTN1__NM_001135041	464	177	610	169
DCTN1__NM_001135041_TGTGGATGTGTATAAGAAAG	DCTN1__NM_001135041	132	32	191	7
DCTN1__NM_004082_GCGGGGACTGAAGCCTAAGA	DCTN1__NM_004082	460	588	515	562
DCTN2__NM_001261412_AAGAGGACAGGATATGAATC	DCTN2__NM_001261412	276	166	259	143
DCTN2__NM_001261412_AGAGTGGGGACAAAGGGACT	DCTN2__NM_001261412	356	222	298	93
DCTN2__NM_001261412_ATCCAAAACTGCAAGGTCTA	DCTN2__NM_001261412	283	100	360	81
DCTN2__NM_001261412_CCACTTAGCCAGGGCGCCAT	DCTN2__NM_001261412	683	283	803	213
DCTN2__NM_001261412_CCGGGAAGGTCGGCGTATTT	DCTN2__NM_001261412	908	491	870	519
DCTN2__NM_001261412_CCTACCTGAGGATGATCAAG	DCTN2__NM_001261412	454	199	510	197
DCTN2__NM_001261412_CGGGAAGGTCGGCGTATTTA	DCTN2__NM_001261412	1124	553	1119	404
DCTN2__NM_001261412_GCAGATTTCTCAGATCGTAT	DCTN2__NM_001261412	480	441	634	561
DCTN2__NM_001261412_TCGCTAGTTTCATAAACATC	DCTN2__NM_001261412	182	151	203	251
DCTN2__NM_001261412_TGAAACTAGCGACCTACCTG	DCTN2__NM_001261412	190	10	214	21
DCTN2__NM_001261412_TGACAAGGCTGCTATCTGGG	DCTN2__NM_001261412	307	38	346	147
DCTN2__NM_001261412_TGTAGAGCTGTTGCAAGCAA	DCTN2__NM_001261412	288	80	190	21
DCTN3__NM_001281425_AATTGCAGCTTAGAGGCATC	DCTN3__NM_001281425	214	22	235	60
DCTN3__NM_001281425_CTCTGCTGGCTTCACTTGCG	DCTN3__NM_001281425	816	908	962	1287
DCTN3__NM_001281425_TCTCCAAGCAATTCGGTGCAG	DCTN3__NM_001281425	750	739	761	1008
DCTN3__NM_001281425_TGAAGATCTGATCAAGTACC	DCTN3__NM_001281425	312	199	402	348
DCTN3__NM_001281425_TGAGCTACTTTGCCAGCTAG	DCTN3__NM_001281425	156	74	95	78
DCTN3__NM_001281426_ATGTGAGCACTGTCCAGCAT	DCTN3__NM_001281426	213	112	175	90
DCTN3__NM_001281426_CTGCTCCAGGAGTGCAACCT	DCTN3__NM_001281426	349	14	363	84
DCTN3__NM_001281426_GTGCTACACTTTGGCCCTAC	DCTN3__NM_001281426	611	575	793	707
DCTN3__NM_001281426_TTCCTTTCTGTGCTACACTT	DCTN3__NM_001281426	69	62	88	75
DCTN3__NM_001281426_TTTCCACACACTGGTCCTGT	DCTN3__NM_001281426	762	344	800	525
DCTN3__NM_001281427_GAGAAGCATTGTCTGGTTGT	DCTN3__NM_001281427	856	679	873	788
DCTN3__NM_024348_GGGAGTGGGAGTCCGTGATG	DCTN3__NM_024348	975	934	967	845
DCTN3__NM_024348_TGATGAGGCAGGAAGTTTAG	DCTN3__NM_024348	188	303	164	222
DCTN4__NM_001135643_ACCGCAGTTCGCTACAATAG	DCTN4__NM_001135643	1234	633	1462	712
DCTN4__NM_001135643_CATGAAGAAAGCCTATTACC	DCTN4__NM_001135643	434	141	600	187
DCTN4__NM_001135643_CCGGGTTCTCTATCTAGTCC	DCTN4__NM_001135643	452	327	505	264
DCTN4__NM_001135643_GCAACATACTATTCATGTAG	DCTN4__NM_001135643	566	710	570	420
DCTN4__NM_001135643_GCATCCATCAGTACCCTTGC	DCTN4__NM_001135643	3820	2654	4008	3514
DCTN4__NM_001135643_GCATGCACACCCTCTCTACT	DCTN4__NM_001135643	7166	2369	7729	2512
DCTN4__NM_001135643_GCCGCTATTGTAGCGAACTG	DCTN4__NM_001135643	490	59	546	193
DCTN4__NM_001135643_TAAACAACTGGGACAATAAT	DCTN4__NM_001135643	367	115	400	202
DCTN4__NM_001135643_TCCAGGGAGAAAAGAAGGTT	DCTN4__NM_001135643	129	127	110	85
DCTN4__NM_001135643_TGAGCGAGATCGCAAGAAAC	DCTN4__NM_001135643	146	74	165	73
DCTN4__NM_001135643_TGCTCGTGGCCCGAGTAGAG	DCTN4__NM_001135643	100	42	124	122
DCTN4__NM_001135643_TTGTAGCGAACTGCGGTCGC	DCTN4__NM_001135643	55	59	54	15
DCTN4__NM_001135644_AACCTTCATGTAGCGAAGGT	DCTN4__NM_001135644	486	828	533	674
DCTN4__NM_001135644_ACCTTCATGTAGCGAAGGTT	DCTN4__NM_001135644	619	756	741	817
DCTN4__NM_001135644_ATCAGAAGATGTTTGTGGCG	DCTN4__NM_001135644	883	705	935	981
DCTN4__NM_001135644_CGAAGAGAGTCACATGGGTG	DCTN4__NM_001135644	320	358	344	488
DCTN4__NM_001135644_CTGGATTTGTAAGAGTCAGG	DCTN4__NM_001135644	238	15	329	22
DCTN4__NM_001135644_GCAGTGCTGTTGATATCATC	DCTN4__NM_001135644	522	608	630	574
DCTN4__NM_001135644_TTCTGAAGGCTATAATGCTA	DCTN4__NM_001135644	49	61	18	48
DCTN5__NM_001199011_AGCCAGAACATCGTTCTCAA	DCTN5__NM_001199011	366	30	417	1
DCTN5__NM_001199011_GACATCTGATATATGGGCTC	DCTN5__NM_001199011	57	49	36	81
DCTN5__NM_001199743_ATGAATGACTGTATTATCCG	DCTN5__NM_001199743	236	57	334	66
DCTN5__NM_001199743_GATCTGGCAAATGTAAGAGT	DCTN5__NM_001199743	837	6	966	22
DCTN5__NM_001199743_GCATTCTTTCCTTTACATAT	DCTN5__NM_001199743	595	784	737	916
DCTN5__NM_001199743_TGAAAAGTCGTAGTGTCATA	DCTN5__NM_001199743	394	81	344	107
DCTN5__NM_001199743_TGAATGACTGTATTATCCGA	DCTN5__NM_001199743	80	0	111	2
DCTN5__NM_032486_AGATGCTAGACTTGCGTCAG	DCTN5__NM_032486	380	477	395	272
DCTN5__NM_032486_ATCAGCTCCTGAGTGCACTC	DCTN5__NM_032486	206	0	289	31
DCTN5__NM_032486_GAGATGCTAGACTTGCGTCA	DCTN5__NM_032486	583	500	628	539
DCTN5__NM_032486_GGAGCTCCGGAGTGCACTC	DCTN5__NM_032486	647	62	577	48
DCTN6__NM_006571_CAATAATTCTTGCTTTAGGG	DCTN6__NM_006571	240	62	250	146
DCTN6__NM_006571_CATAGATCACCGTATTCTCA	DCTN6__NM_006571	192	15	293	28
DCTN6__NM_006571_CCTGAGAATACGGTGATCTA	DCTN6__NM_006571	397	6	467	3
DCTN6__NM_006571_CGGAGAAGACTCAAAAGAGG	DCTN6__NM_006571	256	94	350	111
DCTN6__NM_006571_GAACCAAAACCTATGATCAT	DCTN6__NM_006571	803	25	1012	54
DCTN6__NM_006571_GAAGCCGGGCCAATAGTGAT	DCTN6__NM_006571	349	6	458	1
DCTN6__NM_006571_GCCAACTTCAAACACATTAT	DCTN6__NM_006571	1021	995	1081	861
DCTN6__NM_006571_GGCCAATAGTGATTGGCGAA	DCTN6__NM_006571	227	110	243	207
DCTN6__NM_006571_GGGCCAATAGTGATTGGCGA	DCTN6__NM_006571	109	130	201	213
DCTN6__NM_006571_TAAGGGCCTGTTCTTCTATT	DCTN6__NM_006571	329	0	496	25
DCTN6__NM_006571_TTAGGGTGGATCACTGTCCG	DCTN6__NM_006571	262	0	256	9
DCTN6__NM_006571_TTATTGGTGCCAATGATCAT	DCTN6__NM_006571	177	0	234	1
DCTPP1__NM_024096_AGTTTGCTGCGGAACGAGAC	DCTPP1__NM_024096	300	274	356	522
DCTPP1__NM_024096_GATGGGCTGGGTAGGCGTCGC	DCTPP1__NM_024096	200	357	308	275
DCTPP1__NM_024096_GCGGCAGATCCACACGGCAG	DCTPP1__NM_024096	1099	1077	1151	983
DCTPP1__NM_024096_GGCCGGTGGGGAGATTCGTG	DCTPP1__NM_024096	1104	1463	1324	1492
DCTPP1__NM_024096_GGGGCAATTCTGTATACTTG	DCTPP1__NM_024096	484	719	669	742
DCTPP1__NM_024096_GTGGCCGGTGGGGAGATTCG	DCTPP1__NM_024096	272	184	309	365
DCTPP1__NM_024096_TAGTCAGTGGAAAACCGATG	DCTPP1__NM_024096	638	772	617	583
DCTPP1__NM_024096_TGGCCGGTGGGGAGATTCGT	DCTPP1__NM_024096	287	305	378	315
DCUN1D1__NM_020640_AATAATCAACACACTAATGC	DCUN1D1__NM_020640	107	64	164	33
DCUN1D1__NM_020640_ACAAAGTCATCAATAAGAAC	DCUN1D1__NM_020640	185	143	220	228
DCUN1D1__NM_020640_ATTAAGCACTAAGTTCCAGT	DCUN1D1__NM_020640	1114	928	953	887
DCUN1D1__NM_020640_CTCTCTGTATATAAAGTTC	DCUN1D1__NM_020640	43	37	83	115
DCUN1D1__NM_020640_CTTGTTCCATCTTGGGTATC	DCUN1D1__NM_020640	524	600	568	742
DCUN1D1__NM_020640_GAACAAGAATTGAAAGAACC	DCUN1D1__NM_020640	79	140	90	54
DCUN1D1__NM_020640_GCCATCCATGAACTCCTGTT	DCUN1D1__NM_020640	2156	1939	2347	2226
DCUN1D1__NM_020640_GTGTAAAAGGATCATTGGAC	DCUN1D1__NM_020640	252	362	205	176
DCUN1D1__NM_020640_TGGATGGCATGACAGAATTA	DCUN1D1__NM_020640	16	2	33	43
DCUN1D1__NM_020640_TTGCACGCCCTCAAATTGCT	DCUN1D1__NM_020640	820	985	772	1212
DCUN1D2__NM_001014283_AAAACAAAATTGGAGTCGAT	DCUN1D2__NM_001014283	394	341	415	553
DCUN1D2__NM_001014283_ACTGCTTAACGCAGAATGAG	DCUN1D2__NM_001014283	29	74	30	45
DCUN1D2__NM_001014283_CCTGAGTGCACGCCATAAAC	DCUN1D2__NM_001014283	570	488	566	257
DCUN1D2__NM_001014283_CGGCTGTACGGCAGGTACAA	DCUN1D2__NM_001014283	142	56	172	161
DCUN1D2__NM_001014283_GAAAACAAAATTGGAGTCGA	DCUN1D2__NM_001014283	235	237	303	436
DCUN1D2__NM_001014283_GACTCCCTGTGGAGCGAGTC	DCUN1D2__NM_001014283	419	499	419	453
DCUN1D2__NM_001014283_GGACAAGGTCCGCCAGTTTA	DCUN1D2__NM_001014283	259	403	365	387
DCUN1D2__NM_001014283_TAGAAATGGCTGTTGCGTAT	DCUN1D2__NM_001014283	420	433	382	448
DCUN1D2__NM_001014283_TCACCTTCGCTAAGAACCCA	DCUN1D2__NM_001014283	1355	1043	1492	1138
DCUN1D2__NM_001014283_TCAGTGTATTGGTCATAGCG	DCUN1D2__NM_001014283	23	52	29	39
DCUN1D2__NM_001014283_TCAGTGTGAATTTAGCAGAA	DCUN1D2__NM_001014283	262	274	217	232
DCUN1D2__NM_001014283_TCTCTGGAACACATTCTTAA	DCUN1D2__NM_001014283	436	342	533	384
DCUN1D3__NM_173475_AGAACTGTTCAGGCGCTACA	DCUN1D3__NM_173475	436	412	510	651
DCUN1D3__NM_173475_AGCAGCACTCGAAATTCTGT	DCUN1D3__NM_173475	1442	1766	1412	1766
DCUN1D3__NM_173475_ATTCTGTGGGGTCAACACAC	DCUN1D3__NM_173475	145	282	162	116
DCUN1D3__NM_173475_CAAACAAGAGGATAAATTCA	DCUN1D3__NM_173475	407	542	428	592
DCUN1D3__NM_173475_CAGTTCTTCCAATCTTTGCA	DCUN1D3__NM_173475	441	398	421	688
DCUN1D3__NM_173475_CGATGAGGATTCTTACACT	DCUN1D3__NM_173475	1157	797	1206	892
DCUN1D3__NM_173475_CTTGAATTTATCCTCTTGTT	DCUN1D3__NM_173475	1043	1549	1226	1565
DCUN1D3__NM_173475_GCAACAAGTCACATAGCAGG	DCUN1D3__NM_173475	195	409	222	190
DCUN1D3__NM_173475_GGAGTCCAAGTCCAATGCCG	DCUN1D3__NM_173475	574	466	776	762
DCUN1D3__NM_173475_TAAGAATCCCTCATCGACCC	DCUN1D3__NM_173475	269	268	328	228
DCUN1D3__NM_173475_TGGCTTCTGTTAAGAGGCTA	DCUN1D3__NM_173475	22	66	4	0
DCUN1D3__NM_173475_TTGCTGCCCAGGGTCGATGA	DCUN1D3__NM_173475	329	356	364	334
DCUN1D4__NM_001040402_ACTCTAGGACATTGCACCAC	DCUN1D4__NM_001040402	1091	1319	1222	1362
DCUN1D4__NM_001040402_CCAAGAAAAGTAGACATGAT	DCUN1D4__NM_001040402	616	1260	795	1026
DCUN1D4__NM_001040402_GACTGTTATTAGGAAAAATC	DCUN1D4__NM_001040402	142	83	131	163
DCUN1D4__NM_001040402_TAACAGTCCCAACATGCACT	DCUN1D4__NM_001040402	339	369	352	424
DCUN1D4__NM_001040402_TACCTGTTTGGTGATCGTCT	DCUN1D4__NM_001040402	386	448	446	462
DCUN1D4__NM_001287755_AGCGTTGGGCTGTAGCGGGC	DCUN1D4__NM_001287755	577	698	634	699
DCUN1D5__NM_032299_CATAGTTACTAAGATCAGCA	DCUN1D5__NM_032299	61	30	110	78
DCUN1D5__NM_032299_CCCCCTGCTAGACTAATAAG	DCUN1D5__NM_032299	359	321	279	299
DCUN1D5__NM_032299_CTCCACTTATTAGTCTAGCT	DCUN1D5__NM_032299	478	592	618	620
DCUN1D5__NM_032299_TCTCCACTTATTAGTCTAGC	DCUN1D5__NM_032299	111	134	116	173
DCUN1D5__NM_032299_TGGTAAAATACTGAAAACAG	DCUN1D5__NM_032299	21	3	30	6
DCX__NM_000555_AAAGCTGCGAAAACGGTCAG	DCX__NM_000555	158	218	211	170
DCX__NM_000555_ACGAAAGAGATAAGACATCC	DCX__NM_000555	348	350	407	321
DCX__NM_000555_AGGCGTCAAAGCTGCGAAAA	DCX__NM_000555	289	206	251	428
DCX__NM_000555_ATCCAGGAAGATCGGAAGCA	DCX__NM_000555	476	543	574	722
DCX__NM_000555_CGGGCTAAATACATTAAAAC	DCX__NM_000555	232	368	180	132
DCX__NM_000555_CTACAGTGGGCGCTGTGAGT	DCX__NM_000555	1215	1342	1579	1715
DCX__NM_000555_GCGGATGATGGTAACCAGCT	DCX__NM_000555	732	834	617	703
DCX__NM_000555_GGACTGGGGGGCTTTCATAT	DCX__NM_000555	1107	1074	1271	1091
DCX__NM_000555_TAAGCTGGAGATGCTAACCT	DCX__NM_000555	373	249	331	213
DCX__NM_000555_TCGCAGCTTTGACGCCTTGC	DCX__NM_000555	260	416	356	478
DCX__NM_000555_TGCGAGGCTCCCGGATGAAT	DCX__NM_000555	586	778	631	650
DCX__NM_000555_TGTCAGACAGAGATCGCGTC	DCX__NM_000555	324	595	461	555
DCX__NM_001195553_CTACACTCTGGATGGAAAAC	DCX__NM_001195553	223	142	316	300
DCX__NM_001195553_TGAAAAATTTCGCTATGCTC	DCX__NM_001195553	17	7	20	49
DCXR__NM_001195218_ACAGTGGTGATGACGTCCAT	DCXR__NM_001195218	979	729	971	685
DCXR__NM_001195218_AGAACCGTGTGCGTGGACC	DCXR__NM_001195218	954	770	895	754
DCXR__NM_001195218_ATGCTGAACCGAATCCCACT	DCXR__NM_001195218	776	618	879	607
DCXR__NM_001195218_CTGGCTGGAGACATTCACGA	DCXR__NM_001195218	870	510	897	601
DCXR__NM_001195218_GACAGCCTTGTCCGCGAGGT	DCXR__NM_001195218	454	275	492	394
DCXR__NM_001195218_GCCAGGGGCTTAATAGCCCG	DCXR__NM_001195218	112	216	156	129
DCXR__NM_001195218_TATGGTTAGTTACTGCCCGC	DCXR__NM_001195218	525	434	582	493
DCXR__NM_001195218_TGCCCTGGACATGCTGACCA	DCXR__NM_001195218	483	335	562	460
DCXR__NM_001195218_TGGCCAGGGGCTTAATAGCC	DCXR__NM_001195218	0	0	3	5
DCXR__NM_016286_GGCATGGGGCTTGAGGTGTG	DCXR__NM_016286	1029	979	1078	938
DCXR__NM_016286_GGGTAGGATGAGCACGGCAT	DCXR__NM_016286	604	626	720	930
DCXR__NM_016286_GGTAGGATGAGCACGGCATG	DCXR__NM_016286	326	399	397	524
DDA1__NM_024050_AAGTGTCCTCGTGCATGTCT	DDA1__NM_024050	534	292	607	344
DDA1__NM_024050_CACCTCCCCGCAAGGTGGCG	DDA1__NM_024050	10	5	14	27
DDA1__NM_024050_CGGGTAGGCAGGTAGACTGA	DDA1__NM_024050	441	89	578	111
DDA1__NM_024050_TAAGTGTCCTCGTGCATGTC	DDA1__NM_024050	281	226	308	293
DDA1__NM_024050_TCAGACGGGTACTCGCGGGT	DDA1__NM_024050	1661	437	1828	525
DDA1__NM_024050_TCGGTCCGCGCCACCTTGCG	DDA1__NM_024050	1047	1031	1030	1281
DDA1__NM_024050_TCTGTTCAGACGGGTACTCG	DDA1__NM_024050	325	147	266	105
DDAH1__NM_001134445_AGCAAGACGTGCCCTTTGTT	DDAH1__NM_001134445	2591	1983	2649	1765
DDAH1__NM_001134445_ATCTGCACAGAAGGCCCTTA	DDAH1__NM_001134445	637	677	745	514
DDAH1__NM_001134445_CACTAGACCCAATTGCGATC	DDAH1__NM_001134445	402	314	419	410
DDAH1__NM_001134445_CAGTTTGCTGCTATGTCATC	DDAH1__NM_001134445	674	921	603	894
DDAH1__NM_001134445_CCAGTTCAGACATGCTCACG	DDAH1__NM_001134445	549	405	656	316
DDAH1__NM_001134445_GACCCAATTGCGATCAGGTT	DDAH1__NM_001134445	788	824	822	948
DDAH1__NM_001134445_GATGAAAATGCAACTTTAGA	DDAH1__NM_001134445	506	526	627	703
DDAH1__NM_001134445_GCACAGTGAGTTTGTCGTAG	DDAH1__NM_001134445	1058	1273	1159	1154
DDAH1__NM_001134445_GGCCTAACCTGATCGCAATT	DDAH1__NM_001134445	1005	1646	1007	1295
DDAH1__NM_001134445_GGGCCTAACCTGATCGCAAT	DDAH1__NM_001134445	390	634	596	729
DDAH1__NM_001134445_TCCAAAAGGACAAATCAACG	DDAH1__NM_001134445	476	368	341	381
DDAH1__NM_001134445_TGCTCACGGGGATCAGCATA	DDAH1__NM_001134445	0	0	0	0
DDAH1__NM_012137_CACGCGCTGAGAAGCGCCAA	DDAH1__NM_012137	22	15	22	73
DDAH1__NM_012137_CCACGAAGACGCAGTCCGGA	DDAH1__NM_012137	187	315	275	287
DDAH1__NM_012137_CGCGTGCTGGCCGAGCGACT	DDAH1__NM_012137	1080	833	1284	1053
DDAH1__NM_012137_CGGCCCTCATCACCCGACCC	DDAH1__NM_012137	2429	2485	2503	2333
DDAH1__NM_012137_TCCGGAAGGCTCTCGTCGGC	DDAH1__NM_012137	843	723	1048	753
DDAH1__NM_012137_TCCTCCACGAAGACGCAGTC	DDAH1__NM_012137	57	66	91	83
DDAH1__NM_012137_TGGCGCTTCTCAGCGCGTGC	DDAH1__NM_012137	2	0	1	0
DDB1__NM_001923_AATCAAGGCTTGGTGGTCTA	DDB1__NM_001923	237	235	233	317
DDB1__NM_001923_ACGCAGCCGTTCACGGCGGT	DDB1__NM_001923	177	9	211	11
DDB1__NM_001923_AGGCCAATCATCCGGCACTC	DDB1__NM_001923	526	41	533	78
DDB1__NM_001923_ATTGATGTCAAGTTCCTATA	DDB1__NM_001923	260	10	390	21
DDB1__NM_001923_ATTGGCCTGCGTCTCTATGA	DDB1__NM_001923	419	24	514	28
DDB1__NM_001923_CAGAAGCCCACCGCCGTGAA	DDB1__NM_001923	555	0	662	1
DDB1__NM_001923_CCCGCCCCACTTCCACATAG	DDB1__NM_001923	921	829	1043	874
DDB1__NM_001923_GACCCTGAGTGCCGGATGAT	DDB1__NM_001923	1546	442	1752	663
DDB1__NM_001923_GCCGTGAACGGCTGCGTGAC	DDB1__NM_001923	124	9	139	0
DDB1__NM_001923_GGGGCTTCGGCCCGTCAAAG	DDB1__NM_001923	46	83	32	49
DDB1__NM_001923_GGTCACGCAGCCGTTCACGG	DDB1__NM_001923	477	3	453	15
DDB1__NM_001923_GTCAATGATGCCAATAATGC	DDB1__NM_001923	418	121	409	185
DDB1__NM_001923_TCAAAGAGGTGGGCATGTAT	DDB1__NM_001923	229	2	317	10
DDB1__NM_001923_TCTTTATTATCGCGATCCTAG	DDB1__NM_001923	127	181	126	235
DDB1__NM_001923_TTGGCAGTGTAGGGAGGTTGT	DDB1__NM_001923	576	581	694	894
DDB2__NM_000107_AAAACTGGTTGGTATTGAGA	DDB2__NM_000107	143	98	161	152
DDB2__NM_000107_AAGGGGGAGATATCATGCTC	DDB2__NM_000107	65	37	34	41
DDB2__NM_000107_ACTAGGCTGCAAGACTTTAA	DDB2__NM_000107	293	361	321	306
DDB2__NM_000107_GACCCTCCACCAGCATAAGC	DDB2__NM_000107	0	0	0	0
DDB2__NM_000107_GATGTAGCCCTCCTGTCAAA	DDB2__NM_000107	894	805	1106	715
DDB2__NM_000107_GCCAAGAAGCTCTGTGCGAA	DDB2__NM_000107	329	248	449	369
DDB2__NM_000107_GGAGGGTCCTGACGATGCTG	DDB2__NM_000107	470	662	603	555
DDB2__NM_000107_GGGGCGTAATACAATCTCGG	DDB2__NM_000107	781	991	987	1105
DDB2__NM_000107_GTCTGCTAGTAGCCGAATGG	DDB2__NM_000107	320	305	474	282
DDB2__NM_000107_TGAGGAGGCGTAAAACTGGT	DDB2__NM_000107	474	466	641	736
DDB2__NM_000107_TGCACACTCTGGATTCTTAC	DDB2__NM_000107	478	492	572	558
DDB2__NM_000107_TGTGTCTGCTAGTAGCCGAA	DDB2__NM_000107	250	134	230	234
DDC__NM_000790_ATCAGCGGCCGCAGGTACCC	DDC__NM_000790	418	379	429	224
DDC__NM_000790_ATGATGTCCTCAAACGTGTC	DDC__NM_000790	248	296	353	371
DDC__NM_000790_ATGCTTGCGGACATGCTGTG	DDC__NM_000790	462	591	483	715
DDC__NM_000790_CAACTACATGGAAGGCATTG	DDC__NM_000790	883	987	949	950
DDC__NM_000790_CTCAATGCCTTCCATGTAGT	DDC__NM_000790	379	517	412	591
DDC__NM_000790_CTCGGACCAAAGTGATCCAT	DDC__NM_000790	1101	1144	1177	968
DDC__NM_000790_GCTCACACAGGCCGCTATCA	DDC__NM_000790	1080	1047	1281	1087
DDC__NM_000790_GCTTGCGGACATGCTGTGCG	DDC__NM_000790	1129	1698	1192	1412
DDC__NM_000790_GGGAAGTAGGCGAAGAAGTA	DDC__NM_000790	217	576	262	287
DDC__NM_000790_TACATGGCCAACTACATGGA	DDC__NM_000790	407	390	510	467
DDC__NM_000790_TCGGAATTCACTTGCGTTCA	DDC__NM_000790	363	541	483	609
DDC__NM_000790_TTGAGAAGATAATCATGCCT	DDC__NM_000790	366	258	511	328
DDC__NM_001242886_GATGAATGCACTGCCTGCGT	DDC__NM_001242886	996	1016	1071	1070
DDC__NM_001242886_GTGGAAAGAGCTGGGTTAAT	DDC__NM_001242886	105	84	82	82
DDC__NM_001242886_TTAAAAGCCATCCCCTCAGA	DDC__NM_001242886	828	1105	1051	1096
DDC__NM_001242889_TCAGAAGGTGCCGGAACTCA	DDC__NM_001242889	428	470	360	406
DDHD1__NM_001160147_AGCCCCGAGCATAACGGCCG	DDHD1__NM_001160147	1049	1286	1228	1591
DDHD1__NM_001160147_CCACGGAGCCCCGAGCATAA	DDHD1__NM_001160147	297	332	317	448
DDHD1__NM_001160147_CCCGGGCTGCATTTGGCGCC	DDHD1__NM_001160147	841	1352	1040	1158
DDHD1__NM_001160147_CCGCCTCGGCCGTTATGCTC	DDHD1__NM_001160147	56	29	53	28
DDHD1__NM_001160147_CGCCTCGGCCGTTATGCTCG	DDHD1__NM_001160147	484	517	520	751
DDHD1__NM_001160147_CGGGGAACCCGGGCTGCATT	DDHD1__NM_001160147	1278	1257	1289	1434
DDHD1__NM_001160147_GACTTCAGCTCCGCCGAGTC	DDHD1__NM_001160147	352	402	318	428
DDHD1__NM_001160147_GCCGCCTCGGCCGTTATGCT	DDHD1__NM_001160147	226	137	262	239
DDHD1__NM_001160147_GGGAGCTGGGCTCAGACGCG	DDHD1__NM_001160147	600	729	631	605
DDHD1__NM_001160147_TCAGACGCGAGGCCAGCGTT	DDHD1__NM_001160147	550	483	503	578
DDHD1__NM_001160147_TCCGCTGGTCCCGACGAACT	DDHD1__NM_001160147	746	921	567	743
DDHD1__NM_001160147_TGGTTGTGGTCGTCGGTGCC	DDHD1__NM_001160147	16	0	10	15
DDHD2__NM_001164232_AAGAGGCATATAGCTCTGGT	DDHD2__NM_001164232	24	0	28	10
DDHD2__NM_001164232_ATACTGGGATGAACTGGCAT	DDHD2__NM_001164232	695	829	730	888
DDHD2__NM_001164232_ATCCATGTCTATTAGCTCAA	DDHD2__NM_001164232	64	52	81	79
DDHD2__NM_001164232_CGGAAGTGAGACGATGTACG	DDHD2__NM_001164232	839	764	805	955
DDHD2__NM_001164232_GATGCGGTATGCTGTATACT	DDHD2__NM_001164232	1221	1106	1363	1488
DDHD2__NM_001164232_GCAGATATGATGTTCATTTG	DDHD2__NM_001164232	819	739	851	1020
DDHD2__NM_001164232_GGATGCGGTATGCTGTATAC	DDHD2__NM_001164232	870	948	969	1101
DDHD2__NM_001164232_GGGCAGATATGATGTTCATT	DDHD2__NM_001164232	920	1156	1117	1438
DDHD2__NM_001164232_TAATAGATTCTAAGGAGACA	DDHD2__NM_001164232	93	113	95	60
DDHD2__NM_001164232_TCAAAGGAACTACATGAGTT	DDHD2__NM_001164232	1465	1805	1696	1671
DDHD2__NM_001164232_TGGCTGAAGCTCTCCGAGTA	DDHD2__NM_001164232	29	23	65	89
DDHD2__NM_001164232_TTCCTTTGAGCTAATAGACA	DDHD2__NM_001164232	50	20	46	100
DDI1__NM_001001711_AAGTTTCGGAGCTCAAAGTC	DDI1__NM_001001711	161	184	233	193
DDI1__NM_001001711_AGAAGGACAATGTGGGACCT	DDI1__NM_001001711	134	232	132	124
DDI1__NM_001001711_ATCGCCATCTTTGAGGCCGT	DDI1__NM_001001711	1417	1321	1430	1143
DDI1__NM_001001711_ATGATCTGGATCTCTTCGAC	DDI1__NM_001001711	645	716	692	831
DDI1__NM_001001711_ATGCCACTGAAGTCTACACG	DDI1__NM_001001711	840	867	916	762
DDI1__NM_001001711_CAGCCTCGTGTAGACTTCAG	DDI1__NM_001001711	855	943	869	934
DDI1__NM_001001711_CATGGAGCGACTCCTCATCG	DDI1__NM_001001711	3178	3058	3612	3087
DDI1__NM_001001711_CCTCAAAGATGGCGATATCG	DDI1__NM_001001711	660	946	990	1033
DDI1__NM_001001711_CGCCAAGCCCTGTGACCGCT	DDI1__NM_001001711	3604	3816	3869	4657
DDI1__NM_001001711_CTCTGGACTCCGCTTCGCAG	DDI1__NM_001001711	173	94	244	205
DDI1__NM_001001711_TGAGCAGGGACAGATCGTGG	DDI1__NM_001001711	897	693	959	708
DDI1__NM_001001711_TTCAGTGGCATTGCGGTGCC	DDI1__NM_001001711	53	62	71	26
DDI2__NM_032341_ATACTACTGAAATCTATTCG	DDI2__NM_032341	171	115	292	82
DDI2__NM_032341_ATGTTCCTTCTCTATACTTG	DDI2__NM_032341	444	320	562	448
DDI2__NM_032341_ATTATTGGAAGGGTACATCT	DDI2__NM_032341	202	150	208	89
DDI2__NM_032341_CAAGGACAGCTCATGCGGGT	DDI2__NM_032341	1326	773	1211	780
DDI2__NM_032341_CTGTTCCTCAAGTATAGAGA	DDI2__NM_032341	175	113	189	85
DDI2__NM_032341_CTTCTTATGGCTTGAAAGAT	DDI2__NM_032341	120	188	184	222
DDI2__NM_032341_CTTGGCTCATGATAGTCATC	DDI2__NM_032341	329	140	327	202
DDI2__NM_032341_GCACCCAGAAGATTATTGGA	DDI2__NM_032341	1238	598	1280	872
DDI2__NM_032341_GCTTCTTATGGCTTGAAAGA	DDI2__NM_032341	227	143	206	184
DDI2__NM_032341_GGACATGCTTAAACGGCACC	DDI2__NM_032341	164	126	113	154
DDI2__NM_032341_TGAGACTGGTGGACCGTCGG	DDI2__NM_032341	471	295	449	362
DDI2__NM_032341_TGGGACTGGACATGCTTAAA	DDI2__NM_032341	140	11	161	63
DDIAS__NM_145018_AAGCCTGCAATACCTGGGTC	DDIAS__NM_145018	813	560	866	975
DDIAS__NM_145018_ACAGTAAAGCCTGCAATACC	DDIAS__NM_145018	587	838	542	513
DDIAS__NM_145018_AGAACTTGGCTTACAAGCTA	DDIAS__NM_145018	559	546	488	608
DDIAS__NM_145018_ATGCAAGTGAATTCAAGTGA	DDIAS__NM_145018	283	270	430	402
DDIAS__NM_145018_ATGGAGCCTTGTTTCATATA	DDIAS__NM_145018	415	359	441	451
DDIAS__NM_145018_CTACCAGACCCAGGTATTGC	DDIAS__NM_145018	1515	1438	1722	1649
DDIAS__NM_145018_CTGGCAAGCCACTAGTGCTT	DDIAS__NM_145018	160	256	151	157
DDIAS__NM_145018_CTGGGTCTGGTAGAACAATC	DDIAS__NM_145018	177	185	185	221
DDIAS__NM_145018_GCATTGCTGTAAGAAGTTAC	DDIAS__NM_145018	423	445	447	647
DDIAS__NM_145018_GGAAAGTTTGTATCTGTAAT	DDIAS__NM_145018	232	247	283	336
DDIAS__NM_145018_GTTCTGCTGAAGAGTACCAA	DDIAS__NM_145018	38	12	43	32
DDIAS__NM_145018_TAGCTGCCATGATCCCATTC	DDIAS__NM_145018	640	574	754	831
DDIT3__NM_001195053_AGGGAGCTCTGACTGGAATC	DDIT3__NM_001195053	181	66	167	90
DDIT3__NM_001195053_CCGAGCTCTGATTGACCGAA	DDIT3__NM_001195053	233	368	240	414
DDIT3__NM_001195053_CTTGGTGCAGATTCACCATT	DDIT3__NM_001195053	778	867	748	620
DDIT3__NM_004083_ACCCTCAATCCCACATACGC	DDIT3__NM_004083	472	307	529	281
DDIT3__NM_004083_CATCATTGGCACTAGTGAGA	DDIT3__NM_004083	478	409	388	260
DDIT3__NM_004083_CTCAATCCCACATACGCAGG	DDIT3__NM_004083	745	587	699	304
DDIT3__NM_004083_GAAGGCTTGGAGTAGACAAA	DDIT3__NM_004083	214	176	233	174
DDIT3__NM_004083_GTGGGTAGTGTGGCCCAAGT	DDIT3__NM_004083	123	79	152	117
DDIT4__NM_019058_ACGACGAGAAGCGGTCCCAA	DDIT4__NM_019058	412	479	383	416
DDIT4__NM_019058_CCAACTGGCTAGGCATCAGC	DDIT4__NM_019058	215	174	184	216
DDIT4__NM_019058_CCAGACACGGCTTACCTGGA	DDIT4__NM_019058	2241	2007	2580	3220
DDIT4__NM_019058_CCATCCAGGTAAGCCGTGTC	DDIT4__NM_019058	1954	2035	2233	3524
DDIT4__NM_019058_CCCAAAGGCTAGGCATGGTG	DDIT4__NM_019058	39	18	52	33
DDIT4__NM_019058_CGATCTGGGGTGGAGTTCG	DDIT4__NM_019058	1138	1564	1359	1984
DDIT4__NM_019058_CTGAGCGCGGCGGCCGATCT	DDIT4__NM_019058	1057	1079	1265	1472
DDIT4__NM_019058_GCCTAGCCAGTTGGTAAGCC	DDIT4__NM_019058	754	768	989	1342
DDIT4__NM_019058_TGAGTCCAGGCGCAGCACGA	DDIT4__NM_019058	231	156	253	219
DDIT4__NM_019058_TGCCCACCTGGCTTACCAAC	DDIT4__NM_019058	123	51	185	201
DDIT4__NM_019058_TGCGCCTGGACTCACGACTC	DDIT4__NM_019058	611	567	685	674
DDIT4__NM_019058_TGGGGTGGGAGTTCGGGGCA	DDIT4__NM_019058	52	9	75	30
DDIT4L__NM_145244_AACACAACCTCGCAAGCCGC	DDIT4L__NM_145244	685	828	938	1164
DDIT4L__NM_145244_AATATTACCTCGTTGAGGTT	DDIT4L__NM_145244	133	219	98	91
DDIT4L__NM_145244_ACGGAGCCCTGCGGCTTGCG	DDIT4L__NM_145244	741	936	840	871
DDIT4L__NM_145244_CACCCAGAGAGCCTGCTAAG	DDIT4L__NM_145244	515	417	588	682
DDIT4L__NM_145244_TGATTGAAGGGTCCTAAAAA	DDIT4L__NM_145244	43	5	39	34
DDN__NM_015086_AGCAGGTGAGCCTCGTAGCT	DDN__NM_015086	388	503	303	246
DDN__NM_015086_AGGTGAGCCTCGTAGCTTGG	DDN__NM_015086	372	471	295	265
DDN__NM_015086_CAGGTGAGCCTCGTAGCTTG	DDN__NM_015086	239	185	280	223
DDN__NM_015086_CCAAGGAGACCGAGCGAAAA	DDN__NM_015086	168	66	169	182
DDN__NM_015086_CGCCCCTTCTCGACAGCCCA	DDN__NM_015086	1924	1963	1879	1387
DDN__NM_015086_GCAGGTGAGCCTCGTAGCTT	DDN__NM_015086	233	231	235	107
DDN__NM_015086_GCCGCAGAGCGACCCAGGGT	DDN__NM_015086	0	0	0	0
DDN__NM_015086_GGAAAGCGGCGTCGCAGGAG	DDN__NM_015086	448	649	639	465
DDN__NM_015086_GGCGCGGCGACTATAATGAC	DDN__NM_015086	810	467	932	620
DDN__NM_015086_GGTGGAGCCACGTAGGGTGG	DDN__NM_015086	202	133	211	103
DDN__NM_015086_TCTGCGGCTGCGCGGATGGA	DDN__NM_015086	311	342	450	371
DDN__NM_015086_TTGCTCCCGAGCTCGGACCT	DDN__NM_015086	176	73	107	71
DDO__NM_003649_AAATGATGGTAACGGAGCAT	DDO__NM_003649	397	281	434	416
DDO__NM_003649_AATGATGGTAACGGAGCATC	DDO__NM_003649	214	189	224	144
DDO__NM_003649_AATTGCAAAGAGGTGATTAA	DDO__NM_003649	82	85	168	341
DDO__NM_003649_ACAGCACGGATTGCAGTTGT	DDO__NM_003649	2229	2447	2413	2305
DDO__NM_003649_AGCACGGATTGCAGTTGTCG	DDO__NM_003649	1237	1194	1438	1279
DDO__NM_003649_AGCTGACCAAACACATACT	DDO__NM_003649	469	407	595	471
DDO__NM_003649_AGTGTGAGGAATAAGCATTC	DDO__NM_003649	53	127	76	34
DDO__NM_003649_ATGATGGTAACGGAGCATCG	DDO__NM_003649	290	313	373	257
DDO__NM_003649_CACTGCTTCTGCGTGTGAAT	DDO__NM_003649	1158	1580	1136	1511
DDO__NM_003649_CTGGTGTTCATTTGGTATCA	DDO__NM_003649	767	711	799	873
DDO__NM_003649_CTTGTCTGAAATGATGGTAA	DDO__NM_003649	379	261	340	389
DDO__NM_003649_GCTGGTGTTCATTTGGTATC	DDO__NM_003649	664	791	716	817
DDO__NM_004032_ACAGTTGACCACGATGTCAA	DDO__NM_004032	286	200	352	230
DDOST__NM_005216_ACCATCAGTGCCTTTATTGA	DDOST__NM_005216	2052	139	2433	165
DDOST__NM_005216_AGCCTGTCTCTCATAAAGTA	DDOST__NM_005216	575	725	706	879
DDOST__NM_005216_AGGAGGGGGGTTATTAGGAT	DDOST__NM_005216	244	199	331	322
DDOST__NM_005216_AGTGCCTTTATTGACGGCGG	DDOST__NM_005216	638	971	833	1150
DDOST__NM_005216_ATCAGTGCCTTTATTGACGG	DDOST__NM_005216	708	26	720	134
DDOST__NM_005216_CACCGGAAGTACCCCATCTA	DDOST__NM_005216	3082	3060	3274	3190
DDOST__NM_005216_CCCCATACTTTATGAGAGAC	DDOST__NM_005216	404	473	349	442
DDOST__NM_005216_CCTGTCTCTCATAAAGTATG	DDOST__NM_005216	567	145	496	129
DDOST__NM_005216_GCCTGTCTCTCATAAAGTAT	DDOST__NM_005216	1052	1024	1153	1276
DDOST__NM_005216_GCGCAAACCGCGCCAAGCAA	DDOST__NM_005216	0	0	0	0
DDOST__NM_005216_GGGCAGCAGCAACCAAAAGA	DDOST__NM_005216	141	101	204	12
DDOST__NM_005216_GTGAGCTCAAAGCCCGGGTC	DDOST__NM_005216	547	112	525	130
DDOST__NM_005216_TCATTTCTAAAATAGGGACG	DDOST__NM_005216	116	150	167	165
DDOST__NM_005216_TGTCCAGCAGCACTAAGGTG	DDOST__NM_005216	10	0	14	0
DDR1__NM_001202522_GCCCAGCAGCCGAATGATGT	DDR1__NM_001202522	1213	1305	1267	1494
DDR2__NM_001014796_ACAAATCTGGCTACAATGGG	DDR2__NM_001014796	227	175	259	265
DDR2__NM_001014796_ACGGCTGTGTCTGGCTAGGT	DDR2__NM_001014796	1266	1248	1401	1473
DDR2__NM_001014796_ATCTCTTGGCGGAACCGTCA	DDR2__NM_001014796	122	200	172	145
DDR2__NM_001014796_CACTCTCATACACACATTCA	DDR2__NM_001014796	938	1069	1065	1336
DDR2__NM_001014796_CCACAGCTGCCAAATATGGA	DDR2__NM_001014796	405	199	381	368
DDR2__NM_001014796_CCATTGTAGCCAGATTTGTC	DDR2__NM_001014796	1215	1016	1207	1309
DDR2__NM_001014796_CCGGACAAATCTGGCTACAA	DDR2__NM_001014796	442	455	534	554
DDR2__NM_001014796_CCTTTAGGAAAATGTCATAG	DDR2__NM_001014796	559	469	622	659
DDR2__NM_001014796_CTGTAATTGATCTTGTACAT	DDR2__NM_001014796	702	393	477	275
DDR2__NM_001014796_TCCTTTAGGAAAATGTCATA	DDR2__NM_001014796	108	80	91	103
DDR2__NM_001014796_TGGAATGAACCGGACAAATC	DDR2__NM_001014796	141	172	203	175
DDR2__NM_001014796_TGTATGAGAGTGGAGCTTTA	DDR2__NM_001014796	308	242	240	255
DDR2__NM_006182_AGTGCCTGGCCATGTTCCTA	DDR2__NM_006182	1387	1344	1361	1376
DDR2__NM_006182_GTGTATATGAGTCAGGGGTA	DDR2__NM_006182	133	241	136	184
DDRGK1__NM_023935_AAATTGGAGCTAAGAAACTG	DDRGK1__NM_023935	112	53	130	15
DDRGK1__NM_023935_ACAGGTGTGATTGACGACCG	DDRGK1__NM_023935	361	97	283	61
DDRGK1__NM_023935_CACAGGTGTGATTGACGACC	DDRGK1__NM_023935	87	77	42	29
DDRGK1__NM_023935_CAGGTCCTGGATGCGATTTA	DDRGK1__NM_023935	615	355	688	444
DDRGK1__NM_023935_CTGGCTGAGGGGACTATAAC	DDRGK1__NM_023935	663	577	716	854
DDRGK1__NM_023935_CTTCCACTCAGCTTCGCGCT	DDRGK1__NM_023935	444	457	523	655
DDRGK1__NM_023935_GCCCCGCTGTCGGATGAAGT	DDRGK1__NM_023935	1410	258	1649	292
DDRGK1__NM_023935_GCGCGAAGCTGAGTGGAAGA	DDRGK1__NM_023935	779	934	782	856
DDRGK1__NM_023935_GGAGGAGAAACAAGCGCGAA	DDRGK1__NM_023935	235	306	317	271
DDRGK1__NM_023935_GTTCATCTACATAACCCCAG	DDRGK1__NM_023935	20	5	43	23
DDT__NM_001084392_GCAGATTGGCAAGATAGGGA	DDT__NM_001084392	224	190	200	256
DDTL__NM_001084393_GGATAAGACCGTAGGGAACC	DDTL__NM_001084393	73	61	90	99
DDTL__NM_001084393_GGCTGGTGGATAAGACCGTA	DDTL__NM_001084393	511	538	552	689
DDTL__NM_001084393_GGGCTGGTGGATAAGACCGT	DDTL__NM_001084393	288	235	287	230
DDX1__NM_004939_AAAGCAGAGAAGTAAAGGAA	DDX1__NM_004939	227	100	309	131
DDX1__NM_004939_AAGCAGATCCTCTGTCATAT	DDX1__NM_004939	573	208	499	187
DDX1__NM_004939_AGACCATCTGACCCAATTGC	DDX1__NM_004939	1133	434	1223	497
DDX1__NM_004939_AGGAGGAGGTGATGTACTTA	DDX1__NM_004939	588	0	622	38
DDX1__NM_004939_ATTCAGCCTGGATATCAGTT	DDX1__NM_004939	441	25	445	20
DDX1__NM_004939_CATGGGTGTAGAGCTACTAA	DDX1__NM_004939	578	270	505	37
DDX1__NM_004939_GAAACAGGAAGTGGCAAAAC	DDX1__NM_004939	171	123	148	163
DDX1__NM_004939_GCATAACCCTTGGTCATGAC	DDX1__NM_004939	1205	1490	1342	1577
DDX1__NM_004939_GCTACTAAAGGATTAATGAA	DDX1__NM_004939	285	72	301	60
DDX1__NM_004939_TCAGAGTTTCATAAACTATC	DDX1__NM_004939	238	273	236	241
DDX1__NM_004939_TCATAAACTATCTGGATAAC	DDX1__NM_004939	506	306	554	571
DDX1__NM_004939_TCATGACCAAGGGTTATGCA	DDX1__NM_004939	262	47	357	6
DDX10__NM_004398_ACAGAAGCAGACCATTGGAT	DDX10__NM_004398	1102	29	1091	52
DDX10__NM_004398_AGTCCATTGCAGACGATATA	DDX10__NM_004398	263	1	234	5
DDX10__NM_004398_ATCTAAAACACGAAGCTGAG	DDX10__NM_004398	147	33	179	54
DDX10__NM_004398_CAGCGATTGAAGCTCCGCAC	DDX10__NM_004398	579	127	633	77
DDX10__NM_004398_CCTCATGCAGAACTATGAAA	DDX10__NM_004398	322	59	366	161
DDX10__NM_004398_GAAAAACCCTGAGTATGTCT	DDX10__NM_004398	611	151	704	245
DDX10__NM_004398_GAGAACCTCAAAGGTCTGAT	DDX10__NM_004398	221	17	247	1
DDX10__NM_004398_GGCTCCCGAACCCGGAGAGT	DDX10__NM_004398	640	464	600	317
DDX10__NM_004398_GTAAAGTCTGACGTTTCTTG	DDX10__NM_004398	659	569	757	892
DDX10__NM_004398_TCGGTACAGATACTGGACCT	DDX10__NM_004398	674	23	755	69
DDX10__NM_004398_TGATAGGCCAGTTCTCTCGT	DDX10__NM_004398	1195	10	1294	18
DDX10__NM_004398_TTGCAAGGTAAAGATGTACT	DDX10__NM_004398	515	15	503	4
DDX11__NM_001257144_AAGTCCTTAAGTCTTATTTG	DDX11__NM_001257144	159	132	164	81
DDX11__NM_001257144_AGCAGGGAGATGTCTAGAGAC	DDX11__NM_001257144	108	77	178	78
DDX11__NM_001257144_CATCACTCTCGTATTCGGCG	DDX11__NM_001257144	962	569	719	609
DDX11__NM_001257144_CATCTTTCTCATCATGTAAG	DDX11__NM_001257144	175	57	239	240
DDX11__NM_001257144_CTCCTCATCACTCTCGTATT	DDX11__NM_001257144	674	137	856	79
DDX11__NM_001257144_GAGGCACGACTCCTTGAAAC	DDX11__NM_001257144	538	393	676	544
DDX11__NM_001257144_GCTAGATTTATTACTGTAGT	DDX11__NM_001257144	759	880	887	1003
DDX11__NM_001257144_GGACCTGGTGGACCGACTAA	DDX11__NM_001257144	253	205	296	253
DDX11__NM_001257144_GGAGGAAGAACACATAACTA	DDX11__NM_001257144	311	85	292	21
DDX11__NM_001257144_GGCCCCACAAATAAGACTTA	DDX11__NM_001257144	355	309	470	368
DDX11__NM_001257144_TCTGTCTTCTTCCTGCGAAG	DDX11__NM_001257144	209	278	250	148
DDX11__NM_001257144_TGGATGGAATAGGGTGTGAA	DDX11__NM_001257144	22	34	46	23
DDX11__NM_001257145_CTTCACCTCATGCACAAACT	DDX11__NM_001257145	656	332	824	299
DDX17__NM_001098504_AAAGAAGGAGATTACAGTGA	DDX17__NM_001098504	305	172	264	195
DDX17__NM_001098504_AGCGTTTGCGTAAAAAAAAG	DDX17__NM_001098504	25	12	65	112
DDX17__NM_001098504_AGGATTACCAAATTTCTTCG	DDX17__NM_001098504	311	392	380	549
DDX17__NM_001098504_CCGCCTCTCTCGTCGGAGAC	DDX17__NM_001098504	146	114	245	301
DDX17__NM_001098504_CTGGTGACGACCGATGGCGG	DDX17__NM_001098504	416	633	457	412
DDX17__NM_001098504_GAAAGAAGGAGATTACAGTG	DDX17__NM_001098504	357	263	406	372
DDX17__NM_001098504_GAGAATCGGGGCTACAAAGC	DDX17__NM_001098504	302	344	310	314
DDX17__NM_001098504_GAGCAAAACACAGAGAATCG	DDX17__NM_001098504	262	245	343	288
DDX17__NM_001098504_GCCATCGGTCGTCACCAGAC	DDX17__NM_001098504	859	653	820	684
DDX17__NM_001098504_GGTCTGGTGACGACCGATGG	DDX17__NM_001098504	614	871	586	462
DDX17__NM_001098504_GTCCCAAAAGGATAGAGATC	DDX17__NM_001098504	259	127	218	177
DDX17__NM_001098504_TGGAAGGCAAACACGGGTTT	DDX17__NM_001098504	167	79	187	153
DDX17__NM_006386_AAAAAAAAGAAAAGGCGAAG	DDX17__NM_006386	56	115	47	22
DDX17__NM_006386_AAAACAGCATTACCATGAAG	DDX17__NM_006386	199	238	316	381
DDX17__NM_006386_AAAAGACAAATCACGATGGT	DDX17__NM_006386	396	365	516	609
DDX17__NM_006386_AAAGACAAATCACGATGGTT	DDX17__NM_006386	1078	1396	1114	717
DDX17__NM_006386_AAATGAACATAAATGCTCGC	DDX17__NM_006386	300	297	332	380
DDX17__NM_006386_AACGAATTGTTAGGGAGGAT	DDX17__NM_006386	643	624	660	793
DDX17__NM_006386_AAGACAAATCACGATGGTTG	DDX17__NM_006386	452	353	413	397
DDX17__NM_006386_AATGTTTCTTAAGTTGGAAC	DDX17__NM_006386	70	89	74	84
DDX17__NM_006386_ACCCCTTCCAGATCTGATAT	DDX17__NM_006386	763	876	930	1112
DDX17__NM_006386_ACTCAGAAAAACAAAACATG	DDX17__NM_006386	544	490	562	425
DDX17__NM_006386_AGATGGTCCCCAGTTAAACT	DDX17__NM_006386	736	894	709	754
DDX17__NM_006386_AGTCCACACAACGAATTGTT	DDX17__NM_006386	324	353	371	330
DDX17__NM_006386_ATCTGTAAGATCTATACTCG	DDX17__NM_006386	242	234	373	314
DDX17__NM_006386_CACACAACGAATTGTTAGGG	DDX17__NM_006386	549	398	447	373
DDX17__NM_006386_CATGGTTGAAACTCAACTTA	DDX17__NM_006386	343	214	354	173
DDX17__NM_006386_CCCCAAATTTGTCTAAGCAC	DDX17__NM_006386	514	707	564	561
DDX17__NM_006386_CTCCCTAACAATTCGTTGTG	DDX17__NM_006386	600	658	668	1036
DDX17__NM_006386_CTTCTCATCTAAAAGGTTAG	DDX17__NM_006386	57	94	71	123
DDX17__NM_006386_CTTTCAAGTCTTAATGCAAC	DDX17__NM_006386	420	513	501	684
DDX17__NM_006386_GACAAATCACGATGGTTGGG	DDX17__NM_006386	450	542	696	655
DDX17__NM_006386_GCCAGTGCTTAGACAAATTT	DDX17__NM_006386	522	592	709	1011
DDX17__NM_006386_GGTTCCAATGTATTAAGCAA	DDX17__NM_006386	14	20	24	32
DDX17__NM_006386_GTCCACACAACGAATTGTTA	DDX17__NM_006386	730	802	777	739
DDX17__NM_006386_GTGTGGACTTCTCATCTAAA	DDX17__NM_006386	192	223	238	275
DDX17__NM_006386_GTTCCAATGTATTAAGCAAT	DDX17__NM_006386	430	538	380	417
DDX17__NM_006386_TAATAGTCCCATATCAGATC	DDX17__NM_006386	267	170	238	197
DDX17__NM_006386_TATTTCTTCCCAGTTTAACT	DDX17__NM_006386	141	295	116	108
DDX17__NM_006386_TGAGGCCAAGGGAGAATGGT	DDX17__NM_006386	400	429	488	358
DDX17__NM_006386_TGTGTTCACTACAACAGGAT	DDX17__NM_006386	98	89	93	113
DDX17__NM_006386_TTCAAGAGCTGCGAGAGAGT	DDX17__NM_006386	44	44	62	36
DDX18__NM_006773_AAAAGGTGACCTAGTGTACT	DDX18__NM_006773	336	314	391	408
DDX18__NM_006773_AAATCCACTGTATTAACCAA	DDX18__NM_006773	284	100	323	189
DDX18__NM_006773_AAGAACACCAAAGGTTTGCA	DDX18__NM_006773	130	0	138	0
DDX18__NM_006773_AATGGGATCCTATGATTAAC	DDX18__NM_006773	88	3	118	36
DDX18__NM_006773_ACCTATGGCTTGATAATGGG	DDX18__NM_006773	671	28	790	45
DDX18__NM_006773_ACTCACCACGTGCATACCTA	DDX18__NM_006773	337	27	384	12
DDX18__NM_006773_AGGCATGTCTCAAGAAGCAG	DDX18__NM_006773	93	25	74	63
DDX18__NM_006773_CATACCTATGGCTTGATAAT	DDX18__NM_006773	655	0	695	0
DDX18__NM_006773_CTGTTGTGCCAGTTAATCAT	DDX18__NM_006773	524	488	619	724
DDX18__NM_006773_GAAGCACAGAAACTTGGTAA	DDX18__NM_006773	720	152	766	270
DDX18__NM_006773_GCAATGCCACCCTAATCTTA	DDX18__NM_006773	337	359	405	498
DDX18__NM_006773_GCTTCTCGATCTTCTTACGC	DDX18__NM_006773	651	622	786	940
DDX18__NM_006773_GGAACCTCAAATTGCGGCAG	DDX18__NM_006773	345	360	381	487
DDX18__NM_006773_GTCCAGGACAGGTCCGACCA	DDX18__NM_006773	843	858	977	863
DDX18__NM_006773_TAAAAGGTGACCTAGTGTAC	DDX18__NM_006773	64	20	136	116
DDX18__NM_006773_TAAGCAGTTATAACGTTTGA	DDX18__NM_006773	496	399	521	496
DDX18__NM_006773_TACGCAGGAGTTTCATCGGC	DDX18__NM_006773	942	452	858	489
DDX18__NM_006773_TCAAGCCATAGGTATGCACG	DDX18__NM_006773	223	10	350	1
DDX18__NM_006773_TTCTCCATTGGTTAATACAG	DDX18__NM_006773	0	0	0	0
DDX18__NM_006773_TTGTTTAATTCAAGGTGGTT	DDX18__NM_006773	0	0	7	4
DDX19A__NM_018332_AAGCGCCAGCTCATATGTTG	DDX19A__NM_018332	992	377	883	477
DDX19A__NM_018332_ACTGGGCAATCAGATTCTGT	DDX19A__NM_018332	1111	874	1113	1009
DDX19A__NM_018332_CACCGCCAGGGCCCACGAGT	DDX19A__NM_018332	812	634	917	777
DDX19A__NM_018332_CTCTTCCTTGATCTGCAAAT	DDX19A__NM_018332	180	304	279	266
DDX19A__NM_018332_CTGATTGCCCAGTCTCAGTC	DDX19A__NM_018332	331	421	448	593
DDX19A__NM_018332_GACTGGGCAATCAGATTCTG	DDX19A__NM_018332	714	464	896	632
DDX19A__NM_018332_GATGACCAATTTGCAGATCA	DDX19A__NM_018332	686	934	809	698
DDX19A__NM_018332_GCAGGAAGCGGCTGTCAAGT	DDX19A__NM_018332	1427	1435	1383	1270
DDX19A__NM_018332_GGACTATGGCCACCGACTCG	DDX19A__NM_018332	1397	1347	1469	1508
DDX19A__NM_018332_GGCAATCAGATTCTGTGGGC	DDX19A__NM_018332	799	758	769	942
DDX19A__NM_018332_TGCGTTCTCTTGTATCTTGG	DDX19A__NM_018332	195	255	221	341
DDX19A__NM_018332_TGGCTCCACTCGGCTGAGCA	DDX19A__NM_018332	2	0	5	46
DDX19B__NM_001014449_AGGAAAAGTGATTGAACAAA	DDX19B__NM_001014449	200	213	213	250
DDX19B__NM_001014449_AGGTTCTACTTGGCTAAGCA	DDX19B__NM_001014449	32	43	40	29
DDX19B__NM_001014449_AGTTTGACACGTTTGGGTC	DDX19B__NM_001014449	188	171	336	403
DDX19B__NM_001014449_ATTGGGCAATTAAGTTCTGT	DDX19B__NM_001014449	384	359	422	325
DDX19B__NM_001014449_CGCTTCAGTTTGATAACGTT	DDX19B__NM_001014449	1059	1189	1136	1322
DDX19B__NM_001014449_GAGGGCGAGCTCATACGTTG	DDX19B__NM_001014449	549	508	489	414
DDX19B__NM_001014449_GATTGGGCAATTAAGTTCTG	DDX19B__NM_001014449	380	369	515	421
DDX19B__NM_001014449_GCTTCAGTTTGATAACGTTT	DDX19B__NM_001014449	1758	2460	2015	2650
DDX19B__NM_001014449_GGAGGGCGAGCTCATACGTT	DDX19B__NM_001014449	731	531	679	558
DDX19B__NM_001014449_TGGAGGGCGAGCTCATACGT	DDX19B__NM_001014449	259	198	189	78
DDX19B__NM_001014449_TTAATTGCCCAATCTCAGTC	DDX19B__NM_001014449	167	133	206	172
DDX19B__NM_001014449_TTGGGGAGAGACATAGACAC	DDX19B__NM_001014449	225	138	241	214
DDX19B__NM_001014451_GACCATGGCCACTGACTCAT	DDX19B__NM_001014451	3286	3110	3772	3720
DDX19B__NM_001014451_GGACCATGGCCACTGACTCA	DDX19B__NM_001014451	995	1003	1049	986
DDX20__NM_007204_AACAAGAGAGTCCAAAGCTA	DDX20__NM_007204	37	0	38	0
DDX20__NM_007204_AAGAGAGTCCAAAGCTATGG	DDX20__NM_007204	292	0	372	17
DDX20__NM_007204_AGCAGTGACTCGAAGTCGGC	DDX20__NM_007204	441	34	451	20
DDX20__NM_007204_AGTGTCATGTCTTTATTGGA	DDX20__NM_007204	1034	25	1258	34
DDX20__NM_007204_CAACAGCACATCCCCCGTGC	DDX20__NM_007204	960	215	890	48
DDX20__NM_007204_CACTGCTGCTAAGGCTCCCG	DDX20__NM_007204	730	535	776	586
DDX20__NM_007204_CAGCTCAAGGCCATCCCGTT	DDX20__NM_007204	1593	2035	1765	2341
DDX20__NM_007204_GAACTTGACTACTTGAACCC	DDX20__NM_007204	254	8	273	1
DDX20__NM_007204_GCCTTGAGCTGCACCGGCGA	DDX20__NM_007204	1064	883	1173	1139
DDX20__NM_007204_GGCGGCGGCATTTGAAGCCT	DDX20__NM_007204	571	735	685	650
DDX20__NM_007204_TGTGTTCTCCACCATAGCTT	DDX20__NM_007204	0	0	0	0
DDX20__NM_007204_TTCAAGCTAAATCTGGCACC	DDX20__NM_007204	53	1	132	0
DDX21__NM_001256910_ACATTCCATCATGTTTACAG	DDX21__NM_001256910	381	7	474	2
DDX21__NM_001256910_ATAGTTGAACGCATGAGGAA	DDX21__NM_001256910	132	0	167	11
DDX21__NM_001256910_ATCAAAGACCACATACAGAA	DDX21__NM_001256910	374	15	447	54
DDX21__NM_001256910_CATGAAGTTTCTTCAATCAAA	DDX21__NM_001256910	842	243	1027	382
DDX21__NM_001256910_CCTTCCCGCTGTAAACATGA	DDX21__NM_001256910	936	14	1086	29
DDX21__NM_001256910_CGGGAAGGACTTAATTGCAC	DDX21__NM_001256910	816	2	856	28
DDX21__NM_001256910_CTGGGGTTACAGTCCGGTTC	DDX21__NM_001256910	644	181	583	229
DDX21__NM_001256910_GCTTCACTGGGGTTACAGTC	DDX21__NM_001256910	238	116	347	69
DDX21__NM_001256910_TAGTTGAACGCATGAGGAAT	DDX21__NM_001256910	161	2	171	3
DDX21__NM_001256910_TATGGTGGAACTCCCTATGG	DDX21__NM_001256910	219	3	173	4
DDX21__NM_001256910_TCTTGCACCTACAAGAGAGT	DDX21__NM_001256910	110	1	144	10
DDX21__NM_001256910_TCTTTGCTTGTATAGGAAAT	DDX21__NM_001256910	219	64	225	28
DDX21__NM_004728_AAATATCTAGTCGGGATTCA	DDX21__NM_004728	153	172	129	78
DDX21__NM_004728_AATCCTGGAACTACTATGCT	DDX21__NM_004728	990	778	992	1068
DDX21__NM_004728_ACACTGTCACCTAATACCCA	DDX21__NM_004728	762	645	793	537
DDX21__NM_004728_ACTGCGAAAGCAAACCGAGG	DDX21__NM_004728	137	81	124	59
DDX21__NM_004728_AGAGAAGAACAAGTATGATT	DDX21__NM_004728	269	187	271	152
DDX21__NM_004728_AGCTCAGCTCCTAATCCTTG	DDX21__NM_004728	151	72	157	75
DDX21__NM_004728_AGTACATGGTGATCAAATAA	DDX21__NM_004728	108	162	110	81
DDX21__NM_004728_AGTCTCAGTAGACAAATAAT	DDX21__NM_004728	153	130	194	175
DDX21__NM_004728_ATAGCACAGGAGCTTAAGAG	DDX21__NM_004728	454	300	509	565
DDX21__NM_004728_ATTAAAGCATGCCCAGTATT	DDX21__NM_004728	300	465	410	377
DDX21__NM_004728_ATTCCAAACCAGCGTCACTA	DDX21__NM_004728	300	207	408	423
DDX21__NM_004728_CCCATCAAGCCATGGGTATT	DDX21__NM_004728	940	835	1023	1078
DDX21__NM_004728_CCTACTTACAAATAAAGCTA	DDX21__NM_004728	243	283	304	284

DDX21__NM_004728_CTATCAGAGCTCCTAGTACA	DDX21__NM_004728	922	845	1209	1234
DDX21__NM_004728_CTTGGCAAGTGGTCAAAAGG	DDX21__NM_004728	255	198	192	166
DDX21__NM_004728_GAGGAAACCTTAACTTCCAA	DDX21__NM_004728	137	205	245	294
DDX21__NM_004728_TAGTACATGGTGATCAAATA	DDX21__NM_004728	17	33	35	43
DDX21__NM_004728_TCAGACACCGCAATGAAAAA	DDX21__NM_004728	116	118	124	155
DDX21__NM_004728_TGAGAAATCAGGCAGTTATC	DDX21__NM_004728	257	270	446	567
DDX21__NM_004728_TGTACTCCACTTCTTCCTAT	DDX21__NM_004728	481	311	571	349
DDX21__NM_004728_TTATTTGATCACCATGTACT	DDX21__NM_004728	116	53	114	162
DDX21__NM_004728_TTCAGGGAAGGACACTATTA	DDX21__NM_004728	5	0	6	9
DDX21__NM_004728_TTCATTATTTGGATAGGCAT	DDX21__NM_004728	22	8	14	23
DDX23__NM_004818_AAAGGAGAGGACGACGCTGT	DDX23__NM_004818	864	703	795	424
DDX23__NM_004818_AGGAGGAAAGGAAGCGATCA	DDX23__NM_004818	217	107	267	66
DDX23__NM_004818_ATAGAAAGCGGCATCGTTCA	DDX23__NM_004818	260	248	329	200
DDX23__NM_004818_CAGAGAGCGGGATAGAGACC	DDX23__NM_004818	341	226	295	181
DDX23__NM_004818_CATCAGCTCATGCCAGGCTA	DDX23__NM_004818	272	252	278	225
DDX23__NM_004818_CGTTCAAGGGATAGACGTCG	DDX23__NM_004818	510	373	605	540
DDX23__NM_004818_CTCTTGGGATATGGGGGCTT	DDX23__NM_004818	259	245	187	102
DDX23__NM_004818_GAAGGACCGAGATCGAGACA	DDX23__NM_004818	462	353	433	190
DDX23__NM_004818_GAGGAAAAGACTTTAAATCT	DDX23__NM_004818	427	163	419	142
DDX23__NM_004818_GGAGCGGGATCGGAATAAGA	DDX23__NM_004818	410	354	393	330
DDX23__NM_004818_GGATGGGCACAGACGGGACA	DDX23__NM_004818	298	342	311	362
DDX23__NM_004818_TAGAAAGCGGCATCGTTCAA	DDX23__NM_004818	431	271	404	182
DDX23__NM_004818_TCAAGGGATAGACGTCGAGG	DDX23__NM_004818	135	57	129	27
DDX23__NM_004818_TCTCTATCCCGCTCTCTGTC	DDX23__NM_004818	1578	793	1552	914
DDX23__NM_004818_TGCCGCTTTCTATCTTTAGA	DDX23__NM_004818	574	650	560	872
DDX24__NM_020414_CATGGTTTGTGATGATCCGG	DDX24__NM_020414	362	429	421	414
DDX24__NM_020414_GAACCGGCCTGGGAACAAAC	DDX24__NM_020414	127	75	151	4
DDX24__NM_020414_GATGTCCAGTTTGTCACGGA	DDX24__NM_020414	784	268	1038	449
DDX24__NM_020414_GCCAAGGTCAGGGCTTGGAT	DDX24__NM_020414	810	602	954	681
DDX24__NM_020414_GCCCAGTGATATTGCAGCCG	DDX24__NM_020414	1267	836	1618	1324
DDX24__NM_020414_GCCTCGGCTGCAATATCACT	DDX24__NM_020414	1356	1367	1539	829
DDX24__NM_020414_GGAATTGACAGATTACCAGT	DDX24__NM_020414	392	133	465	218
DDX24__NM_020414_GGAGCTGAGACTAGATCACC	DDX24__NM_020414	393	277	397	221
DDX24__NM_020414_GGTGCTTCGGTGTTACTTGG	DDX24__NM_020414	216	77	258	142
DDX24__NM_020414_GGTGGTGCTTCGGTGTTACT	DDX24__NM_020414	132	163	162	111
DDX24__NM_020414_TCCAGTTTGTCACGGATGGC	DDX24__NM_020414	369	736	338	499
DDX24__NM_020414_TTCAAATTCTTTCTGGGTAC	DDX24__NM_020414	22	18	31	48
DDX25__NM_013264_ACGGTGACTGGATTCTACTA	DDX25__NM_013264	661	853	784	840
DDX25__NM_013264_ACTAAAAGGAATCTATGCAA	DDX25__NM_013264	480	502	503	531
DDX25__NM_013264_AGAAAGGAAGAGTTACTAAA	DDX25__NM_013264	76	121	76	106
DDX25__NM_013264_AGCCATGGCGTCGTTACTGT	DDX25__NM_013264	1151	1261	1171	1453
DDX25__NM_013264_ATGATGAAGAAGATGTAGGT	DDX25__NM_013264	358	232	350	285
DDX25__NM_013264_CCTAGCTCCTACTTATGAAT	DDX25__NM_013264	349	293	462	409
DDX25__NM_013264_GATGGCTCTCCCTATGATGC	DDX25__NM_013264	256	111	192	116
DDX25__NM_013264_GCCATGGCGTCGTTACTGTG	DDX25__NM_013264	620	316	615	689
DDX25__NM_013264_GTTAAGCAGAGTTAATGCCT	DDX25__NM_013264	875	666	963	986
DDX25__NM_013264_TAAAACTTCCACACGGTGAC	DDX25__NM_013264	199	296	249	282
DDX25__NM_013264_TCCCCACAGTAACGACGCCA	DDX25__NM_013264	259	217	315	230
DDX25__NM_013264_TGGATTCTACTAAGGATTGA	DDX25__NM_013264	0	0	0	0
DDX26B__NM_182540_AACAAGTTAACAAGTACTGC	DDX26B__NM_182540	167	260	229	200
DDX26B__NM_182540_ACACAGGAGAACCTCACAAC	DDX26B__NM_182540	724	896	980	1101
DDX26B__NM_182540_AGTGGAAAGTACAATGAACT	DDX26B__NM_182540	325	422	408	453
DDX26B__NM_182540_CTCCAGGCAAACGCAACACC	DDX26B__NM_182540	387	457	404	509
DDX26B__NM_182540_CTGTGTAGATTGTGAGCCAA	DDX26B__NM_182540	489	768	533	584
DDX26B__NM_182540_GCAGTACGGGGGTTCGTCGT	DDX26B__NM_182540	3943	4738	4330	5096
DDX26B__NM_182540_GGATCAAAGGTTATTTGCCC	DDX26B__NM_182540	372	394	433	552
DDX26B__NM_182540_GTACGACCTAACTCTAAAAC	DDX26B__NM_182540	165	223	169	189
DDX26B__NM_182540_TAGAGGCGGACGTGTCTATG	DDX26B__NM_182540	1511	251	1655	290
DDX26B__NM_182540_TCCAGGCAAACGCAACACCA	DDX26B__NM_182540	570	574	584	703
DDX26B__NM_182540_TCTATGAGGAACAGCAGGAT	DDX26B__NM_182540	79	89	144	109
DDX26B__NM_182540_TGGACTGACTACTCTCGGTC	DDX26B__NM_182540	55	66	100	122
DDX27__NM_017895_AGGAGGCTGCGAAAAGTTAA	DDX27__NM_017895	253	316	278	284
DDX27__NM_017895_CACCTCGTCATCCTCGCCTA	DDX27__NM_017895	2090	855	2209	713
DDX27__NM_017895_CAGGGTTGAAATCAGCACTG	DDX27__NM_017895	354	126	329	124
DDX27__NM_017895_CTATGGTTCCGATTAAGCCG	DDX27__NM_017895	295	341	258	188
DDX27__NM_017895_CTCGGCTTAATCGGAACCAT	DDX27__NM_017895	626	340	702	342
DDX27__NM_017895_CTTAATCGGAACCATAGGCG	DDX27__NM_017895	209	67	222	97
DDX27__NM_017895_CTTGCGGACCTCGGCTTAAT	DDX27__NM_017895	951	681	1161	664
DDX27__NM_017895_GAGAAGGAGGGGACGTACGA	DDX27__NM_017895	382	143	378	71
DDX27__NM_017895_GAGGAGGCTGCGAAAAGTTA	DDX27__NM_017895	360	353	464	435
DDX27__NM_017895_GGCTGCGAAAAGTTAAGGGC	DDX27__NM_017895	371	319	374	361
DDX27__NM_017895_TCTCAGTGAAAACGAAATCA	DDX27__NM_017895	59	36	61	12
DDX27__NM_017895_TTCTCAGTGAAAACGAAATC	DDX27__NM_017895	0	0	0	0
DDX28__NM_018380_AAACATGGCTCTAACGCGGC	DDX28__NM_018380	20	18	30	34
DDX28__NM_018380_ACCACCGGCAGGGGTTCGTC	DDX28__NM_018380	731	274	796	297
DDX28__NM_018380_AGGCCGGTGCTGGTTCGACC	DDX28__NM_018380	667	500	695	701
DDX28__NM_018380_CAGGAGCAACCGAGTCACGA	DDX28__NM_018380	662	142	756	158
DDX28__NM_018380_CCAGGAGCAACCGAGTCACG	DDX28__NM_018380	590	188	560	186
DDX28__NM_018380_CGCGCCTCACACTGGGCCGT	DDX28__NM_018380	5142	4035	5161	3756
DDX28__NM_018380_CTAGCGGCGCGCGCTCCCAA	DDX28__NM_018380	374	79	338	121
DDX28__NM_018380_GACCGCTGCTGGTTTCGGCG	DDX28__NM_018380	829	688	952	760
DDX28__NM_018380_GCCACTGGGATGCGCACCAC	DDX28__NM_018380	1367	1160	1397	1125
DDX28__NM_018380_GCGGCCGGAGTTGAACCAGC	DDX28__NM_018380	676	315	693	334
DDX28__NM_018380_GGTCCGGGTCGAACCAGCAC	DDX28__NM_018380	1034	974	1088	1026
DDX28__NM_018380_GGTTGCTCCTGGCGCCGCGA	DDX28__NM_018380	373	330	347	466
DDX31__NM_022779_ATTCGGCCGAGAGTTCACGC	DDX31__NM_022779	573	366	598	453
DDX31__NM_022779_CGCCGGATCTCGCTTCTCAG	DDX31__NM_022779	2296	2168	2561	2628
DDX31__NM_022779_CTGTACGGACTCGTCCCGAG	DDX31__NM_022779	832	802	884	702
DDX31__NM_022779_CTGTGGAGTTCTGGAATGTC	DDX31__NM_022779	424	262	466	546
DDX31__NM_022779_GGTCCTCGCGCGTCCCACGT	DDX31__NM_022779	769	781	917	798
DDX31__NM_022779_GTCCCCAGCCCAGGCGAGT	DDX31__NM_022779	450	481	430	297
DDX31__NM_022779_GTCGAAGAGCGAACCGTCGG	DDX31__NM_022779	645	661	744	644
DDX31__NM_022779_GTGAGGCTCCCCCAGCGAAA	DDX31__NM_022779	290	175	230	100
DDX31__NM_022779_GTTGTCGAAGAGCGAACCGT	DDX31__NM_022779	567	695	535	482
DDX31__NM_022779_GTTTCGTTCCTCCGTTTCGC	DDX31__NM_022779	268	603	216	394
DDX31__NM_022779_TGCCTCTGAGAAGCGAGATC	DDX31__NM_022779	44	62	43	46
DDX31__NM_022779_TTTCGTTCCTCCGTTTCGCT	DDX31__NM_022779	284	406	286	374
DDX39A__NM_005804_ACTGTCCCCATGTCGTGGTG	DDX39A__NM_005804	648	421	716	719
DDX39A__NM_005804_CATGACCAGGACCGTCACCT	DDX39A__NM_005804	1717	1346	1940	1670
DDX39A__NM_005804_CCTGGTCATGTGCCACACGA	DDX39A__NM_005804	616	324	732	541
DDX39A__NM_005804_CGGGGTCCCCACCACGACAT	DDX39A__NM_005804	355	233	379	213
DDX39A__NM_005804_GAAGAACTGTCCCCATGTCG	DDX39A__NM_005804	780	518	753	541
DDX39A__NM_005804_GCCGCATCCTGGCGCTCGTG	DDX39A__NM_005804	1243	1253	1484	1585
DDX39A__NM_005804_GGATGTGCTGGAGTTTAACC	DDX39A__NM_005804	621	362	719	425
DDX39A__NM_005804_GGGGTCCCCACCACGACATG	DDX39A__NM_005804	132	91	157	68
DDX39A__NM_005804_TAGGTGTCTGTGTTCTTCGG	DDX39A__NM_005804	325	147	353	270
DDX39A__NM_005804_TCCCTTGATGTCTTTCTTAG	DDX39A__NM_005804	448	341	630	663
DDX39A__NM_005804_TCCTGGCGCTCGTGCGGAAT	DDX39A__NM_005804	559	242	655	345
DDX39A__NM_005804_TGCCAGGCCAAGTCCGGGAT	DDX39A__NM_005804	872	682	1008	890
DDX3X__NM_001193416_ATTAGCACCAACGAGAGAGT	DDX3X__NM_001193416	48	1	38	0
DDX3X__NM_001193416_CGGAGTGATTACGATGGCAT	DDX3X__NM_001193416	813	810	808	863
DDX3X__NM_001193416_GATCTCGTAGTGATTCAAGA	DDX3X__NM_001193416	355	220	400	248
DDX3X__NM_001193416_GCTATATTCCTCCTCATTTA	DDX3X__NM_001193416	800	113	989	64
DDX3X__NM_001193416_GCTTCTCGGTTCCTTAAATG	DDX3X__NM_001193416	238	43	333	41
DDX3X__NM_001193416_GGCTTTGGCAAATTTGAACG	DDX3X__NM_001193416	232	345	338	495
DDX3X__NM_001193416_GTGGAGTTCTAGCAAAGATA	DDX3X__NM_001193416	13	11	31	0
DDX3X__NM_001193416_GTGGCAGTGGAAAATGCGCT	DDX3X__NM_001193416	240	252	218	146
DDX3X__NM_001193416_TCTACGATAAAGACAGTTCA	DDX3X__NM_001193416	192	41	173	61
DDX3X__NM_001193416_TCTCGGTTCCTTAAATGAGG	DDX3X__NM_001193416	228	18	124	6
DDX3X__NM_001193416_TCTCGTTGGTGCTAATACCA	DDX3X__NM_001193416	710	31	770	29
DDX3X__NM_001193416_TCTTCAGATAATCAGAGTGG	DDX3X__NM_001193416	59	10	83	30
DDX3X__NM_001193417_ATCTAGAGTTCGTCCTTGCG	DDX3X__NM_001193417	727	206	763	202
DDX3X__NM_001193417_GTTCGTCCTTGCGTGGTTTA	DDX3X__NM_001193417	179	18	165	26
DDX3X__NM_001193417_TGTACTGCCAACTCTCTCGT	DDX3X__NM_001193417	733	6	703	43
DDX3Y__NM_001122665_AACGGAGTGGACATAGTCGT	DDX3Y__NM_001122665	980	1209	1239	1505
DDX3Y__NM_001122665_AGGAACAGAGAAGCATCTAA	DDX3Y__NM_001122665	229	392	231	363
DDX3Y__NM_001122665_ATATGATGATATACCAGTAG	DDX3Y__NM_001122665	322	406	389	489
DDX3Y__NM_001122665_ATTGGCAATCGTGAAAGACC	DDX3Y__NM_001122665	268	220	370	434
DDX3Y__NM_001122665_CCTGGTTATTTCAGTGAACG	DDX3Y__NM_001122665	311	308	310	451
DDX3Y__NM_001122665_CGGAGTGACTATGATGGTAT	DDX3Y__NM_001122665	777	1191	1028	1041
DDX3Y__NM_001122665_GCAGTTTAGCGATATTGACA	DDX3Y__NM_001122665	287	514	287	531
DDX3Y__NM_001122665_TCTCTGTTCCTTAAGTGAGG	DDX3Y__NM_001122665	332	325	369	370
DDX3Y__NM_001122665_TTACTGCCGGTTGCCTCTAC	DDX3Y__NM_001122665	707	1318	714	1062
DDX3Y__NM_001122665_TTCAGGGATGAGTCATGTGG	DDX3Y__NM_001122665	5	0	2	7
DDX3Y__NM_001122665_TTTGGCAGATTTGAACGGAG	DDX3Y__NM_001122665	193	384	261	285
DDX3Y__NM_001122665_TTTGGGTCTCGAGATTCTAG	DDX3Y__NM_001122665	65	68	62	91
DDX4__NM_001142549_ACTGCGAAGATAATCCAACA	DDX4__NM_001142549	154	280	176	161
DDX4__NM_001142549_AGACCAGTATTAAGTGGCAC	DDX4__NM_001142549	394	393	377	309
DDX4__NM_001142549_ATGAAATGATCTCTTCGAGA	DDX4__NM_001142549	118	103	132	152
DDX4__NM_001142549_CCAATCTTCATCTCCCATGG	DDX4__NM_001142549	680	475	739	575
DDX4__NM_001142549_CTTCTCAAATATGGGAACAT	DDX4__NM_001142549	104	134	141	109
DDX4__NM_001142549_GAACATAGGAAGACATATGA	DDX4__NM_001142549	341	498	373	288
DDX4__NM_001142549_GAAGAAGTAATAACAGGCTC	DDX4__NM_001142549	20	32	12	6
DDX4__NM_001142549_GGAGAAAGTAGTGATACTCA	DDX4__NM_001142549	247	193	237	272
DDX4__NM_001142549_GTGCGCTGCATACATTCGTC	DDX4__NM_001142549	542	544	620	624
DDX4__NM_001142549_TCACCTGTGCCACTTAATAC	DDX4__NM_001142549	636	632	758	815
DDX4__NM_001142549_TGCGCTGCATACATTCGTCT	DDX4__NM_001142549	986	944	1051	1184
DDX4__NM_001142549_TTTGAAGATGGTGATAGCTC	DDX4__NM_001142549	0	0	0	0
DDX4__NM_001166533_CCTCTTCCACCTCTTCTGTA	DDX4__NM_001166533	1541	1496	1713	1429
DDX4__NM_001166533_GCTTCAGGGCCATACAGAAG	DDX4__NM_001166533	157	156	161	150
DDX4__NM_001166534_CAAATACGACACTATTCTTG	DDX4__NM_001166534	1650	1709	1818	1415
DDX4__NM_001166534_GCTTGCGCTCAAACAGGGTC	DDX4__NM_001166534	120	180	102	148
DDX4__NM_001166534_TGATGGCTTGCGCTCAAACA	DDX4__NM_001166534	148	170	175	221
DDX4__NM_001166534_TGCATCATATGAGCCAAAAT	DDX4__NM_001166534	364	438	364	482
DDX4__NM_001166534_TGTCTGATAATGTGCAAAGA	DDX4__NM_001166534	203	195	281	205
DDX4__NM_001166534_TTGATGGCTTGCGCTCAAAC	DDX4__NM_001166534	83	108	114	108
DDX41__NM_016222_ACGTGCCCTATGTGCCGTTA	DDX41__NM_016222	1184	363	1277	250
DDX41__NM_016222_AGGACAGCGGTAGTGAACCC	DDX41__NM_016222	131	93	149	114
DDX41__NM_016222_ATCCTGGAGAGTGTTGCCGA	DDX41__NM_016222	568	79	651	190
DDX41__NM_016222_CAGCCTTCTCTTTAAGGTGC	DDX41__NM_016222	340	458	330	448
DDX41__NM_016222_CCCTGGATCTGAATGGGTGT	DDX41__NM_016222	1359	6	1434	35
DDX41__NM_016222_CTGCTGTATGTGTAGACTGG	DDX41__NM_016222	693	916	786	746
DDX41__NM_016222_GATCCTGGAGAGTGTTGCCG	DDX41__NM_016222	621	158	716	287
DDX41__NM_016222_GATGAGGACGACATCCCGCT	DDX41__NM_016222	932	297	1253	414
DDX41__NM_016222_GGAGTCGGAACCCGAACGGA	DDX41__NM_016222	100	50	181	92
DDX41__NM_016222_TCAGTGAAGGAGATGGCTAA	DDX41__NM_016222	295	8	433	5
DDX41__NM_016222_TCCTCCGGCAGGCACCTCGT	DDX41__NM_016222	899	470	958	608
DDX41__NM_016222_TCGGAACCCGAACGGAAGGT	DDX41__NM_016222	145	70	174	97
DDX41__NM_016222_TGGAGGAGTCGGAACCCGAA	DDX41__NM_016222	72	95	81	59
DDX42__NM_007372_AATCAACATGGGCCAAATGA	DDX42__NM_007372	111	0	144	14
DDX42__NM_007372_ACCACCTTTATTCCAGTTCA	DDX42__NM_007372	1465	1205	1814	1480
DDX42__NM_007372_ACCTCTGAATGATCAATGGG	DDX42__NM_007372	169	15	251	26
DDX42__NM_007372_ACCTGGCACTGTATTGGAGT	DDX42__NM_007372	1327	26	1303	100
DDX42__NM_007372_CCTGGCACTAAGCGAGGATT	DDX42__NM_007372	1070	643	1164	723
DDX42__NM_007372_CGGAGATCTATTAACTGCTG	DDX42__NM_007372	284	247	295	222
DDX42__NM_007372_CTACAAAATTGGATCTAAGC	DDX42__NM_007372	218	229	309	157
DDX42__NM_007372_GCCCATGTTGATTCATATAA	DDX42__NM_007372	570	759	908	769
DDX42__NM_007372_TCCCCCCATTGATCATTCAG	DDX42__NM_007372	799	214	818	281
DDX42__NM_007372_TCTCTACCACTTAATGCCAC	DDX42__NM_007372	327	39	375	29
DDX42__NM_007372_TGTATTCAGATTTCCGAATC	DDX42__NM_007372	140	31	146	50
DDX42__NM_007372_TTAAGTGGTAGAGACATGAT	DDX42__NM_007372	183	34	228	7
DDX43__NM_018665_AGATCGGCCATTGATAGATT	DDX43__NM_018665	678	906	717	753
DDX43__NM_018665_AGCTCCCAAGGCCTCTACGT	DDX43__NM_018665	1143	872	1007	579
DDX43__NM_018665_ATTTGATCCCAATCTATCAA	DDX43__NM_018665	67	9	78	67
DDX43__NM_018665_CAAAAGTAGAAGCAGATAGT	DDX43__NM_018665	618	465	570	511
DDX43__NM_018665_CGATTACCGCGCCAACAAAG	DDX43__NM_018665	0	0	0	0
DDX43__NM_018665_CTACGTGGGTCGTTGCTAGT	DDX43__NM_018665	1158	894	1162	1122
DDX43__NM_018665_GAGATCGGCCATTGATAGAT	DDX43__NM_018665	503	676	550	669
DDX43__NM_018665_GAGCTCCCAAGGCCTCTACG	DDX43__NM_018665	534	794	540	576
DDX43__NM_018665_GCGGAGGAGTTGAATCGAAC	DDX43__NM_018665	484	774	551	676
DDX43__NM_018665_GGTCCTGAGGGATATAGTGT	DDX43__NM_018665	460	391	473	394
DDX43__NM_018665_TGGGATCAAATTAGAGAGGA	DDX43__NM_018665	21	12	28	58
DDX43__NM_018665_TTCCTCGTGACCAGCGGCCA	DDX43__NM_018665	153	193	158	194
DDX46__NM_014829_AATACAAAAATTAGTCGGCA	DDX46__NM_014829	589	696	529	573
DDX46__NM_014829_AGAGGTAAATGTGTTTCGAT	DDX46__NM_014829	379	370	488	467
DDX46__NM_014829_AGAGTCATGTAACATCAAGG	DDX46__NM_014829	125	246	184	76
DDX46__NM_014829_AGATCCATTAGATGCTTACA	DDX46__NM_014829	218	3	257	0
DDX46__NM_014829_AGGGAAAAAGTGGAGTTTAG	DDX46__NM_014829	262	56	271	65
DDX46__NM_014829_AGTCCCACATCGATGATAAT	DDX46__NM_014829	259	140	276	300
DDX46__NM_014829_ATAAAGTACACAAGTCGTTA	DDX46__NM_014829	43	30	77	65
DDX46__NM_014829_ATGAGAAGTGTAAAAGGTGG	DDX46__NM_014829	165	78	120	42
DDX46__NM_014829_ATGTCCGGATAATATTCTAG	DDX46__NM_014829	613	488	740	519
DDX46__NM_014829_ATTCAGTGGCTGAAGTGATA	DDX46__NM_014829	94	53	140	82
DDX46__NM_014829_CACAATTTCATTGAAAGTAC	DDX46__NM_014829	205	390	146	158
DDX46__NM_014829_CAGTCATCCTTTGGTATGAG	DDX46__NM_014829	645	811	874	786
DDX46__NM_014829_CAGTCCCCCTCTCATACCAA	DDX46__NM_014829	2609	2175	2856	2172
DDX46__NM_014829_CGAAAACGATCGGCATCCCG	DDX46__NM_014829	2571	1447	2915	1462
DDX46__NM_014829_CTGAAAAAGTAAGATATACC	DDX46__NM_014829	409	679	418	609
DDX46__NM_014829_GAAGTAGAGATAGAGATAGG	DDX46__NM_014829	71	0	81	2
DDX46__NM_014829_GACTCTGGAACAATATGAAC	DDX46__NM_014829	308	258	311	393
DDX46__NM_014829_GACTGGAGGATCGGGATCGC	DDX46__NM_014829	724	403	854	547
DDX46__NM_014829_GAGAAGTGTAAAAGGTGGTG	DDX46__NM_014829	112	113	152	118
DDX46__NM_014829_GCGAGAAGAGCAACGTAAAA	DDX46__NM_014829	334	278	348	269
DDX46__NM_014829_TAAAGTACACAAGTCGTTAA	DDX46__NM_014829	79	84	66	100
DDX46__NM_014829_TAGCCACTATCGAAAACGAT	DDX46__NM_014829	479	319	533	319
DDX46__NM_014829_TCTTCCATGTAAGCATCTAA	DDX46__NM_014829	131	42	174	29
DDX46__NM_014829_TGAGCTGATGGAGAATGACC	DDX46__NM_014829	216	24	199	12
DDX46__NM_014829_TGCTCATCTTTATCACCTAA	DDX46__NM_014829	249	205	320	521
DDX46__NM_014829_TTCAAGCCTCTTATATGAAA	DDX46__NM_014829	38	83	39	60
DDX47__NM_016355_AAAGGCCAGCTCCCGAGTCG	DDX47__NM_016355	622	13	616	35
DDX47__NM_016355_ACTAGGGCAAACAAACGCTG	DDX47__NM_016355	311	12	316	4
DDX47__NM_016355_ATCGGCTGGGACGCTTCGGT	DDX47__NM_016355	1958	1629	2170	1511
DDX47__NM_016355_CACAATCGGCTGGGACGCTT	DDX47__NM_016355	550	452	665	471
DDX47__NM_016355_CCAAAGGAATAGCTTCAATC	DDX47__NM_016355	443	326	571	353
DDX47__NM_016355_CCAAGCTTCTTTACAATTAC	DDX47__NM_016355	252	494	368	362
DDX47__NM_016355_CCCTAGTTCTTACCCCGACT	DDX47__NM_016355	1166	63	1305	101
DDX47__NM_016355_CCGAGTCGGGGTAAGAACTA	DDX47__NM_016355	172	0	228	6
DDX47__NM_016355_CCTTGCTCATGACATGAGTA	DDX47__NM_016355	277	497	394	455
DDX47__NM_016355_CTAGGGCAAACAAACGCTGC	DDX47__NM_016355	441	27	449	16
DDX47__NM_016355_CTCCAGCAGTGCGTTTAGAA	DDX47__NM_016355	145	135	125	213
DDX47__NM_016355_GCCCATTCTAAACGCACTGC	DDX47__NM_016355	93	199	113	117
DDX47__NM_016355_TAGGGCAAACAAACGCTGCG	DDX47__NM_016355	560	92	543	57
DDX47__NM_016355_TCCAGCAGTGCGTTTAGAAT	DDX47__NM_016355	348	39	595	52
DDX47__NM_016355_TTCTTTACAATTACAGGTCA	DDX47__NM_016355	0	0	3	26
DDX49__NM_019070_AAAGACTGCATCATCGTCGG	DDX49__NM_019070	599	0	634	10
DDX49__NM_019070_ACGAGGCAGAAGATGCCATA	DDX49__NM_019070	637	14	625	15
DDX49__NM_019070_AGCCCAAGCAGTCTCGACCT	DDX49__NM_019070	891	1	1022	3
DDX49__NM_019070_CAGCCGAGCTGCACGGGCGT	DDX49__NM_019070	152	0	144	16
DDX49__NM_019070_CCACGCTGACCGACACACTC	DDX49__NM_019070	1009	153	1087	174
DDX49__NM_019070_CCTGGACATCCCTACGGTAC	DDX49__NM_019070	621	7	571	23
DDX49__NM_019070_CGTGGAACAATGTCGGCAGC	DDX49__NM_019070	486	578	601	736
DDX49__NM_019070_CGTGGCTCGTGGAACAATGT	DDX49__NM_019070	64	0	101	1
DDX49__NM_019070_GAACTGCTCTGCGATCTGGT	DDX49__NM_019070	768	27	1092	14
DDX49__NM_019070_GCTCTCTCGGAAACCACACG	DDX49__NM_019070	200	0	206	4
DDX49__NM_019070_GGGTCCTGGGGAAGCCTCTA	DDX49__NM_019070	254	34	258	63
DDX49__NM_019070_TGCTTGGGCTGTGCTAAGAC	DDX49__NM_019070	136	56	173	32
DDX5__NM_004396_ACCTCGATTTGGAGGAAGTA	DDX5__NM_004396	245	109	290	237
DDX5__NM_004396_AGCAAGGAAATTACAGTTAG	DDX5__NM_004396	248	53	233	86
DDX5__NM_004396_AGCTACTTGCTGCACCTGTT	DDX5__NM_004396	1251	484	1397	785
DDX5__NM_004396_AGGTTTGGTGCACCTCGATT	DDX5__NM_004396	603	407	565	392
DDX5__NM_004396_GCCCTACTTCCTCCAAATCG	DDX5__NM_004396	1584	877	1639	1058
DDX5__NM_004396_GGATGGCCAGTTGCTCTAAG	DDX5__NM_004396	254	120	270	225
DDX5__NM_004396_GTCACTCGAATAACCCGACA	DDX5__NM_004396	205	136	241	119
DDX5__NM_004396_TCCAATCCACTTAGAGCAAC	DDX5__NM_004396	327	260	350	303
DDX5__NM_004396_TGCTCTAAGTGGATTGGATA	DDX5__NM_004396	177	319	204	276
DDX5__NM_004396_TGGATTGGATATGGTTGGAG	DDX5__NM_004396	52	7	65	21
DDX5__NM_004396_TGTTGGGCCAGTTCCCGAGT	DDX5__NM_004396	719	112	541	131
DDX5__NM_004396_TTATCAAGAGCACCCTGATT	DDX5__NM_004396	229	82	197	69
DDX50__NM_024045_AAGATTTAATAGCTCAAGCA	DDX50__NM_024045	67	45	86	66
DDX50__NM_024045_AAGTGATAATAAACTAGAGG	DDX50__NM_024045	44	66	73	19
DDX50__NM_024045_ACTCCTCTGGGGGGACATTA	DDX50__NM_024045	460	336	500	413
DDX50__NM_024045_ATTTCTCATCCGAGTCATAA	DDX50__NM_024045	137	141	144	161
DDX50__NM_024045_CCAAAGGTCTTAACTTGAAT	DDX50__NM_024045	47	35	74	86
DDX50__NM_024045_CTCTTCAGAAATAGGAAAAT	DDX50__NM_024045	159	195	131	153
DDX50__NM_024045_CTGGGGGGACATTATGGAGC	DDX50__NM_024045	467	296	432	358
DDX50__NM_024045_CTTTGGAGTCTTTCAATTAA	DDX50__NM_024045	123	177	234	312
DDX50__NM_024045_GTCAAGGCACCATTATGACT	DDX50__NM_024045	492	342	563	563
DDX50__NM_024045_TCCTTCATATACAGGACCAA	DDX50__NM_024045	15	35	60	27
DDX50__NM_024045_TTCTTTCTGTTCACGTGTTA	DDX50__NM_024045	0	0	0	0
DDX50__NM_024045_TTTGGCTCCAACAAGGGAAC	DDX50__NM_024045	0	0	0	0
DDX51__NM_175066_AAACACAGAGGTCGCTAGGC	DDX51__NM_175066	182	0	228	20
DDX51__NM_175066_AAACTTCACACTTCCACAAT	DDX51__NM_175066	351	337	539	365
DDX51__NM_175066_AACACAGTCCACAGGTTCAG	DDX51__NM_175066	440	551	425	431
DDX51__NM_175066_AAGGAATGTCACCGAAGACC	DDX51__NM_175066	158	14	215	19
DDX51__NM_175066_ACAAATGGGCTAGTGACGAG	DDX51__NM_175066	968	919	976	988
DDX51__NM_175066_ACACAAAAGGCGACTGTTAC	DDX51__NM_175066	379	332	343	342
DDX51__NM_175066_ACCTGACGCTAACTGAAGGA	DDX51__NM_175066	255	172	304	324
DDX51__NM_175066_ACTCTGTGTTTAACGCCCCA	DDX51__NM_175066	1137	907	1181	746
DDX51__NM_175066_AGAAATGATTTAGGTAGACG	DDX51__NM_175066	240	390	333	204
DDX51__NM_175066_AGAAGAGCTCTCCGTTGCAG	DDX51__NM_175066	227	104	217	166
DDX51__NM_175066_AGCCTTCCTCCCCAAGCACG	DDX51__NM_175066	518	352	583	402
DDX51__NM_175066_AGCTTCCCTTAGTGCACCTA	DDX51__NM_175066	481	442	644	574
DDX51__NM_175066_AGGCAGGAGAATCACTTGCC	DDX51__NM_175066	300	7	350	49
DDX51__NM_175066_CAAAAGGCGACTGTTACAGG	DDX51__NM_175066	501	276	510	539
DDX51__NM_175066_CATCCACGGGGTGCCTATGC	DDX51__NM_175066	277	171	269	222
DDX51__NM_175066_CCACCGTGCCCGGCCTGTCA	DDX51__NM_175066	1766	971	2093	1526
DDX51__NM_175066_CCTCCAGGGCCGGTCCATCT	DDX51__NM_175066	835	450	809	464
DDX51__NM_175066_CCTGGGACACTCCCTATAGC	DDX51__NM_175066	638	527	670	559
DDX51__NM_175066_CGCGACGTAGAACAGCGCCA	DDX51__NM_175066	25	8	21	5
DDX51__NM_175066_CGGAACCTGACGCTAACTGA	DDX51__NM_175066	690	409	818	481
DDX51__NM_175066_CGGTGAAAAGTACTCCCAGA	DDX51__NM_175066	229	153	280	291
DDX51__NM_175066_CTGAGCCTAACTGTGTCAGA	DDX51__NM_175066	550	173	527	244
DDX51__NM_175066_CTGCACCACAGGGATGACGA	DDX51__NM_175066	250	20	278	0
DDX51__NM_175066_CTTCAGTTAGCGTCAGGTTC	DDX51__NM_175066	125	120	301	341
DDX51__NM_175066_GAAGAGCTCTCCGTTGCAGA	DDX51__NM_175066	298	210	381	265
DDX51__NM_175066_GCAATGAAGCCTTAGTAAAA	DDX51__NM_175066	136	114	130	133
DDX51__NM_175066_GCACCAGCATAGGCACCCCG	DDX51__NM_175066	11	0	13	16
DDX51__NM_175066_GCAGACCACTCTCGAAAGCA	DDX51__NM_175066	391	117	411	225
DDX51__NM_175066_GCCGCAGGGAAAGCGACGGA	DDX51__NM_175066	472	142	564	95
DDX51__NM_175066_GCCTCAGCCTCTTGAGTAGT	DDX51__NM_175066	1046	952	1149	1245
DDX51__NM_175066_GGATGTAGGTTTCATTGATC	DDX51__NM_175066	529	516	550	464
DDX51__NM_175066_GGCAGACCACTCTCGAAAGC	DDX51__NM_175066	942	1595	1016	1316
DDX51__NM_175066_GGCTAGTGACGAGGGGTTAG	DDX51__NM_175066	324	279	311	276
DDX51__NM_175066_TACAAATGGGCTAGTGACGA	DDX51__NM_175066	682	508	761	547
DDX51__NM_175066_TCAGGCCCTGCTTTCGAGAG	DDX51__NM_175066	555	27	552	85
DDX51__NM_175066_TGCCGGGGCCGGGAGTTGGT	DDX51__NM_175066	1143	658	1262	569
DDX51__NM_175066_TGGCGCAGAGACCACGTGCT	DDX51__NM_175066	572	576	532	430
DDX51__NM_175066_TGGCGCTGTTCTACGTCGCG	DDX51__NM_175066	218	48	205	48
DDX51__NM_175066_TTACAAATGGGCTAGTGACG	DDX51__NM_175066	237	150	315	186
DDX51__NM_175066_TTCTACGTCGCGCGGTACCC	DDX51__NM_175066	9	4	6	0
DDX51__NM_175066_TTGCTGGTGGGAAGGTCACG	DDX51__NM_175066	214	69	269	113
DDX51__NM_175066_TTTACTAAAAACCATGGACT	DDX51__NM_175066	29	62	32	44
DDX51__NM_175066_TTTGAACCCCGAGGTTAGAA	DDX51__NM_175066	15	46	29	26
DDX52__NM_001291476_AAACACAAAATTCACGTCCA	DDX52__NM_001291476	618	139	603	165
DDX52__NM_001291476_ACCTTGCTAGGTCGATTCCG	DDX52__NM_001291476	796	320	796	170
DDX52__NM_001291476_AGTTGCTGAAATGTAGCAAT	DDX52__NM_001291476	272	226	365	485
DDX52__NM_001291476_CCCCCGGAATCGACCTAGCA	DDX52__NM_001291476	658	638	906	846
DDX52__NM_001291476_CCTTGCTAGGTCGATTCCGG	DDX52__NM_001291476	860	77	934	304
DDX52__NM_001291476_CTCGTGTTGGTGATATAATC	DDX52__NM_001291476	241	177	231	320
DDX52__NM_001291476_CTTCAGAACATTCTAGATGC	DDX52__NM_001291476	221	179	240	264
DDX52__NM_001291476_GCAGTGGCAGCCAAGAAATT	DDX52__NM_001291476	367	230	395	152
DDX52__NM_001291476_GCTTGCATTTGGATTGGCGT	DDX52__NM_001291476	1225	292	1279	335
DDX52__NM_001291476_GTTGCTGAAATGTAGCAATT	DDX52__NM_001291476	418	16	410	76
DDX52__NM_001291476_TAAACTAACTTCCGGAAAGT	DDX52__NM_001291476	105	178	149	174
DDX52__NM_001291476_TTTGGATTGGCGTAGGCATT	DDX52__NM_001291476	327	320	338	181
DDX52__NM_007010_AACATTACATTGTAAGGGTA	DDX52__NM_007010	214	247	165	186
DDX52__NM_007010_AAGAAAGATCTACTGGTACA	DDX52__NM_007010	191	294	322	355
DDX52__NM_007010_AAGAAGAGAGCCTAACTGAA	DDX52__NM_007010	156	112	152	80
DDX52__NM_007010_AAGAAGGTGCTACTATACAG	DDX52__NM_007010	34	28	33	21
DDX52__NM_007010_AAGGAGTGAAATTAGTGAAC	DDX52__NM_007010	151	125	131	117
DDX52__NM_007010_AAGTCACACAGCTGGCAAAT	DDX52__NM_007010	312	340	329	379
DDX52__NM_007010_ACAGGGTCTGTTACCTAATT	DDX52__NM_007010	859	996	1044	1041
DDX52__NM_007010_ACCATATAGCCTAGGTGTAC	DDX52__NM_007010	681	397	680	293
DDX52__NM_007010_AGAGGGCATCTAACCCAGAT	DDX52__NM_007010	455	455	490	394
DDX52__NM_007010_AGTAGCTGGTACTATAGGTG	DDX52__NM_007010	107	6	133	14
DDX52__NM_007010_ATATGACTTTGATTCTTCGG	DDX52__NM_007010	358	270	465	219
DDX52__NM_007010_CAAGGTGTACTACTCTTGTT	DDX52__NM_007010	110	74	154	64
DDX52__NM_007010_CAAGTGCTCTGTTAGATGTA	DDX52__NM_007010	419	421	482	408
DDX52__NM_007010_CACGATCTCTTTCGCCGGCT	DDX52__NM_007010	3077	1396	3490	1925
DDX52__NM_007010_CAGACAGCAGATACAATGGT	DDX52__NM_007010	678	335	624	488
DDX52__NM_007010_CAGGACAGTAACATGCTATA	DDX52__NM_007010	303	390	371	280
DDX52__NM_007010_CAGTGCTAGGTACTCACAAT	DDX52__NM_007010	996	928	953	797
DDX52__NM_007010_CATGTAGCACATGACTAGTG	DDX52__NM_007010	273	290	269	276
DDX52__NM_007010_CCAGAATATATATTGGGTAA	DDX52__NM_007010	339	255	263	258
DDX52__NM_007010_GAAGCGTCTCGTGTCGAATT	DDX52__NM_007010	786	375	747	358
DDX52__NM_007010_GAGGGCATCTAACCCAGATT	DDX52__NM_007010	627	208	668	255
DDX52__NM_007010_GATGCCAATTGTAACTCCTA	DDX52__NM_007010	502	442	594	459
DDX52__NM_007010_GCTCCCTCTTCCTTTCAGTT	DDX52__NM_007010	1391	582	1258	686
DDX52__NM_007010_GGATTATGATCATATGGTGT	DDX52__NM_007010	398	300	472	493
DDX52__NM_007010_GGTATTACGTATCCAATACA	DDX52__NM_007010	602	383	464	752
DDX52__NM_007010_GTATCCTTAGGAGTTACAAT	DDX52__NM_007010	239	191	304	220
DDX52__NM_007010_GTTTGCTTGCTAACAAGTAA	DDX52__NM_007010	159	165	130	238
DDX52__NM_007010_TACACTGACTAAGTTGATAC	DDX52__NM_007010	350	368	345	363
DDX52__NM_007010_TAGATCTCTACTTAAGCTAA	DDX52__NM_007010	176	271	213	145
DDX52__NM_007010_TATTCTGGAGGTAGGTATAC	DDX52__NM_007010	79	48	86	39
DDX52__NM_007010_TCCAACTGTCTATAGCATGC	DDX52__NM_007010	159	154	182	155
DDX52__NM_007010_TCCTGCATGCTATAGACAGT	DDX52__NM_007010	467	266	539	604
DDX52__NM_007010_TGACTTCATATACGTAAGAC	DDX52__NM_007010	172	207	172	146
DDX52__NM_007010_TGATTTCAAATGTTTGGTAC	DDX52__NM_007010	346	463	413	424
DDX52__NM_007010_TTTATAAAGGCCAGATATAG	DDX52__NM_007010	1	0	3	3
DDX52__NM_007010_TTTCAGCTTCACACAAGGAA	DDX52__NM_007010	71	225	93	71
DDX53__NM_182699_AAGTGTTTCTATAGCCGCTT	DDX53__NM_182699	125	135	84	90
DDX53__NM_182699_CTCGGTCCCTGATGGCCGAA	DDX53__NM_182699	457	710	621	798
DDX53__NM_182699_CTGAAAAGCATAATAAGGGT	DDX53__NM_182699	423	492	411	575
DDX53__NM_182699_CTGAAATGCAGGTGATTAAC	DDX53__NM_182699	226	308	298	378
DDX53__NM_182699_CTGCTGCTCTCGGTCCCTGA	DDX53__NM_182699	907	1190	1018	1043
DDX53__NM_182699_GATTTCTTCCAATAGGGGTT	DDX53__NM_182699	68	109	38	196
DDX53__NM_182699_GCCTGTGACTGAATTGGCGT	DDX53__NM_182699	1610	1845	1721	2424
DDX53__NM_182699_GCTGCATCCCAAACCCCTAT	DDX53__NM_182699	1650	1650	1792	1653
DDX53__NM_182699_GGCTAATCCAAGAGACCTTG	DDX53__NM_182699	202	316	231	354
DDX53__NM_182699_GGTGTGGTCATTGGTTACAG	DDX53__NM_182699	421	487	527	649
DDX53__NM_182699_GTCAAATTGGGATCGCATTA	DDX53__NM_182699	280	278	400	395
DDX53__NM_182699_TGTCAAATTGGGATCGCATT	DDX53__NM_182699	227	275	228	248
DDX54__NM_001111322_ACCTCGGTCGCGAGCTGCCA	DDX54__NM_001111322	1178	805	1296	526
DDX54__NM_001111322_CACATCCGAGGTGCATTCCG	DDX54__NM_001111322	1161	1146	1304	1260
DDX54__NM_001111322_CCCCACCTCGGAATGCACCT	DDX54__NM_001111322	2287	2326	2787	2255

DDX54__NM_001111322_CCGAGGTGCATTCCGAGGTG	DDX54__NM_001111322	467	465	420	220
DDX54__NM_001111322_CTGAGCTACCCGGTGTTCAA	DDX54__NM_001111322	272	20	267	26
DDX54__NM_001111322_CTTTGAACACCGGGTAGCTC	DDX54__NM_001111322	3	0	0	0
DDX54__NM_001111322_GCCATGGCAGCTCGCGACCG	DDX54__NM_001111322	1082	811	1154	828
DDX54__NM_001111322_GCCCGAATGGCGGCCGACAA	DDX54__NM_001111322	41	18	23	25
DDX54__NM_001111322_GGCCCTTGTCGGCCGCCATT	DDX54__NM_001111322	214	166	320	337
DDX54__NM_001111322_TCCAGGCGGAAGATGACGCC	DDX54__NM_001111322	86	40	93	83
DDX54__NM_001111322_TCTTGAGCCGCTCGAACATT	DDX54__NM_001111322	554	741	694	831
[illegible]					
DDX6__NM_001257191_GGATCTAAGAATCAAAACTT	DDX6__NM_001257191	89	5	52	14
DDX6__NM_001257191_GGCCACCAGTGGGTTTCACA	DDX6__NM_001257191	1040	686	1080	907
DDX6__NM_001257191_TCCCTACCAGATAAAGCAAT	DDX6__NM_001257191	232	10	262	27
DDX6__NM_001257191_TGGACAGACCCATTATAACA	DDX6__NM_001257191	285	65	300	149
DDX60__NM_017631_ATCTTTCCAACTGGTTAAGC	DDX60__NM_017631	79	152	100	102
DDX60__NM_017631_ATGGAATAATATTACGGAAG	DDX60__NM_017631	94	104	132	106
DDX60__NM_017631_CGAGATGCTTATCAAAAGAG	DDX60__NM_017631	289	540	302	385
DDX60__NM_017631_CTGCAACTATCAGGAAATAT	DDX60__NM_017631	356	518	265	302
[illegible]					
DEF6__NM_022047_CAGCGCTTTCCGTCGCGGTC	DEF6__NM_022047	1623	1577	1972	2082
DEF6__NM_022047_CAGGTGCTGCCAGACCGCGA	DEF6__NM_022047	380	236	368	277
DEF6__NM_022047_CTCCACGTCCAGCGCGGTAA	DEF6__NM_022047	393	393	482	284
DEF6__NM_022047_CTCCAGGGCCACGGGGTCAT	DEF6__NM_022047	226	199	276	198
DEF6__NM_022047_GAAAAGGGGCATTATCCCGC	DEF6__NM_022047	520	446	602	545
DEF6__NM_022047_GCTCATCTCATACGTGCGGT	DEF6__NM_022047	1582	2225	1884	2621
DEF6__NM_022047_GCTGTCCCACAACCTGTACA	DEF6__NM_022047	961	1269	1142	1422
DEF6__NM_022047_GGATGTGCAGGACCGTGTAC	DEF6__NM_022047	440	438	634	649
[illegible]					
DEFB125__NM_153325_CATAGTAGTCTCGGGAGTAG	DEFB125__NM_153325	620	442	740	924
DEFB125__NM_153325_GACGATGTTTAGATACTGAA	DEFB125__NM_153325	387	431	408	506
DEFB125__NM_153325_GATCATTCAACATAGATACT	DEFB125__NM_153325	128	121	178	142
DEFB125__NM_153325_TATCTGTGGGTTGCTAACTC	DEFB125__NM_153325	175	167	222	137
DEFB125__NM_153325_TCACCTAGAGGATATAACAT	DEFB125__NM_153325	249	318	223	271
DEFB126__NM_030931_AAAAAGTGTCTAAACGACGT	DEFB126__NM_030931	167	151	156	225
DEFB126__NM_030931_AAACTGCAAGGGTGAACAGT	DEFB126__NM_030931	841	904	796	988
DEFB126__NM_030931_AAATTCTTGTAGTCTTTGTC	DEFB126__NM_030931	481	658	525	726
[illegible]					

[illegible]

DHDDS__NM_001243565_AGCATTGCTGATCTCATGAC	DHDDS__NM_001243565	479	6	527	33
DHDH__NM_014475_AAGTCCTGTGATTCCCCTGT	DHDH__NM_014475	1005	1077	1115	748
DHDH__NM_014475_CATGCCCTTGGTGCCGCTCA	DHDH__NM_014475	1544	1765	1883	1993
DHDH__NM_014475_CCAGAGAAGATTTCTGTCGT	DHDH__NM_014475	1325	1822	1602	2120
DHDH__NM_014475_CCGCGATCTGAGCCGTGCGA	DHDH__NM_014475	583	561	673	925
DHDH__NM_014475_CGGGGCAGGCATGAGTTATG	DHDH__NM_014475	757	1060	955	1139
DHDH__NM_014475_CTGGCCAAGGACCCGAGCGT	DHDH__NM_014475	724	595	768	710
DHDH__NM_014475_GTCTGGGAGTGCCTACGCAA	DHDH__NM_014475	313	213	325	235
DHDH__NM_014475_GTTATGAGGCCAAGCACGTC	DHDH__NM_014475	143	94	230	190
DHDH__NM_014475_TAGGCACTCCCAGACGTGCT	DHDH__NM_014475	298	423	523	356
DHDH__NM_014475_TCCGCCGCGTTCACGCCCGT	DHDH__NM_014475	2077	1902	2543	2278
DHDH__NM_014475_TCTGTGCAAACTCCTTCGCA	DHDH__NM_014475	888	1033	1136	1071
DHDH__NM_014475_TTATGAGGCCAAGCACGTCT	DHDH__NM_014475	151	209	211	152
DHFR__NM_000791_AAGGAAGCCATGAATCACCC	DHFR__NM_000791	280	3	305	26
DHFR__NM_000791_ACAAATAGTTTAAGATGGCC	DHFR__NM_000791	149	196	269	370
DHFR__NM_000791_CAAATAAAGTAGACATGGTC	DHFR__NM_000791	151	144	136	336
DHFR__NM_000791_CTCCTTGTGGAGGTTCCCTA	DHFR__NM_000791	343	275	300	269
DHFR__NM_000791_GAGAAGAATCGACCTTTAAA	DHFR__NM_000791	101	0	57	0
DHFRL1__NM_001195643_AAGGAAGCCATGAATCACCT	DHFRL1__NM_001195643	910	895	994	1083
DHFRL1__NM_001195643_CGTCGCTGTGTCCCAAAACA	DHFRL1__NM_001195643	530	700	449	537
DHH__NM_021044_ACAAATTGCTTGTAGAGTAG	DHH__NM_021044	169	240	167	187
DHH__NM_021044_ACAGGGGCAGTAGATTGGTC	DHH__NM_021044	393	295	343	328
DHH__NM_021044_ACCGCCTGATGACCGAGGTA	DHH__NM_021044	1475	1213	1589	639
DHH__NM_021044_AGGGTCCGCTCTGGCACGCC	DHH__NM_021044	464	689	506	735
DHH__NM_021044_AGTCACTCGTAGGCGCACTC	DHH__NM_021044	503	322	484	390
DHH__NM_021044_ATGATGTCGGGGTTGTAGTT	DHH__NM_021044	495	644	539	350
DHH__NM_021044_CAGGGTCCGCTCTGGCACGC	DHH__NM_021044	771	746	830	581
DHH__NM_021044_CGAGCTGCTTGCGCGCATAG	DHH__NM_021044	14	78	61	26
DHH__NM_021044_CTCTACAAGCAATTTGTGCC	DHH__NM_021044	161	220	183	219
DHH__NM_021044_GCTTGCGCGCATAGCGGCGC	DHH__NM_021044	0	0	0	0
DHH__NM_021044_TAAGGAGCGGGTGAACGCTT	DHH__NM_021044	223	172	200	112
DHH__NM_021044_TCACCCGCTCCTTACAACGC	DHH__NM_021044	679	596	694	306
DHODH__NM_001361_AGGACCAAGCTGTCATTAAC	DHODH__NM_001361	256	0	210	4
DHODH__NM_001361_CAGCAATTCCTACTGGATTT	DHODH__NM_001361	1137	261	1278	396
DHODH__NM_001361_CAGTGGTGGAACACAGGTTA	DHODH__NM_001361	183	32	243	64
DHODH__NM_001361_CATCTTATAAAGTCCGTCCA	DHODH__NM_001361	444	32	599	9
DHODH__NM_001361_CATGTCAGAGTCTTGAAATC	DHODH__NM_001361	320	128	419	174
DHODH__NM_001361_CCTACCTGTTAATGACAGCT	DHODH__NM_001361	1127	106	1446	140
DHODH__NM_001361_CGTGGACGGACTTTATAAGA	DHODH__NM_001361	633	8	601	27
DHODH__NM_001361_CTACTGGATTTCGGAATTTA	DHODH__NM_001361	221	171	231	176
DHODH__NM_001361_CTCAGGGAGGCGGAAGACTC	DHODH__NM_001361	87	5	133	3
DHODH__NM_001361_CTTGAAATCTGGCCCGTGGA	DHODH__NM_001361	1782	1898	2262	2369
DHODH__NM_001361_GGTATGGATTTAACAGTCAC	DHODH__NM_001361	462	72	559	116
DHODH__NM_001361_GTGGACGGACTTTATAAGAT	DHODH__NM_001361	72	1	111	3
DHPS__NM_001206974_AAACTTGCAGTAATTCTCAT	DHPS__NM_001206974	352	361	364	551
DHPS__NM_001206974_AACTTGCAGTAATTCTCATT	DHPS__NM_001206974	1137	34	1251	25
DHPS__NM_001206974_ACCCAGAGTCCGTGTATTAC	DHPS__NM_001206974	800	932	950	961
DHPS__NM_001206974_ATCACCATCTGGTCCAGAAT	DHPS__NM_001206974	494	7	646	4
DHPS__NM_001206974_CCCAGAGTCCGTGTATTACT	DHPS__NM_001206974	2294	47	2698	69
DHPS__NM_001206974_CTTCTGGGCCCAGTAATACA	DHPS__NM_001206974	380	0	431	0
DHPS__NM_001206974_CTTGTGCAGCACAACATGGT	DHPS__NM_001206974	0	0	0	0
DHPS__NM_001206974_GCCCAGTAATACACGGACTC	DHPS__NM_001206974	578	646	473	666
DHPS__NM_001206974_GTGCTGCACAAGGTAGCGAA	DHPS__NM_001206974	39	32	34	34
DHPS__NM_001206974_TAAGTGCGGGACTAAACACA	DHPS__NM_001206974	332	11	383	1
DHPS__NM_001206974_TCCGGGAGAACGGGATCAAT	DHPS__NM_001206974	303	22	397	0
DHPS__NM_001206974_TGGGCGAGTTTAGCCTCAGG	DHPS__NM_001206974	139	83	199	0
DHPS__NM_001930_CGAGCTGTGCTTTAGCACGG	DHPS__NM_001930	80	23	83	7
DHPS__NM_001930_CGCGGTTGAAGTCGTAGCCC	DHPS__NM_001930	621	0	716	61
DHPS__NM_001930_CGGGGCTACGACTTCAACCG	DHPS__NM_001930	1646	57	1740	4
DHPS__NM_001930_GGCTTCCAAGCAACCAACTT	DHPS__NM_001930	301	86	357	121
DHRS1__NM_001136050_ACTGGTGCCTCCAGGGGTAT	DHRS1__NM_001136050	627	732	641	464
DHRS1__NM_001136050_CACACACACTTGGCCATTCA	DHRS1__NM_001136050	900	999	1191	989
DHRS1__NM_001136050_CCTCCTGAGCAACAACGCGA	DHRS1__NM_001136050	169	181	203	156
DHRS1__NM_001136050_CCTGTTTGAGCAAGTGGATC	DHRS1__NM_001136050	288	441	316	266
DHRS1__NM_001136050_CTCCAGGCACAATCCCTCGG	DHRS1__NM_001136050	904	989	1050	883
DHRS1__NM_001136050_CTCCTGAGCAACAACGCGAA	DHRS1__NM_001136050	34	51	48	91
DHRS1__NM_001136050_GCAAGGGCGTCTAGATGTGC	DHRS1__NM_001136050	554	488	638	702
DHRS1__NM_001136050_GCCTCCAGGGGTATTGGCCG	DHRS1__NM_001136050	404	396	491	467
DHRS1__NM_001136050_GCCTGTTTGAGCAAGTGGAT	DHRS1__NM_001136050	588	801	654	739
DHRS1__NM_001136050_GCGGCCAGTGATGTAAACTG	DHRS1__NM_001136050	379	477	441	434
DHRS1__NM_001136050_TGGCTTGAATCGCACACCAC	DHRS1__NM_001136050	85	103	118	143
DHRS1__NM_001136050_TGTGGTGTGCGATTCAAGCC	DHRS1__NM_001136050	147	142	139	107
DHRS11__NM_024308_AACTCCTTTCCCATGTCAAA	DHRS11__NM_024308	632	488	654	397
DHRS11__NM_024308_AGGAGATAGATAAGGGGGAG	DHRS11__NM_024308	127	34	101	48
DHRS11__NM_024308_ATCATTAACATCAATAGGTG	DHRS11__NM_024308	22	22	26	21
DHRS11__NM_024308_ATGATCACCATGAAATCCAC	DHRS11__NM_024308	388	223	397	278
DHRS11__NM_024308_CACACCTGGAGAGATGCACT	DHRS11__NM_024308	1053	1008	1032	935
DHRS11__NM_024308_CAGACAGGGGTAACACTCGG	DHRS11__NM_024308	860	557	988	561
DHRS11__NM_024308_CAGCGCAGTGACGGCATACT	DHRS11__NM_024308	558	561	449	459
DHRS11__NM_024308_CCGAGAAGGAGATAGATAAG	DHRS11__NM_024308	362	374	403	325
DHRS11__NM_024308_CCTGTCCACACCCCGACCAG	DHRS11__NM_024308	1651	1011	1542	1095
DHRS11__NM_024308_CGCCTTCAAACTCCACGACA	DHRS11__NM_024308	820	904	1048	1160
DHRS11__NM_024308_CTGCATCAACAATGCTGGCT	DHRS11__NM_024308	141	322	285	474
DHRS11__NM_024308_TCTCAGGCAGCACCAGTGGT	DHRS11__NM_024308	328	257	342	221
DHRS11__NM_024308_TGATCACCATGAAATCCACT	DHRS11__NM_024308	626	419	678	600
DHRS11__NM_024308_TGGATAACAACACAGATATA	DHRS11__NM_024308	50	143	65	57
DHRS11__NM_024308_TTCTTGCACCTCAACGTCTG	DHRS11__NM_024308	24	24	35	21
DHRS12__NM_001031719_AAACTTTGCTGCCAATACTC	DHRS12__NM_001031719	217	249	214	309
DHRS12__NM_001031719_AACAAATTTCCAGATTTGCT	DHRS12__NM_001031719	134	292	132	178
DHRS12__NM_001031719_AGGACACAGCTCGAATGATC	DHRS12__NM_001031719	148	123	126	127
DHRS12__NM_001031719_GGTGTGTACATTCTCACGAC	DHRS12__NM_001031719	572	519	568	368
DHRS12__NM_001031719_TACCTCGCTTGGCGATTTCA	DHRS12__NM_001031719	549	812	702	736
DHRS12__NM_001031719_TCCGAAAGAACACCATTTGA	DHRS12__NM_001031719	799	924	978	806
DHRS12__NM_001031719_TGGAAGAAAAATGGATGGCC	DHRS12__NM_001031719	68	18	93	84
DHRS12__NM_001270424_CATCCGGGAGAGCGGTAACC	DHRS12__NM_001270424	518	637	548	585
DHRS12__NM_001270424_CCGAACCTGGCGTGGAACCC	DHRS12__NM_001270424	547	835	680	640
DHRS12__NM_001270424_CGATGCCGGGGTTCCACGCC	DHRS12__NM_001270424	736	1019	904	1215
DHRS12__NM_001270424_GCGTCGTGTGGTTCGCCAAG	DHRS12__NM_001270424	338	273	355	216
DHRS12__NM_001270424_GGGCTTGATCTCGACAAACC	DHRS12__NM_001270424	337	263	296	408
DHRS12__NM_001270424_TTTCTCCTCTTCGGCCGGTG	DHRS12__NM_001270424	47	68	66	101
DHRS13__NM_144683_AAGCCAATGGGCGCAAAAGT	DHRS13__NM_144683	419	420	434	399
DHRS13__NM_144683_ACGTCTTGACTTCAAACGCC	DHRS13__NM_144683	325	413	446	533
DHRS13__NM_144683_ATGTGCCCCTAGCCGCGTGG	DHRS13__NM_144683	1730	1541	1689	1456
DHRS13__NM_144683_CAGCAGGTTAAACGCCTCAC	DHRS13__NM_144683	151	85	193	115
DHRS13__NM_144683_CAGCGGCATCGGAAAGATGA	DHRS13__NM_144683	385	383	371	366
DHRS13__NM_144683_CGGCCACAGGAACTGATACC	DHRS13__NM_144683	391	326	234	310
DHRS13__NM_144683_GATGCCGCTGTTGGCGCCTG	DHRS13__NM_144683	664	750	745	810
DHRS13__NM_144683_GCAGCAGGTTAAACGCCTCA	DHRS13__NM_144683	873	690	979	883
DHRS13__NM_144683_GCCACAGGAACTGATACCTG	DHRS13__NM_144683	1006	699	976	1025
DHRS13__NM_144683_GGCATATGCTGACACTAAGC	DHRS13__NM_144683	277	147	357	253
DHRS13__NM_144683_GTCAAGACGTCCCCGACAGT	DHRS13__NM_144683	805	329	551	401
DHRS13__NM_144683_TGGCTAATGTACTGTTTGCC	DHRS13__NM_144683	93	66	100	77
DHRS2__NM_005794_ACTTCCTGGTGTGCAGCGCA	DHRS2__NM_005794	242	215	324	377
DHRS2__NM_005794_AGCACCGGGATAGACAGGAA	DHRS2__NM_005794	423	359	499	376
DHRS2__NM_005794_CAGGGGTCAACCCTCTGGTA	DHRS2__NM_005794	493	484	551	421
DHRS2__NM_005794_CCACGGCTACCCGGTTAGCC	DHRS2__NM_005794	11	5	20	32
DHRS2__NM_005794_CCAGAGTGCTCCCTACCAGA	DHRS2__NM_005794	834	1311	1109	974
DHRS2__NM_005794_CCATACCTGTTCTCCATGTA	DHRS2__NM_005794	1800	1811	1913	2119
DHRS2__NM_005794_GAAGGGCGTCCTGGCTAACC	DHRS2__NM_005794	504	459	662	593
DHRS2__NM_005794_GCAGGGGTCAACCCTCTGGT	DHRS2__NM_005794	1203	941	1143	842
DHRS2__NM_005794_GCTGGGTCTCACTAGAACAC	DHRS2__NM_005794	273	261	312	348
DHRS2__NM_005794_GGACATCCGGGTAAACTGCG	DHRS2__NM_005794	307	291	352	372
DHRS2__NM_005794_TACTGGATTATAAGCTGCAA	DHRS2__NM_005794	284	223	230	260
DHRS2__NM_005794_TCTCACTAGAACACTGGCAT	DHRS2__NM_005794	535	558	573	618
DHRS3__NM_004753_AACAATGCCGCCGTGGTCCA	DHRS3__NM_004753	861	795	857	1013
DHRS3__NM_004753_AACGTCCTCATCACCGGCGG	DHRS3__NM_004753	1150	1072	1280	1295
DHRS3__NM_004753_ACGGAGGAGATCCGGCAGAT	DHRS3__NM_004753	994	736	1071	874
DHRS3__NM_004753_CCCTCTACAGATGATCTATC	DHRS3__NM_004753	803	731	692	643
DHRS3__NM_004753_GAAAGTGTTCATGCAGGTGT	DHRS3__NM_004753	493	396	628	562
DHRS3__NM_004753_GAGGATGGTGTGGAAACGGC	DHRS3__NM_004753	227	161	178	109
DHRS3__NM_004753_GCAGCTCCAGCATACGTGGC	DHRS3__NM_004753	242	251	264	291
DHRS3__NM_004753_GCCCAGGGTGTTGATGTGTT	DHRS3__NM_004753	1635	1536	1636	1703
DHRS3__NM_004753_GGCCTTCCTGCCACGTATGC	DHRS3__NM_004753	397	515	467	583
DHRS3__NM_004753_TGCAGCCTGTGGAAGTATGC	DHRS3__NM_004753	238	194	306	265
DHRS3__NM_004753_TGTGCAGTAGTCGATGGCAC	DHRS3__NM_004753	467	476	595	426
DHRS4__NM_001282987_AGGGCCACCTTATTTGCGAG	DHRS4__NM_001282987	715	1247	1029	1143
DHRS4__NM_001282987_CCGGGACCCGCTCGCAAATA	DHRS4__NM_001282987	434	693	501	494
DHRS4__NM_001282987_CGCCTTCCCCACATGGCACA	DHRS4__NM_001282987	2542	2615	2755	2840
DHRS4__NM_001282987_GACGGGCACCGTGTGCCATG	DHRS4__NM_001282987	153	140	101	61
DHRS4__NM_001282987_GGACCCGCTCGCAAATAAGG	DHRS4__NM_001282987	97	103	151	75
DHRS4__NM_001282987_GGCTTGGAATTCGGTGCGGA	DHRS4__NM_001282987	758	968	897	1155
DHRS4__NM_001282987_GGGCCACCTTATTTGCGAGC	DHRS4__NM_001282987	385	494	493	572
DHRS4__NM_001282987_TAGCAGCCCCGCCTTGTGCA	DHRS4__NM_001282987	1000	1292	1086	1302
DHRS4__NM_001282987_TCCGGCTGCTGACGACCACA	DHRS4__NM_001282987	575	360	624	650
DHRS4__NM_001282988_TGTTTCCCCAGTGATGTAGC	DHRS4__NM_001282988	0	0	0	0
DHRS4L2__NM_001193635_CCGGCTGCTGACGACCACGT	DHRS4L2__NM_001193635	2221	2065	2434	2372
DHRS4L2__NM_001193635_GCTGGGCCTCAACAATACCC	DHRS4L2__NM_001193635	169	242	127	194
DHRS4L2__NM_001193636_CCTCCTCGGTGACATCCATT	DHRS4L2__NM_001193636	1399	1620	1610	1462
DHRS4L2__NM_198083_CTGTGCCTGGGCACGGAAGT	DHRS4L2__NM_198083	236	269	245	357
DHRS7__NM_016029_AACCAGTGTCGGTCAGGTCA	DHRS7__NM_016029	1766	1619	1882	2397
DHRS7__NM_016029_ACGGCGACCTGACGCTACTA	DHRS7__NM_016029	832	820	838	850
DHRS7__NM_016029_ACTGGAGCCTCGAGTGGAAT	DHRS7__NM_016029	638	866	552	732
DHRS7__NM_016029_AGAGCTTAACTACTTAGGGA	DHRS7__NM_016029	75	97	104	90
DHRS7__NM_016029_AGCTTTGGTAGCCGCTTCAT	DHRS7__NM_016029	1085	896	1043	888
DHRS7__NM_016029_CATGGGAACCAGTGTCGGTC	DHRS7__NM_016029	791	1173	920	931
DHRS7__NM_016029_CGCTTCATGGGAACCAGTGT	DHRS7__NM_016029	564	622	731	770
DHRS7__NM_016029_CGGCGACCTGACGCTACTAT	DHRS7__NM_016029	794	848	983	1072
DHRS7__NM_016029_CTCCTCACCAATTCCACTCG	DHRS7__NM_016029	948	1221	1268	1431
DHRS7__NM_016029_TATCCATGCACAGAGAACGC	DHRS7__NM_016029	500	534	465	608
DHRS7__NM_016029_TGCTTCCTCTCGATCATGTG	DHRS7__NM_016029	43	17	117	123
DHRS7__NM_016029_TTCTGCCTCACATGATCGAG	DHRS7__NM_016029	0	0	0	0
DHRS7B__NM_015510_AGCCTCCAAGCACGCAACCC	DHRS7B__NM_015510	410	265	424	395
DHRS7B__NM_015510_AGGTCGAAGGTCACCAAGTA	DHRS7B__NM_015510	388	354	403	408
DHRS7B__NM_015510_CACCACAGCATTCCGCAGGT	DHRS7B__NM_015510	925	1038	959	850
DHRS7B__NM_015510_CCCCTGCTGTTCGGCTGCCT	DHRS7B__NM_015510	2615	3510	2832	3799
DHRS7B__NM_015510_CTATGCTGCGGGTGCTAAAC	DHRS7B__NM_015510	442	392	460	493
DHRS7B__NM_015510_GAAAGCCTGGGTTGCGTGCT	DHRS7B__NM_015510	963	1070	994	1247
DHRS7B__NM_015510_GCTCTGTGGCCGGAATGGTG	DHRS7B__NM_015510	11	25	21	35
DHRS7B__NM_015510_GGCCCAGTTGCTCTAACGAA	DHRS7B__NM_015510	58	100	76	65
DHRS7B__NM_015510_GTCATGGAGACAAACTACTT	DHRS7B__NM_015510	366	429	421	564
DHRS7B__NM_015510_TACCTTTCGTTAGAGCAACT	DHRS7B__NM_015510	549	533	650	633
DHRS7B__NM_015510_TCACCACCACAGCATTCCGC	DHRS7B__NM_015510	591	456	499	445
DHRS7B__NM_015510_TGCCCTCCATGATCAAGAGG	DHRS7B__NM_015510	149	236	169	157
DHRS7C__NM_001105571_AACATGATCTCCCGGAGAAC	DHRS7C__NM_001105571	418	417	434	239
DHRS7C__NM_001105571_ACACCTCTTGGTAAATGAAG	DHRS7C__NM_001105571	182	187	167	242
DHRS7C__NM_001105571_ACTTACAAGTCGTACGGAAC	DHRS7C__NM_001105571	168	78	194	259
DHRS7C__NM_001105571_AGACCTTTCGTCAATGTGAT	DHRS7C__NM_001105571	480	1024	774	858
DHRS7C__NM_001105571_CCGTGAGCCCGACTTTCATC	DHRS7C__NM_001105571	4566	5054	5240	6314
DHRS7C__NM_001105571_CCTCCTCTTCATTTACCAAG	DHRS7C__NM_001105571	506	477	513	492
DHRS7C__NM_001105571_GAGACCTTTCGTCAATGTGA	DHRS7C__NM_001105571	332	405	424	560
DHRS7C__NM_001105571_GGCCCCATCACATTGACGAA	DHRS7C__NM_001105571	131	268	250	257
DHRS7C__NM_001105571_GTGTTAGTGAATAATATCCA	DHRS7C__NM_001105571	81	112	118	77
DHRS7C__NM_001105571_TCCAGAGAAATCTTATGGGC	DHRS7C__NM_001105571	707	521	669	711
DHRS7C__NM_001105571_TGATGTCTGAGAGGTCCAAC	DHRS7C__NM_001105571	69	112	110	146
DHRS7C__NM_001105571_TGTGATGGGGCCAAAGTAAT	DHRS7C__NM_001105571	363	545	463	355
DHRS9__NM_001142270_AGGATTGTTCAAAACAAACT	DHRS9__NM_001142270	100	160	103	145
DHRS9__NM_001142270_AGTGGGTGAAGAACCAAGTT	DHRS9__NM_001142270	218	94	263	302
DHRS9__NM_001142270_CCTATTGAAGTGAACCTGTT	DHRS9__NM_001142270	1537	1801	1732	2041
DHRS9__NM_001142270_CGCCTTGCAATCGTTGGAGG	DHRS9__NM_001142270	1109	1208	1323	1434
DHRS9__NM_001142270_CTAGGCCTCCTAATCCTCTG	DHRS9__NM_001142270	311	459	375	692
DHRS9__NM_001142270_GAAAACCACAGAGGATTAGG	DHRS9__NM_001142270	207	311	216	138
DHRS9__NM_001142270_GGAGGTCGCCTTGCAATCGT	DHRS9__NM_001142270	1463	1367	1741	1744
DHRS9__NM_001142270_GGTCGCCTTGCAATCGTTGG	DHRS9__NM_001142270	795	884	1023	1284
DHRS9__NM_001142270_GTCGCCTTGCAATCGTTGGA	DHRS9__NM_001142270	1275	1026	1368	1680
DHRS9__NM_001142270_TCGCCTTGCAATCGTTGGAG	DHRS9__NM_001142270	1059	1279	1074	1350
DHRS9__NM_001142270_TGATCCTGATTCAGTCAGAC	DHRS9__NM_001142270	202	319	229	457
DHRS9__NM_001142270_TGGGGTCTGATCAATAATGC	DHRS9__NM_001142270	11	21	39	43
DHRS9__NM_001289763_TATCCAGTGTCACTCTTGTA	DHRS9__NM_001289763	1242	1192	1237	1242
DHRS9__NM_001289763_TCACTCTTGTAAGGCTGACC	DHRS9__NM_001289763	97	112	134	96
DHTKD1__NM_018706_AGCGGGGCGTTTACGGCTAC	DHTKD1__NM_018706	492	562	773	708
DHTKD1__NM_018706_ATCTCAATCAAATCTACTGT	DHTKD1__NM_018706	505	600	512	585
DHTKD1__NM_018706_CAGACCGAGCGGGGCGTTTA	DHTKD1__NM_018706	1046	1115	973	1223
DHTKD1__NM_018706_CAGGGACCCTTCCACACGGC	DHTKD1__NM_018706	716	646	780	937
DHTKD1__NM_018706_CGAAGGGGCTGAAAGCATGA	DHTKD1__NM_018706	223	252	310	494
DHTKD1__NM_018706_GCAGGGCTTGTCCGGTGAAG	DHTKD1__NM_018706	1079	861	1125	1198
DHTKD1__NM_018706_GCGGTTTGAGGAACTGCAAA	DHTKD1__NM_018706	107	79	110	108
DHTKD1__NM_018706_GGCGTGGCTACCAGACCGAG	DHTKD1__NM_018706	1181	1421	1322	1402
DHTKD1__NM_018706_TCACAGGATTATTGAACATG	DHTKD1__NM_018706	599	825	791	723
DHTKD1__NM_018706_TCGCTTCACTGTCGAGAACT	DHTKD1__NM_018706	476	507	548	729
DHTKD1__NM_018706_TTCTCGACAGTGAAGCGATA	DHTKD1__NM_018706	0	0	0	0
DHTKD1__NM_018706_TTGCAGTTCCTCAAACCGCT	DHTKD1__NM_018706	293	485	277	333
DHX15__NM_001358_AACGTCTTCAGCTCCCTGTT	DHX15__NM_001358	1278	895	1404	1234
DHX15__NM_001358_AGCGGCACCGGTTGGACCTA	DHX15__NM_001358	1548	523	1517	521
DHX15__NM_001358_CCCCGCACGCTTCTTGCCAG	DHX15__NM_001358	2398	1974	2896	2417
DHX15__NM_001358_CGAGACCGAGAACGTGATAG	DHX15__NM_001358	637	548	871	730
DHX15__NM_001358_GAGACCGGGATCGTGAAGAT	DHX15__NM_001358	388	637	513	533
DHX15__NM_001358_TAGACCGATCTTCACGATCC	DHX15__NM_001358	438	414	493	406
DHX15__NM_001358_TATCATAGTATCGAGGAGTA	DHX15__NM_001358	123	10	141	96
DHX15__NM_001358_TATCCTTGTATTCCCAAACA	DHX15__NM_001358	216	4	270	0
DHX15__NM_001358_TCAACGCATGCCGGACATGC	DHX15__NM_001358	0	0	0	0
DHX15__NM_001358_TCGAGGAGTATGGGGTAAGT	DHX15__NM_001358	1305	296	1415	376
DHX15__NM_001358_TCTGCTCATTCAACGCATGC	DHX15__NM_001358	36	2	62	41
DHX15__NM_001358_TGGGTCTGGTAAAACAACAC	DHX15__NM_001358	40	0	28	0
DHX29__NM_019030_AAGAGCAGCAACCTAAAAGT	DHX29__NM_019030	880	427	793	426
DHX29__NM_019030_AAGAGGCTCAGGAAAAAATA	DHX29__NM_019030	62	64	69	46
DHX29__NM_019030_AATTCCAGCCTCGGCAGATT	DHX29__NM_019030	440	463	442	404
DHX29__NM_019030_ACCTCCATATAAGAGTGTAT	DHX29__NM_019030	523	190	630	205
DHX29__NM_019030_ACTTACTGCCAAAAAATTGC	DHX29__NM_019030	228	497	266	293
DHX29__NM_019030_AGAGCCAAATCTGCCGAGGC	DHX29__NM_019030	2225	2058	2417	2239
DHX29__NM_019030_AGGTGACCTTCATTCTGCCT	DHX29__NM_019030	1530	1842	1554	2147
DHX29__NM_019030_ATATAAGAGTGTATTGGTCA	DHX29__NM_019030	232	64	228	118
DHX29__NM_019030_CGAGGCTGGAATTGCCGGGG	DHX29__NM_019030	1279	1327	1341	1117
DHX29__NM_019030_TTCATGGCTGGGTTAAATAC	DHX29__NM_019030	35	70	70	74
DHX29__NM_019030_TTCCAGAGCCAAATCTGCCG	DHX29__NM_019030	80	64	125	107
DHX29__NM_019030_TTGCAATGGAGGTGAAATAG	DHX29__NM_019030	117	51	119	50
DHX30__NM_014966_AAAATGGCCCAAGAGCGTGG	DHX30__NM_014966	175	75	129	217
DHX30__NM_014966_AACGTCATCCTCCACAGCTA	DHX30__NM_014966	354	131	319	146
DHX30__NM_014966_AATCCAGAGAGTATTCGACC	DHX30__NM_014966	434	141	440	290
DHX30__NM_014966_AGCGTGGAGGTAGAAGGCTA	DHX30__NM_014966	293	417	364	204
DHX30__NM_014966_ATCCAGAGAGTATTCGACCA	DHX30__NM_014966	414	379	439	173
DHX30__NM_014966_CCAGAGAGTATTCGACCAGG	DHX30__NM_014966	983	495	1048	512
DHX30__NM_014966_CTAGTCTACGTGCACACAAA	DHX30__NM_014966	156	123	217	93
DHX30__NM_014966_CTTCTTCCCGGCCTAAAGAG	DHX30__NM_014966	177	113	194	178
DHX30__NM_014966_GCAAGAAGATCGATGCTGAG	DHX30__NM_014966	646	396	557	478
DHX30__NM_014966_GCGTCAAACAACTCATTCCG	DHX30__NM_014966	1281	1391	1505	1256
DHX30__NM_014966_TCCAGAGAGTATTCGACCAG	DHX30__NM_014966	297	186	388	252
DHX30__NM_014966_TGAGTCCCTGAGTGGAGCGT	DHX30__NM_014966	250	129	222	130
DHX32__NM_018180_AATCCCTGGATTCCAGCGAT	DHX32__NM_018180	64	118	52	103
DHX32__NM_018180_AGTGAAAAATAAACACCCTG	DHX32__NM_018180	320	390	366	288
DHX32__NM_018180_ATCCCTGGATTCCAGCGATG	DHX32__NM_018180	113	52	140	134
DHX32__NM_018180_CAACCTCAGGATTGTTTCGT	DHX32__NM_018180	1774	1648	2137	2268
DHX32__NM_018180_CAACGAAACAATCCTGAGGT	DHX32__NM_018180	1316	1300	1556	1729
DHX32__NM_018180_GAATATGGCAATCCATCAAA	DHX32__NM_018180	99	106	96	54
DHX32__NM_018180_GATGAAATGGATGTTAACAT	DHX32__NM_018180	803	679	829	896
DHX32__NM_018180_GCTGGACCACAGTCTGCTTG	DHX32__NM_018180	1404	1647	1493	1663
DHX32__NM_018180_GTTTCAGGAGATGCTAAATG	DHX32__NM_018180	323	365	374	412
DHX32__NM_018180_TCCATCCACTACCAGCACGG	DHX32__NM_018180	511	644	491	461
DHX32__NM_018180_TTGAAGCAGGTTCTCCATAA	DHX32__NM_018180	189	244	189	188
DHX32__NM_018180_TTTCCATCCACTACCAGCAC	DHX32__NM_018180	262	332	317	313
DHX33__NM_001199699_ACATCCTGGTGTTCCTCACT	DHX33__NM_001199699	1736	117	2007	299
DHX33__NM_001199699_ACCCTCTAGGTAGAGGACGG	DHX33__NM_001199699	104	13	97	2
DHX33__NM_001199699_CATTGAAATACTGAGAGAAC	DHX33__NM_001199699	472	41	634	137
DHX33__NM_001199699_CCGTCCTCTACCTAGAGGGT	DHX33__NM_001199699	1735	17	1915	135
DHX33__NM_001199699_GAGCTGCTGTGCATAGGGCA	DHX33__NM_001199699	160	1	142	34
DHX33__NM_001199699_GATGTCAGCTACGATGGATG	DHX33__NM_001199699	728	38	955	63
DHX33__NM_001199699_GATTGTGATGTCAGCTACGA	DHX33__NM_001199699	494	138	471	302
DHX33__NM_001199699_GCAGGGAGGCGTACAGAGGA	DHX33__NM_001199699	500	150	617	288
DHX33__NM_001199699_GCCCCCGTCCTCTACCTAGA	DHX33__NM_001199699	986	52	1053	26
DHX33__NM_001199699_GCGTGCAGGTAATCATTCTG	DHX33__NM_001199699	1595	1779	1867	2298
DHX33__NM_001199699_GCTGCCGACCCTCTAGGTAG	DHX33__NM_001199699	650	1	550	3
DHX33__NM_001199699_TAGGGCAGGGAGGCGTACAG	DHX33__NM_001199699	299	0	265	0
DHX33__NM_020162_CCCGCCTCCTCCGGCATGTC	DHX33__NM_020162	1635	2045	1867	2000
DHX33__NM_020162_CCTCAGTACCTGTATGAAGG	DHX33__NM_020162	811	45	764	58
DHX33__NM_020162_CCTCCTTCATACAGGTACTG	DHX33__NM_020162	254	4	349	3
DHX33__NM_020162_CGATGAGGACCGCGTTGTCC	DHX33__NM_020162	1015	151	1168	130
DHX33__NM_020162_GAGATGGCAGCTACTCGACG	DHX33__NM_020162	843	18	1053	23
DHX33__NM_020162_GATGAGAAGAGAACTGAACT	DHX33__NM_020162	500	18	526	71
DHX33__NM_020162_GGCAGCTACTCGACGAGGCT	DHX33__NM_020162	664	214	561	308
DHX33__NM_020162_GTTGTCCAGGTTTCGGAGCT	DHX33__NM_020162	261	42	479	79
DHX33__NM_020162_TCCCGCCGGCCAAGAGATTC	DHX33__NM_020162	403	452	551	608
DHX33__NM_020162_TGGCAGCTACTCGACGAGGC	DHX33__NM_020162	10	0	10	3
DHX34__NM_014681_AGCGCTGGGATGCTGTGCTT	DHX34__NM_014681	895	929	854	752
DHX34__NM_014681_CAAAGTCCAGGTAGTGCAAC	DHX34__NM_014681	207	252	245	342
DHX34__NM_014681_CATGCCTCCTCCTAGAACAA	DHX34__NM_014681	556	592	623	533
DHX34__NM_014681_CCGAGCCCTGTTGCACTACC	DHX34__NM_014681	1213	1105	1174	1356
DHX34__NM_014681_CTGCCCTCCCTTGTTCTAGG	DHX34__NM_014681	1124	1064	1400	1685
DHX34__NM_014681_GAAGAGGATTACATCCGTCA	DHX34__NM_014681	431	513	583	432
DHX34__NM_014681_GAGAGGTTGATGCGGTAACG	DHX34__NM_014681	816	1141	1036	824
DHX34__NM_014681_GCCTCCTCCTAGAACAAGGG	DHX34__NM_014681	268	277	243	280
DHX34__NM_014681_TAACGTGGGTCGTAAGTGCG	DHX34__NM_014681	382	340	422	332
DHX34__NM_014681_TCCTGGAGGTCTTGAGATTC	DHX34__NM_014681	322	460	473	559
DHX34__NM_014681_TGAAGAGGATTACATCCGTC	DHX34__NM_014681	256	269	240	251
DHX34__NM_014681_TGGCAGGGCCAAGAACAGAG	DHX34__NM_014681	83	64	83	76
DHX35__NM_001190809_AATGGCAATGTCAGTGTACA	DHX35__NM_001190809	1310	365	1471	387
DHX35__NM_001190809_ATCACACATGTATCCCTTGC	DHX35__NM_001190809	735	817	802	804
DHX35__NM_001190809_ATGTTCTCCCTTCCACTGTA	DHX35__NM_001190809	335	73	391	48
DHX35__NM_001190809_CAAATCAACTGTCGAAACTG	DHX35__NM_001190809	1091	1105	1186	1410
DHX35__NM_001190809_CAGCAGCCACTCTTCGAGGC	DHX35__NM_001190809	681	181	750	219
DHX35__NM_001190809_CCAGCTGGCCACGAGAATTA	DHX35__NM_001190809	7	0	11	1
DHX35__NM_001190809_CCGGTGAAGTTCTGGCGACC	DHX35__NM_001190809	518	117	647	76
DHX35__NM_001190809_GAAGTTCTGGCGACCCGGTA	DHX35__NM_001190809	227	1	220	29
DHX35__NM_001190809_GATTTGATATAATCTGGAAC	DHX35__NM_001190809	82	83	53	93
DHX35__NM_001190809_GCAGAAGCTGCCGGTATTCA	DHX35__NM_001190809	402	320	412	381
DHX35__NM_001190809_TCTTCAGAGATGCTTACACC	DHX35__NM_001190809	219	58	332	146
DHX35__NM_001190809_TGAAACCAGTGATCCAGCAA	DHX35__NM_001190809	143	153	178	233
DHX35__NM_021931_GATAGAAAATTATCAGACAG	DHX35__NM_021931	94	3	157	5
DHX35__NM_021931_TTGTTGGTGAAACAGGATGT	DHX35__NM_021931	288	54	248	17
DHX36__NM_001114397_AAGGCCGCGAAATCGGCATG	DHX36__NM_001114397	320	87	331	114
DHX36__NM_001114397_AGAGAAAAGCTGCCTTCGTA	DHX36__NM_001114397	888	115	985	237
DHX36__NM_001114397_ATGTGGTACGCGAAAAAACA	DHX36__NM_001114397	56	97	95	54
DHX36__NM_001114397_CATCAGGTAACAGTAATAAG	DHX36__NM_001114397	322	16	356	11
DHX36__NM_001114397_CATGTGGTACGCGAAAAAAC	DHX36__NM_001114397	123	46	134	206
DHX36__NM_001114397_CCTGCATTTCAATATACCGA	DHX36__NM_001114397	1149	696	1384	928
DHX36__NM_001114397_CCTTCGGTATATTGAAATGC	DHX36__NM_001114397	387	278	397	276
DHX36__NM_001114397_CGTACCACATGCCGATTTCG	DHX36__NM_001114397	875	937	1098	927
DHX36__NM_001114397_TATGACTACCATCAGAACTG	DHX36__NM_001114397	153	148	87	79
DHX36__NM_001114397_TGTGGTACGCGAAAAAACAG	DHX36__NM_001114397	45	62	47	3
DHX36__NM_001114397_TTCGCGGCCTTTCAGGTGCC	DHX36__NM_001114397	15	67	35	41
DHX36__NM_001114397_TTGGATAACTACATTGAAAG	DHX36__NM_001114397	163	150	107	156
DHX37__NM_032656_AAAGAGTCTCATCTCAGCTT	DHX37__NM_032656	344	116	320	76
DHX37__NM_032656_AAGCAACGCGCTCGTTCTAC	DHX37__NM_032656	233	72	255	45
DHX37__NM_032656_ACCACTTCCAAGCTAGGCAC	DHX37__NM_032656	216	242	290	160
DHX37__NM_032656_AGCAACGCGCTCGTTCTACC	DHX37__NM_032656	336	33	387	29
DHX37__NM_032656_CCAGCGGCGACGCTTCCGGT	DHX37__NM_032656	2334	984	2693	575
DHX37__NM_032656_GAGAAGATCAGTAGCCTCAG	DHX37__NM_032656	243	265	324	146
DHX37__NM_032656_GCAACGCGCTCGTTCTACCG	DHX37__NM_032656	1199	220	1252	153
DHX37__NM_032656_GCGGTTCCCAGTGCCTAGCT	DHX37__NM_032656	4	29	5	21
DHX37__NM_032656_GCTTCTTCTCCTTCTTCGAC	DHX37__NM_032656	556	837	790	950
DHX37__NM_032656_TTCTCCTTCTTCGACAGGGG	DHX37__NM_032656	3	5	4	0
DHX37__NM_032656_TTCTTCTCCTTCTTCGACAG	DHX37__NM_032656	10	2	8	2
DHX37__NM_032656_TTTCCGGGGAGCGGTTCACG	DHX37__NM_032656	38	6	63	7
DHX38__NM_014003_ACAGTGGCTATGGCTCCTCA	DHX38__NM_014003	589	457	828	528
DHX38__NM_014003_AGCAGCAGATCAGAGCGAGA	DHX38__NM_014003	122	181	141	42
DHX38__NM_014003_AGTCCAGTCCGAGTAATGAA	DHX38__NM_014003	224	18	279	98
DHX38__NM_014003_CAGCGAGCAGCATGTCTTCA	DHX38__NM_014003	501	554	654	558
DHX38__NM_014003_CCGCCCTTCATTACTCGGAC	DHX38__NM_014003	3620	4890	4098	5487
DHX38__NM_014003_CCTCCCAGGTAGACCTTGAA	DHX38__NM_014003	628	380	629	645
DHX38__NM_014003_CGCTCAGAATCCCGATAGGA	DHX38__NM_014003	285	152	360	276
DHX38__NM_014003_GAATCCCGATAGGAAGGCGT	DHX38__NM_014003	455	371	458	208
DHX38__NM_014003_GATCAGTGCCTTCCAATCGA	DHX38__NM_014003	463	288	525	254
DHX38__NM_014003_GCTCCGCTCAGAATCCCGAT	DHX38__NM_014003	633	360	1036	495
DHX38__NM_014003_GGATGCCTCGATCCATCGAT	DHX38__NM_014003	782	986	1006	1339
DHX38__NM_014003_TACCTTTAGGTCGATGTCGT	DHX38__NM_014003	831	1010	962	877
DHX40__NM_001166301_AAAAGATTATTCAAGCTGTG	DHX40__NM_001166301	44	145	47	140
DHX40__NM_001166301_AAATACTGCGTATATTCAAG	DHX40__NM_001166301	63	69	68	23
DHX40__NM_001166301_AGGATGTCCAACCCTAATCT	DHX40__NM_001166301	567	394	482	246
DHX40__NM_001166301_CTTCCAGGTATATCAAATAT	DHX40__NM_001166301	628	891	750	1078
DHX40__NM_001166301_GCAATATTAACAAGCCATCG	DHX40__NM_001166301	522	279	669	440
DHX40__NM_001166301_GCTGTTTGCATCGCCGATAG	DHX40__NM_001166301	257	248	203	348
DHX40__NM_001166301_GGATGTCCAACCCTAATCTG	DHX40__NM_001166301	471	261	435	312
DHX40__NM_001166301_GTTGTCATTGATCCATAACA	DHX40__NM_001166301	251	86	312	134
DHX40__NM_001166301_TGTCCAATATTTGATATACC	DHX40__NM_001166301	131	137	164	54
DHX40__NM_001166301_TGTTTCTGAATAGGAAAAGT	DHX40__NM_001166301	0	0	0	0
DHX40__NM_001166301_TTGCCATACACGATGTCATA	DHX40__NM_001166301	379	457	351	254
DHX40__NM_001166301_TTGTTAATATTGCCGTGTTA	DHX40__NM_001166301	88	91	182	163
DHX40__NM_024612_ACGAACTTGGTATCCTACTT	DHX40__NM_024612	269	176	330	337
DHX57__NM_198963_AAGGATCCAAGTCGGGAACG	DHX57__NM_198963	68	123	68	134
DHX57__NM_198963_AATGGGGAGACTGTAAATTC	DHX57__NM_198963	237	164	296	252
DHX57__NM_198963_AGTCATATGTAGAGTCTGAA	DHX57__NM_198963	152	54	109	116
DHX57__NM_198963_AGTTCTTCAGTAAGAAGAAA	DHX57__NM_198963	43	10	42	84
DHX57__NM_198963_ATCATCCCATATTCTACTGG	DHX57__NM_198963	682	565	659	619
DHX57__NM_198963_CAATACTGAACGCTGTCAAG	DHX57__NM_198963	780	796	880	999
DHX57__NM_198963_CGCTCATCGTTACAGCAATC	DHX57__NM_198963	308	643	430	684
DHX57__NM_198963_CTGAAGGGGTACTTTGGCTT	DHX57__NM_198963	612	765	831	927
DHX57__NM_198963_GAAAGGCCTCCAGTAGAATA	DHX57__NM_198963	191	130	144	171
DHX57__NM_198963_GCTAAGCCAGCATATTCCAA	DHX57__NM_198963	397	396	462	496
DHX57__NM_198963_GCTGTAACGATGAGCGGTAC	DHX57__NM_198963	576	613	457	601
DHX57__NM_198963_TCCAGTAGAATATGGGATGA	DHX57__NM_198963	514	509	455	701
DHX58__NM_024119_AGGGCAAGAATATCATCATC	DHX58__NM_024119	440	308	385	389
DHX58__NM_024119_AGGTGGTTGTATTGGTCAAC	DHX58__NM_024119	16	22	18	28
DHX58__NM_024119_CAAGCGGCACCTAGAGACTG	DHX58__NM_024119	952	1012	990	772
DHX58__NM_024119_CACAGTCTCTAGGTGCCGCT	DHX58__NM_024119	1010	843	864	1170
DHX58__NM_024119_GACCCGGGCGGCTGCTTATG	DHX58__NM_024119	4241	3970	4236	3947
DHX58__NM_024119_GCACTCATCCACCACGATCA	DHX58__NM_024119	1197	1312	1302	1223
DHX58__NM_024119_GCTTCGGTCCTACCAATGGG	DHX58__NM_024119	308	208	226	218
DHX58__NM_024119_GGAGCTTCGGTCCTACCAAT	DHX58__NM_024119	363	294	334	312
DHX58__NM_024119_GGCGCATGCTGGATGGACGC	DHX58__NM_024119	711	781	950	1093
DHX58__NM_024119_GGTGGTTGTATTGGTCAACA	DHX58__NM_024119	5	11	17	38
DHX58__NM_024119_TGGAGCCAAGGTGGTTGTAT	DHX58__NM_024119	0	0	0	0
DHX58__NM_024119_TGGAGCTTCGGTCCTACCAA	DHX58__NM_024119	105	60	119	63
DHX8__NM_004941_AGAAGCCTTAAAAGTATCAA	DHX8__NM_004941	50	9	82	32
DHX8__NM_004941_AGTTTGGCAAGTTCTTCCGC	DHX8__NM_004941	347	180	448	130
DHX8__NM_004941_CAAAGACAGGTACTCGAGTT	DHX8__NM_004941	1200	603	1446	958
DHX8__NM_004941_CCATGGCGGGAGCCTTAATC	DHX8__NM_004941	93	13	60	17
DHX8__NM_004941_CCTGGAACGTGATCGAGAGC	DHX8__NM_004941	556	289	431	195
DHX8__NM_004941_CGGAAGGACCGGGACAAATA	DHX8__NM_004941	363	260	300	214
DHX8__NM_004941_CTGAGCTGGACAATCACTTG	DHX8__NM_004941	596	79	742	59
DHX8__NM_004941_GAACAGAAGGGTTGTCCGGT	DHX8__NM_004941	974	229	1113	256
DHX8__NM_004941_GCCATGGCGGGAGCCTTAAT	DHX8__NM_004941	113	56	75	35
DHX8__NM_004941_GCCTGGCTCCGACCCGATTA	DHX8__NM_004941	911	134	860	162
DHX8__NM_004941_GGCGGGAGCCTTAATCGGGT	DHX8__NM_004941	322	205	312	156
DHX8__NM_004941_TGCGTCTCATACAAACCATG	DHX8__NM_004941	242	42	253	29
DHX9__NM_001357_AGAAAATAATTCTGAGGTAG	DHX9__NM_001357	121	315	141	100
DHX9__NM_001357_CAGAGACTTTGTTAACTATT	DHX9__NM_001357	506	311	548	484
DHX9__NM_001357_CAGGCAGAAATTCATGTGTG	DHX9__NM_001357	998	12	1120	15
DHX9__NM_001357_CCCCACTGCTCTAATTTCAT	DHX9__NM_001357	1358	41	1463	55
DHX9__NM_001357_GATTTACCAACAACCATGGG	DHX9__NM_001357	38	8	49	12
DHX9__NM_001357_GGAGATTTACCAACAACCAT	DHX9__NM_001357	113	16	128	18
DHX9__NM_001357_GGTTATAATTACACTGGCAT	DHX9__NM_001357	136	118	176	164
DHX9__NM_001357_GTCAGGAGTATCAGTAAGTG	DHX9__NM_001357	408	71	479	105
DHX9__NM_001357_GTGTCAGGAGTATCAGTAAG	DHX9__NM_001357	453	41	648	100
DHX9__NM_001357_TCCTATGAAATTAGAGCAGT	DHX9__NM_001357	193	24	185	35
DHX9__NM_001357_TGCTCTAATTTCATAGGATG	DHX9__NM_001357	76	51	138	61
DHX9__NM_001357_TGTCAGGAGTATCAGTAAGT	DHX9__NM_001357	351	7	279	38
DIABLO__NM_001278302_AGTAGAAGTCAGATTTCATA	DIABLO__NM_001278302	69	169	90	62
DIABLO__NM_001278302_CGACAATATACAAGTTTACT	DIABLO__NM_001278302	594	658	639	748
DIABLO__NM_001278302_GCAGAAGCTGCATATCAAAC	DIABLO__NM_001278302	397	590	399	549
DIABLO__NM_001278302_TGGGAAAATGAATTCAGAGG	DIABLO__NM_001278302	32	6	42	16
DIABLO__NM_001278303_AGCTTCAATCAACGCATATG	DIABLO__NM_001278303	405	430	485	287
DIABLO__NM_001278304_CATCAATGCTTCACTACTAA	DIABLO__NM_001278304	595	665	662	756
DIABLO__NM_001278304_TCATCAATGCTTCACTACTA	DIABLO__NM_001278304	820	1043	839	1133
DIABLO__NM_001278342_AAAACTGTGACGATTGGCTT	DIABLO__NM_001278342	405	402	498	689
DIABLO__NM_001278342_GTGTGCGGTTCCTATTGCAC	DIABLO__NM_001278342	513	376	409	270
DIABLO__NM_001278342_TCTCAGAATTGATAAGACCA	DIABLO__NM_001278342	211	188	248	110
DIABLO__NM_001278342_TGCAATAGGAACCGCACACA	DIABLO__NM_001278342	522	421	545	519
DIAPH1__NM_001079812_ACATGGTCTTGATTCCAAAC	DIAPH1__NM_001079812	221	113	278	127
DIAPH1__NM_001079812_AGAATTTCTATGAGCAGAAT	DIAPH1__NM_001079812	128	160	107	165
DIAPH1__NM_001079812_AGCTGCATCAATCATCATGT	DIAPH1__NM_001079812	1353	1350	1645	1738
DIAPH1__NM_001079812_CAACTCCTGAATATACATCA	DIAPH1__NM_001079812	696	699	748	655
DIAPH1__NM_001079812_CGTTTCCAGCCGCTGCTGGA	DIAPH1__NM_001079812	260	187	328	392
DIAPH1__NM_001079812_GGAGAAACAGCAACCTTTGA	DIAPH1__NM_001079812	481	431	565	432
DIAPH1__NM_001079812_GGAGTTGAGGTCAGGCTTGC	DIAPH1__NM_001079812	805	758	836	752
DIAPH1__NM_001079812_GTCTGCCATGATGTATATTC	DIAPH1__NM_001079812	242	140	219	118
DIAPH1__NM_001079812_GTTGTTGAGAGACACACGAA	DIAPH1__NM_001079812	154	158	131	49
DIAPH1__NM_001079812_TGAAGGCTTGGCCTCCTTAT	DIAPH1__NM_001079812	528	175	608	274
DIAPH1__NM_001079812_TGGAACCACTATTGCACTGA	DIAPH1__NM_001079812	50	122	88	54
DIAPH1__NM_001079812_TTTAAGAATGTCCAATAAGG	DIAPH1__NM_001079812	5	0	12	0
DIAPH2__NM_006729_AGTCATCATGTTGGGTTGTT	DIAPH2__NM_006729	972	1005	948	1052
DIAPH2__NM_006729_ATCGCGACTTGAGAATCAAT	DIAPH2__NM_006729	474	331	458	421
DIAPH2__NM_006729_ATTCGAAAATCAAGCTCATA	DIAPH2__NM_006729	130	198	109	148
DIAPH2__NM_006729_CTCAGCTGGGTTAACAACTT	DIAPH2__NM_006729	224	432	325	304
DIAPH2__NM_006729_CTGGACAACCATCTCACGTT	DIAPH2__NM_006729	1788	1699	1867	1471
DIAPH2__NM_006729_CTTTAACCAGCAATCCGGTC	DIAPH2__NM_006729	362	513	501	584
DIAPH2__NM_006729_CTTTACCACCAAACGTGAGA	DIAPH2__NM_006729	257	320	352	485
DIAPH2__NM_006729_GACAACCATCTCACGTTTGG	DIAPH2__NM_006729	1239	1557	1294	1162
DIAPH2__NM_006729_GGCGGCCCTACACCTGGCAA	DIAPH2__NM_006729	1215	1503	1389	1521
DIAPH2__NM_006729_TATGTTCATGAATTACGATC	DIAPH2__NM_006729	135	257	235	172
DIAPH2__NM_006729_TCAATAGGATGTTGAATAAG	DIAPH2__NM_006729	178	176	195	192
DIAPH2__NM_006729_TTACCTGGGTTCGAAGTTGC	DIAPH2__NM_006729	158	248	203	201
DIAPH3__NM_001042517_AAAGGCACCATTGCGGGAAA	DIAPH3__NM_001042517	123	211	146	145
DIAPH3__NM_001042517_AAGAGTTCATTCTCTGACAG	DIAPH3__NM_001042517	89	38	115	121
DIAPH3__NM_001042517_AATCATCAGGAGATGTAACC	DIAPH3__NM_001042517	250	122	238	175
DIAPH3__NM_001042517_AGAAATGAATTTATGCGTTG	DIAPH3__NM_001042517	343	375	364	459
DIAPH3__NM_001042517_ATCATCAGGAGATGTAACCA	DIAPH3__NM_001042517	116	115	160	109
DIAPH3__NM_001042517_CCACATCTGTCATCATATTG	DIAPH3__NM_001042517	932	672	1002	873
DIAPH3__NM_001042517_CTCTCTGCGGTATGCATTGT	DIAPH3__NM_001042517	415	603	399	464
DIAPH3__NM_001042517_CTCTGCGGTATGCATTGTAG	DIAPH3__NM_001042517	183	100	279	204
DIAPH3__NM_001042517_GATTGCTGGTCAAAGACACT	DIAPH3__NM_001042517	588	350	488	302
DIAPH3__NM_001042517_GCTCATCAAAGACTTTAAGT	DIAPH3__NM_001042517	441	272	559	352
DIAPH3__NM_001042517_TCTCTGCGGTATGCATTGTA	DIAPH3__NM_001042517	321	253	386	303
DIAPH3__NM_001042517_TGGAAAGCTTTGGACATGAA	DIAPH3__NM_001042517	68	18	106	48
DIAPH3__NM_001258367_AGCATATGATGTTTACAACA	DIAPH3__NM_001258367	301	181	250	292
DIAPH3__NM_001258370_CAGATTGTATTGCATAGAGA	DIAPH3__NM_001258370	88	91	151	117
DIAPH3__NM_001258370_CATCTCTATGCAATACAATC	DIAPH3__NM_001258370	153	257	163	326
DIAPH3__NM_001258370_GTTAAAGAAACTAGAGCAGA	DIAPH3__NM_001258370	65	53	91	127
DICER1__NM_001195573_AAATGGGAAATGTGATCCAG	DICER1__NM_001195573	220	103	230	111
DICER1__NM_001195573_AAGAGCTGTCCTATCAGATC	DICER1__NM_001195573	208	46	260	63
DICER1__NM_001195573_ACCATCGTCTGTTTAAACAC	DICER1__NM_001195573	730	642	801	413
DICER1__NM_001195573_ACCTTCATAATTTCTCGATA	DICER1__NM_001195573	897	826	842	894
DICER1__NM_001195573_AGAGCTGTCCTATCAGATCA	DICER1__NM_001195573	340	154	414	127
DICER1__NM_001195573_GAAGTCTCCCCTGATCTGAT	DICER1__NM_001195573	814	160	939	207
DICER1__NM_001195573_GAGCTGTCCTATCAGATCAG	DICER1__NM_001195573	193	31	230	47
DICER1__NM_001195573_GCAGTTAGTCCCAAAATGCG	DICER1__NM_001195573	184	37	228	20
DICER1__NM_001195573_GCCAGTGTTTAAACAGACGA	DICER1__NM_001195573	264	295	302	256
DICER1__NM_001195573_TACCTTCATAATTTCTCGAT	DICER1__NM_001195573	675	919	651	898
DICER1__NM_001195573_TCATTCAGATCTCAAGGTTG	DICER1__NM_001195573	38	3	124	0
DICER1__NM_001195573_TTATACGCCAAGAAAATATC	DICER1__NM_001195573	120	26	91	132
DIDO1__NM_001193369_CAAAGGCGACCCGAGCAATG	DIDO1__NM_001193369	592	453	732	724
DIDO1__NM_001193369_CCTGTTCCCGCTTCCTGCGA	DIDO1__NM_001193369	934	1066	1266	1334
DIDO1__NM_001193369_CCTTCGCAGGAAGCGGGAAC	DIDO1__NM_001193369	476	418	509	925
DIDO1__NM_001193369_CTCCACGCGCTCAGTGCGCT	DIDO1__NM_001193369	37	39	39	86
DIDO1__NM_001193369_GACGCCGAGACAGCCTCCGA	DIDO1__NM_001193369	1738	1061	1879	1564
DIDO1__NM_001193369_GAGCAATGAGGAGGCACCTA	DIDO1__NM_001193369	277	172	313	312
DIDO1__NM_001193369_GCCATCGCTGTCACTATCGG	DIDO1__NM_001193369	1657	2488	1787	2313
DIDO1__NM_001193369_GCCCTCTCGCTTGGCGATAG	DIDO1__NM_001193369	338	281	372	319
DIDO1__NM_001193369_GGGCGTCCTGCCCAGTAAGC	DIDO1__NM_001193369	57	29	155	139
DIDO1__NM_001193369_GTTCTCGGGCTCCTGCTTAC	DIDO1__NM_001193369	85	110	137	90
DIDO1__NM_001193369_TCCACGCGCTCAGTGCGCTT	DIDO1__NM_001193369	109	20	120	101
DIDO1__NM_001193369_TTCTCGGGCTCCTGCTTACT	DIDO1__NM_001193369	55	25	79	3
DIEXF__NM_014388_AAAGCCACAGATTTGTCAAC	DIEXF__NM_014388	344	68	331	33
DIEXF__NM_014388_AAGAAACATCTTCGAGATTT	DIEXF__NM_014388	120	107	142	110
DIEXF__NM_014388_ACATCTTCGAGATTTCGGCG	DIEXF__NM_014388	768	499	862	463
DIEXF__NM_014388_ACCAACAGCCAGTTCCTATC	DIEXF__NM_014388	1956	695	1938	520
DIEXF__NM_014388_ACTTAGAGGAAAAGAAAAGC	DIEXF__NM_014388	71	4	133	48
DIEXF__NM_014388_CAGTCCTTTCCGGGTAGAAC	DIEXF__NM_014388	482	193	572	223
DIEXF__NM_014388_GGGGATGTTTGTGATGTGCC	DIEXF__NM_014388	404	746	418	623
DIEXF__NM_014388_TCATTGTGAGCAACAAAAAG	DIEXF__NM_014388	16	32	58	32
DIEXF__NM_014388_TCTTCTTTGAAAGCCTCTCA	DIEXF__NM_014388	180	212	176	196
DIEXF__NM_014388_TGGTGGTAGCGATGTCAGTG	DIEXF__NM_014388	14	30	4	0
DIEXF__NM_014388_TTACCGGGACCTGTTCTACC	DIEXF__NM_014388	244	0	207	0
DIEXF__NM_014388_TTGCATCTGTGAACTCTTCG	DIEXF__NM_014388	272	309	262	488
DIMT1__NM_014473_ACTTCCAGCACTACATCAGT	DIMT1__NM_014473	564	18	624	9
DIMT1__NM_014473_ACTTGAAGTTTGCTGGCCAC	DIMT1__NM_014473	622	273	921	497
DIMT1__NM_014473_AGCAAACTTCAAGTACTGGT	DIMT1__NM_014473	1040	163	1163	112
DIMT1__NM_014473_CACTACATCAGTTGGTCTTA	DIMT1__NM_014473	967	194	1277	141
DIMT1__NM_014473_CAGGACTCATGTTCAACACG	DIMT1__NM_014473	643	199	651	218
DIMT1__NM_014473_CTCATGTTCAACACGGGGAT	DIMT1__NM_014473	259	565	267	410
DIMT1__NM_014473_GCCATTCTTTGATACTTGTG	DIMT1__NM_014473	1256	1201	1521	1804
DIMT1__NM_014473_GGCCAGCAAACTTCAAGTAC	DIMT1__NM_014473	961	634	1216	1200
DIMT1__NM_014473_TAACTTCAGACCACCGCCCA	DIMT1__NM_014473	675	221	810	227
DIMT1__NM_014473_TACACAGCTGTTGGCACGTG	DIMT1__NM_014473	1030	19	1137	112
DIMT1__NM_014473_TCATGTTCAACACGGGGATT	DIMT1__NM_014473	100	94	137	94
DIMT1__NM_014473_TTGCTTGTGAACTTGACCCA	DIMT1__NM_014473	266	42	299	62
DIO1__NM_000792_AACATTTGGGAGTTTATGCA	DIO1__NM_000792	265	357	380	315
DIO1__NM_000792_AATGACAAGAAAATCTGCTA	DIO1__NM_000792	151	33	228	135
DIO1__NM_000792_CACGACTGAGCTAGGGGGTC	DIO1__NM_000792	782	1095	1003	1337
DIO1__NM_000792_CAGGACAGAGGTGCAACATT	DIO1__NM_000792	483	326	479	413
DIO1__NM_000792_GAAAAGCTCCACAGTTAATC	DIO1__NM_000792	332	512	413	478
DIO1__NM_000792_GCTCCTGGCCAGCAGTAGAT	DIO1__NM_000792	275	226	328	392
DIO1__NM_000792_GGTTCGTCTTGAAGGTCCGT	DIO1__NM_000792	124	118	97	66
DIO1__NM_000792_GTATTTCTGGTTCGTCTTGA	DIO1__NM_000792	46	82	67	44
DIO1__NM_000792_TCTGGCCCCAAACTGCCCGG	DIO1__NM_000792	1015	700	1035	671
DIO1__NM_000792_TGTTTCCAGACAGAGTCAAG	DIO1__NM_000792	7	14	9	23
DIO2__NM_000793_AATAACGCCAACATAGCTTA	DIO2__NM_000793	0	0	0	0
DIO2__NM_000793_AGCCGCTCCAAGTCCACTCG	DIO2__NM_000793	1217	1222	1387	1884
DIO2__NM_000793_AGGCTCATCCATCAGATGGC	DIO2__NM_000793	136	111	140	179
DIO2__NM_000793_ATCAGATGGCTGGGCGATAC	DIO2__NM_000793	290	191	286	344
DIO2__NM_000793_CAACAGTGGCAATGGTACCC	DIO2__NM_000793	650	624	490	328
DIO2__NM_000793_CGGCGCATGCTGACCTCAGA	DIO2__NM_000793	550	913	840	976
DIO2__NM_000793_GAGCCAAAGTTGACCACTAG	DIO2__NM_000793	513	584	605	774
DIO2__NM_000793_GGCAGCTGGCTCGTGAAAGG	DIO2__NM_000793	263	256	240	273
DIO2__NM_000793_GGCTCATCCATCAGATGGCT	DIO2__NM_000793	251	460	343	412
DIO2__NM_000793_TCAGATGGCTGGGCGATACC	DIO2__NM_000793	337	474	305	308
DIO2__NM_000793_TGATCAGCAAGTCTACGCTG	DIO2__NM_000793	492	585	645	673
DIO2__NM_000793_TGTTGGCGTTATTGTCCATG	DIO2__NM_000793	357	510	505	673
DIO2__NM_001007023_ACATTTGGATTTATGGATGG	DIO2__NM_001007023	517	609	545	742
DIO2__NM_001007023_ACTACTGGTCTACTCACATT	DIO2__NM_001007023	4088	3814	4346	4353
DIO2__NM_001007023_AGCTTATAAAAGCAGACTAC	DIO2__NM_001007023	146	129	157	198
DIO2__NM_001007023_TCTACTCACATTTGGATTTA	DIO2__NM_001007023	245	281	235	346
DIO2__NM_001242502_AAACACAGACTAATTTGCCT	DIO2__NM_001242502	756	1123	749	936
DIO3__NM_001362_ACACCGCCTTGAGCGACGCC	DIO3__NM_001362	683	871	837	941
DIO3__NM_001362_ACGCAGATGGGCGGGTCATC	DIO3__NM_001362	979	1415	1047	1178
DIO3__NM_001362_CACGAGGCACGAGGCGGTCT	DIO3__NM_001362	1245	1339	1509	1548
DIO3__NM_001362_CCAGGCCACGTCGCGGTTGG	DIO3__NM_001362	1099	1373	1217	1539
DIO3__NM_001362_CGGGAAGAGCACGAGGCACG	DIO3__NM_001362	752	1043	952	898
DIO3__NM_001362_GTGCACCCTGGCGTCGCTCA	DIO3__NM_001362	551	823	761	719
DIO3__NM_001362_GTTGAGCTCCACTTCGGGCT	DIO3__NM_001362	968	1073	1033	1274
DIO3__NM_001362_TCGCCAGGCCACGTCGCGGT	DIO3__NM_001362	2767	2578	3051	3107
DIO3__NM_001362_TGCCTCGCCAGGCCACGTCG	DIO3__NM_001362	637	674	689	585
DIO3__NM_001362_TGGCGTCGCTCAAGGCGGTG	DIO3__NM_001362	51	5	42	54
DIO3__NM_001362_TTGAGCTCCACTTCGGGCTC	DIO3__NM_001362	3	0	15	13
DIO3__NM_001362_TTGTCGTCGGACACGCAGAT	DIO3__NM_001362	297	396	245	350
DIP2A__NM_001146115_CGTTATATACCGCTTATTCA	DIP2A__NM_001146115	766	607	869	904
DIP2A__NM_001146115_GAAGCGCTCATCCCTCGAGG	DIP2A__NM_001146115	558	453	601	560
DIP2A__NM_001146115_TGCAGCGGCCGTGGTTTGTG	DIP2A__NM_001146115	1242	1559	1448	1724
DIP2B__NM_173602_AAGTACCACCGAACTCGATC	DIP2B__NM_173602	496	324	453	427
DIP2B__NM_173602_AGCTGCGCCCGCACTTCAGG	DIP2B__NM_173602	211	113	116	78
DIP2B__NM_173602_AGGGATGAACGATATCGATC	DIP2B__NM_173602	70	90	72	25
DIP2B__NM_173602_AGTACCACCGAACTCGATCT	DIP2B__NM_173602	807	690	926	501
DIP2B__NM_173602_CCCCCCTCGGGGATAGTTAA	DIP2B__NM_173602	422	416	355	323
DIP2B__NM_173602_CGAGTTCGGTGGTACTTAGA	DIP2B__NM_173602	165	195	208	96
DIP2B__NM_173602_CTCCCCCAGATCGAGTTCGG	DIP2B__NM_173602	1032	693	1016	941
DIP2B__NM_173602_CTGGATCAACCGTTCAATTC	DIP2B__NM_173602	326	477	352	393
DIP2B__NM_173602_GAGGTAGTTGTAGTGACATC	DIP2B__NM_173602	152	194	158	87
DIP2B__NM_173602_GCATCTTCTACGCTGTCCCA	DIP2B__NM_173602	873	725	890	608
DIP2B__NM_173602_GTATTTGCCAATACTCGAAT	DIP2B__NM_173602	364	547	505	426
DIP2B__NM_173602_TACCACCGAACTCGATCTGG	DIP2B__NM_173602	961	555	993	322
DIP2C__NM_014974_AAGCTTTGCCGCAAGAACGC	DIP2C__NM_014974	132	203	246	244
DIP2C__NM_014974_CATCGAGGTCTGCACGACCA	DIP2C__NM_014974	759	573	786	942
DIP2C__NM_014974_CCACAGGCTGGCGGACGTCA	DIP2C__NM_014974	315	241	425	594
DIP2C__NM_014974_CCGTCGGCGGTGGTAGCGAG	DIP2C__NM_014974	182	99	165	221
DIP2C__NM_014974_CGAGATGAGCGCTATCGGTC	DIP2C__NM_014974	815	818	1172	1178
DIP2C__NM_014974_CTCGTCCTCTACGCAGAGCG	DIP2C__NM_014974	108	228	157	243
DIP2C__NM_014974_CTCGTGACCCTGAAGACCGT	DIP2C__NM_014974	334	308	359	394
DIP2C__NM_014974_CTCTGGCCAAACACAAAGAG	DIP2C__NM_014974	20	0	25	11
DIP2C__NM_014974_GAGGCGGAGGAAGGAGTGAC	DIP2C__NM_014974	591	417	542	605
DIP2C__NM_014974_GAGGTGTACGTGGTTACGTC	DIP2C__NM_014974	229	353	277	266
DIP2C__NM_014974_GGTCACGAGATGAGCGCTAT	DIP2C__NM_014974	391	561	492	553
DIP2C__NM_014974_GTGACCCTGAAGACCGTCGG	DIP2C__NM_014974	305	444	453	406
DIRAS1__NM_145173_ACAGAGTAACGATTACCGCG	DIRAS1__NM_145173	772	860	725	615
DIRAS1__NM_145173_CCACCATCGAGGACACCTAC	DIRAS1__NM_145173	844	851	1089	971
DIRAS1__NM_145173_CGGCGTGGGCAAGAGCTCGC	DIRAS1__NM_145173	265	146	329	371
DIRAS1__NM_145173_CGTCACCAGCAAGCAGTCGC	DIRAS1__NM_145173	346	428	383	404
DIRAS1__NM_145173_CTGGTGCTGCGCTTCGTGAA	DIRAS1__NM_145173	226	236	249	226
DIRAS1__NM_145173_CTTGTTGCCCACGAGCATCA	DIRAS1__NM_145173	322	418	271	319
DIRAS1__NM_145173_GACATCCCCGTGATGCTCGT	DIRAS1__NM_145173	531	342	604	603
DIRAS1__NM_145173_GAGTAACGATTACCGCGTGG	DIRAS1__NM_145173	1089	678	1019	941
DIRAS1__NM_145173_GCTGGTGCTGCGCTTCGTGA	DIRAS1__NM_145173	671	950	856	710
DIRAS1__NM_145173_TGGTGGGGATGTAGGTGTCG	DIRAS1__NM_145173	5	13	9	2
DIRAS1__NM_145173_TGTTGCCCACGAGCATCACG	DIRAS1__NM_145173	421	381	358	329
DIRAS1__NM_145173_TTGTTGCCCACGAGCATCAC	DIRAS1__NM_145173	471	572	488	609
DIRAS2__NM_017594_ACAGCTGATCACTTGCCGGT	DIRAS2__NM_017594	4414	3709	5153	3854
DIRAS2__NM_017594_CAAGGACTGTCGGCTGGTAA	DIRAS2__NM_017594	828	728	921	908
DIRAS2__NM_017594_CCGGGAGAGCTACATCCCGA	DIRAS2__NM_017594	480	279	589	520
DIRAS2__NM_017594_CTGAGCAGAGTAACGATTAC	DIRAS2__NM_017594	344	553	347	456
DIRAS2__NM_017594_CTGGTGGCTCCCCGTCGTGT	DIRAS2__NM_017594	847	1216	966	1144
DIRAS2__NM_017594_CTGGTGTTGAGGTTTGTGAA	DIRAS2__NM_017594	180	217	174	208
DIRAS2__NM_017594_GCAGAGTAACGATTACCGGG	DIRAS2__NM_017594	371	207	508	222
DIRAS2__NM_017594_TCGCAGATTTGTTCGTAGAT	DIRAS2__NM_017594	504	588	474	670
DIRAS2__NM_017594_TGAAGGCACACTTCCATGTG	DIRAS2__NM_017594	484	731	625	834
DIRAS2__NM_017594_TGAGCAGAGTAACGATTACC	DIRAS2__NM_017594	209	144	330	376
DIRAS2__NM_017594_TGCAGATCACCGACACGACG	DIRAS2__NM_017594	223	168	347	313
DIRAS2__NM_017594_TTGCAGATCACCGACACGAC	DIRAS2__NM_017594	313	516	382	455
DIRAS3__NM_004675_AAGCTGGCGTTACCCATCGT	DIRAS3__NM_004675	919	1179	939	1011
DIRAS3__NM_004675_ACCGACAGCAAGAGTGGCGA	DIRAS3__NM_004675	598	540	701	785
DIRAS3__NM_004675_CAGCGCCACGTTATAGCCCG	DIRAS3__NM_004675	928	1033	1009	1146
DIRAS3__NM_004675_GATCAGAGATTACCGCGTCG	DIRAS3__NM_004675	424	298	356	519
DIRAS3__NM_004675_GATTACCGCGTCGTGGTAGT	DIRAS3__NM_004675	402	563	616	483
DIRAS3__NM_004675_GCGCGGAGGATAAGCAGGGC	DIRAS3__NM_004675	257	183	246	297
DIRAS3__NM_004675_GTAGTCGGCACCGCTGGTGT	DIRAS3__NM_004675	703	525	735	572
DIRAS3__NM_004675_GTGCCACCTGTGCGATGGAG	DIRAS3__NM_004675	714	1031	816	1054
DIRAS3__NM_004675_TCTTGCTGTCGGTGATGTGC	DIRAS3__NM_004675	484	525	518	658
DIRAS3__NM_004675_TCTTGGTGACTGAGTAGACC	DIRAS3__NM_004675	450	248	454	341
DIRAS3__NM_004675_TGCAGCGCCACGTTATAGCC	DIRAS3__NM_004675	134	152	171	265
DIRAS3__NM_004675_TGGTCGGCAGGTACTCATGA	DIRAS3__NM_004675	24	4	6	15
DIRC1__NM_052952_AAGTGTCTTAACTCATTGAG	DIRC1__NM_052952	262	210	343	326
DIRC1__NM_052952_AGTGTCTTAACTCATTGAGG	DIRC1__NM_052952	128	37	245	129
DIRC1__NM_052952_GAAAGTTGATCCTTTGTTAT	DIRC1__NM_052952	363	737	484	488
DIRC1__NM_052952_GACCTCTATACACATGGGGT	DIRC1__NM_052952	417	344	410	574
DIRC1__NM_052952_GCTGAGTGGTGGCAAGTAAC	DIRC1__NM_052952	448	658	598	867
DIRC1__NM_052952_GGTCTGAATTGAATACTGTG	DIRC1__NM_052952	444	401	557	607
DIRC1__NM_052952_GTAATTAGGTAAGGCTTAGT	DIRC1__NM_052952	221	89	210	204
DIRC1__NM_052952_TCTGGACCTCTATACACATG	DIRC1__NM_052952	1005	809	934	969
DIRC2__NM_032839_AAGAAGAGGTGATGTCCCAT	DIRC2__NM_032839	474	653	410	402
DIRC2__NM_032839_AGTTCCCAAAACCATAAGGA	DIRC2__NM_032839	311	311	246	233
DIRC2__NM_032839_CACCATTTCTCTCTACGACG	DIRC2__NM_032839	1109	1401	1389	1637
DIRC2__NM_032839_CAGACTTAATCCTTAAAAGA	DIRC2__NM_032839	224	383	300	405
DIRC2__NM_032839_CTCGCTGCTGGCGTTCGTTC	DIRC2__NM_032839	748	984	972	1223
DIRC2__NM_032839_GAGCCACATGAACGCGAAGC	DIRC2__NM_032839	13	1	3	2
DIRC2__NM_032839_GCATATTAAAGATCGCATAG	DIRC2__NM_032839	48	149	64	71
DIRC2__NM_032839_GCATCAATGCTCAGTTATCT	DIRC2__NM_032839	1805	2342	1702	2193
DIRC2__NM_032839_TCAATGCTCAGTTATCTTGG	DIRC2__NM_032839	602	551	681	555
DIRC2__NM_032839_TCGCTGCTGGCGTTCGTTCA	DIRC2__NM_032839	944	1236	1127	1486
DIRC2__NM_032839_TGCCCTGCTTCGCGTTCATG	DIRC2__NM_032839	369	401	356	370
DIRC2__NM_032839_TGGCGCGCCGAGTTCTGGAT	DIRC2__NM_032839	305	240	342	272
DIS3__NM_001128226_AAGCCTACAGGTAGAGTTGT	DIS3__NM_001128226	362	6	441	14
DIS3__NM_001128226_AGAAGAAGAGACAGAACGAA	DIS3__NM_001128226	45	22	42	5
DIS3__NM_001128226_AGCTGTTCACGAAGATATTG	DIS3__NM_001128226	416	21	446	15
DIS3__NM_001128226_CAAGACGATCTATGAGTTCG	DIS3__NM_001128226	909	179	999	239
DIS3__NM_001128226_CCCTTAAGTAAGCTACAGCA	DIS3__NM_001128226	354	25	447	53
DIS3__NM_001128226_CCTTGCTGTAGCTTACTTAA	DIS3__NM_001128226	420	43	437	79
DIS3__NM_001128226_CGCAGCCGATGTCGTCTCGC	DIS3__NM_001128226	2262	187	2461	192
DIS3__NM_001128226_GAGAAAATGTTGAAGCCTAC	DIS3__NM_001128226	470	185	513	142
DIS3__NM_001128226_GCAGCAAGTAGTGCGGTTGC	DIS3__NM_001128226	1042	546	1072	540
DIS3__NM_001128226_GCCTTGCTGTAGCTTACTTA	DIS3__NM_001128226	679	143	935	252
DIS3__NM_001128226_TCCCTGTTCTTGTTCTACAT	DIS3__NM_001128226	357	6	406	10
DIS3__NM_001128226_TGAGTTCGGGGTTAGCAGTT	DIS3__NM_001128226	69	9	118	10
DIS3__NM_014953_ATCTCGGATGCGTTTATATA	DIS3__NM_014953	213	3	280	0
DIS3__NM_014953_CTCGGATGCGTTTATATACG	DIS3__NM_014953	235	6	242	55
DIS3__NM_014953_CTTGGTTATTAGTCACATCT	DIS3__NM_014953	480	50	623	27
DIS3__NM_014953_TCGGATGCGTTTATATACGG	DIS3__NM_014953	291	6	326	3
DIS3L__NM_001143688_AGCAATGCTGCTATCTGCCA	DIS3L__NM_001143688	754	558	751	781
DIS3L__NM_001143688_ATTCTATCCTTCAGTCTCGA	DIS3L__NM_001143688	188	151	134	200
DIS3L__NM_001143688_CCCCTGGAAGTGTTAGAAGC	DIS3L__NM_001143688	421	444	500	443
DIS3L__NM_001143688_CCCTGGAAGTGTTAGAAGCT	DIS3L__NM_001143688	565	417	530	496
DIS3L__NM_001143688_CCTTGAGTTTCCTGAGTTGA	DIS3L__NM_001143688	741	1080	851	1105
DIS3L__NM_001143688_CTCTCTCCCGTCGAGACTGA	DIS3L__NM_001143688	125	142	125	103
DIS3L__NM_001143688_GATAGAATCACAAAGCTCGT	DIS3L__NM_001143688	14	14	50	57
DIS3L__NM_001143688_GGACAGGATGCCAATTGTTA	DIS3L__NM_001143688	729	506	859	558
DIS3L__NM_001143688_GGATAGAATCACAAAGCTCG	DIS3L__NM_001143688	75	33	61	85
DIS3L__NM_001143688_TAAATCTGGACGCTATATCC	DIS3L__NM_001143688	119	120	153	95
DIS3L__NM_001143688_TCTTCTATCCTTCAGTCTCGAC	DIS3L__NM_001143688	139	154	199	163
DIS3L__NM_001143688_TTCTATCCTTCAGTCTCGAC	DIS3L__NM_001143688	0	0	5	3
DIS3L__NM_133375_GTCGAACAAAAGCTTCTATT	DIS3L__NM_133375	796	811	839	831
DIS3L2__NM_001257281_AAACATTCTCGAGCAGCAAC	DIS3L2__NM_001257281	199	270	217	330
DIS3L2__NM_001257281_ATAAGAACAGCGAACTGTTT	DIS3L2__NM_001257281	458	482	499	684
DIS3L2__NM_001257281_CATGACATTGGTGCTTCGCC	DIS3L2__NM_001257281	1407	1215	1249	1485
DIS3L2__NM_001257281_CTCGTAATAGAGCCTTAAAT	DIS3L2__NM_001257281	142	267	122	194
DIS3L2__NM_001257281_GACCACCAGATCCCCATTTA	DIS3L2__NM_001257281	784	753	773	655
DIS3L2__NM_001257281_GCTTCCCGAGGAGCATTGGA	DIS3L2__NM_001257281	757	855	935	886
DIS3L2__NM_001257281_GTATGAATCAGATATCCCCG	DIS3L2__NM_001257281	524	306	617	532
DIS3L2__NM_001257281_GTGGCTGGTCCACATGACAT	DIS3L2__NM_001257281	109	51	112	82
DIS3L2__NM_001257281_TAGAGCCTTAAATGGGGATC	DIS3L2__NM_001257281	52	30	48	55
DIS3L2__NM_001257281_TAGTCAGGATGGCTCATTAT	DIS3L2__NM_001257281	5	18	39	20
DIS3L2__NM_001257281_TCAAAGAACAGGTCCACACG	DIS3L2__NM_001257281	436	456	572	515
DIS3L2__NM_001257281_TCGTAATAGAGCCTTAAATG	DIS3L2__NM_001257281	235	190	220	352
DIS3L2__NM_001257282_GTGAAAGCCATTTGGACACG	DIS3L2__NM_001257282	909	1199	1077	1390
DISC1__NM_001012957_AAAGCTAAAGCTTGAGGTAA	DISC1__NM_001012957	39	90	64	27
DISC1__NM_001012957_AGAGGAACCTCGGCGCACTT	DISC1__NM_001012957	1407	1816	1739	1859
DISC1__NM_001012957_AGTCCCACCACTCGGAGTCC	DISC1__NM_001012957	621	629	808	561
DISC1__NM_001012957_ATGCATGTCACGCTCTGGCC	DISC1__NM_001012957	423	534	463	459
DISC1__NM_001012957_CACGGCCAGCTAAGCCTGTC	DISC1__NM_001012957	997	1140	1259	1133
DISC1__NM_001012957_CAGTGTGGCCTTGACTCGAG	DISC1__NM_001012957	2275	2256	2858	2779
DISC1__NM_001012957_CATGCATTCTTTACCAGACA	DISC1__NM_001012957	1124	1258	1234	1335
DISC1__NM_001012957_CCAAGAGGCCTCTCGAGTCA	DISC1__NM_001012957	372	355	430	467
DISC1__NM_001012957_CCTTCAGTCTCTTGGCTACA	DISC1__NM_001012957	781	934	951	1032
DISC1__NM_001012957_CTTCAGTCTCTTGGCTACAC	DISC1__NM_001012957	322	173	310	216
DISC1__NM_001012957_GGCTACATGAGAAGCTCGAC	DISC1__NM_001012957	279	317	399	505
DISC1__NM_001012957_TCTCTGGATCCCTCACTGGC	DISC1__NM_001012957	830	904	986	1008
DISC1__NM_001164556_AAGCCAGTCTCGTCTCGTGA	DISC1__NM_001164556	443	223	497	423
DISC1__NM_001164556_CAGAGCCACCGGAAACCATA	DISC1__NM_001164556	172	144	209	198
DISC1__NM_001164556_GGTGTGGGTGTCATCTCCGC	DISC1__NM_001164556	805	449	995	739
DISC1__NM_001164556_TCCGGTGGCTCTGCATGGAA	DISC1__NM_001164556	81	66	85	96
DISP1__NM_032890_AACAGCACATAGCCAACATA	DISP1__NM_032890	207	208	211	129
DISP1__NM_032890_ATGCAGCCCTGATAGCCGAC	DISP1__NM_032890	493	616	649	665
DISP1__NM_032890_CAGCATTGGGGTAACATCTG	DISP1__NM_032890	309	263	360	494
DISP1__NM_032890_CCTTCTGCCTGCATCATCCG	DISP1__NM_032890	640	633	735	729
DISP1__NM_032890_GACTGCTATGGCTAGTCAAA	DISP1__NM_032890	236	300	354	325
DISP1__NM_032890_GATGCAGAATACTCGGAGTG	DISP1__NM_032890	878	1393	1018	1389
DISP1__NM_032890_GGCATTCTTTGGTTGTCTAA	DISP1__NM_032890	243	367	401	548
DISP1__NM_032890_GGGACTCGGGTTAGCAGCAC	DISP1__NM_032890	417	494	481	492
DISP1__NM_032890_TGACCGTGCCATTAAGTTGC	DISP1__NM_032890	1112	1307	1167	1487
DISP1__NM_032890_TGACTGCTATGGCTAGTCAA	DISP1__NM_032890	341	410	287	362
DISP1__NM_032890_TGATACACAGGTGAATGATT	DISP1__NM_032890	331	237	327	416
DISP1__NM_032890_TTAAGTTGCAGGCATCCATT	DISP1__NM_032890	274	537	255	193
DISP2__NM_033510_AAGGGGAGCAACGGCCCGAG	DISP2__NM_033510	245	233	180	245
DISP2__NM_033510_ACATGGTGCTGCACAGCGGG	DISP2__NM_033510	136	230	193	209
DISP2__NM_033510_AGCAATCAGCTGGGAATAGC	DISP2__NM_033510	136	301	166	198
DISP2__NM_033510_AGGTCCCATCGCGCTGTGAG	DISP2__NM_033510	603	629	651	794
DISP2__NM_033510_CAGCCCCCTCACAGCGCGAT	DISP2__NM_033510	495	321	602	656
DISP2__NM_033510_CAGGTCCCATCGCGCTGTGA	DISP2__NM_033510	2986	2668	3131	2164
DISP2__NM_033510_CCAGGTCCCATCGCGCTGTG	DISP2__NM_033510	1687	1997	1895	2250
DISP2__NM_033510_GAAGGGGAGCAACGGCCCGA	DISP2__NM_033510	590	747	700	699
DISP2__NM_033510_GGAAGGGGAGCAACGGCCCG	DISP2__NM_033510	520	528	502	564
DISP2__NM_033510_GTGCCCGGGGATAGGTGAAG	DISP2__NM_033510	115	84	156	96
DISP2__NM_033510_TGTCCGGGGAGCCGCCGTCT	DISP2__NM_033510	757	723	876	634
DISP2__NM_033510_TTGGGAGCAAGTTAGTGGTC	DISP2__NM_033510	253	228	235	236
DIXDC1__NM_001037954_AAAGACATCCCGTCCTGATC	DIXDC1__NM_001037954	578	482	561	440
DIXDC1__NM_001037954_ACAGCAGGAGAAAAGCTGAG	DIXDC1__NM_001037954	456	263	480	432
DIXDC1__NM_001037954_ATGACATGTCCCGATCAGGA	DIXDC1__NM_001037954	419	342	447	494
DIXDC1__NM_001037954_ATGTCTTTCGATATAGACAG	DIXDC1__NM_001037954	119	151	196	294
DIXDC1__NM_001037954_CAGGACGGTGAACCAAGGAC	DIXDC1__NM_001037954	169	175	230	154
DIXDC1__NM_001037954_CATCCCGGAGATCTTGTCGC	DIXDC1__NM_001037954	4770	4838	5345	5058
DIXDC1__NM_001037954_CTGCGACAAGATCTCCGGGA	DIXDC1__NM_001037954	691	575	929	790
DIXDC1__NM_001037954_GAAACCTGAAGTCTATCATG	DIXDC1__NM_001037954	240	251	293	293
DIXDC1__NM_001037954_GCATATCTCATCGAGATTGT	DIXDC1__NM_001037954	352	469	437	421
DIXDC1__NM_001037954_GCTGAGTCCCGGTAACCAAC	DIXDC1__NM_001037954	1242	1235	1183	1322
DIXDC1__NM_001037954_TGACATGTCCCGATCAGGAC	DIXDC1__NM_001037954	344	517	421	398
DIXDC1__NM_001037954_TGTCATGACATGTCCCGATC	DIXDC1__NM_001037954	252	118	240	242
DIXDC1__NM_001278542_TATTGAATCTCATAACCACT	DIXDC1__NM_001278542	584	465	574	768
DIXDC1__NM_033425_ATTCAGGCCTTACTGCTCAA	DIXDC1__NM_033425	266	260	279	277
DIXDC1__NM_033425_ATTCTCCATTATCTCGAGAC	DIXDC1__NM_033425	101	68	80	142
DIXDC1__NM_033425_CTCCATTATCTCGAGACTGG	DIXDC1__NM_033425	147	185	198	245
DIXDC1__NM_033425_CTCTTCTGACTGGGTTATAA	DIXDC1__NM_033425	80	104	61	165
DIXDC1__NM_033425_CTTTGCACTGTGGATTGGAC	DIXDC1__NM_033425	453	459	519	729
DIXDC1__NM_033425_GAAAATGATATCAGGACTAC	DIXDC1__NM_033425	207	312	229	313
DIXDC1__NM_033425_GAAGCAAAGAAAATGATATC	DIXDC1__NM_033425	349	178	401	427
DIXDC1__NM_033425_GGCCGCCAGTCTCGAGATAA	DIXDC1__NM_033425	750	852	734	1091
DIXDC1__NM_033425_TACCTGAAGATGAACAGGAG	DIXDC1__NM_033425	158	158	199	209
DIXDC1__NM_033425_TGAACCAGGTGTCAATCCCG	DIXDC1__NM_033425	187	141	260	267
DIXDC1__NM_033425_TGTTCCTCGGGATTGACACC	DIXDC1__NM_033425	128	115	137	173
DIXDC1__NM_033425_TTTGTGATTATTCCCGCTGA	DIXDC1__NM_033425	156	184	250	149
DKC1__NM_001142463_AAGTGGGGGTCGCTGGAATA	DKC1__NM_001142463	468	30	372	2
DKC1__NM_001142463_AATCCTCTGAAGAGAGAGAT	DKC1__NM_001142463	36	38	45	87
DKC1__NM_001142463_CACACATAATGTCCGAATGT	DKC1__NM_001142463	763	13	843	5
DKC1__NM_001142463_CCGAAGTATCCGTCGAATCC	DKC1__NM_001142463	944	42	969	81
DKC1__NM_001142463_CGTGTCCAACTTAGCAACTT	DKC1__NM_001142463	1104	258	1096	499
DKC1__NM_001142463_CTAAGTTGGACACGTCTCAG	DKC1__NM_001142463	399	28	408	73
DKC1__NM_001142463_GACAGGGCACAGTGGTACGC	DKC1__NM_001142463	698	1344	974	1293
DKC1__NM_001142463_GATTCGACGGATACTTCGGG	DKC1__NM_001142463	608	101	614	65
DKC1__NM_001142463_GTGAGGCTGGCACCTACATT	DKC1__NM_001142463	930	6	1154	25
DKC1__NM_001142463_TCATGGGAAGAGGGGTTAGA	DKC1__NM_001142463	244	0	259	0
DKC1__NM_001142463_TGAATCCAAAGTTGCTAAGT	DKC1__NM_001142463	159	200	277	217
DKC1__NM_001142463_TGCACAATGCTATTGAAGGG	DKC1__NM_001142463	292	12	441	33
DKC1__NM_001363_ACTCTTCTACCACTTATTTA	DKC1__NM_001363	529	713	551	513
DKC1__NM_001363_AGACAGCTGCGGACAGTTAC	DKC1__NM_001363	518	537	527	585
DKC1__NM_001363_CACAGGAACAAGTAGTCAGA	DKC1__NM_001363	325	308	334	238
DKC1__NM_001363_CACTCTTCTACCACTTATTT	DKC1__NM_001363	837	992	915	1151
DKC1__NM_001363_GCTGTAGAACCCTAAATAAG	DKC1__NM_001363	201	297	203	226
DKC1__NM_001363_TCCATCTTGTTCTGGCCTAG	DKC1__NM_001363	676	665	857	966
DKK1__NM_012242_AAAAACTATCACAGCCTAAA	DKK1__NM_012242	91	111	103	118
DKK1__NM_012242_ATGATCATAGCACCTTGGAT	DKK1__NM_012242	227	236	308	323
DKK1__NM_012242_CAGGACAGGTTTACAGATCT	DKK1__NM_012242	302	321	448	414
DKK1__NM_012242_CTACCATCGCGACAAAGACC	DKK1__NM_012242	363	262	405	402
DKK1__NM_012242_GCGCCGGGAATCCTGTACCC	DKK1__NM_012242	695	754	710	774
DKK1__NM_012242_GGAATTGAGAACCGAGTTCA	DKK1__NM_012242	168	79	198	175
DKK1__NM_012242_GGGCAGGTTCTTGATAGCGT	DKK1__NM_012242	145	163	198	280
DKK1__NM_012242_GTAATTTACTGCAATCACAG	DKK1__NM_012242	74	140	102	122
DKK1__NM_012242_GTTCACTGCATTTGGATAGC	DKK1__NM_012242	98	92	92	111
[illegible]	[illegible]	[illegible]	[illegible]	[illegible]	[illegible]
DLC1__NM_006094_ATATTTCCAGGGCATTCTAA	DLC1__NM_006094	61	31	59	90
DLC1__NM_006094_CACTGCAAATAAAGCGACAC	DLC1__NM_006094	643	741	709	1059
DLC1__NM_006094_CAGGGTTCCTGTTTGGGAG	DLC1__NM_006094	344	260	414	545
DLC1__NM_006094_CTTGACCAAGGAAATATCGA	DLC1__NM_006094	267	256	297	489
DLC1__NM_006094_CTTTATTTGCAGTGTGTTGA	DLC1__NM_006094	175	234	238	278
DLC1__NM_006094_GCCGTTAACAACCAAAGGCG	DLC1__NM_006094	229	256	313	310
DLC1__NM_006094_GGGAGTGAGATGCAATATTA	DLC1__NM_006094	283	391	285	265
[illegible]	[illegible]	[illegible]	[illegible]	[illegible]	[illegible]
DLG1__NM_001204387_CTGAGCCAGAACCTTCACGC	DLG1__NM_001204387	1099	1232	1239	1045
DLG1__NM_001204387_GGGCTAACATGGTTATCAAC	DLG1__NM_001204387	183	177	205	308
DLG1__NM_001204387_TACGCTTGTATGTAAAAAGA	DLG1__NM_001204387	115	91	106	185
DLG1__NM_001204387_TGAAGGAGGTGCAGCACATA	DLG1__NM_001204387	113	43	112	63
DLG1__NM_001204387_TGCTGCCAGCGTGAAGGTTC	DLG1__NM_001204387	392	377	476	705
DLG1__NM_001204387_TTGGAGATGATGAAATTACA	DLG1__NM_001204387	143	30	115	84
DLG2__NM_001142699_ATCTTCGTAATAAATATGCC	DLG2__NM_001142699	144	245	237	437
[illegible]	[illegible]	[illegible]	[illegible]	[illegible]	[illegible]
DLG4__NM_001365_GAGGGATGGGTCTAGGGAGT	DLG4__NM_001365	379	145	431	160
DLG4__NM_001365_GGGAAGCCTCATAATAGTCC	DLG4__NM_001365	560	638	575	632
DLG4__NM_001365_GGGGTGCCCACCGCAGCAGT	DLG4__NM_001365	432	377	483	517
DLG4__NM_001365_GTGCTGGAGTTGCAGGTGAA	DLG4__NM_001365	299	281	343	400
DLG4__NM_001365_TCTTTGCCGGTTTGCATGAG	DLG4__NM_001365	429	379	440	287
DLG4__NM_001365_TGGGGAATTACGATGAGGGC	DLG4__NM_001365	141	91	157	101
DLG5__NM_004747_AGGACGCTGTAGGTAGAACC	DLG5__NM_004747	175	235	225	273
[illegible]	[illegible]	[illegible]	[illegible]	[illegible]	[illegible]

DMPK__NM_001288765_CTACCTGGCGGAGATTGTCA	DMPK__NM_001288765	582	365	626	421
DMPK__NM_001288765_CTGGTCATGGAGTATTACGT	DMPK__NM_001288765	447	292	509	453
DMPK__NM_001288765_GATTGTCATGGCCATAGACT	DMPK__NM_001288765	436	436	612	427
DMPK__NM_001288765_GCAGCTGCGTGATCCACCGC	DMPK__NM_001288765	1928	1556	1971	1687
DMPK__NM_001288765_GGAGCGGATTCCGGCCGAGA	DMPK__NM_001288765	762	637	725	539
DMPK__NM_001288765_GTCATGGAGTATTACGTGGG	DMPK__NM_001288765	858	995	989	880
DMPK__NM_001288765_TCATGGAGTATTACGTGGGC	DMPK__NM_001288765	889	828	987	989
DMPK__NM_004409_AAATATCCAAACCGCCGAAG	DMPK__NM_004409	49	6	48	26
DMPK__NM_004409_ACCATTTCTTTCTTTCGGCC	DMPK__NM_004409	765	957	984	1410
DMPK__NM_004409_AGAGCAGCGCAAGTGAGGAG	DMPK__NM_004409	268	235	333	254
DMPK__NM_004409_ATAAATACCGAGGAATGTCG	DMPK__NM_004409	44	79	37	38
DMPK__NM_004409_CAATAAATACCGAGGAATGT	DMPK__NM_004409	639	702	675	765
DMPK__NM_004409_CCAGCTCCAGTCCTGTGATC	DMPK__NM_004409	1190	1097	1400	1510
DMPK__NM_004409_CCCCGACCCTCGCGAATAAA	DMPK__NM_004409	407	417	533	553
DMPK__NM_004409_CCTGTAGCCTGTCAGCGAGT	DMPK__NM_004409	1006	935	1049	1065
DMPK__NM_004409_CGAGAGCAGCGCAAGTGAGG	DMPK__NM_004409	554	434	643	395
DMPK__NM_004409_CGCGGGATCCCCGAAAAAGC	DMPK__NM_004409	459	533	465	388
DMPK__NM_004409_CGCTCGGAGCGGTTGTGAAC	DMPK__NM_004409	502	315	557	514
DMPK__NM_004409_GCACTTTGCGAACCAACGAT	DMPK__NM_004409	465	420	516	400
DMPK__NM_004409_GCCGGCGAACGGGGCTCGAA	DMPK__NM_004409	454	396	464	447
DMPK__NM_004409_GCGGAGACCCACGCTCGGAG	DMPK__NM_004409	900	869	1027	1061
DMPK__NM_004409_GCGGGCCCGGATCACAGGAC	DMPK__NM_004409	2392	2474	2596	2625
DMPK__NM_004409_GGGACAGACAATAAATACCG	DMPK__NM_004409	264	255	366	352
DMPK__NM_004409_GTCTCAGTGCATCCAAAACG	DMPK__NM_004409	454	548	456	509
DMPK__NM_004409_TCGTCCTCCGACTCGCTGAC	DMPK__NM_004409	427	770	395	532
DMPK__NM_004409_TGCGAACCAACGATAGGTGG	DMPK__NM_004409	0	0	0	0
DMPK__NM_004409_TTTGCGAACCAACGATAGGT	DMPK__NM_004409	0	0	0	0
DMRT1__NM_021951_AAAGCGTCTGGGGCGCTAGT	DMRT1__NM_021951	760	570	740	852
DMRT1__NM_021951_AAGCGCTTGTGGCCCTTGAG	DMRT1__NM_021951	197	387	240	235
DMRT1__NM_021951_AAGCGTCTGGGGCGCTAGTG	DMRT1__NM_021951	221	192	306	393
DMRT1__NM_021951_AGGGGCTTCCGACGGTGTAG	DMRT1__NM_021951	121	71	127	169
DMRT1__NM_021951_CAGCAAGCCCTCTACACCGT	DMRT1__NM_021951	1510	1870	1710	1728
DMRT1__NM_021951_CGCGCTGCCTCTCGGCGATC	DMRT1__NM_021951	815	1328	973	1207
DMRT1__NM_021951_CTTGCTGAATGCCTCGTCGT	DMRT1__NM_021951	1358	1678	1545	2146
DMRT1__NM_021951_CTTGTGGCCCTTGAGCGGCG	DMRT1__NM_021951	527	716	587	971
DMRT1__NM_021951_GACAAGCAGCTCGGCCGCAC	DMRT1__NM_021951	426	425	477	455
DMRT1__NM_021951_GGGCACCATGCCCAACGACG	DMRT1__NM_021951	43	85	20	25
DMRT1__NM_021951_TCAGGTTGCACTTCTTGCAC	DMRT1__NM_021951	310	397	447	511
DMRT1__NM_021951_TTGCTGAATGCCTCGTCGTT	DMRT1__NM_021951	669	824	666	950
DMRT2__NM_001130865_AATAGCTGTAGAATAATCCA	DMRT2__NM_001130865	74	32	53	45
DMRT2__NM_001130865_CCAGCAGGCAGTTGGCGCAC	DMRT2__NM_001130865	1183	1155	1240	[illegible]
DMRT2__NM_001130865_CTGCCTCCGGAGCGCCACCT	DMRT2__NM_001130865	352	353	438	[illegible]
DMRT2__NM_001130865_GGAGATCGATGTCGAGAGCC	DMRT2__NM_001130865	135	93	110	[illegible]
DMRT2__NM_181872_ACATAAGTCTCCGCTGGAAT	DMRT2__NM_181872	794	1268	905	[illegible]
DMRT2__NM_181872_ATCCTGTCACTAACTGGGGG	DMRT2__NM_181872	849	783	1086	[illegible]
DMRT2__NM_181872_ATTCCAGCGGAGACTTATGT	DMRT2__NM_181872	314	428	313	[illegible]
DMRT2__NM_181872_CAGCGGAGACTTATGTAGGA	DMRT2__NM_181872	758	732	912	[illegible]
DMRT2__NM_181872_CCAGCGGAGACTTATGTAGG	DMRT2__NM_181872	469	326	648	[illegible]
DMRT2__NM_181872_CCTCCTACATAAGTCTCCGC	DMRT2__NM_181872	1232	1262	1375	[illegible]
DMRT2__NM_181872_CTCATCCTGTCACTAACTGG	DMRT2__NM_181872	516	386	641	[illegible]
DMRT2__NM_181872_TACATAAGTCTCCGCTGGAA	DMRT2__NM_181872	450	381	524	[illegible]
DMRT2__NM_181872_TTCCTCATCCTGTCACTAAC	DMRT2__NM_181872	38	74	59	[illegible]
DMRT3__NM_021240_ACGACTTGGAGACGGCAAGT	DMRT3__NM_021240	595	723	596	[illegible]
DMRT3__NM_021240_AGTCGTTCTTCAGTCAAATC	DMRT3__NM_021240	181	188	200	[illegible]
DMRT3__NM_021240_ATTCTGCTCCGCGTGACACT	DMRT3__NM_021240	111	161	98	[illegible]

[illegible]

DOCK3__NM_004947_TACAGCAACTCTACAAGAAT	DOCK3__NM_004947	239	167	304	387
DOCK3__NM_004947_TGCTTTGCTGTACACTCTGC	DOCK3__NM_004947	559	517	520	472
DOCK3__NM_004947_TTCAAGTGAATGTAATTTGC	DOCK3__NM_004947	0	0	11	55
DOCK4__NM_014705_AATGCCTGTGTAAAGAACAA	DOCK4__NM_014705	112	109	153	190
DOCK4__NM_014705_ATTTCCAATGACAGGCCATA	DOCK4__NM_014705	109	66	99	164
DOCK4__NM_014705_CACATAGAGTTGTTTCCACA	DOCK4__NM_014705	520	413	651	754
DOCK4__NM_014705_CACGTCCTTCATCCGGTCGT	DOCK4__NM_014705	1886	2325	2357	2696
DOCK4__NM_014705_CCGTATTTCTCGTGCTCCGT	DOCK4__NM_014705	3403	3436	3390	3438
DOCK4__NM_014705_CCTACGGAGCACGAGAAATA	DOCK4__NM_014705	176	260	150	211
DOCK4__NM_014705_GCCTAGGAAAGAGTACGCAA	DOCK4__NM_014705	640	517	685	577
DOCK4__NM_014705_GGCGCTTCACGTCCTTCATC	DOCK4__NM_014705	769	756	1042	1242
DOCK4__NM_014705_TATCTCCAATTTCCAATGAC	DOCK4__NM_014705	355	199	398	366
DOCK4__NM_014705_TCACGTCCTTCATCCGGTCG	DOCK4__NM_014705	1318	1638	1378	1915
DOCK4__NM_014705_TTCAAGTGAACGTAGCTGGA	DOCK4__NM_014705	3	7	13	30
DOCK4__NM_014705_TTCCGAGGAACCGTTCCATA	DOCK4__NM_014705	70	52	88	37
DOCK5__NM_024940_AAGAGGCAGAAGTACGGGGT	DOCK5__NM_024940	765	503	774	671
DOCK5__NM_024940_CAATGGTGCTGGTTTCGTCA	DOCK5__NM_024940	1403	1830	1521	1601
DOCK5__NM_024940_CACGTCCACTCTGCGAGAAT	DOCK5__NM_024940	1465	972	1446	902
DOCK5__NM_024940_CATCTGGCGAAAGCTCTACG	DOCK5__NM_024940	421	304	446	301
DOCK5__NM_024940_CCAAAATTGATCATGGGAAC	DOCK5__NM_024940	538	357	563	609
DOCK5__NM_024940_CCATTTGAAAGAGGCAACTG	DOCK5__NM_024940	103	179	139	112
DOCK5__NM_024940_CCCGCTGGATCCCGACCAAG	DOCK5__NM_024940	506	296	533	464
DOCK5__NM_024940_CCTTGGGGAGCGTCCCAGAC	DOCK5__NM_024940	297	381	441	324
DOCK5__NM_024940_GCAATGGTGCTGGTTTCGTC	DOCK5__NM_024940	1104	1409	1292	1544
DOCK5__NM_024940_GCATTGTAGTTATAGATCGC	DOCK5__NM_024940	260	221	322	281
DOCK5__NM_024940_TCACATCCTGGAGATGTACG	DOCK5__NM_024940	161	82	175	154
DOCK5__NM_024940_TGACGTACAGCCTGATCGAG	DOCK5__NM_024940	537	513	635	496
DOCK6__NM_020812_ACACAGCGGGAGCGACAGAA	DOCK6__NM_020812	131	66	108	103
DOCK6__NM_020812_CCTCGTCAGGTGCCGGGTAG	DOCK6__NM_020812	528	586	584	748
DOCK6__NM_020812_CGGCCCCAGAAGATGTGGAC	DOCK6__NM_020812	579	758	642	762
DOCK6__NM_020812_CTTTGTCCAAAGTGCTCGCG	DOCK6__NM_020812	245	362	302	516
DOCK6__NM_020812_GCCCCGGGAATGCCGGACCA	DOCK6__NM_020812	455	501	545	450
DOCK6__NM_020812_GCCCTCTCTGCTAGAGCGGG	DOCK6__NM_020812	241	187	245	192
DOCK6__NM_020812_GGAGATGTATATTGAGGACT	DOCK6__NM_020812	578	508	642	627
DOCK6__NM_020812_GGCGCAATGAAACCCTTCGA	DOCK6__NM_020812	1286	910	1421	1332
DOCK6__NM_020812_GGGCCGGTGCTGCCGTCGAA	DOCK6__NM_020812	646	951	785	900
DOCK6__NM_020812_TCTTGGCTCTGTATGATGTG	DOCK6__NM_020812	217	227	273	347
DOCK6__NM_020812_TGGAGATGTATATTGAGGAC	DOCK6__NM_020812	155	121	160	160
DOCK6__NM_020812_TTGAGGATGTACTTCTGAGC	DOCK6__NM_020812	220	159	313	159
DOCK7__NM_001271999_AATTCAATCAAATCCCGTAA	DOCK7__NM_001271999	145	102	193	110
DOCK7__NM_001271999_ATCCACTGGATCTACTGCTT	DOCK7__NM_001271999	238	211	264	150
DOCK7__NM_001271999_CAGCCAAAGGATGAGTAATG	DOCK7__NM_001271999	257	122	308	341
DOCK7__NM_001271999_CTGGATCTACTGCTTCGGTA	DOCK7__NM_001271999	488	272	430	362
DOCK7__NM_001271999_CTGTGGATTCTGGGCCTTTA	DOCK7__NM_001271999	699	903	739	907
DOCK7__NM_001271999_CTTATACAGTCTCTAACATG	DOCK7__NM_001271999	291	166	322	189
DOCK7__NM_001271999_GAACGGTGGCAGCCGAAGTT	DOCK7__NM_001271999	316	322	324	288
DOCK7__NM_001271999_GGAGATCTGCTTCCTAACTT	DOCK7__NM_001271999	356	222	310	298
DOCK7__NM_001271999_GGATCTACTGCTTCGGTAAG	DOCK7__NM_001271999	264	286	275	335
DOCK7__NM_001271999_GGGAGAACCACTATATTGTC	DOCK7__NM_001271999	762	861	821	549
DOCK7__NM_001271999_TGGATCTACTGCTTCGGTAA	DOCK7__NM_001271999	0	0	6	0
DOCK7__NM_001271999_TGTGGATTCTGGGCCTTTAC	DOCK7__NM_001271999	173	175	138	92
DOCK8__NM_001190458_AACGTGCTGTGCGACGTGTC	DOCK8__NM_001190458	469	658	578	724
DOCK8__NM_001190458_ACAGCACGTTTAAGTGGCGG	DOCK8__NM_001190458	767	885	864	809
DOCK8__NM_001190458_ACGTGCTGTGCGACGTGTCT	DOCK8__NM_001190458	2001	2077	2123	2663
DOCK8__NM_001190458_CACAGCACGTTTAAGTGGCG	DOCK8__NM_001190458	727	1196	809	1056
DOCK8__NM_001190458_CAGCCCTCTTTGCCGGAGGA	DOCK8__NM_001190458	707	1013	716	897
DOCK8__NM_001190458_CCTCGTCCACTGATGGGTAA	DOCK8__NM_001190458	835	1062	1121	1457
DOCK8__NM_001190458_CTTTGCCCTTTACCCATCAG	DOCK8__NM_001190458	175	170	192	319
DOCK8__NM_001190458_GGACGTGGTGTTCACGCCAA	DOCK8__NM_001190458	233	482	269	280
DOCK8__NM_001190458_TCCGAAACAGACGTTTGAGT	DOCK8__NM_001190458	1248	1175	1327	1377
DOCK8__NM_001190458_TCCGACTCAAACGTCTGTTT	DOCK8__NM_001190458	659	539	834	847
DOCK8__NM_001190458_TGAACTGGACCCTCATGTCA	DOCK8__NM_001190458	245	187	366	348
DOCK8__NM_001190458_TTGAACTGGACCCTCATGTC	DOCK8__NM_001190458	496	272	539	632
DOCK8__NM_203447_AGGTACTTATGCTCTGTCGA	DOCK8__NM_203447	405	526	463	577
DOCK8__NM_203447_GGTACTGGCCAAGGTTTGGT	DOCK8__NM_203447	510	617	628	504
DOCK8__NM_203447_GGTATTCTTCAGCGGAAATA	DOCK8__NM_203447	24	54	36	79
DOCK8__NM_203447_TCTGTCGATGGTACTGGCCA	DOCK8__NM_203447	432	465	541	567
DOCK8__NM_203447_TTTACTCTCCCACCAAACCT	DOCK8__NM_203447	89	90	88	95
DOCK9__NM_001130048_ACGGCCATCCTGAGACGACA	DOCK9__NM_001130048	256	179	196	168
DOCK9__NM_001130048_AGCTGCATCCTTGTATTGCA	DOCK9__NM_001130048	631	534	742	779
DOCK9__NM_001130048_CTTACCTGAAAGTCATCGTA	DOCK9__NM_001130048	333	497	351	471
DOCK9__NM_001130048_GACGGCCATCCTGAGACGAC	DOCK9__NM_001130048	558	550	665	632
DOCK9__NM_001130048_GAGCTGCATCCTTGTATTGC	DOCK9__NM_001130048	818	676	905	945
DOCK9__NM_001130048_GATCCTGAACGACTGTTTAC	DOCK9__NM_001130048	525	410	489	477
DOCK9__NM_001130048_GCTGCATCCTTGTATTGCAG	DOCK9__NM_001130048	838	560	777	619
DOCK9__NM_001130048_TCAAAACCTATAACTCTGAC	DOCK9__NM_001130048	547	668	730	802
DOCK9__NM_001130048_TCGAGTGGCTCAATTAGCTT	DOCK9__NM_001130048	300	314	413	385
DOCK9__NM_001130048_TCTCCCGTAAACAGTCGTTC	DOCK9__NM_001130048	708	703	888	844
DOCK9__NM_001130048_TTACCTGAAAGTCATCGTAA	DOCK9__NM_001130048	112	84	132	61
DOCK9__NM_001130048_TTATAGCAAAGTGGTCAAGT	DOCK9__NM_001130048	131	99	142	125
DOCK9__NM_001130049_AAGCATGGCTGGCTGTACAA	DOCK9__NM_001130049	122	79	164	74
DOCK9__NM_001130049_GGCGCTGAGTAAGCCGGGCA	DOCK9__NM_001130049	1153	1330	1130	1368
DOCK9__NM_001130049_TCAACTTCATAGACATGAAC	DOCK9__NM_001130049	199	293	183	256
DOHH__NM_001145165_CCGGCTGAGGAGCGTGACGT	DOHH__NM_001145165	1533	429	1687	840
DOHH__NM_001145165_CCTGCCTCATGGCGCACCAT	DOHH__NM_001145165	565	9	665	46
DOHH__NM_001145165_CGCCATTGCATGGATCAGCC	DOHH__NM_001145165	311	303	297	415
DOHH__NM_001145165_CGGGCGCTGTTCACGCTGCG	DOHH__NM_001145165	1268	1551	1221	1737
DOHH__NM_001145165_CGTGCTGCAAGACACCCGTC	DOHH__NM_001145165	858	43	1092	77
DOHH__NM_001145165_GCGCGGTATCGCTCGAAGAG	DOHH__NM_001145165	380	267	350	357
DOHH__NM_001145165_GCGGCCCAGGCGCCATTGCA	DOHH__NM_001145165	125	155	96	89
DOHH__NM_001145165_GGCCTGGCTGATCCATGCAA	DOHH__NM_001145165	553	134	577	128
DOHH__NM_001145165_GGTATCGCTCGAAGAGCGGC	DOHH__NM_001145165	0	0	0	0
DOHH__NM_001145165_TACCTCGATGACGGGGTCCG	DOHH__NM_001145165	576	115	520	296
DOHH__NM_001145165_TTCCTCGGACCCCGTCATCG	DOHH__NM_001145165	224	4	225	14
DOHH__NM_001145165_TTCGCCCTGCGCAACGCGGG	DOHH__NM_001145165	0	0	9	4
DOK1__NM_001197260_AGGGCATGGAGCAATGCGCA	DOK1__NM_001197260	369	320	281	245
DOK1__NM_001197260_AGTCTAAGGGCTCAGCATAC	DOK1__NM_001197260	63	43	115	74
DOK1__NM_001197260_CTGCACCGTTACCCAGAATT	DOK1__NM_001197260	2142	1844	2495	2039
DOK1__NM_001197260_GCAATGCGCAGGGAGTCTAA	DOK1__NM_001197260	354	217	400	271
DOK1__NM_001197260_GCATGGCTCCTACGTGCTGA	DOK1__NM_001197260	231	238	309	277
DOK1__NM_001197260_GGGGGCCCAGAGTCAGATAC	DOK1__NM_001197260	236	368	309	252
DOK1__NM_001197260_GTCCAAGGGGTCTGAGTATA	DOK1__NM_001197260	172	148	211	149
DOK1__NM_001197260_GTCTAAGGGCTCAGCATACA	DOK1__NM_001197260	36	8	51	21
DOK1__NM_001197260_TACACTCTGTTGCGTCGCTA	DOK1__NM_001197260	292	323	374	337
DOK1__NM_001197260_TCTGCACCGTTACCCAGAAT	DOK1__NM_001197260	746	786	1083	807
DOK1__NM_001197260_TCTGTTGCGTCGCTATGGCC	DOK1__NM_001197260	338	261	411	354
DOK1__NM_001197260_TTAGGTCATGTTCTCTTTCG	DOK1__NM_001197260	237	199	293	259
DOK1__NM_001381_ACCATGGGGAGGTGGCACTA	DOK1__NM_001381	518	328	474	205
DOK1__NM_001381_AGCTTAGGTGGGTTATCGGT	DOK1__NM_001381	316	223	296	323
DOK1__NM_001381_CACTCACACACTCAGCCAGA	DOK1__NM_001381	947	914	1057	758
DOK1__NM_001381_CCAGGAAGTCCTAAGAAGTG	DOK1__NM_001381	252	132	242	149
DOK1__NM_001381_CCAGTCCCCACGGCGTAGCG	DOK1__NM_001381	213	211	198	288
DOK1__NM_001381_CCATAAGGGGTCGAGCTCTG	DOK1__NM_001381	134	211	95	218
DOK1__NM_001381_CCCCAGAGCTCGACCCCTTA	DOK1__NM_001381	626	672	643	543
DOK1__NM_001381_GCAAGGCTGAGGTGGCTAAG	DOK1__NM_001381	317	392	330	352
DOK1__NM_001381_GCTCGAGTTCTTTGACCATA	DOK1__NM_001381	598	643	786	804
DOK1__NM_001381_GGGACTGGCCGGGTAGAGCA	DOK1__NM_001381	654	437	716	596
DOK1__NM_001381_GGGGGGTCTCCACGGTGACG	DOK1__NM_001381	211	171	212	212
DOK1__NM_001381_TCCAGGGCAGAAAGCTTAGG	DOK1__NM_001381	277	341	331	296
DOK2__NM_003974_AAGCGCCGCAGAAACCTGTA	DOK2__NM_003974	293	296	418	370
DOK2__NM_003974_CAGGGACCCAGCTGTACGAC	DOK2__NM_003974	886	1265	1022	1369
DOK2__NM_003974_CGCCTCACTGTATGGAGGGT	DOK2__NM_003974	3196	3549	3781	4729
DOK2__NM_003974_GAGTAGGGGCTATCAGGCCG	DOK2__NM_003974	394	417	421	431
DOK2__NM_003974_GCAACTTTGAGTTCGAAACC	DOK2__NM_003974	228	171	261	304
DOK2__NM_003974_GCACAGAAATGGCGCCGCTT	DOK2__NM_003974	1506	1487	1674	2227
DOK2__NM_003974_GGAGACGGGGCAGTGAAACA	DOK2__NM_003974	362	403	380	397
DOK2__NM_003974_GGCCGAGAGTAGGGGCTATC	DOK2__NM_003974	408	448	446	652
DOK2__NM_003974_GGCGGCGGCAGTGAGTCATG	DOK2__NM_003974	1533	1839	1596	1931
DOK2__NM_003974_TCGGCGCCTCACTGTATGGA	DOK2__NM_003974	1019	561	978	766
DOK2__NM_003974_TGCCGTGCCCTTCGATGCGG	DOK2__NM_003974	919	1097	905	1234
DOK2__NM_003974_TTGTACAGCAGCGCAGTCAC	DOK2__NM_003974	296	491	343	278
DOK3__NM_001144875_AACTTGCGCAGGAAGTGGTA	DOK3__NM_001144875	371	132	432	304
DOK3__NM_001144875_AGAGGATGCCATCCTTGATA	DOK3__NM_001144875	275	127	298	229
DOK3__NM_001144875_AGGAAGGCACCGGTGTCCCG	DOK3__NM_001144875	276	264	204	194
DOK3__NM_001144875_CACCGAGCGAAGCCATCTAC	DOK3__NM_001144875	851	687	948	906
DOK3__NM_001144875_CAGTAGATGGCTTCGCTCGG	DOK3__NM_001144875	626	559	668	648
DOK3__NM_001144875_CCCATCAGGCGTGGCACGGC	DOK3__NM_001144875	971	1298	1229	1402
DOK3__NM_001144875_CCCTCTGGAGACCCCTATCA	DOK3__NM_001144875	1217	1105	1392	1195
DOK3__NM_001144875_CTGGAGACCCCTATCAAGGA	DOK3__NM_001144875	241	199	254	222
DOK3__NM_001144875_GCAGCGGGTGACAGGTCGGC	DOK3__NM_001144875	2160	2034	2338	2332
DOK3__NM_001144875_GCAGGAAGGCACCGGTGTCC	DOK3__NM_001144875	1211	1133	1220	1150
DOK3__NM_001144875_TACCAGCAGCATGTCAAGTT	DOK3__NM_001144875	356	217	377	440
DOK3__NM_001144875_TCCAGCCGTGCCACGCCTGA	DOK3__NM_001144875	465	537	581	755
DOK3__NM_001144876_ACGCAAGCGTCATGCCTCCA	DOK3__NM_001144876	335	286	383	305
DOK3__NM_001144876_AGGCATGACGCTTGCGTGCG	DOK3__NM_001144876	591	527	552	738
DOK3__NM_024872_TGCAGCTGTCTCTAGACGGA	DOK3__NM_024872	74	55	134	165
DOK4__NM_018110_AGCCTCGAAGGTAAAGCGTG	DOK4__NM_018110	244	386	354	338
DOK4__NM_018110_AGGAAGACATTGAAGCGATC	DOK4__NM_018110	191	141	144	236
DOK4__NM_018110_AGGTAAAGCGTGTGGCATCC	DOK4__NM_018110	475	431	460	496
DOK4__NM_018110_ATACCTTCCAGACACAAGAG	DOK4__NM_018110	155	43	143	108
DOK4__NM_018110_CACAGATCTACCGGAGGTGC	DOK4__NM_018110	214	208	252	252
DOK4__NM_018110_CCCGTGTGAAGCTCGTCTCG	DOK4__NM_018110	1230	1378	1265	1499
DOK4__NM_018110_CGAGCTTCACACGGGGGTTG	DOK4__NM_018110	715	835	841	919
DOK4__NM_018110_CTCTGCTCACTGCGCCGCTA	DOK4__NM_018110	307	429	479	642
DOK4__NM_018110_GCCACGAGACGAGCTTCACA	DOK4__NM_018110	1377	1332	1361	1353
DOK4__NM_018110_GTGGTACAAGACACTATCTG	DOK4__NM_018110	98	36	88	201
DOK4__NM_018110_TGCCACACGCTTTACCTTCG	DOK4__NM_018110	693	453	879	728
DOK4__NM_018110_TGGAAATGGAGAAGAACGTG	DOK4__NM_018110	21	62	26	61
DOK5__NM_018431_AGCACCAAGAAACATGCCAT	DOK5__NM_018431	520	594	603	574
DOK5__NM_018431_ATCACGTCCATACCGCCGCA	DOK5__NM_018431	772	842	944	1114
DOK5__NM_018431_CCAAGAGATGAGTTTGACTC	DOK5__NM_018431	623	651	708	984
DOK5__NM_018431_CCTTGGAGAGCCTGACTTAC	DOK5__NM_018431	1133	1523	1170	1377
DOK5__NM_018431_GATCCGGAGCAGACGCCTCG	DOK5__NM_018431	996	977	1081	1090
DOK5__NM_018431_GCACCAAGAAACATGCCATA	DOK5__NM_018431	337	283	456	350
DOK5__NM_018431_TACCGCCGCAGGGCGCTTAG	DOK5__NM_018431	265	218	309	225
DOK5__NM_018431_TATCAGTCCATACCGCCGC	DOK5__NM_018431	1462	1755	1795	2115
DOK5__NM_018431_TCTCCTAACTTAGATGTACA	DOK5__NM_018431	455	480	530	503
DOK5__NM_018431_TGTACATCTAAGTTAGGAGA	DOK5__NM_018431	52	95	147	91
DOK5__NM_018431_TTATCTTTCAGACCCGAGAC	DOK5__NM_018431	79	154	178	225
DOK5__NM_018431_TTTAATGACATAGTGAAGCA	DOK5__NM_018431	34	57	48	81
DOK6__NM_152721_ACGTGGTTCACGTTTGAGTC	DOK6__NM_152721	136	219	144	159
DOK6__NM_152721_AGAGACAAAGAAGCATGCGG	DOK6__NM_152721	283	229	250	190
DOK6__NM_152721_AGGAGAAATGATCTATCAGA	DOK6__NM_152721	334	326	406	418
DOK6__NM_152721_CTCACTGAGGAGATACGGTC	DOK6__NM_152721	417	531	343	493
DOK6__NM_152721_CTTGACTATGTCGTTAAAGT	DOK6__NM_152721	513	842	560	533
DOK6__NM_152721_GCATGCTTCTTTGTCTCTCG	DOK6__NM_152721	1235	1745	1378	1854
DOK6__NM_152721_GCTCACTGAGGAGATACGGT	DOK6__NM_152721	949	1074	985	918
DOK6__NM_152721_GGTTCACGTTTGAGTCAGGA	DOK6__NM_152721	68	148	86	157
DOK6__NM_152721_TCACCATAAATATCCAGGTT	DOK6__NM_152721	990	1051	1073	1133
DOK6__NM_152721_TCTTATGCCTACACCAAACC	DOK6__NM_152721	152	195	161	182
DOK6__NM_152721_TCTTTCATGTTGCTCAGCTA	DOK6__NM_152721	84	49	88	121
DOK6__NM_152721_TGCAGCGGGAACAGAATGGT	DOK6__NM_152721	760	839	933	854
DOK7__NM_001164673_ATGATGTCCTCGTCTTGGCC	DOK7__NM_001164673	822	1053	871	947
DOK7__NM_001164673_CACCCTCGCCGAGCGCATAG	DOK7__NM_001164673	182	73	157	238
DOK7__NM_001164673_CCGCTCTCCAACTTGGTGCC	DOK7__NM_001164673	778	806	836	1070
DOK7__NM_001164673_CCTTCAAAGATGAATCCGCT	DOK7__NM_001164673	698	770	610	700
DOK7__NM_001164673_CTCGGACGATGCAGTCGAAC	DOK7__NM_001164673	326	401	444	362
DOK7__NM_001164673_GACAAGTCGGAGCGTATCAA	DOK7__NM_001164673	139	218	220	248
DOK7__NM_001164673_GCAGTGGAAGAGTAGGTGGC	DOK7__NM_001164673	607	419	651	855
DOK7__NM_001164673_GCCCGGATCCGCTATGCGCT	DOK7__NM_001164673	1191	1289	1363	1468
DOK7__NM_001164673_GCCGAGCGCATAGCGGATCC	DOK7__NM_001164673	60	101	45	85
DOK7__NM_001164673_GGACAAGTCGGAGCGTATCA	DOK7__NM_001164673	454	371	499	747
DOK7__NM_001164673_GGGCTGCGGCCGGTTCTACC	DOK7__NM_001164673	316	363	338	507
DOK7__NM_001164673_GTGTGGACCAAGCCCTCGTA	DOK7__NM_001164673	742	579	668	875
DOK7__NM_001256896_AGACCACAGCCCCCCCTCAC	DOK7__NM_001256896	2757	2226	2893	2621
DOK7__NM_001256896_AGCAGCCTCTCGTCCTACGC	DOK7__NM_001256896	1344	1425	1281	1141
DOK7__NM_001256896_CACCTGGTACTCGACTGTCC	DOK7__NM_001256896	4285	4137	4469	4473
DOK7__NM_001256896_CAGTGAGCCCAGTTCATCTG	DOK7__NM_001256896	1808	2854	2214	2665
DOK7__NM_001256896_CTGCTTCCCAGGGCTCGCCA	DOK7__NM_001256896	750	1004	770	1143
DOK7__NM_001256896_GCAGCAGCCTCGGACGTGTGG	DOK7__NM_001256896	2213	2040	2162	2335
DOK7__NM_001256896_GCCCGGGACAGTCGAGTACC	DOK7__NM_001256896	587	531	494	556
DOK7__NM_001256896_GGCTGCGTGGTGTGTCATAG	DOK7__NM_001256896	1045	1175	1092	1311
DOK7__NM_001256896_TCAGGCGGCCAGACGTCCGC	DOK7__NM_001256896	57	53	45	97
DOK7__NM_001256896_TGCTGCCCGCGTAGGACGAG	DOK7__NM_001256896	441	720	402	417
DOK7__NM_001256896_TGTGTCATAGTGGGCCCGCA	DOK7__NM_001256896	406	400	542	490
DOLK__NM_014908_AGAGTGCCCATCTCCGGCCC	DOLK__NM_014908	691	356	739	424
DOLK__NM_014908_ATCGGTCCCATACGGTTGCG	DOLK__NM_014908	402	54	503	186
DOLK__NM_014908_CCACGAGTATCGGTCCCATA	DOLK__NM_014908	650	418	690	195
DOLK__NM_014908_CCACGGCGCACCACGAGTAT	DOLK__NM_014908	2322	1000	2232	1522
DOLK__NM_014908_CCACTTGTATTGGACGTAGA	DOLK__NM_014908	1391	77	1403	216
DOLK__NM_014908_CCTTCTACGTCCAATACAAG	DOLK__NM_014908	52	2	110	71
DOLK__NM_014908_CTTCTACGTCCAATACAAGT	DOLK__NM_014908	268	54	262	11
DOLK__NM_014908_GAGCATCCACGCAACCGTAT	DOLK__NM_014908	1094	31	1312	57
DOLK__NM_014908_GGCCTGCACTGCGAGGGCCA	DOLK__NM_014908	329	441	479	414
DOLK__NM_014908_TGACCATGGAGGCGGGCAAT	DOLK__NM_014908	523	28	516	0
DOLK__NM_014908_TGAGCATCCACGCAACCGTA	DOLK__NM_014908	508	87	498	37
DOLK__NM_014908_TGTTTGCGGACATTCGGAAC	DOLK__NM_014908	169	148	180	95
DOLPP1__NM_001135917_AACCGACGATGACAAATACA	DOLPP1__NM_001135917	455	128	499	362
DOLPP1__NM_001135917_ATTAGGAAGAACTCGGAGAC	DOLPP1__NM_001135917	172	126	219	171
DOLPP1__NM_001135917_CGTACTCTGGTTTGAGTACA	DOLPP1__NM_001135917	331	273	409	432
DOLPP1__NM_001135917_CGTGCCTCCACAGCAAGTCC	DOLPP1__NM_001135917	1185	844	1267	985
DOLPP1__NM_001135917_CTAGTCTCCTACAGCAGGTA	DOLPP1__NM_001135917	170	70	213	134
DOLPP1__NM_001135917_GCTGGAGGGCATCCCGTACT	DOLPP1__NM_001135917	1820	732	1966	1326
DOLPP1__NM_001135917_GGAGAAGAACCACATAAACT	DOLPP1__NM_001135917	441	269	559	461
DOLPP1__NM_001135917_GGGCCTGGCACTGAACGAGG	DOLPP1__NM_001135917	584	305	731	497
DOLPP1__NM_001135917_GGTTCCTGGACTTGCTGTGG	DOLPP1__NM_001135917	520	256	571	626
DOLPP1__NM_001135917_GGTTTGAGTACACGGTAACC	DOLPP1__NM_001135917	35	17	92	89
DOLPP1__NM_001135917_TACTCAAACCAGAGTACGTT	DOLPP1__NM_001135917	711	527	685	580
DOLPP1__NM_001135917_TGCACCAAACAAACAACGCC	DOLPP1__NM_001135917	236	243	314	334
DOLPP1__NM_020438_GTTACCAGGCTGCTATCCTG	DOLPP1__NM_020438	366	276	482	431
DONSON__NM_017613_AGGTCTTGTCCAGCATTGTA	DONSON__NM_017613	5	16	18	1
DONSON__NM_017613_CCCGCCTGGACAACCGACCG	DONSON__NM_017613	638	392	846	448
DONSON__NM_017613_CCGCCTGGACAACCGACCGC	DONSON__NM_017613	604	369	573	367
DONSON__NM_017613_CGGAGCCGCACTACCTCGGG	DONSON__NM_017613	781	398	952	504
DONSON__NM_017613_CTCTTCTCAACCCTTTACCT	DONSON__NM_017613	32	0	47	2
DONSON__NM_017613_CTTCTGTTGCCCTACAATGC	DONSON__NM_017613	144	0	144	0
DONSON__NM_017613_GCCGGCTCCGTCAGCTCACG	DONSON__NM_017613	891	665	1039	702
DONSON__NM_017613_TACTTTGCCTAAAAGTATAC	DONSON__NM_017613	95	29	88	26
DONSON__NM_017613_TCGGAAGCCCGGTGAGTAGC	DONSON__NM_017613	221	104	217	196
DONSON__NM_017613_TCGGGCGGCTTTCGGAAGCC	DONSON__NM_017613	5	0	0	0
DONSON__NM_017613_TGAGGACGGAATATCAGGCT	DONSON__NM_017613	329	364	335	367
DONSON__NM_017613_TTCGGTGCCCGGCTACTCAC	DONSON__NM_017613	153	214	81	99
DOPEY1__NM_001199942_ACCAGGTGGAGTTCATCGGA	DOPEY1__NM_001199942	63	18	86	39
DOPEY1__NM_001199942_AGAAAGCTTCCTGTTTAAAT	DOPEY1__NM_001199942	536	634	668	488
DOPEY1__NM_001199942_AGATCTTTGGCAAGTCGCTT	DOPEY1__NM_001199942	203	184	165	220
DOPEY1__NM_001199942_AGTCCTGCTGTGCGTTTACC	DOPEY1__NM_001199942	716	618	780	747
DOPEY1__NM_001199942_ATGTTCTTGCCCATTTAAAC	DOPEY1__NM_001199942	146	175	81	157
DOPEY1__NM_001199942_CCAACAACATATTTGTTCTG	DOPEY1__NM_001199942	607	604	837	819
DOPEY1__NM_001199942_CTACATCCAGCATTACCAGG	DOPEY1__NM_001199942	784	776	755	585
DOPEY1__NM_001199942_TGGCAGTGATATTGAGCTAA	DOPEY1__NM_001199942	29	29	27	32
DOPEY1__NM_001199942_TGGTGAGAAGACTACCCCAC	DOPEY1__NM_001199942	64	47	81	60
DOPEY1__NM_001199942_TTGAGCTAGGCGTTTGCCTA	DOPEY1__NM_001199942	56	34	41	75
DOPEY1__NM_001199942_TTGGGTAAAACACTGAAACC	DOPEY1__NM_001199942	59	55	86	89
DOPEY1__NM_001199942_TTTAAACAGGAAGCTTTCTA	DOPEY1__NM_001199942	41	59	77	124
DOPEY1__NM_015018_TTGCAGGTTTAAGACAACTC	DOPEY1__NM_015018	192	300	217	269
DOPEY2__NM_005128_AATCGTGGGGACCAAATGGC	DOPEY2__NM_005128	436	375	419	458
DOPEY2__NM_005128_AGGGCTCCGAGATCTCCGAC	DOPEY2__NM_005128	584	766	835	856
DOPEY2__NM_005128_CAAGTCCTTGGCCAGCCATT	DOPEY2__NM_005128	794	900	1100	1168
DOPEY2__NM_005128_CACACGCGGCGGTGTCGGTG	DOPEY2__NM_005128	205	357	277	351
DOPEY2__NM_005128_CGGGAGCAGAAGTACATGCT	DOPEY2__NM_005128	529	565	652	866
DOPEY2__NM_005128_GAGATCCGCCCATTCACTCG	DOPEY2__NM_005128	1141	1295	1319	1350
DOPEY2__NM_005128_GCTCCTGAGACTGTCGCTGG	DOPEY2__NM_005128	1034	1007	1153	1091
DOPEY2__NM_005128_GGAGGAAGTACTTCTCGTAC	DOPEY2__NM_005128	436	542	513	602
DOPEY2__NM_005128_TCCTCTCCTGGCACACGCGG	DOPEY2__NM_005128	159	143	216	145
DOPEY2__NM_005128_TCGTGGTGGGCCACATCAAC	DOPEY2__NM_005128	223	188	249	180
DOPEY2__NM_005128_TCTGCTCCTGAGACTGTCGC	DOPEY2__NM_005128	392	593	522	670
DOPEY2__NM_005128_TTGCTGATGAGGAGCCGTCT	DOPEY2__NM_005128	303	349	249	304
DOT1L__NM_032482_AGCGGCCACGGTAGACGGC	DOT1L__NM_032482	884	656	992	1703
DOT1L__NM_032482_CAGAGGTGCAAAGGGTTTCG	DOT1L__NM_032482	599	417	660	775
DOT1L__NM_032482_CCCGGATCTCAAGCTCGCTA	DOT1L__NM_032482	394	521	416	521
DOT1L__NM_032482_CCCTTCTCCCCCGAGGTGTA	DOT1L__NM_032482	828	470	872	1633
DOT1L__NM_032482_CGAATCGCCAACACGAGGTA	DOT1L__NM_032482	0	0	0	0
DOT1L__NM_032482_CGGCAGCGGCCACGGTAGA	DOT1L__NM_032482	1227	867	1350	2059
DOT1L__NM_032482_CTGAGACTGAAGTCGCCCGT	DOT1L__NM_032482	1059	431	1188	1679
DOT1L__NM_032482_GACCGACGACGACCTGTTTG	DOT1L__NM_032482	1187	2020	1525	2220
DOT1L__NM_032482_GGGAGCGAATCGCCAACACG	DOT1L__NM_032482	302	205	280	352
DOT1L__NM_032482_GGTGGATGCTGTCGATGGCA	DOT1L__NM_032482	431	284	434	603
DOT1L__NM_032482_TCAGCTTCGAGAGCATGCAG	DOT1L__NM_032482	146	91	144	138
DOT1L__NM_032482_TGAGACTGAAGTCGCCCGTG	DOT1L__NM_032482	516	315	651	922
DPAGT1__NM_001382_AATATCCTAGCAGGAATTAA	DPAGT1__NM_001382	51	4	39	1
DPAGT1__NM_001382_AATTCCTGCTAGGATATTGA	DPAGT1__NM_001382	334	0	506	0
DPAGT1__NM_001382_AGCAGGAATTAACGGCCTAG	DPAGT1__NM_001382	632	33	722	28
[illegible]	[illegible]	[illegible]	[illegible]	[illegible]	[illegible]

DTX3L__NM_138287_AGAGACAGGGCAATATAGTA	DTX3L__NM_138287	253	260	297	383
DTX3L__NM_138287_AGCTATGGGGGCAGACTACC	DTX3L__NM_138287	487	741	655	667
DTX3L__NM_138287_AGTCCGGCCCCCGAGTACGA	DTX3L__NM_138287	539	743	686	739
DTX3L__NM_138287_ATTTGCTTTGAATACTGTGT	DTX3L__NM_138287	574	632	546	636
DTX3L__NM_138287_CAGGGCAAGTGCACCCAATA	DTX3L__NM_138287	472	407	587	636
DTX3L__NM_138287_CAGGTCACCTGATCGACTTG	DTX3L__NM_138287	704	618	825	840
DTX3L__NM_138287_CCAAGAGCCTAGTATATAGT	DTX3L__NM_138287	784	709	878	940
DTX3L__NM_138287_CCATGGAGAAGTACCCTCAT	DTX3L__NM_138287	1914	2316	2172	2263
DTX3L__NM_138287_CCCCCGAGTACGAAGGAAGC	DTX3L__NM_138287	155	141	123	164
DTX3L__NM_138287_CCTCAGCCTCTCAAGTGGCT	DTX3L__NM_138287	67	0	25	0
DTX3L__NM_138287_CCTTGGCCTCTCGAGTAGAT	DTX3L__NM_138287	802	766	989	913
DTX3L__NM_138287_CGCCCGAGGACTTAGAGCTC	DTX3L__NM_138287	48	64	46	82
DTX3L__NM_138287_CTCGTGCGGGTGTACAAGTC	DTX3L__NM_138287	522	354	584	455
DTX3L__NM_138287_CTGAAAAACTACTACAATGT	DTX3L__NM_138287	428	557	414	368
DTX3L__NM_138287_CTGTGTAAAATCTGCTGCAA	DTX3L__NM_138287	463	751	613	753
DTX3L__NM_138287_GAAATGCTGGGGTATGAACC	DTX3L__NM_138287	100	104	142	68
DTX3L__NM_138287_GAGGAAGCCACTCAGTCAGC	DTX3L__NM_138287	475	470	610	386
DTX3L__NM_138287_GAGGTGAAGCCTAACGGGAG	DTX3L__NM_138287	415	323	479	436
DTX3L__NM_138287_GCTGCTTCCATTCATAGCAA	DTX3L__NM_138287	291	357	276	336
DTX3L__NM_138287_GCTTCCTCTCTGTCATTGAA	DTX3L__NM_138287	173	295	322	335
DTX3L__NM_138287_GGAGGTGAAGCCTAACGGGA	DTX3L__NM_138287	341	463	351	318
DTX3L__NM_138287_GGTGAAATAAGCCTACTATA	DTX3L__NM_138287	347	141	304	140
DTX3L__NM_138287_GTCTAAATCCTTATGTAGAA	DTX3L__NM_138287	197	392	257	228
DTX3L__NM_138287_GTGGGAGATTGCAGAGCATT	DTX3L__NM_138287	79	117	30	63
DTX3L__NM_138287_TATAATCCCATCTACTCGAG	DTX3L__NM_138287	112	1	160	4
DTX3L__NM_138287_TATATAGCATTACTGTGGCA	DTX3L__NM_138287	525	527	521	510
DTX3L__NM_138287_TCAGTGGGTACCAGGAAAAT	DTX3L__NM_138287	970	1160	975	1068
DTX3L__NM_138287_TCCAGCTTCCTTCGTACTCG	DTX3L__NM_138287	489	630	638	801
DTX3L__NM_138287_TCTCCAGCTTCCTTCGTACT	DTX3L__NM_138287	1537	1923	1720	1583
DTX3L__NM_138287_TGGAGAACAGGTTACAGTTC	DTX3L__NM_138287	329	339	294	153
DTX3L__NM_138287_TGGAGGTGAAGCCTAACGGG	DTX3L__NM_138287	71	19	59	13
DTX4__NM_015177_AACCAGTTCCGCCAAGACAC	DTX4__NM_015177	75	33	90	82
DTX4__NM_015177_ATGCCCACTTCCATGTCGTA	DTX4__NM_015177	1212	1608	1435	1688
DTX4__NM_015177_ATGGAAGTGAGGTCGATCCA	DTX4__NM_015177	661	673	793	687
DTX4__NM_015177_CACCCGTGTCTTGGCGGAAC	DTX4__NM_015177	1032	1419	1213	1520
DTX4__NM_015177_CGTAGTAGTTGCGGCGAACT	DTX4__NM_015177	843	985	1006	1169
DTX4__NM_015177_GACGCCCTACGACATGGAAG	DTX4__NM_015177	393	431	408	524
DTX4__NM_015177_GATGCCCACTTCCATGTCGT	DTX4__NM_015177	3210	3214	3358	3602
DTX4__NM_015177_GCTGGGTCTGACGGTTGATC	DTX4__NM_015177	524	296	539	634
DTX4__NM_015177_GGCAAGGTCCCTGTGACCAT	DTX4__NM_015177	750	962	863	829
DTX4__NM_015177_TCGTAGTAGTTGCGGCGAAC	DTX4__NM_015177	272	378	318	444
DTX4__NM_015177_TGGAAGTGAGGTCGATCCAG	DTX4__NM_015177	70	63	59	63
DTX4__NM_015177_TGTCTTGGTGATGAGTGTTA	DTX4__NM_015177	280	309	366	273
DTYMK__NM_001165031_AAGTCGTGTCTTTCATGAGC	DTYMK__NM_001165031	394	199	499	314
DTYMK__NM_001165031_AGCTGGCGGATGCTGCCAAG	DTYMK__NM_001165031	498	0	594	12
DTYMK__NM_001165031_CACCTCCTTGGCACCGGTGA	DTYMK__NM_001165031	1500	1513	1833	1885
DTYMK__NM_001165031_CCAGGGCGTGACCCTCGTCG	DTYMK__NM_001165031	1342	709	1343	977
DTYMK__NM_001165031_CCCCGTTCTCATAGCGCTCA	DTYMK__NM_001165031	111	74	109	70
DTYMK__NM_001165031_CTAGAAAGATCAACTGAAAT	DTYMK__NM_001165031	427	591	458	615
DTYMK__NM_001165031_GCCATGAGCGCTATGAGAAC	DTYMK__NM_001165031	587	643	643	817
DTYMK__NM_012145_AGAGGTGGGAGACTCGCGGA	DTYMK__NM_012145	317	213	329	237
DTYMK__NM_012145_CTGGCTGTTTACACCAATCT	DTYMK__NM_012145	1571	591	1791	660
DTYMK__NM_012145_GGGCCTTCCCAAACCCGACC	DTYMK__NM_012145	526	90	617	222
DTYMK__NM_012145_GTTCCTCCAGTTACAGCTGG	DTYMK__NM_012145	134	54	174	70
DTYMK__NM_012145_TGGCTGTTTACACCAATCTA	DTYMK__NM_012145	86	0	84	0
DUOX1__NM_017434_AGCTCAGAACCCCATTTCGT	DUOX1__NM_017434	831	514	839	685
DUOX1__NM_017434_ATGGTGTTGCTAAGGTCTCG	DUOX1__NM_017434	339	459	444	447
DUOX1__NM_017434_CAACACTGTGCGGTTTCTCA	DUOX1__NM_017434	1149	1210	1344	1307
DUOX1__NM_017434_CCCTTGAGATGGTGTTGCTA	DUOX1__NM_017434	676	700	932	950
DUOX1__NM_017434_CCTGAGAAACCGCACAGTGT	DUOX1__NM_017434	1511	1009	1566	1154
DUOX1__NM_017434_CGCTGCACCTCCCACGAAAT	DUOX1__NM_017434	2470	3164	2789	3622
DUOX1__NM_017434_CGCTGGTCGGGGTCGAACAT	DUOX1__NM_017434	888	723	1042	994
DUOX1__NM_017434_GCTTCTGCCTGGCTCTAGCA	DUOX1__NM_017434	217	384	390	431
DUOX1__NM_017434_GGGAGGTGCAGCGATTTGAT	DUOX1__NM_017434	602	547	599	588
DUOX1__NM_017434_TCGCTGCACCTCCCACGAAA	DUOX1__NM_017434	799	1217	933	968
DUOX1__NM_017434_TGAGAAACCGCACAGTGTTG	DUOX1__NM_017434	410	278	393	281
DUOX1__NM_017434_TTGCTAAGGTCTCGGGGGTT	DUOX1__NM_017434	303	440	397	482
DUOX2__NM_014080_AAGTGCAGCGCTATGACGGC	DUOX2__NM_014080	1129	1154	1289	1071
DUOX2__NM_014080_ACACCGTCGGCGTAATTGGC	DUOX2__NM_014080	567	735	695	863
DUOX2__NM_014080_ATACACACCGTCGGCGTAAT	DUOX2__NM_014080	630	940	694	715
DUOX2__NM_014080_CAGAGCCTGATACACACCGT	DUOX2__NM_014080	104	55	119	113
DUOX2__NM_014080_CCACAACCGCACCGTACTGG	DUOX2__NM_014080	1674	1705	1772	1212
DUOX2__NM_014080_CCCCCAGTACGGTGCGGTTG	DUOX2__NM_014080	1669	1598	1777	2191
DUOX2__NM_014080_CGGCGTAATTGGCTGGTACG	DUOX2__NM_014080	891	1190	992	833
DUOX2__NM_014080_CTACCATGTTCTTTCCGACG	DUOX2__NM_014080	1016	948	1281	1489
DUOX2__NM_014080_GTACCAGCCAATTACGCCGA	DUOX2__NM_014080	1061	1190	1270	1166
DUOX2__NM_014080_TCCACAACCGCACCGTACTG	DUOX2__NM_014080	1523	1087	1523	1245
DUOX2__NM_014080_TGGGAAGTGCAGCGCTATGA	DUOX2__NM_014080	58	74	94	90
DUOX2__NM_014080_TTACGCCGACGGTGTGTATC	DUOX2__NM_014080	363	228	367	225
DUOXA1__NM_001276264_AGGCAGGATGACGATGAACG	DUOXA1__NM_001276264	338	467	369	444
DUOXA1__NM_001276264_AGTGGATCAGCGCTGATATT	DUOXA1__NM_001276264	523	448	397	321
DUOXA1__NM_001276264_CAAAGTGGTGTCCATCGGGA	DUOXA1__NM_001276264	458	324	506	361
DUOXA1__NM_001276264_CCCCAAGCCAACCTTCCCGA	DUOXA1__NM_001276264	135	299	189	266
DUOXA1__NM_001276264_GAAGGCCTTGTATGATGTGT	DUOXA1__NM_001276264	1102	1116	1150	1391
DUOXA1__NM_001276264_GAGTGGATCAGCGCTGATAT	DUOXA1__NM_001276264	619	627	794	862
DUOXA1__NM_001276264_GGTGACCAGCTTATTCATCG	DUOXA1__NM_001276264	1112	987	1282	1277
DUOXA1__NM_001276264_GTGGTGACCAGCTTATTCAT	DUOXA1__NM_001276264	25	0	22	0
DUOXA1__NM_001276264_TGCAGCCCCGATGAATAAGC	DUOXA1__NM_001276264	193	206	239	219
DUOXA1__NM_001276264_TGGCTTGGGGCCAGCATAGA	DUOXA1__NM_001276264	0	0	0	0
DUOXA1__NM_001276264_TGGTGACCAGCTTATTCATC	DUOXA1__NM_001276264	69	24	54	47
DUOXA1__NM_001276264_TTCAGTTCTGAGTGGTCTGT	DUOXA1__NM_001276264	38	148	85	90
DUOXA1__NM_001276265_CCTGGGTGAGAACTATGCTG	DUOXA1__NM_001276265	951	927	1051	1192
DUOXA1__NM_001276265_CTATGCTGAGGAGTATGCAA	DUOXA1__NM_001276265	412	490	524	567
DUOXA1__NM_001276265_GAACTCCTCGTTGTAATTGA	DUOXA1__NM_001276265	642	546	662	778
DUOXA1__NM_001276265_TGAGACCATCAATTACAACG	DUOXA1__NM_001276265	752	810	921	803
DUOXA2__NM_207581_ACTGGTGGTACAGGCCGCAA	DUOXA2__NM_207581	97	70	96	62
DUOXA2__NM_207581_CAGTGCGTTCGCGTACTCCG	DUOXA2__NM_207581	1319	1603	1507	1402
DUOXA2__NM_207581_CTTCAGTGCAGAATGGTTCG	DUOXA2__NM_207581	548	741	557	636
DUOXA2__NM_207581_GAAGGCTTTGTAGGATGTGT	DUOXA2__NM_207581	835	863	1011	1126
DUOXA2__NM_207581_GCCGGACCCAGTGCTCTACC	DUOXA2__NM_207581	1022	1053	1154	1348
DUOXA2__NM_207581_GCTTCCTGCTCATCTTGCCG	DUOXA2__NM_207581	1650	1745	2004	1636
DUOXA2__NM_207581_GTTCTTCTCAGTCTGTTCAT	DUOXA2__NM_207581	123	102	149	78
DUOXA2__NM_207581_TACAGGCCGCAAGGGCTACT	DUOXA2__NM_207581	429	268	289	300
DUOXA2__NM_207581_TCTCCGCCAGGTAGAGCACT	DUOXA2__NM_207581	176	185	211	141
DUOXA2__NM_207581_TCTGAAAGAGAATTACGCCG	DUOXA2__NM_207581	537	423	600	520
DUOXA2__NM_207581_TTCAGTGCAGAATGGTTCGT	DUOXA2__NM_207581	175	210	143	190
DUOXA2__NM_207581_TTCTCCGCCAGGTAGAGCAC	DUOXA2__NM_207581	10	6	17	1
DUPD1__NM_001003892_ACACCCACGTCAACGAGGTC	DUPD1__NM_001003892	240	305	259	353
DUPD1__NM_001003892_ACCGCTGCGTCCTCCCGAAC	DUPD1__NM_001003892	1304	1244	1387	1433
DUPD1__NM_001003892_CCTCGTTGACGTGGGTGTAC	DUPD1__NM_001003892	475	427	414	478
DUPD1__NM_001003892_CGACGGCGCTGGACCGCTAT	DUPD1__NM_001003892	2324	2862	2775	2721
DUPD1__NM_001003892_GACATGGACATCCAGTACCA	DUPD1__NM_001003892	470	533	434	490
DUPD1__NM_001003892_GGCCCATGACGCAGTGAACC	DUPD1__NM_001003892	944	1303	873	1139
DUPD1__NM_001003892_GTCTGGCCCAAGCTCTACAT	DUPD1__NM_001003892	323	17	585	87
DUPD1__NM_001003892_TCAAAAAGCCCCGGTTCGGG	DUPD1__NM_001003892	68	182	103	170
DUPD1__NM_001003892_TCCATGTCGCGGTAGTAGTC	DUPD1__NM_001003892	568	705	650	699
DUPD1__NM_001003892_TGGGCCAGACCTCGTTGACG	DUPD1__NM_001003892	344	333	318	404
DUS1L__NM_022156_ATGTTCCCGTTAGCAAACAC	DUS1L__NM_022156	208	167	148	135
DUS1L__NM_022156_CAGCATCTGGGCGTACCTCA	DUS1L__NM_022156	595	449	628	574
DUS1L__NM_022156_CCAGGACGCTGCACCCGACA	DUS1L__NM_022156	954	885	1180	971
DUS1L__NM_022156_CCATCCCTGTGTTTGCTAAC	DUS1L__NM_022156	1153	1203	1405	1629
DUS1L__NM_022156_CCTCTCTTGGCTATCATCTG	DUS1L__NM_022156	732	672	754	850
DUS1L__NM_022156_CCTTCCGGTAGTTGGCGTCG	DUS1L__NM_022156	1159	869	1311	1415
DUS1L__NM_022156_GACGGTGCACGGACGCACCA	DUS1L__NM_022156	204	170	214	161
DUS1L__NM_022156_GTTCTGTGCCAATGACCCGG	DUS1L__NM_022156	107	64	110	77
DUS1L__NM_022156_TCCGTGCACCGTCAGCAACT	DUS1L__NM_022156	1254	1056	1378	1224
DUS1L__NM_022156_TCCTAGTTGCTGACGGTGCA	DUS1L__NM_022156	400	347	503	637
DUS1L__NM_022156_TGTCAATCTCCGGGAAGACA	DUS1L__NM_022156	145	222	194	122
DUS1L__NM_022156_TTGTCCGCGACGCCAACTAC	DUS1L__NM_022156	76	69	110	66
DUS2__NM_001271762_AATCCTGGCCCCAATGGTTC	DUS2__NM_001271762	434	464	546	428
DUS2__NM_001271762_AATGTCCGCTCCATAATCCA	DUS2__NM_001271762	717	501	743	670
DUS2__NM_001271762_AGTCCCTACCCGAACCATTG	DUS2__NM_001271762	612	550	692	615
DUS2__NM_001271762_CTGGCCCCAATGGTTCGGGT	DUS2__NM_001271762	1645	1715	1909	1556
DUS2__NM_001271762_GAAGACAACTCGATCATCAG	DUS2__NM_001271762	517	456	555	420
DUS2__NM_001271762_GAGTCCCTACCCGAACCATT	DUS2__NM_001271762	651	602	693	571
DUS2__NM_001271762_GCTGGCCCTGGATTATGGAG	DUS2__NM_001271762	468	404	620	594
DUS2__NM_001271762_GGGCCAGGATTAGCTTATTA	DUS2__NM_001271762	191	434	217	138
DUS2__NM_001271762_TAATCCTGGCCCCAATGGTT	DUS2__NM_001271762	950	779	999	608
DUS2__NM_001271762_TCTTGCACTGAATCATCTTG	DUS2__NM_001271762	625	567	647	544
DUS2__NM_001271762_TGGCCCCAATGGTTCGGGTA	DUS2__NM_001271762	109	115	142	84
DUS2__NM_001271762_TTACCATAATAAGCTAATCC	DUS2__NM_001271762	38	43	57	47
DUS3L__NM_001161619_AAGGGCAGGAGAAGACTTGC	DUS3L__NM_001161619	373	296	397	495
DUS3L__NM_001161619_ACACTGGTGGCGTTTGAGTA	DUS3L__NM_001161619	566	366	524	523
DUS3L__NM_001161619_CACACTGGTGGCGTTTGAGT	DUS3L__NM_001161619	445	317	515	358
DUS3L__NM_001161619_CGAAGCGCTTGCAGATCCGT	DUS3L__NM_001161619	4833	3002	5937	4230
DUS3L__NM_001161619_CGACGGATCTGCAAGCGCTT	DUS3L__NM_001161619	267	233	233	302
DUS3L__NM_001161619_CGACTCGGGAGCCGGAGCTT	DUS3L__NM_001161619	669	877	794	826
DUS3L__NM_001161619_CGCTTGCAGATCCGTCGGAA	DUS3L__NM_001161619	1068	1140	1097	1703
DUS3L__NM_001161619_CTGGCCCCCTCACCACGGT	DUS3L__NM_001161619	1635	1268	1733	1369
DUS3L__NM_001161619_GAATGGTGGTGGTGGCGACT	DUS3L__NM_001161619	878	659	940	932
DUS3L__NM_001161619_GCGCTTGCAGATCCGTCGGA	DUS3L__NM_001161619	1073	1157	1133	1446
DUS3L__NM_001161619_GGAGCCGGAGCTTTGGAACG	DUS3L__NM_001161619	841	927	924	867
DUS3L__NM_001161619_GGATGTGACATGTGGAGAGA	DUS3L__NM_001161619	218	144	233	360
DUS3L__NM_020175_ACTGGAGGATGGACAGACGG	DUS3L__NM_020175	436	282	554	170
DUS3L__NM_020175_CCTGTTCTTGTCGTAGTTCG	DUS3L__NM_020175	536	708	731	755
DUS3L__NM_020175_CTGCCGCTTTCTGCACGACG	DUS3L__NM_020175	661	1138	864	1147
DUS3L__NM_020175_CTGTTCTTGTCGTAGTTCGT	DUS3L__NM_020175	438	433	583	544
DUS3L__NM_020175_GAGGGCCCGGGGACAAAACA	DUS3L__NM_020175	310	166	441	302
DUS3L__NM_020175_GCCGCTTTCTGCACGACGTG	DUS3L__NM_020175	962	1055	1006	1230
DUS3L__NM_020175_GCTGTGTCCCTCCCTAATCC	DUS3L__NM_020175	922	682	1186	595
DUS3L__NM_020175_TGAGGCTAAGCGGATCCGAC	DUS3L__NM_020175	435	273	429	492
DUS4L__NM_001270419_ACTTTGAATATCGAACCATT	DUS4L__NM_001270419	386	433	498	372
DUS4L__NM_001270419_AGCGAATTTACCACAAATCA	DUS4L__NM_001270419	505	831	598	620
DUS4L__NM_001270419_ATAGTCTGTCCTTATGCGAA	DUS4L__NM_001270419	231	462	247	271
DUS4L__NM_001270419_ATGTCTCCATTAGCAATTAC	DUS4L__NM_001270419	134	152	127	141
DUS4L__NM_001270419_CCAGAATGAAACATTTCTAT	DUS4L__NM_001270419	167	379	240	294
DUS4L__NM_001270419_CTTTGAATATCGAACCATTG	DUS4L__NM_001270419	189	252	164	253
DUS4L__NM_001270419_GAAAATGTGTGGCGGATTAC	DUS4L__NM_001270419	226	242	197	203
DUS4L__NM_001270419_GCAGCAAACTGAACAATCAA	DUS4L__NM_001270419	321	252	380	469
DUS4L__NM_001270419_GGCAATGGCAGAAGGTTATG	DUS4L__NM_001270419	397	542	422	439
DUS4L__NM_001270419_GTTTCATGGATTACAGTCCA	DUS4L__NM_001270419	230	368	330	427
DUS4L__NM_001270419_TACTTTGAATATCGAACCAT	DUS4L__NM_001270419	163	336	194	243
DUS4L__NM_001270419_TCTATACCTGTAATTGCTAA	DUS4L__NM_001270419	510	739	647	892
DUSP1__NM_004417_AAGCGTGATACGCACTGCCC	DUSP1__NM_004417	709	864	792	639
DUSP1__NM_004417_ACTAACCTGATCGTAGAGTG	DUSP1__NM_004417	289	299	392	504
DUSP1__NM_004417_AGCAAACAGTCGACCCCCAT	DUSP1__NM_004417	2807	2515	3262	3322
DUSP1__NM_004417_ATTGGCTGAGACGTTGATCA	DUSP1__NM_004417	856	759	980	886
DUSP1__NM_004417_CAGTACCCCACTCTACGATC	DUSP1__NM_004417	1059	954	1222	958
DUSP1__NM_004417_CCGCAGGCCTCCAGCGTCCA	DUSP1__NM_004417	131	215	172	123
DUSP1__NM_004417_CGTTGACAGAGCCGGCGATG	DUSP1__NM_004417	753	867	779	926
DUSP1__NM_004417_CTCAAAATGGTTGGGACAAT	DUSP1__NM_004417	358	477	461	499
DUSP1__NM_004417_GCACTGCCCAGGTACAGAAA	DUSP1__NM_004417	165	94	153	141
DUSP1__NM_004417_GTCAGGGACGCTAGTACTCA	DUSP1__NM_004417	732	732	779	898
DUSP1__NM_004417_TCCTTCTTCGCTTTCAACGC	DUSP1__NM_004417	305	240	371	494
DUSP1__NM_004417_TGTCAGGGACGCTAGTACTC	DUSP1__NM_004417	119	57	110	83
DUSP10__NM_007207_ACAACGGTGGTGGCGATGAC	DUSP10__NM_007207	55	86	76	69
DUSP10__NM_007207_ACCCTGTCGTCTAAAGGAGA	DUSP10__NM_007207	571	573	465	531
DUSP10__NM_007207_ACGTCAGATTCGCAGCCTTG	DUSP10__NM_007207	1314	2010	1598	2127
DUSP10__NM_007207_ACTACTACCCTGTCGTCTAA	DUSP10__NM_007207	706	795	917	1149
DUSP10__NM_007207_AGCCTTGAGGGACACAACGG	DUSP10__NM_007207	424	331	379	386
DUSP10__NM_007207_CACTGTGGCAACCTACGACA	DUSP10__NM_007207	826	498	961	1138
DUSP10__NM_007207_CTCCGTCTCCTTTAGACGAC	DUSP10__NM_007207	167	259	213	348
DUSP10__NM_007207_GCATCCACAATTCAGCGAGC	DUSP10__NM_007207	346	312	272	480
DUSP10__NM_007207_GGCCTGATTGTCCTTGTCGT	DUSP10__NM_007207	2934	3320	3276	3870
DUSP10__NM_007207_TCCGTCTCCTTTAGACGACA	DUSP10__NM_007207	99	104	121	129
DUSP10__NM_007207_TCTGCCCGCTCGCTGAATTG	DUSP10__NM_007207	4244	4936	4931	4903
DUSP10__NM_007207_TGTTTAGACTCTAGTTACCT	DUSP10__NM_007207	224	306	201	225
DUSP11__NM_003584_AAATTCAAACACGCTGTTAA	DUSP11__NM_003584	220	332	246	356
DUSP11__NM_003584_AATTCAAACACGCTGTTAAT	DUSP11__NM_003584	445	603	510	608
DUSP11__NM_003584_ACCCATGGTTTAAACAGGAC	DUSP11__NM_003584	220	247	326	401
DUSP11__NM_003584_ATCTGATGTAGATTATACCT	DUSP11__NM_003584	408	318	461	354
DUSP11__NM_003584_CACAATAAGCCTGTTAAACA	DUSP11__NM_003584	394	531	360	419
DUSP11__NM_003584_CATGCGCAATAGCGAGACGC	DUSP11__NM_003584	279	195	353	280
DUSP11__NM_003584_CCGGCACCTATTGAATACTG	DUSP11__NM_003584	2443	2211	2552	2438
DUSP11__NM_003584_CCTCAGTATTCAATAGGTGC	DUSP11__NM_003584	1123	1347	1479	1524
DUSP11__NM_003584_GTCTTCAAAATCACTTGACC	DUSP11__NM_003584	309	310	297	310
DUSP11__NM_003584_TGTTTAAACCATGGGTACAG	DUSP11__NM_003584	0	0	7	23
DUSP11__NM_003584_TTACATTCAATTGCATCATC	DUSP11__NM_003584	62	61	95	85
DUSP11__NM_003584_TTTCAAAGTTCCTTTGCAAA	DUSP11__NM_003584	83	97	61	110
DUSP12__NM_007240_AGCCAGGATTGTATTTCGGT	DUSP12__NM_007240	1602	908	1930	1384
DUSP12__NM_007240_AGCGCTGGACAAACCCGAGA	DUSP12__NM_007240	669	286	546	280
DUSP12__NM_007240_AGGCCACGCACCGGTCCAGA	DUSP12__NM_007240	1186	328	991	456
DUSP12__NM_007240_ATGGCTGAGTAGGTCCGTCT	DUSP12__NM_007240	480	247	489	233
DUSP12__NM_007240_CAGCCAGGATTGTATTTCGG	DUSP12__NM_007240	1946	940	2145	1470
DUSP12__NM_007240_CATCACGGCCGTGCTAACAG	DUSP12__NM_007240	394	222	374	149
DUSP12__NM_007240_CCCGCTTCCCTCAGGTGATC	DUSP12__NM_007240	1012	586	1281	943
DUSP12__NM_007240_CCTGAGGGAAGCGGGCATCA	DUSP12__NM_007240	850	305	896	442
DUSP12__NM_007240_CGCGGAGCCAGATCACCTGA	DUSP12__NM_007240	2077	2533	2307	2934
DUSP12__NM_007240_GCCAGGATTGTATTTCGGTG	DUSP12__NM_007240	974	526	1003	562
DUSP12__NM_007240_GGCCGTGCTAACAGTGGACT	DUSP12__NM_007240	918	860	942	737
DUSP12__NM_007240_GGCCTGGGGTCGAGGATCTA	DUSP12__NM_007240	814	560	837	786
DUSP13__NM_001007271_AAGGGCCTCTACTGTCAGGG	DUSP13__NM_001007271	489	376	499	356
DUSP13__NM_001007271_ACCGTGCCCTCAACACGCCT	DUSP13__NM_001007271	2431	2103	2515	1933
DUSP13__NM_001007271_CACTGCTGCCGTAGAAGTCA	DUSP13__NM_001007271	708	658	870	972
DUSP13__NM_001007271_CAGGGCGGCCCTGACTTCTA	DUSP13__NM_001007271	889	770	976	833
DUSP13__NM_001007271_CCGTGCCCTCAACACGCCTG	DUSP13__NM_001007271	1634	1431	1907	1670
DUSP13__NM_001007271_CCTACCCCCAGGCGTGTTGA	DUSP13__NM_001007271	1437	1521	1588	1499
DUSP13__NM_001007271_CTCAAAGCGGTTGTTTGCCG	DUSP13__NM_001007271	392	574	360	436
DUSP13__NM_001007271_CTCACGCATCTCCTATGAAA	DUSP13__NM_001007271	240	172	375	204
DUSP13__NM_001007271_GCAGCCGTGTGGACGAAGTT	DUSP13__NM_001007271	720	942	775	744
DUSP13__NM_001007271_GGCAGCAGTGTGAGCTACCT	DUSP13__NM_001007271	1391	888	1379	1360
DUSP13__NM_001007271_GGGAGAGAGGTCTCAGCCAT	DUSP13__NM_001007271	840	679	933	791
DUSP13__NM_001007271_TGTGGGCGGCGTTCAGCACG	DUSP13__NM_001007271	587	488	755	520
DUSP13__NM_001007272_ACAGGTGCCAAATTCTACCG	DUSP13__NM_001007272	662	672	665	1096
DUSP13__NM_001007272_CACACTGAACCATATCGATG	DUSP13__NM_001007272	417	231	482	312
DUSP13__NM_001007272_CCAGGTTCTGGACAACCGAC	DUSP13__NM_001007272	85	52	77	124
DUSP13__NM_001007272_CTCATCGATATGGTTCAGTG	DUSP13__NM_001007272	1809	2239	2305	2690
DUSP13__NM_001007272_GCGCTTGCTGTGGGTCCGTC	DUSP13__NM_001007272	2041	2627	2319	3143
DUSP13__NM_001007272_GGAATGTCCCTGGAGTACTA	DUSP13__NM_001007272	471	452	451	443
DUSP13__NM_001007272_GGACATTCCACGGTAGAATT	DUSP13__NM_001007272	259	311	261	305
DUSP13__NM_001007272_GTTAGGGCAGATATTGCGGT	DUSP13__NM_001007272	480	417	528	408
DUSP13__NM_001007272_TGCAGCGGCATTCACAACGT	DUSP13__NM_001007272	419	450	559	466
DUSP13__NM_001007273_CAGGTTCTGGACAACCGACT	DUSP13__NM_001007273	546	673	503	513
DUSP13__NM_001007273_GAGACGGGGCGGTTCTGATC	DUSP13__NM_001007273	703	866	779	806
DUSP13__NM_001007273_GAGGGGGTGTAGGCGCCAGG	DUSP13__NM_001007273	578	582	538	551
DUSP14__NM_007026_AGCCTGGAGGAGGTGCCGAT	DUSP14__NM_007026	936	851	1088	1241
DUSP14__NM_007026_CAGGTACGCGATACACAGCG	DUSP14__NM_007026	842	1020	935	1022
DUSP14__NM_007026_CTCCCTCGGAAATCATCCGA	DUSP14__NM_007026	180	67	201	122
DUSP14__NM_007026_CTGGAGGAGGTGCCGATTGG	DUSP14__NM_007026	1794	1727	2030	2066
DUSP14__NM_007026_GAGGCAGTGTGGCCTCCAAT	DUSP14__NM_007026	167	157	176	147
DUSP14__NM_007026_GAGGTCACAGCACGCTACCA	DUSP14__NM_007026	299	216	257	318
DUSP14__NM_007026_GCACTTTAACATACTCAAAT	DUSP14__NM_007026	182	174	265	225
DUSP14__NM_007026_GCCTGCTGGAGGCGTACAAC	DUSP14__NM_007026	547	358	535	585
DUSP14__NM_007026_GGTGTCAAAGTACAGTCCAA	DUSP14__NM_007026	368	307	337	321
DUSP14__NM_007026_GTGTCAAAGTACAGTCCAAT	DUSP14__NM_007026	580	658	679	774
DUSP14__NM_007026_GTTGAAATTAGGGATCTCAA	DUSP14__NM_007026	238	139	178	142
DUSP14__NM_007026_TCCCTCGGAAATCATCCGAG	DUSP14__NM_007026	21	33	40	29
DUSP15__NM_001012644_AACTCCTCCTCGTCGCGGAA	DUSP15__NM_001012644	561	393	633	749
DUSP15__NM_001012644_AGCAGCTTGAAGAGTTTGGC	DUSP15__NM_001012644	793	744	940	1407
DUSP15__NM_001012644_CAAGCTGCTGCCTAAAGCCT	DUSP15__NM_001012644	830	929	954	878
DUSP15__NM_001012644_CCCCCGGTGTCTGTCCCGCA	DUSP15__NM_001012644	2193	2232	2444	2800
DUSP15__NM_001012644_CGGTGTCTGTCCCGCAAGGG	DUSP15__NM_001012644	460	605	503	503
DUSP15__NM_001012644_TCAAGCTGCTGCCTAAAGCC	DUSP15__NM_001012644	943	730	998	1462
DUSP15__NM_080611_ACTGCTGCCGCCTTAATGGG	DUSP15__NM_080611	748	607	740	677
DUSP15__NM_080611_CAAGGCAGTTCCCCCCATTA	DUSP15__NM_080611	446	405	550	395
DUSP15__NM_080611_CCTCAGGGGTATCAGCGACC	DUSP15__NM_080611	618	311	570	571
DUSP16__NM_030640_AACACAGACCGACTTGGTTA	DUSP16__NM_030640	727	712	997	1113
DUSP16__NM_030640_AAGAGGTGGAAGTGAGATAG	DUSP16__NM_030640	29	91	36	83
DUSP16__NM_030640_AAGTGCTGCTAATTGATAGC	DUSP16__NM_030640	125	165	159	182
DUSP16__NM_030640_AATGATGTTACGATTCCTAC	DUSP16__NM_030640	139	196	200	278
DUSP16__NM_030640_AATGCAGGTAGGGACTAGAG	DUSP16__NM_030640	606	717	554	579
DUSP16__NM_030640_AATTGATAGCCGGCCATTTG	DUSP16__NM_030640	609	646	693	592
DUSP16__NM_030640_ACACAGACCGACTTGGTTAT	DUSP16__NM_030640	1027	1192	1344	1415
DUSP16__NM_030640_ACTCTGCTGGACGGACCATT	DUSP16__NM_030640	1453	1789	1675	1403
DUSP16__NM_030640_AGAACACACAAGTACATGGT	DUSP16__NM_030640	422	402	440	456
DUSP16__NM_030640_AGACTTTCACATTTACTAGT	DUSP16__NM_030640	460	513	702	719
DUSP16__NM_030640_AGAGAAAAACCTGAGATACG	DUSP16__NM_030640	268	179	419	378
DUSP16__NM_030640_AGCACAACGTGAAAATGGTA	DUSP16__NM_030640	224	345	294	419
DUSP16__NM_030640_AGCCGCATTGTTATGATACT	DUSP16__NM_030640	1619	1865	1892	2290
DUSP16__NM_030640_AGGACATGTGTAAAGTTAGA	DUSP16__NM_030640	326	360	412	402
DUSP16__NM_030640_ATCACCCCTAGTAGACGCGA	DUSP16__NM_030640	361	472	397	335
DUSP16__NM_030640_ATCAGCCCCATCGCATGCCG	DUSP16__NM_030640	899	756	915	1002
DUSP16__NM_030640_ATTATCTCCATCTAGGCCCA	DUSP16__NM_030640	239	128	238	210
DUSP16__NM_030640_CAACATCACGATTTAAGCTA	DUSP16__NM_030640	697	1095	992	1240
DUSP16__NM_030640_CACAGACCGACTTGGTTATG	DUSP16__NM_030640	1181	1395	1332	1228
DUSP16__NM_030640_CACTAGGCTCTGTCCCTAAC	DUSP16__NM_030640	407	508	452	624
DUSP16__NM_030640_CAGCAACTAGCGACACTTAC	DUSP16__NM_030640	275	410	439	508
DUSP16__NM_030640_CATCATCCCCATAACCAAGT	DUSP16__NM_030640	699	975	690	682
DUSP16__NM_030640_CCAGCATTCAGCGAAACATA	DUSP16__NM_030640	48	54	88	60
DUSP16__NM_030640_CCTCAGCAGGCTATTGTTGG	DUSP16__NM_030640	981	1004	925	1179
DUSP16__NM_030640_CCTCGGCTGTCAGGTGAGCG	DUSP16__NM_030640	850	951	811	1016
DUSP16__NM_030640_CTCAACCTACAATCACTGTA	DUSP16__NM_030640	370	388	425	499
DUSP16__NM_030640_CTCAGCGCTAAGGTGTACTA	DUSP16__NM_030640	163	204	242	318
DUSP16__NM_030640_CTCCTGAAACATCGTTAGCA	DUSP16__NM_030640	354	570	364	441
DUSP16__NM_030640_CTCGGCTGTCAGGTGAGCGT	DUSP16__NM_030640	1253	1806	1414	1640
DUSP16__NM_030640_CTCTGCTGGACGGACCATTA	DUSP16__NM_030640	279	358	325	228
DUSP16__NM_030640_CTCTTCTAGTCTAGCTCACC	DUSP16__NM_030640	35	18	29	9
DUSP16__NM_030640_CTGGCTCAGTCTCAGCGCTA	DUSP16__NM_030640	224	321	243	344
DUSP16__NM_030640_CTTCCTAGGGAGGTAAATTA	DUSP16__NM_030640	143	237	236	202
DUSP16__NM_030640_CTTGGAGCAGTTGATATTAA	DUSP16__NM_030640	483	602	490	633
DUSP16__NM_030640_CTTTGCAGCCATAAACTCGG	DUSP16__NM_030640	104	118	94	124
DUSP16__NM_030640_GAAACCCTCGCGTCTACTAG	DUSP16__NM_030640	427	646	554	698
DUSP16__NM_030640_GAACCAGCACTAAAGGCTGT	DUSP16__NM_030640	599	449	558	543
DUSP16__NM_030640_GAACTCAAATTGTTACTGAG	DUSP16__NM_030640	360	273	439	513
DUSP16__NM_030640_GACCTTGGCAAGGCCCCTAA	DUSP16__NM_030640	17	23	37	14
DUSP16__NM_030640_GACTTCTATGTGAGAAGTGC	DUSP16__NM_030640	195	151	166	352
DUSP16__NM_030640_GATGTATTGTATTCCACAAA	DUSP16__NM_030640	262	213	310	356
DUSP16__NM_030640_GATGTTAAGAGAGTAACAAC	DUSP16__NM_030640	110	63	128	112
DUSP16__NM_030640_GCTCCAAGCTTATGAAGCGA	DUSP16__NM_030640	457	550	506	670
DUSP16__NM_030640_GCTCTCTTCAGCATGTAATT	DUSP16__NM_030640	67	88	109	105
DUSP16__NM_030640_GCTTTGACCACTACCATATC	DUSP16__NM_030640	463	576	459	701
DUSP16__NM_030640_GGAAACCCTCGCGTCTACTA	DUSP16__NM_030640	1860	2519	1936	2608
DUSP16__NM_030640_GGCTGAGAGAGGCTTTAGTG	DUSP16__NM_030640	678	624	863	954
DUSP16__NM_030640_GGGAAACCCTCGCGTCTACT	DUSP16__NM_030640	1247	1825	1319	1429
DUSP16__NM_030640_GTGAAAAAGCCTAAGACACT	DUSP16__NM_030640	367	344	373	251
DUSP16__NM_030640_TAAACTGTTAGCTAATCATC	DUSP16__NM_030640	91	87	144	126
DUSP16__NM_030640_TATCACCCCTAGTAGACGCG	DUSP16__NM_030640	350	406	405	340
DUSP16__NM_030640_TATGAAGCGAAGGTTGCAAC	DUSP16__NM_030640	399	214	339	325
DUSP16__NM_030640_TATTCACAATGCCTAGAAAA	DUSP16__NM_030640	109	175	196	245
DUSP16__NM_030640_TATTGTATTCCACAAATGGC	DUSP16__NM_030640	144	228	213	128
DUSP16__NM_030640_TCAAATTGTTACTGAGAGGT	DUSP16__NM_030640	292	414	319	539
DUSP16__NM_030640_TCACGATTTAAGCTAAGGTT	DUSP16__NM_030640	1191	1219	1359	1182
DUSP16__NM_030640_TCAGCCCCATCGCATGCCGA	DUSP16__NM_030640	588	677	642	736
DUSP16__NM_030640_TCAGGTAGGTAGACCCTGTT	DUSP16__NM_030640	959	1004	1174	1436
DUSP16__NM_030640_TCTGCTGGACGGACCATTAG	DUSP16__NM_030640	122	51	145	87
DUSP16__NM_030640_TGACTCAACACAGACCGACT	DUSP16__NM_030640	487	531	591	463
DUSP16__NM_030640_TGATTGTAGGTTGAGGACTG	DUSP16__NM_030640	160	231	175	198
DUSP16__NM_030640_TGGGCTACCAGATATGGTAG	DUSP16__NM_030640	68	37	50	62
DUSP16__NM_030640_TGGTGTTCCCACCGAGTTTA	DUSP16__NM_030640	87	80	85	80
DUSP16__NM_030640_TTATAGGAAACTTCCTAGGG	DUSP16__NM_030640	71	54	70	55
DUSP16__NM_030640_TTATCTCCATCTAGGCCCAC	DUSP16__NM_030640	202	218	210	323
DUSP16__NM_030640_TTATTTGTGCACAACTAGTG	DUSP16__NM_030640	35	59	40	95
DUSP16__NM_030640_TTCCAGAACACACAAGTACA	DUSP16__NM_030640	65	84	46	197
DUSP16__NM_030640_TTCCCAGTATCATAACAATG	DUSP16__NM_030640	79	129	95	79
DUSP16__NM_030640_TTCCTAGGGAGGTAAATTAT	DUSP16__NM_030640	123	62	96	101
DUSP16__NM_030640_TTTATCCCCTCGGCATGCGA	DUSP16__NM_030640	7	4	5	4
DUSP16__NM_030640_TTTGAGTTCCAATCATCTCA	DUSP16__NM_030640	278	374	266	308
DUSP16__NM_030640_TTTGCAGCCATAAACTCGGT	DUSP16__NM_030640	403	602	562	663
DUSP18__NM_152511_AAGTCACAGAGACGTGAGTT	DUSP18__NM_152511	1497	1853	1505	1622
DUSP18__NM_152511_ACATGGCGTGGTACTTCATG	DUSP18__NM_152511	2345	2100	2479	2490
DUSP18__NM_152511_AGTCACAGAGACGTGAGTTA	DUSP18__NM_152511	589	656	690	571
DUSP18__NM_152511_CCACACCATTGCTGATATAC	DUSP18__NM_152511	1416	1810	1520	1561
DUSP18__NM_152511_CCCTGCTGGACGCCCACACG	DUSP18__NM_152511	1341	1325	1513	1737
DUSP18__NM_152511_CCGCTGTTGGGTCGGATGAT	DUSP18__NM_152511	1621	1741	1708	2154
DUSP18__NM_152511_CTGAACTGGGAAGGCACACG	DUSP18__NM_152511	514	467	615	433
DUSP18__NM_152511_GAGGGCTGCCGGAACTGAAC	DUSP18__NM_152511	200	240	245	216
DUSP18__NM_152511_GCATGACTTGGTCCACGTGT	DUSP18__NM_152511	1533	1432	1718	1248
DUSP18__NM_152511_GCCGCTGTTGGGTCGGATGA	DUSP18__NM_152511	1527	2244	1646	2021
DUSP18__NM_152511_GTCACAGAGACGTGAGTTAG	DUSP18__NM_152511	1064	825	1427	1180
DUSP18__NM_152511_TGCTCCCAAAAGCCGCTGTT	DUSP18__NM_152511	329	559	395	504
DUSP19__NM_001142314_AAGTTGGCGTTATTAAGCCA	DUSP19__NM_001142314	381	509	318	298
DUSP19__NM_001142314_ACCAGGGTGACAACGCTAAC	DUSP19__NM_001142314	503	670	706	915
DUSP19__NM_001142314_AGATGCCAGAATTCATGTTG	DUSP19__NM_001142314	1189	1141	1201	1388
DUSP19__NM_001142314_ATTGCTTCCTGAGATTATTC	DUSP19__NM_001142314	215	397	238	277
DUSP19__NM_001142314_CAACTTGCAGGTCCGAGCTA	DUSP19__NM_001142314	706	963	715	921
DUSP19__NM_001142314_CAGCTTCGTACATATCAAGA	DUSP19__NM_001142314	453	317	413	403
DUSP19__NM_001142314_CCAGAATTTGGACATATGGA	DUSP19__NM_001142314	255	221	334	326
DUSP19__NM_001142314_CCTTCCATATGTCCAAATTC	DUSP19__NM_001142314	588	587	713	866
DUSP19__NM_001142314_GCAGCTTCGTACATATCAAG	DUSP19__NM_001142314	742	575	751	766
DUSP19__NM_001142314_GGGTGGTTGTGGTTATGTGC	DUSP19__NM_001142314	29	26	41	21
DUSP19__NM_001142314_GTTCTTGTTCATTGTAATGC	DUSP19__NM_001142314	49	39	48	61
DUSP19__NM_001142314_TGTAATGTACTCCCTTAACC	DUSP19__NM_001142314	279	296	360	441
DUSP19__NM_080876_TCTTATATGTAAAGTCACTG	DUSP19__NM_080876	429	414	500	452
DUSP2__NM_004418_CAGAGCGCGAGATACCCGCC	DUSP2__NM_004418	1674	1798	1852	1966
DUSP2__NM_004418_CAGGAGGCCATAGGCTTCAT	DUSP2__NM_004418	769	796	864	803
DUSP2__NM_004418_CATGAGGTATGCCAGACAGA	DUSP2__NM_004418	111	258	122	119
DUSP2__NM_004418_CCACAGGGATACTCTTGTAG	DUSP2__NM_004418	1034	1085	1226	903
DUSP2__NM_004418_CCCTGTGGAGGACAACCAGA	DUSP2__NM_004418	273	294	283	223
DUSP2__NM_004418_CCGCTACAAGAGTATCCCTG	DUSP2__NM_004418	1450	1395	1407	1397
DUSP2__NM_004418_CGCAGGAAGTACACGGCAGT	DUSP2__NM_004418	154	315	154	114
DUSP2__NM_004418_TCATGCAGAGTCGCCGTGTG	DUSP2__NM_004418	1844	2566	2171	2536
DUSP2__NM_004418_TGTGGAGGACAACCAGATGG	DUSP2__NM_004418	638	562	538	542
DUSP2__NM_004418_TTGACTTCGTTAAGCAGCGC	DUSP2__NM_004418	13	3	16	27
DUSP21__NM_022076_AAACAAGCTTCTGGTTATTT	DUSP21__NM_022076	301	371	313	477
DUSP21__NM_022076_AATGGCGGTGATGCGATTGC	DUSP21__NM_022076	496	889	564	686
DUSP21__NM_022076_ACGGCCCTGCCTCATATCGA	DUSP21__NM_022076	1227	1850	1275	1577
DUSP21__NM_022076_AGTGGTCAACGTATTCTTCG	DUSP21__NM_022076	467	598	621	846
DUSP21__NM_022076_CAGAAGGAGTTTGTCGTTGG	DUSP21__NM_022076	1443	2048	1932	2356
DUSP21__NM_022076_CCACACCATCGATATGAGGC	DUSP21__NM_022076	861	1209	1090	1196
DUSP21__NM_022076_CCTGCCTCATATCGATGGTG	DUSP21__NM_022076	1219	1636	1498	1712
DUSP21__NM_022076_GAGTCACGAGCATCGGTAAC	DUSP21__NM_022076	791	883	800	973
DUSP21__NM_022076_GGTATTTCATGAGGTACGCA	DUSP21__NM_022076	396	569	479	600
DUSP21__NM_022076_GTGGTCAACGTATTCTTCGA	DUSP21__NM_022076	76	118	89	132
DUSP21__NM_022076_GTGTGGATAAGATCAGCAAT	DUSP21__NM_022076	105	162	101	161
DUSP21__NM_022076_TTATCCACACCATCGATATG	DUSP21__NM_022076	897	1023	1050	1345
DUSP22__NM_001286555_ACATAGGCCTGGCACTATCG	DUSP22__NM_001286555	1259	1194	1237	967
DUSP22__NM_001286555_CCCTGCACACCGTGCGTGCT	DUSP22__NM_001286555	752	942	892	1084
DUSP22__NM_001286555_CTGGGTAAATATAAGGAGCA	DUSP22__NM_001286555	307	264	361	466
DUSP22__NM_001286555_CTTTGAAGTTGCCGATGTAC	DUSP22__NM_001286555	728	926	1033	1211
DUSP22__NM_001286555_GACCTACGATAATTATACGA	DUSP22__NM_001286555	344	393	428	313
DUSP22__NM_001286555_GCTTCTTCTGCATCCTGCAA	DUSP22__NM_001286555	367	465	399	496
DUSP22__NM_001286555_GGTCTCCAGGAGCGTGACAC	DUSP22__NM_001286555	254	440	422	510
DUSP22__NM_001286555_GTATAATTATCGTAGGTCAG	DUSP22__NM_001286555	163	58	161	190
DUSP22__NM_001286555_TCCTGTGCCAACCCCAACGT	DUSP22__NM_001286555	792	752	741	522
DUSP22__NM_001286555_TCTTACCTCCAACATAGGCC	DUSP22__NM_001286555	52	48	57	38
DUSP22__NM_001286555_TGTCTGTCCACGATAGTGCC	DUSP22__NM_001286555	165	257	258	323
DUSP22__NM_001286555_TTAAATTCATTCACGAGTGC	DUSP22__NM_001286555	113	152	108	282
DUSP22__NM_020185_GAACTTCAGAATTCCCGGAG	DUSP22__NM_020185	187	126	229	276
DUSP23__NM_017823_CACCATGCTGGCCTGTTACC	DUSP23__NM_017823	1194	1282	1350	1231
DUSP23__NM_017823_CACGAAGCGGTCGATCTGGT	DUSP23__NM_017823	2297	1875	2260	2616
DUSP23__NM_017823_CCGCTCCTTCACCAGGTAAC	DUSP23__NM_017823	600	628	667	740
DUSP23__NM_017823_CGACCGCTTCGTGCAGATCG	DUSP23__NM_017823	2400	2663	2494	2751
DUSP23__NM_017823_CTCCATCGAGACCTATGAGC	DUSP23__NM_017823	767	594	779	546
DUSP23__NM_017823_CTCCTGCTCATAGGTCTCGA	DUSP23__NM_017823	125	241	170	232
DUSP23__NM_017823_GAAGCGGTCGATCTGGTCGG	DUSP23__NM_017823	2162	2137	2147	1804
DUSP23__NM_017823_GTCCAACAGGAACTGGTAGT	DUSP23__NM_017823	722	477	754	546
DUSP23__NM_017823_TCGTGGACGAGGCCAACGCA	DUSP23__NM_017823	232	202	245	307
DUSP23__NM_017823_TCTACCAGCGAACGAAATAA	DUSP23__NM_017823	76	31	104	89
DUSP23__NM_017823_TGGAGCCGGGTCGTAGTCGT	DUSP23__NM_017823	921	974	976	935
DUSP23__NM_017823_TTCTACCAGCGAACGAAATA	DUSP23__NM_017823	0	0	0	0
DUSP26__NM_024025_AAGCCCAAAGCCAGTTACCA	DUSP26__NM_024025	574	572	625	617
DUSP26__NM_024025_AGGAAAGGATGTTGAACGGT	DUSP26__NM_024025	311	202	377	256
DUSP26__NM_024025_AGGGTCCCTCGAGTTCGAAC	DUSP26__NM_024025	340	100	463	322
DUSP26__NM_024025_AGGTCTCCTGTTCGAACTCG	DUSP26__NM_024025	516	762	499	682
DUSP26__NM_024025_CAGGAGACCTTGAGCTACTC	DUSP26__NM_024025	452	214	604	397
DUSP26__NM_024025_CTAAGATGTGCCCTGGTAAC	DUSP26__NM_024025	864	493	885	718
DUSP26__NM_024025_GAAGCCCAAAGCCAGTTACC	DUSP26__NM_024025	441	341	565	348
DUSP26__NM_024025_GACCTCGTCGGCATGGTTAC	DUSP26__NM_024025	603	400	782	632
DUSP26__NM_024025_GGTCTCCTGTTCGAACTCGA	DUSP26__NM_024025	301	393	390	408
DUSP26__NM_024025_TGTGCCCTGGTAACTGGCTT	DUSP26__NM_024025	938	920	1122	1031
DUSP26__NM_024025_TTGGAGCGGCTCCTCTACAC	DUSP26__NM_024025	1301	584	1652	913
DUSP26__NM_024025_TTTCCTCAATGTCTTCGAGT	DUSP26__NM_024025	544	281	593	417
DUSP27__NM_001080426_ACAGGGCGTATTATAGAGGC	DUSP27__NM_001080426	435	339	488	647
DUSP27__NM_001080426_ACCTCATCCACCTCATTCCA	DUSP27__NM_001080426	2193	1877	2194	2015
DUSP27__NM_001080426_AGGACCTGTACAACCGCGTC	DUSP27__NM_001080426	834	904	775	688
DUSP27__NM_001080426_CACATTCTGAATGCTGCGCA	DUSP27__NM_001080426	1053	1317	1192	1706
DUSP27__NM_001080426_CCAGGCCAGTGTAGAATTCG	DUSP27__NM_001080426	957	819	942	1226
DUSP27__NM_001080426_CTGAGACCATCGAATACCTA	DUSP27__NM_001080426	899	1044	1033	998
DUSP27__NM_001080426_CTTCTCAGCTATGAAGACAT	DUSP27__NM_001080426	385	497	504	483
DUSP27__NM_001080426_GGACACAGGGCGTATTATAG	DUSP27__NM_001080426	262	232	207	320
DUSP27__NM_001080426_GGACCTGTACAACCGCGTCA	DUSP27__NM_001080426	841	834	1009	811
DUSP27__NM_001080426_TCCAGGCCAGTGTAGAATTC	DUSP27__NM_001080426	204	175	208	266
DUSP27__NM_001080426_TCTCCCTGACGCGGTTGTAC	DUSP27__NM_001080426	693	578	755	749
DUSP27__NM_001080426_TTCGGGGCCAGTGTAAACGC	DUSP27__NM_001080426	12	21	18	85
DUSP28__NM_001033575_CCCGGTAGCAGAACCGAACC	DUSP28__NM_001033575	908	979	1040	1435
DUSP28__NM_001033575_CGGTGGAGGTACGGGCGAGG	DUSP28__NM_001033575	601	489	512	543
DUSP28__NM_001033575_CTCAGCCGGGTCGTCGAACA	DUSP28__NM_001033575	991	1436	1211	1814
DUSP28__NM_001033575_GAGGGCGCGACGCGCACCAA	DUSP28__NM_001033575	0	0	0	0
DUSP28__NM_001033575_GCGACGCGCACCAACGGTGG	DUSP28__NM_001033575	0	0	0	0
DUSP28__NM_001033575_GCGCACCAACGGTGGAGGTA	DUSP28__NM_001033575	235	139	284	278
DUSP28__NM_001033575_GCGGTGCCGCATGAGGTACG	DUSP28__NM_001033575	711	1101	766	832
DUSP28__NM_001033575_GCGTACCTCATGCGGCACCG	DUSP28__NM_001033575	1781	2097	1867	2357
DUSP28__NM_001033575_GTTCTTGCAGTAGACTAGGC	DUSP28__NM_001033575	124	50	67	95
DUSP28__NM_001033575_TCAGCCGGGTCGTCGAACAC	DUSP28__NM_001033575	794	624	662	551
DUSP28__NM_001033575_TCTGCACCGCGTACCTCATG	DUSP28__NM_001033575	1431	1320	1684	1615
DUSP28__NM_001033575_TGAGGCCGCGGTGCCGCATG	DUSP28__NM_001033575	328	331	392	485
DUSP3__NM_004090_ACCTCAGCGCTTACTTTGAA	DUSP3__NM_004090	1227	1388	1466	1991
DUSP3__NM_004090_ATTGGTGTTGACGTGCATGA	DUSP3__NM_004090	516	609	512	574
DUSP3__NM_004090_CATCAAGGCCAACGACACAC	DUSP3__NM_004090	189	155	234	254
DUSP3__NM_004090_CGCGGGGTGACCTCGTTGCA	DUSP3__NM_004090	3785	5119	3950	4542
DUSP3__NM_004090_CTCGAACGAGCCCGACATGG	DUSP3__NM_004090	38	27	95	106
DUSP3__NM_004090_GACTCCGGCATCACATACCT	DUSP3__NM_004090	2106	2243	2176	1869
DUSP3__NM_004090_GCCAACTTCTACAAGGACTC	DUSP3__NM_004090	295	344	401	301
DUSP3__NM_004090_GCGTTGCCCACGTAGATCCG	DUSP3__NM_004090	14	36	8	0
DUSP3__NM_004090_GGTCACCCCGCGGATCTACG	DUSP3__NM_004090	770	882	834	748
DUSP3__NM_004090_GTCACCCCGCGGATCTACGT	DUSP3__NM_004090	1361	951	1457	1334
DUSP3__NM_004090_GTTGAACTCCTGTGTGTCGT	DUSP3__NM_004090	2393	2624	2682	3543
DUSP3__NM_004090_GTTGCCCACGTAGATCCGCG	DUSP3__NM_004090	984	717	1047	958
DUSP4__NM_001394_AAAGTGGTTTGGGCAGTCCG	DUSP4__NM_001394	404	231	349	282
DUSP4__NM_001394_ACACGGTGCTGTCCTCGCGG	DUSP4__NM_001394	1617	1172	1602	1664
DUSP4__NM_001394_ATCGTGCGGCGGCGGGCTAA	DUSP4__NM_001394	1437	1103	1782	1918
DUSP4__NM_001394_ATGAACCGGGACGAGAATGG	DUSP4__NM_001394	196	116	217	222
DUSP4__NM_001394_ATGGAAGCCATAGAGTACAT	DUSP4__NM_001394	594	617	659	718
DUSP4__NM_001394_CGCGGGCTACATCCTAGGTT	DUSP4__NM_001394	1335	799	1624	1553
DUSP4__NM_001394_CTGATGAACCGGGACGAGAA	DUSP4__NM_001394	609	967	628	793
DUSP4__NM_001394_GCGCTCCGGCCTCTACTCGG	DUSP4__NM_001394	2174	1876	2538	2875
DUSP4__NM_001394_GTCTACCTGGTCGTGTAGTG	DUSP4__NM_001394	439	353	585	721
DUSP4__NM_001394_TCAGTACAAGTGCATCCCAG	DUSP4__NM_001394	758	546	880	862
DUSP4__NM_001394_TGCGCTGTAACACCATCGTG	DUSP4__NM_001394	289	247	424	442
DUSP4__NM_001394_TGTCGGTGCGCTCGGCGTTG	DUSP4__NM_001394	668	539	815	688
DUSP4__NM_057158_AACATAGCAGATCGCCCAGG	DUSP4__NM_057158	763	685	1010	991
DUSP4__NM_057158_AAGCCATAGAGTACATCGGT	DUSP4__NM_057158	1754	960	2101	1994
DUSP4__NM_057158_ATGCCGTGAAGGACTGCCGT	DUSP4__NM_057158	1782	1164	1797	2071
DUSP4__NM_057158_CCAGGCGGGCATCTCGCGGT	DUSP4__NM_057158	4389	3607	5029	5120
DUSP4__NM_057158_CGAGCCCCCAGCATGGCGTT	DUSP4__NM_057158	0	0	1	7
DUSP4__NM_057158_GCTGACACCTAACGCCATGC	DUSP4__NM_057158	189	168	214	164
DUSP4__NM_057158_GGAAGAAAAGTTCACTCCAA	DUSP4__NM_057158	214	292	220	222
DUSP4__NM_057158_GGCAGTCCTTCACGGCATCT	DUSP4__NM_057158	2254	2871	2629	3329
DUSP5__NM_004419_ATGAAGGTCACGTCGCTCGA	DUSP5__NM_004419	778	1056	937	1376
DUSP5__NM_004419_CCTTTGAGGAAGTAGACCCG	DUSP5__NM_004419	598	616	735	658
DUSP5__NM_004419_CGAGGTGAGGACGACACGCG	DUSP5__NM_004419	1525	1609	1850	1496
DUSP5__NM_004419_CTACAGGCCAGCTTATGACC	DUSP5__NM_004419	431	589	594	752
DUSP5__NM_004419_CTCAATCTTCTCTTGTGAAA	DUSP5__NM_004419	108	159	116	172
DUSP5__NM_004419_GCTCGACTGCCGGCCCTATC	DUSP5__NM_004419	524	574	733	772
DUSP5__NM_004419_GGAACTCGCACTTGGATGCA	DUSP5__NM_004419	1549	1466	1704	1916
DUSP5__NM_004419_GGCAAGCGAGTAGCGAGGTG	DUSP5__NM_004419	151	158	141	178
DUSP5__NM_004419_GGGTAGGCAAGCGAGTAGCG	DUSP5__NM_004419	74	43	91	76
DUSP5__NM_004419_GGTAGGCACTTCCAAGGTAG	DUSP5__NM_004419	53	38	152	85
DUSP5__NM_004419_TTCGCTGCCTCGAACGTGCG	DUSP5__NM_004419	341	297	297	363
DUSP5__NM_004419_TTTACATCCACGCAACACTC	DUSP5__NM_004419	183	198	247	384
DUSP6__NM_001946_AGCGACTGGAACGAGAATAC	DUSP6__NM_001946	172	201	253	221
DUSP6__NM_001946_ATCGCCATTTCCGACGCGAA	DUSP6__NM_001946	544	491	566	696
DUSP6__NM_001946_CAGACCCGTGCCCTTCGCGT	DUSP6__NM_001946	8401	8725	9185	8479
DUSP6__NM_001946_CAGCGACTGGAACGAGAATA	DUSP6__NM_001946	297	316	300	314
DUSP6__NM_001946_CGTGCCCTTCGCGTCGGAAA	DUSP6__NM_001946	719	838	812	847
DUSP6__NM_001946_CTCAAGAAGCTCAAGGACGA	DUSP6__NM_001946	151	236	209	127
DUSP6__NM_001946_GACTGGAACGAGAATACGGG	DUSP6__NM_001946	1088	946	1050	811
DUSP6__NM_001946_GATCGCCATTTCCGACGCGA	DUSP6__NM_001946	772	852	764	1051
DUSP6__NM_001946_GGGCTGCCGGGCGTTCTACC	DUSP6__NM_001946	829	645	994	685
DUSP6__NM_001946_GGGTCTGAGCGTATCTATCA	DUSP6__NM_001946	411	600	566	491
DUSP6__NM_001946_GTGCGCGCGCTCTTCACGCG	DUSP6__NM_001946	431	526	513	574
DUSP6__NM_001946_TAGATTGGTCTCGCAATGCA	DUSP6__NM_001946	486	561	475	439
DUSP6__NM_022652_GTTGAAGTTAGGGGATATGT	DUSP6__NM_022652	546	474	572	537
DUSP7__NM_001947_AATGCAGAGCTAAGTCAGAC	DUSP7__NM_001947	178	198	242	284
DUSP7__NM_001947_ACAAAGTCCCGTTGTTGGTT	DUSP7__NM_001947	456	636	436	548
DUSP7__NM_001947_ACCCATTTCAACCCTAGGGC	DUSP7__NM_001947	394	613	502	531
DUSP7__NM_001947_ACGCCTACGACTTTGTCAAG	DUSP7__NM_001947	1196	1504	1256	1716
DUSP7__NM_001947_AGGCATCAGCCGCTCAGTGA	DUSP7__NM_001947	736	521	582	360
DUSP7__NM_001947_ATATATCCCGAACCAACAAC	DUSP7__NM_001947	103	43	116	132
DUSP7__NM_001947_ATCCTGCCCTACCTCTACCT	DUSP7__NM_001947	1644	1091	1560	923
DUSP7__NM_001947_CAAAGTCCCGTTGTTGGTTC	DUSP7__NM_001947	844	1117	819	1027
DUSP7__NM_001947_CAACTTGCAGCAGTCATCAC	DUSP7__NM_001947	668	629	769	820
DUSP7__NM_001947_CACAGGGCTGCCGTCTGACT	DUSP7__NM_001947	2383	2035	2423	1876
DUSP7__NM_001947_CCACTGCTTCTTACCTCACG	DUSP7__NM_001947	1176	814	1160	855
DUSP7__NM_001947_CCATTTCAACCCTAGGGCGG	DUSP7__NM_001947	402	337	448	419
DUSP7__NM_001947_CCCATTTCAACCCTAGGGCG	DUSP7__NM_001947	613	622	585	665
DUSP7__NM_001947_CCCCCGCCCTAGGGTTGAAA	DUSP7__NM_001947	398	319	447	551
DUSP7__NM_001947_CGCGCAGCTTCTGTAGGAGC	DUSP7__NM_001947	1066	849	1120	1032
DUSP7__NM_001947_CGTGCTCGAAGGCGTTGGGT	DUSP7__NM_001947	2102	1718	2111	1796
DUSP7__NM_001947_CTGGACGTGCTCGGCAAGTA	DUSP7__NM_001947	400	414	499	456
DUSP7__NM_001947_CTTACTGCTCTTCAGGTATT	DUSP7__NM_001947	355	511	431	536
DUSP7__NM_001947_GAGGAAGATAACGGAATCCG	DUSP7__NM_001947	177	79	198	197
DUSP7__NM_001947_GAGGGCAGGGCAAGCTTCCA	DUSP7__NM_001947	668	510	747	427
DUSP7__NM_001947_GCTCCGACACCGATCAGCCT	DUSP7__NM_001947	1127	779	1211	927
DUSP7__NM_001947_GCTGGGGAGACATTAGGTGG	DUSP7__NM_001947	394	266	431	241
DUSP7__NM_001947_GGACCAGCAGAAGGGGGGTA	DUSP7__NM_001947	315	435	432	575
DUSP7__NM_001947_GGCTCTTGGACACGTGGCTT	DUSP7__NM_001947	193	242	224	270
DUSP7__NM_001947_GGTGGGAGGCGGCACGACTG	DUSP7__NM_001947	538	567	627	460
DUSP7__NM_001947_GTAAATGAGGGTATGCCAGA	DUSP7__NM_001947	144	162	125	77
DUSP7__NM_001947_GTGTGTGTATATATATACGT	DUSP7__NM_001947	298	242	328	100
DUSP7__NM_001947_GTTGAAGTTGGGCGAGATGT	DUSP7__NM_001947	997	1196	1298	1529
DUSP7__NM_001947_TACCCTCATTTACACAAATC	DUSP7__NM_001947	484	441	397	430
DUSP7__NM_001947_TATAAACAAAGTCCCGTTGT	DUSP7__NM_001947	609	437	597	399
DUSP7__NM_001947_TATATATCCCGAACCAACAA	DUSP7__NM_001947	0	0	0	0
DUSP7__NM_001947_TCCTCTCTCTTGTTGTAACT	DUSP7__NM_001947	221	117	279	258
DUSP7__NM_001947_TCGAAGGCGTTGGGTAGGTT	DUSP7__NM_001947	990	1116	1080	1306
DUSP7__NM_001947_TCGACGCGTGGTTGTCGCAC	DUSP7__NM_001947	596	325	578	235
DUSP7__NM_001947_TGACTGCTCCGACGGCGAGT	DUSP7__NM_001947	1290	684	1326	769
DUSP7__NM_001947_TGTTGTCAATTTCTCGACTG	DUSP7__NM_001947	420	421	602	516
DUSP7__NM_001947_TTGCACCCATTTCAACCCTA	DUSP7__NM_001947	591	368	577	319
DUSP7__NM_001947_TTGGGGATGATGGAGCGGAT	DUSP7__NM_001947	199	79	194	184
DUSP7__NM_001947_TTTCAGGCTACATGCAACAG	DUSP7__NM_001947	230	336	266	239
DUSP8__NM_004420_AGGCTTGCCCTCGCAGAGGC	DUSP8__NM_004420	82	118	84	109
DUSP8__NM_004420_CATCCTGCCTCACCTCTACC	DUSP8__NM_004420	394	195	539	319
DUSP8__NM_004420_GAGGCTCCGGCGTCCCTGAG	DUSP8__NM_004420	111	117	179	114
DUSP8__NM_004420_GCAAGCTGGACAGCCCGTCG	DUSP8__NM_004420	1275	2017	1532	1672
DUSP8__NM_004420_GCAGAAGGACGTCCTAAACA	DUSP8__NM_004420	769	868	832	724
DUSP8__NM_004420_GCAGCGCTCTCTGAGGTAGG	DUSP8__NM_004420	1062	537	1132	815
DUSP8__NM_004420_GGGAGAGGCTCATGGGTAGC	DUSP8__NM_004420	535	745	597	647
DUSP8__NM_004420_GTGGCTCCGTAGCCTCCACC	DUSP8__NM_004420	169	88	119	75
DUSP8__NM_004420_TCACAGTAGTTGTCGTTGAT	DUSP8__NM_004420	712	779	850	946
DUSP8__NM_004420_TGCAAGCTGGACAGCCCGTC	DUSP8__NM_004420	1077	1561	1061	1721
DUSP8__NM_004420_TGGTCTATGACCAGAGCACG	DUSP8__NM_004420	13	0	10	0
DUSP8__NM_004420_TGTCCTCCGACGACGCCTAC	DUSP8__NM_004420	197	151	124	195
DUSP9__NM_001395_ACGAGTCGGCGCGCATCGGT	DUSP9__NM_001395	7194	6957	7782	7348
DUSP9__NM_001395_ACTGGAGCCAGAACCTGTCG	DUSP9__NM_001395	1819	2074	1918	2395
DUSP9__NM_001395_ATCCTGCCCAACCTCTATCT	DUSP9__NM_001395	1586	1705	1834	1484
DUSP9__NM_001395_CATTCTTCTCGAAGAAGTTT	DUSP9__NM_001395	566	628	594	874
DUSP9__NM_001395_CCATTCTTCTCGAAGAAGTT	DUSP9__NM_001395	517	792	525	739
DUSP9__NM_001395_CCGGAGGCCATTGAGTTCAT	DUSP9__NM_001395	82	122	112	133
DUSP9__NM_001395_CCTCCGGAAAGAACCGCGAC	DUSP9__NM_001395	469	464	559	560
DUSP9__NM_001395_CGAGTCGGCGCGCATCGGTG	DUSP9__NM_001395	739	765	801	865
DUSP9__NM_001395_GATCCTGCCCAACCTCTATC	DUSP9__NM_001395	957	936	1230	1490
DUSP9__NM_001395_GCTCGTGCCTGTGGCTGCGT	DUSP9__NM_001395	1099	1367	1441	1706
DUSP9__NM_001395_TCGAAGAAGTTTGGGAGGTT	DUSP9__NM_001395	668	1198	683	897
DUSP9__NM_001395_TGGCTCCAGTGGTCGGAGAT	DUSP9__NM_001395	16	17	22	20
DUT__NM_001025248_AAGGGCGAGCTTCCTAAGGC	DUT__NM_001025248	175	91	181	115
DUT__NM_001025248_ACTCTTCCATAACACCCAGA	DUT__NM_001025248	423	53	428	10
DUT__NM_001025248_AGGAAATGGTAGCAGAGCGC	DUT__NM_001025248	14	9	8	9
DUT__NM_001025248_AGGGCGAGCTTCCTAAGGCG	DUT__NM_001025248	480	332	587	647
DUT__NM_001025248_CCCGGAGAGTACCGCGGCTT	DUT__NM_001025248	2775	3444	3349	3405
DUT__NM_001025248_CCGCTTACTGGGTGAAATGG	DUT__NM_001025248	789	778	916	979
DUT__NM_001025248_GCAGGCCGGGCCCGCTTACT	DUT__NM_001025248	1438	439	1392	653
DUT__NM_001025248_GCCTGGCCCGGAGAGTACCG	DUT__NM_001025248	405	494	461	523
DUT__NM_001025248_GCCTTGGATGACACCGAAAG	DUT__NM_001025248	235	142	327	193
DUT__NM_001025248_GGCGAGCTTCCTAAGGCGGG	DUT__NM_001025248	1207	989	1508	1342
DUT__NM_001025248_TTGCACAGCTCATTTGCGAA	DUT__NM_001025248	128	201	150	165
DUT__NM_001025248_TTTGCAGCCAAGCCTGACCG	DUT__NM_001025248	402	2	505	13
DUXA__NM_001012729_CTCACTTGGACTCTTGACTC	DUXA__NM_001012729	369	565	389	597
DUXA__NM_001012729_GAAGAACTTGCTAAAGAAAT	DUXA__NM_001012729	155	162	142	85
DUXA__NM_001012729_GATGAGAGTGTGTAACTGAG	DUXA__NM_001012729	165	294	227	233
DUXA__NM_001012729_GGTTTCAGAATCGAAGAGCT	DUXA__NM_001012729	173	61	202	89
DUXA__NM_001012729_GTAGGTGGTACGACACCGTC	DUXA__NM_001012729	7	10	19	25
DUXA__NM_001012729_TACAGAAGAGTCCAGAATCC	DUXA__NM_001012729	495	578	479	439
DUXA__NM_001012729_TTGGTAGCATAACCTGGGTA	DUXA__NM_001012729	183	68	159	38
DVL1__NM_004421_AAGAATTTGTAGGCGTGCAC	DVL1__NM_004421	94	62	105	63
DVL1__NM_004421_AGGGTGCTCACTCGGATGCG	DVL1__NM_004421	595	510	568	544
DVL1__NM_004421_CCGCCTGTCCGCTCAAGAGG	DVL1__NM_004421	888	380	897	648
DVL1__NM_004421_CGAGGAGGAGACGCCGTACC	DVL1__NM_004421	921	722	1076	825
DVL1__NM_004421_CTCCCGCCGGTGACTGACCA	DVL1__NM_004421	185	175	295	444
DVL1__NM_004421_CTTGAGCGGACAGGCGGCAT	DVL1__NM_004421	887	651	1093	793
DVL1__NM_004421_GAAGAATTTGTAGGCGTGCA	DVL1__NM_004421	448	561	615	637
DVL1__NM_004421_GACCAAGATTATCTACCACA	DVL1__NM_004421	551	324	612	540
DVL1__NM_004421_GACGGGATGGACAACGAGAC	DVL1__NM_004421	194	176	273	170
DVL1__NM_004421_GGAGACCGACGGCGGGATGT	DVL1__NM_004421	1352	967	1769	1556
DVL1__NM_004421_GGCCAGCAGCCGTGACGGGA	DVL1__NM_004421	483	409	486	685
DVL1__NM_004421_TGAGGGTGCTCACTCGGATG	DVL1__NM_004421	241	145	266	254
DVL2__NM_004422_AGATAATCCCCAACCCGAGA	DVL2__NM_004422	217	368	277	306
DVL2__NM_004422_AGGTCCCTTGTCTACCCCTG	DVL2__NM_004422	442	514	569	384
DVL2__NM_004422_ATCGCCGAGGGTGATGCGCT	DVL2__NM_004422	687	762	891	769
DVL2__NM_004422_CATAGACTTGAAAAAGTACT	DVL2__NM_004422	104	316	118	115
DVL2__NM_004422_CATGGCTGCTGGACACATTA	DVL2__NM_004422	488	722	742	690
DVL2__NM_004422_CCAGCGGCATTGGGGACTCA	DVL2__NM_004422	955	693	1192	1263
DVL2__NM_004422_CCGAGAGGACCAGCGGCATT	DVL2__NM_004422	3438	3692	3872	3083
DVL2__NM_004422_CCGAGGCTCATGGACTGGAG	DVL2__NM_004422	697	1024	859	1241
DVL2__NM_004422_CCTTGAGTCCCCAATGCCGC	DVL2__NM_004422	818	903	838	998
DVL2__NM_004422_CTCGGGTTGGGGATTATCTG	DVL2__NM_004422	400	628	488	472
DVL2__NM_004422_CTGAGGAAGCTGGGAGATTT	DVL2__NM_004422	442	383	473	487
DVL2__NM_004422_GCCCTGCCCTTTGCAGGGTT	DVL2__NM_004422	178	229	132	207
DVL2__NM_004422_GCCTCCCCTGCTTCAACGGA	DVL2__NM_004422	752	783	846	968
DVL2__NM_004422_GGACGCTCTTGAAATCGCCG	DVL2__NM_004422	638	621	583	594
DVL2__NM_004422_GGTCACATTCTGGGGTTAGA	DVL2__NM_004422	470	587	569	552
DVL2__NM_004422_GTCTAGGATGCCTAACCCCG	DVL2__NM_004422	220	304	245	353
DVL2__NM_004422_GTGCTTGCTCTTACAGTGCC	DVL2__NM_004422	130	89	127	114
DVL2__NM_004422_TGGAGGGGCCATCTCGGGTT	DVL2__NM_004422	598	648	733	730
DVL3__NM_004423_AAGTCTATGGACGACGATTT	DVL3__NM_004423	223	310	339	269
DVL3__NM_004423_AGAAGGTTTCTCGGATTGAG	DVL3__NM_004423	328	412	330	381
DVL3__NM_004423_AGATCATCTACCACTTGGAT	DVL3__NM_004423	726	739	736	734
DVL3__NM_004423_AGGGGCAGCTTCACAAGGTA	DVL3__NM_004423	195	267	227	195
DVL3__NM_004423_ATGGACGACGATTTCGGGTG	DVL3__NM_004423	414	452	416	349
DVL3__NM_004423_CAGCTCACTGCTCATAAGGG	DVL3__NM_004423	181	231	176	118
DVL3__NM_004423_CCTCCCGTGCGCTCCATAGG	DVL3__NM_004423	1351	1441	1153	987
DVL3__NM_004423_CGGCTAAATGGAACTGCGAA	DVL3__NM_004423	134	193	127	103
DVL3__NM_004423_CGTCACCTTGGCGGACTTTA	DVL3__NM_004423	861	1676	1026	1240
DVL3__NM_004423_GAAGGTTTCTCGGATTGAGC	DVL3__NM_004423	338	465	355	362
DVL3__NM_004423_GTCACCTTGGCGGACTTTAA	DVL3__NM_004423	326	300	359	332
DVL3__NM_004423_TTGAAGAAGAACTTATAGCT	DVL3__NM_004423	67	35	41	34
DYDC1__NM_001269053_AGAGGAGCTCTTACTTCAGC	DYDC1__NM_001269053	180	98	190	304
DYDC1__NM_001269053_AGCTCAAGAACAATTAGGCA	DYDC1__NM_001269053	435	484	594	672
DYDC1__NM_001269053_CGATAGAATATTTAGCATTG	DYDC1__NM_001269053	303	320	363	326
DYDC1__NM_001269053_CTTGGGGCCTGTTTAACTCA	DYDC1__NM_001269053	408	296	424	449
DYDC1__NM_001269053_GCTGAAATCAGCGATCGTTA	DYDC1__NM_001269053	1978	2019	2120	2109
DYDC1__NM_001269053_TAGGAAGCAACACTAGACTC	DYDC1__NM_001269053	201	296	271	351
DYDC1__NM_001269053_TGCAAGACCTTGAGTTAAAC	DYDC1__NM_001269053	214	231	223	299
DYDC2__NM_001270041_AGTGAGCCAGGTATTCTATT	DYDC2__NM_001270041	696	724	731	871
DYDC2__NM_001270041_GAGGTGCTTTGGAAATTGCC	DYDC2__NM_001270041	207	208	193	297
DYDC2__NM_001270041_GATTCCAGGAATGCCTCAAC	DYDC2__NM_001270041	440	559	313	447
DYDC2__NM_001270041_GCCACAAGAAATAAATTACA	DYDC2__NM_001270041	484	550	494	608
DYDC2__NM_001270041_GGACACAAACCCCCTTGAGA	DYDC2__NM_001270041	517	343	562	488
DYDC2__NM_001270041_TGGCAGAGGTGGCGAAGGTT	DYDC2__NM_001270041	115	113	99	140
DYDC2__NM_032372_ACAATGAAAACACATCTGTT	DYDC2__NM_032372	2017	1893	2029	1631
DYDC2__NM_032372_ATATGGCTACTTGAGATAAT	DYDC2__NM_032372	49	0	37	0
DYDC2__NM_032372_CACAGCCTCCATACTAGCAT	DYDC2__NM_032372	501	211	530	286
DYDC2__NM_032372_CAGACCCAACACCCGGCCAT	DYDC2__NM_032372	2147	1761	2314	2310
DYDC2__NM_032372_CAGGGCTTGGAACATGTCCG	DYDC2__NM_032372	1133	433	1141	529
DYDC2__NM_032372_GGGGGCCAATGCTAGTATGG	DYDC2__NM_032372	157	0	213	22
DYDC2__NM_032372_TAATGGGAGTGCTAGAAGCA	DYDC2__NM_032372	25	0	18	2
DYDC2__NM_032372_TCTCTTTCCTTTGACTCGGC	DYDC2__NM_032372	329	542	323	531
DYDC2__NM_032372_TGGAAGCCCACACTATTTGT	DYDC2__NM_032372	221	182	300	118
DYDC2__NM_032372_TTGATCACCAACAAATAGTG	DYDC2__NM_032372	2	3	20	20
DYDC2__NM_032372_TTGGGAGAAAAGTTGAGGCA	DYDC2__NM_032372	137	36	186	47
DYDC2__NM_032372_TTGGGAGGAAAGCTTAGTGT	DYDC2__NM_032372	489	440	677	601
DYM__NM_017653_AAATCTTGGTGCACTAATTA	DYM__NM_017653	182	197	267	293
DYM__NM_017653_ACATAATGGCTTGTCTGTAG	DYM__NM_017653	819	1026	873	754
DYM__NM_017653_AGCCACAAGTATTTGATGCG	DYM__NM_017653	719	876	888	819
DYM__NM_017653_CATGTTGGCTCGCACAGATA	DYM__NM_017653	635	526	696	581
DYM__NM_017653_CGACTGCTGAGGGAAAACAT	DYM__NM_017653	844	742	740	636
DYM__NM_017653_CTTGAAAAAGTTATCAGGCA	DYM__NM_017653	440	516	531	528
DYM__NM_017653_GACATAATGGCTTGTCTGTA	DYM__NM_017653	698	814	733	1009
DYM__NM_017653_GAGTACTTGAAAAAGTTATC	DYM__NM_017653	132	143	202	198
DYM__NM_017653_GATGGAGCAAGGTATACAAG	DYM__NM_017653	291	325	347	362
DYM__NM_017653_GCACCAAGATTTCCTGTTCG	DYM__NM_017653	2112	2911	2281	3017
DYM__NM_017653_TCTCCTGCTTCTGCTGGTGT	DYM__NM_017653	1539	1430	1611	1688
DYM__NM_017653_TCTCTGAGAATGACCCGTTC	DYM__NM_017653	297	183	256	307
DYNAP__NM_173629_ACGCAGACCCCAGTTAAGTT	DYNAP__NM_173629	1269	1504	1491	1456
DYNAP__NM_173629_ACTGGGGTCTGCGTGAACCC	DYNAP__NM_173629	219	119	260	118
[illegible]					
DYNC1LI2__NM_001286157_GCTAGGTCCCAACGGGCCCG	DYNC1LI2__NM_001286157	71	66	61	32
DYNC1LI2__NM_001286157_GCTGGTCCACGACAAAGAGT	DYNC1LI2__NM_001286157	348	193	379	360
[illegible]					
DYRK1A__NM_001396_AGACGATTCTAGTCATAAGA	DYRK1A__NM_001396	625	1009	715	676
DYRK1A__NM_001396_ATATTGTCATGTTACAGAGG	DYRK1A__NM_001396	65	103	83	57
[illegible]					
DYTN__NM_001093730_TTGCACTGATTGTAATTTGA	DYTN__NM_001093730	371	501	434	434
DYTN__NM_001093730_TTGCAGATCCCAACTTTCGT	DYTN__NM_001093730	445	446	416	342
[illegible]					

ECSIT__NM_001142465_TCCACTTAGCCATCGTCGCC	ECSIT__NM_001142465	1080	1075	1148	1731
ECT2__NM_001258315_AAGTTTCTTTGAAGGTTCAA	ECT2__NM_001258315	62	44	76	35
ECT2__NM_001258315_AATCTAGTACTATGCTTTAC	ECT2__NM_001258315	451	43	519	46
ECT2__NM_001258315_AGTTTCTTTGAAGGTTCAAA	ECT2__NM_001258315	39	12	48	10
ECT2__NM_001258315_ATCCATTCTGGCTTCATAAT	ECT2__NM_001258315	364	59	309	58
ECT2__NM_001258315_CAAGCTTTATAAATCCATTC	ECT2__NM_001258315	227	6	241	29
ECT2__NM_001258315_GAACAATTTAATACAACTGG	ECT2__NM_001258315	128	0	108	12
ECT2__NM_001258315_GGAGGTAAATATTTACCGCT	ECT2__NM_001258315	370	139	410	100
ECT2__NM_001258315_GGATCTACTTCATATGTAGA	ECT2__NM_001258315	263	309	253	321
ECT2__NM_001258315_GTGACATTGGTCCATCACAT	ECT2__NM_001258315	467	1	455	2
ECT2__NM_001258315_TACTATGCTTTACTGGATTT	ECT2__NM_001258315	543	97	777	206
ECT2__NM_001258315_TCCAAGGAAAACTTACTTAT	ECT2__NM_001258315	213	204	252	333
ECT2__NM_001258315_TTCAAAAGTTACACATTTGG	ECT2__NM_001258315	15	0	11	31
ECT2__NM_018098_GAGAGAGTACGGTATATTTA	ECT2__NM_018098	189	147	284	259
ECT2__NM_018098_TAGCTGTTTCAGAGAGAGTA	ECT2__NM_018098	71	163	99	83
ECT2__NM_018098_TGATTTAGTTTGATAGTGTG	ECT2__NM_018098	319	395	377	483
ECT2L__NM_001077706_ACTCCAACAGATAATGAGTA	ECT2L__NM_001077706	357	306	377	372
ECT2L__NM_001077706_AGACTGGCTGACACCTAGGG	ECT2L__NM_001077706	482	248	563	476
ECT2L__NM_001077706_AGGTGTCCAGGCACTAAATC	ECT2L__NM_001077706	263	272	321	164
ECT2L__NM_001077706_ATGCCCAAATGCGTTAAGTT	ECT2L__NM_001077706	734	758	799	857
ECT2L__NM_001077706_CAAATCTTTCGGACTCAAAA	ECT2L__NM_001077706	250	312	318	370
ECT2L__NM_001077706_CAACAAGTCATTAAATAGAC	ECT2L__NM_001077706	276	260	386	399
ECT2L__NM_001077706_CATCCGAACTTAACGCATTT	ECT2L__NM_001077706	1275	1424	1496	1936
ECT2L__NM_001077706_CCATCCGAACTTAACGCATT	ECT2L__NM_001077706	1757	1921	2088	2391
ECT2L__NM_001077706_CTTAGACTGGCTGACACCTA	ECT2L__NM_001077706	311	335	368	330
ECT2L__NM_001077706_GACGTTGCTTGTTAGTCCAG	ECT2L__NM_001077706	649	550	719	657
ECT2L__NM_001077706_GGGACACACAAGCAATGTAA	ECT2L__NM_001077706	180	135	194	142
ECT2L__NM_001077706_TCCACACCAGATTTAGTGCC	ECT2L__NM_001077706	517	427	537	605
EDA__NM_001005609_AAGCAGCAGCCATTGGAACC	EDA__NM_001005609	465	763	593	510
EDA__NM_001005609_AGGGAAGAAGAAATTCAATA	EDA__NM_001005609	101	61	108	80
EDA__NM_001005609_CAAGCACACCACGTTCTTTG	EDA__NM_001005609	1239	1030	1371	1549
EDA__NM_001005609_CCAGCTGTGGTGCATCTACA	EDA__NM_001005609	864	908	890	1047
EDA__NM_001005609_GATGTAGCTTAAACACCTTG	EDA__NM_001005609	516	528	491	457
EDA__NM_001005609_GCAGGTGCTGCTGATAAAGC	EDA__NM_001005609	219	324	262	278
EDA__NM_001005609_GCGGCTTCTCCCGGTTCCAA	EDA__NM_001005609	398	332	384	304
EDA__NM_001005609_GGGATGTAGCTTAAACACCT	EDA__NM_001005609	149	109	151	148
EDA__NM_001005609_TACAACACTTGCTATACCGC	EDA__NM_001005609	577	714	691	490
EDA__NM_001005609_TGCACACGCAGCATCGAGAC	EDA__NM_001005609	200	163	170	206
EDA2R__NM_001199687_AAAATGAGTACTGGGACCAA	EDA2R__NM_001199687	280	221	296	318
EDA2R__NM_001199687_ACTGGTGAGCAGCCTGCTAG	EDA2R__NM_001199687	552	470	588	629
EDA2R__NM_001199687_CCAGGGTAAACACCACTAGC	EDA2R__NM_001199687	1051	1161	1298	1492
EDA2R__NM_001199687_CTGCCTCCACTAAGCTCAAC	EDA2R__NM_001199687	351	226	319	292
EDA2R__NM_001199687_CTTCCAGTTGAGCTTAGTGG	EDA2R__NM_001199687	883	897	1041	1002
EDA2R__NM_001199687_GCTACCTCTAATGCTGTCTG	EDA2R__NM_001199687	2836	4104	2858	3692
EDA2R__NM_001199687_GTGGGGGTCTGCTTCGTGCA	EDA2R__NM_001199687	375	473	396	499
EDA2R__NM_001199687_TACCGAAAGACACGCATTGG	EDA2R__NM_001199687	807	834	967	952
EDA2R__NM_001199687_TCCAGGACCACACCGTTGGC	EDA2R__NM_001199687	386	405	425	526
EDA2R__NM_001199687_TGTCATCAATCGTGTTCAGA	EDA2R__NM_001199687	218	284	296	290
EDA2R__NM_001199687_TTCTACCGAAAGACACGCAT	EDA2R__NM_001199687	13	18	0	0
EDA2R__NM_001199687_TTGCCAAGAAAATGAGTACT	EDA2R__NM_001199687	358	407	338	468
EDAR__NM_022336_AACAGACCGAGGAACATCTA	EDAR__NM_022336	278	350	328	329
EDAR__NM_022336_AGTACTACAACCAGACTACG	EDAR__NM_022336	367	311	275	329
EDAR__NM_022336_CAGCTGCTCATTCTCGGATG	EDAR__NM_022336	525	521	617	577
EDAR__NM_022336_CCTCACCTTTGTGGGCGTGC	EDAR__NM_022336	516	1198	710	771
EDAR__NM_022336_CCTTTGTGGGCGTGCTGGAA	EDAR__NM_022336	484	459	473	477
EDAR__NM_022336_CGAGTACTACAACCAGACTA	EDAR__NM_022336	554	426	658	776
EDAR__NM_022336_CGCAGTTTGAGTATTCCGCT	EDAR__NM_022336	1291	1757	1628	2018
EDAR__NM_022336_CTCCTCGTCCTTGCTCACTT	EDAR__NM_022336	1323	1294	1718	2052
EDAR__NM_022336_GGCACCAAAGACGAGGACTA	EDAR__NM_022336	376	405	473	481
EDAR__NM_022336_GTCTTTGGTGCCGTAGCCAC	EDAR__NM_022336	245	225	302	259
EDAR__NM_022336_TCTCCTCGTCCTTGCTCACT	EDAR__NM_022336	1231	1728	1412	1784
EDAR__NM_022336_TGCCAGGAGTGCCCCCCGTG	EDAR__NM_022336	466	774	614	695
EDARADD__NM_080738_AGGACTTACTAGACGTGATC	EDARADD__NM_080738	206	289	227	257
EDARADD__NM_080738_ATCATTGAGCAAGTCACTTA	EDARADD__NM_080738	625	582	591	674
EDARADD__NM_080738_CAAATATCCCATTCAAGATA	EDARADD__NM_080738	339	526	391	382
EDARADD__NM_080738_CAGGAAGCACAATTCGTCAT	EDARADD__NM_080738	903	720	1012	763
EDARADD__NM_080738_CGGAACAGTCAGAGGACGGT	EDARADD__NM_080738	947	746	1012	1104
EDARADD__NM_080738_GCTGGATCCGTGTCACCCAA	EDARADD__NM_080738	532	844	723	756
EDARADD__NM_080738_GGACGAGGAGTGGCCCAAGC	EDARADD__NM_080738	227	376	334	318
EDARADD__NM_080738_GGGAGTTCCGTATCTTGAAT	EDARADD__NM_080738	556	874	656	880
EDARADD__NM_080738_TGAGCAAGTCACTTATGGTG	EDARADD__NM_080738	180	89	160	100
EDARADD__NM_080738_TTGAGCAAGTCACTTATGGT	EDARADD__NM_080738	634	260	632	615
EDARADD__NM_145861_CCATCTGTTTAGTCGTCCTG	EDARADD__NM_145861	1341	1540	1585	1783
EDARADD__NM_145861_TCAGGACGACTAAACAGATG	EDARADD__NM_145861	140	143	163	150
EDC3__NM_001142443_AGTATTGTGTCCATCAATTG	EDC3__NM_001142443	355	425	528	530
EDC3__NM_001142443_CAAGTGGGCATCAATCAGAA	EDC3__NM_001142443	458	356	418	353
EDC3__NM_001142443_CACTTCACTCCATTATGGAA	EDC3__NM_001142443	415	425	488	318
EDC3__NM_001142443_CATTACGGAGTTAAAAATTC	EDC3__NM_001142443	73	108	115	99
EDC3__NM_001142443_CCCCTCAGAATATCCCTAAG	EDC3__NM_001142443	572	412	684	666
EDC3__NM_001142443_GATAGCTTGGGTGTCTATCA	EDC3__NM_001142443	127	76	179	228
EDC3__NM_001142443_GCCAGACCATTTCTCTCACC	EDC3__NM_001142443	1772	2144	2123	2501
EDC3__NM_001142443_GCCGGGTGAGAGAAATGGTC	EDC3__NM_001142443	904	575	956	1128
EDC3__NM_001142443_GCTCAAAGAGCTATGTCGAC	EDC3__NM_001142443	742	629	749	782
EDC3__NM_001142443_GTCCTCTTAGGGATATTCTG	EDC3__NM_001142443	399	391	431	464
EDC3__NM_001142443_TCCTCTTAGGGATATTCTGA	EDC3__NM_001142443	68	151	110	85
EDC3__NM_001142443_TCTTCACATCTGTCCTCTTA	EDC3__NM_001142443	778	924	914	1091
EDC4__NM_014329_AGCATTTCAAGCAAGGCCCG	EDC4__NM_014329	125	70	152	58
EDC4__NM_014329_AGGCAGGCAACCTGTTCGTG	EDC4__NM_014329	740	140	878	493
EDC4__NM_014329_CCAGCTTGAGGATGTCCCGC	EDC4__NM_014329	1006	697	1195	815
EDC4__NM_014329_CCGAATGGCATAGGCCAAGA	EDC4__NM_014329	122	85	218	127
EDC4__NM_014329_CGCCACTGAACCACTTTCGC	EDC4__NM_014329	2469	2829	2377	3104
EDC4__NM_014329_CTTCTGTTCCCAGTCATACT	EDC4__NM_014329	149	32	250	73
EDC4__NM_014329_GAGCCAAGCGCCACACGAAC	EDC4__NM_014329	524	603	474	623
EDC4__NM_014329_GCCATCCAGTGCCTACAATG	EDC4__NM_014329	465	353	522	402

EDC4__NM_014329_GGCCAAAATCCCAATGCAGG	EDC4__NM_014329	336	398	399	540
EDC4__NM_014329_TCCCAGTCATACTTGGCGAC	EDC4__NM_014329	668	66	747	328
EDC4__NM_014329_TGCCCTCTGGCTGCCGAATA	EDC4__NM_014329	483	497	576	844
EDC4__NM_014329_TGCTTCTCTTGCAGGTTAAT	EDC4__NM_014329	0	0	8	4
EDDM3A__NM_006683_AGCTACATTGAATTCCATTG	EDDM3A__NM_006683	820	1005	859	807
EDDM3A__NM_006683_CAAAGTACTCGAGTGTCACT	EDDM3A__NM_006683	1000	1033	1048	1106
EDDM3A__NM_006683_CATATCCATCTACGCCACAA	EDDM3A__NM_006683	367	199	468	362
EDDM3A__NM_006683_CGAGTACTTTGAGGGCACCT	EDDM3A__NM_006683	735	884	735	687
EDDM3A__NM_006683_GAATTCCATTGTGGCGTAGA	EDDM3A__NM_006683	616	743	703	768
EDDM3A__NM_006683_TGACATCCTCTCTAAAGATT	EDDM3A__NM_006683	215	127	303	370
EDDM3B__NM_022360_AATTCCATTGTAGCATGGAC	EDDM3B__NM_022360	195	203	147	195
EDDM3B__NM_022360_CATACCCGTCCATGCTACAA	EDDM3B__NM_022360	651	622	712	437
EDDM3B__NM_022360_CTAAAGATGGTAGAACCTAT	EDDM3B__NM_022360	543	525	533	610
EDDM3B__NM_022360_GAATTCCATTGTAGCATGGA	EDDM3B__NM_022360	203	421	308	179
EDDM3B__NM_022360_GCATCGTCTCTAAAGATCTG	EDDM3B__NM_022360	664	723	638	719
EDDM3B__NM_022360_GGACCCATACATATGCATTT	EDDM3B__NM_022360	2141	2078	2291	2257
EDDM3B__NM_022360_GTGTGCATAGGATGCAAAGT	EDDM3B__NM_022360	615	626	768	591
EDDM3B__NM_022360_TAGCATAGAAGACCTAAAGA	EDDM3B__NM_022360	61	48	43	32
EDDM3B__NM_022360_TGACACTTGAGTACTTTGAG	EDDM3B__NM_022360	218	325	324	383
EDDM3B__NM_022360_TGGCATCGTCTCTAAAGATC	EDDM3B__NM_022360	20	34	25	49
EDEM1__NM_014674_AGGGATTCCATATCCTCGGG	EDEM1__NM_014674	230	180	347	280
EDEM1__NM_014674_ATTGTGGCTCGTCTTCGGGC	EDEM1__NM_014674	501	432	593	523
EDEM1__NM_014674_CAGCGCTTTCCGCTCAGCTT	EDEM1__NM_014674	586	964	559	575
EDEM1__NM_014674_CCCCAGGTGAATCTAAAGAC	EDEM1__NM_014674	117	117	134	198
EDEM1__NM_014674_CCGTGCCAGGTCGCGCATCT	EDEM1__NM_014674	3035	3308	3615	4278
EDEM1__NM_014674_CTGAACATCAATGATGTACT	EDEM1__NM_014674	388	323	451	348
EDEM1__NM_014674_CTTTGGCTACGACAACTACA	EDEM1__NM_014674	332	448	337	378
EDEM1__NM_014674_GACGAGCGCTCGCCATTGCA	EDEM1__NM_014674	1299	1686	1335	1501
EDEM1__NM_014674_GAGTACGAGAAGCGCTACAG	EDEM1__NM_014674	295	325	306	347
EDEM1__NM_014674_GGGATTCCATATCCTCGGGT	EDEM1__NM_014674	666	661	764	506
EDEM1__NM_014674_TGAACATCAATGATGTACTA	EDEM1__NM_014674	248	234	211	161
EDEM1__NM_014674_TTCCACCGTCCAAGTCTTTG	EDEM1__NM_014674	298	405	412	384
EDEM2__NM_001145025_ACCCCCACTGGCATGCCATA	EDEM2__NM_001145025	779	665	754	670
EDEM2__NM_001145025_ACCCCTGTCACCTGTACGGC	EDEM2__NM_001145025	1673	2677	1897	2634
EDEM2__NM_001145025_AGTAAGTTCACTGTTCCATA	EDEM2__NM_001145025	151	111	159	86
EDEM2__NM_001145025_CGCCAGATCCCGCCCACTAC	EDEM2__NM_001145025	2284	2599	2523	2786
EDEM2__NM_001145025_CTACCAGCCCGATATCTGAC	EDEM2__NM_001145025	1395	1418	1532	1493
EDEM2__NM_001145025_GAGCCGGTCAGATATCGGGC	EDEM2__NM_001145025	889	553	923	856
EDEM2__NM_001145025_GGAACAGTGAACTTACTTCA	EDEM2__NM_001145025	417	494	521	464
EDEM2__NM_001145025_GGGAGAGCCGGTCAGATATC	EDEM2__NM_001145025	942	650	931	811
EDEM2__NM_001145025_GGTCCCAATCCCTGCCGTAC	EDEM2__NM_001145025	1056	750	1242	1096
EDEM2__NM_001145025_GTGTTTGAAACAAACATTCG	EDEM2__NM_001145025	172	86	170	213
EDEM2__NM_001145025_TGGGAGAGCCGGTCAGATAT	EDEM2__NM_001145025	230	142	283	158
EDEM2__NM_001145025_TGTTCCATATGGCATGCCAG	EDEM2__NM_001145025	209	155	234	175
EDEM2__NM_018217_AGAGGTCGCAGCTCATCGAA	EDEM2__NM_018217	494	249	499	259
EDEM2__NM_018217_CTGTGACGGGCACGACACCT	EDEM2__NM_018217	1249	1019	1479	1333
EDEM3__NM_025191_ACCTGTAGAGGTCGAGTTAG	EDEM3__NM_025191	239	419	217	264
EDEM3__NM_025191_ACTCGACCTCTACAGGTTAA	EDEM3__NM_025191	272	205	324	308
EDEM3__NM_025191_CTCATGCCTTTAACCTGTAG	EDEM3__NM_025191	455	629	549	620
EDEM3__NM_025191_GAAATGTTTGATCATGCTTA	EDEM3__NM_025191	143	309	184	202
EDEM3__NM_025191_GCCTCTAACTCGACCTCTAC	EDEM3__NM_025191	726	773	777	793
EDEM3__NM_025191_GGCCACCTCCGTGTGGACGG	EDEM3__NM_025191	348	343	409	380
EDEM3__NM_025191_GGCCGAGCCCATGAGTAGGG	EDEM3__NM_025191	103	162	137	89
EDEM3__NM_025191_GGGGGCCGAGCCCATGAGTA	EDEM3__NM_025191	188	288	246	207
EDEM3__NM_025191_TCATCAACGTCACCGCGACT	EDEM3__NM_025191	1093	1563	1040	1075
EDEM3__NM_025191_TCATGCTTATGGTAACTATA	EDEM3__NM_025191	124	203	123	193
EDEM3__NM_025191_TGTTTCTCCTCCCTACTCAT	EDEM3__NM_025191	21	3	27	33
EDEM3__NM_025191_TTTCTTTCAGCATGATTGCC	EDEM3__NM_025191	11	5	34	37
EDF1__NM_001281297_AAGCACCTGGTTATTGGGTA	EDF1__NM_001281297	860	1134	948	1226
EDF1__NM_001281297_ACGGGCCATACCCAATAACC	EDF1__NM_001281297	380	292	485	431
EDF1__NM_001281297_AGGTGCTTGGCAAAATCGAG	EDF1__NM_001281297	146	195	154	294
EDF1__NM_001281297_ATACCCAATAACCAGGTGCT	EDF1__NM_001281297	190	83	164	165
EDF1__NM_001281297_CCTCCAGGGTCACCCTGTCA	EDF1__NM_001281297	2430	1529	2709	2234
EDF1__NM_001281297_CCTTGCTCTGCCGACCTTGC	EDF1__NM_001281297	696	738	600	843
EDF1__NM_001281297_GAGCAAGGGGCTTACGCAGA	EDF1__NM_001281297	259	195	301	227
EDF1__NM_001281297_GCTGCGCAAGAAGGGCCCTA	EDF1__NM_001281297	245	368	284	322
EDF1__NM_001281297_GTGACGGTGCTGCGCAAGAA	EDF1__NM_001281297	201	219	226	249
EDF1__NM_001281297_TGAGAAGCCACAGGTGATCG	EDF1__NM_001281297	291	111	366	287
EDF1__NM_001281297_TTGCCAAGCACCTGGTTATT	EDF1__NM_001281297	1075	858	1034	1062
EDF1__NM_001281297_TTTGCCAAGCACCTGGTTAT	EDF1__NM_001281297	518	460	630	613
EDF1__NM_001281298_ATTGGAAAGCCCATCGAGAA	EDF1__NM_001281298	293	170	371	397
EDF1__NM_001281298_GGCAAAATCGAGCGGGCCAT	EDF1__NM_001281298	901	915	1009	1445
EDIL3__NM_001278642_AAATAAGTGAAGCATACCGA	EDIL3__NM_001278642	243	170	220	187
EDIL3__NM_001278642_AATAAGTGAAGCATACCGAG	EDIL3__NM_001278642	177	125	186	143
EDIL3__NM_001278642_AATGTCCCCGAGGATTTAAT	EDIL3__NM_001278642	471	636	545	503
EDIL3__NM_001278642_ATGGCATGGATTAGGAGTGC	EDIL3__NM_001278642	836	888	973	998
EDIL3__NM_001278642_CCCCAACTGTTCTAGTGTTG	EDIL3__NM_001278642	2236	2199	2477	2999
EDIL3__NM_001278642_CCTTACCTTTGCCGAACTGG	EDIL3__NM_001278642	987	651	1040	838
EDIL3__NM_001278642_GAAATAAGTGAAGCATACCG	EDIL3__NM_001278642	633	619	683	568
EDIL3__NM_001278642_GAAGCCATCTGGACACTCAC	EDIL3__NM_001278642	2234	2371	2519	2668
EDIL3__NM_001278642_GTATGCTTCACTTATTTCAC	EDIL3__NM_001278642	562	698	733	828
EDIL3__NM_001278642_TGAAGCGCTCGGTAGCCGTC	EDIL3__NM_001278642	565	513	769	900
EDIL3__NM_001278642_TGAATCCCATTAAATCCTCG	EDIL3__NM_001278642	457	257	472	357
EDIL3__NM_001278642_TGGCATGGATTAGGAGTGCA	EDIL3__NM_001278642	84	149	91	92
EDN1__NM_001168319_AATAGGCACGTTGTTCCGTA	EDN1__NM_001168319	483	502	544	460
EDN1__NM_001168319_AGGAACACCTAAGACAAACC	EDN1__NM_001168319	333	391	267	277
EDN1__NM_001168319_CACGTTGTTCCGTATGGACT	EDN1__NM_001168319	921	1206	1134	1205
EDN1__NM_001168319_CTAGGGCTTCCAAGTCCATA	EDN1__NM_001168319	124	166	157	181
EDN1__NM_001168319_GACTGGAATAATCATAAGAA	EDN1__NM_001168319	364	487	404	492
EDN1__NM_001168319_GTCACCAATGTGCTCGGTTG	EDN1__NM_001168319	338	522	451	515
EDN1__NM_001168319_TAACAGGGCTGAAGACATTA	EDN1__NM_001168319	356	465	418	432
EDN1__NM_001168319_TCACCAATGTGCTCGGTTGT	EDN1__NM_001168319	1071	1213	1032	1310
EDN1__NM_001168319_TGTGGGAAGTAAATTCTCCA	EDN1__NM_001168319	429	635	409	418
EDN1__NM_001955_GCTCAGCGCCTAAGACTGCT	EDN1__NM_001955	579	629	631	670

EDN2__NM_001956_ACAGACAGCTCCTTACGGCC	EDN2__NM_001956	504	531	793	943
EDN2__NM_001956_CACAGACAGACAGCTCCTTA	EDN2__NM_001956	281	326	344	255
EDN2__NM_001956_CCTTCTGCCTTCGAAGGCCC	EDN2__NM_001956	53	58	25	25
EDN2__NM_001956_GCGTCCTCATCTCATGCCCA	EDN2__NM_001956	696	622	901	672
EDN2__NM_001956_TGGCCCCTGTCTTGCCAGTC	EDN2__NM_001956	51	33	39	61
EDN3__NM_207032_AAGGTGAGAGGTGCCAACAG	EDN3__NM_207032	49	70	67	80
EDN3__NM_207033_CCCAAGACAGGTTGAAGTCA	EDN3__NM_207033	505	646	542	633
EDNRA__NM_001166055_AAGACTGGCTATCAGCGCGT	EDNRA__NM_001166055	2138	2054	2158	2238
EDNRA__NM_001166055_AATTTGGTCCTACCCAGCAA	EDNRA__NM_001166055	227	235	306	338
EDNRA__NM_001166055_CACTCATCAACCCACTAATT	EDNRA__NM_001166055	3080	3095	3338	2982
EDNRA__NM_001166055_CCTACCTTAAATACATTGAT	EDNRA__NM_001166055	1357	1963	1621	1949
EDNRA__NM_001166055_CCTATCAATGTATTTAAGGT	EDNRA__NM_001166055	2229	2383	2426	2999
EDNRA__NM_001166055_CTGGGTAGGACCAAATTAGT	EDNRA__NM_001166055	952	787	866	854
EDNRA__NM_001166055_GACTGGCTATCAGCGCGTTG	EDNRA__NM_001166055	3093	3106	3415	3925
EDNRA__NM_001166055_GATCAATGACCACATAGATA	EDNRA__NM_001166055	438	588	499	597
EDNRA__NM_001166055_GATGGAAACCCTTTGCCTCA	EDNRA__NM_001166055	3310	3529	3682	3655
EDNRA__NM_001166055_GTTGTGCATTGAGCCATTGC	EDNRA__NM_001166055	1089	1249	1273	1719
EDNRA__NM_001166055_TCTCCCTATCAATGTATTTA	EDNRA__NM_001166055	639	837	618	766
EDNRA__NM_001166055_TGGGTTGATGAGTGGTAACC	EDNRA__NM_001166055	129	242	175	203
EDNRA__NM_001256283_AATATACGGCTTAAATGAAG	EDNRA__NM_001256283	240	406	241	297
EDNRA__NM_001256283_CATACCTAAGTAATTCACAT	EDNRA__NM_001256283	2494	2739	2887	3218
EDNRA__NM_001256283_CTCACAAAATACAGAGCTAT	EDNRA__NM_001256283	1076	1418	1233	1799
EDNRA__NM_001256283_GAAAACTGTGTATAACGAGA	EDNRA__NM_001256283	245	167	245	288
EDNRA__NM_001256283_GCAGCAGCAGAGGCATGACT	EDNRA__NM_001256283	1984	2017	1953	1701
EDNRA__NM_001256283_GCTCACAAAATACAGAGCTA	EDNRA__NM_001256283	601	606	644	739
EDNRA__NM_001256283_GGATGCTTGTTCCGTTCATG	EDNRA__NM_001256283	2981	4309	3442	4196
EDNRA__NM_001256283_GGATTACATCGGTATTAACT	EDNRA__NM_001256283	686	666	826	1066
EDNRA__NM_001256283_GGGGTTTATACATGAATTCA	EDNRA__NM_001256283	196	327	222	258
EDNRA__NM_001256283_TCAACATCTCACAAGTCATG	EDNRA__NM_001256283	1316	1491	1492	2019
EDNRA__NM_001256283_TCATGGTACCCTTTGAATAT	EDNRA__NM_001256283	876	1077	1029	948
EDNRA__NM_001256283_TGGATGCTTGTTCCGTTCAT	EDNRA__NM_001256283	441	356	574	431
EDNRB__NM_000115_AAGAACAAGTGCATGCGAAA	EDNRB__NM_000115	68	125	114	152
EDNRB__NM_000115_AAGCTGGCGATCAAGATATT	EDNRB__NM_000115	804	739	817	601
EDNRB__NM_000115_ACTAAGACCTTATGGCCCAA	EDNRB__NM_000115	696	552	757	697
EDNRB__NM_000115_CATCCCTATCAATGTCTACA	EDNRB__NM_000115	2526	2824	2507	2885
EDNRB__NM_000115_CGACCGCGCCAGACTGGCGT	EDNRB__NM_000115	697	735	768	637
EDNRB__NM_000115_CGCCACCCACTAAGACCTTA	EDNRB__NM_000115	1731	2120	2005	2585
EDNRB__NM_000115_GGCAAGAACCAGCGCAACCA	EDNRB__NM_000115	1175	1376	1159	1292
EDNRB__NM_000115_GGGGGAGGGGAGATGGTGCG	EDNRB__NM_000115	1427	1814	1719	2238
EDNRB__NM_000115_TATGTGCTCTGAGTATTGAC	EDNRB__NM_000115	313	384	402	330
EDNRB__NM_000115_TGCGGTTTGCAAAAGCGGAG	EDNRB__NM_000115	194	173	279	317
EDNRB__NM_000115_TGGAGCTGAGATGTGTAAGC	EDNRB__NM_000115	131	142	134	196
EDNRB__NM_000115_TGTTCGTGCTGGGGATCATC	EDNRB__NM_000115	167	192	227	301
EDNRB__NM_001201397_ACCAGATGCAGAGCGACGAA	EDNRB__NM_001201397	120	56	111	65
EDNRB__NM_001201397_ACGGTGGAGGCTACACTGTC	EDNRB__NM_001201397	966	886	1148	1551
EDNRB__NM_001201397_CAGAGACCCCGAGCAAACGG	EDNRB__NM_001201397	820	848	878	617
EDNRB__NM_001201397_CTGCTCCAGAAGGCGTCCGG	EDNRB__NM_001201397	2777	2666	3095	2822
EDNRB__NM_001201397_TAGCCTCCACCGTTTGCTCG	EDNRB__NM_001201397	589	641	771	813
EDNRB__NM_001201397_TAGGCTCTGAAACTGCGGAG	EDNRB__NM_001201397	8	31	4	11
EDNRB__NM_001201397_TCGCAGCGTTTCGTCAGAGC	EDNRB__NM_001201397	807	810	839	1291
EDNRB__NM_001201397_TGTAGCCTCCACCGTTTGCT	EDNRB__NM_001201397	1634	1429	1709	1337
EDRF1__NM_001202438_AACTGAAGAAGGCATTTCGA	EDRF1__NM_001202438	910	668	860	474
EDRF1__NM_001202438_ATCAGACAAATGATTCGGAA	EDRF1__NM_001202438	208	249	313	349
EDRF1__NM_001202438_GAGGCTGAGCCCTCCTCGAG	EDRF1__NM_001202438	60	75	112	110
EDRF1__NM_001202438_GATCAGACAAATGATTCGGA	EDRF1__NM_001202438	322	183	376	334
EDRF1__NM_001202438_GCTCACGTGCGACTTGCTGT	EDRF1__NM_001202438	1534	1542	1716	1838
EDRF1__NM_001202438_GGCACTCTCTCGTAACCAGT	EDRF1__NM_001202438	691	324	633	352
EDRF1__NM_001202438_GTAAGAGAGTCCTTCCTATG	EDRF1__NM_001202438	466	428	659	530
EDRF1__NM_001202438_TCAGACAAATGATTCGGAAG	EDRF1__NM_001202438	299	169	306	257
EDRF1__NM_001202438_TCTTGAACCCTCATACATAG	EDRF1__NM_001202438	217	294	208	259
EDRF1__NM_001202438_TGGCAGTACACCGCATAGGA	EDRF1__NM_001202438	51	16	70	46
EDRF1__NM_001202438_TTCAGATCAGACAAATGATT	EDRF1__NM_001202438	64	95	42	75
EDRF1__NM_001202438_TTGAACCCTCATACATAGTG	EDRF1__NM_001202438	139	106	163	230
EDRF1__NM_015608_GAAGATATCCATATGTTGGT	EDRF1__NM_015608	530	233	676	306
EDRF1__NM_015608_TGTTGGAGCCGACCAACATA	EDRF1__NM_015608	170	13	119	27
EEA1__NM_003566_AATCTTGCTTTGAAGCGGTA	EEA1__NM_003566	146	54	145	109
EEA1__NM_003566_ACAAGACTATACTAAGTTAG	EEA1__NM_003566	200	283	230	214
EEA1__NM_003566_ACGGCAACATCTTCTATACC	EEA1__NM_003566	754	683	853	804
EEA1__NM_003566_AGACCTACAGGCTTCACTTA	EEA1__NM_003566	659	465	769	657
EEA1__NM_003566_CATGTAGAACTAAGTGAAAA	EEA1__NM_003566	187	134	194	265
EEA1__NM_003566_CTGCAATTTCAGTAGCAAGT	EEA1__NM_003566	818	1096	928	1320
EEA1__NM_003566_CTGTAAAATCCTCCTTAACA	EEA1__NM_003566	694	622	574	592
EEA1__NM_003566_GAATCAGTCACCAACCCATC	EEA1__NM_003566	519	489	474	350
EEA1__NM_003566_GGAAGTTGCTGTATATGTAC	EEA1__NM_003566	520	658	518	520
EEA1__NM_003566_GGTCCAAGTTCAAACACTAA	EEA1__NM_003566	141	186	265	200
EEA1__NM_003566_TTGAGGAGGCCAAACCTGAT	EEA1__NM_003566	754	964	871	808
EEA1__NM_003566_TTGGTGACTGATTCATCAGC	EEA1__NM_003566	275	191	245	239
EED__NM_003797_AGCTTCTGCTTCTTGGCCGC	EED__NM_003797	1080	815	1258	1250
EED__NM_003797_ATGGCTCGTATTGCTATCAT	EED__NM_003797	301	291	303	240
EED__NM_003797_CCGGTTGTTGCAATCTTACG	EED__NM_003797	1059	700	984	1103
EED__NM_003797_CCTACAGTTGCAAACACTAA	EED__NM_003797	454	260	636	530
EED__NM_003797_CTGGCTGTAGCTGGATCTAG	EED__NM_003797	364	256	369	473
EED__NM_003797_GAAGTGTCGACTGCGCCGGC	EED__NM_003797	764	760	861	816
EED__NM_003797_GACGCTGTCAGTATAGAAAG	EED__NM_003797	442	804	395	409
EED__NM_003797_GAGGGAAGTGTCGACTGCGC	EED__NM_003797	661	479	615	490
EED__NM_003797_GATCATAACCAACCATTGTT	EED__NM_003797	484	153	579	580
EED__NM_003797_GGCGTGTTTGTAGGTGTATC	EED__NM_003797	672	935	891	1106
EED__NM_003797_GGTGCATTTGGCGTGTTTGT	EED__NM_003797	1344	1126	1583	1611
EED__NM_003797_TGTTTGGAGTTCAGTTTAAC	EED__NM_003797	26	14	75	69
EEF1A1__NM_001402_CACCGCATTTATAGATCAGA	EEF1A1__NM_001402	345	55	388	97
EEF1A1__NM_001402_CATATCAACATTGTCGTCAT	EEF1A1__NM_001402	1148	13	1222	57
EEF1A1__NM_001402_CGTCATTGGACACGTAGATT	EEF1A1__NM_001402	458	12	555	80
EEF1A1__NM_001402_CTACAGCCAGAAGAGATATG	EEF1A1__NM_001402	84	1	38	0
EEF1A1__NM_001402_CTGAAAGCTGAGCGTGAACG	EEF1A1__NM_001402	361	33	289	141

EEF1A1__NM_001402_GATAGTCACATAGTACTTGC	EEF1A1__NM_001402	154	6	203	3
EEF1A1__NM_001402_GCACAAATGCTACTGTGTCG	EEF1A1__NM_001402	1073	17	1094	55
EEF1A1__NM_001402_GCCCTTCTGGCTTACACACT	EEF1A1__NM_001402	250	0	202	4
EEF1A1__NM_001402_GCTGTCCTGATTGTTGCTGC	EEF1A1__NM_001402	1172	1054	1209	1478
EEF1A1__NM_001402_GGCCATCTGATCTATAAATG	EEF1A1__NM_001402	490	30	601	139
EEF1A1__NM_001402_GGTGTGAAACAACTAATTGT	EEF1A1__NM_001402	116	0	94	12
EEF1A1__NM_001402_TCACCATTGATATCTCCTTG	EEF1A1__NM_001402	1315	328	1728	484
EEF1A2__NM_001958_CACCTCATCTACAAATGCGG	EEF1A2__NM_001958	1129	985	1269	1101
EEF1A2__NM_001958_CATCGTGGTCATCGGCCACG	EEF1A2__NM_001958	98	132	102	182
EEF1A2__NM_001958_CGTAAGGAGGGCAACGCAAG	EEF1A2__NM_001958	284	211	286	191
EEF1A2__NM_001958_CTCCAAGAATGGGCAGACGC	EEF1A2__NM_001958	91	58	176	168
EEF1A2__NM_001958_CTGGAAGGTGGAGCGTAAGG	EEF1A2__NM_001958	94	79	108	143
EEF1A2__NM_001958_CTTGATGAAGTCGCGGTGGC	EEF1A2__NM_001958	499	794	587	793
EEF1A2__NM_001958_GATGATGGTGATGTAGTACT	EEF1A2__NM_001958	313	235	441	238
EEF1A2__NM_001958_GGACTGCGCAGTGCTGATCG	EEF1A2__NM_001958	1207	811	1294	1215
EEF1A2__NM_001958_GGCGCAGGGGCTTGTCCGTG	EEF1A2__NM_001958	3932	4146	4110	4703
EEF1A2__NM_001958_GTCATCGGCCACGTGGACTC	EEF1A2__NM_001958	433	385	494	459
EEF1A2__NM_001958_GTCCAGATGCCGTGGTTCAA	EEF1A2__NM_001958	435	336	486	435
EEF1A2__NM_001958_TCTCCAAGAATGGGCAGACG	EEF1A2__NM_001958	151	284	218	219
EEF1B2__NM_001037663_AAGAAAGCTTTGGGCAAATA	EEF1B2__NM_001037663	172	156	403	278
EEF1B2__NM_001037663_ACGTAGGGCATGACACAAGT	EEF1B2__NM_001037663	459	316	543	433
EEF1B2__NM_001037663_ACTTGTGTCATGCCCTACGT	EEF1B2__NM_001037663	1367	340	1350	479
EEF1B2__NM_001037663_AGGGCATGACACAAGTCGGC	EEF1B2__NM_001037663	1224	882	1277	830
EEF1B2__NM_001037663_CCAGGTGCTCAACGATTACC	EEF1B2__NM_001037663	302	66	238	114
EEF1B2__NM_001037663_GACAGATATGGCGAAATTAG	EEF1B2__NM_001037663	153	15	150	59
EEF1B2__NM_001037663_GATGTGATTATACCAACGTA	EEF1B2__NM_001037663	108	73	110	25
EEF1B2__NM_001037663_GCGGACAAGAGCTACATCGA	EEF1B2__NM_001037663	644	163	651	174
EEF1B2__NM_001037663_GGTGCTCAACGATTACCTGG	EEF1B2__NM_001037663	334	81	331	74
EEF1B2__NM_001037663_GTCAGAAGCATTCAAGCAGA	EEF1B2__NM_001037663	128	45	182	91
EEF1B2__NM_001037663_TGATGTGATTATACCAACGT	EEF1B2__NM_001037663	224	200	294	128
EEF1B2__NM_001037663_TTTCAGGTCTCCGAAACCCA	EEF1B2__NM_001037663	23	6	14	17
EEF1D__NM_001130053_CAGCCCAGCCCACCGGTCAA	EEF1D__NM_001130053	908	715	1052	1168
EEF1D__NM_001130053_CCTCTCGGCTGCATCATACC	EEF1D__NM_001130053	720	582	729	488
EEF1D__NM_001130053_CCTGCCACCACGTGACCTGG	EEF1D__NM_001130053	1470	990	1774	1374
EEF1D__NM_001130053_CTCGGCCGAACGCGTGTGGC	EEF1D__NM_001130053	24	16	13	29
EEF1D__NM_001130053_CTCTCGGCTGCATCATACCG	EEF1D__NM_001130053	285	212	323	286
EEF1D__NM_001130053_CTGGCCATTGACCGGTGGGC	EEF1D__NM_001130053	493	764	674	827
EEF1D__NM_001130053_CTGGGGGATGGCCGTCAAAC	EEF1D__NM_001130053	810	417	713	832
EEF1D__NM_001130053_CTTGTCCAGCCACACGCGTT	EEF1D__NM_001130053	290	281	306	246
EEF1D__NM_001130053_GGCCCGCTCAGCCTGGTCGA	EEF1D__NM_001130053	1158	724	1327	1023
EEF1D__NM_001130053_GGGGCTGGCCATTGACCGGT	EEF1D__NM_001130053	1143	710	1234	1152
EEF1D__NM_001130053_GGGGGCTGGCCATTGACCGG	EEF1D__NM_001130053	811	665	808	804
EEF1D__NM_001130053_TTCTACGAGGCCCTGTTTGA	EEF1D__NM_001130053	0	0	3	0
EEF1D__NM_001130055_AACATCCAGAAATCCCTGGC	EEF1D__NM_001130055	1063	1037	1308	1340
EEF1D__NM_001130055_AAGCTGGTGCCCGTGGGCTA	EEF1D__NM_001130055	129	11	213	11
EEF1D__NM_001130055_CCACTTCCAGACTGGCAATC	EEF1D__NM_001130055	499	291	485	397
EEF1D__NM_001130055_CCTCACCGCCACGCAGACTC	EEF1D__NM_001130055	197	326	187	261
EEF1D__NM_001130055_CGTCGTCCGGATTGCCAGTC	EEF1D__NM_001130055	1078	949	1201	1125
EEF1D__NM_001130055_CTCTGGCTCTCGCAATGTCA	EEF1D__NM_001130055	215	174	243	160
EEF1D__NM_001130055_GATGATGACATTGACCTGTT	EEF1D__NM_001130055	652	254	742	236
EEF1D__NM_001130055_GGAGCGGCTACGGCAGTACG	EEF1D__NM_001130055	457	455	502	450
EEF1D__NM_001130055_GTGGAGAACCAGAGTCTGCG	EEF1D__NM_001130055	184	250	161	139
EEF1D__NM_001130055_TGCCCGTGGGCTACGGTATC	EEF1D__NM_001130055	133	131	161	114
EEF1D__NM_001130055_TGGCTCTCGCAATGTCACGG	EEF1D__NM_001130055	4	0	2	0
EEF1D__NM_001130055_TGGGGGCTCCACTTGGCGCA	EEF1D__NM_001130055	98	43	90	37
EEF1D__NM_001130056_GGAGGAGATCACCAAGTTTG	EEF1D__NM_001130056	421	284	576	212
EEF1D__NM_001130056_GTGGTGGAGGACGACAAGGT	EEF1D__NM_001130056	40	0	44	4
EEF1E1__NM_001135650_AAAAAGCAATCGTTCAGCAG	EEF1E1__NM_001135650	248	320	258	323
EEF1E1__NM_001135650_AAGTCCCTGGGACTGAGTAA	EEF1E1__NM_001135650	25	37	44	23
EEF1E1__NM_001135650_ACAGGGTCACTCAAGTAGAT	EEF1E1__NM_001135650	251	370	347	364
EEF1E1__NM_001135650_AGTCCCTGGGACTGAGTAAG	EEF1E1__NM_001135650	97	41	164	105
EEF1E1__NM_001135650_CCCCAGCAAATATTCTTTGT	EEF1E1__NM_001135650	1313	1328	1483	1593
EEF1E1__NM_001135650_GAAGATAAAGTCTACCTTAC	EEF1E1__NM_001135650	264	251	273	234
EEF1E1__NM_001135650_GCAGATATACTATTGTACTA	EEF1E1__NM_001135650	277	211	393	492
EEF1E1__NM_001135650_GCCAACAAAGAATATTTGCT	EEF1E1__NM_001135650	516	618	650	523
EEF1E1__NM_001135650_GGCCGCAGAGTTGTCGCTAC	EEF1E1__NM_001135650	1702	1921	2120	2102
EEF1E1__NM_001135650_TCAGCAGTGGTTAGAATACA	EEF1E1__NM_001135650	242	167	192	223
EEF1E1__NM_001135650_TTCAGCAGTGGTTAGAATAC	EEF1E1__NM_001135650	17	8	45	36
EEF1E1__NM_004280_AATATCTTAATGTGTCTCGC	EEF1E1__NM_004280	238	211	251	335
EEF1G__NM_001404_AGCAGCGATGAGAGCCTTGA	EEF1G__NM_001404	478	57	531	83
EEF1G__NM_001404_AGGAACCAGCGGTTGGTATT	EEF1G__NM_001404	575	0	566	5
EEF1G__NM_001404_CAGTTGTCTGCACCCTGTTG	EEF1G__NM_001404	1114	742	1325	731
EEF1G__NM_001404_CCTGTTGTGGCTCTATAAGC	EEF1G__NM_001404	264	65	225	5
EEF1G__NM_001404_CCTTTCCCAATACCAACCGC	EEF1G__NM_001404	393	37	496	18
EEF1G__NM_001404_CTAGGCCACTGAGAATGCAA	EEF1G__NM_001404	146	48	185	53
EEF1G__NM_001404_CTGCTTATAGAGCCACAACA	EEF1G__NM_001404	57	12	96	28
[illegible]	[illegible]	[illegible]	[illegible]	[illegible]	[illegible]

EEF2__NM_001961_GAAAGGGGAAAGGAACACGC	EEF2__NM_001961	180	165	191	154
EEF2__NM_001961_GACAGACATGTTGCGGATGT	EEF2__NM_001961	645	67	851	29
EEF2__NM_001961_GCAGACACGCCCTCTTAGTA	EEF2__NM_001961	334	261	459	409
EEF2__NM_001961_GCTGCCCTCCGAGTCACCGA	EEF2__NM_001961	4002	87	4906	210
EEF2__NM_001961_GGACTTGCCATGGTCCACGT	EEF2__NM_001961	637	0	781	28
EEF2__NM_001961_TAGGGACTTAATGGGCCGGT	EEF2__NM_001961	289	296	328	269
EEF2__NM_001961_TCCAATGGCACTAGTACAGC	EEF2__NM_001961	783	601	933	744
[illegible]	[illegible]	[illegible]	[illegible]	[illegible]	[illegible]

EFCAB13__NM_001195192_GTACTACTCTTCAAGCTTTG	EFCAB13__NM_001195192	289	551	329	351
EFCAB13__NM_001195192_TAACCACATGGTGGATATTG	EFCAB13__NM_001195192	45	16	33	33
EFCAB13__NM_001195192_TTATATGATGAAGTAACCCA	EFCAB13__NM_001195192	56	100	25	37
EFCAB13__NM_001195192_TTGTGAACGCTGTACTGATT	EFCAB13__NM_001195192	524	502	663	462
EFCAB13__NM_001195192_TTTGCAACTGACTTGTCATC	EFCAB13__NM_001195192	694	504	766	1045
EFCAB13__NM_152347_CTAAAGACTGTTGATATTGA	EFCAB13__NM_152347	381	393	430	337
[illegible]	[illegible]	[illegible]	[illegible]	[illegible]	[illegible]

EFCC1__NM_024768_ACCCACGAGATGGGGCACGG	EFCC1__NM_024768	654	557	719	740
EFCC1__NM_024768_AGAGCTGCTGTCTGACTGCC	EFCC1__NM_024768	1191	1565	1452	1798
EFCC1__NM_024768_AGCGCCCTGGCGCACCACTA	EFCC1__NM_024768	9	59	6	7
EFCC1__NM_024768_CAGGCGAGCACCCACGAGAT	EFCC1__NM_024768	1000	1052	1290	1268
EFCC1__NM_024768_CCCGCCGTGCCCCATCTCGT	EFCC1__NM_024768	3801	3707	4184	3126
EFCC1__NM_024768_CGGCGTGGAGAACGAGATCG	EFCC1__NM_024768	661	682	641	729
[illegible]	[illegible]	[illegible]	[illegible]	[illegible]	[illegible]

[illegible]

EIF2S2__NM_003908_GAGCTGCTTAGTACGAAGTC	EIF2S2__NM_003908	271	183	260	318
EIF2S2__NM_003908_GATGACCTTGACATTATGCT	EIF2S2__NM_003908	442	457	473	488
EIF2S2__NM_003908_GCACCTTGTATTCCCTCATG	EIF2S2__NM_003908	1341	1220	1471	841
EIF2S2__NM_003908_GCCTCCCACGCGAGTGTGGT	EIF2S2__NM_003908	829	1069	896	1095
EIF2S2__NM_003908_GCTGGAAGGGGATTCTCCGC	EIF2S2__NM_003908	626	385	818	689
EIF2S2__NM_003908_GGAAGGGGATACCCAAACAG	EIF2S2__NM_003908	51	18	23	7
EIF2S2__NM_003908_GGGATTCTCCGCTGGCACAT	EIF2S2__NM_003908	501	395	531	602
EIF2S2__NM_003908_GTTCCTACTCGGACGACTTG	EIF2S2__NM_003908	135	14	130	31
EIF2S2__NM_003908_TATTGCCAAGCATAATGTCA	EIF2S2__NM_003908	483	487	516	488
EIF2S2__NM_003908_TCCAACATTGGGAATCGTGT	EIF2S2__NM_003908	664	534	771	511
EIF2S2__NM_003908_TCCCACCACACTCGCGTGGG	EIF2S2__NM_003908	482	478	394	451
EIF2S2__NM_003908_TGAAGCTGAAGAAGGTGTAA	EIF2S2__NM_003908	119	138	116	70
EIF2S2__NM_003908_TGAGCTGCTTAGTACGAAGT	EIF2S2__NM_003908	106	83	158	117
EIF2S2__NM_003908_TTCTCCCCAGCAACCATATC	EIF2S2__NM_003908	0	0	0	0
EIF2S3__NM_001415_AAAATATGGGGCCACATAAA	EIF2S3__NM_001415	272	168	260	312
EIF2S3__NM_001415_AAATTTGTTTCTCAACACTG	EIF2S3__NM_001415	38	27	19	49
EIF2S3__NM_001415_AACCTTACCGTTACCTACTA	EIF2S3__NM_001415	370	325	420	519
EIF2S3__NM_001415_AAGGGAACTTCAAATTAGTC	EIF2S3__NM_001415	131	30	126	69
EIF2S3__NM_001415_AATTCCTCTTAACAACCAAG	EIF2S3__NM_001415	23	54	75	74
EIF2S3__NM_001415_AGAAATAGCTTTGACGACTG	EIF2S3__NM_001415	98	77	139	0
EIF2S3__NM_001415_AGACAAGCCACAATTAACAT	EIF2S3__NM_001415	224	0	297	10
EIF2S3__NM_001415_AGATCCTGACGCGAAAGATG	EIF2S3__NM_001415	274	202	333	170
EIF2S3__NM_001415_AGGAAAAGCACACGAAGTAT	EIF2S3__NM_001415	662	653	640	504
EIF2S3__NM_001415_AGTTTCCTACGGACATTCCA	EIF2S3__NM_001415	74	47	69	38
EIF2S3__NM_001415_ATAATACATTCGGATGGATT	EIF2S3__NM_001415	172	180	170	176
EIF2S3__NM_001415_ATTGGCTTGGAGTAGAATTC	EIF2S3__NM_001415	68	76	129	70
EIF2S3__NM_001415_CACACGAAGTATAGGGACAT	EIF2S3__NM_001415	2442	2751	2686	2658
EIF2S3__NM_001415_CAGAATGTTATAGATCTTGT	EIF2S3__NM_001415	204	0	245	46
EIF2S3__NM_001415_CCACAAGATCTATAACATTC	EIF2S3__NM_001415	192	23	284	99
EIF2S3__NM_001415_CCAGAATGTTATAGATCTTG	EIF2S3__NM_001415	231	5	363	1
EIF2S3__NM_001415_GAAGGTTGTGTTGTAGCATG	EIF2S3__NM_001415	87	156	144	112
EIF2S3__NM_001415_GAGAAGCTGGAGTGACTCTA	EIF2S3__NM_001415	86	1	108	63
EIF2S3__NM_001415_GAGAATCACTTGAATCAGGG	EIF2S3__NM_001415	44	0	21	0
EIF2S3__NM_001415_GCACACGAAGTATAGGGACA	EIF2S3__NM_001415	680	617	692	830
EIF2S3__NM_001415_GCAGCCGCATCTTTCGCGTC	EIF2S3__NM_001415	2388	3071	2525	3512
EIF2S3__NM_001415_GCTTGGATATGCTAATGCTA	EIF2S3__NM_001415	248	14	313	25
EIF2S3__NM_001415_GGAAAAGCACACGAAGTATA	EIF2S3__NM_001415	645	692	616	736
EIF2S3__NM_001415_TACATGACCAATTGTACCTA	EIF2S3__NM_001415	58	0	74	19
EIF2S3__NM_001415_TACCTTAGTAGGTAACGGTA	EIF2S3__NM_001415	130	70	101	48
EIF2S3__NM_001415_TCACAGTTCGTTACCTTAGT	EIF2S3__NM_001415	635	507	743	569
EIF2S3__NM_001415_TTGTAGAAGTTTAGAAGCTC	EIF2S3__NM_001415	17	0	6	26
EIF3A__NM_003750_AATCTCTGGAGGATGTTGTT	EIF3A__NM_003750	386	1	459	28
EIF3A__NM_003750_AATGCCCTCAAACGCGCCAA	EIF3A__NM_003750	272	0	370	36
EIF3A__NM_003750_ACTCACCGTTGGCGCGTTTG	EIF3A__NM_003750	1647	148	1847	314
EIF3A__NM_003750_AGGTACGTATCAACCACTTA	EIF3A__NM_003750	647	360	636	345
EIF3A__NM_003750_AGGTGTTACTCAATACCTGA	EIF3A__NM_003750	130	56	171	85
EIF3A__NM_003750_ATGGAAAAGCAGTTTGCTAT	EIF3A__NM_003750	418	536	493	592
EIF3A__NM_003750_ATTACAAACATTGGATACGG	EIF3A__NM_003750	394	187	445	265
EIF3A__NM_003750_ATTCAGCCTTACGCGTGTAT	EIF3A__NM_003750	347	26	380	19
EIF3A__NM_003750_CAGCCTTACGCGTGTATTGG	EIF3A__NM_003750	481	6	561	22
EIF3A__NM_003750_CTGCCTCCAATACACGCGTA	EIF3A__NM_003750	394	32	487	35
EIF3A__NM_003750_CTTCACCACTTACAGCACTT	EIF3A__NM_003750	254	37	324	50
EIF3A__NM_003750_GAAATACTTGGAACTTTGCG	EIF3A__NM_003750	259	4	314	10
EIF3A__NM_003750_GCTGGGCAATATCATGGTAC	EIF3A__NM_003750	343	11	361	11
EIF3A__NM_003750_GGTGTTACTCAATACCTGAA	EIF3A__NM_003750	341	567	392	409
EIF3A__NM_003750_GTTGACAAATGTTCTTATAC	EIF3A__NM_003750	244	13	251	31
EIF3A__NM_003750_TACAAACATTGGATACGGTG	EIF3A__NM_003750	131	151	188	162
EIF3A__NM_003750_TCCATGGGTTAAATTCCTGT	EIF3A__NM_003750	514	38	509	35
EIF3A__NM_003750_TCCCACAGGAATTTAACCCA	EIF3A__NM_003750	155	0	146	14
EIF3A__NM_003750_TTACAAACATTGGATACGGT	EIF3A__NM_003750	148	81	185	301
EIF3B__NM_001037283_AAAGAGGCATTGCTCTATGG	EIF3B__NM_001037283	13	0	21	0
EIF3B__NM_001037283_ACGTTCTCCGCGTCCTGCAT	EIF3B__NM_001037283	185	0	172	2
EIF3B__NM_001037283_ACTTGTCAAAATCCGTAAAG	EIF3B__NM_001037283	302	18	356	78
EIF3B__NM_001037283_AGGGGGCTCAGCATGCGACC	EIF3B__NM_001037283	11	0	24	0
EIF3B__NM_001037283_CTCTTTCTTCAATTGAGACA	EIF3B__NM_001037283	21	8	81	48
EIF3B__NM_001037283_GCTCTTTCTTCAATTGAGAC	EIF3B__NM_001037283	170	228	192	237
EIF3B__NM_001037283_GGAAGCAGATGGAATCGATT	EIF3B__NM_001037283	700	48	745	50
EIF3B__NM_001037283_GGACAGAGACGTATGTGCGT	EIF3B__NM_001037283	945	8	854	47
EIF3B__NM_001037283_GTCACTGATCGTCATATACC	EIF3B__NM_001037283	314	14	308	17
EIF3B__NM_001037283_TGGGAGATGTACTCAAAGAT	EIF3B__NM_001037283	60	6	81	17
EIF3B__NM_001037283_TTGACTTCTCACCTTGTGAA	EIF3B__NM_001037283	146	15	109	29
EIF3B__NM_001037283_TTTGAGTTTCTCAAGTCGGT	EIF3B__NM_001037283	145	223	199	290
EIF3B__NM_003751_AATGCTGCTCGGCTGTGTAC	EIF3B__NM_003751	572	636	568	446
EIF3B__NM_003751_CCGAGCAGCATTTCCGTTGA	EIF3B__NM_003751	1840	2334	1965	2520
EIF3B__NM_003751_CGAAGACTTAGCGACGCCAC	EIF3B__NM_003751	452	355	458	364
[illegible]	[illegible]	[illegible]	[illegible]	[illegible]	[illegible]

EN1__NM_001426_GGTGGGCCACAGTTCGCGTC	EN1__NM_001426	564	615	525	551
EN1__NM_001426_GTTGCCGTGCTCCGGGTATT	EN1__NM_001426	1656	1731	1767	2116
EN1__NM_001426_TGCGCCCCGGACGCGAACTG	EN1__NM_001426	514	775	567	620
EN1__NM_001426_TGGGCCACAGTTCGCGTCCG	EN1__NM_001426	481	722	530	597
EN1__NM_001426_TGTCCACCCGTTGGGCACCC	EN1__NM_001426	74	67	41	63
EN2__NM_001427_ACCGTGCAGCGAGAGCGTCT	EN2__NM_001427	284	286	307	349
EN2__NM_001427_ACTGTACGCGCTACTCGGAC	EN2__NM_001427	889	1270	1094	1174
EN2__NM_001427_AGAAGTTGGTGATGCGGTGC	EN2__NM_001427	304	315	327	390
EN2__NM_001427_GGCCTTGAGCGACCCGTCCA	EN2__NM_001427	862	1186	1070	1303
EN2__NM_001427_GGTCTACTGTACGCGCTACT	EN2__NM_001427	835	1070	1008	1394
EN2__NM_001427_TCGCAAGCCGGCGCCAACCT	EN2__NM_001427	831	811	899	745
EN2__NM_001427_TCGGCGGTAAAGGCCGTGCG	EN2__NM_001427	603	638	757	898
EN2__NM_001427_TTCGGCCGGCGAAAGGACGC	EN2__NM_001427	48	43	47	25
ENAH__NM_001008493_AAGGAAGGGGTCCATTTCCA	ENAH__NM_001008493	402	305	454	274
ENAH__NM_001008493_CAATAGCATTCCCTCCACTT	ENAH__NM_001008493	1813	1420	1853	1452
ENAH__NM_001008493_CCAAGTGGAGGGAATGCTAT	ENAH__NM_001008493	664	707	679	668
ENAH__NM_001008493_CCAGTGGCGAGATGCTAGAC	ENAH__NM_001008493	403	267	397	319
ENAH__NM_001008493_CTTAGAAGTGTTAAATTCAC	ENAH__NM_001008493	97	110	175	218
ENAH__NM_001008493_GAAGACAATCGCCCTTTAAC	ENAH__NM_001008493	323	156	289	225
ENAH__NM_001008493_GAGCGCAGAATATCAAGTGC	ENAH__NM_001008493	576	443	702	593
ENAH__NM_001008493_GGACTTGCAGCTGCAATTGC	ENAH__NM_001008493	205	160	232	175
ENAH__NM_001008493_TCTCCCAGCACAGAGTTTAG	ENAH__NM_001008493	189	195	238	257
ENAH__NM_001008493_TGAATGTGTTGTTGCCTGTA	ENAH__NM_001008493	502	400	511	645
ENAH__NM_001008493_TGTTGGGATGGAGTCTCGGC	ENAH__NM_001008493	345	187	356	303
ENAH__NM_001008493_TTTCCAATTCTTCTTGGGAT	ENAH__NM_001008493	268	380	248	352
ENAM__NM_031889_AGGTTACCACCACCAGGTTA	ENAM__NM_031889	38	18	35	39
ENAM__NM_031889_ATCAGTCTTGTTATGACGTT	ENAM__NM_031889	878	977	845	1382
ENAM__NM_031889_ATTGGTGGGCGTCCATAACC	ENAM__NM_031889	187	252	258	287
ENAM__NM_031889_CAAAAAAGCCACCACAAAAG	ENAM__NM_031889	172	352	175	194
ENAM__NM_031889_CACATGGGCATCTGGTACTG	ENAM__NM_031889	891	1076	1055	1298
ENAM__NM_031889_CGTGGATGCCATGTGTTGGG	ENAM__NM_031889	3317	3754	3868	4345
ENAM__NM_031889_CTCACCAAGTTATCTAGTTT	ENAM__NM_031889	698	1045	742	1097
ENAM__NM_031889_CTCTAGGCATTCCCACCATT	ENAM__NM_031889	589	765	757	822
ENAM__NM_031889_GCATTCGGGGCATGTGCATC	ENAM__NM_031889	655	654	696	962
ENAM__NM_031889_TGCCATGGTGGTTGTTGATA	ENAM__NM_031889	125	174	83	82
ENAM__NM_031889_TTCCGTGGATGCCATGTGTT	ENAM__NM_031889	236	154	179	193
ENAM__NM_031889_TTTCCGTGGATGCCATGTGT	ENAM__NM_031889	791	917	820	1001
ENC1__NM_001256574_ACTGTTGCAGACAGTAAGGC	ENC1__NM_001256574	148	237	207	221
ENC1__NM_001256574_CCAAGATAGTTCGTACAGCT	ENC1__NM_001256574	255	399	270	368
ENC1__NM_001256574_CCAAGCTGTACGAACTATCT	ENC1__NM_001256574	164	241	166	177
ENC1__NM_001256574_CCAGCCTCTGTGCCCGACCT	ENC1__NM_001256574	1484	1985	1717	1784
ENC1__NM_001256574_CTGCAAAGTGTACATTACTG	ENC1__NM_001256574	934	762	888	817
ENC1__NM_001256574_GAGGACGCTGTCAGCGTAGG	ENC1__NM_001256574	43	112	36	63
ENC1__NM_001256574_GCATGAGAACCGCAAGTCCA	ENC1__NM_001256574	543	644	721	640
ENC1__NM_001256574_GCTGCAAAGTGTACATTACT	ENC1__NM_001256574	1125	1263	1216	1228
ENC1__NM_001256574_GGAAGGTCCTATTTCCGGCA	ENC1__NM_001256574	490	588	554	484
ENC1__NM_001256574_TCCTTCTCCATGCCGGAAAT	ENC1__NM_001256574	280	258	292	350
ENC1__NM_001256574_TGCATGCAGTCGCTACTTTG	ENC1__NM_001256574	740	816	830	685
ENC1__NM_001256574_TGTACGAGTCTGCAATTAAC	ENC1__NM_001256574	136	60	178	137
ENC1__NM_001256576_AAGTGTACATTACTGGGGGG	ENC1__NM_001256576	305	365	458	545
ENC1__NM_001256576_AAGTTTGGCACTCACTACTG	ENC1__NM_001256576	446	914	433	305
ENC1__NM_001256576_CCCCTCAGTCTCTCTAAAGC	ENC1__NM_001256576	911	936	929	756
ENC1__NM_001256576_CCTTGGACCACTCCTCGTGC	ENC1__NM_001256576	1261	1602	1663	1408
ENC1__NM_001256576_GAGCTTGTCATGACTGACAC	ENC1__NM_001256576	456	515	618	663
ENDOD1__NM_015036_ACGCGGAGTACACGGGGATG	ENDOD1__NM_015036	578	509	758	731
ENDOD1__NM_015036_AGTCCCTGAGTTTGTTTGGC	ENDOD1__NM_015036	1105	1515	1344	1805
ENDOD1__NM_015036_CCTGGAAGGACTGAGTCATT	ENDOD1__NM_015036	1471	1265	1423	966
ENDOD1__NM_015036_CCTTGAGGAGGCGATTAATG	ENDOD1__NM_015036	289	240	271	256
ENDOD1__NM_015036_CTGCCAGCCAAACAAACTCA	ENDOD1__NM_015036	91	143	131	103
ENDOD1__NM_015036_CTGTGCCTGTGAGGATATAT	ENDOD1__NM_015036	261	189	307	261
ENDOD1__NM_015036_CTTGATTCTGATTACCAAAG	ENDOD1__NM_015036	87	125	89	48
ENDOD1__NM_015036_GAAGACCTATATATCCTCAC	ENDOD1__NM_015036	448	224	593	446
ENDOD1__NM_015036_GCGCTGACAGATCTTCACGT	ENDOD1__NM_015036	1812	1822	2298	1847
ENDOD1__NM_015036_GGGTCAAAGCCCGGTCCATT	ENDOD1__NM_015036	1189	1397	1369	1485
ENDOD1__NM_015036_GTGACAAGTTCTTCTACGCC	ENDOD1__NM_015036	310	489	349	776
ENDOD1__NM_015036_TCCTGGAAGGACTGAGTCAT	ENDOD1__NM_015036	261	192	208	239
ENDOG__NM_004435_AGCACAGCACGTACGACTCG	ENDOG__NM_004435	1026	848	1226	1137
ENDOG__NM_004435_CAAAACGTCTATGTCTGCAC	ENDOG__NM_004435	685	832	773	1052
ENDOG__NM_004435_CCTGGGGCGCGACGTTGCTC	ENDOG__NM_004435	149	78	118	109
ENDOG__NM_004435_CGCGGCAGTGGCTTCGACCG	ENDOG__NM_004435	2507	2116	2647	2655
ENDOG__NM_004435_CGTGATGCCCAACGCACCTG	ENDOG__NM_004435	1262	1523	1239	1184
ENDOG__NM_004435_GAAATCCTACGTAAAGTACC	ENDOG__NM_004435	148	169	233	234
ENDOG__NM_004435_GCACCAGGAAGCGCTCCAGT	ENDOG__NM_004435	1913	651	1998	1005
ENDOG__NM_004435_GCCACCAACGCCGACTACCG	ENDOG__NM_004435	1442	1097	1513	1448
ENDOG__NM_004435_GCCTCATCCACAGGTGCGTT	ENDOG__NM_004435	971	774	1031	1072
ENDOG__NM_004435_GGCCTCATCCACAGGTGCGT	ENDOG__NM_004435	807	835	739	935
ENDOG__NM_004435_GGGCTGGGTGCGGTCGTCGA	ENDOG__NM_004435	926	900	1064	1150
ENDOG__NM_004435_TACGTAAAGTACCAGGTCAT	ENDOG__NM_004435	313	181	384	429
ENDOG__NM_004435_TCACGGCGGGCAGTAAGTGA	ENDOG__NM_004435	571	333	669	587
ENDOU__NM_001172439_ACATTGGTGGTGCTTGTCAA	ENDOU__NM_001172439	158	196	165	101
ENDOU__NM_001172439_ACTAACCGTGGTCACTACAC	ENDOU__NM_001172439	228	141	228	112
ENDOU__NM_001172439_AGAGACACCGGCGGTCACAC	ENDOU__NM_001172439	454	603	463	317
ENDOU__NM_001172439_CACACAGCACGGCCAATACC	ENDOU__NM_001172439	422	330	361	331
ENDOU__NM_001172439_CAGTGATGCCATAACAAAAG	ENDOU__NM_001172439	85	71	36	42
ENDOU__NM_001172439_CCCCATGGCCTGTTGCCCGC	ENDOU__NM_001172439	1047	1443	1158	1518
ENDOU__NM_001172439_CTCATTACATCGAGATGCAC	ENDOU__NM_001172439	414	598	500	456
ENDOU__NM_001172439_CTGCCTGCCAGTGTGACCGC	ENDOU__NM_001172439	1925	2726	2075	2228
ENDOU__NM_001172439_GAAGGCTGCATAGGTGGGCT	ENDOU__NM_001172439	357	462	414	420
ENDOU__NM_001172439_GTTGATGAAGGCTGCATAGG	ENDOU__NM_001172439	107	44	94	84
ENDOU__NM_001172439_TGGTTTGGGCTCTATTCAAG	ENDOU__NM_001172439	5	1	8	10
ENDOU__NM_001172439_TGTCAAAGGCTTCGTAGCAG	ENDOU__NM_001172439	228	189	191	190
ENDOU__NM_001172440_AATGAAGAGGGGGACTCGAG	ENDOU__NM_001172440	257	245	275	187
ENDOU__NM_001172440_GTAAAAAAAGGCAAGGTTAC	ENDOU__NM_001172440	113	66	131	64
ENDOU__NM_001172440_TATTACAGTCACATCTACGA	ENDOU__NM_001172440	764	612	764	394
ENDOV__NM_001164637_AGGCCAGTGGCATGCCCCAA	ENDOV__NM_001164637	303	143	311	161
ENDOV__NM_001164637_CCACCAGGGAAGCACAAGCG	ENDOV__NM_001164637	372	248	255	188
ENDOV__NM_001164637_CCAGGAGCCCGAAGGCGCAG	ENDOV__NM_001164637	81	82	76	63
ENDOV__NM_001164637_CGGGAGCAGATGTCAGCCTG	ENDOV__NM_001164637	834	417	776	515
ENDOV__NM_001164637_GCAGGTCTGTAAGGACGCCA	ENDOV__NM_001164637	192	233	190	142
ENDOV__NM_001164637_GGTGTCCCGGTCTACGACGT	ENDOV__NM_001164637	706	588	612	384
ENDOV__NM_001164637_GTTGACGTGTCCTTCGTGAA	ENDOV__NM_001164637	837	707	882	886
ENDOV__NM_001164637_TAGACCGGGACACCGAGGCG	ENDOV__NM_001164637	491	390	382	324
ENDOV__NM_001164637_TGGCCACCCCAACACACGGC	ENDOV__NM_001164637	74	124	92	51
ENDOV__NM_001164637_TGGCCCACGGAGATGTAGAG	ENDOV__NM_001164637	38	36	88	59
ENDOV__NM_001164637_TGTGGCCCACGGAGATGTAG	ENDOV__NM_001164637	180	139	201	227
ENDOV__NM_001164638_GCGGGCAAGGACTGGCAGTA	ENDOV__NM_001164638	956	656	946	919
ENDOV__NM_173627_CAGGAAGCCCGACACGTAGG	ENDOV__NM_173627	467	268	492	297
ENDOV__NM_173627_CCAGGAAGCCCGACACGTAG	ENDOV__NM_173627	492	270	507	355
ENDOV__NM_173627_GCCAGGAAGCCCGACACGTA	ENDOV__NM_173627	110	31	135	69
ENG__NM_000118_AACTTGCCTAACTAACTGGC	ENG__NM_000118	351	641	371	475
ENG__NM_000118_AATGCTGGCATTGAGCATCC	ENG__NM_000118	236	128	298	207
ENG__NM_000118_ACAAGGAGGCGCACATCCTG	ENG__NM_000118	444	153	431	408
ENG__NM_000118_ACATGGGCCGCACGCTCGAG	ENG__NM_000118	802	1225	1040	873
ENG__NM_000118_AGGCTCCATTCTGGGTCGAG	ENG__NM_000118	288	272	310	321
ENG__NM_000118_AGGGCCCCTCAATCCCTCAG	ENG__NM_000118	478	446	535	555
ENG__NM_000118_AGGGTCCCCCCTACGTGTCC	ENG__NM_000118	239	310	238	180
ENG__NM_000118_ATCCTTCGTGGAGCTACCGC	ENG__NM_000118	483	337	547	579
ENG__NM_000118_CAAGACCCAGATCCTTGAGT	ENG__NM_000118	1840	1743	2146	1662
ENG__NM_000118_CACAGAACCTTCAACCCAGT	ENG__NM_000118	1189	852	1325	803
ENG__NM_000118_CCTGAACTTGCCTAACTAAC	ENG__NM_000118	621	892	644	700
ENG__NM_000118_CTCTGTGGTGTTGACCCCCG	ENG__NM_000118	151	41	162	144
ENG__NM_000118_GAAGCCTCTGAGGGATTGAG	ENG__NM_000118	250	170	254	275
ENG__NM_000118_GCCACTGGCTTGGAACACCT	ENG__NM_000118	1099	1297	1207	1050
ENG__NM_000118_GCCCTCTTCTCTTCCCAGCG	ENG__NM_000118	712	1065	697	935
ENG__NM_000118_GCCCTTCGAGACCTGGCTAG	ENG__NM_000118	626	411	747	677
ENG__NM_000118_GGAGGCGGGAAGGGTAGGCG	ENG__NM_000118	336	413	482	392
ENG__NM_000118_GGCCAGCGGTAGCTCCACGA	ENG__NM_000118	262	228	273	229
ENG__NM_000118_GGTGACATATACCACTAGCC	ENG__NM_000118	314	383	485	629
ENG__NM_000118_GTCTCCCCTGCCAGTTAGTT	ENG__NM_000118	620	529	656	914
ENG__NM_000118_GTGGTTGGCGTCGATGAGCC	ENG__NM_000118	14	10	24	55
ENG__NM_000118_TGGCAAGAACAGTGGGCGTT	ENG__NM_000118	53	45	101	84
ENG__NM_000118_TGGGCCGCACGCTCGAGTGG	ENG__NM_000118	92	83	152	148
ENG__NM_001278138_CAAGCTCTTTCTTTAGTACC	ENG__NM_001278138	255	320	273	396
ENG__NM_001278138_CGTCGTCGGCACACTTTGTC	ENG__NM_001278138	1903	2055	2166	2535
ENG__NM_001278138_TCCCAAGGACACTTGTAGCC	ENG__NM_001278138	252	319	301	286
ENG__NM_001278138_TTTCTTTAGTACCAGGGTCA	ENG__NM_001278138	9	23	9	2
ENGASE__NM_001042573_ACCAGTTAGATATTATGACA	ENGASE__NM_001042573	513	356	473	433
ENGASE__NM_001042573_AGAAGAGACAGTCTTTCGAG	ENGASE__NM_001042573	492	486	516	390
ENGASE__NM_001042573_AGGGGGCTGGCGACACGCCA	ENGASE__NM_001042573	8	23	16	60
ENGASE__NM_001042573_CAAGTAAAAGCTGATTGGTT	ENGASE__NM_001042573	1247	1078	1198	1387
ENGASE__NM_001042573_CAGGCATGGGGTCTGCGTGC	ENGASE__NM_001042573	1255	1836	1268	1817
ENGASE__NM_001042573_CGCTGGAGGAGCTCTTGGCG	ENGASE__NM_001042573	678	715	745	819
ENGASE__NM_001042573_CTGCCAGTGGTAGAAAGCAT	ENGASE__NM_001042573	325	468	391	384
ENGASE__NM_001042573_GGATGGCTTTAATGTGGCCC	ENGASE__NM_001042573	612	864	690	642
ENGASE__NM_001042573_TCATGACACAACAAAGTCCG	ENGASE__NM_001042573	278	218	416	423
ENGASE__NM_001042573_TGCCAGTGGTAGAAAGCATA	ENGASE__NM_001042573	19	4	22	14
ENGASE__NM_001042573_TGTCATGACACAACAAAGTC	ENGASE__NM_001042573	1	0	0	0
ENGASE__NM_001042573_TTAAAGCCATCCTCCAAGCG	ENGASE__NM_001042573	191	175	240	265
ENHO__NM_198573_ATCGTCTGCAACGGTCTCGT	ENHO__NM_198573	2456	2434	2835	2804
ENHO__NM_198573_CATCGTCTGCAACGGTCTCG	ENHO__NM_198573	633	893	881	927
ENHO__NM_198573_GAGATGGCTGCCCCCATGAC	ENHO__NM_198573	854	848	888	651
ENHO__NM_198573_TTCAGAGAGAGAGTCAACGT	ENHO__NM_198573	28	55	58	98
ENKD1__NM_032140_AGCGTCGGGTCTGGGGGGAT	ENKD1__NM_032140	1007	1009	1241	1482
ENKD1__NM_032140_AGGACAGAGCGTCGGGTCTG	ENKD1__NM_032140	578	762	702	708
ENKD1__NM_032140_CCGGCACCGGGACCGATGCA	ENKD1__NM_032140	1507	1595	1673	1948
ENKD1__NM_032140_CGCTCTGTCCTGACAACTAC	ENKD1__NM_032140	388	310	368	456
ENKD1__NM_032140_CGTGGCCCCTGCATCGGTCC	ENKD1__NM_032140	914	1044	940	1021
ENKD1__NM_032140_GACGTGCTGTTGCAACTCGA	ENKD1__NM_032140	1727	1670	1904	1745
ENKD1__NM_032140_GCTTCAGCGCGTTTCCCTCG	ENKD1__NM_032140	1318	1475	1737	1791
ENKD1__NM_032140_GGACGTGCTGTTGCAACTCG	ENKD1__NM_032140	990	1001	1026	1186
ENKD1__NM_032140_GGAGAACCTGAGGCGGATCA	ENKD1__NM_032140	693	979	868	800
ENKD1__NM_032140_GGGGATCTCCCTAGGTCCTG	ENKD1__NM_032140	296	260	334	334
ENKD1__NM_032140_TCAGGACAGAGCGTCGGGTC	ENKD1__NM_032140	512	509	491	569
ENKD1__NM_032140_TGGGCCCCGAGATGCGGGAC	ENKD1__NM_032140	59	34	75	74
ENKUR__NM_001270383_ATACATATGTAAGCGAAACG	ENKUR__NM_001270383	260	332	375	522
ENKUR__NM_001270383_CAAAGAAGATCCGCAAGCAG	ENKUR__NM_001270383	363	686	483	596
ENKUR__NM_145010_AAAAAACTCTACCACCCAGT	ENKUR__NM_145010	390	346	462	355
ENKUR__NM_145010_AGAAGAGCACGTTGGATCCA	ENKUR__NM_145010	407	417	498	351
ENO1__NM_001201483_AACCTCTGCTCCAATGCGCA	ENO1__NM_001201483	612	171	573	276
ENO1__NM_001201483_AGCTGCTGAAGACTGCTATT	ENO1__NM_001201483	818	20	893	35
ENO1__NM_001201483_AGCTGGTGCCGTTGAGAAGG	ENO1__NM_001201483	228	1	181	21
ENO1__NM_001201483_ATTGATGACATTGAACGCCT	ENO1__NM_001201483	221	0	282	14
ENO1__NM_001201483_CAAAGCTGGTGCCGTTGAGA	ENO1__NM_001201483	434	511	516	492
ENO1__NM_001201483_CATTGATGACATTGAACGCC	ENO1__NM_001201483	583	658	540	565
ENO1__NM_001201483_CGGGTTTGCTCCCAACATCC	ENO1__NM_001201483	142	67	216	126
ENO1__NM_001201483_GAGCTGCTGAAGACTGCTAT	ENO1__NM_001201483	511	289	604	373
ENO1__NM_001201483_GGTCATCGGCATGGACGTAG	ENO1__NM_001201483	360	75	407	75
ENO1__NM_001201483_GTACCGCCACATCGCTGACT	ENO1__NM_001201483	1598	79	1740	244
ENO1__NM_001201483_GTTTGGTGCGAACGCCATTC	ENO1__NM_001201483	103	131	113	122
ENO1__NM_001201483_TTGATGACATTGAACGCCTG	ENO1__NM_001201483	304	16	399	2
ENO1__NM_001428_AAGACTCGCTATATGGGGAA	ENO1__NM_001428	327	235	379	264
ENO1__NM_001428_AAGAGATCAACCTCAACAGT	ENO1__NM_001428	322	0	291	11
ENO1__NM_001428_AATGATAAGACTCGCTATAT	ENO1__NM_001428	600	145	633	81
ENO1__NM_001428_AGCGAGTCTTATCATTGTCC	ENO1__NM_001428	984	504	1171	393
ENO1__NM_001428_ATCATTGTCCCGGAGCTCTA	ENO1__NM_001428	193	0	190	4
ENO1__NM_001428_ATGATAAGACTCGCTATATG	ENO1__NM_001428	438	51	506	39
ENO1__NM_001428_CAATGATAAGACTCGCTATA	ENO1__NM_001428	440	292	486	238
ENO1__NM_001428_CCTGCCCTGGTTAGCAAGGT	ENO1__NM_001428	371	398	433	597
ENO1__NM_001428_GGGAGATCTTTGACTCTCGC	ENO1__NM_001428	376	686	434	335
ENO1__NM_001428_TAAGACTCGCTATATGGGGA	ENO1__NM_001428	361	33	343	87
ENO1__NM_001428_TGCGCCTGCCCTGGTTAGCA	ENO1__NM_001428	915	368	1189	180
ENO1__NM_001428_TGCTTCAACTGGTATCTATG	ENO1__NM_001428	85	1	138	4
ENO2__NM_001975_CTGAGCAATGTGGCGATACA	ENO2__NM_001975	507	258	414	512
ENO2__NM_001975_GACCTCTGCACCTAGTCGCA	ENO2__NM_001975	610	522	811	772
ENO2__NM_001975_GAGGTCTACCATACACTCAA	ENO2__NM_001975	312	248	322	250
ENO2__NM_001975_GCCACCATTGATCACGTTGA	ENO2__NM_001975	1733	2286	1986	1979
ENO2__NM_001975_GCCTTCAACGTGATCAATGG	ENO2__NM_001975	846	634	902	918
ENO2__NM_001975_GGAGACAAACAGCGTTACTT	ENO2__NM_001975	616	609	618	661
ENO2__NM_001975_GGCCTCATAGATGCCCGTAG	ENO2__NM_001975	112	56	111	141
ENO2__NM_001975_GTGCCCAGTGGAGCCTCTAC	ENO2__NM_001975	704	717	749	728
ENO2__NM_001975_GTGGATCTCTATACTGCCAA	ENO2__NM_001975	11	6	12	7
ENO2__NM_001975_TGAGCAATGTGGCGATACAG	ENO2__NM_001975	148	144	158	166
ENO2__NM_001975_TGCCATGCGACTAGGTGCAG	ENO2__NM_001975	383	581	308	253
ENO2__NM_001975_TTTCGGGATGCCATGCGACT	ENO2__NM_001975	160	103	211	213
ENO3__NM_001193503_AATCTTTGCCCGGGAAATCT	ENO3__NM_001193503	205	281	213	212
ENO3__NM_001193503_ACAGCTGCTCGGAATCGGCC	ENO3__NM_001193503	644	973	928	1116
ENO3__NM_001193503_ATGTGCCGTGCGGGATCATC	ENO3__NM_001193503	1006	1416	1276	1233
ENO3__NM_001193503_CAGAGCCTCATAGATACCCG	ENO3__NM_001193503	548	415	496	326
ENO3__NM_001193503_CATCTGAGTTCTATCGCAAT	ENO3__NM_001193503	779	791	1140	937
ENO3__NM_001193503_CTCCAGGGGCAACCCCACGG	ENO3__NM_001193503	140	265	185	155
ENO3__NM_001193503_GAGGCTCTGGAACTAAGAGA	ENO3__NM_001193503	25	16	47	35
ENO3__NM_001193503_GCATCTGAGTTCTATCGCAA	ENO3__NM_001193503	495	429	555	793
ENO3__NM_001193503_GGCCTTCAATGTGATCAACG	ENO3__NM_001193503	462	436	615	485
ENO3__NM_001193503_GGCGGCTGGTTACCCAGACA	ENO3__NM_001193503	371	440	465	464
ENO3__NM_001193503_GGTGGTGATCGGCATGGATG	ENO3__NM_001193503	60	45	93	55
ENO3__NM_001193503_TCCCAGCGAGATCTGCGATG	ENO3__NM_001193503	409	584	493	390
ENO4__NM_001242699_ACTTGTTTGGTTGTTAATTC	ENO4__NM_001242699	358	433	296	489
ENO4__NM_001242699_AGGTGCCCGTAGACGTCGGC	ENO4__NM_001242699	337	287	478	406
ENO4__NM_001242699_CAGAGGTTTATTAAGCAGCA	ENO4__NM_001242699	161	305	178	294
ENO4__NM_001242699_CAGTGGCAGTATGGCCATAG	ENO4__NM_001242699	442	763	499	531
ENO4__NM_001242699_CCACCATATGCAAAATAGTG	ENO4__NM_001242699	285	273	272	356
ENO4__NM_001242699_CCCACCATATGCAAAATAGT	ENO4__NM_001242699	1113	1253	1169	1194
ENO4__NM_001242699_CCCCTGGAGCTCGTGCGTGA	ENO4__NM_001242699	549	621	769	909
ENO4__NM_001242699_GAGACAAAAAAAGGGCACGA	ENO4__NM_001242699	492	707	484	505
ENO4__NM_001242699_GCAGAATATGTCCACTTGCA	ENO4__NM_001242699	1196	1214	1064	1303
ENO4__NM_001242699_GCCTTTGCTGATGGTATCGC	ENO4__NM_001242699	798	1106	737	1115
ENO4__NM_001242699_GTGGGGAAAGACGTACTAGA	ENO4__NM_001242699	160	164	161	180
ENO4__NM_001242699_TACCAGGTGCCCGTAGACGT	ENO4__NM_001242699	542	862	648	596
ENOPH1__NM_021204_AACCCCGATTGCTTTCGTGA	ENOPH1__NM_021204	2230	1821	2511	2888
ENOPH1__NM_021204_AGAATAAACCTAGACACTAC	ENOPH1__NM_021204	788	827	873	864
ENOPH1__NM_021204_AGACACTACAGGGACAACTC	ENOPH1__NM_021204	329	193	348	399
ENOPH1__NM_021204_ATAGATGTACACCTTCATTC	ENOPH1__NM_021204	942	1105	1048	987
ENOPH1__NM_021204_CACCTTCACGAAAGCAATCG	ENOPH1__NM_021204	1065	1014	1119	1054
ENOPH1__NM_021204_CACGAAAGCAATCGGGGTTG	ENOPH1__NM_021204	1106	1199	1137	1195
ENOPH1__NM_021204_CCAGCACACATTATCTACCA	ENOPH1__NM_021204	1940	2241	2111	2296
ENOPH1__NM_021204_CTATAATGATAACACGTGGC	ENOPH1__NM_021204	608	624	579	827
ENOPH1__NM_021204_CTCACCTTCACGAAAGCAAT	ENOPH1__NM_021204	758	696	808	1013
ENOPH1__NM_021204_GAATAAACCTAGACACTACA	ENOPH1__NM_021204	329	429	384	409
ENOPH1__NM_021204_GGATCACGGTGACTTCGGCG	ENOPH1__NM_021204	1270	1517	1365	1600
ENOPH1__NM_021204_GTGATCCTGTTAGATATCGA	ENOPH1__NM_021204	143	44	170	172
ENOPH1__NM_021204_GTGCAGTGGTCTTTCGATCC	ENOPH1__NM_021204	501	611	597	708
ENOPH1__NM_021204_TCACCTTCACGAAAGCAATC	ENOPH1__NM_021204	404	379	467	525
ENOPH1__NM_021204_TCATACTCTAACCCTTAGTG	ENOPH1__NM_021204	178	184	220	177
ENOPH1__NM_021204_TGAAGCAGAAGGAAATACCT	ENOPH1__NM_021204	335	340	282	309
ENOPH1__NM_021204_TGGTACCTTCGATATCTAAC	ENOPH1__NM_021204	0	0	8	7
ENOPH1__NM_021204_TTCGATATCTAACAGGATCA	ENOPH1__NM_021204	8	0	9	9
ENOPH1__NM_021204_TTCTTTCACTTATGTACCTA	ENOPH1__NM_021204	0	0	0	0
ENOSF1__NM_001126123_ACAAGTCCCACACCGCGTTT	ENOSF1__NM_001126123	1275	1035	1374	1154
ENOSF1__NM_001126123_ATGCAGAAGATGGAATCAAG	ENOSF1__NM_001126123	298	235	380	214
ENOSF1__NM_001126123_CGCACGATGTCGTGTAAGCA	ENOSF1__NM_001126123	1331	1413	1582	1929
ENOSF1__NM_001126123_CGGCCGTCCTAAACGCGGTG	ENOSF1__NM_001126123	263	168	320	374
ENOSF1__NM_001126123_CTCAGATGACACGTTGAAGC	ENOSF1__NM_001126123	430	549	563	710
ENOSF1__NM_001126123_CTTACACGACATCGTGCGCC	ENOSF1__NM_001126123	482	518	565	855
ENOSF1__NM_001126123_GACAGCGGCCGTCCTAAACG	ENOSF1__NM_001126123	471	604	533	455
ENOSF1__NM_001126123_GATGCAGAAGATGGAATCAA	ENOSF1__NM_001126123	170	179	262	242
ENOSF1__NM_001126123_GCGCACGATGTCGTGTAAGC	ENOSF1__NM_001126123	504	732	500	796
ENOSF1__NM_001126123_GGCCGTCCTAAACGCGGTGT	ENOSF1__NM_001126123	1127	1129	1263	1433
ENOSF1__NM_001126123_GTCCCACACCGCGTTTAGGA	ENOSF1__NM_001126123	716	776	669	708
ENOSF1__NM_001126123_GTGTGGAATTACCTTCACTC	ENOSF1__NM_001126123	447	447	520	612
ENOSF1__NM_017512_ACATAGGCAGCCGAGTAGTC	ENOSF1__NM_017512	577	423	513	399
ENOSF1__NM_017512_ACGTGGGGAAGCGCACGTCC	ENOSF1__NM_017512	22	0	20	1
ENOSF1__NM_017512_AGTCACCAACAATGTCCTTG	ENOSF1__NM_017512	144	147	157	154
ENOSF1__NM_017512_CATAGGCAGCCGAGTAGTCA	ENOSF1__NM_017512	724	782	807	806
ENOSF1__NM_017512_CCGTGGCCCCCAAGCGACGT	ENOSF1__NM_017512	179	251	252	305
ENOSF1__NM_017512_CGCTTCCCCACGTCGCTTGG	ENOSF1__NM_017512	1249	1097	1425	1100
ENOSF1__NM_017512_CGTGGCCCCCAAGCGACGTG	ENOSF1__NM_017512	188	139	217	166
ENOSF1__NM_017512_GCGCTTCCCCACGTCGCTTG	ENOSF1__NM_017512	997	1014	1019	1274
ENOSF1__NM_017512_GTCCTTGTTGAGCACATGGT	ENOSF1__NM_017512	927	658	993	922
ENOSF1__NM_202758_ATGGGCAGCTCAGATGGGTA	ENOSF1__NM_202758	448	416	563	387
ENOSF1__NM_202758_GTGACTTCAGAGGCTTCTAT	ENOSF1__NM_202758	376	255	483	416
ENOX1__NM_001127615_ACCCTACGGGAACCATGCCC	ENOX1__NM_001127615	73	49	77	44
ENOX1__NM_001127615_ACCTGGGCATGGTTCCCGTA	ENOX1__NM_001127615	1122	1245	1157	1090
ENOX1__NM_001127615_CCACAGCAGCCGATGGTTTG	ENOX1__NM_001127615	1615	2188	1665	2103
ENOX1__NM_001127615_CCCAGGAGGTCGTTCTCTTG	ENOX1__NM_001127615	923	1002	1014	1055
ENOX1__NM_001127615_CCTGGACAGCAGCTCGTGTC	ENOX1__NM_001127615	199	329	292	274
ENOX1__NM_001127615_CCTGGTATCATTGGGTTAAT	ENOX1__NM_001127615	727	923	740	734
ENOX1__NM_001127615_CGCTATACTCCCCAAACCAT	ENOX1__NM_001127615	1475	1655	1758	1313
ENOX1__NM_001127615_GCCTGGTATCATTGGGTTAA	ENOX1__NM_001127615	987	1377	917	1319
ENOX1__NM_001127615_GCTACAGCCATGAATAACCT	ENOX1__NM_001127615	585	700	507	503
ENOX1__NM_001127615_GTGGGGGAGGTACCAGTCCA	ENOX1__NM_001127615	328	339	322	253
ENOX1__NM_001127615_TGGTATCATTGGGTTAATGG	ENOX1__NM_001127615	1	0	2	0
ENOX1__NM_001127615_TTGACAACAGCCACTTCTGT	ENOX1__NM_001127615	1247	1298	1313	1171
ENOX2__NM_001281736_AACTCCAATGATGCCTGGTT	ENOX2__NM_001281736	915	1122	1014	1477
ENOX2__NM_001281736_ACCAGGTAAAATTGGTTGTC	ENOX2__NM_001281736	574	806	530	631
ENOX2__NM_001281736_ACTCCAATGATGCCTGGTTT	ENOX2__NM_001281736	1470	1727	1828	1855
ENOX2__NM_001281736_ACTCCACGTTGATTTCGCAC	ENOX2__NM_001281736	1301	1536	1463	1429
ENOX2__NM_001281736_ATTGCTGTGGCCCATGCAGT	ENOX2__NM_001281736	332	308	331	374
ENOX2__NM_001281736_GACAAAGCCCTGTATCTGTC	ENOX2__NM_001281736	1305	1553	1613	1904
ENOX2__NM_001281736_GGAGGTACTATTCCCAAACC	ENOX2__NM_001281736	136	106	155	169
ENOX2__NM_001281736_GGGCTCTAGTACTGACAAGA	ENOX2__NM_001281736	25	24	79	37
ENOX2__NM_001281736_TAATCTTGGAATGGCACCGC	ENOX2__NM_001281736	206	257	248	207
ENOX2__NM_001281736_TGCAAAACAGTATTTGTGGG	ENOX2__NM_001281736	383	356	476	601
ENOX2__NM_001281736_TGGGACAGAGCAAATCATTG	ENOX2__NM_001281736	328	353	284	277
ENOX2__NM_001281736_TTTACTACTGGCATATCTGG	ENOX2__NM_001281736	171	175	196	258
ENOX2__NM_182314_GTCTACGAAATAGGCTATGC	ENOX2__NM_182314	170	151	211	286
ENOX2__NM_182314_TAACAGTAGAACTCTGAACG	ENOX2__NM_182314	433	600	523	590
ENOX2__NM_182314_TGGCTGTGGGTCTACGAAAT	ENOX2__NM_182314	187	179	141	148
ENPEP__NM_001977_ACGTGCAGGTCGTAGTGGAC	ENPEP__NM_001977	1146	1140	1111	1348
ENPEP__NM_001977_ATTAATAGTGGGACTTGCCG	ENPEP__NM_001977	135	165	187	282
ENPEP__NM_001977_CACCACCACCGCACAGAGAA	ENPEP__NM_001977	759	738	731	1053
ENPEP__NM_001977_CGTGCAGGTCGTAGTGGACT	ENPEP__NM_001977	2118	2403	2368	2265
ENPEP__NM_001977_CTCCTGACCATGGAGTTCGC	ENPEP__NM_001977	402	204	482	302
ENPEP__NM_001977_CTGCATTCAAACGAAACATG	ENPEP__NM_001977	319	484	308	389
ENPEP__NM_001977_GTGCAGCCACAGGTACCGGG	ENPEP__NM_001977	971	979	1154	1364
ENPEP__NM_001977_GTGGGTGTAGGATTAATAGT	ENPEP__NM_001977	165	227	203	138
ENPEP__NM_001977_TGACCATGGAGTTCGCCGGC	ENPEP__NM_001977	908	932	923	1109
ENPEP__NM_001977_TGCAGCCACAGGTACCGGGT	ENPEP__NM_001977	1411	1511	1498	1148
ENPEP__NM_001977_TGGCTGAACGGCTCCCTCGT	ENPEP__NM_001977	336	203	383	415
ENPEP__NM_001977_TGTGGCCATTCTCTGTGCGG	ENPEP__NM_001977	879	1125	826	1029
ENPP1__NM_006208_AGAGTACTTTATAGGTGTTG	ENPP1__NM_006208	354	466	502	353
ENPP1__NM_006208_CACCTATAAAGTACTCTCGC	ENPP1__NM_006208	1819	1405	2189	1924
ENPP1__NM_006208_CGAGAGTACTTTATAGGTGT	ENPP1__NM_006208	611	889	629	599
ENPP1__NM_006208_CTCCTCCCCCACGTCCATAG	ENPP1__NM_006208	708	461	835	688
ENPP1__NM_006208_CTTGCTGGCCCCTATGGACG	ENPP1__NM_006208	611	544	596	510
ENPP1__NM_006208_GAGAGTACTTTATAGGTGTT	ENPP1__NM_006208	259	368	379	468
ENPP1__NM_006208_GCTCCTCCCCCACGTCCATA	ENPP1__NM_006208	571	869	697	814
ENPP1__NM_006208_GGCTCCTCCCCCACGTCCAT	ENPP1__NM_006208	994	1006	1010	909
ENPP1__NM_006208_TATGGACTTGCAACAAATTC	ENPP1__NM_006208	243	173	290	378
ENPP1__NM_006208_TGCTGGCCCCTATGGACGTG	ENPP1__NM_006208	921	975	878	1198
ENPP1__NM_006208_TGTGTGTTAACAACAATACT	ENPP1__NM_006208	248	156	181	123
ENPP1__NM_006208_TTGCTGGCCCCTATGGACGT	ENPP1__NM_006208	946	781	866	951
ENPP2__NM_001040092_AACATCTCCGGATCTTGCAA	ENPP2__NM_001040092	744	470	752	483
ENPP2__NM_001040092_AAGTTGTCACAGCGACAATC	ENPP2__NM_001040092	133	54	178	184
ENPP2__NM_001040092_ACGGAGAAGATGATTAATGG	ENPP2__NM_001040092	615	551	744	688
ENPP2__NM_001040092_AGATCGGAGATGTTGGTCC	ENPP2__NM_001040092	655	574	720	701
ENPP2__NM_001040092_CCGTGGCTGGGAGTGTACTA	ENPP2__NM_001040092	1322	1566	1479	1624
ENPP2__NM_001040092_CGGAGAAGATGATTAATGGA	ENPP2__NM_001040092	939	890	1073	995
ENPP2__NM_001040092_CTGCTGTACCAATTACCAAG	ENPP2__NM_001040092	420	418	542	372
ENPP2__NM_001040092_CTTGCAAGATCCGGAGATGT	ENPP2__NM_001040092	1340	1215	1358	1334
ENPP2__NM_001040092_CTTTCCTAACTTATACACTT	ENPP2__NM_001040092	314	515	456	607
ENPP2__NM_001040092_CTTTCTTCATGTATGATGCA	ENPP2__NM_001040092	324	586	425	561
ENPP2__NM_001040092_TGATGACTGTGAGGAAATAA	ENPP2__NM_001040092	279	296	247	296
ENPP2__NM_001040092_TTGTCACAGCGACAATCAGG	ENPP2__NM_001040092	580	489	625	382
ENPP3__NM_005021_AATCCCAGCAGCAATCACCT	ENPP3__NM_005021	1158	1433	1383	1350
ENPP3__NM_005021_ATGTCACTTGGATTAGGCCT	ENPP3__NM_005021	537	503	583	828
ENPP3__NM_005021_ATGTGCAATAAATTTCGTTG	ENPP3__NM_005021	983	976	1227	888
ENPP3__NM_005021_GAGCTGAATATTTATACACA	ENPP3__NM_005021	387	610	451	482
ENPP3__NM_005021_GATGTGGCATGTAAAGACCG	ENPP3__NM_005021	688	572	733	790
ENPP3__NM_005021_GCAAAGGCTGGCCTCTAATC	ENPP3__NM_005021	979	1365	1123	919
ENPP3__NM_005021_GGAGAACTGCCGGTGTGATG	ENPP3__NM_005021	1024	948	1074	951
ENPP3__NM_005021_GTGACAATGGTGTAATGATT	ENPP3__NM_005021	310	240	376	559
ENPP3__NM_005021_TGATGCATCATTTAGAGGAC	ENPP3__NM_005021	563	773	677	798
ENPP3__NM_005021_TGCTTTGATGCATCATTTAG	ENPP3__NM_005021	178	237	232	340
ENPP3__NM_005021_TTCAGGAGAAACCTCATGGC	ENPP3__NM_005021	40	64	76	41
ENPP3__NM_005021_TTGTGGAGAGACCAGATTAG	ENPP3__NM_005021	169	135	198	190
ENPP4__NM_014936_AACCACTACAGTATTGTGAC	ENPP4__NM_014936	681	897	824	1273
ENPP4__NM_014936_AAGGATACTAGTAGTAACTT	ENPP4__NM_014936	68	68	94	62
ENPP4__NM_014936_AGCCTGTCACAATACTGTAG	ENPP4__NM_014936	606	402	681	640
ENPP4__NM_014936_GAAGAGATGGTATCGTGAAT	ENPP4__NM_014936	1104	1472	1280	1208
ENPP4__NM_014936_GAATGAGGCAGTACCTATTT	ENPP4__NM_014936	1177	915	1214	1064
ENPP4__NM_014936_GGAAGAGATGGTATCGTGAA	ENPP4__NM_014936	500	482	495	559
ENPP4__NM_014936_GGAATGAGGCAGTACCTATT	ENPP4__NM_014936	752	550	698	585
ENPP4__NM_014936_GTCACAATACTGTAGTGGTT	ENPP4__NM_014936	672	772	672	936
ENPP4__NM_014936_GTCCAAAGACTCAAGATGTT	ENPP4__NM_014936	1200	1065	1417	1354
ENPP4__NM_014936_TCACCTTTGCAACACTATAT	ENPP4__NM_014936	711	876	887	1125
ENPP4__NM_014936_TCCAAAGACTCAAGATGTTA	ENPP4__NM_014936	514	851	696	1062
ENPP4__NM_014936_TCTTTCCTCAAATGACACTG	ENPP4__NM_014936	161	211	220	173
ENPP5__NM_001290072_AACCATTATACTTTGGTAAC	ENPP5__NM_001290072	292	538	303	303
ENPP5__NM_001290072_AATGAAACTGACTCATTGTA	ENPP5__NM_001290072	671	755	720	723
ENPP5__NM_001290072_ATAGAGAAGACCAAGATTTA	ENPP5__NM_001290072	170	148	194	121
ENPP5__NM_001290072_ATATAATGAAAATGGGGCGT	ENPP5__NM_001290072	284	271	328	310
ENPP5__NM_001290072_CAGAGGGCAGGACATACTAG	ENPP5__NM_001290072	1092	1142	1220	1067
ENPP5__NM_001290072_CTACCCTAACCATTATACTT	ENPP5__NM_001290072	930	811	1052	1200
ENPP5__NM_001290072_CTCTGGTTTGTGATCCATAT	ENPP5__NM_001290072	355	379	372	586
ENPP5__NM_001290072_GCCTCTTTGCAGAGAATCAT	ENPP5__NM_001290072	632	762	740	748
ENPP5__NM_001290072_GGGAAGAAGCGACACCAATA	ENPP5__NM_001290072	352	297	415	347
ENPP5__NM_001290072_GTTACCAAAGTATAATGGTT	ENPP5__NM_001290072	423	436	449	339
ENPP5__NM_001290072_TAGAGAAGACCAAGATTTAT	ENPP5__NM_001290072	486	746	546	427
ENPP5__NM_001290072_TTGTAAGGCATGTAATGAGT	ENPP5__NM_001290072	243	272	305	349
ENPP5__NM_001290073_AAATCTTGGTCTTCTCTATT	ENPP5__NM_001290073	418	806	504	533
ENPP5__NM_001290073_ATTGATCAATCAGGGTATAG	ENPP5__NM_001290073	191	109	226	236
ENPP5__NM_001290073_GGCCCCATGAGCGGACTGTC	ENPP5__NM_001290073	843	1327	907	1074
ENPP5__NM_001290073_GGGACCTGACAGTCCGCTCA	ENPP5__NM_001290073	392	478	468	408
ENPP5__NM_001290073_TACTGGAGATTGATCAATCA	ENPP5__NM_001290073	407	213	457	408
ENPP6__NM_153343_AATTGGGATACGAGAGACTA	ENPP6__NM_153343	308	259	283	302
ENPP6__NM_153343_GATGATCGGGAACTACATGT	ENPP6__NM_153343	1475	1573	1547	1431
ENPP6__NM_153343_GGAAGGTCTACATGTACTAC	ENPP6__NM_153343	463	699	656	593
ENPP6__NM_153343_GTGAAGTCCATCAGATGATC	ENPP6__NM_153343	368	272	414	435
ENPP6__NM_153343_GTTTCAAAGAGATTGTGAGC	ENPP6__NM_153343	352	276	360	314
ENPP6__NM_153343_TAATTGGGATACGAGAGACT	ENPP6__NM_153343	334	331	489	350
ENPP6__NM_153343_TCATCTGATGGACTTCACAA	ENPP6__NM_153343	404	334	465	353
ENPP6__NM_153343_TGGCAAAATTGATATCCGTT	ENPP6__NM_153343	197	284	229	294
ENPP6__NM_153343_TGTAGTTCCCGATCATCTGA	ENPP6__NM_153343	301	221	343	243
ENPP6__NM_153343_TTGGCAAAATTGATATCCGT	ENPP6__NM_153343	839	918	881	1216
ENPP6__NM_153343_TTTCAAAGAGATTGTGAGCA	ENPP6__NM_153343	96	95	72	76
ENPP6__NM_153343_TTTGTTGACGCCAATGTCAA	ENPP6__NM_153343	329	274	380	443
ENPP7__NM_178543_ACTACAAAAATGAGACGGAG	ENPP7__NM_178543	700	630	782	755
ENPP7__NM_178543_ATCCAGAGGTGGTGGGACAA	ENPP7__NM_178543	38	18	33	17
ENPP7__NM_178543_CGTCACAGCCACCCCTTGGT	ENPP7__NM_178543	1459	804	1340	884
ENPP7__NM_178543_CTGACTTTGTACCGGTGCTC	ENPP7__NM_178543	128	73	116	109
ENPP7__NM_178543_GAACGTCACCTACCAAGGGG	ENPP7__NM_178543	89	35	80	93
ENPP7__NM_178543_GCAAATATATCGAGAACCAC	ENPP7__NM_178543	704	441	784	583
ENPP7__NM_178543_GGCTCCTTCTTCTACCCGGG	ENPP7__NM_178543	922	1009	1065	906
ENPP7__NM_178543_GTTCCAGCGGAAGCCGTCGA	ENPP7__NM_178543	231	185	267	251
ENPP7__NM_178543_TCTGGTCACACTCTACTTCG	ENPP7__NM_178543	576	570	702	576
ENPP7__NM_178543_TGGGAGCCCTGACTTTGTAC	ENPP7__NM_178543	458	172	323	208
ENPP7__NM_178543_TGGTAGGTGACGTTCCCGCC	ENPP7__NM_178543	34	86	25	23
ENPP7__NM_178543_TGTCCTTCGACGGCTTCCGC	ENPP7__NM_178543	358	335	390	393
ENSA__NM_004436_AGCTGAAGAGGCAAAGCTAA	ENSA__NM_004436	183	151	281	228
ENSA__NM_004436_AGGTTACCCCGCAAGCTTGC	ENSA__NM_004436	81	139	88	211
ENSA__NM_004436_GGATGTGATCACCAGTCACC	ENSA__NM_004436	252	186	265	227
ENSA__NM_004436_TCGTCACCAGCAAGCTTGCG	ENSA__NM_004436	185	206	187	169
ENSA__NM_004436_TTGTCCACCTAGGCTTGGGTATT	ENSA__NM_004436	118	35	201	209
ENSA__NM_207042_CTCTGTGCGGATGAAATGGT	ENSA__NM_207042	285	320	266	238
ENSA__NM_207045_TGTGTGTTATTGGTTTGTAG	ENSA__NM_207045	324	249	322	367
ENTHD1__NM_152512_ACCATTGCTGTGTAAAGAGA	ENTHD1__NM_152512	293	184	374	355
ENTHD1__NM_152512_AGAAAACAATATAGAGTGGG	ENTHD1__NM_152512	141	136	89	85
ENTHD1__NM_152512_AGATGCTGAAATAAAAGTCA	ENTHD1__NM_152512	359	230	403	260
ENTHD1__NM_152512_AGCAGAAATATCGGTGTGG	ENTHD1__NM_152512	601	499	581	500
ENTHD1__NM_152512_CTTGATGAGATAATCATTA	ENTHD1__NM_152512	155	158	229	283
ENTHD1__NM_152512_GAGATAATCCATTAGGTAA	ENTHD1__NM_152512	112	49	105	112
ENTHD1__NM_152512_GTATAAATCCCTTACCCTAA	ENTHD1__NM_152512	187	130	257	224
ENTHD1__NM_152512_TCCCTCTCTTTACACAGCAA	ENTHD1__NM_152512	248	241	260	192
ENTHD1__NM_152512_TCTAACATCAGAGAACTAGA	ENTHD1__NM_152512	236	198	258	217
ENTHD1__NM_152512_TGAAGCAGAAATATCCGGTG	ENTHD1__NM_152512	752	586	822	776
ENTHD1__NM_152512_TGAGATAATCCATTAGGGTA	ENTHD1__NM_152512	56	50	148	177
ENTHD1__NM_152512_TTTCAGCACATAGATGAAGC	ENTHD1__NM_152512	27	43	34	28
ENTHD2__NM_144679_AAGCTCAGGCGGTCCCGTAG	ENTHD2__NM_144679	202	199	202	162
ENTHD2__NM_144679_ACCGCCTGAGCTTTCTACAC	ENTHD2__NM_144679	1209	744	1202	964
ENTHD2__NM_144679_CAACACGGTGTCCGAGAACA	ENTHD2__NM_144679	285	288	271	246
ENTHD2__NM_144679_CCAGCTGTGAGGCATCAGCC	ENTHD2__NM_144679	307	459	293	400
ENTHD2__NM_144679_CCGGCAGCGACAGCCATTCA	ENTHD2__NM_144679	396	666	488	547
ENTHD2__NM_144679_CGATGATGATGTCCCGTGTC	ENTHD2__NM_144679	1555	1493	1642	1992
ENTHD2__NM_144679_CTTGTACCAGAAGGTTCGCG	ENTHD2__NM_144679	618	349	797	402
ENTHD2__NM_144679_GCTCCCGGCTGGCCCCTGAA	ENTHD2__NM_144679	1131	1070	1112	945
ENTHD2__NM_144679_GCTGCTGCCCGGAGACTCGT	ENTHD2__NM_144679	262	401	328	255
ENTHD2__NM_144679_GCTGTAGCCGAAACCCTGGA	ENTHD2__NM_144679	612	708	673	554
ENTHD2__NM_144679_TACAAGCTGTTCCCGTGCAG	ENTHD2__NM_144679	68	70	89	39
ENTHD2__NM_144679_TCACCCGGTGTAGAAAGCTC	ENTHD2__NM_144679	19	0	8	0
ENTPD1__NM_001098175_AAGAGGAAGGTGCCTATGGC	ENTPD1__NM_001098175	566	709	747	943
ENTPD1__NM_001098175_AGCATTGCCAGAAAACGTTA	ENTPD1__NM_001098175	46	26	50	130
ENTPD1__NM_001098175_AGCTATCACAGCTATGATAG	ENTPD1__NM_001098175	142	151	179	229
ENTPD1__NM_001098175_CAAGAGACACCCGTTTACCT	ENTPD1__NM_001098175	2342	2463	2818	2580
ENTPD1__NM_001098175_CCAAGAGACACCCGTTTACC	ENTPD1__NM_001098175	819	837	818	795
ENTPD1__NM_001098175_CCCATATGAAACCAATAATC	ENTPD1__NM_001098175	753	943	735	878
ENTPD1__NM_001098175_CCTGATTATTGGTTTCATAT	ENTPD1__NM_001098175	842	1078	979	1364
ENTPD1__NM_001098175_CTCTTGGCCAGTAATGATCC	ENTPD1__NM_001098175	317	371	365	452
ENTPD1__NM_001098175_CTCTTTCCATGCAATCAGTC	ENTPD1__NM_001098175	346	450	276	392
ENTPD1__NM_001098175_TATCTGGGGACTCGATAGTC	ENTPD1__NM_001098175	426	448	501	445
ENTPD1__NM_001098175_TCTGCTGGGCAAATTCAGTC	ENTPD1__NM_001098175	631	738	655	775
ENTPD1__NM_001098175_TTTCTGGCAATGCTTTGTTC	ENTPD1__NM_001098175	88	162	150	201
ENTPD1__NM_001164181_CACATAGCTTCTTGTGCTAT	ENTPD1__NM_001164181	631	845	722	900
ENTPD1__NM_001164181_CGAAATTGCAGAGCATTATC	ENTPD1__NM_001164181	391	277	443	635
ENTPD1__NM_001164181_CTTTCATCCTGGATATAAGA	ENTPD1__NM_001164181	572	528	674	532
ENTPD1__NM_001164181_GAAATTGCAGAGCATTATCT	ENTPD1__NM_001164181	275	213	322	380
ENTPD1__NM_001164182_ATCTCAAATCTCTTGGTGCA	ENTPD1__NM_001164182	654	945	753	987
ENTPD1__NM_001164182_ATTCCAGCAGTTTGAAATCC	ENTPD1__NM_001164182	501	501	498	539
ENTPD2__NM_001246_AACCAGATCTCGGCAGAGGT	ENTPD2__NM_001246	392	361	423	382
ENTPD2__NM_001246_AAGCTGCCAAGCACCATTTA	ENTPD2__NM_001246	445	616	663	600
ENTPD2__NM_001246_CAGTACCCCTTGACTTCCG	ENTPD2__NM_001246	1081	912	954	1085
ENTPD2__NM_001246_CATCCTGAAGCGCCTGTTCG	ENTPD2__NM_001246	2628	2848	2847	2920
ENTPD2__NM_001246_CCTGGTCACGGCCATAGCAG	ENTPD2__NM_001246	184	198	245	260
ENTPD2__NM_001246_CGGCCACTTGTAGAAAACA	ENTPD2__NM_001246	277	317	316	330
ENTPD2__NM_001246_CGGTGGTTCCGGCCACGGAA	ENTPD2__NM_001246	0	0	0	0
ENTPD2__NM_001246_GCAGACAAGGAGAACGACAC	ENTPD2__NM_001246	376	259	473	377
ENTPD2__NM_001246_TCGAGGCCTCTGGATTGGTC	ENTPD2__NM_001246	1015	556	1077	1083
ENTPD2__NM_001246_TGGGCCGGTGGTTCCGGCCA	ENTPD2__NM_001246	0	0	0	0
ENTPD2__NM_001246_TGGGGACGACGACCAGTAGG	ENTPD2__NM_001246	96	42	92	101
ENTPD2__NM_001246_TGGTTGTCTCAAAAGTGATC	ENTPD2__NM_001246	0	0	0	0
ENTPD2__NM_203468_CGCCTGGCCGACTACTGCGC	ENTPD2__NM_203468	2509	2491	2825	2553
ENTPD3__NM_001248_AAAAGAGAATAATACCGGAG	ENTPD3__NM_001248	7	8	12	4
ENTPD3__NM_001248_ACCACCAAGGCAATGATGGT	ENTPD3__NM_001248	1191	1181	1306	1339
ENTPD3__NM_001248_AGTGTATGCAAAAAGTCAAG	ENTPD3__NM_001248	310	203	390	432
ENTPD3__NM_001248_CCACAGTCTACGTGTATCAA	ENTPD3__NM_001248	1032	784	961	1111
ENTPD3__NM_001248_CTGCTTGCTCACATGGTTGG	ENTPD3__NM_001248	777	1271	776	1061
ENTPD3__NM_001248_GATGGTTGGAGTTCGGTAGA	ENTPD3__NM_001248	322	294	289	330
ENTPD3__NM_001248_GCAGAAAAAGAGAATAATAC	ENTPD3__NM_001248	271	320	281	159
ENTPD3__NM_001248_GGGCAAGAAGAAGGGGTATA	ENTPD3__NM_001248	355	274	402	468
ENTPD3__NM_001248_GGTCTTGCTTGTGAGTATTG	ENTPD3__NM_001248	524	781	594	785
ENTPD3__NM_001248_TGATGGTTGGAGTTCGGTAG	ENTPD3__NM_001248	332	559	410	556
ENTPD3__NM_001248_TGCTTGCTCACATGGTTGGC	ENTPD3__NM_001248	351	429	374	427
ENTPD3__NM_001248_TTGGGGGTTATTTCCATAGC	ENTPD3__NM_001248	44	38	62	37
ENTPD4__NM_001128930_AAATTGGTATTCAGAATTCG	ENTPD4__NM_001128930	337	467	428	497
ENTPD4__NM_001128930_AATAACCCCAATGTGAACTA	ENTPD4__NM_001128930	157	232	200	246
ENTPD4__NM_001128930_ACGATCCCATAGTCACATT	ENTPD4__NM_001128930	2028	2030	2299	2274
ENTPD4__NM_001128930_ATAACCCCAATGTGAACTAT	ENTPD4__NM_001128930	279	296	307	440
ENTPD4__NM_001128930_ATAGTTCACATTGGGGTTAT	ENTPD4__NM_001128930	426	618	463	578
ENTPD4__NM_001128930_CAATGTCGGTAACTCGTGCC	ENTPD4__NM_001128930	1575	2292	1794	2084
ENTPD4__NM_001128930_CAATGTGAACTATGGGATCG	ENTPD4__NM_001128930	2035	2361	2072	2532
ENTPD4__NM_001128930_CACGATCCCATAGTTCACAT	ENTPD4__NM_001128930	1193	1400	1355	1604
ENTPD4__NM_001128930_CTCGAGTATTTGTTTACTGC	ENTPD4__NM_001128930	951	1402	1045	1257
ENTPD4__NM_001128930_GACCATAATTTGGCGTAAAT	ENTPD4__NM_001128930	409	596	571	633
ENTPD4__NM_001128930_TACCAATTTACGCCAAATTA	ENTPD4__NM_001128930	403	587	472	867
ENTPD4__NM_001128930_TACTGCTGGCCAAGGCATAA	ENTPD4__NM_001128930	264	353	253	335
ENTPD5__NM_001249_AACCAGCAGACTTGGTTTGA	ENTPD5__NM_001249	632	645	627	792
ENTPD5__NM_001249_AAGGGTGCTGAGACCGTTCA	ENTPD5__NM_001249	486	444	452	460
ENTPD5__NM_001249_ACAGCTTCCAAATTCTAGAAG	ENTPD5__NM_001249	316	428	335	450
ENTPD5__NM_001249_ACTGTGGGGACCTTGGACCT	ENTPD5__NM_001249	1071	936	1253	1184
ENTPD5__NM_001249_AGGGTGCTGAGACCGTTCAA	ENTPD5__NM_001249	393	163	528	380
ENTPD5__NM_001249_ATCATGGATGGATCCGACGA	ENTPD5__NM_001249	179	74	161	97
ENTPD5__NM_001249_ATGGATGGATCCGACGAAGG	ENTPD5__NM_001249	76	131	65	78
ENTPD5__NM_001249_CACCTCTAAGAGCCCTTGAA	ENTPD5__NM_001249	928	1269	992	1222
ENTPD5__NM_001249_GTGCTGGCGCTGACATTGAT	ENTPD5__NM_001249	573	656	610	653
ENTPD5__NM_001249_TCACCTTCCTGGTACCAAA	ENTPD5__NM_001249	230	400	273	261
ENTPD5__NM_001249_TGGATGGATCCGACGAAGGT	ENTPD5__NM_001249	25	9	45	30
ENTPD5__NM_001249_TGTTGCCTTTAGGACCACTG	ENTPD5__NM_001249	112	139	163	117
ENTPD6__NM_001114089_AAGCATCGCCTTTCCTTGTA	ENTPD6__NM_001114089	906	836	1004	902
ENTPD6__NM_001114089_AAGGTTTCGTGGGTTAACGT	ENTPD6__NM_001114089	178	175	188	226
ENTPD6__NM_001114089_ATGAGAAAAATATCCAACCA	ENTPD6__NM_001114089	28	101	29	9
ENTPD6__NM_001114089_CGAAGGTGGCATACCCCCTG	ENTPD6__NM_001114089	716	848	711	844
ENTPD6__NM_001114089_GAACTGGAAGACGTGTACTC	ENTPD6__NM_001114089	225	268	212	280
ENTPD6__NM_001114089_GAAGGTTTCGTGGGTTAACG	ENTPD6__NM_001114089	72	127	76	89
ENTPD6__NM_001114089_GGCCTTGAGGACCAGAGGGG	ENTPD6__NM_001114089	73	76	88	108
ENTPD6__NM_001114089_GTGGCCTTCCAGAAGTCGAA	ENTPD6__NM_001114089	96	58	99	91
ENTPD6__NM_001114089_TCAGGGAATCCGGGAACTAC	ENTPD6__NM_001114089	224	163	242	175
ENTPD6__NM_001114089_TGAACTGGAAGACGTGTACT	ENTPD6__NM_001114089	310	441	397	289
ENTPD6__NM_001114089_TGAGAAAAATATCCAACCAC	ENTPD6__NM_001114089	133	67	174	234
ENTPD6__NM_001114089_TTGCCTACATCAAGTGGCAC	ENTPD6__NM_001114089	95	111	107	110
ENTPD7__NM_020354_AAAGAAAACGTTACCTGAGT	ENTPD7__NM_020354	218	229	263	283
ENTPD7__NM_020354_AACATCAAAATAGGAGTCCG	ENTPD7__NM_020354	469	444	559	469
ENTPD7__NM_020354_AACATCATCAAACTACGAC	ENTPD7__NM_020354	693	1030	738	974
ENTPD7__NM_020354_AACCAGAGATGGTAAGTGAC	ENTPD7__NM_020354	374	410	462	464
ENTPD7__NM_020354_AAGTGAGTGGTATCACTTAA	ENTPD7__NM_020354	171	146	132	227
ENTPD7__NM_020354_AATTCAGGGGGTACAAGTGC	ENTPD7__NM_020354	408	72	453	74
ENTPD7__NM_020354_ACAAGTCATCACATACCTAT	ENTPD7__NM_020354	196	167	284	254
ENTPD7__NM_020354_ACAAGTCCATAATTCAGATT	ENTPD7__NM_020354	503	807	499	563
ENTPD7__NM_020354_ACACTGTGGACTACTAGAGC	ENTPD7__NM_020354	255	42	243	18
ENTPD7__NM_020354_ACAGAGCACTTGCCGGCATT	ENTPD7__NM_020354	1943	1780	1685	2126
ENTPD7__NM_020354_ACAGGAGATTGGTGCTAATA	ENTPD7__NM_020354	308	217	323	238
ENTPD7__NM_020354_ACATCATCAACACTACGACA	ENTPD7__NM_020354	385	513	338	258
ENTPD7__NM_020354_ACATTTATGAAGCTAGTAAG	ENTPD7__NM_020354	286	301	339	298
ENTPD7__NM_020354_ACCACTTGTATACCCTCTGG	ENTPD7__NM_020354	464	217	458	337
ENTPD7__NM_020354_ACCAGCAGCAGGCCAAATGC	ENTPD7__NM_020354	221	292	337	306
ENTPD7__NM_020354_ACTCAGCAGTCTCTTAATAG	ENTPD7__NM_020354	133	128	179	176
ENTPD7__NM_020354_ACTTCAGGACTTCAACTAAA	ENTPD7__NM_020354	230	369	269	365
ENTPD7__NM_020354_ACTTCCATTAATGAAGCGAG	ENTPD7__NM_020354	856	838	1001	1073
ENTPD7__NM_020354_AGATGGGGCATGGTTAGTTG	ENTPD7__NM_020354	780	739	924	782
ENTPD7__NM_020354_AGGAATCTCTTAGGATGAGG	ENTPD7__NM_020354	221	340	254	274
ENTPD7__NM_020354_AGGAGATTGGTGCTAATACG	ENTPD7__NM_020354	264	199	323	261
ENTPD7__NM_020354_ATCACCCATATAAATCAAGG	ENTPD7__NM_020354	226	282	259	128
ENTPD7__NM_020354_ATGAAAGAAACCCTAATGAA	ENTPD7__NM_020354	212	197	212	220
ENTPD7__NM_020354_ATGTGTGATGCATGTTAGAG	ENTPD7__NM_020354	152	168	190	239
ENTPD7__NM_020354_CAAATACATCTATTGTAGC	ENTPD7__NM_020354	291	327	375	450
ENTPD7__NM_020354_CAAGTGAGTGGTATCACTTA	ENTPD7__NM_020354	382	395	465	418
ENTPD7__NM_020354_CAATCTGTTAATAAGCCATC	ENTPD7__NM_020354	321	340	400	481
ENTPD7__NM_020354_CACGAGATAGGCAATACGAA	ENTPD7__NM_020354	312	385	280	297
ENTPD7__NM_020354_CAGAGGACGAAGGTAATCAC	ENTPD7__NM_020354	563	508	611	520
ENTPD7__NM_020354_CAGCCGTGGGAGCATATTCG	ENTPD7__NM_020354	796	1058	906	1184
ENTPD7__NM_020354_CAGGAGTTCCACAGTACTGA	ENTPD7__NM_020354	608	375	636	671
ENTPD7__NM_020354_CCACAGTGTCTATTCTGGTC	ENTPD7__NM_020354	1223	1571	1375	1555
ENTPD7__NM_020354_CCATCCTACTAACACACTTA	ENTPD7__NM_020354	1041	786	1160	1194
ENTPD7__NM_020354_CCCACCAACCTTATCATCAG	ENTPD7__NM_020354	1127	1250	1179	1213
ENTPD7__NM_020354_CCCCTGATGATAAGGTTGGT	ENTPD7__NM_020354	1149	1363	1277	1401
ENTPD7__NM_020354_CCTCACAAGTTATTGCCCCA	ENTPD7__NM_020354	1495	1362	1690	1577
ENTPD7__NM_020354_CCTTAAGTGTGTTAGTAGGA	ENTPD7__NM_020354	871	884	915	876
ENTPD7__NM_020354_CTGCCTGTATTACATTGCTT	ENTPD7__NM_020354	288	667	447	493
ENTPD7__NM_020354_CTTAGGTATTTGGCTCGAGT	ENTPD7__NM_020354	256	245	203	274
ENTPD7__NM_020354_CTTTCGTATTGCCTATCTCG	ENTPD7__NM_020354	923	1005	886	915
ENTPD7__NM_020354_GAAATCTCTTACCTTTCATT	ENTPD7__NM_020354	322	347	371	246
ENTPD7__NM_020354_GAATCATTACTCATACTACA	ENTPD7__NM_020354	537	689	656	834
ENTPD7__NM_020354_GAGTGGTGGTAAGATTCAA	ENTPD7__NM_020354	95	66	114	91
ENTPD7__NM_020354_GAGTGTCTGCTACATAACAA	ENTPD7__NM_020354	176	353	194	142
ENTPD7__NM_020354_GATGAGAACCGAACCAGAGA	ENTPD7__NM_020354	238	161	214	209
ENTPD7__NM_020354_GCAGACCTGGATTCTAGTTC	ENTPD7__NM_020354	978	1014	1110	1390
ENTPD7__NM_020354_GCCACCAGAGGGTATACAAG	ENTPD7__NM_020354	189	276	288	264
ENTPD7__NM_020354_GCTAAAATCAGTATGTAGTA	ENTPD7__NM_020354	753	729	672	926
ENTPD7__NM_020354_GCTTTGTCATTTACTAGCTC	ENTPD7__NM_020354	12	13	32	21
ENTPD7__NM_020354_GGAGATCCACAACCTTTAAC	ENTPD7__NM_020354	283	300	329	322
ENTPD7__NM_020354_GGAGATTGGTGCTAATACGG	ENTPD7__NM_020354	419	255	451	291
ENTPD7__NM_020354_GGATTAAAGAACTATCTGTT	ENTPD7__NM_020354	247	233	184	163
ENTPD7__NM_020354_GGCCACTTAGGAAAGTACCA	ENTPD7__NM_020354	261	203	317	224
ENTPD7__NM_020354_GGTAGATGGGACACCCAAAC	ENTPD7__NM_020354	133	149	175	153
ENTPD7__NM_020354_GGTGCCTGGAAATTCCTATG	ENTPD7__NM_020354	446	574	401	476
ENTPD7__NM_020354_GTAGTATGGGGAATGTATTT	ENTPD7__NM_020354	536	408	521	463
ENTPD7__NM_020354_GTTTGATGTCCAGCAAGTCA	ENTPD7__NM_020354	267	172	250	349
ENTPD7__NM_020354_TAATCATAGACTTTCGACAT	ENTPD7__NM_020354	357	602	450	353
ENTPD7__NM_020354_TACTTCAGGACTTCAACTAA	ENTPD7__NM_020354	382	392	474	453
ENTPD7__NM_020354_TATCACCCATATAAATCAAG	ENTPD7__NM_020354	193	284	287	142
ENTPD7__NM_020354_TATTTCTGGCCAAGACATAA	ENTPD7__NM_020354	23	52	44	30
ENTPD7__NM_020354_TCCCACCAACCTTATCATCA	ENTPD7__NM_020354	625	431	608	701
ENTPD7__NM_020354_TCCTTGAATTTCCTTAATCT	ENTPD7__NM_020354	717	725	713	737
ENTPD7__NM_020354_TCTGAGGCTATATAAGAAAG	ENTPD7__NM_020354	121	113	102	234
ENTPD7__NM_020354_TCTGGTGAATGATTATCTTA	ENTPD7__NM_020354	149	218	211	182
ENTPD7__NM_020354_TCTGTGAGGAACTAAATGAC	ENTPD7__NM_020354	277	334	322	276
ENTPD7__NM_020354_TGACACCATAATTATCTTCA	ENTPD7__NM_020354	535	751	593	768
ENTPD7__NM_020354_TGACTCTTGGAATGTCTGAA	ENTPD7__NM_020354	117	99	135	155
ENTPD7__NM_020354_TGAGCCTTAAGTGTGTTAGT	ENTPD7__NM_020354	596	826	647	888
ENTPD7__NM_020354_TGATAACGGTACTGACAAAC	ENTPD7__NM_020354	415	594	492	724
ENTPD7__NM_020354_TGATGTCCAGCAAGTCATGG	ENTPD7__NM_020354	631	477	662	772
ENTPD7__NM_020354_TGCAGTTCAATCGACCACAT	ENTPD7__NM_020354	404	574	566	426
ENTPD7__NM_020354_TGGATCCCTGTTAAAGGTTG	ENTPD7__NM_020354	12	22	23	29
ENTPD7__NM_020354_TGGCTCATTCTCTCATAACC	ENTPD7__NM_020354	7	20	26	22
ENTPD7__NM_020354_TGGGAACCCCCATGACTTGC	ENTPD7__NM_020354	216	60	297	303
ENTPD7__NM_020354_TGTCTTTATTACCATGAGCA	ENTPD7__NM_020354	70	93	130	123
ENTPD7__NM_020354_TGTGGTCCTGGGATGAATGG	ENTPD7__NM_020354	139	251	111	191
ENTPD7__NM_020354_TTAGGTATTTGGCTCGAGTA	ENTPD7__NM_020354	52	24	76	124
ENTPD7__NM_020354_TTATGTAGCAGACACTCTTA	ENTPD7__NM_020354	453	466	400	310
ENTPD7__NM_020354_TTGATGTCCAGCAAGTCATG	ENTPD7__NM_020354	89	67	88	79
ENTPD7__NM_020354_TTTGTCAGTACCGTTATCAG	ENTPD7__NM_020354	1688	1703	1953	1567
ENTPD8__NM_001033113_ACCCAGATCACGTTCGTGCC	ENTPD8__NM_001033113	1673	2044	2060	2423
ENTPD8__NM_001033113_AGGCCGAAGGTGCCTTTGGT	ENTPD8__NM_001033113	1203	977	1443	1207
ENTPD8__NM_001033113_ATCACTGTCAACTACGGCTT	ENTPD8__NM_001033113	393	586	465	489
ENTPD8__NM_001033113_CAGCCACTGATACAGGAAGA	ENTPD8__NM_001033113	478	682	672	605
ENTPD8__NM_001033113_CCCAAAAGTCCACGGGAGAC	ENTPD8__NM_001033113	849	756	908	1319
ENTPD8__NM_001033113_CCCAGATCACGTTCGTGCCT	ENTPD8__NM_001033113	1150	1173	1203	1601
ENTPD8__NM_001033113_CCCCAGGCACGAACGTGATC	ENTPD8__NM_001033113	580	980	518	815
ENTPD8__NM_001033113_CGGCTTGGGGACGCTGGTCA	ENTPD8__NM_001033113	700	612	972	692
ENTPD8__NM_001033113_CTGTGCAGCATTAGAAGTGT	ENTPD8__NM_001033113	609	587	687	699
ENTPD8__NM_001033113_GAGCAACCTCATGCCAGCCG	ENTPD8__NM_001033113	853	794	938	898
ENTPD8__NM_001033113_GATCACTGTCAACTACGGCT	ENTPD8__NM_001033113	892	779	1268	1417
ENTPD8__NM_001033113_TCACTGTCAACTACGGCTTG	ENTPD8__NM_001033113	501	537	621	706
ENY2__NM_001193557_AACCAAAAGTTGATAGAAAC	ENY2__NM_001193557	104	135	145	138
ENY2__NM_001193557_CAGTTGAAGGCACACTGTAA	ENY2__NM_001193557	334	8	516	52
ENY2__NM_001193557_GTGGCTGAAATCACTCCAAA	ENY2__NM_001193557	144	6	143	28
EOGT__NM_001278689_ACATCAAGAGAAATCATGAC	EOGT__NM_001278689	510	575	588	862
EOGT__NM_001278689_AGTTTGCAGCTATGTCGACA	EOGT__NM_001278689	220	209	245	284
EOGT__NM_001278689_ATGCTGGCATAGTTATACAG	EOGT__NM_001278689	266	284	265	257
EOGT__NM_001278689_CCTGGAATGCTGTGAGTATT	EOGT__NM_001278689	685	960	755	910
EOGT__NM_001278689_CTGTCAGCCTAAGGAAACGG	EOGT__NM_001278689	158	366	232	268
EOGT__NM_001278689_GAAAAAGTCCTCCTTAAATC	EOGT__NM_001278689	242	212	228	347
EOGT__NM_001278689_GACCTTCAGACGTCAATGTA	EOGT__NM_001278689	568	635	703	729
EOGT__NM_001278689_GCAGCTATGTCGACATGGGA	EOGT__NM_001278689	649	675	752	1024
EOGT__NM_001278689_GCATGTGCTCTGTCAGCCTA	EOGT__NM_001278689	301	327	301	341
EOGT__NM_001278689_GCTCTGTCAGCCTAAGGAAA	EOGT__NM_001278689	263	247	210	227
EOGT__NM_001278689_GGAATGCTGTGAGTATTAGG	EOGT__NM_001278689	554	752	608	631
EOGT__NM_001278689_TGCCAGCATCCGCTTGCCAG	EOGT__NM_001278689	207	99	206	207
EOMES__NM_001278182_AGCCGGGTACACAGGTCCGT	EOMES__NM_001278182	1736	1375	2015	1513
EOMES__NM_001278182_CCGCTCTACGGGCCGTACCC	EOMES__NM_001278182	199	132	210	235
EOMES__NM_001278182_GAGATGATCATTACGAAACA	EOMES__NM_001278182	574	607	576	604
EOMES__NM_001278182_GAGCCGGGTACACAGGTCCG	EOMES__NM_001278182	559	597	604	479
EOMES__NM_001278182_GCCACAGAGGCCGGTTGCAC	EOMES__NM_001278182	783	636	959	648
EOMES__NM_001278182_GCCAGGGGGCTCGCCTCTAC	EOMES__NM_001278182	937	549	921	693
EOMES__NM_001278182_GGACTGGAGGTAGTACCGCT	EOMES__NM_001278182	1392	1302	1679	1518
EOMES__NM_001278182_GGCGTCGGTGTCACTAAGCA	EOMES__NM_001278182	676	807	731	737
EOMES__NM_001278182_GGTGTCACTAAGCATGGCCG	EOMES__NM_001278182	32	42	89	62
EOMES__NM_001278182_TAGGGGTAGCGCGCCCCGTT	EOMES__NM_001278182	684	552	752	544
EOMES__NM_001278182_TCGTAATGATCATCTCAGTT	EOMES__NM_001278182	1102	1213	1383	1713
EOMES__NM_001278182_TGAGATGATCATTACGAAAC	EOMES__NM_001278182	201	174	228	148
EOMES__NM_001278183_AAAGCCGACAATAACATGCA	EOMES__NM_001278183	434	472	462	363
EOMES__NM_001278183_ATCCAGTGGGAACCAGTATT	EOMES__NM_001278183	686	553	642	591
EOMES__NM_001278183_CAAAGCCGACAATAACATGC	EOMES__NM_001278183	220	414	300	285
EOMES__NM_001278183_CACCCAGAGTCTCCTAATAC	EOMES__NM_001278183	1160	1068	1488	1284
EOMES__NM_001278183_CCACTACAATGTGTTCGTAG	EOMES__NM_001278183	722	532	857	645
EOMES__NM_001278183_CCCAACCACTGGCGCTTCCA	EOMES__NM_001278183	910	760	1040	937
EOMES__NM_001278183_CCCCTGGAAGCGCCAGTGGT	EOMES__NM_001278183	745	593	961	911
EOMES__NM_001278183_CCTCTACGAACACATTGTAG	EOMES__NM_001278183	688	411	680	436
EOMES__NM_001278183_CTACAATGTGTTCGTAGAGG	EOMES__NM_001278183	146	97	109	133
EOMES__NM_001278183_GAACCAGTATTAGGAGACTC	EOMES__NM_001278183	290	392	289	302
EOMES__NM_001278183_GCGCTTCCAGGGGGGCAAAT	EOMES__NM_001278183	278	440	330	331
EOMES__NM_001278183_TTCTTGAGCTTCAACATAAA	EOMES__NM_001278183	1	20	8	0
EP300__NM_001429_AACTCCCATATTGAGGTTAG	EP300__NM_001429	616	523	622	893
EP300__NM_001429_ACAGAATTGGGACTAACCAA	EP300__NM_001429	263	179	314	276
EP300__NM_001429_AGATGAGAGTTTAGGCCGCT	EP300__NM_001429	802	537	1014	1182
EP300__NM_001429_AGGGCGGAGATGAGAGTTT	EP300__NM_001429	482	323	518	389
EP300__NM_001429_AGGGTAAGAAACGATTCCGG	EP300__NM_001429	462	277	680	462
EP300__NM_001429_AGTTCCCCTAACCTCAATAT	EP300__NM_001429	861	752	752	625
EP300__NM_001429_AGTTTAGGCCGCTTGGCTGA	EP300__NM_001429	733	490	760	896
EP300__NM_001429_CAACTCCCATATTGAGGTTA	EP300__NM_001429	963	654	1166	1368
EP300__NM_001429_CAAGGCAGGAGCATATTGTT	EP300__NM_001429	562	460	690	611
EP300__NM_001429_CCAACTCCCATATTGAGGTT	EP300__NM_001429	1256	923	1577	1641
EP300__NM_001429_CCTAACCTCAATATGGGAGT	EP300__NM_001429	1009	713	1088	1375
EP300__NM_001429_CTGTCAGAATTGCTGCGATC	EP300__NM_001429	358	405	458	619
EP300__NM_001429_GGAGCACAAAAGTTGACTTG	EP300__NM_001429	541	388	728	812
EP300__NM_001429_GGGTAAGAAACGATTCCGGT	EP300__NM_001429	513	408	486	399
EP300__NM_001429_TAGTTCCCCTAACCTCAATA	EP300__NM_001429	110	59	93	152
EP300__NM_001429_TGCATGATTTACAACAGTTG	EP300__NM_001429	178	199	191	262
EP300__NM_001429_TGGTGGCCAGGTCAAGTCA	EP300__NM_001429	37	21	67	121
EP300__NM_001429_TTTAACTCTTCCATAAAGTG	EP300__NM_001429	25	19	33	20
EP400__NM_015409_AGAGCGTGGGCCCTGTCGTC	EP400__NM_015409	1210	884	1216	1259
EP400__NM_015409_AGCGTGGGCCCTGTCGTCGG	EP400__NM_015409	2114	1592	2195	1193
EP400__NM_015409_CACAAAGTGTCCACCACTGG	EP400__NM_015409	164	28	199	71
EP400__NM_015409_CAGCCTCGGCGGTTTGAGCA	EP400__NM_015409	501	345	519	480
EP400__NM_015409_CAGCTGCTGTATTTGATAAC	EP400__NM_015409	422	369	628	601
EP400__NM_015409_CGCTCCCTCAGCAAGCCCGT	EP400__NM_015409	886	611	1017	697
EP400__NM_015409_GACGTGACCTGAATGTATGA	EP400__NM_015409	590	257	662	207
EP400__NM_015409_GCCGAGGCTGAGCGCTGAAC	EP400__NM_015409	599	239	686	277
EP400__NM_015409_GCCTTGCAGAATGTGCGTGC	EP400__NM_015409	2804	1249	3520	1583
EP400__NM_015409_GCTGATGCTGGACGTTCTGG	EP400__NM_015409	933	755	1049	779
EP400__NM_015409_TGGGTCTCCATCATACATTC	EP400__NM_015409	45	0	24	18
EP400__NM_015409_TTGTGTTTCAGGATGGGTCA	EP400__NM_015409	50	69	69	55
EPAS1__NM_001430_ACGAATCTCCTCATGGTCGC	EPAS1__NM_001430	715	1047	776	1328
EPAS1__NM_001430_AGATGGGAGCTCACACTGTG	EPAS1__NM_001430	532	500	473	537
EPAS1__NM_001430_CAAGGCCTCCATCATGCGAC	EPAS1__NM_001430	245	396	380	461
EPAS1__NM_001430_CAAGTTCATGGGACTTACAC	EPAS1__NM_001430	503	481	473	599
EPAS1__NM_001430_CACGGCAATGAAACCCTCCA	EPAS1__NM_001430	1047	1054	997	1087
EPAS1__NM_001430_CACTCATCCCTGCGACCATG	EPAS1__NM_001430	1930	2326	2211	2376
EPAS1__NM_001430_CGAATCTCCTCATGGTCGCA	EPAS1__NM_001430	1114	1323	1260	1616
EPAS1__NM_001430_CTTCATGAGGATGAAGTGCA	EPAS1__NM_001430	264	292	277	328
EPAS1__NM_001430_GAAAGATCATGTCGCCATCT	EPAS1__NM_001430	1172	1122	1288	1826
EPAS1__NM_001430_GACGGAGGTGTTCTATGAGC	EPAS1__NM_001430	444	385	531	404
EPAS1__NM_001430_GAGAACCTGAGTCTCAAAAA	EPAS1__NM_001430	171	135	198	269
EPAS1__NM_001430_GCTGATTGCCAGTCGCATGA	EPAS1__NM_001430	877	1366	982	1256
EPB41__NM_001166005_AATGAATGAAGATCCAAAGA	EPB41__NM_001166005	121	108	168	179
EPB41__NM_001166005_AATGATCTCTTCATCAAGAC	EPB41__NM_001166005	183	186	133	201
EPB41__NM_001166005_AGTTAACAGAAGACATAACA	EPB41__NM_001166005	113	283	158	206
EPB41__NM_001166005_AGTTTATGAATGTGTTGTGG	EPB41__NM_001166005	256	576	598	609
EPB41__NM_001166005_ATGAATGAAGATCCAAAGAT	EPB41__NM_001166005	299	268	248	214
EPB41__NM_001166005_CAGCAGCTGAAGGAGATAAT	EPB41__NM_001166005	281	223	301	304
EPB41__NM_001166005_CTTCGGCAGGACATAGTTGC	EPB41__NM_001166005	321	564	293	400
EPB41__NM_001166005_GCACTGCAAGGTTTCTTTGT	EPB41__NM_001166005	2141	2136	2241	1983
EPB41__NM_001166005_GCCCAAATCTCAGGTGTCCG	EPB41__NM_001166005	1233	1551	1449	1641
EPB41__NM_001166005_TATTCTCCTCGTTTCTCAAA	EPB41__NM_001166005	167	508	174	352
EPB41__NM_001166005_TGTCTTCTGTTAACTGTGCT	EPB41__NM_001166005	105	108	187	212
EPB41__NM_001166005_TTGGGATAACGCAACCTCTA	EPB41__NM_001166005	148	188	199	178
EPB41__NM_001166007_AAGTTCCTTGGTCTGATTCG	EPB41__NM_001166007	267	173	283	234
EPB41__NM_001166007_AGTTGATCTTCATAAAGCAA	EPB41__NM_001166007	96	79	84	174
EPB41__NM_001166007_CAAGTTCCTTGGTCTGATTC	EPB41__NM_001166007	518	486	629	769
EPB41__NM_001166007_CGACCCAGAACTCCATGGCG	EPB41__NM_001166007	1536	1580	1745	1511
EPB41__NM_001166007_GTAAGAACCTAATAATGCTA	EPB41__NM_001166007	574	469	577	595
EPB41__NM_001166007_TAATAATGCTAAGGTTGCAA	EPB41__NM_001166007	298	355	358	616
EPB41L1__NM_001258329_CAGTAGGGCATCGTGACAA	EPB41L1__NM_001258329	575	555	505	500
EPB41L1__NM_001258329_CCAGCTCCGGGTCTGGTTA	EPB41L1__NM_001258329	971	1006	1162	905
EPB41L1__NM_001258329_CTCTTGGCTGACCGGTGGTT	EPB41L1__NM_001258329	99	67	122	135
EPB41L1__NM_001258329_CTTTGTCACGCATGCCCTAC	EPB41L1__NM_001258329	458	885	530	561
EPB41L1__NM_001258329_GCTTCGCCCCTAACCAGACC	EPB41L1__NM_001258329	7	0	10	10
EPB41L1__NM_001258329_GGGTCTGGTTAGGGGCGAAG	EPB41L1__NM_001258329	414	281	380	326
EPB41L1__NM_001258329_GTGCAGGTCTACTCCGTACA	EPB41L1__NM_001258329	1098	1113	1310	1149
EPB41L1__NM_001258329_TCTTGGCATTCTCTAAGAAG	EPB41L1__NM_001258329	194	225	214	202
EPB41L1__NM_001258329_TGCTCTCAAATTGCTCATAC	EPB41L1__NM_001258329	156	249	201	276
EPB41L1__NM_001258329_TGGAGCTGCATAAGACATAT	EPB41L1__NM_001258329	105	91	107	124
EPB41L1__NM_001258329_TGGCCCCGGCCATGTTTCTA	EPB41L1__NM_001258329	84	59	112	141
EPB41L1__NM_001258329_TTTGTCACGCATGCCCTACT	EPB41L1__NM_001258329	516	485	560	448
EPB41L2__NM_001135554_AACTGGGAATCAGCTTGTGC	EPB41L2__NM_001135554	687	477	793	759
EPB41L2__NM_001135554_AAGAAGGACTCTAGCCAGTT	EPB41L2__NM_001135554	415	326	476	432
EPB41L2__NM_001135554_ACACAGGAAGACAAATTAGA	EPB41L2__NM_001135554	520	405	506	414
EPB41L2__NM_001135554_ATATCTTCAGTCAATTGAGA	EPB41L2__NM_001135554	91	87	104	118
EPB41L2__NM_001135554_ATGTAGGTCAACACCATACA	EPB41L2__NM_001135554	390	273	380	272
EPB41L2__NM_001135554_CTAAGAGGGTCACTTTACAC	EPB41L2__NM_001135554	512	409	494	396
EPB41L2__NM_001135554_CTTCTCTGCTTTCAGCTCAT	EPB41L2__NM_001135554	194	213	220	248
EPB41L2__NM_001135554_GCATGAGTCACAAAAGAGCA	EPB41L2__NM_001135554	406	479	397	416
EPB41L2__NM_001135554_GCCTACTGAATTAGTAAGTA	EPB41L2__NM_001135554	392	465	482	453
EPB41L2__NM_001135554_TAGTCTGAGTAGGGGCAAAC	EPB41L2__NM_001135554	110	142	197	239
EPB41L2__NM_001135554_TCCTTACTTACTAATTCAGT	EPB41L2__NM_001135554	375	186	325	354
EPB41L2__NM_001135554_TGTAAAGTGACCCTCTTAGA	EPB41L2__NM_001135554	806	523	748	554
EPB41L3__NM_001281533_ACCAAACCACACTAAAGAAC	EPB41L3__NM_001281533	344	334	405	366
EPB41L3__NM_001281533_CAAATCTGTTTATTCGCAGC	EPB41L3__NM_001281533	732	966	805	1046
EPB41L3__NM_001281533_CATAGTCTCCGAGCTCTGAC	EPB41L3__NM_001281533	349	614	427	495
EPB41L3__NM_001281533_GAGATGACATCGTGTCCGGA	EPB41L3__NM_001281533	688	1124	908	806
EPB41L3__NM_001281533_GCCCTGCTCCTTTGTTACCC	EPB41L3__NM_001281533	1418	2259	1467	1678
EPB41L3__NM_001281533_GTCTGTTGATATATCGCGAC	EPB41L3__NM_001281533	345	315	489	383
EPB41L3__NM_001281533_TCAACCATGACGACCGAATC	EPB41L3__NM_001281533	1317	1640	1536	1917
EPB41L3__NM_001281533_TCCAGTTCTTTAGTGTGGTT	EPB41L3__NM_001281533	253	355	333	487
EPB41L3__NM_001281533_TGATCCAGATTCGGTCGTCA	EPB41L3__NM_001281533	467	489	496	549
EPB41L3__NM_001281533_TGCAAAGTGATACTTCTCGA	EPB41L3__NM_001281533	756	888	792	1055
EPB41L3__NM_001281533_TGCGAATAAACAGATTTGCC	EPB41L3__NM_001281533	179	171	200	245
EPB41L3__NM_001281533_TGTCTTCCAGTTCTTTAGTG	EPB41L3__NM_001281533	392	296	445	458
EPB41L3__NM_001281535_ATCGAGCTGCCAAGCGTTTA	EPB41L3__NM_001281535	626	927	694	1067
EPB41L3__NM_001281535_GAACAATTTGAAAGCACCAT	EPB41L3__NM_001281535	405	525	349	512
EPB41L4A__NM_022140_AAAACTGTCTCAAAAGTAAG	EPB41L4A__NM_022140	32	50	76	87
EPB41L4A__NM_022140_AACACAGTGATTGATATTAG	EPB41L4A__NM_022140	251	244	360	264
EPB41L4A__NM_022140_AAGGACTCAGTTCTAAAACA	EPB41L4A__NM_022140	206	230	243	262
EPB41L4A__NM_022140_AAGTGAACCTTTGTAATCCG	EPB41L4A__NM_022140	618	317	535	542
EPB41L4A__NM_022140_AATGACATATACAGAGGCTT	EPB41L4A__NM_022140	189	180	169	225
EPB41L4A__NM_022140_AGAGAACTGTACGTGGTAAG	EPB41L4A__NM_022140	719	720	747	696
EPB41L4A__NM_022140_AGAGGAAAATCTTAGTTGCT	EPB41L4A__NM_022140	376	360	438	499
EPB41L4A__NM_022140_AGATGACTTTGCAATCTACA	EPB41L4A__NM_022140	228	304	406	425
EPB41L4A__NM_022140_AGCAGCAGTGTTGACGGGAC	EPB41L4A__NM_022140	397	521	433	540
EPB41L4A__NM_022140_AGCTGGACACCTAGCAGTTA	EPB41L4A__NM_022140	628	669	699	661
EPB41L4A__NM_022140_AGGCTGAGCTGAATTACTTG	EPB41L4A__NM_022140	647	787	638	778
EPB41L4A__NM_022140_ATAATGACCCTAGATTCAGC	EPB41L4A__NM_022140	323	208	242	259
EPB41L4A__NM_022140_ATTCCATCACGTAAACCTTG	EPB41L4A__NM_022140	37	25	46	121
EPB41L4A__NM_022140_CACCGCAGCCTCCACTTACT	EPB41L4A__NM_022140	3167	2679	3289	2633
EPB41L4A__NM_022140_CACTTGTACTATTGCACAGC	EPB41L4A__NM_022140	380	270	464	446
EPB41L4A__NM_022140_CCAAAATACAAAGTATATGG	EPB41L4A__NM_022140	554	597	579	369
EPB41L4A__NM_022140_CCACTATTACCACGTCTAGT	EPB41L4A__NM_022140	613	501	654	570
EPB41L4A__NM_022140_CCTTTGGAAACATAGATAAG	EPB41L4A__NM_022140	141	165	166	127
EPB41L4A__NM_022140_CGTGATGGAATACGTGGTCA	EPB41L4A__NM_022140	894	1132	1164	1195
EPB41L4A__NM_022140_CTCAAAAGTAAGTGGACTAA	EPB41L4A__NM_022140	308	309	407	442
EPB41L4A__NM_022140_CTGCTCCCCAGCTGAATCTA	EPB41L4A__NM_022140	183	201	194	171
EPB41L4A__NM_022140_CTGCTGTGCAATAGTACAAG	EPB41L4A__NM_022140	280	191	336	330
EPB41L4A__NM_022140_CTGGGTGGTAAGGGTTAACT	EPB41L4A__NM_022140	189	182	209	191
EPB41L4A__NM_022140_GAATCGCTTAACCCAGTAAG	EPB41L4A__NM_022140	220	145	158	175
EPB41L4A__NM_022140_GACAAAAAGGTTAGGGCTGC	EPB41L4A__NM_022140	199	221	182	163
EPB41L4A__NM_022140_GAGAAGAGCATCCAAAGCAC	EPB41L4A__NM_022140	321	237	281	304
EPB41L4A__NM_022140_GAGAGAACTGTACGTGGTAA	EPB41L4A__NM_022140	1714	1407	1749	1540
EPB41L4A__NM_022140_GATTACAAAGGTTCACTTCA	EPB41L4A__NM_022140	301	431	303	403
EPB41L4A__NM_022140_GGAAAATCTTAGTTGCTTGG	EPB41L4A__NM_022140	621	722	657	928
EPB41L4A__NM_022140_GGAGAGAACTGTACGTGGTA	EPB41L4A__NM_022140	564	854	604	668
EPB41L4A__NM_022140_GTAGCGGTGCTTATAACGTA	EPB41L4A__NM_022140	69	89	72	44
EPB41L4A__NM_022140_GTTAGAGATAGCTTAAGATA	EPB41L4A__NM_022140	143	36	114	108
EPB41L4A__NM_022140_TATGAGATGAGTTCCGTGAT	EPB41L4A__NM_022140	681	719	532	697
EPB41L4A__NM_022140_TCAATTTGAACTCAGAGTAC	EPB41L4A__NM_022140	164	248	204	245
EPB41L4A__NM_022140_TCTCCACAAGGTTTACGTGA	EPB41L4A__NM_022140	62	44	127	118
EPB41L4A__NM_022140_TGAGAAAGAGTTGAGACTAA	EPB41L4A__NM_022140	239	215	251	225
EPB41L4A__NM_022140_TGATGAAGGGTCAAGGGTAG	EPB41L4A__NM_022140	131	86	144	115
EPB41L4A__NM_022140_TGCTGTGCAATAGTACAAGT	EPB41L4A__NM_022140	583	567	599	554
EPB41L4A__NM_022140_TGGAAACATAGATAAGTGGA	EPB41L4A__NM_022140	36	13	52	100
EPB41L4A__NM_022140_TGTAAGATGGCTATATGGG	EPB41L4A__NM_022140	373	519	418	326
EPB41L4A__NM_022140_TGTTAGAGATAGCTTAAGAT	EPB41L4A__NM_022140	45	66	91	61
EPB41L4A__NM_022140_TTCTTAGGATTAACTCCGGT	EPB41L4A__NM_022140	0	0	1	0
EPB41L4A__NM_022140_TTGCAAAGTCATCTATGGAG	EPB41L4A__NM_022140	385	357	316	307
EPB41L4A__NM_022140_TTTACCTTGCCATTAATTAG	EPB41L4A__NM_022140	213	175	236	312
EPB41L4B__NM_018424_ACCTTCGTGAGGAGTTTACA	EPB41L4B__NM_018424	537	506	577	639
EPB41L4B__NM_018424_CAAAGATTAATATGCCTGTC	EPB41L4B__NM_018424	602	692	609	701
EPB41L4B__NM_018424_CGAAACTCAGACACAAGCTC	EPB41L4B__NM_018424	19	5	8	25
EPB41L4B__NM_018424_GCAGGAAGACGCGGCAGTAG	EPB41L4B__NM_018424	710	614	742	492
EPB41L4B__NM_018424_GCCGCGTCTTCCTGCTCGAC	EPB41L4B__NM_018424	1895	1724	2187	1627
EPB41L4B__NM_018424_GGGTAGACATGCACGTTGTC	EPB41L4B__NM_018424	313	367	313	431
EPB41L4B__NM_018424_GGTAGACATGCACGTTGTCA	EPB41L4B__NM_018424	683	642	735	672
EPB41L4B__NM_018424_TCACTTCGGTCCCGTCGAGC	EPB41L4B__NM_018424	327	317	433	364
EPB41L4B__NM_018424_TCTCTTGGACTGACCCCGAC	EPB41L4B__NM_018424	613	749	651	893
EPB41L4B__NM_018424_TCTGAATAAAGCGAAGTGGC	EPB41L4B__NM_018424	327	453	319	408
EPB41L4B__NM_018424_TGATCAGATTGTGTACCACT	EPB41L4B__NM_018424	510	697	584	738
EPB41L4B__NM_018424_TTAGCTGCTCTCTGTCTACA	EPB41L4B__NM_018424	323	100	376	206
EPB41L5__NM_001184937_AATTGCACTGCTGTATCAAA	EPB41L5__NM_001184937	259	179	260	236
EPB41L5__NM_001184937_ACCGACGCCCTAGTGTTCTA	EPB41L5__NM_001184937	1198	924	1299	1085
EPB41L5__NM_001184937_ACCGGGTTAGCTGCTCCTCACGA	EPB41L5__NM_001184937	415	430	595	578
EPB41L5__NM_001184937_AGAACCAAATAACCTTCGTG	EPB41L5__NM_001184937	423	273	516	340
EPB41L5__NM_001184937_AGCTCCTCACGAAGGTTATT	EPB41L5__NM_001184937	327	344	474	420
EPB41L5__NM_001184937_AGTTTCTTCCGTAGAACACT	EPB41L5__NM_001184937	89	51	70	86
EPB41L5__NM_001184937_CAAAATAGTCGCTTTCAATC	EPB41L5__NM_001184937	659	1146	819	1025
EPB41L5__NM_001184937_GGACACCCGACACGTGATGA	EPB41L5__NM_001184937	854	1185	838	1005
EPB41L5__NM_001184937_GGATGGTACTGATGTTAGTG	EPB41L5__NM_001184937	324	305	323	383
EPB41L5__NM_001184937_TAAGTCCATCATCACGTGTC	EPB41L5__NM_001184937	290	392	341	274
EPB41L5__NM_001184937_TCCGTAGAACACTAGGGCGT	EPB41L5__NM_001184937	305	246	359	240
EPB41L5__NM_001184937_TGATCAGATTATGTACCACC	EPB41L5__NM_001184937	422	477	482	575
EPB42__NM_000119_AGCCAAGCCAGCGCTCGACA	EPB42__NM_000119	469	715	561	716
EPB42__NM_000119_CAGCAAGCCCTGTCGAGCGC	EPB42__NM_000119	585	741	618	666
EPB42__NM_000119_CAGCGCTCGACAGGGCTTGC	EPB42__NM_000119	646	960	711	801
EPB42__NM_000119_CCAGAATGGTCTCATCTACC	EPB42__NM_000119	995	1006	1040	889
EPB42__NM_000119_CTAACCAGTTTGTGCAGTGA	EPB42__NM_000119	739	682	929	833
EPB42__NM_000119_GAGCCAAGCCAGCGCTCGAC	EPB42__NM_000119	883	967	955	1259
EPB42__NM_000119_GGACTGGAGCGCGGAAGTAC	EPB42__NM_000119	519	439	584	334
EPB42__NM_000119_GGCCCGTGTGTTGGGTGCCT	EPB42__NM_000119	1075	1672	983	1071
EPB42__NM_000119_GTCACAGCTCTTGATACCCA	EPB42__NM_000119	167	92	161	144
EPB42__NM_000119_GTCTGGGGGACCGAAAGTGG	EPB42__NM_000119	105	161	103	59
EPB42__NM_000119_TACCAAGGCACCCAACACAC	EPB42__NM_000119	375	460	384	378
EPB42__NM_000119_TGAAAACAGGTGATGCTGCG	EPB42__NM_000119	171	189	194	362
EPB42__NM_001114134_AGTCCTGGGGTCCTGTGATC	EPB42__NM_001114134	850	550	681	493
EPB42__NM_001114134_CACCTCCATTAGGAGCACTT	EPB42__NM_001114134	873	900	794	1020
EPB42__NM_001114134_CCAAGTGCTCCTAATGGAGG	EPB42__NM_001114134	273	385	301	326
EPB42__NM_001114134_CTGCACCCAAGTGCTCCTAA	EPB42__NM_001114134	167	170	184	189
EPB42__NM_001114134_TTGGCTGCTGTTGCTTGCAC	EPB42__NM_001114134	348	343	324	443
EPC1__NM_001272004_AAAAGAGAAAAAACTGTCGA	EPC1__NM_001272004	202	164	280	320
EPC1__NM_001272004_ACGAATACGCCTCGATAAAC	EPC1__NM_001272004	255	264	263	399
EPC1__NM_001272004_ATGATTGACCGCCTAGAAAA	EPC1__NM_001272004	41	17	73	52
EPC1__NM_001272004_CGAATACGCCTCGATAAACA	EPC1__NM_001272004	249	374	324	349
EPC1__NM_001272004_CTATGAGTCTATATATCCTG	EPC1__NM_001272004	402	254	557	377
EPC1__NM_001272004_CTCTTCCTTCTCCATTCCGG	EPC1__NM_001272004	325	189	380	291
EPC1__NM_001272004_GAGGGATAATATGATTATAC	EPC1__NM_001272004	89	103	138	112
EPC1__NM_001272004_GTAAACTGTCGTTTCGGGCG	EPC1__NM_001272004	118	222	176	160
EPC1__NM_001272004_TACTATGAGTCTATATATCC	EPC1__NM_001272004	97	112	140	142
EPC1__NM_001272004_TCAGAATCCAAATCATAATC	EPC1__NM_001272004	520	438	636	790
EPC1__NM_001272004_TCTTCCTTCTCCATTCCGGT	EPC1__NM_001272004	661	465	687	622
EPC1__NM_001272004_TTATCGAGGCGTATTCGTGC	EPC1__NM_001272004	109	66	155	110
EPC1__NM_001272019_AGAGATCATGTCTGAGGTTA	EPC1__NM_001272019	239	248	325	249
EPC1__NM_001272019_AGAGCTATTGCACTTAACAC	EPC1__NM_001272019	148	31	140	89
EPC1__NM_001272019_ATGATGGGGATGGCATAAGT	EPC1__NM_001272019	428	199	414	300
EPC1__NM_001272019_CAGCACAAATGATCCTTATG	EPC1__NM_001272019	1598	1584	1800	1438
EPC1__NM_001272019_CGCCTTCTAAAAGCCACATA	EPC1__NM_001272019	385	346	431	339
EPC1__NM_001272019_CTATTAGTAATAGGGATGAT	EPC1__NM_001272019	253	208	350	303
EPC1__NM_001272019_TAGCAGTCAATTTAAACACC	EPC1__NM_001272019	229	236	332	441
EPC2__NM_015630_AAAAGTCTGACTGTAAGCGT	EPC2__NM_015630	400	558	423	485
EPC2__NM_015630_AAGCCGCTGCCTATCTACCG	EPC2__NM_015630	1572	1313	1455	1039
EPC2__NM_015630_ACTTATCAAGAACAAGTAAC	EPC2__NM_015630	316	417	354	433
EPC2__NM_015630_AGAAGGAGGAGGAATCGGTA	EPC2__NM_015630	492	307	545	451
EPC2__NM_015630_ATGTCCTTGCCGCGGTAGAT	EPC2__NM_015630	468	357	503	294
EPC2__NM_015630_CACATAAAAGATACTTTACC	EPC2__NM_015630	536	628	467	419
EPC2__NM_015630_CTACAAGGCAAGTAACGGGT	EPC2__NM_015630	590	681	752	813
EPC2__NM_015630_GAGAAGGAGGAGGAATCGGT	EPC2__NM_015630	423	189	484	336
EPC2__NM_015630_GAGACGCAGTCGTTGAGATC	EPC2__NM_015630	339	175	383	272
EPC2__NM_015630_GATATAATGCAATACATACC	EPC2__NM_015630	384	387	370	285
EPC2__NM_015630_TAAAGCTGTATATGACTACT	EPC2__NM_015630	352	285	402	302
EPC2__NM_015630_TATTACAATCGCTTGTACAA	EPC2__NM_015630	221	131	253	160
EPC2__NM_015630_TCCTCCTTCTCCATCCCGGT	EPC2__NM_015630	467	271	656	331
EPC2__NM_015630_TCTCCATCCCGGTGGGCATC	EPC2__NM_015630	383	370	339	300
EPC2__NM_015630_TCTGAATCCATATCATAATC	EPC2__NM_015630	263	252	242	207
EPC2__NM_015630_TGAAAAAGCCAGTTCTAATC	EPC2__NM_015630	347	269	368	463
EPC2__NM_015630_TGTAAATTAGATACGTGTAG	EPC2__NM_015630	173	125	189	301
EPC2__NM_015630_TGTTTCACAATTTGACTAGA	EPC2__NM_015630	8	5	16	13
EPC2__NM_015630_TTAAAGCTGTATATGACTAC	EPC2__NM_015630	154	129	158	156
EPCAM__NM_002354_AAAATGGACCTGACAGTAAA	EPCAM__NM_002354	228	167	310	283
EPCAM__NM_002354_AAATGAATGGCTCAAAACTT	EPCAM__NM_002354	225	239	247	348
EPCAM__NM_002354_AGGAGATGGGTGAGATGCAT	EPCAM__NM_002354	281	246	379	364
EPCAM__NM_002354_CAATGCCAGTGTACTTCAGT	EPCAM__NM_002354	1623	1110	1794	1853
EPCAM__NM_002354_CCCCCGCAGGTCCTCGCGTT	EPCAM__NM_002354	3362	3828	4325	4583
EPCAM__NM_002354_GATCACAACGCGTTATCAAC	EPCAM__NM_002354	866	1019	987	1159
EPCAM__NM_002354_GATCCTGACTGCGATGAGAG	EPCAM__NM_002354	487	550	507	701
EPCAM__NM_002354_TGCCTTCATCACCAAACATT	EPCAM__NM_002354	158	122	221	145
EPDR1__NM_001242946_GCGCCCTGCTCTCCTACGAC	EPDR1__NM_001242946	1358	2228	1389	1840
EPDR1__NM_001242946_TACTTTGCTGGTACATAACC	EPDR1__NM_001242946	475	530	413	403
EPDR1__NM_001242948_AAAGTCAGCTAGATCCTGTA	EPDR1__NM_001242948	105	76	87	63
EPDR1__NM_001242948_AGGGGTCTTTAATACCCAGC	EPDR1__NM_001242948	98	55	69	64
EPDR1__NM_001242948_CACACTGTAGTTTATGGTAA	EPDR1__NM_001242948	978	1367	1202	1759
EPDR1__NM_001242948_CATCTGGCACGTGCTTGGAG	EPDR1__NM_001242948	706	601	804	602
EPDR1__NM_001242948_CCATCTGGCACGTGCTTGGA	EPDR1__NM_001242948	837	1040	777	1056
EPDR1__NM_001242948_CCCTCCAAGCACGTGCCAGA	EPDR1__NM_001242948	1748	2402	2237	2944
EPDR1__NM_001242948_GATTGGCATCTATACAGTCA	EPDR1__NM_001242948	352	517	360	489
EPDR1__NM_001242948_GCCATCTGGCACGTGCTTGG	EPDR1__NM_001242948	1203	1906	1293	1458
EPDR1__NM_001242948_TCAGGAGCAGATCACCGTCC	EPDR1__NM_001242948	946	1083	1329	1298
EPDR1__NM_001242948_TCTTCTCCAGTTGGGCATCC	EPDR1__NM_001242948	329	450	228	358
EPDR1__NM_001242948_TTGGAGGGGTAAACACCGAG	EPDR1__NM_001242948	195	435	277	324
EPG5__NM_020964_AGTCGCTCACTGCGAAGCAA	EPG5__NM_020964	6	6	15	61
EPG5__NM_020964_CACAGAGCCCCCAAAGGAAG	EPG5__NM_020964	117	27	95	102
EPG5__NM_020964_CCCAGAACTCCCGTCTCAAC	EPG5__NM_020964	1578	1346	1805	1190
EPG5__NM_020964_CTCTTCATTGCTTATAGTTA	EPG5__NM_020964	394	443	457	595
EPG5__NM_020964_GAAAATATGTCGGTACAAGG	EPG5__NM_020964	86	35	141	90
EPG5__NM_020964_GAAGTATGAAACTCCTCAGA	EPG5__NM_020964	69	33	99	29
EPG5__NM_020964_GAGGAAAATATGTCGGTACA	EPG5__NM_020964	364	319	416	302
EPG5__NM_020964_GAGTCGCTCACTGCGAAGCA	EPG5__NM_020964	26	8	11	28
EPG5__NM_020964_GGCCAGCCGGACTAAAACAA	EPG5__NM_020964	151	107	127	106
EPG5__NM_020964_GGGCTCTGTGTTACACGTCA	EPG5__NM_020964	373	438	330	411
EPG5__NM_020964_GGGGCTCTGTGTTACACGTC	EPG5__NM_020964	297	476	308	465
EPG5__NM_020964_TTGAATTCACAGGCTAGAGA	EPG5__NM_020964	70	68	101	123
EPGN__NM_001270989_AATGACAGCACTGACCGAAG	EPGN__NM_001270989	38	45	29	45
EPGN__NM_001270989_ATTGCAATTGGGATTGGTGT	EPGN__NM_001270989	885	985	998	969
EPGN__NM_001270989_GGTGTTGGATTACTATTAAG	EPGN__NM_001270989	240	288	315	265
EPGN__NM_001270989_TAACTGTCCAGTTACCTTGC	EPGN__NM_001270989	101	51	98	108
EPGN__NM_001270989_TCACAGCCAGCAAGGTAAC	EPGN__NM_001270989	5	26	2	6
EPGN__NM_001270989_TGGCTTCTCTAGCTCATGG	EPGN__NM_001270989	0	0	0	0
EPHA1__NM_005232_ACCACTGGCTTCGCTCCAAT	EPHA1__NM_005232	900	1260	1152	1541
EPHA1__NM_005232_ACCATTCGAGACCTTGTGTC	EPHA1__NM_005232	670	1070	951	1233
EPHA1__NM_005232_CAGTCCTGGTACATGTACAG	EPHA1__NM_005232	591	647	572	643
EPHA1__NM_005232_CCTTCTGGAACAAGGGCCGT	EPHA1__NM_005232	596	707	694	570
EPHA1__NM_005232_CGCTCCAATTGGATCTACCG	EPHA1__NM_005232	955	1252	1029	1417
EPHA1__NM_005232_CTCCAATTGGATCTACCGCG	EPHA1__NM_005232	420	296	431	328
EPHA1__NM_005232_CTCCCCGCCCGGTAGATCAAT	EPHA1__NM_005232	675	670	693	724
EPHA1__NM_005232_GCTCCAATTGGATCTACCGC	EPHA1__NM_005232	507	510	601	448
EPHA1__NM_005232_GTGTCTCTGCGTCCTTGCAT	EPHA1__NM_005232	447	463	448	458
EPHA1__NM_005232_TACCAGGACTGCCCAATGCA	EPHA1__NM_005232	199	165	186	132
EPHA1__NM_005232_TGCAGCCACCGTGGTTACCT	EPHA1__NM_005232	789	1098	946	1002
EPHA1__NM_005232_TGTCTCTGCGTCCTTGCATT	EPHA1__NM_005232	397	475	444	488
EPHA10__NM_001099439_AACACAGAGGTGCGCGAGAT	EPHA10__NM_001099439	1019	939	1031	1396
EPHA10__NM_001099439_ACGTGCGTGGCGCACTCGGA	EPHA10__NM_001099439	319	236	389	393
EPHA10__NM_001099439_CAAAATCGACACGATCGCGG	EPHA10__NM_001099439	3354	2856	3890	3470
EPHA10__NM_001099439_CACCTGGCCTTTCAGGACGT	EPHA10__NM_001099439	1302	1191	1581	973
EPHA10__NM_001099439_CCGCAAAATCGACACGATCG	EPHA10__NM_001099439	1020	1269	1106	1669
EPHA10__NM_001099439_CGAGTGCGCCACGCACGTTC	EPHA10__NM_001099439	385	280	437	397
EPHA10__NM_001099439_CGGTGGCTGGGAACGTGGCC	EPHA10__NM_001099439	555	765	657	878
EPHA10__NM_001099439_GCGGACGAGAGCTTCACGCA	EPHA10__NM_001099439	534	572	508	468
EPHA10__NM_001099439_GGAATCCAGGAGGATAACTA	EPHA10__NM_001099439	151	206	197	259
EPHA10__NM_001099439_GGACTGCACTGCCAAGTAAT	EPHA10__NM_001099439	430	470	431	287
EPHA10__NM_001099439_TGGACTGCACTGCCAAGTAA	EPHA10__NM_001099439	119	60	171	82
EPHA10__NM_001099439_TTCCAGGTAGTAGACGTTGA	EPHA10__NM_001099439	5	6	17	6
EPHA2__NM_004431_AACGTGGAGGAGCGCTCCGT	EPHA2__NM_004431	700	741	957	874
EPHA2__NM_004431_ACAACTGGCTCCGCACCAAC	EPHA2__NM_004431	388	645	432	537
EPHA2__NM_004431_ATCCACTGCACAGTGCATAC	EPHA2__NM_004431	619	556	641	606
EPHA2__NM_004431_CAACGTGATGTCTGGCGACC	EPHA2__NM_004431	666	602	669	886
EPHA2__NM_004431_CAACTGGGTGTACCGAGGAG	EPHA2__NM_004431	584	574	596	676
EPHA2__NM_004431_CACCGTCAGCAGCGACTTCG	EPHA2__NM_004431	882	812	1068	1344
EPHA2__NM_004431_CACCTGTGTGGACATCGCCG	EPHA2__NM_004431	1681	1883	1616	1981
EPHA2__NM_004431_CTACTATGCCGAGTCGGACC	EPHA2__NM_004431	334	429	389	515
EPHA2__NM_004431_GGCTGGCTCACACACCCGTA	EPHA2__NM_004431	748	979	887	1216
EPHA2__NM_004431_GTGGGGCCGCTCACCCGCAA	EPHA2__NM_004431	340	365	331	282
EPHA2__NM_004431_TCACACACCCGTATGGCAAA	EPHA2__NM_004431	355	462	352	478
EPHA2__NM_004431_TCCGTGTGCAACGTGATGTC	EPHA2__NM_004431	825	1187	760	1138
EPHA3__NM_005233_ACAATTGGCTGAGAACAAAC	EPHA3__NM_005233	236	211	226	196
EPHA3__NM_005233_AGACTGCAATAGCATTCCAT	EPHA3__NM_005233	561	815	690	832
EPHA3__NM_005233_ATGAACATTACACACCCATC	EPHA3__NM_005233	260	225	283	262
EPHA3__NM_005233_CACACCCATCAGGACTTACC	EPHA3__NM_005233	710	561	929	992
EPHA3__NM_005233_CAGTCCCTGGTGGAGGTTAG	EPHA3__NM_005233	260	494	340	322
EPHA3__NM_005233_GAGTCCATGGGTACCGTGTC	EPHA3__NM_005233	611	821	664	775
EPHA3__NM_005233_GCACACCTGGTAAGTCCTGA	EPHA3__NM_005233	1414	1527	1809	2349
EPHA3__NM_005233_GGATCTCTTATCCATCACAT	EPHA3__NM_005233	106	90	131	89
EPHA3__NM_005233_TGATGAAAGTTTCACTCAAA	EPHA3__NM_005233	208	405	250	423
EPHA3__NM_005233_TGTTGGTGCTTGTGTTGCCT	EPHA3__NM_005233	470	574	559	747
EPHA3__NM_005233_TTACCAGGTGTGCAATGTCA	EPHA3__NM_005233	116	39	102	130
EPHA3__NM_005233_TTGTGTCAACAATTCTAAGG	EPHA3__NM_005233	171	44	159	112
EPHA4__NM_004438_AACAGATCTGGAATCCAATA	EPHA4__NM_004438	53	107	105	125
EPHA4__NM_004438_ACTGATTGGATCACCCGAGA	EPHA4__NM_004438	560	366	631	524
EPHA4__NM_004438_AGGGTATACCCCGCGAATGA	EPHA4__NM_004438	317	406	325	293
EPHA4__NM_004438_ATAACTGGCTACGAACTGAT	EPHA4__NM_004438	454	508	558	556
EPHA4__NM_004438_CACACTTGGTAGGTTCGGAT	EPHA4__NM_004438	688	825	744	926
EPHA4__NM_004438_CAGTTCGTAGCCAGTTATTC	EPHA4__NM_004438	496	550	504	922
EPHA4__NM_004438_CCCGAAGAGACACGAAAATA	EPHA4__NM_004438	508	779	611	724
EPHA4__NM_004438_CGTAGCCAGTTATTCTGGCT	EPHA4__NM_004438	345	564	367	388
EPHA4__NM_004438_CTGATTGGATCACCCGAGAA	EPHA4__NM_004438	223	192	257	475
EPHA4__NM_004438_GTGGGAGGAAGTGAGTATCA	EPHA4__NM_004438	65	49	81	70
EPHA4__NM_004438_TCGTAGCCAGTTATTCTGGC	EPHA4__NM_004438	80	32	110	143
EPHA4__NM_004438_TGGAACCCAGCCAGAATAAC	EPHA4__NM_004438	72	97	51	47
EPHA5__NM_001281765_ACAGGAGAAGGCACGTCCAG	EPHA5__NM_001281765	439	393	449	282
EPHA5__NM_001281765_AGAACTCAAATTTACCCTGC	EPHA5__NM_001281765	395	592	608	809
EPHA5__NM_001281765_CAGCCCCAGCAACGAAGGTA	EPHA5__NM_001281765	182	232	202	351
EPHA5__NM_001281765_CATATTAAAGGTTTCCTTAC	EPHA5__NM_001281765	287	293	393	397
EPHA5__NM_001281765_CCAAGTGTCGTACCACAGAA	EPHA5__NM_001281765	240	267	253	433
EPHA5__NM_001281765_CTGCTACTCTGCACCTCGAC	EPHA5__NM_001281765	1107	1609	1072	1404
EPHA5__NM_001281765_CTGGCCAGCCCCAGCAACGA	EPHA5__NM_001281765	176	187	143	155
EPHA5__NM_001281765_TAGAACTCAAATTTACCCTG	EPHA5__NM_001281765	101	144	76	93
EPHA5__NM_001281765_TGGAGGACTGGGGACCTGTA	EPHA5__NM_001281765	169	129	225	195
EPHA5__NM_001281765_TGTAGGACCTCTAAGCAAAA	EPHA5__NM_001281765	45	46	44	33
EPHA5__NM_001281765_TGTTATGAAACTGAATACAG	EPHA5__NM_001281765	74	254	93	55
EPHA5__NM_001281765_TGTTGGTGCTGCATTGCTC	EPHA5__NM_001281765	0	0	0	0
EPHA6__NM_001080448_AACACTGAAATTCGTGAGGT	EPHA6__NM_001080448	462	338	580	808
EPHA6__NM_001080448_ACAACTGGCTTCGTACAAAC	EPHA6__NM_001080448	393	432	525	649
EPHA6__NM_001080448_ACATACCTGGTATGTGTGAA	EPHA6__NM_001080448	287	502	279	354
EPHA6__NM_001080448_AGGTGCAGCTGCTTCGGAGG	EPHA6__NM_001080448	1363	1975	1392	1371
EPHA6__NM_001080448_CAACACTGAAATTCGTGAGG	EPHA6__NM_001080448	723	907	853	995
EPHA6__NM_001080448_CTGTACTGGGAGAGCTAGGA	EPHA6__NM_001080448	326	374	429	468
EPHA6__NM_001080448_GCTTGATACAACAACTGTAC	EPHA6__NM_001080448	610	820	597	803
EPHA6__NM_001080448_GGAAATGAAATTCACACTAA	EPHA6__NM_001080448	376	374	552	761
EPHA6__NM_001080448_GTTCCATTACATTACATACC	EPHA6__NM_001080448	99	184	148	222
EPHA6__NM_001080448_TCCTCTCCCTTAAAGAGAAG	EPHA6__NM_001080448	39	27	71	48
EPHA6__NM_001080448_TGCAAGTCCCCAAGACCCAT	EPHA6__NM_001080448	776	1058	835	854
EPHA6__NM_001080448_TTGTGTCGATCTTGTATAC	EPHA6__NM_001080448	1148	1497	1244	1553
EPHA6__NM_001278300_AAACATCATTCGCCTAGAAG	EPHA6__NM_001278300	462	665	558	526
EPHA6__NM_001278300_AGGATCCTTCCCGGCCATTG	EPHA6__NM_001278300	691	942	820	627
EPHA6__NM_001278300_ATTCATGGACTGCTAGGGAT	EPHA6__NM_001278300	127	96	161	121
EPHA6__NM_001278300_ATTGAGAGAGTCATTGGGGC	EPHA6__NM_001278300	269	380	297	425
EPHA6__NM_001278300_CAAACATCATTCGCCTAGAA	EPHA6__NM_001278300	384	442	419	543
EPHA6__NM_001278300_CCAAACATCATTCGCCTAGA	EPHA6__NM_001278300	1256	1660	1516	1946
EPHA6__NM_001278300_CTCTCAATACGAATTCTTGA	EPHA6__NM_001278300	165	155	165	132
EPHA6__NM_001278300_CTCTTCTTTGCCGATCCATG	EPHA6__NM_001278300	202	291	314	369
EPHA6__NM_001278300_GAGAGTGAATCCGCCAACAG	EPHA6__NM_001278300	199	251	162	173
EPHA6__NM_001278300_GCCATTAAAACTTTGAAAGG	EPHA6__NM_001278300	285	450	295	314
EPHA6__NM_001278300_GTGATAGCCACCGCCGCTGT	EPHA6__NM_001278300	1299	1311	1432	1565
EPHA6__NM_001278300_TCTAAGAGAAGCTAGTATCA	EPHA6__NM_001278300	130	53	127	90
EPHA7__NM_001288629_AAACAGACTATGACACTGGC	EPHA7__NM_001288629	535	1048	587	665
EPHA7__NM_001288629_AAGACTGTTACAATCCCTCA	EPHA7__NM_001288629	208	215	321	316
EPHA7__NM_001288629_ACAACTGGCTGCGGACTAAC	EPHA7__NM_001288629	568	847	708	1065
EPHA7__NM_001288629_CACACCTGGTATGTTCGTAT	EPHA7__NM_001288629	1038	1203	1330	1261
EPHA7__NM_001288629_CACCTGGTATGTTCGTATCG	EPHA7__NM_001288629	664	626	813	786
EPHA7__NM_001288629_CGGACTAACTGGATTTCCAA	EPHA7__NM_001288629	478	713	510	616
EPHA7__NM_001288629_GTACCGAGTTTGAAAAACCA	EPHA7__NM_001288629	133	75	117	84
EPHA7__NM_001288629_TAAAGCACAACAAACAGAGT	EPHA7__NM_001288629	123	163	101	89
EPHA7__NM_001288629_TACCCCGATACGAACATCAC	EPHA7__NM_001288629	459	461	449	447
EPHA7__NM_001288629_TAGAATTGAAATTCACCCTG	EPHA7__NM_001288629	192	192	251	208
EPHA7__NM_001288629_TATGAAACAGACTATGACAC	EPHA7__NM_001288629	733	719	794	671
EPHA7__NM_001288629_TGGATTTCCTCTCCACCCAA	EPHA7__NM_001288629	14	22	27	0
EPHA8__NM_001006943_ACAACTGGCTGCGCACGAGC	EPHA8__NM_001006943	281	315	375	354
EPHA8__NM_001006943_AGCTTCACAGGTGCCGACCT	EPHA8__NM_001006943	125	159	205	219
EPHA8__NM_001006943_CAAACCTGGTACGTGTGGAT	EPHA8__NM_001006943	1404	1761	1586	2263
EPHA8__NM_001006943_CAACTGGCTGCGCACGAGCT	EPHA8__NM_001006943	464	547	679	704
EPHA8__NM_001006943_CCTCAAAATCGACACCATTG	EPHA8__NM_001006943	935	1058	1148	1184
EPHA8__NM_001006943_CTGGACACGTCGACCATCCA	EPHA8__NM_001006943	738	911	838	1086
EPHA8__NM_001006943_GAAGCTCTCGTCGGCCGCAA	EPHA8__NM_001006943	551	547	647	561
EPHA8__NM_001006943_GCAAACCTGGTACGTGTGGA	EPHA8__NM_001006943	3096	4004	3036	4280
EPHA8__NM_001006943_GCTCACGTATCCGGCTCATG	EPHA8__NM_001006943	1478	1710	1507	1423
EPHA8__NM_001006943_GTGTGCTGGCCCCCAGGTCG	EPHA8__NM_001006943	784	771	822	964
EPHA8__NM_001006943_TGATCTCAGCATAGACGCGC	EPHA8__NM_001006943	0	0	0	0
EPHA8__NM_001006943_TGGCTCACGTATCCGGCTCA	EPHA8__NM_001006943	56	79	31	30
EPHB1__NM_004441_AACTCTACTGCAACGGGGAT	EPHB1__NM_004441	602	532	584	717
EPHB1__NM_004441_AATGGTGTCTACTTTGAGGT	EPHB1__NM_004441	482	481	559	581
EPHB1__NM_004441_ACCACCTTCATCAACCGGCG	EPHB1__NM_004441	590	625	700	601
EPHB1__NM_004441_CCGTTGCAGTAGAGTTTGAT	EPHB1__NM_004441	954	1045	935	1240
EPHB1__NM_004441_CTCCATAATCCTGAAAAGCG	EPHB1__NM_004441	111	205	179	251
EPHB1__NM_004441_GAGCAGCCAATTGTTCTGGT	EPHB1__NM_004441	1077	1095	1174	1455
EPHB1__NM_004441_GAGCAGTAGTAGATAATCCA	EPHB1__NM_004441	225	263	274	185
EPHB1__NM_004441_GGAGCAGTAGTAGATAATCC	EPHB1__NM_004441	204	205	283	270
EPHB1__NM_004441_GGCCCCCCGCCGGTTGATGA	EPHB1__NM_004441	947	877	1172	1589
EPHB1__NM_004441_GGGGAATGGATGGTGCCTAT	EPHB1__NM_004441	591	829	722	841
EPHB1__NM_004441_GGTGTCTACTTTGAGGTAGG	EPHB1__NM_004441	149	214	174	155
EPHB1__NM_004441_TGGACGGCCAATCCTGCGTC	EPHB1__NM_004441	307	275	406	417
EPHB2__NM_004442_AAAACATGCAACTCAAACGA	EPHB2__NM_004442	660	685	769	752
EPHB2__NM_004442_AACACCGAGGTGCGGAGCTT	EPHB2__NM_004442	237	420	270	288
EPHB2__NM_004442_ACGCCCTCAGTCTAGGGATC	EPHB2__NM_004442	736	694	818	840
EPHB2__NM_004442_ACTGATGTAAATGCGTGGCT	EPHB2__NM_004442	418	528	477	590
EPHB2__NM_004442_ATTCATGCGATGTGTCCAAT	EPHB2__NM_004442	308	334	362	288
EPHB2__NM_004442_CAAGCCAGAACAACTGGCTA	EPHB2__NM_004442	256	337	274	271
EPHB2__NM_004442_CCAGCCCAATGGCGTGATCC	EPHB2__NM_004442	1726	2240	1851	1881
EPHB2__NM_004442_CCCATCAAGCTCTACTGTAA	EPHB2__NM_004442	218	133	304	228
EPHB2__NM_004442_CTGACTTAGGCAATACACCA	EPHB2__NM_004442	499	368	397	409
EPHB2__NM_004442_CTGTAAACAAACCCAGATGC	EPHB2__NM_004442	610	793	616	433
EPHB2__NM_004442_CTTCAACCTCTATTACTATG	EPHB2__NM_004442	441	696	544	800
EPHB2__NM_004442_CTTCCAGGAAACCCTGTCGG	EPHB2__NM_004442	91	70	102	57
EPHB2__NM_004442_GAGAATGGCACCGTCTGCCG	EPHB2__NM_004442	817	1016	863	874
EPHB2__NM_004442_GGCTACGGACAAGTTTATC	EPHB2__NM_004442	293	245	343	444
EPHB2__NM_004442_GGGAGGGGGCGGGAAATACA	EPHB2__NM_004442	166	91	132	178
EPHB2__NM_004442_GGGCACTCAGGAGCCTCAAA	EPHB2__NM_004442	430	352	494	594
EPHB2__NM_004442_GGGTGTCTGTGTTGGTTCAC	EPHB2__NM_004442	293	266	318	430
EPHB2__NM_004442_GGTCCGGCTGGGACCACGAC	EPHB2__NM_004442	83	117	149	256
EPHB2__NM_004442_GTTTCTCCTGTTGGCTCACA	EPHB2__NM_004442	298	428	363	474
EPHB2__NM_004442_TCACTCAACAACCAAGCGCC	EPHB2__NM_004442	290	421	377	458
EPHB2__NM_004442_TCTTCCAGGAAACCCTGTCG	EPHB2__NM_004442	369	239	444	414
EPHB2__NM_004442_TCTTGGTGACGTCTCGTGGC	EPHB2__NM_004442	297	427	316	456
EPHB2__NM_004442_TGACTTAGGCAATACACCAA	EPHB2__NM_004442	195	456	242	250
EPHB2__NM_004442_TGGACAGCATTACCCTGTCG	EPHB2__NM_004442	231	311	264	306
EPHB3__NM_004443_AAAATGGGTAACATCTGAGT	EPHB3__NM_004443	228	101	215	242
EPHB3__NM_004443_AACACCAAGGTGCGCAGCTT	EPHB3__NM_004443	7	5	6	0
EPHB3__NM_004443_AGACCCTCATGGACACAAAA	EPHB3__NM_004443	160	170	191	200
EPHB3__NM_004443_AGCCGCGAGGAAGCTCTATC	EPHB3__NM_004443	212	132	286	209
EPHB3__NM_004443_CACACCTGGTATGTGCGGAT	EPHB3__NM_004443	1057	1169	1246	1257
EPHB3__NM_004443_CACCTTGGTGTTGACACGGC	EPHB3__NM_004443	964	764	990	1047
EPHB3__NM_004443_CTCTTCCCCGAGACCCTCAC	EPHB3__NM_004443	376	355	323	342
EPHB3__NM_004443_GACCCTCATGGACACAAAAT	EPHB3__NM_004443	395	610	444	473
EPHB3__NM_004443_GCACGGGGTTCATCTGGCGG	EPHB3__NM_004443	613	575	746	858
EPHB3__NM_004443_GGCTTCGCACGGGGTTCATC	EPHB3__NM_004443	153	179	142	192
EPHB3__NM_004443_GTATGTGCGGATGGGTTCA	EPHB3__NM_004443	358	323	416	446
EPHB3__NM_004443_TGGTGTCCACTTTCACGTAG	EPHB3__NM_004443	26	0	27	39
EPHB4__NM_004444_ACCACGCAGCTACCGGCCAC	EPHB4__NM_004444	1919	2012	2079	2448
EPHB4__NM_004444_AGCGCAGCGTGGCGTACACG	EPHB4__NM_004444	842	969	863	989
EPHB4__NM_004444_CAGAAACGGACGCCGGTGCT	EPHB4__NM_004444	1816	2232	2030	2648
EPHB4__NM_004444_CAGGCGGGGTCCGGGGATGT	EPHB4__NM_004444	1686	1435	1836	1617
EPHB4__NM_004444_CAGTCTCCGGGAATCGAGTC	EPHB4__NM_004444	730	864	888	1132
EPHB4__NM_004444_CCACTGGCTTCGCACAGGTT	EPHB4__NM_004444	1080	1493	1395	1801
EPHB4__NM_004444_CCCCAAGCTGTGTCCTATGA	EPHB4__NM_004444	1007	1679	1262	1761
EPHB4__NM_004444_CCCCAGCCTCTACTGCCGTG	EPHB4__NM_004444	2164	2560	2512	2687
EPHB4__NM_004444_CCTTCATAGGACACAGCTTG	EPHB4__NM_004444	1097	1365	1215	1561
EPHB4__NM_004444_CGTGGCCGGTAGCTGCGTGG	EPHB4__NM_004444	6587	6552	6802	5839
EPHB4__NM_004444_CTGCACGTCACACACTTCGT	EPHB4__NM_004444	658	658	612	506
EPHB4__NM_004444_CTGCGGGAACGCCACCATCA	EPHB4__NM_004444	41	139	53	31
EPHB4__NM_004444_CTGTTTCCAGAACAGTGCCT	EPHB4__NM_004444	354	556	355	507
EPHB4__NM_004444_GACCCTGCTGAACACAAAAT	EPHB4__NM_004444	487	634	602	867
EPHB4__NM_004444_GATGGAGAACCCCTACATCA	EPHB4__NM_004444	660	465	545	325
EPHB4__NM_004444_GATTGCACTTTGAGCCCGTG	EPHB4__NM_004444	1034	1371	1113	1139
EPHB4__NM_004444_GCGTCTGGGACCGCTCAGCA	EPHB4__NM_004444	560	880	652	938
EPHB4__NM_004444_GCTCAAAGTGCAATCCAGCG	EPHB4__NM_004444	616	1079	725	777
EPHB4__NM_004444_GGCCATCCTCACGGCAGTAG	EPHB4__NM_004444	757	1078	810	927
EPHB4__NM_004444_GGGAAAAAAATGATTGAGTT	EPHB4__NM_004444	591	702	665	854
EPHB4__NM_004444_GTAGTGAAAAGGGCGGTAGT	EPHB4__NM_004444	261	347	290	245
EPHB4__NM_004444_TCGGGAGCTGGTTGTGCCCG	EPHB4__NM_004444	1647	1564	1716	1894
EPHB4__NM_004444_TTTGGGGGTTCTGCCATAAT	EPHB4__NM_004444	179	206	161	184
EPHB6__NM_001280794_ACCCTACATCTTAGGAGTGC	EPHB6__NM_001280794	722	803	744	767
EPHB6__NM_001280794_ACTGAGAGCCGAGTGTTAGT	EPHB6__NM_001280794	1724	2092	1953	1767
EPHB6__NM_001280794_AGAGGTGCAGGCTGTTAATG	EPHB6__NM_001280794	616	476	643	494
EPHB6__NM_001280794_AGCCCCCGTTTGCCCCTGCC	EPHB6__NM_001280794	2481	4052	3141	3826
EPHB6__NM_001280794_CCTGCCTGGAGGGCTTCTAC	EPHB6__NM_001280794	611	560	751	915
EPHB6__NM_001280794_CCTGGATACCAACCAGCACG	EPHB6__NM_001280794	196	211	229	227
EPHB6__NM_001280794_CTGAGAGCCGAGTGTTAGTG	EPHB6__NM_001280794	494	418	515	790
EPHB6__NM_001280794_GACTGAGAGCCGAGTGTTAG	EPHB6__NM_001280794	625	1067	779	679
EPHB6__NM_001280794_GCCTGCACCTCTAAGATGTA	EPHB6__NM_001280794	1570	1909	1718	2019
EPHB6__NM_001280794_GCTCTTCAATGTCGTGTGCA	EPHB6__NM_001280794	290	408	428	540
EPHB6__NM_001280794_GGCCTCTGGTGGGTCGGAAC	EPHB6__NM_001280794	301	287	266	307
EPHB6__NM_001280794_TGAGAGCCGAGTGTTAGTGG	EPHB6__NM_001280794	714	634	741	628
EPHB6__NM_004445_AATCTCAGATGTCTCTCCGG	EPHB6__NM_004445	790	1000	1041	860
EPHB6__NM_004445_ACGCCTGACTCGGACCTTTG	EPHB6__NM_004445	1585	1925	1828	1899
EPHB6__NM_004445_ATGCCTCAAAGGTCCGAGTC	EPHB6__NM_004445	481	437	635	894
EPHB6__NM_004445_CCGCCACTCTGTTCCCTAAC	EPHB6__NM_004445	1023	1058	1091	1513
EPHB6__NM_004445_CGGGCATGGTGTGTAGCCTA	EPHB6__NM_004445	433	332	549	428
EPHB6__NM_004445_CTCAGCCTGACGGTAGTAAA	EPHB6__NM_004445	96	145	150	130
EPHB6__NM_004445_CTGAAGGGGCTGCCCAGTTA	EPHB6__NM_004445	469	479	546	541
EPHB6__NM_004445_CTTCACCCTTTACTACCGTC	EPHB6__NM_004445	451	428	581	462
EPHB6__NM_004445_GGCTGGCTCACCTACCCACC	EPHB6__NM_004445	939	1132	1034	1019
EPHB6__NM_004445_GGGCATGGTGTGTAGCCTAT	EPHB6__NM_004445	375	527	401	415
EPHB6__NM_004445_TGTGCGGGCATGCTCCAGCC	EPHB6__NM_004445	199	332	286	430
EPHB6__NM_004445_TTTGAGGCATGTCATGTGGC	EPHB6__NM_004445	422	476	522	510
EPHX1__NM_000120_AAACTTTGCCCACTTGAAGAT	EPHX1__NM_000120	128	313	221	379
EPHX1__NM_000120_AGGAATTCTACATTCAAGGA	EPHX1__NM_000120	63	30	59	70
EPHX1__NM_000120_CAAGGTGGAAACGTCAGATG	EPHX1__NM_000120	26	33	14	2
EPHX1__NM_000120_CACTTCAAGACTAAGATTGA	EPHX1__NM_000120	656	795	719	875
EPHX1__NM_000120_CAGGAATTCTACATTCAAGG	EPHX1__NM_000120	694	751	831	693
EPHX1__NM_000120_CCGGGAGATGAACCAGTAGA	EPHX1__NM_000120	590	565	630	657
EPHX1__NM_000120_CGACAGCATCCGCCCTTTCA	EPHX1__NM_000120	1662	1110	1722	1048
EPHX1__NM_000120_GCCACTTGAAGATGGGTGGT	EPHX1__NM_000120	827	777	933	951
EPHX1__NM_000120_GTCAAATTCATTCCGCCAGT	EPHX1__NM_000120	1146	619	1139	720
EPHX1__NM_000120_GTCCCTCATCTGCACTAATA	EPHX1__NM_000120	556	368	587	1097
EPHX1__NM_000120_GTGGCCACCGAGTTGAACCC	EPHX1__NM_000120	152	140	176	245
EPHX1__NM_000120_TGCTGGGCTTTGCCATCTAC	EPHX1__NM_000120	414	422	436	394
EPHX2__NM_001256482_AGCCTTTCATGTCCATAGCT	EPHX2__NM_001256482	41	25	45	51
EPHX2__NM_001256482_AGGTTACCGGGTCCTAGCTA	EPHX2__NM_001256482	296	222	251	315
EPHX2__NM_001256482_CCACCTGACACGACTCTATC	EPHX2__NM_001256482	906	1087	916	1181
EPHX2__NM_001256482_CCGAGAGTTGGTATTCTTGG	EPHX2__NM_001256482	960	1156	991	1317
EPHX2__NM_001256482_CCTTTACTGTCACGTACCCA	EPHX2__NM_001256482	1028	1446	1062	923
EPHX2__NM_001256482_CTTCCTGATAGAGTCGTGTC	EPHX2__NM_001256482	383	473	418	524
EPHX2__NM_001256482_GAGGCAGACTTTAGCGGTCT	EPHX2__NM_001256482	486	493	557	751
EPHX2__NM_001256482_GCCATGGATTTCCCGAGAGT	EPHX2__NM_001256482	923	1037	957	1086
EPHX2__NM_001256482_TCATGTCCATAGCTAGGACC	EPHX2__NM_001256482	265	356	279	348
EPHX2__NM_001256482_TCTGGCCCAGGCAGGTTACC	EPHX2__NM_001256482	547	606	445	588
EPHX2__NM_001256482_TTATGGAGAAATTCTTGGGG	EPHX2__NM_001256482	170	172	218	187
EPHX2__NM_001256482_TTGCATGGAAGTGTTATGTA	EPHX2__NM_001256482	153	223	186	145
EPHX2__NM_001979_GCAGCGCCAGCACCCCGTCA	EPHX2__NM_001979	999	798	986	1211
EPHX2__NM_001979_GCCACTACCCGGCTTATGAA	EPHX2__NM_001979	1141	1202	1360	1084
EPHX2__NM_001979_GCGGCCGTCTTCGACCTTGA	EPHX2__NM_001979	1527	2112	1660	1885
EPHX2__NM_001979_GGCCGTCTTCGACCTTGACG	EPHX2__NM_001979	657	612	744	642
EPHX3__NM_001142886_AGATGGAGAAATGCCAGGCA	EPHX3__NM_001142886	379	298	446	433
EPHX3__NM_001142886_CACAACCATCCGCTCGACCA	EPHX3__NM_001142886	817	435	841	636
EPHX3__NM_001142886_CCACAACCATCCGCTCGACC	EPHX3__NM_001142886	990	558	1124	575
EPHX3__NM_001142886_CCATCCGCTCGACCAGGGAT	EPHX3__NM_001142886	1305	1270	1377	1120
EPHX3__NM_001142886_CCGTGCAGAAACAGCATGAG	EPHX3__NM_001142886	623	611	702	830
EPHX3__NM_001142886_CGTCTGCACTATGTCTCGGC	EPHX3__NM_001142886	1416	1470	1792	2121
EPHX3__NM_001142886_CTCCCGGAGCTGGTAACGCC	EPHX3__NM_001142886	182	146	278	266
EPHX3__NM_001142886_CTGGTGGCCGCGGCGGTCTA	EPHX3__NM_001142886	347	511	467	496
EPHX3__NM_001142886_GAACACCAGGCTCCACATGA	EPHX3__NM_001142886	1155	1407	1318	1253
EPHX3__NM_001142886_GACCAGGGATGGGTAGTAGA	EPHX3__NM_001142886	553	654	625	599
EPHX3__NM_001142886_GATGCACTTCGAGTAACCTG	EPHX3__NM_001142886	1307	1382	1423	1387
EPHX3__NM_001142886_TATGCAGCCGTAGACCGCCG	EPHX3__NM_001142886	576	515	733	587
EPHX4__NM_173567_ACCTGAAGATATTTCGGTAA	EPHX4__NM_173567	1282	1540	1412	1628
EPHX4__NM_173567_AGAGAATGACGCATTCATGG	EPHX4__NM_173567	626	305	648	384
EPHX4__NM_173567_ATCAATTTGTTCACTATGTC	EPHX4__NM_173567	263	292	248	316
EPHX4__NM_173567_ATTCTGGGAACCATGGTATT	EPHX4__NM_173567	311	397	386	446
EPHX4__NM_173567_CACTCCAACACTACTACTGT	EPHX4__NM_173567	1423	906	1674	1430
EPHX4__NM_173567_CCACAGTAGTAGTGTTGGAG	EPHX4__NM_173567	624	603	721	817
EPHX4__NM_173567_CCTCATCCAAATGTATTTAC	EPHX4__NM_173567	491	516	593	564
EPHX4__NM_173567_CTGTTTACCAGTCACAGCAC	EPHX4__NM_173567	394	331	291	346
EPHX4__NM_173567_GCACTGGATTTGAGAGGTTA	EPHX4__NM_173567	325	489	471	442
EPHX4__NM_173567_TGGGCACCCACTGCTACGTG	EPHX4__NM_173567	185	63	176	188
EPHX4__NM_173567_TTCATGGATTTCCAGAATTC	EPHX4__NM_173567	17	40	29	29
EPM2A__NM_001018041_AAGAAGGCTTGGCCTACATC	EPM2A__NM_001018041	462	314	477	341
EPM2A__NM_001018041_AATGAATTTCCAGACTGAAT	EPM2A__NM_001018041	319	338	341	384
EPM2A__NM_001018041_ACCAAGCCATGCATTATTCA	EPM2A__NM_001018041	3358	3896	4113	4006
EPM2A__NM_001018041_ATCAAACTGAAGCATGAATT	EPM2A__NM_001018041	404	331	356	411
EPM2A__NM_001018041_ATTGTCTCCCAATAGGACAC	EPM2A__NM_001018041	250	127	259	197
EPM2A__NM_001018041_CAGCATCTGTACTCGGCCTG	EPM2A__NM_001018041	1646	1898	2054	2346
EPM2A__NM_001018041_CATCAAACTGAAGCATGAAT	EPM2A__NM_001018041	571	739	653	860
EPM2A__NM_001018041_CCTCAATCCAGTGTCCTATT	EPM2A__NM_001018041	2349	2183	2442	2168
EPM2A__NM_001018041_GACACTGGATTGAGGCCACT	EPM2A__NM_001018041	1487	1564	1566	1839
EPM2A__NM_001018041_GTACAATATCCCATTCAGTC	EPM2A__NM_001018041	492	297	502	368
EPM2A__NM_001018041_TCGGTGCTCATATCTGGTGT	EPM2A__NM_001018041	1526	1548	1647	1826
EPM2A__NM_001018041_TTTCATTGTAAGTACAGCAA	EPM2A__NM_001018041	28	36	19	0
EPM2AIP1__NM_014805_CGACGTCAGGCGCCACTACG	EPM2AIP1__NM_014805	9	0	15	0
EPM2AIP1__NM_014805_CGAGTGGACCCAGCGTTATT	EPM2AIP1__NM_014805	1566	977	1589	1002
EPM2AIP1__NM_014805_CGATGAGGCGGCGACAGACC	EPM2AIP1__NM_014805	387	499	393	307
EPM2AIP1__NM_014805_CTACCCGCGAACGCGACGTC	EPM2AIP1__NM_014805	237	144	247	351
EPM2AIP1__NM_014805_CTCCACCACCAAATAACGCT	EPM2AIP1__NM_014805	279	269	352	171
EPM2AIP1__NM_014805_CTGAGCACGAATACTACGAG	EPM2AIP1__NM_014805	729	540	742	674
EPM2AIP1__NM_014805_GACCCAGCGTTATTTGGTGG	EPM2AIP1__NM_014805	505	469	621	461
EPM2AIP1__NM_014805_GCGCCTGACGTCGCGTTCGC	EPM2AIP1__NM_014805	141	90	184	159
EPM2AIP1__NM_014805_GCTCCACCACCAAATAACGC	EPM2AIP1__NM_014805	85	46	49	47
EPM2AIP1__NM_014805_GTGGACCCAGCGTTATTTGG	EPM2AIP1__NM_014805	173	119	191	116
EPM2AIP1__NM_014805_GTTCGCGGGTAGCTACGATG	EPM2AIP1__NM_014805	292	93	290	128
EPM2AIP1__NM_014805_TGGAGCCTCGGAGGGCGAT	EPM2AIP1__NM_014805	160	128	217	194
EPN1__NM_001130071_AGGCGACAACGTTGTAGGTG	EPN1__NM_001130071	106	51	66	87
EPN1__NM_001130071_ATGGGGCGACTCCCGGACGA	EPN1__NM_001130071	944	900	889	785
EPN1__NM_001130071_CATCGTCCACAACTACTCAG	EPN1__NM_001130071	608	667	892	591
EPN1__NM_001130071_CCAGAAGACCAGACGTCCCA	EPN1__NM_001130071	523	476	730	679
EPN1__NM_001130071_CCCCATCTCTCCACGCATCG	EPN1__NM_001130071	1694	1553	1700	1808
EPN1__NM_001130071_CCTCAAGGACGAGGTCGACA	EPN1__NM_001130071	334	260	279	300
EPN1__NM_001130071_GAACTGGCGTCACGTTTACA	EPN1__NM_001130071	727	605	806	769
EPN1__NM_001130071_GCCCCATCTCTCCACGCATC	EPN1__NM_001130071	1427	1274	1464	1063
EPN1__NM_001130071_GCCCCGATGCGTGGAGAGAT	EPN1__NM_001130071	1187	1361	1167	1517
EPN1__NM_001130071_GTACCTCATCAAGACCGGCT	EPN1__NM_001130071	778	726	931	995
EPN1__NM_001130071_TCCGCTATCCTTGGGACGTC	EPN1__NM_001130071	1014	1074	1050	1313
EPN1__NM_001130071_TCTCCGCCTCTGAGTAGTTG	EPN1__NM_001130071	455	363	465	362
EPN1__NM_001130072_AAGCTGGCACAGACCGCCAC	EPN1__NM_001130072	644	574	654	663
EPN1__NM_001130072_AGGAGGAGCGGATCCGTCGC	EPN1__NM_001130072	1320	1335	1440	1325
EPN1__NM_001130072_CAGGAGGAGCGGATCCGTCG	EPN1__NM_001130072	1483	1412	1801	1594
EPN1__NM_001130072_GCTCTTCTCGGCTCAAACTA	EPN1__NM_001130072	203	203	189	159
EPN1__NM_001130072_GGAGGAGCGGATCCGTCGCG	EPN1__NM_001130072	1891	2062	2171	1858
EPN1__NM_001130072_TGCCGTCGCGGTCCACGTAC	EPN1__NM_001130072	416	565	495	563
EPN1__NM_013333_GCCGCAGGTCATCCCCGCGA	EPN1__NM_013333	6448	5854	6998	5235
EPN2__NM_001102664_AGCTGCCTCCATTGACCCAT	EPN2__NM_001102664	1191	1589	1358	1785
EPN2__NM_001102664_CAGCTGCCTCCATTGACCCA	EPN2__NM_001102664	699	604	831	610
EPN2__NM_001102664_CCCACAGCAGACTACGCTGT	EPN2__NM_001102664	360	462	482	393
EPN2__NM_001102664_CTCCAGTGGGCACCCCCCAT	EPN2__NM_001102664	885	904	1006	1011
EPN2__NM_001102664_CTGCCTCCATTGACCCATGG	EPN2__NM_001102664	378	499	426	520
EPN2__NM_001102664_CTTGGGGACAGATTGTACGG	EPN2__NM_001102664	511	667	597	689
EPN2__NM_001102664_GCTGCCTCCATTGACCCATG	EPN2__NM_001102664	693	968	929	671
EPN2__NM_001102664_GGGCACCCCCCATGGGTCAA	EPN2__NM_001102664	175	275	161	105
EPN2__NM_001102664_GGGGTTGGTCTGGTTAGTGG	EPN2__NM_001102664	411	536	513	479
EPN2__NM_001102664_TCAGACCCCTGGCCATCGTT	EPN2__NM_001102664	859	794	1004	921
EPN2__NM_001102664_TCCAACAGCGTAGTCTGCTG	EPN2__NM_001102664	91	114	91	142
EPN2__NM_001102664_TGCCTCCATTGACCCATGGG	EPN2__NM_001102664	307	338	356	382
EPN2__NM_014964_AAGCCACCTCCAATGACCCG	EPN2__NM_014964	932	970	1023	763
EPN2__NM_014964_AGCCACCTCCAATGACCCGT	EPN2__NM_014964	1153	886	1299	1109
EPN2__NM_014964_CAAGACAGGCTCCGAACGTG	EPN2__NM_014964	1638	1646	1809	1532
EPN2__NM_014964_CATCGTGAACAATTACTCAG	EPN2__NM_014964	678	465	543	398
EPN2__NM_014964_CTCCTCTTCTCCACTAGTCT	EPN2__NM_014964	398	416	423	446
EPN2__NM_014964_GCTCCTCTTCTCCACTAGTC	EPN2__NM_014964	694	751	831	693
EPN2__NM_014964_GTAGGTCAGGTCGGCAATCT	EPN2__NM_014964	319	223	448	371
EPN2__NM_014964_TCCAGCTCGGAGGACACTCG	EPN2__NM_014964	427	501	476	480
EPN2__NM_014964_TCCTCTTCTCCACTAGTCTG	EPN2__NM_014964	439	396	458	489
EPN2__NM_014964_TGACGACTTCGTCTATCAGA	EPN2__NM_014964	44	91	60	15
EPN2__NM_014964_TGCCGACCTGACCTACAACG	EPN2__NM_014964	583	618	837	1170
EPN2__NM_014964_TTCCAGTACATTGACCGAGA	EPN2__NM_014964	76	50	121	47
EPN3__NM_017957_ACCCCGCTATACCTCCGACC	EPN3__NM_017957	2324	2274	2596	2370
EPN3__NM_017957_ACTGGAAGTCCTTGAGTGTC	EPN3__NM_017957	383	343	449	429
EPN3__NM_017957_AGTGGCCTTCACCGAAGTCA	EPN3__NM_017957	451	363	369	340
EPN3__NM_017957_ATCTCGGACATGAGCGAACT	EPN3__NM_017957	304	320	351	352
EPN3__NM_017957_CATGCCCATGACTTCGGTGA	EPN3__NM_017957	1258	1275	1464	1214
EPN3__NM_017957_CGGACATGAGCGAACTAGGG	EPN3__NM_017957	364	325	398	378
EPN3__NM_017957_GAAGGTCAGGTCAGCGATCT	EPN3__NM_017957	293	500	437	298
EPN3__NM_017957_GCTCCAGGTCGGAGGTATAG	EPN3__NM_017957	480	437	519	606
EPN3__NM_017957_GTGGCCTTCACCGAAGTCAT	EPN3__NM_017957	119	173	136	190
EPN3__NM_017957_TCCAGGTCGGAGGTATAGCG	EPN3__NM_017957	45	14	48	42
EPN3__NM_017957_TCGGACATGAGCGAACTAGG	EPN3__NM_017957	218	91	184	90
EPN3__NM_017957_TGTAGAGGTTCTCGCGGCAC	EPN3__NM_017957	155	159	189	163
EPO__NM_000799_AAGTGTCAGCAGTGATTGTT	EPO__NM_000799	427	352	481	507
EPO__NM_000799_CAAGGAGGCCGAGAATATCA	EPO__NM_000799	223	254	285	267
EPO__NM_000799_CTGTCACAGATGAGGCGTGG	EPO__NM_000799	483	557	534	514
EPO__NM_000799_GAAAGCTGAAGCTGTACACA	EPO__NM_000799	156	124	201	114
EPO__NM_000799_GCAGGACAGCTTCCGACAGC	EPO__NM_000799	695	1007	736	1272
EPO__NM_000799_GCTGGGAAGAGTTGACCAAC	EPO__NM_000799	204	163	239	244
EPO__NM_000799_GGAAAGCTGAAGCTGTACAC	EPO__NM_000799	313	248	326	294
EPO__NM_000799_GGAGGAAATTGGAGTAGACT	EPO__NM_000799	254	250	272	284
EPO__NM_000799_GGTCAACTCTTCCCAGCCGT	EPO__NM_000799	2298	2891	2546	2222
EPO__NM_000799_TCAGCAGTGATTGTTCGGAG	EPO__NM_000799	651	965	876	555
EPOR__NM_000121_ACCCTCAATGGGCCATTTGA	EPOR__NM_000121	438	411	575	405
EPOR__NM_000121_CGCACCGCACCACGAGCCGT	EPOR__NM_000121	1654	1762	1964	1520
EPOR__NM_000121_CTTCTCTTAGGGTACTTAGA	EPOR__NM_000121	84	54	85	87
EPOR__NM_000121_GACTGGCAGTTTGTAGAACA	EPOR__NM_000121	705	680	793	791
EPOR__NM_000121_GCGGCCACGTAGTGTTGCGC	EPOR__NM_000121	732	466	800	716
EPOR__NM_000121_GCTGTGACGCGCAACTCTAG	EPOR__NM_000121	1105	758	1268	813
EPOR__NM_000121_GGAGCCAGCGCAACACTACG	EPOR__NM_000121	1295	1190	1553	1213
EPOR__NM_000121_GGGCACGAAGCTCGACGTGT	EPOR__NM_000121	889	697	972	952
EPOR__NM_000121_GGGTCCGGGAGGTTAGGCGG	EPOR__NM_000121	388	351	364	256
EPOR__NM_000121_TACCTTTGCTCTCGAACTTG	EPOR__NM_000121	327	290	287	306
EPOR__NM_000121_TCCCTCAAATGGCCCATTGA	EPOR__NM_000121	710	426	934	520
EPOR__NM_000121_TCCTGGCCTTTCATGTGACT	EPOR__NM_000121	877	1235	1065	1413
EPOR__NM_000121_TCTGTGCTTCACCGAGCGGT	EPOR__NM_000121	326	181	348	291
EPOR__NM_000121_TGAACCTCTGACTCATCCCA	EPOR__NM_000121	522	440	555	508
EPOR__NM_000121_TGCTCCTAGACGCCCCCGTG	EPOR__NM_000121	381	268	383	457
EPOR__NM_000121_TGCTGTGACGCGCAACTCTA	EPOR__NM_000121	467	633	523	569
EPOR__NM_000121_TGGATGACACGGTGATATCG	EPOR__NM_000121	55	42	65	59
EPOR__NM_000121_TGTAGCTCTACCTTCTCTTA	EPOR__NM_000121	56	26	107	85
EPOR__NM_000121_TTGCTCTCGAACTTGGGGTC	EPOR__NM_000121	94	221	86	107
EPPIN-WFDC6__NM_001198986_AAGTGTTGCCCGTTCAGCC	EPPIN-WFDC6__NM_001198986	930	969	971	1059
EPPIN-WFDC6__NM_001198986_AATCTCTGGGTTTGGTACA	EPPIN-WFDC6__NM_001198986	402	274	415	414
EPPIN-WFDC6__NM_001198986_AGGGACCTGGTCTGACTG	EPPIN-WFDC6__NM_001198986	633	573	705	651
EPPIN-WFDC6__NM_001198986_ATTTCTTTCCACGGCTGAA	EPPIN-WFDC6__NM_001198986	99	126	158	223
EPPIN-WFDC6__NM_001198986_CGTCCTCTTAGCGAATGTC	EPPIN-WFDC6__NM_001198986	379	420	520	552
EPPIN-WFDC6__NM_001198986_CTAAGAGGACGAATAGCA	EPPIN-WFDC6__NM_001198986	252	262	235	228
EPPIN-WFDC6__NM_001198986_GTCCCTGGACATTCGCTAA	EPPIN-WFDC6__NM_001198986	633	777	716	1010
EPPK1__NM_031308_CCAGAGTGTCTACGCCGCCA	EPPK1__NM_031308	922	849	1138	753
EPPK1__NM_031308_CCATGGCGGCGTAGACACTC	EPPK1__NM_031308	293	198	369	358
EPPK1__NM_031308_CCCAGGCCAGGAGCATAGCT	EPPK1__NM_031308	0	0	0	0
EPPK1__NM_031308_CCCCAGGCCAGGAGCATAGC	EPPK1__NM_031308	0	0	0	0
EPPK1__NM_031308_CGGGAAGGAGGTTGTGGACA	EPPK1__NM_031308	799	709	841	843
EPPK1__NM_031308_GAGCGGAAGGTGATCTTGAG	EPPK1__NM_031308	536	359	553	411
EPPK1__NM_031308_GCCCGTAGTGGCACGCTCAG	EPPK1__NM_031308	748	658	783	648
EPPK1__NM_031308_GCCGCTGAGCGTGCCACTAC	EPPK1__NM_031308	202	225	295	210
EPPK1__NM_031308_GCTCGGGCAGGCTCTGCTAG	EPPK1__NM_031308	672	501	697	682
EPPK1__NM_031308_GGCCGCTGAGCGTGCCACTA	EPPK1__NM_031308	68	127	83	126
EPPK1__NM_031308_GGGGTCAGGATAGCCCGTAG	EPPK1__NM_031308	129	93	179	107
EPPK1__NM_031308_TCGGGAAGGAGGTTGTGGAC	EPPK1__NM_031308	700	728	735	754
EPRS__NM_004446_AGGAGACCCTCCGCTAGGTA	EPRS__NM_004446	123	3	98	0
EPRS__NM_004446_CAGCAGGCCTTCCAGTCAGT	EPRS__NM_004446	1054	271	805	239
EPRS__NM_004446_CCAGTCAGTAGGTACCAAGT	EPRS__NM_004446	642	165	502	154
EPRS__NM_004446_CCCACTTGGTACCTACTGAC	EPRS__NM_004446	1389	489	1321	423
EPRS__NM_004446_CTCCAGTTCATGTAAAACGT	EPRS__NM_004446	456	121	276	63
EPRS__NM_004446_GAATTCTATACTTCGCTACT	EPRS__NM_004446	192	191	234	326
EPRS__NM_004446_GCAACTACAGCTGGGTTATA	EPRS__NM_004446	354	167	264	132
EPRS__NM_004446_GGCCTCTGGAGGAAATCTGA	EPRS__NM_004446	300	5	320	1
EPRS__NM_004446_GTTCATGTAAAACGTTGGTT	EPRS__NM_004446	288	92	338	130
EPRS__NM_004446_GTTTCCCTTTAAAGTTAACC	EPRS__NM_004446	12	3	19	14
EPRS__NM_004446_TAATCTGATGGAACATACTG	EPRS__NM_004446	105	8	75	38
EPRS__NM_004446_TCAATGCTTTAAAAAGTGAG	EPRS__NM_004446	53	73	110	162
EPRS__NM_004446_TCCAGTCAGTAGGTACCAAG	EPRS__NM_004446	281	156	258	105
EPRS__NM_004446_TCTTATGCCTAAGTTAACAG	EPRS__NM_004446	322	363	224	241
EPS15__NM_001159969_AAAAGCGATTCAGGATTAGG	EPS15__NM_001159969	172	289	245	244
EPS15__NM_001159969_ACTGGAAGATTCCTGGTGTAT	EPS15__NM_001159969	1670	1555	1823	1556
EPS15__NM_001159969_AGAGTCAGGGAAGGCTCAGT	EPS15__NM_001159969	555	439	527	395
EPS15__NM_001159969_AGAGTCTGAGCCCATACACC	EPS15__NM_001159969	494	666	546	345
EPS15__NM_001159969_AGGAAGATACTATTAAACAG	EPS15__NM_001159969	93	139	76	21
EPS15__NM_001159969_CAGAAAGAAATGTGCTGAGG	EPS15__NM_001159969	368	276	539	481
EPS15__NM_001159969_CATTGTTTGCTGGTCTTCTA	EPS15__NM_001159969	640	849	676	658
EPS15__NM_001159969_CCACACAGCATTCTTGTAAA	EPS15__NM_001159969	471	721	563	565
EPS15__NM_001159969_GAAAGCTGAATTAACTAGTC	EPS15__NM_001159969	367	499	391	378
EPS15__NM_001159969_GCACCTACAAGATTCACAAC	EPS15__NM_001159969	650	581	784	624
EPS15__NM_001159969_TACTAATCTGCAAAAACTAC	EPS15__NM_001159969	167	96	289	291
EPS15__NM_001159969_TTGGAGGAGAGTGTAGAGTC	EPS15__NM_001159969	88	223	100	122
EPS15__NM_001981_ACCTTTCCAAGTATCAAGTC	EPS15__NM_001981	328	376	392	442
EPS15__NM_001981_ACTGATTAGCAAAGGACTAC	EPS15__NM_001981	145	178	193	124
EPS15__NM_001981_AGTAGTCCTTTGCTAATCAG	EPS15__NM_001981	315	309	328	351
EPS15__NM_001981_CAGGTTGATACAGGCAATAC	EPS15__NM_001981	276	369	310	385
EPS15__NM_001981_CCAGTGAATGGATTTCTGTC	EPS15__NM_001981	794	782	812	809
EPS15__NM_001981_CTTTGTTGCTTTGCGTCTTG	EPS15__NM_001981	991	1238	1193	1646
EPS15__NM_001981_GATATGGACGGATTTGTGTC	EPS15__NM_001981	1059	1319	914	1224
EPS15__NM_001981_GCTGCTTTCCTGAAAAAATC	EPS15__NM_001981	106	91	127	132
EPS15__NM_001981_GGGCTTCCAGACTTGATACT	EPS15__NM_001981	349	451	340	444
EPS15__NM_001981_TGCACAGTATACCAAAAACA	EPS15__NM_001981	95	58	155	140
EPS15__NM_001981_TTACCATATATGGGCTAGTA	EPS15__NM_001981	185	409	196	334
EPS15__NM_001981_TTCACTAAGTAGTTTGAACC	EPS15__NM_001981	31	76	32	29
EPS15L1__NM_001258374_AGAACCTTCTAGCACACATA	EPS15L1__NM_001258374	141	95	170	153
EPS15L1__NM_001258374_CAGGTCGATCGGCATACAC	EPS15L1__NM_001258374	419	396	345	345
EPS15L1__NM_001258374_CCAGGGCCCTGGCCGATACG	EPS15L1__NM_001258374	0	0	4	0
EPS15L1__NM_001258374_CGATCGGCATACACAGGGA	EPS15L1__NM_001258374	695	418	885	647
[illegible]					
EPSTI1__NM_001002264_CGGAGTTCACCACTCTATTG	EPSTI1__NM_001002264	954	974	953	798
EPSTI1__NM_001002264_CTCCCGCGAAGGGCCCTTAG	EPSTI1__NM_001002264	197	178	201	169
EPSTI1__NM_001002264_CTGTGATCCCTAGGCAGGAT	EPSTI1__NM_001002264	570	770	671	726
EPSTI1__NM_001002264_GCACGACGCTCTCCCGCGAA	EPSTI1__NM_001002264	798	1048	875	936
EPSTI1__NM_001002264_GGAGCAGAACAGAGCTAAAC	EPSTI1__NM_001002264	347	443	409	364
EPSTI1__NM_001002264_GGGTCCTGGGGATCCCGGGT	EPSTI1__NM_001002264	398	730	516	619
EPSTI1__NM_001002264_GGTTTGGAGGCAGCCCCTAA	EPSTI1__NM_001002264	191	104	128	202
EPSTI1__NM_001002264_TCATTTCTCCGGTTTATATT	EPSTI1__NM_001002264	860	1052	904	1142
EPSTI1__NM_001002264_TGGCCCACAATCCTCAACAT	EPSTI1__NM_001002264	105	210	131	92
EPSTI1__NM_033255_AAATCCACCAGACTGAACAC	EPSTI1__NM_033255	364	359	335	363
EPSTI1__NM_033255_TTTACCTCCTGTGTTCAGTC	EPSTI1__NM_033255	321	263	313	347
EPT1__NM_033505_AAACAGGAAAGGTTCATACC	EPT1__NM_033505	191	188	206	155
EPT1__NM_033505_ACTGGGAAAAGTATAACACA	EPT1__NM_033505	328	368	375	309
EPT1__NM_033505_ATGATCAAAAAGCTCCCCTA	EPT1__NM_033505	295	258	267	284
EPT1__NM_033505_ATTCTGGAACACTATAGTAA	EPT1__NM_033505	189	95	219	157
EPT1__NM_033505_CAATTCTAGCACTCCCTTAG	EPT1__NM_033505	530	441	561	554
EPT1__NM_033505_CCAATTCTAGCACTCCCTTA	EPT1__NM_033505	1344	1252	1438	1414
EPT1__NM_033505_CCCTAAGGAGTGCTAGAAT	EPT1__NM_033505	1016	514	904	908
EPT1__NM_033505_GTCACAAGCACGTGCCTGAC	EPT1__NM_033505	637	421	837	988
[illegible]					

ERAS__NM_181532_CAGCAGGGCAGGCCATCCAT	ERAS__NM_181532	705	531	604	557
ERAS__NM_181532_GGACTGCATTCTGAATGTGC	ERAS__NM_181532	582	413	467	642
ERBB2__NM_001005862_ACAATACCACCCCTGTCACA	ERBB2__NM_001005862	1490	1432	1667	1426
ERBB2__NM_001005862_ACAGGGGTGGTATTGTTCAG	ERBB2__NM_001005862	142	136	143	201
ERBB2__NM_001005862_ACCAGGCAGTCAGAGTGCTT	ERBB2__NM_001005862	598	718	834	607
ERBB2__NM_001005862_AGGTGGGTCTCGGGACTGGC	ERBB2__NM_001005862	213	191	133	159
ERBB2__NM_001005862_CAGAGGCTGCGGATTGTGCG	ERBB2__NM_001005862	859	1383	984	1121
ERBB2__NM_001005862_CAGGGGTGGTATTGTTCAGC	ERBB2__NM_001005862	243	225	211	301
ERBB2__NM_001005862_CCCCTGTTCTCCGATGTGTA	ERBB2__NM_001005862	1697	1966	2047	2079
ERBB2__NM_001005862_CCCGAGGGCCGGTATACATT	ERBB2__NM_001005862	125	144	180	97
ERBB2__NM_001005862_CCCTTACACATCGGAGAACA	ERBB2__NM_001005862	1433	1819	1429	1612
ERBB2__NM_001005862_CTCCATTGTCTAGCACGGCC	ERBB2__NM_001005862	369	399	478	481
ERBB2__NM_001005862_CTGGGGAGGCCCCTGTGACA	ERBB2__NM_001005862	640	95	493	76
ERBB2__NM_001005862_GTATACCGGCCCTCGGGATT	ERBB2__NM_001005862	999	1284	1105	1148
ERBB2__NM_004448_ACCCTAGAGTCTCATTGGGC	ERBB2__NM_004448	1100	990	1290	1213
ERBB2__NM_004448_AGATGAAATAAAGACCCAGG	ERBB2__NM_004448	322	185	343	265
ERBB2__NM_004448_CCCTAAGGCTTTCAGTACCC	ERBB2__NM_004448	949	1030	914	1004
ERBB2__NM_004448_CCCTGCCCAATGAGACTCTA	ERBB2__NM_004448	465	481	612	571
ERBB2__NM_004448_CCTGGGTACTGAAAGCCTTA	ERBB2__NM_004448	316	255	405	326
ERBB2__NM_004448_GAGAGGGGAAGCGGCCCTAA	ERBB2__NM_004448	682	417	659	591
ERBB2__NM_004448_GGGAGAATGGGTGTTGTATG	ERBB2__NM_004448	694	1044	707	958
ERBB2__NM_004448_GGGGAGAATGGGTGTTGTAT	ERBB2__NM_004448	867	998	844	992
ERBB2__NM_004448_TAAAGATGAAATAAAGACCC	ERBB2__NM_004448	96	66	127	61
ERBB2__NM_004448_TGAGAGGGGAAGCGGCCCTA	ERBB2__NM_004448	479	390	586	276
ERBB2IP__NM_001006600_AAGCATCATTCAGATACAAC	ERBB2IP__NM_001006600	216	215	229	218
ERBB2IP__NM_001006600_ACGAAGTTTGTTTGTGCGGT	ERBB2IP__NM_001006600	672	867	961	1361
ERBB2IP__NM_001006600_AGCATCATTCAGATACAACT	ERBB2IP__NM_001006600	650	557	809	681
ERBB2IP__NM_001006600_AGTCTGGCAGATACATTAAC	ERBB2IP__NM_001006600	710	717	625	516
ERBB2IP__NM_001006600_ATGTATCTGCCAGACTCTAT	ERBB2IP__NM_001006600	480	458	465	469
ERBB2IP__NM_001006600_CATTCAGATACAACTGGGTT	ERBB2IP__NM_001006600	531	578	550	635
ERBB2IP__NM_001006600_CTTGGAAATAGGGTTTACAC	ERBB2IP__NM_001006600	178	150	174	83
ERBB2IP__NM_001006600_GAAGTACTTGAGCAACTAAG	ERBB2IP__NM_001006600	99	103	171	158
ERBB2IP__NM_001006600_TACCATGTCGCTGTCTACGA	ERBB2IP__NM_001006600	822	816	878	707
ERBB2IP__NM_001006600_TATCTGCCAGACTCTATAGG	ERBB2IP__NM_001006600	696	872	618	405
ERBB2IP__NM_001006600_TGCAAACCTTATTAATCTCA	ERBB2IP__NM_001006600	990	1168	1067	1388
ERBB2IP__NM_001006600_TTTGGGAAGTAACGAATTCA	ERBB2IP__NM_001006600	35	35	42	50
ERBB2IP__NM_018695_AAATAAAGGGCTGTTTCTAC	ERBB2IP__NM_018695	191	176	130	164
ERBB2IP__NM_018695_ACAAAATGTTACGAAGGTTT	ERBB2IP__NM_018695	227	247	270	264
ERBB2IP__NM_018695_AGGATTAATGCATGGTTATT	ERBB2IP__NM_018695	239	224	313	313
ERBB2IP__NM_018695_AGTAGCAAATGTATAGTCAA	ERBB2IP__NM_018695	260	127	239	205
ERBB2IP__NM_018695_CCAAATATTGCTCTAATGAA	ERBB2IP__NM_018695	266	401	201	362
ERBB2IP__NM_018695_TAATGGTAAGGATTAATGCA	ERBB2IP__NM_018695	276	261	249	310
ERBB2IP__NM_018695_TAATTGGTATATGTGGTTCA	ERBB2IP__NM_018695	232	240	247	239
ERBB2IP__NM_018695_TGAAGATCCATTGTTCCGTG	ERBB2IP__NM_018695	1585	1827	1767	1605
ERBB2IP__NM_018695_TGTGGAGAGTTATAGAGCAA	ERBB2IP__NM_018695	133	162	201	213
ERBB2IP__NM_018695_TGTTTGGCCTTGCATTGCCT	ERBB2IP__NM_018695	71	94	65	50
ERBB3__NM_001005915_AAACTTCCCATCGTAGACCT	ERBB3__NM_001005915	232	213	238	197
ERBB3__NM_001005915_AGGAACCATCGGGAACTGAC	ERBB3__NM_001005915	285	197	370	313
ERBB3__NM_001005915_CGAGGGACCCAGGTCTACGA	ERBB3__NM_001005915	800	623	905	577
ERBB3__NM_001005915_CGTAGAGCTTGTACAGTGTC	ERBB3__NM_001005915	650	761	727	699
ERBB3__NM_001005915_GAGGGACCCAGGTCTACGAT	ERBB3__NM_001005915	743	393	767	304
ERBB3__NM_001005915_GCTCACCGGTCAGTTCCCGA	ERBB3__NM_001005915	1593	1542	1650	1639
ERBB3__NM_001005915_TCACCTTGGCCGCTGTCTAA	ERBB3__NM_001005915	856	676	931	491
ERBB3__NM_001005915_TGCCTGAGAGTTGCCCACCT	ERBB3__NM_001005915	992	788	922	707
ERBB3__NM_001982_AAACAGTGCCCCTTAAACAA	ERBB3__NM_001982	323	351	313	309
ERBB3__NM_001982_AAGATCATTTACGGCACATG	ERBB3__NM_001982	251	192	297	254
ERBB3__NM_001982_AAGTTACAGCCCTTGTTTAA	ERBB3__NM_001982	200	177	178	116
ERBB3__NM_001982_ACATGGACACAATTGACTGG	ERBB3__NM_001982	948	839	989	526
ERBB3__NM_001982_ACCAGGTCCCCCCTGTCATG	ERBB3__NM_001982	786	477	774	477
ERBB3__NM_001982_AGATGGGGGTCTTACTATGT	ERBB3__NM_001982	253	0	211	0
ERBB3__NM_001982_AGGTTTGCAAGGGGCGATGC	ERBB3__NM_001982	152	237	160	137
ERBB3__NM_001982_ATGAGATGTCTAGGTATAGG	ERBB3__NM_001982	305	289	313	335
ERBB3__NM_001982_CAGAAGACTGCCAGACATGT	ERBB3__NM_001982	33	31	56	19
ERBB3__NM_001982_CAGAATTAGAGATAAGCCTA	ERBB3__NM_001982	268	242	279	201
ERBB3__NM_001982_CAGTGTAATGGTCACTGCTT	ERBB3__NM_001982	2190	2411	2413	2078
ERBB3__NM_001982_CCCTTGCAAACCTCATGACA	ERBB3__NM_001982	900	843	915	354
ERBB3__NM_001982_CTCTCAGGCTCTTGACTACT	ERBB3__NM_001982	192	155	217	351
ERBB3__NM_001982_CTTCCTGAGATGAGATGTCT	ERBB3__NM_001982	386	486	477	429
ERBB3__NM_001982_GAAACAGTGCCCCTTAAACA	ERBB3__NM_001982	385	269	491	409
ERBB3__NM_001982_GAGAAGACGGTACCCTCTAA	ERBB3__NM_001982	422	446	499	276
ERBB3__NM_001982_GATCTTCACTCCTTATCCGA	ERBB3__NM_001982	501	666	489	285
ERBB3__NM_001982_GCAAGACACTGTCTCTACAG	ERBB3__NM_001982	528	360	465	222
ERBB3__NM_001982_GGCACTCAGGGAGCATTTAA	ERBB3__NM_001982	404	355	328	213
ERBB3__NM_001982_GTGCATGTGCTCTTATTGTA	ERBB3__NM_001982	99	115	100	77
ERBB3__NM_001982_GTGTCTTGCTATGTTTCCAT	ERBB3__NM_001982	283	119	230	119
ERBB3__NM_001982_TCAGAAGACTGCCAGACATG	ERBB3__NM_001982	149	41	136	55
ERBB3__NM_001982_TGATCTTCACTCCTTATCCG	ERBB3__NM_001982	816	584	803	546
ERBB3__NM_001982_TGGCAGCTAGTGCCTTTAGA	ERBB3__NM_001982	86	116	74	39
ERBB4__NM_001042599_AACAATATCTTGCCAATGAA	ERBB4__NM_001042599	203	299	259	416
ERBB4__NM_001042599_AACGGCCAGTACAGGACTTA	ERBB4__NM_001042599	935	765	880	1075
ERBB4__NM_001042599_AGAATTTACGCATTATTCGT	ERBB4__NM_001042599	316	276	313	261
ERBB4__NM_001042599_CCACCTGACTGAGAATCGCT	ERBB4__NM_001042599	1319	915	1378	1145
ERBB4__NM_001042599_CTGGGTGAGCCTTCTCGTGG	ERBB4__NM_001042599	851	761	947	827
ERBB4__NM_001042599_GAGAATTTACGCATTATTCG	ERBB4__NM_001042599	368	762	391	467
ERBB4__NM_001042599_GCAGGCACGCACACAAGAAC	ERBB4__NM_001042599	476	426	543	769
ERBB4__NM_001042599_GGATTGTAGACAAAGGTTTG	ERBB4__NM_001042599	151	207	200	246
ERBB4__NM_001042599_TGAAGCCGGCGACAGGACTT	ERBB4__NM_001042599	643	473	784	702
ERBB4__NM_001042599_TGCTGCCATCGAGAATGTGC	ERBB4__NM_001042599	405	423	414	483
ERBB4__NM_001042599_TTATGAGGATCGATATGCCT	ERBB4__NM_001042599	124	140	175	141
ERBB4__NM_001042599_TTGACTCTTGTGTCAACAAA	ERBB4__NM_001042599	41	56	63	78
ERC1__NM_178039_ACCATGACACTTGGCCGTTC	ERC1__NM_178039	1955	1654	2262	2184
ERC1__NM_178039_CACCGTCGAACCAACAGTAC	ERC1__NM_178039	493	569	516	328
ERC1__NM_178039_CATGGTCACTTAGATACATA	ERC1__NM_178039	462	425	435	599
ERC1__NM_178039_CGAACTCCCTCCCGTACTGT	ERC1__NM_178039	2728	2404	3029	2706
ERC1__NM_178039_CGTCGAACCAACAGTACGGG	ERC1__NM_178039	317	373	361	430
ERC1__NM_178039_CTTCCACGTTCCCCTCGCTT	ERC1__NM_178039	1194	1072	1280	988
ERC1__NM_178039_GCAGTCATCCGAACACCGTA	ERC1__NM_178039	1068	1025	1228	1080
ERC1__NM_178039_GTGGATGCCATACTCACAGG	ERC1__NM_178039	167	103	203	97
ERC1__NM_178039_GTTATCTCTCGCCTGACGAA	ERC1__NM_178039	31	12	37	16
ERC1__NM_178039_GTTCGACGGTGACCCAAGCG	ERC1__NM_178039	467	723	549	460
ERC1__NM_178039_TCATGGTCACTTAGATACAT	ERC1__NM_178039	399	327	489	729
ERC1__NM_178039_TCTGGGGGACGTCTGCCTTA	ERC1__NM_178039	844	1029	911	975
ERC2__NM_015576_AACAGGCAAGACTCTGTCTA	ERC2__NM_015576	621	440	731	530
ERC2__NM_015576_AATGCAGCCTATGCTACGTC	ERC2__NM_015576	1134	952	1221	1262
ERC2__NM_015576_AGGAGACCTTGGCAAACGAG	ERC2__NM_015576	168	68	167	98
ERC2__NM_015576_CATGGGTCCAGACGTAGCAT	ERC2__NM_015576	420	343	317	439
ERC2__NM_015576_CCACCGAAGAACAAGTAGTG	ERC2__NM_015576	394	361	310	337
ERC2__NM_015576_CCAGATCCCCTCGTTTGCCA	ERC2__NM_015576	1577	1361	1752	1641
ERC2__NM_015576_CCCCACTACTTGTTCTTCGG	ERC2__NM_015576	1967	1686	2238	1916
ERC2__NM_015576_CCTTGGCAAACGAGGGGATC	ERC2__NM_015576	28	68	72	64
ERC2__NM_015576_CGAGGAGACCTTGGCAAACG	ERC2__NM_015576	1112	878	1105	921
ERC2__NM_015576_GGCCACCGAAGAACAAGTAG	ERC2__NM_015576	264	255	330	261
ERC2__NM_015576_GTTCTTCGGTGGCCCAAACG	ERC2__NM_015576	81	37	67	79
ERC2__NM_015576_TTTGCCAAGGTCTCCTCGTT	ERC2__NM_015576	538	778	636	668
ERCC1__NM_001166049_ATATTCGGCGTAGGTCTGAG	ERCC1__NM_001166049	252	141	293	299
ERCC1__NM_001166049_ATTTGTGATACCCCTCGACG	ERCC1__NM_001166049	404	304	302	248
ERCC1__NM_001166049_CATATTCGGCGTAGGTCTGA	ERCC1__NM_001166049	23	3	23	10
ERCC1__NM_001166049_CGTAATTCCCGACTATGTGC	ERCC1__NM_001166049	885	1085	906	1313
ERCC1__NM_001166049_CTCTGTGTAGATCGGAATAA	ERCC1__NM_001166049	99	155	135	76
ERCC1__NM_001166049_TGAGATGGCATATTCGGCGT	ERCC1__NM_001166049	378	255	414	388
ERCC1__NM_001166049_TGGGGAAGAACTTCGCCTTG	ERCC1__NM_001166049	189	195	228	113
ERCC1__NM_001166049_TTGCGCACGAACTTCAGTAC	ERCC1__NM_001166049	599	275	758	340
ERCC1__NM_001983_AATGAGCAAGCTTAGGTTGC	ERCC1__NM_001983	161	103	215	195
ERCC1__NM_001983_ACGATTTATTATTACACTGG	ERCC1__NM_001983	424	356	460	546
ERCC1__NM_001983_ACTTACAAGGTTCATGCTTA	ERCC1__NM_001983	163	116	138	95
ERCC1__NM_001983_AGGACGATTTATTATTACAC	ERCC1__NM_001983	168	356	159	175
ERCC1__NM_001983_CGTGAAGTCAGTCAACAAAA	ERCC1__NM_001983	367	385	442	540
ERCC1__NM_001983_CTCTCTGTAGCTTCAATGGG	ERCC1__NM_001983	261	18	379	79
ERCC1__NM_001983_GGACGATTTATTATTACACT	ERCC1__NM_001983	775	1463	1007	1294
ERCC1__NM_001983_TCACTAAAGGTAGGGGCTAT	ERCC1__NM_001983	448	269	597	221
ERCC1__NM_001983_TCCTGACCACATTTGGAGTA	ERCC1__NM_001983	367	238	499	334
ERCC1__NM_001983_TGGTCAGACATTCAGTCACC	ERCC1__NM_001983	13	0	1	5
ERCC1__NM_001983_TTACTCCAAATGTGGTCAGG	ERCC1__NM_001983	193	223	253	179
ERCC2__NM_000400_ACCTCATAGAATCGGCAGTG	ERCC2__NM_000400	469	58	715	276
ERCC2__NM_000400_CAGGTGACACCCCTGCGCTT	ERCC2__NM_000400	589	244	592	96
ERCC2__NM_000400_CGAAGACCACGACGGCCTTG	ERCC2__NM_000400	3158	3588	3603	4665
ERCC2__NM_000400_CGGGGTAGATGTAGTCGTAC	ERCC2__NM_000400	448	0	564	5
ERCC2__NM_000400_CTGGTACTGCGCCCGCACAT	ERCC2__NM_000400	495	108	593	121
ERCC2__NM_000400_GCCCCACTGCCGATTCTATG	ERCC2__NM_000400	1414	9	1915	619
ERCC2__NM_000400_GGCCGTCGTGGTCTTCGACG	ERCC2__NM_000400	127	70	72	62
ERCC2__NM_000400_GTAGCTATAAACCACCACAT	ERCC2__NM_000400	151	0	179	5
ERCC2__NM_000400_TAACCTCATAGAATCGGCAG	ERCC2__NM_000400	396	178	485	150
ERCC2__NM_000400_TCGGGGTAGATGTAGTCGTA	ERCC2__NM_000400	484	0	621	17
ERCC2__NM_000400_TCTTGAGCAGTAGATGAGTT	ERCC2__NM_000400	416	5	567	75
ERCC2__NM_000400_TTCGACGAGGCCCACAACAT	ERCC2__NM_000400	167	7	167	64
ERCC2__NM_001130867_CGAGGCCCACAACATTGGTG	ERCC2__NM_001130867	64	1	84	38
ERCC2__NM_001130867_GAGTCGATGCAGACGTTGTC	ERCC2__NM_001130867	558	288	538	395
ERCC3__NM_000122_ACTCATGCACATGGGTTGGT	ERCC3__NM_000122	1141	6	1268	148
ERCC3__NM_000122_ATAAACTGCATAATTCCATC	ERCC3__NM_000122	339	397	364	306
ERCC3__NM_000122_CAGGCTGCAAATGCCGCTGA	ERCC3__NM_000122	567	291	583	287
ERCC3__NM_000122_CAGTTGTGTACTGTCAGCTA	ERCC3__NM_000122	379	0	472	15
ERCC3__NM_000122_CCCCTTCAGAGTTTCTTAAG	ERCC3__NM_000122	127	21	121	30
ERCC3__NM_000122_GACAGCTGCATACAAGGAGT	ERCC3__NM_000122	167	0	241	0
ERCC3__NM_000122_GATGCTGGATTACATCAGGG	ERCC3__NM_000122	358	81	466	55
ERCC3__NM_000122_GCTGAGCTTCCTGAGGTACT	ERCC3__NM_000122	1054	107	1217	95
ERCC3__NM_000122_GGGATGTCAGATTTACCCTG	ERCC3__NM_000122	548	176	623	147
ERCC3__NM_000122_TCTTGGGCATATTTGTAAAC	ERCC3__NM_000122	88	36	116	75
ERCC3__NM_000122_TGTATGCAGCTGTCAGCGTT	ERCC3__NM_000122	223	62	169	31
ERCC3__NM_000122_TTGTACTCATGCACATGGGT	ERCC3__NM_000122	236	91	270	97
ERCC4__NM_005236_AAAATGAGTTTACTGCTACA	ERCC4__NM_005236	640	524	668	669
ERCC4__NM_005236_ACAAGACTCGATTATTCTGT	ERCC4__NM_005236	847	647	968	615
ERCC4__NM_005236_ATGACAAAAACCAGTATCAA	ERCC4__NM_005236	206	160	211	262
ERCC4__NM_005236_CGCTATGAAGTTTACACACA	ERCC4__NM_005236	993	960	1304	1326
ERCC4__NM_005236_GACAAGACTCGATTATTCTG	ERCC4__NM_005236	431	358	504	577
ERCC4__NM_005236_GCAGCTGGAGAAAGTGGTAG	ERCC4__NM_005236	383	289	447	298
ERCC4__NM_005236_GCCATGGCAATCCGTCGAGC	ERCC4__NM_005236	1075	977	1236	1099
ERCC4__NM_005236_GCCGGCTCGACGGATTGCCA	ERCC4__NM_005236	1358	1246	1497	1282
ERCC4__NM_005236_GTGTTATATTTGCGACAAGT	ERCC4__NM_005236	72	85	96	250
ERCC4__NM_005236_TGGAACTGCTCGACACTGAC	ERCC4__NM_005236	46	129	91	67
ERCC4__NM_005236_TTGTGAGGAAACTGTATCTG	ERCC4__NM_005236	283	247	406	358
ERCC4__NM_005236_TTTCATTTGTTACACGGCGA	ERCC4__NM_005236	53	26	63	71
ERCC5__NM_000123_AAAGGAGTCCGGGATCGCCA	ERCC5__NM_000123	572	343	623	415
ERCC5__NM_000123_AAATAATGTTCTTCTGCGCT	ERCC5__NM_000123	373	668	452	544
ERCC5__NM_000123_AACATTCAGGACACATCCGA	ERCC5__NM_000123	614	311	817	528
ERCC5__NM_000123_ACGTAGCCAGCACTAATGAG	ERCC5__NM_000123	339	406	344	335
ERCC5__NM_000123_CCTTCTTCCAGCAAAATGCA	ERCC5__NM_000123	209	218	143	246
ERCC5__NM_000123_GAATCAAAAACAAGCATTAC	ERCC5__NM_000123	305	263	388	314
ERCC5__NM_000123_GACTCAATATCTATCGCTTG	ERCC5__NM_000123	1231	1142	1443	1426
ERCC5__NM_000123_GGAGAAGGAGGGGTAGCATC	ERCC5__NM_000123	239	89	224	75
ERCC5__NM_000123_GGAGAGTGAAAATCGAAGGC	ERCC5__NM_000123	457	551	508	490
ERCC5__NM_000123_TCAGCCCCGAAGCGCTGGAA	ERCC5__NM_000123	314	314	365	437
ERCC5__NM_000123_TCCTTCATCACTGTTCTCGG	ERCC5__NM_000123	634	485	552	371
ERCC5__NM_000123_TTTGGACATGTTCTATATGC	ERCC5__NM_000123	107	162	146	155
ERCC6__NM_000124_ACTAGATCACGCCAGTCTGG	ERCC6__NM_000124	763	675	694	663
ERCC6__NM_000124_ATCAGGCTTCGAAAAGTATT	ERCC6__NM_000124	283	586	323	439
ERCC6__NM_000124_CAGAGTTCTGTCCAAAAAAG	ERCC6__NM_000124	70	151	118	154
ERCC6__NM_000124_CCCCCACTCAAGTCAAACTC	ERCC6__NM_000124	493	559	414	511
ERCC6__NM_000124_CGTGGAGAAGGAGTATCGGT	ERCC6__NM_000124	1006	1200	1094	1132
ERCC6__NM_000124_CTACTGCCTGGATCTGATGT	ERCC6__NM_000124	947	1069	921	727
ERCC6__NM_000124_GAAGGAGTATCGGTCGGTCC	ERCC6__NM_000124	760	1026	974	822
ERCC6__NM_000124_GACCAGGACGTGCTGGAACA	ERCC6__NM_000124	465	513	453	472
ERCC6__NM_000124_GAGCCGGGGCCATCCAGTCT	ERCC6__NM_000124	1055	1342	1260	1535
ERCC6__NM_000124_GGTCATGTACGACATCCCTA	ERCC6__NM_000124	594	360	646	602
ERCC6__NM_000124_TGAACTAGATCACGCCAGTC	ERCC6__NM_000124	327	310	383	377
ERCC6__NM_000124_TGAGGGATCTGGGTACCAAA	ERCC6__NM_000124	133	143	252	227
ERCC6L__NM_017669_AACCTTTCATGGTCCTAGCA	ERCC6L__NM_017669	244	300	257	459
ERCC6L__NM_017669_AACGGACCAGAAACCTCAAT	ERCC6L__NM_017669	542	182	482	180
ERCC6L__NM_017669_AAGTGATGCATCAAACATAC	ERCC6L__NM_017669	199	59	230	33
ERCC6L__NM_017669_AGCAGGCTGCTCATTACCTA	ERCC6L__NM_017669	577	181	662	228
ERCC6L__NM_017669_ATGCATCAAACATACCGGAA	ERCC6L__NM_017669	287	282	310	357
ERCC6L__NM_017669_ATGGTCCTAGCAAGGATGAA	ERCC6L__NM_017669	69	40	76	9
ERCC6L__NM_017669_CAACCAATCTTATTAACACA	ERCC6L__NM_017669	1014	745	920	1169
ERCC6L__NM_017669_CATCCCTATACAGGCTATAG	ERCC6L__NM_017669	369	163	449	79
ERCC6L__NM_017669_CATGTGTTAATAAGATTGGT	ERCC6L__NM_017669	682	216	854	141
ERCC6L__NM_017669_CTCTATAGCCTGTATAGGGA	ERCC6L__NM_017669	34	4	27	51
ERCC6L__NM_017669_CTTTCCTCTATAGCCTGTAT	ERCC6L__NM_017669	503	564	600	540
ERCC6L__NM_017669_TTTCCTCTATAGCCTGTATA	ERCC6L__NM_017669	98	58	84	74
ERCC6L2__NM_001010895_AATTAATTCGTGTAAAGCAG	ERCC6L2__NM_001010895	106	115	140	242
ERCC6L2__NM_001010895_ACTTCTTGAAGCTGTTTAAG	ERCC6L2__NM_001010895	201	249	244	253
ERCC6L2__NM_001010895_AGTAGGTCCCACTCCCTAAA	ERCC6L2__NM_001010895	69	77	73	128
ERCC6L2__NM_001010895_CAGCCAGAAATGAAATAACC	ERCC6L2__NM_001010895	434	223	454	463
ERCC6L2__NM_001010895_CTTTGCCTGAGGTTTCCGCG	ERCC6L2__NM_001010895	1633	2059	2045	2122
ERCC6L2__NM_001010895_GAAGGAACCCGGTTTCTTTA	ERCC6L2__NM_001010895	119	130	168	207
ERCC6L2__NM_001010895_GAAGGAACTGTGGTGTGTTA	ERCC6L2__NM_001010895	335	478	433	568
ERCC6L2__NM_001010895_GGAATGGTCAGCTGTCATTG	ERCC6L2__NM_001010895	631	641	702	777
ERCC6L2__NM_001010895_TGAAACACTACGCTTATGCC	ERCC6L2__NM_001010895	165	194	273	306
ERCC6L2__NM_001010895_TTCTGTCCTCTACAACTGGA	ERCC6L2__NM_001010895	22	17	25	22
ERCC6L2__NM_001010895_TTGCCTGAGGTTTCCGCGCG	ERCC6L2__NM_001010895	317	283	345	456
ERCC6L2__NM_001010895_TTTGCCTGAGGTTTCCGCGC	ERCC6L2__NM_001010895	579	403	636	568
ERCC8__NM_000082_ACAGCAATGGTGCTACCATA	ERCC8__NM_000082	209	251	202	221
ERCC8__NM_000082_ACCAAGCACTGTTTGGTAGC	ERCC8__NM_000082	1041	1210	989	1346
ERCC8__NM_000082_AGTCTCCACCAAGCACTGTT	ERCC8__NM_000082	366	383	386	398
ERCC8__NM_000082_CTGACAGTAGAGTAAAATTA	ERCC8__NM_000082	128	154	108	125
ERCC8__NM_000082_GAACAGATAACTATGCTTAA	ERCC8__NM_000082	231	346	232	301
ERCC8__NM_000082_GATGTTGAAAGAATCCACGG	ERCC8__NM_000082	508	317	506	319
ERCC8__NM_000082_GCCAAGATATAGTCATAACG	ERCC8__NM_000082	492	519	420	609
ERCC8__NM_000082_GGGTGTTAATTCCACCGCCG	ERCC8__NM_000082	245	358	286	350
ERCC8__NM_000082_GTCCGCACGCCAAACGGGTT	ERCC8__NM_000082	1023	958	1196	1077
ERCC8__NM_000082_TCCTGTTCTCACATTCTACA	ERCC8__NM_000082	325	368	474	514
ERCC8__NM_000082_TGATGTTCACAGATACAGTG	ERCC8__NM_000082	231	259	210	284
ERCC8__NM_000082_TGTAAAGCAGTGTGTTCCAT	ERCC8__NM_000082	768	562	839	732
EREG__NM_001432_AAGTGTAGCTCTGACATGAA	EREG__NM_001432	53	62	41	47
EREG__NM_001432_AGAGTTGCCGCAAGTCTGAA	EREG__NM_001432	241	387	271	384
EREG__NM_001432_AGGTGTGAAGTGGGTTATAC	EREG__NM_001432	238	350	296	261
EREG__NM_001432_GACTCTCCTGGGATACATGA	EREG__NM_001432	559	574	576	592
EREG__NM_001432_GCATGGACAGTGCATCTATC	EREG__NM_001432	802	1033	841	1051
EREG__NM_001432_GCCACATATTCTTTGCTTAA	EREG__NM_001432	1515	2047	1812	2273
EREG__NM_001432_TGGAGATGCTCTGTGCCGGC	EREG__NM_001432	216	108	204	224
ERF__NM_006494_AGTACCAGGGCGTCATTGCC	ERF__NM_006494	1018	1480	1057	1129
ERF__NM_006494_AGTTGTAGACGCTTTGGGTG	ERF__NM_006494	1017	1122	1242	1608
ERF__NM_006494_ATTGCCTGGCAGGGGGACTA	ERF__NM_006494	1426	1997	1648	2008
ERF__NM_006494_CAGCTTGTCGTAATTCATCT	ERF__NM_006494	3163	4212	3373	4229
ERF__NM_006494_CCAGCTGTGGCACTTTATCC	ERF__NM_006494	2601	1932	2992	2662
ERF__NM_006494_CGGAAGTGGCTACCACCCGA	ERF__NM_006494	2524	2313	2890	2834
ERF__NM_006494_CTGGAGCTTAAACTTGAATG	ERF__NM_006494	501	383	445	388
ERF__NM_006494_GACCGGCCAGAGGCGACACA	ERF__NM_006494	1000	853	1021	983
ERF__NM_006494_GCGCATTCTGCACAAGACCA	ERF__NM_006494	884	912	874	1200
ERF__NM_006494_GCTTGTTATAGTAATAGCTG	ERF__NM_006494	237	277	283	400
ERF__NM_006494_GGCCAGAGGCGACACAGGGA	ERF__NM_006494	1052	1183	1237	1482
ERF__NM_006494_GGGCTTCCAGTTTGGTTTCA	ERF__NM_006494	860	1416	1016	1165
ERF__NM_006494_GGTCAAGCCTTATTCTGTAT	ERF__NM_006494	1598	2040	1866	2325
ERF__NM_006494_TCAAGCCTTATTCTGTATTG	ERF__NM_006494	371	420	536	547
ERF__NM_006494_TCAGCTTGTCGTAATTCATC	ERF__NM_006494	700	735	837	941
ERF__NM_006494_TGCTCCTAGGACTGGCCCAT	ERF__NM_006494	305	478	360	545
ERF__NM_006494_TGTAGAGATTTGTGGCCCAT	ERF__NM_006494	536	537	686	607
ERG__NM_001136154_AATGGAATGTAACCCTAGCC	ERG__NM_001136154	1345	2142	1494	1916
ERG__NM_001136154_ACTCCAGCCACTGCCGCACA	ERG__NM_001136154	3634	4108	3945	3732
ERG__NM_001136154_AGCCAGGTGTGGCGTTCCGT	ERG__NM_001136154	3590	4366	3873	3762
ERG__NM_001136154_AGGCACACTCAAACAACGAC	ERG__NM_001136154	414	380	477	601
ERG__NM_001136154_CACGCGTCCCTCAGCAGGAT	ERG__NM_001136154	2109	2339	2409	2810
ERG__NM_001136154_GCGTCCTCCTCCAGCGACTA	ERG__NM_001136154	1817	2097	2068	2580
ERG__NM_001136154_GCTGCCGTAGTTCATCCCAA	ERG__NM_001136154	2073	2656	2409	2833
ERG__NM_001136154_GGAAGTCTGTCCATAGTCGC	ERG__NM_001136154	762	1114	850	1056
ERG__NM_001136154_TGCCTACGGAACGCCACACC	ERG__NM_001136154	1131	1562	1266	1679
ERG__NM_001136154_TGGGCAGCCCAGACACCGTT	ERG__NM_001136154	1876	2108	2004	2116
ERG__NM_001136154_TGGTCTGTACTCCATAGCGT	ERG__NM_001136154	0	0	0	0
ERG__NM_001136154_TTACCTTGATATGAGCTGCT	ERG__NM_001136154	346	450	448	465
ERG__NM_001136155_ACAAGATGTTGACGTCTGGA	ERG__NM_001136155	969	1123	1057	1264
ERG__NM_001136155_ACATCATCTGAAGTCAAATG	ERG__NM_001136155	804	904	955	1193
ERG__NM_001136155_AGGATGTCGGCGTTGTAGCT	ERG__NM_001136155	597	407	675	842
ERG__NM_001136155_ATTCCAGAACATCGATGGGA	ERG__NM_001136155	1049	929	1296	1242
ERG__NM_001136155_CTACGCAAAGAATTACAACT	ERG__NM_001136155	753	800	840	1123
ERG__NM_001136155_GAGTGGGCGGTGAAAGAATA	ERG__NM_001136155	504	504	626	729
ERG__NM_001136155_GATCTCCTGGGGGGCTCATA	ERG__NM_001136155	837	1088	851	875
ERG__NM_001136155_TTTCTAGCATGCATTAACCG	ERG__NM_001136155	207	232	250	250
ERG__NM_001243429_GAAGACCAGCGTCCTCAGTT	ERG__NM_001243429	2386	2857	2877	2929
ERG__NM_182918_TGGCCAGCACTATTAAGGTA	ERG__NM_182918	123	206	134	126
ERGIC1__NM_001031711_AGTGGTTGGGCTTGACATTC	ERGIC1__NM_001031711	283	275	240	208
ERGIC1__NM_001031711_CACTTCTGTCGTTATAAATC	ERGIC1__NM_001031711	468	394	402	288
ERGIC1__NM_001031711_CATAGAGCTCGTTCACACTA	ERGIC1__NM_001031711	363	389	492	388
ERGIC1__NM_001031711_CGGAGCTTTCAATGCTCTCG	ERGIC1__NM_001031711	806	818	894	1168
ERGIC1__NM_001031711_CGGTGTACGTTGGCTGCGTA	ERGIC1__NM_001031711	419	808	397	445
ERGIC1__NM_001031711_CTATGTCGATGACCCAGACA	ERGIC1__NM_001031711	333	318	461	587
ERGIC1__NM_001031711_CTTACGCAGCCAACGTACAC	ERGIC1__NM_001031711	0	0	0	0
ERGIC1__NM_001031711_GAGATGGGCAGGCACGAAGT	ERGIC1__NM_001031711	63	58	64	102
ERGIC1__NM_001031711_GGATGACATGCGTCATGTCT	ERGIC1__NM_001031711	242	399	287	356
ERGIC1__NM_001031711_TACGCAGCCAACGTACACCG	ERGIC1__NM_001031711	1140	1169	1300	984
ERGIC1__NM_001031711_TGAAGATCCCGCTGAACAAT	ERGIC1__NM_001031711	506	631	576	703
ERGIC1__NM_001031711_TTACGCAGCCAACGTACACC	ERGIC1__NM_001031711	741	642	609	623
ERGIC2__NM_016570_ACTTCAGCCAGTGGAGGTAC	ERGIC2__NM_016570	628	622	663	829
ERGIC2__NM_016570_AGACTCTGTGGTATTGTTGG	ERGIC2__NM_016570	418	528	482	576
ERGIC2__NM_016570_AGATGTTGGAGCGGATGTAT	ERGIC2__NM_016570	919	1200	1119	1125
ERGIC2__NM_016570_ATATGATCTCAGTTCTCTTA	ERGIC2__NM_016570	322	543	400	288
ERGIC2__NM_016570_ATTTGCTGTCGTTTCAGACT	ERGIC2__NM_016570	388	617	523	596
ERGIC2__NM_016570_CTATATGTCAATAAAGTAGC	ERGIC2__NM_016570	197	257	251	298
ERGIC2__NM_016570_CTGAGGAGCACATGCCATTC	ERGIC2__NM_016570	3101	3259	3294	3044
ERGIC2__NM_016570_GCTCTCAGGAACCTTCGGAA	ERGIC2__NM_016570	412	262	368	342
ERGIC2__NM_016570_GTATGAATACGAAGTAGACA	ERGIC2__NM_016570	137	154	130	139
ERGIC2__NM_016570_TCTTATGGTGACAGTTACTG	ERGIC2__NM_016570	323	236	380	283
ERGIC2__NM_016570_TTCTGTCACAGAAAACTGAT	ERGIC2__NM_016570	0	0	0	0
ERGIC3__NM_015966_AAGCAACGACTAGATAAAGA	ERGIC3__NM_015966	130	127	141	128
ERGIC3__NM_015966_AGCAGCTCTCACAGCGATCA	ERGIC3__NM_015966	317	211	314	347
ERGIC3__NM_015966_AGCTCTACGTGGACAAGTCG	ERGIC3__NM_015966	206	160	213	331
ERGIC3__NM_015966_ATTGAGCAGTGCCGGCGAGA	ERGIC3__NM_015966	335	270	347	276
ERGIC3__NM_015966_CGCTCAGCCTCTGAGCTCAC	ERGIC3__NM_015966	530	834	521	742
ERGIC3__NM_015966_CGGGAGGCATATCGCCGTAG	ERGIC3__NM_015966	296	196	309	265
ERGIC3__NM_015966_CTCTACGTGGACAAGTCGCG	ERGIC3__NM_015966	155	252	229	192
ERGIC3__NM_015966_CTTAGAGCTTGGGAAAGTCG	ERGIC3__NM_015966	301	289	299	460
ERGIC3__NM_015966_GGCACTGCTCAATAGTATCT	ERGIC3__NM_015966	741	669	721	591
ERGIC3__NM_015966_GGCATATCGCCGTAGAGGCT	ERGIC3__NM_015966	494	368	440	613
ERGIC3__NM_015966_TATCTAGTCGTTGCTTGAAC	ERGIC3__NM_015966	112	50	129	101
ERGIC3__NM_015966_TGGCTTCTTGGAAGTCAATA	ERGIC3__NM_015966	0	0	1	0
ERH__NM_004450_ACCAGCCTTATAACAAAGAC	ERH__NM_004450	443	552	598	774
ERH__NM_004450_AGAAAAAGAGGAGGTAACGG	ERH__NM_004450	155	81	190	95
ERH__NM_004450_AGATCTACGTGCTCCTTCGT	ERH__NM_004450	650	119	704	115
ERH__NM_004450_CCTGTTGGGCCTGCCGACGA	ERH__NM_004450	507	278	555	232
ERH__NM_004450_CTACGTGCTCCTTCGTCGGC	ERH__NM_004450	505	937	646	860
ERH__NM_004450_TATGTGATAGAGGGACTGTT	ERH__NM_004450	190	18	193	19
ERH__NM_004450_TCATCTAATACAGTCAATCT	ERH__NM_004450	652	470	834	755
ERH__NM_004450_TGTTATAAGGCTGGTATGTC	ERH__NM_004450	272	48	298	150
ERH__NM_004450_TTGGGGTGGGCTTGGAACAC	ERH__NM_004450	131	34	125	21
ERI1__NM_153332_AAAGAGATTGCCATTACGAA	ERI1__NM_153332	160	283	188	234
ERI1__NM_153332_ACATTCAGTGTCAACTCAGC	ERI1__NM_153332	496	561	531	532
ERI1__NM_153332_ATCAATATTCGGAAGTCATA	ERI1__NM_153332	79	45	92	190
ERI1__NM_153332_ATTAATACAGCCATTCGTAA	ERI1__NM_153332	284	295	240	277
ERI1__NM_153332_CGAAAAAGTGGATCAATATT	ERI1__NM_153332	921	859	963	1107
ERI1__NM_153332_CGCACTGGAGGTAATGAACT	ERI1__NM_153332	2169	2526	2305	2369
ERI1__NM_153332_GATTTCTGCATCAGTCTAAC	ERI1__NM_153332	308	452	308	445
ERI1__NM_153332_GCATTATTGTCAGTTTGGTT	ERI1__NM_153332	673	1013	700	1078
ERI1__NM_153332_GTCACTGAAGTCACTCGCAC	ERI1__NM_153332	840	1143	835	1181
ERI1__NM_153332_GTCCTCTTCCTTACCAATAG	ERI1__NM_153332	88	205	173	141
ERI1__NM_153332_TGGCAATCTCTTTGTAAACC	ERI1__NM_153332	21	14	21	29
ERI1__NM_153332_TTCAAGCATTATTGTCAGTT	ERI1__NM_153332	40	56	60	77
ERI2__NM_001142725_AATTACAAGGTCGTTGAACA	ERI2__NM_001142725	39	14	40	87
ERI2__NM_001142725_ACCCTGGATGCTAATGTTAC	ERI2__NM_001142725	450	451	418	414
ERI2__NM_001142725_ACTTACTTATTTCCTGGCTA	ERI2__NM_001142725	551	586	795	787
ERI2__NM_001142725_AGAAAACCAAAAGGACTAAG	ERI2__NM_001142725	1	0	14	0
ERI2__NM_001142725_AGGCTCCTTATTATATATGC	ERI2__NM_001142725	216	221	256	235
ERI2__NM_001142725_CCTGAGGTTGAACATAAGCC	ERI2__NM_001142725	439	507	431	413
ERI2__NM_001142725_GATGTGTTCAGCAACACTGC	ERI2__NM_001142725	512	441	693	469
ERI2__NM_001142725_GCCTGTAACATTAGCATCCA	ERI2__NM_001142725	1000	983	1116	1166
ERI2__NM_001142725_GGCTCCTTATTATATATGCT	ERI2__NM_001142725	243	325	249	293
ERI2__NM_001142725_TGATCCTGTTGTGCCTTTAT	ERI2__NM_001142725	245	289	427	384
ERI2__NM_001142725_TGATCTCAGAGCAACTTACA	ERI2__NM_001142725	238	292	160	194
ERI2__NM_001142725_TTCTCAGGACGAGAACATTC	ERI2__NM_001142725	0	0	0	0
ERI2__NM_080663_TCCAGGTCCACGAAGGCAAG	ERI2__NM_080663	155	137	179	218
ERI3__NM_024066_ACTTGGATGGGCCCGAGTTG	ERI3__NM_024066	858	864	905	642
ERI3__NM_024066_AGGCTGGACATACATGTGAA	ERI3__NM_024066	470	280	554	449
ERI3__NM_024066_CATGGATGCGCCAAAGCCGT	ERI3__NM_024066	664	599	674	439
ERI3__NM_024066_CCACGGCTTTGGCGCATCCA	ERI3__NM_024066	1005	1115	1258	1153
ERI3__NM_024066_CCGGCCATTTAGCTTTAGGA	ERI3__NM_024066	1031	658	890	743
ERI3__NM_024066_GATGAATGGATGGCGAAGGA	ERI3__NM_024066	264	213	212	195
ERI3__NM_024066_GGCCATTTAGCTTTAGGATG	ERI3__NM_024066	399	350	376	568
ERI3__NM_024066_GTAAGCTGTGGATGGACTAC	ERI3__NM_024066	805	818	820	841
ERI3__NM_024066_GTCCAGGGGAGAGTAAGGGG	ERI3__NM_024066	252	42	330	131
ERI3__NM_024066_GTCTTGGCATCTTCGAAGTA	ERI3__NM_024066	307	224	310	239
ERI3__NM_024066_TCCAGAAGCATCTAAAACTT	ERI3__NM_024066	611	356	638	380
ERI3__NM_024066_TTCCCCATCCTAAAGCTAAA	ERI3__NM_024066	29	0	33	33
ERICH1__NM_207332_AGACGTTAAAGATACCAGGG	ERICH1__NM_207332	163	262	184	226
ERICH1__NM_207332_AGGAAGACCCGACATGGGCT	ERICH1__NM_207332	815	1048	973	1335
ERICH1__NM_207332_AGGAAGACGTTAAAGACGCC	ERICH1__NM_207332	368	507	299	445
ERICH1__NM_207332_AGGAAGACGTTAAAGATACC	ERICH1__NM_207332	87	116	87	119
ERICH1__NM_207332_CAGCGAGGAAGACCCGACAT	ERICH1__NM_207332	1388	1975	1442	2102
ERICH1__NM_207332_CCAGCGAGGAAGACCCGACA	ERICH1__NM_207332	595	926	673	931
ERICH1__NM_207332_CGCTGGCAGTGTAGAGCCGT	ERICH1__NM_207332	583	740	642	929
ERICH1__NM_207332_GCAGCATGCTGTGTGACGCA	ERICH1__NM_207332	82	67	81	66
ERICH1__NM_207332_GGAAGACGTTAAAGACGCCA	ERICH1__NM_207332	240	191	259	380
ERICH1__NM_207332_GGAAGACGTTAAAGATACCA	ERICH1__NM_207332	281	395	373	442
ERICH1__NM_207332_GTAGAGCCGTCGGGCAGTCG	ERICH1__NM_207332	1439	1447	1445	1950
ERICH1__NM_207332_TCTCAGCTTTCTTTAGTTAC	ERICH1__NM_207332	551	815	608	760
ERICH2__NM_001289947_CTTCTGCCATTAACTCTAGT	ERICH2__NM_001289947	812	737	854	1029
ERICH2__NM_001289947_TCAGGCCCCACTAGAGTTAA	ERICH2__NM_001289947	167	164	193	223
ERICH2__NM_001289947_TTCTGCCATTAACTCTAGTG	ERICH2__NM_001289947	34	110	68	75
ERICH2__NM_001290030_ACAGCAAAGAACAAACAGAA	ERICH2__NM_001290030	77	67	113	140
ERICH2__NM_001290030_ACATTTACAGAGACCTGTGT	ERICH2__NM_001290030	1002	860	1069	1231
ERICH2__NM_001290030_AGGAAAGAAGGTTGGAATGT	ERICH2__NM_001290030	563	491	727	711
ERICH2__NM_001290030_CGCGTGCGCACGTCAATCCG	ERICH2__NM_001290030	838	709	879	999
ERICH2__NM_001290030_CGGGGAACCACGGAGAGGCA	ERICH2__NM_001290030	584	501	604	612
ERICH2__NM_001290030_GCGTGCGCACGTCAATCCGA	ERICH2__NM_001290030	1261	1929	1277	1451
ERICH2__NM_001290030_GTCCCGCGGCGCCGGAAACT	ERICH2__NM_001290030	1613	1510	1568	1703
ERICH2__NM_001290030_TTGCTAATGTTTGATCCAAA	ERICH2__NM_001290030	119	124	151	261
ERICH3__NM_001002912_AGAAATTGGAGACCTTAGCT	ERICH3__NM_001002912	116	126	147	83
ERICH3__NM_001002912_AGGCGTCATCTCTTAAGATC	ERICH3__NM_001002912	283	296	285	329
ERICH3__NM_001002912_CAGCCAGGTGTTTATCCATA	ERICH3__NM_001002912	399	444	537	595
ERICH3__NM_001002912_CTAGGAAGGAGCGAATCCAG	ERICH3__NM_001002912	209	232	221	259
ERICH3__NM_001002912_CTTTGGGCCAACTGGTGGGT	ERICH3__NM_001002912	0	0	0	0
ERICH3__NM_001002912_GAACTTTATGAAAAATTGCC	ERICH3__NM_001002912	47	27	84	156
ERICH3__NM_001002912_GATTCGCTCCTTCCTAGCTA	ERICH3__NM_001002912	297	201	299	348
ERICH3__NM_001002912_GTTGGCCCAAAGAGTAATCG	ERICH3__NM_001002912	309	228	262	292
ERICH3__NM_001002912_TAATCGTGGCCATAGTGTTC	ERICH3__NM_001002912	990	1001	1036	967
ERICH3__NM_001002912_TATCCGGGAATGCTTAGCCC	ERICH3__NM_001002912	259	144	304	150
ERICH3__NM_001002912_TCTAGGGAGAGCACACAAGA	ERICH3__NM_001002912	110	66	75	105
ERICH3__NM_001002912_TGCTGCATATAATAGCCTTA	ERICH3__NM_001002912	142	122	161	169
ERICH4__NM_001130514_GATCCACAGCAGACTATCGC	ERICH4__NM_001130514	1491	1149	1767	1708
ERICH4__NM_001130514_GTGCCTGCGATAGTCTGCTG	ERICH4__NM_001130514	1038	1204	1216	1266
ERICH5__NM_173549_ACGTTCTCAGTTGTTCCTAG	ERICH5__NM_173549	535	385	478	409
ERICH5__NM_173549_ATGCTCATTTGAAGTTACTT	ERICH5__NM_173549	273	277	175	256
ERICH5__NM_173549_ATGGAGCAGATTCAACCTGA	ERICH5__NM_173549	650	525	702	719
ERICH5__NM_173549_CCCTTGGCTTCTGGTCCTAA	ERICH5__NM_173549	704	1158	818	1344
ERICH5__NM_173549_CTGTTGATGGCAATGTACAA	ERICH5__NM_173549	143	153	135	202
ERICH5__NM_173549_GCATCCAGCACGAAATGTAG	ERICH5__NM_173549	447	487	505	515
ERICH5__NM_173549_GGAAGAGAATCTACTGTTGA	ERICH5__NM_173549	954	902	971	1159
ERICH5__NM_173549_GGTTTAACACCATTAGCTGT	ERICH5__NM_173549	196	243	225	205
ERICH5__NM_173549_TCAGCAGAGCCTACAGCTAA	ERICH5__NM_173549	24	11	22	7
ERICH5__NM_173549_TCCTCCCTTACAAAAGCTCA	ERICH5__NM_173549	38	49	44	49
ERICH5__NM_173549_TGGAATGGATCATTCATAAC	ERICH5__NM_173549	36	70	51	31
ERICH5__NM_173549_TGTTGATGGCAATGTACAAA	ERICH5__NM_173549	140	121	194	143
ERICH6__NM_152394_AAAGAAGAGAGCTCCAATTT	ERICH6__NM_152394	264	330	317	222
ERICH6__NM_152394_AATGGATGGTGGTCCGATGC	ERICH6__NM_152394	396	399	331	386
ERICH6__NM_152394_CAATTTAGGCATTAACTTCA	ERICH6__NM_152394	432	660	608	722
ERICH6__NM_152394_CATCGGACCACCATCCATTC	ERICH6__NM_152394	1733	1888	1898	1597
ERICH6__NM_152394_CGAACGTAGAGGTCAGGCTA	ERICH6__NM_152394	475	375	470	468
ERICH6__NM_152394_CTAGCGGCTTCGGAGACCCG	ERICH6__NM_152394	517	558	626	383
ERICH6__NM_152394_CTGGCTGGGCACGAACGTAG	ERICH6__NM_152394	363	466	358	353
ERICH6__NM_152394_GCACTTTATCAGACAAATCT	ERICH6__NM_152394	477	279	444	513
ERICH6__NM_152394_TGGGCACGAACGTAGAGGTC	ERICH6__NM_152394	29	7	19	9
ERICH6__NM_152394_TGTCTGAAATGAGTATAGAT	ERICH6__NM_152394	125	285	136	124
ERICH6__NM_152394_TGTCTGGCATCTAAGTTAAG	ERICH6__NM_152394	144	133	179	198
ERICH6__NM_152394_TTGCGCTCGCCTAGCGGCTT	ERICH6__NM_152394	109	188	178	190
ERICH6B__NM_182542_AAGGGGTAGCGAAAGTCAAC	ERICH6B__NM_182542	118	187	127	110
ERICH6B__NM_182542_ACTAGATGAGGAAAGTCTAC	ERICH6B__NM_182542	214	237	222	130
ERICH6B__NM_182542_AGAAGGTGGTCACCTGAGAG	ERICH6B__NM_182542	40	25	28	23
ERICH6B__NM_182542_CCATGCTTCTTCGTGATGCA	ERICH6B__NM_182542	567	726	709	779
ERICH6B__NM_182542_CTGGAAGAGGAAGAGTATCT	ERICH6B__NM_182542	351	546	514	489
ERICH6B__NM_182542_GAAGAAATCATATCTAGAAG	ERICH6B__NM_182542	245	286	282	290
ERICH6B__NM_182542_GAAGGGGTAGCGAAAGTCAA	ERICH6B__NM_182542	123	189	188	141
ERICH6B__NM_182542_GCTGAAAATAATCAGTTATC	ERICH6B__NM_182542	364	415	433	724
ERICH6B__NM_182542_TCTGGAGGACAAAGAGTATC	ERICH6B__NM_182542	116	205	135	192
ERICH6B__NM_182542_TCTGGGGAAGAAAGCGTATC	ERICH6B__NM_182542	248	219	307	305
ERICH6B__NM_182542_TTGAGGAGGTAGATTATCTG	ERICH6B__NM_182542	452	671	513	687
ERICH6B__NM_182542_TTGTGATGAATAACTTGCCT	ERICH6B__NM_182542	242	328	404	464
ERLEC1__NM_001127397_AATATTCACGAGTACTACCT	ERLEC1__NM_001127397	368	297	300	321
ERLEC1__NM_001127397_ACCAGAGAGAACTCGGTGCC	ERLEC1__NM_001127397	612	884	741	751
ERLEC1__NM_001127397_ATACTTCCCCTTGTGACAAG	ERLEC1__NM_001127397	231	267	209	325
ERLEC1__NM_001127397_CCGGGCCAGTTGACTCGGAA	ERLEC1__NM_001127397	848	1391	1076	1213
ERLEC1__NM_001127397_CCTCATCCCCACTTGTCACA	ERLEC1__NM_001127397	818	1149	1058	1259
ERLEC1__NM_001127397_CCTTTCCGAGTCAACTGGCC	ERLEC1__NM_001127397	626	694	839	846
ERLEC1__NM_001127397_GATGACACCATACTATCCTG	ERLEC1__NM_001127397	897	1070	1082	1213
ERLEC1__NM_001127397_GGGATGTCATCGCTGAGTTG	ERLEC1__NM_001127397	398	612	465	508
ERLEC1__NM_001127397_TACTTCCCCTTGTGACAAGT	ERLEC1__NM_001127397	880	665	985	939
ERLEC1__NM_001127397_TCATCCCCACTTGTCACAAG	ERLEC1__NM_001127397	660	749	845	962
ERLEC1__NM_001127397_TCTTGGCCAACATATTCCCA	ERLEC1__NM_001127397	249	168	205	198
ERLEC1__NM_001127397_TGTCATCGCTGAGTTGAGGA	ERLEC1__NM_001127397	221	177	217	178
ERLIN1__NM_001100626_AATATGTTGGCTCCTTATGC	ERLIN1__NM_001100626	362	282	446	443
ERLIN1__NM_001100626_AATGAATATGACTCAAGCCC	ERLIN1__NM_001100626	290	308	311	344
ERLIN1__NM_001100626_CGCCTCCATCCACAAGATTG	ERLIN1__NM_001100626	409	378	496	413
ERLIN1__NM_001100626_CTATATTGACCGAATAGAAG	ERLIN1__NM_001100626	95	79	179	168
ERLIN1__NM_001100626_CTTCAGTGTTTGATATCGTG	ERLIN1__NM_001100626	683	744	694	567
ERLIN1__NM_001100626_GAGAGGAAAAAGGCAGTTAT	ERLIN1__NM_001100626	610	605	636	675
ERLIN1__NM_001100626_GATGGCCCTCCTCAATCTTG	ERLIN1__NM_001100626	875	660	992	702
ERLIN1__NM_001100626_GGCAACATGATATGATAGCC	ERLIN1__NM_001100626	132	252	151	230
ERLIN1__NM_001100626_TGCACAGAAACAAAAGGTTG	ERLIN1__NM_001100626	75	100	79	103
ERLIN1__NM_001100626_TTACCTGTATAGTGAGACCT	ERLIN1__NM_001100626	69	113	164	128
ERLIN1__NM_001100626_TTTGAGCTCCAGATATTCCG	ERLIN1__NM_001100626	474	646	516	456
ERLIN1__NM_001100626_TTTGTTTCTGTGCAGCTATA	ERLIN1__NM_001100626	24	60	18	14
ERLIN2__NM_001003790_AACTTCCTGGTCCCGAACGC	ERLIN2__NM_001003790	548	432	461	473
ERLIN2__NM_001003790_AAGATAGAAGAGGGACATAT	ERLIN2__NM_001003790	180	149	156	121
ERLIN2__NM_001003790_AGAACTGGTTCAGTTCGTGG	ERLIN2__NM_001003790	237	281	419	403
ERLIN2__NM_001003790_AGGTGAAGAATGTACCTTGT	ERLIN2__NM_001003790	834	612	801	647
ERLIN2__NM_001003790_CACATCATATAAGTCTGTGC	ERLIN2__NM_001003790	506	408	520	557
ERLIN2__NM_001003790_CAGTGTGCACACGCTTCAAG	ERLIN2__NM_001003790	324	187	379	297
ERLIN2__NM_001003790_CTACTTTGACAGAATTGAAG	ERLIN2__NM_001003790	81	31	118	74
ERLIN2__NM_001003790_GATAGAAGAGGGACATATTG	ERLIN2__NM_001003790	339	314	359	440
ERLIN2__NM_001003790_GCCGCTGGTCGAAGTCAGCA	ERLIN2__NM_001003790	492	747	790	813
ERLIN2__NM_001003790_GGAGCATGAGATGGAAACCA	ERLIN2__NM_001003790	352	304	287	300
ERLIN2__NM_001003790_TGATGAAAGGGAGCATGAGA	ERLIN2__NM_001003790	113	55	100	115
ERLIN2__NM_007175_GATTGATGAAAATCTCAAAC	ERLIN2__NM_007175	134	143	131	178
ERMAP__NM_001017922_AAGTTCCACGTGGCCCTACT	ERMAP__NM_001017922	553	480	576	663
ERMAP__NM_001017922_ACACATGCACAGACAGCCGG	ERMAP__NM_001017922	703	495	785	458
ERMAP__NM_001017922_AGATCTGATGCCGGAATATA	ERMAP__NM_001017922	398	255	507	360
ERMAP__NM_001017922_AGTTCCACGTGGCCCTACTA	ERMAP__NM_001017922	371	336	439	274
ERMAP__NM_001017922_ATCTGATGCCGGAATATAAG	ERMAP__NM_001017922	229	69	222	122
ERMAP__NM_001017922_CACCTCCTTGGGTACCGTCC	ERMAP__NM_001017922	820	870	837	641
ERMAP__NM_001017922_CGGCTGTCTGTGCATGTGTC	ERMAP__NM_001017922	777	1234	935	1222
ERMAP__NM_001017922_CTCGGCTGTGCCCCCTAGTA	ERMAP__NM_001017922	594	528	706	811
ERMAP__NM_001017922_CTGTTCACATATTCCGGGAT	ERMAP__NM_001017922	705	888	898	1081
ERMAP__NM_001017922_GATCTGATGCCGGAATATAA	ERMAP__NM_001017922	107	36	113	55
ERMAP__NM_001017922_GGATGCCGGCAAGTTCCACG	ERMAP__NM_001017922	1200	1696	1552	1816
ERMAP__NM_001017922_GTTCCACGTGGCCCTACTAG	ERMAP__NM_001017922	191	68	141	65
ERMAP__NM_018538_AAAAAAAGCTTCTACGTTGG	ERMAP__NM_018538	432	598	427	561
ERMAP__NM_018538_AAATAGGAAGTTGGTAGACT	ERMAP__NM_018538	221	211	249	172
ERMAP__NM_018538_AAGCTTTAGTCAAGAAGTTA	ERMAP__NM_018538	241	240	307	336
ERMAP__NM_018538_AAGGCCCGCCCACATTTGGG	ERMAP__NM_018538	801	873	849	817
ERMAP__NM_018538_ACTTCTTACTTATCCCATTG	ERMAP__NM_018538	1774	1583	1909	2044
ERMAP__NM_018538_ATGGGATAAGTAAGAAGTCA	ERMAP__NM_018538	106	142	173	141
ERMAP__NM_018538_ATTGGCTCACGTGATTATGG	ERMAP__NM_018538	61	0	44	0
ERMAP__NM_018538_CAAACAATCAGTTTAGGTGC	ERMAP__NM_018538	697	769	846	1035
ERMAP__NM_018538_CAGACTGTCCTCCCAAATGT	ERMAP__NM_018538	847	473	869	307
ERMAP__NM_018538_CATCTTGTGACCCAGTTAAG	ERMAP__NM_018538	317	282	390	248
ERMAP__NM_018538_CTGTCCAAAGGCTATTAGGC	ERMAP__NM_018538	161	222	209	248
ERMAP__NM_018538_GAAAAGAGTTCGCTACTCCA	ERMAP__NM_018538	322	396	482	457
ERMAP__NM_018538_GAAGAGACGTTCCATAATCA	ERMAP__NM_018538	319	285	338	416
ERMAP__NM_018538_GATGTGACTTGAAAGCGACG	ERMAP__NM_018538	339	557	419	501
ERMAP__NM_018538_GGAAAAAAAAGCTTCTACGT	ERMAP__NM_018538	264	125	314	116
ERMAP__NM_018538_GGAAACCAAACAATCAGTTT	ERMAP__NM_018538	1214	1203	1390	1662
ERMAP__NM_018538_GGAATTGGCTCACGTGATTA	ERMAP__NM_018538	734	408	683	358
ERMAP__NM_018538_GGCCTTGCTGAATCAGTTGA	ERMAP__NM_018538	1890	2342	2445	2998
ERMAP__NM_018538_GTCACATCATATATGCGATC	ERMAP__NM_018538	474	760	522	826
ERMAP__NM_018538_TATGCGATCTGGCCTAACCA	ERMAP__NM_018538	387	208	390	226
ERMAP__NM_018538_TGCAGTACAATGGTCTTGTG	ERMAP__NM_018538	412	366	480	517
ERMAP__NM_018538_TGGAAAACCTTGACTAATAC	ERMAP__NM_018538	52	66	73	44
ERMAP__NM_018538_TGGCAGCAGGCTTGACGCCA	ERMAP__NM_018538	5	0	7	0
ERMAP__NM_018538_TGGCCTAACCATGGAGATAT	ERMAP__NM_018538	14	14	39	41
ERMARD__NM_001278531_AAGAGCAGGGTCTGGATTAC	ERMARD__NM_001278531	607	606	831	754
ERMARD__NM_001278531_AATAGCATTGTAACTCAGAA	ERMARD__NM_001278531	104	55	105	179
ERMARD__NM_001278531_AATGTGCTAAAAGTCTTCGT	ERMARD__NM_001278531	766	660	894	774
ERMARD__NM_001278531_ACCAGTGCAACTTCCCAATA	ERMARD__NM_001278531	1117	1024	1268	1051
ERMARD__NM_001278531_ACCATATTGGGAAGTTGCAC	ERMARD__NM_001278531	575	636	647	658
ERMARD__NM_001278531_ATGATGATACTGTTGACGGC	ERMARD__NM_001278531	348	453	304	402
ERMARD__NM_001278531_CATATCTCTTACAAACCTCG	ERMARD__NM_001278531	51	63	43	37
ERMARD__NM_001278531_GGAGAAAGACATGTGGTAAT	ERMARD__NM_001278531	537	528	584	560
ERMARD__NM_001278531_TGCGTAACGTCTTATGGCAT	ERMARD__NM_001278531	1263	1373	1447	1630
ERMARD__NM_001278531_TGGTCAAGTTCAAGTCACAC	ERMARD__NM_001278531	13	5	3	12
ERMARD__NM_001278531_TTACGCAGGTTGAGACCACA	ERMARD__NM_001278531	345	398	448	425
ERMARD__NM_001278531_TTGCTGACACAACTGGAGAC	ERMARD__NM_001278531	378	561	463	655
ERMARD__NM_001278532_ATAAGAGATCATTTAAGCCA	ERMARD__NM_001278532	172	193	192	64
ERMARD__NM_001278532_CTTAAATGATCTCTTATGCG	ERMARD__NM_001278532	734	489	773	802
ERMARD__NM_001278532_GGCTTAAATGATCTCTTATG	ERMARD__NM_001278532	496	425	501	419
ERMN__NM_001009959_AGAGGTTTCTCAATTTAAAG	ERMN__NM_001009959	182	314	137	113
ERMN__NM_001009959_CTGATGAGCAGCCAACCTTA	ERMN__NM_001009959	0	0	0	0
ERMN__NM_001009959_GAGGAAAGAAGAAAATTACA	ERMN__NM_001009959	206	260	192	179
ERMN__NM_001009959_GGCAGCTGAAATTGAATGGC	ERMN__NM_001009959	693	806	715	841
ERMN__NM_001009959_TACCCAGGCTGAGTGTAATG	ERMN__NM_001009959	748	603	796	674
ERMN__NM_001009959_TAGAATGTAACATGTCAGCT	ERMN__NM_001009959	346	218	332	363
ERMN__NM_001009959_TTCTACCCTGTAGTGTGGCA	ERMN__NM_001009959	19	81	31	48
ERMP1__NM_024896_AGATTTCAGTAGATGTACAA	ERMP1__NM_024896	130	32	159	175
ERMP1__NM_024896_AGTAGAGCGTCGAGAGGGAG	ERMP1__NM_024896	239	198	250	243
ERMP1__NM_024896_CATCTACTGAAATCTTATGA	ERMP1__NM_024896	333	235	498	513
ERMP1__NM_024896_CGACTCAGAACCCCACTCCA	ERMP1__NM_024896	1451	1504	1808	1737
ERMP1__NM_024896_CTCGACGCTCTACTCCGACG	ERMP1__NM_024896	812	1002	964	1110
ERMP1__NM_024896_CTGTTCCAAAAGGTAGTGCA	ERMP1__NM_024896	68	65	101	193
ERMP1__NM_024896_CTTGAACACATAACTCCAT	ERMP1__NM_024896	951	1230	1294	1579
ERMP1__NM_024896_GCAGCAGCTCGTGCTACGCG	ERMP1__NM_024896	741	710	714	923
ERMP1__NM_024896_GCTGTGAGGCGGCACCGCGT	ERMP1__NM_024896	1135	1178	1320	1278
ERMP1__NM_024896_GTCGGAGTAGAGCGTCGAGA	ERMP1__NM_024896	537	610	527	552
ERMP1__NM_024896_TGCAGCAGCTCGTGCTACGC	ERMP1__NM_024896	273	101	220	240
ERMP1__NM_024896_TTCCAGCTTTACCACAACAT	ERMP1__NM_024896	44	39	83	90
ERN1__NM_001433_CGCTATCTGACCTTCATGTC	ERN1__NM_001433	2399	3247	2827	3529
ERN1__NM_001433_CTCGTGTACCATTGAGGGAG	ERN1__NM_001433	216	323	242	284
ERN1__NM_001433_CTCTCCCTCAATGGTACACG	ERN1__NM_001433	622	335	512	421
ERN1__NM_001433_CTTACTCTTCCACATGTGTT	ERN1__NM_001433	775	1187	869	1138
ERN1__NM_001433_CTTGTTGTTTGTGTCAACGC	ERN1__NM_001433	771	554	800	780
ERN1__NM_001433_GAAGCAGCAGACTTTGTCAT	ERN1__NM_001433	1966	2144	2043	2328
ERN1__NM_001433_GATGGCAGCCTGTATACGCT	ERN1__NM_001433	1047	610	1099	858
ERN1__NM_001433_GGGGATGCCTGCACCAATTC	ERN1__NM_001433	1225	1284	1357	1414
ERN1__NM_001433_TCGCCCAAGATACAGAAGAG	ERN1__NM_001433	132	179	253	289
ERN1__NM_001433_TCTTGCTTCCAAGCGTATAC	ERN1__NM_001433	149	153	228	180
ERN1__NM_001433_TGGGCAGAGACTATCTGCAA	ERN1__NM_001433	45	0	62	34
ERN1__NM_001433_TTGTTTGTGTCAACGCTGGA	ERN1__NM_001433	184	267	245	256
ERN2__NM_033266_CAGGTCCCTGGGTGCCTCCA	ERN2__NM_033266	904	577	988	423
ERN2__NM_033266_CCCAGGCGCTATGGCGAGTG	ERN2__NM_033266	543	930	557	612
ERN2__NM_033266_CCGCACTCGCCATAGCGCCT	ERN2__NM_033266	300	313	357	291
ERN2__NM_033266_CGCTATGGCGAGTGCGGTCA	ERN2__NM_033266	993	1046	1063	1397
ERN2__NM_033266_CTATGGCGAGTGCGGTCAGG	ERN2__NM_033266	1514	1568	1563	1439
ERN2__NM_033266_CTGTCTGCTTGCTTAGTGCG	ERN2__NM_033266	1207	1264	1500	1902
ERN2__NM_033266_GAGAAGGGCGGGGATCGGCG	ERN2__NM_033266	1028	996	1171	1003
ERN2__NM_033266_GCGCTATGGCGAGTGCGGTC	ERN2__NM_033266	1234	1300	1487	1772
ERN2__NM_033266_GGCGGAGCCTTCTCCGGGTC	ERN2__NM_033266	949	1307	1109	1164
ERN2__NM_033266_GTACATTGGTCCTTCGATGA	ERN2__NM_033266	356	360	446	515
ERN2__NM_033266_TCCACCCCCGCCTCTACAT	ERN2__NM_033266	2525	2381	2802	1974
ERN2__NM_033266_TTTGGGTCCCCAAGATGTAC	ERN2__NM_033266	12	3	47	45
ERO1L__NM_014584_AAATCTGGGCGCTCGAAGAA	ERO1L__NM_014584	104	127	145	175
ERO1L__NM_014584_AATTAAATCTGCGAGCTACA	ERO1L__NM_014584	233	270	257	308
ERO1L__NM_014584_CAAACAAGAATCCCCAGCCG	ERO1L__NM_014584	850	1100	938	1213
ERO1L__NM_014584_CTATTTCTTGTCTGGTTAGA	ERO1L__NM_014584	249	497	392	460
ERO1L__NM_014584_CTTAATCCTGAGCGCTACAC	ERO1L__NM_014584	300	311	372	390
ERO1L__NM_014584_GAGCAGTGGATGAATCTCTG	ERO1L__NM_014584	252	318	297	260
ERO1L__NM_014584_GTATCATTATTCAACGCATT	ERO1L__NM_014584	480	474	495	388
ERO1L__NM_014584_GTTAGATGGAATTCATAACT	ERO1L__NM_014584	343	219	344	277
ERO1L__NM_014584_TCAAGAATTATGGATTGTGT	ERO1L__NM_014584	466	582	514	644
ERO1L__NM_014584_TGACTACTTTAGGTATTACA	ERO1L__NM_014584	201	138	322	369
ERO1L__NM_014584_TGAGCGCTACACTGGTTACA	ERO1L__NM_014584	229	234	271	230
ERO1LB__NM_019891_AAAAGCTGGGCATTCTAATA	ERO1LB__NM_019891	207	188	240	326
ERO1LB__NM_019891_AAAGGACGATAAACAGATCG	ERO1LB__NM_019891	175	52	176	205
ERO1LB__NM_019891_AACGCTCTGGGTTCAGCAAT	ERO1LB__NM_019891	757	670	904	795
ERO1LB__NM_019891_ATTCCGGTTGGAATAAAAGC	ERO1LB__NM_019891	175	292	218	193
ERO1LB__NM_019891_CTGAACCCAGAGCGTTACAC	ERO1LB__NM_019891	799	852	803	817
ERO1LB__NM_019891_CTTCCTGCGGAGCGTCGTCG	ERO1LB__NM_019891	441	370	520	465
ERO1LB__NM_019891_CTTCTTTGCTTTGATTACTA	ERO1LB__NM_019891	117	60	219	220
ERO1LB__NM_019891_GAGCAATTAACAGCACATTA	ERO1LB__NM_019891	679	1017	853	1036
ERO1LB__NM_019891_GGCAAGATATGATGATTCAC	ERO1LB__NM_019891	348	356	349	460
ERO1LB__NM_019891_TGTGAGCAAGCTAATAAACT	ERO1LB__NM_019891	99	110	151	111
ERO1LB__NM_019891_TTGTGAGCAAGCTAATAAAC	ERO1LB__NM_019891	83	185	78	99
ERO1LB__NM_019891_TTTAAGAGTAAAATTCCGGT	ERO1LB__NM_019891	88	109	89	98
ERP27__NM_152321_AAAGAAAATCGTGAATCAGA	ERP27__NM_152321	319	380	256	294
ERP27__NM_152321_AAATTCCCAGGCGTGTCATT	ERP27__NM_152321	564	845	577	679
ERP27__NM_152321_AATTCCCAGGCGTGTCATTT	ERP27__NM_152321	512	829	510	562
ERP27__NM_152321_ACCAGACTCTAGATGACGAG	ERP27__NM_152321	398	492	643	661
ERP27__NM_152321_AGTGGCAGCAATGAATTCCA	ERP27__NM_152321	580	526	541	476
ERP27__NM_152321_ATTCCTAAGTGGAAAATTGT	ERP27__NM_152321	380	573	439	551
ERP27__NM_152321_CAATGAACAACTGAATTTAG	ERP27__NM_152321	438	450	507	738
ERP27__NM_152321_CACCATCTGCCTCTTTCGCC	ERP27__NM_152321	1580	2169	1925	2151
ERP27__NM_152321_CCAGACTCTAGATGACGAGT	ERP27__NM_152321	935	655	1015	836
ERP27__NM_152321_TCTCCTCCTGATAATGAACA	ERP27__NM_152321	243	296	266	256
ERP27__NM_152321_TGATCCCAAATGACACGCCT	ERP27__NM_152321	638	997	693	883
ERP27__NM_152321_TTGCACCATGCTATGGAGTA	ERP27__NM_152321	364	393	313	415
ERP29__NM_001034025_AAGTGACCGTATCCAGGGGA	ERP29__NM_001034025	568	298	617	342
ERP29__NM_001034025_CGGGGAGAGAAATGCGGCGC	ERP29__NM_001034025	451	618	513	446
ERP29__NM_001034025_GGATACGGTCACTTTCTACA	ERP29__NM_001034025	582	552	599	560
ERP29__NM_001034025_GTAGAAAGTGACCGTATCCA	ERP29__NM_001034025	354	184	365	147
ERP29__NM_001034025_TAGAAAGTGACCGTATCCAG	ERP29__NM_001034025	251	186	319	112
ERP29__NM_001034025_TGTAGAAAGTGACCGTATCC	ERP29__NM_001034025	103	77	96	77
ERP29__NM_006817_ACTGCCCCAGTGTATGGGAC	ERP29__NM_006817	1160	1229	1293	1390
ERP29__NM_006817_AGAACCCAGTCCCATACACT	ERP29__NM_006817	827	899	837	822
ERP29__NM_006817_CATCCTGCTTCTCACCGTAG	ERP29__NM_006817	171	66	178	82
ERP29__NM_006817_CCTTAACTGCCCCAGTGTAT	ERP29__NM_006817	1062	930	1107	952
ERP29__NM_006817_TCATCCTGCTTCTCACCGTA	ERP29__NM_006817	1193	1081	1339	984
ERP29__NM_006817_TCCCGGAAGAGGTAGAAGAC	ERP29__NM_006817	131	98	154	160
ERP44__NM_015051_AAGCATTGGCAGATTACATC	ERP44__NM_015051	251	202	174	227
ERP44__NM_015051_ATAGCATTCTGCTCCGGATA	ERP44__NM_015051	195	170	316	172
ERP44__NM_015051_CAGAAGGAGCATCTGAGGT	ERP44__NM_015051	718	711	854	701
ERP44__NM_015051_CATAATCTACAAACCACCAG	ERP44__NM_015051	130	100	213	236
ERP44__NM_015051_CCCTCAAATTGTTTCGTAAT	ERP44__NM_015051	1623	2393	1739	2574
ERP44__NM_015051_CTTGTTATTTCAGTTGTTAC	ERP44__NM_015051	195	314	227	301
ERP44__NM_015051_GCTCCCAAGTACACCATATC	ERP44__NM_015051	841	774	873	699
ERP44__NM_015051_GGAAGCTTCCGATGTCATTA	ERP44__NM_015051	549	491	645	752
ERP44__NM_015051_TAAGTCCCGAATTTCTTGAA	ERP44__NM_015051	564	370	499	369
ERP44__NM_015051_TACGAAACAATTTGAGGGTT	ERP44__NM_015051	516	644	522	690
ERP44__NM_015051_TCAAAACCGGAAAGATATAG	ERP44__NM_015051	139	225	202	59
ERP44__NM_015051_TGCTCCGGATATGGTGTACT	ERP44__NM_015051	552	489	578	665
ERRFI1__NM_018948_AAATATTGACCCGATAACCA	ERRFI1__NM_018948	168	296	150	206
ERRFI1__NM_018948_AGACTGTAGGCCATGGTTAT	ERRFI1__NM_018948	892	953	938	961
ERRFI1__NM_018948_AGCTATGTGTCTGACCAAAA	ERRFI1__NM_018948	373	437	394	607
ERRFI1__NM_018948_AGGGCTTCAGAGATTGGCAA	ERRFI1__NM_018948	379	216	400	396
ERRFI1__NM_018948_CAGGAGCGCCTAATACCACT	ERRFI1__NM_018948	602	567	848	772
ERRFI1__NM_018948_CCTTATGGCTGGCTTGTTAA	ERRFI1__NM_018948	849	1280	1050	1230
ERRFI1__NM_018948_CTGGCAGGCGAAGCTTCCGT	ERRFI1__NM_018948	1113	865	1284	726
ERRFI1__NM_018948_GACTGTAGGCCATGGTTATC	ERRFI1__NM_018948	501	423	619	606
ERRFI1__NM_018948_GAGACCTTCTTAATCTTCGG	ERRFI1__NM_018948	525	744	694	667
ERRFI1__NM_018948_GCTTCAGAGATTGGCAACGG	ERRFI1__NM_018948	444	586	543	494
ERRFI1__NM_018948_TCCAAATCTGCTCCGATGAA	ERRFI1__NM_018948	451	701	534	651
ERRFI1__NM_018948_TGTGCCCTCTTTGTGAACG	ERRFI1__NM_018948	1039	1141	1123	1636
ERVFRD-1__NM_207582_AAGATGGGTCCGAAAATGGG	ERVFRD-1__NM_207582	112	135	131	146
ERVFRD-1__NM_207582_AGAATGGACAAGCATAGAGG	ERVFRD-1__NM_207582	22	0	74	59
ERVFRD-1__NM_207582_CAGAGAATGGACAAGCATAG	ERVFRD-1__NM_207582	14	1	3	0
ERVFRD-1__NM_207582_CAGATTAGGGTCCCATCGAT	ERVFRD-1__NM_207582	1041	1008	1061	1050
ERVFRD-1__NM_207582_CCAGCAATTGGTGGAGTAAG	ERVFRD-1__NM_207582	202	197	223	218
ERVFRD-1__NM_207582_CCATCCTGATTTCCCGTTAT	ERVFRD-1__NM_207582	3765	4538	3982	4797
ERVFRD-1__NM_207582_CCCCTTACTCCACCAATTGC	ERVFRD-1__NM_207582	1266	1288	1560	1314
ERVFRD-1__NM_207582_CGATGGGACCCTAATCTGAA	ERVFRD-1__NM_207582	782	850	862	925
ERVFRD-1__NM_207582_CTCATCAGTCCTTTCAGATT	ERVFRD-1__NM_207582	995	1002	1274	1444
ERVFRD-1__NM_207582_GGAGGTTTCTGGCGGATATC	ERVFRD-1__NM_207582	320	466	343	312
ERVFRD-1__NM_207582_GTTGAAAGAAGACTATTTGC	ERVFRD-1__NM_207582	417	400	350	429
ERVFRD-1__NM_207582_TGGCGGTAGGCTGCTAGTGA	ERVFRD-1__NM_207582	106	160	138	112
ERVMER34-1__NM_001242690_TAGACTTGTGTCATATTGAT	ERVMER34-1__NM_001242690	215	263	276	275
ERVV-2__NM_001191055_AAGATTCCGGGAAACTATGG	ERVV-2__NM_001191055	96	136	138	101
ERVV-2__NM_001191055_AGATATCAGCTATCTGTCAT	ERVV-2__NM_001191055	655	734	736	1056
ERVV-2__NM_001191055_AGATGTGCTTATAGGTGCTG	ERVV-2__NM_001191055	255	322	269	274
ERVV-2__NM_001191055_ATAGCTGATATCTTTCCATT	ERVV-2__NM_001191055	536	573	666	680
ERVV-2__NM_001191055_ATGCCAGTGGGCAAAGATTC	ERVV-2__NM_001191055	235	315	249	296
ERVV-2__NM_001191055_CAAGGGGGAGATGTGCTTAT	ERVV-2__NM_001191055	419	436	389	474
ERVV-2__NM_001191055_CTGATATCTTTCCATTTGGG	ERVV-2__NM_001191055	621	567	658	676
ERVV-2__NM_001191055_GAGATGTGCTTATAGGTGCT	ERVV-2__NM_001191055	256	281	348	500
ERVV-2__NM_001191055_GCAAAGATTCCGGGAAACTA	ERVV-2__NM_001191055	297	299	330	329
ESAM__NM_138961_AACCTTAGAACTCAATGTAC	ESAM__NM_138961	984	1035	1175	1277
ESAM__NM_138961_ACCGCCTGCAACCGGTTGGC	ESAM__NM_138961	1431	1402	1569	1705
ESAM__NM_138961_ACCTTAGAACTCAATGTACT	ESAM__NM_138961	837	782	1024	932
ESAM__NM_138961_AGCGTGGTACACCTTGCACG	ESAM__NM_138961	1137	997	1174	900
ESAM__NM_138961_CACCGCCTGCAACCGGTTGG	ESAM__NM_138961	1397	1364	1648	1707
ESAM__NM_138961_CAGGTGTTGTCCTACATCAA	ESAM__NM_138961	440	387	548	419
ESAM__NM_138961_CCGTGCAAGGTGTACCACGC	ESAM__NM_138961	717	714	737	992
ESAM__NM_138961_CTCCACCGCCTGCAACCGGT	ESAM__NM_138961	326	298	312	271
ESAM__NM_138961_CTCCCTCCACCGCCTGCAAC	ESAM__NM_138961	1219	1009	1378	1178
ESAM__NM_138961_CTTGCCCGCCAACCGGTTGC	ESAM__NM_138961	22	7	38	42
ESAM__NM_138961_GACAAACAAGGCAAATCTAG	ESAM__NM_138961	72	30	94	53
ESAM__NM_138961_GGGCCCCGGGAGGGAAATCA	ESAM__NM_138961	281	392	296	330
ESCO1__NM_052911_AAGCAAATGAACAGCTTAAC	ESCO1__NM_052911	471	244	495	524
ESCO1__NM_052911_ACTGAGAATACTATGCTGTT	ESCO1__NM_052911	978	1065	1085	1024
ESCO1__NM_052911_ATCAAAAATGGCTACTTCAG	ESCO1__NM_052911	396	276	496	322
ESCO1__NM_052911_CCATTTAGTATACATTTCAC	ESCO1__NM_052911	523	283	748	483
ESCO1__NM_052911_CCTGTGAAATGTATACTAAA	ESCO1__NM_052911	490	432	477	515
ESCO1__NM_052911_CTCTGTGCAGCACAATAAGT	ESCO1__NM_052911	68	100	143	118
ESCO1__NM_052911_TCATCAGAAGGAAACAAATC	ESCO1__NM_052911	139	180	209	228
ESCO1__NM_052911_TCATCTGATTATCCAATGGT	ESCO1__NM_052911	511	250	785	436
ESCO1__NM_052911_TCCCAAAGGGAAAAAACGCA	ESCO1__NM_052911	64	24	107	84
ESCO1__NM_052911_TCGTAATAAGCCTAATTTAG	ESCO1__NM_052911	287	223	310	360
ESCO1__NM_052911_TGAACAAAGTGATAATGAGC	ESCO1__NM_052911	93	101	124	47
ESCO1__NM_052911_TGTTCAGGCACTGATGGCTG	ESCO1__NM_052911	129	84	193	162
ESCO2__NM_001017420_AATTGCAGAACCCATCAAAC	ESCO2__NM_001017420	535	293	652	357
ESCO2__NM_001017420_AGGATTCTTTGAAGTGTGAC	ESCO2__NM_001017420	44	40	93	125
ESCO2__NM_001017420_AGTGATCTATAAGCCAATTG	ESCO2__NM_001017420	250	92	206	161
ESCO2__NM_001017420_CATCTTCAGTCAATTCAAAA	ESCO2__NM_001017420	161	130	262	262
ESCO2__NM_001017420_CGTGTCCTGTCTGAACCAAT	ESCO2__NM_001017420	1275	1007	1241	992
ESCO2__NM_001017420_CTGGAAAATGAGCCGTCACT	ESCO2__NM_001017420	824	467	1032	761
ESCO2__NM_001017420_GACACATCAAGCCTGTATCA	ESCO2__NM_001017420	625	312	716	548
ESCO2__NM_001017420_GCTACTGTGTGCAAGTCTTG	ESCO2__NM_001017420	762	487	931	737
ESCO2__NM_001017420_GGCTTGATGTGTCTATACTT	ESCO2__NM_001017420	466	230	475	381
ESCO2__NM_001017420_TCCAAGCTTTGCTATCAAAA	ESCO2__NM_001017420	147	132	168	228
ESCO2__NM_001017420_TTGGGATGGGAAAATCGTGT	ESCO2__NM_001017420	176	117	224	190
ESCO2__NM_001017420_TTTCTAGAATTCCTTGATAC	ESCO2__NM_001017420	88	59	67	82
ESD__NM_001984_ATAAGAGTACATTCTGTAGT	ESD__NM_001984	579	681	571	728
ESD__NM_001984_ATAGGTGGCTGCAATATTAA	ESD__NM_001984	35	6	47	59
ESD__NM_001984_ATTTCCAGCAACAAGTGCTT	ESD__NM_001984	229	169	174	150
ESD__NM_001984_CACCTTGTGAAATCCTATCC	ESD__NM_001984	686	604	822	653
ESD__NM_001984_CCAAAAATAGACATCCTTTG	ESD__NM_001984	1210	1226	1615	1847
ESD__NM_001984_CTCTTGCAATCGAAAAACAA	ESD__NM_001984	44	54	59	70
ESD__NM_001984_CTTGCAATCGAAAAACAACG	ESD__NM_001984	711	683	714	636
ESD__NM_001984_GAAAATTGGCATTTATGAGT	ESD__NM_001984	671	607	834	956
ESD__NM_001984_GGCCACTCCATGGGAGTGCA	ESD__NM_001984	172	122	212	445
ESD__NM_001984_TGGACATACTAATTGATCAA	ESD__NM_001984	37	9	47	52
ESD__NM_001984_TGTTCCCAAATATCCACTAA	ESD__NM_001984	309	254	259	272
ESD__NM_001984_TTCCAGCAACAAGTGCTTTG	ESD__NM_001984	83	44	40	54
ESF1__NM_001276380_ACCAAGAAGGCTAATCACAA	ESF1__NM_001276380	268	88	308	163
ESF1__NM_001276380_AGATGCTCCTCGTGCTGATG	ESF1__NM_001276380	683	128	686	159
ESF1__NM_001276380_GAAGAGAAATGCAATCAGGT	ESF1__NM_001276380	316	31	262	15
ESF1__NM_001276380_GCGGTTTAGACGGGTTGCAA	ESF1__NM_001276380	140	15	111	2
ESF1__NM_001276380_GGGAGATTTCACAATTTCAG	ESF1__NM_001276380	229	253	292	294
ESF1__NM_001276380_GGTTATGAAAACTCAACAGA	ESF1__NM_001276380	48	30	42	51
ESF1__NM_001276380_TCAAATCCTCTGTAGTGCTA	ESF1__NM_001276380	375	450	425	600
ESF1__NM_001276380_TCAGAAAGCGTTAGTGAAAT	ESF1__NM_001276380	347	529	577	663
ESF1__NM_001276380_TCTGTAGTGCTATGGCTAAT	ESF1__NM_001276380	285	21	316	39
ESF1__NM_001276380_TGAGTGACCAGCGGTTTAGA	ESF1__NM_001276380	278	16	288	11
ESF1__NM_001276380_TGTCTTAGAACACTCGATTC	ESF1__NM_001276380	214	58	334	135
ESF1__NM_001276380_TTGCAACCCGTCTAAACCGC	ESF1__NM_001276380	266	307	296	265
ESM1__NM_001135604_AAAGGATCCTCCCCATTAGA	ESM1__NM_001135604	221	241	265	291
ESM1__NM_001135604_CTGAGGTGTCAGCCTTCTAA	ESM1__NM_001135604	130	67	193	159
ESM1__NM_001135604_CTGCCGGGTCTCCCGTAATG	ESM1__NM_001135604	1283	1199	1157	1004
ESM1__NM_001135604_CTGGAGCAATAATTATGCGG	ESM1__NM_001135604	860	869	785	720
ESM1__NM_001135604_GAGGTGTCAGCCTTCTAATG	ESM1__NM_001135604	149	139	124	207
ESM1__NM_001135604_GGTTAAATCCACGCTGATCC	ESM1__NM_001135604	412	546	333	612
ESM1__NM_001135604_GTGTCAGCCTTCTAATGGGG	ESM1__NM_001135604	191	180	297	371
ESM1__NM_001135604_TCACTGCTGTCACAGTGTTG	ESM1__NM_001135604	486	177	543	384
ESM1__NM_001135604_TTTAACCATTTCCTCATTAC	ESM1__NM_001135604	265	318	299	301
ESM1__NM_001135604_TTTCCTCATTACGGGAGACC	ESM1__NM_001135604	4	0	3	7
ESM1__NM_007036_AAGACTTGGTTACTGAATAT	ESM1__NM_007036	399	468	432	577
ESM1__NM_007036_CGTGAGAGAAACAAATCTGT	ESM1__NM_007036	564	484	632	667
ESM1__NM_007036_TCCATCCCGAAGGTGCCGTA	ESM1__NM_007036	228	266	324	281
ESPL1__NM_012291_ACTGGCATCAAATAGCTGAT	ESPL1__NM_012291	101	2	110	2
ESPL1__NM_012291_AGAGTTGCTGCCCGCCTTGA	ESPL1__NM_012291	1428	1746	1563	1822
ESPL1__NM_012291_AGCTTCAAAAGAGTCAACTT	ESPL1__NM_012291	163	53	155	29
ESPL1__NM_012291_ATGGGCAGCTACCGCTCGAC	ESPL1__NM_012291	302	0	321	15
ESPL1__NM_012291_CAAGAGTACTAGGAAGCTCA	ESPL1__NM_012291	27	1	38	0
ESPL1__NM_012291_CATCTGCTTCATTTAGACCA	ESPL1__NM_012291	577	158	488	95
ESPL1__NM_012291_CTGAAGGCTAGGGGCTAGAT	ESPL1__NM_012291	547	829	604	751
ESPL1__NM_012291_CTGACATAGCTGAAGGCTAG	ESPL1__NM_012291	38	1	45	9
ESPL1__NM_012291_GCTACCGCTCGACAGGCTGT	ESPL1__NM_012291	663	39	613	19
ESPL1__NM_012291_GGATGAAAGTACCCCTTGTG	ESPL1__NM_012291	169	48	203	45
ESPL1__NM_012291_GTAGAGGGGAGGACGCTGTG	ESPL1__NM_012291	765	6	635	11
ESPL1__NM_012291_TTCTCCAACAGCCTGTCGAG	ESPL1__NM_012291	1	0	0	0
ESPN__NM_031475_CAGATGAGAGCGCTTTAATG	ESPN__NM_031475	628	525	636	637
ESPN__NM_031475_CAGCCCAGTCATCGTGTGGT	ESPN__NM_031475	2345	1799	2327	2120
ESPN__NM_031475_CAGCGGGGTCATGCCGTCGT	ESPN__NM_031475	1347	676	1384	532
ESPN__NM_031475_CCAGATGAGAGCGCTTTAAT	ESPN__NM_031475	531	454	601	531
ESPN__NM_031475_CCATTAAAGCGCTCTCATCT	ESPN__NM_031475	1087	1324	1452	1346
ESPN__NM_031475_CCCAGATGAGAGCGCTTTAA	ESPN__NM_031475	576	416	582	621
ESPN__NM_031475_CCGCGACCGCGACGGGTACA	ESPN__NM_031475	302	163	433	382
ESPN__NM_031475_CTCACCAACCACACGATGAC	ESPN__NM_031475	322	76	367	204
ESPN__NM_031475_CTGGAGTGCACACCGCCGCA	ESPN__NM_031475	1237	1418	1138	1243
ESPN__NM_031475_GCAGCGGGGTCATGCCGTCG	ESPN__NM_031475	1734	831	1901	1249
ESPN__NM_031475_GCCGACCTGTCGGACTTCAA	ESPN__NM_031475	88	57	166	93
ESPN__NM_031475_GCGTGGCACCGTTCTTGGTT	ESPN__NM_031475	2733	3398	3157	3972
ESPN__NM_031475_GGCAGCGTAGTGGATAGGCA	ESPN__NM_031475	291	160	364	262
[illegible]	[illegible]	[illegible]	[illegible]	[illegible]	[illegible]

EVX2__NM_001080458_AAGAAAAGAGATGATTCTGA	EVX2__NM_001080458	153	166	151	103
EVX2__NM_001080458_ACCGGGAGAACTATGTGTCG	EVX2__NM_001080458	1068	712	1091	778
EVX2__NM_001080458_AGAATCTCTTGCCCGCCGTA	EVX2__NM_001080458	772	901	961	916
EVX2__NM_001080458_AGCGGCACGTGCGAGTGGAA	EVX2__NM_001080458	1083	1309	1192	1613
EVX2__NM_001080458_ATCATGTAGGTGTAGAAGCT	EVX2__NM_001080458	283	406	285	339
EVX2__NM_001080458_CCTCGGCGCCGCTCAGCTTA	EVX2__NM_001080458	336	374	330	450
EVX2__NM_001080458_CCTTGATGGTGGTTTCGGGC	EVX2__NM_001080458	501	213	538	549
EVX2__NM_001080458_GAAGCCGGGCCATTATTCAG	EVX2__NM_001080458	498	425	606	590
EVX2__NM_001080458_GCCGGGCCATTATTCAGAGG	EVX2__NM_001080458	1065	1335	1284	1079
EVX2__NM_001080458_GCTCGCGGGTGAACGCCGTA	EVX2__NM_001080458	628	670	665	596
EVX2__NM_001080458_GGCCGCGTGCGTCATCATGT	EVX2__NM_001080458	2204	1605	2394	2171
EVX2__NM_001080458_TCAAGTGCGGCGCTACCGTA	EVX2__NM_001080458	438	525	510	562
EWSR1__NM_001163285_CAACAAAGCTATGGAACCTA	EWSR1__NM_001163285	572	550	586	417
EWSR1__NM_001163285_CAGCCTGCTTATCCAGCCTA	EWSR1__NM_001163285	1026	594	1079	584
EWSR1__NM_001163285_CCAGTGCCATACCCCTGGAC	EWSR1__NM_001163285	1079	259	1121	134
EWSR1__NM_001163285_CTGTCCATAAGAAGTTGCAT	EWSR1__NM_001163285	456	148	465	196
EWSR1__NM_001163285_GCATATGGGCAACAAAGCTA	EWSR1__NM_001163285	135	49	157	48
EWSR1__NM_001163285_GGTCTGCCCATAGGTTGCAG	EWSR1__NM_001163285	388	118	406	236
EWSR1__NM_001163285_GGTCTGTGCATATCCTTGAG	EWSR1__NM_001163285	417	181	484	267
EWSR1__NM_001163285_GTCTGTGCATATCCTTGAGT	EWSR1__NM_001163285	715	122	721	139
EWSR1__NM_001163285_TGAGGTTGACTAGTCTCAGT	EWSR1__NM_001163285	514	94	512	161
EWSR1__NM_001163285_TGCAGACTGAGCTGCATAGG	EWSR1__NM_001163285	73	54	113	39
EWSR1__NM_001163285_TGCCCATAGGCTGGATAAGC	EWSR1__NM_001163285	173	115	234	91
EWSR1__NM_001163285_TTGAGGTTGACTAGTCTCAG	EWSR1__NM_001163285	204	72	203	48
EWSR1__NM_001163286_ACTCTCAGCAGAACACCTAT	EWSR1__NM_001163286	225	70	232	33
EWSR1__NM_001163286_CTGCTCTGATCATAACTAGT	EWSR1__NM_001163286	94	14	156	18
EXD1__NM_001286441_AAGCAGGCTCACATACATTT	EXD1__NM_001286441	276	183	398	271
EXD1__NM_001286441_AGGAAATGGTAGTCACTGCT	EXD1__NM_001286441	350	386	430	361
EXD1__NM_001286441_AGGTTATCCATGATTGTCGT	EXD1__NM_001286441	175	237	248	184
EXD1__NM_001286441_AGTGGAACTACTAGATGAAG	EXD1__NM_001286441	118	168	147	162
EXD1__NM_001286441_CAGAAAGCCAACGACAATCA	EXD1__NM_001286441	224	367	213	427
EXD1__NM_001286441_CGACAATCATGGATAACCTA	EXD1__NM_001286441	502	701	493	416
EXD1__NM_001286441_GAAGAGGGTGAAACTCACAT	EXD1__NM_001286441	406	341	450	432
EXD1__NM_001286441_GGAGCGAATGTATGTCGCCA	EXD1__NM_001286441	739	733	904	878
EXD1__NM_001286441_TAAGTAAACTCGGCAATTTG	EXD1__NM_001286441	488	342	694	665
EXD1__NM_001286441_TCAGGACAACAATCTTATTA	EXD1__NM_001286441	160	92	159	168
EXD1__NM_001286441_TTCAGGACAACAATCTTATT	EXD1__NM_001286441	27	129	37	12
EXD1__NM_001286441_TTGGTCTGTGGTGTCTTCGA	EXD1__NM_001286441	185	220	194	282
EXD1__NM_152596_CAAGCGAAGGGGTAACAGGT	EXD1__NM_152596	420	338	536	449
EXD1__NM_152596_GAATAATGTCTTTGACACAC	EXD1__NM_152596	274	278	330	347
EXD1__NM_152596_GACAGTGAATTCCTAGCTTA	EXD1__NM_152596	261	270	216	152
EXD1__NM_152596_GAGACAAAAACTAATTCAGG	EXD1__NM_152596	426	452	539	620
EXD1__NM_152596_TCTAGGAGTGCCAAGCGAAG	EXD1__NM_152596	129	90	101	89
EXD2__NM_001193360_AACACTACCAAGAACGTTAT	EXD2__NM_001193360	531	786	701	818
EXD2__NM_001193360_AGGATTATGGCCTCGTTGTT	EXD2__NM_001193360	841	681	918	1075
EXD2__NM_001193360_ATTATGGCCTCGTTGTTAGG	EXD2__NM_001193360	290	267	327	280
EXD2__NM_001193360_CCAGGCACCCCCTAACAACG	EXD2__NM_001193360	792	700	806	682
EXD2__NM_001193360_CGCCTGCCCAAGCTAATCTG	EXD2__NM_001193360	1018	982	1285	1281
EXD2__NM_001193360_CTCCACAGATTAGCTTGGGC	EXD2__NM_001193360	319	441	313	262
EXD2__NM_001193360_CTGCCCAAGCTAATCTGTGG	EXD2__NM_001193360	562	580	584	573
EXD2__NM_001193360_GATGTCTAGACAGAACTTAG	EXD2__NM_001193360	168	299	281	315
EXD2__NM_001193360_GATTATGGCCTCGTTGTTAG	EXD2__NM_001193360	446	458	605	552
EXD2__NM_001193360_GGATTATGGCCTCGTTGTTA	EXD2__NM_001193360	812	846	878	1078
EXD2__NM_001193360_TCACAGTCAATTCCAAGTAC	EXD2__NM_001193360	180	261	341	261
EXD2__NM_001193360_TTTCCTCCACAGATTAGCTT	EXD2__NM_001193360	27	2	25	22
EXD2__NM_018199_ACCTCCGATACCTAGCCATG	EXD2__NM_018199	849	912	863	644
EXD2__NM_018199_TCTGCCGCATGGCTAGGTAT	EXD2__NM_018199	354	364	355	267
EXD3__NM_001286823_ACGTCCTTGATGTCAAAGCC	EXD3__NM_001286823	296	284	260	282
EXD3__NM_001286823_AGTGAGGACTTTGACCGCTC	EXD3__NM_001286823	248	179	307	234
EXD3__NM_001286823_CAGTGAGGACTTTGACCGCT	EXD3__NM_001286823	1291	1537	1386	1495
EXD3__NM_001286823_GCTGGCGAGCGCCACCGCAT	EXD3__NM_001286823	522	218	583	492
EXD3__NM_017820_CAAGAGTTCCATGGCACACT	EXD3__NM_017820	1292	1522	1542	1712
EXD3__NM_017820_CACACTGGGCGGCCGTGACT	EXD3__NM_017820	2421	1981	2545	2551
EXD3__NM_017820_CGCTGCTGAATGGCCGCGTT	EXD3__NM_017820	2075	2657	2385	3177
EXD3__NM_017820_CTCGGGCAGCAAGAGTTCCA	EXD3__NM_017820	471	435	518	583
EXD3__NM_017820_GAACCTCCACAAACCGCTTG	EXD3__NM_017820	615	433	750	619
EXD3__NM_017820_GAACTGGACCGACCATGTGC	EXD3__NM_017820	994	717	975	707
EXD3__NM_017820_GGATGGGCAGCTGGTAGTAA	EXD3__NM_017820	285	380	401	433
EXD3__NM_017820_TGCGGCACCTGTGCCACAAG	EXD3__NM_017820	21	8	38	27
EXO1__NM_003686_AACTGCGCATCAGCTTCATA	EXO1__NM_003686	388	101	281	204
EXO1__NM_003686_ATCCATGTGAGGAAGTATAA	EXO1__NM_003686	201	130	183	131
EXO1__NM_003686_ATGTGTGATATTGATAGACC	EXO1__NM_003686	121	57	127	147
EXO1__NM_003686_CATCCATCAAATACGAGAAT	EXO1__NM_003686	616	532	767	787
EXO1__NM_003686_CCAAAGGTGAACCTACTGAT	EXO1__NM_003686	1023	839	1135	1213
EXO1__NM_003686_GAAAGCAACTTCTTCGTGAG	EXO1__NM_003686	534	749	592	710
EXO1__NM_003686_GCCCTTTATACTTCCTCACA	EXO1__NM_003686	1343	716	1737	1664
EXO1__NM_003686_TACAAAATCCTACATACCTA	EXO1__NM_003686	357	423	359	392
EXO1__NM_003686_TATCAATATCACACATGCCA	EXO1__NM_003686	97	99	243	211
EXO1__NM_003686_TCAGGGGGTAGATTGCCTCG	EXO1__NM_003686	728	486	910	813
EXO1__NM_003686_TGTGCTGAAAAACTAGCCAA	EXO1__NM_003686	55	20	66	41
EXO1__NM_003686_TTGCTTTCCCTTAAGAAGAT	EXO1__NM_003686	104	76	78	148
EXO5__NM_022774_AAGGCTTTAGTTAACATGCC	EXO5__NM_022774	123	175	186	163
EXO5__NM_022774_ACAAGAAGACAAGTTCCATG	EXO5__NM_022774	658	634	602	474
EXO5__NM_022774_AGAGAAGGAGGTGAGAGCCA	EXO5__NM_022774	290	61	245	81
EXO5__NM_022774_AGCCAAGGTGCAGCATTATA	EXO5__NM_022774	12	0	27	4
EXO5__NM_022774_AGCTGCACTATACAGCCAAG	EXO5__NM_022774	267	65	287	278
EXO5__NM_022774_ATCTTCTGAATCCCTTGGGA	EXO5__NM_022774	219	98	188	122
EXO5__NM_022774_CTGACCTGGCTACTCAGAAC	EXO5__NM_022774	1882	1860	2145	2311
EXO5__NM_022774_GAAGTTCTAGTTCTCTAGCT	EXO5__NM_022774	99	105	110	169
EXO5__NM_022774_GAGCTGCACTATACAGCCAA	EXO5__NM_022774	565	374	755	429
EXO5__NM_022774_GAGCTTCCAGAGGGAGCATA	EXO5__NM_022774	24	0	28	45
EXO5__NM_022774_GTGAACTGCAAACAGCATAT	EXO5__NM_022774	72	79	139	163
EXO5__NM_022774_TGCCCAAGCATCTTCTTTAG	EXO5__NM_022774	846	830	918	1049
EXOC1__NM_001024924_AAAGTCAAGAAATCCGATAA	EXOC1__NM_001024924	158	70	299	85
EXOC1__NM_001024924_ACAGGATATCGAAATAATGA	EXOC1__NM_001024924	264	96	258	115
EXOC1__NM_001024924_ACGCCCTGTGCAGGTTAAGG	EXOC1__NM_001024924	550	607	638	576
EXOC1__NM_001024924_ACTCCATCAGCTTGGCATAT	EXOC1__NM_001024924	394	141	448	275
EXOC1__NM_001024924_CAAAGTCAAGAAATCCGATA	EXOC1__NM_001024924	284	497	414	605
EXOC1__NM_001024924_CTCAGCAGGCGTTCATCATT	EXOC1__NM_001024924	471	543	663	583
EXOC1__NM_001024924_GTTGAACAAAAACATTGTTG	EXOC1__NM_001024924	520	339	560	515
EXOC1__NM_001024924_TAGGTCTTCATGGAAGTTCG	EXOC1__NM_001024924	76	38	86	53
EXOC1__NM_001024924_TCTACAAAAGGCAGATTGCA	EXOC1__NM_001024924	319	242	366	269
EXOC1__NM_001024924_TGAATATGCAATCTCGAATG	EXOC1__NM_001024924	481	293	506	260
EXOC1__NM_001024924_TGGCTAAAGAGTACAGATTA	EXOC1__NM_001024924	43	49	43	15
EXOC1__NM_001024924_TTTGTAGAAATCTCCCTTAT	EXOC1__NM_001024924	632	416	682	520
EXOC1__NM_018261_AAGTTAGTATGCAACCTATA	EXOC1__NM_018261	348	442	246	286
EXOC1__NM_018261_ACTCCAAATGCATTGCTCAT	EXOC1__NM_018261	44	16	33	11
EXOC1__NM_018261_CACAAGCAGCTAGGCAATAA	EXOC1__NM_018261	420	333	433	545
EXOC1__NM_018261_CACACTAACAAATGTGTATA	EXOC1__NM_018261	886	1068	959	1207
EXOC1__NM_018261_GGCAAATAAAACTGCTATAT	EXOC1__NM_018261	532	585	647	730
EXOC1__NM_018261_TAAGTTAGTATGCAACCTAT	EXOC1__NM_018261	286	233	327	330
EXOC2__NM_018303_AAGTAAAATAGTATGTCGAG	EXOC2__NM_018303	179	315	300	450
EXOC2__NM_018303_AGAGATGCCGGTCACAAGGG	EXOC2__NM_018303	325	172	305	158
EXOC2__NM_018303_CTCCAAATGAAGGGATACCA	EXOC2__NM_018303	87	38	126	105
EXOC2__NM_018303_GACTTAGAAATGCTATTCCA	EXOC2__NM_018303	818	625	815	590
EXOC2__NM_018303_GAGAGATGCCGGTCACAAGG	EXOC2__NM_018303	195	94	239	116
EXOC2__NM_018303_GTCCATGGTATCCCTTCATT	EXOC2__NM_018303	933	428	1086	607
EXOC2__NM_018303_GTTAGCAGGACGTAAGGACA	EXOC2__NM_018303	519	299	583	328
EXOC2__NM_018303_TAGCTTGAAAGAGACTGTTG	EXOC2__NM_018303	183	371	221	150
EXOC2__NM_018303_TCAATGCCAAGCGGGTTAGC	EXOC2__NM_018303	107	49	110	176
EXOC2__NM_018303_TGAAGGGATACCATGGACGA	EXOC2__NM_018303	301	218	358	237
EXOC2__NM_018303_TGGACGAAGGTCACAATCAG	EXOC2__NM_018303	28	49	14	3
EXOC2__NM_018303_TTACGTCCTGCTAACCCGCT	EXOC2__NM_018303	640	194	764	374
EXOC3__NM_007277_ACAGCAGTGCAAAGGGTTGC	EXOC3__NM_007277	678	699	811	969
EXOC3__NM_007277_ACCCAGGACCTAATTGAACA	EXOC3__NM_007277	1291	1389	1505	1643
EXOC3__NM_007277_CAGCAGTGCAAAGGGTTGCT	EXOC3__NM_007277	2111	2013	1936	2073
EXOC3__NM_007277_CAGTTGGACGGGGTGCGCAC	EXOC3__NM_007277	721	354	709	349
EXOC3__NM_007277_CATCCAGTCACAGTTGGACG	EXOC3__NM_007277	1515	924	1673	756
EXOC3__NM_007277_CATTCAGGGCGTTGTGGAGC	EXOC3__NM_007277	2994	2934	3332	3514
EXOC3__NM_007277_CCATCCAGTCACAGTTGGAC	EXOC3__NM_007277	1669	995	1648	948
EXOC3__NM_007277_CCCAGGACCTAATTGAACAA	EXOC3__NM_007277	544	159	452	116
EXOC3__NM_007277_CCTCACAATCTCAGGCACTA	EXOC3__NM_007277	1199	163	1373	244
EXOC3__NM_007277_CTCCGTGGAGGCCAGATTGA	EXOC3__NM_007277	317	178	457	255
EXOC3__NM_007277_GGCCGCCATCCAGTCACAGT	EXOC3__NM_007277	2362	2458	2496	1977
EXOC3__NM_007277_GGCTACTTTGGCAGCACGCA	EXOC3__NM_007277	242	287	264	400
EXOC3L1__NM_178516_CAGGCCCGTGGGTTGCTCCA	EXOC3L1__NM_178516	1146	1398	1378	1209
EXOC3L1__NM_178516_CATATGCCTCCAAGAACTGT	EXOC3L1__NM_178516	132	33	195	134
EXOC3L1__NM_178516_CCCGGGGTGCAGCGCTCAAG	EXOC3L1__NM_178516	813	715	905	1010
EXOC3L1__NM_178516_CCGGGGTGCAGCGCTCAAGT	EXOC3L1__NM_178516	1038	1003	1233	988
EXOC3L1__NM_178516_CTCCCTGGAATCGCGCCTCA	EXOC3L1__NM_178516	907	810	1145	1179
EXOC3L1__NM_178516_GCAGCTGCGAGAGGATACGT	EXOC3L1__NM_178516	733	853	780	749
EXOC3L1__NM_178516_GCTCTAGAGTCTGTAAGGCC	EXOC3L1__NM_178516	122	241	136	216
EXOC3L1__NM_178516_GGGGCTGGACCTTCTGTTCG	EXOC3L1__NM_178516	1307	1884	1687	2175
EXOC3L1__NM_178516_GTCAGTGATGCAGTCATACC	EXOC3L1__NM_178516	262	274	272	293
EXOC3L1__NM_178516_GTGATGCAGTCATACCTGGA	EXOC3L1__NM_178516	633	562	715	661
EXOC3L1__NM_178516_TACAGACTCTAGAGCCCCTA	EXOC3L1__NM_178516	488	512	553	623
EXOC3L1__NM_178516_TAGGGGCTCTAGAGTCTGTA	EXOC3L1__NM_178516	181	187	186	228
EXOC3L2__NM_138568_AATGCAGGACGAGCCTTACC	EXOC3L2__NM_138568	934	844	993	777
EXOC3L2__NM_138568_ACAGCAGGTTGGCCACGACA	EXOC3L2__NM_138568	782	716	896	797
EXOC3L2__NM_138568_ATGGCCCACTGCTGCCTAGG	EXOC3L2__NM_138568	1050	911	1110	1097
EXOC3L2__NM_138568_CAGCGCCCGCCGGTGTAGCT	EXOC3L2__NM_138568	459	608	550	665
EXOC3L2__NM_138568_CGCTGGTAGCCGAGCTACAC	EXOC3L2__NM_138568	510	454	489	443
EXOC3L2__NM_138568_CTCGTCCTGCATTCTGCGCA	EXOC3L2__NM_138568	286	621	416	712
EXOC3L2__NM_138568_GCAGGTTGGCCACGACACGG	EXOC3L2__NM_138568	691	866	793	686
EXOC3L2__NM_138568_GGATGCGTGGTGCCCCCATT	EXOC3L2__NM_138568	1674	1622	1944	2009
EXOC3L2__NM_138568_GGTAGCCGAGCTACACCGGC	EXOC3L2__NM_138568	112	127	151	98
EXOC3L2__NM_138568_GGTGGCAGAGCCGGGTCACA	EXOC3L2__NM_138568	256	123	301	96
EXOC3L2__NM_138568_GTCTTGCTGATATAGGTGTC	EXOC3L2__NM_138568	454	831	533	666
EXOC3L2__NM_138568_TTCATCCTCCAAACCCCGCA	EXOC3L2__NM_138568	107	116	170	176
EXOC3L4__NM_001077594_ACACCATTCATGACCCCCGA	EXOC3L4__NM_001077594	1064	955	1176	1004
EXOC3L4__NM_001077594_AGACCTGGGTCAAAGCCCGC	EXOC3L4__NM_001077594	219	158	336	305
EXOC3L4__NM_001077594_CATTACTGAACGGCAACTGC	EXOC3L4__NM_001077594	474	390	611	677
EXOC3L4__NM_001077594_CCAGGTCTCCAAGGAAGATA	EXOC3L4__NM_001077594	191	174	238	334
EXOC3L4__NM_001077594_CCGCAAGGATGGCACAAGGC	EXOC3L4__NM_001077594	193	148	178	210
EXOC3L4__NM_001077594_CCTTGTGCCATCCTTGCGGT	EXOC3L4__NM_001077594	1318	1931	1528	2039
EXOC3L4__NM_001077594_CGGTAGCTGGGCCGTCATTC	EXOC3L4__NM_001077594	958	957	1035	1139
EXOC3L4__NM_001077594_CTGCCAGCCCGTCGTAAAGC	EXOC3L4__NM_001077594	453	471	465	642
EXOC3L4__NM_001077594_GAGGAACTGAAACCCGAGGC	EXOC3L4__NM_001077594	500	486	524	524
EXOC3L4__NM_001077594_GTGCCCTCGGGGGTCATGAA	EXOC3L4__NM_001077594	206	303	231	272
EXOC3L4__NM_001077594_GTGCCTGCTTTACGACGGGC	EXOC3L4__NM_001077594	574	863	704	995
EXOC3L4__NM_001077594_TGGCCGACCTCATTACTGAA	EXOC3L4__NM_001077594	99	104	137	163
EXOC4__NM_001037126_AATGAGGATACCCATAAACA	EXOC4__NM_001037126	421	108	428	135
EXOC4__NM_001037126_CATCTATGAGAACCAAGTGA	EXOC4__NM_001037126	474	175	580	213
EXOC4__NM_001037126_GATGCTCTGGTATGTGCGAA	EXOC4__NM_001037126	561	932	689	902
EXOC4__NM_001037126_GGAAAACAGCACCCTGTTTA	EXOC4__NM_001037126	774	580	1037	925
EXOC4__NM_001037126_GGCACTGAGATAGTGCTTGC	EXOC4__NM_001037126	608	486	760	638
EXOC4__NM_001037126_GGCTAGTCGATTTGATGTAC	EXOC4__NM_001037126	806	878	956	998
EXOC4__NM_001037126_GTCACGGTCACATTTCTCGT	EXOC4__NM_001037126	1818	1226	1863	981
EXOC4__NM_001037126_GTTTATGGGTATCCTCATTA	EXOC4__NM_001037126	227	175	259	202
EXOC4__NM_001037126_TAAGCATGTCCTGAACTTGT	EXOC4__NM_001037126	743	486	632	372
EXOC4__NM_001037126_TAATGAGGATACCCATAAAC	EXOC4__NM_001037126	254	40	324	161
EXOC4__NM_001037126_TAGTGACGATGTCGAAGACA	EXOC4__NM_001037126	245	199	226	280
EXOC4__NM_001037126_TGCTGTGAAGCTCTAGTCGA	EXOC4__NM_001037126	135	173	147	109
EXOC5__NM_006544_AAGAGCAGTTAGAAGAATGT	EXOC5__NM_006544	351	119	376	117
EXOC5__NM_006544_AAGGTAACTCTTGGGCAATT	EXOC5__NM_006544	422	25	489	46
EXOC5__NM_006544_AATGATATATTTGAAGACGC	EXOC5__NM_006544	120	47	120	74
EXOC5__NM_006544_AGCCTCCACTGCCCGTTGTC	EXOC5__NM_006544	2250	1751	2338	2306
EXOC5__NM_006544_CAAAGAGTGAACAAACAAGT	EXOC5__NM_006544	267	151	310	241
EXOC5__NM_006544_GAATCCTTTCATCCATTATC	EXOC5__NM_006544	207	40	337	93
EXOC5__NM_006544_GCCTCCACTGCCCGTTGTCT	EXOC5__NM_006544	3231	2037	3700	2788
EXOC5__NM_006544_GTGTGTTTACCCCCTCTAAC	EXOC5__NM_006544	286	386	360	524
EXOC5__NM_006544_TAGTTCTTGGAAATGTTGGA	EXOC5__NM_006544	7	4	12	18
EXOC5__NM_006544_TATTCAGGAACTCCAGATAA	EXOC5__NM_006544	149	4	134	22
EXOC5__NM_006544_TCAGCTGGCATTCTAAATCA	EXOC5__NM_006544	396	177	389	280
EXOC5__NM_006544_TTTGAGATATTGCTCTGCAT	EXOC5__NM_006544	33	16	37	28
EXOC6__NM_001013848_AAAGTCTTTGAGATCAGACA	EXOC6__NM_001013848	193	151	301	364
EXOC6__NM_001013848_AACCGGTATTGACTAACCCA	EXOC6__NM_001013848	276	217	296	269
EXOC6__NM_001013848_AAGTTACTGATACCAACCGA	EXOC6__NM_001013848	419	331	364	409
EXOC6__NM_001013848_ACTTCTTGTGCGCATTTGGT	EXOC6__NM_001013848	1012	812	955	1143
EXOC6__NM_001013848_ATTGCAACCCCAGTCGAATA	EXOC6__NM_001013848	525	314	583	409
EXOC6__NM_001013848_CTGCATCAGTCCTTACTTTA	EXOC6__NM_001013848	927	1131	1034	1366
EXOC6__NM_001013848_GTGCAAACATCGATAAACAG	EXOC6__NM_001013848	814	944	1025	1125
EXOC6__NM_001013848_GTTGAAGCTACTTTAAGGTA	EXOC6__NM_001013848	277	239	299	252
EXOC6__NM_001013848_TACCTTAAAGTAGCTTCAAC	EXOC6__NM_001013848	100	152	110	94
EXOC6__NM_001013848_TGTGCAAACATCGATAAACA	EXOC6__NM_001013848	261	260	383	493
EXOC6__NM_001013848_TTCCCTGGGTTAGTCAATAC	EXOC6__NM_001013848	14	30	42	31
EXOC6__NM_001013848_TTTAATATCCTCACGGAGTT	EXOC6__NM_001013848	16	18	17	65
EXOC6__NM_019053_CACTGATGAACTTTGGAACA	EXOC6__NM_019053	695	888	600	740
EXOC6__NM_019053_TGACCCAAGGATTAGTAACC	EXOC6__NM_019053	107	85	60	64
EXOC6B__NM_015189_AAGCCACTATCGATTCTGCA	EXOC6B__NM_015189	718	895	851	920
EXOC6B__NM_015189_AATCGATAGTGGCTTACTTG	EXOC6B__NM_015189	1477	1191	1551	1319
EXOC6B__NM_015189_AGACAGCGGCAGAGCACGAG	EXOC6B__NM_015189	224	260	217	207
EXOC6B__NM_015189_AGCACTGACACGGCCTGCAT	EXOC6B__NM_015189	824	576	795	588
EXOC6B__NM_015189_ATGTGCAACTTTCATTACCA	EXOC6B__NM_015189	722	731	847	639
EXOC6B__NM_015189_CAATTTCTCGGTCGTGATTA	EXOC6B__NM_015189	659	982	711	855
EXOC6B__NM_015189_CAGCATTAATTTATCAACAG	EXOC6B__NM_015189	247	301	416	398
EXOC6B__NM_015189_CAGTGCTCTCGATCTCTCGC	EXOC6B__NM_015189	1102	1419	1260	1592
EXOC6B__NM_015189_CGCAAACATTCAGACAAAAT	EXOC6B__NM_015189	486	755	511	738
EXOC6B__NM_015189_GATAGTGGCTTACTTGAGGC	EXOC6B__NM_015189	493	367	492	575
EXOC6B__NM_015189_TCACCTTGCAGAATCGATAG	EXOC6B__NM_015189	938	835	908	918
EXOC6B__NM_015189_TTCCATTGCTATTACCAGCT	EXOC6B__NM_015189	5	7	2	0
EXOC7__NM_001013839_AGGAGGCATCCGCTCGACGG	EXOC7__NM_001013839	500	411	566	315
EXOC7__NM_001013839_ATTCCCAGCATGGTCTAGAT	EXOC7__NM_001013839	136	214	144	118
EXOC7__NM_001013839_CAGCCCGGAACTCAACAAAG	EXOC7__NM_001013839	249	150	325	152
EXOC7__NM_001013839_CCAGCTGGCTGGAGCGTATC	EXOC7__NM_001013839	249	187	373	282
EXOC7__NM_001013839_CCTGATGACGCGGCACAGTA	EXOC7__NM_001013839	810	501	976	564
EXOC7__NM_001013839_CGTCATCAGGCTGCGAAATT	EXOC7__NM_001013839	779	987	797	724
EXOC7__NM_001013839_CTCATCTTGGATCTGATCAG	EXOC7__NM_001013839	317	130	245	79
EXOC7__NM_001013839_CTTACCACTTTGTTGAGTTC	EXOC7__NM_001013839	433	332	496	777
EXOC7__NM_001013839_TACTCTTCCAGCCTACCTGT	EXOC7__NM_001013839	504	158	431	189
EXOC7__NM_001013839_TATTCCCAGCATGGTCTAGA	EXOC7__NM_001013839	27	10	36	13
EXOC7__NM_001013839_TCAGAAGGTTCTGAGCCTTA	EXOC7__NM_001013839	225	98	228	196
EXOC7__NM_001013839_TCCCAGCCCCACAGGTAGGC	EXOC7__NM_001013839	184	32	192	148
EXOC7__NM_001145298_CGGAAATCATGCTCGTGACC	EXOC7__NM_001145298	446	527	559	509
EXOC7__NM_001145298_CGGCCCGTGCTTGTCGTTCA	EXOC7__NM_001145298	2095	2753	2321	3195
EXOC7__NM_001145298_TGTCGTTCAAGGCCTCGGAC	EXOC7__NM_001145298	106	64	91	80
EXOC7__NM_001282313_TTTCCCATCTAGACCATGCT	EXOC7__NM_001282313	383	396	453	405
EXOC7__NM_001282314_AAGCTTCTCACGTTATTCTG	EXOC7__NM_001282314	418	257	443	277
EXOC7__NM_001282314_CAGAATAACGTGAGAAGCTT	EXOC7__NM_001282314	293	328	276	444
EXOC7__NM_001282314_GAACTCAACAAAGTGGTAAG	EXOC7__NM_001282314	2	0	8	3
EXOC7__NM_001282314_GTCTCTGCCCTGGTCTCATG	EXOC7__NM_001282314	752	825	774	811
EXOC7__NM_001282314_TCTCTGCCCTGGTCTCATGA	EXOC7__NM_001282314	793	368	765	660
EXOC7__NM_015219_CGTCAGTGCCTTCGTCAAGC	EXOC7__NM_015219	613	745	554	690
EXOC7__NM_015219_GGAGAAGTCGTGTCGCACAA	EXOC7__NM_015219	1015	1229	1173	1303
EXOC7__NM_015219_TGGATGGGCTGATGCTTGAA	EXOC7__NM_015219	34	9	29	25
EXOC8__NM_175876_CCTGGAAAATACGGCTCTCG	EXOC8__NM_175876	1333	814	1479	632
EXOC8__NM_175876_CCTGGCTGCCTCAGCGGCGT	EXOC8__NM_175876	1002	670	1090	609
EXOC8__NM_175876_CTACCGGCAGTTCATAGAGA	EXOC8__NM_175876	343	57	395	83
EXOC8__NM_175876_GACTACGGCCAAACCATCTA	EXOC8__NM_175876	341	265	447	219
EXOC8__NM_175876_GAGGGGAGGAGGGAGTCGGT	EXOC8__NM_175876	642	117	736	144
EXOC8__NM_175876_GCAACGTCTACCAGAACTAC	EXOC8__NM_175876	395	220	378	193
EXOC8__NM_175876_GCGATGGCGATGTCGGACAG	EXOC8__NM_175876	1277	1159	1531	1512
EXOC8__NM_175876_GGAATACGATGCGGACCACA	EXOC8__NM_175876	460	335	506	192
EXOC8__NM_175876_GGACAGTACTTGGTGTACAA	EXOC8__NM_175876	233	234	238	150
EXOC8__NM_175876_GTTGTCCTTGACATTGACTA	EXOC8__NM_175876	177	127	189	159
EXOC8__NM_175876_TCTCATGAACGATTGCTTGT	EXOC8__NM_175876	760	679	786	969
EXOC8__NM_175876_TGACTACGGCCAAACCATCT	EXOC8__NM_175876	594	326	685	335
EXOG__NM_001145464_AATGAAGATTATGTTGGAAG	EXOG__NM_001145464	373	427	459	517
EXOG__NM_001145464_ACTGAAGGTTGGAGGGATAT	EXOG__NM_001145464	664	400	611	471
EXOG__NM_001145464_AGTCCCCTCACACCTTTATA	EXOG__NM_001145464	391	208	382	424
EXOG__NM_001145464_ATTACCTTATAAAGGTGTGA	EXOG__NM_001145464	104	124	84	156
EXOG__NM_001145464_CCTGAGGCACAATGTTAGAA	EXOG__NM_001145464	315	224	311	321
EXOG__NM_001145464_CGAAGCCGCTCAGAAAACGA	EXOG__NM_001145464	644	622	584	656
EXOG__NM_001145464_GATTACCTTATAAAGGTGTG	EXOG__NM_001145464	101	255	157	192
EXOG__NM_001145464_GCGCTGAGGGAGCGTTGACA	EXOG__NM_001145464	576	520	610	458
EXOG__NM_001145464_GGCGCTGAGGGAGCGTTGAC	EXOG__NM_001145464	388	180	419	355
EXOG__NM_001145464_GTTGGAGGGATATTGGGATC	EXOG__NM_001145464	442	379	449	316
EXOG__NM_001145464_TTACCTCAGACTAGAGGCGA	EXOG__NM_001145464	60	17	34	43
EXOG__NM_001145464_TTGACCTTACCTCAGACTAG	EXOG__NM_001145464	87	89	110	99
EXOG__NM_005107_AAGGCTGTCTTGGAACAATT	EXOG__NM_005107	173	159	256	318
EXOG__NM_005107_AGGCAAAGCGGGTGCCTAGA	EXOG__NM_005107	266	171	403	259
EXOG__NM_005107_CTTGCCTCTGTTCCAGTTAA	EXOG__NM_005107	452	313	498	478
EXOG__NM_005107_GGCAAAGCGGGTGCCTAGAT	EXOG__NM_005107	1011	544	1196	1097
EXOG__NM_005107_GTCTTATGATCAGGCAAAGC	EXOG__NM_005107	81	73	62	12
EXOG__NM_005107_TGTCTTATGATCAGGCAAAG	EXOG__NM_005107	245	202	247	180
EXOSC1__NM_016046_AGACCCACACTAAAGAATTC	EXOSC1__NM_016046	157	57	144	102
EXOSC1__NM_016046_ATTCTTTAGTGTGGGTCTTA	EXOSC1__NM_016046	38	60	107	79
EXOSC1__NM_016046_CAAGAATTCGGGTTGTACTC	EXOSC1__NM_016046	528	595	544	638
EXOSC1__NM_016046_CACATACAGGATGTGTACTT	EXOSC1__NM_016046	585	197	648	115
EXOSC1__NM_016046_CAGCGAGAATGGCGCGGTAA	EXOSC1__NM_016046	698	251	849	293
EXOSC1__NM_016046_CGTCTGTGTAACTTGGAGGA	EXOSC1__NM_016046	764	812	741	489
EXOSC1__NM_016046_CGTTCTCGGCGGTGGTTAGC	EXOSC1__NM_016046	80	12	108	35
EXOSC1__NM_016046_CTGATGAAGAGCAGCGAGAA	EXOSC1__NM_016046	63	134	49	39
EXOSC1__NM_016046_GAGGCATGGACCCCACATAC	EXOSC1__NM_016046	488	302	586	310
EXOSC1__NM_016046_GGCGGTGGTTAGCAGGTAGT	EXOSC1__NM_016046	86	68	100	60
EXOSC1__NM_016046_GGGATGCAGTATCTCACAGG	EXOSC1__NM_016046	560	232	683	184
EXOSC1__NM_016046_TTTCCGGAATTCTTTAGTGT	EXOSC1__NM_016046	374	475	333	429
EXOSC10__NM_001001998_AACCAAATCCGACGGAGAGA	EXOSC10__NM_001001998	572	201	757	182
EXOSC10__NM_001001998_ACCAGCGCAACCAAATCCGA	EXOSC10__NM_001001998	902	446	1030	519
EXOSC10__NM_001001998_AGGGAGACAGGTTGCTTCAG	EXOSC10__NM_001001998	323	35	304	18
EXOSC10__NM_001001998_AGTGTCCAGCTGGAACCGTA	EXOSC10__NM_001001998	600	273	602	345
EXOSC10__NM_001001998_CACCATCTCTCCGTCGGATT	EXOSC10__NM_001001998	2152	1614	2212	2322
EXOSC10__NM_001001998_CCTGTGTTTCGCAAAATGCT	EXOSC10__NM_001001998	733	840	915	878
EXOSC10__NM_001001998_GCATTACTCTGCTCATGCTA	EXOSC10__NM_001001998	1405	102	1861	168
EXOSC10__NM_001001998_TAATGTTGCTGCGACACCCA	EXOSC10__NM_001001998	487	101	491	93
EXOSC10__NM_001001998_TGGCAGCACCATCTCTCCGT	EXOSC10__NM_001001998	143	42	177	81
EXOSC10__NM_001001998_TGGTTGCGCTGGTCGCCGAC	EXOSC10__NM_001001998	4	8	9	2
EXOSC10__NM_001001998_TGTCCTCCCTGCCGGCTTGC	EXOSC10__NM_001001998	572	405	597	330
EXOSC10__NM_001001998_TTGATTCTTGTTTACACCTG	EXOSC10__NM_001001998	125	163	104	160
EXOSC2__NM_001282708_AGAAACATCTAGTGGTGCCG	EXOSC2__NM_001282708	272	48	395	19
EXOSC2__NM_001282708_AGGGGCCATGGAACGTATAT	EXOSC2__NM_001282708	344	0	409	23
EXOSC2__NM_001282708_CACTTTCATGATTTGCCATG	EXOSC2__NM_001282708	1019	1322	1184	1370
EXOSC2__NM_001282708_CAGGGGCCATGGAACGTATA	EXOSC2__NM_001282708	249	6	262	13
EXOSC2__NM_001282708_CGGCCCAGTCTCTCGCTAAG	EXOSC2__NM_001282708	822	237	941	251
EXOSC2__NM_001282708_CTAAGAGGCTTGCGAGCCAC	EXOSC2__NM_001282708	77	3	136	30
EXOSC2__NM_001282708_CTAGATGTTTCTTAGTGTCG	EXOSC2__NM_001282708	768	396	921	282
EXOSC2__NM_001282708_CTTCTCCCATATACGTTCCA	EXOSC2__NM_001282708	600	190	609	211
EXOSC2__NM_001282708_GAAGTAGGAGACATCGTAGT	EXOSC2__NM_001282708	637	5	781	13
EXOSC2__NM_001282708_GCCGGGGGATACAATCACTA	EXOSC2__NM_001282708	471	49	560	95
EXOSC2__NM_001282708_TAAGAAACATCTAGTGGTGC	EXOSC2__NM_001282708	635	383	866	371
EXOSC2__NM_001282708_TGAAGTAGGAGACATCGTAG	EXOSC2__NM_001282708	273	10	376	15
EXOSC2__NM_001282709_GAGCTGTCTCTTTGCACACG	EXOSC2__NM_001282709	1005	190	1100	342
EXOSC2__NM_014285_AGCAAAGAAAAATGGAGACT	EXOSC2__NM_014285	175	187	226	207
EXOSC2__NM_014285_ATCCTATAATTGAAGTAGTG	EXOSC2__NM_014285	87	43	121	128
EXOSC2__NM_014285_CCAGCTGAGTCCCGGTATTA	EXOSC2__NM_014285	865	516	894	577
EXOSC2__NM_014285_CTCAGTCATTTAAAGGATAT	EXOSC2__NM_014285	100	72	93	137
EXOSC2__NM_014285_CTGCATTGACGGCCCTGTGA	EXOSC2__NM_014285	642	793	808	911
EXOSC2__NM_014285_GCACAGTGTTACAGTCGAAT	EXOSC2__NM_014285	1583	1407	1755	1345
EXOSC2__NM_014285_GCCAGCTGAGTCCCGGTATT	EXOSC2__NM_014285	2232	1997	2310	2335
EXOSC2__NM_014285_GCTCCAGAAGCACGGGACTG	EXOSC2__NM_014285	627	1022	734	1029
EXOSC2__NM_014285_GGAGGCCAAAACAGACCATT	EXOSC2__NM_014285	985	810	1102	908
EXOSC2__NM_014285_GGAGTGAAGACTGTGATGTG	EXOSC2__NM_014285	1039	1227	958	1145
EXOSC2__NM_014285_TCTCCATATTATTCCAGGAT	EXOSC2__NM_014285	381	317	416	335
EXOSC2__NM_014285_TCTTTGCTGAGCCACATATG	EXOSC2__NM_014285	386	403	421	412
EXOSC2__NM_014285_TGAAACTATTCCCTAATACC	EXOSC2__NM_014285	199	204	310	243
EXOSC2__NM_014285_TGCACAGTGTTACAGTCGAA	EXOSC2__NM_014285	397	584	422	584
EXOSC2__NM_014285_TGCCACCCAGGCTTGGTGTT	EXOSC2__NM_014285	1198	1123	1260	1271
EXOSC2__NM_014285_TTCATGTTTATCTGAGTACA	EXOSC2__NM_014285	34	40	47	125
EXOSC3__NM_001002269_AGACGCAGGTTCGGCCATCG	EXOSC3__NM_001002269	2481	67	3166	129
EXOSC3__NM_001002269_CAGCGGCGGCGGTGTTTACT	EXOSC3__NM_001002269	804	82	990	38
EXOSC3__NM_001002269_CGGCGGCGGTGTTTACTGGG	EXOSC3__NM_001002269	770	90	922	44
EXOSC3__NM_001002269_GAATGCTAGAGCGTGCTCGC	EXOSC3__NM_001002269	142	19	232	112
EXOSC3__NM_001002269_GATTCAGCCGCGACAGACGC	EXOSC3__NM_001002269	85	51	95	50
EXOSC3__NM_001002269_GCGAGCACGCTCTAGCATTC	EXOSC3__NM_001002269	668	191	800	390
EXOSC3__NM_001002269_GTCCGGGCCTTCGGCGCTGT	EXOSC3__NM_001002269	224	25	233	7
EXOSC3__NM_001002269_GTCGCGGCTGAATCTCTCGC	EXOSC3__NM_001002269	1089	616	1414	942
EXOSC3__NM_001002269_TCGCGGGTGCGCGTTGTATG	EXOSC3__NM_001002269	391	82	481	35
EXOSC3__NM_001002269_TGAATGCTAGAGCGTGCTCG	EXOSC3__NM_001002269	52	67	45	5
EXOSC3__NM_001002269_TGCACGCACAGTACTAGGTC	EXOSC3__NM_001002269	98	24	158	45
EXOSC3__NM_001002269_TGCCGGGCTCCTTGTGACGG	EXOSC3__NM_001002269	824	199	978	126
EXOSC4__NM_019037_ACTATATTGACAGTTCACTA	EXOSC4__NM_019037	324	7	297	34
EXOSC4__NM_019037_AGATATCAATCTGGAGCGT	EXOSC4__NM_019037	111	0	160	53
EXOSC4__NM_019037_AGCGCAAGCGACGGCCACAT	EXOSC4__NM_019037	687	316	900	306
EXOSC4__NM_019037_CACTAGGGCCCTGTCCGGCA	EXOSC4__NM_019037	1428	328	1611	498
EXOSC4__NM_019037_CAGGACTTACGGTCCCCATG	EXOSC4__NM_019037	585	267	502	152
EXOSC4__NM_019037_GACCAGGGCTACCGGGTGGA	EXOSC4__NM_019037	335	14	432	3
EXOSC4__NM_019037_GCACAGGTGAGCGCAAGCGA	EXOSC4__NM_019037	71	32	76	50
EXOSC4__NM_019037_GCGCAAGCGACGGCCACATG	EXOSC4__NM_019037	600	325	719	370
EXOSC4__NM_019037_GCGGATGGGCGTGTTCGCGC	EXOSC4__NM_019037	614	20	734	50
EXOSC4__NM_019037_GCTGGAGCTCTTGTCGGACC	EXOSC4__NM_019037	450	170	548	205
EXOSC4__NM_019037_GGCGGGGCTGGAGCTCTTGT	EXOSC4__NM_019037	333	138	406	187
EXOSC4__NM_019037_GGCTCGGCCTACATTGAGCA	EXOSC4__NM_019037	765	668	807	790
EXOSC5__NM_020158_ACGGAAGACGTGTTGCGAAG	EXOSC5__NM_020158	1465	189	1788	258
EXOSC5__NM_020158_AGCCATCTGGCCGCGACAGC	EXOSC5__NM_020158	56	49	41	67
EXOSC5__NM_020158_AGGACTGCCCGGGCCTCCTA	EXOSC5__NM_020158	185	0	215	1
EXOSC5__NM_020158_CCACAGAAGAGAGCCCGCAT	EXOSC5__NM_020158	645	62	752	27
EXOSC5__NM_020158_CCTCAGGATCACTTCGAGTG	EXOSC5__NM_020158	438	50	587	101
EXOSC5__NM_020158_CTGTCCTGGCGGGTGTGTAC	EXOSC5__NM_020158	757	68	839	89
EXOSC5__NM_020158_GCAGCGATTCCCGGTAGAAA	EXOSC5__NM_020158	465	226	486	273
EXOSC5__NM_020158_GCCAAAATCCGTGCTGAAAA	EXOSC5__NM_020158	498	515	571	706
EXOSC5__NM_020158_GTGATCCTGAGGCCGAAGAT	EXOSC5__NM_020158	143	48	127	143
EXOSC5__NM_020158_TGCCGCCTGCATGGCATTGG	EXOSC5__NM_020158	573	35	567	16
EXOSC6__NM_058219_ACACGGGCCGTAGCCGCGTT	EXOSC6__NM_058219	462	8	550	83
EXOSC6__NM_058219_CATGCCTGTGCTGAATCAGG	EXOSC6__NM_058219	597	6	854	11
EXOSC6__NM_058219_CCCGCGACCCAACGCGGCTA	EXOSC6__NM_058219	37	17	60	13
EXOSC6__NM_058219_CGCTCTTCCTCGAGCCGCGT	EXOSC6__NM_058219	1106	123	1412	198
EXOSC6__NM_058219_CGTGGAGATGTACGACCTGG	EXOSC6__NM_058219	1008	16	1408	15
EXOSC6__NM_058219_GCGGCGGCTGCGATTCTTCA	EXOSC6__NM_058219	1494	340	1885	513
EXOSC6__NM_058219_GCTCATGCCTGTGCTGAATC	EXOSC6__NM_058219	662	660	810	785
EXOSC6__NM_058219_TACACGGGCCGTAGCCGCGT	EXOSC6__NM_058219	7	0	23	0
EXOSC6__NM_058219_TCGGCCGCGTACAGCTGCGG	EXOSC6__NM_058219	1189	535	1295	605
EXOSC6__NM_058219_TGGGCGGAGGCCGTACGCCT	EXOSC6__NM_058219	101	0	123	0
EXOSC7__NM_015004_AACTCCAAGTAGCCTTCATT	EXOSC7__NM_015004	674	896	742	645
EXOSC7__NM_015004_AGAACCCAGCAGTGCTCCCG	EXOSC7__NM_015004	111	42	133	3
EXOSC7__NM_015004_AGATCGCTAACACCCTCTAT	EXOSC7__NM_015004	1087	146	1156	105
EXOSC7__NM_015004_AGTGCTACCCCTGAATTTGA	EXOSC7__NM_015004	647	581	642	522
EXOSC7__NM_015004_ATAGAGGGTGTTAGCGATCT	EXOSC7__NM_015004	199	175	125	63
EXOSC7__NM_015004_CTTAGTCGTATGCAGTCATA	EXOSC7__NM_015004	151	71	159	164
EXOSC7__NM_015004_GACCTCCGTGTGGATGGCCG	EXOSC7__NM_015004	618	918	767	822
EXOSC7__NM_015004_GAGAAGGTGTACATCGTGCA	EXOSC7__NM_015004	588	0	715	9
EXOSC7__NM_015004_GAGAGCAGCCTTTACAGCAA	EXOSC7__NM_015004	298	28	338	18
EXOSC7__NM_015004_GATGTGGTGTCCAACACTAG	EXOSC7__NM_015004	202	25	218	51
EXOSC7__NM_015004_GCTCCCGAGGACTAATGCAG	EXOSC7__NM_015004	470	41	481	53
EXOSC7__NM_015004_TGACTGCATACGACTAAGTG	EXOSC7__NM_015004	147	26	149	26
EXOSC8__NM_181503_ACCGGAATCTCGATGAACAC	EXOSC8__NM_181503	281	98	306	92
EXOSC8__NM_181503_AGAACCACAACTGTCAACAT	EXOSC8__NM_181503	518	51	631	44
EXOSC8__NM_181503_CACAACTGTCAACATCGGTA	EXOSC8__NM_181503	107	0	101	5
EXOSC8__NM_181503_CCCTGTGTTCATCGAGATTC	EXOSC8__NM_181503	850	1178	992	1257
EXOSC8__NM_181503_CTTACCGATGTTGACAGTTG	EXOSC8__NM_181503	415	20	434	54
EXOSC8__NM_181503_GCCCCTGATAAAGGATACGT	EXOSC8__NM_181503	373	163	404	55
EXOSC8__NM_181503_GGTTCAATTAGTACCGCAGA	EXOSC8__NM_181503	52	0	108	6
EXOSC8__NM_181503_GGTTCTGCTTTAGTGAAGTT	EXOSC8__NM_181503	32	15	48	11
EXOSC8__NM_181503_TCAACAGATGCCCCTGATAA	EXOSC8__NM_181503	409	174	530	210
EXOSC8__NM_181503_TGGAACCTCTGGAGTATTAC	EXOSC8__NM_181503	10	0	10	0
EXOSC8__NM_181503_TGTTCATCGAGATTCCGGTC	EXOSC8__NM_181503	247	105	166	105
EXOSC8__NM_181503_TTATCAGGGGCATCTGTTGA	EXOSC8__NM_181503	340	98	416	177
EXOSC9__NM_001034194_AAACTCAATCGGGCAACAGA	EXOSC9__NM_001034194	67	8	58	9
EXOSC9__NM_001034194_ACACGTTCTTCTCGTTCATT	EXOSC9__NM_001034194	1251	1707	1489	1829
EXOSC9__NM_001034194_AGGAACATCAGGATCTCATT	EXOSC9__NM_001034194	157	12	279	5
EXOSC9__NM_001034194_AGGTCTTCGGAAATGACATA	EXOSC9__NM_001034194	107	1	138	7
EXOSC9__NM_001034194_ATCTCATTTGGAACAGATTA	EXOSC9__NM_001034194	60	35	150	57
EXOSC9__NM_001034194_ATTTGTACTATCCAGTCCAG	EXOSC9__NM_001034194	168	5	154	6
EXOSC9__NM_001034194_CCTGATGTTCCTATAATCAT	EXOSC9__NM_001034194	766	261	855	413
EXOSC9__NM_001034194_CCTTCTTCTCTTCGATGGCA	EXOSC9__NM_001034194	426	457	477	645
EXOSC9__NM_001034194_GCCGCTCCAGCTTTCGAACC	EXOSC9__NM_001034194	1425	315	1695	475
EXOSC9__NM_001034194_GGTGAAGTTGAATCGACTCA	EXOSC9__NM_001034194	1626	609	2117	967
EXOSC9__NM_001034194_TGCCAGCATTGCTGCAATCG	EXOSC9__NM_001034194	84	5	120	0
EXOSC9__NM_001034194_TTGTGTCTCCAAAACTCAAT	EXOSC9__NM_001034194	299	480	320	356
EXPH5__NM_015065_AAAGCCAGCAGTCATGAGGG	EXPH5__NM_015065	310	190	393	280
EXPH5__NM_015065_AAGAAACTGAAATCAAACGC	EXPH5__NM_015065	219	103	256	296
EXPH5__NM_015065_ACTTACATACAGGCTAAGTA	EXPH5__NM_015065	369	270	427	435
EXPH5__NM_015065_AGACAAAGAGGGATATCAGA	EXPH5__NM_015065	308	229	444	304
EXPH5__NM_015065_ATCCGGGAAGAAAAAGGTGT	EXPH5__NM_015065	107	109	130	103
EXPH5__NM_015065_ATCTATGACATCCTAAGACC	EXPH5__NM_015065	85	161	125	69
EXPH5__NM_015065_ATGACGATCTGAAGCTCATC	EXPH5__NM_015065	145	91	161	103
EXPH5__NM_015065_ATTACCATGTCTGTTACCAC	EXPH5__NM_015065	1182	1314	1182	1329
EXPH5__NM_015065_CAGATTTCCTGAATGAGAAC	EXPH5__NM_015065	268	240	228	308
EXPH5__NM_015065_GATGTCATAGATAGACATAT	EXPH5__NM_015065	214	195	202	156
EXPH5__NM_015065_GGGCCGAGAAGGACAGGATC	EXPH5__NM_015065	357	315	513	420
EXPH5__NM_015065_TAAAACAACCACTTACATAC	EXPH5__NM_015065	487	424	414	618
EXT1__NM_000127_AAACATTCTAGCGGCCATCG	EXT1__NM_000127	195	206	235	162
EXT1__NM_000127_ACGAAGGGGCGCAGAGCGTC	EXT1__NM_000127	99	274	108	108
EXT1__NM_000127_CACTTGGCCTGACTACACCG	EXT1__NM_000127	1105	1111	1319	963
EXT1__NM_000127_CCGGGGGGAAATGTGCACGC	EXT1__NM_000127	1061	1211	1038	1048
EXT1__NM_000127_CCTGACTACACCGAGGACGT	EXT1__NM_000127	1531	1360	1549	974
EXT1__NM_000127_GCAAAGGGTGAAATCGAAGC	EXT1__NM_000127	236	264	202	205
EXT1__NM_000127_GCTTGCAGTTTAGGGCATCG	EXT1__NM_000127	947	973	1108	1100
EXT1__NM_000127_GGGCCAGAAATGATCCGGAC	EXT1__NM_000127	609	839	639	652
EXT1__NM_000127_GGGGTCCGAGGTGTAGAACC	EXT1__NM_000127	125	127	154	121
EXT1__NM_000127_TCCACTTGTGGAACAATGGT	EXT1__NM_000127	96	67	76	17
EXT1__NM_000127_TTGGGATCAATTGGAAAACG	EXT1__NM_000127	77	64	98	71
EXT1__NM_000127_TTTCGGAGGCTTGCAGTTTA	EXT1__NM_000127	139	150	119	177
EXT2__NM_000401_AGAGGGTGATATAGTAGATT	EXT2__NM_000401	215	208	264	107
EXT2__NM_000401_ATTTGAGGACTCGATAGAAT	EXT2__NM_000401	326	292	306	356
EXT2__NM_000401_CATTTGAGGACTCGATAGAA	EXT2__NM_000401	266	224	229	320
EXT2__NM_000401_CCAAAACTGAAACATGCCAG	EXT2__NM_000401	705	746	763	652
EXT2__NM_000401_CGTCGGTCAAGTATAATATC	EXT2__NM_000401	264	268	313	241
EXT2__NM_000401_CTATCGAGTCCTCAAATGAC	EXT2__NM_000401	486	609	468	579
EXT2__NM_000401_CTGTGTCATTATGTGTGCGT	EXT2__NM_000401	649	704	752	744
EXT2__NM_000401_GATATAGTAGATTCGGTGCT	EXT2__NM_000401	468	559	551	453
EXT2__NM_000401_GCCTAACAACCGGCACATCA	EXT2__NM_000401	748	1094	835	916
EXT2__NM_000401_GTCGGTCAAGTATAATATCC	EXT2__NM_000401	135	181	152	130
EXT2__NM_000401_TCGGTCAAGTATAATATCCG	EXT2__NM_000401	440	476	585	451
EXT2__NM_000401_TTTGAGGACTCGATAGAATG	EXT2__NM_000401	73	164	128	145
EXT2__NM_001178083_TCGGCTGGCAGCCTAACAAC	EXT2__NM_001178083	68	42	35	61
EXTL1__NM_004455_AAGCCCACCCGTTGCGAGGT	EXTL1__NM_004455	883	1222	1034	1075
EXTL1__NM_004455_AGCCCCACCTCGCAACGGGT	EXTL1__NM_004455	990	1024	1040	1025
EXTL1__NM_004455_CAATGCCTCTGCAATGGAAC	EXTL1__NM_004455	287	297	323	480
EXTL1__NM_004455_CAGGAGCCCCACCTCGCAAC	EXTL1__NM_004455	1532	1206	1464	670
EXTL1__NM_004455_CCATCTCTGAGACTCATCGC	EXTL1__NM_004455	624	698	769	490
EXTL1__NM_004455_CCTGAAGCCCACCCGTTGCG	EXTL1__NM_004455	710	1148	817	1061
EXTL1__NM_004455_CTGGGTACACGAATACCTTA	EXTL1__NM_004455	323	296	363	280
EXTL1__NM_004455_GAGCCCCACCTCGCAACGGG	EXTL1__NM_004455	375	329	363	213
EXTL1__NM_004455_GTGCAGGGGCGATGGCCTTA	EXTL1__NM_004455	491	337	438	274
EXTL1__NM_004455_TCAGAGATGGTTCCAACCGC	EXTL1__NM_004455	166	150	191	154
EXTL1__NM_004455_TGAGACTCATCGCAGGATCC	EXTL1__NM_004455	147	105	186	145
EXTL1__NM_004455_TTCTCCTCCACGACTGCATG	EXTL1__NM_004455	2	2	2	0
EXTL2__NM_001033025_AAACTTCCTGGGAGAGTAAT	EXTL2__NM_001033025	156	107	178	204
EXTL2__NM_001033025_AAATCTGCACAAAGTGATTG	EXTL2__NM_001033025	188	89	243	253
EXTL2__NM_001033025_AACTTCCTGGGAGAGTAATG	EXTL2__NM_001033025	182	192	186	190
EXTL2__NM_001033025_ACAAGATGCTCATGTTGCGT	EXTL2__NM_001033025	621	477	566	399
EXTL2__NM_001033025_ACAATCACTTTGTGCAGATT	EXTL2__NM_001033025	340	325	404	350
EXTL2__NM_001033025_AGAGAATTCCATAATTCATC	EXTL2__NM_001033025	81	40	76	46
EXTL2__NM_001033025_ATTGTGGTATGGAACAATAT	EXTL2__NM_001033025	558	307	558	355
EXTL2__NM_001033025_CAGCTACCAGTAATAATACG	EXTL2__NM_001033025	1203	805	1365	1238
EXTL2__NM_001033025_CGTAGGGAAATAAAATCCCA	EXTL2__NM_001033025	435	416	455	383
EXTL2__NM_001033025_CGTCTGCATTATGAGAGTAA	EXTL2__NM_001033025	157	223	228	125
EXTL2__NM_001033025_CTCGTATTATTACTGGTAGC	EXTL2__NM_001033025	310	489	333	317
EXTL2__NM_001033025_GGTCATCCTCGTATTATTAC	EXTL2__NM_001033025	268	372	278	402
EXTL3__NM_001440_CAGAGTGGTGAGGTAATAGT	EXTL3__NM_001440	567	725	658	605
EXTL3__NM_001440_CAGTACTGACGTTATAGAGA	EXTL3__NM_001440	296	353	480	474
EXTL3__NM_001440_CCTCCCCCGAAGGCCACTCG	EXTL3__NM_001440	170	184	250	289
EXTL3__NM_001440_CCTTGTAGGAATGCTCGGTC	EXTL3__NM_001440	603	723	617	564
EXTL3__NM_001440_CTCCCACTAGTATCACGTAA	EXTL3__NM_001440	232	282	376	282
EXTL3__NM_001440_CTGCCTTTACGTGATACTAG	EXTL3__NM_001440	466	358	434	554
EXTL3__NM_001440_CTGGGAGCAGTCGGATGGGC	EXTL3__NM_001440	662	759	722	945
EXTL3__NM_001440_CTTCTCTATAACGTCAGTAC	EXTL3__NM_001440	127	128	145	236
EXTL3__NM_001440_GCTATACCATGCTGCGGAAT	EXTL3__NM_001440	1725	2177	1662	2084
EXTL3__NM_001440_TCACTGTCATAGACGTAGAC	EXTL3__NM_001440	348	400	245	440
EXTL3__NM_001440_TGCCTTTACGTGATACTAGT	EXTL3__NM_001440	239	240	304	313
EXTL3__NM_001440_TGTCCATCCGTCCGCCAGTG	EXTL3__NM_001440	1211	1636	1583	1149
EYA1__NM_000503_AAACTACTGCTCCCAATTGC	EYA1__NM_000503	568	399	461	473
EYA1__NM_000503_ACAGCTTCAACGACAGCCGA	EYA1__NM_000503	543	749	641	823
EYA1__NM_000503_ACTCAGACGGCTATGCGGGC	EYA1__NM_000503	404	385	393	464
EYA1__NM_000503_AGAACTGGTGAGTTGGTCGT	EYA1__NM_000503	710	812	726	650
EYA1__NM_000503_AGCCATAGTTGTGAGGAAG	EYA1__NM_000503	379	505	471	529
EYA1__NM_000503_CAGCTTCAACGACAGCCGAC	EYA1__NM_000503	398	464	530	536
EYA1__NM_000503_GAAATTGTTTAAAGACCCGT	EYA1__NM_000503	256	142	204	202
EYA1__NM_000503_GGTGGAGAGAACTGGTGAGT	EYA1__NM_000503	658	541	696	464
EYA1__NM_000503_TACCACTCAGACGGCTATGC	EYA1__NM_000503	230	337	276	164
EYA1__NM_000503_TACCCCTTCCTCACAAACTA	EYA1__NM_000503	237	197	394	352
EYA1__NM_000503_TATATGAGAGTTACCGAGTT	EYA1__NM_000503	265	170	276	240
EYA1__NM_000503_TATGAGAGTTACCGAGTTTG	EYA1__NM_000503	415	354	381	472
EYA1__NM_001288575_ACATCATCAGGAATATATAC	EYA1__NM_001288575	392	559	412	309
EYA1__NM_001288575_AGATGGGAAATCACGTGGAC	EYA1__NM_001288575	730	900	830	836
EYA1__NM_001288575_AGGACCCAGTAAGCAAGGAG	EYA1__NM_001288575	87	53	83	85
EYA1__NM_001288575_CAGCTTCAAGAACCGCCATC	EYA1__NM_001288575	679	592	801	687
EYA1__NM_001288575_CGTACCTCAAGATCAGAATC	EYA1__NM_001288575	352	465	477	564
EYA1__NM_001288575_CTGCTGGTCATATAATGTGC	EYA1__NM_001288575	514	526	582	539
EYA1__NM_001288575_GAATCTTTAATGGGTGTTGA	EYA1__NM_001288575	141	199	103	239
EYA1__NM_001288575_GATTCTGATCGATTGCGTCG	EYA1__NM_001288575	145	212	145	192
EYA1__NM_001288575_GATTGCGTCGAGGTTCAGAT	EYA1__NM_001288575	603	704	636	815
EYA1__NM_001288575_GCTTGGCTGGTGATGCCAGA	EYA1__NM_001288575	561	703	786	969
EYA1__NM_001288575_GTACCTCAAGATCAGAATCT	EYA1__NM_001288575	313	445	329	374
EYA1__NM_001288575_TGGTAAGTGGCATTGGTGGA	EYA1__NM_001288575	0	0	2	14
EYA2__NM_005244_AAGAGCTGGGACACTCTCAG	EYA2__NM_005244	827	770	1067	1032
EYA2__NM_005244_ACTGTACTGCGTCTGGCCGT	EYA2__NM_005244	1584	1673	1651	1734
EYA2__NM_005244_CACTGTAAACAGCGATTGTC	EYA2__NM_005244	406	595	392	574
EYA2__NM_005244_CCACCTCCAGCACAAGCCTA	EYA2__NM_005244	275	126	451	466
EYA2__NM_005244_CGTGCATCTGGTAGGTGTAT	EYA2__NM_005244	1793	1600	2173	2228
EYA2__NM_005244_CTGGGCAACGCAGCCGGTTT	EYA2__NM_005244	610	725	729	1031
EYA2__NM_005244_GAGGTGGGTAAGCTGTATAG	EYA2__NM_005244	73	149	94	52
EYA2__NM_005244_GGAGGTGGGTAAGCTGTATA	EYA2__NM_005244	77	92	83	117
EYA2__NM_005244_GGCACAACAGGGTTCTATCA	EYA2__NM_005244	219	265	339	288
EYA2__NM_005244_TATCAAGGAGGAAATGGACT	EYA2__NM_005244	260	191	284	214
EYA2__NM_005244_TCCACAGCCATGGCAGCCTA	EYA2__NM_005244	277	425	448	333
EYA2__NM_005244_TCCCCGCACTGTACTGCGTC	EYA2__NM_005244	1034	1115	1141	1349
EYA3__NM_001282560_AATCAGGAGAGCAAACTATA	EYA3__NM_001282560	140	127	156	128
EYA3__NM_001282560_AGGAGGTAGTCCATACGTTT	EYA3__NM_001282560	612	536	558	406
EYA3__NM_001282560_ATCTGGATTGCTTACTTGAC	EYA3__NM_001282560	414	332	401	333
EYA3__NM_001282560_CATTTGTGAGGTATAATCAT	EYA3__NM_001282560	399	585	552	643
EYA3__NM_001282560_CCTCAGGCAACCCAAACGTA	EYA3__NM_001282560	167	98	170	93
EYA3__NM_001282560_CTTTGCAGAATACATTTGTG	EYA3__NM_001282560	770	768	855	948
EYA3__NM_001282560_GCCTGAGGGTAGACAGCATA	EYA3__NM_001282560	5	0	2	9
EYA3__NM_001282560_GTTTCGGAAACTGCTTACCC	EYA3__NM_001282560	189	110	325	310
EYA3__NM_001282560_TACTTTCCTCTGACATGGGA	EYA3__NM_001282560	182	174	250	294
EYA3__NM_001282560_TCTGGATTGCTTACTTGACT	EYA3__NM_001282560	698	890	909	640
EYA3__NM_001282560_TTCAAACCTTCCCATGTCAG	EYA3__NM_001282560	15	4	7	12
EYA3__NM_001282560_TTGTAGAAGAAGTAGATATC	EYA3__NM_001282560	15	41	15	4
EYA3__NM_001282562_AGTAGCCAGCATCTCAAACC	EYA3__NM_001282562	288	200	267	341
EYA3__NM_001282562_ATGGGATAAGAATAATGTGC	EYA3__NM_001282562	137	165	190	189
EYA3__NM_001282562_CATTATTCTTATCCCATTCA	EYA3__NM_001282562	200	349	281	412
EYA3__NM_001282562_CCACTTTCAGGTTTCATACC	EYA3__NM_001282562	310	263	296	233
EYA3__NM_001282562_TCATACCTGGTTTGAGATGC	EYA3__NM_001282562	808	569	848	952
EYA4__NM_004100_ACCACTGAAATTCGTTGAAT	EYA4__NM_004100	1174	2278	1453	1691
EYA4__NM_004100_AGAACCCTGGGCTGTAACTG	EYA4__NM_004100	709	715	783	557
EYA4__NM_004100_AGCACATCAGTTACTACAAA	EYA4__NM_004100	391	651	406	484
EYA4__NM_004100_CCTTATTCTTACCAAATGCC	EYA4__NM_004100	329	648	414	368
EYA4__NM_004100_GCAAGTCCTCATACTCTTGT	EYA4__NM_004100	1236	1723	1468	1752
EYA4__NM_004100_GCACATCAGTTACTACAAAT	EYA4__NM_004100	1015	932	1197	1133
EYA4__NM_004100_GCAGGCCAGACTCAGTATTC	EYA4__NM_004100	631	325	690	539
EYA4__NM_004100_GCATCCCCGAATACTGAGTC	EYA4__NM_004100	878	1033	1098	1219
EYA4__NM_004100_GGCTGTGGGGTAGAGAACCC	EYA4__NM_004100	195	106	269	178
EYA4__NM_004100_GTCCTGCATTTCCATAGACC	EYA4__NM_004100	398	215	401	301
EYA4__NM_004100_TCCAATTCAACGAATTTCAG	EYA4__NM_004100	142	142	162	280
EYA4__NM_004100_TTTGGTAAGAATAAGGTGTC	EYA4__NM_004100	8	30	13	14
EYS__NM_001142800_AAAAACATGTAGACGGCAAT	EYS__NM_001142800	480	622	502	528
EYS__NM_001142800_AACATGTAGACGGCAATTGG	EYS__NM_001142800	721	801	754	679
EYS__NM_001142800_AAGAGCTGTGAAAAACCATC	EYS__NM_001142800	247	151	311	187
EYS__NM_001142800_AAGTAACTGATCTTCCGTTG	EYS__NM_001142800	493	537	506	601
EYS__NM_001142800_ACATTTAGTCGAAGTCCCAG	EYS__NM_001142800	827	784	811	928
EYS__NM_001142800_AGGTGACTCCACAGTAGCAG	EYS__NM_001142800	152	114	172	165
EYS__NM_001142800_ATCTTGGCAAATACCATGGA	EYS__NM_001142800	316	449	388	393
EYS__NM_001142800_CAATTTACCACATATGATGA	EYS__NM_001142800	286	416	327	468
EYS__NM_001142800_CCTCATCATATGTGGTAAAT	EYS__NM_001142800	327	432	310	448
EYS__NM_001142800_GTTTCTGAAACATCTTTCGT	EYS__NM_001142800	95	137	106	169
EYS__NM_001142800_TTTATCACAGTTATGGCAAG	EYS__NM_001142800	154	286	204	235
EYS__NM_001142800_TTTCACAGCTCTTTCATAAA	EYS__NM_001142800	71	131	63	93
EZH1__NM_001991_ACAGGCTTCATTGACTGAAC	EZH1__NM_001991	608	638	589	569
EZH1__NM_001991_AGCTGATCAATAACTATGAT	EZH1__NM_001991	492	412	475	560
EZH1__NM_001991_CCAGTAAGTGATACATTTGG	EZH1__NM_001991	944	630	860	824
EZH1__NM_001991_CCCTCTCCAACAGAACTTTA	EZH1__NM_001991	101	165	127	163
EZH1__NM_001991_CCTCCAAATGTATCACTTAC	EZH1__NM_001991	732	978	878	1113
EZH1__NM_001991_GAGCTGATCAATAACTATGA	EZH1__NM_001991	380	399	440	417
EZH1__NM_001991_GGGGACCAGGAATACATGAT	EZH1__NM_001991	615	484	778	850
EZH1__NM_001991_GTGACCTCATTAACATATGT	EZH1__NM_001991	292	415	414	331
EZH1__NM_001991_TCCCGGGAAAATGCTCTCTA	EZH1__NM_001991	387	559	524	526
EZH1__NM_001991_TTCACTTCATCTCCCATGTA	EZH1__NM_001991	48	63	47	25
EZH1__NM_001991_TTTCACTTCATCTCCCATGT	EZH1__NM_001991	457	594	631	663
EZH1__NM_001991_TTTCCAGTAAGTGATACATT	EZH1__NM_001991	235	221	228	283
EZH2__NM_001203247_AAAACTTCATCTCCCATATA	EZH2__NM_001203247	191	195	245	296
EZH2__NM_001203247_ACTGCATTCAGAGTCTTTAA	EZH2__NM_001203247	422	374	432	431
EZH2__NM_001203247_ATCTGGAAACATTGAGGAAA	EZH2__NM_001203247	192	230	158	198
EZH2__NM_001203247_ATTCAGGAGGAAGTGCGCCT	EZH2__NM_001203247	350	190	420	347
EZH2__NM_001203247_CAGAAGGAAATTTCCGAGGT	EZH2__NM_001203247	483	284	575	405
EZH2__NM_001203247_GAATGTACCCCCAACATAGA	EZH2__NM_001203247	60	23	62	67
EZH2__NM_001203247_GGGAAAGTACACGGGGATAG	EZH2__NM_001203247	93	26	78	92
EZH2__NM_001203247_TACCATAAAATTCTGCTGTA	EZH2__NM_001203247	914	599	947	1057
EZH2__NM_001203247_TCAGAAGGAAATTTCCGAGG	EZH2__NM_001203247	540	229	566	572
EZH2__NM_001203247_TCTATGTTGGGGGTACATTC	EZH2__NM_001203247	247	109	204	218
EZH2__NM_001203247_TTGCGCGGGACTAGGGAGGT	EZH2__NM_001203247	465	490	511	590
EZH2__NM_001203247_TTTAATGGGATGACTTGTGT	EZH2__NM_001203247	303	394	374	458
EZH2__NM_001203248_CCTACAGAAAAGCGTATGAA	EZH2__NM_001203248	756	656	953	1199
EZH2__NM_152998_CAACACCCAACACTTATAAG	EZH2__NM_152998	323	164	411	437
EZH2__NM_152998_TTCTTCCGCTTATAAGTGTT	EZH2__NM_152998	24	15	43	77
EZR__NM_001111077_ACAAAGAAGTGCACAAGTCT	EZR__NM_001111077	301	199	433	484
EZR__NM_001111077_ATAATAAAGGATTTCCTACC	EZR__NM_001111077	148	115	245	197
EZR__NM_001111077_CCAAGAGCACGGCAGTCTCA	EZR__NM_001111077	669	662	826	804
EZR__NM_001111077_CCAAGGAAAGCCAATCTTTG	EZR__NM_001111077	415	546	399	378
EZR__NM_001111077_CTCAGGTTAACCCCAAAGAT	EZR__NM_001111077	312	277	393	578
EZR__NM_001111077_CTCCACTATGTGGATAATAA	EZR__NM_001111077	36	54	31	53
EZR__NM_001111077_CTGCATCCATGGTGGTAACT	EZR__NM_001111077	219	240	240	201
EZR__NM_001111077_CTTGTTGATTCTCAGACGTG	EZR__NM_001111077	297	225	468	266
EZR__NM_001111077_GCCCGGAACTTGAACTGGAG	EZR__NM_001111077	1165	1431	1306	1564
EZR__NM_001111077_GTGTGGCATGCGGAACACCG	EZR__NM_001111077	333	376	516	346
EZR__NM_001111077_TGGACCAGCACAAACTTACC	EZR__NM_001111077	98	89	105	139
EZR__NM_001111077_TGTGGCATGCGGAACACCGT	EZR__NM_001111077	414	423	510	526
EZR__NM_003379_ACTAAAGACACAAGCGTGGC	EZR__NM_003379	98	118	110	217
EZR__NM_003379_CAGTTGTAATAGTGCCAAGC	EZR__NM_003379	166	263	227	155
EZR__NM_003379_CATCAAGTGCCATGGTTTAG	EZR__NM_003379	309	271	333	488
EZR__NM_003379_CCATGGCACTTGATGTGATG	EZR__NM_003379	1185	1317	1441	1696
EZR__NM_003379_CGAGAATAATCGCGAGAATC	EZR__NM_003379	597	590	587	735
EZR__NM_003379_CTAAAGACACAAGCGTGGCG	EZR__NM_003379	89	138	131	146
EZR__NM_003379_GTATATTTACATGCTTAGAG	EZR__NM_003379	249	381	259	243
EZR__NM_003379_TCCTCCTCCACCACTCACGC	EZR__NM_003379	801	762	972	900
EZR__NM_003379_TGCCAGCTGCGTGGAGTGAC	EZR__NM_003379	436	408	483	513
F10__NM_000504_ACCTGTTTAGAAGGATTCGA	F10__NM_000504	654	872	770	850
F10__NM_000504_AGGACAGCGACAAGACGGTA	F10__NM_000504	166	81	130	99
F10__NM_000504_AGGCCAGGAATGCAAGGACG	F10__NM_000504	288	325	369	358
F10__NM_000504_AGGCCTTGCCGTTGTCAGCC	F10__NM_000504	349	467	374	412
F10__NM_000504_AGGTGCAGGTGTATTCCCCG	F10__NM_000504	1203	778	1422	1158
F10__NM_000504_CCACAGAGTTCTGTTCCTCG	F10__NM_000504	1174	1070	1366	1541
F10__NM_000504_CTTCCATGCACTCTCTTTCG	F10__NM_000504	1761	2017	1964	2103
F10__NM_000504_GAATCCTTCTAAACAGGTGC	F10__NM_000504	58	97	93	133
F10__NM_000504_GCGGGCCTCTTCGTATGAGC	F10__NM_000504	535	583	575	822
F10__NM_000504_GGCCGTCTTTACATTTGCCC	F10__NM_000504	907	1623	1004	1209
F10__NM_000504_GTCAGCCAGGGTGTACCCGC	F10__NM_000504	316	338	271	195
F10__NM_000504_TCCAGGTCGGCTGCATCATA	F10__NM_000504	511	361	631	661
F11__NM_000128_AAACACTGCCAAGAGTGAAT	F11__NM_000128	188	189	245	362
F11__NM_000128_CAATGCTCACACCAAATAAG	F11__NM_000128	253	354	266	268
F11__NM_000128_CAGGCTGGGAAACTGCCTTG	F11__NM_000128	779	719	771	799
F11__NM_000128_CTGTCTGCAAACACCGTATT	F11__NM_000128	898	1269	1127	1067
F11__NM_000128_TAAATGTCTTTGTTGCAAGC	F11__NM_000128	121	167	159	180
F11__NM_000128_TCCTGTGCACTTTCTAATCT	F11__NM_000128	1271	1842	1535	1895
F11__NM_000128_TGAAGGAGGGGACATTACTA	F11__NM_000128	139	201	140	132
F11__NM_000128_TGTCTGCAAACACCGTATTA	F11__NM_000128	321	292	410	357
F11__NM_000128_TTCTGGTTGGTGTCCCTGTT	F11__NM_000128	10	0	25	30
F11__NM_000128_TTGCAACAAAGACATTTATG	F11__NM_000128	209	286	250	336
F11__NM_000128_TTGTGGCGTACGTGAAAAAG	F11__NM_000128	119	217	147	156
F11__NM_000128_TTTATCGAGCTTCGTTATTC	F11__NM_000128	302	438	459	447
F11R__NM_016946_AAGTTGTCCTGTGCCTACTC	F11R__NM_016946	499	413	511	322
F11R__NM_016946_ACAGCTGTGAGGCACGGAAT	F11R__NM_016946	351	370	375	308
F11R__NM_016946_AGAAAAGCCCGAGTAGGCAC	F11R__NM_016946	360	380	479	372
F11R__NM_016946_ATTATAGCAAACGAGTCTGG	F11R__NM_016946	236	151	283	196
F11R__NM_016946_CCCCACCTTCTGAATACACC	F11R__NM_016946	1069	1836	1226	1578
F11R__NM_016946_CCCCTGTCAGCCTCTGATAC	F11R__NM_016946	1163	1240	1343	1321
F11R__NM_016946_CTGTAACACTGCCCAATGCC	F11R__NM_016946	498	568	491	618
F11R__NM_016946_GACTTGAAGGTGATACCAGT	F11R__NM_016946	666	393	637	562
F11R__NM_016946_GGACAAAGGCGCAAGTCGAG	F11R__NM_016946	514	457	491	574
F11R__NM_016946_GGGGATGTTAACTGTAGGCT	F11R__NM_016946	447	553	604	637
F11R__NM_016946_TCAAACTTCCACTCCACACG	F11R__NM_016946	587	401	496	353
F11R__NM_016946_TGTGAGGCACGGAATGGGTA	F11R__NM_016946	332	557	380	379
F12__NM_000505_ACCGGAGCCTTCTGCGACGT	F12__NM_000505	873	773	874	1169
F12__NM_000505_CCAGCGATGGGGATACTGTT	F12__NM_000505	886	1115	1031	1102
F12__NM_000505_CGCTGGTCCTGATCAAAGTT	F12__NM_000505	1325	1653	1569	1820
F12__NM_000505_CTCGGCAGTCACGTTCCGGT	F12__NM_000505	2813	2455	2991	2174
F12__NM_000505_CTCTGTCCACAACACCTCAC	F12__NM_000505	763	1015	836	798
F12__NM_000505_CTTGCTCGGCAGTCACGTTC	F12__NM_000505	476	554	735	666
F12__NM_000505_CTTTGTACTTATGCTCCTTG	F12__NM_000505	285	351	441	464
F12__NM_000505_GAGGCACGGGTTGGTGCGGC	F12__NM_000505	939	929	1027	986
F12__NM_000505_GCTGGTCCTGATCAAAGTTG	F12__NM_000505	459	584	554	636
F12__NM_000505_GGTACAGCTGCCGGTGGTAC	F12__NM_000505	779	819	979	899
F12__NM_000505_TGGTTTCCAGTGAGGTGTTG	F12__NM_000505	0	0	0	0
F12__NM_000505_TTTGATCAGGACCAGCGATG	F12__NM_000505	147	67	194	81
F13A1__NM_000129_AACAAGCTGATTGTCCGCAG	F13A1__NM_000129	119	48	151	80
F13A1__NM_000129_ACAAGCTGATTGTCCGCAGA	F13A1__NM_000129	111	118	122	125
F13A1__NM_000129_CACGAGCGTTCACCTGTTCA	F13A1__NM_000129	1801	2293	2217	2682
F13A1__NM_000129_CCTTGCAGGTTGACGCCCCG	F13A1__NM_000129	2014	2509	2763	3114
F13A1__NM_000129_CTTCCCCCAAATGTATTGTG	F13A1__NM_000129	714	988	796	1015
F13A1__NM_000129_CTTTGTAACTCTGAGACTAT	F13A1__NM_000129	259	235	288	248
F13A1__NM_000129_GAGATGGGACACTAACAAGG	F13A1__NM_000129	49	18	53	25
F13A1__NM_000129_GGAGAGATGGGACACTAACA	F13A1__NM_000129	428	520	534	464
F13A1__NM_000129_GTCCTCTCTCATGACAATCT	F13A1__NM_000129	296	376	330	335
F13A1__NM_000129_TCCGCTGCATTAGAGTTATT	F13A1__NM_000129	1191	1265	1225	1338
F13A1__NM_000129_TCGCTACCCACAGGAGAACA	F13A1__NM_000129	140	278	230	313
F13A1__NM_000129_TTCAGGGTGGAATACGTCAT	F13A1__NM_000129	46	60	47	52
F13B__NM_001994_ACTGGCTACTACACAGCTGG	F13B__NM_001994	465	423	623	515
F13B__NM_001994_ATGTAACCATTACTCAGGTC	F13B__NM_001994	256	187	355	383
F13B__NM_001994_ATTCAAGAGAACATGCGTTA	F13B__NM_001994	187	187	224	233
F13B__NM_001994_CATTCAACAACTTATCGTCA	F13B__NM_001994	822	974	805	855
F13B__NM_001994_CCAGAATCTCCTGTATGCGA	F13B__NM_001994	927	734	938	828
F13B__NM_001994_GCAGAAATACGTTGTGAAGA	F13B__NM_001994	328	424	346	471
F13B__NM_001994_GCTTCAGGGTACAAAACCAC	F13B__NM_001994	387	324	448	342
F13B__NM_001994_GGTCTTCTCAACCAACCTGT	F13B__NM_001994	513	251	456	347
F13B__NM_001994_TCACACCAAAATGTACCAGT	F13B__NM_001994	894	1052	995	1018
F13B__NM_001994_TCAGGAGAACTCTATGCAGA	F13B__NM_001994	436	539	468	469
F13B__NM_001994_TCTTCATAGGTTTGCTTTAC	F13B__NM_001994	416	439	476	544
F13B__NM_001994_TGCGTTATGGTTGCGCTTCA	F13B__NM_001994	329	426	368	456
F2__NM_000506_ACACTGGAGCCTTCGGAGCG	F2__NM_000506	854	688	921	1179
F2__NM_000506_CATGCAGCAAGCTTATCTCG	F2__NM_000506	864	736	881	817
F2__NM_000506_CATGTGAACATCACCCGGTC	F2__NM_000506	1386	1125	1458	972
F2__NM_000506_CCAAGAAGGTGTTGGCTCGC	F2__NM_000506	752	893	904	1133
F2__NM_000506_CCATCCTGGGGCCGACCTAC	F2__NM_000506	191	140	255	281
F2__NM_000506_GCTGGCACTCAATGCCTGAC	F2__NM_000506	591	617	734	762
F2__NM_000506_GGGGTCTGTAGTGTAGCACC	F2__NM_000506	287	248	296	331
F2__NM_000506_GGTCTGGGTACGAACTACCG	F2__NM_000506	317	410	284	334
F2__NM_000506_GTCGGCCCCAGGATGGGTAG	F2__NM_000506	293	385	379	380
F2__NM_000506_GTCTGGGTACGAACTACCGA	F2__NM_000506	279	218	269	360
F2__NM_000506_TCGCTACAGTGACTTGATCC	F2__NM_000506	990	833	958	1114
F2__NM_000506_TCTGGAGTCCTCCACGGCTA	F2__NM_000506	589	453	722	680
F2R__NM_001992_AAGAAATGACCGGGGATCTA	F2R__NM_001992	228	294	301	367
F2R__NM_001992_AATGAAGGTCAAGAAGCCGG	F2R__NM_001992	757	630	706	691
F2R__NM_001992_ACGATGGCCATGATGTTTAG	F2R__NM_001992	1054	1685	1140	1177
F2R__NM_001992_AGTAATAGCTGATCTTAAAG	F2R__NM_001992	34	18	40	55
F2R__NM_001992_CGATGGCCATGATGTTTAGT	F2R__NM_001992	2820	3126	3258	3360
F2R__NM_001992_GAGGCTGACTACAAACACTC	F2R__NM_001992	267	521	273	187
F2R__NM_001992_GGAGCTGGTCAAATATCCGG	F2R__NM_001992	668	686	571	424
F2R__NM_001992_TCCGGCAGTGATTGGCAGTT	F2R__NM_001992	1913	2529	2035	2576
F2R__NM_001992_TGGCCATGATGTTTAGTGGG	F2R__NM_001992	62	82	52	55
F2R__NM_001992_TTCCTGAGAAGAAATGACCG	F2R__NM_001992	12	1	32	31
F2R__NM_001992_TTTCAGGATGAACACAACGA	F2R__NM_001992	109	73	109	39
F2R__NM_001992_TTTGTCCCATCTGTGTACAC	F2R__NM_001992	434	230	427	238
F2RL1__NM_005242_AACACAATTGTGTAGACAAT	F2RL1__NM_005242	426	663	548	526
F2RL1__NM_005242_AAGATTGCCTATCACATACA	F2RL1__NM_005242	396	384	442	403
F2RL1__NM_005242_AATAAGGCTTCTTCCTTTAG	F2RL1__NM_005242	567	715	529	463
F2RL1__NM_005242_ATGTGATAGGCAATCTTCAA	F2RL1__NM_005242	391	534	451	364
F2RL1__NM_005242_CAATTGTGTAGACAATTGGA	F2RL1__NM_005242	237	342	363	330
F2RL1__NM_005242_GGAACCAGTAGATCCTCTAA	F2RL1__NM_005242	547	547	504	506
F2RL1__NM_005242_GGGTTTGCCAAGTAACGGCA	F2RL1__NM_005242	103	201	154	218
F2RL1__NM_005242_GTTGTTGCCATGTATGTGAT	F2RL1__NM_005242	995	1037	1127	1229
F2RL1__NM_005242_TCTAAAGGAAGAAGCCTTAT	F2RL1__NM_005242	336	325	302	345
F2RL1__NM_005242_TGTGATAGGCAATCTTCAAG	F2RL1__NM_005242	439	378	433	277
F2RL1__NM_005242_TTACATGGCCAATCTGGCCT	F2RL1__NM_005242	13	4	6	12
F2RL1__NM_005242_TTGGCCATGTAAATCACAGC	F2RL1__NM_005242	223	458	291	273
F2RL2__NM_001256566_AAGATAGCTTATCATCTCAA	F2RL2__NM_001256566	135	225	195	109
F2RL2__NM_001256566_AAGGTCTTAATGGGTAAGGT	F2RL2__NM_001256566	109	82	93	124
F2RL2__NM_001256566_ACGAAAGGTCTTAATGGGTA	F2RL2__NM_001256566	1285	1900	1553	2014
F2RL2__NM_001256566_AGATAGCTTATCATCTCAAT	F2RL2__NM_001256566	583	673	713	902
F2RL2__NM_001256566_AGGTATCAGTTTAGTACTTA	F2RL2__NM_001256566	175	174	255	225
F2RL2__NM_001256566_ATGTGAAAAATGCTACCATG	F2RL2__NM_001256566	1107	912	1104	1070
F2RL2__NM_001256566_CATGTGAAAAATGCTACCAT	F2RL2__NM_001256566	1397	1496	1671	1458
F2RL2__NM_001256566_GCTCCACGAAAGGTCTTAAT	F2RL2__NM_001256566	302	303	355	360
F2RL2__NM_001256566_GGAACTCTTCAAAAGAATTT	F2RL2__NM_001256566	57	91	65	83
F2RL2__NM_001256566_TACCTCCTGGTGTTTGTAGT	F2RL2__NM_001256566	1565	1895	1792	2069
F2RL2__NM_001256566_TCCTGAAGAAAAGCATCCAC	F2RL2__NM_001256566	471	463	483	414
F2RL2__NM_001256566_TTACCCATTAAGACCTTTCG	F2RL2__NM_001256566	157	166	195	292
F2RL2__NM_004101_AGAATTAAGGCAAAGACGTT	F2RL2__NM_004101	336	476	293	276
F2RL2__NM_004101_AGGCATGAGCCACTACGTCC	F2RL2__NM_004101	856	320	644	308
F2RL2__NM_004101_AGTACCAAGTATAATGGAGA	F2RL2__NM_004101	259	263	302	240
F2RL2__NM_004101_AGTGCCACTGCACGCCAGTC	F2RL2__NM_004101	229	38	257	35
F2RL2__NM_004101_CAGTATGAAATATACAGTCA	F2RL2__NM_004101	164	111	172	159
F2RL2__NM_004101_CAGTGGAGAGAATGGTTAGA	F2RL2__NM_004101	356	399	332	362
F2RL2__NM_004101_CAGTGTCTCGCTTCGTCACC	F2RL2__NM_004101	1447	2064	1509	2238
F2RL2__NM_004101_CCTGTCTAATCACTGAGTTA	F2RL2__NM_004101	932	1246	1446	1302
F2RL2__NM_004101_CTAAGGAAGATTAATCTGGC	F2RL2__NM_004101	464	802	616	809
F2RL2__NM_004101_GACATTAGTGATTAGACAGG	F2RL2__NM_004101	191	347	234	203
F2RL2__NM_004101_GATATTAGTGATTAGACAGC	F2RL2__NM_004101	134	32	58	102
F2RL2__NM_004101_GCAACATACTGTGATGATAC	F2RL2__NM_004101	969	909	984	1199
F2RL2__NM_004101_GTGCCACTGCACGCCAGTCT	F2RL2__NM_004101	111	0	56	0
F2RL2__NM_004101_TGAAACTAGATAAAAGACCC	F2RL2__NM_004101	34	16	34	25
F2RL2__NM_004101_TGCCTGTAGTCTTAGTTACT	F2RL2__NM_004101	181	36	214	49
F2RL3__NM_003950_AGCAGTGCCCGTGAGCTGTC	F2RL3__NM_003950	1451	1827	1646	1822
F2RL3__NM_003950_AGGATTGAGGGCGTGCTGTC	F2RL3__NM_003950	233	161	265	222
F2RL3__NM_003950_AGGTGGTAGGCGATCCGCGG	F2RL3__NM_003950	48	42	59	63
F2RL3__NM_003950_CCAGCGTCTACGACGAGAGC	F2RL3__NM_003950	165	190	232	269
F2RL3__NM_003950_CCCAGCGTCTACGACGAGAG	F2RL3__NM_003950	237	420	268	286
F2RL3__NM_003950_CCGCTCTCGTCGTAGACGCT	F2RL3__NM_003950	838	658	792	913
F2RL3__NM_003950_CGCAGGTGGTAGGCGATCCG	F2RL3__NM_003950	435	474	493	400
F2RL3__NM_003950_CTGCGGTCAGCCTCAGCGCG	F2RL3__NM_003950	339	586	465	512
F2RL3__NM_003950_CTGGCCACGGCCGCACTCTA	F2RL3__NM_003950	244	319	258	177
F2RL3__NM_003950_GCACTCTATGGTCACATGTA	F2RL3__NM_003950	986	1238	1110	1183
F2RL3__NM_003950_GTCATGGCAGAGCACGCGAT	F2RL3__NM_003950	241	467	258	213
F2RL3__NM_003950_TCAGCGCGTGGCCGTAGCGC	F2RL3__NM_003950	205	197	185	105
F3__NM_001178096_AAGTCTACACTGTTCAAATA	F3__NM_001178096	310	320	356	396
F3__NM_001178096_ACTTGTGTAGAGATATAGCC	F3__NM_001178096	142	284	202	194
[illegible]	[illegible]	[illegible]	[illegible]	[illegible]	[illegible]
FA2H__NM_024306_ACCAGGCGGGAGCCGTCGAA	FA2H__NM_024306	180	175	130	129
FA2H__NM_024306_AGGGCCTCTCTAAGACTGTC	FA2H__NM_024306	100	62	113	115
FA2H__NM_024306_AGTAGAAGACGCCGATCACC	FA2H__NM_024306	448	630	650	697
[illegible]	[illegible]	[illegible]	[illegible]	[illegible]	[illegible]
FABP9__NM_001080526_CAGCACCAGAATCTACGAAA	FABP9__NM_001080526	368	322	375	235
FABP9__NM_001080526_CAGTAACTATTAGTGTTGAT	FABP9__NM_001080526	600	726	867	915
FABP9__NM_001080526_TCAACACTAATAGTTACTGT	FABP9__NM_001080526	738	735	709	801
[illegible]	[illegible]	[illegible]	[illegible]	[illegible]	[illegible]
FADS2__NM_004265_TGGTGATGTTGTAAACCTTG	FADS2__NM_004265	8	7	5	9
FADS3__NM_021727_ACCCCAGGACGCCGTAGAA	FADS3__NM_021727	517	501	568	423
FADS3__NM_021727_ACCTTCTTTGCTTTCCTACT	FADS3__NM_021727	1523	2026	1819	1962
[illegible]	[illegible]	[illegible]	[illegible]	[illegible]	[illegible]

FAM120AOS__NM_198841_ACACTGTCGCCTCCGAATTC	FAM120AOS__NM_198841	1236	1375	1550	1649
FAM120AOS__NM_198841_AGGAGCAGGATGCAACCGAC	FAM120AOS__NM_198841	257	302	416	382
FAM120AOS__NM_198841_ATGGGAAAAACTAAGGATAT	FAM120AOS__NM_198841	184	48	258	185
FAM120AOS__NM_198841_CCAGGGCGCAGAATGTCGCC	FAM120AOS__NM_198841	233	146	226	202
FAM120AOS__NM_198841_CGCCCTGGGAAGGGGGATAG	FAM120AOS__NM_198841	276	302	324	464
FAM120AOS__NM_198841_GAGAGCCCCGGACCAGAATT	FAM120AOS__NM_198841	283	333	318	386
FAM120AOS__NM_198841_TCTCCAGCTGTCCCTGTTCG	FAM120AOS__NM_198841	2336	2449	2734	2984
FAM120AOS__NM_198841_TGGGAAAAACTAAGGATATT	FAM120AOS__NM_198841	4	0	10	6
FAM120B__NM_001286379_CAGAAAAGTATTCTCGCCAC	FAM120B__NM_001286379	780	759	922	798
FAM120B__NM_001286379_CCAATATCTGAGACAACACA	FAM120B__NM_001286379	145	125	192	251
FAM120B__NM_001286379_CCAGAATCTTGGATCTGCGG	FAM120B__NM_001286379	960	809	1008	754
FAM120B__NM_001286379_CCATAACTGAACTCCGGGAA	FAM120B__NM_001286379	524	684	482	554
FAM120B__NM_001286379_CCATTTGGCGATCCATTGAG	FAM120B__NM_001286379	614	728	600	738
FAM120B__NM_001286379_CCTCTCAATGGATCGCCAAA	FAM120B__NM_001286379	339	362	376	503
FAM120B__NM_001286379_CCTTTCCCGGAGTTCAGTTA	FAM120B__NM_001286379	805	994	771	822
FAM120B__NM_001286379_CTTTCCCGGAGTTCAGTTAT	FAM120B__NM_001286379	1423	1801	1530	1353
FAM120B__NM_001286379_GGCATCAACCACAATGGTAG	FAM120B__NM_001286379	160	44	158	126
FAM120B__NM_001286379_TACATCCAGGATACTTGCTT	FAM120B__NM_001286379	776	978	955	1112
FAM120B__NM_001286379_TACTGTACATATATGTGGGC	FAM120B__NM_001286379	204	204	268	298
FAM120B__NM_001286379_TCACACCCATAACTGAACTC	FAM120B__NM_001286379	594	517	523	508
FAM120B__NM_001286380_AGAACAACAGGGAGATATCC	FAM120B__NM_001286380	107	96	82	182
FAM120B__NM_001286380_GAGATGAATGGGTGAAACGA	FAM120B__NM_001286380	129	124	133	154
FAM120B__NM_001286381_ACGGCTGTCCCTGACTGGAA	FAM120B__NM_001286381	392	463	448	443
FAM120B__NM_001286381_AGAAATACAGGTTCGGCGCT	FAM120B__NM_001286381	483	506	748	788
FAM120B__NM_001286381_CCTGAAGGCAGTCGTCTGCA	FAM120B__NM_001286381	981	893	1113	1064
FAM120B__NM_001286381_GCTGTTGACTAAAACCAGAG	FAM120B__NM_001286381	439	204	409	338
FAM120B__NM_001286381_GGCTCCAGCTACCACAGGAC	FAM120B__NM_001286381	740	1094	852	1161
FAM120B__NM_001286381_GGGTATAGCCGTTCCAGTCA	FAM120B__NM_001286381	1335	1413	1488	1759
FAM120B__NM_001286381_TGCCTTCAGGTTGTGGAATT	FAM120B__NM_001286381	606	717	680	839
FAM120B__NM_001286381_TGGGTATAGCCGTTCCAGTC	FAM120B__NM_001286381	123	54	110	85
FAM120B__NM_001286381_TTTGCATGCGTTTATTGCGC	FAM120B__NM_001286381	243	458	349	558
FAM120B__NM_032448_ATATGTTCTTCATCCCCTCA	FAM120B__NM_032448	309	328	479	449
FAM120B__NM_032448_ATGAAGAACATATTTCTGCC	FAM120B__NM_032448	525	429	542	553
FAM120B__NM_032448_CGATTTGCTCTAAAGACACT	FAM120B__NM_032448	301	225	366	554
FAM120B__NM_032448_CTTGATGTAATGAAAAATCC	FAM120B__NM_032448	196	121	211	251
FAM120C__NM_017848_AATATTGTAGAGAGCATACT	FAM120C__NM_017848	58	12	69	35
FAM120C__NM_017848_AGAGAGCATACTCGGAGTCA	FAM120C__NM_017848	501	676	614	570
FAM120C__NM_017848_AGCCCAGCATGGCGTTCCAT	FAM120C__NM_017848	0	0	0	0
FAM120C__NM_017848_CGCCACACACCCAATCCGTC	FAM120C__NM_017848	1777	2120	1989	1780
FAM120C__NM_017848_GCAGACAGCGCAACTGATCG	FAM120C__NM_017848	1095	1455	1366	1773
FAM120C__NM_017848_GCATGGGAACTGTAGTAAGA	FAM120C__NM_017848	810	1009	795	859
FAM120C__NM_017848_GCGCTGCCGCGGCTCTATGG	FAM120C__NM_017848	331	553	292	392
FAM120C__NM_017848_GCGGGGTGATGGTGCCGAAT	FAM120C__NM_017848	787	1069	890	945
FAM120C__NM_017848_TCGGCGCTGCCGCGGCTCTA	FAM120C__NM_017848	112	86	90	86
FAM120C__NM_017848_TGCCTTGGGTGCCTACTCCG	FAM120C__NM_017848	547	630	658	759
FAM120C__NM_017848_TGGCGGCTACCAGACGGATT	FAM120C__NM_017848	155	99	138	135
FAM120C__NM_017848_TGGTAGCCGCCATAGAGCCG	FAM120C__NM_017848	11	0	8	0
FAM122A__NM_138333_CCAATTCCCCGAGTGGGTGA	FAM122A__NM_138333	337	171	394	309
FAM122A__NM_138333_CCAGCACCGTCACCCACTCG	FAM122A__NM_138333	2406	1828	2866	2381
FAM122A__NM_138333_CCGTCACCCACTCGGGGAAT	FAM122A__NM_138333	827	523	876	626
FAM122A__NM_138333_CTGCTTCCCAATTCCCCGAG	FAM122A__NM_138333	791	580	739	730
FAM122A__NM_138333_CTTGTTTGATCTGGTGCAAG	FAM122A__NM_138333	201	201	260	334
FAM122A__NM_138333_GAGCTGCCTCCGGGTACGGG	FAM122A__NM_138333	394	245	415	315
FAM122A__NM_138333_GCTGCTGTGCATGCGGACAG	FAM122A__NM_138333	338	263	359	278
FAM122A__NM_138333_GGAATGAAATCGATGCGCTT	FAM122A__NM_138333	264	530	361	461
FAM122A__NM_138333_GGACTTGATCAACCGAGAGA	FAM122A__NM_138333	239	303	282	156
FAM122A__NM_138333_TCTTCTCCTGAGCCATGTCG	FAM122A__NM_138333	173	72	189	169
FAM122A__NM_138333_TGCCCTCTTCTTGTTTGATC	FAM122A__NM_138333	499	528	501	762
FAM122A__NM_138333_TGCTTCCCAATTCCCCGAGT	FAM122A__NM_138333	280	216	348	188
FAM122B__NM_001166599_AACCTTACACACTTAGAACT	FAM122B__NM_001166599	664	648	655	748
FAM122B__NM_001166599_CAATGCAGATAAGCCAATCA	FAM122B__NM_001166599	431	618	581	779
FAM122B__NM_001166599_CACCTGAGCCCATGGATTAG	FAM122B__NM_001166599	375	366	526	402
FAM122B__NM_001166599_CTCCGAGTTCTAAGTGTGTA	FAM122B__NM_001166599	594	822	670	1006
FAM122B__NM_001166599_CTTACACACTTAGAACTCGG	FAM122B__NM_001166599	750	574	754	707
FAM122B__NM_001166599_CTTGAGCCTGACACATCTTA	FAM122B__NM_001166599	261	327	416	408
FAM122B__NM_001166599_GCAGCGCTCCCCTAATCCAT	FAM122B__NM_001166599	1794	2197	1931	1756
FAM122B__NM_001166599_GCAGGAAGAAGGCCTGGATA	FAM122B__NM_001166599	410	180	445	296
FAM122B__NM_001166599_GTTCCCCCATAAGATGTGTC	FAM122B__NM_001166599	103	122	118	138
FAM122B__NM_001166599_TCAAGCTCTCATCCCATGAT	FAM122B__NM_001166599	190	334	228	268
FAM122B__NM_001166599_TCACCTGAGCCCATGGATTA	FAM122B__NM_001166599	988	1171	1032	861
FAM122B__NM_001166599_TTGAGCCTGACACATCTTAT	FAM122B__NM_001166599	249	197	419	365
FAM122B__NM_001166600_ACTAGGAATACGATTAGGTG	FAM122B__NM_001166600	104	170	152	105
FAM122B__NM_001166600_ATCTGATGCAGTCTGCTACT	FAM122B__NM_001166600	351	305	336	405
FAM122B__NM_001170757_ACTGAGATCAGACAGTTGCG	FAM122B__NM_001170757	601	787	639	594
FAM122B__NM_001170757_CTGGGTCTAATGCACTTGAC	FAM122B__NM_001170757	154	148	162	170
FAM122B__NM_001170757_GGCATCTGGAGATAACATAT	FAM122B__NM_001170757	781	693	786	581
FAM122C__NM_001170779_AAATGAGAACCAGTATCAGA	FAM122C__NM_001170779	188	141	185	255
FAM122C__NM_001170779_AACAGTGCCCCTTTGATCAA	FAM122C__NM_001170779	787	1080	862	892
FAM122C__NM_001170779_AATGTTGCCGTCTGCGGTAG	FAM122C__NM_001170779	369	413	404	516
FAM122C__NM_001170779_CCGAGTTCCACTACCGCAGA	FAM122C__NM_001170779	940	1146	1158	1354
FAM122C__NM_001170779_CCTGTGTCACCTTCTGATAC	FAM122C__NM_001170779	1638	2442	1757	1962
FAM122C__NM_001170779_GCGGCTGATGGAACCATGAA	FAM122C__NM_001170779	263	350	313	421
FAM122C__NM_001170779_GGACAAACAGAACAACATTT	FAM122C__NM_001170779	996	1007	1098	1154
FAM122C__NM_001170779_TCGTTTCCATCAAAAGTAGC	FAM122C__NM_001170779	134	147	163	189
FAM122C__NM_001170780_ACTGATCAAGAAACACAGCT	FAM122C__NM_001170780	436	560	577	657
FAM122C__NM_138819_GCTTCTTCCATCAAGAAGAC	FAM122C__NM_138819	87	153	114	114
FAM122C__NM_138819_TTTAACTCCAGTATCCTCAA	FAM122C__NM_138819	83	81	121	112
FAM124A__NM_001242312_ACCCCGACCTCCCGCTGTTC	FAM124A__NM_001242312	2365	1879	2593	2015
FAM124A__NM_001242312_AGGTGAGCTTTCCGTTGAAG	FAM124A__NM_001242312	415	362	349	522
FAM124A__NM_001242312_ATCCACATAATCGCAGACCC	FAM124A__NM_001242312	778	952	864	806
FAM124A__NM_001242312_ATTTCCTTGCCGTAGTGCAC	FAM124A__NM_001242312	149	216	275	144
FAM124A__NM_001242312_CCCCTGGGTCTGCGATTATG	FAM124A__NM_001242312	700	797	821	1038
FAM124A__NM_001242312_CCGGCCCGTGCACTACGGCA	FAM124A__NM_001242312	712	1095	837	1109
FAM124A__NM_001242312_CTCTCGGACACCCGGAACAG	FAM124A__NM_001242312	283	281	169	190
FAM124A__NM_001242312_GATTTCCTTGCCGTAGTGCA	FAM124A__NM_001242312	366	491	298	321
FAM124A__NM_001242312_GCCATCCGGCCCGTGCACTA	FAM124A__NM_001242312	21	8	3	0
FAM124A__NM_001242312_GCGCTGGCTGAGCGCCCTTG	FAM124A__NM_001242312	0	0	0	0
FAM124A__NM_001242312_GGAGGCCATCGACAACGTCC	FAM124A__NM_001242312	640	526	699	629
FAM124A__NM_001242312_TGGCCCAAAGCGGCGTCCCA	FAM124A__NM_001242312	89	87	91	91
FAM124B__NM_001122779_AAACCGGGACCGCTTGGAAT	FAM124B__NM_001122779	583	758	786	799
FAM124B__NM_001122779_AGCACTGCCATGGCGAATGC	FAM124B__NM_001122779	232	177	266	249
FAM124B__NM_001122779_CCACCCAGGACACTCGGGGA	FAM124B__NM_001122779	1140	1042	1427	1334
FAM124B__NM_001122779_CCTCTCCCGGGCTTTCGTGC	FAM124B__NM_001122779	956	826	1064	936
FAM124B__NM_001122779_CTCTTCCTGCACGAAAGCCC	FAM124B__NM_001122779	151	104	168	143
FAM124B__NM_001122779_GCTCTTCCTGCACGAAAGCC	FAM124B__NM_001122779	254	338	379	410
FAM124B__NM_001122779_GCTGATAGGCATGCATGGAT	FAM124B__NM_001122779	492	308	564	521
FAM124B__NM_001122779_GGATAGGCTATTTCGCGTCC	FAM124B__NM_001122779	1293	2010	1839	2387
FAM124B__NM_001122779_GTGCTGCTGATAGGCATGCA	FAM124B__NM_001122779	280	430	342	406
FAM124B__NM_001122779_GTTCAGCTGAATCCAGAACT	FAM124B__NM_001122779	209	172	253	270
FAM124B__NM_001122779_TGGCAGACTCAGGACTACGA	FAM124B__NM_001122779	41	25	52	29
FAM124B__NM_001122779_TGGGTGGGGTAGCACTGCCA	FAM124B__NM_001122779	53	86	48	47
FAM126A__NM_032581_AAAAAAAGACTCAAACCAAG	FAM126A__NM_032581	60	46	86	79
FAM126A__NM_032581_AACACTGGGCATGTAAGTTA	FAM126A__NM_032581	645	611	697	686
FAM126A__NM_032581_AAGCTGATTTACCCTACTGC	FAM126A__NM_032581	457	470	579	606
FAM126A__NM_032581_AAGGATTCCACACAAAATTG	FAM126A__NM_032581	87	120	54	102
FAM126A__NM_032581_AGATGCTGACAGCACAGAAT	FAM126A__NM_032581	202	197	204	234
FAM126A__NM_032581_AGTTGGTTATGTCTCATGTG	FAM126A__NM_032581	218	315	166	248
FAM126A__NM_032581_ATCTTCCCTTCATAAAGTCA	FAM126A__NM_032581	224	196	207	249
FAM126A__NM_032581_ATGTCTCATGTGAGGTTGTA	FAM126A__NM_032581	286	495	342	319
FAM126A__NM_032581_CAAAATCCACATTGTAAACA	FAM126A__NM_032581	212	154	226	320
[truncated: 9,225,875 more chars]
